# Mobile-Health based physical activities co-production policies towards cardiovascular diseases prevention: findings from a mixed-method systematic review

**DOI:** 10.1186/s12913-022-07637-8

**Published:** 2022-03-01

**Authors:** Gabriele Palozzi, Gianluca Antonucci

**Affiliations:** 1 Department Management & Law, University of Rome Tor Vergata, Rome, Italy; 2 DEA Department, “G. d’Annunzio” University of Chieti-Pescara, Viale Pindaro, 42, Pescara, 65127 Italy

**Keywords:** Co-production, Healthcare, Cardiovascular Disease, Physical activity, Sports, Mobile-health, Bibliometric analysis, Health Policies

## Abstract

**Background:**

Cardiovascular disease (CVD) is the first cause of death globally, with huge costs worldwide. Most cases of CVD could be prevented by addressing behavioural risk factors. Among these factors, there is physical and amateur sports activity (PASA), which has a linear negative correlation with the risk of CVD. Nevertheless, attempts to encourage PASA, as exercise prescription programmes, achieved little impact at the community-wide level. A new frontier to promote PASA is represented by mobile health tools, such as exergaming, mobile device apps, health wearables, GPS/GIS and virtual reality. Nevertheless, there has not yet been any evident turnabout in patient active involvement towards CVD prevention, and inactivity rates are even increasing. This study aims at framing the state of the art of the literature about the use of m-health in supporting PASA, as a user-centric innovation strategy, to promote co-production health policies aiming at CVD prevention.

**Methods:**

A mixed-method systematic literature review was conducted in the fields of health and healthcare management to highlight the intersections between PASA promotion and m-health tools in fostering co-produced services focused on CVD prevention. The literature has been extracted by the PRISMA logic application. The resulting sample has been first statistically described by a bibliometric approach and then further investigated with a conceptual analysis of the most relevant contributions, which have been qualitatively analysed.

**Results:**

We identified 2,295 studies, on which we ran the bibliometric analysis. After narrowing the research around the co-production field, we found 10 papers relevant for the concept analysis of contents. The interest about the theme has increased in the last two decades, with a high prevalence of contributions from higher income countries and those with higher CVD incidence. The field of research is highly multi-disciplinary; most of documents belong to the medical field, with only a few interconnections with the technology and health policy spheres. Although the involvement of patients is recognized as fundamental for CVD prevention through PASA, co-design schemes are still lacking at the public management level.

**Conclusions:**

While the link between the subjects of motor activity, medicine and technology is clear, the involvement of citizens in the service delivery process is still underinvestigated, especially the issue concerning how “value co-creation” could effectively be applied by public agencies. In synthesis, the analysis of the role of co-production as a system coordination method, which is so important in designing and implementing preventive care, is still lacking.

**Supplementary Information:**

The online version contains supplementary material available at 10.1186/s12913-022-07637-8.

## Background

Cardiovascular disease (CVD) is the cause of 31% of all global deaths [1] and determines huge burdens and high costs of medical care worldwide [2–4]. For instance, in the USA, CVD costs $555 billion per year [5]; the total costs of CVD in the main European economies were, instead, estimated as €122.6 billion in 2020 [6]. This is because CVD costs have impacts not only in terms of deaths and medical interventions but also in terms of complications [7] and risk factors [8], which makes CVD a leading cause of disability [9]. Most cases of CVD could be prevented by addressing behavioural risk factors through proper population-wide strategies [9]. People with CVD, especially those who are at high cardiovascular risk, need early detection and management, using counselling and medicines, as well as behavioural changes [10]. Effective behavioural changes could indeed prevent 75% of all cardiac events [11].

Among the behavioural change factors, there is physical and amateur sports activity (PASA), which shows a linear negative correlation with the risk of CVD, regardless of age and gender [12]. Nevertheless, levels of inactivity in high-income Western countries are increasing [5, 13], even in children and youngsters [14], thus precluding any virtuous trajectory. Programmes to reduce physical inactivity have made little impact at the community-wide level [15, 16], because the promotion of PASA is not an easy process. It involves several aspects and deserves proper investments in terms of policy programmes in different fields (communication, public education, sport and recreation, etc.), ranging from infrastructure and urban design to healthcare and health education, thus necessitating the design, financing, and implementation of proper community-wide programmes [17].

A new frontier to promote PASA might be mobile (m)-health [18], which is a system that improves healthcare service delivery processes through the use of various apps, as well as the basic utilities of mobile or wireless devices [19, 20]. M-health changes the traditional modes of information sharing and dissemination [21], thus increasing health promotion, disease prevention, provision of care, and monitoring [22]. New technologies have been implemented to promote PASA [23–25], and, specifically, PASA to prevent CVD [26, 27]. These digital healthcare tools include exergaming (active video games), social media, mobile device apps, health wearables, mobile games, augmented reality games, global positioning and geographic information systems (GPS/GIS), and virtual reality [28].

An effective application of digital health requires an active role by the patient, especially if the target is the promotion of his/her daily PASA, because the active lifestyle is his/her responsibility. The effective participation of patients in health care, found different labels in the last decades such as: patient centredness, patient engagement, patient experience [29]. A common baseline has been found in co-production, which is having greater attention in health care [30], gaining recognition to an approach to create patient partnerships that enable better functioning healthcare systems [31]. Indeed, the active patients’ involvement is increasingly pursued in order to improve the quality of life [32], as in the case of being active in doing regular PASA as a preventive care. In cases such as this, policymakers can reduce costs while concurrently improve the quality of the service, through an imperative patients’ engagement, asking them to interact with healthcare professionals and other decision-makers in healthcare [33].

Despite the above reported considerations, the linkage among literatures belonging to PASA and m-health fields in addressing CVD related issues in a co-production scheme is still missing. Accordingly, as reported by the review from Abu-Omar et al. [34], existing evidence about cost-effective initiatives of PASA promotion in CVD prevention appears scant and scattered; hence, physical activities have not been largely used yet as a support for conventional treatments [35]. Moreover, the role of e-tools in PASA prescription remains under-investigated, according to Piwek et al. [36]; particularly, e-health involvement in co-produced prevention policies still seems to hold much promise towards expanding the clinical repertoire of the future, rather than being already commonly used strategies [37]. This is, probably, the result of still existing barriers towards digital health technology diffusion, which should be properly tackled, such as the needs for social connectedness, access, and programme availability [38].

In synthesis, although there has been an increase of pilots demonstrating the effectiveness of m-health programmes to promote PASA to prevent CVD, they do not yet account for or guide any process of change for implementing and adopting effective community-wide programmes. This is because studies remain fragmented, thus lacking in knowledge about, for instance, the frequency of practices used, the produced outcomes, and, especially, if and how the pilots can be enlarged and replicated, thus driving the needed change at the wider community level [39]. In other words, to date, matters of PASA, m-health and CVD are considered in literature as separate sub-fields of medicine and biomedical engineering without any clear connection with public policies and citizenship involvement. In our opinion, instead, a complex issue of decreasing charge related to CVD management needs to be tackled by a holistic approach considering the cause-effect relationship among the problem (CVD prevention), the clinical intervention (PASA prescription) and the operating solution for monitoring patient engagement and literacy (m-health strategies).

Particularly, starting from the above considerations, we investigate the issue in an integrated way, considering the relevance of all the involved research fields and having, as common denominator, the co-creation scheme [40] as reference for the implementation of the needed effective community-wide programmes. Indeed, given the relevance of behavioural changes, we define a basic element in the useful innovation. That is the one given by patients, to serve their own needs and to develop and improve tools for medical care needs [41] based on the innovation theories of users [42, 43]. Hence. the concept of patient and public involvement (PPI) in driving both the design and implementation of healthcare services and products, as well as the governance of healthcare provider organizations [44, 45].

Accordingly, our study intends to fill this gap, giving an integrated overview of the situation in the different specific involved fields towards public management overview. Aiming at finding indications for a cohesive learning, rather than an increase in knowledge in each separate scientific field, the goal of this manuscript is to frame the state of the art of the literature about the use of PASA, monitored by m-health technologies, as a user-centric innovation strategy to promote co-production health policies aiming at CVD prevention.

Consequently, this research presents twofold aims; the first one, developed through quantitative literature review, investigates the existing intersections, among the involved research fields, in studies seeking to foster services for CVD prevention. Starting from the result of the first one, the second enquiry focuses on qualitative understanding upon the role of patients, as part of co-production schemes, to boost the use of PASA in prevention policies.

As far as we know, the relevant innovative aspect in this paper is not only in the comprehensive literature review per se but especially in the fact that it has been properly designed in order to consider the presence (or absence) and the relevance of “interconnections” among different fields. The research indeed covers health policy and health services management on one side and sport policies and PASA promotion on the other one, passing through the field of ICT development for the design and implementation of proper apps. All the above depicted within an outline able to foster co-production schemes in a co-creation environment, where participants could have an active role in the realization of a healthy lifestyle for themselves in an innovation framework for users.

## Materials and methods

A mixed-method systematic literature review [46] was conducted in the fields of health and healthcare management in order to understand how the use of e-tools, to promote and monitor PASA, could be actually considered as a m-health based innovation strategy to promote health policies aiming at CVD prevention.

### Data Collection

Scopus was the database used for conducting the research. The keywords (KWs) used for the whole inquiry are contained in Table 1. We aimed at investigating the significance of physical activities applications (1st KW) within the field of Health Policy (2nd KW) through m-health infrastructures (3rd KW) for enhancing prevention (4th KW) in patients with CVD (5th KW).

At the first stage of enquiry, the keywords search criteria was steered by the goal to understand how PASA, as boosted by forms of digital technologies, are considered within public bodies of interventions as a leverage to improve CVD care. As a consequence, as drawn by the existing literature the search includes synonyms of: (i) physical activities; (ii) health policy; (iii) mobile health; (iv) prevention; (v) cardiovascular diseases. Conversely, the search did not include any explicit reference to the word “management” (“manag*”), because of its too-broad meaning which would risk to include source results not pertinent to the enquiry aims. Moreover, the sphere of public management and governance, towards co-production schemes, has been touched by adding the sixth keywords string, according to the qualitative conceptual analysis of the literature provided in the second step of investigation.

**Table 1:** Keywords used for the inquiry

1st Keywords	‘physical activity’ or ‘motor activity’ or ‘sport activity’
**And**	
**2nd Keywords**	‘health policy’ or ‘policy’ or ‘healthcare’ or ‘health care’ or ‘public’
**And**	
**3rd Keywords**	‘mobile health’ or ‘m-health’ or ‘e-health’ or ‘ehealth’ or ‘mhealth’ or ‘telemedicine’ or ‘tele care’ or ‘telecare’
**And**	
**4th Keywords**	‘prevention’ or ‘life’ or ‘quality’
**And**	
**5th Keywords**	‘cardiovascular’ or ‘cardio’ or ‘CVD’

Source: Authors’ illustration

Keywords contained in the same column are an alternative within them. Papers that contained at least one keyword belonging to each column, within the title and/or abstract and/or keywords, were considered relevant for this study. Other search criteria used to define the selection of papers were the following:

- Language: only studies published in English were selected;
- Document type: only peer-reviewed articles, reviews and book chapters were considered. The other categories of sources from Scopus, such as conference papers, editorials, articles in press, conference proceedings, and letters, were excluded. No further grey literature, as guidelines and clinical protocols, were considered.

Disciplines excluded from the research setting were: Agricultural and Biological Sciences, Arts and Humanities, Earth and Planetary Sciences, Energy, Environmental Science, Materials Science, Mathematics, Physics and Astronomy, and Veterinary.

**Fig. 1:**
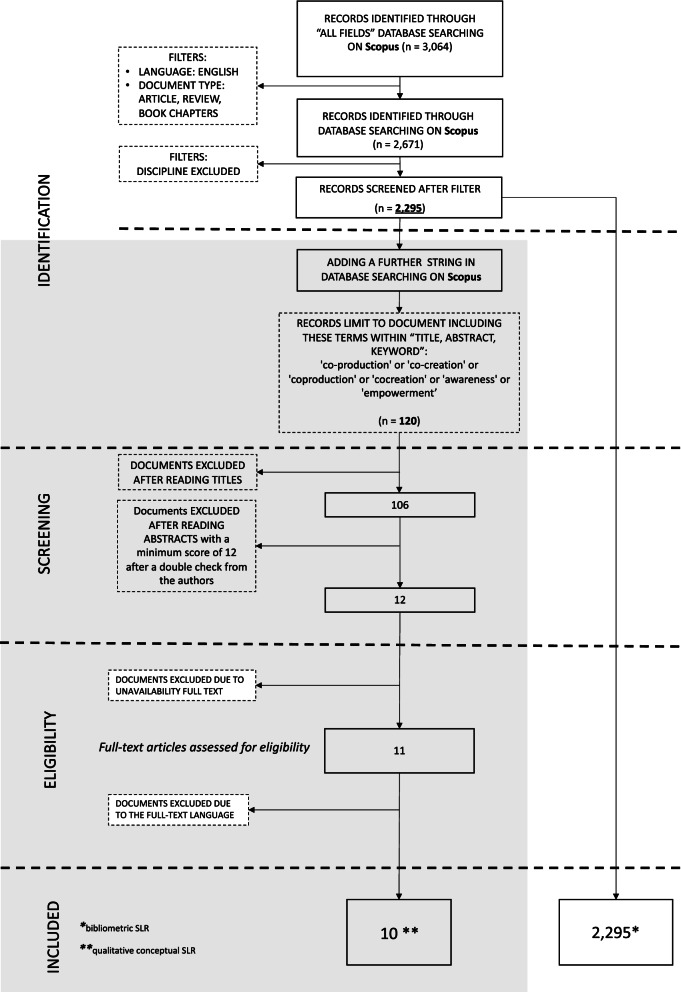
Paper selection and extraction template. White side: bibliometric SLR; Grey side: conceptual SLR. Source: Authors’ elaboration from the PRISMA model

According to the results there were no publications before 1989, and this is in line with the fact that first indications about relevance and effectiveness of Physical Activity as preventive care arose at the end of the ‘70s and the first studies about PASA as preventive factor for CVD arrived about ten years later [47]. Hence, we considered the whole spectrum of relevant articles. After this process of selection, as shown in Fig. 1 (white side), the entire dataset included 2,295 studies at the end of April 2021.

### Bibliometric Data Analysis

The bibliometric analysis of the literature is a relatively new statistical method whose goals are studying the editorial and textual information of written documents; this method appears to be a systematic, transparent, and replicable process for literature reviews [48, 49], allowing users to quantitatively examine the bibliographic state of the art of a topic or issue by statistically analysing interrelated information within a dataset (composed by metadata of the sample of selected documents).

According to Duriex [50], the bibliometric analysis is employed by scholars to statistically/mathematically measure the relative importance of a particular issue within a paper (e.g., scientific fields, keywords, authors, or country). There are three main types of bibliometric indicators: (i) *Quantity* (e.g., number of publications); (ii) *Performance* (e.g., citations); and (iii) *Structural-Networking* (e.g., connections between publications, authors, or research fields) [51]. Unlike a more common meta-analysis, a bibliometric analysis is only aimed at quantitatively describing objective variables among a group of selected studies, without any reference to their contents or findings [52].

The bibliometric analysis has been conducted on the entire dataset of 2,295 selected papers.

Depending on the different needs in data representation, the bibliometric analysis of the entire dataset was conducted on the metadata downloaded from Scopus by using: (i) a statistical-descriptive spreadsheet tool, (ii) VOSviewer software; and (iii) the Biblioshiny web-interface of the Bibliometrix R Package.

### Quantity and Performance Analyses

The bibliometric quantity and performance analyses focus on sample features by quantifying the research into the specific field, identifying the most important actors and evaluating groups of scientific producers. Moreover, a citation analysis was performed at this stage, and this was based on the hypothesis that most cited actors can be considered as leading experts for the field of research.

In particular, the following dimensions have been analysed:

i. Documents/years;
ii. Documents/years publication growth rate in the last two decades.
iii. Documents/leading country.
iv. Frequency of documents/country.
v. Most cited countries;
vi. Most cited journals.

### Structural/Networking Analysis

The bibliometric structural/networking analysis aims at understanding the scientific mapping [33] of the intellectual structure of the field together with the scientific collaboration among actors.

The scientific collaboration, instead identifies the social structure of the field, by networking the actors via nodes concerning links to the subject.

The co-citation analysis, finally, aims at capturing the intellectual structure of the field. Co-citation is defined as the frequency with which two subjects are cited together; it is based on the hypothesis that what is co-cited is conceptually close.

The co-occurrence analysis shows the statistical correlation among two terms within an examined dataset [53]—the higher the frequency with which the two terms are simultaneously cited is, the stronger their expected logical connection. Specifically, the co-word analysis is based on the precondition that the co-occurrence of key terms, by describing the contents of documents, captures those semantic or conceptual groups of topics able to depict a field.

Accordingly, the following dimensions have been analysed:

i. Country collaboration;
ii. Co-citation among journals;
iii. Co-occurrence of keywords;
iv. Thematic maps of authors’ keywords.

### Qualitative Conceptual Analysis

Additionally, we reflected upon the fact that the bibliometric analysis, although rigorous and complete, may not be able to directly capture the relevance of co-production policies and experiences in CVD prevention through PASA using e-tools.

Therefore, we decided to deepen our research by running a selective content analysis.

Thus, in order to understand, more specifically, which research has touched the specific theme of fostering PASA in order to “co-produce” prevention of CVD, a further qualitative descriptive and conceptual analysis [54, 55] was conducted.

Accordingly, starting from the entire dataset (par. 2.1), we added the following further keyword strings in Scopus (in Title, Abstract & Keywords):

*‘co-production’ or ‘co-creation’ or ‘coproduction’ or ‘cocreation’ or ‘awareness’ or ‘empowerment’.*

In order to intercept the entire knowledge base regarding health policy promotion from every standpoint about the field, no further selection of disciplines was applied. Thus, we obtained 120 documents to be screened.

We initiated this further step of analysis considering the sample of 120 articles. We moved towards the evaluation of documents by applying the PRISMA logic [56] for the selection of papers included in the conceptual review. For the selection, particularly, we constructed an evaluation framework of all the selected abstracts as a type of applied active research [57], starting from the text analysis [58]. The text analysis of the abstracts was run by two different evaluators providing double-blind scoring (adapted from [59, 60]) about aspects deductively and logically identified on the four main themes of the research: (i) CVD, (ii) physical activities, (iii) m-health, and (iv) co-production. The two evaluators scored, separately, all the documents, giving, for each theme, a score from 1 (low adherence) to 5 (high adherence), according to the relevance of the presence of the evaluated thematic within the abstract [61, 62]. The goal of this analysis was to understand which co-production experiences have already promoted wellbeing and CVD prevention through the use of PASA supported by m-health technologies. The 11 documents that have reached more than 12 as an average score in the evaluation were selected. A score of 12, representing 60% of the highest assessment score, was considered as the “passing mark”.

Afterwards, we checked the full texts of the selected papers, and we had to reject one document because of misclassification by Scopus as an English full-text, but was actually written in the Slovenian language with an abstract in English.

As a result, we selected 10 papers for the qualitative conceptual analysis, as reported in Fig. 1 (grey side).

## Results

This section presents results of the systematic literature review proposed in this manuscript, by following the three analyses as presented in the previous section.

An appropriate understanding of bibliometric findings including in the following Figures from 4 to 9, has to take into consideration that the graphic representation of the results highlights a correlation between variables, whose meaning should be read by the following general rules:

- the greater the circle representing the variable, the higher the recurrence of that variable in the sample of documents;
- the closer the circles representing two or more variables, the higher the correlation among the variables;
- the thicker the connection line among two variables, the stronger the relationship among those variables in the sample of documents.

### Quantity and Performance Analyses

Table 2 briefly reports the main quantitative information about the entire dataset of documents for the bibliometric sample.

**Table 2:** Quantitative information about the bibliometric sample

Description	Results
MAIN INFORMATION ABOUT THE DATA	
Timespan	1989:2021
Documents	2,295
Average years from publication	3.65
Average citations per document	38.14
Average citations per year per document	6.17
Total references	250,622
DOCUMENT TYPES	
Article	1,569
Book chapter	90
Review	636
AUTHORS & COLLABORATION	
Authors	14,890
Authors of single-authored documents	80
Average authors per document	6.49
Collaboration Index	6.74

Source: Authors’ elaboration Biblioshiny web-interface of Bibliometrix R Package

**Fig. 2:**
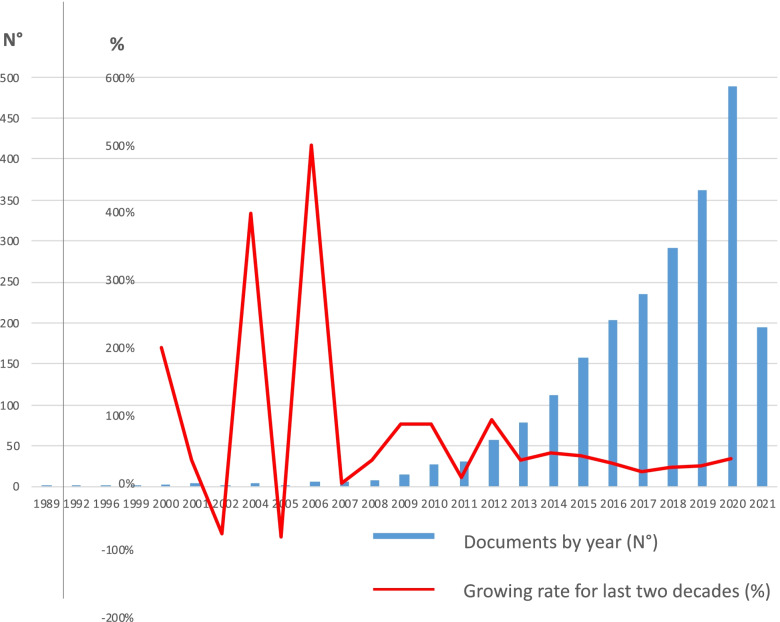
Number of documents by year and growth rate for the last two decades. Source: Authors’ elaboration

As highlighted in Fig. 2, the number of documents published upon the topic has considerably increased in the last two decades. It has gone from a few documents published in the late 1990s to about 480 papers published in 2020. The scientific production growth rate highly increased in the period from 2004 to 2006, by settling about 40% per year since 2013, according to m-health technology advancement [63] and the new theory of value based healthcare [64]. This performance shows an always increasing interest in the topic among scholars, indicating the scientific attractiveness of a social issue, such as CVD, following the availability of new technologies and strategies in addressing health policy solutions to complex problems [65].

**Fig. 3:**
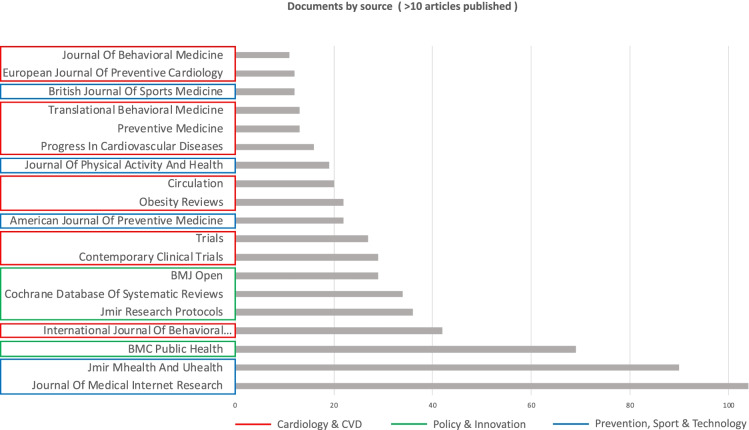
Documents by sources. Coloured labels represent clusters of disciplines. Colours have been assigned by the authors. Source: Authors’ elaboration

In terms of scientific production, the ranks of the most productive sources were also significant. Accordingly, Fig. 3 shows that main sources (> 10 documents published) exclusively regards three fields of research: Cardiology and CVD; Policy and Innovation; and Prevention, Sport and Technology. According to Scopus categorization of disciplines, the three coloured clusters included into Fig. 3 were obtained by grouping the different sources per macro-areas, pointed-out by the authors, by reading each journal aims and scopes. As a consequence, no managerial or public governance implications about the topic have been highlighted.

This means that the topic of CVD prevention is still considered a mostly clinical issue, and its disentanglement through multidisciplinary approaches has yet to be undertaken by public agencies.

The productivity analysis per country showed that the highest number of documents were published referring to cases in the USA, followed by the UK, Australia and Canada.

**Fig. 4:**
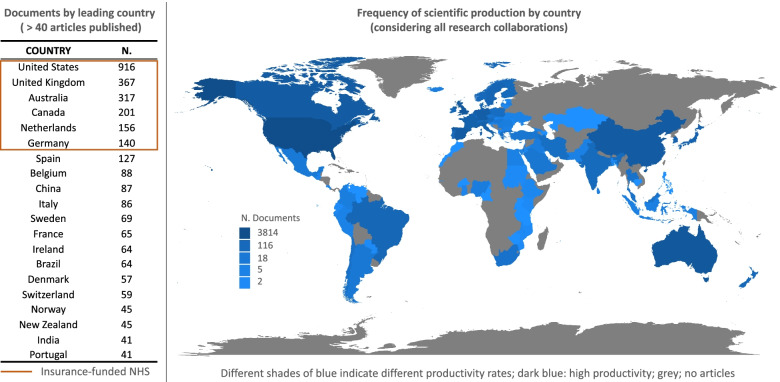
Documents by country. Source: Authors’ elaboration from Biblioshiny web-interface of Bibliometrix R Package

Particularly, the left side of Fig. 4 shows the number of papers provided by each country as research leader (selection in the Fig. 4 with > 40 documents published); it highlights that the most productive leading countries have, at least, two of the following features:

1. Western country/high-income country;
2. Huge dimension and extended rural areas (far from city hospitals);
3. NHS funded/co-funded by compulsory insurance systems.

The map at the right side of the Fig. 4, instead, shows the global scientific production of each country, including all research collaborations among authors, according their geographical provenience.

As a result, the theme of CVD management seems to be more relevant in those countries that have a higher pressure to reduce the costs of healthcare, including chronic diseases [66], compatibly with multi-payer systems financing management [67].

**Fig. 5:**
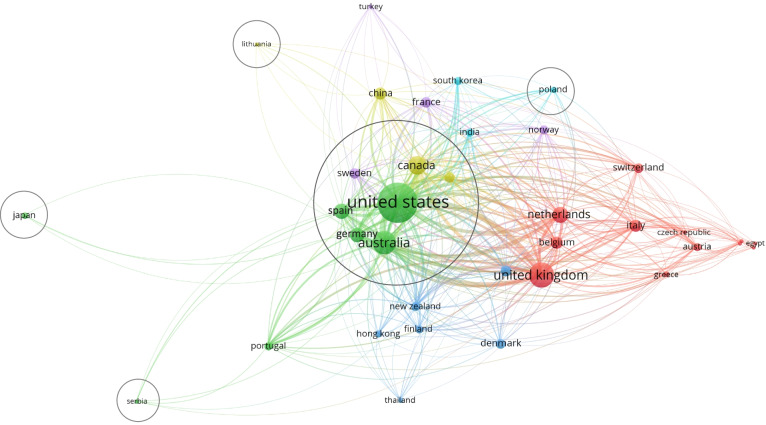
Most cited countries and interactions. Minimum number of citations of a country = 500; Number of countries = 30. Label colours have been automatically assigned by the software. Source: Authors’ elaboration from VOSviewer

As expected, the most productive countries were also the most cited. Particularly, as shown in Fig. 5, countries corresponding to greatest circles (the most cited) are the same with both higher productivity and frequency of scientific production, as shown in the previous Fig. 4.

Furthermore, Fig. 5 shows that exists a polarization of citations (the circle in the centre of the figure that marks “closest” countries per research interaction) for documents coming from countries, which are larger, with high income and co-funded by health insurances.

This, perfectly coherent with the previous findings, can be seen also by the connection lines between countries; thicker lines indicate that the two connected countries have reciprocally cited themselves many times. Thus, when the proximity between the circles representing two countries is high and their connection line is thick, we can expect that research interest about the topic of the review are quite similar.

Particularly, the analysis of the most cited countries adds a further lens of investigation to our inquiry. The most cited countries seem to be also those with a higher prevalence of chronic diseases [68] and obesity-related pathologies (e.g., higher body mass index (BMI) [69]). Accordingly, scholars agree that high-income countries (those with higher life expectancy) tend to develop higher risk factors for non-communicable diseases [70] and, in turn, they observe higher per capita expenditures on CVD [71]; this might explain the stimulus in scientific production with regard to new strategies for CVD care that we found in our analysis.

The less cited countries (small circles in Fig. 5), instead, are those which already use PASA for enhancing wellbeing. Particularly, Lithuania, Poland, Serbia are former Soviet countries, where the sport practices were imposed by the regime and comes into the current practice of the lifestyles of citizens [72, 73]. Japan, instead, is a country where the respect for one’s own body and dietary habits are cornerstones of education and policies, which, in the last 15 years, have pushed people towards carrying out physical activity as the gold standard for the correct lifestyle [74–76].

Hence, we can deduce that the interest about “how to fight CVD through PASA” seems to be correlated with the demographic/epidemiologic features of a country, together with their legacy regarding sport practice culture.

**Fig. 6:**
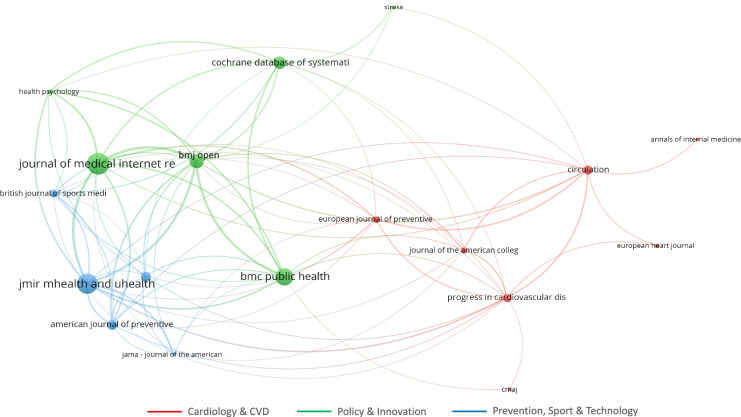
Most cited sources and interactions. Minimum number of citations of a source = 650; Number of sources = 18. Coloured labels represent clusters of disciplines. Colours have automatically been assigned by the software. Names of labels are assigned by the author according to Fig. 3 categorization. Source: Authors’ elaboration from VOSviewer

The analysis of the most cited sources (Fig. 6) integrates the previous investigations by showing that the most interesting areas of research regard the following three main fields (already mentioned in Fig. 3):

- e-health (blue);
- Innovative policy (green);
- Cardiology (red).

All these sources refer to the sphere of medicine and clinical science. Connection lines indicate that the two sources have mutually cited themselves. Thus, thicker is the line, higher is the expected research interest. Moreover, the distance among circles representing sources in Fig. 6 reveals that the use of m-health tracked PASA in CVD prevention is a thematic that mainly concerns new strategies (green and blue sources) for general advancement in ordinary chronic healthcare, rather than a highly specialized heart condition (red sources).

Although the connection between digital technology, PASA and CVD is noted [77], the analysis of the most cited journal confirms that the public management standpoint in addressing the theme of this work is still lacking.

### Structural/Network Analysis

**Fig. 7:**
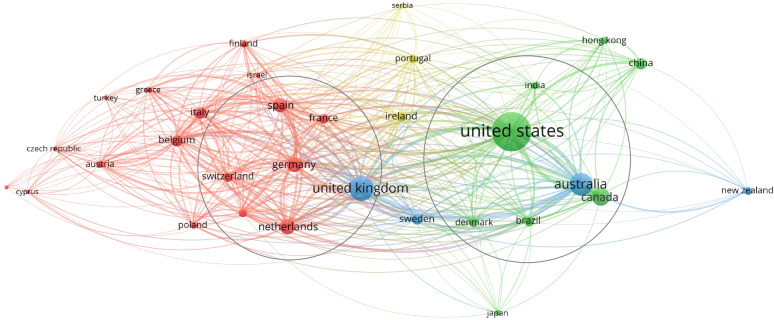
Scientific collaboration among countries. Minimum number of documents of a country = 20; Number of countries = 31. Label colours have been automatically assigned by the software. Source: Authors’ elaboration from VOSviewer

The first structural analysis of the network, regarding research and scientific collaboration among countries, shows two main groups of “collaborators” (Fig. 7), which intercepts similar research necessities:

- North America group and Australia;
- Central Europe group.

These groups of countries have similar features in terms of dimension, income, NHS funding, epidemiology, and culture. These justify research collaborations and cross-country investigations [78]. Accordingly, the UK might be seen as the linking bridge between the two groups, having some common features with both.

**Fig. 8:**
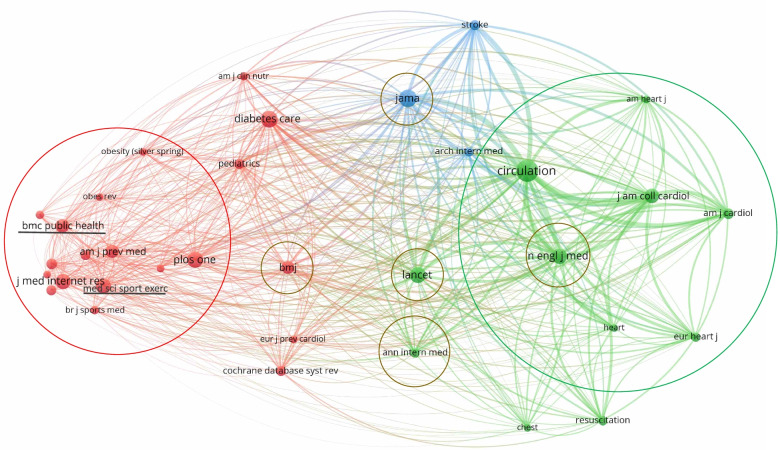
Source co-citation. Minimum number of citations of a source = 520; Number of sources = 34. Label colours have been automatically assigned by the software. Circle colours have been assigned by the authors. Source: Authors’ elaboration from VOSviewer

Moving to the co-citation analysis, this investigation indicates how much two specified objects (sources, authors, keywords, etc.) are simultaneously cited in a third document. Thus, within the heterogeneous medical field, the source co-citation showed an evident prevalence of collaboration of documents belonging to two main sub-fields of interest about the topic. As highlighted by Fig. 8, we can find two main collaboration groups:

- cardiological area (green circle);
- innovation and preventive medicine area (red circle).

In the middle between the two circles, the top five medical journals (Annuals of Internal Medicine, BMJ, Jama, Lancet, and New England Journal of Medicine) [79], whose focus is more generalist, represent a “proved” connection between the sphere of specialist heart disease and new strategies in CVD care.

Particularly, from the co-citation analysis of sources, our results showed that the research about non-communicable disease prevention is strongly related to the subject of innovation in healthcare through telemedicine and sports (red coloured journals: e.g., Journal of Medical Internet Research, American Journal of Preventive Medicine, PLoS One, etc.). Moreover, telemedicine and PASA seem to be directly effective in CVD management [80, 81], while they represent only an accessory support for serious heart conditions [82] (green coloured journals: e.g., Circulation, Journal of American College of Cardiology, European Heart Journal, etc.).

Furthermore, the co-citation analysis connection of the following two journals (highlighted in Fig. 8) is interesting:

- BMC Public Health is strongly correlated with sources concerning physical activities. This confirms that PASA starts to be considered, from a policy standpoint, as leverage for spreading wellbeing among citizens.
- Medicine & Science in Sport & Exercise is correlated with all the sources within the medical field. This confirms again that PASA is considered at powerful tool for enhancing health and healthy lifestyles, with particular benefits on CVD management and prevention.

Nevertheless, as we discuss in the next sections, the critical point of PASA prescription as a medical treatment implicates a deep involvement of citizens and their monitoring within a structured healthcare pathway.

**Fig. 9:**
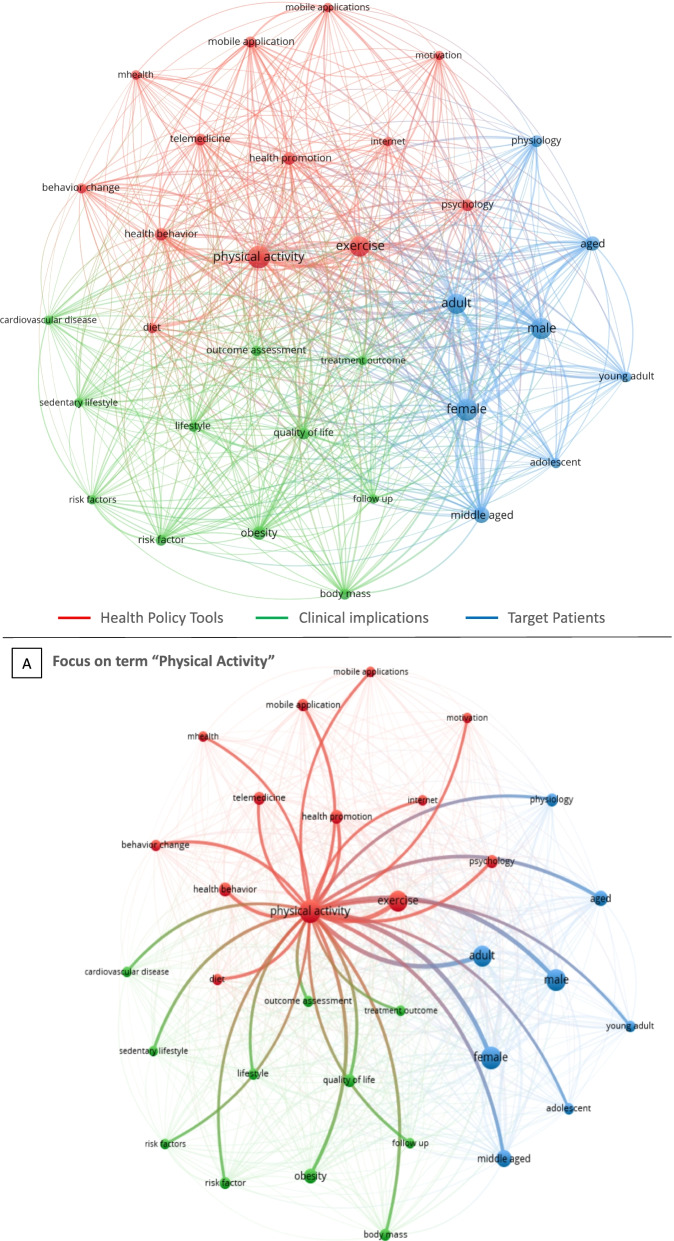
Keywords co-occurrence. Minimum number of occurrences of a keyword = 150; Number of keywords = 45; Number of items = 32. The following 13 keywords have been excluded because they are too general: article, controlled study, human, humans, major clinical study, pathophysiology, priority journal, procedures, questionnaire, randomized controlled trial (topic), randomized controlled trial, review, and systematic review. Label colours have been automatically assigned by the software. The names of labels have been assigned by the authors through macro-grouping of keywords. Source: Authors’ elaboration from VOSviewer

The network inquiry continues with the keyword co-occurrence, as included in Fig. 9.

The analysis depicts very well the state of the art about the topic. Figure 9 indeed reveals the connections among a clinical issue with social implications and the levers handled by public agencies for its resolution.

Particularly, the keywords interconnection shows three clusters of terms, according the macro-grouping provided by the authors considering the meaning of resulting keywords towards research endpoints:

1. Tools in the hands of public agencies (red).
2. Clinical implications (diagnosis, prognosis and outcomes expectations) for implementation of policies (green).
3. Target populations of the health policy interventions (blue).

In other words, the literature recognized that public organizations, in order to obtain an improvement in CVD healthcare and wellbeing promotion (green) for citizens (blue), have three main clusters of levers:

- PASA (e.g., keywords: physical activities, exercise, diet, etc.);
- Technologies (e.g., keywords: telemedicine, mobile health, internet, etc.);
- Cultural change (e.g., keywords: health behaviour, health promotion, motivation, etc.).

This statistical finding confirms the necessity of a multidisciplinary approach in addressing such a complex social and health issue as CVD management. Particularly, the necessity of a cultural change, by motivating and involving patients in healthcare pathways, launches the theme of co-production schemes for quality improvement in healthcare [83], which we analyse in depth in the following section.

Furthermore, it is interesting the keyword “physical activities” and its interconnection with all the other keywords. Figure 9, BOX A shows that this term is the most quoted and is particularly “barycentric” about the topic. This confirms that PASA is recognized, in the literature, as a strategy to address clinical issues related to CVD for a large target population.

Nevertheless, the co-occurrence test on authors’ keywords confirm that the subject is still mostly related to medicine-oriented disciplines; thus, no words related to the co-production sphere are present in the analysis. The statistical results show that policies aiming at involving active patients’ participation towards better CVD management are still an understudied matter from a government point of view. Accordingly, such an issue deserves to be furtherly enquired. Therefore, we decided to deepen our enquiry through a conceptual qualitative analysis of the literature.

Finally, the last analysis concerning the structural network about the topic, refers to the thematic map of the keywords of the authors. This inquiry allows us to synthetize the map of the main themes in the field. Properly, the choice of using the keywords of the authors is appropriate to understand the trend of publishing streams about the topic in the intentions of the authors. Using the visualization technique proposed by Cobo et al. [84, 85], the thematic map is developed by plotting the themes into four quadrants according to density and centrality of each main theme within the sample of documents analysed. Particularly:

- the centrality measures “*the importance of a theme in the development of an entire research field*” ([84] p. 150);
- the density measures “*the internal strength of the network and identifies the degree of development of a theme*” ([33] p. 11).

**Fig. 10:**
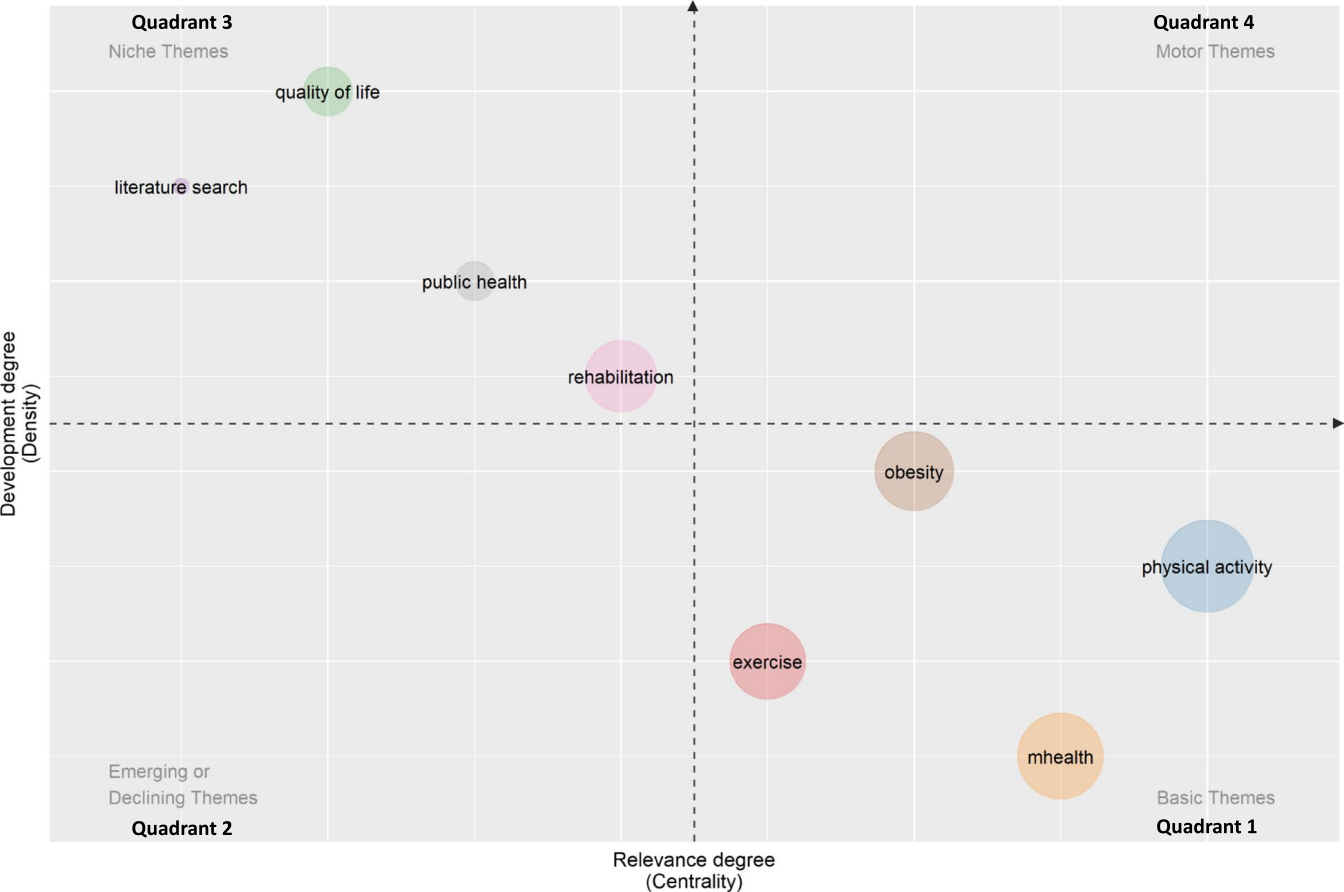
Thematic map of the field. Minimum cluster frequency (per thousand docs) = 2; Number of words = 750. Label colours and widths have been automatically assigned by the software. The size of the cluster is provided by the software based on the number of keyword occurrences; the names of labels are provided by the software as corresponding to the most significant keywords. Source: Authors’ elaboration from Biblioshiny web-interface of Bibliometrix R Package

According to the clusters of themes as presented in Fig. 10, we present, hereafter, the discussion about themes addressing the field we analysed.

- Quadrant 1 - Basic themes (low centrality and high density) regard central subjects about prevention of CVD, but they are still underdeveloped within the field.

They mostly concern umbrella terms, such as “mhealth”, “exercise”, and “physical activities”, which are only generically recognized as levers for contrasting CVD diffusion. The position of these three terms into the thematic map reveals that many scholars write about m-health and PASA for healthcare improvement [86]; nevertheless, their direct inferences with CVD management and prevention are still underinvestigated.

The term “obesity”, instead, is the closest to the “motor theme” quadrant: literature recognizes that nutrition issues, as related to CVD, are those more influenced by carrying out physical activities and by digital tracking for healthcare evolvement and monitoring [87].

- Quadrant 2 - Emerging or declining themes (low centrality and low density) are absent. There are not themes simultaneously irrelevant and underdeveloped within the entire research field. This confirms there are no topics considered both as emerging and as declining in the research area focusing at discovering operating strategies based on PASA and m-health to address the complex theme of CVD prevention. According to what is represented in Fig. 2, the field of investigation is constantly growing; thus the streams of research should be characterized at least by one between relevance (centrality) and dissemination (density). In synthesis the research field, which is not surely brand new, seems to be underinvestigated considering its potential relevance for scholars.
- Quadrant 3 - Niche themes (high centrality and low density) regard highly developed subjects, but they are still seen as accessory to the main field. “Rehabilitation” is the most recurrent term in this cluster. It highlights how PASA, tracked by mobile technology, is recognized by scholars as effective for patient recovery [88]. “Rehabilitation”, with its highest density, confirms that the leading relevance of the field is clinical; that is why the term is the closest to the “motor themes” quadrant.

Nevertheless, among the niche themes, specific topics such as “quality of life” and “public health” have arisen as central ones in addressing public policies [89, 90]. Even if still understudied, the presence of these two keywords confirm that scholars are moving towards new methodologies for addressing complex issues (such as CVD) thanks to “old” implementation strategies (PASA) in “new” pathways (m-health) [91, 92]. Accordingly, the presence of the term “literature search”, confirms that some streams of research have recognized the field as multidisciplinary, and it deserves to be analysed by several literature standpoints.

- Quadrant 4 - Motor themes (high centrality and high density) are absent. This result confirms that the macro topic of “using PASA, supported by mobile health technologies, as a strategy to promote health policies aiming at CVD prevention” is not actually a field of research as a whole. The thematic map shows that the field is still fragmentated within different disciplines, as confirmed by the citation and co-citation analyses of sources. As a consequence, the integration among the different subfields (CVD prevention, PASA, and m-health) is still missing (from a statistical point of view). This explains why the motor theme quadrant is empty: none of the themes is simultaneously central and widespread into the field. Accordingly, from these results, the challenge is to look for a “bridge” topic able to integrate the different mentioned subfields of research towards a public policy logic able to integrate the clinical, technical, and managerial aspects of the holistic theme addressed by this work [93].

#### Qualitative Conceptual Analysis

Table 3 reports the content analysis of the 10 most significant articles, according to the coherence, with the aim of this study (please, see methodology section). Each analysed document, ordered per year of publication (newest first), is described according to the methodology (nine documents are empirical research, and one is theoretical research) and main findings.

**Table 3:** Main findings from the paper included in the narrative content analysis

			Scores^a^				Total	Methodology	Main findings
Year	Authors	Country	CVD	PA	MH	CP			
2021	Okop K.J. et al. [94]	South Africa	5	1.5	4.5	4.5	15.5	Case study on participatory action research (PAR)	Co-creative approach with scientists and citizens who participate together in creating new understandings and a shared agenda, using tablets to facilitate data gathering discussion. The involvement of relevant stakeholders facilitates the training of “citizen scientists”, operating to increase the awareness of CVD risk and the importance of proper lifestyles.
2021	Maxwell et al. [95]	Australia	2	4	4.5	4.5	15	Interview based pilot case study	Focus on the relevance of digital tools in fostering PASA. The study reveals that m-health enhances health by increasing: (i) health literacy; (ii) motivation to exercise; and (iii) accountability about impact of PASA in wellbeing. It identifies technical issues as the main barrier for the use of m-health for fostering PASA.
2020	Monteiro-Guerra F. et al. [96]	Spain	2	4.5	1.5	5	13	Scoping literature review	User engagement via m-health enhances better results of PASA towards wellbeing. The engagement and awareness (co-production) of users are due to the following features to be contained in a physical training APP: (1) Feedback; (2) User Targeting; (3) Goal Setting; (4) Inter-Human Interaction; (5) Adaptation; and (6) Context Awareness; Self Learning.
2020	Harris M.A. & Crone D. [97]	United Kingdom	3.5	5	1	4.5	14	Case study on mobile app physical activity development. Based on focus groups to enucleate positive points and barriers.	The study describes a pilot intervention aiming at a community-wide gamification based physical activity intervention on behalf of Sport England in the City of Wolverhampton. It identifies why participants decided to be engaged and the barriers to participation they encountered.
2020	Kim Y. et al. [98]	Republic of Korea	4	4.5	1	3.5	13	Literature review + explorative case study on real-life setting by focus group interviews.	Based on LR findings, the study analyses the co-development of a mobile app to foster PASA in a sedentary population of migrants with high CVD risk. The remote monitoring the physical, mental, and social health (considering also their specific cultural features) of users led to a change in their behaviours, also favouring inclusion and access to healthcare for early detection of illnesses.
2019	Su J.J. & Yu D.S.F. [99]	China	3	2	4.5	4	13.5	Case study designed on single-blinded two-arm parallel randomized controlled trials comparing the effects of an eHealth CR with usual care on the behavioural and clinical outcomes of patients with CHD who were admitted for disease exacerbation.	The study shows an ongoing nurse-led eHealth cardiac rehabilitation intervention investigating its effects on: lifestyle behaviour, self-efficacy, health-related quality of life and service use. The study employs a hybrid approach guided by an empowerment model. The intervention content and web-design are based on international guidelines and national culturally appropriated recommendations.
2018	Sankaran S. et al. [100]	Belgium	3.5	4	5	5	17	Case study on hearth rehabilitation with two lenses of observation: - Medication adherence - Physical activities.	The paper highlights how the intelligibility of improvements (or failures) through a mobile app in physical rehabilitation is a pre-requisite for protocol success. This happens when you: (i) inform the users; (ii) provide feedback; (iii) enforce identity and action disclosure; and (iv) provide control. Both medication and physical exercise adherence behave in the same way within rehabilitation protocols. A greater training adherence is due to making the reasoning behind PASA in heart disease tele-rehabilitation intelligible.
2017	Zhang H. et al. [101]	Singapore	3	2	4	4	13	Pilot case study based on a randomized controlled trial (RCT) aimed at examining the feasibility and efficacy of a newly developed 4-week smartphone based coronary heart disease prevention (SBCHDP) programme in improving awareness and knowledge of CHD, perceived stress and heart-related lifestyle behaviours among the working population of Singapore.	Compared to the control group, there were more participants in the intervention group who were aware that CHD is the second leading cause of death. The study affirmed that chronic disease prevention via mobile devices is feasible and effective due to its convenience. It represents a convenient, affordable, and accessible method for a substantial proportion of the population.
2017	Jennings C.A. et al. [102]	Canada	2	4.5	1	5	12.5	Case study description of the development of UWALK (eHealth based physical activities promotion program), which is based on the RE-AIM model	The paper highlights the importance of the involvement of citizens and promotion of healthy lifestyles. The UWALK program, aimed at fostering wellbeing by monitoring physical activities through mobile apps (either connected to tracking sensor devices or manually filled by information about exercises carried out), was strongly based on a communication campaign that enhanced the participation and awareness of patients about the issue and predisposition to usage of the app.
2016	Mercer K. et al. [103]	Canada	2	5	1	4	12	Case study based on qualitative research with 32 participants aged between 52 and 85 years who were at risk or had a pathology, using the most common (in 2014) trackers in Canada	The study examines the usability and usefulness of wearable activity trackers for older adults with chronic illnesses, as a first step to better understand how wearable fitness trackers can help older adults become healthier. All the wearable activity trackers tested had a similar score for each item, and 22 of the 30 participants who completed the study said they would purchase a wearable activity tracker.

^a^ Scores (1 min; 5 max) are averaged after a double check from the authors regarding the coherence and pertinence of each paper about the following topics concerning this study: (i) cardiovascular disease (CVD); (ii) physical activity (PA); (iii) mobile-health (MH); and (iv) co-production (CP)

Source: Authors’ elaboration

Source: Authors’ elaboration.

The comprehensive analysis reveals that the use of PASA in fostering wellbeing and healthy lifestyles (also towards CVD prevention by challenging obesity and sedentary lifestyles) is an effective and widespread strategy [88]. Nevertheless, the main limit for prescribing PASA for therapeutic aims is the monitoring of patients in evaluating both the adherence to an exercise protocol and the accuracy of gestures/movements. Without surprise all the analysed documents report on the effectiveness of digital technologies, mostly smartphone based, for activity tracking and monitoring (Parker et al., 2018). The real value of our conceptual analysis relies, however, in proving the awareness of users about the usefulness of m-health applications for healthy living is the critical success factor for their effective employment as boosters of PASA.

Particularly, most of the studies showed that a sort of “virtuous cycle” exists where users, who use training activity mobile apps for enhancing their health status, improve their:

- motivation in doing PASA.
- awareness about their impact on healthy living.
- wellbeing and correct lifestyle.
- learning and growth, both in medical knowledge and digital technology competences.

To this end, the critical issue is the commitment of users. According to the analysed scholars, this should be pursued by:

- policies of participation in mobile app development.
- considering the opinions of users in mobile app features, which makes users more comfortable.
- making the clinical issues and endpoints resulting from carrying out PASA monitored by mobile app intelligible to users/patients.
- considering gaming and interactive challenges as self-reinforcing strategies for improving both performance and constancy of the PASA being carried out.

In this sense, the qualitative conceptual analysis revealed that the participation of users is, unquestionably, the most critical factor for fostering the use of PASA for therapeutic purposes via smartphone applications for tracking their activities.

In other words, a medical subject (such as CVD prevention through PASA) becomes a public management topic inherent in co-production; this implicates the user’s involvement in obtaining his/her clinical results.

The co-production approach, according to our review of the literature, concerns several aspects.

*First*, *user’s participation* is fundamental for the development of public service networks, especially those concerned with healthcare endpoints. Indeed, the analysed work by Okop et al. [94] underlines the importance of co-creating shared programmes between scientists and citizens, with the aim of fostering shared cultures and agendas towards awareness of appropriate lifestyles and CVD risk reduction. Also, Kim et al. [98] emphasized the appropriateness of a co-productive approach in designing health-related programmes. Their study explores the critical factors of co-design and co-development of a smartphone application aimed at stimulating migrant workers, with a sedentary lifestyle, to participate in physical training. The study shows that the involvement of users in the digital platform design not only improved their health but also enhanced their interaction, resulting in higher social inclusion. Moreover, Breeman et al. [104] confirmed that a multi-stakeholder designed eHealth intervention enhances healthy living in populations with CVD. This is mostly due to the consideration of stakeholders needs, preferences and abilities in digital e-platform development, which contributes in aligning the values of patients and providers in healthcare delivery. These points perfectly fit with the work of Geelen et al. [105] whose results prove that, in developing physical activity protocols for patients with long lengths of stay, the co-creation approach is successful and effective in improving muscle mass and strength and in reducing malnutrition and new limitations in daily activities.

*Second*, *interaction between the user and digital technology* is essential for the engagement and awareness of users. The case study from Monteiro-Guerra et al. [96] shows that physical training mobile applications are successfully used only if they can be personalized, thus allowing users to: (i) give/receive feedback; (ii) fix their own targets; (iii) set personal goals; (iv) trigger human interactions among users and clinicians; (v) adapt exercises to specific body needs; (vi) gather information from the surrounding context; and (vii) foster self-learning.

Furthermore, the *third* relevant point, which is concerned with co-production, is related to *the intelligibility and affordability of clinical data to users*. Sankaran et al. [100] stress the possibility, via m-health, of making the reasoning behind physical activities intelligible for enhancing health status a pre-requisite of every e-health intervention. Accordingly, Mercer et al. [103] underline that explaining the operation of wearable fitness trackers to elders could surely help them to become healthier thanks to physical exercise.

Finally, the *fourth* point relates to *gaming* [106]. Harris and Crone [97] demonstrated, by the case study of the “Beat the Street” app, that the gamification of physical training in group challenges contributes to the commitment of users and reduces barriers to participation in community-wide interventions based on motor activity. This research implies that interventions need to be simple to engage with; capable to facilitate social interaction, cooperation, and encourage outdoor activity [107]; and provide a degree of healthy competition, where possible.

The four endpoints of our study also refer to the accessibility of healthcare. According to Chehade et al. [108], innovative applications particularly improve access to musculoskeletal care and rehabilitation by supporting the monitoring of physical activities. As a result, the engagement of users in physical activities, via digital technologies, improve health. Su and Yu [99], in a cardiac rehabilitation case study, show that eHealth protocols, with nursing coaching, foster the change of lifestyle behaviour of patients towards exercise, diet, and quitting smoking. Moreover, Maxwell et al. [95] confirm that the employment of digital tools in the healthcare pathways of patients directly fosters motivation to exercise and stimulates awareness about the effectiveness of PASA on personal wellbeing and health. Simply, digitalization of healthcare improves outcomes [109, 110]. Accordingly, Zhang et al. [101] demonstrated that the use of a smartphone app for patient coaching towards better lifestyle behaviours is a convenient, affordable, and accessible methodology to spread culture and examples about quality of life. In the same direction, the review by McMahon et al. [111] highlights how an eHealth approach reduces clinician bias in the evaluation of CVD risk. The study also reports a moderate improvement in physical activity and diet towards a better lifestyle in patients whose healthcare were steered by telemedicine arms.

## Discussion

This literature analysis provides a comprehensive picture about CVD prevention through the implementation of PASA with the support of m-health tracking and monitoring applications in the healthcare sector. It confirms that the issue of using PASA as a support for the prevention of CVD risk has grown exponentially in the last 20 years [111], following technological advancement [63]; our results show specifically that the use of digital tools as a booster for carrying out PASA is recognized as effective and able to stimulate physical exercise for clinical-therapeutic purposes worldwide [105, 110]. If properly implemented, digital tools (m-health, e-health, and wellness apps) allow monitoring of the user during PASA and can also promote its continuity in daily activities over time, contributing to preventing the high widespread dropout rate of PASA as a therapeutic prescription, which is especially high in adults and the elderly [112].

Nevertheless, apart from confirming both the magnitude and effectiveness of the investigated theme, our study emphasizes other relevant factors. Its innovative aspect lays in being properly framed as a scoping review that brings together, for the first time, a comprehensive state of the art picture in the different scientific fields involved in this research.

By analysing the different steps of our research (from the performance of the quantitative analysis to the conceptual analysis), we draw several insights to contribute to the work of both academic and health policy practitioners.

Firstly, high-income countries, which mainly finance healthcare through private (and often mandatory) insurances, are those that create greater scientific production about the positive impacts of PASA as a safeguard of health. These countries suffer more from the impact of CVD with obesity-related pathologies [68, 69] and related costs [71]. The analysis seems to reaffirm, also in this specific field, Porter’s 2008 statements. The author indeed affirmed, referring to Western countries, “*Today, we are trying to deliver 21st-century medicines with 19th-century organizational structures, management processes, and measurement systems”* ([113] p. 503). With his theory about value based healthcare [64], the American author criticizes those healthcare systems that have focused too much on delivering in-hospital care (reimbursed by payers) to the detriment of services with higher value-added for patients (i.e., home care and remote monitoring). On this wave, since the last decade, Western countries seemed to be pushed into looking for sustainable strategies for providing healthcare to patients outside hospital institutions and clinics. This is confirmed by the widespread implementation of telemedicine policies aimed at delocalizing healthcare and increasing access to services [114].

Thus, the theme of the m-health based implementation of PASA towards CVD prevention requires high interdisciplinarity to be properly investigated. High inferences and connections were recognized between medical and technical disciplines [77]. Nevertheless, the promotion of PASA as a vehicle for “wellbeing” and “healthy lifestyle” appears to be an issue still underinvestigated at levels of public policy and public management [83].

Despite the evident increasing interest upon the issue, however, our analysis shows that the field remains fragmented in the investigations of its different components, as shown in the analysis of documents by source and especially in the one upon centrality and density. The absence of motor themes able to encompass all the different components on which researchers are deepening their analyses appears evident.

Accordingly, the most relevant insight of this work emerges from the analysis of the different possible links and interconnections. More specifically, there would seem to be an implicit recognition of the link between the carrying out of PASA and the expectation of the economic sustainability of healthcare, especially in those aspects related to the cardiovascular and nutrition spheres [90, 115]. This link is based on the use of e-health and m-health tools capable of measuring and managing physical performance in the perspective of achievement of health goals [88].

This aspect is proven both by the keywords co-occurrence analysis and by the co-citation analysis among sources. The investigations show that technology represents a lever that makes possible the effectiveness of the prescription of PASA for therapeutic purposes [108], especially as preventive strategies for people at risk for CVD.

The real critical issue arising in our work, however, can be summarized by the following statement:

*How users (patients) might be pushed to employ digital technology in order to support the monitoring of their PASA for achievement of clinical outcomes*.

The commitment and active participation of patients in healthcare is a prerequisite for obtaining high adherence to training protocols (as it happens for medication adherence [116]). This is valid both for telerehabilitation interventions [100] and, generally, for healthcare campaigns [117].

In order to obtain this result, however, public agencies should be able to engage with citizens by communicating to them the value of PASA on health policy interventions. Public agencies should foster, by health and social policies, the behavioural change [91] of patients towards their co-production of services. Accordingly, Jennings et al. [102] highlight that the success of a programme aimed at fostering wellbeing, by monitoring physical activities through mobile apps, is based on users receiving the correct information about healthy lifestyles, which is aimed at increasing the literacy of patients. This can be obtained through public communication campaigns that enhance both patient participation and awareness about usage of the app and expected health outcomes.

From the academia point of view, our quantitative investigation highlights a polarization of the disciplines within the literature. There are the following clear links between, respectively:

- PASA and prevention of cardiovascular risks.
- CVD prevention and m-health for clinical monitoring.
- PASA and m-health for monitoring performing the activities and the accuracy of gestures.

Nevertheless, no strong connection was noted among the three themes (CVD, PASA, and m-health) together. This explains the necessity of the conceptual analyses of further selected documents; these showed that the only interconnection among previous themes is possible through policies and strategies for patient involvement, which makes them a co-producer of the service.

Indeed, if we look at the evaluation of the scientific contributions selected for the conceptual analysis, we note that there is never, simultaneously, a high score (e.g., 4-5) for all of the three themes: PASA, CVD, and m-health, as depicted by the above framed links. Particularly, m-health becomes the “connecting” element that makes the prescription of PASA in patients with CVD an effective preventive factor, but the only theme that has a high score in all the selected documents is co-production. This means that the extracted literature is recognized in the co-production sphere, linking the involvement of patients in m-health usage in order to foster adherence in carrying out PASA towards CVD prevention.

**Fig. 11:**
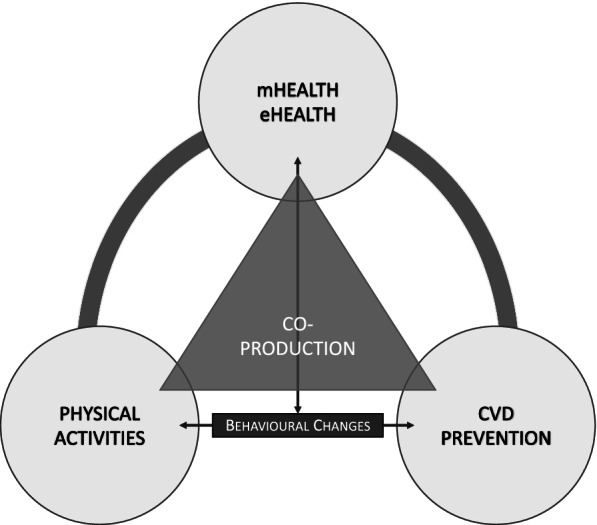
Connections among subjects: towards co-production policies for CVD prevention through physical activity. Source: Authors’ elaboration

Figure 11 explains this finding of the study, which highlights the necessity to move further research towards co-production policies. This aspect is relevant both at the academic and, especially, policy levels. In particular, we highlight that, in CVD prevention, while the link between the subjects of motor activity, medicine and technology is clear, there is not yet any deep investigation upon the connection with the ways citizens are involved in the service delivery process [118].

In our representation, an extension of the research upon co-production is necessary. This might represent the motor theme able to encompass all the different investigated components that is still lacking in the results of our quantitative and performance analyses.

According to Fusco et al. [33], the issue concerning how “value co-creation” [119] can effectively be applied by public agencies in order to explain the dynamics of co-production in the public sector is still underinvestigated.

Co-production is a system coordination method, which is still lacking public policies to foster public value towards physical activity as cultural leverage for wellbeing and healthy living and not only professional sport performance.

Our results appear to be in line with these statements, highlighting an ambivalence in dealing with the issue. On one hand, there is, always (independently from which of the above reported links is considered) the recognition of the importance of co-production. On the other hand, there is a low investigation on its relevance.

As a matter of fact, we can highlight that, when adding the 6th keyword string in Scopus, our sample changed from 2,295 to 120 documents, with as many as 84 of these being published in the last 5 years. This means that the issue is still underinvestigated, but its relevance is growing.

Co-production is relevant because, as stated since our background analysis, initiatives aiming at behavioural changes [91] are effective only if developed at a wider community level. Moreover, most of our reviewed articles showed the necessity of people commitment and the relevance of e-tools as supporting instruments.

In synthesis, our investigation highlights the need for more consumer engagement and collaborative patient-provider relationships [120]. The increasing analysis on the role of co-production in fostering PASA to prevent CVD using e-tools is, in this regard, promising but absolutely insufficient. Our review indicates the presence of several interesting and promising pilot studies, but there has not yet been any change of pace. In most of the cases, patients are still seen as passive recipients of care, while the future, especially for the effectiveness of preventive initiatives, is their involvement as active co-creators of their healthcare service experiences [121] because engaged patients achieve higher success from their healthcare experiences [122]. A pivotal theme regards how consumers can take control of their healthcare service experiences [123]. In this sense, our qualitative conceptual analysis showed that m-health improves outcomes; a simple proper use of specific apps can lead to better lifestyle behaviours, thus representing a convenient, affordable, and accessible methodology to spread culture and examples about quality of life.

Nevertheless, the therapeutic prescription of PASA, monitored by m-health, is not so common worldwide [77]. Accordingly, as shown in the 1 section, inactivity rates are growing in Western countries, even among youngsters. Therefore, it seems that the demonstrated effectiveness of the different numerous pilots, which have been tested in several countries, has not yet led to any structural changes in healthcare services to transform them into user-friendly ones [124–126]. Services remain complex and confusing to consumers who do not know explicitly ‘how’ to engage in these services [127] and must often be guided extensively by service providers [128].

On the contrary, the best medical outcomes occur when consumers comply with provider requests [120], and our research highlights not only the relevance of m-health as a stimulus for the participation of consumers [129] but also that it is not sufficient for assuring a long-term commitment to behavioural change.

Therefore, it is fundamental to identify how healthcare service designers and providers can learn from consumers about the technical and functional aspects of healthcare services that are important to them [130]. That is why our results highlight the relevance of initiatives where consumers engage and co-create the service process to foster PASA to prevent CVD, because consumer engagement is critical to the success of such complex, long-term effective preventive service experiences in healthcare [122, 131, 132].

To improve service success, consumers should participate by sharing information, providing input and suggestions, and engaging in shared decision making [131]. Our extensive literature review indicates the use of m-health to foster PASA for CVD prevention, especially consumer involvement, although defined as fundamental, is still underinvestigated.

## Conclusions

Even though some clear indications arise from the analysis of the results of the systematic review we conducted, the study has some limitations. A first limitation regards the access to the data and dataset; in this work, we referred to the sole Scopus research engine. Although some significant studies might have been outside that dataset, we believe that Scopus is among the most complete datasets to identify a large section of articles for managerial research applied to interdisciplinary fields, according to several scholars addressing recent literature studies [133–135]. Another limitation concerns with the fact the keywords search did not include an explicit connection to the sphere of management, with the consequence to potentially miss significant sources. Nevertheless, “manag*” word exclusion has prevented from influencing the results of the enquiry. Thus, managerial implications of PASA-based strategies for CVD prevention were the expected findings of the bibliometric literature review, with the aim to understand how to support public agencies to the complex challenge of caring chronic disease by new interventions. Moreover, managerial implication at public governance level have been recovered within the conceptual analysis of the literature by the addition of the sixth keyword, concerning co-production and its relation with policies.

A further limitation, concerning the paper selection, is due to the fact that we had to limit our research to English written publications, and this might have given no access to some interesting contributions. For instance, in the conceptual analysis, we excluded one of the selected articles after the English abstract screening, because the full text was only in Slovene. Moreover, no grey literature was considered for this review. This choice was principally steered by the reason that the specific field of inquiry, as demonstrated, is still underinvestigated; thus, insights from non-peer-reviewed sources or practical documents might not be non-reliable for the aims of this paper at this exploratory stage of investigation.

A last limitation concerns the internal coding we adopted in the double-blind scoring of the assessment of abstracts for the inclusion of documents for the conceptual analysis. Although based on a rigorous scoring process (extracted by the adaptation of previous research [59, 60] and agreeing upon common grading rules [61, 62]), this method is, in some sense, original and designed for our research aims; thus, it has not been tested in any previous research.

Nevertheless, this study gives some interesting indications both for future research and for health policy planning, as well as for practitioners. Designed to consider the presence (or absence) and the relevance of “interconnections” among different fields, our systematic literature review clearly showed the need for a greater interaction between research and policy planning. Indeed, while the link between the subjects of motor activity, medicine and technology is clear, the involvement of citizens in the service delivery process is still underinvestigated, especially the issue concerning how “value co-creation” could effectively be applied by public agencies [25, 136]. From the results of the analysis, the following insights are evident and useful both for future research and for designing future health policy planning.

Firstly, the study highlights the current relevance of the theme, which is confirmed by the exponential increase of studies in the last years. The geographical distribution shows that this relevance of the theme is higher in countries with greater pressure in reducing the healthcare costs of chronic diseases [71], especially if conceived in multi-payer financing systems [67].

Secondly, the theme of m-health based PASA implementation towards CVD prevention requires high interdisciplinarity to be properly investigated, as confirmed by the inferences and connections recognized among the different disciplines. Nevertheless, our analysis shows that the field remains fragmented in the investigations of its different components; most of all, its promotion as a vehicle for “wellbeing” and “healthy lifestyle” is still underinvestigated at the levels of public policy and public management.

Thirdly, the study reveals that co-production becomes a health policy lever that encourages patients in changing their habits towards their active involvement in healthcare processes aimed at CVD prevention.

Nevertheless, the analysis of the role of co-production as a system coordination method, which is so important in designing and implementing preventive care, is still lacking. This aspect should be considered not only at the academic level but also, and especially, at the policy planning level. Indeed, our findings report interesting trials and protocol testing, but there is not yet any systematic application at the generalized system level. The study by Harris and Crone [97], in fact, represents the only case of an application upon an entire territory of technology that enabled the engagement of citizens towards physical activities; nevertheless, the study is an experiment whose transmigration in actual general policy planning that has not yet appeared in the very near future. This transmigration in general policy planning appears even more important in the very near future, with the necessity to implement wellbeing measures that match with COVID-19 restrictions. Accordingly, the very recent protocol by Okop et al. [94] about m-health design has already given some indications that they had to change some aspects in order to avoid the risk of COVID-19 infection, which is also coherent with the interesting protocol foreseen by Su and Yu [99].

In synthesis, it appears that an actual full application, in a co-productive scheme, of m-health to promote PA to prevent CVD is not yet right around the corner. Nevertheless, the responsibilities are not anymore on the academic research, which has already shown the effectiveness of different protocols, it lies rather with health policy planning, which should now give more effort to create proper environments able to foster the validity of this preventive scheme.

To conclude, we shall remember that public services should be designed to obtain “*the maximum feasible participation of residents of the areas and members of the groups served*” ([137], p. 303). Thus, co-production schemes are involved in understanding how user’s participation can be “added into” the process of service planning and operating, with the aim of improving these services. This perspective creates values among users, which “*comprises their satisfaction with the service, the impact of the service experience upon their well-being and the extent to which it meets their social, health or economic needs*” ([40], p.643).

Particularly, from a public management standpoint, the involvement of patients in CVD co-prevention policies based on physical exercise means to trigger a conscious and voluntary act from the users [40] that concerns how to co-create capacity within service delivery systems and to co-design the service itself [138, 139]. Healthcare based on patient participation is expected to improve the performance of existing public services by also enhancing their sustainability [140]. By entering into the new sphere of experience of healthcare improvement, patients could make a transition from simple users and choosers to makers and shapers of health services [141, 142].

Therefore, how could public policies foster the transition of patients from passive to active subjects towards CVD prevention? Surely, this work showed that PASA, especially if tracked through m-health that is made by consumer-centred and consumer-driven tools [143], represents an effective strategy to reduce CVD risk and enhance the wellbeing of citizens. Nevertheless, giving the lack of structured and continuative experiences in the field, future research should analyse the aspects of managerial feasibility (both technical and operating) concerned with the design of widespread public health policies, addressing the prevention of most common diseases (e.g., CVD and chronic diseases) through active patient involvement, thanks to their behavioural changes supported by the availability of new digital technologies.

## Supplementary Information

**Additional file 1.** List of dataset papers for quantitative bibliometric analysis.

**Additional file 2.** List of dataset papers for qualitative conceptual analysis.

## Data Availability

All data generated or analysed during this study are included in this published article (and its supplementary information files).

## Abbreviations

CVD: Cardiovascular Disease
PASA: Physical Amateur Sport Activity
GPS/GIS: Global Positioning System/ Geographic Information System
PPI: Patient and Public Involvement
ICT: Information and Communication Technology
KW: Keyword
