# Supplementary material for: Mobile-Health based physical activities co-production policies towards cardiovascular diseases prevention: findings from a mixed-method systematic review
Source: BMC Health Serv Res. 2022 Mar 1;22:277. doi: 10.1186/s12913-022-07637-8 (PMC8886562; doi:10.1186/s12913-022-07637-8)
Supplement: Supplementary file 2 — Additional file 2. List of dataset papers for qualitative conceptual analysis. [file 12913_2022_7637_MOESM2_ESM.pdf]

Yellow colored numbers indicate papers included into the narrative content analysis	
**Colonna1**	**Colonna2**
	Authors,Title,Year,Link,Abstract,Author B5
**1**	Okop K.J., Murphy K., Lambert E.V., Kedir K., Getachew H., Howe R., Niyibizi J.B., Ntawuyirushintege S., Bavuma C., Rulisa S., Kasenda S., Chipeta E., Bunn C., Crampin A.C., Chapotera G., King A.C., Banchoff A., Winter S.J., Levitt N.S., "**Community-driven citizen science approach to explore cardiovascular disease risk perception, and develop prevention advocacy strategies in sub-Saharan Africa: a programme protocol**",2021,"https://www.scopus.com/inward/record.uri?eid=2-s2.0-85101862235&doi=10.1186%2fs40900-020-00246-x&partnerID=40&md5=490451af0501f2f4633954f0ceb6bb39", "Background: In sub-Saharan Africa (SSA), which experiences a disproportionately high cardiovascular disease (CVD) burden, population-based screening and prevention measures are hampered by low levels of knowledge about CVD and associated risk factors, and inaccurate perceptions of severity of risk. Methods: This protocol describes the planned processes for implementing community-driven participatory research, using a citizen science method to explore CVD risk perceptions and to develop community-specific advocacy and prevention strategies in the rural and urban SSA settings. Multi-disciplinary research teams in four selected African countries will engage with and train community members living in rural and urban communities as citizen scientists to facilitate conceptualization, co-designing of research, data gathering, and co-creation of knowledge that can lead to a shared agenda to support collaborative participation in community-engaged science. The emphasis is on robust community engagement, using mobile technology to support data gathering, participatory learning, and co-creation of knowledge and disease prevention advocacy. Discussion: Contextual processes applied and lessons learned in specific settings will support redefining or disassembling boundaries in participatory science to foster effective implementation of sustainable prevention intervention programmes in Low- and Middle-income countries. © 2021, The Author(s).","Advocacy
**2**	Wang Z., Li C., Huang W., Chen Y., Li Y., Huang L., Zhang M., Wu D., Wang L., Duan H., An J., Deng N.,"**Effectiveness of a pathway-driven eHealth-based integrated care model (PEICM) for community-based hypertension management in China: study protocol for a randomized controlled trial**",**2021**,"https://www.scopus.com/inward/record.uri?eid=2-s2.0-85099738242&doi=10.1186%2fs13063-021-05020-2&partnerID=40&md5=4e4ae8fc936ea52289c7d434ee52ee99", "Background: The prevalence of hypertension is high and increasing in China in recent years. The treatment and control of hypertension calls for long-term management beyond hospital, which is hard to implement in traditional care settings. Integrated care combined with information technology can promote high-quality healthcare services across the life-course. However, few studies have applied a customized integrated care model in community-based hypertension management in China, catering to the emerging “three-manager” mode. This study aims to identify the effectiveness of a pathway-driven eHealth-based integrated model that implemented as a full-featured telehealth system to facilitate standardized management of hypertension in China. Methods: The trial has been designed as a 1-year, non-blinded superiority trial with two parallel groups. A total of 402 hypertensive patients who meet the eligibility criteria will be recruited and randomized with a 1:1 allocation. All the participants will receive a mobile device for self-management, which is a part of our telehealth system. Participants in the control group will only use the device for BP measurement and receive regular follow-ups from care providers according to the guidelines. Participants in the intervention group will gain full access to the system and receive intervention based on the proposed model (a well-designed coordinated care pathway consisting of 9 tasks). Outcomes will be measured mainly on three occasions (at inclusion, at 6 months, and at 12 months). The primary outcome is mean change in systolic blood pressure over a 12-month period. Secondary outcomes include changes in diastolic blood pressure, biochemical indexes related to hypertension, lifestyles, self-management adherence, and hypertension awareness, as well as work efficiency of care providers. Discussion: This study aims to investigate whether a pathway-driven eHealth-based integrated care model based on the “three-manager” mode will improve hypertension control in China. Success of the model would help improve the quality of present community-based management procedures and benefit more patients with uncontrolled hypertension. Trial registration: Chinese Clinical Trial Registry ChiCTR1900027645. Registered on November 22, 2019. © 2021, The Author(s).","Community
**3**	Strozzi M.M., Varrica A., Colivicchi M., Pelazzo C., Negri R., Galante A., Ianniello P., Sterpone R., Nannini P., Leo D., Mannarino F., Striani M., Montani S., Gazzolo D.,"**High-Fidelity simulation-based program improves flow state scale in the perinatal team**",**2021**,"https://www.scopus.com/inward/record.uri?eid=2-s2.0-85101709323&doi=10.1186%2fs13052-021-00972-2&partnerID=40&md5=a419ede5af024fd29d652b6d9cdb4451", "Background: We aimed to evaluate the degree of realism and involvement, stress management and awareness of performance improvement in practitioners taking part in high fidelity simulation (HFS) training program for delivery room (DR) management, by means of a self-report test such as flow state scale (FSS). Methods: This is an observational pretest-test study. Between March 2016 and May 2019, fourty-three practitioners (physicians, midwives, nurses) grouped in multidisciplinary teams were admitted to our training High Fidelity Simulation center. In a time-period of 1 month, practitioners attended two HFS courses (model 1, 2) focusing on DR management and resuscitation maneuvers. FSS test was administred at the end of M1 and M2 course, respectively. Results: FSS scale items such as unambiguous feed-back, loss of self consciousness and loss of time reality, merging of action and awareness significantly improved (P < 0.05, for all) between M1 and M2. Conclusions: The present results showing the high level of practitioner involvement during DR management-based HFS courses support the usefulness of HFS as a trustworthy tool for improving the awareness of practitioner performances and feed-back. The data open the way to the usefulness of FSS as a trustworthy tool for the evaluation of the efficacy of training programs in a multidisciplinary team. © 2021, The Author(s).","Delivery room
**4**	Page-Reeves J., Murray-Krezan C., Regino L., Perez J., Bleecker M., Perez D., Wagner B., Tigert S., Bearer E.L., Willging C.E.,"**A randomized control trial to test a peer support group approach for reducing social isolation and depression among female Mexican immigrants**",**2021**,"https://www.scopus.com/inward/record.uri?eid=2-s2.0-85099236925&doi=10.1186%2fs12889-020-09867-z&partnerID=40&md5=c712797e524ed8414cbf4904d2124854", "Background: Female Mexican Immigrants (FMIs) experience high rates of depression compared with other populations. For this population, depression is often exacerbated by social isolation associated with the experience of immigration. Aim 1. To measure whether a culturally situated peer group intervention will reduce depression and stress associated with the experience of immigration. Aim 2. To test whether an intervention using a “women’s funds of knowledge” approach results in improved resilience, knowledge and empowerment. Aim 3. To investigate whether a culturally situated peer group intervention using a women’s funds of knowledge approach can give participants a sense and experience of social and physical connection (“emplacement”) that is lost in the process of immigration. Methods: This mixed-methods study will implement “Tertulias” (“conversational gatherings” in Spanish), a peer support group intervention designed to improve health outcomes for FMI participants in Albuquerque, New Mexico. We will document results of the intervention on our primary hypotheses of a decrease in depression, and increases in resilience and social support, as well as on our secondary hypotheses of decreased stress (including testing of hair cortisol as a biomarker for chronic stress), and an increase in social connectedness and positive assessment of knowledge and empowerment. Discussion: This project will address mental health disparities in an underserved population that experiences high rates of social isolation. Successful completion of this project will demonstrate that health challenges that may appear too complex and too hard to address can be using a multi-level, holistic approach. Our use of hair samples to test for the 3-month average levels of systemic cortisol will contribute to the literature on an emerging biomarker for analyzing chronic stress. Trial registration: This study was registered with ClinicalTrials.gov on 2/3/20, Identifier #NCT04254198. © 2021, The Author(s).","Depression
**5**	Meltzer L.J., Williamson A.A., Mindell J.A.,"**Pediatric sleep health: It matters, and so does how we define it**",**2021**,"https://www.scopus.com/inward/record.uri?eid=2-s2.0-85101019037&doi=10.1016%2fj.smrv.2021.101425&partnerID=40&md5=c9c368d4e89e2e933a81ca860c86992d", "In 2014, Buysse published a novel definition of sleep health, raising awareness for the importance of this construct for individuals, populations, clinical care, and research. However, the original definition focused on adults, with the recommendation that it should be adapted for children and adolescents. As children live within a complex and dynamic system, and may not always have control over their own sleep, this theoretical review will examine and apply Buysse's five dimensions of sleep health within the context of pediatrics. In addition, using examples from the pediatric sleep literature we introduce a modified definition that takes into consideration the influence of the socio-ecological system within which children live, and the sleep-related behaviors that are critical in supporting or hindering sleep health. Finally, we discuss how the proposed theoretical framework, Peds B-SATED, can be applied to clinical practice, research, and training in the field of pediatric sleep. © 2021 Elsevier Ltd","Adolescents
**6**	Zahid A., Poulsen J.K., Sharma R., Wingreen S.C.,"**A systematic review of emerging information technologies for sustainable data-centric health-care**",2021,"https://www.scopus.com/inward/record.uri?eid=2-s2.0-85102066719&doi=10.1016%2fj.ijmedinf.2021.104420&partnerID=40&md5=c3a9012ffda190ac9aed2d85b367b740", "Background: Of the Sustainable Development Goals (SDGs), the third presents the opportunity for a predictive universal digital healthcare ecosystem, capable of informing early warning, assisting in risk reduction and guiding management of national and global health risks. However, in reality, the existing technology infrastructure of digital healthcare systems is insufficient, failing to satisfy current and future data needs. Objective: This paper systematically reviews emerging information technologies for data modelling and analytics that have potential to achieve Data-Centric Health-Care (DCHC) for the envisioned objective of sustainable healthcare. The goal of this review is to: 1) identify emerging information technologies with potential for data modelling and analytics, and 2) explore recent research of these technologies in DCHC. Findings: A total of 1619 relevant papers have been identified and analysed in this review. Of these, 69 were probed deeply. Our analysis found that the extant research focused on elder care, rehabilitation, chronic diseases, and healthcare service delivery. Use-cases of the emerging information technologies included providing assistance, monitoring, self-care and self-management, diagnosis, risk prediction, well-being awareness, personalized healthcare, and qualitative and/or quantitative service enhancement. Limitations identified in the studies included vendor hardware specificity, issues with user interface and usability, inadequate features, interoperability, scalability, and compatibility, unjustifiable costs and insufficient evaluation in terms of validation. Conclusion: Achievement of a predictive universal digital healthcare ecosystem in the current context is a challenge. State-of-the-art technologies demand user centric design, data privacy and protection measures, transparency, interoperability, scalability, and compatibility to achieve the SDG objective of sustainable healthcare by 2030. © 2021 Elsevier B.V.","Data analytics

7	Almarcha M., Balagué N., Torrents C.,"**Healthy teleworking: Towards personalized exercise recommendations**",2021,"https://www.scopus.com/inward/record.uri?eid=2-s2.0-85103108325&doi=10.3390%2fsu13063192&partnerID=40&md5=5d8fbe1e25a7740ea6deae70b99e1bfe","Home-based teleworking, associated with sedentary behavior, may impair self-reported adult health status. Current exercise recommendations, based on universal recipes, may be insufficient or even misleading to promote healthy teleworking. From the Network Physiology of Exercise perspective, health is redefined as an adaptive emergent state, product of dynamic interactions among multiple levels (from genetic to social) that cannot be reduced to a few dimensions. Under such a perspective, fitness development is focused on enhancing the individual functional diversity potential, which is better achieved through varied and personalized exercise proposals. This paper discusses some myths related to ideal or unique recommendations, like the ideal exercise or posture, and the contribution of recent computer technologies and applications for prescribing exercise and assessing fitness. Highlighting the need for creating personalized working environments and strengthening the active contribution of users in the process, new recommendations related to teleworking posture, home exercise counselling, exercise monitoring and to the roles of healthcare and exercise professionals are proposed. Instead of exercise prescribers, professionals act as co-designers that help users to learn, co-adapt and adequately contextualize exercise in order to promote their somatic awareness, job satisfaction, productivity, work–life balance, wellbeing and health. © 2021 by the authors. Licensee MDPI, Basel, Switzerland.","Affordances
8	Chatterjee A., Gerdes M., Prinz A., Martinez S.,"**Human coaching methodologies for automatic electronic coaching (eCoaching) as behavioral interventions with information and communication technology: Systematic review**",2021,"https://www.scopus.com/inward/record.uri?eid=2-s2.0-85103593582&doi=10.2196%2f23533&partnerID=40&md5=6ac6df2c746f2095bf3199e3fb7b65b6","Background: We systematically reviewed the literature on human coaching to identify different coaching processes as behavioral interventions and methods within those processes. We then reviewed how those identified coaching processes and the used methods can be utilized to improve an electronic coaching (eCoaching) process for the promotion of a healthy lifestyle with the support of information and communication technology (ICT). Objective: This study aimed to identify coaching and eCoaching processes as behavioral interventions and the methods behind these processes. Here, we mainly looked at processes (and corresponding models that describe coaching as certain processes) and the methods that were used within the different processes. Several methods will be part of multiple processes. Certain processes (or the corresponding models) will be applicable for both human coaching and eCoaching. Methods: We performed a systematic literature review to search the scientific databases EBSCOhost, Scopus, ACM, Nature, SpringerLink, IEEE Xplore, MDPI, Google Scholar, and PubMed for publications that included personal coaching (from 2000 to 2019) and persuasive eCoaching as behavioral interventions for a healthy lifestyle (from 2014 to 2019). The PRISMA (Preferred Reporting Items for Systematic Reviews and Meta-Analyses) framework was used for the evidence-based systematic review and meta-analysis. Results: The systematic search resulted in 79 publications, including 72 papers and seven books. Of these, 53 were related to behavioral interventions by eCoaching and the remaining 26 were related to human coaching. The most utilized persuasive eCoaching methods were personalization (n=19), interaction and cocreation (n=17), technology adoption for behavior change (n=17), goal setting and evaluation (n=16), persuasion (n=15), automation (n=14), and lifestyle change (n=14). The most relevant methods for human coaching were behavior (n=23), methodology (n=10), psychology (n=9), and mentoring (n=6). Here, ""n"" signifies the total number of articles where the respective method was identified. In this study, we focused on different coaching methods to understand the psychology, behavioral science, coaching philosophy, and essential coaching processes for effective coaching. We have discussed how we can integrate the obtained knowledge into the eCoaching process for healthy lifestyle management using ICT. We identified that knowledge, coaching skills, observation, interaction, ethics, trust, efficacy study, coaching experience, pragmatism, intervention, goal setting, and evaluation of coaching processes are relevant for eCoaching. Conclusions: This systematic literature review selected processes, associated methods, strengths, and limitations for behavioral interventions from established coaching models. The identified methods of coaching point toward integrating human psychology in eCoaching to develop effective intervention plans for healthy lifestyle management and overcome the existing limitations of human coaching. © 2021 Journal of Medical Internet Research. All rights reserved.","Coaching
9	Maxwell H., O'Shea M., Stronach M., Pearce S.,"**Empowerment through digital health trackers: an exploration of Indigenous Australian women and physical activity in leisure settings**",2021,"https://www.scopus.com/inward/record.uri?eid=2-s2.0-85074038587&doi=10.1080%2f11745398.2019.1674677&partnerID=40&md5=4d8dcca15e073078ac046454146d6d9a","Drawing on a strengths-based empowerment approach and Indigenous traditions of 'yarning', this research explores how digital health technologies might contribute to Indigenous Australian women's increased participation in physical activity in leisure settings. While people have long controlled their bodies, conventionally through diaries and weight scales, digital self-tracking of one's bodily states and activities continues to expand. No previous studies have addressed how these technologies influence physical activity among this population and there is limited research about Indigenous Australian people's leisure experiences and the meanings they attribute to them. Accordingly, this research contributes to an under researched area of health and leisure studies concerned with understanding the social, narrative and affective facets of individuals' practices and experiences. Key findings from the pilot study include intersections between the use of health trackers and Indigenous women's prioritizing time for leisure, increased physical activity, enhanced health literacy, and greater personal accountability for lifestyle choices. © 2019 Australia and New Zealand Association of Leisure Studies.","Aboriginal
10	Syrjälä M.B., Fharm E., Dempsey P.C., Nordendahl M., Wennberg P.,"**Reducing occupational sitting time in adults with type 2 diabetes: Qualitative experiences of an office-adapted mHealth intervention**",**2021**,"https://www.scopus.com/inward/record.uri?eid=2-s2.0-85099959754&doi=10.1111%2fdme.14514&partnerID=40&md5=0e536cd278026f59ecb53340d5f766e7",<br>"Aim: Understanding barriers and facilitators for limiting occupational sitting and what impact it has on health on those with type 2 diabetes is essential for future trials and intervention development in primary healthcare settings. This study aimed to explore the feasibility and acceptability of an intervention using mobile health (mHealth) technology, together with counselling by a diabetes specialist nurse, to reduce occupational sitting in adults with type 2 diabetes. Methods: Individual semi-structured interviews were conducted in 15 participants with type 2 diabetes who completed a 3-month intervention including mHealth<br>activity tracker (Garmin Vivofit3) and SMS reminders, one initial face-to-face patient-centred counselling session and three telephone follow-up calls by a diabetes specialist nurse within the primary healthcare system in Sweden. The interviews were recorded, transcribed verbatim and analysed using qualitative content analysis. Results: Two themes were identified: (1) 'From baby steps to milestones' reflecting three categories 'Small changes make it easier to reduce sitting', 'Encouraged by trustworthy coaching', 'Physical and mental rewards matter' and (2) 'Tailoring strategies that fit me and my workplace' reflecting four categories 'It's up to me', 'Taking advantage of the support', 'Using creativity to find practical solutions for interrupting sitting' and 'Living up to expectations'. Conclusion: The intervention was perceived as feasible and acceptable in different office workplaces, and led to increased awareness of sedentary behaviour in adults with type 2 diabetes. Stepwise goal setting together with personalization of the mHealth intervention should be emphasized in individual type 2 diabetes programmes aiming to reduce workplace sitting. © 2021 The Authors. Diabetic Medicine published by John Wiley & Sons Ltd on behalf of Diabetes UK.","activity tracker
11	Niknejad N., Ismail W., Bahari M., Nazari B.,"**Understanding Telerehabilitation Technology to Evaluate Stakeholders' Adoption of Telerehabilitation Services: A Systematic Literature Review and Directions for Further Research**",**2021**,"https://www.scopus.com/inward/record.uri?eid=2-s2.0-85101175050&doi=10.1016%2fj.apmr.2020.12.014&partnerID=40&md5=30da55067f49d6796877d77b86b3725e",<br>"Objectives: To examine the adoption of telerehabilitation services from the stakeholders' perspective and to investigate recent advances and future challenges. Data Sources: A systematic review of English articles indexed by PubMed, Thomson Institute of Scientific Information's Web of Science, and Elsevier's Scopus between 1998 and 2020. Study Selection: The first author (N.N.) screened all titles and abstracts based on the eligibility criteria. Experimental and empirical articles such as randomized and nonrandomized controlled trials, pre-experimental studies, case studies, surveys, feasibility studies, qualitative descriptive studies, and cohort studies were all included in this review. Data Extraction: The first, second, and fourth authors (N.N., W.I., B.N.) independently extracted data using data fields predefined by the third author (M.B.). The data extracted through this review included study objective, study design, purpose of telerehabilitation, telerehabilitation equipment, patient/sample, age, disease, data collection methods, theory/framework, and adoption themes. Data Synthesis: A telerehabilitation adoption process model was proposed to highlight the significance of the readiness stage and to classify the primary studies. The articles were classified based on 6 adoption themes, namely users' perception, perspective, and experience<br>and users' motivation and awareness. Results: A total of 133 of 914 articles met the eligibility criteria. The majority of papers were randomized controlled trials (27%), followed by surveys (15%). Almost 49% of the papers examined the use of telerehabilitation technology in patients with nervous system problems, 23% examined physical disability disorders, 10% examined cardiovascular diseases, and 8% inspected pulmonary diseases. Conclusion: Research on the adoption of telerehabilitation is still in its infancy and needs further attention from researchers working in health care, especially in resource-limited countries. Indeed, studies on the adoption of telerehabilitation are essential to minimize implementation failure, as these studies will help to inform health care personnel and clients about successful adoption strategies. © 2021 American Congress of Rehabilitation Medicine","Patient satisfaction
12	Gaudet L.,"**The piety of optimization: The rhetoric of health awareness in ParticipACTION and Fitbit**",**2021**,"https://www.scopus.com/inward/record.uri?eid=2-s2.0-85100469248&doi=10.1177%2f1363459320988886&partnerID=40&md5=500cf17da751e1c85047736bf092467b",<br>"This article uses the tools of rhetorical study to investigate how health awareness, as both a concept and a set of beliefs that reinforce ideals of health, permeates everyday life and affects ways of being. I explore how health awareness is communicated through both public health and commercial marketing campaigns, and argue that as the sources of information change, so too do the ideas of health that we are asked to be aware of. Through an analysis of the websites of ParticipACTION, a publicly funded health and fitness campaign, and Fitbit, a corporation that produces wearable technologies, I show that these organizations provide their audiences with instructions for self-conduct in the pursuit of health through the piety that time is a resource to be managed. Through this piety, ParticipACTION and Fitbit's websites each reify an altar of health where health is represented as a socially and physically fitter (optimized) self, always just out of reach and attainable in the future. I conclude with a call for critical descriptions of health awareness to move beyond the explanatory power of neoliberalization of health, and turn to the work of Rachel Sanders, Annmarie Mol, and Donna Haraway as possible avenues for resisting optimization. © The Author(s) 2021.","Fitbit

**13**	Zaharieva D.P., Addala A., Simmons K.M., Maahs D.M.,"**Weight Management in Youth with Type 1 Diabetes and Obesity: Challenges and Possible Solutions**",**2020**,"https://www.scopus.com/inward/record.uri?eid=2-s2.0-85094127651&doi=10.1007%2fs13679-020-00411-z&partnerID=40&md5=9ecf7453e7f45b58d4502737f0b643a7", "Purpose of Review: This review highlights challenges associated with weight management in children and adolescents with type 1 diabetes (T1D). Our purpose is to propose potential solutions to improve weight outcomes in youth with T1D. Recent Findings: A common barrier to weight management in T1D is reluctance to engage in exercise for fear of hypoglycemia. Healthcare practitioners generally provide limited guidance for insulin dosing and carbohydrate modifications to maintain stable glycemia during exercise. Adherence to dietary guidelines is associated with improved glycemia however, youth struggle to meet recommendations. When psychosocial factors are addressed in combination with glucose trends, this often leads to successful T1D management. Newer medications also hold promise to potentially aid in glycemia and weight management, but further research is necessary. Summary: Properly addressing physical activity, nutrition, pharmacotherapy, and psychosocial factors while emphasizing weight management may reduce the likelihood of obesity development and its perpetuation in this population. © 2020, Springer Science+Business Media, LLC, part of Springer Nature.","Exercise however, youth struggle to meet recommendations. When psychosocial factors are addressed in combination with glucose trends, this often leads to successful T1D management. Newer medications also hold promise to potentially aid in glycemia and weight management, but further research is necessary. Summary: Properly addressing physical activity, nutrition, pharmacotherapy, and psychosocial factors while emphasizing weight management may reduce the likelihood of obesity development and its perpetuation in this population. © 2020, Springer Science+Business Media, LLC, part of Springer Nature.","Exercise
**14**	Anirudh B.V.M., Mohanraj K.G., Nivethigaa B.,"**Awareness on sedentary lifestyle, obesity and cardiovascular diseases among undergraduate student population**",**2020**,"https://www.scopus.com/inward/record.uri?eid=2-s2.0-85096921459&partnerID=40&md5=9ceec11e72e2fa37f36d00233e7432df", "Sedentary lifestyle has become a huge concern in one's life, in which physical inactivity has become a major health problem. Prevalence of sedentary life is increasing nowadays and is seen more commonly in the student population. Sedentary lifestyle leads to obesity is an important health concern which is increasing day by day around the globe. It affects the whole body which mainly causes cardiovascular problems.The Aim of this research is to bring awareness about sedentary lifestyle, obesity and cardiovascular disease in the student population.A questionnaire of a total 15 questions were prepared and was uploaded through surveyplanet. The responses were collected and then interpreted as pie charts and bar graphs. A total of 100 undergraduate college students have taken the survey. The data was analysed by IBM-SPSS software where descriptive statistics and chi square test was done, where the results are plotted as graphs and pie charts.A total of 100 college students have taken up the survey in which 47% were male and 53% were female. In a question pertaining to how many people were following a healthy diet- 74 % have answered yes and 26% said no. The next question was about exercising everyday in which 62 % said yes while 38% said no. In which 47% males and 15% females have answered yes.97% agree that sedentary lifestyle leads to complications such as obesity. 90% of the students are aware about the increase in sedentary lifestyle among students. 69% have answered that unhealthy diet, lack of physical activity,smoking etc were the common causes of obesity. 95% agree that obesity causes respiratory problems. Almost 95 % students agree that obesity causes systemic diseases such as diabetes and hypertension which will ultimately lead to cardiovascular problems.Chi square test was done between gender and people who are following a healthy diet and exercising daily where p<0.05 which is statistically significant.Our study showed that almost all students were aware about sedentary lifestyle and its complications, but the females in the study have shown that they do not follow a healthy diet and do not exercise regularly which makes them prone to obesity and ultimately cardiovascular problems. To avoid these problems, awareness has to be spread in colleges about the seriousness of obesity and its harmful effects and how to prevent it. © 2020 Ubiquity Press. All rights reserved.","Awareness
**15**	Fang E.F., Xie C., Schenkel J.A., Wu C., Long Q., Cui H., Aman Y., Frank J., Liao J., Zou H., Wang N.Y., Wu J., Liu X., Li T., Fang Y., Niu Z., Yang G., Hong J., Wang Q., Chen G., Li J., Chen H.-Z., Kang L., Su H., Gilmour B.C., Zhu X., Jiang H., He N., Tao J., Leng S.X., Tong T., Woo J.,"**A research agenda for ageing in China in the 21st century (2nd edition): Focusing on basic and translational research, long-term care, policy and social networks**",**2020**,"https://www.scopus.com/inward/record.uri?eid=2-s2.0-85091870837&doi=10.1016%2fj.arr.2020.101174&partnerID=40&md5=ab1fecdf2054a153668e3d92bcf7b41d", "One of the key issues facing public healthcare is the global trend of an increasingly ageing society which continues to present policy makers and caregivers with formidable healthcare and socio-economic challenges. Ageing is the primary contributor to a broad spectrum of chronic disorders all associated with a lower quality of life in the elderly. In 2019, the Chinese population constituted 18 % of the world population, with 164.5 million Chinese citizens aged 65 and above (65+), and 26 million aged 80 or above (80+). China has become an ageing society, and as it continues to age it will continue to exacerbate the burden borne by current family and public healthcare systems. Major healthcare challenges involved with caring for the elderly in China include the management of chronic non-communicable diseases (CNCDs), physical frailty, neurodegenerative diseases, cardiovascular diseases, with emerging challenges such as providing sufficient dental care, combating the rising prevalence of sexually transmitted diseases among nursing home communities, providing support for increased incidences of immune diseases, and the growing necessity to provide palliative care for the elderly. At the governmental level, it is necessary to make long-term strategic plans to respond to the pressures of an ageing society, especially to establish a nationwide, affordable, annual health check system to facilitate early diagnosis and provide access to affordable treatments. China has begun work on several activities to address these issues including the recent completion of the of the Ten-year Health-Care Reform project, the implementation of the Healthy China 2030 Action Plan, and the opening of the National Clinical Research Center for Geriatric Disorders. There are also societal challenges, namely the shift from an extended family system in which the younger provide home care for their elderly family members, to the current trend in which young people are increasingly migrating towards major cities for work, increasing reliance on nursing homes to compensate, especially following the outcomes of the 'one child policy' and the 'empty-nest elderly' phenomenon. At the individual level, it is important to provide avenues for people to seek and improve their own knowledge of health and disease, to encourage them to seek medical check-ups to prevent/manage illness, and to find ways to promote modifiable health-related behaviors (social activity, exercise, healthy diets, reasonable diet supplements) to enable healthier, happier, longer, and more productive lives in the elderly. Finally, at the technological or treatment level, there is a focus on modern technologies to counteract the negative effects of ageing. Researchers are striving to produce drugs that can mimic the effects of 'exercising more, eating less', while other anti-ageing molecules from molecular gerontologists could help to improve 'healthspan' in the elderly. Machine learning, 'Big Data', and other novel technologies can also be used to monitor disease patterns at the population level and may be used to inform policy design in the future. Collectively, synergies across disciplines on policies, geriatric care, drug development, personal awareness, the use of big data, machine learning and personalized medicine will transform China into a country that enables the most for its elderly, maximizing and celebrating their longevity in the coming decades. This is the 2nd edition of the review paper (Fang EF et al., Ageing Re. Rev. 2015). © 2020 Elsevier B.V.","Ageing policy
**16**	Topothai T., Suphanchaimat R., Tangcharoensathien V., Putthasri W., Sukaew T., Asawutmangkul U., Topothai C., Piancharoen P., Piyathawornanan C.,"**Daily step counts from the first thailand national steps challenge in 2020: A cross-sectional study**",**2020**,"https://www.scopus.com/inward/record.uri?eid=2-s2.0-85096206515&doi=10.3390%2fijerph17228433&partnerID=40&md5=05db5d2db1860963cbd8c9ecafc85562", "Thailand's first national steps challenge has been implemented in 2020 with the goal to raise the level of physical activity nationwide by monitoring achievements through a smartphone application. This study examined the daily step counts of participants in the first national steps challenge. Six data points from 186,653 valid participants were retrieved and analyzed in five periods using Poisson regression. The mean daily steps peaked at 3196 in Period 1, and steadily dropped to 1205 in Period 5. The daily steps per period were analyzed using the participants' characteristics, such as the type of participant, sex, age, body mass index, and area of residence. The overall mean daily steps of the participants meant physical activity was far below the recommended level and tended to drop in later periods. The general population achieved significantly higher mean daily steps than public health officers or village health volunteers (24.0% by multivariate analysis). Participants who were female, younger (<45 years), obese (body mass index > 30), and living in rural areas had fewer mean daily steps (13.8%, 44.3%, 12.7%, and 14.7% by multivariate analysis, respectively), with statistical significance. In the future, the national steps challenge should be continuously implemented by counting all steps throughout a day, using more strategies to draw attention and raise motivation, advocating for more participants, as well as reporting the whole day step counts instead of distance. © 2020, MDPI AG. All rights reserved.","Intervention
**17**	Middleton M., Somerset S., Evans C., Blake H.,"**Test@work texts: Mobile phone messaging to increase awareness of HIV and HIV testing in UK construction employees during the COVID-19 pandemic**",**2020**,"https://www.scopus.com/inward/record.uri?eid=2-s2.0-85094208611&doi=10.3390%2fijerph17217819&partnerID=40&md5=a721bf78680fb5c33d851219d3f901ef", "Background: HIV poses a threat to global health. With effective treatment options available, education and testing strategies are essential in preventing transmission. Text messaging is an effective tool for health promotion and can be used to target higher risk populations. This study reports on the design, delivery and testing of a mobile text messaging SMS intervention for HIV prevention and awareness, aimed at adults in the construction industry and delivered during the COVID-19 pandemic. Method: Participants were recruited at Test@Work workplace health promotion events (21 sites, n = 464 employees), including health checks with HIV testing. Message development was based on a participatory design and included a focus group (n = 9) and message fidelity testing (n = 291) with assessment of intervention uptake, reach, acceptability, and engagement. Barriers to HIV testing were identified and mapped to the COM-B behavioural model. 23 one-way push SMS messages (19 included short web links) were generated and fidelity tested, then sent via automated SMS to two employee cohorts over a 10-week period during the COVID-19 pandemic. Engagement metrics measured were: opt-outs, SMS delivered/read, number of clicks per web link, four two-way pull messages exploring repeat HIV testing, learning new information, perceived usefulness and behaviour change. Results: 291 people participated (68.3% of eligible attendees). A total of 7726 messages were sent between March and June 2020, with 91.6% successfully delivered (100% read). 12.4% of participants opted out over 10 weeks. Of delivered messages, links were clicked an average of 14.4% times, max 24.1% for HIV related links. The number of clicks on web links declined over time (r = −6.24, p = 0.01). Response rate for two-way pull messages was 13.7% of participants. Since the workplace HIV test offer at recruitment, 21.6% reported having taken a further HIV test. Qualitative replies indicated behavioural influence of messaging on exercise, lifestyle behaviours and intention to HIV test. Conclusions: SMS messaging for HIV prevention and awareness is acceptable to adults in the construction industry, has high uptake, low attrition and good engagement with message content, when delivered during a global pandemic. Data collection methods may need refinement for audience, and effect of COVID-19 on results is yet to be understood. © 2020 by the authors. Licensee MDPI, Basel, Switzerland.","Construction

**18**	Ho T.-W., Tsai H.-H., Lai J.-F., Chu S.-M., Liao W.-C., Chiu H.-M.,"**Physical fitness cognition, assessment, and promotion: A cross-sectional study in Taiwan**",**2020**,"https://www.scopus.com/inward/record.uri?eid=2-s2.0-85092646464&doi=10.1371%2fjournal.pone.0240137&partnerID=40&md5=3dc5568efad05d2d124f95f0f7f3ef92",<br>"Introduction Many health organizations have promoted the importance of the health-related benefits of physical fitness and physical activity. Studies have evaluated effective public health practice aiming to understand the cognition of physical activity among youths and adolescents. However, studies investigating the level of cognition and knowledge of physical fitness among Asian adults are lacking. Purpose This study aimed to investigate the self-awareness level of physical fitness and exercise prescription and the demand for physical fitness assessment among Taiwanese adults. Methods In January-July 2019, a cross-sectional anonymous survey was conducted using Research Electronic Data Capture to gather data on demographic data, cognition investigation of physical fitness and exercise prescription, cognitive test of physical fitness and exercise prescription, and demand for physical fitness assessment. Results The questionnaire was answered by 200 respondents. The rating for cognition investigation of physical fitness was 2.63-3.13 (unclear to mostly clear) and for exercise prescription was 2.05-2.76 (unclear) (rated on a 5-point Likert scale). Results show that lack of awareness was highest for health-related physical fitness, exercise prescription, and exercise progress planning. 98% of subjects did not know the latest recommended guidelines for physical activity, despite most agreeing that physical fitness and exercise are good for health. Most subjects (72%) indicated a willingness to accept self-pay service for physical fitness assessments. Conclusions This is the first study to report on the demand for cognition, assessment, and promotion of physical fitness among Taiwanese adults. The study shows that the subjects widely lack knowledge in the cognition of physical fitness and exercise prescription. Furthermore, a selfpay service for the physical fitness assessment and individualized exercise prescription were acceptable to most subjects, especially those undergoing regular health examinations. The findings are encouraging and will aid support for health organizations and professionals in the development and management of promotion strategies on health-related physical fitness in preventive medicine and health promotion. © 2020 Ho et al. This is an open access article distributed under the terms of the Creative Commons Attribution License, which permits unrestricted use, distribution, and reproduction in any medium, provided the original author and source are credited.",
**19**	Besenyi G.M., Hayashi E.B., Christiana R.W.,"**Prescribing physical activity in parks and nature: Health care provider insights on park prescription programs**",**2020**,"https://www.scopus.com/inward/record.uri?eid=2-s2.0-85092277685&doi=10.1123%2fjpah.2019-0479&partnerID=40&md5=c055b21961f87dc872988fe3de1ad69c",<br>"Background: Health care providers (HCPs) promoting physical activity (PA) through programs such as Park Prescriptions (ParkRx) are gaining momentum. However, it is difficult to realize provider PA practices and program interest, and differences in program success exist by provider type (eg, primary vs secondary). This study explored HCPs' (1) PA counseling practices, (2) knowledge/interest in ParkRx, (3) barriers and resources needed to implement PA counseling and ParkRx programs, and (4) differences in primary versus secondary HCPs. Methods: An e-survey administered in Spring/Summer 2018 to HCPs in 3 states examined study objectives. Results: Respondents (n = 278) were mostly primary (58.3%) HCPs. The majority asked about patient PA habits and offered PA counseling (mean = 5.0, SD = 1.5<br>mean = 4.8, SD = 1.5), but few provided written prescriptions (mean = 2.5, SD = 1.6). Providers were satisfied with their PA counseling knowledge (mean = 3.8, SD = 1.0) but not with prescribing practices (mean = 3.2, SD = 1.1). Secondary HCPs placed higher importance (P =.012) and provided significantly more written PA prescriptions (P =.005). Time was a common barrier to prescribing PA (mean = 3.4, SD = 1.2), though more so for primary HCPs (P =.000). Although few HCPs knew about ParkRx programs, 81.6% expressed interest. Access to park information and community partnerships was an important resource for program implementation. Conclusions: HCPs underutilize PA prescriptions. Despite little awareness, HCPs were interested in ParkRx programs. © 2020 Human Kinetics Publishers Inc.. All rights reserved.","Counseling
**20**	Wasserman D., Iosue M., Wuestefeld A., Carli V.,"**Adaptation of evidence-based suicide prevention strategies during and after the COVID-19 pandemic**",**2020**,"https://www.scopus.com/inward/record.uri?eid=2-s2.0-85091034872&doi=10.1002%2fwps.20801&partnerID=40&md5=b4f21f6351c5c093a85a9279461bcedb","Suicide is preventable. Nevertheless, each year 800,000 people die of suicide in the world. While there is evidence indicating that suicide rates de-crease during times of crises, they are expected to increase once the immediate crisis has passed. The COVID-19 pandemic affects risk and pro-tective factors for suicide at each level of the socio-ecological model. Economic downturn, augmented barriers to accessing health care, increased access to suicidal means, inappropriate media reporting at the societal level<br>deprioritization of mental health and preventive activities at the community level interpersonal conflicts, neglect and violence at the relationship level unemployment, poverty, loneliness and hopelessness at the individual level: all these variables contribute to an increase of depression, anxiety, post-traumatic stress disorder, harmful use of alcohol, substance abuse, and ultimately suicide risk. Suicide should be prevented by strengthening universal strategies directed to the entire population, including mitigation of unemployment, poverty and inequalities prioritization of access to mental health care
**21**	Pardhan S., Nakafero G., Raman R., Sapkota R.,"**Barriers to diabetes awareness and self-help are influenced by people's demographics: perspectives of South Asians with type 2 diabetes**",**2020**,"https://www.scopus.com/inward/record.uri?eid=2-s2.0-85044471084&doi=10.1080%2f13557858.2018.1455809&partnerID=40&md5=b5da4eb88b596d7be1bb74eb3105f03b",<br>"Aim: To determine whether barriers to diabetes awareness and self-help differ in South Asian participants of different demographic characteristics (age, gender, and literacy) with type 2 diabetes living in the United Kingdom. Methods: Six focus group discussions (FGDs) were carried out in patients who were categorized according to age (30–60 years, ≥60 years), gender (male, female) and literacy status (literate, illiterate). Data were analysed following the iterative process of thematic analysis techniques. Results: Barriers were demographic-specific. The illiterate groups reported language as the major barrier to improved diabetes awareness and self-help. The literate groups reported that information provided by healthcare providers was general, and not specific to their diet/culture. Major barriers to adherence to the recommended diet for diabetes included: insufficient knowledge/awareness about nutritional content of food (all groups)<br>lack of self-will to resist eating sweets, especially during weddings/festivals (literate older groups/literate younger females/illiterate older males) difficulty cooking separate meals for diabetic and non-diabetic family members (illiterate/literate older females). Other barriers to seeking advice/help ranged from not wanting to disclose their diabetes as it may affect employment/work (literate groups) to fear of being singled out at social gatherings (illiterate groups). General lack of motivation to exercise was reported by all groups. Time constraints and not knowing what/how to exercise was reported by literate younger groups whilst the illiterate older groups reported to not having suitable exercising facilities at local communities. Different barriers were also reported when accessing healthcare language barriers (illiterate groups), restricted access to doctors' appointments/difficulty attending specific appointment slots offered by General Practitioners (literate females). Conclusion: Different barriers exist to improved awareness about diabetes and self-help in different patient demographics. Lack of culturally appropriate diabetes educational/awareness programs in the community appeared to be a major barrier in most older and illiterate participants while younger participants reported time constraint. © 2018 Informa UK Limited, trading as Taylor & Francis Group."
**22**	Cahan E.M., Mittal V., Shah N.R., Thadaney-Israni S.,"**Achieving a Quintuple Aim for Telehealth in Pediatrics**",**2020**,"https://www.scopus.com/inward/record.uri?eid=2-s2.0-85086364069&doi=10.1016%2fj.pcl.2020.04.015&partnerID=40&md5=b09aff463177b4613031917c4cc900dd",<br>"Pediatric practice increasingly involves providing care for children with medical complexity. Telehealth offers a strategy for providers and health care systems to improve care for these patients and their families. However, lack of awareness related to the unintended negative consequences of telehealth on vulnerable populations–coupled with failure to intentional design best practices for telehealth initiatives–implies that these novel technologies may worsen health disparities in the long run. This article reviews the positive and negative implications of telehealth. In addition, to achieve optimal implementation of telehealth, it discusses 10 considerations to promote optimal care of children using these technologies. © 2020 Elsevier Inc.","Bioethics
**23**	Chehade M.J., Yadav L., Jayatilaka A., Gill T.K., Palmer E.,"**Personal digital health hubs for multiple conditions [Centres de santé numériques et personnalisés pour pathologies multiples] [Centros de salud virtuales personalizados para múltiples afecciones]**",**2020**,"https://www.scopus.com/inward/record.uri?eid=2-s2.0-85088793147&doi=10.2471%2fBLT.19.249136&partnerID=40&md5=0fd938ff4907436cc24e747eaf17b8a1",<br>"Multimorbidity is the presence of more than one chronic disease condition in an individual. Health-related, socioeconomic, cultural and environmental factors, as well as patient behaviour, all influence the outcomes of multimorbidity. Addressing these complex and often interacting biopsychosocial factors therefore requires a shift in treatment from a physical damage model towards person-centred integrated care with increased patient agency. Education influences behaviour and can be used to empower patients and their carers with greater agency, thus allowing greater responsibility for and control over the management of patient care. In this paper we reflect on our own learning as a community of health practitioners from different disciplines. Recognizing the increasing importance of patient agency in driving the evolution of health care, we describe the concept of a web-based personal digital health hub for integrated patient care. Informed by collaboration between patient, health and education communities, we share our early experience in the implementation of a health hub around a cohort of patients with hip fractures. We also describe a vision for future health care based on the co-creation of digital health hubs centred on patients' and carers' needs. The health hub could allow important advances and efficiencies to be achieved in workforce practice and education<br>patient and carer engagement in self-care and the collection of patient-reported health data required for ongoing research and improvements in health care. © 2020, World Health Organization. All rights reserved.",
**24**	Sala M., Rochefort C., Lui P.P., Baldwin A.S.,"**Trait mindfulness and health behaviours: a meta-analysis**",**2020**,"https://www.scopus.com/inward/record.uri?eid=2-s2.0-85070811877&doi=10.1080%2f17437199.2019.1650290&partnerID=40&md5=1ddb064ec73c280051bc66f8e3dfa173",<br>"Mindfulness is defined as bringing one's attention to present-moment experience with acceptance, and is associated with engagement in various health behaviours. To synthesise and evaluate this literature, we conducted a comprehensive meta-analytic review and examined (a) the associations between trait mindfulness and health behaviours and (b) the extent to which these associations were moderated by study and individual differences. A total of 125 independent samples were included (N = 31,697, median male percentage = 38.8%, median age = 28.3). A multilevel random-effects model was used to estimate summary study-level effect sizes, and multilevel mixed-effects models were used to examine moderator effects. Mindfulness had a positive and small association with aggregated health behaviours (r =.08). Mindfulness was positively associated with physical activity, healthy eating, and sleep (rs =.08–.14), and negatively associated with alcohol use (r = –.06). Effects were larger for health promoting behaviours, the acting with awareness facet of mindfulness, and samples involving psychiatric patients. Although findings indicate that individual differences in trait mindfulness do not reliably translate into a pattern of healthful behaviours in general, trait mindfulness shows a stronger associations with health behaviours under certain conditions. © 2019 Informa UK Limited, trading as Taylor & Francis Group.","healthy eating

25	Throuvala M.A., Griffiths M.D., Rennoldson M., Kuss D.J.,"**Mind over matter: Testing the efficacy of an online randomized controlled trial to reduce distraction from smartphone use**",**2020**,"https://www.scopus.com/inward/record.uri?eid=2-s2.0-85087438925&doi=10.3390%2fijerph17134842&partnerID=40&md5=a8c0f6648d45adfa03f6500304f15f84",<br>"Evidence suggests a growing call for the prevention of excessive smartphone and social media use and the ensuing distraction that arises affecting academic achievement and productivity. A ten-day online randomized controlled trial with the use of smartphone apps, engaging participants in mindfulness exercises, self-monitoring and mood tracking, was implemented amongst UK university students (n = 143). Participants were asked to complete online pre-and post-intervention assessments. Results indicated high effect sizes in reduction of smartphone distraction and improvement scores on a number of self-reported secondary psychological outcomes. The intervention was not effective in reducing habitual behaviours, nomophobia, or time spent on social media. Mediation analyses demonstrated that: (i) emotional self-awareness but not mindful attention mediated the relationship between intervention effects and smartphone distraction, and (ii) online vigilance mediated the relationship between smartphone distraction and problematic social media use. The present study provides preliminary evidence of the efficacy of an intervention for decreased smartphone distraction and highlights psychological processes involved in this emergent phenomenon in the smartphone literature. Online interventions may serve as complementary strategies to reduce distraction levels and promote insight into online engagement. More research is required to elucidate the mechanisms of digital distraction and assess its implications in problematic use. © 2020 by the authors.","Distraction
26	Monteiro-Guerra F., Rivera-Romero O., Fernandez-Luque L., Caulfield B.,"**Personalization in Real-Time Physical Activity Coaching Using Mobile Applications: A Scoping Review**",**2020**,"https://www.scopus.com/inward/record.uri?eid=2-s2.0-85086052556&doi=10.1109%2fJBHI.2019.2947243&partnerID=40&md5=c2e4cf7ee8d64bf8d5b0910f2c023b11",<br>"Mobile monitoring for health and wellness is becoming more sophisticated and accurate, with an increased use of real-time personalization technologies that may improve the effectiveness of physical activity coaching systems. This study aimed to review real-time physical activity coaching applications that make use of personalization mechanisms. A scoping review, using the PRISMA-ScR checklist, was conducted on the literature published from July 2007 to July 2018. A data extraction tool was developed to analyze the systems on general characteristics, personalization, design foundations (behavior change and gamification) and evaluation methods. 28 papers describing 17 different mobile applications were included. The most used personalization concepts were Feedback (17/17), Goal Setting (15/17), User Targeting (9/17) and Inter-human Interaction (8/17), while the less commonly covered were Self-Learning (4/17), Context Awareness (3/17) and Adaptation (2/17). Few systems considered behavior change theories for design (6/17). A total of 42 instances of gamification-related elements were found across 15 systems, but only 6 explicitly mention its use. Most systems (15/17) were submitted to some type of evaluation. However, few assessed the effects of particular strategies or overall system effectiveness using randomized experimental designs (5/17). Although personalization is thought to improve user adherence in physical activity coaching applications, it is still far from reaching its full potential. We believe that future work should consider the theory and suggestions reported in prior work<br>leverage the needs of the target users for personalization include behavior change foundations and explore gamification theory and properly evaluate these systems. © 2013 IEEE."
27	Harris M.A., Crone D.,"**Motivations and barriers to engagement with a technology-enabled community wide physical activity intervention**",**2020**,"https://www.scopus.com/inward/record.uri?eid=2-s2.0-85087321093&doi=10.1371%2fjournal.pone.0232317&partnerID=40&md5=b0a452f094f6947aa5a254847f9d97cb",<br>"Previous physical activity interventions have failed to create population change and an alternative approach is needed to support a World Health Organization target of a 15% reduction in global levels of inactivity by 2030. There is growing evidence that gamification-based interventions can reach substantial portions of the community. However, to date, these studies have been predominantly quantitative and as such there is a paucity of research in the area on motivations and barriers to engagement with these programs. Four focus groups conducted with N = 26 players who participated in a gamification-based intervention 'Beat the Street' revealed several varied motives to engagement, including collective reward<br>social influence game reinvention exploration accessibility and awareness. However, several barriers specific to the Beat the Street intervention and outdoor gamification interventions more generally were also identified. This study provides novel insight into the motives which engage individuals into physical activity interventions and the design principles which need to be considered when implementing interventions of this nature. © 2020 Harris, Crone. This is an open access article distributed under the terms of the Creative Commons Attribution License, which permits unrestricted use, distribution, and reproduction in any medium, provided the original author and source are credited.",
28	Guerra J., Smith L., Vicinanza D., Stubbs B., Veronese N., Williams G.,"**The use of sonification for physiotherapy in human movement tasks: A scoping review [Sonification du mouvement en kinésithérapie: une revue exploratoire de la littérature]**",**2020,**"https://www.scopus.com/inward/record.uri?eid=2-s2.0-85078835671&doi=10.1016%2fj.scispo.2019.12.004&partnerID=40&md5=ddeb655cdf2cabb935e6ec654441e508","Objectives: This review aims to: (1) map the use of sonification in human movement tasks for physical therapy<br>(2) identify methods of data capture, tasks and its effects on human subjects (3) suggest future research directions. News: Sonification can be described as a technique to translate data into sound. It has been used for human motion analysis tasks even if it is not part of most physical therapist's lexicon. Prospects and projects: Identify and analyze publications where sonification was used as an audio-feedback technique for physical therapy. Thirty-five papers were included, 13 randomized-control-trials. Thirteen papers reported an investigation on a specific dysfunction, while upper limb movements were investigated in fifteen papers. Inertial measurement units were the most commonly used technology to capture human movement, 10 papers reported improvements in motor control and/or movement quality. Gaps in the literature were identified: (1) absence of sonification framework for rehabilitation, (2) no long-term comparison with gold-standard interventions for specific populations, (3) approaches for cardio-respiratory physical therapy and injury prevention were absent. Conclusion: Sonification has the potential to support rehabilitation for physical therapy. Effects of sonification were varied and ranged from improvements in movement quality/control, increased movement and body-awareness and improvements in performance when compared with activities with audio-visual or non-specific audio-feedback among others. Data for sonification was mainly captured using inertial measurement units, smartphones and optical tracking devices but others are also commonly used. Well-designed clinical trials supported by current promising results need to be developed. We recommend testing different sonification techniques in common physical therapy disfunctions using significant outcome measures to understand and maximize its effects on motor learning and control while scoping for further benefits. © 2020 Elsevier Masson SAS","Movement
29	Carlsten C., Salvi S., Wong G.W.K., Chung K.F.,"**Personal strategies to minimise effects of air pollution on respiratory health: Advice for providers, patients and the public**",**2020**,"https://www.scopus.com/inward/record.uri?eid=2-s2.0-85084648521&doi=10.1183%2f13993003.02056-2019&partnerID=40&md5=316faf4c1ac64705d3eab09ca653daf1",<br>"As global awareness of air pollution rises, so does the imperative to provide evidence-based recommendations for strategies to mitigate its impact. While public policy has a central role in reducing air pollution, exposure can also be reduced by personal choices. Qualified evidence supports limiting physical exertion outdoors on high air pollution days and near air pollution sources, reducing nearroadway exposure while commuting, utilising air quality alert systems to plan activities, and wearing facemasks in prescribed circumstances. Other strategies include avoiding cooking with solid fuels, ventilating and isolating cooking areas, and using portable air cleaners fitted with high-efficiency particulate air filters. We detail recommendations to assist providers and public health officials when advising patients and the public regarding personal-level strategies to mitigate risk imposed by air pollution, while recognising that well-designed prospective studies are urgently needed to better establish and validate interventions that benefit respiratory health in this context. © 2020 ERS.",
30	Hirano R., Yamaguchi S., Waki K., Kimura Y., Chin K., Nannya Y., Nangaku M., Kadowaki T., Ohe K.,"**Willingness of patients prescribed medications for lifestyle-related diseases to use personal health records: Questionnaire study**", **2020**, "https://www.scopus.com/inward/record.uri?eid=2-s2.0-85085664875&doi=10.2196%2f13866&partnerID=40&md5=8d7b2e2cf9fe9bc78d8e7f596df40915",<br>"Background: Personal health record (PHR) systems let individuals utilize their own health information to maintain and improve quality of life. Using PHRs is expected to support self-management in patients with lifestyle-related diseases. Objective: The aim of this study was to identify predictors of the willingness to use PHRs among patients who are prescribed medications for lifestyle-related diseases. Methods: We recruited pharmacy patrons, aged 20 years or older, who had received at least one medication indicated for hypertension, dyslipidemia, or diabetes. Participants completed self-administered questionnaires regarding their previous diseases, awareness of health care, experience in using PHRs, willingness to use PHRs, and barriers to using PHRs. Data were analyzed using multivariate logistic regression models. Results: Of the 3708 subjects meeting eligibility criteria, 2307 replies (62.22%) were collected. While only 174 (7.54%) participants had previous PHR experience, 853 (36.97%) expressed willingness to use PHRs. In the multivariate analysis, considering exercise to be important for health management (odds ratio [OR] 1.57, 95% CI 1.12-2.21<br>P=.009), obtaining medical information from books or magazines (OR 1.23, 95% CI 0.96-1.59 P=.10), and obtaining medical information from the internet (OR 1.45, 95% CI 1.13-1.87 P=.004) were newly identified predictors. These were in addition to known predictors, such as being employed, owning information terminals, and previous PHR experience. Conclusions: Patients who have an active and positive attitude toward health seem to be more willing to use PHRs. Investigating willingness should contribute to the development of more useful PHRs for self-management among patients prescribed medications for lifestyle-related diseases. © Ryoma Hirano, Satoko Yamaguchi, Kayo Waki, Yoshihiko Kimura, Keiichi Chin, Yasuhito Nannya, Masaomi Nangaku, Takashi Kadowaki, Kazuhiko Ohe. Originally published in the Journal of Medical Internet Research (http://www.jmir.org),28.05.2020. This is an open-access article distributed under the terms of the Creative Commons Attribution License (https://creativecommons.org/licenses/by/4.0/), which permits unrestricted use, distribution, and reproduction in any medium, provided the original work, first published in the Journal of Medical Internet Research, is properly cited. The complete bibliographic information, a link to the original publication on http://www.jmir.org/, as well as this copyright and license information must be included.","Chronic disease

**31**	Burge A.T., Cox N.S., Abramson M.J., Holland A.E.,"**Interventions for promoting physical activity in people with chronic obstructive pulmonary disease** (COPD)",**2020,**"https://www.scopus.com/inward/record.uri?eid=2-s2.0-85083479318&doi=10.1002%2f14651858.CD012626.pub2&partnerID=40&md5=5a8809ac641b829504d292b7754ab145", "Background: Escalating awareness of the magnitude of the challenge posed by low levels of physical activity in people with chronic obstructive pulmonary disease (COPD) highlights the need for interventions to increase physical activity participation. The widely-accepted benefits of physical activity, coupled with the increasing availability of wearable monitoring devices to objectively measure participation, has led to a dramatic rise in the number and variety of studies that aimed to improve the physical activity of people with COPD. However, little was known about the relative efficacy of interventions tested so far. Objectives: In people with COPD, which interventions are effective at improving objectively-assessed physical activity?. Search methods: We identified trials from the Cochrane Airways Trials Register Register, which contains records identified from bibliographic databases including the Cochrane Central Register of Controlled Trials, MEDLINE, Embase, CINAHL, AMED, and PsycINFO. We also searched PEDro, ClinicalTrials.gov, the World Health Organization International Clinical Trials Registry Platform portal and the Australian New Zealand Clinical Trials Registry (from inception to June 2019). We checked reference lists of all primary studies and review articles for additional references, as well as respiratory journals and respiratory meeting abstracts, to identify relevant studies. Selection criteria: We included randomised controlled trials of interventions that used objective measures for the assessment of physical activity in people with COPD. Trials compared an intervention with no intervention or a sham/placebo intervention, an intervention in addition to another standard intervention common to both groups, or two different interventions. Data collection and analysis: We used standard methods recommended by Cochrane. Subgroup analyses were possible for supervised compared to unsupervised pulmonary rehabilitation programmes in clinically-stable COPD for a range of physical activity outcomes. Secondary outcomes were health-related quality of life, exercise capacity, adverse events and adherence. Insufficient data were available to perform prespecified subgroup analyses by duration of intervention or disease severity. We undertook sensitivity analyses by removing studies that were at high or unclear risk of bias for the domains of blinding and incomplete outcome data. Main results: We included 76 studies with 8018 participants. Most studies were funded by government bodies, although some were sponsored by equipment or drug manufacturers. Only 38 studies had physical activity as a primary outcome. A diverse range of interventions have been assessed, primarily in single studies, but improvements have not been systematically demonstrated following any particular interventions. Where improvements were demonstrated, results were confined to single studies, or data for maintained improvement were not provided. Step count was the most frequently reported outcome, but it was commonly assessed using devices with documented inaccuracy for this variable. Compared to no intervention, the mean difference (MD) in time in moderate- to vigorous-intensity physical activity (MVPA) following pulmonary rehabilitation was four minutes per day (95% confidence interval (CI) −2 to 9
**32**	Etminani K., Engström A.T., Göransson C., Sant'anna A., Nowaczyk S.,"**How behavior change strategies are used to design digital interventions to improve medication adherence and blood pressure among patients with hypertension: Systematic review**",**2020**,"https://www.scopus.com/inward/record.uri?eid=2-s2.0-85083538249&doi=10.2196%2f17201&partnerID=40&md5=677f1af621dd3a46dbdac51924dd5e34", "Background: Information on how behavior change strategies have been used to design digital interventions (DIs) to improve blood pressure (BP) control or medication adherence (MA) for patients with hypertension is currently limited. Objective: Hypertension is a major modifiable risk factor for cardiovascular diseases and can be controlled with appropriate medication. Many interventions that target MA to improve BP are increasingly using modern digital technologies. This systematic review was conducted to discover how DIs have been designed to improve MA and BP control among patients with hypertension in the recent 10 years. Results were mapped into a matrix of change objectives using the Intervention Mapping framework to guide future development of technologies to improve MA and BP control. Methods: We included all the studies regarding DI development to improve MA or BP control for patients with hypertension published in PubMed from 2008 to 2018. All the DI components were mapped into a matrix of change objectives using the Intervention Mapping technique by eliciting the key determinant factors (from patient and health care team and system levels) and targeted patient behaviors. Results: The analysis included 54 eligible studies. The determinants were considered at two levels: Patient and health care team and system. The most commonly described determinants at the patient level were lack of education, lack of self-awareness, lack of self-efficacy, and forgetfulness. Clinical inertia and an inadequate health workforce were the most commonly targeted determinants at the health care team and system level. Taking medication, interactive patient-provider communication, self-measurement, and lifestyle management were the most cited patient behaviors at both levels. Most of the DIs did not include support from peers or family members, despite its reported effectiveness and the rate of social media penetration. Conclusions: This review highlights the need to design a multifaceted DI that can be personalized according to patient behavior(s) that need to be changed to overcome the key determinant(s) of low adherence to medication or uncontrolled BP among patients with hypertension, considering different levels including patient and healthcare team and system involvement. © 2020 Journal of Medical Internet Research. All rights reserved.","Behavior change
**33**	Kim D.Y., Kwon H., Nam K.-W., Lee Y., Kwon H.-M., Chung Y.S.,"**Remote management of poststroke patients with a smartphone-based management system integrated in clinical care: Prospective, nonrandomized, interventional study**",**2020**,"https://www.scopus.com/inward/record.uri?eid=2-s2.0-85081389629&doi=10.2196%2f15377&partnerID=40&md5=e5e1337e5c54ddc1be3890437f0519a7", "Background: Advances in mobile health (mHealth) have enabled systematic and continuous management of patients with chronic diseases. Objective: We developed a smartphone-based mHealth system and aimed to evaluate its effects on health behavior management and risk factor control in stroke patients. Methods: With a multifaceted stroke aftercare management system that included exercise, medication, and educational materials, we performed a 12-week single-arm intervention among eligible poststroke patients in the stroke clinic from September to December 2016. The intervention consisted of (1) regular blood pressure (BP), blood glucose, and physical activity measurements (2) stroke education (3) an exercise program (4) a medication program and (5) feedback on reviewing of records by clinicians. Clinical assessments consisted of the stroke awareness score, Beck Depression Inventory-II (BDI), EuroQol-5 Dimensions (EQ-5D), and BP at visit 1 (baseline), visit 2 (4 weeks), and visit 3 (12 weeks). Temporal differences in the parameters over 12 weeks were investigated with repeated-measures analysis of variance. Changes in medication adherence at visit 1-2 (from visit 1 to visit 2) and visit 2-3 (from visit 2 to visit 3) were compared. System satisfaction was evaluated with a self-questionnaire using a 5-point Likert scale at visit 3. Results: The study was approved by the Institutional Review Board in September 2016, and participants were enrolled from September to December 2016. Among the 110 patients enrolled for the study, 99 were included in our analyses. The mean stroke awareness score (baseline: 59.6 [SD 18.1] 4 weeks: 67.6 [SD 16.0], P<.001 12 weeks: 74.7 [SD 14.0], P<.001) and BDI score (baseline: 12.7 [SD 10.1]
**34**	Kim Y., Lee H., Lee M.K., Lee H., Jang H.,"**Development of a living lab for a mobile-based health program for korean-chinese working women in South Korea: Mixed methods study**",**2020**,"https://www.scopus.com/inward/record.uri?eid=2-s2.0-85077688155&doi=10.2196%2f15359&partnerID=40&md5=18209df4fbf6b3541648a97ff1f33b6f", "Background: Korean-Chinese (KC) women make up the largest group of female migrants in South Korea. To prevent and manage chronic diseases in middle-aged KC women working full time, it is necessary to develop health promotion programs that utilize an online platform because such a platform would allow individuals to participate in health promotion interventions at their convenience. Objective: This study aimed to develop a living lab for a mobile-based health (LLm Health) program focused on B26 of KC women workers. Methods: We used a mixed methods design. Living lab principles were factored into the LLm Health program, including the use of multiple methods, user engagement, multistakeholder participants, real-life settings, and cocreation. The program was developed using the 4 steps of the intervention mapping method: needs assessment, setting of objectives, identification of intervention strategies, and intervention design. Needs assessment was conducted through a literature review, focus group interviews with a total of 16 middle-aged KC women, and an online survey related to health promotion of migrant workers given to 38 stakeholders. KC middle-aged women participated in the early stages of program development and provided the idea of developing programs and mobile apps to enhance physical activity and acculturation. The mobile app developed in the program was validated with the help of 12 KC women and 4 experts, including 3 nursing professors and a professor of physical education. They were asked to rate each item based on content, interface design, and technology on a 4-point scale using a 23-item Smartphone App Evaluation Tool for Health Care. Results: The LLm Health program comprised a 24-week walking program using Fitbit devices, the mobile app, and social cognitive interventions. The mobile app contained 6 components: A step counter, an exercise timer, an online chat function, health information, level of cardiovascular risk, and health status. The cultural aspects and lifestyles of KC women were accommodated in the entire process of program development. The content validity of the mobile app was found to be 0.90 and 0.96 according to the 12 KC women and 4 experts, respectively. Conclusions: The mobile app was found to be valid and acceptable for KC women. The living lab approach was a useful strategy for developing a culturally adaptive LLm Health program for KC women workers, leading to their active participation in the overall research process, including needs assessment, program composition, and pre-evaluation. © 2020 Journal of Medical Internet Research. All rights reserved.","Health promotion
**35**	Kuo M.-C., Chen C.-M., Wu F.-G., Chen C.-H., Yin Z.-X., Wang C.-Y.,"**Use of photo diary and focus group to explore needs for digital disease management program among community older adults with chronic disease**",**2020**,"https://www.scopus.com/inward/record.uri?eid=2-s2.0-85097601637&doi=10.1111%2fhsc.13241&partnerID=40&md5=ed6e616fc82ed8351ad06984e0ffeb80", "As technology advanced, new e-health solutions are evolved to empower people to manage their care at home. This study explored the needs for disease management in activity tracking using photo diary through older adults' subjective perspective. It further aimed to suggest which lifestyle measures, symptoms and behaviours would be meaningful to include in such a digital diseases care management program for technology design. Both photo diary and focus group discussion were used, 11 older adults with multiple metabolism-related chronic diseases (Mean age, 72.5 ± 6.14 years) were recruited and asked to carry out the photo diary to trace their living situation and needs using a tablet camera. A focus group discussion was applied to identify the needs of chronic disease management, based on the results of living context tracing. Five themes, regular physical activity, smart management of healthy behaviors, healthy diet, regular daily routine and social connection, were identified by content analysis from photo diary and the focus group discussion. The results indicated that the photo diary program can raise awareness and promotes positive behavior changes. It is believed that the E-approach can be applied to the effectively enhance older adults' self-management by monitoring their health status and their daily routine activities. © 2020 John Wiley & Sons Ltd","disease care management

**36**	Zhang T., Yang J., Liang N., Pitts B.J., Prakah-Asante K.O., Curry R., Duerstock B.S., Wachs J.P., Yu D.,"**Physiological Measurements of Situation Awareness: A Systematic Review**",**2020**,"https://www.scopus.com/inward/record.uri?eid=2-s2.0-85096753526&doi=10.1177%2f0018720820969071&partnerID=40&md5=e172e8e5c4b77857011106d054c1aff3", "Objective: The goal of this review is to investigate the relationship between indirect physiological measurements and direct measures of situation awareness (SA). Background: Assessments of SA are often performed using techniques designed specifically to directly measure SA, such as SA global assessment technique (SAGAT), situation present assessment method (SPAM), and/or SA rating technique (SART). However, research suggests that physiological sensing methods may also be capable of inferring SA. Method: Seven databases were searched. Eligibility criteria included human–subject experiments that used at least one direct SA assessment technique as well as at least one physiological measurement. Information extracted from each article were the physiological metric(s), direct SA measurement(s), correlation between these two metrics, and experimental task(s). Results: Twenty-five articles were included in this review. Eye tracking techniques were the most commonly used physiological measures, and correlations between conscious aspects of eye movement measures and direct SA scores were observed. Evidence for cardiovascular predictors of SA was mixed. Only three electroencephalography (EEG) studies were identified, and their results suggest that EEG was sensitive to changes in SA. Overall, medium correlations were observed among the studies that reported a correlation coefficient between physiological and direct SA measures. Conclusion: Reviewed studies observed relationships between a wide range of physiological measurements and direct assessments of SA. However, further investigations are needed to methodically collect more evidence. Application: This review provides researchers and practitioners a summary of observed methods to indirectly assess SA with sensors and highlights research gaps to be addressed in future work. © The Author(s) 2020.","driver behavior
**37**	Choi S.H., McClintock C.H.-Y., Lau E., Miller L.,"**The dynamic universal profiles of spiritual awareness: A latent profile analysis**",**2020**,"https://www.scopus.com/inward/record.uri?eid=2-s2.0-85090646621&doi=10.3390%2fREL11060288&partnerID=40&md5=e79d3d71a63239ef7744d9f7a5926ad2", The aim of the current investigation was to identify universal profiles of lived spirituality. A study on a large sample of participants (N = 5512) across three countries, India, China, and the United States, suggested there are at least five cross-cultural phenotypic dimensions of personal spiritual capacity—spiritual reflection and commitment contemplative practice perception of interconnectedness perception of love and practice of altruism—that are protective against pathology in a community sample and have been replicated in matched clinical and non-clinical samples. Based on the highest frequency combinations of these five capacities in the same sample, we explored potentially dynamic profiles of spiritual engagement. We inductively derived five profiles using Latent Profile Analysis (LPA): non-seeking socially disconnected spiritual emergence virtuous humanist
**38**	Vettori V., Lorini C., Milani C., Bonaccorsi G.,"**Towards the implementation of a conceptual framework of food and nutrition literacy: Providing healthy eating for the population**",**2019**,"https://www.scopus.com/inward/record.uri?eid=2-s2.0-85076457730&doi=10.3390%2fijerph16245041&partnerID=40&md5=ad8922cd1cdaf5353ec48955642fc147", "Existing definitions of food literacy (FL) and nutrition literacy (NL) in particular refer to individual knowledge, motivation, competences, and awareness, which determine the relationship between individuals and food, the food system, and nutrition information. Several authors proposed specific conceptualization of the terms. Nevertheless, the description of analogies and differences between FL and NL is still lacking, as is an integrated framework which highlights the meaning of the concepts. This work aims to describe and discuss evidence provided by the literature in order to develop and propose a comprehensive conceptualization of FL and NL to the scientific community. We systematically reviewed six databases, considering the search terms of FL and NL. We collected the antecedents, components, and consequences of both FL and NL. We underlined and traced similarities of the concepts as well as prerogative features through the content analysis of definitions. We obtained 14 definitions of NL and 12 definitions of FL 42 papers presented antecedents and 53 papers contained consequences. We observed that NL could be considered a specific form of FL. In addition, we noted that the consequences of NL are included in the subset of the consequences of FL and the conceptual limits of FL correspond to the outcome of healthful diet. We conclude that FL and NL build a multifaceted concept which implies both individual and public perspectives. We propose a conceptualization which could be useful to develop an executive framework aimed at providing healthy eating for the population. © 2019 by the authors. Licensee MDPI, Basel, Switzerland."
**39**	Evans W.D., Harrington C., Patchen L., Andrews V., Gaminian A., Ellis L.P., Napolitano M.A.,"**Design of a novel digital intervention to promote healthy weight management among postpartum African American women**",**2019**,"https://www.scopus.com/inward/record.uri?eid=2-s2.0-85072937021&doi=10.1016%2fj.conctc.2019.100460&partnerID=40&md5=9e386a836dbc547bd667b8a85f43809c" "Background: Excess weight retention among postpartum women is a risk factor for long term obesity, and African American women are at heightened risk. New solutions, including digital technologies and community-based approaches are needed. Digital platforms, like social media, provide opportunity for participant co-creation (i.e., content co-generated by users and investigators) of health messages and may allow for adaptation of evidence-based weight management interventions to reduce participant burden. The BeFAB intervention, a branded, digital weight management program, tests this hypothesis. Methods: BeFAB content comprises culturally-specific nutrition, physical activity, stress management, health information seeking and related weight management messages and content designed for African American women. The intervention is 12 weeks in duration, delivered through a mobile phone app, and is designed to target specific behavioral predictor beliefs and attitudinal measures (e.g., self-efficacy to achieve weight management goals) based on the culturally-specific content. Use of personal, culturally-specific video-based narratives in the app, and through a secret Facebook group, are included to help model HEAL behaviors and brand BeFAB. Intervention development consisted of iterative formative research steps to engage African American women. The program will be evaluated in a small randomized trial among patients recruited at a clinical facility. Conclusions: BeFAB applies evidence-based content using a promising digital approach. It is novel in its use of branding, culturally-tailored content, and digital technology for behavior change.Evaluation of BeFAB will contribute to the growing literature on digital health behavior change interventions for weight management. © 2019","African American
**40**	Su J.J., Yu D.S.F.,"**Effectiveness of eHealth cardiac rehabilitation on health outcomes of coronary heart disease patients: A randomized controlled trial protocol**",**2019**,"https://www.scopus.com/inward/record.uri?eid=2-s2.0-85075775844&doi=10.1186%2fs12872-019-1262-5&partnerID=40&md5=599391e07cb0da1ce9933725a7bd8ca2", "Background: Cardiac rehabilitation (CR) uptake and adherence remain sub-optimal despite the apparent health benefits of modifying healthy behavior and slowing disease progression. eHealth is the use of information and communication technology (ICT) for health. eHealth lifestyle interventions and disease management have emerged as modalities to enhance CR accessibility, enable an individualized progress page, and enrich real-time contact, video-based information, and technology monitored functionality. This study aims to develop a nurse-led eHealth cardiac rehabilitation (NeCR) intervention and investigate its effectiveness on coronary heart disease (CHD) patients' health outcomes. Methods: This single-blinded two-arm parallel randomized controlled trial will randomize 146 patients from the inpatient cardiovascular units of a hospital in Wuhan, China to receive either the NeCR or the usual care. The NeCR intervention uses a hybrid approach consisting of a brief face-to-face preparatory phase and an empowerment phase delivered by health technology. The preparatory phase aims at identifying self-care needs, developing a goal-oriented patient centered action plan, incorporating a peer support network and orientation to the use of the e-platform. The empowerment phase includes use of the multi-media interactive NeCR for promoting symptom management, monitoring lifestyle changes and offering psychological support. A tele-care platform is also integrated to enhance health care dialogue with health professionals and peer groups. The control group will receive the usual care. An evaluation of lifestyle behavioral changes, self-efficacy, health-related quality of life, anxiety and depression, cardiovascular risk parameters, and unplanned health services use will be conducted at baseline, 6 weeks and 12 weeks post-intervention. Discussion: This protocol proposes an individualized, comprehensive, and interactive NeCR delivered using a hybrid approach and guided by an empowerment model to optimize health outcomes of CHD patients. The intervention content and web-design is based on international health guidelines to improve credibility, comprehensibility and implementation. This study also proposes a new method of peer support in which the researcher shares participants' progress toward goal attainment with the peer group. Results of this research have the potential to increase accessibility and availability of CR, improve cardiac rehabilitation service development in China, and inform eHealth lifestyle interventions. Trial registration: Chinese Clinical Trial Registry: ChiCTR1800020411 Date of registration: December 28, 2018. © 2019 The Author(s)
**41**	Rasoul A.M., Jalali R., Abdi A., Salari N., Rahimi M., Mohammadi M.,"**The effect of self-management education through weblogs on the quality of life of diabetic patients**",2019,"https://www.scopus.com/inward/record.uri?eid=2-s2.0-85074356179&doi=10.1186%2fs12911-019-0941-6&partnerID=40&md5=9e6f685155ffa070d7f25442b569284f", "Background: Self-management education of diabetes which is one of the most important noncommunicable diseases worldwide involves facilitating knowledge, skills, and ability required for self-care in these patients. Concerning the progressive growth of use of Internet for educating patients and absence of different studies about education through use of weblogs in patients with diabetes in Iran, the present study was conducted with the aim of determining the effect of self-management education through weblogs on the quality of life of affect the patients. Methods: This study was performed as intervention on patients referring to diabetes clinic of Talghani hospital in Kermanshah in winter 2018 and spring 2019. The samples consisted of 98 patients with diabetes chosen through available sampling and randomly assigned into study and control groups. For data collection, diabetes quality of life (DQOL) short form clinical questionnaire, Persian version, was used. The intervention involved training self-management conducted through 60 sessions via a designed weblog. The obtained information was introduced into SPSS 21, and analyzed through Mann-Whitney, t-test, and paired t-test. Results: According to the results of this study, the mean age of the examined patients was 32.1 ± 4.9 years, where the major participants were male (n = 52 in the test group, 52.5%). The results showed that after the intervention, the test and control groups were different in terms of anthropometric variables and metabolic indicators the mean waist circumference in the test and control groups was 98.6 ± 9.8 and 101.5 ± 7.8, respectively the mean FBS following the intervention in the test and control groups was 131.08 ± 16.04 and 238.2 ± 40, respectively and the mean BMI postintervention in the test and control groups was obtained as 27.3 ± 3.4 and 30.1 ± 3.8 respectively, where these differences were significant according to independent t-test (p < 0.05). The mean score of quality of life postintervention in the test and control groups was obtained as 56.1 and 49.9 respectively according to Mann-Whitney test, the difference between the two groups was significant (p < 0.05). Conclusion: The results of the present study revealed the positive effect of weblog based self-management on the quality of life of patients with diabetes following the intervention. Further, reduced levels of FBS, BMI, as well as systolic and diastolic blood pressure were also observed, which could be due to increased awareness of patients about their abilities, its risks, as well as the ways to control and treat it. © 2019 The Author(s).",""

**42**	Zhao M., Zhang M., Ying J., Wang S., Shi Y., Li H., Li Y., Xing Z., Sun J.,"**Knowledge, attitudes, practices and information demand in relation to haze in China: A cross-sectional study**",**2019**,"https://www.scopus.com/inward/record.uri?eid=2-s2.0-85074226335&doi=10.1186%2fs12889-019-7772-3&partnerID=40&md5=ae79f4bb3025a53336ccb01b98e8b7f2",<br>"Background: Information on the effects of haze on health and corresponding knowledge, attitudes, and practices (KAP) will improve self-care ability against the ill effects of haze pollution and make environmental health policies more targeted and effective. In this study, we aim to determine the KAP and information demand in the context of haze and its effects on health. Methods: A cross-sectional survey was conducted in Changchun, China, between October and November 2016. A purposive sample comprising four groups (college students, occupational population, traffic police, and elderly population) were investigated. Personal information and questions pertaining to KAP and information demand on haze pollution and health were collected through questionnaire. Results: The questionnaire was completed by 888 residents. The awareness rate was 57.7% and varied significantly with education, exercise, and population group ($p < 0.05$). Among the four groups, college students scored the lowest in attitudes and practices, whereas traffic police scored the highest. Concerning the source of information demand, the elderly and traffic police preferred traditional information access (i.e., television and newspaper), whereas college students and the occupational population preferred new social media (i.e., mobile newspaper and social media sites). Regarding the most trusted department that releases information, all residents relied on the haze information released by the environmental protection department and meteorological department. The latest air quality was the most desired information of the residents. Conclusions: Changchun residents have a relatively high knowledge awareness rate. The elderly and less-educated residents are the targetable population for haze education. Education should be disseminated in such manner as to meet the needs of different people. © 2019 The Author(s).","Attitudes
**43**	Gabrić I.D.,"**How to monitor your physical activity?** [Kako pratiti svoju tjelesnu aktivnost]",**2019**,"https://www.scopus.com/inward/record.uri?eid=2-s2.0-85078616809&partnerID=40&md5=4dbbc7b474210283dc4bee4613001000",<br>"Regular physical activity at any age is connected with a reduced risk of almost all diseases. However, despite the increased awareness of the need for physical activity, the incidence of obesity and cardiovascular diseases is on the rise. The cause for this is the lack of adherence and regular tracking of physical activity. The development of mobile technology for tracking physical activity has been of great help in the last couple of years. The benefits of using such technology have been confirmed in numerous controlled studies, as well as in everyday life. No matter what device, programme or mobile application is used to track physical activity, regular usage is essential. Systematic tracking of daily, as well as weekly and monthly activity trends enables better control of variation between the planned and performed activity. As a result, healthcare professionals are provided with an individual approach to the determination of necessary activity, depending on age, physical condition and possible chronic diseases. © 2019, PLIVA d.d.. All rights reserved.","Physical activity
**44**	Niksirat K.S., Silpasuwanchai C., Cheng P., Ren X.,"**Attention regulation framework: Designing self-regulated mindfulness technologies**",**2019**,"https://www.scopus.com/inward/record.uri?eid=2-s2.0-85074886177&doi=10.1145%2f3359593&partnerID=40&md5=00952c1ec625f3973958a713260e53e7",<br>"Mindfulness practices are well-known for their benefits to mental and physical well-being. Given the prevalence of smartphones,mindfulness applications have attracted growing global interest. However, themajority of existing applications use guided meditation that is not adaptable to each user's unique needs or pace. This article proposes a novel framework called Attention Regulation Framework (ARF), which studies how more flexible and adaptable mindfulness applications could be designed, beyond guided meditation and toward self-regulated meditation. ARF proposes mindfulness interaction design guidelines and interfaces whereby practitioners naturally and constantly bring their attention back to the present moment and develop nonjudgmental awareness. This is achieved by the performance of subtle movements, which are supported by non-intrusive detection-feedback mechanisms. We used two design cases to demonstrate ARF in static and kinetic meditation conditions. We conducted four user evaluation studies in unique situations where ARF is particularly effective, vis-à-vis mindfulness practice in busy environments and mindfulness interfaces that adapt to the pace of the user. The studies show that the design cases, compared with guided meditation applications, are more effective in improving attention, mindfulness, mood, well-being, and physical balance. Our work contributes to the development of self-regulated mindfulness technologies. © 2019 Association for Computing Machinery.","Attention
**45**	Sutherland R., Nathan N., Brown A., Yoong S., Finch M., Lecathelinais C., Reynolds R., Walton A., Janssen L., Desmet C., Gillham K., Herrmann V., Hall A., Wiggers J., Wolfenden L.,"**A randomized controlled trial to assess the potential efficacy, feasibility and acceptability of an m-health intervention targeting parents of school aged children to improve the nutritional quality of foods packed in the lunchbox 'SWAP IT'**",**2019**,"https://www.scopus.com/inward/record.uri?eid=2-s2.0-85068529468&doi=10.1186%2fs12966-019-0812-7&partnerID=40&md5=6c32a320f620c23bab6c44348dc09910",<br>"Background: Scalable interventions that improve the nutritional quality of foods in children's lunchboxes have considerable potential to improve child public health nutrition. This study assessed the potential efficacy, feasibility and acceptability of an m-health intervention, 'SWAP IT', to improve the energy and nutritional quality of foods packed in children's lunchboxes. Methods: The study employed a 2X2 factorial cluster randomized-controlled trial design. Twelve primary schools in New South Wales, Australia were randomly allocated to one of four groups: (i) no intervention<br>(ii) physical activity intervention only (iii) lunchbox intervention only or(iv) physical activity and lunchbox intervention combined. The two intervention strategies were evaluated separately. This paper focuses on the effects of the lunchbox intervention only. The lunchbox intervention comprised four strategies: 1) school nutrition guidelines 2) lunchbox lessons 3) information pushed to parents via a school-communication app and 4) parent resources addressing barriers to packing healthy lunchboxes. Outcome measures were taken at baseline and immediately post-intervention (10 weeks) and included measures of effectiveness (mean energy (kJ) packed in lunchboxes, total energy and percentage energy from recommended foods consistent with Australian Dietary Guidelines), feasibility (of delivering intervention to schools, parent app engagement and behaviour change) and acceptability to school staff and parents. Linear mixed models were used to assess intervention efficacy. Results: Of the 1915 lunchbox observations, at follow-up there was no significant differences between intervention and control group in mean energy of foods packed within lunchboxes (- 118.39 kJ, CI = -307.08, 70.30, $p = 0.22$). There was a significant increase favouring the intervention in the secondary outcome of mean lunchbox energy from recommended foods (79.21 kJ, CI = 1.99, 156.43, $p = 0.04$), and a non-significant increase in percentage of lunchbox energy from recommended foods in intervention schools (4.57%, CI = -0.52, 9.66, $p = 0.08$). The views of the messages pushed via the app ranged from 387 to 1550 views per week (mean views =1025 per week). A large proportion (71%) of parents reported awareness of the intervention, making healthier swaps in the lunchbox (55%), and pushed content was helpful (84%). Conclusion: The study is the first RCT to assess the potential of a multi-component m-health lunchbox intervention. The intervention was feasible, acceptable and potentially effective in improving the nutritional quality of foods packed within children's lunchboxes. Trial registration: Australian Clinical Trials Registry ACTRN: ACTRN12616001228471. © 2019 The Author(s).","Child nutrition
**46**	Majumder S., Deen M.J.,"**Smartphone sensors for health monitoring and diagnosis**", **2019**, "https://www.scopus.com/inward/record.uri?eid=2-s2.0-85065954138&doi=10.3390%2fs19092164&partnerID=40&md5=167afeefe0ec2148312e3fdb91f0a996",<br>"Over the past few decades, we have witnessed a dramatic rise in life expectancy owing to significant advances in medical science and technology, medicine as well as increased awareness about nutrition, education, and environmental and personal hygiene. Consequently, the elderly population in many countries are expected to rise rapidly in the coming years. A rapidly rising elderly demographics is expected to adversely affect the socioeconomic systems of many nations in terms of costs associated with their healthcare and wellbeing. In addition, diseases related to the cardiovascular system, eye, respiratory system, skin and mental health are widespread globally. However, most of these diseases can be avoided and/or properly managed through continuous monitoring. In order to enable continuous health monitoring as well as to serve growing healthcare needs affordable, non-invasive and easy-to-use healthcare solutions are critical. The ever-increasing penetration of smartphones, coupled with embedded sensors and modern communication technologies, make it an attractive technology for enabling continuous and remote monitoring of an individual's health and wellbeing with negligible additional costs. In this paper, we present a comprehensive review of the state-of-the-art research and developments in smartphone-sensor based healthcare technologies. A discussion on regulatory policies for medical devices and their implications in smartphone-based healthcare systems is presented. Finally, some future research perspectives and concerns regarding smartphone-based healthcare systems are described. © 2019 by the authors. Licensee MDPI, Basel, Switzerland.","Medical device
**47**	Thomsen T., Esbensen B.A., Hetland M.L., Aadahl M.,"**Motivational Counseling and Text Message Reminders: For Reduction of Daily Sitting Time and Promotion of Everyday Physical Activity in People with Rheumatoid Arthritis**",**2019**,"https://www.scopus.com/inward/record.uri?eid=2-s2.0-85063740443&doi=10.1016%2fj.rdc.2019.01.005&partnerID=40&md5=60a92b53ed00cd9d1bb874136454d7ee","Most patients with rheumatoid arthritis tend to be physically inactive and spend more time in sedentary behaviors compared with the general population. This inactive lifestyle can lead to serious health consequences, for example, increased risk of cardiovascular disease. For this reason, there is an interest in increasing participation in physical activity in patients with rheumatoid arthritis. The relatively new approach of reducing sedentary behavior and replacing it with light-intensity physical activity has been shown to be feasible and effective in promoting physical activity in patients with rheumatoid arthritis. However, methods to facilitate this behavior have not yet been fully explored. © 2019 Elsevier Inc.",

48	Goodale B.M., Shilaih M., Falco L., Dammeier F., Hamvas G., Leeners B.,"**Wearable sensors reveal menses-driven changes in physiology and enable prediction of the fertile window: Observational study**",**2019**, "https://www.scopus.com/inward/record.uri?eid=2-s2.0-85065062914&doi=10.2196%2f13404&partnerID=40&md5=cafb7ed893e7967a426af6ea16a373fb", "Background: Previous research examining physiological changes across the menstrual cycle has considered biological responses to shifting hormones in isolation. Clinical studies, for example, have shown that women's nightly basal body temperature increases from 0.28 to 0.56 °C following postovulation progesterone production. Women's resting pulse rate, respiratory rate, and heart rate variability (HRV) are similarly elevated in the luteal phase, whereas skin perfusion decreases significantly following the fertile window's closing. Past research probed only 1 or 2 of these physiological features in a given study, requiring participants to come to a laboratory or hospital clinic multiple times throughout their cycle. Although initially designed for recreational purposes, wearable technology could enable more ambulatory studies of physiological changes across the menstrual cycle. Early research suggests that wearables can detect phase-based shifts in pulse rate and wrist skin temperature (WST). To date, previous work has studied these features separately, with the ability of wearables to accurately pinpoint the fertile window using multiple physiological parameters simultaneously yet unknown. Objective: In this study, we probed what phase-based differences a wearable bracelet could detect in users' WST, heart rate, HRV, respiratory rate, and skin perfusion. Drawing on insight from artificial intelligence and machine learning, we then sought to develop an algorithm that could identify the fertile window in real time. Methods: We conducted a prospective longitudinal study, recruiting 237 conception-seeking Swiss women. Participants wore the Ava bracelet (Ava AG) nightly while sleeping for up to a year or until they became pregnant. In addition to syncing the device to the corresponding smartphone app daily, women also completed an electronic diary about their activities in the past 24 hours. Finally, women took a urinary luteinizing hormone test at several points in a given cycle to determine the close of the fertile window. We assessed phase-based changes in physiological parameters using cross-classified mixed-effects models with random intercepts and random slopes. We then trained a machine learning algorithm to recognize the fertile window. Results: We have demonstrated that wearable technology can detect significant, concurrent phase-based shifts in WST, heart rate, and respiratory rate (all P<.001). HRV and skin perfusion similarly varied across the menstrual cycle (all P<.05), although these effects only trended toward significance following a Bonferroni correction to maintain a family-wise alpha level. Our findings were robust to daily, individual, and cycle-level covariates. Furthermore, we developed a machine learning algorithm that can detect the fertile window with 90% accuracy (95% CI 0.89 to 0.92). Conclusions: Our contributions highlight the impact of artificial intelligence and machine learning's integration into health care. By monitoring numerous physiological parameters simultaneously, wearable technology uniquely improves upon retrospective methods for fertility awareness and enables the first real-time predictive model of ovulation. © 2019 Journal of Medical Internet Research. All rights reserved.","Algorithms
49	Sankaran S., Luyten K., Hansen D., Dendale P., Coninx K.,"**Enhancing Patient Motivation through Intelligibility in Cardiac Tele-rehabilitation**",**2019**,"https://www.scopus.com/inward/record.uri?eid=2-s2.0-85083759975&doi=10.1093%2fiwc%2fiwz008&partnerID=40&md5=983be9854c6074c1ac0b35712dfd4078", "Physical exercise training and medication compliance are primary components of cardiac rehabilitation. When rehabilitating independently at home, patients often fail to comply with their prescribed medication and find it challenging to interpret exercise targets or be aware of the expected efforts. Our work aims to assist cardiac patients in understanding their condition better, promoting medication adherence and motivating them to achieve their exercise targets in a tele-rehabilitation setting. We introduce a patient-centric intelligible visualization approach to present prescribed medication and exercise targets to patients. We assessed efficacy of intelligible visualizations on patients' comprehension in two lab studies. We evaluated the impact on patient motivation and health outcomes in field studies. Patients were able to adhere to medication prescriptions, manage their physical exercises, monitor their progress and gained better self-awareness on how they achieved their rehabilitation targets. Patients confirmed that the intelligible visualizations motivated them to achieve their targets better. We observed an improvement in overall physical activity levels and health outcomes of patients. Research Highlights: Presents challenges currently faced in cardiac tele-rehabilitation. Demonstrates how intelligibility was applied to two core aspects of cardiac rehabilitation- promoting medication adherence and physical exercise training. Lab., field and clinical studies to demonstrate efficacy of intelligible visualization, impact on patient motivation and resultant health outcomes. Reflection on how similar HCI approaches could be leveraged for technology-supported management of critical health conditions such as cardiac diseases. © 2019 The Author(s) 2019. Published by Oxford University Press on behalf of The British Computer Society. All rights reserved. For Permissions, please email: journals.permissions@oup.com."
50	Westland H., Sluiter J., Te Dorsthorst S., Schröder C.D., Trappenburg J.C.A., Vervoort S.C.J.M., Schuurmans M.J.,"**Patients' experiences with a behaviour change intervention to enhance physical activity in primary care: A mixed methods study**",**2019**,"https://www.scopus.com/inward/record.uri?eid=2-s2.0-85061495300&doi=10.1371%2fjournal.pone.0212169&partnerID=40&md5=964d024044b9432ffee6ac9a621a0c4a", "Objective :To explore the experiences of patients at risk for cardiovascular disease in primary care with the Activate intervention in relation to their success in increasing their physical activity. Methods: A convergent mixed methods study was conducted, parallel to a cluster-randomised controlled trial in primary care, using a questionnaire and semi-structured interviews. Questionnaires from 67 patients were analysed, and semi-structured interviews of 22 patients were thematically analysed. Experiences of patients who had objectively increased their physical activity (responders) were compared to those who had not (non-responders). Objective success was analysed in relation to self-perceived success. Results: The questionnaire and interview data corresponded, and no substantial differences among responders and non-responders emerged. Participating in the intervention increased patients' awareness of their physical activity and their physical activity level. Key components of the intervention were the subsequent support of nurses with whom patients' have a trustful relationship and the use of self-monitoring tools. Patients highly valued jointly setting goals, planning actions, receiving feedback and review on their goal attainment and jointly solving problems. Nurses' support, the use of self-monitoring tools, and involving others incentivised patients to increase their physical activity. Internal circumstances and external circumstances challenged patients' engagement in increasing and maintaining their physical activity. Conclusion: Patients experienced the Activate intervention as valuable to increase and maintain their physical activity, irrespective of their objective change in physical activity. The findings enable the understanding of the effectiveness of the intervention and implementation in primary care. © 2019 Westland et al. This is an open access article distributed under the terms of the Creative Commons Attribution License, which permits unrestricted use, distribution, and reproduction in any medium, provided the original author and source are credited.",
51	Nepper M.J., McAtee J.R., Wheeler L., Chai W.,"**Mobile phone text message intervention on diabetes self-care activities, cardiovascular disease risk awareness, and food choices among type 2 diabetes patients**",**2019**,"https://www.scopus.com/inward/record.uri?eid=2-s2.0-85068485051&doi=10.3390%2fnu11061314&partnerID=40&md5=399c543635bdd6c148355598bb979937", "This study examines the effects of educational text messages on diabetes self-care activities, cardiovascular disease (CVD) risk awareness, and home food availabilities related to food choices among patients with type 2 diabetes. Quasi-experimental design was used with 40 patients (58.0 ± 10.6 years) in the intervention group and 39 (55.7 ± 12.2 years) in the control group. In addition to the usual care provided for all participants, the intervention group received three educational text messages weekly for 12 weeks. Pre-and post-intervention measures were collected for both groups. Ninety-four percent of the participants receiving text messages indicated the usefulness of this program. The intervention group either maintained the same level or demonstrated small improvements in diabetes self-care activities after the intervention. Significant increases in scores of CVD risk awareness (57% increase<br>p = 0.04) and availabilities of fresh fruits (320% increase p = 0.01) and fresh vegetables (250% increase p = 0.02) in the home and weekly total (16% increase p = 0.006) physical activity levels were observed for the intervention group relative to the control group. The pilot results suggest the feasibility and usefulness of the text message program for diabetes education. The study is registered with Clinical Trials.gov (NCT03039569). © 2019 by the authors. Licensee MDPI, Basel, Switzerland.","Cardiovascular disease risk awareness
52	Mahmood A., Kedia S., Wyant D.K., Ahn S., Bhuyan S.S.,"**Use of mobile health applications for health-promoting behavior among individuals with chronic medical conditions**",**2019**,"https://www.scopus.com/inward/record.uri?eid=2-s2.0-85073613740&doi=10.1177%2f2055207619882181&partnerID=40&md5=ce41df38143b3d15482b1e6b981f720c", "Background: Chronic medical conditions (CCs) are leading causes of morbidity and mortality in the United States. Strategies to control CCs include targeting unhealthy behaviors, often through the use of patient empowerment tools, such as mobile health (mHealth) technology. However, no conclusive evidence exists that mHealth applications (apps) are effective among individuals with CCs for chronic disease self-management. Methods: We used data from the Health Information National Trends Survey (HINTS 5, Cycle 1, 2017). A sample of 1864 non-institutionalized US adults (≥18 years) who had a smartphone and/or a tablet computer and at least one CC was analyzed. Using multivariable logistic regressions, we assessed predisposing, enabling, and need predictors of three health-promoting behaviors (HPBs): tracking progress on a health-related goal, making a health-related decision, and health-related discussions with a care provider among smart device and mHealth apps owners. Results: Compared to those without mHealth apps, individuals with mHealth apps had significantly higher odds of using their smart devices to track progress on a health-related goal (adjusted odds ratio (aOR) 8.74, 95% confidence interval (CI): 5.66–13.50, P <.001), to make a health-related decision (aOR 1.77, 95% CI: 1.16–2.71, P <.01) and in health-related discussions with care providers (aOR 2.0, 95% CI: 1.26–3.19, P <.01). Other significant factors of at least one type of HPB among smart device and mHealth apps users were age, gender, education, occupational status, having a regular provider, and self-rated general health. Conclusion: mHealth apps are associated with increased rates of HPB among individuals with CCs. However, certain groups, like older adults, are most affected by a digital divide where they have lower access to mHealth apps and thus are not able to take advantage of these tools. Rigorous randomized clinical trials among various segments of the population and different health conditions are needed to establish the effectiveness of these mHealth apps. Healthcare providers should encourage validated mHealth apps for patients with CCs. © The Author(s) 2019.","Chronic disease

**53**	Jiang Y., Jiao N., Nguyen H.D., Lopez V., Wu V.X., Kowitlawakul Y., Poo D.C.C., Wang W.,"**Effect of a mHealth programme on coronary heart disease prevention among working population in Singapore: A single group pretest–post-test design** [新加坡工作人口mhealth计划对冠心病预防的影响:单组试验前-试验后设计]",**2019**,"https://www.scopus.com/inward/record.uri?eid=2-s2.0-85065156805&doi=10.1111%2fjan.13980&partnerID=40&md5=ec76b4891c10b805b3fa04b78723a5c5",<br>"Aim: The aim of this study was to develop and examine the effectiveness of a 4-week Mobile Health (mHealth) programme using the smartphone app Care4Heart on awareness, knowledge, perceived stress, and a heart-related lifestyle among working population in Singapore. Design: A quasi-experimental single group pretest and repeated post-test design. Methods: A total of 160 nonhealth-related working adults were recruited from February 2016–April 2016. The Care4Heart app was installed on the participants' phones. Outcome measures, including the Heart Disease Fact Questionnaire-2, the Behavioral Risk Factor Surveillance System, and the Perceived Stress Scale were collected online at baseline, 4th week, and 6th month from baseline. Results: Compared with the baseline, the participants had significantly higher total mean scores for awareness and knowledge of coronary heart disease (CHD) after study intervention at 4 weeks and 6 months from baseline. However, there were no significant differences in perceived stress levels and heart-related lifestyle behaviours before and after the intervention. Conclusions: The study contributed some evidence that the mHealth app helped to improve awareness and knowledge of CHD. Impact: Coronary heart disease remains one of the major causes of death and disability. It imposes a heavy financial burden on working adults. However, working adults were less able to identify the risk factors and adopt healthy lifestyles due to gaps in knowledge and awareness. This study demonstrated some effects of a mHealth programme in improving awareness and knowledge of CHD among the working population. mHealth provides a potential avenue for primary prevention of CHD. © 2019 John Wiley & Sons Ltd","awareness
**54**	Maxwell H., O'Shea M., Stronach M., Pearce S.,"**Empowerment through digital health trackers: an exploration of Indigenous Australian women and physical activity in leisure settings**",**2019**,"https://www.scopus.com/inward/record.uri?eid=2-s2.0-85074038587&doi=10.1080%2f11745398.2019.1674677&partnerID=40&md5=4d8dcca15e073078ac046454146d6d9a","Drawing on a strengths-based empowerment approach and Indigenous traditions of 'yarning', this research explores how digital health technologies might contribute to Indigenous Australian women's increased participation in physical activity in leisure settings. While people have long controlled their bodies, conventionally through diaries and weight scales, digital self-tracking of one's bodily states and activities continues to expand. No previous studies have addressed how these technologies influence physical activity among this population and there is limited research about Indigenous Australian people's leisure experiences and the meanings they attribute to them. Accordingly, this research contributes to an under researched area of health and leisure studies concerned with understanding the social, narrative and affective facets of individuals' practices and experiences. Key findings from the pilot study include intersections between the use of health trackers and Indigenous women's prioritizing time for leisure, increased physical activity, enhanced health literacy, and greater personal accountability for lifestyle choices. © 2019, © 2019 Australia and New Zealand Association of Leisure Studies.","Aboriginal
**55**	Kim E.J., Hwang S.Y.,"**Awareness and utilization of mobile health and preventive health behavior according to cardiovascular risk factor cluster type in early middle-aged male workers**",**2019**,"https://www.scopus.com/inward/record.uri?eid=2-s2.0-85077615413&doi=10.7475%2fkjan.2019.31.5.562&partnerID=40&md5=a59c660554a56766ad610735f1f20708",<br>"Purpose: This study was conducted to identify cardiovascular risk factor cluster types in early middle-aged male workers in their 30s and 40s, and to identify differences in awareness of mobile health and preventive health behaviors by cluster type. Methods: This study adopted a cross-sectional descriptive design. Male workers aged 30~49 years with cardiovascular risk factors (n=166) at three medical device manufacturers in June, 2019 were recruited. Self-reported questionnaires were administered. K-means cluster analysis was performed using four measurement tools: e-health literacy, behavior of seeking health information on the internet, intent to use mobile health, and preventive health behavior. Results: Three cluster groups were identified based on 7 risk factors: ""unhealthy behavior (51.8%)"", ""chronic disease (28.9%)"", and ""dyslipid family history (19.3%)"". In the ""unhealthy behavior"" group where more than 70% of the participants were smoking and drinking heavily, the awareness of mobile health utilization such as behavior of seeking information on the internet and intent to use mobile health, especially usefulness, was significantly lower than that in the other two groups. The preventive health behavior was also the lowest among the three groups. Conclusion: We suggest that when planning for mobile-use cardiovascular prevention education for early middle-aged male workers, it is necessary to consider a cluster of risk factors. Strategies for raising positive awareness of the use of mobile health should be included prior to cardiovascular health education for workers with unhealthy lifestyles such as smoking and excessive drinking alcohol. © 2019 Korean Society of Adult Nursing.","Cardiovascular diseases
**56**	Petsani D., Kostantinidis E.I., Diaz-Orueta U., Hopper L., Bamidis P.D.,"**Extending Exergame-Based Physical Activity for Older Adults: The e-Coaching Approach for Increased Adherence**",**2019**,"https://www.scopus.com/inward/record.uri?eid=2-s2.0-85064879280&doi=10.1007%2f978-3-030-15736-4_6&partnerID=40&md5=b08f80cb2d4bd3704f54e46407466806",<br>"e-Coaching approaches have recently received a lot of attention, as technology led healthy ageing solutions depend on empowering older people's motivation. While the beneficial impact of physical activity for older populations is indisputable, this work aims at presenting first designing considerations to be followed if increased adherence to physical activity through an e-coaching system were to be aimed for older adults. The work plan kicks-off on the basis of an existing exergame platform, especially designed and widely tested for older adults (webFitForAll) which is forced to align with notion of behavior change techniques (BCTs) that have been developed for physical activity enhancement. New advances in micro-projector technologies provide extra opportunities for tweaking the accessibility burden while augmenting and blending the real with the coaching environment. Quite reasonably, these are not easy tasks to follow and success depends much on multi-disciplinary approaches encompassing new ideas from co-creation and co-design with the actual users. © 2019, Springer Nature Switzerland AG.","e-Coaching
**57**	Douka S., Zilidou V.I., Lilou O., Tsolaki M.,"**Greek traditional dances: A way to support intellectual, psychological, and motor functions in senior citizens at risk of neurodegeneration**",**2019**,"https://www.scopus.com/inward/record.uri?eid=2-s2.0-85064246129&doi=10.3389%2ffnagi.2019.00006&partnerID=40&md5=bc150afca899e87767a57bcb2ebc3d57","One of the major problems that elderly people are facing is dementia. For scientist's dementia is a medical, social and economic problem, as it has been characterized as the epidemic of the 21st century. Prevention and treatment in the initial stages of dementia are essential, and community awareness and specialization of health professionals are required, with the aim of early and valid diagnosis of the disease. Activities are recommended to the senior citizens to improve their physical and mental health. Dance has been suggested as an appropriate recreational activity for the elderly that brings functional adjustments to the various systems of the body, psychological benefits, and makes exercise to seem interesting and entertaining as it combines the performance of multiple animations with musical accompaniment. A Greek traditional dance program was performed where our sample consisted of 30 healthy elderly and 30 with Mild Cognitive Impairment - MCI. It lasted 24 weeks, two times a week for 60 min. Specific traditional dances from all over Greece were selected. The dances were of a moderate intensity at the beginning with a gradual increase in intensity, according to the age and physical abilities of the participants. The results showed a significant improvement in: attention (S4viac-Healthy: z = -3.085, p = 0.002<br>MCI: z = -3.695, p < 0.001, S4viti-Healthy: z = -2.800, p = 0.005 MCI: z = -3.538, p < 0.001), anxiety (Healthy: z = -2.042, p = 0.041
**58**	Choi B., Li Z.,"**The longitudinal effects of social sharing on physical health**",**2019**,"https://www.scopus.com/inward/record.uri?eid=2-s2.0-85089209608&partnerID=40&md5=ed121518179c8989ac796351a4b092a7",<br>A key strategy to combat sedentary lifestyle is to encourage fitness exercises. Traditional wellness and lifestyle management programs focus on inducing health awareness and providing fitness activity support. The growing pervasive use of smartphones allows individualized and cost-effective digital wellness programs to be administrated through mobile fitness apps. Drawing on the self-promotion literature, this paper elucidates the effects of mobile fitness app on health outcomes. Specifically, this study examines a social feature of mobile fitness app usage - social sharing. The results of our longitudinal field experiment reveal strong evidence that mobile fitness apps help improve health outcomes. Furthermore, compared the absence of social sharing, social sharing leads to lower body fatness, however the effects of social sharing diminish over time. © Proceedings of the 23rd Pacific Asia Conference on Information Systems: Secure ICT Platform for the 4th Industrial Revolution, PACIS 2019.","Field experiment
**59**	Joshi P., Shukla S.,"**Child development and education in the twenty-first century**",**2019**,"https://www.scopus.com/inward/record.uri?eid=2-s2.0-85084888043&doi=10.1007%2f978-981-13-9258-0&partnerID=40&md5=9f008004283a0d589d1d4706a0e61bc3",<br>"Introduction This book provides an in-depth understanding of how children's development at different stages of their lives interfaces with the kind of education and support they need at school and home. It examines closely how education, in turn, influences their development and prepares them for an uncertain future. The chapters focus on the rapid developments of the 21st century that are changing the nature of education, especially the shift needed to being able to sift through and meaningfully deal with overwhelming volumes of information now available. This book helps readers understand how children can benefit from the digital environment while avoiding its pitfalls. Keeping in mind that in today's world parents are getting to spend less time with their children, the authors provide research-backed ideas on how they can best enable children's development, including their thoughts, feelings and notions of self. Given the increasing disparities, there is a perceptive analysis of how education can build an awareness of equity in a context marked by diversity and disadvantage. This book addresses issues such as these in a reliable, scholarly yet accessible manner, for students, young researchers and lay readers. Consequently, it is a valuable source of fundamental insights and understanding for educators, policy-makers, educational administrators and students of human and child development, education, and teacher training courses. © Springer Nature Singapore Pte Ltd. 2019.","21st century learning needs

**60**	Carter D.D., Robinson K., Forbes J., Hayes S.,"**Experiences of mobile health in promoting physical activity: A qualitative systematic review and meta-ethnography**",**2018**,"https://www.scopus.com/inward/record.uri?eid=2-s2.0-85058570784&doi=10.1371%2fjournal.pone.0208759&partnerID=40&md5=209e5d9c70581cb9f155af62cd40ab43", "Objective Despite evidence supporting physical activity in primary and secondary prevention, many individuals do not meet recommended levels. Mobile health is a field with a growing evidence base and is proposed as a convenient method for delivering health interventions. Despite qualitative exploration of stakeholder perspectives, there is a lack of synthesis to inform evidence-based design. This study aims to resolve this by identifying and synthesising qualitative research on the experience of using mobile health applications to promote physical activity. Method A systematic review focused on qualitative research, mobile health and physical activity was conducted in October 2017 using CINAHL, ERIC, EMBASE, MEDLINE and PsycINFO databases. The protocol was registered with the Prospero database (Registration: CRD4201808 0610). Results were synthesised as a meta-ethnography. Results Fifteen studies were included, covering a variety of populations, including people with diabetes, obesity, and serious mental illness. Five themes emerged: (a) personal factors and the experience of using mobile health, (b) mobile health and changes in thinking that support physical activity, (c) the experience of mobile health features, including prompts, goal setting and gamification, (d) the experience of personalised mobile health and physical activity, (e) technical and user issues in mobile health and their effect on experience. Conclusion Personal factors and features of the device influenced the experience of using mobile health to support physical activity. The two mechanisms through which mobile health use facilitated physical activity were strengthening of motivation and changes in self-awareness and strategising. Experiences were not entirely unproblematic as technical issues and adverse effects related to self-monitoring were noted. This synthesis provides insight into the experience of mobile health and is useful for researchers and healthcare practitioners interested in designing user-informed mobile health interventions for promoting physical activity. © 2018 Carter et al. This is an open access article distributed under the terms of the Creative Commons Attribution License, which permits unrestricted use, distribution, and reproduction in any medium, provided the original author and source are credited.",
**61**	Brakenridge C.L., Healy G.N., Hadgraft N.T., Young D.C., Fjeldsoe B.S.,"**Australian employee perceptions of an organizational-level intervention to reduce sitting**",**2018**,"https://www.scopus.com/inward/record.uri?eid=2-s2.0-85052868094&doi=10.1093%2fheapro%2fdax037&partnerID=40&md5=fc5820f1cac136cc97f7026a4ee9704f","Stand Up Lendlease—a cluster-randomized trial targeting reductions in sitting time in Australian office workers (n ¼ 153, 18 manager-led teams, 1 organization)—effectively reduced sitting time during work hours and across the day after 12 months. The trial included two arms: organizational-support strategies (e.g. manager support, emails) with or without an activity tracker. The current study aimed to examine participant perceptions of the intervention, and perceived barriers and facilitators for reducing sitting time. Telephone interviews (n ¼ 50 participants conducted at 6–10 months) and three focus groups (n ¼ 21 participants conducted at 16 months) evaluated the intervention with qualitative data analysed thematically. Several consistent themes emerged across both short and long-term time points and intervention groups. Support and role modelling of desired behaviours from important organization personnel and receiving feedback on sitting levels were key drivers of change. Improvements in awareness about sitting, and workplace culture changes supporting active work practices were positive impacts of the intervention, but some participants also reported that initial cultural effects had dissipated and the intervention needed 'reinvigoration'. Participants desired additional 'tools' to maintain sitting less and being active, such as sit–stand desks, standing meeting tables and activity trackers. In summary, the intervention raised awareness and initiated cultural changes towards active work practices, however, additional support may be required to maintain changes in organizational culture long term. Practical tools to support sitting changes, organizational and management support and role modelling, as well as ongoing 'reinvigoration' are key strategies for short and long-term intervention success in office workplaces. © The Author 2018. Published by Oxford University Press. All rights reserved.","Intervention
**62**	Besenyi G.M., Schooley B., Turner-McGrievy G.M., Wilcox S., Wilhelm Stanis S.A., Kaczynski A.T.,"**The electronic community park audit tool (eCPAT): Exploring the use of mobile technology for youth empowerment and advocacy for healthy community policy, systems, and environmental Change**",**2018**,"https://www.scopus.com/inward/record.uri?eid=2-s2.0-85059230531&doi=10.3389%2ffpubh.2018.00332&partnerID=40&md5=013ec7422539ad7fc9170a49c7979c9a","Empowering and engaging youth in advocacy and participatory action research (PAR) for healthy community environments is an emerging approach to reducing the childhood obesity epidemic. Technology is a promising strategy for engaging youth in such efforts. The Community Park Audit Tool (CPAT) is user-friendly tool for evaluating the ability of parks to promote youth physical activity. Recently an electronic version of the tool (eCPAT) was developed and validated. The purpose of this study was to explore the use of eCPAT mobile technology on youth empowerment and advocacy. This study examined tool usability, youths' technology access, use, and readiness for PAR efforts, effectiveness of mobile technology on youth empowerment and advocacy, interaction effects between tool format and regular technology use, and tool format preferences. Youth ages 11-18 years were recruited and randomized into one of three study conditions: Control (no audit), paper (CPAT), and mobile technology (eCPAT). Intervention youth completed two park audits using assigned format. A subsample of youth in the Control group completed both CPAT and eCPAT audits for comparison. Independent samples t-tests and MANCOVAs explored differences in post-project levels of tool usability and empowerment and advocacy scores between groups. Multivariate linear regression analysis explored the interaction between Control, Paper, or eCPAT group membership and mean technology use in predicting empowerment and advocacy. Youth (n = 124) completed pre and post surveys. The majority of youth had access to technology (smartphone 77.4%, tablet/iPad 67.7%). Youth used mobile technology at least once a day to use apps (M = 7.8, SD = 3.2), browse the web (M = 6.3, SD = 3.3), and search for information (M = 6.3, SD = 3.5). Youth were also ready and willing to use technology for PAR (M = 3.42-3.59). No main or interaction effects were found for post-project levels of youth empowerment or advocacy. However, the eCPAT tool had high usability scores, was better liked, and was preferred by youth over paper-pencil methods. Mobile technologies are ubiquitous and a preferred strategy among youth for engagement in community change. Future studies should explore mobile technology as a potential strategy for engaging youth in ongoing PAR efforts to achieve successful engagement and advocacy in community healthy environmental change. © 2018 Besenyi, Schooley, Turner-McGrievy, Wilcox, Wilhelm Stanis and Kaczynski.","Advocacy
**63**	Eanes L.,"**CE: Too much sitting: A newly recognized health risk**",**2018**,"https://www.scopus.com/inward/record.uri?eid=2-s2.0-85052734253&doi=10.1097%2f01.NAJ.0000544948.27593.9b&partnerID=40&md5=82639dc251bbc14d990057bf8a126259", "While moderate-to-vigorous physical activity (MVPA) has been widely accepted as a major factor in promoting optimal health, emerging research specific to sedentary behavior suggests that MVPA alone may not be enough. This integrative literature review examines the evidence on sedentary behavior as an independent health risk for cardiometabolic health conditions, certain cancers, and all-cause mortality. In so doing, it reveals new insights into high-volume sitting and prolonged uninterrupted sitting and their relationship to adverse health conditions in order to increase awareness of sedentary behavior as an independent health risk factor, examine the potential effects of displacing sedentary time with light-intensity physical activity, and encourage nurses to advance the overall reduction of sedentary behavior. © 2018 Lippincott Williams and Wilkins. All rights reserved.","high-volume sitting
**64**	Baharum A., Pitchay S.A., Ismail R., Fabeil N.F., Rusli N.M., Bahar I.A.A.,"**Demuse: Releasing stress using music mobile application**",**2018**,"https://www.scopus.com/inward/record.uri?eid=2-s2.0-85051140150&doi=10.1109%2fICTC.2017.8191001&partnerID=40&md5=30f0adb8ea4ffba1d8a108019d24897d", "It can be seen that, conflicts, negative revolution, suicides, and other crimes becoming more common worldwide. Several studies and investigations have been conducted due to this case. Thus, it has been found that one of the root cause is stress, especially among the youth. Although stress can improve work performance and awareness for those who can manage it properly, however if someone is unable to cope with the stressful situation when it becomes excessive, the reaction might be disastrous. In tackling this unfavourable situation, several lifestyle changes have been prescribed such as listening to music, physical activities, doing desired activities, surfing, and others. This study uses the power of music to reduce stress. A mobile application named as "DeMuse" was developed and in its development, Mobile-D step-by-step methodology was applied. At explore phase, a number of existing applications have been compared. At the second phase, the initialize stage, a quantitative analysis was carried out to study the music and mood categories respectively. During the third and fourth phases, which were Productionize and Stabilise, the completion of Data Flow Diagram and Entity Relationship Diagram were established based on the quantitative analysis done. In the final phase, the System Test and Fix, the prototype were reviewed by 148 potential users. DeMuse showed to be one of the alternative ways to relieve stress. From this finding, DeMuse highlight the main feature which is the music and mood categories. In conclusion, DeMuse is a valid mobile apps that could be used to help reduce stress of its user. With this app, it hopes greatly to help in decreasing and eliminating the tension, dissatisfaction, and others negative feelings of users in their daily life. © 2005 – on going JATIT & LLS.","Mobile application
**65**	Holmberg C., Larsson C., Korp P., Lindgren E.-C., Jonsson L., Fröberg A., Chaplin J.E., Berg C.,"**Empowering aspects for healthy food and physical activity habits: adolescents' experiences of a school-based intervention in a disadvantaged urban community**",**2018**,"https://www.scopus.com/inward/record.uri?eid=2-s2.0-85049570269&doi=10.1080%2f17482631.2018.1487759&partnerID=40&md5=43e783c1806ccbe84b026f105f4451b5", "Purpose:This study aimed to describe adolescents' experiences of participating in a health-promoting school-based intervention regarding food and physical activity, with a focus on empowering aspects. Method: The school was located in a urban disadvantaged community in Sweden, characterized by poorer self-reported health and lower life expectancy than the municipality average. Focus group interviews with adolescents (29 girls, 20 boys, 14–15 years) and their teachers (n = 4) were conducted two years after intervention. Data were categorized using qualitative content analysis. Results: A theme was generated, intersecting with all the categories: Gaining control over one's health: deciding, trying, and practicing together, in new ways, using reflective tools. The adolescents appreciated influencing the components of the intervention and collaborating with peers in active learning activities such as practicing sports and preparing meals. They also reported acquiring new health information, that trying new activities was inspiring, and the use of pedometers and photo-food diaries helped them reflect on their health behaviours. The adolescents' experiences were also echoed by their teachers. Conclusions: To facilitate empowerment and stimulate learning, health-promotion interventions targeting adolescents could enable active learning activities in groups, by using visualizing tools to facilitate self-reflection, and allowing adolescents to influence intervention activities. © 2018, © 2018 The Author(s). Published by Informa UK Limited, trading as Taylor & Francis Group.","Adolescence

**66**	Dietrich D., Dekova R., Davy S., Fahrni G., Geissbühler A.,"**Applications of space technologies to global health: Scoping review**",**2018**,"https://www.scopus.com/inward/record.uri?eid=2-s2.0-85054987274&doi=10.2196%2fjmir.9458&partnerID=40&md5=79b98828772f73429bab3cf7e0cf2850", "Background: Space technology has an impact on many domains of activity on earth, including in the field of global health. With the recent adoption of the United Nations' Sustainable Development Goals that highlight the need for strengthening partnerships in different domains, it is useful to better characterize the relationship between space technology and global health. Objective: The aim of this study was to identify the applications of space technologies to global health, the key stakeholders in the field, as well as gaps and challenges. Methods: We used a scoping review methodology, including a literature review and the involvement of stakeholders, via a brief self-administered, open-response questionnaire. A distinct search on several search engines was conducted for each of the four key technological domains that were previously identified by the UN Office for Outer Space Affairs' Expert Group on Space and Global Health (Domain A: Remote sensing<br>Domain B: Global navigation satellite systems Domain C: Satellite communication and Domain D: Human space flight). Themes in which space technologies are of benefit to global health were extracted. Key stakeholders, as well as gaps, challenges, and perspectives were identified. Results: A total of 222 sources were included for Domain A, 82 sources for Domain B, 144 sources for Domain C, and 31 sources for Domain D. A total of 3 questionnaires out of 16 sent were answered. Global navigation satellite systems and geographic information systems are used for the study and forecasting of communicable and noncommunicable diseases satellite communication and global navigation satellite systems for disaster response and global navigation satellite systems for autonomy improvement, access to health care, as well as for safe and efficient transportation. Various health research and technologies developed for inhabited space flights have been adapted for terrestrial use. Conclusions: Although numerous examples of space technology applications to global health exist, improved awareness, training, and collaboration of the research community is needed. © 2018 Journal of Medical Internet Research. All rights
**67**	Gupta A., Heng T., Shaw C., Li L., Feehan L.,"**Designing pervasive technology for physical activity self-management in arthritis patients**",**2018**,"https://www.scopus.com/inward/record.uri?eid=2-s2.0-85055719770&doi=10.1145%2f3240925.3240956&partnerID=40&md5=18917b94f2ef12dba6e0df6bbec31ce9","reduces the quality of life. A physically active lifestyle is crucial for the successful management of the disease. Pervasive technology such as activity trackers can make patients more aware of their physical activity (PA), and help clinicians in getting an objective view of their patients' lifestyle.We developed a web application called FitViz which gathers data from an arthritis patient's Fitbit device and allows her clinician to use this data in setting personalized goals for the patient.We conducted a pilot study with 10 knee Osteoarthritis patients and 10 Rheumatoid Arthritis patients to test the feasibility of the application. 11 participants were interviewed to share their experiences after using FitViz for a month. The use of pervasive technology - Fitbit and FitViz - increased PA awareness, and helped in realistic goal-setting. Participants expressed different emotions - including mistrust in technology - concerning goal achievement.We use these findings to draw design implications for future pervasive technologies for arthritis patients. © 2018 Copyright held by the owner/author(s).","Arthritis
**68**	Barroso M., Pérez-Fernández S., Vila M.M., Zomeño M.D., Martí-Lluch R., Cordon F., Ramos R., Elosua R., Degano I.R., Fitó M., Cabezas C., Salvador G., Castell C., Grau M.,"**Validity of a method for the self-screening of cardiovascular risk**",**2018**,"https://www.scopus.com/inward/record.uri?eid=2-s2.0-85047755850&doi=10.2147%2fCLEP.S158358&partnerID=40&md5=408afe78a7c531a92f3a4b686ffdce65", "Background: The validity of a cardiovascular risk self-screening method was assessed. The results obtained for self-measurement of blood pressure, a point-of-care system's assessment of lipid profile and glycated hemoglobin, and a self-administered questionnaire (sex, age, diabetes, tobacco consumption) were compared with the standard screening (gold standard) conducted by a health professional. Methods: Crossover clinical trial on a population-based sample from Girona (north-eastern Spain), aged 35–74, with no cardiovascular disease at recruitment. Participants were randomized to one of the two risk assessment sequences (standard screening followed by self-screening or vice versa). Cardiovascular risk was estimated with the Framingham-REGICOR function. Concordance between methods was estimated with the intraclass correlation coefficient (ICC). Sensitivity, specificity, and positive and negative predictive values were estimated, considering 5% cardiovascular risk as the cutoff point. ClinicalTrials.gov Registration #NCT02373319. Clinical Research Ethic Committee of the Parc de Salut Mar Registration #2014/5815/I. Results: The median cardiovascular risk in men was 2.56 (interquartile range: 1.42–4.35) estimated by standard methods and 2.25 (1.28–4.07) by self-screening with ICC=0.92 (95% CI: 0.90–0.93). In women, the cardiovascular risk was 1.14 (0.61–2.10) by standard methods and 1.10 (0.56–2.00) by self-screening, with ICC=0.89 (0.87–0.90). The sensitivity, specificity, and positive and negative predictive values for the self-screening method were 0.74 (0.63–0.82), 0.97 (0.95–0.99), 0.86 (0.77–0.93), and 0.94 (0.91–0.96), respectively, in men. In women, these values were 0.50 (0.30–0.70), 0.99 (0.98–1), 0.81 (0.54–0.96), and 0.97 (0.95–0.99), respectively. Conclusion: The self-screening method for assessing cardiovascular risk provided similar results to the standard method. Self-screening had high clinical performance to rule out intermediate or high cardiovascular risk. © 2018 Barroso et al.","Cardiovascular diseases
**69**	Gupta S., Shapiro A.D.,"**Optimizing bleed prevention throughout the lifespan: Womb to Tomb**",**2018**,"https://www.scopus.com/inward/record.uri?eid=2-s2.0-85048009543&doi=10.1111%2fhae.13471&partnerID=40&md5=4aa49bd2f5d1d4325dcaac410077a9ac", "The focus of care providers, patients and families is the ability to tailor care for persons with haemophilia (PWH) across the lifespan. Care requires knowledge of the bleeding disorder and age-related complications, risk of therapeutic interventions, and evaluation of individual characteristics that contribute to outcomes. The ultimate goal is to live a normal life without the burden of bleeding, for PWH and carriers. A wide range of therapeutic options is required to achieve personalized care. Over the last decade, substantial therapeutic advantages have been achieved in the treatment of haemophilia that include the development of a robust array of factor concentrates, novel haemostatic agents, and increased knowledge and awareness of disease associated outcomes and risk factors. Significant strides on the road to accessible gene therapy have been realized. This increased range of therapeutic modalities provides options for development and implementation of care plans for each patient at each stage of life that are more flexible compared to prior care regimens. Paradigms for management of haemophilia are changing. As a community, we must work together to use these resources wisely, to learn from outcomes with new therapies and diagnostic tools, to assure all patients can achieve improved care and outcomes regardless of disease state or country of origin. © 2018 John Wiley & Sons Ltd","gene therapy
**70**	Hallam K.T., Bilsborough S., de Courten M.,"""**Happy feet""**: Evaluating the benefits of a 100-day 10,000 step challenge on mental health and wellbeing**",**2018**,"https://www.scopus.com/inward/record.uri?eid=2-s2.0-85043454271&doi=10.1186%2fs12888-018-1609-y&partnerID=40&md5=38051a9f7e3d93527a74aa717edd742a", "Background: An increased awareness of the health benefits of walking has emerged with the development and refinement of accelerometer equipment. Evidence is beginning to highlight the value of promoting walking, particularly focusing on the Japanese mark of obtaining 10,000 steps per day. Workplace based step challenges have become popular to engage large cohorts in increasing their daily physical activity in a sustainable and enjoyable way. Findings are now highlighting the positive health effects of these medium-term programs (typically conducted over a few months) in terms of cardiovascular health, reducing diabetes risk and improving lifestyle factors such as weight and blood pressure. As yet, research has not focused on whether similar improvements in psychological health and wellbeing are present. Methods: This study investigated the impact of a 100-day, 10,000 step program on signs of depression, anxiety and stress as well as general wellbeing using standardised psychological scales. Results: The results indicated a small but consistent effect on all of these measures of mental health over the term of the program. This effect appeared irrespective of whether a person reached the 10,000 step mark. Conclusions: These results highlight improved mental health and wellbeing in people undertaking this 100-day 10,000 step program and indicates the efficacy and potential of these programs for a modest, yet important improvement in mental health. Notably, targets reached may be less important than participation itself. © 2018 The Author(s).","Anxiety
**71**	van Ommen B., Wopereis S., van Empelen P., van Keulen H.M., Otten W., Kasteleyn M., Molema J.J.W., de Hoogh I.M., Chavannes N.H., Numans M.E., Evers A.W.M., Pijl H.,"**From diabetes care to diabetes cure-the integration of systems biology, ehealth, and behavioral change**",**2018**,"https://www.scopus.com/inward/record.uri?eid=2-s2.0-85041126365&doi=10.3389%2ffendo.2017.00381&partnerID=40&md5=9aec0bbd08b8e918ada8d823d9bf6b09", "From a biological view, most of the processes involved in insulin resistance, which drives the pathobiology of type 2 diabetes, are reversible. This theoretically makes the disease reversible and curable by changing dietary habits and physical activity, particularly when adopted early in the disease process. Yet, this is not fully implemented and exploited in health care due to numerous obstacles. This article reviews the state of the art in all areas involved in a diabetes cure-focused therapy and discusses the scientific and technological advancements that need to be integrated into a systems approach sustainable lifestyle-based healthcare system and economy. The implementation of lifestyle as cure necessitates personalized and sustained lifestyle adaptations, which can only be established by a systems approach, including all relevant aspects (personalized diagnosis and diet, physical activity and stress management, self-empowerment, motivation, participation and health literacy, all facilitated by blended care and ehealth). Introduction of such a systems approach in type 2 diabetes therapy not only requires a concerted action of many stakeholders but also a change in healthcare economy, with new winners and losers. A ""call for action"" is put forward to actually initiate this transition. The solution provided for type 2 diabetes is translatable to other lifestyle-related disorders. © 2018 van Ommen, Wopereis, van Empelen, van Keulen, Otten, Kasteleyn, Molema, de Hoogh, Chavannes, Numans, Evers and Pijl.","Cure

**72**	Reis A., Paulino D., Martins P., Paredes H., Barroso J.,"**eHealth context inference a review of open source frameworks initiatives**",**2018**,"https://www.scopus.com/inward/record.uri?eid=2-s2.0-85051744749&doi=10.5220%2f0006752707070714&partnerID=40&md5=7953b0ade28adaf3a1182b1593fba531",<br>"The collection of health and fitness longitudinal data can be used to model disease progression and shape new algorithms to diagnose and predict health hazards. Continuously tracking vital signs, in particular heart rate and skin temperature, can be very informative by using models and algorithms to predict and notify the user about when he might be falling ill. With the current wearable devices and the proper algorithms, the individual can be permanently monitored, which might be much more interesting than a one-off reading comparison with the population average, made by a doctor. It would be possible to intervene earlier and to prevent somebody from becoming seriously ill. From a broader perspective, the knowledge about a user's health can be considered as an element of that user's context and be used by context aware applications to provide higher value to the user. After the trivialization of the data acquisition sensors, wearable devices, and raw data, the next logical step is the development of contained software components that can infer and produce knowledge from the raw data. These components and the knowledge they produce can be used by all sorts of applications in order to further customize their usage by a specific user. Customization and context awareness, in regard to health, is a wide field for research and there are a multitude of proposals for models and algorithms. In this review work we searched for software components (frameworks, software libraries, etc.), freely available and that can be used as building blocks for other computer systems by software developers. Copyright © 2018 by SCITEPRESS – Science and Technology Publications, Lda. All rights reserved.","Context Awareness
**73**	Daykin N., Mansfield L., Meads C., Julier G., Tomlinson A., Payne A., Grigsby Duffy L., Lane J., D'Innocenzo G., Burnett A., Kay T., Dolan P., Testoni S., Victor C.,"What works for wellbeing? A systematic review of wellbeing outcomes for music and singing in adults",**2018**,"https://www.scopus.com/inward/record.uri?eid=2-s2.0-85039924262&doi=10.1177%2f1757913917740391&partnerID=40&md5=775c78ca96ba7df07f4b06d989650684",<br>"Aims: The role of arts and music in supporting subjective wellbeing (SWB) is increasingly recognised. Robust evidence is needed to support policy and practice. This article reports on the first of four reviews of Culture, Sport and Wellbeing (CSW) commissioned by the Economic and Social Research Council (ESRC)-funded What Works Centre for Wellbeing (https://whatworkswellbeing.org/). Objective: To identify SWB outcomes for music and singing in adults. Methods: Comprehensive literature searches were conducted in PsychInfo, Medline, ERIC, Arts and Humanities, Social Science and Science Citation Indexes, Scopus, PILOTS and CINAHL databases. From 5,397 records identified, 61 relevant records were assessed using GRADE and CERQual schema. Results: A wide range of wellbeing measures was used, with no consistency in how SWB was measured across the studies. A wide range of activities was reported, most commonly music listening and regular group singing. Music has been associated with reduced anxiety in young adults, enhanced mood and purpose in adults and mental wellbeing, quality of life, self-awareness and coping in people with diagnosed health conditions. Music and singing have been shown to be effective in enhancing morale and reducing risk of depression in older people. Few studies address SWB in people with dementia. While there are a few studies of music with marginalised communities, participants in community choirs tend to be female, white and relatively well educated. Research challenges include recruiting participants with baseline wellbeing scores that are low enough to record any significant or noteworthy change following a music or singing intervention. Conclusions: There is reliable evidence for positive effects of music and singing on wellbeing in adults. There remains a need for research with sub-groups who are at greater risk of lower levels of wellbeing, and on the processes by which wellbeing outcomes are, or are not, achieved. © 2017, © Royal Society for Public Health 2017.","depression
**74**	Ahmed H.O., Hama Marif M.A., sabah abid abdulhakim, Ali Omer M.A., majeed nuri D.A., Hamasur A.F., Ahmed S.H., Abddalqadir K.M.,"**The life styles causing overweight or obesity: Based on 5 years of experience in two centers in Sulaimani Governorate, Kurdistan Region/Iraq**",**2018**,"https://www.scopus.com/inward/record.uri?eid=2-s2.0-85046474685&doi=10.1016%2fj.ijso.2018.04.002&partnerID=40&md5=164c856f20e32f2f4b003f56532fc1ba",<br>"Background: One of the top worldwide health problems is overweight and obesity, Body weight gain over a number of years could be achieved through cumulative positive energy balances, which may be the result of habitual behaviors adopted through personal choice. Despite the increased in physical activity, the percentage of people who are overweight has increased, after decades-old efforts and awareness on the issue to nudge consumers towards healthier lifestyles through dietary guidelines, diet-related diseases are still on the rise. Is there potential variables need to be evaluated for any relation with gaining weight and obesity in the patient's life styles? Objectives: Exploration of the possible factors in the life style of overweight and obese patients, resulting in weight gain. Patients and Methods: A prospective randomized study including 440 overweight or obese patients from a total of 689 patients, who consulted for advice, diet and or drugs and various bariatric operations. This was conducted in the period of 5 years from January 2, 2012 to October 1, 2017. For the information collection, each patient was interviewed face-to-face, by three Surgical Board Trainees were interviewing patients face-to-face to fill an originally-designed questionnaire in English translated to the Kurdish language. The questionnaire was composed of demographic data, medical data, biological data, and potential factors of their overweight or obesity. A comparison was made between these patients in the conception of case matching to 446 patients who were of normal body weight, and a normal BMI. Results: Most of the patients (females n = 200, 86.95% and males n = 167, 79.52%) in group A, while (females n = 173, 82.77%) from group B patients were drinking water 60 min after every meals. Nearly half of the patients have a Television in their kitchen/dining room (n = 239/440, 54.39%), however only (51.59%), (13.90%) of the patients from group A, and B respectively were watching TV while eating, A great number of the patients (Group A: n = 358, 81.36%) were operating their computers while eating, versus minority (n = 53, 11.88%), in Group B, and majority of the patients in group A were using their smart phones or making phone calls while eating (n = 390/440, 88.63%) versus small number (n = 49/460, 10.65%) in group B. Conclusion: Excessive habitual usage of a cell phone, early drinking (within 30 min) of water or other liquids after meals may be the potential factors in the initiation of becoming overweight and obese. University students living in the areas where entertainments are not affordable for all, the only remaining entertainment is using cellphones for social media and communication may be more vulnerable to gain weight, overweight and obesity. © 2018 The Authors","Cellphone
**75**	Deshmukh S.K., Srivastava S.K., Tyagi N., Ahmad A., Singh A.P., Ghadhban A.A., Dyess D.L., Carter J.E., Dugger K., Singh S.,"**Emerging evidence for the role of differential tumor microenvironment in breast cancer racial disparity: A closer look at the surroundings**",**2017**,"https://www.scopus.com/inward/record.uri?eid=2-s2.0-85028377243&doi=10.1093%2fcarcin%2fbgx037&partnerID=40&md5=ae1a2cc3982d16991f4c84b3d2a03d0b",<br>"Although increased awareness leading to early detection and prevention, as well as advancements in treatment strategies, have resulted in superior clinical outcomes, African American women with breast cancer continue to have greater mortality rates, compared to Caucasian American counterparts. Moreover, African American women are more likely to have breast cancer at a younger age and be diagnosed with aggressive tumor sub-types. Such racial disparities can be attributed to socioeconomic differences, but it is increasingly being recognized that these disparities may indeed be due to certain genetic and other non-genetic biological differences. Tumor microenvironment, which provides a favorable niche for the growth of tumor cells, is comprised of several types of stromal cells and the various proteins secreted as a consequence of bi-directional tumor-stromal cross-talk. Emerging evidence suggests inherent biological differences in the tumor microenvironment of breast cancer patients from different racial backgrounds. Tumor microenvironment components, affected by the genetic make-up of the tumor cells as well as other nontumor- associated factors, may also render patients more susceptible to the development of aggressive tumors and faster progression of disease resulting in early onset, thus adversely affecting patients' survival. This review provides an overview of breast cancer racial disparity and discusses the existence of race-associated differential tumor microenvironment and its underlying genetic and non-genetic causal factors. A better understanding of these aspects would help further research on effective cancer management and improved approaches for reducing the racial disparities gaps in breast cancer patients. © The Author 2017. Published by Oxford University Press.",
**76**	Deepa M., Anjana R.M., Mohan V.,"**Role of lifestyle factors in the epidemic of diabetes: Lessons learnt from India**",**2017**,"https://www.scopus.com/inward/record.uri?eid=2-s2.0-85017593567&doi=10.1038%2fejcn.2017.19&partnerID=40&md5=31dfa81f929a7fa77abf81a47eefd199",<br>"The prevalence of type 2 diabetes (T2D) is increasing steadily globally with the largest increases occurring in developing countries like India. This is attributed to the changes in the lifestyle factors, including physical inactivity and unhealthy diet, both of which are modifiable. Existing evidence suggests that increasing physical activity reduces the risk of T2D. Improving the built environment can make it more conducive to people to increase physical activity. There is also a rapid nutrition transition with consumption of diets with higher intake of refined grains, higher fat, increased consumption of sugar and sweetened beverages, and lower intake of fruits and vegetables. A multisectoral approach promoting healthier diets and increasing physical activity can help in slowing down the diabetic epidemic. However, this requires political will to make necessary policy changes, as well as empowerment of the community, if the preventive measures are to be sustainable and scalable. © 2017 Macmillan Publishers Limited, part of Springer Nature. All rights reserved.",
**77**	Cho H.N., Choi E., Seo D.H., Suh M., Lee H.-Y., Park B., Park S., Cho J., Kim S., Park Y.-R., Lim J.-Y., Ahn Y., Park H.-Y., Choi K.S., Rhee Y.,"**The Korean Study of Women's Health-Related Issues (K-Stori): Rationale and Study Design**",**2017**,"https://www.scopus.com/inward/record.uri?eid=2-s2.0-85021651993&doi=10.1186%2fs12889-017-4531-1&partnerID=40&md5=f504dfd5fb4291c5db8ead41029c1b49"<br>"Background: Measures to address gender-specific health issues are essential due to fundamental, biological differences between the sexes. Studies have increasingly stressed the importance of customizing approaches directed at women's health issues according to stages in the female life cycle. In Korea, however, gender-specific studies on issues affecting Korean women in relation to stages in their life cycle are lacking. Accordingly, the Korean Study of Women's Health-Related Issues (K-Stori) was designed to investigate life cycle-specific health issues among women, covering health status, awareness, and risk perceptions. Methods: K-Stori was conducted as a nationwide cross-sectional survey targeting Korean women aged 14-79 years. Per each stage in the female life cycle (adolescence, childbearing age, pregnancy & postpartum, menopause, and older adult stage), 3000 women (total 15,000) were recruited by stratified multistage random sampling for geographic area based on the 2010 Resident Registration Population in Korea. Specialized questionnaires per each stage (total of five) were developed in consultation with multidisciplinary experts and by reflecting upon current interests into health among the general population of women in Korea. This survey was conducted from April 1 to June 31, 2016, at which time investigators from a professional research agency went door-to-door to recruit residents and conducted in-person interviews. Discussion: The study's findings may help with elucidating health issues and unmet needs specific to each stage in the life cycle of Korean women that have yet to be identified in present surveys. © 2017 The Author(s).","Adolescence

78	Zhang H., Jiang Y., Nguyen H.D., Poo D.C.C., Wang W.,"**The effect of a smartphone-based coronary heart disease prevention (SBCHDP) programme on awareness and knowledge of CHD, stress, and cardiac-related lifestyle behaviours among the working population in Singapore: A pilot randomised controlled trial**",**2017**,"https://www.scopus.com/inward/record.uri?eid=2-s2.0-85014878089&doi=10.1186%2fs12955-017-0623-y&partnerID=40&md5=380a97fab0c265ac96e9836b9cbbf2ab", "Background: Coronary heart disease (CHD) is the most prevalent type of cardiac disease among adults worldwide, including those in Singapore. Most of its risk factors, such as smoking, physical inactivity and high blood pressure, are preventable. mHealth has improved in the last decade, showing promising results in chronic disease prevention and health promotion worldwide. Our aim was to develop and examine the effect of a 4-week Smartphone-Based Coronary Heart Disease Prevention (SBCHDP) programme in improving awareness and knowledge of CHD, perceived stress as well as cardiac-related lifestyle behaviours in the working population of Singapore. Methods: The smartphone app ""Care4Heart"" was developed as the main component of the programme. App content was reviewed and validated by a panel of experts, including two cardiologists and two experienced cardiology-trained nurses. A pilot randomised controlled trial was conducted. Eighty working people were recruited and randomised to either the intervention group (n = 40) or the control group (n = 40). The intervention group underwent a 4-week SBCHDP programme, whereas the control group were offered health promotion websites only. The participants' CHD knowledge, perceived stress and behavioural risk factors were measured at baseline and on the 4th week using the Heart Disease Fact Questionnaire-2, Perceived Stress Scale, and Behavioural Risk Factor Surveillance System. Results: After the SBCHDP programme, participants in the intervention group had a better awareness of CHD being the second leading cause of death in Singapore (X 2 = 6.486, p = 0.039), a better overall CHD knowledge level (t = 3.171, p = 0.002), and better behaviour concerning blood cholesterol control (X 2 = 4.54, p = 0.033) than participants in the control group. Conclusion: This pilot study partially confirmed the positive effects of the SBCHDP programme in improving awareness and knowledge of CHD among the working population. Due to the small sample size and short follow-up period, this study was underpowered to detect significant differences between groups. A full-scale longitudinal study is required in the future to confirm the effectiveness of the SBCHDP programme. © 2017 The Author(s).","Coronary heart disease
79	Eisenhauer C.M., Hageman P.A., Rowland S., Becker B.J., Barnason S.A., Pullen C.H.,"**Acceptability of mHealth Technology for Self-Monitoring Eating and Activity among Rural Men**",**2017**,"https://www.scopus.com/inward/record.uri?eid=2-s2.0-84991624839&doi=10.1111%2fphn.12297&partnerID=40&md5=89ef44b5cf23c2c64eaffa881bd61613","Objective: To examine rural men's use and perceptions of mobile and wireless devices to self-monitor eating and physical activity (mHealth). Design and Sample: Men in this 3-week pilot study used FitBit One® to log daily food intake and monitor activity. A companion application (app) allowed activity monitoring of fellow participants. Health-related text messages were received 1–3 times daily. A purposive sample of 12 rural men (ages 40–67) was recruited by community leaders. Measures: (1) baseline heart rate, blood pressure, and BMI, (2) FitBit One® usage, (3) investigator-generated surveys on acceptability of mHealth, and (4) focus group on experience with mHealth. Results: Men were overweight (n = 3) or obese (n = 9) and 9 of 12 were hypertensive. Nine of twelve wore FitBit One® all 21 days. Eleven of 12 men logged food, with 9 of 12 doing this at least 15 of 21 days. Self-monitoring and daily text messaging increased awareness of energy intake and output. Companion app's food log needed targeting for rural foods. Rotating seasons (occupational, religious, recreational) and weak cellular signals created contextual barriers to self-monitoring eating and activity. Conclusions: FitBit One® and text messaging were perceived as useful among the rural men, while the companion apps require adaptation to reflect dietary norms. © 2016 Wiley Periodicals, Inc.","eating
80	Jennings C.A., Berry T.R., Carson V., Culos-Reed S.N., Duncan M.J., Loitz C.C., McCormack G.R., McHugh T.-L.F., Spence J.C., Vallance J.K., Mummery W.K.,"**UWALK: the development of a multi-strategy, community-wide physical activity program**",**2017**,"https://www.scopus.com/inward/record.uri?eid=2-s2.0-85015718366&doi=10.1007%2fs13142-016-0417-5&partnerID=40&md5=671e7088b1ccd4fc093d1fbf263366fd", "UWALK is a multi-strategy, multi-sector, theory-informed, community-wide approach using e and mHealth to promote physical activity in Alberta, Canada. The aim of UWALK is to promote physical activity, primarily via the accumulation of steps and flights of stairs, through a single over-arching brand. This paper describes the development of the UWALK program. A social ecological model and the social cognitive theory guided the development of key strategies, including the marketing and communication activities, establishing partnerships with key stakeholders, and e and mHealth programs. The program promotes the use of physical activity monitoring devices to self-monitor physical activity. This includes pedometers, electronic devices, and smartphone applications. In addition to entering physical activity data manually, the e and mHealth program provides the function for objective data to be automatically uploaded from select electronic devices (Fitbit®, Garmin and the smartphone application Moves) The RE-AIM framework is used to guide the evaluation of UWALK. Funding for the program commenced in February 2013. The UWALK brand was introduced on April 12, 2013 with the official launch, including the UWALK website on September 20, 2013. This paper describes the development and evaluation framework of a physical activity promotion program. This program has the potential for population level dissemination and uptake of an ecologically valid physical activity promotion program that is evidence-based and theoretically framed. © 2016, Society of Behavioral Medicine.","Community
81	Towfighi A., Cheng E.M., Ayala-Rivera M., McCreath H., Sanossian N., Dutta T., Mehta B., Bryg R., Rao N., Song S., Razmara A., Ramirez M., Sivers-Teixeira T., Tran J., Mojarro-Huang E., Montoya A., Corrales M., Martinez B., Willis P., Macias M., Ibrahim N., Wu S., Wacksman J., Haber H., Richards A., Barry F., Hill V., Mittman B., Cunningham W., Liu H., Ganz D.A., Factor D., Vickrey B.G.,"**Randomized controlled trial of a coordinated care intervention to improve risk factor control after stroke or transient ischemic attack in the safety net: Secondary stroke prevention by Uniting Community and Chronic care model teams Early to End Disparities (SUCCEED)**",**2017**,"https://www.scopus.com/inward/record.uri?eid=2-s2.0-85011649786&doi=10.1186%2fs12883-017-0792-7&partnerID=40&md5=3a6dd1e95d55f3e61118e3ecebba6c18", "Background: Recurrent strokes are preventable through awareness and control of risk factors such as hypertension, and through lifestyle changes such as healthier diets, greater physical activity, and smoking cessation. However, vascular risk factor control is frequently poor among stroke survivors, particularly among socio-economically disadvantaged blacks, Latinos and other people of color. The Chronic Care Model (CCM) is an effective framework for multi-component interventions aimed at improving care processes and outcomes for individuals with chronic disease. In addition, community health workers (CHWs) have played an integral role in reducing health disparities hypertension, and through lifestyle changes such as healthier diets, greater physical activity, and smoking cessation. However, vascular risk factor control is frequently poor among stroke survivors, particularly among socio-economically disadvantaged blacks, Latinos and other people of color. The Chronic Care Model (CCM) is an effective framework for multi-component interventions aimed at improving care processes and outcomes for individuals with chronic disease. In addition, community health workers (CHWs) have played an integral role in reducing health disparities (2) control of other vascular risk factors including lipids and hemoglobin A1c, (3) inflammation (C reactive protein [CRP]), (4) medication adherence, (5) lifestyle factors (smoking, diet, and physical activity), (6) estimated relative reduction in risk for recurrent stroke or myocardial infarction (MI), and (7) cost-effectiveness of the intervention versus usual care. Discussion: If this multi-component interdisciplinary intervention is shown to be effective in improving risk factor control after stroke, it may serve as a model that can be used internationally to reduce race/ethnic and socioeconomic disparities in stroke in resource-constrained settings. Trial registration: ClinicalTrials.gov Identifier NCT01763203. © 2017 The Author(s).","Biomarkers
82	Brouwers R.W.M., Kraal J.J., Traa S.C.J., Spee R.F., Oostveen L.M.L.C., Kemps H.M.C.,"**Effects of cardiac telerehabilitation in patients with coronary artery disease using a personalised patient-centred web application: Protocol for the SmartCare-CAD randomised controlled trial**",**2017**,"https://www.scopus.com/inward/record.uri?eid=2-s2.0-85011043556&doi=10.1186%2fs12872-017-0477-6&partnerID=40&md5=2e129082cf2fd9b14e65af3320f7de8f", "Background: Cardiac rehabilitation has beneficial effects on morbidity and mortality in patients with coronary artery disease, but is vastly underutilised and short-term improvements are often not sustained. Telerehabilitation has the potential to overcome these barriers, but its superiority has not been convincingly demonstrated yet. This may be due to insufficient focus on behavioural change and development of patients' self-management skills. Moreover, potentially beneficial communication methods, such as internet and video consultation, are rarely used. We hypothesise that, when compared to centre-based cardiac rehabilitation, cardiac telerehabilitation using evidence-based behavioural change strategies, modern communication methods and on-demand coaching will result in improved self-management skills and sustainable behavioural change, which translates to higher physical activity levels in a cost-effective way. Methods: This randomised controlled trial compares cardiac telerehabilitation with centre-based cardiac rehabilitation in patients with coronary artery disease. We randomise 300 patients entering cardiac rehabilitation to centre-based cardiac rehabilitation (control group) or cardiac telerehabilitation (intervention group). The core component of the intervention is a patient-centred web application, which enables patients to adjust rehabilitation goals, inspect training and physical activity data, share data with other caregivers and to use video consultation. After six supervised training sessions, the intervention group continues exercise training at home, wearing an accelerometer and heart rate monitor. In addition, physical activity levels are assessed by the accelerometer for four days per week. Patients upload training and physical activity data weekly and receive feedback through video consultation once a week. After completion of the rehabilitation programme, on-demand coaching is performed when training adherence or physical activity levels decline with 50% or more. The primary outcome measure is physical activity level, assessed at baseline, three months and twelve months, and is calculated from accelerometer and heart rate data. Secondary outcome measures include physical fitness, quality of life, anxiety and depression, patient empowerment, patient satisfaction and cost-effectiveness. Discussion: This study is one of the first studies evaluating effects and costs of a cardiac telerehabilitation intervention comprising a combination of modern technology and evidence-based behavioural change strategies including relapse prevention. We hypothesise that this intervention has superior effects on exercise behaviour without exceeding the costs of a traditional centre-based intervention. Trial registration: Netherlands Trial Register NTR5156. Registered 22 April 2015. © 2017 The Author(s).","Behavioural change

83	Pettinico G., Milne G.R.,"**Living by the numbers: understanding the "quantification effect"**",**2017**,"https://www.scopus.com/inward/record.uri?eid=2-s2.0-85020850162&doi=10.1108%2fJCM-06-2016-1839&partnerID=40&md5=c7dccbbb36a782c74271d6db3debbbe8",<br>"Purpose: This paper aims to establish if quantified self-data positively impact motivation in a goal pursuit across a broad cross-section of consumers and in multiple contexts and to understand the underlying causal mechanism and identify boundary conditions. Design/methodology/approach: Exploratory qualitative research helped direct the hypotheses development. Two quantitative experiments were then conducted via MTURK, involving 331 respondents, to test the hypotheses in two different personal goal areas (fitness and carbon footprint reduction). Findings: Self-quantification has a significant and positive impact on anticipated motivation in both contexts studied. The mediated model provides insight into the psychological process underlying self-quantification's motivational impact, which involves strengthening user perceptions regarding feedback meaningfulness, self-empowerment and goal focus. Age (>50) was found to be a boundary condition however, distance to goal was not. Research limitations/implications: This paper focuses on initial (anticipated) motivation, which is the vital first step in behavior change. However, more work is needed to understand quantification's long-term impact over the course of a behavior change process. Practical implications: This research encourages firms to incorporate self-quantification features into products/services aimed at behavior change and helps firms better understand consumer-perceived benefits. It alerts firms regarding the extra effort needed to convince older consumers of these benefits. Originality/value: This is the first study to confirm the "quantification effect" on motivation in multiple life areas and provide a causal model to explain how it works. It is also the first to highlight age as a boundary condition. © 2017, © Emerald Publishing Limited.","Behavior modification
84	Croskerry P., Cosby K.S., Graber M.L., Singh H.,"**Diagnosis: Interpreting the shadows**",**2017**,"https://www.scopus.com/inward/record.uri?eid=2-s2.0-85050514040&doi=10.1201%2f9781315116334&partnerID=40&md5=aa120d71bcb3a89ad1bd8577b6afaf03",<br>"Despite diagnosis being the key feature of a physician's clinical performance, this is the first book that deals specifically with the topic. In recent years, however, considerable interest has been shown in this area and significant developments have occurred in two main areas: a) an awareness and increasing understanding of the critical role of clinical decision making in the process of diagnosis, and of the multiple factors that impact it, and b) a similar appreciation of the role of the healthcare system in supporting clinicians in their efforts to make accurate diagnoses. Although medicine has seen major gains in knowledge and technology over the last few decades, there is a consensus that the diagnostic failure rate remains in the order of 10-15%. This book provides an overview of the major issues in this area, in particular focusing on where the diagnostic process fails, and where improvements might be made. © 2007 by Taylor & Francis Group, LLC.",
85	Martin J., Schneider F., Kowalewskij A., Jordan D., Hapfelmeier A., Kochs E.F., Wagner K.J., Schulz C.M.,"**Linear and non-linear heart rate metrics for the assessment of anaesthetists' workload during general anaesthesia**",**2016**,"https://www.scopus.com/inward/record.uri?eid=2-s2.0-85036475372&doi=10.1093%2fbja%2faew342&partnerID=40&md5=aa8791990f0426b41c3866790d8c5a06",<br>"Background: Excessive workload may impact the anaesthetists' ability to adequately process information during clinical practice in the operation room and may result in inaccurate situational awareness and performance. This exploratory study investigated heart rate (HR), linear and non-linear heart rate variability (HRV) metrics and subjective ratings scales for the assessment of workload associated with the anaesthesia stages induction, maintenance and emergence. Methods: HR and HRV metrics were calculated based on five min segments from each of the three anaesthesia stages. The area under the receiver operating characteristics curve (AUC) of the investigated metrics was calculated to assess their ability to discriminate between the stages of anaesthesia. Additionally, a multiparametric approach based on logistic regression models was performed to further evaluate whether linear or non-linear heart rate metrics are suitable for the assessment of workload. Results: Mean HR and several linear and non-linear HRV metrics including subjective workload ratings differed significantly between stages of anaesthesia. Permutation Entropy (PeEn, AUC=0.828) and mean HR (AUC=0.826) discriminated best between the anaesthesia stages induction and maintenance. In the multiparametric approach using logistic regression models, the model based on non-linear heart rate metrics provided a higher AUC compared with the models based on linear metrics. Conclusions: In this exploratory study based on short ECG segment analysis, PeEn and HR seem to be promising to separate workload levels between different stages of anaesthesia. The multiparametric analysis of the regression models favours non-linear heart rate metrics over linear metrics. © 2016 The Author(s)","anaesthesia
86	Banos O., Bilal Amin M., Ali Khan W., Afzal M., Hussain M., Kang B.H., Lee S.,"**The Mining Minds digital health and wellness framework**",**2016**,"https://www.scopus.com/inward/record.uri?eid=2-s2.0-84978235809&doi=10.1186%2fs12938-016-0179-9&partnerID=40&md5=6f0816e970377d50408d009dac2bf567",<br>"Background: The provision of health and wellness care is undergoing an enormous transformation. A key element of this revolution consists in prioritizing prevention and proactivity based on the analysis of people's conducts and the empowerment of individuals in their self-management. Digital technologies are unquestionably destined to be the main engine of this change, with an increasing number of domain-specific applications and devices commercialized every year however, there is an apparent lack of frameworks capable of orchestrating and intelligently leveraging, all the data, information and knowledge generated through these systems. Methods: This work presents Mining Minds, a novel framework that builds on the core ideas of the digital health and wellness paradigms to enable the provision of personalized support. Mining Minds embraces some of the most prominent digital technologies, ranging from Big Data and Cloud Computing to Wearables and Internet of Things, as well as modern concepts and methods, such as context-awareness, knowledge bases or analytics, to holistically and continuously investigate on people's lifestyles and provide a variety of smart coaching and support services. Results: This paper comprehensively describes the efficient and rational combination and interoperation of these technologies and methods through Mining Minds, while meeting the essential requirements posed by a framework for personalized health and wellness support. Moreover, this work presents a realization of the key architectural components of Mining Minds, as well as various exemplary user applications and expert tools to illustrate some of the potential services supported by the proposed framework. Conclusions: Mining Minds constitutes an innovative holistic means to inspect human behavior and provide personalized health and wellness support. The principles behind this framework uncover new research ideas and may serve as a reference for similar initiatives. © 2016 The Author(s).","Big data
87	Verdezoto N., Grönvall E.,"**On preventive blood pressure self-monitoring at home**",**2016**,"https://www.scopus.com/inward/record.uri?eid=2-s2.0-84948137859&doi=10.1007%2fs10111-015-0358-7&partnerID=40&md5=4751910f6e372fdefd1e27a518916ae5",<br>"Self-monitoring activities are increasingly becoming part of people's everyday lives. Some of these measurements are taken voluntarily rather than being referred by a physician and conducted because of either a preventive health interest or to better understand the body and its functions (the so-called Quantified Self). In this article, we explore socio-technical complexities that may occur when introducing preventive health-measurement technologies into older adults' daily routines and everyday lives. In particular, the original study investigated blood pressure (BP) measurement in non-clinical settings, to understand existing challenges, and uncover opportunities for self-monitoring technologies to support preventive healthcare activities among older adults. From our study, several important aspects emerged to consider when designing preventive self-monitoring technology, such as the complexity of guidelines for self-measuring, the importance of interpretation, understanding and health awareness, sharing self-monitoring information for prevention, various motivational factors, the role of the doctor in prevention, and the home as a distributed information space. An awareness of these aspects can help designers to develop better tools to support people's preventive self-monitoring needs, compared to existing solutions. Supporting the active and informed individual can help improve people's self-care, awareness, and implementation of preventive care. Based on our study, we also reflect on the findings to illustrate how these aspects can both inform people engaged in Quantified Self activities and designers alike, and the tools and approaches that have sprung from the so-called Quantified Self movement. © 2015, Springer-Verlag London.","Challenges
88	Mcbain H., Mulligan K., Haddad M., Flood C., Jones J., Simpson A.,"**Self management interventions for type 2 diabetes in adult people with severe mental illness**",**2016**,"https://www.scopus.com/inward/record.uri?eid=2-s2.0-84964422594&doi=10.1002%2f14651858.CD011361.pub2&partnerID=40&md5=359a82bd26571afd03de6c014498b910",<br>"Background: People with severe mental illness are twice as likely to develop type 2 diabetes as those without severe mental illness. Treatment guidelines for type 2 diabetes recommend that structured education should be integrated into routine care and should be offered to all. However, for people with severe mental illness, physical health may be a low priority, and motivation to change may be limited. These additional challenges mean that the findings reported in previous systematic reviews of diabetes self management interventions may not be generalised to those with severe mental illness, and that tailored approaches to effective diabetes education may be required for this population. Objectives: To assess the effects of diabetes self management interventions specifically tailored for people with type 2 diabetes and severe mental illness. Search methods: We searched the Cochrane Library, MEDLINE, EMBASE, PsycINFO, the Cumulative Index to Nursing and Allied Health Literature (CINAHL), the International Clinical Trials Registry Platform (ICTRP) Search Portal, ClinicalTrials.gov and grey literature. The date of the last search of all databases was 07 March 2016. Selection criteria: Randomised controlled trials of diabetes self management interventions for people with type 2 diabetes and severe mental illness. Data collection and analysis: Two review authors independently screened abstracts and full-text articles, extracted data and conducted the risk of bias assessment. We used a taxonomy of behaviour change techniques and the framework for behaviour change theory to describe the theoretical basis of the interventions and active ingredients. We used the GRADE method (Grades of Recommendation, Assessment, Development and Evaluation Working Group) to assess trials for overall quality of evidence. Main results: We included one randomised controlled trial involving 64 participants with schizophrenia or schizoaffective disorder. The average age of participants was 54 years<br>participants had been living with type 2 diabetes for on average nine years, and with their psychiatric diagnosis since they were on average 28 years of age. Investigators evaluated the 24-week Diabetes Awareness and Rehabilitation Training (DART) programme in comparison with usual care plus information (UCI). Follow-up after trial completion was six months. Risk of bias was mostly unclear but was high for selective reporting. Trial authors did not report on diabetes-related complications, all-cause mortality, adverse events, health-related quality of life nor socioeconomic effects. Twelve months of data on self care behaviours as measured by total energy expenditure showed a mean of 2148 kcal for DART and 1496 kcal for UCI (52 participants very low-quality evidence), indicating no substantial improvement. The intervention did not have a substantial effect on glycosylated haemoglobin A1c (HbA1c) at 6 or 12 months of follow-up (12-month HbA1c data 7.9% for DART vs 6.9% for UCI 52 participants very low-quality evidence). Researchers noted small improvements in body mass index immediately after the intervention was provided and at six months, along with improved weight post intervention. Diabetes knowledge and self efficacy improved immediately following receipt of the intervention, and knowledge also at six months. The intervention did not improve blood pressure. Authors' conclusions: Evidence is insufficient to show whether type 2 diabetes self management interventions for people with severe mental illness are effective in improving outcomes. Researchers must conduct additional trials to establish efficacy, and to identify the active ingredients in these interventions and the people most likely to benefit from them. © 2016 The Cochrane Collaboration. John Wiley & Sons, Ltd.",

89	Mercer K., Giangregorio L., Schneider E., Chilana P., Li M., Grindrod K.,"**Acceptance of commercially available wearable activity trackers among adults aged over 50 and with chronic illness: A mixed-methods evaluation**",**2016**,"https://www.scopus.com/inward/record.uri?eid=2-s2.0-84983470070&doi=10.2196%2fmhealth.4225&partnerID=40&md5=ebc0c6f5f2cf7df8850cbdba48335e62",<br>"Background: Physical inactivity and sedentary behavior increase the risk of chronic illness and death. The newest generation of ""wearable""activity trackers offers potential as a multifaceted intervention to help people become more active. Objective: To examine the usability and usefulness of wearable activity trackers for older adults living with chronic illness. Methods: We recruited a purposive sample of 32 participants over the age of 50, who had been previously diagnosed with a chronic illness, including vascular disease, diabetes, arthritis, and osteoporosis. Participants were between 52 and 84 years of age (mean 64)
90	Goyal S., Morita P., Lewis G.F., Yu C., Seto E., Cafazzo J.A.,"**The Systematic Design of a Behavioural Mobile Health Application for the Self-Management of Type 2 Diabetes**",**2016**,"https://www.scopus.com/inward/record.uri?eid=2-s2.0-84977461587&doi=10.1016%2fj.jcjd.2015.06.007&partnerID=40&md5=358005492cf4427c0e73a9b01699e456","Patients with diabetes often face serious complications due to limited self-management skills, the inability to adhere to care regimens, and psychosocial factors. Although regular self-monitoring of blood glucose is known to benefit patients receiving insulin therapy, its role in patients not treated with insulin has been unclear. However, recent studies have demonstrated that structured self-monitoring of blood glucose can significantly benefit patients who are not taking insulin, facilitating improved self-awareness and clinical decision making. We hypothesize that effective self-management by patients with type 2 diabetes who do not need insulin requires a behavioural intervention that enables the association between lifestyle behaviours, such as dietary intake and physical activity, and overall glycemic control. Mobile health applications (apps), coupled with wireless medical peripheral devices, can facilitate self-monitoring<br>deliver tailored, actionable knowledge elicit positive behaviour changes and promote effective self-management of diabetes. Although existing apps incorporate tracking and feedback from healthcare providers, few attempt to elicit positive behaviour changes for the purposes of developing patients' self-care skills. The purpose of this article is to present a systematic approach to the design and development a diabetes self-management mobile app, which included 1) a scoping review of literature 2) the development of an overarching theoretical approach and 3) validation of the app features through user-centred design methods. The resulting app, bant II, facilitates 1) self-monitoring of blood glucose, physical activity, diet and weight 2) identification of glycemic patterns in relation to lifestyle 3) remedial decision making and 4) positive behaviour change through incentives. © 2015 Canadian Diabetes Association.","Blood glucose
91	Forster H., Walsh M.C., Gibney M.J., Brennan L., Gibney E.R.,"**Personalised nutrition: The role of new dietary assessment methods**",**2016**,"https://www.scopus.com/inward/record.uri?eid=2-s2.0-84959463094&doi=10.1017%2fS0029665115002086&partnerID=40&md5=57eadea56c482cc23d9792142449df32","Food records or diaries, dietary recalls and FFQ are methods traditionally used to measure dietary intake<br>however, advancing technologies and growing awareness in personalised health have heightened interest in the application of new technologies to assess dietary intake. Dietary intake data can be used in epidemiology, dietary interventions and in the delivery of personalised nutrition advice. Compared with traditional dietary assessment methods, new technologies have many advantages, including their ability to automatically process data and provide personalised dietary feedback advice. This review examines the new technologies presently under development for the assessment of dietary intakes, and their utilisation and efficacy for personalising dietary advice. New technology-based methods of dietary assessment can broadly be categorised into three key areas: online (web-based) methods, mobile methods and sensor technologies. Several studies have demonstrated that utilising new technologies to provide tailored advice can result in positive dietary changes and have a significant impact on selected nutrient and food group intakes. However, comparison across studies indicates that the magnitude of change is variable and may be influenced by several factors, including the frequency and type of feedback provided. Future work should establish the most effective combinations of these factors in facilitating dietary changes across different population groups. © The Authors 2015.","Diet
92	Tagliente I., Solvoll T., Trieste L., De Cecco C.N., Murgia F., Bella S.,"**Which indicators for measuring the daily physical activity? An overview on the challenges and technology limits for Telehealth applications**",**2016**,"https://www.scopus.com/inward/record.uri?eid=2-s2.0-84988557271&doi=10.3233%2fTHC-161216&partnerID=40&md5=b856c1a3bd5131a296afb79f59351bd0",<br>"BACKGROUND: Obesity is one of the biggest drivers of preventable chronic diseases and healthcare costs inWorldwide. Different prevention activities are suggested. By monitoring daily energy expenditure (EE) could be possible make personalized diets and programming physical activity. In this, physical inactivity is one of the most important public health problems. Some studies refer the effort of the international community in promoting physical activities. Physical activity can be promoted only by increasing citizens' empowerment on taking care of their health, and it passes from the improving of individual information. Technology can offer solutions and metrics for monitoring and measuring daily activity by interacting with individuals, sharing information and feedbacks. OBJECTIVE: In this study we review indicators of total energy expenditure and weaknesses of available devices in assessing these parameters. METHODS: Literature review and technology testing EuNetHta core model. RESULTS: For the clinical aspects, it is fundamental to take into account all the factor that can influence the personal energy expenditure as: heart rate, blood pressure and thermoregulation (influenced by the body temperature). DISCUSSION: In this study we focused the attention on the importance of tools to encourage the physical activity. We made an analysis of the factor that can influence the right analysis of energy expenditure and at the same time the energy regime. A punctual monitoring of the exercise regime could be helpful in Telemedicine application as Telemonitorig. More study are needed to value the impact of physical activity tracker in Telemonitorig protocols. CONCLUSION: On the assessment of the energy expenditure, critical issues are related to the physiological data acquisition. Sensors connected with mobile devices could be important tools for disease prevention and interventions affecting health behaviors. New devices applications are potential useful for telemedicine assistance, but security of data and the related communication protocol limits should be taking into account. © 2016 -IOS Press and the authors. All rights reserved.","energy expenditure
93	Banos O., Amin M.B., Ali Khan W., Ali T., Afzal M., Kang B.H., Lee S.,"**Mining Minds: An innovative framework for personalized health and wellness support**",**2015**,"https://www.scopus.com/inward/record.uri?eid=2-s2.0-84963721156&doi=10.4108%2ficst.pervasivehealth.2015.259083&partnerID=40&md5=3fc898c4e5c982df74706a16d7a3998e",<br>"The world is witnessing a spectacular shift in the delivery of health and wellness care. The key ingredient of this transformation consists in the use of revolutionary digital technologies to empower people in their self-management as well as to enhance traditional care procedures. While substantial domain-specific contributions have been provided to that end in the recent years, there is a clear lack of platforms that may orchestrate, and intelligently leverage, all the data, information and knowledge generated through these technologies. This work presents Mining Minds, an innovative framework that builds on the core ideas of the digital health and wellness paradigms to enable the provision of personalized healthcare and wellness support. Mining Minds embraces some of the currently most prominent digital technologies, ranging from Big Data and Cloud Computing to Wearables and Internet of Things, and state-of-the-art concepts and methods, such as Context-Awareness, Knowledge Bases or Analytics, among others. This paper aims at thoroughly describing the efficient and rational combination and interoperation of these modern technologies and methods through Mining Minds, while meeting the essential requirements posed by a framework for personalized health and wellness support. © 2015 ICST.","big data
94	Blazer D.G., Yaffe K., Liverman C.T., Committee on the Public Health Dimensions of Cognitive Aging, Board on Health Sciences Policy, Institute of Medicine,"**Cognitive aging: Progress in understanding and opportunities for action**",**2015**,"https://www.scopus.com/inward/record.uri?eid=2-s2.0-85014417227&doi=10.17226%2f21693&partnerID=40&md5=20056065a811de6528b2c7baf7ff8fa3",<br>"For most Americans, staying ""mentally sharp"" as they age is a very high priority. Declines in memory and decision-making abilities may trigger fears of Alzheimer's disease or other neurodegenerative diseases. However, cognitive aging is a natural process that can have both positive and negative effects on cognitive function in older adults - effects that vary widely among individuals. At this point in time, when the older population is rapidly growing in the United States and across the globe, it is important to examine what is known about cognitive aging and to identify and promote actions that individuals, organizations, communities, and society can take to help older adults maintain and improve their cognitive health. Cognitive Aging assesses the public health dimensions of cognitive aging with an emphasis on definitions and terminology, epidemiology and surveillance, prevention and intervention, education of health professionals, and public awareness and education. This report makes specific recommendations for individuals to reduce the risks of cognitive decline with aging. Aging is inevitable, but there are actions that can be taken by individuals, families, communities, and society that may help to prevent or ameliorate the impact of aging on the brain, understand more about its impact, and help older adults live more fully and independent lives. Cognitive aging is not just an individual or a family or a health care system challenge. It is an issue that affects the fabric of society and requires actions by many and varied stakeholders. Cognitive Aging offers clear steps that individuals, families, communities, health care providers and systems, financial organizations, community groups, public health agencies, and others can take to promote cognitive health and to help older adults live fuller and more independent lives. Ultimately, this report calls for a societal commitment to cognitive aging as a public health issue that requires prompt action across many sectors. © 2015 by the National Academy of Sciences. All rights reserved.",
95	Bird M.-L., Clark B., Millar J., Whetton S., Smith S.,"**Exposure to ""exergames"" increases older adults' perception of the usefulness of technology for improving health and physical activity: A pilot study**",**2015**,"https://www.scopus.com/inward/record.uri?eid=2-s2.0-85016885944&doi=10.2196%2fgames.4275&partnerID=40&md5=92ffa6d029b6d1432ece5a47cee55356",<br>"Background: High rates of sedentary behaviors in older adults can lead to poor health outcomes. However, new technologies, namely exercise-based videogames (""exergames""), may provide ways of stimulating uptake and ongoing participation in physical activities. Older adults' perceptions of the use of technology to improve health are not known. Objective: The study aimed to determine use and perceptions of technology before and after using a 5-week exergame. Methods: Focus groups determined habitual use of technology and the participant's perceptions of technology to assist with health and physical activity. Surveys were developed to quantitatively measure these perceptions and were administered before and after a 5-week intervention. The intervention was an exergame that focused on postural balance (""Your Shape Fitness Evolved 2012""). Games scores, rates of game participation, and enjoyment were also recorded. Results: A total of 24 healthy participants aged between 55 and 82 years (mean 70, SD 6 years) indicated that after the intervention there was an increased awareness that technology (in the form of exergames) can assist with maintaining physical activity (P<.001). High levels of enjoyment (Physical Activity Enjoyment Scale [PACES-8] score mean 53.0, SE 0.7) and participation rates over the whole study (83%-100%) were recorded. Conclusions: Older adults' have low perception of the use of technology for improving health outcomes until after exposure to exergames. Technology, in the form of enjoyable exergames, may be useful for improving participation in physical activity that is relevant for older adults. © 2015 JMIR Publications Inc. All right reserved.","Exercise

**96**	Apovian C.M., Garvey W.T., Ryan D.H.,"**Challenging obesity: Patient, provider, and expert perspectives on the roles of available and emerging nonsurgical therapies**",**2015**,"https://www.scopus.com/inward/record.uri?eid=2-s2.0-84936761068&doi=10.1002%2foby.21140&partnerID=40&md5=a604297b82a822833f0f4f1ab1de09b3", "Objective Adult obesity is recognized as a chronic disease. According to principles of chronic disease management, healthcare professionals should work collaboratively with patients to determine appropriate therapeutic strategies that address overweight and obesity, specifically considering a patient's disease status in addition to their individual needs, preferences, and attitudes regarding treatment. A central role and responsibility of healthcare professionals in this process is to inform and educate patients about their treatment options. Although current recommendations for the management of adult obesity provide general guidance regarding safe and proper implementation of lifestyle, pharmacological, and surgical interventions, healthcare professionals need awareness of specific evidence-based information that supports individualized clinical application of these therapies. More specifically, healthcare professionals should be up-to-date on approaches that promote successful lifestyle management and be knowledgeable about newer weight loss pharmacotherapies, so they can offer patients with obesity a wide range of options to personalize their treatment. Accordingly, this educational activity has been developed to provide participants with the latest information on treatment recommendations and therapeutic advances in lifestyle intervention and pharmacotherapy for adult obesity management. Design and Methods This supplement is based on the content presented at a live CME symposium held in conjunction with ObesityWeek 2014. Results This supplement provides an expert summary of current treatment recommendations and recent advances in nonsurgical therapies for the management of adult obesity. Patient and provider perspectives on obesity management are highlighted in embedded video clips available via QR codes, and new evidence will be applied using clinically relevant case studies. Conclusions This supplement provides a topical update of obesity management, including clinical practice examples, for healthcare professionals who treat or provide care for adults with obesity. © 2015 The Obesity Society.",
**97**	Barwais F.A., Cuddihy T.F.,"**Empowering sedentary adults to reduce sedentary behavior and increase physical activity levels and energy expenditure: A pilot study**",**2015**,"https://www.scopus.com/inward/record.uri?eid=2-s2.0-84920391336&doi=10.3390%2fijerph120100414&partnerID=40&md5=cdf0995c04a6508bdc0ad72a205bc8ae", "Objective: The purpose of this study was to assess the effectiveness of a 4-week intervention in which an online personal activity monitor (Gruve-Technologies™) was used to reduce sedentary behavior among sedentary adults. Method: Eighteen, sedentary adult volunteers (12 men, six women, mean age 29 ± 4.0 years) were recruited to participate in the study. Time spent in sedentary activities and light-, moderate-, and vigorous-intensity physical activity and energy expenditure were assessed during waking hours using the monitor and the 7-day SLIPA Log at both baseline and post-intervention. Results: A significant decrease of 33% (3.1 h/day<br>p < 0.001) was found between the time spent in sedentary activities measured at baseline (9.4 ± 1.1 h/day) and at the end of the 4-week intervention (6.3 ± 0.8 h/day). Consequent to the changes in sedentary time, significant increases were found in the amount of time spent in light- (45% (2.6 h/day), p < 0.001), moderate- (33% (1 h/day) p < 0.001), vigorous-intensity physical activity (39% (0.16 h/day), p < 0.001), and energy expenditure (47% (216.7 kcal/day), p < 0.001). Conclusion: This monitor contributes to a meaningful reduction in time spent in sedentary activities and has a large effect on energy expenditure and physical activity patterns. © 2015 by the authors licensee MDPI, Basel, Switzerland.","7-day SLIPA log
**98**	Dave S.S., Craft L.L., Mehta P., Naval S., Kumar S., Kandula N.R.,"**Life stage influences on U.S. South Asian women's physical activity**",**2015**,"https://www.scopus.com/inward/record.uri?eid=2-s2.0-84921033844&doi=10.4278%2fajhp.130415-QUAL-175&partnerID=40&md5=872a38a0066c64d3ca358531c2a1b152", "Purpose. South Asian (SA) women in the United States report extremely low rates of leisure time physical activity (PA) compared with women in other ethnic minority groups. This study explored SA women's perspectives on PA during different life stages. Design. This is a community-based participatory research study that used focus groups. Setting. The study setting was a community-based organization that provides social services to SA immigrants in Chicago, Illinois. Participants. The study team conducted six focus groups (in English and Hindi) with 42 SA women, ages 18 to 71 years. Method. A semistructured interview guide was used to foster discussion about perceptions of, barriers to/facilitators of, and suggestions for PA programs. Discussions were transcribed and independently coded by two reviewers using thematic content analysis and guided by a coding scheme that was developed a priori. Results. Participants said that different life stages strongly influenced their PA. PA decreased after marriage and having children. Chronic diseases constrained older women from more vigorous PA. Barriers to PA among younger women were family disapproval and perceptions that PA is unnecessary if you are ""skinny."" Women agreed that PA is not a priority within the culture, and that interventions must take into account cultural, religious, and family context. Conclusion. Sociocultural norms, family constraints, and lack of awareness about the benefits of PA strongly influenced PA among SA women. Culturally salient intervention strategies might include programs in trusted community settings where women can exercise in women-only classes with their children, and targeted education campaigns to increase awareness about the benefits of PA across life stages. Copyright © 2015 by American Journal of Health Promotion, Inc.","Community-based research
**99**	Silver M.P.,"**Patient perspectives on online health information and communication with doctors: A qualitative study of patients 50 years old and over**",**2015**,"https://www.scopus.com/inward/record.uri?eid=2-s2.0-84922319699&doi=10.2196%2fjmir.3588&partnerID=40&md5=ccb821a159d993f6c7f471559079c4dc", "Background: As health care systems around the world shift toward models that emphasize self-care management, there is increasing pressure for patients to obtain health information online. It is critical that patients are able to identify potential problems with using the Internet to diagnose and treat a health issue and that they feel comfortable communicating with their doctor about the health information they acquire from the Internet. Objective: Our aim was to examine patient-identified (1) problems with using the Internet to identify and treat a health issue, (2) barriers to communication with a doctor about online health information seeking, and (3) facilitators of communication with a doctor about patient searches for health information on the Internet. Methods: For this qualitative exploratory study, semistructured interviews were conducted with a sample of 56 adults age 50 years old and over. General concerns regarding use of the Internet to diagnose and treat a health issue were examined separately for participants based on whether they had ever discussed health information obtained through the Internet with a doctor. Discussions about barriers to and facilitators of communication about patient searches for health information on the Internet with a doctor were analyzed using thematic analysis. Results: Six higher-level general concerns emerged: (1) limitations in own ability, (2) credibility/limitations of online information, (3) anxiety, (4) time consumption, (5) conflict, and (6) non-physical harm. The most prevalent concern raised by participants who communicated with a doctor about their online health information seeking related to the credibility or limitations in online information. Participants who had never communicated with a doctor about their online health information seeking most commonly reported concerns about non-physical harm. Four barriers to communication emerged: (1) concerns about embarrassment, (2) concerns that the doctor doesn't want to hear about it, (3) belief that there is no need to bring it up, and (4) forgetting to bring it up. Facilitators of communication included: (1) having a family member present at doctor visits, (2) doctor-initiated inquiries, and (3) encountering an advertisement that suggested talking with a doctor. Conclusions: Overall, participants displayed awareness of potential problems related to online health information seeking. Findings from this study point to a set of barriers as well as facilitators of communication about online health information seeking between patients and doctors. This study highlights the need for enhanced patient communication skills, eHealth literacy assessments that are accompanied by targeted resources pointing individuals to high-quality credible online health information, and the need to remind patients of the importance of consulting a medical professional when they use online health resources to diagnose and treat a health issue.","Adults 50 years old and over
**100**	Giabbanelli P.J., Crutzen R.,"**Supporting self-management of obesity using a novel game architecture**",**2015**,"https://www.scopus.com/inward/record.uri?eid=2-s2.0-84940486864&doi=10.1177%2f1460458214521051&partnerID=40&md5=d7cae750f9f8c229c17805ee8a400f61","Obesity has commonly been addressed using a one size fits all approach centred on a combination of diet and exercise. This has not succeeded in halting the obesity epidemic, as two-thirds of American adults are now obese or overweight. Practitioners are increasingly highlighting that ones weight is shaped by myriad factors, suggesting that interventions should be tailored to the specific needs of individuals. Health games have potential to provide such tailored approach. However, they currently tend to focus on communicating and/or reinforcing knowledge, in order to suscitate learning in the participants. We argue that it would be equally, if not more valuable, that games learn from participants using recommender systems. This would allow treatments to be comprehensive, as games can deduce from the participants behaviour which factors seem to be most relevant to his or her weight and focus on them. We introduce a novel game architecture and discuss its implications on facilitating the self-management of obesity. © The Author(s) 2014.","health games
**101**	Mendoza M., Han M., Meyring-Wösten A., Wilund K., Kotanko P.,"**It's a non-dialysis day... do you know how your patient is doing? a case for research into interdialytic activity**",**2015**,"https://www.scopus.com/inward/record.uri?eid=2-s2.0-84923072042&doi=10.1159%2f000369430&partnerID=40&md5=901551fd485520563886a5275176c249", "Hemodialysis (HD) patients are less active than their healthy counterparts<br>this is associated with higher mortality. Healthcare workers observe their patients only during HD, which accounts for about 7% of the week. Knowing more about what occurs in between sessions, particularly with respect to physical activity, may improve patient care and prognosis. Yet without a standard method to measure interdialytic activity, it is difficult to compare the effect of interventions. However, it is unclear how interdialytic activity can be accurately measured. Since activity associated with quality of life is multi-dimensional, objective and subjective tools should be used in conjunction. While commercially available tracking devices can be seamlessly incorporated into everyday life and can increase awareness of user's activity, their validation is needed in the HD population. Fertile topics for research should include the relationship between objective and subjective measures in HD patients, and the investigation of physical activity in non-ambulatory HD patients. © 2015 S. Karger AG, Basel.","CKD

**102**	Hermens H., op den Akker H., Tabak M., Wijsman J., Vollenbroek M.,"**Personalized Coaching Systems to support healthy behavior in people with chronic conditions**",**2014**,"https://www.scopus.com/inward/record.uri?eid=2-s2.0-84909630841&doi=10.1016%2fj.jelekin.2014.10.003&partnerID=40&md5=0beb799259e49d41a91205c7b528595d",<br>"Chronic conditions cannot be cured but daily behavior has a major effect on the severity of secondary problems and quality of life. Changing behavior however requires intensive support in daily life, which is not feasible with a human coach. A new coaching approach - so-called Personal Coaching Systems (PCSs) - use on-body sensing, combined with smart reasoning and context-aware feedback to support users in developing and maintaining a healthier behavior. Three different PCSs will be used to illustrate the different aspects of this approach:. (1)Treatment of neck/shoulder pain. EMG patterns of the Trapezius muscles are used to estimate their level of relaxation. Personal vibrotactile feedback is given, to create awareness and enable learning when muscles are insufficiently relaxed.(2)Promoting a healthy activity pattern. Using a 3D accelerometer to measure activity and a smartphone to provide feedback. Timing and content of the feedback are adapted real-time, using machine-learning techniques, to optimize adherence.(3)Management of stress during daily living. The level of stress is quantified using a personal model involving a combination of different sensor signals (EMG, ECG, skin conductance, respiration).Results show that Personal Coaching Systems are feasible and a promising and challenging way forward to coach people with chronic conditions. © 2014 Elsevier Ltd.","Ambulant sensing
**103**	Mindell J.S., Cohen D.L., Shelton N.J., Sutaria S., Hayward A., Watkins S.J.,"**Transport and clinical practice**",**2014**,"https://www.scopus.com/inward/record.uri?eid=2-s2.0-84899850201&doi=10.1016%2fj.jth.2013.08.001&partnerID=40&md5=1fb9a07d8a65a18112eef908282b4fea",<br>"This article summarises the transport and health agenda for health care practitioners who seek to understand how transport-related issues affect the well-being of their patients, and how disease and symptoms affects their patients' ability to travel. It is a resource for general medical education<br>it may also be useful in specialist training and in the training of other health professionals, particularly nurses and therapists. There is a lack of awareness among many health care professionals of the health benefits of active travel, adverse consequences of car use, and the financial cost to health services in providing car parking spaces. © 2013 Elsevier Ltd.","Disease
**104**	Nadeau D.A.,"**Management of type 2 diabetes mellitus in self- motivated patients: Optimized diet, exercise, and medication for weight loss and cardiometabolic fitness**",**2014**,"https://www.scopus.com/inward/record.uri?eid=2-s2.0-84922480117&doi=10.3810%2fpsm.2014.11.2091&partnerID=40&md5=db74a8187376cd42caccd683d6ad83d0",<br>"Type 2 diabetes mellitus (T2DM) is a growing public health problem with significant lifetime health care costs. The majority of Americans do not achieve minimal targets for exercise, and individuals with T2DM typically engage in less exercise than the general adult population. However, those patients with T2DM who are sufficiently self-motivated to manage their condition have the potential to reverse diabetes and prevent its complications through behavioral and pharmacologic interventions. Marked improvements are possible through increased awareness and selection of healthy eating options, a willingness to incorporate vigorous exercise into their lifestyle, and the use of newer medications that essentially eliminate the risk of hypoglycemia while facilitating weight loss and the achievement of ideal glucose targets. For self-motivated patients, daily aerobic activity of 45 to 60 minutes per day may be a suitable target. For those who have cardiovascular clearance, high-intensity interval training accomplishes high levels of cardiometabolic fitness with shorter training periods by alternating moderate and intense exertion. Suitable medications that have a low risk of hypoglycemia during exercise include metformin, glucagon-like peptide-1 receptor agonists, dipeptidyl peptidase-4 inhibitors, and sodium-glucose linked transporter-2 inhibitors. Specific daily caloric goals and incorporation of a mainly plant-based diet should be considered as a primary target for diabetes management. Self-management is important to achieving diabetes treatment goals, and mobile applications can be useful tools to support lifestyle changes in patients with T2DM. © The Physician and Sportsmedicine.","Diabetes mellitus
**105**	Campbell K.R.,"**Women and cardiovascular disease: Addressing disparities in care**",**2014**,"https://www.scopus.com/inward/record.uri?eid=2-s2.0-84986597318&doi=10.1142%2f9781783265022_fmatter&partnerID=40&md5=6d6844c882e2656d2b7d583f33453e4b",<br>"Cardiovascular disease is the number one killer of both men and women in the US and Europe today. Over the last 20 years, many innovations in technology have allowed for improved therapies for heart attack and stroke and overall outcomes have significantly improved. Death rates from cardiovascular disease in men are declining. However, even with these improvements in care, death rates for women remain unchanged. This book will explore these gender disparities in care in depth - specific sections will be devoted to answering the questions of why they are occurring and what healthcare providers and women can do to effect change and narrow the gender gap in cardiovascular care. Ultimately, this book is designed to open the eyes of healthcare providers, medical professionals and policy makers - as well as potential female patients - and should serve as a “call to action” to promote better cardiovascular care for women. Through education, awareness and advocacy women worldwide will benefit, the gap will begin to close and better cardiovascular care can be provided for all. © 2015 by Imperial College Press. All rights reserved.",
**106**	Valinsky L., Mishali M., Endevelt R., Preiss R., Dopelt K., Heymann A.D.,"**Reducing resistance to treatment, through group intervention, improves clinical measurements in patients with type 2 diabetes**",**2013**,"https://www.scopus.com/inward/record.uri?eid=2-s2.0-84890992559&doi=10.1186%2f1472-6823-13-61&partnerID=40&md5=3eaa97710b99c979bf9e9b0ecb49b0c5",<br>"Background: Studies have shown that group Therapeutic Patient Education (TPE) may empower patients with type 2 diabetes to better manage their disease. The mechanism of these interventions is not fully understood. A reduction in resistance to treatment may explain the mechanism by which TPE empowers participants to improve self-management. The Objective of this study was to examine the effectiveness of diabetes groups in reducing resistance to treatment and the association between reduced resistance and better management of the disease.Methods: In a program evaluation study, we administered validated questionnaires to measure resistance to treatment (RTQ) in 3 time periods: before the intervention (T1), immediately after the intervention (T2) and six months later (T3). Clinical measures (HbA1C, blood pressure, HDL, LDL and total cholesterol, Triglycerides and BMI) were retrieved from Maccabi Healthcare Services computerized systems, for T1<br>T2 and a year post intervention (T3). Linear mixed models were used adjusting for age, gender, social support and family status.Results: 157 156 and 106 TPE participants completed the RTQ in T1 T2 and T3 respectively. HbA1C and systolic and diastolic blood pressure were significantly reduced in the group which achieved a reduction in three out of the five RTQ components. For the other clinical measurements no significant changes were observed.Conclusion: Our findings suggest that reducing resistance to treatment, through an educational program for patients with diabetes, is associated with a better disease control. Identifying patients with higher resistance to treatment, and including components that reduce resistance in patient education programs, have the potential to increase the effectiveness of these programs. © 2013 Valinsky et al. licensee BioMed Central Ltd."
**107**	Grönvall E., Verdezoto N.,"**Understanding challenges and opportunities of preventive blood pressure self-monitoring at home**",**2013**,"https://www.scopus.com/inward/record.uri?eid=2-s2.0-84884639905&doi=10.1145%2f2501907.2501962&partnerID=40&md5=2de7995adb82a9223483b0e7038cc11c",<br>"The herein presented study explores socio-technical complexities that may occur when introducing preventive health-measurement technologies in older adults' daily routines and everyday lives. Using mainly blood pressure measurements in private homes, the study applied qualitative and quantitative methods to understand existing challenges and uncover opportunities of self-monitoring technologies to support preventive healthcare activities among older adults. Emerging challenges from our study were: rule complexity for self-measuring, reliability of measurements, interpretation, understanding and health awareness, the sharing activity for prevention, motivational sources, the role of the doctor for prevention, older adult's use of technology, the home as a distributed information space, and visualization of health data. An awareness of these challenges can help designers to develop better tools to support people's preventive self-monitoring needs compared with existing solutions. Furthermore, supporting the active and informed citizen can improve older adult's care abilities, awareness and activation towards preventive care. © 2013 ACM.","home-based technology
**108**	Van Dantzig S., Geleijnse G., Van Halteren A.T.,"**Toward a persuasive mobile application to reduce sedentary behavior**",**2013**,"https://www.scopus.com/inward/record.uri?eid=2-s2.0-84883446816&doi=10.1007%2fs00779-012-0588-0&partnerID=40&md5=74af6757fc74ae2510c6a514ae5c8dfb",<br>"Prolonged sitting is a potential health risk, not only for people with an inactive lifestyle but also for those who meet the daily physical activity recommendations. Mobile applications that trigger people to take regular breaks from sitting seem promising. In this paper, we present the results of our quest to create effective persuasive mobile applications aimed at reducing sedentary behavior. First, we developed SitCoach, a mobile application to nudge office workers from their seats. SitCoach monitors physical activity and sedentary behavior and provides timely persuasive messages suggesting active breaks. A user test showed that users had little awareness of the risks of prolonged sitting and considered their ability to take active breaks to be highly dependent on external factors. The results from this study formed the basis for a second experiment, which was more extensive in duration and number of participants. In this 6-week experiment, office workers received timely persuasive messages on their smart phones, advising them to take an active break whenever they were sitting behind their computer for too long. Compared to a Control group who did not receive these messages, a significant decrease in computer activity was achieved. The studies show the potential and limitations of using a smart phone as a platform for reducing sedentary behavior. We conclude with recommendations to create effective mobile applications that motivate people to take regular breaks from sitting. © 2012 Springer-Verlag London Limited.","Mobile application
**109**	Rao A.L., Asif I.M., Drezner J.A.,"**Secondary prevention of sudden death in athletes: The essential role of automated external defibrillators**",**2013**,"https://www.scopus.com/inward/record.uri?eid=2-s2.0-84874515119&doi=10.1016%2fj.ccep.2012.11.004&partnerID=40&md5=896706a04fce6f8b6bed94bb02cb530b",<br>"Sudden cardiac arrest (SCA) is the leading cause of death in exercising young athletes. Automated external defibrillators (AEDs) are an integral link in the ""chain of survival"" and their prompt use promotes higher survival rates for SCA. Public access defibrillation programs shorten the time interval between SCA and shock delivery and train likely responders in CPR and AED use. SCA should be assumed in any collapsed and unresponsive athlete. Prompt management of SCA can be life saving for athletes with SCA. This article reviews strategies for effective secondary prevention of sudden death in athletes and the critical role of AEDs. © 2013 Elsevier Inc.","Cardiac arrest

**110**	Bozo D., Pano G., Çitozi R.,"Assessment of physical activity level in office employees groups in Albania",2013,"https://www.scopus.com/inward/record.uri?eid=2-s2.0-85086494759&doi=10.4100%2fjhse.2012.8.proc2.18&partnerID=40&md5=696780b911afed1bf63aa2fc086096fb","Insufficient PA is very common in office employees and due to this they are at higher risk for metabolic, cardiovascular and many more other health related risk factor diseases. There is a lack of data regarding the PA level of office employees groups in Albania. Thus the aim of this paper was to identify the PA level in typical representative groups of this category. A total of 164 randomly selected office employees, out of which 97 (52 females and 45 males) from one mobile company and 67 (37 females and 30 males) from a bank, participated in this study. An Albanian modified and adapted version of the an International Physical Activity Questionnaire was used to assess some indicators dealing with all kinds of PA jobs, everyday life and leisure and time expenditure on PAs of different intensity. The weekly activity level was measured through time, frequency and intensity, expressed in MET*minute/week. The subjects were classified in 3 main PA categories: HIGH (1500-3000 MET*min), MODERATE (600-1300 MET*min) and LOW (<600 MET*min). The results show that both groups declared an average PA Level classified under the LOW category. The values of the mobile company group was around 950 MET*min with a difference between the genders of 280 MET*min (1070 in males and 790 in females)<br>while the bank group revealed lower values, i.e. 890 MET*min as average and a difference of 220 MET*min between the two genders (980 in males against 760 of females). The differences between the two groups under investigation can be explained through various factors such as the general PA gender differences, type of sedentary work, type of different work position/role and working spaces. These results may be also a consequence of improper individual activity habits, low information and awareness of health benefits of PA and lack of proper promotion for a healthy and active lifestyle. © Faculty of Education. University of Alicante.","Albania
**111**	Fitzpatrick K., LaGory M.,"**Unhealthy cities: Poverty, race, and place in america**",**2013**,"https://www.scopus.com/inward/record.uri?eid=2-s2.0-84909039853&doi=10.4324%2f9780203843765&partnerID=40&md5=bcc95781dead0e876d16d5ba74394ff2",<br>"The purpose of this book is to show the important role that space and place plays in the health of urban residents, particularly those living in high poverty ghettos. The book brings together research and writing from a variety of disciplines to demonstrate the health costs of being poor in America's cities. Both authors are committed to raising awareness of structural factors that promote poverty and injustice in a society that proclaims its commitment to equality of opportunity. Our health is often dramatically affected by where we live<br>some parts of the city seem to be designed to make people sick. The book is intended for students and professionals in urban sociology, medical sociology, public health, and community planning. © 2011 Taylor & Francis.",
**112**	Go A.S., Mozaffarian D., Roger V.L., Benjamin E.J., Berry J.D., Borden W.B., Bravata D.M., Dai S., Ford E.S., Fox C.S., Franco S., Fullerton H.J., Gillespie C., Hailpern S.M., Heit J.A., Howard V.J., Huffman M.D., Kissela B.M., Kittner S.J., Lackland D.T., Lichtman J.H., Lisabeth L.D., Magid D., Marcus G.M., Marelli A., Matchar D.B., McGuire D.K., Mohler E.R., Moy C.S., Mussolino M.E., Nichol G., Paynter N.P., Schreiner P.J., Sorlie P.D., Stein J., Turan T.N., Virani S.S., Wong N.D., Woo D., Turner M.B.,"**Heart disease and stroke statistics-2013 update: A Report from the American Heart Association**",**2013**,"https://www.scopus.com/inward/record.uri?eid=2-s2.0-84872091789&doi=10.1161%2fCIR.0b013e31828124ad&partnerID=40&md5=65f616ef3ff98b662466cee266c720f4",<br>"Each year, the American Heart Association (AHA), in conjunction with the Centers for Disease Control and Prevention, the National Institutes of Health, and other government agencies, brings together the most up-to-date statistics on heart disease, stroke, other vascular diseases, and their risk factors and presents them in its Heart Disease and Stroke Statistical Update*The Statistical Update is a valuable resource for researchers, clinicians, healthcare policy makers, media professionals, the lay public, and many others who seek the best national data available on heart disease, stroke, and other cardiovascular disease-related morbidity and mortality and the risks, quality of care, medical procedures and operations, and costs associated with the management of these diseases in a single document*Indeed, since 1999, the Statistical Update has been cited >10 500 times in the literature, based on citations of all annual versions*In 2011 alone, the various Statistical Updates were cited ≈1500 times (data from ISI Web of Science)*In recent years, the Statistical Update has undergone some major changes with the addition of new chapters and major updates across multiple areas, as well as increasing the number of ways to access and use the information assembled*For this year's edition, the Statistics Committee, which produces the document for the AHA, updated all of the current chapters with the most recent nationally representative data and inclusion of relevant articles from the literature over the past year*This year's edition also implements a new chapter organization to reflect the spectrum of cardiovascular health behaviors and health factors and risks, as well as subsequent complicating conditions, disease states, and outcomes*Also, the 2013 Statistical Update contains new data on the monitoring and benefits of cardiovascular health in the population, with additional new focus on evidence-based approaches to changing behaviors, implementation strategies, and implications of the AHA's 2020 Impact Goals*Below are a few highlights from this year's Update . © 2013 American Heart Association, Inc.",
**113**	Guastello S.J.,"**Human factorsengineering and ergonomicsa: A systems approach**",**2013**,"https://www.scopus.com/inward/record.uri?eid=2-s2.0-85054279115&partnerID=40&md5=7d760db8702ff6c90f8bc21161729fb9",<br>"Although still true to its original focus on the person-machine interface, the field of human factors psychology (ergonomics) has expanded to include stress research, accident analysis and prevention, and nonlinear dynamical systems theory (how systems change over time), human group dynamics, and environmental psychology. Reflecting new developments in the field, Human Factors Engineering and Ergonomics: A Systems Approach, Second Edition addresses a wide range of human factors and ergonomics principles found in conventional and twenty-first century technologies and environments. Based on the author's thirty years of experience, the text emphasizes fundamental concepts, systems thinking, the changing nature of the person-machine interface, and the dynamics of systems as they change over time. See What's New in the Second Edition: •Developments in working memory, degrees of freedom in cognitive processes, subjective workload, decision-making, and situation awareness •Updated information on cognitive workload and fatigue •Additional principles for HFE, networks, multiple person-machine systems, and human-robot swarms •Accident analysis and prevention includes resilience, new developments in safety climate, and an update to the inventory of accident prevention techniques and their relative effectiveness •Problems in "big data" mining •Psychomotor control and its relevance to human-robot systems •Navigation in real-world environment •Trust in automation and augmented cognition Computer technology permeates every aspect of the human-machine system, and has only become more ubiquitous since the previous edition. The systems are becoming more complex, so it should stand to reason that theories need to evolve to cope with the new sources of complexity. While many books cover traditional topics and theory, they to not focus on the practical problems students will face in the future. With broad coverage that ranges from physical ergonomics to cognitive aspects of human-machine interaction and includes dynamic approaches to system failure, this book increases the number of methods and analytical tools that are available for the human factors researcher. © 2014 by Taylor & Francis Group, LLC.",
**114**	Fasinu P.S., Bouic P.J., Rosenkranz B.,"**An overview of the evidence and mechanisms of herb-drug interactions**",**2012**,"https://www.scopus.com/inward/record.uri?eid=2-s2.0-84866040241&doi=10.3389%2ffphar.2012.00069&partnerID=40&md5=8e0bd7434aa12db5e173a9219c948f76",<br>"Despite the lack of sufficient information on the safety of herbal products, their use as alternative and/or complementary medicine is globally popular. There is also an increasing interest in medicinal herbs as precursor for pharmacological actives. Of serious concern is the concurrent consumption of herbal products and conventional drugs. Herb-drug interaction (HDI) is the single most important clinical consequence of this practice. Using a structured assessment procedure, the evidence of HDI presents with varying degree of clinical significance. While the potential for HDI for a number of herbal products is inferred from non-human studies, certain HDIs are well established through human studies and documented case reports. Various mechanisms of pharmacokinetic HDI have been identified and include the alteration in the gastrointestinal functions with consequent effects on drug absorption<br>induction and inhibition of metabolic enzymes and transport proteins and alteration of renal excretion of drugs and their metabolites. Due to the intrinsic pharmacologic properties of phytochemicals, pharmacodynamic HDIs are also known to occur. The effects could be synergistic, additive, and/or antagonistic. Poor reporting on the part of patients and the inability to promptly identify HDI by health providers are identified as major factors limiting the extensive compilation of clinically relevant HDIs. A general overview and the significance of pharmacokinetic and pharmacodynamic HDI are provided, detailing basic mechanism, and nature of evidence available. An increased level of awareness of HDI is necessary among health professionals and drug discovery scientists.With the increasing number of plant-sourced pharmacological actives, the potential for HDI should always be assessed in the non-clinical safety assessment phase of drug development process. More clinically relevant research is also required in this area as current information on HDI is insufficient for clinical applications. © 2012 Fasinu, Bouic and Rosenkranz.","Cytochrome P450
**115**	England M.J., Liverman C.T., Schultz A.M., Strawbridge L.M., Committee on the Public Health Dimensions of the Epilepsies, Board on Health Sciences Policy, Institute of Medicine,"**Epilepsy across the spectrum: Promoting health and understanding**",**2012**,"https://www.scopus.com/inward/record.uri?eid=2-s2.0-84990171527&doi=10.17226%2f13379&partnerID=40&md5=354b8fac30b3e9e6f1f51e5a8df08ec7",<br>"Although epilepsy is one of the nation's most common neurological disorders, public understanding of it is limited. Many people do not know the causes of epilepsy or what they should do if they see someone having a seizure. Epilepsy is a complex spectrum of disorders that affects an estimated 2.2 million Americans in a variety of ways, and is characterized by unpredictable seizures that differ in type, cause, and severity. Yet living with epilepsy is about much more than just seizures<br>the disorder is often defined in practical terms, such as challenges in school, uncertainties about social situations and employment, limitations on driving, and questions about independent living. The Institute of Medicine was asked to examine the public health dimensions of the epilepsies, focusing on public health surveillance and data collection population and public health research health policy, health care, and human services and education for people with the disorder and their families, health care providers, and the public. In Epilepsy Across the Spectrum, the IOM makes recommendations ranging from the expansion of collaborative epilepsy surveillance efforts, to the coordination of public awareness efforts, to the engagement of people with epilepsy and their families in education, dissemination, and advocacy for improved care and services. Taking action across multiple dimensions will improve the lives of people with epilepsy and their families. The realistic, feasible, and action-oriented recommendations in this report can help enable short- and long-term improvements for people with epilepsy. For all epilepsy organizations and advocates, local, state, and federal agencies, researchers, health care professionals, people with epilepsy, as well as the public, Epilepsy Across the Spectrum is an essential resource. © 2012 by the National Academy of Sciences. All rights reserved.",

**116**	Honka A., Kaipainen K., Hietala H., Saranummi N.,"**Rethinking health: ICT-enabled services to empower people to manage their health**",**2011**,"https://www.scopus.com/inward/record.uri?eid=2-s2.0-84855964067&doi=10.1109%2fRBME.2011.2174217&partnerID=40&md5=7d98f2dd62c8074fffdf2bb56820413f", "Lifestyle is a key determinant in the prevention and management of chronic diseases. If we would exercise regularly, eat healthy, control our weight, sleep enough, manage stress, not smoke and use alcohol only moderately, 90% of type II diabetes, 80% of coronary heart disease, and 70% of stroke could be prevented. Health statistics show that lifestyle related diseases are increasing at an alarming rate. Public health promotion campaigns and healthcare together are not effective enough to stop this tsunami. The solution that is offered is to empower people to manage their health with the assistance of ICT-enabled services. A lot of R&D and engineering effort is being invested in Personal Health Systems. Although some progress has been made, the market for such systems has not yet emerged. The aim of this critical review is to identify the barriers which are holding back the growth of the market. It looks into the theoretical foundations of behavior change support, the maturity of the technologies for behavior change support, and the business context in which behavior change support systems are used. © 2008 IEEE.","Behavior change support
**117**	Merrill R.M., Hyatt B., Aldana S.G., Kinnersley D.,"**Lowering employee health care costs through the healthy lifestyle incentive program**",**2011**,"https://www.scopus.com/inward/record.uri?eid=2-s2.0-79955051354&doi=10.1097%2fPHH.0b013e3181f54128&partnerID=40&md5=aa9da37042d5b5081d78431a8ec47371", "Objective: To evaluate the impact of the Healthy Lifestyle Incentive Program (HLIP), a worksite health program, on lowering prescription drug and medical costs. Design: Health care cost data for Salt Lake County employees during 2004 through 2008 were linked with HLIP enrollment status. Additional program information was obtained from a cross-sectional survey administered in 2008. Intervention: The program includes free annual screenings, tailored feedback on screening results, financial incentives for maintaining and modifying certain behaviors, and periodic educational programs and promotions to raise awareness of health topics. Main Outcome MeasureS: Frequency and cost of prescription drug and medical claims. Results: Participation increased from 16% to 23% in men and 34% to 45% in women over the 5-year study period and was associated with a significantly greater level of physical activity and improved general health. Participants were generally satisfied with the HLIP (43% were very satisfied, 51% satisfied, 5% dissatisfied, and 1% very dissatisfied). The primary factors contributing to participation were financial incentives (more so among younger employees), followed by a desire to improve health (more so among older employees). Over the study period, the cost savings in lower prescription drug and medical costs was $3 568 837. For every dollar spent on the HLIP the county saved $3.85. Conclusion: Financial incentives and then a desire for better health were the primary reasons for participation. The HLIP resulted in substantial health care cost savings for Salt Lake County Government. Copyright © 2011 Wolters Kluwer Health | Lippincott Williams & Wilkins.","Cost-effectiveness
**118**	Liu W.M.,"**Social class and classism in the helping professions: Research, theory, and practice**",**2011**,"https://www.scopus.com/inward/record.uri?eid=2-s2.0-84949809793&doi=10.4135%2f9781452230504&partnerID=40&md5=6e9d2cf30a15ebc7ab309c43aa5a54f0", "In this text author William Ming Liu presents theory and research on the impact of classism and social class on mental health. He provides an original framework—the Social Class Worldview Model—for exploring each person's individual and subjective life experiences. These experiences form a perspective that is unique to the individual. The author then helps the reader integrate this realization into the study of poverty, economic inequality, wealth, and the often overlooked implications of greed, materialism, and consumerism for a more complete understanding of social class and classism. Liu's original Social Class Worldview Model–Revised provides a theoretical framework for integrating each individual's reaction to social class and classism experiences and addressing that worldview within counseling and psychology work. Readers receive guidance in additional ways to act as advocates for their clients—regardless of affluence—through a study of privilege, social justice, empowerment, and competence. © 2011 by SAGE Publications, Inc. All rights reserved.",
**119**	Maitland J., Chalmers M.,"**Self-monitoring, self-awareness, and self-determination in cardiac rehabilitation**",**2010**,"https://www.scopus.com/inward/record.uri?eid=2-s2.0-77953976072&doi=10.1145%2f1753326.1753508&partnerID=40&md5=dcaaef864531fdbaa74ea32a0c1a7d9a", "The application of self-monitoring technologies to the problem of promoting health-related behavioural change has been an active area of research for many years. This paper reports on our investigations into health-related behavioural change within the context of a cardiac rehabilitation programme, and considers the role that self-monitoring currently plays and may play in the future. We carried out semi-structured interviews with nineteen cardiac rehabilitation participants. Our main findings relate to distinctions between implicit and conscious change, tensions between cardiac rehabilitation and everyday life, the importance of self-awareness and self-determination, and an overall reluctance towards unnecessary self-monitoring. In view of these findings, we then offer suggestions as to how self-monitoring technologies can be designed to suit this particular context of use. © 2010 ACM.","cardiac rehabilitation
**120**	Thiruvengada H., Srinivasan S., Gacic A.,"**Design and implementation of an automated human activity monitoring application for wearable devices**",**2008**,"https://www.scopus.com/inward/record.uri?eid=2-s2.0-69949119245&doi=10.1109%2fICSMC.2008.4811628&partnerID=40&md5=4384984187aea0877ee70659a98ad94b", "We present the design and implementation of a system that monitors physical activities of a human user wearing a portable device equipped with inertial sensors. This system uses inertial sensor data to automatically classify human activities using a trained classifier. The portable device is used both as a sensing as well as an application platform with user interface. The software application includes the classifier and a programmable graphical user interface that displays feedback on physical fitness related metrics such as activity type, duration, and the amount of energy expended while performing the action. The application also enables users to review historical data and set future goals. Processed activity information is stored and transmitted using a wireless connection to a remote web-based server, which can publish this feedback to the users on a secure website as needed. This application aims at improving user's awareness and lifestyle by providing on-demand feedback using real-time, reflective and motivational modes. In short, we propose a system that integrates sensing, computing, and user interaction into a wearable platform with the purpose of monitoring and reporting users' physical performance metrics with the goal of motivating physical fitness or active lifestyle without interfering with their day-to-day activities. © 2008 IEEE.","Activity monitoring
