# Supplementary material for: Mobile-Health based physical activities co-production policies towards cardiovascular diseases prevention: findings from a mixed-method systematic review
Source: BMC Health Serv Res. 2022 Mar 1;22:277. doi: 10.1186/s12913-022-07637-8 (PMC8886562; doi:10.1186/s12913-022-07637-8)
Supplement: Supplementary file 1 — Additional file 1. List of dataset papers for quantitative bibliometric analysis. [file 12913_2022_7637_MOESM1_ESM.pdf]

@ARTICLE{Smuck2021,
author={Smuck, M. and Odonkor, C.A. and Wilt, J.K. and Schmidt, N. and Swiernik, M.A.},
title={The emerging clinical role of wearables: factors for successful implementation in healthcare},
journal={npj Digital Medicine},
year={2021},
volume={4},
number={1},
doi={10.1038/s41746-021-00418-3},
art_number={45},
note={cited By 0},
url={https://www.scopus.com/inward/record.uri?eid=2-s2.0-85102501558&doi=10.1038%2fs41746-021-00418-3&partnerID=40&md5=bbc7158fab0cec02ba629165a56b692f},
affiliation={Division of Physical Medicine & Rehabilitation, Department of Orthopaedic Surgery, Stanford University, Stanford, CA, United States; Department of Orthopaedics and Rehabilitation, Yale University School of Medicine, New Haven, CT, United States; Chief Technology Officer of Innovation, Ochsner, Ochsner Health System, New Orleans, LA, United States; Head of Healthcare Products, Digital Health, Nokia Technologies, Cambridge, MA, United States; Medical Informatics, Kaiser Permanente Southern California, Pasadena, CA, United States},
abstract={Wearable technologies promise to redefine assessment of health behaviors, yet their clinical implementation remains a challenge. To address this gap, two of the NIH’s Big Data to Knowledge Centers of Excellence organized a workshop on potential clinical applications of wearables. A workgroup comprised of 14 stakeholders from diverse backgrounds (hospital administration, clinical medicine, academia, insurance, and the commercial device industry) discussed two successful digital health interventions that involve wearables to identify common features responsible for their success. Seven features were identified including: a clearly defined problem, integration into a system of healthcare delivery, technology support, personalized experience, focus on end-user experience, alignment with reimbursement models, and inclusion of clinician champions. Health providers and systems keen to establish new models of care inclusive of wearables may consider these features during program design. A better understanding of these features is necessary to guide future clinical applications of wearable technology. © 2021, The Author(s).},
keywords={big data; clinical medicine; health care delivery; hospital management; human; reimbursement; review},
references={Sung, D., (2015) What is Wearable Technology? Everything You Need to Know Explained, , https://www.wareable.com/wearable-tech/what-is-wearable-tech-753; Piwek, L., Ellis, D.A., Andrews, S., Joinson, A., The rise of consumer health wearables: promises and barriers (2016) PLoS Med., 13; Butte, N.F., Ekelund, U., Westerterp, K.R., Assessing physical activity using wearable monitors: measures of physical activity (2012) Med. Sci. Sports. Exerc., 44, pp. S5-S12; Schrack, J.A., Assessing daily physical activity in older adults: unraveling the complexity of monitors, measures and methods (2016) J. Gerontol. A Biol. Sci. Med. Sci., 71, pp. 1039-1048; Freedson, P., Bowles, H.R., Trojano, R., Haskell, W., Assessment of physical activity using wearable monitors: recommendations for monitor calibration and use in the field (2012) Med. Sci. Sports. Exerc., 44, pp. S1-S4; Smuck, M., Tomkins-Lane, C., Ith, M., Jarosz, R., Kao, M.C.,

Physical performance analysis: a new approach to assessing free-living physical activity in musculoskeletal pain and mobility-limited populations (2017) PLoS ONE, 12; Troiano, R.P., Physical activity in the United States measured by accelerometer (2008) Med. Sci. Sports Exerc., 40, pp. 181-188; Jakicic, J.M., Effect of wearable technology combined with a lifestyle intervention on long-term weight loss: the IDEA randomized clinical trial (2016) J. Am. Med. Assoc., 316, pp. 1161-1171; Tomkins-Lane, C.C., Haig, A.J., A review of activity monitors as a new technology for objectifying function in lumbar spinal stenosis (2012) J. Back. Musculoskelet. Rehabil., 25, pp. 177-185; Yang, C.C., Hsu, Y.L., A review of accelerometry-based wearable motion detectors for physical activity monitoring (2010) Sensors, 10, pp. 7772-7788; Gresham, G., Earable activity monitors to assess performance status and predict clinical outcomes in advanced cancer patients (2018) Npj Digital Med.; Gresham, G., Wearable activity monitors in oncology trials: current use of an emerging technology (2018) Contemp. Clin. Trials, 64, pp. 13-21; Schrack, J.A., Gresham, G., Wanigatunga, A.A., Understanding physical activity in cancer patients and survivors: New methodology, new challenges, and new opportunities (2017) Cold Spring Harb Mol. Case Stud.; John, D., Feedson, P., ActiGraph and Actical physical activity monitors: a peek under the hood (2012) Med. Sci. Sports. Exerc., 44, pp. S86-S89; Oresti, B., PhysioDroid: Combining wearable health sensors and mobile devices for a ubiquitous, continuous, and personal monitoring (2014) Sci. World J.; Ajami, S., Tejmouri, F., Features and application of wearable biosensors in medical care (2015) J. Res. Med. Sci., 20, pp. 1208-1215. , COI: 1:CAS:528:DC%2BC28XhsFOqtbvN; Noah, B., Impact of remote patient monitoring on clinical outcomes: An updated meta-analysis of randomized controlled trials (2018) Npj Digital Med.; Majumder, S., Smart homes for elderly healthcare—recent advances and research challenges (2017) Sensors, 17, p. 2496; Anderson, K., Buford, O., Emmerton, L., Mobile health apps to facilitate self-care: A qualitative study of user experiences (2016) Plos ONE; Car, J., Tan, W.S., Huang, Z., Sloot, P., Franklin, B.D., eHealth in the future of medications management: personalization, monitoring and adherence (2017) BMC Med., 15; Simpson, C.C., Mazzeo, S.E., Calorie counting and fitness tracking technology: associations with eating disorder symptomatology (2017) Eat. Behav., 26, pp. 89-92; Kim, J., Analysis of health consumers’ behavior using self-tracker for activity, sleep, and diet (2014) Telemed. J. E Health, 20, pp. 552-558; Mercer, K., Li, M., Giangregorio, L., Burns, C., Grindrod, K., Behavior change techniques present in wearable activity trackers: a critical analysis (2016) JMIR MHealth UHealth, 4; Schukat, M., Uninteded consequences of wearable sensor use in healthcareL contribution of the IMIA werable sensors in healthcare WG (2016) Yearb. Med. Inform., 1, pp. 73-86; Middelweerd, A., Mollee, J.S., van der Wal, C.N., Brug, J., Te Velde, S.J., Apps to promote physical activity among adults: a review and content analysis (2014) Int. J. Behav. Nutr. Phys. Act., 11, p. 97; Hilty, D.M., Chan, S., Hwang, T., Wong, A., Bauer, A.M., Advances in mobile mental health: opportunities and implications for the spectrum of e-mental health services (2017) Mhealth, 3, p. 34; Labrique, A.B., Vasudevan, L., Kochi, E., Fabricant, R., Mehl, G., mHealth innovations as a health system strengthening tools: 12 common applications and a visual framework (2013) Glob. Health. Sci. Pract., 1, pp. 160-171; Ridgers, N.D., McNarry, M.A., Mackintosh, K.A., Feasibility and effectiveness of using wearable activity trackers in youth: a systematic review (2016) JMIR Mhealth Uhealth, 4; Shantz, J.A.S., Veillette, C.J.H., The application of wearable technology in surgery: ensuring positive impact of the wearable revolution on surgical patients (2014) Front. Surg., 1, p. 39; Chiauzzi, E., Rodarte, C., DasMahapatra, P., Patient-centered activity monitoring in the self-management

of chronic health conditions (2015) BMC. Med., 1; Agarwal, V., Smuck, M., Shah, N.H., Quantifying the relative change in physical activity after Total Knee Arthroplasty using accelerometer based measurements (2017) AMIA. Jt. Summits. Transl. Sci. Proc., 2017, pp. 463-472. , PID: 28815146; Honkoop, P.J., MyAirCoach: the use of home-monitoring and mHealth systems to predict deterioration in asthma control and the occurrence of asthma exacerbations; study protocol of an observational study (2017) BMJ Open, , https://doi.org/10.1136/bmjopen-2016-013935; Field, M.J., Grigsby, J., Telemedicine and remote patient monitoring (2002) J. Am. Med. Assoc., 288, pp. 423-425; Ku, J.P., The mobilize center: an NIH big data to knowledge center to advance human movement research and improve mobility (2015) J. Am. Med. Inform. Assoc., 22, pp. 1120-1125; Kumar, S., Center of excellence for mobile sensor data-to-knowledge (MD2K) (2015) J. Am. Med. Inform. Assoc., 22, pp. 1137-1142; Milani, R.V., Lavie, C.J., Bober, R.M., Milani, A.R., Ventura, H.O., Improving hypertension control and patient engagement using digital tools (2017) Am. J. Med., 130, pp. 14-20; Milani, R.V., Lavie, C.J., Wilt, J.K., Bober, R.M., Ventura, H.O., New concepts in hypertension management: a population-based perspective (2016) Prog. Cardiovasc. Dis., 59, pp. 289-294; Jim, R., Dudl, M.D., Diabetes Lead Care Management Institute & Community Benefit Kaiser Permanente, , Personal Communication, 2/14/2018; Beach, R.D., Kuster, F.V., Moussy, F., Subminiature implantable potentiostat and modified commercial telemetry device for remote glucose monitoring (1999) IEEE Trans. Instrument. Measure., 48, pp. 1239-1245. , COI: 1:CAS:528:DC%2BD3cXptVWqsg%3D%3D; Glynn, L.G., Murphy, A.W., Smith, S.M., Schroeder, K., Fahey, T., Interventions used to improve control of blood pressure in patients with hypertension (2010) Cochrane Database Syst Rev.; Walker, W., Polk, T., Hande, A., Bhatia, D., Remote blood pressure monitoring using a wireless sensor network (2006) In 6Th Annual IEEE Emerging Information Technology Conference; Jethwani, K., Diabetes connect: an evaluation of patient adoption and engagement in a web-based remote glucose monitoring program (2012) J. Diabetes Sci. Technol., 6, pp. 1328-1336; Michael, A., Swiernik, M.D., Kaiser Permanente, Medical Informatics, Southern California, Health Connect, , Personal Communication, 4/17/2018; Lewy, H., Wearable technologies–future challenges for implementation in healthcare services (2015) Healthc. Technol. Lett., 2, pp. 2-5; Walker, R.K., Hickey, A.M., Freedson, P.S., Advantages and limitations of wearable activity trackers: Considerations for patients and clinicians (2016) Clin. J. Oncol. Nurs., 20, pp. 606-610; Liao, Y., Thompson, C., Peterson, S., Mandrola, J., Beg, M.S., The future of wearable technologies and remote monitoring in health care (2019) Am. Soc. Clin. Oncol. Educ. Book, 39, pp. 115-121; Smuck, M., Does physical activity influence the relationship between low back pain and obesity? (2014) Spine J., 14, pp. 209-216; Smuck, M., Objective measurement of function following lumbar spinal stenosis decompression reveals improved functional capacity with stagnant real-life physical activity (2018) Spine J, 18, pp. 15-21; Zheng, P.Z., Discriminating physical performance phenotypes of patients with chronic low back pain (2017) PM&R, 9, p. S169},
correspondence_address1={Smuck, M.; Division of Physical Medicine & Rehabilitation, United States; email: msmuck@stanford.edu},
publisher={Nature Research},
issn={23986352},
language={English},
abbrev_source_title={npj Digit. Med.},
document_type={Review},
source={Scopus},
}

@ARTICLE{Følling2021,
author={Følling, I.S. and Oldervoll, L.M. and Hilmarsen, C. and Ersfjord, E.M.I.},
title={Exploring use of activity monitors for patients with obesity during weight-loss treatment - a qualitative study},
journal={BMC Sports Science, Medicine and Rehabilitation},
year={2021},
volume={13},
number={1},
doi={10.1186/s13102-021-00253-9},
art_number={25},
note={cited By 0},
url={https://www.scopus.com/inward/record.uri?eid=2-s2.0-85102698409&doi=10.1186%2fs13102-021-00253-9&partnerID=40&md5=b2da910d0e19b2b3e5cf118b30b065fa},
affiliation={Centre for Obesity Research, Department of Surgery, Forsyningssenteret, St. Olavs Hospital, Trondheim, 7006, Norway; Department of Public Health and Nursing, Faculty of Medicine and Health Sciences, Norwegian University of Science and Technology, Trondheim, 7491, Norway; Centre for Crisis Psychology, Faculty of Psychology, University of Bergen, Bergen, 5020, Norway; Department of Circulation and Medical Imaging Faculty of Medicine and Health Sciences, Norwegian University of Science and Technology, Trondheim, 7491, Norway; Centre for eHealth, University of Agder, Campus Grimstad, 4879, Norway},
abstract={Background: Obesity is a major health concern in western countries. In Norway, patients with obesity can attend weight-loss programmes, which focus on changes in dietary and physical activity habits. Use of self-monitoring is advocated when changing dietary and physical activity habits for adults with obesity. This study aimed to explore the experiences of patients with obesity who used activity monitors while attending a weight-loss programme. Methods: Patients with body mass index (BMI) > 35 kg/m2 with weight related comorbidities or a BMI > 40 kg/m2 referred to an intermittent weight-loss programme were recruited into this study. They were introduced to one of three different activity monitors, Fitbit Zip™, Mio Fuse™, or Mio Slice™. Semi-structured interviews were performed with patients six months into the weight-loss programme. Thematic analysis was applied when analysing the data. Results: Of the 29 informants (aged 21 to 66 years) interviewed, 59% were female. Their experience with activity monitors was related to their adherence to the weight-loss programme. Two main themes emerged from the informants stories: 1. “Activity monitors visualize proof of effort or failure to change health habits”. 2. “Activity monitors act as a positive or negative enforcer when incorporating change”. Conclusions: Using activity monitors either strengthens or undermines patients’ attempts to change health habits when attending a weight-loss program. Our findings suggest a need for more individualized weight-loss programmes for patients with obesity. © 2021, The Author(s).},
author_keywords={Activity monitors; Health habits; Obesity; Qualitative research; Weight-loss programme},
references={(2020) Fact Sheets; Obesity and Overweight, , https://www.who.int/news-room/fact-sheets/detail/obesity-and-overweight; Hruby, A., Hu, F.B., The epidemiology of obesity: a big picture (2015) Pharmacoeconomics, 33, pp. 673-689; Wadden, T.A., Butryn, M.L., Wilson, C., Lifestyle modification for the management of obesity (2007) Gastroenterology., 132 (6), pp. 2226-2238; Peirson, L., Douketis, J., Ciliska, D.,

Fitzpatrick-Lewis, D., Ali, M.U., Raina, P., Treatment for overweight and obesity in adult populations: a systematic review and meta-analysis (2014) CMAJ Open, 2, pp. 306-317; Sharma, A., Iacobellis, G., Treatment of obesity: a challenging task (2006) Obesity and the kidney, pp. 212-220. , Wolf G, (ed), Karger, Jena; Kay, M., Santos, J., Takane, M., (2011) New Horizons for Health through Mobile Technologies. Global Observatory for Ehealth Series, 3. , World Health Organization; Rapp, A., Cena, F., Personal informatics for everyday life: how users without prior self-tracking experience engage with personal data (2016) Int J Human Comp Stud, 94, pp. 1-17; Rich, E., Miah, A., Mobile, wearable and ingestible health technologies: towards a critical research agenda (2017) Health Sociol Rev, 26, pp. 84-97; Foster, G.D., Makris, A.P., Bailer, B.A., Behavioral treatment of obesity (2005) Am J Clin Nutr, 82, pp. 230-235; Parish, J.M., The patient will see you now: the future of medicine is in your hands (2015) J Clin Sleep Med, 11 (6), pp. 689-690; Steinhubl, S.R., Muse, E.D., Topol, E.J., Can mobile health technologies transform health care? (2013) JAMA., 310 (22), pp. 2395-2396. , COI: 1:CAS:528:DC%2BC2cXosFGitQ%3D%3D; Marcengo, A., Rapp, A., Visualization of human behavior data: The quantified self (2014) Innovative Approaches of Data Visualization and Visual Analytics. Information Science Reference. Global, pp. 236-265. , Huang, ML, Huang W; De Vries, H.J., Kooiman, T.J., Van Ittersum, M.W., Van Brussel, M., De Groot, M., Do activity monitors increase physical activity in adults with overweight or obesity? A systematic review and meta-analysis (2016) Obesity, 24 (10), pp. 2078-2091; Yen, H.Y., Chiu, H.L., The effectiveness of wearable technologies as physical activity interventions in weight control: A systematic review and meta-analysis of randomized controlled trials (2019) Obes Rev, 20 (10), pp. 1485-1493; Pantzar, M., Ruckenstein, M., Social rhythms of the heart (2017) Health Sociol Rev, 26 (1), pp. 22-37; Donnachie, C., Wyke, S., Mutrie, N., Hunt, K., It's like having a personal motivator that you carried around wi' you": Utilising selfdetermination theory to understand men’s experiences of using pedometers to increase physical activity in a weight management programme (2017) IJBNPA, 14, p. 61; Khaodhiar, L., McCowen, K.C., Blackburn, G.L., Obesity and its comorbid conditions (1999) Clin Cornerstone, 2 (3), pp. 17-31. , COI: 1:STN:280:DC%2BD3c7mtFaksA%3D%3D; Tsigos, C., Hainer, V., Basdevant, A., Finer, N., Fried, M., Mathus-vliegen, E., Managements of obesity in adults: European clinical pratice guidelines (2008) Obes Facts, 1 (2), pp. 106-116; Beck, A.T., Cognitive theraphy:nature and relation to behavior therapy (1970) Behv Ther, 1 (2), pp. 184-200; Nes, B.M., Gutvik, C., Lavie, C., Nauman, J., Wisløff, U., Personalized activity intelligence (PAI) for prevention of cardiovascular disease and promotion of physical activity (2017) Am J Med, 130 (3), pp. 328-336; Braun, V., Clarke, V., Using thematic analysis in psychology (2006) Qual Res Psychol, 3 (2), pp. 77-101; Tong, A., Sainsbury, P., Craig, J., (2007) Consolidated Criteria for Reporting Qualitative Research (COREQ): A 32-Item Checklist for Interviews and Focus Groups. International Journal for Quality in Health Care: Journal of the International Society for Quality in Health Care / Isqua, 19, pp. 349-357; Hartmann-Boyce, J., Johns, D.J., Jebb, S.A., Aveyard, P., Behavioural weight management review group. Effect of behavioural techniques and delivery mode on effectiveness of weight management: systematic review, meta-analysis and meta-regressio (2014) Obes Rev, 15, pp. 598-609. , COI: 1:STN:280:DC%2BC2crktleiug%3D%3D; Samdal, G.B., Eide, G.E., Barth, T., Williams, G., Meland, E., Effective behaviour change techniques for physical activity and healthy eating in overweight and obese adults; systematic review and meta-regression analyses (2017) Int J Behav Nutr Phys Act, 14; Rothman, A.J., Baldwin, A.S., Hertel, A.W., Fuglestad, P.T., Self-regulation and behavior change: disentangling behavioral initiation and behavioral

maintenance (2011) Handbook of self-regulation: research, theory, and applications, pp. 106-122. , Baumeister RF, Vohs KD, (eds), Guilford Press, New York; Prochaska, J.O., Redding, C.A., Evers, K.E., Health behavior and health education (2008) Theory, research, and practice; the transtheoretical model and stages of change, , Glanz K, Rimer B, Viswanath K, (eds), Jossey Bass, San Francisco; Jeffery, R.W., Drewnowski, A., Epstein, L.H., Stunkard, A.J., Wilson, G.T., Wing, R.R., Long-term maintenance of weight loss: current status (2000) Health Psychol Behav Med, 19 (1S), pp. 5-16. , COI: 1:STN:280:DC%2BD3c7ntlWkug%3D%3D; Thomas, S.L., Hyde, J., Karunaratne, A., Kausman, R., Komeraroff, P.A., "They all work ….When you stick to them": A qualitative investigation of dieting. weight-loss, and physical exercise, in obese individuals (2008) Nutr J, 7 (34); Divine, C.M., Barnhill, A., The ethical and public health importance of unintended consequences: the case of behavioral weight loss interventions (2018) Public Health Ethics, 11 (3), pp. 356-361; Venditti, E.M., Wylie-Rosett, J., Delahanty, L.M., Mele, L., Hoskin, M.A., Edelstein, S.L., Short and Long-term Lifestyle Coaching Approaches Used to Address Diverse Participant Barriers to Weight Loss and Physical Activity (2014) IJBNPA, (16), p. 11; Mensinger, J.L., Calogero, R.M., Tylka, T.L., Internalized weight stigma moderates eating behavior outcomes in women with high BMI participating in a healthy living program (2016) Appetite., 102, pp. 32-43; Følling, I.S., Solbjør, M., Helvik, A.S., Previous experiences and emotional baggage as barriers to lifestyle change - a qualitative study of Norwegian Healthy Life Centre participants (2015) BMC Fam Pract, 16; Dato, S., Rose, G., Crocco, P., Monti, D., Garagnani, P., Franceschi, C., The genetics of human longevity: an intricacy of genes, environment, culture and microbiome (2017) Mech Ageing Dev, 165, pp. 147-155. , COI: 1:CAS:528:DC%2BC2sXmtlSksLs%3D; Wu, Q., Sum, K., Nathan-Roberts, D., How fitness trackers facilitate health behavior change (2016) Proceedings Human Factors Ergonomics Society Annual Meeting, 60 (1), pp. 1068-1072},
correspondence_address1={Følling, I.S.; Centre for Obesity Research, Norway; email: ingfoll@gmail.com},
publisher={BioMed Central Ltd},
issn={20521847},
language={English},
abbrev_source_title={BMC Sports Sci. Med. Rehabil.},
document_type={Article},
source={Scopus},
}

@ARTICLE{Ding2021,
author={Ding, D. and Cheng, M. and del Pozo Cruz, B. and Lin, T. and Sun, S. and Zhang, L. and Yang, Q. and Ma, Z. and Wang, J. and Jia, Y. and Shi, Y.},
title={How COVID-19 lockdown and reopening affected daily steps: evidence based on 164,630 person-days of prospectively collected data from Shanghai, China},
journal={International Journal of Behavioral Nutrition and Physical Activity},
year={2021},
volume={18},
number={1},
doi={10.1186/s12966-021-01106-x},
art_number={40},
note={cited By 0},
url={https://www.scopus.com/inward/record.uri?eid=2-s2.0-85102711874&doi=10.1186%2fs12966-021-01106-x&partnerID=40&md5=bbf360cb5f58b8d4f97137341aca19ea},
affiliation={Prevention Research Collaboration, Sydney School of Public Health,

Faculty of Medicine and Health, The University of Sydney, Camperdown, NSW, Australia; Charles Perkins Centre, The University of Sydney, Camperdown, NSW, Australia; Shanghai Municipal Center for Disease Control and Prevention, 1380 West Zhongshan Road, Shanghai, 200336, China; Centre for Active and Healthy Ageing, Department of Sports Science and Clinical Biomechanics, University of Southern Denmark, Campusvej 55, Odense, 5230, Denmark; Shanghai Pudong New Area Center for Disease Control and Prevention, Shanghai, 200136, China; School of Public Health, Key Lab of Public Health Safety of the Ministry of Education, Fudan University, 130 Dongan Road, Shanghai, 200032, China; Health Communication Institute, Fudan University, Shanghai, 200032, China; National Clinical Research Center for Aging and Medicine, Huashan Hospital, Fudan University, Shanghai, 200040, China},
abstract={Background: COVID-19 lockdowns may lead to physical inactivity, a major risk factor for non-communicable diseases. This study aims to determine: 1) the trajectory in daily step counts before, during and after the lockdown in China, and 2) the characteristics associated with the trajectories. Methods: From December 2019 to July 2020, smartphone-based step counts were continuously collected in 815 Chinese adults residing in Shanghai over 202 days across three phases: before, during, and after the lockdown. Participant characteristics were reported, and height, weight and body composition measured before the lockdown. A ‘sharp’ regression discontinuity design with cluster robust standard errors was used to test the effect of the lockdown and reopening on daily steps and a linear mixed model was used to examine the characteristics associated with trajectories during the observed period. Results: Based on 164,630 person-days of data, we found a sharp decline in daily step counts upon the lockdown (24/01/2020) by an average of 3796 (SE = 88) steps, followed by a significant trend of increase by 34 steps/day (SE = 2.5; p <.001) until the end of the lockdown (22/03/2020). This increasing trend continued into the reopening phase at a slower rate of 5 steps per day (SE = 2.3; p = 0.029). Those who were older, married, university educated, insufficiently active, had an ‘at risk’ body composition, and those in the control group, were slower at recovering step counts during the lockdown, and those who were older, married, without university education and with an ‘at risk’ body composition recovered step counts at a slower pace after the reopening. Conclusions: Despite later increases in step counts, COVID-19 lockdown led to a sustained period of reduced physical activity, which may have adverse health implications. Governments and health professionals around the world should continue to encourage and facilitate physical activity during the pandemic. © 2021, The Author(s).},
keywords={adult; Article; body composition; body height; body weight; China; Chinese; coronavirus disease 2019; female; human; lockdown; major clinical study; male; physical activity; prospective study; quarantine; activity tracker; communicable disease control; epidemiology; exercise; information processing; middle aged; mobile application; prevention and control; procedures; smartphone, Adult; China; Communicable Disease Control; COVID-19; Data Collection; Exercise; Female; Fitness Trackers; Humans; Male; Middle Aged; Mobile Applications; SARS-CoV-2; Sedentary Behavior; Smartphone},
references={WHO Director-General's Opening Remarks at the Media Briefing on COVID-19 - 11 2020 2020, , https://www.who.int/dg/speeches/detail/who-director-general-s-opening-remarks-at-the-media-briefing-on-covid-19%2D%2D-11-march-2020; (2020) WHO Coronavirus Disease (COVID-19) Dashboard, , https://covid19.who.int/, cited 2020 01/10, Available from; Maringe, C., Spicer, J., Morris, M., Purushotham, A., Nolte,

E., Sullivan, R., The impact of the COVID-19 pandemic on cancer deaths due to delays in diagnosis in England, UK: a national, population-based, modelling study (2020) The Lancet Oncol, 21 (8), pp. 1023-1034. , COI: 1:CAS:528:DC%2BB3cXhsVags7jN; Boserup, B., McKenney, M., Elkbuli, A., Alarming trends in US domestic violence during the COVID-19 pandemic (2020) Am J Emerg Med, 38 (12), pp. 2753-2755. , (,):, –; Pfefferbaum, B., North, C.S., Mental health and the Covid-19 pandemic (2020) N Engl J Med, 383 (6), pp. 510-512. , COI: 1:CAS:528:DC%2BB3cXhsFKjt7zN; Hartmann-Boyce, J., Morris, E., Goyder, C., Kinton, J., Perring, J., Nunan, D., Diabetes and COVID-19: risks, management, and learnings from other National Disasters (2020) Diabetes Care, 43 (8), pp. 1695-1703. , COI: 1:CAS:528:DC%2BB3cXitVCjs7nO; (2018) 2018 physical activity guidelines advisory committee scientific report, , U.S. Department of Health and Human Services, Washington, DC; Lee, I.M., Shiroma, E.J., Lobelo, F., Puska, P., Blair, S.N., Katzmarzyk, P.T., Effect of physical inactivity on major non-communicable diseases worldwide: an analysis of burden of disease and life expectancy (2012) Lancet, 380 (9838), pp. 219-229; Hamer, M., Kivimaki, M., Gale, C.R., Batty, G.D., Lifestyle risk factors, inflammatory mechanisms, and COVID-19 hospitalization: a community-based cohort study of 387,109 adults in UK (2020) Brain Behav Immun, 87, pp. 184-187. , COI: 1:CAS:528:DC%2BB3cXhtVWmtLrE; Ammar, A., Brach, M., Trabelsi, K., Chtourou, H., Boukhris, O., Masmoudi, L., Effects of COVID-19 home confinement on eating behaviour and physical activity: results of the ECLB-COVID19 international online survey (2020) Nutrients., 12 (6), p. 28; Ruiz-Roso, M.B., Knott-Torcal, C., Matilla-Escalante, D.C., Garcimartin, A., Sampedro-Nunez, M.A., Davalos, A., COVID-19 lockdown and changes of the dietary pattern and physical activity habits in a cohort of patients with type 2 diabetes mellitus (2020) Nutrients., 12 (8), p. 04; Stanton, R., To, Q.G., Khalesi, S., Williams, S.L., Alley, S.J., Thwaite, T.L., Depression, anxiety and stress during COVID-19: associations with changes in physical activity, sleep, tobacco and alcohol use in Australian adults (2020) Int J Environ Res Public Health, 17 (11), p. 07; Reyes-Olavarria, D., Latorre-Roman, P.A., Guzman-Guzman, I.P., Jerez-Mayorga, D., Caamano-Navarrete, F., Delgado-Floody, P., Positive and negative changes in food habits, physical activity patterns, and weight status during COVID-19 confinement: associated factors in the Chilean population (2020) Int J Environ Res Public Health, 17 (15), p. 28; Constandt, B., Thibaut, E., De Bosscher, V., Scheerder, J., Ricour, M., Willem, A., Exercising in times of lockdown: an analysis of the impact of COVID-19 on levels and patterns of exercise among adults in Belgium (2020) Int J Environ Res Public Health, 17 (11), p. 10; Gallo, L.A., Gallo, T.F., Young, S.L., Moritz, K.M., Akison, L.K., The impact of isolation measures due to COVID-19 on energy intake and physical activity levels in Australian University students (2020) Nutrients., 12 (6), p. 23; Lesser, I.A., Nienhuis, C.P., The impact of COVID-19 on physical activity behavior and well-being of Canadians (2020) Int J Environ Res Public Health, 17 (11), p. 31; Tison, G.H., Avram, R., Kuhar, P., Abreau, S., Marcus, G.M., Pletcher, M.J., Worldwide effect of COVID-19 on physical activity: A descriptive study Ann Intern Med, , https://doi.org/10.7326/M20-2665; Gui, X., Chen, Y., Caldeira, C., Xiao, D., Chen, Y., When Fitness Meets Social Networks: Investigating Fitness Tracking and Social Practices on WeRun (2017) Proceedings of the 2017 CHI Conference on Human Factors in Computing Systems, pp. 1647-1659. , Denver, Colorado, USA, Association for Computing Machinery; Macfarlane, D.J., Lee, C.C.Y., Ho, E.Y.K., Chan, K.L., Chan, D.T.S., Reliability and validity of the Chinese version of IPAQ (short, last 7 days) (2007) J Sci Med Sport, 10 (1), pp. 45-51; Jeong, S.-W., Kim, S.-H., Kang, S.-H., Kim, H.-J., Yoon, C.-H., Youn, T.-J., Mortality reduction with physical

activity in patients with and without cardiovascular disease (2019) Eur Heart J, 40 (43), pp. 3547-3555; Appropriate body-mass index for Asian populations and its implications for policy and intervention strategies (2004) Lancet., 363 (9403), pp. 157-163; Vasold, K.L., Parks, A.C., Phelan, D.M.L., Pontifex, M.B., Pivarnik, J.M., Reliability and validity of commercially available low-cost bioelectrical impedance analysis (2019) Int J Sport Nutr Exerc Metab, 29 (4), pp. 406-410. , PID: 30507268; Wang, C., Hou, X.H., Zhang, M.L., Bao, Y.Q., Zou, Y.H., Zhong, W.H., Comparison of body mass index with body fat percentage in the evaluation of obesity in Chinese (2010) Biomed Environ Sci, 23 (3), pp. 173-179; Oldenburg, C.E., Moscoe, E., Bärnighausen, T., Regression discontinuity for causal effect estimation in epidemiology (2016) Curr Epidemiol Rep, 3 (3), pp. 233-241; Imbens, G., Kalyanaraman, K., Optimal bandwidth choice for the regression discontinuity estimator (2011) Rev Econ Stud, 79 (3), pp. 933-959; Yan, Q., Yao, H., Xu, J., Cheng, M., Shi, Y., Zhong, W., Influencing factors of physical inactivity among non-agricultural population in Shanghai in 2013 (2017) J Environ Occup Med, 34 (8), pp. 681-686; Gornicka, M., Drywien, M.E., Zielinska, M.A., Hamulka, J., Dietary and lifestyle changes during COVID-19 and the subsequent lockdowns among polish adults: a cross-sectional online survey PLifeCOVID-19 study (2020) Nutrients., 12 (8), p. 03; (2020) New exercise habits forming during coronavirus crisis, , Sport England, London, UK; Ekelund, U., Kolle, E., Steene-Johannessen, J., Dalene, K.E., Nilsen, A.K.O., Anderssen, S.A., Objectively measured sedentary time and physical activity and associations with body weight gain: does body weight determine a decline in moderate and vigorous intensity physical activity? (2017) Int J Obes, 41 (12), pp. 1769-1774. , COI: 1:STN:280:DC%2BC1cfktlSksQ%3D%3D; Bauman, A.E., Reis, R.S., Sallis, J.F., Wells, J.C., Loos, R.J., Martin, B.W., Correlates of physical activity: why are some people physically active and others not? (2012) Lancet., 380 (9838), pp. 258-271; Ding, D., Del Pozo, C.B., Green, M.A., Bauman, A.E., Is the COVID-19 lockdown nudging people to be more active: A big data analysis Br J Sports Med, , https://doi.org/10.1136/bjsports-2020-102575; Liu, Z., Kemperman, A., Timmermans, H., Social-ecological correlates of older adults’ outdoor activity patterns (2020) J Transp Health, 16, p. 100840; Jiang, N., Li, S., Cao, S., Wei, J., Wang, B.B., Qin, N., Duan, X.L., Transportation Activity Patterns of Chinese Population during the COVID-19 Epidemic (2020) Res Environ Sci, 33, pp. 1675-1682. , in Chinese; Mattioli, A.V., Ballerini Puviani, M., Nasi, M., Farinetti, A., COVID-19 pandemic: the effects of quarantine on cardiovascular risk (2020) Eur J Clin Nutr, 74 (6), pp. 852-855. , COI: 1:CAS:528:DC%2BB3cXos1Gnt7k%3D; Bowden Davies, K.A., Sprung, V.S., Norman, J.A., Thompson, A., Mitchell, K.L., Halford, J.C.G., Short-term decreased physical activity with increased sedentary behaviour causes metabolic derangements and altered body composition: effects in individuals with and without a first-degree relative with type 2 diabetes (2018) Diabetologia., 61 (6), pp. 1282-1294; Nieman, D.C., Wentz, L.M., The compelling link between physical activity and the body's defense system (2019) J Sport Health Sci, 8 (3), pp. 201-217; Amagasa, S., Machida, M., Ding, D., Inoue, S., Preventing infectious diseases outbreaks at exercise facilities (2020) Managing Sport Leisure, pp. 1-4; Brooks, J.H.M., Tingay, R., Varney, J., Social distancing and COVID-19: An unprecedented active transport public health opportunity Br J Sports Med, , https://doi.org/10.1136/bjsports-2020-102856},
correspondence_address1={Shi, Y.; Shanghai Municipal Center for Disease Control and Prevention, 1380 West Zhongshan Road, China; email: shiyan@scdc.sh.cn; Jia, Y.; School of Public Health, 130 Dongan Road, China; email: jyn@fudan.edu.cn},
publisher={BioMed Central Ltd},

issn={14795868},
pubmed_id={33731132},
language={English},
abbrev_source_title={Int. J. Behav. Nutr. Phys. Act.},
document_type={Article},
source={Scopus},
}

@ARTICLE{Okop2021,
author={Okop, K.J. and Murphy, K. and Lambert, E.V. and Kedir, K. and Getachew, H. and Howe, R. and Niyibizi, J.B. and Ntawuyirushintege, S. and Bavuma, C. and Rulisa, S. and Kasenda, S. and Chipeta, E. and Bunn, C. and Crampin, A.C. and Chapotera, G. and King, A.C. and Banchoff, A. and Winter, S.J. and Levitt, N.S.},
title={Community-driven citizen science approach to explore cardiovascular disease risk perception, and develop prevention advocacy strategies in sub-Saharan Africa: a programme protocol},
journal={Research Involvement and Engagement},
year={2021},
volume={7},
number={1},
doi={10.1186/s40900-020-00246-x},
art_number={11},
note={cited By 0},
url={https://www.scopus.com/inward/record.uri?eid=2-s2.0-85101862235&doi=10.1186%2fs40900-020-00246-x&partnerID=40&md5=490451af0501f2f4633954f0ceb6bb39},
affiliation={Chronic Disease Initiative for Africa, Department of Medicine, University of Cape Town, Cape Town, South Africa; Centre for Social Science Research, University of Cape Town, Cape Town, South Africa; UCT Research Centre for Health through Physical Activity, Lifestyle and Sport, Division of Exercise Science and Sports Medicine, Faculty of Health Sciences, University of Cape Town, Cape Town, South Africa; Armauer Hansen Research Institute (AHRI), Addis Ababa, Ethiopia; Single Project Implementation Unit, University of Rwanda, Kigali, Rwanda; School of Medicine and Pharmacy, College of Medicine and Health Sciences, University of Rwanda, Kigali, Rwanda; Malawi Epidemiology and Intervention Research Unit, Lilongwe, Malawi; Centre for Reproductive Health, College of Medicine, University of Malawi, Blantyre, Malawi; College of Social Sciences, University of Glasgow, Glasgow, United Kingdom; School of Public Health and Family Medicine, College of Medicine, University of Malawi, Zomba, Malawi; Department of Epidemiology and Population Health, Stanford University School of Medicine, Stanford, CA, United States; Stanford Prevention Research Center, Stanford University School of Medicine, Stanford, CA, United States},
abstract={Background: In sub-Saharan Africa (SSA), which experiences a disproportionately high cardiovascular disease (CVD) burden, population-based screening and prevention measures are hampered by low levels of knowledge about CVD and associated risk factors, and inaccurate perceptions of severity of risk. Methods: This protocol describes the planned processes for implementing community-driven participatory research, using a citizen science method to explore CVD risk perceptions and to develop community-specific advocacy and prevention strategies in the rural and urban SSA settings. Multi-disciplinary research teams in four selected African countries will engage with and train community members living in rural and urban communities as citizen scientists to facilitate conceptualization, co-designing of research, data gathering, and co-creation of knowledge that can lead to a shared agenda to support

collaborative participation in community-engaged science. The emphasis is on robust community engagement, using mobile technology to support data gathering, participatory learning, and co-creation of knowledge and disease prevention advocacy. Discussion: Contextual processes applied and lessons learned in specific settings will support redefining or disassembling boundaries in participatory science to foster effective implementation of sustainable prevention intervention programmes in Low- and Middle-income countries. © 2021, The Author(s).},
author_keywords={Advocacy; Cardiovascular disease; Citizen science; Community engagement; Community-driven; Participatory learning; Risk perception; Sub-Saharan Africa},
references={Yusuf, S., Rangarajan, S., Teo, K., Islam, S., Li, W., Liu, L., Cardiovascular risk and events in 17 low-, middle-, and high-income countries (2014) N Engl J Med, 371 (9), pp. 818-827. , COI: 1:CAS:528:DC%2BC2cXhs12jtLbF; Keates, A.K., Mocumbi, A.O., Ntsekhe, M., Sliwa, K., Stewart, S., Cardiovascular disease in Africa: Epidemiological profile and challenges (2017) Nature Reviews Cardiology, 14, pp. 273-293; Cooney, M.T., Cooney, H.C., Dudina, A., Graham, I.M., Total cardiovascular disease risk assessment: a review (2011) Curr Opin Cardiol [Internet], 26 (5), pp. 429-437. , http://www.ncbi.nlm.nih.gov/pubmed/21822139; Lim, S.S., Gaziano, T.A., Gakidou, E., Reddy, K.S., Farzadfar, F., Lozano, R., Prevention of cardiovascular disease in high-risk individuals in low-income and middle-income countries: health effects and costs (2007) Lancet., 370 (9604), pp. 2054-2062; Boateng, D., Wekesah, F., Browne, J.L., Agyemang, C., Agyei-Baffour, P., De-Graft Aikins, A., Knowledge and awareness of and perception towards cardiovascular disease risk in sub-Saharan Africa: a systematic review (2017) PLoS One, 12 (12), pp. 1-21; Surka, S., Edirippulige, S., Steyn, K., Gaziano, T., Puoane, T., Levitt, N., Evaluating the use of Mobile phone technology to enhance cardiovascular disease screening by community health workers (2014) Int J Med Inform [Internet], 83 (9), pp. 6-12. , http://www.ncbi.nlm.nih.gov/pubmed/25002305; Gaziano, T.A., Abrahams-Gessel, S., Denman, C.A., Montano, C.M., Khanam, M., Puoane, T., An assessment of community health workers’ ability to screen for cardiovascular disease risk with a simple, non-invasive risk assessment instrument in Bangladesh, Guatemala, Mexico, and South Africa: an observational study (2015) Lancet Glob Heal, 3 (9), pp. e556-e563; Abrahams-Gessel, S., Denman, C.A., Montano, C.M., Gaziano, T.A., Levitt, N., Rivera-Andrade, A., The training and fieldwork experiences of community health workers conducting population-based, noninvasive screening for CVD in LMIC (2015) Glob Heart, 10 (1), pp. 45-54; Levitt, N.S., Puoane, T., Denman, C.A., Abrahams-gessel, S., Surka, S., Mendoza, C., Referral outcomes of individuals identified at high risk of cardiovascular disease by community health workers in Bangladesh, Guatemala, Mexico, and South Africa (2015) Glob Health Action, 8 (1), pp. 1-7; Surka, S., Steyn, K., Everett-Murphy, K., Gaziano, T.A., Levitt, N., Knowledge and perceptions of risk for cardiovascular disease: Findings of a qualitative investigation from a low-income peri-urban community in the Western Cape, South Africa (2015) African J Prim Heal Care Fam Med, 7 (1). , http://phcfm.org/index.php/phcfm/article/view/891; Okop, K.J., Mukumbang, F.C., Mathole, T., Levitt, N., Puoane, T., Perceptions of body size, obesity threat and the willingness to lose weight among black south African adults: a qualitative study (2016) BMC Public Health, 16 (1), p. 365. , http://bmcpublichealth.biomedcentral.com/articles/10.1186/s12889-016-3028-7; Gigerenzer, G., Gaissmaier, W., Kurz-milcke, E., Schwartz, L.M., Gigerenzer, G., Gaissmaier, W., Helping doctors and patients make sense of health

statistics (2007) Assoc Psychol Sci, 8 (2), pp. 53-96; Okop, K.J., Exploring the association between body image, body fat, and total cardiovascular disease risk among adults in a rural and an urban community of South Africa (2017) University of the Western Cape.; Bovet, P., Chiolero, A., Paccaud, F., Banatvala, N., Screening for cardiovascular disease risk and subsequent management in low and middle income countries: Challenges and opportunities (2015) Public Health Reviews, 36; Payne, Y.A., Participatory action research (2017) The Wiley-Blackwell Encyclopedia of Social Theory, 20, pp. 1-5; Morton Ninomiya, M., George, N., George, J., Linklater, R., Bull, J., Plain, S., A community-driven and evidence-based approach to developing mental wellness strategies in first nations: a program protocol (2020) Res Involv Engagem, 6 (1), pp. 1-12; Abayneh, S., Lempp, H., Hanlon, C., Branquinho, C., Tomé, G., Grothausen, T., Community-based youth participatory action research studies with a focus on youth health and well-being: a systematic review (2020) J Community Psychol, 6 (December 2019), pp. 1-14; King, A.C., Winter, S.J., Chrisinger, B.W., Hua, J., Banchoff, A.W., Maximizing the promise of citizen science to advance health and prevent disease (2019) Prev Med (Baltim), 119 (December 2018), pp. 44-47; Den Broeder, L., Devilee, J., Van Oers, H., Schuit, A.J., Wagemakers, A., Citizen science for public health (2018) Health Promot Int, 33 (3), pp. 505-514; McKinley, D.C., Miller-Rushing, A.J., Ballard, H.L., Bonney, R., Brown, H., Cook-Patton, S.C., Citizen science can improve conservation science, natural resource management, and environmental protection (2017) Biol Conserv, 208, pp. 15-28; Barrie, H., Soebarto, V., Lange, J., Corry-breen, F.M., Walker, L., Katapally, T.R., The rise of citizen science in health and biomedical research (2019) Am J Bioeth [Internet], 7 (1), pp. 44-47; Dickinson, J.L., Shirk, J., Bonter, D., Bonney, R., Crain, R.L., Martin, J., The current state of citizen science as a tool for ecological research and public engagement (2012) Front Ecol Environ, 10 (6), pp. 291-297; Becker, A., (1997) Book reviews: Alan Irwin: citizen science. A study of people, expertise and sustainable development. In: organization studies, , Routledge, London and New York; Wallerstein, N., Duran, B., Oetzel, J.G., Minkler, M., (2017) Community-Based Participatory Research for Health: Advancing Social and Health Equity: Wiley, pp. 19-29; Rehfuess, E.A., Durão, S., Kyamanywa, P., Meerpohl, J.J., Young, T., Rohwer, A., An approach for setting evidence-based and stakeholder-informed research priorities in low- and middle-income countries (2016) Bull World Health Organ, 94 (4), pp. 297-305; Macdonald, C.D., Understanding participatory action research: a qualitative research methodology option (2012) Can J Action Res, 13 (2), pp. 34-50; Vollman, A.R., Anderson, E.T., McFarlane, J., Canadian community as partner: Theory and multidisciplinary practice (2016) First. (ESP)., pp. 1-908. , WKHA, editor. Canadian Community As Partner: Theory and Multidisciplinary Practice; Kemmis, S., Wilkinson, M., Participatory action research and the study of practice (1998) Action Research in Practice; Partnership for Social Justice in Education, pp. 336-396. , In, Eds.) D& YSL, editor; Beauchemin, C., González-Ferrier, A., Sampling international migrants with origin-based snowballing method (2011) Demogr Res, 25, pp. 103-134; Hinckson, E., Schneider, M., Winter, S.J., Stone, E., Puhan, M., Stathi, A., Citizen science applied to building healthier community environments: advancing the field through shared construct and measurement development (2017) Int J Behav Nutr Phys Act, 14 (1). , http://ijbnpa.biomedcentral.com/articles/10.1186/s12966-017-0588-6, [, Internet,].;, (,):,., http://ijbnpa.biomedcentral.com/articles/10.1186/s12966-017-0588-6; Heigl, F., Zaller, J.G., Using a citizen science approach in higher education: a case study reporting Roadkills in Austria (2014) Hum Comput, 1 (2), pp. 165-175;

Doane, J., Schoenhals, S., Sherpa, A., Lama, L., Fassl, B., Levy, D., Implementing a mobile app for field global health research: successes, challenges, and future directions (2019) J Investig Med, 67 (1), p. 195; Odunitan-Wayas, F.A., Hamann, N., Sinyanya, N.A., King, A.C., Banchoff, A., Winter, S.J., A citizen science approach to determine perceived barriers and promoters of physical activity in a low-income south African community (2020) Glob Public Health, pp. 1-14. , https://doi.org/10.1080/17441692.2020.1712449, Internet]; King, A.C., King, D.K., Banchoff, A., Solomonov, S., Ben, N.O., Hua, J., Employing participatory citizen science methods to promote age-friendly environments worldwide (2020) Int J Environ Res Public Health, 17, p. 1541; Braun, V., Clarke, V., Using thematic analysis in psychology (2006) Qual Res Psychol, 3 (2), pp. 77-101; Asingizwe, D., Poortvliet, P.M., Koenraadt, C.J.M., Van Vliet, A.J.H., Murindahabi, M.M., Ingabire, C., Applying citizen science for malaria prevention in Rwanda: an integrated conceptual framework (2018) NJAS - Wageningen J Life Sci, 86-87 (August 2017), pp. 111-122},
correspondence_address1={Okop, K.J.; Centre for Social Science Research, South Africa; email: kufreokop@gmail.com},
publisher={BioMed Central Ltd},
issn={20567529},
language={English},
abbrev_source_title={Res. Involv. Engagem.},
document_type={Article},
source={Scopus},
}

@ARTICLE{Batsis2021,
author={Batsis, J.A. and Petersen, C.L. and Clark, M.M. and Cook, S.B. and Kotz, D. and Gooding, T.L. and Roderka, M.N. and Al-Nimr, R.I. and Pidgeon, D. and Haedrich, A. and Wright, K.C. and Aquila, C. and Mackenzie, T.A.},
title={Feasibility and acceptability of a technology-based, rural weight management intervention in older adults with obesity},
journal={BMC Geriatrics},
year={2021},
volume={21},
number={1},
doi={10.1186/s12877-020-01978-x},
art_number={44},
note={cited By 0},
url={https://www.scopus.com/inward/record.uri?eid=2-s2.0-85099228070&doi=10.1186%2fs12877-020-01978-x&partnerID=40&md5=b15224435a2c6f1d1bd6c276e02f7dcf},
affiliation={Division of Geriatric Medicine, School of Medicine, and Department of Nutrition, Gillings School of Global Public Health, University of North Carolina at Chapel Hill, 5017 Old Clinic Building, Chapel Hill, NC 27599, United States; Dartmouth-Hitchcock, Geisel School of Medicine, and The Dartmouth Institute for Health Policy, Hanover, NH, United States; Dartmouth College, Hanover, NH, United States; Mayo Clinic Rochester, Department of Psychiatry and Psychology, and Division of Endocrinology, Rochester, MN, United States; University of New Hampshire, Durham, NH, United States},
abstract={Background: Older adults with obesity residing in rural areas have reduced access to weight management programs. We determined the feasibility, acceptability and preliminary outcomes of an integrated technology-based health promotion intervention in rural-living, older adults using remote monitoring and synchronous video-based technology. Methods: A 6-month, non-randomized, non-blinded, single-arm study was conducted from October 2018 to May 2020 at a

community-based aging center of adults aged ≥65 years with a body mass index (BMI) ≥30 kg/m2. Weekly dietitian visits focusing on behavior therapy and caloric restriction and twice-weekly physical therapist-led group strength, flexibility and balance training classes were delivered using video-conferencing to participants in their homes. Participants used a Fitbit Alta HR for remote monitoring with data feedback provided by the interventionists. An aerobic activity prescription was provided and monitored. Results: Mean age was 72.9±3.9 years (82% female). Baseline anthropometric measures of weight, BMI, and waist circumference were 97.8±16.3 kg, 36.5±5.2 kg/m2, and 115.5±13.0 cm, respectively. A total of 142 participants were screened (n=27 ineligible), and 53 consented. There were nine dropouts (17%). Overall satisfaction with the trial (4.7+ 0.6, scale: 1 (low) to 5 (high)) and with Fitbit (4.2+ 0.9) were high. Fitbit was worn an average of 81.7±19.3% of intervention days. In completers, mean weight loss was 4.6±3.5 kg or 4.7±3.5% (p< 0.001). Physical function measures of 30-s sit-to-stand repetitions increased from 13.5±5.7 to 16.7±5.9 (p< 0.001), 6-min walk improved by 42.0±77.3 m (p=0.005) but no differences were observed in gait speed or grip strength. Subjective measures of late-life function improved (3.4±4.7 points, p< 0.001). Conclusions: A technology-based obesity intervention is feasible and acceptable to older adults with obesity and may lead to weight loss and improved physical function. Clinical trial registration: Registered on Clinicaltrials.gov #NCT03104205. Registered on April 7, 2017. First participant enrolled on October 1st, 2018. © 2021, The Author(s).},
author_keywords={Disparities; Telehealth; Weight},
references={Hales, C.M., Fryar, C.D., Carroll, M.D., Freedman, D.S., Ogden, C.L., Trends in obesity and severe obesity prevalence in US youth and adults by sex and age, 2007-2008 to 2015-2016 (2018) JAMA, 319 (16), pp. 1723-1725. , PID: 29570750; Jensen, G.L., Friedmann, J.M., Obesity is associated with functional decline in community-dwelling rural older persons (2002) J Am Geriatr Soc, 50 (5), pp. 918-923. , PID: 12028181; Zizza, C.A., Herring, A., Stevens, J., Popkin, B.M., Obesity affects nursing-care facility admission among whites but not blacks (2002) Obes Res, 10 (8), pp. 816-823. , PID: 12181391; Flegal, K.M., Kit, B.K., Orpana, H., Graubard, B.I., Association of all-cause mortality with overweight and obesity using standard body mass index categories: a systematic review and meta-analysis (2013) JAMA, 309 (1), pp. 71-82. , COI: 1:CAS:528:DC%2BC3sXlvVWjuw%3D%3D, PID: 23280227; Musich, S., MacLeod, S., Bhattarai, G.R., Wang, S.S., Hawkins, K., Bottone, F.G., Jr., Yeh, C.S., The impact of obesity on health care utilization and expenditures in a Medicare supplement population (2016) Gerontol Geriatr Med, 2. , PID: 28138482; Batsis, J.A., Gill, L.E., Masutani, R.K., Adachi-Mejia, A.M., Blunt, H.B., Bagley, P.J., Lopez-Jimenez, F., Bartels, S.J., Weight loss interventions in older adults with obesity: a systematic review of randomized controlled trials since 2005 (2017) J Am Geriatr Soc, 65 (2), pp. 257-268. , PID: 27641543; Villareal, D.T., Aguirre, L., Gurney, A.B., Waters, D.L., Sinacore, D.R., Colombo, E., Armamento-Villareal, R., Qualls, C., Aerobic or resistance exercise, or both in dieting obese older adults (2017) N Engl J Med, 376, pp. 1943-1955. , PID: 28514618; Nicklas, B.J., Brinkley, T.E., Houston, D.K., Lyles, M.F., Hugenschmidt, C.E., Beavers, K.M., Leng, X., Effects of caloric restriction on cardiorespiratory fitness, fatigue, and disability responses to aerobic exercise in older adults with obesity: a randomized controlled trial (2019) J Gerontol A Biol Sci Med Sci, 74 (7), pp. 1084-1090. , COI: 1:CAS:528:DC%2BB3cXhtlGit7rM, PID: 29982294; Strandberg, T.E., Strandberg, A.Y., Salomaa, V.V., Pitkala, K.H., Tilvis, R.S., Sirola, J., Miettinen, T.A., Explaining the obesity paradox: cardiovascular risk, weight change, and

mortality during long-term follow-up in men (2009) Eur Heart J, 30 (14), pp. 1720-1727. , PID: 19429917; Aleem, S., Lasky, R., Brooks, W.B., Batsis, J.A., Obesity perceptions and documentation among primary care clinicians at a rural academic health center (2015) Obes Res Clin Pract, 9 (4), pp. 408-415. , PID: 26359306; Kahan, S., Kushner, R.F., Obesity Medicine: A Core Competency for Primary Care Providers (2018) Med Clin North Am, 102 (1), pp. xvii-xix. , PID: 29156191; Dewar, S., Bynum, J., Batsis, J.A., Uptake of Obesity Intensive Behavioral Treatment Codes in Medicare Beneficiaries, 2012-2015 (2020) J Gen Intern Med, 35 (1), pp. 368-370. , https://doi.org/10.1007/s11606-019-05437-1; Trivedi, T., Liu, J., Probst, J., Merchant, A., Jhones, S., Martin, A.B., Obesity and obesity-related behaviors among rural and urban adults in the USA (2015) Rural Remote Health, 15 (4), p. 3267. , PID: 26458564; Bolin, J.N., Bellamy, G.R., Ferdinand, A.O., Vuong, A.M., Kash, B.A., Schulze, A., Helduser, J.W., Rural healthy people 2020: new decade, Same Challenges (2015) J Rural Health, 31 (3), pp. 326-333. , PID: 25953431; Foley, D.J., Heimovitz, H.K., Guralnik, J.M., Brock, D.B., Driving life expectancy of persons aged 70 years and older in the United States (2002) Am J Public Health, 92 (8), pp. 1284-1289. , PID: 12144985; (2018) Achieving Rural Health Equity and Well-Being: Proceedings of a Workshop, , The National Academies Press, Washington, DC; Batsis, J.A., Naslund, J.A., Zagaria, A.B., Kotz, D., Dokko, R., Bartels, S.J., Carpenter-Song, E., Technology for Behavioral Change in rural older adults with obesity (2019) J Nutr Gerontol Geriatr, 38 (2), pp. 130-148. , PID: 30971189; Befort, C.A., Klemp, J.R., Austin, H.L., Perri, M.G., Schmitz, K.H., Sullivan, D.K., Fabian, C.J., Outcomes of a weight loss intervention among rural breast cancer survivors (2012) Breast Cancer Res Treat, 132 (2), pp. 631-639. , PID: 22198470; Batsis, J.A., Petersen, C.L., Clark, M.M., Cook, S.B., Lopez-Jimenez, F., Al-Nimr, R.I., Pidgeon, D., Bartels, S.J., A Weight Loss Intervention Augmented by a Wearable Device in Rural Older Adults With Obesity: A Feasibility Study (2021) J Gerontol A Biol Sci Med Sci, 76 (1), pp. 95-100. , (,):, -,., https://doi.org/10.1093/gerona/glaa115; Venditti, E.M., Zgibor, J.C., Vander Bilt, J., Kieffer, L.A., Boudreau, R.M., Burke, L.E., Glynn, N.W., Semler, L.N., Mobility and Vitality Lifestyle Program (MOVE UP): A Community Health Worker Intervention for Older Adults With Obesity to Improve Weight, Health, and Physical Function (2018) Innov Aging, 2 (2), p. igy012. , PID: 30480135; Leahey, T.M., Wing, R.R., A randomized controlled pilot study testing three types of health coaches for obesity treatment: professional, peer, and mentor (2013) Obesity (Silver Spring), 21 (5), pp. 928-934; Batsis, J.A., Zbehlik, A.J., Barre, L.K., Bynum, J.P., Pidgeon, D., Bartels, S.J., Impact of obesity on disability, function, and physical activity: data from the osteoarthritis initiative (2015) Scand J Rheumatol, 44 (6), pp. 495-502. , COI: 1:STN:280:DC%2BC2Mbkt1Shug%3D%3D, PID: 26083472; Visser, M., Obesity, sarcopenia and their functional consequences in old age (2011) Proc Nutr Soc, 70 (1), pp. 114-118. , PID: 21092365; Wing, R.R., Crane, M.M., Thomas, J.G., Kumar, R., Weinberg, B., Improving weight loss outcomes of community interventions by incorporating behavioral strategies (2010) Am J Public Health, 100 (12), pp. 2513-2519. , PID: 20966375; Gamm, L.D., Hutchison, L.L., Dabney, B.J., Dorsey, A.M., (2003) Rural health people 2010: a companion document to health people 2010, vol. 1, , The Texas A&M University System Health Science Center, School of Rural Public Health, Southwest Rural Health Research Center, College Station; Davis, A.M., Sampilo, M., Gallagher, K.S., Dean, K., Saroja, M.B., Yu, Q., He, J., Sporn, N., Treating rural paediatric obesity through telemedicine vs. telephone: outcomes from a cluster randomized controlled trial (2016) J Telemed Telecare, 22 (2), pp. 86-95. , PID: 26026186; (2019),

https://www.pewresearch.org/internet/chart/internet-use-by-age/, Pew Internet Survey,. Accessed 9 Jan 2020; Batsis, J.A., Dimilia, P.R., Seo, L.M., Fortuna, K.L., Kennedy, M.A., Blunt, H.B., Bagley, P.J., Bartels, S.J., Effectiveness of Ambulatory Telemedicine Care in Older Adults: A Systematic Review (2019) J am Geriatr Soc, 67 (8), pp. 1737-1749. , https://doi.org/10.1111/jgs.15959, Aug, Epub 2019; Batsis, J.A., Naslund, J.A., Gill, L.E., Masutani, R.K., Agarwal, N., Bartels, S.J., Use of a wearable activity device in rural older obese adults: a pilot study (2016) Gerontol Geriatr Med, 2. , PID: 28138502; Callahan, C.M., Unverzagt, F.W., Hui, S.L., Perkins, A.J., Hendrie, H.C., Six-item screener to identify cognitive impairment among potential subjects for clinical research (2002) Med Care, 40 (9), pp. 771-781. , PID: 12218768; Doble, S.E., Fisher, A.G., The dimensionality and validity of the older Americans resources and services (OARS) activities of daily living (ADL) scale (1998) J Outcome Meas, 2 (1), pp. 4-24. , COI: 1:STN:280:DyaK1czis1ektg%3D%3D, PID: 9661728; Bandura, A., Health promotion by social cognitive means (2004) Health Educ Behav, 31 (2), pp. 143-164; Venkatesh, V., Determinants of perceived ease ofUse: integrating control, intrinsic motivation, and emotion into the technology acceptance model (2000) Inf Syst Res, 11 (4), pp. 342-368; Siervo, M., Bertoli, S., Battezzati, A., Wells, J.C., Lara, J., Ferraris, C., Tagliabue, A., Accuracy of predictive equations for the measurement of resting energy expenditure in older subjects (2014) Clin Nutr, 33 (4), pp. 613-619. , COI: 1:STN:280:DC%2BC2c%2FjtVGjtg%3D%3D, PID: 24094813; Riebe, D., Franklin, B.A., Thompson, P.D., Garber, C.E., Whitfield, G.P., Magal, M., Pescatello, L.S., Updating ACSM's recommendations for exercise Preparticipation health screening (2015) Med Sci Sports Exerc, 47 (11), pp. 2473-2479. , COI: 1:CAS:528:DC%2BC2MXhslSqu7bI, PID: 26473759; Garber, C.E., Blissmer, B., Deschenes, M.R., Franklin, B.A., Lamonte, M.J., Lee, I.M., Nieman, D.C., Swain, D.P., American College of Sports M: American College of Sports Medicine position stand. Quantity and quality of exercise for developing and maintaining cardiorespiratory, musculoskeletal, and neuromotor fitness in apparently healthy adults: guidance for prescribing exercise (2011) Med Sci Sports Exerc, 43 (7), pp. 1334-1359; Garber, C.E., Blissmer, B., Deschenes, M.R., Franklin, B.A., Lamonte, M.J., Lee, I.M., Nieman, D.C., Swain, D.P., Quantity and Quality of Exercise for Developing and Maintaining Cardiorespiratory, Musculoskeletal, and Neuromotor Fitness in Apparently Healthy Adults: Guidance for Prescribing Exercise (2011) Med Sci Sports Exerc, 43 (7), pp. 1334-1359; Borg, G.A., Psychophysical bases of perceived exertion (1982) Med Sci Sports Exerc, 14 (5), pp. 377-381. , COI: 1:STN:280:DyaL3s7hsVChsw%3D%3D, PID: 7154893; Piercy Katrina, L., Troiano Richard, P., Physical activity guidelines for Americans from the US Department of Health and Human Services (2018) Circ Cardiovasc Qual Outcomes, 11 (11). , COI: 1:STN:280:DC%2BB3cnktF2jtw%3D%3D, PID: 30571339; Nicklas, B.J., Chmelo, E., Delbono, O., Carr, J.J., Lyles, M.F., Marsh, A.P., Effects of resistance training with and without caloric restriction on physical function and mobility in overweight and obese older adults: a randomized controlled trial (2015) Am J Clin Nutr, 101 (5), pp. 991-999. , COI: 1:CAS:528:DC%2BC2MXptlCgs74%3D, PID: 25762810; Pahor, M., Guralnik, J.M., Ambrosius, W.T., Blair, S., Bonds, D.E., Church, T.S., Espeland, M.A., Groessl, E.J., Effect of structured physical activity on prevention of major mobility disability in older adults: the LIFE study randomized clinical trial (2014) JAMA, 311 (23), pp. 2387-2396. , COI: 1:CAS:528:DC%2BC2cXhsFemt7%2FO, PID: 24866862; Stiggelbout, M., Hopman-Rock, M., Tak, E., Lechner, L., Van Mechelen, W., Dropout from exercise programs for seniors: a prospective cohort study (2005) J Aging Phys Act, 13 (4), pp. 409-421; Hawley-Hague, H., Horne, M., Skelton, D.A., Todd, C., Review of how we should define (and measure) adherence

in studies examining older adults' participation in exercise classes (2016) BMJ Open, 6 (6). , COI: 1:STN:280:DC%2BC2s7jsFegtg%3D%3D, PID: 27338884; Prescott, S., Traynor, J.P., Shilliday, I., Zanotto, T., Rush, R., Mercer, T.H., Minimum accelerometer wear-time for reliable estimates of physical activity and sedentary behaviour of people receiving haemodialysis (2020) BMC Nephrol, 21 (1), p. 230. , PID: 32546225; Aadland, E., Ylvisåker, E., Reliability of the Actigraph GT3X+ accelerometer in adults under free-living conditions (2015) PLoS One, 10 (8). , PID: 26274586, COI: 1:CAS:528:DC%2BC28XkvVertg%3D%3D; Subar, A.F., Kirkpatrick, S.I., Mittl, B., Zimmerman, T.P., Thompson, F.E., Bingley, C., Willis, G., McNutt, S., The automated self-administered 24-hour dietary recall (ASA24): a resource for researchers, clinicians and educators from the National Cancer Institute (2012) J Acad Nutr Diet, 112 (8), pp. 1134-1137. , PID: 22704899; Jensen, M.D., Ryan, D.H., Apovian, C.M., Ard, J.D., Comuzzie, A.G., Donato, K.A., Hu, F.B., Kushner, R.F., 2013 AHA/ACC/TOS guideline for the Management of Overweight and Obesity in adults (2014) Circulation, 129, pp. S102-S138. , PID: 24222017; Lauretani, F., Russo, C.R., Bandinelli, S., Bartali, B., Cavazzini, C., Di Iorio, A., Corsi, A.M., Ferrucci, L., Age-associated changes in skeletal muscles and their effect on mobility: an operational diagnosis of sarcopenia (2003) J Appl Physiol, 95 (5), pp. 1851-1860. , PID: 14555665; Jones, C.J., Rikli, R.E., Beam, W.C., A 30-s chair-stand test as a measure of lower body strength in community-residing older adults (1999) Res Q Exerc Sport, 70 (2), pp. 113-119. , COI: 1:STN:280:DyaK1MzgvFensg%3D%3D, PID: 10380242; Zhang, Q., Li, Y.X., Li, X.L., Yin, Y., Li, R.L., Qiao, X., Li, W., Han, Y.F., A comparative study of the five-repetition sit-to-stand test and the 30-second sit-to-stand test to assess exercise tolerance in COPD patients (2018) Int J Chron Obstruct Pulmon Dis, 13, pp. 2833-2839. , PID: 30237707; Petersen, C., Steffen, T., Paly, E., Dvorak, L., Nelson, R., Reliability and minimal detectable change for sit-to-stand tests and the functional gait assessment for individuals with Parkinson disease (2017) J Geriatr Phys Ther, 40 (4), pp. 223-226. , PID: 27805924; Wright, A.A., Cook, C.E., Baxter, G.D., Dockerty, J.D., Abbott, J.H., A comparison of 3 methodological approaches to defining major clinically important improvement of 4 performance measures in patients with hip osteoarthritis (2011) J Orthop Sports Phys Ther, 41 (5), pp. 319-327. , PID: 21335930; Bohannon, R.W., Minimal clinically important difference for grip strength: a systematic review (2019) J Phys Ther Sci, 31 (1), pp. 75-78. , PID: 30774209; ATS statement: guidelines for the six-minute walk test (2002) Am J Respir Crit Care Med, 166 (1), pp. 111-117; Bohannon, R.W., Crouch, R., Minimal clinically important difference for change in 6-minute walk test distance of adults with pathology: a systematic review (2017) J Eval Clin Pract, 23 (2), pp. 377-381. , PID: 27592691; Sayers, S.P., Jette, A.M., Haley, S.M., Heeren, T.C., Guralnik, J.M., Fielding, R.A., Validation of the late-life function and disability instrument (2004) J Am Geriatr Soc, 52 (9), pp. 1554-1559. , PID: 15341561; Van Domelen, D.R., (2018) Accelerometry: functions for processing minute-to-minute accelerometer data. R Package version 3.1.2; Vaportzis, E., Clausen, M.G., Gow, A.J., Older adults perceptions of technology and barriers to interacting with tablet computers: a focus group study (2017) Front Psychol, 8, p. 1687. , PID: 29071004; Mody, L., Miller, D.K., McGloin, J.M., Freeman, M., Marcantonio, E.R., Magaziner, J., Studenski, S., Recruitment and retention of older adults in aging research (2008) J Am Geriatr Soc, 56 (12), pp. 2340-2348. , PID: 19093934; Acton, G., Health-promoting self-Care in Family Caregivers (2002) West J Nurs Res, 24, pp. 73-86. , PID: 11829276; Bernard, M.A., Clayton, J.A., Lauer, M.S., Inclusion Across the Lifespan: NIH Policy for Clinical Research (2018) JAMA, 320 (15), pp. 1535-1536; Brown, J.D., Hales, S., Evans, T.E.,

Turner, T., Sword, D.O., O'neil, P.M., Ballentine, S., Dubose-Morris, R.A., Description, utilisation and results from a telehealth primary care weight management intervention for adults with obesity in South Carolina (2020) J Telemed Telecare, 26 (1-2), pp. 28-35. , https://doi.org/10.1177/1357633X18789562, Epub 2018; Chung, L.M., Law, Q.P., Fong, S.S., Chung, J.W., Yuen, P.P., A cost-effectiveness analysis of teledietetics in short-, intermediate-, and long-term weight reduction (2015) J Telemed Telecare, 21 (5), pp. 268-275. , COI: 1:STN:280:DC%2BC2MnjtF2ntQ%3D%3D, PID: 25761467; Huang, J.W., Lin, Y.Y., Wu, N.Y., The effectiveness of telemedicine on body mass index: A systematic review and meta-analysis (2019) J Telemed Telecare, 25 (7), pp. 389-401. , https://doi.org/10.1177/1357633X18775564, . Epub 2018; Rutledge, T., Skoyen, J.A., Wiese, J.A., Ober, K.M., Woods, G.N., A comparison of MOVE! Versus TeleMOVE programs for weight loss in veterans with obesity (2017) Obes Res Clin Pract, 11 (3), pp. 344-351. , PID: 27931766; Barnason, S., Zimmerman, L., Schulz, P., Pullen, C., Schuelke, S., Weight management telehealth intervention for overweight and obese rural cardiac rehabilitation participants: A randomised trial (2019) J Clin Nurs., 28 (9-10), pp. 1808-1818. , https://doi.org/10.1111/jocn.14784, Epub 2019; Hong, J., Kim, J., Kim, S.W., Kong, H.J., Effects of home-based tele-exercise on sarcopenia among community-dwelling elderly adults: Body composition and functional fitness (2017) Exp Gerontol, 87, pp. 33-39. , PID: 27838369; Haugen, H.A., Tran, Z.V., Wyatt, H.R., Barry, M.J., Hill, J.O., Using telehealth to increase participation in weight maintenance programs (2007) Obesity (Silver Spring), 15 (12), pp. 3067-3077; Labiris, G., Panagiotopoulou, E.K., Kozobolis, V.P., A systematic review of teleophthalmological studies in Europe (2018) Int J Ophthalmol, 11 (2), pp. 314-325. , PID: 29487825; de Swanepoel, W., Hall, J.W., 3rd, A systematic review of telehealth applications in audiology (2010) Telemed J E Health, 16 (2), pp. 181-200; Narasimha, S., Madathil, K.C., Agnisarman, S., Rogers, H., Welch, B., Ashok, A., Nair, A., McElligott, J., Designing telemedicine Systems for Geriatric Patients: a review of the usability studies (2016) Telemed e-Health, 23 (6), pp. 459-472; Van Orden, K.A., Bower, E., Lutz, J., Silva, C., Gallegos, A.M., Podgorski, C.A., Santos, E.J., Conwell, Y., Strategies to Promote Social Connections Among Older Adults During 'Social Distancing' Restrictions (2020) Am J Geriatr Psychiatry, , https://doi.org/10.1016/j.jagp.2020.1005.1004; Fedele, D.A., Cushing, C.C., Fritz, A., Amaro, C.M., Ortega, A., Mobile health interventions for improving health outcomes in youth: a meta-analysis (2017) JAMA Pediatr, 171 (5), pp. 461-469. , PID: 28319239; Romeo, A., Edney, S., Plotnikoff, R., Curtis, R., Ryan, J., Sanders, I., Crozier, A., Maher, C., Can smartphone apps increase physical activity? Systematic review and meta-analysis (2019) J Med Internet Res, 21 (3). , PID: 30888321; Jakicic, J.M., Davis, K.K., Rogers, R.J., King, W.C., Marcus, M.D., Helsel, D., Rickman, A.D., Belle, S.H., Effect of wearable technology combined with a lifestyle intervention on long-term weight loss: the IDEA randomized clinical trial (2016) JAMA, 316 (11), pp. 1161-1171. , PID: 27654602; Spring, B., Duncan, J.M., Janke, E.A., Kozak, A.T., McFadden, H.G., DeMott, A., Pictor, A., Pellegrini, C.A., Integrating technology into standard weight loss treatment: a randomized controlled trial (2013) JAMA Intern Med, 173 (2), pp. 105-111. , PID: 23229890; McDermott, M.M., Spring, B., Berger, J.S., Treat-Jacobson, D., Conte, M.S., Creager, M.A., Criqui, M.H., Guralnik, J.M., Effect of a home-based exercise intervention of wearable technology and telephone coaching on walking performance in peripheral artery disease: the HONOR randomized clinical trial (2018) JAMA, 319 (16), pp. 1665-1676. , PID: 29710165; Hutchesson, M.J., Rollo, M.E., Krukowski, R., Ells, L., Harvey, J.,

Morgan, P.J., Callister, R., Collins, C.E., eHealth interventions for the prevention and treatment of overweight and obesity in adults: a systematic review with meta-analysis (2015) Obes Rev, 16 (5), pp. 376-392. , COI: 1:STN:280:DC%2BC2MnitlegtQ%3D%3D, PID: 25753009; Patel, M.L., Wakayama, L.N., Bass, M.B., Breland, J.Y., Motivational interviewing in eHealth and telehealth interventions for weight loss: a systematic review (2019) Prev Med, 126, p. 105738. , PID: 31153917; (2020) Internet Service in New Hampshire, , https://www.pewresearch.org/internet/chart/internet-use-by-age/, . Accessed 1 Oct 2020},
correspondence_address1={Batsis, J.A.; Division of Geriatric Medicine, 5017 Old Clinic Building, United States; email: john.batsis@gmail.com},
publisher={BioMed Central Ltd},
issn={14712318},
pubmed_id={33435877},
language={English},
abbrev_source_title={BMC Geriatr.},
document_type={Article},
source={Scopus},
}

@ARTICLE{Wang2021,
author={Wang, Z. and Li, C. and Huang, W. and Chen, Y. and Li, Y. and Huang, L. and Zhang, M. and Wu, D. and Wang, L. and Duan, H. and An, J. and Deng, N.},
title={Effectiveness of a pathway-driven eHealth-based integrated care model (PEICM) for community-based hypertension management in China: study protocol for a randomized controlled trial},
journal={Trials},
year={2021},
volume={22},
number={1},
doi={10.1186/s13063-021-05020-2},
art_number={81},
note={cited By 0},
url={https://www.scopus.com/inward/record.uri?eid=2-s2.0-85099738242&doi=10.1186%2fs13063-021-05020-2&partnerID=40&md5=4e4ae8fc936ea52289c7d434ee52ee99},
affiliation={College of Biomedical Engineering and Instrument Science, The Ministry of Education Key Laboratory of Biomedical Engineering, Zhejiang University, Hangzhou, 310027, China; The First People’s Hospital of Yibin, Yibin, China; ZICT Technology Co.,Ltd., Shenzhen, China},
abstract={Background: The prevalence of hypertension is high and increasing in China in recent years. The treatment and control of hypertension calls for long-term management beyond hospital, which is hard to implement in traditional care settings. Integrated care combined with information technology can promote high-quality healthcare services across the life-course. However, few studies have applied a customized integrated care model in community-based hypertension management in China, catering to the emerging “three-manager” mode. This study aims to identify the effectiveness of a pathway-driven eHealth-based integrated model that implemented as a full-featured telehealth system to facilitate standardized management of hypertension in China. Methods: The trial has been designed as a 1-year, non-blinded superiority trial with two parallel groups. A total of 402 hypertensive patients who meet the eligibility criteria will be recruited and randomized with a 1:1 allocation. All the participants will receive a mobile device for self-management, which is a part of our telehealth system. Participants in the control group will only use the device for BP

measurement and receive regular follow-ups from care providers according to the guidelines. Participants in the intervention group will gain full access to the system and receive intervention based on the proposed model (a well-designed coordinated care pathway consisting of 9 tasks). Outcomes will be measured mainly on three occasions (at inclusion, at 6 months, and at 12 months). The primary outcome is mean change in systolic blood pressure over a 12-month period. Secondary outcomes include changes in diastolic blood pressure, biochemical indexes related to hypertension, lifestyles, self-management adherence, and hypertension awareness, as well as work efficiency of care providers. Discussion: This study aims to investigate whether a pathway-driven eHealth-based integrated care model based on the “three-manager” mode will improve hypertension control in China. Success of the model would help improve the quality of present community-based management procedures and benefit more patients with uncontrolled hypertension. Trial registration: Chinese Clinical Trial Registry ChiCTR1900027645. Registered on November 22, 2019. © 2021, The Author(s).},
author_keywords={Community; Hypertension management; Integrated care; Pathway; Randomized controlled trial; Study protocol; Telehealth},
keywords={adult; article; awareness; blood pressure monitoring; catering service; China; clinical trial registry; controlled study; diastolic blood pressure; eligibility criteria; female; human; hypertensive patient; lifestyle; major clinical study; male; manager; outcome assessment; parallel design; practice guideline; randomized controlled trial; self care; single blind procedure; superiority trial; telehealth},
references={Wang, Z., Chen, Z., Zhang, L., Wang, X., Hao, G., Zhang, Z., Status of hypertension in China (2018) Circulation, 137 (22), pp. 2344-2356; Reynolds, R., Dennis, S., Hasan, I., Slewa, J., Chen, W., Tian, D., A systematic review of chronic disease management interventions in primary care (2018) BMC Fam Pract, 19 (1), p. 11; Nguyen, Q., Dominguez, J., Nguyen, L., Gullapalli, N., Hypertension management: an update (2010) Am Heal Drug Benefits, 3 (1), pp. 47-56. , http://www.ncbi.nlm.nih.gov/pubmed/25126308, Available from: http://www.ncbi.nlm.nih.gov/pubmed/25126308; (2016) Integrated Care Models: An Overview, , https://www.euro.who.int/__data/assets/pdf_file/0005/322475/Integrated-care-models-overview.pdf.Accessed18Jan2020, Internet, available from; (2016), https://www.euro.who.int/__data/assets/pdf_file/0004/315787/66wd15e_FFA_IHSD_160535.pdf, WHO Regional Office for Europe. Strengthening people-centred health systems in the WHO European Region: framework for action on integrated health services delivery [Internet], available from:, Accessed 18 Jan 2020; Hamine, S., Gerth-Guyette, E., Faulx, D., Green, B.B., Ginsburg, A.S., Impact of mHealth chronic disease management on treatment adherence and patient outcomes: a systematic review (2015) J Med Internet Res, 17 (2); Kumar, N., Khunger, M., Gupta, A., Garg, N., A content analysis of smartphone-based applications for hypertension management (2015) J Am Soc Hypertens, 9 (2), pp. 130-136; Harrison, C.E., Wild, K., Using telehealth in the management of hypertension (2017) Nurs Stand, 31 (48), pp. 44-49; Curry, N., Ham, C., Clinical and service integration: The route to improved outcomes. [Internet] (2010) The King’s Fund, , https://www.kingsfund.org.uk/sites/files/kf/Clinical-and-service-integration-Natasha-Curry-Chris-Ham-22-November-2010.pdf, Accessed 19 Jan 2020; Wagner, E.H., Austin, B.T., Von Korff, M., Organizing care for patients with chronic illness (1996) Milbank Q, 74 (4), pp. 511-544. , http://www.ncbi.nlm.nih.gov/pubmed/8941260, COI: 1:STN:280:DyaK2s%2Fps1agtA%3D%3D, Available from:

http://www.ncbi.nlm.nih.gov/pubmed/8941260; Wagner, E.H., Austin, B.T., Davis, C., Hindmarsh, M., Schaefer, J., Bonomi, A., Improving chronic illness care: translating evidence into action (2001) Health Aff, 20 (6), pp. 64-78; Bodenheimer, T., Wagner, E.H., Grumbach, K., Improving primary care for patients with chronic illness (2002) JAMA, 288 (14), pp. 1775-1779. , http://www.ncbi.nlm.nih.gov/pubmed/12365965, Available from: http://www.ncbi.nlm.nih.gov/pubmed/12365965; Bodenheimer, T., Wagner, E.H., Grumbach, K., Improving primary care for patients with chronic illness: the chronic care model, Part 2 (2002) JAMA, 288 (15), pp. 1909-1914. , http://www.ncbi.nlm.nih.gov/pubmed/12377092, Available from: http://www.ncbi.nlm.nih.gov/pubmed/12377092; Coleman, K., Austin, B.T., Brach, C., Wagner, E.H., Evidence on the Chronic Care Model in the new millennium (2009) Health Aff (Millwood), 28 (1), pp. 75-85; Gee, P.M., Greenwood, D.A., Paterniti, D.A., Ward, D., Miller, L.M.S., The eHealth Enhanced Chronic Care Model: a theory derivation approach (2015) J Med Internet Res, 17 (4); 2018 Chinese Guidelines for Prevention and Treatment of Hypertension-a report of the Revision Committee of Chinese Guidelines for Prevention and Treatment of Hypertension (2019) J Geriatr Cardiol, 16 (3), pp. 182-241; Yang, S., Introduction of general practitioner education and chronic disease management model in Xiamen (2017) Chinese Gen Pract, 20 (202), pp. 2526-2527; Bourgueil, Y., Marek, A., Mousques, J., Practice, role and position of nurses in primary care in six European countries, in Ontario and in Quebec (2008) Rech Soins Infirm, (93), pp. 94-105. , http://www.ncbi.nlm.nih.gov/pubmed/18678084.Accessed1Feb2020, Available from; van Dillen, S.M.E., Hiddink, G.J., To what extent do primary care practice nurses act as case managers lifestyle counselling regarding weight management? A systematic review (2014) BMC Fam Pract, 15, p. 197; Feng, X.L., Pang, M., Beard, J., Health system strengthening and hypertension awareness, treatment and control: data from the China Health and Retirement Longitudinal Study (2014) Bull World Health Organ, 92 (1), pp. 29-41; Hernandez, J., Anderson, S., Storied experiences of nurse practitioners managing prehypertension in primary care (2012) J Am Acad Nurse Pract, 24 (2), pp. 89-96; Li, H., Hypertension management in primary care in China: still a long way to proceed (2015) J Gen Pract, 4 (2), pp. 2-3; Li, H., Wei, X., Wong, M.C., Yang, N., Wong, S.Y., Lao, X., A comparison of the quality of hypertension management in primary care between Shanghai and Shenzhen: a cohort study of 3196 patients (2015) Medicine (Baltimore), 94 (5); Chan, A.-W., Tetzlaff, J.M., Altman, D.G., Laupacis, A., Gøtzsche, P.C., Krleža-Jerić, K., SPIRIT 2013 statement: defining standard protocol items for clinical trials (2013) Ann Intern Med, 158 (3), pp. 200-207; Hoffmann, T.C., Glasziou, P.P., Boutron, I., Milne, R., Perera, R., Moher, D., Better reporting of interventions: template for intervention description and replication (TIDieR) checklist and guide (2014) BMJ, 348, p. g1687; (2017) China City Statistical Yearbook 2017, , http://www.stats.gov.cn/tjsj/ndsj/2017/indexeh.htm, [Internet]. China Statistics Press;, Accessed 3 Feb 2020; Wang, X., Li, W., Li, X., An, N., Chen, H., Jan, S., Effects and cost-effectiveness of a guideline-oriented primary healthcare hypertension management program in Beijing, China: results from a 1-year controlled trial (2013) Hypertens Res, 36 (4), pp. 313-321; Duan, H., Wang, Z., Ji, Y., Ma, L., Liu, F., Chi, M., Using goal-directed design to create a mobile health app to improve patient compliance with hypertension self-management: development and deployment (2020) JMIR mHealth uHealth, 8 (2); McManus, R.J., Mant, J., Bray, E.P., Holder, R., Jones, M.I., Greenfield, S., Telemonitoring and self-management in the control of hypertension (TASMINH2): a randomised controlled trial (2010) Lancet, 376 (9736), pp. 163-172; Dolan, E.,

Stanton, A., Thijs, L., Hinedi, K., Atkins, N., McClory, S., Superiority of ambulatory over clinic blood pressure measurement in predicting mortality: the Dublin outcome study (2005) Hypertens (Dallas, Tex 1979), 46 (1), pp. 156-161; Niiranen, T.J., Hänninen, M.-R., Johansson, J., Reunanen, A., Jula, A.M., Home-measured blood pressure is a stronger predictor of cardiovascular risk than office blood pressure: the Finn-Home study (2010) Hypertens (Dallas, Tex 1979), 55 (6), pp. 1346-1351; O’Shea, P.M., Griffin, T.P., Fitzgibbon, M., Hypertension: The role of biochemistry in the diagnosis and management (2017) Clin Chim Acta, 465, pp. 131-143. , –,. doi:, https://doi.org/; Craig, C.L., Marshall, A.L., Sjöström, M., Bauman, A.E., Booth, M.L., Ainsworth, B.E., International physical activity questionnaire: 12-country reliability and validity (2003) Med Sci Sports Exerc, 35 (8), pp. 1381-1395; McLean, R.M., Measuring population sodium intake: a review of methods (2014) Nutrients, 6 (11), pp. 4651-4662; Aburto, N.J., Hanson, S., Gutierrez, H., Hooper, L., Elliott, P., Cappuccio, F.P., Effect of increased potassium intake on cardiovascular risk factors and disease: systematic review and meta-analyses (2013) BMJ, 346 (apr03 3), p. f1378; Kim, M.T., Hill, M.N., Bone, L.R., Levine, D.M., Development and testing of the Hill-Bone Compliance to High Blood Pressure Therapy Scale (2000) Prog Cardiovasc Nurs, 15 (3), pp. 90-96; Erkoc, S.B., Isikli, B., Metintas, S., Kalyoncu, C., Hypertension Knowledge-Level Scale (HK-LS): a study on development, validity and reliability (2012) Int J Environ Res Public Health, 9 (3), pp. 1018-1029; Xu, L., Fang, W.-Y., Zhu, F., Zhang, H.-G., Liu, K., A coordinated PCP-Cardiologist Telemedicine Model (PCTM) in China’s community hypertension care: study protocol for a randomized controlled trial (2017) Trials, 18 (1), p. 236; Tu, Q., Xiao, L.D., Ullah, S., Fuller, J., Du, H., Hypertension management for community-dwelling older people with diabetes in Nanchang, China: study protocol for a cluster randomized controlled trial (2018) Trials, 19 (1), p. 385; Yin, M., Augustin, B., Fu, Z., Yan, M., Fu, A., Yin, P., Geographic distributions in hypertension diagnosis, measurement, prevalence, awareness, treatment and control rates among middle-aged and older adults in China (2016) Sci Rep, 6 (1), p. 37020},
correspondence_address1={Deng, N.; College of Biomedical Engineering and Instrument Science, China; email: zju.dengning@gmail.com},
publisher={BioMed Central Ltd},
issn={17456215},
pubmed_id={33482896},
language={English},
abbrev_source_title={Trials},
document_type={Article},
source={Scopus},
}

@ARTICLE{Lambert2021,
author={Lambert, S.D. and Duncan, L.R. and Ellis, J. and Robinson, J.W. and Sears, C. and Culos-Reed, N. and Matthew, A. and De Raad, M. and Schaffler, J.L. and Mina, D.S. and Saha-Chaudhuri, P. and McTaggart-Cowan, H. and Peacock, S.},
title={A study protocol for a multicenter randomized pilot trial of a dyadic, tailored, web-based, psychosocial, and physical activity self-management program (TEMPO) for men with prostate cancer and their caregivers},
journal={Pilot and Feasibility Studies},
year={2021},
volume={7},
number={1},

doi={10.1186/s40814-021-00791-6},
art_number={78},
note={cited By 0},
url={https://www.scopus.com/inward/record.uri?eid=2-s2.0-85103204816&doi=10.1186%2fs40814-021-00791-6&partnerID=40&md5=edfcba9c37d98ccea15bbdf508f16c1d},
affiliation={Ingram School of Nursing, McGill University, Montreal, QC, Canada; St. Mary’s Research Centre, Montreal, QC, Canada; Department of Kinesiology and Physical Education, McGill University, Montreal, QC, Canada; Department of Psychiatry, University of Toronto, Toronto, Canada; Psychosocial Care in Trauma, Sunnybrook Health Sciences Centre, Toronto, Canada; Department of Psychology, University of Calgary, Calgary, Canada; Department of Oncology, University of Calgary, Calgary, Canada; University of Calgary, Calgary, Canada; Health and Exercise Psychology, Faculty of Kinesiology, University of Calgary, Calgary, Canada; Department of Oncology, Cumming School of Medicine, University of Calgary, Calgary, Canada; Psychosocial Resources, Tom Baker Cancer Centre, Cancer Care, Alberta Health Services, Edmonton, Canada; Faculty of Medicine, Department of Surgery, University of Toronto, Toronto, Canada; Department of Psychiatry, University of Toronto, Toronto, Canada; Department of Surgery, Princess Margaret Cancer Centre, Toronto, Canada; Faculty of Kinesiology and Physical Education, University of Toronto, Toronto, Canada; Toronto General Hospital, Toronto, Canada; Department of Mathematics and Statistics, University of Vermont, Burlington, VT, United States; Canadian Centre for Applied Research in Cancer Control, Toronto, Canada; Cancer Control Research, BC Cancer, Vancouver, Canada; Faculty of Health Sciences, Simon Fraser University, Burnaby, Canada},
abstract={Background: Prostate cancer predisposes patients and caregivers to a wide range of complex physical and psychosocial challenges, and interventions must incorporate a wide range of self-management strategies to help patients and their caregivers effectively cope with cancer challenges. To palliate this need, our team recently developed and evaluated the initial acceptability of a dyadic, Tailored, wEb-based, psychosocial, and physical activity self-Management PrOgram (TEMPO). TEMPO is a 10-week, interactive, web-based intervention consisting of five modules designed to help dyads manage their physical and psychosocial needs. It aims to teach dyads new self-management strategies and encourages them to increase their physical activity (PA) levels, mainly through walking and strength-based exercises. Initial acceptability evaluation of TEMPO revealed high user satisfaction, in addition to having a number of potential benefits for participants. After integrating suggested changes to TEMPO, the proposed pilot study aims to further test the acceptability and feasibility of TEMPO. Methods: This study is a multicenter, stratified, parallel, two-group, pilot randomized control trial (RCT), where patient–caregiver dyads are randomized (stratified by anxiety level) to receive (a) TEMPO or (b) usual care. Participants (n goal = 40) are recruited across Canada at participating cancer centers and through self-referral (e.g., online recruitment). Patient inclusion criteria are (a) having received prostate cancer treatment within the past 2 years or scheduled to receive treatment, (b) identified a primary caregiver willing to participate in the study, and (c) has access to the Internet. Eligible caregivers are those identified by the patient as his primary source of support. Dyads complete a baseline questionnaire (T1) and another one 3 months later (T2) assessing various aspects of physical and emotional functioning (e.g., the Medical Outcomes Study (MOS) 12-item Short Form Health Survey (SF-12), the Hospital Anxiety and Depression Scale (HADS), and the Perceived Stress Scale (PSS)), self-management behaviors (e.g., the Health Education Impact Questionnaire (heiQ)), physical activity (the

International Physical Activity Questionnaires (IPAQ) and the Multidimensional Self-efficacy for Exercise Scale (MSES)), and dyadic coping (the Dyadic Coping Inventory (DCI)). Dyads that used TEMPO are also asked to participate in a semi-structured exit interview exploring their overall experience with the program. Discussion: This feasibility analysis will begin to develop the knowledge base on TEMPO’s value for men with prostate cancer and their caregivers to inform a larger trial. Trial registration: NCT04304196. © 2021, The Author(s).},
author_keywords={Cancer rehabilitation; Cancer survivorship; Caregivers; Dyadic intervention; Prostate cancer; Self-management},
references={Chambers, S.K., Hyde, M.K., Smith, D.P., Hughes, S., Yuill, S., Egger, S., O'Connell, D.L., Wittert, G., New challenges in Psycho-Oncology Research III: a systematic review of psychological interventions for prostate cancer survivors and their partners: clinical and research implications (2017) Psycho Oncol, 26 (7), p. 873; Girgis, A., Lambert, S., Johnson, C., Waller, A., Currow, D., Physical, psychosocial, relationship, and economic burden of caring for people with cancer: a review (2013) Journal of Oncology Practice, 9 (4), pp. 197-202. , PID: 23942921; Lambert, S.D., Harrison, J.D., Smith, E., Bonevski, B., Carey, M., Lawsin, C., Paul, C., Girgis, A., The unmet needs of partners and caregivers of adults diagnosed with cancer: a systematic review (2012) BMJ supportive & palliative care, 2 (3), pp. 224-230; Coumoundouros, C., Ould Brahim, L., Lambert, S.D., McCusker, J., The direct and indirect financial costs of informal cancer care: a scoping review (2019) Health Soc Care Commun, 27 (5), pp. e622-e636; Lambert, S., Girgis, A., Descallar, J., Levesque, J.V., Jones, B., Trajectories of mental and physical functioning among spouse caregivers of cancer survivors over the first five years following the diagnosis (2017) Patient education and counseling, 100 (6), pp. 1213-1221. , PID: 28089132; Sánchez, E.S., Baena, A.C.G., Cáliz, C.G., Paredes, F.C., Calvo, J.L.M., Fernández, J.C., Prevalence of anxiety and depression in prostate cancer patients and their spouses: An unaddressed reality (2020) Prostate Cancer, 2020 (4393), p. 175; Lambert, S.D., Caregivers’ psychological well-being over the first five years after the patients’ diagnosis: results from Australia’s partners and caregivers longitudinal well-being study (2016) European Oncology Nurses Society Annual Conference: Balancing Health Care Needs: October 17 – 18 2016; Dublin, Ireland; Fu, F., Zhao, H., Tong, F., Chi, I., A systematic review of psychosocial interventions to cancer caregivers (2017) Front Psychol, 8, p. 834. , PID: 28596746; Gabriel, I., Creedy, D., Coyne, E., A systematic review of psychosocial interventions to improve quality of life of people with cancer and their family caregivers (2020) Nursing Open, 7 (5), pp. 1299-1312. , PID: 32802350; Northouse, L.L., Katapodi, M.C., Song, L., Zhang, L., Mood, D.W., Interventions with family caregivers of cancer patients: meta-analysis of randomized trials (2010) CA: a cancer journal for clinicians, 60 (5), pp. 317-339; Lambert, S.D., Girgis, A., Turner, J., Regan, T., Candler, H., Britton, B., Chambers, S., Lawsin, C., Kayser K: “You need something like this to give you guidelines on what to do”: patients' and partners' use and perceptions of a self-directed coping skills training resource (2013) Support Care Cancer, 21 (12), pp. 3451-3460. , PID: 23955024; Swartz, J.J., Keir, S.T., Program preferences to reduce stress in caregivers of patients with brain tumors (2007) Clinical journal of oncology nursing, 11 (5), p. 723. , PID: 17962180; Lambert, S.D., Duncan, L.R., Kapellas, S., Bruson, A.-M., Myrand, M., Santa Mina, D., Culos-Reed, N., Lambrou, A., A descriptive systematic review of physical activity interventions for caregivers: effects on caregivers’ and care recipients’ psychosocial outcomes, physical activity levels, and physical health (2016) Annals of Behavioral Medicine, 50 (6), pp.

907-919. , PID: 27439530; Cuthbert, C.A., King-Shier, K., Ruether, D., Tapp, D.M., Culos-Reed, S.N., What is the state of the science on physical activity interventions for family caregivers? A systematic review and RE-AIM evaluation (2017) Journal of Physical Activity and Health, 14 (7), pp. 578-595. , PID: 28290737; Badger, T., Segrin, C., Dorros, S.M., Meek, P., Lopez, A.M., Depression and anxiety in women with breast cancer and their partners (2007) Nursing research, 56 (1), pp. 44-53. , PID: 17179873; Martin, A.C., Keats, M.R., The impact of yoga on quality of life and psychological distress in caregivers for patients with cancer (2014) Oncol Nurs Forum, 2014, pp. 257-264; Milbury, K., Mallaiah, S., Lopez, G., Liao, Z., Yang, C., Carmack, C., Chaoul, A., Cohen, L., Vivekananda yoga program for patients with advanced lung cancer and their family caregivers (2015) Integrative cancer therapies, 14 (5), pp. 446-451. , COI: 1:CAS:528:DC%2BC28XnsFCnsbY%3D, PID: 25917816; Nezu, A.M., Nezu, C.M., Felgoise, S.H., McClure, K.S., Houts, P.S., Project Genesis: assessing the efficacy of problem-solving therapy for distressed adult cancer patients (2003) J Consult Clin Psychol, 71 (6), p. 1036. , PID: 14622079; Scott, J.L., Halford, W.K., Ward, B.G., United we stand? The effects of a couple-coping intervention on adjustment to early stage breast or gynecological cancer (2004) Journal of consulting and clinical psychology, 72 (6), p. 1122. , PID: 15612858; Cuthbert, C.A., King-Shier, K.M., Ruether, J.D., Tapp, D.M., Wytsma-Fisher, K., Fung, T.S., Culos-Reed, S.N., The effects of exercise on physical and psychological outcomes in cancer caregivers: results from the RECHARGE randomized controlled trial (2018) Annals of Behavioral Medicine, 52 (8), pp. 645-661. , PID: 30010704; Beatty, L.J., Koczwara, B., Rice, J., Wade, T.D., A randomised controlled trial to evaluate the effects of a self-help workbook intervention on distress, coping and quality of life after breast cancer diagnosis (2010) Med J Australia, 193, pp. S68-S73. , PID: 21542450; Jacobsen, P.B., Phillips, K.M., Jim, H.S., Small, B.J., Faul, L.A., Meade, C.D., Thompson, L., Fishman, M., Effects of self-directed stress management training and home-based exercise on quality of life in cancer patients receiving chemotherapy: a randomized controlled trial (2013) Psycho Oncol, 22 (6), pp. 1229-1235; Northouse, L., Schafenacker, A., Barr, K.L., Katapodi, M., Yoon, H., Brittain, K., Song, L., An, L., A tailored web-based psycho-educational intervention for cancer patients and their family caregivers (2014) Cancer Nurs, 37 (5), p. 321. , PID: 4164300; Badr, H., Lipnick, D., Diefenbach, M.A., Posner, M., Kotz, T., Miles, B., Genden, E., Development and usability testing of a web-based self-management intervention for oral cancer survivors and their family caregivers (2016) European journal of cancer care, 25 (5), pp. 806-821. , COI: 1:STN:280:DC%2BC28zmtVWhtg%3D%3D, PID: 26507369; Sotirova, M., McCaughan, E., Ramsey, L., Flannagan, C., Kerr, D.P., O'connor, S., Blackburn, N.E., Wilson, I., Acceptability of online exercise-based interventions after breast cancer surgery: Systematic review and narrative synthesis (2020) J Cancer Survivor; Lambert, S.D., Duncan, L.R., Ellis, J., Schaffler, J.L., Loban, E., Robinson, J.W., Culos-Reed, N., Santa Mina, D., (2020) Acceptability and usefulness of a dyadic, tailored, web-based, psychosocial and physical activity self-management programme (TEMPO): a qualitative study. Under Review in Journal of Clinical Medicine (Cancer Rehabilitation and Survivorship); Sidani, S., Braden, C.J., (2011) Design, evaluation, and translation of nursing interventions, , UK: Wiley-Blackwell, West Sussex; Thabane, L., Hopewell, S., Lancaster, G., Bond, C., Coleman, C., Campbell, M.J., Eldridge, S.M., Methods and processes for development of a CONSORT extension for reporting pilot randomized controlled trials (2016) Pilot Feasibility Stud, 2, p. 25. , PID: 27965844; Bennett, D.A., How can I deal with missing data in my study? (2001) Australian and New Zealand Journal of Public

Health, 25 (5), pp. 464-469. , COI: 1:STN:280:DC%2BD3MnjsFCjtQ%3D%3D, PID: 11688629; Regan, T., Lambert, S., Kelly, B., Uptake and attrition in couple-based interventions for cancer: perspectives from the literature (2013) Psycho Oncol, 22 (12), pp. 2639-2647; Harden, J., Falahee, M., Bickes, J., Schafenacker, A., Walker, J., Mood, D., Northouse, L.L., Factors associated with prostate cancer patients' and their spouses' satisfaction with a family-based intervention (2009) Cancer Nurs, 32 (6), pp. 482-492. , PID: 19816159; Cohen, J.H., (1988) Statistical power analysis for the behavioral sciences, , 2, Lawrence Earlbaum Associates, Hillsdale; Moher, D., Hopewell, S., Schulz, K.F., Montori, V., Gotzsche, P.C., Devereaux, P.J., Elbourne, D., Altman, D.G., CONSORT 2010 explanation and elaboration: updated guidelines for reporting parallel group randomised trials (2010) BMJ, 340, p. c869. , PID: 20332511; Chan, A.-W., Tetzlaff, J.M., Gøtzsche, P.C., Altman, D.G., Mann, H., Berlin, J.A., Dickersin, K., Parulekar, W.R., SPIRIT 2013 explanation and elaboration: guidance for protocols of clinical trials (2013) Bmj, 346; Craig, P., Dieppe, P., Macintyre, S., Michie, S., Nazareth, I., Petticrew, M., Developing and evaluating complex interventions: the new Medical Research Council guidance (2013) Int J Nurs Stud, 50 (5), pp. 587-592. , PID: 23159157; Lambert, S.D., Jones, B., Girgis, A., Lecathelinais, C., Distressed partners and caregivers do not recover easily: adjustment trajectories among partners and caregivers of cancer survivors (2012) Ann Behav Med, 44 (2), pp. 225-235. , PID: 22740365; Northouse, L.L., Mood, D.W., Schafenacker, A., Kalemkerian, G., Zalupski, M., LoRusso, P., Hayes, D.F., Fendrick, A.M., Randomized clinical trial of a brief and extensive dyadic intervention for advanced cancer patients and their family caregivers (2013) Psychooncology, 22 (3), pp. 555-563. , PID: 22290823; Northouse, L.L., Mood, D.W., Schafenacker, A., Montie, J.E., Sandler, H.M., Forman, J.D., Hussain, M., Kershaw, T., Randomized clinical trial of a family intervention for prostate cancer patients and their spouses (2007) Cancer, 110 (12), pp. 2809-2818. , PID: 17999405; Northouse, L., Walker, J., Schafenacker, A., Mood, D., Mellon, S., Galvin, E., Harden, J., Freeman-Gibb, L., A family-based program of care for women with recurrent breast cancer and their family members (2002) Oncol Nurs Forum, 29 (10), pp. 1411-1419. , PID: 12432412; Lambert, S.D., McElduff, P., Girgis, A., Levesque, J.V., Regan, T.W., Turner, J., Candler, H., Kayser, K., A pilot, multisite, randomized controlled trial of a self-directed coping skills training intervention for couples facing prostate cancer: accrual, retention, and data collection issues (2016) Support Care Cancer, 24 (2), pp. 711-722. , PID: 26184499; Bell, M.L., Whitehead, A.L., Julious, S.A., Guidance for using pilot studies to inform the design of intervention trials with continuous outcomes (2018) Clin Epidemiol, 10, pp. 153-157. , PID: 29403314; Zigmond, A.S., Snaith, R.P., The hospital anxiety and depression scale (1983) Acta Psychiatr Scand, 67 (6), pp. 361-370. , COI: 1:STN:280:DyaL3s3nvFWjug%3D%3D; Brush, B.L., Baiardi, J.M., Lapides, S., Moving toward synergy: lessons learned in developing and sustaining community-academic partnerships (2011) Progress in community health partnerships: research, education, and action, 5 (1), pp. 27-34; Hoffmann, T.C., Glasziou, P.P., Boutron, I., Milne, R., Perera, R., Moher, D., Altman, D.G., Johnston, M., Better reporting of interventions: template for intervention description and replication (TIDieR) checklist and guide (2014) BMJ: British Medical Journal, 348, p. g1687. , PID: 24609605; Lambert, S.D., Duncan, L.R., Ellis, J., Schaffler, J.L., Loban, E., Robinson, J.W., Culos-Reed, N., Santa Mina, D., Acceptability and usefulness of a dyadic, tailored, web-based, psychosocial and physical activity self-management program (TEMPO): a qualitative study (2020) J Clin Med, 9 (10), p. 3284; Courneya, K.S., Segal, R.J., Reid, R.D., Jones, L.W., Malone, S.C., Venner, P.M., Parliament, M.B., Wells, G.A., Three

independent factors predicted adherence in a randomized controlled trial of resistance exercise training among prostate cancer survivors (2004) Journal of clinical epidemiology, 57 (6), pp. 571-579. , PID: 15246125; Demark-Wahnefried, W., Clipp, E.C., Morey, M.C., Pieper, C.F., Sloane, R., Snyder, D.C., Cohen, H.J., Lifestyle intervention development study to improve physical function in older adults with cancer: outcomes from Project LEAD (2006) Journal of clinical oncology: official journal of the American Society of Clinical Oncology, 24 (21), p. 3465; Schmitz, K.H., Courneya, K.S., Matthews, C., Demark-Wahnefried, W., Galvão, D.A., Pinto, B.M., Irwin, M.L., Lucia, A., American College of Sports Medicine roundtable on exercise guidelines for cancer survivors (2010) Med Sci Sport Exercise, 42 (7), pp. 1409-1426; Fogg, B.J., Persuasive technology: using computers to change what we think and do (2002) Ubiquity, 2002 (December), p. 2; Lazarus, R., Folkman, S., (1984) Stress, appraisal, and coping, , Springer Publishing Company Inc, New York; Stanton, A.L., Danoff-Burg, S., Huggins, M., The first year after breast cancer diagnosis: hope and coping strategies as predictors of adjustment (2002) Psycho Oncol, 11 (2), pp. 93-102; Manne, S.L., Coping with cancer: findings of research and intervention studies (2007) Coping with Chronic Illness and Disability, pp. 191-293. , Martz E, Livneh H, (eds), Springer Science+Business Media, New York; Bodenmann, G., A systemic-transactional conceptualization of stress and coping in couples (1995) Swiss J Psychology, 54 (1), pp. 34-49; Regan, T., Lambert, S.D., Kelly, B., Falconier, M., Kissane, D., Levesque, J.V., Couples coping with cancer: exploration of theoretical frameworks from dyadic studies (2015) Psycho Oncol, 24 (12), pp. 1605-1617; Bandura, A., (1986) Social foundations of thought and action: a social cognitive theory, , Prentice-Hall, Englewood Cliffs, NJ; Page, S.J., Persch, A.C., Recruitment, retention, and blinding in clinical trials (2013) Am J Occup Ther, 67 (2), pp. 154-161. , PID: 23433269; Ware, J.E., Kosinski, M., Turner-Bowker, D.M., Gandeck, B., (2002) User's manual for the SF-12v2TM health survey with a supplement documenting SF-12R health survey In, , QualityMetric Incorporated, Lincoln; Kershaw, T., Mood, D., Newth, G., Ronis, D.L., Sanda, M.G., Vaishampayan, U., Northouse, L., Longitudinal analysis of a model to predict quality of life in prostate cancer patients and their spouses (2008) Ann Behav Med, 36 (2), pp. 117-128. , PID: 18830672; Mitchell, G., Girgis, A., Jiwa, M., Sibbritt, D., Burridge, L., A GP caregiver needs toolkit versus usual care in the management of the needs of caregivers of patients with advanced cancer: a randomized controlled trial (2010) Trials, 11 (1), p. 115. , PID: 21114863; Busija, L., Pausenberger, E., Haines, T.P., Haymes, S., Buchbinder, R., Osborne, R.H., Adult measures of general health and health-related quality of life: Medical Outcomes Study Short Form 36-Item (SF-36) and Short Form 12-Item (SF-12) Health Surveys, Nottingham Health Profile (NHP), Sickness Impact Profile (SIP), Medical Outcomes Study Short Form 6D (SF-6D), Health Utilities Index Mark 3 (HUI3), Quality of Well-Being Scale (QWB), and Assessment of Quality of Life (AQOL) (2011) Arthritis Care Res, 63 (S11), pp. S383-S412; Gandek, B., Ware, J.E., Aaronson, N.K., Apolone, G., Bjorner, J.B., Brazier, J.E., Bullinger, M., Prieto, L., Cross-validation of item selection and scoring for the SF-12 health survey in nine countries: results from the IQOLA Project (1998) J Clin Epidemiol, 51 (11), pp. 1171-1178. , COI: 1:STN:280:DyaK1M%2Fjt1Shtw%3D%3D; Bjelland, I., Dahl, A., Haug, T., Neckelmann, D., The validity of the Hospital Anxiety and Depression Scale: an updated literature review (2002) J Psychosomatic Res, 52 (2), pp. 69-77; Luckett, T., Butow, P.N., King, M.T., Oguchi, M., Heading, G., Hackl, N.A., Rankin, N., Price, M.A., A review and recommendations for optimal outcome measures of anxiety, depression and general distress in studies evaluating psychosocial interventions for English-speaking adults with

heterogeneous cancer diagnoses (2010) Supp Care Cancer, 18, pp. 1241-1262; Coyne, J.C., van Sonderen, E., No further research needed: abandoning the Hospital and Anxiety Depression Scale (HADS) (2012) Journal of psychosomatic research, 72 (3), pp. 173-174. , PID: 22325694; Lambert, S., Pallant, J.F., Boyes, A., King, M., Britton, B., Girgis, A., A Rasch analysis of the Hospital Anxiety and Depression Scale (HADS) among cancer survivors (2013) Psychol Assess, 25 (2), pp. 379-390. , PID: 23356678; Lambert, S.D., Pallant, J.F., Girgis, A., Rasch analysis of the Hospital Anxiety and Depression Scale among caregivers of cancer survivors: implications for its use in psycho-oncology (2011) Psycho-Oncology, 20 (9), pp. 919-925. , PID: 20669337; Roberge, P., Doré, I., Menear, M., Chartrand, É., Ciampi, A., Duhoux, A., Fournier, L., A psychometric evaluation of the French Canadian version of the Hospital Anxiety and Depression Scale in a large primary care population (2013) Journal of affective disorders, 147 (1-3), pp. 171-179. , PID: 23218249; Cohen, S., Kamarck, T., Mermelstein, R., A global measure of perceived stress (1983) Journal of Health and Social Behavior, (4), pp. 385-396; Lee, E.-H., Review of the psychometric evidence of the perceived stress scale (2012) Asian nursing research, 6 (4), pp. 121-127. , PID: 25031113; Lesage, F.-X., Berjot, S., Deschamps, F., Psychometric properties of the French versions of the Perceived Stress Scale (2012) International journal of occupational medicine and environmental health, 25 (2), pp. 178-184. , PID: 22528542; Osborne, R., Elsworth, G., Whitfield, K., The Health Education Impact Questionnaire (heiQ): an outcomes and evaluation measure for patient education and self-management interventions for people with chronic conditions (2007) Pat Educ Counsel, 66 (2), pp. 192-201; Bélanger, A., Hudon, C., Fortin, M., Amirall, J., Bouhali, T., Chouinard, M.-C., Validation of a French-language version of the health education impact Questionnaire (heiQ) among chronic disease patients seen in primary care: a cross-sectional study (2015) Health and quality of life outcomes, 13 (1), p. 64. , PID: 26306793; Maunsell, E., Lauzier, S., Brunet, J., Pelletier, S., Osborne, R.H., Campbell, H.S., Health-related empowerment in cancer: validity of scales from the Health Education Impact Questionnaire (2014) Cancer, 120 (20), pp. 3228-3236. , PID: 24988944; Osborne, R.H., Batterham, R.W., Elsworth, G.R., Hawkins, M., Buchbinder, R., The grounded psychometric development and initial validation of the Health Literacy Questionnaire (HLQ) (2013) BMC Public Health, 13 (1), p. 658. , PID: 23855504; Debussche, X., Lenclume, V., Balcou-Debussche, M., Alakian, D., Sokolowsky, C., Ballet, D., Elsworth, G.R., Huiart, L., Characterisation of health literacy strengths and weaknesses among people at metabolic and cardiovascular risk: validity testing of the Health Literacy Questionnaire (2018) SAGE Open Med, 6. , PID: 30319778; Craig, C., Marshall, A., Sjostrom, M., Bauman, A., Booth, M., Ainsworth, B., Yngre, A., Oja, P., International physical activity questionnaire: 12-country reliability and validity (2003) Med Sci Sports Exerc, 35, pp. 1381-1395; Ajzen, I., (2002) Constructing a TPB questionnaire: conceptual and methodological considerations; Rodgers, W., Wilson, P., Hall, C., Fraser, S., Murray, T., Evidence for a multidimensional self-efficacy for exercise scale (2008) Res Q Exerc Sport, 79 (2), pp. 222-234. , COI: 1:STN:280:DC%2BD1cvosVygsA%3D%3D, PID: 18664046; Bodenmann, G., (2008) Dyadisches Coping Inventar: Testmanual [Dyadic Coping Inventory: Test manual], , Huber, Bern; Feldman, B.N., Broussard, C.A., The influence of relational factors on men's adjustment to their partners' newly-diagnosed breast cancer (2005) J Psychosocial Oncol, 23 (2-3), pp. 23-43; Bodenmann, G., Arista, L.J., Walsh, K.J., Randall, A.K., Dyadic Coping Inventory (2018) Encyclopedia of couple and family therapy, pp. 1-5. , Springer, Cham; Spanier, G.B., Measuring dyadic adjustment: new scales for assessing the quality of marriage and similar

dyads (1976) J Marriage Fam, 38 (1), pp. 15-28; Manne, S.L., Norton, T.R., Ostroff, J.S., Winkel, G., Fox, K., Grana, G., Protective buffering and psychological distress among couples coping with breast cancer: the moderating role of relationship satisfaction (2007) Journal of Family Psychology, 21 (3), pp. 380-388. , PID: 17874923; Lambert, S.D., Girgis, A., McElduff, P., Turner, J., Levesque, J.V., Kayser, K., Mihalopoulos, C., Barker, D., A parallel-group, randomised controlled trial of a multimedia, self-directed, coping skills training intervention for patients with cancer and their partners: design and rationale (2013) BMJ Open, 3 (7), pp. 2013-003337; Abu-Dalbouh, H., A questionnaire approach based on the Technology Acceptance Model for mobile tracking on patient progress applications (2013) J Comput Sci, 9, pp. 763-770; Brooke, J., SUS: a "quick and dirty" usability scale (1996) Usability Evaluation in Industry, , Jordan PW, Thomas B, Weerdmeester BA, AL MC, (eds), Taylor and Francis, London; Puhan, M., Frey, M., Buchi, S., Schunemann, H., The minimal important difference of the hospital anxiety and depression scale in patients with chronic obstructive pulmonary disease (2008) Health Qual Life Outcomes, 6 (46), pp. 1-6; Díaz-Arribas, M.J., Fernández-Serrano, M., Royuela, A., Kovacs, F.M., Gallego-Izquierdo, T., Ramos-Sánchez, M., Llorca-Palomera, R., Martín-Pariente, O.S., Minimal clinically important difference in quality of life for patients with low back pain (2017) Spine (Phila Pa 1976), 42 (24), pp. 1908-1916; Hanmer, J., Predicting an SF-6D preference-based score using MCS and PCS scores from the SF-12 or SF-36 (2009) Value Health, 12, pp. 958-966. , PID: 19490549; Braun, V., Clarke, V., Using thematic analysis in psychology (2006) Qualitative Research in Psychology, 3 (2), pp. 77-101; Streubert Speziale, H., Carpenter, D., (2003) Qualitative research in Nursing, 3rd Ed. edn, , Lippincott Williams & Wilkins, Philadelphia; Beatty, L., Lambert, S., A systematic review of internet-based self-help therapeutic interventions to improve distress and disease-control among adults with chronic health conditions (2013) Clinical Psychology Review, 33 (4), pp. 609-622. , PID: 23603521; Council, M.R., (2000) A framework for development and evaluation of RCTs for complex interventions to improve health, , MRC, London; Craig, P., Dieppe, P., Macintyre, S., Michie, S., Nazareth, I., Petticrew, M., Developing and evaluating complex interventions: The new Medical Research Council guidance (2008) BMJ, 337},
correspondence_address1={Lambert, S.D.; St. Mary’s Research CentreCanada; email: sylvie.lambert@mcgill.ca},
publisher={BioMed Central Ltd},
issn={20555784},
language={English},
abbrev_source_title={Pilot Feasibility Stud.},
document_type={Article},
source={Scopus},
}

@ARTICLE{Langer2021,
author={Langer, A. and Gassner, L. and Flotz, A. and Hasenauer, S. and Gruber, J. and Wizany, L. and Pokan, R. and Maetzler, W. and Zach, H.},
title={How COVID-19 will boost remote exercise-based treatment in Parkinson’s disease: a narrative review},
journal={npj Parkinson's Disease},
year={2021},
volume={7},
number={1},
doi={10.1038/s41531-021-00160-3},

art_number={25},
note={cited By 0},
url={https://www.scopus.com/inward/record.uri?eid=2-s2.0-85104369470&doi=10.1038%2fs41531-021-00160-3&partnerID=40&md5=4ab445b76bdab182abd0bfb69dc9d65b},
affiliation={Department of Neurology, Medical University of Vienna, Vienna, Austria; Department of Sport Physiology, Institute of Sports Sciences, University of Vienna, Vienna, Austria; School of Engineering, RMIT University, Melbourne, VIC, Australia; Department of Neurology, University Medical Centre Schleswig-Holstein, Kiel University, Kiel, Germany},
abstract={The lack of physical exercise during the COVID-19 pandemic-related quarantine measures is challenging, especially for patients with Parkinson’s disease (PD). Without regular exercise not only patients, but also nursing staff and physicians soon noticed a deterioration of motor and non-motor symptoms. Reduced functional mobility, increased falls, increased frailty, and decreased quality of life were identified as consequences of increased sedentary behavior. This work overviews the current literature on problems of supplying conventional physiotherapy and the potential of telerehabilitation, allied health services, and patient-initiated exercise for PD patients during the COVID-19 period. We discuss recent studies on approaches that can improve remote provision of exercise to patients, including telerehabilitation, motivational tools, apps, exergaming, and virtual reality (VR) exercise. Additionally, we provide a case report about a 69-year-old PD patient who took part in a 12-week guided climbing course for PD patients prior to the pandemic and found a solution to continue her climbing training independently with an outdoor rope ladder. This case can serve as a best practice example for non-instructed, creative, and patient-initiated exercise in the domestic environment in difficult times, as are the current. Overall, many recent studies on telemedicine, telerehabilitation, and patient-initiated exercises have been published, giving rise to optimism that facilitating remote exercise can help PD patients maintain physical mobility and emotional well-being, even in phases such as the COVID-19 pandemic. The pandemic itself may even boost the need to establish comprehensive and easy-to-do telerehabilitation programs. © 2021, The Author(s).},
keywords={alpha synuclein; levodopa; neurotrophic factor; rasagiline; rotigotine, aged; case report; climbing; clinical article; coronavirus disease 2019; exercise; female; health care personnel; health service; human; lockdown; medication compliance; mobile application; mortality; motor dysfunction; muscle strength; neck pain; pandemic; Parkinson disease; patient satisfaction; physical activity; physical mobility; physiotherapy; quality of life; Review; sedentary lifestyle; sleep quality; social distancing; stretching exercise; telecommunication; telemedicine; telerehabilitation; training; virtual reality; wellbeing; yoga},
references={Antonini, A., Leta, V., Teo, J., Chaudhuri, K.R., Outcome of Parkinson’s disease patients affected by COVID-19 (2020) Mov Disord, 35, pp. 905-908; Ferini-Strambi, L., Salsone, M., COVID-19 and neurological disorders: Are neurodegenerative or neuroimmunological diseases more vulnerable? (2020) J Neurol, pp. 1-11. , https://doi.org/10.1007/s00415-020-10070-8, Advance online publication; Bhidayasiri, R., Virameteekul, S., Kim, J.M., Pal, P.K., Chung, S.J., COVID-19: an early review of its global impact and considerations for Parkinson’s disease patient care (2020) J. Mov. Disord., 13, pp. 105-114. , PID: 32344993; Chaudhuri, K.R., COVID_19 and Parkinson’s disease (2020) Kinetic, 2, pp. 4-5; Sulzer, D., COVID-19 and possible links with Parkinson’s disease and parkinsonism: from bench to bedside (2020) NPJ Parkinsons Dis., 6, p. 18. , COI: 1:CAS:528:DC%2BB3cXhs1Kltb%2FE, PID: 32885037; van de Venis, L.,

COVID-19 reveals influence of physical activity on symptom severity in hereditary spastic paraplegia (2020) J. Neurol., 267 (1-3); Boldrini, P., Living with a disability during the pandemic. “Instant paper from the field” on rehabilitation answers to the COVID-19 emergency (2020) Eur. J. Phys. Rehabil. Med., 56, pp. 331-334. , PID: 32406226; Manto, M., Management of patients with cerebellar ataxia during the COVID-19 pandemic: current concerns and future implications (2020) Cerebellum, 19, pp. 562-568. , COI: 1:CAS:528:DC%2BB3cXptlGqsb0%3D, PID: 32405955; Karri, J., Seymour, M.L., Verduzco-Gutierrez, M., Jayaram, P., Point-of-care procedures in physiatry: practice considerations during the Covid-19 pandemic (2020) Am. J. Phys. Med. Rehabil., 99, pp. 567-570. , &; Carda, S., The role of physical and rehabilitation medicine in the COVID-19 pandemic: the clinician’s view (2020) Ann. Phys. Rehabil. Med., 63, pp. 554-556; Helmich, R.C., Bloem, B.R., The impact of the COVID-19 pandemic on Parkinson’s disease: hidden sorrows and emerging opportunities (2020) J. Parkinsons Dis., 10, pp. 351-354. , COI: 1:CAS:528:DC%2BB3cXmvFOntrk%3D, PID: 32250324; Stoessl, A.J., Bhatia, K.P., Merello, M., Movement disorders in the World of COVID-19 (2020) Mov. Disord., 35, pp. 709-710. , COI: 1:CAS:528:DC%2BB3cXps1Oisrw%3D, PID: 32250468; Prasad, S., Parkinson’s disease and COVID-19: Perceptions and implications in patients and caregivers (2020) Mov Disord, 35, pp. 912-914; Uhrbrand, A., Stenager, E., Pedersen, M.S., Dalgas, U., Parkinson’s disease and intensive exercise therapy–a systematic review and meta-analysis of randomized controlled trials (2015) J. Neurol. Sci., 353, pp. 9-19. , PID: 25936252; Shen, X., Wong-Yu, I.S., Mak, M.K., Effects of exercise on falls, balance, and gait ability in Parkinson’s disease: a meta-analysis (2016) Neurorehabil. Neural. Repair., 30, pp. 512-527. , PID: 26493731; Mak, M.K., Wong-Yu, I.S., Shen, X., Chung, C.L., Long-term effects of exercise and physical therapy in people with Parkinson disease (2017) Nat. Rev. Neurol., 13, pp. 689-703. , PID: 29027544; Tomlinson, C.L., Physiotherapy for Parkinson’s disease: a comparison of techniques (2014) Cochrane Database Syst. Rev., 2014, p. Cd002815. , PID: 7120367; Cusso, M.E., Donald, K.J., Khoo, T.K., The impact of physical activity on non-motor symptoms in Parkinson’s disease: a systematic review (2016) Front. Med., 3, p. 35; Klein, P.J., Tai Chi Chuan in the management of Parkinson’s disease and Alzheimer’s disease (2008) Med. Sport Sci., 52, pp. 173-181. , PID: 18487897; Song, R., The impact of Tai Chi and Qigong mind-body exercises on motor and non-motor function and quality of life in Parkinson’s disease: a systematic review and meta-analysis (2017) Parkinsonism Relat. Disord., 41, pp. 3-13. , COI: 1:STN:280:DC%2BC1cnntFWisQ%3D%3D, PID: 28602515; Šumec, R., Filip, P., Sheardová, K., Bareš, M., Psychological benefits of nonpharmacological methods aimed for improving balance in Parkinson’s disease: a systematic review (2015) Behav. Neurol., 2015, p. 620674. , PID: 26236107; Chen, K., Effect of exercise on quality of life in Parkinson’s disease: a systematic review and meta-analysis (2020) Parkinsons Dis., 2020, p. 3257623. , PID: 32695306; Crotty, G.F., Schwarzschild, M.A., Chasing protection in Parkinson’s disease: does exercise reduce risk and progression? (2020) Front. Aging Neurosci., 12, p. 186. , PID: 32636740; Petzinger, G.M., Exercise-enhanced neuroplasticity targeting motor and cognitive circuitry in Parkinson’s disease (2013) Lancet Neurol., 12, pp. 716-726. , PID: 23769598; Schenkman, M., Effect of high-intensity treadmill exercise on motor symptoms in patients with De Novo Parkinson disease: a phase 2 randomized clinical trial (2018) JAMA Neurol., 75, pp. 219-226. , PID: 29228079; Francardo, V., Schmitz, Y., Sulzer, D., Cenci, M.A., Neuroprotection and neurorestoration as experimental therapeutics for Parkinson’s disease (2017) Exp. Neurol., 298, pp. 137-147. , COI: 1:CAS:528:DC%2BC2sXhs1Ciu7jF, PID: 28988910; O’Callaghan, A., Comparing the

influence of exercise intensity on brain-derived neurotrophic factor serum levels in people with Parkinson’s disease: a pilot study (2020) Aging Clin. Exp. Res., 32, pp. 1731-1738. , PID: 31606860; Ellis, T., Rochester, L., Mobilizing Parkinson’s disease: the future of exercise (2018) J. Parkinsons Dis., 8, pp. S95-S100. , PID: 30584167; Hirsch, M.A., Iyer, S.S., Sanjak, M., Exercise-induced neuroplasticity in human Parkinson’s disease: what is the evidence telling us? (2016) Parkinsonism Relat. Disord., 22, pp. S78-S81. , PID: 26439945; Radder, D.L.M., Physiotherapy in Parkinson’s Disease: A Meta-Analysis of Present Treatment Modalities (2020) Neurorehabil Neural Repair., 34, pp. 871-880. , PID: 32917125; Ellis, T.D., Dibble, L.E., Peterson, D.S., Moving Beyond Effectiveness (2019) J. Neurol. Phys. Ther., 43, pp. 1-2. , PID: 30531380; Abbruzzese, G., Marchese, R., Avanzino, L., Pelosin, E., Rehabilitation for Parkinson’s disease: Current outlook and future challenges (2016) Parkinsonism Relat. Disord., 22, pp. S60-S64. , PID: 26360239; Carvalho, A., Comparison of strength training, aerobic training, and additional physical therapy as supplementary treatments for Parkinson’s disease: pilot study (2015) Clin. Inter. Aging, 10, pp. 183-191; Rawson, K.S., Exercise and Parkinson disease: comparing tango, treadmill, and stretching (2019) J. Neurol. Phys. Ther., 43, pp. 26-32. , PID: 30531383; McNeely, M.E., Duncan, R.P., Earhart, G.M., A comparison of dance interventions in people with Parkinson disease and older adults (2015) Maturitas, 81, pp. 10-16. , COI: 1:STN:280:DC%2BC2MnjvFWruw%3D%3D, PID: 25771040; Choi, H.Y., Exercise therapies for Parkinson’s disease: a systematic review and meta-analysis (2020) Parkinsons Dis., 2020, p. 2565320. , PID: 32963753; Hall, M.E., Church, F.C., Exercise for older adults improves the quality of life in Parkinson’s disease and potentially enhances the immune response to COVID-19 (2020) Brain Sci, 10, p. 612. , &; Zach, H., Dirkx, M.F., Pasman, J.W., Bloem, B.R., Helmich, R.C., Cognitive stress reduces the effect of levodopa on Parkinson’s resting tremor (2017) CNS Neurosci. Ther, 23, pp. 209-215; Subramanian, I., Farahnik, J., Mischley, L.K., Synergy of pandemics-social isolation is associated with worsened Parkinson severity and quality of life (2020) npj Parkinsons Dis., 6, p. 28. , COI: 1:CAS:528:DC%2BB3cXitVKhu7rL, PID: 33083522; Prasad, S., Parkinson’s disease and COVID-19: perceptions and implications in patients and caregivers (2020) Mov. Disord., 35, pp. 912-914. , COI: 1:CAS:528:DC%2BB3cXht1amu7zI, PID: 32304118; Schirinzi, T., Self-reported needs of patients with Parkinson’s disease during COVID-19 emergency in Italy (2020) Neurol. Sci., 41, pp. 1373-1375. , PID: 32363506; Song, J., The changes of exercise pattern and clinical symptoms in patients with Parkinson’s disease in the era of COVID-19 pandemic (2020) Parkinsonism Relat. Disord., 80, pp. 148-151. , PID: 33002722; Brown, E.G., The effect of the COVID-19 pandemic on people with Parkinson’s disease (2020) J. Parkinsons Dis., 10, pp. 1365-1377. , COI: 1:CAS:528:DC%2BB3cXit1ags7jL, PID: 32925107; Chaudhuri, K.R., Healy, D.G., Schapira, A.H.V., Non-motor symptoms of Parkinson’s disease: diagnosis and management (2006) Lancet Neurol., 5, pp. 235-245. , PID: 16488379; Pontone, G.M., Report from a multidisciplinary meeting on anxiety as a non-motor manifestation of Parkinson’s disease (2019) npj Parkinsons Dis., 5, p. 30. , PID: 31840044; (2020) Interim Infection Prevention and Control Recommendations for Patients with Suspected Or Confirmed Coronavirus Disease 2019 (COVID-19) in Healthcare Settings, , https://www.cdc.gov/coronavirus/2019-ncov/hcp/infection-control-recommendations.html; (2020) Non-Emergent, Elective Medical Services, and Treatment Recommendations, , https://www.cms.gov/files/document/cms-non-emergent-elective-medical-recommendations.pdf; Hcp, H.P., Interim Infection Prevention and Control Recommendations

for Patients with Suspected or Confirmed Coronavirus Disease 2019 (COVID-19) in Healthcare Settings (2020) National Center for Chronic Disease Prevention and Health Promotion, , https://stacks.cdc.gov/view/cdc/86043, Division of Diabetes, Translation, Atlanta, GA; Ceravolo, M.G., de Sire, A., Andrenelli, E., Negrini, F., Negrini, S., Systematic rapid “living” review on rehabilitation needs due to COVID-19: update to March 31st, 2020 (2020) Eur. J. Phys. Rehabil. Med., 56, pp. 347-353. , PID: 32316718; Leocani, L., Diserens, K., Moccia, M., Caltagirone, C., Disability through COVID-19 pandemic: Neurorehabilitation cannot wait (2020) Eur. J. Neurol, 27, pp. e50-e51; Chen, P., Coronavirus disease (COVID-19): the need to maintain regular physical activity while taking precautions (2020) J. Sport Health Sci., 9, pp. 103-104. , PID: 32099716; Amekran, Y., El Hangouche, A.J., Coronavirus disease (COVID-19) and the need to maintain regular physical activity (2021) J. Sports Med. Phys. Fitness., 61, pp. 159-160; Jiménez-Pavón, D., Carbonell-Baeza, A., Lavie, C.J., Physical exercise as therapy to fight against the mental and physical consequences of COVID-19 quarantine: Special focus in older people (2020) Prog. Cardiovasc. Dis., 63, pp. 386-388. , PID: 32220590; https://www.csp.org.uk/publications/covid-19-guide-rapid-implementation-remote-consultations; Lei, C., Effects of virtual reality rehabilitation training on gait and balance in patients with Parkinson’s disease: a systematic review (2019) PLoS ONE, 14. , COI: 1:CAS:528:DC%2BC1MXit1emtrbP, PID: 31697777; Constantinescu, G., Treating disordered speech and voice in Parkinson’s disease online: a randomized controlled non-inferiority trial (2011) Int J. Lang. Commun. Disord., 46, pp. 1-16. , PID: 21281410; Theodoros, D.G., Hill, A.J., Russell, T.G., Clinical and quality of life outcomes of speech treatment for Parkinson’s disease delivered to the home via telerehabilitation: a noninferiority randomized controlled trial (2016) Am. J. Speech Lang. Pathol., 25, pp. 214-232. , PID: 27145396; Garcia-Agundez, A., Recent advances in rehabilitation for Parkinson’s disease with exergames: a systematic review (2019) J. Neuroeng. Rehabil., 16, p. 17. , PID: 30696453; van der Kolk, N.M., Effectiveness of home-based and remotely supervised aerobic exercise in Parkinson’s disease: a double-blind, randomised controlled trial (2019) Lancet Neurol., 18, pp. 998-1008. , PID: 31521532; Chen, Y.Y., Application of telehealth intervention in Parkinson’s disease: a systematic review and meta-analysis (2020) J. Telemed. Telecare., 26, pp. 3-13. , PID: 30153767; Schneider, R.B., Biglan, K.M., The promise of telemedicine for chronic neurological disorders: the example of Parkinson’s disease (2017) Lancet Neurol., 16, pp. 541-551. , PID: 28566190; https://www.apta.org/your-practice/practice-models-and-settings/telehealth-practice; Lee, A., (2019) Report of the WCPT/INPTRA Digital Physical Therapy Practice Task Force.; Chang, M.C., Boudier-Revéret, M., Usefulness of telerehabilitation for stroke patients during the COVID-19 pandemic (2020) Am. J. Phys. Med. Rehabil., 99, p. 582. , PID: 32404640; Tenforde, A.S., Evidence-based physiatry: efficacy of home-based telerehabilitation versus in-clinic therapy for adults after stroke (2020) Am. J. Phys. Med. Rehabil, 99, pp. 764-765. , PID: 32433244; Laver, K.E., Telerehabilitation services for stroke (2020) Cochrane Database Syst. Rev., 1, p. CD010255. , PID: 32002991; Dial, H.R., Investigating the utility of teletherapy in individuals with primary progressive aphasia (2019) Clin. Inter. Aging, 14, pp. 453-471; Veerapandiyan, A., The care of patients with Duchenne, Becker, and other muscular dystrophies in the COVID-19 pandemic (2020) Muscle Nerve., 62, pp. 41-45. , COI: 1:CAS:528:DC%2BB3cXht1SltrvN, PID: 32329920; Lai, B., COVID-19 modifications for remote teleassessment and teletraining of a complementary alternative medicine intervention for people with multiple sclerosis: protocol

for a randomized controlled trial (2020) JMIR Res. Protoc., 9. , PID: 32540838; Quinn, L., Macpherson, C., Long, K., Shah, H., Promoting physical activity via telehealth in people with Parkinson disease: the path forward after the COVID-19 pandemic? (2020) Phys Ther, 100, pp. 1730-1736. , &; Isernia, S., Effects of an innovative telerehabilitation intervention for people with Parkinson’s disease on quality of life, motor, and non-motor abilities (2020) Front. Neurol., 11, p. 846. , PID: 32903506; Parkinson’s on the Move., , https://pdonthemove.com; Davis Phinney Foundation, , https://davisphinneyfoundation.org/parkinsons-exercise-essentials-video/#ExerciseGetStarted; https://www.apdaparkinson.org/community/st-louis/resources-support-stl/exercise-classes/joy-of-movement/; The Brian Grant Foundation, , https://briangrant.org/exercise-videos/; Dockx, K., Virtual reality for rehabilitation in Parkinson’s disease (2016) Cochrane Database Syst. Rev., 12, p. CD010760. , PID: 28000926; Gandolfi, M., Virtual reality telerehabilitation for postural instability in Parkinson’s disease: a multicenter, single-blind, randomized, controlled trial (2017) Biomed. Res. Int., 2017, p. 7962826. , PID: 29333454; van der Kolk, N.M., King, L.A., Effects of exercise on mobility in people with Parkinson’s disease (2013) Mov. Disord., 28, pp. 1587-1596. , PID: 24132847; Speelman, A.D., How might physical activity benefit patients with Parkinson disease? (2011) Nat. Rev. Neurol., 7, pp. 528-534. , PID: 21750523; Mele, B., Diagnosis, treatment and management of apathy in Parkinson’s disease: a scoping review (2020) BMJ Open., 10. , PID: 32907903; Friedman, J.H., Fatigue in Parkinson’s disease (2016) J Parkinsons Dis., 2, p. 15025. , COI: 1:CAS:528:DC%2BB3cXhsVarsb4%3D; (2018) Global Action Plan on Physical Activity 2018–2030: More Active People for a Healthier World, , World Health Organization; Carl, J., Sudeck, G., Pfeifer, K., Competencies for a healthy physically active lifestyle-reflections on the model of physical activity-related health competence (2020) J. Phys. Act. Health., pp. 1-10. , https://doi-org.ez.srv.meduniwien.ac.at/10.1123/jpah.2019-0442; Buetow, S.A., Martínez-Martín, P., Hirsch, M.A., Okun, M.S., Beyond patient-centered care: person-centered care for Parkinson’s disease (2016) NPJ Parkinson’s Dis., 2, p. 16019; Dancing through Parkinson’s ®., , https://www.invertigodance.org/dtp; https://www.epda.eu.com/exercisecast; The Parkinson’s Fitness Project: The Daily Dose PD., , https://www.dailydosepd.com/free-workouts-2?_ga=2.251054467.426691946.1588676011-35375576.1588676011; twitter.com/michaeljfoxorg; (2019) Parkinson’s Social Network Myhealthteams, Inc V. 12.0.4(Mobile Application Software, , https://apps.apple.com/us/app/parkinsons-social-network/id1339366057; Exercises for People with Parkinson’s., , https://www.parkinsons.va.gov; A Body Guide to Parkinson’s Disease: Parkinson’s Foundation, , https://www.parkinson.org/sites/default/files/Fitness_Counts.pdf; How to Stay Active and Exercise at Home Parkinson’s Disease Society of the United Kingdom, , https://www.parkinsons.org.uk/information-and-support/exercise-progressing-symptoms; Srivastav, A.K., Samuel, A.J., E-rehabilitation: one solution for patients with Parkinson’s disease in COVID-19 era (2020) Parkinsonism Relat. Disord., 75, pp. 128-129. , PID: 32532625; Lakshminarayana, R., Using a smartphone-based self-management platform to support medication adherence and clinical consultation in Parkinson’s disease (2017) NPJ Parkinson’s Dis., 3, p. 2; Ginis, P., Feasibility and effects of home-based smartphone-delivered automated feedback training for gait in people with Parkinson’s disease: a pilot randomized controlled trial (2016) Parkinsonism Relat. Disord., 22, pp. 28-34. , PID: 26777408; Bot, B.M., The mPower study, Parkinson disease mobile

data collected using ResearchKit (2016) Sci. Data., 3. , COI: 1:CAS:528:DC%2BC28XjvVymsbY%3D, PID: 26938265; Trister, A.D., Dorsey, E.R., Friend, S.H., Smartphones as new tools in the management and understanding of Parkinson's disease (2016) NPJ Parkinson's Dis., 2, p. 16006; Ivkovic, V., Fisher, S., Paloski, W.H., Smartphone-based tactile cueing improves motor performance in Parkinson's disease (2016) Parkinsonism Relat. Disord., 22, pp. 42-47. , PID: 26589004; Samà, A., A double closed loop to enhance the quality of life of Parkinson's disease patients: REMPARK system (2014) Stud. Health Technol. Inform., 207, pp. 115-124. , PID: 25488217; Landers, M.R., Ellis, T.D., A mobile app specifically designed to facilitate exercise in Parkinson disease: single-cohort pilot study on feasibility, safety, and signal of efficacy (2020) JMIR Mhealth Uhealth., 8. , PID: 33016887; Siegert, C., Hauptmann, B., Jochems, N., Schrader, A., Deck, R., ParkProTrain: an individualized, tablet-based physiotherapy training programme aimed at improving quality of life and participation restrictions in PD patients - a study protocol for a quasi-randomized, longitudinal and sequential multi-method study (2019) BMC Neurol., 19. , PID: 31238908; Hulbert, S., Fullam, J., Hunt, C., Goodwin, V.A., 'Digital dancing' - "can you see, what I feel" - an exploration of the physical 'experience' of dance for Parkinson's through 3-dimensional motion analysis (2020) Complement Ther. Med., 52, p. 102508. , PID: 32951755; Ellis, T.D., Comparative effectiveness of mHealth-supported exercise compared with exercise alone for people with Parkinson disease: randomized controlled pilot study (2019) Phys Ther., 99, pp. 203-216. , PID: 30715489; (2020) Charity Miles Charity Miles LLC V. 7.2.1 (Mobile Application Software, , https://apps.apple.com/us/app/charity-miles/id505253234; (2020) Parkinson's Moving Day Charity Dynamics, Inc. V. 1.3, , https://play.google.com/store/apps/details?id=com.charitydynamics.parkinson&hl=de_AT, (Mobile application software; (2020) APDA Symptom Tracker American Parkinson Disease Association V. 1.0.1 (Mobile Application Software, , https://play.google.com/store/apps/details?id=com.aoic.apdahealthcaretrackerapp&hl=de_AT; (2018) Parkinson Mpower 2 Sage Bionetworks, a Not-For-Profit Research Organization V. 2.1, , https://apps.apple.com/us/app/parkinson-mpower-2/id1375781575, (Mobile application software; (2020) Umotif: Umotif V.3.9.0, , https://play.google.com/store/apps/details?id=com.umotif.umotif_wellbeing&hl=en_US, (Mobile application software; (2019) Beats Medical Parkinsons Treat: Beats Medical V. 3.20 (Mobile Application Software, , https://www.parkinsons.org.uk/information-and-support/exercise-progressing-symptoms; (2018) 9Zest Parkinson's Therapy & Exercises V.3.2.2 Ed. 9Zest, , (Mobile application software; (2013) Parkinson Exercises Mobile European Foundation for Health and Exercise V. 1.0.1 (Mobile Application Software, , https://play.google.com/store/apps/details?id=nl.efox.parkinsonss.en.phone&hl=en_US; (2019) NEURO PHYSIO V.1.0.6, , https://play.google.com/store/apps/details?id=com.pd.warrior, (Mobile application software; (2018) Yoga against Parkinson's Physup Ehealth V. 1.0, , https://play.google.com/store/apps/details?id=com.mr_ingrisano.Yoga_against_Parkinsons&hl=de_AT, Mobile application software; (2020) Voice Analyst: Pitch & Volume Speechtools Ltd 2014-2020 V. 2.31, , https://apps.apple.com/us/app/voice-analyst-pitch-volume, Mobile application software; Chao, Y.Y., Scherer, Y.K., Montgomery, C.A., Effects of using Nintendo Wii™ exergames in older adults: a review of the literature (2015) J. Aging Health, 27, pp. 379-402. , PID: 25245519; Barry, G., Galna, B., Rochester, L., The role of exergaming in Parkinson's disease rehabilitation: a systematic review of the evidence (2014) J. Neuroeng. Rehabil., 11, p. 33. ,

PID: 24602325; Shih, M.C., Wang, R.Y., Cheng, S.J., Yang, Y.R., Effects of a balance-based exergaming intervention using the Kinect sensor on posture stability in individuals with Parkinson's disease: a single-blinded randomized controlled trial (2016) J. Neuroeng. Rehabil., 13, p. 78. , PID: 27568011; Ribas, C.G., Alves da Silva, L., Corrêa, M.R., Teive, H.G., Valderramas, S., Effectiveness of exergaming in improving functional balance, fatigue and quality of life in Parkinson's disease: a pilot randomized controlled trial (2017) Parkinsonism Relat. Disord., 38, pp. 13-18. , PID: 28190675; Ferraz, D.D., The effects of functional training, bicycle exercise, and exergaming on walking capacity of elderly patients with Parkinson disease: a pilot randomized controlled single-blinded trial (2018) Arch. Phys. Med. Rehabil., 99, pp. 826-833. , PID: 29337023; van Beek, J.J.W., van Wegen, E.E.H., Bohlhalter, S., Vanbellingen, T., Exergaming-based dexterity training in persons With Parkinson disease: a pilot feasibility study (2019) J. Neurol. Phys. Ther., 43, pp. 168-174. , PID: 31136450; Cikajlo, I., Can telerehabilitation games lead to functional improvement of upper extremities in individuals with Parkinson's disease? (2018) Int. J. Rehabil. Res., 41, pp. 230-238. , PID: 29757774; Allen, N.E., An interactive videogame for arm and hand exercise in people with Parkinson's disease: a randomized controlled trial (2017) Parkinsonism Relat. Disord., 41, pp. 66-72. , PID: 28528804; Schaeffer, E., Effects of exergaming on attentional deficits and dual-tasking in Parkinson's disease (2019) Front. Neurol., 10, p. 646. , PID: 31275234; Hatcher-Martin, J.M., Telemedicine in neurology: Telemedicine Work Group of the American Academy of Neurology update (2020) Neurology, 94, pp. 30-38. , PID: 31801829; Spear, K.L., Auinger, P., Simone, R., Dorsey, E.R., Francis, J., Patient views on telemedicine for Parkinson disease (2019) J. Parkinsons Dis., 9, pp. 401-404. , PID: 31127732; Block, V.A., Remote physical activity monitoring in neurological disease: a systematic review (2016) PLoS ONE, 11. , PID: 27124611, COI: 1:CAS:528:DC%2BC28XhslarsLjJ; Mammen, J.R., Patient and physician perceptions of virtual visits for Parkinson's disease: a qualitative study (2018) Telemed. J. E. Health, 24, pp. 255-267. , PID: 28787250; Beck, C.A., National randomized controlled trial of virtual house calls for Parkinson disease (2017) Neurology, 89, pp. 1152-1161. , PID: 28814455; Chirra, M., Telemedicine in neurological disorders: opportunities and challenges (2019) Telemed. J. E. Health, 25, pp. 541-550. , PID: 30136898; Peacock, D., Perception of healthcare access and utility of telehealth among Parkinson's disease patients (2020) Can. J. Neurol. Sci., 47, pp. 700-704. , PID: 32450924; https://www.apple.com/app-store; https://play.google.com/store; Chaudhuri, K.R., International multicenter pilot study of the first comprehensive self-completed nonmotor symptoms questionnaire for Parkinson's disease: the NMSQuest study (2006) Mov. Disord., 21, pp. 916-923. , PID: 16547944},
correspondence_address1={Langer, A.; Department of Neurology, Austria; email: agnes.langer@meduniwien.ac.at},
publisher={Nature Research},
issn={23738057},
language={English},
abbrev_source_title={NPJ Parkinson's Dis.},
document_type={Review},
source={Scopus},
}

@ARTICLE{Curtis2021,
author={Curtis, R.G. and Olds, T. and Fraysse, F. and Dumuid, D. and Hendrie, G.A. and Esterman, A. and Brown, W.J. and Ferguson, T. and Lagiseti, R. and

Maher, C.A.},
title={Annual rhythms in adults’ lifestyle and health (ARIA): protocol for a 12-month longitudinal study examining temporal patterns in weight, activity, diet, and wellbeing in Australian adults},
journal={BMC Public Health},
year={2021},
volume={21},
number={1},
doi={10.1186/s12889-020-10054-3},
art_number={70},
note={cited By 0},
url={https://www.scopus.com/inward/record.uri?eid=2-s2.0-85098866881&doi=10.1186%2fs12889-020-10054-3&partnerID=40&md5=571e6d10742957bb3a16b0911f7b3c1d},
affiliation={Alliance for Research in Exercise, Nutrition and Activity, UniSA Allied Health and Human Performance, University of South Australia, Adelaide, SA, Australia; Health and Biosecurity, Commonwealth Scientific and Industrial Research Organisation, Adelaide, SA, Australia; UniSA Clinical and Health Sciences, University of South Australia, Adelaide, SA, Australia; School of Human Movement and Nutrition Sciences, University of Queensland, Brisbane, QLD, Australia},
abstract={Background: Almost one in three Australian adults are now obese, and the rate continues to rise. The causes of obesity are multifaceted and include environmental, cultural and lifestyle factors. Emerging evidence suggests there may be temporal patterns in weight gain related, for example, to season and major festivals such as Christmas, potentially due to changes in diet, daily activity patterns or both. The aim of this study is to track the annual rhythm in body weight, 24 h activity patterns, dietary patterns, and wellbeing in a cohort of Australian adults. In addition, through data linkage with a concurrent children’s cohort study, we aim to examine whether changes in children’s body mass index, activity and diet are related to those of their parents. Methods: A community-based sample of 375 parents aged 18 to 65 years old, residing in or near Adelaide, Australia, and who have access to a Bluetooth-enabled mobile device or a computer and home internet, will be recruited. Across a full year, daily activities (minutes of moderate to vigorous physical activity, light physical activity, sedentary behaviour and sleep) will be measured using wrist-worn accelerometry (Fitbit Charge 3). Body weight will be measured daily using Fitbit wifi scales. Self-reported dietary intake (Dietary Questionnaire for Epidemiological Studies V3.2), and psychological wellbeing (WHOQOL-BREF and DASS-21) will be assessed eight times throughout the 12-month period. Annual patterns in weight will be examined using Lowess curves. Associations between changes in weight and changes in activity and diet compositions will be examined using repeated measures multi-level models. The associations between parent’s and children’s weight, activity and diet will be investigated using multi-level models. Discussion: Temporal factors, such as day type (weekday or weekend day), cultural celebrations and season, may play a key role in weight gain. The aim is to identify critical opportunities for intervention to assist the prevention of weight gain. Family-based interventions may be an important intervention strategy. Trial registration: Australia New Zealand Clinical Trials Registry, identifier ACTRN12619001430123. Prospectively registered on 16 October 2019. © 2021, The Author(s).},
author_keywords={Compositional data; Diet; Epidemiology; Obesity; Overweight; Physical activity; Sedentary behaviour; Sleep; Weight},
references={(2019) National Health Survey: First Results, 2017–18: Canberra,

Commonwealth of Australia, , https://www.abs.gov.au/statistics/health/health-conditions-and-risks/national-health-survey-first-results/latest-release; (2017) Organisation for Economic Co-Operation and Development. Obesity Update 2017. OECD, , https://wwwoecdorg/health/health-systems/Obesity-Update-2017pdf; Weiderpass, E., Persson, I., Adami, H.-O., Magnusson, C., Lindgren, A., Baron, J.A., Body size in different periods of life, diabetes mellitus, hypertension, and risk of postmenopausal endometrial cancer (2000) Cancer Causes Control, 11 (2), pp. 185-192. , COI: 1:STN:280:DC%2BD3c7ntlCgug%3D%3D, PID: 10710204; Separate and combined associations of body-mass index and abdominal adiposity with cardiovascular disease: collaborative analysis of 58 prospective studies (2011) Lancet., 377 (9771), pp. 1085-1095; Anandacoomarasamy, A., Caterson, I., Sambrook, P., Fransen, M., March, L.J., The impact of obesity on the musculoskeletal system (2008) Curr Opin Rheumatol, 32 (2), pp. 211-222. , COI: 1:STN:280:DC%2BD1c7hvFaruw%3D%3D; Weighing (2019) Australia's Growing Obesity Epidemic, , https://static1.squarespace.com/static/57e9ebb16a4963ef7adfafdb/t/5c9a8961f4e1fc9deceb1ae4/1553631602322/Obesity+Collective_Australias+Growing+Obesity+Epidemic+report+27+03+19.pdf, https://static1.squarespace.com/static/57e9ebb16a4963ef7adfafdb/t/5c9a8961f4e1fc9deceb1ae4/1553631602322/Obesity+Collective_Australias+Growing+Obesity+Epidemic+report+27+03+19.pdf Accessed 11 November 2020; Maher, C.A., Mire, E., Harrington, D.M., Staiano, A.E., Katzmarzyk, P.T., The independent and combined associations of physical activity and sedentary behavior with obesity in adults: NHANES 2003-06 (2013) Obesity., 21 (12), pp. e730-e737. , PID: 23512825; Cappuccio, F.P., Taggart, F.M., Kandala, N.-B., Currie, A., Peile, E., Stranges, S., Meta-analysis of short sleep duration and obesity in children and adults (2008) Sleep., 31 (5), pp. 619-626. , PID: 18517032; (2019) Overweight and Obesity: An Interactive Insight, , https://www.aihw.gov.au/reports/overweight-obesity/overweight-and-obesity-an-interactive-insight/contents/differences-between-groups, AIHW, Canberra: https://www.aihw.gov.au/reports/overweight-obesity/overweight-and-obesity-an-interactive-insight/contents/differences-between-groups Accessed 3 November 2020; Sahoo, K., Sahoo, B., Choudhury, A.K., Sofi, N.Y., Kumar, R., Bhadoria, A.S., Childhood obesity: causes and consequences (2015) J Family Med Prim Care, 4 (2), pp. 187-192. , PID: 25949965; Tanamas, S.K., Shaw, J.E., Backholer, K., Magliano, D.J., Peeters, A., Twelve-year weight change, waist circumference change and incident obesity: the Australian diabetes, obesity and lifestyle study (2014) Obesity., 22 (6), pp. 1538-1545. , PID: 24436317; Brown, W.J., Williams, L., Ford, J.H., Ball, K., Dobson, A.J., Identifying the energy gap: magnitude and determinants of 5-year weight gain in midage women (2005) Obes Res, 13 (8), pp. 1431-1441. , PID: 16129726; Haines, P.S., Hama, M.Y., Guilkey, D.K., Popkin, B.M., Weekend eating in the United States is linked with greater energy, fat, and alcohol intake (2003) Obes Res, 11 (8), pp. 945-949. , PID: 12917498; Tudor-Locke, C., Ham, S.A., Macera, C.A., Ainsworth, B.E., Kirtland, K.A., Reis, J.P., Descriptive epidemiology of pedometer-determined physical activity (2004) Med Sci Sports Exerc, 36 (9), pp. 1567-1573. , PID: 15354039; Miller, R., Brown, W., Steps and sitting in a working population (2004) Int J Behav Med, 11 (4), pp. 219-224. , PID: 15657022; Im, H.-J., Baek, S.-H., Chu, M.K., Yang, K.I., Kim, W.-J., Park, S.-H., Association between weekend catch-up sleep and lower body mass: population-based study (2017) Sleep, 40 (7), p. zsx089; Roepke, S.E., Duffy, J.F., Differential impact of chronotype on weekday and weekend sleep timing and duration (2010) Nat Sci Sleep, 2010 (2), pp. 213-220. , PID: 20890372; Díaz-Zavala, R.G., Castro-Cantú, M.F., Valencia,

M.E., Álvarez-Hernández, G., Haby, M.M., Esparza-Romero, J., Effect of the holiday season on weight gain: a narrative review (2017) J Obes, 2017, p. 2085136. , PID: 28744374; Yanovski, J.A., Yanovski, S.Z., Sovik, K.N., Nguyen, T.T., O'Neil, P.M., Sebring, N.G., A prospective study of holiday weight gain (2000) N Engl J Med, 342 (12), pp. 861-867. , COI: 1:STN:280:DC%2BD3c7ntVGitQ%3D%3D, PID: 10727591; Helander, E.E., Wansink, B., Chieh, A., Weight gain over the holidays in three countries (2016) N Engl J Med, 375 (12), pp. 1200-1202. , PID: 27653588; Visscher, T.L., Seidell, J.C., Time trends (1993-1997) and seasonal variation in body mass index and waist circumference in the Netherlands (2004) Int J Obes Relat Metab Disord, 28 (10), pp. 1309-1316. , COI: 1:STN:280:DC%2BD2cvosVGhug%3D%3D, PID: 15314624; Shahar, D.R., Froom, P., Harari, G., Yerushalmi, N., Lubin, F., Kristal-Boneh, E., Changes in dietary intake account for seasonal changes in cardiovascular disease risk factors (1999) Eur J Clin Nutr, 53 (5), pp. 395-400. , COI: 1:STN:280:DyaK1M3ps1WrtQ%3D%3D, PID: 10369496; Adamsson, M., Laike, T., Morita, T., Seasonal variation in bright daylight exposure, mood and behavior among a group of office workers in Sweden (2018) J Circadian Rhythms, 16, p. 2. , PID: 30210562; Suzuki, M., Taniguchi, T., Furihata, R., Yoshita, K., Arai, Y., Yoshiike, N., Seasonal changes in sleep duration and sleep problems: a prospective study in Japanese community residents (2019) PLoS One, 14 (4). , COI: 1:CAS:528:DC%2BC1MXps1Sgur8%3D, PID: 30998709; Friborg, O., Bjorvatn, B., Amponsah, B., Pallesen, S., Associations between seasonal variations in day length (photoperiod), sleep timing, sleep quality and mood: a comparison between Ghana (5°) and Norway (69°) (2012) J Sleep Res, 21 (2), pp. 176-184. , PID: 22074234; O’Connell, S.E., Griffiths, P.L., Clemes, S.A., Seasonal variation in physical activity, sedentary behaviour and sleep in a sample of UK adults (2014) Ann Hum Biol, 41 (1), pp. 1-8. , PID: 23992280; Hamilton, S.L., Clemes, S.A., Griffiths, P.L., UK adults exhibit higher step counts in summer compared to winter months (2008) Ann Hum Biol, 35 (2), pp. 154-169. , PID: 18428010; Matthews, C.E., Freedson, P.S., Hebert, J.R., Stanek, E.J., III, Merriam, P.A., Rosal, M.C., Seasonal variation in household, occupational, and leisure time physical activity: longitudinal analyses from the seasonal variation of blood cholesterol study (2001) Am J Epidemiol, 153 (2), pp. 172-183. , COI: 1:STN:280:DC%2BD3M7is1Krsg%3D%3D, PID: 11159163; Plasqui, G., Westerterp, K.R., Seasonal variation in total energy expenditure and physical activity in Dutch young adults (2004) Obes Res, 12 (4), pp. 688-694; Lewis, L.K., Maher, C., Belanger, K., Tremblay, M., Chaput, J.P., Olds, T., At the mercy of the gods: associations between weather, physical activity, and sedentary time in children (2016) Pediatr Exerc Sci, 28 (1), pp. 152-163. , PID: 26098393; Watson, A., Maher, C., Tomkinson, G.R., Golley, R., Fraysse, F., Dumuid, D., Life on holidays: study protocol for a 3-year longitudinal study tracking changes in children’s fitness and fatness during the in-school versus summer holiday period (2019) BMC Public Health, 19 (1), p. 1353. , PID: 31646994; Shaffer, J.A., Diaz, K., Alcántara, C., Edmondson, D., Krupka, D.J., Chaplin, W.F., An inexpensive device for monitoring patients' weights via automated hovering (2014) Int J Cardiol, 172 (2), pp. e263-e264. , PID: 24508493; Yorkin, M., Spaccarotella, K., Martin-Biggers, J., Quick, V., Byrd-Bredbenner, C., Accuracy and consistency of weights provided by home bathroom scales (2013) BMC Public Health, 13 (1), p. 1194. , PID: 24341761; Marfell-Jones, M.J., Stewart, A.D., de Ridder, J.H., (2012) International standards for anthropometric assessment, , International Society for the Advancement of Kinanthropometry, Wellington; Wright, S.P., Collier, S.R., Brown, T.S., Sandberg, K., An analysis of how consumer physical activity monitors are used in biomedical research (2017) FASEB J, 31, p. 1020.1024;

Henriksen, A., Haugen Mikalsen, M., Woldaregay, A.Z., Muzny, M., Hartvigsen, G., Hopstock, L.A., Using fitness trackers and smartwatches to measure physical activity in research: analysis of consumer wrist-worn wearables (2018) J Med Internet Res, 20 (3). , PID: 29567635; Reid, R.E.R., Insogna, J.A., Carver, T.E., Comptour, A.M., Bewski, N.A., Sciortino, C., Validity and reliability of Fitbit activity monitors compared to ActiGraph GT3X+ with female adults in a free-living environment (2017) J Sci Med Sport, 20 (6), pp. 578-582. , PID: 27887786; de Zambotti, M., Goldstone, A., Claudatos, S., Colrain, I.M., Baker, F.C., A validation study of Fitbit charge 2™ compared with polysomnography in adults (2018) Chronobiol Int, 35 (4), pp. 465-476. , PID: 29235907; Mantua, J., Gravel, N., Spencer, R.M.C., Reliability of sleep measures from four personal health monitoring devices compared to research-based actigraphy and polysomnography (2016) Sensors, 16 (5), p. 646; Murakami, H., Kawakami, R., Nakae, S., Nakata, Y., Ishikawa-Takata, K., Tanaka, S., Accuracy of wearable devices for estimating total energy expenditure: comparison with metabolic chamber and doubly labeled water method (2016) JAMA Intern Med, 176 (5), pp. 702-703. , PID: 26999758; Burton, N.W., Haynes, M., Wilson, L.-A.M., Giles-Corti, B., Oldenburg, B.F., Brown, W.J., HABITAT: a longitudinal multilevel study of physical activity change in mid-aged adults (2009) BMC Public Health, 9, p. 76. , PID: 19265552; Milne, R.L., Fletcher, A.S., MacInnis, R.J., Hodge, A.M., Hopkins, A.H., Bassett, J.K., Cohort profile: The Melbourne Collaborative Cohort Study (Health 2020) (2017) Int J Epidemiol, 46 (6), pp. 1757-1757i. , COI: 1:STN:280:DC%2BC1cjgvFGjuw%3D%3D, PID: 28641380; (2013) NUTTAB 2010 – Australian Food Composition Tables, , FSANZ, Canberra; (2008) AUSNUT 2007—Australian Food Supplement and Nutrient Database for Estimation of Population Nutrient Intakes, , FSANZ, Canberra; Hebden, L., Kostan, E., O'Leary, F., Hodge, A., Allman-Farinelli, M., Validity and reproducibility of a food frequency questionnaire as a measure of recent dietary intake in young adults (2013) PLoS One, 8 (9). , COI: 1:CAS:528:DC%2BC3sXhsFeitb3N, PID: 24058660; Xinying, P.X., Noakes, M., Keogh, J., Can a food frequency questionnaire be used to capture dietary intake data in a 4 week clinical intervention trial? (2004) Asia Pac J Clin Nutr, 13 (4), p. 318. , PID: 15563434; Skevington, S.M., Lotfy, M., O'Connell, K.A., The World Health Organization's WHOQOL-BREF quality of life assessment: psychometric properties and results of the international field trial. A report from the WHOQOL group (2004) Qual Life Res, 13 (2), pp. 299-310. , COI: 1:STN:280:DC%2BD2c7ovF2itQ%3D%3D; Hawthorne, G., Herrman, H., Murphy, B., Interpreting the WHOQOL-Brèf: preliminary population norms and effect sizes (2006) Soc Indic Res, 77 (1), pp. 37-59; Krägeloh, C.U., Kersten, P., Rex Billington, D., Hsu, P.H.-C., Shepherd, D., Landon, J., Validation of the WHOQOL-BREF quality of life questionnaire for general use in New Zealand: confirmatory factor analysis and Rasch analysis (2013) Qual Life Res, 22 (6), pp. 1451-1457. , PID: 22983781; Chen, W.-C., Wang, J.-D., Hwang, J.-S., Chen, C.-C., Wu, C.-H., Yao, G., Can the web-form WHOQOL-BREF be an alternative to the paper-form? (2009) Soc Indic Res, 94 (1), pp. 97-114; Lovibond, P.F., Lovibond, S.H., (1995) Manual for the depression anxiety stress scales, , 2, Psychology Foundation, Sydney; Henry, J.D., Crawford, J.R., The short-form version of the depression anxiety stress scales (DASS-21): construct validity and normative data in a large non-clinical sample (2005) Br J Clin Psychol, 44 (2), pp. 227-239. , PID: 16004657; Lovibond, P.F., Lovibond, S.H., The structure of negative emotional states: comparison of the depression anxiety stress scales (DASS) with the Beck depression and anxiety inventories (1995) Behav Res Ther, 33 (3), pp. 335-343. , COI: 1:STN:280:DyaK2M3ktl2jsA%3D%3D, PID: 7726811; Herbert, J., Peterson, K.L., Alston, L., Allender, S., Nichols,

M., Comparison between measured and perceived weight status in a nationally representative sample of Australian adults (2017) Obes Res Clin Pract, 11 (4), pp. 414-425. , PID: 28089396; Serdula, M.K., Williamson, D.F., Anda, R.F., Levy, A., Heaton, A., Byers, T., Weight control practices in adults: results of a multistate telephone survey (1994) Am J Public Health, 84 (11), pp. 1821-1824. , COI: 1:STN:280:DyaK2M%2FmvVGltA%3D%3D, PID: 7977925; Wharton, S., Raiber, L., Serodio, K.J., Lee, J., Christensen, R.A., Medications that cause weight gain and alternatives in Canada: a narrative review (2018) Diabetes Metab Syndr Obes, 11, pp. 427-438. , COI: 1:CAS:528:DC%2BC1MXhs1OnsrjM, PID: 30174450; Domecq, J.P., Prutsky, G., Leppin, A., Sonbol, M.B., Altayar, O., Undavalli, C., Clinical review: drugs commonly associated with weight change: a systematic review and meta-analysis (2015) J Clin Endocrinol Metab, 100 (2), pp. 363-370. , COI: 1:CAS:528:DC%2BC2MXjtVWqsL4%3D, PID: 25590213; Zubrick, S.R., Lucas, N., Westrupp, E.M., (2014) Nicholson. Parenting measures in the longitudinal study of Australian children: construct validity and measurement quality, waves 1 to 4, , Australian Government, Canberra; Katzmarzyk, P.T., Barreira, T.V., Broyles, S.T., Champagne, C.M., Chaput, J.-P., Fogelholm, M., The international study of childhood obesity, lifestyle and the environment (ISCOLE): design and methods (2013) BMC Public Health, 13 (1), p. 900. , PID: 24079373; Anderson, S.E., Must, A., Curtin, C., Bandini, L.G., Meals in our household: reliability and initial validation of a questionnaire to assess child mealtime behaviors and family mealtime environments (2012) J Acad Nutr Diet, 112 (2), pp. 276-284. , PID: 22741169; Horne, J.A., Östberg, O., A self-assessment questionnaire to determine morningness-eveningness in human circadian rhythms (1976) Int J Chronobiol, 4 (2), pp. 97-110. , COI: 1:STN:280:DyaE2s3gs1eqtg%3D%3D, PID: 1027738; Sullivan, K.L., Mortimer, J.A., Wang, W., Zesiewicz, T.A., Brownlee, H.J., Jr., Borenstein, A.R., Early-adult life correlates of personality in Parkinson’s disease (2014) J Neurol Res, 4 (2-3), pp. 51-62; Dumuid, D., Pedišić, Ž., Stanford, T.E., Martín-Fernández, J.-A., Hron, K., Maher, C.A., The compositional isotemporal substitution model: a method for estimating changes in a health outcome for reallocation of time between sleep, physical activity and sedentary behaviour (2017) Stat Methods Med Res, 28 (3), pp. 846-857. , PID: 29157152; Dumuid, D., Stanford, T.E., Martin-Fernández, J.-A., Pedišić, Ž., Maher, C.A., Lewis, L.K., Compositional data analysis for physical activity, sedentary time and sleep research (2017) Stat Methods Med Res, 27 (12), pp. 3726-3738. , PID: 28555522; (2002) Australian social trends, 2002, , https://www.abs.gov.au/AUSSTATS/abs@.nsf/bb8db737e2af84b8ca2571780015701e/1e8c8e4887c33955ca2570ec000a9fe5!OpenDocument, Commonwealth of Australia, Canberra: https://www.abs.gov.au/AUSSTATS/abs@.nsf/bb8db737e2af84b8ca2571780015701e/1e8c8e4887c33955ca2570ec000a9fe5!OpenDocument. Accessed 11 November 2020; Moores, C.J., Maeder, A., Miller, J., Prichard, I., Lewis, L.K., Bell, L.K., A digital intervention for Australian adolescents above a healthy weight (health online for teens): protocol for an implementation and user experience study (2019) JMIR Res Protoc, 8 (10). , PID: 31603435; Yackobovitch-Gavan, M., Wolf Linhard, D., Nagelberg, N., Poraz, I., Shalitin, S., Phillip, M., Intervention for childhood obesity based on parents only or parents and child compared with follow-up alone (2018) Pediatr Obes, 13 (11), pp. 647-655. , COI: 1:STN:280:DC%2BC1MvhvVeltA%3D%3D, PID: 29345113},
correspondence_address1={Maher, C.A.; Alliance for Research in Exercise, Australia; email: Carol.Maher@unisa.edu.au},
publisher={BioMed Central Ltd},
issn={14712458},
pubmed_id={33413247},

language={English},
abbrev_source_title={BMC Public Health},
document_type={Article},
source={Scopus},
}

@ARTICLE{Elavsky2021,
author={Elavsky, S. and Jandačková, V. and Knapová, L. and Vašendová, V. and Sebera, M. and Kaštovská, B. and Blaschová, D. and Kühnová, J. and Cimler, R. and Vilímek, D. and Bosek, T. and Koenig, J. and Jandačka, D.},
title={Physical activity in an air-polluted environment: behavioral, psychological and neuroimaging protocol for a prospective cohort study (Healthy Aging in Industrial Environment study – Program 4)},
journal={BMC Public Health},
year={2021},
volume={21},
number={1},
doi={10.1186/s12889-021-10166-4},
art_number={126},
note={cited By 0},
url={https://www.scopus.com/inward/record.uri?eid=2-s2.0-85099212625&doi=10.1186%2fs12889-021-10166-4&partnerID=40&md5=bf9fd136f75a7076e3f6b1d7c3ae051e},
affiliation={Faculty of Education, University of Ostrava, Ostrava, Czech Republic; Faculty of Informatics, Masaryk University, Brno, Czech Republic; Faculty of Medicine, University of Ostrava, Ostrava, Czech Republic; Faculty of Life Sciences, University of Hradec Kralove, Hradec Králové, Czech Republic; VSB-Technical University of Ostrava, Ostrava, Czech Republic; University Hospital of Child and Adolescent Psychiatry and Psychotherapy, University of Bern, Bern, Switzerland; Section for Experimental Child and Adolescent Psychiatry, Department of Child and Adolescent Psychiatry, Centre for Psychosocial Medicine, University of Heidelberg, Heidelberg, Germany},
abstract={Background: Air pollution has been linked to increased mortality and morbidity. The Program 4 of the Healthy Aging in Industrial Environment study investigates whether the health and wellbeing benefits of physical activity (PA) can be fully realized in individuals living in highly polluted environments. Herein, we introduce the behavioral, psychological and neuroimaging protocol of the study. Methods: This is a prospective cohort study of N = 1500 individuals aged 18–65 years comparing: (1) individuals living in the highly polluted, industrial region surrounding the city of Ostrava (n = 750), and (2) controls from the comparison region with relative low pollution levels in Southern Bohemia (n = 750). Quota sampling is used to obtain samples balanced on age, gender, PA status (60% active runners vs. 40% insufficiently active). Participants are screened and complete baseline assessments through online questionnaires and in-person lab-based assessments of physiological, biomechanical, neuroimaging and cognitive function parameters. Prospective 12-month intensive monitoring of air pollution and behavioral parameters (PA, inactivity, and sleep) follows, with a focus on PA-related injuries and psychological factors through fitness trackers, smartphones, and mobile apps. Subsequently, there will be a 5-year follow-up of the study cohort. Discussion: The design of the study will allow for (1) the assessment of both short-term variation and long-term change in behavioral parameters, (2) evaluation of the incidence of musculoskeletal injuries and psychological factors impacting behavior and injury recovery, and (3) the impact that air pollution status (and change) has on behavior, psychological resilience, and injury recovery.

Furthermore, the integration of MRI techniques and cognitive assessment in combination with data on behavioral, biological and environmental variables will provide an opportunity to examine brain structure and cognitive function in relation to health behavior and air pollution, as well as other factors affecting resilience against and vulnerability to adverse changes in brain structure and cognitive aging. This study will help inform individuals about personal risk factors and decision-makers about the impact of environmental factors on negative health outcomes and potential underlying biological, behavioral and psychological mechanisms. Challenges and opportunities stemming from the timing of the study that coincided with the COVID-19 pandemic are also discussed. © 2021, The Author(s).},
author_keywords={Aging; Air pollution; Cognition; Environment; Health; Neuroimaging; Physical activity},
keywords={H 0294; pyrimidine derivative, adolescent; adult; adverse event; aged; air pollutant; air pollution; brain; chemistry; cognition; diagnostic imaging; exercise; female; health behavior; healthy aging; human; male; methodology; middle aged; neuroimaging; physiology; prospective study; psychological resilience; questionnaire; young adult, Adolescent; Adult; Aged; Air Pollutants; Air Pollution; Brain; Cognition; COVID-19; Exercise; Female; Health Behavior; Healthy Aging; Humans; Male; Middle Aged; Neuroimaging; Prospective Studies; Pyrimidines; Research Design; Resilience, Psychological; Surveys and Questionnaires; Young Adult},
references={(2019) World population ageing 2019: highlights, , https://www.un.org/en/development/desa/population/publications/pdf/ageing/WorldPopulationAgeing2019-Highlights.pdf, https://www.un.org/en/development/desa/population/publications/pdf/ageing/WorldPopulationAgeing2019-Highlights.pdf. Accessed 25 May 2020; (2017) Air Quality Guidelines, , http://www.euro.who.int/__data/assets/pdf_file/0005/78638/E90038.pdf?ua=1, Global update 2005. Particulate matter, ozone, nitrogen dioxide and sulfur dioxide: World Health Organization;, . Accessed 25 May 2020; (2005) WHO Air quality guidelines for particulate matter, ozone, nitrogen dioxide and sulfur dioxide: global update 2005; Kampa, M., Castanas, E., Human health effects of air pollution (2008) Environ Pollut, 151, pp. 362-367. , COI: 1:CAS:528:DC%2BD1cXhsVGqsbg%3D, PID: 17646040; Agency, E.E., (2019) Air quality in Europe: 2019 report, , https://www.eea.europa.eu/publications/air-quality-in-europe-2019, https://www.eea.europa.eu/publications/air-quality-in-europe-2019. Accessed 25 May 2020; (2018) Ambient (outdoor) air pollution. Ambient (outdoor) air pollution, , https://www.who.int/en/news-room/fact-sheets/detail/ambient-(outdoor)-air-quality-and-health, https://www.who.int/en/news-room/fact-sheets/detail/ambient-(outdoor)-air-quality-and-health. Accessed 25 May 2020; Ogen, Y., Assessing nitrogen dioxide (NO2) levels as a contributing factor to coronavirus (COVID-19) fatality (2020) Sci Total Environ, 726, p. 138605; Zhu, Y., Xie, J., Huang, F., Cao, L., Association between short-term exposure to air pollution and COVID-19 infection: evidence from China (2020) Sci Total Environ, 727. , https://doi.org/10.1016/j.scitotenv.2020.138704; Raaschou-Nielsen, O., Andersen, Z.J., Beelen, R., Samoli, E., Stafoggia, M., Weinmayr, G., Air pollution and lung cancer incidence in 17 European cohorts: prospective analyses from the European study of cohorts for air pollution effects (ESCAPE) (2013) Lancet Oncol, 14, pp. 813-822. , PID: 23849838; Cowie, H., Crawford, J.,

Davis, A., Steinle, S., Reis, S., Dixon, K., (2015) Air quality, health, wellbeing and behaviour; Giles, L.V., Koehle, M.S., The health effects of exercising in air pollution (2014) Sports Med, 44, pp. 223-249; Pasqua, L.A., Damasceno, M.V., Cruz, R., Matsuda, M., Martins, M.G., Lima-Silva, A.E., Exercising in air pollution: the cleanest versus dirtiest cities challenge (2018) Int J Environ Res Public Health, 15 (7), p. 1502. , COI: 1:CAS:528:DC%2BC1cXisV2jtrrK; Slezakova, K., Pereira, M.C., Morais, S., Ultrafine particles: levels in ambient air during outdoor sport activities (2020) Environ Pollut, 258, p. 113648. , COI: 1:CAS:528:DC%2BC1MXitlSqt7bK, PID: 31806467; An, R., Zhang, S., Ji, M., Guan, C., Impact of ambient air pollution on physical activity among adults: a systematic review and meta-analysis (2018) Perspect Public Health, 138, pp. 111-121. , PID: 28829249; An, R., Xiang, X., Ambient fine particulate matter air pollution and leisure-time physical inactivity among US adults (2015) Public Health, 129, pp. 1637-1644. , COI: 1:STN:280:DC%2BC287jtlarsQ%3D%3D, PID: 26277287; Hu, L., Zhu, L., Xu, Y., Lyu, J., Imm, K., Yang, L., Relationship between air quality and outdoor exercise behavior in China: a novel mobile-based study (2017) Int J Behav Med, 24, pp. 520-527. , PID: 28534316; Roberts, J.D., Voss, J.D., Knight, B., The association of ambient air pollution and physical inactivity in the United States (2014) PLoS One, 9 (3). , PID: 24598907, COI: 1:CAS:528:DC%2BC2cXhsVKhsbrI; Yu, H., Cheng, J., Gordon, S.P., An, R., Yu, M., Chen, X., Impact of air pollution on sedentary behavior: a cohort study of freshmen at a university in Beijing, China (2018) Int J Environ Res Public Health, 15. , https://doi.org/10.3390/ijerph15122811; Lawrence, W.R., Yang, M., Zhang, C., Liu, R.Q., Lin, S., Wang, S.Q., Association between long-term exposure to air pollution and sleep disorder in Chinese children: the Seven Northeastern Cities study (2018) Sleep, 41 (9), p. zsy122; Yu, H., Chen, P., Paige Gordon, S., Yu, M., Wang, Y., The association between air pollution and sleep duration: A cohort study of freshmen at a University in Beijing, China (2019) Int J Environ Res Public Health, 16. , https://doi.org/10.3390/ijerph16183362; Bloemsma, L.D., Wijga, A.H., Klompmaker, J.O., Janssen, N.A.H., Smit, H.A., Koppelman, G.H., The associations of air pollution, traffic noise and green space with overweight throughout childhood: the PIAMA birth cohort study (2019) Environ Res, 169, pp. 348-356. , COI: 1:CAS:528:DC%2BC1cXitl2itLbI, PID: 30504077; Bloemsma, L.D., Gehring, U., Klompmaker, J.O., Hoek, G., Janssen, N.A.H., Lebret, E., Green space, air pollution, traffic noise and cardiometabolic health in adolescents: the PIAMA birth cohort (2019) Environ Int, 131, p. 104991. , COI: 1:CAS:528:DC%2BC1MXhtlKqtbvL, PID: 31302482; del Pozo, C.B., Alfonso-Rosa, R.M., McGregor, D., Chastin, S.F., Palarea-Albaladejo, J., del Pozo Cruz, J., Sedentary behaviour is associated with depression symptoms: compositional data analysis from a representative sample of 3233 US adults and older adults assessed with accelerometers (2020) J Affect Disord, 265, pp. 59-62; Costa, L.G., Cole, T.B., Dao, K., Chang, Y.C., Coburn, J., Garrick, J.M., Effects of air pollution on the nervous system and its possible role in neurodevelopmental and neurodegenerative disorders (2020) Pharmacol Ther, 210; Braithwaite, I., Zhang, S., Kirkbride, J.B., Osborn, D.P.J., Hayes, J.F., Air pollution (particulate matter) exposure and associations with depression, anxiety, bipolar, psychosis and suicide risk: a systematic review and meta-analysis (2019) Environ Health Perspect, 127. , https://doi.org/10.1289/EHP4595; Newbury, J.B., Arseneault, L., Beevers, S., Kitwiroon, N., Roberts, S., Pariante, C.M., Association of air pollution exposure with psychotic experiences during adolescence (2019) JAMA Psychiatry, 76, pp. 614-623; Petkus, A.J., Younan, D., Wang, X., Serre, M., Vizuete, W., Resnick, S., Particulate

air pollutants and trajectories of depressive symptoms in older women (2019) Am J Geriatr Psychiatry, 27, pp. 1083-1096. , PID: 31311712; Pun, V.C., Manjourides, J., Suh, H., Association of ambient air pollution with depressive and anxiety symptoms in older adults: results from the NSHAP study (2017) Environ Health Perspect, 125, pp. 342-348. , COI: 1:CAS:528:DC%2BC1cXhsVagtbzE, PID: 27517877; Lim, Y.H., Kim, H., Kim, J.H., Bae, S., Park, H.Y., Hong, Y.C., Air pollution and symptoms of depression in elderly adults (2012) Environ Health Perspect, 120, pp. 1023-1028. , COI: 1:CAS:528:DC%2BC38Xht1Sht7bL, PID: 22514209; Sass, V., Kravitz-Wirtz, N., Karceski, S.M., Hajat, A., Crowder, K., Takeuchi, D., The effects of air pollution on individual psychological distress (2017) Health Place, 48, pp. 72-79. , PID: 28987650; Zijlema, W.L., Wolf, K., Emeny, R., Ladwig, K.H., Peters, A., Kongsgård, H., The association of air pollution and depressed mood in 70,928 individuals from four European cohorts (2016) Int J Hyg Environ Health, 219, pp. 212-219. , COI: 1:STN:280:DC%2BC28rivVCruw%3D%3D, PID: 26682644; Kawada, T., Air pollution and depressed mood: consistency of association (2016) Int J Hyg Environ Health, 219, p. 477; Zijlema, W.L., Stolk, R.P., Rosmalen, J.G.M., Response to “air pollution and depressed mood: consistency of association. Air pollution and cardiovascular mortality: caution for meta-analysis (2016) Int J Hyg Environ Health, 219, pp. 478-479; Gu, D., Huang, N., Zhang, M., Wang, F., Under the dome: air pollution, wellbeing, and pro-environmental behaviour among Beijing residents (2015) J Pacific Rim Psychol, 9, pp. 65-77; Perach, R., Allen, C.K., Kapantai, I., Madrid-Valero, J.J., Miles, E., Charlton, R.A., The psychological wellbeing outcomes of nonpharmacological interventions for older persons with insomnia symptoms: a systematic review and meta-analysis (2019) Sleep Med Rev, 43, pp. 1-13. , PID: 30408706; Murawski, B., Wade, L., Plotnikoff, R.C., Lubans, D.R., Duncan, M.J., A systematic review and meta-analysis of cognitive and behavioral interventions to improve sleep health in adults without sleep disorders (2018) Sleep Med Rev, 40, pp. 160-169. , PID: 29397329; Zhai, L., Zhang, H., Zhang, D., Sleep duration and depression among adults: a meta-analysis of prospective studies (2015) Depress Anxiety, 32, pp. 664-670. , PID: 26047492; McDowell, C.P., Dishman, R.K., Gordon, B.R., Herring, M.P., Physical activity and anxiety: a systematic review and meta-analysis of prospective cohort studies (2019) Am J Prev Med, 57, pp. 545-556. , PID: 31542132; Rebar, A.L., Stanton, R., Geard, D., Short, C., Duncan, M.J., Vandelanotte, C., A meta-meta-analysis of the effect of physical activity on depression and anxiety in non-clinical adult populations (2015) Health Psychol Rev, 9, pp. 366-378. , PID: 25739893; Clifford, A., Lang, L., Chen, R., Anstey, K.J., Seaton, A., Exposure to air pollution and cognitive functioning across the life course - a systematic literature review (2016) Environ Res, 147, pp. 383-398. , COI: 1:CAS:528:DC%2BC28Xjslymsrg%3D, PID: 26945620; Underwood, E., Brain pollution: Evidence builds that dirty air causes Alzheimer’s, dementia (2017) Science, 355 (6323), pp. 342-345. , https://doi.org/10.1126/science.355.6323.342; Chen, H., Kwong, J.C., Copes, R., Tu, K., Villeneuve, P.J., van Donkelaar, A., Living near major roads and the incidence of dementia, Parkinson’s disease, and multiple sclerosis: a population-based cohort study (2017) Lancet, 389, pp. 718-726; Kilian, J., Kitazawa, M., The emerging risk of exposure to air pollution on cognitive decline and Alzheimer’s disease – evidence from epidemiological and animal studies (2018) Biom J, 41, pp. 141-162; Russ, T.C., Reis, S., Van Tongeren, M., Air pollution and brain health: defining the research agenda (2019) Curr Opin Psychiatry, 32, pp. 97-104; Younan, D., Petkus, A.J., Widaman, K.F., Wang, X., Casanova, R., Espeland, M.A., Particulate matter and episodic memory decline mediated by early neuroanatomic biomarkers of Alzheimer’s disease (2020) Brain,

143, pp. 289-302; de Prado, B.P., Mercader, E.M.H., Pujol, J., Sunyer, J., Mortamais, M., The effects of air pollution on the brain: a review of studies interfacing environmental epidemiology and neuroimaging (2018) Curr Environ Health Rep, 5, pp. 351-364; Hahad, O., Lelieveld, J., Birklein, F., Lieb, K., Daiber, A., Münzel, T., Ambient air pollution increases the risk of cerebrovascular and neuropsychiatric disorders through induction of inflammation and oxidative stress (2020) Int J Mol Sci, 21, pp. 1-24; Kulick, E.R., Wellenius, G.A., Boehme, A.K., Joyce, N.R., Schupf, N., Kaufman, J.D., Long-term exposure to air pollution and trajectories of cognitive decline among older adults (2020) Neurology, 94, pp. E1782-E1792; Gatto, N.M., Henderson, V.W., Hodis, H.N., St. John, J.A., Lurmann, F., Chen, J.C., Components of air pollution and cognitive function in middle-aged and older adults in Los Angeles (2014) Neurotoxicology, 40, pp. 1-7. , COI: 1:CAS:528:DC%2BC2cXhsFCqtbg%3D, PID: 24148924; Weuve, J., Puett, R.C., Schwartz, J., Yanosky, J.D., Laden, F., Grodstein, F., Exposure to particulate air pollution and cognitive decline in older women (2012) Arch Intern Med, 172, pp. 219-227. , PID: 22332151; Cserbik, D., Chen, J.C., McConnell, R., Berhane, K., Sowell, E.R., Schwartz, J., Fine particulate matter exposure during childhood relates to hemispheric-specific differences in brain structure (2020) Environ Int, 143. , https://doi.org/10.1016/j.envint.2020.105933; Allen, J.L., Klocke, C., Morris-Schaffer, K., Conrad, K., Sobolewski, M., Cory-Slechta, D.A., Cognitive effects of air pollution exposures and potential mechanistic underpinnings (2017) Curr Environ Health Rep, 4, pp. 180-191. , COI: 1:CAS:528:DC%2BC2sXmsVakt70%3D, PID: 28435996; Hamer, M., Chida, Y., Physical activity and risk of neurodegenerative disease: A systematic review of prospective evidence (2009) Psychol Med, pp. 39-42. , https://doi.org/10.1017/S0033291708003681; Erickson, K.I., Hillman, C., Stillman, C.M., Ballard, R.M., Bloodgood, B., Conroy, D.E., Physical activity, cognition, and brain outcomes: a review of the 2018 physical activity guidelines (2019) Med Sci Sports Exerc, 51, pp. 1242-1251. , PID: 31095081; Vaynman, S., Ying, Z., Gomez-Pinilla, F., Hippocampal BDNF mediates the efficacy of exercise on synaptic plasticity and cognition (2004) Eur J Neurosci, 20, pp. 2580-2590; Cipriani, G., Danti, S., Carlesi, C., Borin, G., Danger in the air: air pollution and cognitive dysfunction (2018) Am J Alzheimers Dis Other Dement, 33, pp. 333-341; Bos, I., De Boever, P., Int Panis, L., Meeusen, R., Physical activity, air pollution and the brain (2014) Sports Med, 44, pp. 1505-1518; Buoli, M., Grassi, S., Caldiroli, A., Carnevali, G.S., Mucci, F., Iodice, S., Is there a link between air pollution and mental disorders? (2018) Environ Int, 118, pp. 154-168. , COI: 1:CAS:528:DC%2BC1cXhtV2mur7P, PID: 29883762; Altuğ, H., Fuks, K.B., Hüls, A., Mayer, A.-K., Tham, R., Krutmann, J., Air pollution is associated with depressive symptoms in elderly women with cognitive impairment (2020) Environ Int, 136, p. 105448; Center, P.R., (2016) Smartphone ownership and internet usage continues to climb in emerging economies, , http://www.pewglobal.org/2016/02/22/smartphone-ownership-and-internet-usage-continues-to-climb-in-emerging-economies/; Stone, A., Shiffman, S., Atienza, A., Nebeling, L., (2007) The science of real-time data capture: self-reports in health research, , Oxford University Press, New York; (2020) Life Tables for the Czech Republic, Cohesions Regions, and Regions, , https://www.czso.cz/csu/czso/life-tables-for-the-czech-republic-cohesion-regions-and-regions-2017-2018, . Accessed 1 Oct; Hůnová, I., Ambient air quality in the Czech Republic: past and present (2020) Atmosphere (Basel), 11; Sram, R.J., Binkova, B., Dostal, M., Merkerova-Dostalova, M., Libalova, H., Milcova, A., Health impact of air pollution to children (2013) Int J Hyg Environ Health,

216, pp. 533-540. , COI: 1:CAS:528:DC%2BC3sXntFykuw%3D%3D, PID: 23312845; (2020), www.euro.who.int/physical-activity, Fact Sheet Physical Activity Global recommendations on physical activity for health Consequences of physical inactivity, . Accessed 4 Nov; Staufenbiel, S.M., Penninx, B.W.J.H., Spijker, A.T., Elzinga, B.M., van Rossum, E.F.C., Hair cortisol, stress exposure, and mental health in humans: a systematic review (2013) Psychoneuroendocrinology, 38, pp. 1220-1235. , COI: 1:CAS:528:DC%2BC38XhvVCrsr7E, PID: 23253896; Sapolsky, R.M., (2000) Glucocorticoids and hippocampal atrophy in neuropsychiatric disorders; Stalder, T., Steudte-Schmiedgen, S., Alexander, N., Klucken, T., Vater, A., Wichmann, S., Stress-related and basic determinants of hair cortisol in humans: a meta-analysis (2017) Psychoneuroendocrinology, 77, pp. 261-274. , COI: 1:CAS:528:DC%2BC2sXhslemtLg%3D, PID: 28135674; Manenschijn, L., Koper, J.W., Lamberts, S.W.J., Van Rossum, E.F.C., Evaluation of a method to measure long term cortisol levels (2011) Steroids, 76, pp. 1032-1036. , COI: 1:CAS:528:DC%2BC3MXptVWiur8%3D, PID: 21515299; Cooper, G.A.A., Kronstrand, R., Kintz, P., Society of hair testing guidelines for drug testing in hair (2012) Forensic Sci Int, 218, pp. 20-24. , COI: 1:CAS:528:DC%2BC38XmtFCjsL8%3D, PID: 22088946; Wennig, R., (2000) Potential problems with the interpretation of hair analysis results, , www.elsevier.com/locate/forsciint, www.elsevier.com/locate/forsciint; Gonzalez, D., Jacobsen, D., Ibar, C., Pavan, C., Monti, J., Fernandez Machulsky, N., Hair cortisol measurement by an automated method (2019) Sci Rep, 9 (1), pp. 1-6. , COI: 1:CAS:528:DC%2BC1MXmvFSqtLg%3D; Russell, E., Kirschbaum, C., Laudenslager, M.L., Stalder, T., De Rijke, Y., Van Rossum, E.F.C., (2014) Toward standardization of hair cortisol measurement: results of the first international interlaboratory round robin; Rey, A., (1964) L’examen clinique en psychologie (The Clinical Psychological Examination), , Presse Universitaires de France, Paris; Schoenberg, M.R., Dawson, K.A., Duff, K., Patton, D., Scott, J.G., Adams, R.L., Test performance and classification statistics for the Rey Auditory Verbal Learning Test in selected clinical samples (2006) Arch Clin Neuropsychol, 21, pp. 693-703; Preiss, M., Bartoš, A., Čermáková, R., Nondek, M., Benešová, M., Rodriguez, M., (2012) Neuropsychologická baterie Psychiatrického centra Praha, Klinické vyšetření základních kognitivních funkcí, , 3, Psychiatrické centrum Praha, Praha; Mioshi, E., Dawson, K., Mitchell, J., Arnold, R., Hodges, J.R., The Addenbrooke’s Cognitive Examination revised (ACE-R): a brief cognitive test battery for dementia screening (2006) Int J Geriatr Psychiatry, 21, pp. 1078-1085; Kramer, J.H., Mungas, D., Possin, K.L., Rankin, K.P., Boxer, A.L., Rosen, H.J., NIH EXAMINER: conceptualization and development of an executive function battery (2014) J Int Neuropsychol Soc, 20, pp. 11-19; Peirce, J., Gray, J.R., Simpson, S., MacAskill, M., Höchenberger, R., Sogo, H., PsychoPy2: experiments in behavior made easy (2019) Behav Res Methods, 51, pp. 195-203; Hagler, D.J., Hatton, S.N., Cornejo, M.D., Makowski, C., Fair, D.A., Dick, A.S., Image processing and analysis methods for the adolescent brain cognitive development study (2019) Neuroimage, 202. , https://doi.org/10.1016/j.neuroimage.2019.116091; Adler, S., Whitaker, K., Semmelroch, M., Wagstyl, K., (2018) MELD protocol 3 - FreeSurfer quality control, , https://www.protocols.io/view/meld-protocol-3-freesurfer-quality-control-pdadi2e, https://www.protocols.io/view/meld-protocol-3-freesurfer-quality-control-pdadi2e. Accessed 21 Nov 2020; Messier, S.P., Martin, D.F., Mihalko, S.L., Ip, E., DeVita, P., Cannon, D.W., A 2-year prospective cohort study of overuse running injuries: the runners and injury longitudinal study (TRAILS) (2018) Am J Sports Med, 46, pp. 2211-2221. , PID: 29791183; Bauman, A.E., Reis, R.S., Sallis,

J.F., Wells, J.C., Loos, R.J.F., Martin, B.W., Correlates of physical activity: why are some people physically active and others not? (2012) Lancet, 380, pp. 258-271; Choi, J., Lee, M., Lee, J.K., Kang, D., Choi, J.Y., Correlates associated with participation in physical activity among adults: a systematic review of reviews and update (2017) BMC Public Health, 17. , https://doi.org/10.1186/s12889-017-4255-2; Geidl, W., Schlesinger, S., Mino, E., Miranda, L., Pfeifer, K., Dose-response relationship between physical activity and mortality in adults with noncommunicable diseases: a systematic review and meta-analysis of prospective observational studies (2020) Int J Behav Nutr Phys Act, 17, p. 109; Livingston, G., Huntley, J., Sommerlad, A., Ames, D., Ballard, C., Banerjee, S., Dementia prevention, intervention, and care: 2020 report of the lancet commission (2020) Lancet, 396, pp. 413-446; Kumari, P., Toshniwal, D., Impact of lockdown on air quality over major cities across the globe during COVID-19 pandemic (2020) Urban Clim, 34, p. 100719; Zajenkowski, M., Jonason, P.K., Leniarska, M., Kozakiewicz, Z., Who complies with the restrictions to reduce the spread of COVID-19?: personality and perceptions of the COVID-19 situation (2020) Pers Individ Dif, 166, p. 110199; Dawel, A., Shou, Y., Smithson, M., Cherbuin, N., Banfield, M., Calear, A.L., The effect of COVID-19 on mental health and wellbeing in a representative sample of Australian adults (2020) Front Psychiatry, 11, p. 579985; He, G., Pan, Y., Tanaka, T., Urban air pollution in China (2020) Nat Sustain; Martinez, E.Z., Silva, F.M., Morigi, T.Z., Zucoloto, M.L., Silva, T.L., Joaquim, A.G., Physical activity in periods of social distancing due to covid-19: a cross-sectional survey (2020) Cienc e Saude Coletiva, 25, pp. 4157-4168; Meyer, J., McDowell, C., Lansing, J., Brower, C., Smith, L., Tully, M., Changes in physical activity and sedentary behavior in response to covid-19 and their associations with mental health in 3052 us adults (2020) Int J Environ Res Public Health, 17, pp. 1-13; Dunca, G.E., Aver, A.R., Seto, E., Tsang, S., Perceived change in physical activity levels and mental health during COVID-19: findings among adult twin pairs (2020) PLoS One, 15 (8 August), pp. 1-12; Faulkner, G., Rhodes, R.E., Vanderloo, L.M., Chulak-Bozer, T., O’Reilly, N., Ferguson, L., Physical activity as a coping strategy for mental health due to the COVID-19 virus: a potential disconnect among Canadian adults? (2020) Front Commun, 5 (September), pp. 1-5; (2020), https://doi.org/10.1787/69096873-en, . Education at a glance 2020: OECD; Napa Scollon, C., Prieto, C.-K., Diener, E., (2009) Experience sampling: promises and pitfalls, strength and weaknesses, pp. 157-180. , Springer, Dordrecht; Doherty, K., Balaskas, A., Doherty, G., The design of ecological momentary assessment technologies (2020) Interact Comput, , https://doi.org/10.1093/iwcomp/iwaa019; Heron, K.E., Smyth, J.M., Ecological momentary interventions: incorporating mobile technology into psychosocial and health behaviour treatments (2010) Br J Health Psychol, 15, pp. 1-39; Heron, K.E., Everhart, R.S., McHale, S.M., Smyth, J.M., Using mobile-technology-based ecological momentary assessment (EMA) methods with youth: a systematic review and recommendations (2017) J Pediatr Psychol, 42, pp. 1087-1107; Degroote, L., Desmet, A., De Bourdeaudhuij, I., Van Dyck, D., Crombez, G., Content validity and methodological considerations in ecological momentary assessment studies on physical activity and sedentary behaviour: a systematic review (2020) Int J Behav Nutr Phys Act, 17, p. 35},
correspondence_address1={Elavsky, S.; Faculty of Education, Czech Republic; email: steriani.elavsky@osu.cz},
publisher={BioMed Central Ltd},
issn={14712458},
pubmed_id={33435943},
language={English},

```
abbrev_source_title={BMC Public Health},
document_type={Article},
source={Scopus},
}

@ARTICLE{Clays2021,
author={Clays, E. and Puddu, P.E. and Luštrek, M. and Pioggia, G. and Derboven, J. and Vrana, M. and De Sutter, J. and Le Donne, R. and Baert, A. and Bohanec, M. and Ciancarelli, M.C. and Dawodu, A.A. and De Pauw, M. and De Smedt, D. and Marino, F. and Pardaens, S. and Schiariti, M.S. and Valič, J. and Vanderheyden, M. and Vodopija, A. and Tartarisco, G.},
title={Proof-of-concept trial results of the HeartMan mobile personal health system for self-management in congestive heart failure},
journal={Scientific Reports},
year={2021},
volume={11},
number={1},
doi={10.1038/s41598-021-84920-4},
art_number={5663},
note={cited By 0},
url={https://www.scopus.com/inward/record.uri?eid=2-s2.0-85102467873&doi=10.1038%2fs41598-021-84920-4&partnerID=40&md5=a069a5dbe95d8b152068c9998ded8b64},
affiliation={Department of Public Health and Primary Care, Ghent University, Campus University Hospital Ghent, entrance 42, 4K3, Corneel Heymanslaan 10, Ghent, 9000, Belgium; Department of Cardiovascular, Respiratory, Nephrological, Anesthesiological and Geriatric Sciences, Sapienza University of Rome, Rome, Italy; Department of Intelligent Systems, Jožef Stefan Institute, Ljubljana, Slovenia; Institute for Biomedical Research and Innovation, Italian National Research Council, Messina, Italy; Meaningful Interactions Lab, KU Leuven, Leuven, Belgium; European Heart Network, Brussels, Belgium; Department of Cardiology, AZ Maria Middelares Ghent, Ghent, Belgium; Department of Internal Medicine, Ghent University, Ghent, Belgium; Complex Operative Unit of Pneumology and Telemedicine, San Camillo de Lellis General Hospital, Rieti, Italy; Department of Knowledge Technologies, Jožef Stefan Institute, Ljubljana, Slovenia; Cardiovascular Center, Onze-Lieve-Vrouw Hospital Aalst, Aalst, Belgium},
abstract={This study tested the effectiveness of HeartMan—a mobile personal health system offering decisional support for management of congestive heart failure (CHF)—on health-related quality of life (HRQoL), self-management, exercise capacity, illness perception, mental and sexual health. A randomized controlled proof-of-concept trial (1:2 ratio of control:intervention) was set up with ambulatory CHF patients in stable condition in Belgium and Italy. Data were collected by means of a 6-min walking test and a number of standardized questionnaire instruments. A total of 56 (34 intervention and 22 control group) participants completed the study (77% male; mean age 63 years, sd 10.5). All depression and anxiety dimensions decreased in the intervention group (p < 0.001), while the need for sexual counselling decreased in the control group (p < 0.05). Although the group differences were not significant, self-care increased (p < 0.05), and sexual problems decreased (p < 0.05) in the intervention group only. No significant intervention effects were observed for HRQoL, self-care confidence, illness perception and exercise capacity. Overall, results of this proof-of-concept trial suggest that the HeartMan personal health system significantly improved mental and sexual health and self-care behaviour in CHF patients. These observations were in contrast to the lack of
```

intervention effects on HRQoL, illness perception and exercise capacity. © 2021, The Author(s).},
references={Ponikowski, P., Heart failure: preventing disease and death worldwide (2014) ESC Heart Failure, 1, pp. 4-25; Ponikowski, P., ESC guidelines for the diagnosis and treatment of acute and chronic heart failure: the task force for the diagnosis and treatment of acute and chronic heart failure of the European Society of Cardiology (ESC). Developed with the special contribution of the Heart Failure Association (HFA) of the ESC (2016) Eur. J. Heart Failure, 18, pp. 891-975; Roger, V.L., Epidemiology of heart failure (2013) Circ. Res., 113, pp. 646-659. , COI: 1:CAS:528:DC%2BC3sXhtlChs7nK; Zannad, F., Clinical outcome endpoints in heart failure trials: a European Society of Cardiology Heart Failure Association consensus document (2013) Eur. J. Heart Fail, 15, pp. 1082-1094; Piepoli, M.F., Exercise training in heart failure: from theory to practice. A consensus document of the Heart Failure Association and the European Association for Cardiovascular Prevention and Rehabilitation (2011) Eur. J. Heart Fail, 13, pp. 347-357; Lainscak, M., Self-care management of heart failure: practical recommendations from the patient care committee of the heart failure association of the European society of cardiology (2011) Eur. J. Heart Fail, 13, pp. 115-126; Chaudhry, S.I., Telemonitoring in patients with heart failure (2010) N. Engl. J. Med., 363, pp. 2301-2309. , COI: 1:CAS:528:DC%2BC3cXhsFGkt7%2FM; Inglis, S.C., Clark, R.A., Dierckx, R., Prieto-Merino, D., Cleland, J.G., Structured telephone support or non - invasive telemonitoring for patients with heart failure (2015) Cochrane Database Syst. Rev.; Koehler, F., Impact of remote telemedical management on mortality and hospitalizations in ambulatory patients with chronic heart failure: the telemedical interventional monitoring in heart failure study (2011) Circulation, 123, pp. 1873-1880; Ong, M.K., Effectiveness of remote patient monitoring after discharge of hospitalized patients with heart failure: the better effectiveness after transition–heart failure (BEAT-HF) randomized clinical trial (2016) JAMA Int. Med., 176, pp. 310-318; Baert, A., A personal decision support system for heart failure management (heartman): study protocol of the heartman randomized controlled trial (2018) BMC Cardiovasc. Disord., 18, p. 186; Anker, S.D., The importance of patient-reported outcomes: a call for their comprehensive integration in cardiovascular clinical trials (2014) Eur. Heart J., 35, pp. 2001-2009; Chamberlain, A.M., Self-rated health predicts healthcare utilization in heart failure (2014) J. Am. Heart Assoc, 3. , PID: 24870937; Monzo, L., Association of patient-reported outcomes and heart rate trends in heart failure: a report from the Chiron project (2020) Sci. Rep., 10, pp. 1-8; Mlakar, M., Mining telemonitored physiological data and patient-reported outcomes of congestive heart failure patients (2018) PLoS ONE, 13; Riegel, B., Lee, C.S., Dickson, V.V., Carlson, B., An update on the self-care of heart failure index (2009) J. Cardiovasc. Nurs., 24, p. 485; Rector, T., Francis, G., Cohn, J., Patients self-assessment of their congestive heart failure Part 1: patient perceived dysfunction and its poor correlation with maximal exercise tests (1987) Heart Failure, 3, pp. 192-196; Broadbent, E., Petrie, K.J., Main, J., Weinman, J., The brief illness perception questionnaire (2006) J. Psychosom. Res., 60, pp. 631-637; Beck, A.T., Steer, R.A., Brown, G.K., Beck depression inventory-II (1996) San Antonio, 78, pp. 490-498; Spielberger, C.D., (1983) State-Trait Anxiety Inventory for Adults; Derogatis, L.R., The psychosocial adjustment to illness scale (PAIS) (1986) J. Psychosom. Res., 30, pp. 77-91. , COI: 1:STN:280:DyaL287pvFyisg%3D%3D; Driel, A.G., de Hosson, M.J.J., Gamel, C., Sexuality of patients with chronic heart failure and their spouses and the need for information regarding sexuality (2014) Eur. J. Cardiovascular Nursing, 13, pp. 227-234; Pocock, S.J.,

Predicting survival in heart failure: a risk score based on 39 372 patients from 30 studies (2013) Eur. Heart J., 34, pp. 1404-1413; Senni, M., Predicting heart failure outcome from cardiac and comorbid conditions: the 3C-HF score (2013) Int. J. Cardiol., 163, pp. 206-211; Monzo, L., Schiariti, M., Puddu, P.E., Wireless Telecardiology (2019) Health Monitoring Systems: An Enabling Technology for Patient Care, , (eds. Gupta, R. & Biswas, D.) (CRC Press; Lin, M.-H., Clinical effectiveness of telemedicine for chronic heart failure: a systematic review and meta-analysis (2017) J. Investig. Med., 65, pp. 899-911; Marschollek, M., Decision support at home (DS@ HOME)–system architectures and requirements (2012) BMC Med. Inform. Decis. Mak., 12, p. 43; Greeson, J.M., Chin, G.R., Mindfulness and physical disease: a concise review (2019) Current Opin. Psychol., 28, pp. 204-210; Norman, J., Fu, M., Ekman, I., Björck, L., Falk, K., Effects of a mindfulness-based intervention on symptoms and signs in chronic heart failure: A feasibility study (2018) Eur. J. Cardiovasc. Nurs., 17, pp. 54-65; Jeyanantham, K., Kotecha, D., Thanki, D., Dekker, R., Lane, D.A., Effects of cognitive behavioural therapy for depression in heart failure patients: a systematic review and meta-analysis (2017) Heart Fail. Rev., 22, pp. 731-741; Kwekkeboom, K.L., Bratzke, L.C., A systematic review of relaxation, meditation, and guided imagery strategies for symptom management in heart failure (2016) J. Cardiovasc. Nurs., 31, p. 457; Lundgren, J., Andersson, G., Johansson, P., Can cognitive behaviour therapy be beneficial for heart failure patients? (2015) Curr. Heart Fail. Rep., 12, pp. 166-172; Corotto, P.S., McCarey, M.M., Adams, S., Khazanie, P., Whellan, D.J., Heart failure patient adherence: epidemiology, cause, and treatment (2013) Heart Fail. Clin, 9, pp. 49-58; Baert, A., Sexual activity in heart failure patients: information needs and association with health-related quality of life (2019) Int. J. Environ. Res. Public Health, 16, p. 1570; Schwarz, E., The prevalence and clinical relevance of sexual dysfunction in women and men with chronic heart failure (2008) Int. J. Impot. Res., 20, pp. 85-91. , COI: 1:STN:280:DC%2BD1c7gtF2gsA%3D%3D; Baert, A., Factors associated with health-related quality of life in stable ambulatory congestive heart failure patients: Systematic review (2018) Eur. J. Prevent. Cardiol., 25, pp. 472-481; Purves, D., (2001) Physiological changes associated with emotion, , Neuroscience2nd, Sinauer Associates; Hannan, A.L., Impact of wearable physical activity monitoring devices with exercise prescription or advice in the maintenance phase of cardiac rehabilitation: systematic review and meta-analysis (2019) BMC Sports Science, Medicine and Rehabilitation, 11, p. 14; Lunde, P., Long-term follow-up with a smartphone application improves exercise capacity post cardiac rehabilitation: A randomized controlled trial (2020) Eur. J. Prevent. Cardiol., , 2047487320905717; Hagger, M.S., Orbell, S., A meta-analytic review of the common-sense model of illness representations (2003) Psychol. Health, 18, pp. 141-184},
correspondence_address1={Clays, E.; Department of Public Health and Primary Care, Campus University Hospital Ghent, entrance 42, 4K3, Corneel Heymanslaan 10, Belgium; email: els.clays@UGent.be},
publisher={Nature Research},
issn={20452322},
pubmed_id={33707523},
language={English},
abbrev_source_title={Sci. Rep.},
document_type={Article},
source={Scopus},
}

@ARTICLE{Strozzi2021,
author={Strozzi, M.M. and Varrica, A. and Colivicchi, M. and Pelazzo, C. and Negri, R. and Galante, A. and Ianniello, P. and Sterpone, R. and Nannini, P. and Leo, D. and Mannarino, F. and Striani, M. and Montani, S. and Gazzolo, D.},
title={High-Fidelity simulation-based program improves flow state scale in the perinatal team},
journal={Italian Journal of Pediatrics},
year={2021},
volume={47},
number={1},
doi={10.1186/s13052-021-00972-2},
art_number={42},
note={cited By 0},
url={https://www.scopus.com/inward/record.uri?eid=2-s2.0-85101709323&doi=10.1186%2fs13052-021-00972-2&partnerID=40&md5=a419ede5af024fd29d652b6d9cdb4451},
affiliation={Neonatal Intensive Care Unit AO S.S. Antonio, Biagio and C. Arrigo Hospital, Alessandria, Italy; Department of Paediatric Cardiovascular Surgery, Laboratory Research, San Donato Milanese University Hospital, San Donato, Italy; Psychology Unit AO S.S. Antonio, Biagio and C. Arrigo Hospital, Alessandria, Italy; Science and Technological Innovation Department, University of Piemonte Orientale, Alessandria, Italy; Neonatal Intensive Care Unit G. D’Annunzio University of Chieti, Chieti, Italy; Department of Maternal, Fetal and Neonatal Medicine, C. Arrigo Children’s Hospital, Alessandria, I-15100, Italy},
abstract={Background: We aimed to evaluate the degree of realism and involvement, stress management and awareness of performance improvement in practitioners taking part in high fidelity simulation (HFS) training program for delivery room (DR) management, by means of a self-report test such as flow state scale (FSS). Methods: This is an observational pretest-test study. Between March 2016 and May 2019, fourty-three practitioners (physicians, midwives, nurses) grouped in multidisciplinary teams were admitted to our training High Fidelity Simulation center. In a time-period of 1 month, practitioners attended two HFS courses (model 1, 2) focusing on DR management and resuscitation maneuvers. FSS test was administred at the end of M1 and M2 course, respectively. Results: FSS scale items such as unambiguous feed-back, loss of self consciousness and loss of time reality, merging of action and awareness significantly improved (P < 0.05, for all) between M1 and M2. Conclusions: The present results showing the high level of practitioner involvement during DR management-based HFS courses support the usefulness of HFS as a trustworthy tool for improving the awareness of practitioner performances and feed-back. The data open the way to the usefulness of FSS as a trustworthy tool for the evaluation of the efficacy of training programs in a multidisciplinary team. © 2021, The Author(s).},
author_keywords={Delivery room; Flow state scale test; Neonatal resuscitation; Simulation based training},
keywords={article; awareness; comparative effectiveness; consciousness; controlled study; delivery room; high fidelity simulation training; human; human experiment; midwife; multidisciplinary team; nurse; physician; resuscitation; self report; stress management},
references={Ersdal, H.L., Mduma, E., Svensen, E., Perlman, J.M., Early initiation of basic resuscitation interventions including face mask ventilation may reduce birth asphyxia related mortality in low-income countries: a prospective descriptive observational study (2012) Resuscitation., 83, pp. 869-887. , PID: 22198423; Wyckoff, M.H., Aziz, K., Escobedo, M.B., Kapadia,

V.S., Kattwinkel, J., Perlman, J.M., Part 13: neonatal resuscitation: 2015 American Heart Association guidelines update for cardiopulmonary resuscitation and emergency cardiovascular care (2015) Circulation., 132, pp. S543-S560. , PID: 26473001; Perlman, J.M., Wyllie, J., Kattwinkel, J., Wyckoff, M.H., Aziz, K., Guinsburg, R., Part 7: neonatal resuscitation: 2015 international consensus on cardiopulmonary resuscitation and emergency cardiovascular care science with treatment recommendations (2015) Circulation., 132, pp. S204-S241. , PID: 26472855; Weiner, G.M., Delfino, A., (2016) Textbook of Neonatal Resuscitation, , editors, American Academy of Pediatrics, Florence; Thomas, E.J., Williams, A.L., Reichman, E.F., Lasky, R.E., Crandell, S., Taggart, W.R., Team training in the neonatal resuscitation program for interns: teamwork and quality of resuscitations (2010) Pediatrics., 125, pp. 539-546. , PID: 20156896; Halamek, L.P., Kaegi, D.M., Gaba, D.M., Sowb, Y.A., Smith, B.C., Smith, B.E., Howard, S.K., Time for a new paradigm in pediatric medical education: teaching neonatal resuscitation in a simulated delivery room environment (2000) Pediatrics., 106, pp. E45-E51. , COI: 1:STN:280:DC%2BD3cvmslalsA%3D%3D, PID: 11015540; Ades, A., Lee, H.C., Update on simulation for the neonatal resuscitation program (2016) Semin Perinatol, 40, pp. 447-454. , PID: 27823817; Pammi, M., Dempsey, M.D., Ryan, C.A., Barrington, K.J., Newborn resuscitation training programmes reduce early neonatal mortality (2016) Neonatology., 110, pp. 210-224. , PID: 27222260; Kohn, L.T., Corrigan, J., Donaldson, M.S., (2000) To Err Is Human: Building A Safer Health System, , National Academies Press, Washington (DC; (2004) Sentinel Event Alert, Issue 30. Preventing Infant Death and Injury during Delivery; 1995–2004, , Washington (DC, Joint Commission on Accreditation of Healthcare Organizations; Manser, T., Teamwork and patient safety in dynamic domains of healthcare: a review of the literature (2009) Acta Anaesthesiol Scand, 53, pp. 143-151. , COI: 1:STN:280:DC%2BD1M7gtFGltQ%3D%3D, PID: 19032571; Dempsey, E., Pammi, M., Ryan, A.C., Barrington, K.J., Standardised formal resuscitation training programmes for reducing mortality and morbidity in newborn infants (2015) Cochrane Database Syst Rev, 9, p. CD009106; Arnold, J., Neonatal resuscitation program comes of age (2011) J Pediatr, 159, pp. 357-358. , PID: 21846521; O'Currain, E., Davis, P.G., Thio, M., Educational perspectives: toward more effective neonatal resuscitation: assessing and improving clinical skills (2019) Neoreviews., 20, pp. e248-e257. , PID: 31261077; Murphy, A., Halamek, L., Simulation-based training in neonatal resuscitation (2005) Neoreviews., 11, pp. e489-e491; Rakshasbhuvankar, A.A., Patole, S.K., Benefits of simulation based training for neonatal resuscitation education: a systematic review (2014) Resuscitation., 85, pp. 1320-1323. , COI: 1:STN:280:DC%2BC2cbktlahug%3D%3D, PID: 25046744; Rubio-Gurung, S., Putet, G., Touzet, S., Gauthier-Moulinier, H., Jordan, I., Beissel, A., In situ simulation training for neonatal resuscitation: an RCT (2014) Pediatrics., 134, pp. e790-e797. , PID: 25092937; Huang, J., Tang, Y., Tang, J., Shi, J., Wang, H., Xiong, T., Educational efficacy of high-fidelity simulation in neonatal resuscitation training: a systematic review and meta-analysis (2019) BMC Med Educ, 19, pp. 323-333. , PID: 31464614; Weis, J.J., Croft, C.L., Bhoja, R., Lee, J.H., Scott, D.J., Multidisciplinary simulation activity effectively prepares residents for participation in patient safety activities (2019) J Surg Educ, 76, pp. e232-e237. , PID: 31488345; Cheng, A., Lockey, A., Bhanji, F., Lin, Y., Hunt, E.A., Lang, E., The use of high-fidelity manikins for advanced life support training--a systematic review and meta-analysis (2015) Resuscitation., 93, pp. 142-149. , PID: 25888241; Crofts, J., Bartlett, C., Ellis, D., Hunt, L., Fox, R., Draycott, T., Training for shoulder dystocia: a trial of simulation using low-fidelity and high-fidelity mannequins (2006) Obstet Gynecol, 108, pp. 1477-1485. , PID: 17138783; Jackson, K.S., The

importance of non-technical skills and risk reduction in the operating theatre (2016) Obstetrician Gynaecol, 18, pp. 309-314; Kunzle, B., Zala-Mezo, E., Wacker, J., Kolbe, M., Spahn, D.R., Grote, G., Leadership in anaesthesia teams: the most effective leadership is shared (2010) Qual Saf Health Care, 19, pp. 1-6; Flin, R., Maran, N., Basic concepts for crew resource management and non-technical skills (2015) Best Pract Res Clin Anaesthesiol, 29, pp. 27-39. , PID: 25902464; Meurling, L., Hedman, L., Lidefelt, K.J., Escher, C., Felländer-Tsai, L., Wallin, C.J., Comparison of high- and low equipment fidelity during paediatric simulation team training: a case control study (2014) BMC Med Educ., 14, pp. 221-228. , PID: 25326794; Lizotte, M.H., Janvier, A., Latraverse, V., Lachance, C., Walker, C.D., Barrington, K.J., Moussa, A., The impact of neonatal simulations on Trainees’ stress and performance: a parallel-group randomized trial (2017) Pediatr Crit Care Med, 18, pp. 434-441. , PID: 28282325; Krage, R., Zwaan, L., Tjon Soei Len, L., Kolenbrander, M.W., van Groeningen, D., Loer, S.A., Relationship between non-technical skills and technical performance during cardiopulmonary resuscitation: does stress have an influence? (2017) Emerg Med J, 34, pp. 728-733. , PID: 28844039; Judd, B.K., Currie, J., Dodds, K.L., Fethney, J., Gordon, C.J., Registered nurses psychophysiological stress and confidence during high-fidelity emergency simulation: effects on performance (2019) Nurse Educ Today, 78, pp. 44-49. , PID: 31071584; Geeraerts, T., Roulleau, P., Cheisson, G., Marhar, F., Aidan, K., Lallali, K., Physiological and self-assessed psychological stress induced by a high fidelity simulation course among third year anesthesia and critical care residents: an observational study (2017) Anaesth Crit Care Pain Med, 36, pp. 403-406. , PID: 28648752; Koehn, S., Effects of confidence and anxiety on flow state in competition (2013) Eur J of Sport Sci, 13, pp. 543-550; Nakamura, J., Csikszentmihalyi, M., (2014) The concept of flow. Flow and the Foundation of Positive Psychology, pp. 239-263; Jackson, S.A., Marsh, H.W., Development and validation of a scale to measure optimal experience: the flow state scale (1996) J Sport Exercise Psychol, 18, pp. 17-35; Jackson, S., Martin, A.J., Eklund, R.C., Long and short measures of flow: the construct validity of the FSS-2. DFS-2, and new brief counterparts (2008) J Sport Exerc Psychol, 30, pp. 561-587. , PID: 18971512; Csikszentmihalyi, M., LeFevre, J., Optimal experience in work and leisure (1989) J Pers Soc Psychol, 56, pp. 815-822. , COI: 1:STN:280:DyaL1M3lvVagtg%3D%3D, PID: 2724069; Edmondson, A., Psychological safety and learning behavior in work teams (1999) Adm Sci Q, 44, pp. 350-383; Anderson, D.R., Crous, A.F., Schepers, J.M., Flow and quality of work life in a diverse workforce (1996) J Indust Psychol, 22, pp. 13-20; Engeser, S., Rheinberg, F., Flow, performance and moderators of challenge-skill balance (2008) Motiv Emot, 32, pp. 158-172; DeLaVega, R., Pérez García, J., Ruiz-Barquín, R., Marquez, S., Flow in firefighters and its relationship with performance in high-risk tasks: preliminar research (2015) Psychologia., 58, pp. 167-175; Salanova, M., Bakker, A.B., Llorensm, S., Flow at work: evidence for an upward spiral of personal and organizational resources (2006) J Happiness Stud, 7, pp. 1-22; Fullagar, C.J., Kelloway, E.K., Flow at work: an experience sampling approach (2009) J Occup Organ Psychol, 82, pp. 595-615; van Den Hout, J.J., Davis, O.C., Team flow: The psychology of optimal collaboration. SpringerBriefs in well-being and quality of life research (2019) Springer Nature Social Sciences, pp. 1-136. , Chapter 3. Senior Publishing Editor; Jackson, S.A., Factors influencing the occurrence of flow state in elite athletes (1995) J Appl Sport Psychol, 7, pp. 138-166; Koehn, S., Morris, T., The relationship between performance and flow state in tennis competition (2012) J Sports Med Phys Fitness, 52, pp. 1-11; Jackson, S.A., Thomas, P.R., Marsh, H.W., Smethurst, C.J., Relationships between flow, self-concept,

psychological skills, and performance (2001) J Appl Sport Psychol, 13, pp. 129-153; Stavrou, N.A., Zervas, Y., Karteroliotis, K., Jackson, S.A., Flow experience and athletes' performance with reference to the orthogonal model of flow (2007) Sport Psychol, 21, pp. 438-457; van den Hout, J.J., Davisb, O.C., Weggeman, M., The conceptualization of team flow (2018) J Psychol, 152, pp. 388-423. , PID: 30089084; Mannella, P., Palla, G., Cuttano, A., Boldrini, A., Simoncini, T., Effect of high-fidelity shoulder dystocia simulation on emergency obstetric skills and crew resource management skills among residents (2016) Int J Gynaecol Obstet, 135, pp. 338-342. , PID: 27622684; Bracco, F., de Tonetti, G., Masini, M., Passarelli, M., Geretto, F., Celleno, D., Crisis resource Management in the Delivery Room: development of behavioral markers for team performance in emergency simulation (2018) Int J Environ Res Public Health, 15, pp. 439-449; Dadiz, R., Weinschreider, J., Schriefer, J., Arnold, C., Greves, C.D., Crosby, E.C., Wang, H., Guillet, R., Interdisciplinary simulation-based training to improve delivery room communication (2013) Simul Healthc, 8, pp. 279-291. , PID: 23842120; Montani, S., Canonico, M., Gazzolo, D., Strozzi, M.C., Striani, M., TEEM: technology-enhanced emergency Management for Supporting Data Communication during Patient Transportation (2017) Int J Mob Comp Mult Comm, 8, pp. 49-65},
correspondence_address1={Gazzolo, D.; Department of Maternal, Italy; email: dgazzolo@hotmail.com},
publisher={BioMed Central Ltd},
issn={17208424},
coden={IJPTB},
pubmed_id={33632265},
language={English},
abbrev_source_title={Ital. J. Pediatr.},
document_type={Article},
source={Scopus},
}

@ARTICLE{Page-Reeves2021,
author={Page-Reeves, J. and Murray-Krezan, C. and Regino, L. and Perez, J. and Bleecker, M. and Perez, D. and Wagner, B. and Tigert, S. and Bearer, E.L. and Willging, C.E.},
title={A randomized control trial to test a peer support group approach for reducing social isolation and depression among female Mexican immigrants},
journal={BMC Public Health},
year={2021},
volume={21},
number={1},
doi={10.1186/s12889-020-09867-z},
art_number={119},
note={cited By 0},
url={https://www.scopus.com/inward/record.uri?eid=2-s2.0-85099236925&doi=10.1186%2fs12889-020-09867-z&partnerID=40&md5=c712797e524ed8414cbf4904d2124854},
affiliation={University of New Mexico, Albuquerque, New Mexico, United States; Centro Sávila, Albuquerque, New Mexico, United States; Pacific Institute for Research & Evaluation, Albuquerque, New Mexico, United States},
abstract={Background: Female Mexican Immigrants (FMIs) experience high rates of depression compared with other populations. For this population, depression is often exacerbated by social isolation associated with the experience of immigration. Aim 1. To measure whether a culturally situated peer group intervention will reduce depression and stress associated with the experience

of immigration. Aim 2. To test whether an intervention using a “women’s funds of knowledge” approach results in improved resilience, knowledge and empowerment. Aim 3. To investigate whether a culturally situated peer group intervention using a women’s funds of knowledge approach can give participants a sense and experience of social and physical connection (“emplacement”) that is lost in the process of immigration. Methods: This mixed-methods study will implement “Tertulias” (“conversational gatherings” in Spanish), a peer support group intervention designed to improve health outcomes for FMI participants in Albuquerque, New Mexico. We will document results of the intervention on our primary hypotheses of a decrease in depression, and increases in resilience and social support, as well as on our secondary hypotheses of decreased stress (including testing of hair cortisol as a biomarker for chronic stress), and an increase in social connectedness and positive assessment of knowledge and empowerment. Discussion: This project will address mental health disparities in an underserved population that experiences high rates of social isolation. Successful completion of this project will demonstrate that health challenges that may appear too complex and too hard to address can be using a multi-level, holistic approach. Our use of hair samples to test for the 3-month average levels of systemic cortisol will contribute to the literature on an emerging biomarker for analyzing chronic stress. Trial registration: This study was registered with ClinicalTrials.gov on 2/3/20, Identifier #NCT04254198. © 2021, The Author(s).},
author_keywords={Depression; Mexican immigrant; Peer support group; Social isolation; Women},
references={Cruz, F.C., Duarte, J.O., Leão, R.M., Hummel, L.F.V., Planeta, C.S., Crestani, C.C., Adolescent vulnerability to cardiovascular consequences of chronic social stress: Immediate and long-term effects of social isolation during adolescence (2016) Dev Neurobiol, 76 (1), pp. 34-46. , COI: 1:CAS:528:DC%2BC2MXitVagsLzP, PID: 25914339; Chow, P.I., Fua, K., Huang, Y., Bonelli, W., Xiong, H., Barnes, L.E., Using Mobile Sensing to Test Clinical Models of Depression, Social Anxiety, State Affect, and Social Isolation Among College Students (2017) J Med Internet Res, 19 (3). , PID: 28258049; Pedersen, P.V., Andersen, P.T., Curtis, T., Social relations and experiences of social isolation among socially marginalized people (2012) J Soc Pers Relat, 29 (6), pp. 839-858; Turner, R.J., Social support as a contingency in psychological well-being (1981) J Health Soc Behav, pp. 357-367; D’Arcy, C., Siddique, C.M., Social support and mental health among mothers of preschool and school age children (1984) Soc Psychiatry, 19 (4), pp. 155-162. , PID: 6494984; Wethington, E., Kessler, R.C., Perceived Support, Received Support, and Adjustment to Stressful Life Events (1986) J Health Soc Behav, 27 (1), pp. 78-89. , COI: 1:STN:280:DyaL283isFeiuw%3D%3D, PID: 3711634; Kuo, W.H., Tsai, Y.M., Social networking, hardiness and immigrant’s mental health (1986) J Health Soc Behav, 27 (2), pp. 133-149. , COI: 1:STN:280:DyaL283nvFCktA%3D%3D, PID: 3525660; Miller, D.K., Malmstrom, T.K., Joshi, S., Andresen, E.M., Morley, J.E., Wolinsky, F.D., Clinically relevant levels of depressive symptoms in community-dwelling middle-aged African Americans (2004) J Am Geriatr Soc, 52 (5), pp. 741-748. , PID: 15086655; Kawachi, I., Colditz, G.A., Ascherio, A., Rimm, E.B., Giovannucci, E., Stampfer, M.J., A prospective study of social networks in relation to total mortality and cardiovascular disease in men in the USA (1996) J Epidemiol Community Health, 50 (3), pp. 245-251. , COI: 1:STN:280:DyaK2s%2FovFGntw%3D%3D, PID: 8935453; Murberg, T.A., Long-term effect of social relationships on mortality in patients with congestive heart failure (2004) Int J Psychiatry Med, 34 (3), pp. 207-217. , PID: 15666956; Valtorta, N.K., Kanaan, M., Gilbody, S., Ronzi, S., Hanratty, B., Loneliness and social

isolation as risk factors for coronary heart disease and stroke: systematic review and meta-analysis of longitudinal observational studies (2016) Heart, 102 (13), pp. 1009-1016. , COI: 1:CAS:528:DC%2BC2sXitVWkt7zF, PID: 27091846; Burdette, H.L., Wadden, T.A., Whitaker, R.C., Neighborhood safety, collective efficacy, and obesity in women with young children (2006) Obesity (Silver Spring), 14 (3), pp. 518-525; Berkman, L.F., Syme, S.L., Social networks, host resistance, and mortality: a nine-year follow-up study of Alameda County residents (1979) Am J Epidemiol, 109 (2), pp. 186-204. , COI: 1:STN:280:DyaE1M7kt1CisQ%3D%3D, PID: 425958; House, J.S., Robbins, C., Metzner, H.L., The association of social relationships and activities with mortality: prospective evidence from the Tecumseh Community Health Study (1982) Am J Epidemiol, 116 (1), pp. 123-140. , COI: 1:STN:280:DyaL383ltlyjtA%3D%3D, PID: 7102648; Taylor, H.O., Taylor, R.J., Nguyen, A.W., Chatters, L., Social Isolation, Depression, and Psychological Distress Among Older Adults (2018) J Aging Health, 30 (2), pp. 229-246. , PID: 28553785; Marcus, A.F., Illescas, A.H., Hohl, B.C., Llanos, A.A.M., Relationships between social isolation, neighborhood poverty, and cancer mortality in a population-based study of US adults (2017) PLoS One, 12 (3). , COI: 1:CAS:528:DC%2BC1cXns12l; Holt-Lunstad, J., Smith, T.B., Baker, M., Harris, T., Stephenson, D., Loneliness and social isolation as risk factors for mortality: a meta-analytic review (2015) Perspect Psychol Sci, 10 (2), pp. 227-237. , PID: 25910392; Pantell, M., Rehkopf, D., Jutte, D., Syme, S.L., Balmes, J., Adler, N., Social isolation: a predictor of mortality comparable to traditional clinical risk factors (2013) Am J Public Health, 103 (11), pp. 2056-2062. , PID: 24028260; Holt-Lunstad, J., The Potential Public Health Relevance of Social Isolation and Loneliness: Prevalence, Epidemiology, and Risk Factors (2017) Public Policy Aging Rep, 27 (4), pp. 127-130; Cacioppo, J.T., Cacioppo, S., Social Relationships and Health: The Toxic Effects of Perceived Social Isolation (2014) Soc Personal Psychol Compass, 8 (2), pp. 58-72. , PID: 24839458; Steptoe, A., Shankar, A., Demakakos, P., Wardle, J., Social isolation, loneliness, and all-cause mortality in older men and women (2013) Proc Natl Acad Sci U S A, 110 (15), pp. 5797-5801. , COI: 1:CAS:528:DC%2BC3sXnsFKksL8%3D, PID: 23530191; Tomaka, J., Thompson, S., Palacios, R., The relation of social isolation, loneliness, and social support to disease outcomes among the elderly (2006) J Aging Health, 18 (3), pp. 359-384. , PID: 16648391; Friedmann, E., Thomas, S.A., Liu, F., Morton, P.G., Chapa, D., Gottlieb, S.S., Relationship of depression, anxiety, and social isolation to chronic heart failure outpatient mortality (2006) Am Heart J, 152 (5), pp. 940.e1-940.e8; Hawthorne, G., Perceived social isolation in a community sample: its prevalence and correlates with aspects of peoples' lives (2008) Soc Psychiatry Psychiatr Epidemiol, 43 (2), pp. 140-150. , PID: 17994175; Cacioppo, J.T., Cacioppo, S., Capitanio, J.P., Cole, S.W., The neuroendocrinology of social isolation (2015) Annu Rev Psychol, 66, pp. 733-767. , PID: 25148851; Shekunov, J., Immigration and Risk of Psychiatric Disorders: A Review of Existing Literature (2016) Am J Psychiatr Residents J, 11 (2), pp. 3-5; Hurtado-de-Mendoza, A., Gonzales, F.A., Serrano, A., Kaltman, S., Social isolation and perceived barriers to establishing social networks among Latina immigrants (2014) Am J Community Psychol, 53 (1-2), pp. 73-82. , PID: 24402726; Stewart, M., Anderson, J., Beiser, M., Mwakarimba, E., Neufeld, A., Simich, L., Multicultural Meanings of Social Support among Immigrants and Refugees (2008) Int Migr, 46 (3), pp. 123-159; Bekteshi, V., Xu, Q., Van, T.T., Psychological distress among low-income U.S.- and foreign-born women of Mexican descent: impact of acculturation (2015) Womens Health Issues, 25 (2), pp. 185-192. , PID: 25636382; Breslau, J., Borges, G., Tancredi, D., Saito, N., Kravitz, R., Hinton, L., Migration from Mexico to the United States and

subsequent risk for depressive and anxiety disorders: a cross-national study (2011) Arch Gen Psychiatry, 68 (4), pp. 428-433. , PID: 21464367; Franzini, L., Ribble, J.C., Keddie, A.M., Understanding the Hispanic paradox (2001) Ethn Dis, 11 (3), pp. 496-518. , COI: 1:STN:280:DC%2BD3MrisVylug%3D%3D, PID: 11572416; Potochnick, S.R., Perreira, K.M., Depression and Anxiety among First-Generation Immigrant Latino Youth: Key Correlates and Implications for Future Research (2010) J Nerv Ment Dis, 198 (7), pp. 470-477. , PID: 20611049; Merrill, S., (2009) Introduction to Syndemics: A Critical Systems Approach to Public and Community Health: Wiley, , http://www.myilibrary.com?id=218835&ref=toc.Cited2018Sep25; Sharma, T., Syndemics: health in context (2017) Lancet, 389 (10072), p. 881; Singer, M., Bulled, N., Ostrach, B., Mendenhall, E., Syndemics and the biosocial conception of health (2017) Lancet, 389 (10072), pp. 941-950. , PID: 28271845; (2014) I Took the Lemons and I Made Lemonade: Women’s Quotidian Strategies and the Re-Contouring of Food Insecurity in a Hispanic Community in New Mexico. In: Women Redefining the Experience of Food Insecurity, pp. 85-104. , Page-Reeves J, Life off the edge of the table, Lexington Books of Rowman & Littlefield; Page-Reeves, J., Anixter Scott, A., Moffett, M., Apodaca, V., Apodaca, V., Is always that sense of wanting … never really being satisfied”: Women’s Quotidian Struggles With Food Insecurity in a Hispanic Community in New Mexico (2014) J Hunger Environ Nutr, 9 (2), pp. 183-209. , PID: 27570572; Page-Reeves, J., Bleecker, M., Moffett, M.L., Linder, K., Romero, J., Krause, C., Situating food insecurity in a historic Albuquerque community: The whorled relationship between health and place (2017) J Health Disparities Res Pract.; Viruell-Fuentes, E.A., Schulz, A.J., Toward a Dynamic Conceptualization of Social Ties and Context: Implications for Understanding Immigrant and Latino Health (2009) Am J Public Health, 99 (12), pp. 2167-2175. , PID: 19833986; Hiott, A., Grzywacz, J.G., Arcury, T.A., Quandt, S.A., Gender Differences in Anxiety and Depression Among Immigrant Latinos (2006) Fam Syst Health, 24 (2), pp. 137-146. , PID: 31656394; Alegria, M., Shrout, P.E., Woo, M., Guarnaccia, P., Sribney, W., Vila, D., Understanding differences in past year psychiatric disorders for Latinos living in the US (2007) Soc Sci Med, 65 (2), pp. 214-230. , PID: 17499899; Shattell, M.M., Smith, K.M., Quinlan-Colwell, A., Villalba, J.A., Factors contributing to depression in latinas of mexican origin residing in the United States: implications for nurses (2008) J Am Psychiatr Nurses Assoc, 14 (3), pp. 193-204. , PID: 21665765; Viruell-Fuentes, E.A., Andrade, F.C.D., Testing Immigrant Social Ties Explanations for Latino Health Paradoxes: The Case of Social Support and Depression Symptoms (2016) J Latino/Latin Am Stud, 8 (1), pp. 77-92; Vega, W., Kolody, B., Valle, R., Weir, J., Social Networks, Social Support, and their Relationship to Depression among Immigrant Mexican Women (1991) Hum Organ, 50 (2), pp. 154-162; Goodman, R.D., Vesely, C.K., Letiecq, B., Cleaveland, C.L., Trauma and Resilience Among Refugee and Undocumented Immigrant Women (2017) J Couns Dev, 95 (3), pp. 309-321; Shattell, L., Vega, W., Alegria, M., Latino mental health treatment in the United States (2001) Health Issues in the Latino Community, pp. 179-208. , Jossey-Bass, San Francisco; De Oliveira, G., Cianelli, R., Gattamorta, K., Kowalski, N., Peragallo, N., Social Determinants of Depression Among Hispanic Women (2017) J Am Psychiatr Nurses Assoc, 23 (1), pp. 28-36. , PID: 27624770; McNaughton, D.B., Cowell, J.M., Gross, D., Fogg, L., Ailey, S.H., The relationship between maternal and child mental health in Mexican immigrant families (2004) Res Theor Nurs Pract, 18 (2-3), pp. 229-242; Lewis-Fernández, R., Das, A.K., Alfonso, C., Weissman, M.M., Olfson, M., Depression in US Hispanics: diagnostic and management considerations in family practice (2005) J Am Board Fam Pract, 18 (4), pp. 282-296. , PID: 15994474; Page-Reeves, J., Shrum, S., Rohan-Minjares,

F., Thiedeman, T., Perez, J., Murrietta, A., Addressing Syndemic Health Disparities Among Latin Immigrants Using Peer Support (2019) J Racial Ethn Health Disparities, 6 (2), pp. 380-392. , PID: 30506311; Belliveau, M., Gendered Matters: Undocumented Mexican Mothers in the Current Policy Context (2011) Affilia., 26 (1), pp. 32-46; Meadows, L.M., Thurston, W.E., Melton, C., Immigrant women’s health (2001) Soc Sci Med, 52 (9), pp. 1451-1458. , COI: 1:STN:280:DC%2BD3MzisVCrtw%3D%3D, PID: 11286368; Sanchez-Birkhead, A.C., Kennedy, H.P., Callister, L.C., Miyamoto, T.P., Navigating a New Health Culture: Experiences of Immigrant Hispanic Women (2011) J Immigr Minor Health, 13 (6), pp. 1168-1174. , PID: 20607608; Hagan, J.M., Social Networks, Gender, and Immigrant Incorporation: Resources and Constraints (1998) Am Sociol Rev, 63 (1), pp. 55-67; Trickett, E.J., Multilevel community-based culturally situated interventions and community impact: an ecological perspective (2009) Am J Community Psychol, 43 (3-4), pp. 257-266. , PID: 19333751; Trickett, E.J., Schensul, J.J., Summary comments: multi-level community based culturally situated interventions (2009) Am J Community Psychol, 43 (3-4), pp. 377-381. , PID: 19326207; Trickett, E.J., Beehler, S., Deutsch, C., Green, L.W., Hawe, P., McLeroy, K., Advancing the science of community-level interventions (2011) Am J Public Health, 101 (8), pp. 1410-1419. , PID: 21680923; Moll, L.C., Amanti, C., Neff, D., Gonzalez, N., Funds of knowledge for teaching: Using a qualitative approach to connect homes and classrooms (1992) Theory Pract, 31 (2), pp. 132-141; Moll, L.C., Soto-Santiago, S.L., Schwartz, L., Funds of Knowledge in Changing Communities (2013) International Handbook of Research on Children’s Literacy, Learning, and Culture, pp. 172-183. , https://onlinelibrary.wiley.com/doi/abs/10.1002/9781118323342.ch13, Wiley-Blackwell, Available from, Cited 2018 Sep 24; González, N., Wyman, L., O’Connor, B.H., The Past, Present, and Future of “Funds of Knowledge (2011) A Companion to the Anthropology of Education: Wiley, pp. 479-494. , https://onlinelibrary.wiley.com/doi/abs/10.1002/9781444396713.ch28; Fullagar, S., O’Brien, W., Rethinking women’s experiences of depression and recovery as emplacement: Spatiality, care and gender relations in rural Australia (2018) J Rural Stud, 58, pp. 12-19; Pink, S., From embodiment to emplacement: re-thinking competing bodies, senses and spatialities (2017) Physical Culture, Ethnography and the Body, , Routledge, New York; Morin, K.M., Berg, L.D., Emplacing Current Trends in Feminist Historical Geography (1999) Gend Place Cult, 6 (4), pp. 311-330; Evans, J., Davies, B., New directions, new questions? Social theory, education and embodiment (2011) Sport Educ Soc, 16 (3), pp. 263-278; Raubal, M., Jacquez, G., Wilson, J., Kuhn, W., Synthesizing population, health, and place (2013) J Spat Inf Sci, 2013 (7), pp. 103-108; Ussher, J., Kirsten, L., Butow, P., Sandoval, M., What do cancer support groups provide which other supportive relationships do not? The experience of peer support groups for people with cancer (2006) Soc Sci Med, 62 (10), pp. 2565-2576. , PID: 16303220; Hoddinott, P., Chalmers, M., Pill, R., One-to-one or group-based peer support for breastfeeding? Women’s perceptions of a breastfeeding peer coaching intervention (2006) Birth., 33 (2), pp. 139-146. , PID: 16732780; Dennis, C.-L., Peer support within a health care context: a concept analysis (2003) Int J Nurs Stud, 40 (3), pp. 321-332. , PID: 12605954; Mead, S., Hilton, D., Curtis, L., Peer support: a theoretical perspective (2001) Psychiatr Rehabil J, 25 (2), pp. 134-141. , COI: 1:STN:280:DC%2BD38%2Fkt1yqtg%3D%3D, PID: 11769979; Hurdle, D.E., Social support: a critical factor in women’s health and health promotion (2001) Health Soc Work, 26 (2), pp. 72-79. , COI: 1:STN:280:DC%2BD3M3psVynsQ%3D%3D, PID: 11379000; Aranda, M.P., Castaneda, I., Lee, P.-J., Sobel, E., Stress, social support, and coping as predictors of depressive symptoms: Gender differences

among Mexican Americans (2001) Soc Work Res, 25 (1), pp. 37-48; Marsiglia, F.F., Kulis, S., Perez, H.G., Parsai, M., Hopelessness, Family Stress, and Depression among Mexican-heritage Mothers in the Southwest (2011) Health Soc Work, 36 (1), pp. 7-18. , PID: 21446605; Razack, S., Story-telling for Social Change (1993) Gend Educ, 5 (1), pp. 55-70; Plummer, K., (2002) Telling Sexual Stories : Power, Change and Social Worlds: Routledge, , https://www.taylorfrancis.com/books/9781134850969, Cited 2018 Sep 26; Radloff, L.S., (1977) The CES-D Scale: A self-report depression scale for research in the general population, , http://conservancy.umn.edu/handle/11299/98561, Available from: http://conservancy.umn.edu/handle/11299/98561. Cited 2018 Sep 28; Orme, J.G., Reis, J., Herz, E.J., Factorial and discriminant validity of the Center for Epidemiological Studies Depression (CES-D) scale (1986) J Clin Psychol, 42 (1), pp. 28-33. , COI: 1:STN:280:DyaL287ktVOruw%3D%3D, PID: 3950011; Connor, K.M., Davidson, J.R.T., Development of a new resilience scale: the Connor-Davidson Resilience Scale (CD-RISC) (2003) Depress Anxiety, 18 (2), pp. 76-82. , PID: 12964174; Smith, T., Marsden, P., Hout, M., Kim, J., General Social Surveys, 1972-2014, , http://gss.norc.org, Chicago: NORC at the University of Chicago. Available from; Freeman, L.C., Centrality in social networks conceptual clarification (1978) Soc Networks, 1 (3), pp. 215-239; Cohen, S., Kamarck, T., Mermelstein, R., A global measure of perceived stress (1983) J Health Soc Behav, 24 (4), pp. 385-396. , COI: 1:STN:280:DyaL2c7js12iug%3D%3D, PID: 6668417; Meyer, J.S., Novak, M.A., Minireview: Hair Cortisol: A Novel Biomarker of Hypothalamic-Pituitary-Adrenocortical Activity (2012) Endocrinology., 153 (9), pp. 4120-4127. , COI: 1:CAS:528:DC%2BC38Xhtlagt7fN, PID: 22778226; Russell, E., Koren, G., Rieder, M., Van Uum, S., Hair cortisol as a biological marker of chronic stress: current status, future directions and unanswered questions (2012) Psychoneuroendocrinology., 37 (5), pp. 589-601. , COI: 1:CAS:528:DC%2BC38Xkt1CkurY%3D, PID: 21974976; Sierau, S., Glaesmer, H., Klucken, T., Stalder, T., Hair cortisol, lifetime traumatic experiences and psychopathology in unaccompanied refugee minors (2019) Psychoneuroendocrinology., 104, pp. 191-194. , COI: 1:CAS:528:DC%2BC1MXkslars7s%3D, PID: 30856425; Wester, V.L., van Rossu, E.F.C., Clinical applications of cortisol measurements in hair (2015) Eur J Endocrinol, 173 (4), pp. M1-M10. , COI: 1:CAS:528:DC%2BC2MXhslWqtrjM, PID: 25924811; Wright, K.D., Ford, J.L., Perazzo, J., Jones, L.M., Mahari, S., Sullenbarger, B.A., Collecting Hair Samples for Hair Cortisol Analysis in African Americans (2018) JoVE, (136); Yamada, J., Stevens, B., de Silva, N., Gibbins, S., Beyene, J., Taddio, A., Hair cortisol as a potential biologic marker of chronic stress in hospitalized neonates (2007) Neonatology., 92 (1), pp. 42-49. , COI: 1:CAS:528:DC%2BD2sXntVOlsrs%3D, PID: 17356304; Goodman, L.A., Sullivan, C.M., Serrata, J., Perilla, J., Wilson, J.M., Fauci, J.E., Development and validation of the Trauma-Informed Practice Scales (2016) J Commun Psychol, 44 (6), pp. 747-764; Sullivan, C.M., Goodman, L., A Guide for Using the Trauma-Informed Practice (TIP) Scales, p. 30; Beeber, L.S., Holditch-Davis, D., Perreira, K., Schwartz, T.A., Lewis, V., Blanchard, H., Short-term in-home intervention reduces depressive symptoms in Early Head Start Latina mothers of infants and toddlers (2010) Res Nurs Health, 33 (1), pp. 60-76. , PID: 20043296; Tran, A.N., Ornelas, I.J., Kim, M., Perez, G., Green, M., Lyn, M.J., Results from a pilot promotora program to reduce depression and stress among immigrant Latinas (2014) Health Promot Pract, 15 (3), pp. 365-372. , PID: 24334543; Steinhardt, M., Dolbier, C., Evaluation of a resilience intervention to enhance coping strategies and protective factors and decrease symptomatology (2008) J Am Coll Heal, 56 (4), pp. 445-453; Davidson, J., Baldwin, D., Stein, D.J., Kuper, E.,

Benattia, I., Ahmed, S., Treatment of posttraumatic stress disorder with venlafaxine extended release: a 6-month randomized controlled trial (2006) Arch Gen Psychiatry, 63 (10), pp. 1158-1165. , COI: 1:CAS:528:DC%2BD28XhtFKhtr3N, PID: 17015818; Burton, L.E., Qeadan, F., Burge, M., Efficacy of Equine Assisted Psychotherapy in Veterans with Post-Traumatic Stress Disorder (2018) J Integrat Med, p. 16. , (In Press); Mitschke, D., Aguirre, R., Sharma, B., Common Threads: Improving the Mental Health of Bhutanese Refugee Women Through Shared Learning (2013) Soc Work Ment Health, 11 (3), pp. 249-266; Carlson, L.E., Tamagawa, R., Stephen, J., Drysdale, E., Zhong, L., Speca, M., Randomized-controlled trial of mindfulness-based cancer recovery versus supportive expressive group therapy among distressed breast cancer survivors (MINDSET): long-term follow-up results (2016) Psychooncology., 25 (7), pp. 750-759. , PID: 27193737; Carlson, L.E., Doll, R., Stephen, J., Faris, P., Tamagawa, R., Drysdale, E., Randomized controlled trial of Mindfulness-based cancer recovery versus supportive expressive group therapy for distressed survivors of breast cancer (2013) J Clin Oncol, 31 (25), pp. 3119-3126. , PID: 23918953; Tlapoyawa, K., (2020) Explaining the concept of randomization to Spanish-speaking potential clinical trial participants: A short video and video user guide, , https://www.youtube.com/watch?v=layi60vSQNQ, Available from: https://www.youtube.com/watch?v=layi60vSQNQ; (2014) The R Foundation for Statistical Computing, , https://www.r-project.org/, Cited 2016 Feb 2; Harris, P.A., Taylor, R., Thielke, R., Payne, J., Gonzalez, N., Conde, J.G., Research electronic data capture (REDCap)—a metadata-driven methodology and workflow process for providing translational research informatics support (2009) J Biomed Inform, 42 (2), pp. 377-381. , PID: 18929686; Popp, L., Schneider, S., Attention placebo control in randomized controlled trials of psychosocial interventions: theory and practice (2015) Trials., 16. , PID: 25872619; McEwen, B.S., Stellar, E., Stress and the individual. Mechanisms leading to disease (1993) Arch Intern Med, 153 (18), pp. 2093-2101. , COI: 1:STN:280:DyaK3szptleksA%3D%3D, PID: 8379800; Selye, H., (2013) Stress in Health and Disease: Butterworth-Heinemann, p. 1301; Schneiderman, N., Ironson, G., Siegel, S.D., STRESS AND HEALTH: Psychological, Behavioral, and Biological Determinants (2005) Annu Rev Clin Psychol, 1, pp. 607-628. , PID: 17716101; Shern, D.L., Blanch, A.K., Steverman, S.M., Toxic stress, behavioral health, and the next major era in public health (2016) Am J Orthop, 86 (2), pp. 109-123; Stone, A.A., Bachrach, C.A., Jobe, J.B., Kurtzman, H.S., Cain, V.S., (1999) The Science of Self-Report: Implications for Research and Practice, p. 393. , Psychology Press; Bearer, E., Ji, J., Trickett, P., Kaplan, C., Mennen, F., Towards a Role for Clinical Pathology Diagnostics for Childhood Maltreatment (2015) Austin J Clin Pathol, 2 (2). , https://www.ncbi.nlm.nih.gov/pmc/articles/PMC4667746/.Cited2020, Available from; Dettenborn, L., Tietze, A., Bruckner, F., Kirschbaum, C., Higher cortisol content in hair among long-term unemployed individuals compared to controls (2010) Psychoneuroendocrinology., 35 (9), pp. 1404-1409. , COI: 1:CAS:528:DC%2BC3cXhtFOntb3N, PID: 20471757; Gow, R., Thomson, S., Rieder, M., Van Uum, S., Koren, G., An assessment of cortisol analysis in hair and its clinical applications (2010) Forensic Sci Int, 196 (1), pp. 32-37. , COI: 1:CAS:528:DC%2BC3cXit1KitrY%3D, PID: 20096513; Ehlert, U., Enduring psychobiological effects of childhood adversity (2013) Psychoneuroendocrinology., 38 (9), pp. 1850-1857. , PID: 23850228; Gordis, E.B., Granger, D.A., Susman, E.J., Trickett, P.K., Salivary alpha amylase-cortisol asymmetry in maltreated youth (2008) Horm Behav, 53 (1), pp. 96-103. , COI: 1:CAS:528:DC%2BD1cXht1ylsQ%3D%3D, PID: 17945232; Saxbe, D.E., Negriff, S., Susman, E.J., Trickett, P.K., Attenuated

hypothalamic-pituitary-adrenal axis functioning predicts accelerated pubertal development in girls 1 year later (2015) Dev Psychopathol, 27 (3), pp. 819-828. , PID: 25154521; Trickett, P.K., Noll, J.G., Susman, E.J., Shenk, C.E., Putnam, F.W., Attenuation of cortisol across development for victims of sexual abuse (2010) Dev Psychopathol, 22 (1), pp. 165-175. , PID: 20102654; Staufenbiel, S.M., Penninx, B.W.J.H., Spijker, A.T., Elzinga, B.M., van Rossum, E.F.C., Hair cortisol, stress exposure, and mental health in humans: a systematic review (2013) Psychoneuroendocrinology., 38 (8), pp. 1220-1235. , COI: 1:CAS:528:DC%2BC38XhvVCrsr7E, PID: 23253896; Boyle, S.H., Surwit, R.S., Georgiades, A., Brummett, B.H., Helms, M.J., Williams, R.B., Depressive symptoms, race, and glucose concentrations: the role of cortisol as mediator (2007) Diabetes Care, 30 (10), pp. 2484-2488. , COI: 1:CAS:528:DC%2BD2sXht1CltrjM, PID: 17630268; Hinkelmann, K., Muhtz, C., Dettenborn, L., Agorastos, A., Wingenfeld, K., Spitzer, C., Association between childhood trauma and low hair cortisol in depressed patients and healthy control subjects (2013) Biol Psychiatry, 74 (9), pp. 15-17. , COI: 1:CAS:528:DC%2BC3sXovVSlu78%3D; Noppe, G., Van Rossum, E.F.C., Koper, J.W., Manenschijn, L., Bruining, G.J., de Rijke, Y.B., Validation and reference ranges of hair cortisol measurement in healthy children (2014) Horm Res Paediatr, 82 (2), pp. 97-102. , COI: 1:CAS:528:DC%2BC2cXhsVOisbfM, PID: 25115629; Vliegenthart, J., Noppe, G., van Rossum, E.F.C., Koper, J.W., Raat, H., van den Akker, E.L.T., Socioeconomic status in children is associated with hair cortisol levels as a biological measure of chronic stress (2016) Psychoneuroendocrinology., 65, pp. 9-14. , COI: 1:CAS:528:DC%2BC2MXitVGhur7E, PID: 26708067; Woodruff, S.I., Conway, T.L., Edwards, C.C., Hovell, M.F., Acceptability and validity of hair collection from Latino children to assess exposure to environmental tobacco smoke (2003) Nicotine Tob Res, 5 (3), pp. 375-385. , COI: 1:CAS:528:DC%2BD3sXlt1GhsLY%3D, PID: 12791534; Koerber, A., McMichael, L., Qualitative Sampling Methods (2008) J Bus Tech Commun, 22 (4), pp. 454-473; Campbell, K.S., Collecting Information: Qualitative Research Methods for Solving Workplace Problems (1999) Tech Commun, 46 (4), pp. 532-545; Coyne, I.T., Sampling in qualitative research. Purposeful and theoretical sampling; merging or clear boundaries? (1997) J Adv Nurs, 26 (3), pp. 623-630. , COI: 1:STN:280:DyaK1c%2FitVylsA%3D%3D, PID: 9378886; Borgatti, S., Everett, M., Freeman, L.C., Ucinet for Windows: Software for Social Network Analysis, , . Harvard: Analytic Technologies; Martyn, H., (2008) Questioning Qualitative Inquiry: Critical Essays, , Sage Publications; Gläser, J., Laudel, G., Life with and without coding: Two methods for early-stage data analysis in qualitative research aiming at causal explanations (2013) Forum Qualitative Sozialforschung / Forum: Qualitative Social Research, 14 (2). , http://www.qualitative-research.net/index.php/fqs/article/view/1886, Available from:, Cited 2017 Oct 22; Perry, K., Constant comparison (2003) The SAGE Encyclopedia of Social Science Research Methods, pp. 181-182. , Sage Publications, Thousand Oaks; Centers for Disease Control and Prevention (CDC) Policy Analytical Framework, , https://www.cdc.gov/policy/analysis/process/analysis.html},
correspondence_address1={Page-Reeves, J.; University of New MexicoUnited States; email: JPage-Reeves@salud.unm.edu},
publisher={BioMed Central Ltd},
issn={14712458},
pubmed_id={33430845},
language={English},
abbrev_source_title={BMC Public Health},
document_type={Article},

```
source={Scopus},
}

@ARTICLE{Zuccolo2021,
author={Zuccolo, P.F. and Xavier, M.O. and Matijasevich, A. and Polanczyk, G. and Fatori, D.},
title={A smartphone-assisted brief online cognitive-behavioral intervention for pregnant women with depression: a study protocol of a randomized controlled trial},
journal={Trials},
year={2021},
volume={22},
number={1},
doi={10.1186/s13063-021-05179-8},
art_number={227},
note={cited By 0},
url={https://www.scopus.com/inward/record.uri?eid=2-s2.0-85103226290&doi=10.1186%2fs13063-021-05179-8&partnerID=40&md5=31bd4e6315b45fc302c6c60445338252},
affiliation={Department of Psychiatry, University of Sao Paulo Medical School, Sao Paulo, Brazil; Department of Social Medicine, Post-Graduate Program in Epidemiology, Federal University of Pelotas, Pelotas, Brazil; Department of Preventive Medicine, University of Sao Paulo Medical School, Sao Paulo, Brazil},
abstract={Background: Pregnancy is strongly associated with increased risk for depression. Approximately 25% of pregnant women develop depression. Treatment for depression during pregnancy has several complexities: the use of psychiatric medications during pregnancy might result in developmental problems in the child and must be used with caution. Psychosocial interventions are effective, but they require specialized professionals. Low- and middle-income countries (LMIC) such as Brazil do not have enough mental health professionals needed to meet this demand. In this context, smartphone-based interventions show immense potential. We developed Motherly, a smartphone application (app) designed to treat maternal depression. We aim to test the efficacy of Motherly in addition to brief cognitive-behavioral therapies (CBT) to treat maternal depression. Methods: We will conduct a 2-arm parallel-randomized controlled clinical trial in which 70 pregnant women aged between 16 and 40 years with depression will be randomized to intervention or active control. The intervention group will have access to Motherly, a smartphone app based on three concepts: psychoeducation, behavior monitoring, and gaming elements. Motherly is composed of a package of interventions composed of modules: mental health, sleep, nutrition, physical activity, social support, prenatal/postnatal support, and educational content. The main focus of Motherly is delivering behavioral activation (BA), a brief and structured psychological treatment. The app allows participants to schedule and engage in, and monitor activities according to a plan to avoid acting exclusively according to their mood. The active control group will have access to a simplified version of the app consisting of educational content about various aspects of pregnancy, maternal physical and mental health, and infant development (BA, activity scheduling, sleep hygiene, among other functionalities, will not be present in this version). Both groups will receive four sessions of brief CBT in 8 weeks. Participants will be evaluated by assessors blind to randomization and allocation status. Assessments will occur at baseline (T0), midpoint (T1, week 4–5), posttreatment (T2, week 8), and follow-up (T3, when the child is 2 months old). Maternal mental health (prenatal anxiety, psychological well-being,
```

perceived stress, depression, depression severity, and sleep quality), quality of life, physical activity levels, and infant developmental milestones and social/emotional problems will be measured. Our primary outcome is the change in maternal prenatal depression from baseline to posttreatment (8 weeks). Discussion: The potential of digital technology to deliver mental health interventions has been increasingly recognized worldwide. There is a growing literature on interventions using smartphone applications to promote mental health, both with or without the intermediation of a mental health professional. Our study adds to the literature by testing whether an app providing an intervention package, including CBT, psychoeducation, nutrition, physical activity, and social support, can promote maternal and child health and well-being. In particular, we aim to treat depression, for which the use of digital technologies is still scarce. Smartphone applications designed to treat maternal depression are especially relevant because of the potential to circumvent barriers that prevent pregnant women from accessing mental health care. Trial registration: ClinicalTrials.gov NCT04495166. Prospectively registered on July 29, 2020. © 2021, The Author(s).},
author_keywords={Cognitive behavioral therapy; Digital technology; Maternal depression; Randomized controlled trial; Smartphone application},
references={James, S.L., Abate, D., Abate, K.H., Abay, S.M., Abbafati, C., Abbasi, N., Abbastabar, H., Murray, C.J.L., Global, regional, and national incidence, prevalence, and years lived with disability for 354 diseases and injuries for 195 countries and territories, 1990–2017: a systematic analysis for the global burden of disease study 2017 (2018) Lancet., 392 (10159), pp. 1789-1858; Wang, Y.-P., Chiavegatto, A.D., Campanha, A.M., Malik, A.M., Mogadouro, M.A., Cambraia, M., Patterns and predictors of health service use among people with mental disorders in São Paulo metropolitan area, Brazil (2017) Epidemiol Psychiatr Sci, 26 (1), pp. 89-101; Fatori, D., Salum, G.A., Rohde, L.A., Pan, P.M., Bressan, R., Evans-Lacko, S., Polanczyk, G., Graeff-Martins, A.S., Use of Mental Health Services by Children With Mental Disorders in Two Major Cities in Brazil (2019) Psychiatr Serv, 70 (4), pp. 337-341. , https://doi.org/10.1176/appi.ps.201800389; Belmaker, R.H., Agam, G., Major depressive disorder (2008) N Engl J Med, 358 (1), pp. 55-68; Kessler, R.C., Berglund, P., Demler, O., Jin, R., Koretz, D., Merikangas, K.R., Rush, A.J., Wang, P.S., The epidemiology of major depressive disorder: results from the National Comorbidity Survey Replication (NCS-R) (2003) JAMA., 289 (23), pp. 3095-3105; Gavin, N.I., Gaynes, B.N., Lohr, K.N., Meltzer-Brody, S., Gartlehner, G., Swinson, T., Perinatal depression: a systematic review of prevalence and incidence (2005) Obstet Gynecol, 106 (5), pp. 1071-1083; Gelaye, B., Rondon, M.B., Araya, R., Williams, M.A., Epidemiology of maternal depression, risk factors, and child outcomes in low-income and middle-income countries (2016) Lancet Psychiatry, 3 (10), pp. 973-982; Bauer, A., Parsonage, M., Knapp, M., Iemmi, V., Adelaja, B., (2014) The Costs of Perinatal Mental Health Problems, , http://everyonesbusiness.org.uk/wp-content/uploads/2014/12/Embargoed-20th-Oct-Summary-of-Economic-Report-costs-of-Perinatal-Mental-Health-problems.pdf, London School of Economics, Accessed 21 Sept 2020; Gonçalves, H., Pearson, R.M., Horta, B.L., González-Chica, D.A., Castilho, E., Damiani, M., Lima, R.C., Victora, C.G., Maternal depression and anxiety predicts the pattern of offspring symptoms during their transition to adulthood (2016) Psychol Med, 46 (2), pp. 415-424; Grote, N.K., Bridge, J.A., Gavin, A.R., Melville, J.L., Iyengar, S., Katon, W.J., A meta-analysis of depression during pregnancy and the risk of preterm birth, low birth weight, and intrauterine growth restriction (2010) Arch Gen Psychiatry, 67 (10), pp. 1012-1024; Kingston, D.,

Tough, S., Whitfield, H., Prenatal and postpartum maternal psychological distress and infant development: a systematic review (2012) Child Psychiatry Hum Dev, 43 (5), pp. 683-714; Matijasevich, A., Murray, J., Cooper, P.J., Anselmi, L., Barros, A.J.D., Barros, F.C., Santos, I.S., Trajectories of maternal depression and offspring psychopathology at 6 years: 2004 Pelotas cohort study (2015) J Affect Disord, 174, pp. 424-431; Netsi, E., Pearson, R.M., Murray, L., Cooper, P., Craske, M.G., Stein, A., Association of persistent and severe postnatal depression with child outcomes (2018) JAMA Psychiatry., 75 (3), pp. 247-253; Surkan, P.J., Ettinger, A.K., Ahmed, S., Minkovitz, C.S., Strobino, D., Impact of maternal depressive symptoms on growth of preschool- and school-aged children (2012) Pediatrics., 130 (4), pp. e847-e855; Surkan, P.J., Kennedy, C.E., Hurley, K.M., Black, M.M., Maternal depression and early childhood growth in developing countries: systematic review and meta-analysis (2011) Bull World Health Organ, 89, pp. 607-615; Alwan, S., Friedman, J.M., Safety of selective serotonin reuptake inhibitors in pregnancy (2009) CNS Drugs, 23 (6), pp. 493-509; Lugo-Candelas, C., Cha, J., Hong, S., Bastidas, V., Weissman, M., Fifer, W.P., Myers, M., Posner, J., Associations between brain structure and connectivity in infants and exposure to selective serotonin reuptake inhibitors during pregnancy (2018) JAMA Pediatr, 172 (6), pp. 525-533; Reefhuis, J., Devine, O., Friedman, J.M., Louik, C., Honein, M.A., Specific SSRIs and birth defects: Bayesian analysis to interpret new data in the context of previous reports (2015) BMJ, 351, p. h3190; Ammerman, R.T., Putnam, F.W., Altaye, M., Stevens, J., Teeters, A.R., Van Ginkel, J.B., A clinical trial of in-home CBT for depressed mothers in home visitation (2013) Behav Ther, 44 (3), pp. 359-372; Milgrom, J., Negri, L.M., Gemmill, A.W., McNeil, M., Martin, P.R., A randomized controlled trial of psychological interventions for postnatal depression (2005) Br J Clin Psychol, 44, pp. 529-542; Silverstein, M., Diaz-Linhart, Y., Cabral, H., Beardslee, W., Hegel, M., Haile, W., Sander, J., Feinberg, E., Efficacy of a maternal depression prevention strategy in head start: a randomized clinical trial (2017) JAMA Psychiatry, 74 (8), pp. 781-789; Spinelli, M.G., Endicott, J., Controlled clinical trial of interpersonal psychotherapy versus parenting education program for depressed pregnant women (2003) Am J Psychiatry, 160 (3), pp. 555-562; (2015) Pan American Health Organization. Mental Health Atlas of the Americas. Pan American Health Organization, , http://iris.paho.org/xmlui/bitstream/handle/123456789/28451/9789275119006_eng.pdf?sequence=1&isAllowed=y, Accessed 21 Sept 2020; (2015) WHO | Mental Health Atlas 2014, , http://www.who.int/mental_health/evidence/atlas/mental_health_atlas_2014/en/, WHO, Accessed 11 Sep 2016; Kazdin, A.E., Annual research review: expanding mental health services through novel models of intervention delivery (2019) J Child Psychol Psychiatry, 60 (4), pp. 455-472; (2016) Number of Smartphone Users Worldwide 2014–2020 | Statista. Statista, , https://www.statista.com/statistics/330695/number-of-smartphone-users-worldwide/.Accessed15May2018; (2015) Smartphone Average Selling Price Worldwide 2010–2019 | Statistic. Statista, , https://www.statista.com/statistics/484583/global-average-selling-price-smartphones/.Accessed16May2018; Firth, J., Torous, J., Nicholas, J., Carney, R., Pratap, A., Rosenbaum, S., Sarris, J., The efficacy of smartphone-based mental health interventions for depressive symptoms: a meta-analysis of randomized controlled trials (2017) World Psychiatry, 16 (3), pp. 287-298; Huguet, A., Rao, S., McGrath, P.J., Wozney, L., Wheaton, M., Conrod, J., A systematic review of cognitive behavioral therapy and behavioral activation apps for depression (2016) PLoS One, 11 (5); Domhardt, M., Geßlein, H., von Rezori,

R.E., Baumeister, H., Internet- and mobile-based interventions for anxiety disorders: a meta-analytic review of intervention components (2018) Depress Anxiety, 36 (3), pp. 213-224; Espie, C.A., Emsley, R., Kyle, S.D., Gordon, C., Drake, C.L., Siriwardena, A.N., Cape, J., Luik, A.I., Effect of digital cognitive behavioral therapy for insomnia on health, psychological well-being, and sleep-related quality of life: a randomized clinical trial (2018) JAMA Psychiatry., 76 (1), pp. 21-30; Bostock, S., Crosswell, A.D., Prather, A.A., Steptoe, A., Mindfulness on-the-go: effects of a mindfulness meditation app on work stress and well-being (2019) J Occup Health Psychol, 24 (1), pp. 127-138; Chan, K.L., Chen, M., Effects of social media and mobile health apps on pregnancy care: meta-analysis (2019) JMIR mHealth and uHealth, 7 (1); Cheng, H.-Y., Huang, T.-Y., Chien, L.-Y., Cheng, Y.-F., Chen, F.-J., The effects of a mobile application social support program on postpartum perceived stress and depression (2016) Hu Li Za Zhi, 63 (6), pp. 52-60; Chan, K.L., Leung, W.C., Tiwari, A., Or, K.L., Ip, P., Using smartphone-based psychoeducation to reduce postnatal depression among first-time mothers: randomized controlled trial (2019) JMIR Mhealth Uhealth, 7 (5); Kwong, W., What is government’s role in medical apps? (2015) CMAJ., 187 (11); Bates, D.W., Landman, A., Levine, D.M., Health apps and health policy: what is needed? (2018) JAMA., 320 (19), pp. 1975-1976; PHP Manual: Rand function, , https://www.php.net/manual/en/function.rand.php, PHP, Accessed 10 Jul 2020; Santos, I.S., Matijasevich, A., Tavares, B.F., Barros, A.J.D., Botelho, I.P., Lapolli, C., Magalhães, P.V.S., Barros, F.C., Validation of the Edinburgh Postnatal Depression Scale (EPDS) in a sample of mothers from the 2004 Pelotas Birth Cohort Study (2007) Cad Saude Publica, 23 (11), pp. 2577-2588; Santos, I.S., Tavares, B.F., Munhoz, T.N., Manzolli, P., de Ávila, G.B., Jannke, E., Matijasevich, A., Patient health questionnaire-9 versus Edinburgh postnatal depression scale in screening for major depressive episodes: a cross-sectional population-based study (2016) BMC Res Notes, 9 (1), p. 453; Matijasevich, A., Munhoz, T.N., Tavares, B.F., Barbosa, A.P.P.N., da Silva, D.M., Abitante, M.S., Dall’Agnol, T.A., Santos, I.S., Validation of the Edinburgh Postnatal Depression Scale (EPDS) for screening of major depressive episode among adults from the general population (2014) BMC Psychiatry, 14 (1), p. 284; Plummer, F., Manea, L., Trepel, D., McMillan, D., Screening for anxiety disorders with the GAD-7 and GAD-2: a systematic review and diagnostic metaanalysis (2016) Gen Hosp Psychiatry, 39, pp. 24-31; Moreno, A.L., DeSousa, D.A., Souza, A.M.F.L.P., Manfro, G.G., Salum, G.A., Koller, S.H., Factor structure, reliability, and item parameters of the Brazilian-Portuguese version of the GAD-7 Questionnaire (2016) Temas Psicol, 24 (1), pp. 367-376; Henrique, I.F.S., De Micheli, D., de Lacerda, R.B., de Lacerda, L.A., Formigoni MLO de, S., Validação da versão brasileira do teste de triagem do envolvimento com álcool, cigarro e outras substâncias (ASSIST) (2004) Rev Assoc Med Bras, 50 (2), pp. 199-206; Siqueira Reis, R., Ferreira Hino, A.A., Romélio Rodriguez Añez, C., Perceived Stress Scale (2010) J Health Psychol, 15 (1), pp. 107-114; Woods, S.A., Hampson, S.E., Measuring the big five with single items using a bipolar response scale (2005) Eur J Personal, 19 (5), pp. 373-390; Snyder, E., Cai, B., DeMuro, C., Morrison, M.F., Ball, W., A new single-item sleep quality scale: results of psychometric evaluation in patients with chronic primary insomnia and depression (2018) J Clin Sleep Med, 14 (11), pp. 1849-1857; Craig, C.L., Marshall, A.L., Sjöström, M., Bauman, A.E., Booth, M.L., Ainsworth, B.E., International physical activity questionnaire: 12-country reliability and validity (2003) Med Sci Sports Exerc, 35 (8), pp. 1381-1395; Lear, S.A., Hu, W., Rangarajan, S., Gasevic, D., Leong, D., Iqbal, R., Casanova, A., Yusuf, S., The effect of physical activity on mortality and cardiovascular disease in 130 000 people from 17 high-income,

middle-income, and low-income countries: the PURE study (2017) Lancet., 390 (10113), pp. 2643-2654; Abbott, R.A., Ploubidis, G.B., Huppert, F.A., Kuh, D., Croudace, T.J., An evaluation of the precision of measurement of Ryff's psychological well-being scales in a population sample (2010) Soc Indic Res, 97 (3), pp. 357-373; Ryff, C.D., Keyes, C.L., The structure of psychological well-being revisited (1995) J Pers Soc Psychol, 69 (4), pp. 719-727; Ryff, C.D., Singer, B.H., Know thyself and become what you are: a eudaimonic approach to psychological well-being (2008) J Happiness Stud, 9 (1), pp. 13-39; de Lara, M.W., Ruschel Bandeira, D., Pawlowski, J., Validação da Psychological Well-being Scale em uma amostra de estudantes universitários (2013) Avaliação Psicológica, 12, pp. 263-272; Damásio, B.F., Andrade, T.F., Koller, S.H., Psychometric properties of the Brazilian 12-item short-form health survey version 2 (SF-12v2) (2015) Paidéia (Ribeirão Preto), 25 (60), pp. 29-37; Kortum, P., Sorber, M., Measuring the usability of Mobile applications for phones and tablets (2015) Int J Hum Comput Interact, 31 (8), pp. 518-529; Stoyanov, S.R., Hides, L., Kavanagh, D.J., Zelenko, O., Tjondronegoro, D., Mani, M., Mobile app rating scale: a new tool for assessing the quality of health mobile apps (2015) JMIR Mhealth Uhealth., 3 (1); Moreira, R.S., Magalhães, L.D.C., Siqueira, C.M., Alves, C.R.L., Adaptação Transcultural do instrumento de vigilância do desenvolvimento infantil "Survey of Wellbeing of Young Children (SWYC)" no contexto brasileiro (2019) J Hum Growth Dev, 29 (1), pp. 28-38; Arroll, B., Goodyear-Smith, F., Crengle, S., Gunn, J., Kerse, N., Fishman, T., Falloon, K., Hatcher, S., Validation of PHQ-2 and PHQ-9 to screen for major depression in the primary care population (2010) Ann Fam Med, 8 (4), pp. 348-353; Munhoz, T.N., Nunes, B.P., Wehrmeister, F.C., Santos, I.S., Matijasevich, A., A nationwide population-based study of depression in Brazil (2016) J Affect Disord, 192, pp. 226-233; Pallesen, S., Bjorvatn, B., Nordhus, I.H., Sivertsen, B., Hjørnevik, M., Morin, C.M., A new scale for measuring insomnia: the Bergen insomnia scale (2008) Perceptual Motor Skilk, 107 (7), pp. 691-706; (2000) Diagnostic And Statistical Manual Of Mental Disorders DSM-IV-TR Fourth Edition (Text Revision), , American Psychiatric Association; da Silva, F.G., (2013) Reconhecimento de movimentos humanos utilizando um acelerômetro e inteligência computacional. Master, , Universidade de São Paulo; Kirwan, M., Duncan, M.J., Vandelanotte, C., Mummery, W.K., Using smartphone technology to monitor physical activity in the 10,000 steps program: a matched case-control trial (2012) J Med Internet Res, 14 (2); Tudor-Locke, C., Craig, C.L., Brown, W.J., Clemes, S.A., De Cocker, K., Giles-Corti, B., How many steps/day are enough? For adults (2011) Int J Behav Nutr Phys Act, 8 (1), p. 79; Edwards, E.A., Lumsden, J., Rivas, C., Steed, L., Edwards, L.A., Thiyagarajan, A., Sohanpal, R., Walton, R.T., Gamification for health promotion: systematic review of behaviour change techniques in smartphone apps (2016) BMJ Open, 6 (10); Sardi, L., Idri, A., Fernández-Alemán, J.L., A systematic review of gamification in e-health (2017) J Biomed Inform, 71, pp. 31-48; Ferster, C.B., A functional anlysis of depression (1973) Am Psychol, 28 (10), pp. 857-870; Lewinsohn, P.M., Hoberman, H., Teri, L., Hautzinger, M., An integrative theory of depression (1985) Theoretical Issues in Behavior Therapy, pp. 331-359. , Reiss S, Bootzin RR, (eds), Academic Press; Dimidjian, S., Barrera, M., Jr., Martell, C., Muñoz, R.F., Lewinsohn, P.M., The origins and current status of behavioral activation treatments for depression (2011) Annu Rev Clin Psychol, 7 (1), pp. 1-38; Mazzucchelli, T., Kane, R., Rees, C., Behavioral activation treatments for depression in adults: a meta-analysis and review (2009) Clin Psychol Sci Pract, 16, pp. 383-411; Ekers, D., Webster, L., Van Straten, A., Cuijpers, P., Richards, D., Gilbody, S., Behavioural activation for depression; an update of meta-analysis of effectiveness and sub group analysis (2014) PLoS

One, 9 (6); Kanter, J.W., Manos, R.C., Bowe, W.M., Baruch, D.E., Busch, A.M., Rusch, L.C., What is behavioral activation?: a review of the empirical literature (2010) Clin Psychol Rev, 30 (6), pp. 608-620; Cuijpers, P., Andersson, G., Donker, T., van Straten, A., Psychological treatment of depression: results of a series of meta-analyses (2011) Nord J Psychiatry, 65 (6), pp. 354-364; Cuijpers, P., van Straten, A., Andersson, G., van Oppen, P., Psychotherapy for depression in adults: a meta-analysis of comparative outcome studies (2008) J Consult Clin Psychol, 76 (6), pp. 909-922; Dimidjian, S., Goodman, S.H., Sherwood, N.E., Simon, G.E., Ludman, E., Gallop, R., Welch, S.S., Beck, A., A pragmatic randomized clinical trial of behavioral activation for depressed pregnant women (2017) J Consult Clin Psychol, 85 (1), pp. 26-36; Moradveisi, L., Huibers, M.J.H., Renner, F., Arasteh, M., Arntz, A., Behavioural activation v. antidepressant medication for treating depression in Iran: randomised trial (2013) Br J Psychiatry, 202 (3), pp. 204-211; Dobson, K.S., Hollon, S.D., Dimidjian, S., Schmaling, K.B., Kohlenberg, R.J., Gallop, R.J., Rizvi, S.L., Jacobson, N.S., Randomized trial of behavioral activation, cognitive therapy, and antidepressant medication in the prevention of relapse and recurrence in major depression (2008) J Consult Clin Psychol, 76 (3), pp. 468-477; Richards, D.A., Ekers, D., McMillan, D., Taylor, R.S., Byford, S., Warren, F.C., Barrett, B., Finning, K., Cost and outcome of behavioural activation versus cognitive behavioural therapy for depression (COBRA): a randomised, controlled, non-inferiority trial (2016) Lancet., 388 (10047), pp. 871-880; Dimidjian, S., Hollon, S.D., Dobson, K.S., Schmaling, K.B., Kohlenberg, R.J., Addis, M.E., Gallop, R., Jacobson, N.S., Randomized trial of behavioral activation, cognitive therapy, and antidepressant medication in the acute treatment of adults with major depression (2006) J Consult Clin Psychol, 74 (4), pp. 658-670; Ekers, D., Godfrey, C., Gilbody, S., Parrott, S., Richards, D.A., Hammond, D., Hayes, A., Cost utility of behavioural activation delivered by the non-specialist (2011) Br J Psychiatry, 199 (6), pp. 510-511; Ekers, D., Richards, D., McMillan, D., Bland, J.M., Gilbody, S., Behavioural activation delivered by the non-specialist: phase II randomised controlled trial (2011) Br J Psychiatry, 198 (1), pp. 66-72; Dahne, J., Kustanowitz, J., Lejuez, C.W., Development and preliminary feasibility study of a brief behavioral activation mobile application (Behavioral Apptivation) to be used in conjunction with ongoing therapy (2018) Cogn Behav Pract, 25 (1), pp. 44-56; Dahne, J., Lejuez, C.W., Kustanowitz, J., Felton, J.W., Diaz, V.A., Player, M.S., Carpenter, M.J., Moodivate: a self-help behavioral activation mobile app for utilization in primary care-development and clinical considerations (2017) Int J Psychiatry Med, 52 (2), pp. 160-175; Martell, C.R., Dimidjian, S., Herman-Dunn, R., (2013) Behavioral activation for depression: a clinician’s guide, , Guilford Press; Lejuez, C.W., Hopko, D.R., Acierno, R., Daughters, S.B., Pagoto, S.L., Ten year revision of the brief behavioral activation treatment for depression: revised treatment manual (2011) Behav Modif, 35 (2), pp. 111-161; Perlis, M.L., Aloia, M., Kuhn, B., (2010) Behavioral treatments for sleep disorders: a comprehensive primer of behavioral sleep medicine interventions (practical resources for the mental health professional). 1 edition, , Academic Press; Hauri, P.J., (2004) Sleep Disorders, , Upjohn; Chung, K.-F., Lee, C.-T., Yeung, W.-F., Chan, M.-S., Chung, E.W.-Y., Lin, W.-L., Sleep hygiene education as a treatment of insomnia: a systematic review and meta-analysis (2018) Fam Pract, 35 (4), pp. 365-375; Benson, H., Klipper, M.Z., (1975) The relaxation response. Updated & Expanded ed. edition, , William Morrow Paperbacks; Stremler, R., Hodnett, E., Lee, K., MacMillan, S., Mill, C., Ongcangco, L., Willan, A., A behavioral-educational intervention to promote maternal and infant sleep: a pilot randomized, controlled trial (2006) Sleep.,

29 (12), pp. 1609-1615; Stremler, R., Hodnett, E., Kenton, L., Lee, K., Weiss, S., Weston, J., Willan, A., Effect of behavioural-educational intervention on sleep for primiparous women and their infants in early postpartum: multisite randomised controlled trial (2013) BMJ., 346 (mar20 1), p. f1164; Bernstein, B., Borkovec, T., (1973) Progressive Relaxation Training, , Research Press; Jacobson, E., Progressive relaxation: a physiological & clinical investigation of muscular states & their significance in psychology & medical practice (1929) 2nd Edition - Series: The University of Chicago monographs in medicine. edition, , University of Chicago Press; Bhutta, Z.A., Das, J.K., Rizvi, A., Gaffey, M.F., Walker, N., Horton, S., Webb, P., Black, R.E., Evidence-based interventions for improvement of maternal and child nutrition: what can be done and at what cost? (2013) Lancet., 382 (9890), pp. 452-477; Barker, D.J.P., Sir Richard Doll Lecture. Developmental origins of chronic disease (2012) Public Health, 126 (3), pp. 185-189; Benja, M., Lawrie, T.A., Lumbiganon, P., Laopaiboon, M., Diet or Exercise, or Both, for Preventing Excessive Weight Gain in Pregnancy (2015) Cochrane Database Syst Rev, (6), pp. 1-203. , https://doi.org/10.1002/14651858.CD007145.pub3; Goldstein, R.F., Abell, S.K., Ranasinha, S., Misso, M., Boyle, J.A., Black, M.H., Li, N., Teede, H.J., Association of gestational weight gain with maternal and infant outcomes: a systematic review and meta-analysis (2017) JAMA., 317 (21), pp. 2207-2225; Jacka, F.N., O’Neil, A., Opie, R., Itsiopoulos, C., Cotton, S., Mohebbi, M., Castle, D., Berk, M., A randomised controlled trial of dietary improvement for adults with major depression (the “SMILES” trial) (2017) BMC Med, 15 (1), p. 23; Lassale, C., Batty, G.D., Baghdadli, A., Jacka, F., Sánchez-Villegas, A., Kivimäki, M., Akbaraly, T., Healthy dietary indices and risk of depressive outcomes: a systematic review and meta-analysis of observational studies (2018) Mol Psychiatry, 24 (7), pp. 965-986; Opie, R.S., Itsiopoulos, C., Parletta, N., Sanchez-Villegas, A., Akbaraly, T.N., Ruusunen, A., Jacka, F.N., Dietary recommendations for the prevention of depression (2017) Nutr Neurosci, 20 (3), pp. 161-171; (2014) Guia Alimentar Para a População Brasileira. Ministério Da Saúde; Atenção ao pré-natal de baixo risco. Cadernos de Atenção Básica (2012) Secretaria De Atenção à Saúde D De AB; (2010) Weight gain during pregnancy: reexamining the guidelines, , National Academies Press (US), Washington (DC; (2010) Global recommendations on physical activity for health; Vargas-Terrones, M., Barakat, R., Santacruz, B., Fernandez-Buhigas, I., Mottola, M.F., Physical exercise programme during pregnancy decreases perinatal depression risk: a randomised controlled trial (2019) Br J Sports Med, 53 (6), pp. 348-353; Guthold, R., Stevens, G.A., Riley, L.M., Bull, F.C., Worldwide trends in insufficient physical activity from 2001 to 2016: a pooled analysis of 358 population-based surveys with 1·9 million participants (2018) Lancet Glob Health, 6 (10), pp. e1077-e1086; (2015) Physical activity and exercise during pregnancy and the postpartum period, , The American College of Obstetricians and Gynecologists; da Silva, S.G., Ricardo, L.I., Evenson, K.R., Hallal, P.C., Leisure-time physical activity in pregnancy and maternal-child health: a systematic review and meta-analysis of randomized controlled trials and cohort studies (2017) Sports Med, 47 (2), pp. 295-317; Bravata, D.M., Smith-Spangler, C., Sundaram, V., Gienger, A.L., Lin, N., Lewis, R., Stave, C.D., Sirard, J.R., Using pedometers to increase physical activity and improve health: a systematic review (2007) JAMA., 298 (19), pp. 2296-2304; Uchino, B.N., Social support and health: a review of physiological processes potentially underlying links to disease outcomes (2006) J Behav Med, 29 (4), pp. 377-387; Elsenbruch, S., Benson, S., Rücke, M., Rose, M., Dudenhausen, J., Pincus-Knackstedt, M.K., Klapp, B.F., Arck, P.C., Social support during pregnancy: effects on maternal depressive symptoms, smoking and pregnancy outcome (2007) Hum Reprod, 22 (3),

pp. 869-877; Pfeiffer, P.N., Heisler, M., Piette, J.D., Rogers, M.A.M., Valenstein, M., Efficacy of peer support interventions for depression: a meta-analysis (2011) Gen Hosp Psychiatry, 33 (1), pp. 29-36; Mbuagbaw, L., Medley, N., Darzi, A.J., Richardson, M., Habiba Garga, K., Ongolo-Zogo, P., Health system and community level interventions for improving antenatal care coverage and health outcomes (2015) Cochrane Database Syst Rev, 12; (2016) WHO recommendations on antenatal care for a positive pregnancy experience, , WHO; Sockol, L.E., A systematic review of the efficacy of cognitive behavioral therapy for treating and preventing perinatal depression (2015) J Affect Disord, 177, pp. 7-21; Hayes, A.F., (2013) Introduction to mediation, moderation, and conditional process analysis, first edition: a regression-based approach (methodology in the social sciences). First edition, , The Guilford Press; Cumming, G., Cohen'd (2011) Understanding the new statistics: effect sizes, confidence intervals, and meta-analysis, , Routledge, New York, N.Y; Harris, P.A., Taylor, R., Thielke, R., Payne, J., Gonzalez, N., Conde, J.G., Research electronic data capture (REDCap)—a metadata-driven methodology and workflow process for providing translational research informatics support (2009) J Biomed Inform, 42 (2), pp. 377-381; Everly, G.S., Jr., Lating, J.M., (2017) The Johns Hopkins Guide to Psychological First Aid. 1 edition, , Johns Hopkins University Press; Naslund, J.A., Aschbrenner, K.A., Araya, R., Marsch, L.A., Unützer, J., Patel, V., Bartels, S.J., Digital technology for treating and preventing mental disorders in low-income and middle-income countries: a narrative review of the literature (2017) Lancet Psychiatry, 4 (6), pp. 486-500; Menezes, P., Quayle, J., Garcia Claro, H., da Silva, S., Brandt, L.R., Diez-Canseco, F., Miranda, J.J., Araya, R., Use of a mobile phone app to treat depression comorbid with hypertension or diabetes: a pilot study in Brazil and Peru (2019) JMIR Ment Health., 6 (4); Brandt, L.R., Hidalgo, L., Diez-Canseco, F., Araya, R., Mohr, D.C., Menezes, P.R., Miranda, J.J., Addressing depression comorbid with diabetes or hypertension in resource-poor settings: a qualitative study about user perception of a nurse-supported smartphone app in Peru (2019) JMIR Ment Health, 6 (6); Evans, G.W., Cassells, R.C., Childhood poverty, cumulative risk exposure, and mental health in emerging adults (2014) Clin Psychol Sci, 2 (3), pp. 287-296; Evans, G.W., Kim, P., Childhood poverty and health: cumulative risk exposure and stress dysregulation (2007) Psychol Sci, 18 (11), pp. 953-957; Roberts, G., Bellinger, D., McCormick, M.C., A cumulative risk factor model for early identification of academic difficulties in premature and low birth weight infants (2007) Matern Child Health J, 11 (2), pp. 161-172; Torous, J., Lipschitz, J., Ng, M., Firth, J., Dropout rates in clinical trials of smartphone apps for depressive symptoms: a systematic review and meta-analysis (2020) J Affect Disord, 263, pp. 413-419; Cooper, A.A., Conklin, L.R., Dropout from individual psychotherapy for major depression: a meta-analysis of randomized clinical trials (2015) Clin Psychol Rev, 40, pp. 57-65},
correspondence_address1={Zuccolo, P.F.; Department of Psychiatry, Brazil; email: pedrozuccolo@yahoo.com.br},
publisher={BioMed Central Ltd},
issn={17456215},
pubmed_id={33757591},
language={English},
abbrev_source_title={Trials},
document_type={Article},
source={Scopus},
}

@ARTICLE{Lam2021,
author={Lam, T.M. and Vaartjes, I. and Grobbee, D.E. and Karssenberg, D. and Lakerveld, J.},
title={Associations between the built environment and obesity: an umbrella review},
journal={International Journal of Health Geographics},
year={2021},
volume={20},
number={1},
doi={10.1186/s12942-021-00260-6},
art_number={7},
note={cited By 0},
url={https://www.scopus.com/inward/record.uri?eid=2-s2.0-85100241821&doi=10.1186%2fs12942-021-00260-6&partnerID=40&md5=00dff9e8f1f0321cc19cc624c7439280},
affiliation={Julius Center for Health Sciences and Primary Care, University Medical Center Utrecht & Utrecht University, Utrecht, Netherlands; Department of Physical Geography, Utrecht University, Utrecht, Netherlands; Global Geo Health Data Center, University Medical Center Utrecht & Utrecht University, Utrecht, Netherlands; Department of Epidemiology and Data Science, Amsterdam University Medical Centers (VUmc Location), De Boelelaan 1089a, Amsterdam, 1081HV, Netherlands; Dutch Health Foundation, The Hague, Netherlands; Julius Global Health, University Medical Center Utrecht & Utrecht University, Utrecht, Netherlands},
abstract={Background: In the past two decades, the built environment emerged as a conceptually important determinant of obesity. As a result, an abundance of studies aiming to link environmental characteristics to weight-related outcomes have been published, and multiple reviews have attempted to summarise these studies under different scopes and domains. We set out to summarise the accumulated evidence across domains by conducting a review of systematic reviews on associations between any aspect of the built environment and overweight or obesity. Methods: Seven databases were searched for eligible publications from the year 2000 onwards. We included systematic literature reviews, meta-analyses and pooled analyses of observational studies in the form of cross-sectional, case–control, longitudinal cohort, ecological, descriptive, intervention studies and natural experiments. We assessed risk of bias and summarised results structured by built environmental themes such as food environment, physical activity environment, urban–rural disparity, socioeconomic status and air pollution. Results: From 1850 initial hits, 32 systematic reviews were included, most of which reported equivocal evidence for associations. For food- and physical activity environments, associations were generally very small or absent, although some characteristics within these domains were consistently associated with weight status such as fast-food exposure, urbanisation, land use mix and urban sprawl. Risks of bias were predominantly high. Conclusions: Thus far, while most studies have not been able to confirm the assumed influence of built environments on weight, there is evidence for some obesogenic environmental characteristics. Registration: This umbrella review was registered on PROSPERO under ID CRD42019135857. © 2021, The Author(s).},
author_keywords={Built environment; Food environment; Obesity; Obesogenic environment; Overweight; Physical activity; Umbrella review},
keywords={food safety; literature review; obesity; physical activity; urban area; weight},
references={Amarasinghe, A., D’Souza, G., Individual, social, economic, and environmental model: a paradigm shift for obesity prevention (2012) ISRN Public

Health, 2012, pp. 1-10; (2018) Obesity and Overweight., , https://www.who.int/news-room/fact-sheets/detail/obesity-and-overweight; Afshin, A., Forouzanfar, M.H., Reitsma, M.B., Sur, P., Estep, K., Lee, A., Health effects of overweight and obesity in 195 countries over 25 years (2017) N Engl J Med, 377 (1), pp. 13-27. , PID: 28604169; Ross, S.E., Flynn, J.I., Pate, R.R., What is really causing the obesity epidemic? A review of reviews in children and adults (2016) J Sports Sci., 34, pp. 1148-1153. , PID: 26400631; Romieu, I., Dossus, L., Barquera, S., Blottière, H.M., Franks, P.W., Gunter, M., Energy balance and obesity: what are the main drivers? (2017) Cancer Causes Control, 28 (3), pp. 247-258. , PID: 28210884; Lakerveld, J., Mackenbach, J., The upstream determinants of adult obesity (2017) Obes Facts, 10 (3), pp. 216-222. , PID: 28564658; Roberto, C.A., Swinburn, B., Hawkes, C., Huang, T.T.K., Costa, S.A., Ashe, M., Patchy progress on obesity prevention: emerging examples, entrenched barriers, and new thinking (2015) Lancet, 385, pp. 2400-2409. , PID: 25703111; Swinburn, B., Egger, G., Raza, F., The development and application of a framework for identifying and prioritizing environmental interventions for obesity (1999) Dissecting Obesogenic Environ, 29, pp. 563-570. , COI: 1:STN:280:DC%2BD3c%2FntVWqsw%3D%3D; Kremers, S.P., de Bruijn, G.J., Visscher, T.L., van Mechelen, W., de Vries, N.K., Brug, J., Environmental influences on energy balance-related behaviors: a dual-process view (2006) Int J Behav Nutr Phys Act., 3, p. 9. , PID: 16700907; Booth, S.L., Sallis, J.F., Ritenbaugh, C., Hill, J.O., Birch, L.L., Frank, L.D., Environmental and societal factors affect food choice and physical activity: rationale, influences, and leverage points (2009) Nutr Rev, 59 (3), pp. S21-S36; Northridge, M.E., Sclar, E.D., Biswas, P., Sorting out the connections between the built environment and health: a conceptual framework for navigating pathways and planning healthy cities (2003) J Urban Health., 80, pp. 556-568. , PID: 14709705; Vineis, P., Chadeau-Hyam, M., Gmuender, H., Gulliver, J., Herceg, Z., Kleinjans, J., The exposome in practice: design of the EXPOsOMICS project (2017) Int J Hyg Environ Health, 220 (2), pp. 142-151. , COI: 1:STN:280:DC%2BC2szlvFOkug%3D%3D, PID: 27576363; Riggs, D.W., Yeager, R.A., Bhatnagar, A., Defining the human envirome: An omics approach for assessing the environmental risk of cardiovascular disease (2018) Circ Res, 122 (9), pp. 1259-1275. , COI: 1:CAS:528:DC%2BC1cXosVeqsrs%3D, PID: 29700071; Dai, D., Prussin, A.J., Marr, L.C., Vikesland, P.J., Edwards, M.A., Pruden, A., Factors shaping the human exposome in the built environment: opportunities for engineering control (2017) Environ Sci Technol., 51, pp. 7759-7774. , COI: 1:CAS:528:DC%2BC2sXhtV2qu7vO, PID: 28677960; Buck Louis, G.M., Sundaram, R., Exposome: time for transformative research (2012) Stat Med, 31 (22), pp. 2569-2575. , PID: 22969025; Frank, L.D., Iroz-Elardo, N., MacLeod, K.E., Hong, A., Pathways from built environment to health: A conceptual framework linking behavior and exposure-based impacts (2019) J Trans Health., 12, pp. 319-335; Allender, S., Foster, C., Hutchinson, L., Arambepola, C., Quantification of urbanization in relation to chronic diseases in developing countries: a systematic review (2008) J Urban Heal, 85 (6), pp. 938-951; Lachowycz, K., Jones, A.P., Greenspace and obesity: a systematic review of the evidence (2011) Obes Rev, 12 (501), pp. 183-189; Grasser, G., Van Dyck, D., Titze, S., Stronegger, W., Objectively measured walkability and active transport and weight-related outcomes in adults: a systematic review (2013) Int J Public Health, 58 (4), pp. 615-625. , PID: 23224518; Papas, M.A., Alberg, A.J., Ewing, R., Helzlsouer, K.J., Gary, T.L., Klassen, A.C., The built environment and obesity (2007) Epidemiol Rev, 29, pp. 129-143. , PID: 17533172; Cobb, L.K., Appel, L.J., Manuel Franco, M., Jones-Smith, J.C., Alana Nur, A., Anderson, C.A., The relationship of the local food environment with obesity: systematic

review of methods (2015) Study Qual Results Obes, 23 (7), pp. 1331-1344; Sugiyama, T., Koohsari, M.J., Mavoa, S., Owen, N., Activity-friendly built environment attributes and adult adiposity (2014) Curr Obes Rep, 3 (2), pp. 183-198. , PID: 26626602; Mackenbach, J.D., Rutter, H., Compernolle, S., Glonti, K., Oppert, J.-M., Charreire, H., Obesogenic environments: a systematic review of the association between the physical environment and adult weight status, the SPOTLIGHT project (2014) BMC Public Health, 14, p. 233. , PID: 24602291; Moher, D., Liberati, A., Tetzlaff, J., Altman, D.G., Altman, D., Antes, G., Preferred reporting items for systematic reviews and meta-analyses: the PRISMA statement (2009) PLoS Med, 6, p. 873; Ouzzani, M., Hammady, H., Fedorowicz, Z., Elmagarmid, A., Rayyan-a web and mobile app for systematic reviews (2016) Syst Rev, 5 (1), p. 210. , PID: 27919275; Whiting, P., Savovi, J., Higgins, J.P.T., Caldwell, D.M., Reeves, B.C., Shea, B., ROBIS: a new tool to assess risk of bias in systematic reviews was developed (2016) J Clin Epidemiol, 69, pp. 225-234. , PID: 26092286; Richardson, W.S., Wilson, M.C., Nishikwa, J., Haywood, R.S.A., The well-built clinical question: a key to evidence-based decisions (1995) Am College Phys J Club., 123, pp. 12-13; Guyatt, G., Oxman, A.D., Akl, E.A., Kunz, R., Vist, G., Brozek, J., GRADE guidelines: 1. Introduction—GRADE evidence profiles and summary of findings tables (2011) J Clin Epidemiol., 64 (4), pp. 383-394. , PID: 21195583; Chandrabose, M., Rachele, J.N., Gunn, L., Kavanagh, A., Owen, N., Turrell, G., Built environment and cardio-metabolic health: systematic review and meta-analysis of longitudinal studies (2019) Obes Rev, 20 (1), pp. 41-54. , COI: 1:STN:280:DC%2BB3czisFKjsw%3D%3D, PID: 30253075; Schüle, S.A., Bolte, G., Interactive and independent associations between the socioeconomic and objective built environment on the neighbourhood level and individual health: A systematic review of multilevel studies (2015) PLoS ONE; Black, J.L., Macinko, J., Neighborhoods and obesity (2008) Nutr Rev, 66 (1), pp. 2-20. , PID: 18254880; Leal, C., Chaix, B., The influence of geographic life environments on cardiometabolic risk factors: a systematic review, a methodological assessment and a research agenda (2011) Obes Rev, 12 (3), pp. 217-230. , COI: 1:STN:280:DC%2BC3M3htVOlsQ%3D%3D, PID: 20202135; Feng, J., Glass, T.A., Curriero, F.C., Stewart, W.F., Schwartz, B.S., The built environment and obesity: a systematic review of the epidemiologic evidence (2010) Heal Place, 16 (2), pp. 175-190; Angkurawaranon, C., Jiraporncharoen, W., Chenthanakij, B., Doyle, P., Nitsch, D., Urban environments and obesity in southeast asia: A systematic review, meta-analysis and meta-regression (2014) PLoS ONE, 9 (11), pp. 1-19; Hernández, A.V., Pasupuleti, V., Deshpande, A., Bernabé-Ortiz, A., Miranda, J.J., Effect of rural-to-urban within-country migration on cardiovascular risk factors in low- and middle-income countries: a systematic review (2012) Heart, 98 (3), pp. 185-194. , PID: 21917659; Wilkins, E., Radley, D., Morris, M., Hobbs, M., Christensen, A., Marwa, W.L., A systematic review employing the GeoFERN framework to examine methods, reporting quality and associations between the retail food environment and obesity (2019) Health Place, 57, pp. 186-199. , PID: 31060018; Holsten, J.E., Obesity and the community food environment: a systematic review (2009) Public Health Nutr, 12 (3), pp. 397-405. , PID: 18477414; Fraser, L.K., Edwards, K.L., Cade, J., Clarke, G.P., The geography of fast food outlets: a review (2010) Int J Environ Res Public Health, 7 (5), pp. 2290-2308. , PID: 20623025; Gamba, R.J., Schuchter, J., Rutt, C., Seto, E.Y.W., Measuring the food environment and its effects on obesity in the United States: a systematic review of methods and results (2015) J Community Health, 40 (3), pp. 464-475. , PID: 25326425; Fleischghacker, S.E., Evenson, K.R., Rodriguez, D.A., Ammerman, A.S., A systematic review of fast food access studies (2011) Obes Rev, 12 (501), pp.

460-471; Giskes, K., van Lenthe, F., Avendano-Pabon, M., Brug, J., A systematic review of environmental factors and obesogenic dietary intakes among adults: are we getting closer to understanding obesogenic environments? (2011) Obes Rev, 12 (501), pp. e95-e106. , COI: 1:STN:280:DC%2BC3MvktlClsQ%3D%3D, PID: 20604870; Tseng, E., Zhang, A., Shogbesan, O., Gudzune, K.A., Wilson, R.F., Kharrazi, H., Effectiveness of policies and programs to combat adult obesity: a systematic review (2018) J Gen Intern Med, 33 (11), pp. 1990-2001. , PID: 30206789; McCormack, G.R., Cabaj, J., Orpana, H., Lukic, R., Blackstaffe, A., Goopy, S., A scoping review on the relations between urban form and health: a focus on Canadian quantitative evidence (2019) Heal Promot Chronic Dis Prev Canada, 39 (5), pp. 187-200; Grasser, G., van Dyck, D., Titze, S., Stronegger, W.J., A European perspective on GIS-based walkability and active modes of transport (2017) Eur J Public Health, 27 (1), pp. 145-151. , PID: 28013244; Malambo, P., Kengne, A.P., De Villiers, A., Lambert, E.V., Puoane, T., Built Environment, selected risk factors and major cardiovascular disease outcomes: a systematic review (2016) PLoS ONE, 11 (11). , PID: 27880835; Renalds, A., Smith, T.H., Hale, P.J., A systematic review of built environment and health (2010) Fam Community Heal, 33 (1), pp. 68-78; Kondo, M.C., Fluehr, J.M., McKeon, T., Branas, C.C., Urban green space and its impact on human health (2018) Int J Environ Res Public Health., 15 (3), p. 445; Patterson, R., Webb, E., Hone, T., Millett, C., Laverty, A.A., Associations of public transportation use with cardiometabolic health: a systematic review and meta-analysis (2019) Am J Epidemiol, 188 (4), pp. 785-795. , PID: 30689686; An, R., Ji, M., Yan, H., Guan, C., Impact of ambient air pollution on obesity: a systematic review (2018) Int J Obes., 42 (6), pp. 1112-1126. , COI: 1:CAS:528:DC%2BC1cXhtVOmurvM; Casagrande, S.S., Whitt-Glover, M.C., Lancaster, K.J., Odoms-Young, A.M., Gary, T.L., Built environment and health behaviors among african americans: a systematic review (2009) Am J Prev Med, 36 (2), pp. 174-181. , PID: 19135908; Lovasi, G.S., Hutson, M.A., Guerra, M., Neckerman, K.M., Built environments and obesity in disadvantaged populations (2009) Epidemiol Rev., 31 (1), pp. 7-20. , PID: 19589839; Larson, N.I., Story, M.T., Nelson, M.C., Neighborhood environments: disparities in access to healthy foods in the U.S (2009) Am J Prev Med., 36 (1), pp. 74-81. , PID: 18977112; Ferdinand, A.O., Sen, B., Rahurkar, S., Engler, S., Menachemi, N., The relationship between built environments and physical activity: a systematic review (2012) Am J Public Health, 102 (10), pp. e7-e13. , PID: 22897546; Drewnowski, A., Buszkiewicz, J., Aggarwal, A., Rose, C., Gupta, S., Bradshaw, A., Obesity and the built environment: a reappraisal (2019) Obesity, 28 (1), pp. 22-30. , PID: 31782242; Sleddens, E.F.C., Kroeze, W., Kohl, L.F.M., Bolten, L.M., Velema, E., Kaspers, P., Correlates of dietary behavior in adults: an umbrella review (2015) Nutr Rev, 73 (8), pp. 477-499. , PID: 26106126; Carlin, A., Perchoux, C., Puggina, A., Aleksovska, K., Buck, C., Burns, C., A life course examination of the physical environmental determinants of physical activity behaviour: a “Determinants of Diet and Physical Activity” (DEDIPAC) umbrella systematic literature review (2017) PLoS ONE, 12 (8). , PID: 28787023; Jia, P., Yu, C., Remais, J.V., Stein, A., Liu, Y., Brownson, R.C., Spatial Lifecourse Epidemiology Reporting Standards (ISLE-ReSt) statement (2020) Heal Place, 1 (61), p. 102243; Rutter, H., The complex systems challenge of obesity (2018) Clin Chem., 64, pp. 44-46. , COI: 1:CAS:528:DC%2BC1cXitlSgtb3N, PID: 29097510; (2008) Economic Organization for Cooperation and Development. Handbook on Constructing Composite Indicators: Methodology and User Guide. Handbook on Constructing Composite Indicators: Methodology and User Guide, p. 162; Seehra, J., Pandis, N., Koletsi, D., Fleming, P.S., Use of quality assessment tools in systematic reviews was varied and inconsistent (2016) J Clin Epidemiol, 1 (69),

pp. 179-184.e5; Ding, D., Gebel, K., Built environment, physical activity, and obesity: what have we learned from reviewing the literature? (2012) Heal Place., 18 (1), pp. 100-105; Durand, C.P., Andalib, M., Dunton, G.F., Wolch, J., Pentz, M.A., A systematic review of built environment factors related to physical activity and obesity risk: Implications for smart growth urban planning (2011) Obes Rev, 12 (501), pp. e173-e182. , COI: 1:STN:280:DC%2BC3MvksVGjsA%3D%3D, PID: 21348918; King, D.M., Jacobson, S.H., What is driving obesity? A review on the connections between obesity and motorized transportation (2017) Curr Obes Rep, 6 (1), pp. 3-9. , PID: 28243840; Bixby, H., Bentham, J., Zhou, B., Di Cesare, M., Paciorek, C.J., Bennett, J.E., Rising rural body-mass index is the main driver of the global obesity epidemic in adults (2019) Nature, 569 (7755), pp. 260-264; Serrano Fuentes, N., Rogers, A., Portillo, M.C., Social network influences and the adoption of obesity-related behaviours in adults: A critical interpretative synthesis review (2019) BMC Public Health., 19, p. 1178. , PID: 31455318; Powell, K., Wilcox, J., Clonan, A., Bissell, P., Preston, L., Peacock, M., (2010) The Role of Social Networks in the Development of Overweight and Obesity among Adults: A Scoping Review; Rutter, H., The single most important intervention to tackle obesity… (2012) Int J Public Health, 57 (4), pp. 657-658. , PID: 22752266; Hamid, T.K.A., (2009) Thinking in circles about obesity: applying systems thinking to weight management. Thinking in circles about obesity: applying systems thinking to weight management, pp. 1-467. , Springer, Berlin; VanderWeele, T.J., Explanation in causal inference: developments in mediation and interaction (2016) Int J Epidemiol, 45 (6), pp. 1904-1908. , PID: 27864406; Bourisly, A.K., An obesity agent based model: A new decision support system for the obesity epidemic (2013) Communications in Computer and Information Science, pp. 37-48. , Berlin: Springer Verlag},
correspondence_address1={Lam, T.M.; Julius Center for Health Sciences and Primary Care, Netherlands; email: t.m.lam@amsterdamumc.nl},
publisher={BioMed Central Ltd},
issn={1476072X},
pubmed_id={33526041},
language={English},
abbrev_source_title={Int. J. Health Geogr.},
document_type={Review},
source={Scopus},
}

@ARTICLE{Pizzoli2021,
author={Pizzoli, S.F.M. and Marzorati, C. and Gatti, D. and Monzani, D. and Mazzocco, K. and Pravettoni, G.},
title={A meta-analysis on heart rate variability biofeedback and depressive symptoms},
journal={Scientific Reports},
year={2021},
volume={11},
number={1},
doi={10.1038/s41598-021-86149-7},
art_number={6650},
note={cited By 0},
url={https://www.scopus.com/inward/record.uri?eid=2-s2.0-85103178732&doi=10.1038%2fs41598-021-86149-7&partnerID=40&md5=df75298b6222d01e10272bffcbfa89ca},
affiliation={Department of Oncology and Hemato-Oncology, University of Milan, Via Festa del Perdono 7, Milan, 20122, Italy; Applied Research Division for

Cognitive and Psychological Science, European Institute of Oncology IEO, IRCCS, Milan, Italy; Department of Brain and Behavioral Sciences, University of Pavia, Pavia, Italy},
abstract={Heart rate variability biofeedback (HRVB) has been used for a number of years to treat depressive symptoms, a common mental health issue, which is often comorbid with other psychopathological and medical conditions. The aim of the present meta-analysis is to test whether and to what extent HRVB is effective in reducing depressive symptoms in adult patients. We conducted a literature search on Pubmed, ProQuest, Ovid PsycInfo, and Embase up to October 2020, and identified 721 studies. Fourteen studies were included in the meta-analysis. Three meta-regressions were also performed to further test whether publication year, the questionnaire used to assess depressive symptoms, or the interval of time between T0 and T1 moderated the effect of HRVB. Overall, we analysed 14 RCTs with a total of 794 participants. The random effect analysis yielded a medium mean effect size g = 0.38 [95% CI = 0.16, 0.60; 95% PI = – 0.19, 0.96], z = 3.44, p = 0.0006. The total heterogeneity was significant, QT = 23.49, p = 0.03, I2 = 45%, which suggested a moderate variance among the included studies. The year of publication (χ2(1) = 4.08, p = 0.04) and the questionnaire used to assess symptoms (χ2(4) = 12.65, p = 0.01) significantly moderated the effect of the interventions and reduced heterogeneity. Overall, results showed that HRVB improves depressive symptoms in several psychophysiological conditions in adult samples and should be considered as a valid technique to increase psychological well-being. © 2021, The Author(s).},
references={(2017) Depression and Other Common Mental Disorders Global Health Estimates, , WHO; Hasin, D.S., Sarvet, A.L., Meyers, J.L., Saha, T.D., Ruan, W.J., Stohl, M., Grant, B.F., Epidemiology of adult DSM-5 major depressive disorder and its specifiers in the United States (2018) JAMA Psychiatry, 75 (4), pp. 336-346; Moussavi, S., Chatterji, S., Verdes, E., Tandon, A., Patel, V., Ustun, B., Depression, chronic diseases, and decrements in health: Results from the World Health Surveys (2007) Lancet, 370 (9590), pp. 851-858; Penninx, B.W.J.H., Milaneschi, Y., Lamers, F., Vogelzangs, N., Understanding the somatic consequences of depression: Biological mechanisms and the role of depression symptom profile (2013) BMC Med.; DiMatteo, M.R., Lepper, H.S., Croghan, T.W., Depression is a risk factor for noncompliance with medical treatment meta-analysis of the effects of anxiety and depression on patient adherence (2000) Arch. Intern. Med., 160 (14), pp. 2101-2107. , COI: 1:STN:280:DC%2BD3czptVKgug%3D%3D; Knol, M.J., Twisk, J.W.R., Beekman, A.T.F., Heine, R.J., Snoek, F.J., Pouwer, F., Depression as a risk factor for the onset of type 2 diabetes mellitus. A meta-analysis (2006) Diabetologia, 49 (5), pp. 837-845. , COI: 1:STN:280:DC%2BD283jsVWisw%3D%3D; Green, R.C., Cupples, L.A., Kurz, A., Auerbach, S., Go, R., Sadovnick, D., Duara, R., Farrer, L., Depression as a risk factor for Alzheimer disease: The MIRAGE study (2003) Arch. Neurol., 60 (5), pp. 753-759; Correll, C.U., Solmi, M., Veronese, N., Bortolato, B., Rosson, S., Santonastaso, P., Thapa-Chhetri, N., Stubbs, B., Prevalence, incidence and mortality from cardiovascular disease in patients with pooled and specific severe mental illness: A large-scale meta-analysis of 3,211,768 patients and 113,383,368 controls (2017) World Psychiatry, 16 (2), pp. 163-180; Lett, H.S., Blumenthal, J.A., Babyak, M.A., Sherwoob, A., Strauman, T., Robins, C., Newman, M.F., Depression as a risk factor for coronary artery disease: Evidence, mechanisms, and treatment (2004) Psychosom. Med., 66, pp. 305-315. , PID: 15184688; Kang, H.-J., Kim, S.-Y., Bae, K.-Y., Kim, S.-W., Shin, I.-S., Yoon, J.-S., Kim, J.-M., Comorbidity of depression with physical disorders: Research and clinical implications (2015) Chonnam Med.

J., 51 (1), pp. 8-18; Roth, D.L., Haley, W.E., Sheehan, O.C., Liu, C., Clay, O.J., Rhodes, J.D., Judd, S.E., Dhamoon, M., Depressive symptoms after ischemic stroke: Population-based comparisons of patients and caregivers with matched controls (2020) Stroke, 51 (1), pp. 54-60; Davidson, K.W., Alcántara, C., Miller, G.E., Selected psychological comorbidities in coronary heart disease: Challenges and grand opportunities (2018) Am. Psychol., 73 (8), pp. 1019-1030; Kvam, S., Kleppe, C.L., Nordhus, I.H., Hovland, A., Exercise as a treatment for depression: A meta-analysis (2016) J. Affect. Disord., 202, pp. 67-86; Li, J.M., Zhang, Y., Su, W.J., Liu, L.L., Gong, H., Peng, W., Jiang, C.L., Cognitive behavioral therapy for treatment-resistant depression: A systematic review and meta-analysis (2018) Psychiatry Res., 268, pp. 243-250; Gordon, B.R., McDowell, C.P., Hallgren, M., Meyer, J.D., Lyons, M., Herring, M.P., Association of efficacy of resistance exercise training with depressive symptoms meta-analysis and meta-regression: Analysis of randomized clinical trials (2018) JAMA Psychiatry, 75 (6), pp. 566-576; Möller, H., Henkel, V., (2005) What are the Most Effective Diagnostic and Therapeutic Strategies for the Management of Depression in Specialist Care?, , WHO Regional Office for Europe; DeRubeis, R.J., Siegle, G.J., Hollon, S.D., Cognitive therapy versus medication for depression: Treatment outcomes and neural mechanisms (2008) Nat. Rev. Neurosci., 9 (10), pp. 788-796. , COI: 1:CAS:528:DC%2BD1cXhtFeit7bE; Hagen, R., Hjemdal, O., Solem, S., Kennair, L.E.O., Nordahl, H.M., Fisher, P., Wells, A., Metacognitive therapy for depression in adults: A waiting list randomized controlled trial with six months follow-up (2017) Front. Psychol., 8, p. 31; Donker, T., Batterham, P.J., Warmerdam, L., Bennett, K., Bennett, A., Cuijpers, P., Griffiths, K.M., Christensen, H., Predictors and moderators of response to internet-delivered interpersonal psychotherapy and cognitive behavior therapy for depression (2013) J. Affect. Disord., 151 (1), pp. 343-351; Seligman, L., Reichenberg, L., (2014) Theories of Counseling and Psychotherapy: Systems, Strategies, and Skills, pp. 354-356. , Pearson Higher Education; Lin, H.P., Lin, H.Y., Lin, W.L., Huang, A.C.W., Effects of stress, depression, and their interaction on heart rate, skin conductance, finger temperature, and respiratory rate: Sympathetic-parasympathetic hypothesis of stress and depression (2011) J. Clin. Psychol., 67 (10), pp. 1080-1091; Kemp, A.H., Quintana, D.S., Felmingham, K.L., Matthews, S., Jelinek, H.F., Depression, comorbid anxiety disorders, and heart rate variability in physically healthy, unmedicated patients: Implications for cardiovascular risk (2012) PLoS ONE, 7 (2). , COI: 1:CAS:528:DC%2BC38XjtFWisb0%3D; Sgoifo, A., Carnevali, L., Pico Alfonso, M.D.L.A., Amore, M., Autonomic dysfunction and heart rate variability in depression (2015) Stress, 18 (3), pp. 343-352; Brown, L., Karmakar, C., Gray, R., Jindal, R., Lim, T., Bryant, C., Heart rate variability alterations in late life depression: A meta-analysis (2018) J. Affect. Disord., 235, pp. 456-466; Kidwell, M., Ellenbroek, B.A., Heart and soul: Heart rate variability and major depression (2018) Behav. Pharmacol., 29 (2), pp. 152-164; Caldwell, Y.T., Steffen, P.R., Adding HRV biofeedback to psychotherapy increases heart rate variability and improves the treatment of major depressive disorder (2017) Int. J. Psychophysiol., 2018 (131), pp. 96-101; Hartmann, R., Schmidt, F.M., Sander, C., Hegerl, U., Heart rate variability as indicator of clinical state in depression (2019) Front. Psychiatry, 9, p. 735; Kemp, A.H., Quintana, D.S., Gray, M.A., Felmingham, K.L., Brown, K., Gatt, J.M., Impact of depression and antidepressant treatment on heart rate variability: A review and meta-analysis (2010) Biol. Psychiatry, 67 (11), pp. 1067-1074; Dell'Acqua, C., Dal Bò, E., Messerotti Benvenuti, S., Palomba, D., Reduced heart rate variability is associated with vulnerability to depression (2020) J. Affect. Disord. Rep., 1, p. 100006; Lehrer, P.M.,

Vaschillo, E., Vaschillo, B., Resonant frequency biofeedback training to increase cardiac variability: Rationale and manual for training (2000) Appl. Psychophysiol. Biofeedback, 25, pp. 177-191. , COI: 1:STN:280:DC%2BD3cvksl2gsg%3D%3D; Lehrer, P.M., Gevirtz, R., Heart rate variability biofeedback: How and why does it work? (2014) Front. Psychol., 5, p. 756; Lin, I.M., Fan, S.Y., Yen, C.F., Yeh, Y.C., Tang, T.C., Huang, M.F., Liu, T.L., Tsai, Y.C., Heart rate variability biofeedback increased autonomic activation and improved symptoms of depression and insomnia among patients with major depression disorder (2019) Clin. Psychopharmacol. Neurosci., 17 (2), p. 222; Karavidas, M., (2008) Heart Rate Variability Biofeedback for Major Depression, , Biofeedback; Jimenez Morgan, S., Molina Mora, J.A., Effect of heart rate variability biofeedback on sport performance, a systematic review (2017) Appl. Psychophysiol., 42 (3), pp. 235-245; Yu, L.C., Lin, I.M., Fan, S.Y., Chien, C.L., Lin, T.H., One-year cardiovascular prognosis of the randomized, controlled, short-term heart rate variability biofeedback among patients with coronary artery disease (2018) Int. J. Behav. Med., 25 (3), pp. 271-282; Criswell, S.R., Sherman, R., Krippner, S., Cognitive behavioral therapy with heart rate variability biofeedback for adults with persistent noncombat-related posttraumatic stress disorder (2018) Perm. J.; Drury, R.L., Porges, S.W., Thayer, J.F., Ginsberg, J., Editorial: Heart rate variability, health and well-being: A systems perspective (2019) Front. Public Health, 7, p. 323; Yetwin, A., Marks, K., Bell, T., Gold, J., Heart rate variability biofeedback therapy for children and adolescents with chronic pain (2012) J. Pain, 13 (4), p. S93; Goessl, V.C., Curtiss, J.E., Hofmann, S.G., The effect of heart rate variability biofeedback training on stress and anxiety: A meta-analysis (2017) Psychol. Med., 47 (15), pp. 2578-2586. , COI: 1:STN:280:DC%2BC1crksleqtg%3D%3D; Lehrer, P., Kaur, K., Sharma, A., Shah, K., Huseby, R., Bhavsar, J., Zhang, Y., Heart rate variability biofeedback improves emotional and physical health and performance: A systematic review and meta analysis (2020) Appl. Psychophysiol., 45, pp. 109-129; Climov, D., Lysy, C., Berteau, S., Dutrannois, J., Dereppe, H., Brohet, C., Melin, J., Biofeedback on heart rate variability in cardiac rehabilitation: Practical feasibility and psycho-physiological effects (2014) Acta Cardiol., 69 (3), pp. 299-307; Meyer, P.W., Friederich, H.C., Zastrow, A., Breathe to ease—Respiratory biofeedback to improve heart rate variability and coping with stress in obese patients: A pilot study (2018) Ment. Health Prev., 11, pp. 41-46; Chang, W.L., Lee, J.T., Li, C.R., Davis, A.H.T., Yang, C.C., Chen, Y.J., Effects of heart rate variability biofeedback in patients with acute ischemic stroke: A randomized controlled trial (2020) Biol. Res. Nurs., 22 (1), pp. 34-44; Van der Zwan, J.E., Huizink, A.C., Lehrer, P.M., Koot, H.M., de Vente, W., The effect of heart rate variability biofeedback training on mental health of pregnant and non-pregnant women: A randomized controlled trial (2019) Int. J. Environ. Res. Public Health; Penzlin, A.I., Siepmann, T., Illigens, B.M.W., Weidner, K., Siepmann, M., Heart rate variability biofeedback in patients with alcohol dependence: A randomized controlled study (2015) Neuropsychiatr. Dis. Treat., 11, pp. 2619-2627; Swanson, K.S., Gevirtz, R.N., Brown, M., Spira, J., Guarneri, E., Stoletniy, L., The effect of biofeedback on function in patients with heart failure (2009) Appl. Psychophysiol., 34 (2), pp. 71-91; Van der Zwan, J.E., de Vente, W., Huizink, A.C., Bogels, S.M., de Bruin, E.I., Physical activity, mindfulness meditation, or heart rate variability biofeedback for stress reduction: A randomized controlled trial (2015) Appl. Psychophysiol., 40 (4), pp. 257-268; Gruzelier, J.H., Thompson, T., Redding, E., Brandt, R., Steffert, T., Application of alpha/theta neurofeedback and heart rate variability training to young contemporary dancers: State anxiety and

creativity (2014) Int. J. Psychophysiol., 93 (1), pp. 105-111. , COI: 1:STN:280:DC%2BC3snkslersQ%3D%3D; Windthorst, P., Mazurak, N., Kuske, M., Hipp, A., Giel, K.E., Enck, P., Niess, A., Teufel, M., Heart rate variability biofeedback therapy and graded exercise training in management of chronic fatigue syndrome: An exploratory pilot study (2017) J. Psychosom. Res., 93, pp. 6-13; Hallman, D.M., Olsson, E.M.G., Von Scheéle, B., Melin, L., Lyskov, E., Effects of heart rate variability biofeedback in subjects with stress-related chronic neck pain: A pilot study (2011) Appl. Psychophysiol., 36 (2), pp. 71-80; Amjadian, M., Bahrami Ehsan, H., Saboni, K., Vahedi, S., Rostami, R., Roshani, D., A pilot randomized controlled trial to assess the effect of Islamic spiritual intervention and of breathing technique with heart rate variability feedback on anxiety, depression and psycho-physiologic coherence in patients after coronary artery bypass surgery (2020) Ann. Gen. Psychiatry, 19 (1), pp. 1-10; Brinkmann, A.E., Press, S.A., Helmert, E., Hautzinger, M., Khazan, I., Vagedes, J., Comparing effectiveness of HRV-biofeedback and mindfulness for workplace stress reduction: A randomized controlled trial (2020) Appl. Psychophysiol., 45, p. 307; Tatschl, J.M., Hochfellner, S.M., Schwerdtfeger, A.R., Implementing mobile HRV biofeedback as adjunctive therapy during inpatient psychiatric rehabilitation facilitates recovery of depressive symptoms and enhances autonomic functioning short-term: A 1-year pre–post-intervention follow-up pilot study (2020) Front. Neurosci., 14, pp. 1-14; Burch, J.B., Ginsberg, J.P., McLain, A.C., Franco, R., Stokes, S., Susko, K., Hendry, W., O'Rourke, M.A., Symptom management among cancer survivors: Randomized pilot intervention trial of heart rate variability biofeedback (2020) Appl. Psychophysiol., 45 (2), pp. 99-108; Borenstein, M., Hedges, L.V., Higgins, J.P.T., Rothstein, H.R., (2009) Introduction to Meta-Analysis, , Wiley; (2013) Es: Compute Effect Sizes. R Package Version 0.2–2, , http://cran.r-project.org/web/packages/compute.es, Accessed 5 Sept 2020; Higgins, J.P.T., Altman, D.G., Gøtzsche, P.C., Jüni, P., Moher, D., Oxman, A.D., Savović, J., Sterne, J.A.C., The Cochrane Collaboration's tool for assessing risk of bias in randomised trials (2011) BMJ, 343, p. d5928; Higgins, J.P.T., Thompson, S.G., Deeks, J.J., Altman, D.G., Measuring inconsistency in meta-analyses (2003) Br. Med. J., 327 (7414), pp. 557-560; Egger, M., Smith, G.D., Schneider, M., Minder, C., Bias in meta-analysis detected by a simple, graphical test (1997) Br. Med. J., 315 (7109), pp. 629-634. , COI: 1:STN:280:DyaK2svls1KjtA%3D%3D; Lin, L., Chu, H., Quantifying publication bias in meta-analysis (2018) Biometrics, 74 (3), pp. 785-794; Viechtbauer, W., Conducting meta-analyses in R with the metafor (2010) J. Stat. Softw., 36 (3), pp. 1-48; Beck, A.T., Steer, R.A., Brown, G.K., (1996) Manual for the Beck Depression Inventory-II, , TX Psychol Corp; Lovibond, S.H., Lovibond, P.F., (1995) Manual for the Depression Anxiety Stress Scales, , Psychology Foundation of Australia; Spitzer, R.L., Kroenke, K., Williams, J.B.W., Validation and utility of a self-report version of PRIME-MD: The PHQ primary care study (1999) J. Am. Med. Assoc., 282 (18), pp. 1737-1744; Radloff, L.S., The CES-D scale: A self-report depression scale for research in the general population (1977) Appl. Psychol. Meas., 1, pp. 385-401; Read, J.R., Sharpe, L., Modini, M., Dear, B.F., Multimorbidity and depression: A systematic review and meta-analysis (2017) J. Affect. Disord., 15 (221), pp. 36-46; Cuijpers, P., Berking, M., Andersson, G., Quigley, L., Kleiboer, A., Dobson, K.S., A meta-analysis of cognitive-behavioural therapy for adult depression, alone and in comparison with other treatments (2013) Can. J. Psychiatry, 58 (7), pp. 376-385; Jacobson, N.C., Newman, M.G., Anxiety and depression as bidirectional risk factors for one another: A meta-analysis of longitudinal studies (2017) Psychol. Bull., 143 (11), pp. 1155-1200; Mitchell, A.J., Chan, M., Bhatti, H., Halton, M., Grassi,

L., Johansen, C., Meader, N., Prevalence of depression, anxiety, and adjustment disorder in oncological, haematological, and palliative-care settings: A meta-analysis of 94 interview-based studies (2011) Lancet Oncol., 12 (2), pp. 160-174; Mazzocco, K., Masiero, M., Carriero, M.C., Pravettoni, G., The role of emotions in cancer patients’ decision-making (2019) Ecancermedicalscience.; Pravettoni, G., Gorini, A., A P5 cancer medicine approach: Why personalized medicine cannot ignore psychology (2011) J. Eval. Clin. Pract., 17 (4), pp. 594-596; Kondylakis, H., Kazantzaki, E., Koumakis, L., Genitsaridi, I., Marias, K., Gorini, A., Mazzocco, K., Tsiknakis, M., Development of interactive empowerment services in support of personalised medicine (2014) Ecancermedicalscience.; Salvadore, G., Nugent, A.C., Lemaitre, H., Luckenbaugh, D.A., Tinsley, R., Cannon, D.M., Neumeister, A., Drevets, W.C., Prefrontal cortical abnormalities in currently depressed versus currently remitted patients with major depressive disorder (2011) Neuroimage, 54 (4), pp. 2643-2651},
correspondence_address1={Pizzoli, S.F.M.; Department of Oncology and Hemato-Oncology, Via Festa del Perdono 7, Italy; email: silviafrancescamaria.pizzoli@ieo.it},
publisher={Nature Research},
issn={20452322},
pubmed_id={33758260},
language={English},
abbrev_source_title={Sci. Rep.},
document_type={Article},
source={Scopus},
}

@ARTICLE{Vasquez-Muñoz2021,
author={Vasquez-Muñoz, M. and Arce-Alvarez, A. and von Igel, M. and Veliz, C. and Ruiz-Esquide, G. and Ramirez-Campillo, R. and Alvarez, C. and Ramirez-Velez, R. and Crespo, F.A. and Izquierdo, M. and Del Rio, R. and Andrade, D.C.},
title={Oscillatory pattern of glycemic control in patients with diabetes mellitus},
journal={Scientific Reports},
year={2021},
volume={11},
number={1},
doi={10.1038/s41598-021-84822-5},
art_number={5789},
note={cited By 0},
url={https://www.scopus.com/inward/record.uri?eid=2-s2.0-85102428978&doi=10.1038%2fs41598-021-84822-5&partnerID=40&md5=bf7fbe58c29996762d687ab82961ace0},
affiliation={Clínica Santa María, Santiago, Chile; Navarrabiomed, Complejo Hospitalario de Navarra (CHN), Universidad Pública de Navarra (UPNA), IdiSNA, Pamplona, Navarra, Spain; Escuela de Kinesiología, Facultad de Salud, Universidad Católica Silva Henríquez, Santiago, Chile; Centro de Investigación en Fisiología del Ejercicio, Facultad de Ciencias, Universidad Mayor, Santiago, Chile; Human Performance Laboratory, Quality of Life and Wellness Research Group, Department of Physical Activity Sciences, Universidad de Los Lagos, Osorno, Chile; CIBER of Frailty and Healthy Aging (CIBERFES), Instituto de Salud Carlos III, Madrid, Spain; DAiTA Lab, Facultad de Estudios Interdisciplinarios, Universidad Mayor, Santiago, Chile; Laboratory of Cardiorespiratory Control, Department of Physiology, Pontificia Universidad

Católica de Chile, Santiago, Chile; Centro de Envejecimiento Y Regeneración (CARE), Pontificia Universidad Católica de Chile, Santiago, Chile; Centro de Excelencia en Biomedicina de Magallanes (CEBIMA), Universidad de Magallanes, Punta Arenas, Chile; Centro de Investigación en Fisiología Y Medicina de Altura (MedAlt), Facultad de Ciencias de la Salud, Universidad de Antofagasta, Av. Universidad de Antofagasta #02800, Antofagasta, Chile},
abstract={Daily glucose variability is higher in diabetic mellitus (DM) patients which has been related to the severity of the disease. However, it is unclear whether glycemic variability displays a specific pattern oscillation or if it is completely random. Thus, to determine glycemic variability pattern, we measured and analyzed continuous glucose monitoring (CGM) data, in control subjects and patients with DM type-1 (T1D). CGM data was assessed for 6 days (day: 08:00–20:00-h; and night: 20:00–08:00-h). Participants (n = 172; age = 18–80 years) were assigned to T1D (n = 144, females = 65) and Control (i.e., healthy; n = 28, females = 22) groups. Anthropometry, pharmacologic treatments, glycosylated hemoglobin (HbA1c) and years of evolution were determined. T1D females displayed a higher glycemia at 10:00–14:00-h vs. T1D males and Control females. DM patients displays mainly stationary oscillations (deterministic), with circadian rhythm characteristics. The glycemia oscillated between 2 and 6 days. The predictive model of glycemia showed that it is possible to predict hyper and hypoglycemia (R2 = 0.94 and 0.98, respectively) in DM patients independent of their etiology. Our data showed that glycemic variability had a specific oscillation pattern with circadian characteristics, with episodes of hypoglycemia and hyperglycemia at day phases, which could help therapeutic action for this population. © 2021, The Author(s).},
references={Bloomgarden, Z., Questioning glucose measurements used in the International Diabetes Federation (IDF) Atlas (2016) J. Diabetes., 8, pp. 746-747; Riddle, M.C., Herman, W.H., The cost of diabetes care—An elephant in the room (2018) Diabetes Care, 41, pp. 929-932; Hippisley-Cox, J., Coupland, C., Diabetes treatments and risk of heart failure, cardiovascular disease, and all cause mortality: cohort study in primary care (2016) BMJ, 354, p. 3477; Sharma, A., Causes of death in a contemporary cohort of patients with type 2 diabetes and atherosclerotic cardiovascular disease: insights from the TECOS trial (2017) Diabetes Care, 40, pp. 1763-1770. , COI: 1:CAS:528:DC%2BC1cXitFGksLrO; Bergenstal, R.M., Response to comment on Bergenstal et al. glucose management indicator (GMI): A new term for estimating A1C from continuous glucose monitoring (2019) Diabetes Care, 41, pp. 2275-2280; Mian, Z., Hermayer, K.L., Jenkins, A., Continuous glucose monitoring: Review of an innovation in diabetes management (2019) Am. J. Med. Sci., 358, pp. 332-339; Petrie, J.R., Improving the clinical value and utility of CGM systems: issues and recommendations: A joint statement of the European Association for the Study of Diabetes and the American Diabetes Association Diabetes Technology Working Group (2017) Diabetologia, 60, pp. 2319-2328; Riddlesworth, T.D., Optimal sampling duration for continuous glucose monitoring to determine long-term glycemic control (2018) Diabetes Technol. Ther., 20, pp. 314-316. , COI: 1:CAS:528:DC%2BC1cXot1eqt7o%3D; Kovatchev, B., Cobelli, C., Glucose variability: Timing, risk analysis, and relationship to hypoglycemia in diabetes (2016) Diabetes Care, 39, pp. 502-510. , COI: 1:CAS:528:DC%2BC2sXhslaguw%3D%3D; Kovatchev, B.P., Metrics for glycaemic control—From HbA 1c to continuous glucose monitoring (2017) Nat. Rev. Endocrinol., 13, pp. 425-436. , COI: 1:CAS:528:DC%2BC2sXltl2rtrY%3D; Suh, S., Kim, J.H., Glycemic variability: How do we measure it and why is it important? (2015) Diabetes Metab. J., 39, pp. 273-282; Verma, N., (2018) Nutritional and Therapeutic Interventions for Diabetes and Metabolic Syndrome, pp. 263-268. ,

(ed. Bagchi, D.); Miranda-Massari, J.R., Metabolic correction in patients sample with diabetes: clinical outcomes and costs reductions (2016) Int. J. Diabetes Res., 5, pp. 92-101. , PID: 28936369; Sartore, G., The importance of HbA1c and glucose variability in patients with type 1 and type 2 diabetes: Outcome of continuous glucose monitoring (CGM) (2012) Acta Diabetol., 49, pp. S153-S160; Kuenen, J.C., Does glucose variability influence the relationship between mean plasma glucose and HbA1c levels in type 1 and type 2 diabetic patients? (2011) Diabetes Care, 34, pp. 1843-1847. , COI: 1:CAS:528:DC%2BC3MXhtFKis7jE; Sartore, G., Chilelli, N.C., Burlina, S., Lapolla, A., Association between glucose variability as assessed by continuous glucose monitoring (CGM) and diabetic retinopathy in type 1 and type 2 diabetes (2013) Acta Diabetol., 50, pp. 437-442. , COI: 1:CAS:528:DC%2BC3sXptlCnsLg%3D; Lawton, J., Blackburn, M., Allen, J., Patients' and caregivers' experiences of using continuous glucose monitoring to support diabetes self-management: qualitative study (2018) BMC Endocr. Disord., 18, p. 12. , COI: 1:STN:280:DC%2BC1MrjtFSrtg%3D%3D; Wang, C., Glucose fluctuations in subjects with normal glucose tolerance, impaired glucose regulation and newly diagnosed type 2 diabetes mellitus (2012) Clin. Endocrinol., 76, pp. 810-815. , COI: 1:CAS:528:DC%2BC38XhtVOmtbbN; Bellazzi, R., Abu-Hanna, A., Data mining technologies for blood glucose and diabetes management (2009) J. Diabetes Sci. Technol., 3, pp. 603-612; Saïd, E., Saïd, S.E., Unit-roots test for time-series data with a linear time trend (1991) JoE., 47, pp. 285-303; Guo, C., Association of age at menopause and type 2 diabetes: A systematic review and dose-response meta-analysis of cohort studies (2019) Prim. Care Diabetes., 13, pp. 301-309; Frontoni, S., Glucose variability: An emerging target for the treatment of diabetes mellitus (2013) Diabetes Res. Clin Pract., 102, pp. 86-95. , COI: 1:CAS:528:DC%2BC3sXhs1WjurbF; Brand, J.S., Age at menopause, reproductive life span, and type 2 diabetes risk: Results from the EPIC-InterAct study (2013) Diabetes Care, 36, pp. 1012-1019; Jiang, J., Association between age at natural menopause and risk of type 2 diabetes in postmenopausal women with and without obesity (2019) J. Clin. Endocrinol. Metab., 104, pp. 3039-3048; Johnston, J.D., Physiological links between circadian rhythms, metabolism and nutrition (2014) Exp. Physiol., 99, pp. 1133-1137; Gamble, K.L., Berry, R., Frank, S.J., Young, M.E., Circadian clock control of endocrine factors (2014) Nat. Rev. Endocrinol., 10, pp. 466-475; Wishah, R.A., Al-Khawaldeh, O.A., Albsoul, A.M., Impact of pharmaceutical care interventions on glycemic control and other health-related clinical outcomes in patients with type 2 diabetes: Randomized controlled trial (2015) Diabetes Metab. Syndr., 9, pp. 271-276; Agudo-Tabuenca, A., Gimeno-Orna, J.A., Sáenz-Abad, D., Assessment of the efficacy and safety of a protocol to manage glucocorticoid-induced hyperglycemia in diabetic patients during hospital stay (2019) Endocrinol. Diabetes Nutr., 66, pp. 353-360; Litty, A.C., Chaney, S., Glucocorticoid-induced hyperglycemia (2017) Nurse Pract., 42, pp. 8-11; Álvarez, C., Ramírez-Campillo, R., Lucia, A., Ramírez-Vélez, R., Izquierdo, M., Concurrent exercise training on hyperglycemia and comorbidities associated: Non-responders using clinical cutoff points (2019) Scand. J. Med. Sci. Sports., 29, pp. 952-967; Battelino, T., Clinical targets for continuous glucose monitoring data interpretation: Recommendations from the international consensus on time in range (2019) Diabetes Care, 42, pp. 1593-1603; Li, X., Predictive modeling of hypoglycemia for clinical decision support in evaluating outpatients with diabetes mellitus (2019) Curr. Med. Res. Opin., 35, pp. 1885-1891; Ståhl, F., Johansson, R., Diabetes mellitus modeling and short-term prediction based on blood glucose measurements (2009) Math. Biosci., 217, pp. 101-117; Lai, H., Huang, H., Keshavjee, K., Guergachi, A., Gao, X., Predictive

models for diabetes mellitus using machine learning techniques (2019) BMC Endocr. Disord., 19, p. 101; Alejandro Nava, P., (2015) Procesamiento de series de tiempo, , Fondo de Cultura Economica; Reddy, N., Verma, N., Dungan, K., (2020) Monitoring technologies—continuous Glucose Monitoring, Mobile Technology, Biomarkers of Glycemic Control; Satya Krishna, S.V., Kota, S.K., Modi, K.D., Glycemic variability: Clinical implications (2013) Indian J. Endocrinol. Metab., 17, pp. 611-619; Mechanick, J.I., Diabetes-specific nutrition algorithm: a transcultural program to optimize diabetes and prediabetes care (2012) Curr. Diab. Rep., 12, pp. 180-194. , COI: 1:CAS:528:DC%2BC38XktFGrt7Y%3D; (2020) R: A Language and Environment for Statistical Computing., , https://www.R-project.org/, R Foundation for Statistical Computing, Vienna, Austria; Devasahayam, S.R., (2019) Signals and Systems in Biomedical Engineering: Physiological Systems Modeling and Signal Processing, , https://doi.org/10.1007/978-981-13-3531-0; Northrop, R.B., (2016) Signals and Systems Analysis in Biomedical Engineering, , CRC Press; Puntanen, S., Plane answers to complex questions: The theory of linear models, Fourth Edition by Ronald Christensen (2011) Int. Stat. Rev., 79, pp. 504-505; Kamath, G., Tzamos, C., Anaconda: A non-adaptive conditional sampling algorithm for distribution testing (2019) Proceedings of the Thirtieth Annual ACM-SIAM Symposium on Discrete Algorithms, pp. 679-693},
correspondence_address1={Andrade, D.C.; Centro de Investigación en Fisiología Y Medicina de Altura (MedAlt), Av. Universidad de Antofagasta #02800, Chile; email: dcandrade@uc.cl},
publisher={Nature Research},
issn={20452322},
pubmed_id={33707491},
language={English},
abbrev_source_title={Sci. Rep.},
document_type={Article},
source={Scopus},
}

@ARTICLE{Kim2021,
author={Kim, T.B. and Ho, C.-T.B.},
title={Validating the moderating role of age in multi-perspective acceptance model of wearable healthcare technology},
journal={Telematics and Informatics},
year={2021},
volume={61},
doi={10.1016/j.tele.2021.101603},
art_number={101603},
note={cited By 0},
url={https://www.scopus.com/inward/record.uri?eid=2-s2.0-85102642892&doi=10.1016%2fj.tele.2021.101603&partnerID=40&md5=b57187fe71f8d0ca5c47f9ffdb5ce823},
affiliation={Graduate Institute of Technology Management, National Chung Hsing University, Taichung, 402, Taiwan},
abstract={The recent trend of aging population, not to mention the unprecedented pandemic, draws great attention from the general public about health concerns. Since healthcare information technology is different from non-healthcare information technology, additional contexts should be properly incorporated into technology acceptance research to accurately identify influential factors affecting the acceptance of wearable healthcare technology. Thus, we selectively reconfigured factors from health, privacy, and socio-demographic contexts to formulate a health-aware acceptance model. Then,

it was empirically analyzed using structural equation modeling. Based on the results, whereas privacy concerns were directly associated with intention to use wearable healthcare technology, health concerns were not. Moreover, age had a moderating effect on social influence and facilitating conditions. These findings suggest valuable insights that the adoption rate of healthcare technology is increased by 1) keeping personal information securely, 2) facilitating social interaction among users, and 3) offering intuitive user experience for elderly people. © 2021 Elsevier Ltd},
author_keywords={Aging population; Health belief model; Privacy calculus theory; Technology acceptance model; Unified theory of acceptance and use of technology; Wearable activity tracker},
keywords={Health care; User experience, Facilitating conditions; Healthcare information technology; Healthcare technology; Influential factors; Personal information; Social interactions; Structural equation modeling; Technology acceptance, Wearable technology},
references={Ahadzadeh, A.S., Pahlevan Sharif, S., Ong, F.S., Khong, K.W., Integrating health belief model and technology acceptance model: an investigation of health-related internet use (2015) J. Med. Internet Res., 17; Anderson, C.L., Agarwal, R., The digitization of healthcare: boundary risks, emotion, and consumer willingness to disclose personal health information (2011) Inf. Syst. Res., 22, pp. 469-490; Angosto, S., García-Fernández, J., Valantine, I., Grimaldi-Puyana, M., The intention to use fitness and physical activity apps: a systematic review (2020) Sustainability, 12, p. 6641; Bagozzi, R.P., Yi, Y., Specification, evaluation, and interpretation of structural equation models (2012) J. Acad. Mark. Sci., 40, pp. 8-34; Barkley, J.E., Lepp, A., Santo, A., Glickman, E., Dowdell, B., The relationship between fitness app use and physical activity behavior is mediated by exercise identity (2020) Comput. Hum. Behav., 108; Bhattacherjee, A., Hikmet, N., Reconceptualizing organizational support and its effect on information technology usage: Evidence from the health care sector (2008) J. Comput. Inf. Syst., 48, pp. 69-76; Botsis, T., Hartvigsen, G., Current status and future perspectives in telecare for elderly people suffering from chronic diseases (2008) J. Telemed. Telecare, 14, pp. 195-203; Brown, V., Model of adoption of technology in households: a baseline model test and extension incorporating household life cycle (2005) MIS Q., 29, p. 399; Carrigan, M., Szmigin, I., In pursuit of youth: what's wrong with the older market? (1999) Marketing Intelligence & Planning, 17 (5), pp. 222-231; Casselman, J., Onopa, N., Khansa, L., Wearable healthcare: lessons from the past and a peek into the future (2017) Telematics Inform., 34, pp. 1011-1023; Cheung, M.L., Chau, K.Y., Lam, M.H.S., Tse, G., Ho, K.Y., Flint, S.W., Broom, D.R., Lee, K.Y., Examining consumers’ adoption of wearable healthcare technology: the role of health attributes (2019) Int. J. Environ. Res. Public. Health, 16, p. 2257; Choi, J., Kim, S., Is the smartwatch an IT product or a fashion product? A study on factors affecting the intention to use smartwatches (2016) Comput. Hum. Behav., 63, pp. 777-786; Chung, J.E., Park, N., Wang, H., Fulk, J., McLaughlin, M., Age differences in perceptions of online community participation among non-users: an extension of the Technology Acceptance Model (2010) Comput. Hum. Behav. Online Interactivity: Role of Technology in Behavior Change, 26, pp. 1674-1684; Czaja, S.J., Charness, N., Fisk, A.D., Hertzog, C., Nair, S.N., Rogers, W.A., Sharit, J., Factors Predicting the Use of Technology: Findings From the Center for Research and Education on Aging and Technology Enhancement (CREATE) (2006) Psychology and aging, 21 (2), pp. 333-352; Czaja, S.J., Sharit, J., Age Differences in Attitudes Toward Computers (1998) The Journals of Gerontology Series B: Psychological Sciences and Social Sciences, 53 (5), pp. 329-340; Davis, F.D.,

Perceived usefulness, perceived ease of use, and user acceptance of information technology (1989) MIS Q., 13, pp. 319-350; Demiris, G., Rantz, M.J., Aud, M.A., Marek, K.D., Tyrer, H.W., Skubic, M., Hussam, A.A., Older adults' attitudes towards and perceptions of 'smart home' technologies: a pilot study (2004) Med. Inform. Internet Med., 29, pp. 87-94; Deng, Z., Understanding public users' adoption of mobile health service (2013) Int. J. Mob. Commun., 11, p. 351; (2017), Department of Statistics Ministry of the Interior Statistics, Republic of China; Diefenbach, M.A., Weinstein, N.D., O'Reilly, J., Scales for assessing perceptions of health hazard susceptibility (1993) Health Educ. Res., 8, pp. 181-192; Dillard, A.J., Couper, M.P., Zikmund-Fisher, B.J., Perceived risk of cancer and patient reports of participation in decisions about screening: the DECISIONS study (2010) Med. Decis. Making, 30, pp. 96-105; Dinev, T., Hart, P., An extended privacy calculus model for E-commerce transactions (2006) Inf. Syst. Res., 17, pp. 61-80; El-Amrawy, F., Nounou, M.I., Are currently available wearable devices for activity tracking and heart rate monitoring accurate, precise, and medically beneficial? (2015) Healthc. Inform. Res., 21, pp. 315-320; Ellis, R.D., Allaire, J.C., Modeling Computer Interest in Older Adults: The Role of Age, Education, Computer Knowledge, and Computer Anxiety (1999) Human factors, 41 (3), pp. 345-355; Fornell, C., Larcker, D.F., Evaluating structural equation models with unobservable variables and measurement error (1981) J. Mark. Res., 18, pp. 39-50; Gao, Y., Li, H., Luo, Y., An empirical study of wearable technology acceptance in healthcare (2015) Ind. Manag. Data Syst., 115, pp. 1704-1723; (2020), pp. 1-3. , Garmin among hardest hit in major wave of ransomware attacks Comput. Fraud Secur. 2020; Greaves, F., Rozenblum, R., Chapter 18 - Social and Consumer Informatics (2017) Key Advances in Clinical Informatics, pp. 257-278. , A. Sheikh K.M. Cresswell A. Wright D.W. Bates Academic Press; Hair, J., Anderson, R., Tatham, R., Multivariate Data Analysis (1998), Prentice Hall Upper Saddle River, NJ; Heinz, M., Martin, P., Margrett, J.A., Yearns, M., Franke, W., Yang, H.I., Wong, J., Chang, C.K., Perceptions of Technology among Older Adults (2013) Journal of Gerontological Nursing, 39 (1), pp. 42-51; Hsiao, K.-L., Chen, C.-C., What drives smartwatch purchase intention? Perspectives from hardware, software, design, and value (2018) Telematics Inform., 35, pp. 103-113; Huang, J.-C., Innovative health care delivery system—a questionnaire survey to evaluate the influence of behavioral factors on individuals' acceptance of telecare (2013) Comput. Biol. Med., 43, pp. 281-286; Isakadze, N., Martin, S.S., How useful is the smartwatch ECG? (2020) Trends Cardiovasc. Med., 30, pp. 442-448; Janz, N.K., Becker, M.H., The health belief model: a decade later (1984) Health Educ. Q., 11, pp. 1-47; Jian, W.-S., Syed-Abdul, S., Sood, S.P., Lee, P., Hsu, M.-H., Ho, C.-H., Li, Y.-C., Wen, H.-C., Factors influencing consumer adoption of USB-based Personal Health Records in Taiwan (2012) BMC Health Serv. Res., 12, p. 277; Kang, H.G., Mahoney, D.F., Hoenig, H., Hirth, V.A., Bonato, P., Hajjar, I., Lipsitz, L.A., In situ monitoring of health in older adults: technologies and issues (2010) J. Am. Geriatr. Soc., 58, pp. 1579-1586; Karahoca, A., Karahoca, D., Aksöz, M., Examining intention to adopt to internet of things in healthcare technology products (2018) Kybernetes, 47, pp. 742-770; Kijsanayotin, B., Pannarunothai, S., Speedie, S.M., Factors influencing health information technology adoption in Thailand's community health centers: Applying the UTAUT model (2009) Int. J. Med. Inf., 78, pp. 404-416; Kim, D.D., Basu, A., Estimating the medical care costs of obesity in the united states: systematic review, meta-analysis, and empirical analysis (2016) Value Health, 19, pp. 602-613; Kim, J., Park, H.-A., Development of a health information technology acceptance model using consumers' health behavior intention (2012) J. Med. Internet Res., 14; Kim, K.J., Shin, D.-H., An acceptance model for

smart watches: implications for the adoption of future wearable technology (2015) Internet Res., 25, pp. 527-541; Krasnova, H., Veltri, N.F., Günther, O., Self-disclosure and privacy calculus on social networking sites: the role of culture: intercultural dynamics of privacy calculus (2012) Bus. Inf. Syst. Eng., 4, pp. 127-135; Kruk, J., Physical activity and health (2009) Asian Pac. J. Cancer Prev., 10, pp. 721-728; Laufer, R.S., Wolfe, M., Privacy as a concept and a social issue: a multidimensional developmental theory (1977) J. Soc. Issues, 33, pp. 22-42; Lee, H., Home IoT resistance: extended privacy and vulnerability perspective (2020) Telematics Inform., 49; Lee, I.-M., Shiroma, E.J., Lobelo, F., Puska, P., Blair, S.N., Katzmarzyk, P.T., Effect of physical inactivity on major non-communicable diseases worldwide: an analysis of burden of disease and life expectancy (2012) The Lancet, 380, pp. 219-229; Li, H., Wu, J., Gao, Y., Shi, Y., Examining individuals' adoption of healthcare wearable devices: an empirical study from privacy calculus perspective (2016) Int. J. Med. Inf., 88, pp. 8-17; Lin, Y.-Y., Huang, C.-S., Aging in Taiwan: building a society for active aging and aging in place (2016) The Gerontologist, 56, pp. 176-183; Lu, J., Yao, J.E., Yu, C.-S., Personal innovativeness, social influences and adoption of wireless Internet services via mobile technology (2005) J. Strateg. Inf. Syst., 14, pp. 245-268; Lu, W.-H., Lee, W.-J., Chen, L.-K., Hsiao, F.-Y., Comparisons of annual health care utilization, drug consumption, and medical expenditure between the elderly and general population in Taiwan (2016) J. Clin. Gerontol. Geriatr., 7, pp. 44-47; Lunney, A., Cunningham, N.R., Eastin, M.S., Wearable fitness technology: a structural investigation into acceptance and perceived fitness outcomes (2016) Comput. Hum. Behav., 65, pp. 114-120; Maddox, T., (2016), https://www.techrepublic.com/article/smartwatch-sales-to-hit-17-8-billion-in-2020-but-business-use-lagging/, Smartwatch sales to hit $17.8 billion in 2020, but business use lagging [WWW Document]. TechRepublic. URL (accessed 10.9.20); Mair, F.S., Goldstein, P., Shiels, C., Roberts, C., Angus, R., O'connor, J., Haycox, A., Capewell, S., (2006), Recruitment difficulties in a home telecare trial. J. Telemed. Telecare 12, 26–28. 10.1258/135763306777978371; Mauvais-Jarvis, F., Epidemiology of Gender Differences in Diabetes and Obesity (2017), pp. 3-8. , Mauvais-Jarvis, F. (Ed.), Sex and Gender Factors Affecting Metabolic Homeostasis, Diabetes and Obesity, Advances in Experimental Medicine and Biology. Springer International Publishing, Cham, 10.1007/978-3-319-70178-3_1; McConnell, M.V., Turakhia, M.P., Harrington, R.A., King, A.C., Ashley, E.A., Mobile health advances in physical activity, fitness, and atrial fibrillation: moving hearts (2018) J. Am. Coll. Cardiol., 71, pp. 2691-2701; Michael, K., Michael, M.G., Apple watch temptation: just visit the app store (2015) IEEE Consum. Electron. Mag., 4, pp. 120-122; Miles, L., Physical activity and health (2007) Nutr. Bull., 32, pp. 314-363; Miltgen, C.L., Popovič, A., Oliveira, T., Determinants of end-user acceptance of biometrics: Integrating the “Big 3” of technology acceptance with privacy context (2013) Decis. Support Syst., 56, pp. 103-114; Mitzner, T.L., Boron, J.B., Fausset, C.B., Adams, A.E., Charness, N., Czaja, S.J., Dijkstra, K., Sharit, J., Older adults talk technology: Technology usage and attitudes (2010) Computers in Human Behavior, 26 (6), pp. 1710-1721; Mokdad, A.H., Marks, J.S., Stroup, D.F., Gerberding, J.L., Actual Causes of Death in the United States, 2000 (2004) J. Am. Med. Assoc., 291, pp. 1238-1245; Mothersbaugh, D.L., Foxx, W.K., Beatty, S.E., Wang, S., Disclosure antecedents in an online service context: the role of sensitivity of information (2012) J. Serv. Res., 15, pp. 76-98; Nqweniso, S., Walter, C., du Randt, R., Aerts, A., Adams, L., Degen, J., Gall, S., Gerber, M., Prevention of overweight and hypertension through cardiorespiratory fitness and extracurricular sport participation among south

african schoolchildren (2020) Sustainability, 12, p. 6581; Pai, F.-Y., Huang, K.-I., Applying the technology acceptance model to the introduction of healthcare information systems (2011) Technol. Forecast. Soc. Change, 78, pp. 650-660; Park, E., Kim, K.J., An integrated adoption model of mobile cloud services: exploration of key determinants and extension of technology acceptance model (2014) Telematics Inform., 31, pp. 376-385; Park, S., Chung, K., Jayaraman, S., Wearables: Fundamentals, Advancements, and a Roadmap for the Future (2014), pp. 1-23. , Wearable Sensors: Fundamentals, Implementation and Applications. Elsevier, 10.1016/B978-0-12-418662-0.00001-5; Prentice-Dunn, S., Rogers, R.W., Protection motivation theory and preventive health: beyond the health belief model (1986) Health Educ. Res., 1, pp. 153-161; Rogers, R.W., (1983), pp. 153-176. , Cognitive and psychological processes in fear appeals and attitude change: A revised theory of protection motivation, in: Cacioppo, J.T., Petty, R.E. (Eds.), Social Psychophysiology: A Sourcebook. Guilford; Rohm, A.J., Milne, G.R., Just what the doctor ordered (2004) J. Bus. Res., 57, pp. 1000-1011; Rosenstock, I.M., Historical origins of the health belief model (1974) Health Educ. Monogr., 2, pp. 328-335; Saghafi-Asl, M., Aliasgharzadeh, S., Asghari-Jafarabadi, M., Factors influencing weight management behavior among college students: an application of the Health Belief Model (2020) PLoS ONE, 15; Sanders, C., Rogers, A., Bowen, R., Bower, P., Hirani, S., Cartwright, M., Fitzpatrick, R., Newman, S.P., Exploring barriers to participation and adoption of telehealth and telecare within the Whole System Demonstrator trial: a qualitative study (2012) BMC Health Serv. Res., 12, p. 220; Sauer, P.L., Dick, A., Using moderator variables in structural equation models (1993) NA - Advances in Consumer Research, pp. 636-640. , Leigh McAlister Michael L. Rothschild Association for Consumer Research Provo, UT; Schroeder, S.A., We can do better — improving the health of the American people (2007) N. Engl. J. Med., 357, pp. 1221-1228; Steele, R., Lo, A., Secombe, C., Wong, Y.K., Elderly persons' perception and acceptance of using wireless sensor networks to assist healthcare (2009) Int. J. Med. Inf., 78, pp. 788-801; Sun, Y., Wang, N., Guo, X., Peng, Z., Understanding the acceptance of mobile health services: a comparison and integration of alternative models (2013) J. Electron. Commer. Res., 14, pp. 183-200; Sun, Y., Wang, N., Shen, X.-L., Zhang, J.X., Location information disclosure in location-based social network services: Privacy calculus, benefit structure, and gender differences (2015) Comput. Hum. Behav., 52, pp. 278-292; Susan, A.B., Viswanath, V., (2005), Model of Adoption of Technology in Households: A Baseline Model Test and Extension Incorporating Household Life Cycle. MIS Q. 29, 399–426. 10.2307/25148690; Tacken, M., Marcellini, F., Mollenkopf, H., Ruoppila, I., Szeman, Z., Use and acceptance of new technology by older people. Findings of the international MOBILATE survey: 'Enhancing mobility in later life' (2005) Gerontechnology, 3 (3), pp. 126-137; van der Heijden, User acceptance of hedonic information systems (2004) MIS Q., 28, p. 695; Venkatesh, V., Morris, M.G., Davis, G.B., Davis, F.D., User acceptance of information technology: toward a unified view (2003) MIS Q., 27, pp. 425-478; Venkatesh, V., Thong, J.Y., Xu, X., Consumer acceptance and use of information technology: extending the unified theory of acceptance and use of technology (2012) MIS Q., 36, pp. 157-178; Walsh, K., Callan, A., Perceptions, preferences, and acceptance of information and communication technologies in older-adult community care settings in ireland: a case-study and ranked-care program analysis (2011) Ageing Int., 36, pp. 102-122; https://www.healthit.gov/faq/what-health-it, What is Health IT? | HealthIT.gov [WWW Document], n.d. URL (accessed 12.7.20); Wu, L.-H., Wu, L.-C., Chang, S.-C., Exploring consumers' intention to accept smartwatch (2016) Comput. Hum. Behav., 64, pp. 383-392; Yi, M.Y., Jackson, J.D., Park, J.S., Probst, J.C.,

Understanding information technology acceptance by individual professionals: toward an integrative view (2006) Inf. Manage., 43, pp. 350-363; Zhang, M., Luo, M., Nie, R., Zhang, Y., Technical attributes, health attribute, consumer attributes and their roles in adoption intention of healthcare wearable technology (2017) Int. J. Med. Inf., 108, pp. 97-109},
correspondence_address1={Kim, T.B.; Graduate Institute of Technology Management, Taiwan; email: briankim@smail.nchu.edu.tw},
publisher={Elsevier Ltd},
issn={07365853},
coden={TEINE},
language={English},
abbrev_source_title={Telematics Inf},
document_type={Article},
source={Scopus},
}

@ARTICLE{Tong2021,
author={Tong, H.L. and Quiroz, J.C. and Kocaballi, A.B. and Fat, S.C.M. and Dao, K.P. and Gehringer, H. and Chow, C.K. and Laranjo, L.},
title={Personalized mobile technologies for lifestyle behavior change: A systematic review, meta-analysis, and meta-regression},
journal={Preventive Medicine},
year={2021},
volume={148},
doi={10.1016/j.ypmed.2021.106532},
art_number={106532},
note={cited By 0},
url={https://www.scopus.com/inward/record.uri?eid=2-s2.0-85103653312&doi=10.1016%2fj.ypmed.2021.106532&partnerID=40&md5=9dd87ebbd45cbad64aeee78fa391554b},
affiliation={Westmead Applied Research Centre, Faculty of Medicine and Health, University of Sydney, Sydney, Australia; Centre for Health Informatics, Australian Institute of Health Innovation, Macquarie University, Sydney, Australia; Centre for Big Data Research in Health, University of New South Wales, Sydney, Australia; School of Computer Science, University of Technology Sydney, Sydney, Australia; Department of Biomedical Science, Macquarie University, Sydney, Australia; Capital Health Network, Canberra, Australia},
abstract={Given that the one-size-fits-all approach to mobile health interventions have limited effects, a personalized approach might be necessary to promote healthy behaviors and prevent chronic conditions. Our systematic review aims to evaluate the effectiveness of personalized mobile interventions on lifestyle behaviors (i.e., physical activity, diet, smoking and alcohol consumption), and identify the effective key features of such interventions. We included any experimental trials that tested a personalized mobile app or fitness tracker and reported any lifestyle behavior measures. We conducted a narrative synthesis for all studies, and a meta-analysis of randomized controlled trials. Thirty-nine articles describing 31 interventions were included (n = 77,243, 64% women). All interventions personalized content and rarely personalized other features. Source of data included system-captured (12 interventions), user-reported (11 interventions) or both (8 interventions). The meta-analysis showed a moderate positive effect on lifestyle behavior outcomes (standardized difference in means [SDM] 0.663, 95% CI 0.228 to 1.10). A meta-regression model including source of data found that interventions that used system-captured data for personalization were associated with higher effectiveness than those that used user-reported data (SDM 1.48, 95% CI 0.76 to

2.19). In summary, the field is in its infancy, with preliminary evidence of the potential efficacy of personalization in improving lifestyle behaviors. Source of data for personalization might be important in determining intervention effectiveness. To fully exploit the potential of personalization, future high-quality studies should investigate the integration of multiple data from different sources and include personalized features other than content. © 2021 Elsevier Inc.},
author_keywords={Fitness trackers[MeSH]; Health behavior[MeSH]; Mobile applications[MeSH]; Personalization; Tailoring},
references={Afshin, A., Babalola, D., Mclean, M., Yu, Z., Ma, W., Chen, C.Y., Information technology and lifestyle: a systematic evaluation of internet and mobile interventions for improving diet, physical activity, obesity, tobacco, and alcohol use (2016) J. Am. Heart Assoc., 5 (9); Arigo, D., Jake-Schoffman, D.E., Wolin, K., Beckjord, E., Hekler, E.B., Pagoto, S.L., The history and future of digital health in the field of behavioral medicine (2019) J. Behav. Med., 42 (1), pp. 67-83; Bertholet, N., Godinho, A., Cunningham, J.A., Smartphone application for unhealthy alcohol use: pilot randomized controlled trial in the general population (2019) Drug Alcohol Depend., 195, pp. 101-105; Blom, J., Monk, A., Theory of personalization of appearance: why users personalize their PCs and mobile phones (2003) Human-Comput. Interact., 18 (3), pp. 193-228; Boerema, S., Van Velsen, L., Hermens, H., An intervention study to assess potential effect and user experience of an mHealth intervention to reduce sedentary behaviour among older office workers (2019) BMJ Health Care Inform, 26 (1); Borenstein, M., Common Mistakes in Meta-Analysis and how to Avoid them (2019), Biostat, Incorporated; Broekhuizen, K., Kroeze, W., van Poppel, M.N., Oenema, A., Brug, J., A systematic review of randomized controlled trials on the effectiveness of computer-tailored physical activity and dietary behavior promotion programs: an update (2012) Ann. Behav. Med., 44 (2), pp. 259-286; Buchan, K., Morgan, H.M., Using the Onitor® track for weight loss: a mixed methods study among overweight and obese women (2019) Health Inform. J., 1460458219890790; Businelle, M.S., Ma, P., Kendzor, D.E., Frank, S.G., Vidrine, D.J., Wetter, D.W., An ecological momentary intervention for smoking cessation: evaluation of feasibility and effectiveness (2016) J. Med. Internet Res., 18 (12); Cahan, E.M., Hernandez-Boussard, T., Thadaney-Israni, S., Rubin, D.L., Putting the data before the algorithm in big data addressing personalized healthcare (2019) NPJ. Digital Med., 2 (1), pp. 1-6; Chow, C.K., Redfern, J., Hillis, G.S., Thakkar, J., Santo, K., Hackett, M.L., Effect of lifestyle-focused text messaging on risk factor modification in patients with coronary heart disease: a randomized clinical trial (2015) JAMA., 314 (12), pp. 1255-1263; Collins, L.M., Murphy, S.A., Strecher, V., The multiphase optimization strategy (MOST) and the sequential multiple assignment randomized trial (SMART): new methods for more potent eHealth interventions (2007) Am. J. Prev. Med., 32 (5), pp. S112-S118; Dantzig, S., Bulut, M., Krans, M., van der Lans, A., de Ruyter, B., Enhancing physical activity through context-aware coaching (2018) Proceedings of the 12th EAI International Conference on Pervasive Computing Technologies for Healthcare; Davies, E.L., Lonsdale, A.J., Hennelly, S.E., Winstock, A.R., Foxcroft, D.R., Personalized digital interventions showed no impact on risky drinking in young adults: a pilot randomized controlled trial (2017) Alcohol Alcohol., 52 (6), pp. 671-676; De Bra, P., Challenges in user modeling and personalization (2017) IEEE Intell. Syst., 32 (5), pp. 76-80; Direito, A., Tooley, M., Hinbarji, M., Albatal, R., Jiang, Y., Whittaker, R., Tailored daily activity: an adaptive physical activity smartphone intervention (2019) Telemed. e-Health, 26 (4), pp. 426-437; Elbert, S.P., Dijkstra, A., Oenema, A., A mobile phone app intervention

targeting fruit and vegetable consumption: the efficacy of textual and auditory tailored health information tested in a randomized controlled trial (2016) J. Med. Internet Res., 18 (6); Everett, E., Kane, B., Yoo, A., Dobs, A., Mathioudakis, N., A novel approach for fully automated, personalized health coaching for adults with prediabetes: pilot clinical trial (2018) J. Med. Internet Res., 20 (2); Fan, H., Poole, M.S., What is personalization? Perspectives on the design and implementation of personalization in information systems (2006) J. Organ. Comput. Electron. Commer., 16 (3-4), pp. 179-202; Fanning, J., Roberts, S., Hillman, C.H., Mullen, S.P., Ritterband, L., McAuley, E., A smartphone "app"-delivered randomized factorial trial targeting physical activity in adults (2017) J. Behav. Med., 40 (5), pp. 712-729; Fitbit, What should I know about Fitbit Premium? n.d https://help.fitbit.com/articles/en_US/Help_article/2437, Available from; Forman, E., Goldstein, S., Zhang, F., Evans, B., Manasse, S., Butryn, M., OnTrack: development and feasibility of a smartphone app designed to predict and prevent dietary lapses (2018) Transl. Behav. Med., 9; Forman, E.M., Goldstein, S.P., Crochiere, R.J., Butryn, M.L., Juarascio, A.S., Zhang, F., Randomized controlled trial of OnTrack, a just-in-time adaptive intervention designed to enhance weight loss (2019) Transl. Behav. Med., 9 (6), pp. 989-1001; Fukuoka, Y., Gay, C.L., Joiner, K.L., Vittinghoff, E., A novel diabetes prevention intervention using a mobile app: a randomized controlled trial with overweight adults at risk (2015) Am. J. Prev. Med., 49 (2), pp. 223-237; Ghanvatkar, S., Kankanhalli, A., Rajan, V., User models for personalized physical activity interventions: scoping review (2019) JMIR mHealth and uHealth., 7 (1); Gonul, S., Namli, T., Huisman, S., Laleci Erturkmen, G.B., Toroslu, I.H., Cosar, A., An expandable approach for design and personalization of digital, just-in-time adaptive interventions (2018) J. Am. Med. Inform. Assoc., 26 (3), pp. 198-210; Hardeman, W., Houghton, J., Lane, K., Jones, A., Naughton, F., A systematic review of just-in-time adaptive interventions (JITAIs) to promote physical activity (2019) Int. J. Behav. Nutr. Phys. Activity., 16 (1), p. 31; Hébert, E.T., Ra, C.K., Alexander, A.C., Helt, A., Moisiuc, R., Kendzor, D.E., A Mobile just-in-time adaptive intervention for smoking cessation: pilot randomized controlled trial (2020) J. Med. Internet Res., 22 (3); Hermens, H., op den Akker, H., Tabak, M., Wijsman, J., Vollenbroek, M., Personalized coaching systems to support healthy behavior in people with chronic conditions (2014) J. Electromyogr. Kinesiol., 24 (6), pp. 815-826; Higgins, J.P., Green, S., Cochrane Handbook for Systematic Reviews of Interventions (2011), John Wiley & Sons; Höchsmann, C., Müller, O., Ambühl, M., Klenk, C., Königstein, K., Infanger, D., Novel smartphone game improves physical activity behavior in type 2 diabetes (2019) Am. J. Prev. Med., 57 (1), pp. 41-50; Höchsmann, C., Infanger, D., Klenk, C., Königstein, K., Walz, S.P., Schmidt-Trucksäss, A., Effectiveness of a behavior change technique–based smartphone game to improve intrinsic motivation and physical activity adherence in patients with type 2 diabetes: randomized controlled trial (2019) JMIR Serious Games., 7 (1); Huh, J., Cerrada, C.J., Dzubur, E., Dunton, G.F., Spruijt-Metz, D., Leventhal, A.M., Effect of a mobile just-in-time implementation intention intervention on momentary smoking lapses in smoking cessation attempts among Asian American young adults (2020) Transl. Behav. Med, 11 (1), pp. 216-225; Iacoviello, B.M., Steinerman, J.R., Klein, D.B., Silver, T.L., Berger, A.G., Luo, S.X., Clickotine, a personalized smartphone app for smoking cessation: initial evaluation (2017) JMIR mHealth and uHealth., 5 (4); Insel, T.R., Digital phenotyping: Technology for a new Science of behavior (2017) JAMA., 318 (13), pp. 1215-1216; IntHout, J., Ioannidis, J.P., Borm, G.F., The Hartung-Knapp-Sidik-Jonkman method for random effects meta-analysis

is straightforward and considerably outperforms the standard DerSimonian-Laird method (2014) BMC Med. Res. Methodol., 14 (1), p. 25; Kaner, E.F., Beyer, F.R., Garnett, C., Crane, D., Brown, J., Muirhead, C., Personalised digital interventions for reducing hazardous and harmful alcohol consumption in community-dwelling populations (2017) Cochrane Database Syst. Rev., 9; Kazemi, D.M., Borsari, B., Levine, M.J., Li, S., Lamberson, K.A., Matta, L.A., A systematic review of the mHealth interventions to prevent alcohol and substance abuse (2017) J. Health Commun., 22 (5), pp. 413-432; Kelly, M.P., Barker, M., Why is changing health-related behaviour so difficult? (2016) Public Health, 136, pp. 109-116; Khatri, A., Shastri, D., Tsiamyrtzis, P., Uyanik, I., Akleman, E., Pavlidis, I., Effects of simple personalized goals on the usage of a physical activity app (2016) Proceedings of the 2016 CHI Conference Extended Abstracts on Human Factors in Computing Systems; King, A.C., Hekler, E.B., Grieco, L.A., Winter, S.J., Sheats, J.L., Buman, M.P., Harnessing different motivational frames via mobile phones to promote daily physical activity and reduce sedentary behavior in aging adults (2013) PLoS One, 8 (4); King, A.C., Hekler, E.B., Grieco, L.A., Winter, S.J., Sheats, J.L., Buman, M.P., Effects of three motivationally targeted mobile device applications on initial physical activity and sedentary behavior change in midlife and older adults: a randomized trial (2016) PLoS One, 11 (6); Klasnja, P., Smith, S., Seewald, N.J., Lee, A., Hall, K., Luers, B., Efficacy of contextually tailored suggestions for physical activity: a micro-randomized optimization trial of HeartSteps (2019) Ann. Behav. Med., 53 (6), pp. 573-582; Klein, M., Mogles, N., Van Wissen, A., Intelligent mobile support for therapy adherence and behavior change (2014) J. Biomed. Inform., 51, pp. 137-151; Kocaballi, A.B., Berkovsky, S., Quiroz, J.C., Laranjo, L., Tong, H.L., Rezazadegan, D., The personalization of conversational agents in health care: systematic review (2019) J. Med. Internet Res., 21 (11); Korinek, E.V., Phatak, S.S., Martin, C.A., Freigoun, M.T., Rivera, D.E., Adams, M.A., Adaptive step goals and rewards: a longitudinal growth model of daily steps for a smartphone-based walking intervention (2018) J. Behav. Med., 41 (1), pp. 74-86; Krebs, P., Prochaska, J.O., Rossi, J.S., A meta-analysis of computer-tailored interventions for health behavior change (2010) Prev. Med., 51 (3-4), pp. 214-221; Kreuter, M.W., Strecher, V.J., Glassman, B., One size does not fit all: the case for tailoring print materials (1999) Ann. Behav. Med., 21 (4), p. 276; Kroeze, W., Werkman, A., Brug, J., A systematic review of randomized trials on the effectiveness of computer-tailored education on physical activity and dietary behaviors (2006) Ann. Behav. Med., 31 (3), pp. 205-223; Kvedar, J.C., Fogel, A.L., Elenko, E., Zohar, D., Digital medicine's march on chronic disease (2016) Nat. Biotechnol., 34 (3), p. 239; Laranjo, L., Ding, D., Heleno, B., Kocaballi, B., Quiroz, J.C., Tong, H.L., Do smartphone applications and activity trackers increase physical activity in adults? Systematic review, meta-analysis and metaregression (2020) Br. J. Sports Med., 0, pp. 1-13. , bjsports-2020-102892; Lau, Y., Chee, D.G.H., Chow, X.P., Cheng, L.J., Wong, S.N., Personalised eHealth interventions in adults with overweight and obesity: a systematic review and meta-analysis of randomised controlled trials (2020) Prev. Med., 132, p. 106001; Lee, W., Chae, Y.M., Kim, S., Ho, S.H., Choi, I., Evaluation of a mobile phone-based diet game for weight control (2010) J. Telemed. Telecare, 16 (5), pp. 270-275; Liang, T.-P., Lai, H.-J., Ku, Y.-C., Personalized content recommendation and user satisfaction: theoretical synthesis and empirical findings (2006) J. Manag. Inf. Syst., 23 (3), pp. 45-70; Lustria, M.L.A., Noar, S.M., Cortese, J., Van Stee, S.K., Glueckauf, R.L., Lee, J., A meta-analysis of web-delivered tailored health behavior change interventions (2013) J. Health Commun., 18 (9), pp. 1039-1069; Menictas, M., Rabbi, M., Klasnja, P., Murphy, S., Artificial

intelligence decision-making in mobile health (2019) Biochemist, 41 (5), pp. 20-24; Michie, S., Richardson, M., Johnston, M., Abraham, C., Francis, J., Hardeman, W., The behavior change technique taxonomy (v1) of 93 hierarchically clustered techniques: building an international consensus for the reporting of behavior change interventions (2013) Ann. Behav. Med., 46 (1), pp. 81-95; Michie, S., Wood, C.E., Johnston, M., Abraham, C., Francis, J., Hardeman, W., Behaviour change techniques: the development and evaluation of a taxonomic method for reporting and describing behaviour change interventions (a suite of five studies involving consensus methods, randomised controlled trials and analysis of qualitative data) (2015) Health Technol. Assess., 19 (99); Middelweerd, A., Mollee, J., Klein, M.M., Manzoor, A., Brug, J., te Velde, S.J., The use and effects of an app-based physical activity intervention "Active2Gether" in young adults: quasi-experimental trial (2020) JMIR Formative Res., 4 (1); Mitchell, M., White, L., Lau, E., Leahey, T., Adams, M.A., Faulkner, G., Evaluating the carrot rewards app, a population-level incentive-based intervention promoting step counts across two canadian provinces: Quasi-experimental study (2018) J. Med. Internet Res., 20 (9); Mitchell, M., Lau, E., White, L., Faulkner, G., Commercial app use linked with sustained physical activity in two Canadian provinces: a 12-month quasi-experimental study (2020) Int. J. Behav. Nutr. Phys. Act., 17 (1), pp. 1-9; Moher, D., Liberati, A., Tetzlaff, J., Altman, D.G., Group, P., Preferred reporting items for systematic reviews and meta-analyses: the PRISMA statement (2009) PLoS Med., 6 (7); Monteiro-Guerra, F.M., Rivera-Romero, O., Luque, L.F., Caulfield, B., Personalization in real-time physical activity coaching using mobile applications: a scoping review (2019) IEEE J. Biomed. Health Inform., 24 (6), pp. 1738-1751; Myskja, B.K., Steinsbekk, K.S., Personalized medicine, digital technology and trust: a Kantian account (2020) Med. Health Care Philos., 23 (4), pp. 577-587; Nahum-Shani, I., Smith, S.N., Spring, B.J., Collins, L.M., Witkiewitz, K., Tewari, A., Just-in-time adaptive interventions (JITAIs) in mobile health: key components and design principles for ongoing health behavior support (2016) Ann. Behav. Med., 52 (6), pp. 446-462; Nike, Nike Run Club n.d https://www.nike.com/au/nrc-app, Available from; Noar, S.M., Benac, C.N., Harris, M.S., Does tailoring matter? Meta-analytic review of tailored print health behavior change interventions (2007) Psychol. Bull., 133 (4), p. 673; O'Donnell, R., Richardson, B., Fuller-Tyszkiewicz, M., Staiger, P.K., Delivering personalized protective behavioral drinking strategies via a smartphone intervention: a pilot study (2019) Int. J. Behav. Med., 26 (4), pp. 401-414; op den Akker, H., Jones, V.M., Hermens, H.J., Tailoring real-time physical activity coaching systems: a literature survey and model (2014) User Model. User-Adap. Inter., 24 (5), pp. 351-392; Ouzzani, M., Hammady, H., Fedorowicz, Z., Elmagarmid, A., Rayyan—a web and mobile app for systematic reviews (2016) Syst. Rev., 5 (1), p. 210; Palmer, M., Sutherland, J., Barnard, S., Wynne, A., Rezel, E., Doel, A., The effectiveness of smoking cessation, physical activity/diet and alcohol reduction interventions delivered by mobile phones for the prevention of non-communicable diseases: a systematic review of randomised controlled trials (2018) PLoS One, 13 (1); Puig, E.P., Robles, N., Saigí-Rubió, F., Zamora, A., Moharra, M., Paluzie, G., Assessment of the efficacy, safety, and effectiveness of weight control and obesity management Mobile health interventions: systematic review (2019) JMIR mHealth and uHealth., 7 (10); Rabbi, M., Pfammatter, A., Zhang, M., Spring, B., Choudhury, T., Automated personalized feedback for physical activity and dietary behavior change with mobile phones: a randomized controlled trial on adults (2015) JMIR mHealth and uHealth., 3 (2); Rabbi, M., Aung, M.H., Zhang, M., Choudhury, T., MyBehavior: automatic personalized health feedback from user behaviors and

preferences using smartphones (2015) Proceedings of the 2015 ACM International Joint Conference on Pervasive and Ubiquitous Computing; Rabbi, M., Aung, M.S., Gay, G., Reid, M.C., Choudhury, T., Feasibility and acceptability of mobile phone–based auto-personalized physical activity recommendations for chronic pain self-management: pilot study on adults (2018) J. Med. Internet Res., 20 (10); Recio-Rodriguez, J.I., Agudo Conde, C., Calvo-Aponte, M.J., Gonzalez-Viejo, N., Fernandez-Alonso, C., Mendizabal-Gallastegui, N., The effectiveness of a smartphone application on modifying the intakes of macro and micronutrients in primary care: a randomized controlled trial. The EVIDENT II study (2018) Nutrients., 10 (10), p. 1473; Rodriguez, J.A., Clark, C.R., Bates, D.W., Digital health equity as a necessity in the 21st century cures act era (2020) JAMA., 323 (23), pp. 2381-2382; Romeo, A., Edney, S., Plotnikoff, R., Curtis, R., Ryan, J., Sanders, I., Can smartphone apps increase physical activity? Systematic review and meta-analysis (2019) J. Med. Internet Res., 21 (3); Ryan, K., Dockray, S., Linehan, C., A systematic review of tailored eHealth interventions for weight loss (2019) Digital Health., 5. , 2055207619826685; Samek, W., Wiegand, T., Müller, K.-R., Explainable artificial intelligence: Understanding, visualizing and interpreting deep learning models (2017) arXiv preprint, , arXiv:170808296; Shaw, J., Rudzicz, F., Jamieson, T., Goldfarb, A., Artificial intelligence and the implementation challenge (2019) J. Med. Internet Res., 21 (7); Shiffman, S., Stone, A.A., Hufford, M.R., Ecological momentary assessment (2008) Annu. Rev. Clin. Psychol., 4, pp. 1-32; Simons, D., De Bourdeaudhuij, I., Clarys, P., De Cocker, K., Vandelanotte, C., Deforche, B., A smartphone app to promote an active lifestyle in lower-educated working young adults: development, usability, acceptability, and feasibility study (2018) JMIR mHealth and uHealth., 6 (2); Simons, D., De Bourdeaudhuij, I., Clarys, P., De Cocker, K., Vandelanotte, C., Deforche, B., Effect and process evaluation of a smartphone app to promote an active lifestyle in lower educated working young adults: Cluster randomized controlled trial (2018) JMIR mHealth and uHealth, 6 (8); Spanakis, E.G., Santana, S., Tsiknakis, M., Marias, K., Sakkalis, V., Teixeira, A., Technology-based innovations to foster personalized healthy lifestyles and well-being: a targeted review (2016) J. Med. Internet Res., 18 (6); Stanaway, J.D., Afshin, A., Gakidou, E., Lim, S.S., Abate, D., Abate, K.H., Global, regional, and national comparative risk assessment of 84 behavioural, environmental and occupational, and metabolic risks or clusters of risks for 195 countries and territories, 1990-2017: a systematic analysis for the global burden of disease study 2017 (2018) Lancet, 392 (10159), pp. 1923-1994; Steinhubl, S.R., Muse, E.D., Topol, E.J., Can mobile health technologies transform health care? (2013) JAMA., 310 (22), pp. 2395-2396; Sterne, J.A., Savović, J., Page, M.J., Elbers, R.G., Blencowe, N.S., Boutron, I., RoB 2: a revised tool for assessing risk of bias in randomised trials (2019) BMJ., 366; Strava, Routes: New and improved n.d https://blog.strava.com/routes/, Available from; Thakkar, J., Kurup, R., Laba, T.-L., Santo, K., Thiagalingam, A., Rodgers, A., Mobile telephone text messaging for medication adherence in chronic disease: a meta-analysis (2016) JAMA Intern. Med., 176 (3), pp. 340-349; Thomas, J.G., Wing, R.R., Health-e-call, a smartphone-assisted behavioral obesity treatment: pilot study (2013) JMIR mHealth and uHealth., 1 (1); Tong, H.L., Laranjo, L., The use of social features in mobile health interventions to promote physical activity: a systematic review (2018) npj Digital Med., 1 (43); van Drongelen, A., Boot, C.R., Hlobil, H., Twisk, J.W., Smid, T., van der Beek, A.J., Evaluation of an mHealth intervention aiming to improve health-related behavior and sleep and reduce fatigue among airline pilots (2014) Scand. J. Work Environ. Health, pp. 557-568; Wongvibulsin, S., Martin, S.S., Saria, S., Zeger, S.L., Murphy, S.A.,

An individualized, data-driven digital approach for precision behavior change (2019) Am. J. Lifestyle Med., 14 (3), pp. 289-293; World Health Organization, Noncommunicable Diseases Progress Monitor 2017 (2017), WHO Geneva Contract No.:; Yardley, L., Spring, B.J., Riper, H., Morrison, L.G., Crane, D.H., Curtis, K., Understanding and promoting effective engagement with digital behavior change interventions (2016) Am. J. Prev. Med., 51 (5), pp. 833-842; Yusuf, S., Joseph, P., Rangarajan, S., Islam, S., Mente, A., Hystad, P., Modifiable risk factors, cardiovascular disease, and mortality in 155 722 individuals from 21 high-income, middle-income, and low-income countries (PURE): a prospective cohort study (2020) Lancet, 395 (10226), pp. 795-808; Zhou, M., Fukuoka, Y., Mintz, Y., Goldberg, K., Kaminsky, P., Flowers, E., Evaluating machine learning–based automated personalized daily step goals delivered through a mobile phone app: randomized controlled trial (2018) JMIR mHealth and uHealth., 6 (1); Zhou, M., Mintz, Y., Fukuoka, Y., Goldberg, K., Flowers, E., Kaminsky, P., Personalizing mobile fitness apps using reinforcement learning (2018) IUI Workshops; Zuckerman, O., Gal-Oz, A., Deconstructing gamification: evaluating the effectiveness of continuous measurement, virtual rewards, and social comparison for promoting physical activity (2014) Pers. Ubiquit. Comput., 18 (7), pp. 1705-1719},
correspondence_address1={Tong, H.L.; Westmead Applied Research Centre, Australia; email: hton5658@uni.sydney.edu.au},
publisher={Academic Press Inc.},
issn={00917435},
coden={PVTMA},
pubmed_id={33774008},
language={English},
abbrev_source_title={Prev. Med.},
document_type={Review},
source={Scopus},
}

@ARTICLE{Edmonds2021,
author={Edmonds, C.J. and Foglia, E. and Booth, P. and Fu, C.H.Y. and Gardner, M.},
title={Dehydration in older people: A systematic review of the effects of dehydration on health outcomes, healthcare costs and cognitive performance},
journal={Archives of Gerontology and Geriatrics},
year={2021},
volume={95},
doi={10.1016/j.archger.2021.104380},
art_number={104380},
note={cited By 0},
url={https://www.scopus.com/inward/record.uri?eid=2-s2.0-85101409162&doi=10.1016%2fj.archger.2021.104380&partnerID=40&md5=a47bbae2551077e15098570527643a01},
affiliation={School of Psychology, University of East London, Stratford Campus, Water Lane, London, E15 4LZ, United Kingdom; Centre for Affective Disorders, Institute of Psychiatry, Psychology and Neuroscience, King's College London, De Crespigny Park, London, SE5 8AF, United Kingdom; Department of Psychology, University of Westminster, 309 Regent Street, London, W1B 2UW, United Kingdom},
abstract={Objective: To systematically examine the effect of dehydration on health outcomes, identify associated financial costs and consider impacts on cognitive performance in older adults. Design: A systematic review of English-language articles via OVID using MEDLINE, PsychINFO, EMBASE, and others, to March 2018. Included studies examined the relationship between

hydration status and health, care costs or cognitive outcome. Setting: Cross sectional and cohort data from studies reporting on dehydration in older adults. Participants: Adults aged 60 years and older. Measurements: Independent quality ratings were assessed for all extracted articles. Results: Of 1684 articles screened, 18 papers (N = 33,707) met inclusion criteria. Participants were recruited from hospital settings, medical long-term care centres and the community dwelling population. Data were synthesised using a narrative summary. Mortality rates were higher in dehydrated patients. Furthermore, health outcomes, including frailty, bradyarrhythmia, transient ischemic attacks, oral health and surgery recovery are linked to and worsened by dehydration. Length of hospital stay, either as a principal or secondary diagnosis, is greater in those with dehydration, compared to those who are euhydrated. Finally, neurocognitive functioning may be impacted by dehydration. There are issues with study design, inconsistency in hydration status measurement and different measures used for outcome assessment. Conclusion: Dehydration in older people is associated with increased mortality, poorer course of illness and increased costs for health services. In addition, there is some, but sparse evidence that dehydration in older people is linked to poorer cognitive performance. Intervention studies should test strategies for reducing dehydration in older adults. © 2021 Elsevier B.V.},
author_keywords={Cognitive Performance; Dehydration; Healthcare; Older adults},
references={Abdallah, L., Remington, R., Houde, S., Zhan, L., Devereaux Melillio, K., Dehydration Reduction in Community-Dwelling Older Adults Perspectives of Community Health Care Providers (2009) Research in Gerontological Nursing, 2 (1), pp. 49-58; Ackland, G.L., Harrington, J., Downie, P., Holding, J.W., Singh-Ranger, D., Griva, K., Newman, S.P., Dehydration induced by bowel preparation in older adults does not result in cognitive dysfunction (2008) Anesthesia and Analgesia, 106 (3), pp. 924-929. , table of contents; Armstrong, L.E., Hydration Biomarkers During Daily Life (2012) Nutrition Today, 47 (4), pp. S3-S6; Bak, A., Tsiami, A., Greene, C., Methods of assessment of hydration status and their usefulness in detecting dehydration in the elderly (2017) Current Research in Nutrition and Food Science, 5 (2), pp. 43-54; Bar-David, Y., Urkin, J., Kozminsky, E., The effect of voluntary dehydration on cognitive functions of elementary school children (2005) Acta Paediatrica, 94 (11), pp. 1667-1673; Bar-David, Y., Urkin, J., Landau, D., Bar-David, Z., Pilpel, D., Voluntary dehydration among elementary school children residing in a hot arid environment (2009) Journal of Human Nutrition and Dietetics: The Official Journal of the British Dietetic Association, 22 (5), pp. 455-460; Benelam, B., Wyness, L., Hydration and health: a review (2010) Nutrition Bulletin, 35 (1), pp. 3-25. , Retrieved from; Bennett, J.A., Dehydration: Hazards and Benefits (2000) Geriatric Nursing, 21 (2), pp. 84-88; Benton, D., Dehydration influences mood and cognition: a plausible hypothesis? (2011) Nutrients, 3, pp. 555-573; Benton, D., Braun, H., Cobo, J.C., Edmonds, C.J., Elmadfa, I., El-Sharkawy, A.M., Watson, P., Executive summary and conclusions from the European Hydration Institute Expert Conference on human hydration, health, and performance (2015) Nutrition Reviews, 73, pp. 148-150; Benton, D., Burgess, N., The effect of the consumption of water on the memory and attention of children (2009) Appetite, 53 (1), pp. 143-146; Benton, D., Jenkins, K.T., Watkins, H.T., Young, H.A., Minor degree of hypohydration adversely influences cognition: A mediator analysis (2016) American Journal of Clinical Nutrition, 104 (3), pp. 603-612; Benton, D., Young, H.A., Do small differences in hydration status affect mood and mental performance? (2015) Nutrition Reviews, 73, pp. 83-96; Bhuyan, S.S.,

Lu, N., Chandak, A., Kim, H., Wyant, D., Bhatt, J., Chang, C.F., Use of Mobile Health Applications for Health-Seeking Behavior Among US Adults (2016) Journal of Medical Systems, 40, p. 153; Booth, P., Taylor, B., Edmonds, C.J., Water supplementation improves visual attention and fine motor skills in schoolchildren (2012) Education and Health, 30 (3), pp. 75-79; (2014), British Geriatrics Society. Fit for Frailty - Consensus best practice guidance for the care of older people living with frailty in community and outpatient settings; Chan, H.Y.L., Cheng, A., Cheung, S.S.S., Pang, W., Ma, W., Mok, L., Lee, D.T.F., Association between dehydration on admission and postoperative complications in older persons undergoing orthopaedic surgery (2018) Journal of Clinical Nursing, 27 (19-20), pp. 3679-3686; Coughlin, S.S., Whitehead, M., Sheats, J.Q., Mastromonico, J., Hardy, D., Smith, S.A., Smartphone Applications for Promoting Healthy Diet and Nutrition: A Literature Review (2016) Jacobs Journal of Food and Nutrition, 2 (3), pp. 21-35. , http://www.ncbi.nlm.nih.gov/pubmed/26819969%0Ahttp://www.pubmedcentral.nih.gov/articlerender.fcgi?artid=PMC4725321, Retrieved from; D'Anci, K.E.D., Vibhakar, A., Kanter, J.H., Mahoney, C.R., Taylor, H.A., Voluntary dehydration and cognitive performance in trained college athletes (2009) Perceptual and Motor Skills, 109, pp. 251-269; Edmonds, C.J., Burford, D., Should children drink more water?. The effects of drinking water on cognition in children (2009) Appetite, 52 (3), pp. 776-779; Edmonds, C.J., Crombie, R., Ballieux, H., Gardner, M.R., Dawkins, L., Water consumption, not expectancies about water consumption, affects cognitive performance in adults (2013) Appetite, 60 (1), pp. 148-153; Edmonds, C.J., Crombie, R., Gardner, M.R., Subjective thirst moderates changes in speed of responding associated Subjective thirst moderates changes in speed of responding associated with water consumption (2013) Frontiers in Human Neuroscience, (May); Edmonds, C.J., Crombie, R., Gardner, M.R., Subjective thirst moderates changes in speed of responding associated with water consumption (2013) Frontiers in Human Neuroscience, 7 (July), pp. 1-8; Edmonds, C.J., Crosbie, L., Fatima, F., Hussain, M., Jacob, N., Gardner, M., Dose-response effects of water supplementation on cognitive performance and mood in children and adults (2017) Appetite, 108, pp. 464-470; Edmonds, C.J., Harte, N., Gardner, M., How does drinking water affect attention and memory? The effect of mouth rinsing and mouth drying on children's performance (2018) Physiology & Behavior, 194, pp. 233-238; Edmonds, C.J., Jeffes, B., Does having a drink help you think? 6-7-Year-old children show improvements in cognitive performance from baseline to test after having a drink of water (2009) Appetite, 53 (3), pp. 469-472; El-Sharkawy, A.M., Sahota, O., Lobo, D.N., Acute and chronic effects of hydration status on health (2015) Nutrition Reviews; El-Sharkawy, A.M., Watson, P., Neal, K.R., Ljungqvist, O., Maughan, R.J., Sahota, O., Lobo, D.N., Hydration and outcome in older patients admitted to hospital (The HOOP prospective cohort study) (2015) Age and Ageing, 44 (6), pp. 943-947; Fadda, R., Rapinett, G., Grathwohl, D., Parisi, M., Fanari, R., Maria, C., Schmitt, J., Effects of drinking supplementary water at school on cognitive performance in children (2012) Appetite, 59 (3), pp. 730-737; Ferry, M., Strategies for ensuring good hydration in the elderly (2005) Nutrition Reviews, 63 (6), pp. 22-30; Foglia, E., Schoeler, T., Klamerus, E., Morgan, K., Bhattacharyya, S., Cannabis use and adherence to antipsychotic medication: a systematic review and meta-analysis (2017) Psychological Medicine, 47 (10), pp. 1691-1705; Frangeskou, M., Lopez-Valcarcel, B., Serra-Majem, L., Dehydration in the elderly: A review focused on economic burden (2015) Journal of Nutrition, Health and Aging, 19 (6), pp. 619-627; Godfrey, H., Cloete, J., Dymond, E., Long, A., An exploration of the hydration care of older people: A qualitative study (2012) International Journal of Nursing Studies, 49 (10), pp. 1200-1211;

Gopinathan, P.M., Pichan, G., Sharma, V.M., Role of dehydration in heat stress-induced variations in mental performance (1988) Archives of Environmental Health, 43 (1), pp. 15-17; Harada, C.N., Natelson Love, M.C., Triebel, K., Normal Cognitive Aging (2013) Clinics in Geriatric Medicine, 29 (4), pp. 737-752; Hodgkinson, B., Evans, D., Wood, J., Maintaining oral hydration in older adults: A systematic review (2003) International Journal of Nursing Practice, 9, pp. S19-S28; Hooper, L., Why, Oh Why, Are So Many Older Adults Not Drinking Enough Fluid? (2016) Journal of the Academy of Nutrition and Dietetics, 116 (5), pp. 774-778; Hooper, L., Abdelhamid, A., Attreed, N.J., Campbell, W., Channell, A., Chassagne, P., Hunter, P., Clinical symptoms, signs and tests for identification of impending and current water-loss dehydration in older people (2015) The Cochrane Database of Systematic Reviews, 4. , CD009647; Hooper, L., Bunn, D.K., Downing, A., Jimoh, F.O., Groves, J., Free, C., Shepstone, L., Which Frail Older People Are Dehydrated? the UK DRIE Study (2016) Journals of Gerontology - Series A Biological Sciences and Medical Sciences, 71 (10), pp. 1341-1347; Hooper, L., Whitelock, S., Bunn, D., Reducing dehydration in residents of care homes (2015) Nursing Times, 111 (34-35), pp. 16-19; Johnson, P., Waldreus, N., Hahn, R.G., Stenström, H., Sjöstrand, F., Fluid retention index predicts the 30-day mortality in geriatric care (2015) Scandinavian Journal of Clinical and Laboratory Investigation, 75 (6), pp. 444-451; Kenney, W.L., Chiu, P., Influence of age on thirst and fluid intake (2000) Medicine and Science in Sports and Exercise, 33 (9), pp. 1254-1532; Kenny, G.P., Yardley, J., Brown, C., Sigal, R.J., Jay, O., Heat stress in older individuals and patients with common chronic diseases (2010) Canadian Medical Association Journal, 182 (10), pp. 1053-1060; Khan, M.A., Hossain, F.S., Dashti, Z., Muthukumar, N., Causes and predictors of early re-admission after surgery for a fracture of the hip (2012) The Journal of Bone and Joint Surgery, 94-B, pp. 690-697; Knapton, S., Care home staff may leave pensioners dehydrated to stop incontinence, Oxford University warns (2015) The Daily Telegraph, p. 1. , https://www.telegraph.co.uk/news/science/science-news/11347733/Care-home-staff-may-leave-pensioners-dehydrated-to-stop-incontinence-Oxford-University-warns.html, Retrieved from; Kolasa, K.M., Lackey, C.J., Grandjean, A.C., Hydration and Health Promotion (2009) Nutrition Today, 44 (5), pp. 190-201; Lawrence, T., Hundreds have died of dehydration in care homes (2011) The Independent, p. 1; Lean, K., Nawaz, R.F., Jawad, S., Vincent, C., Reducing urinary tract infections in care homes by improving hydration (2019) BMJ Open Quality, 8 (3); Licchetta, M., Stelmach, M., Fiscal sustainability analytical paper: Fiscal sustainability and public spending on health (2016), Retrieved from _; Masento, N.A., Golightly, M., Field, D.T., Butler, L.T., van Reekum, C.M., Effects of hydration status on cognitive performance and mood (2014) British Journal of Nutrition, 111 (10), pp. 1841-1852; Maughan, R.J., (2003), Impact of mild dehydration on wellness and on exercise performance, 19–23. 10.1038/sj.ejcn.1601897; McCrow, J., Morton, M., Travers, C., Harvey, K., Eeles, E., Associations between dehydration, cognitive impairment, and frailty in older hospitalized patients: An exploratory study (2016) Journal of Gerontological Nursing, 42 (5), pp. 19-27; Mentes, J.C., Oral hydration in older adults (2006) American Journal of Nursing, 106 (6), pp. 40-49; Mentes, J.C., Wang, J., Measuring Risk for Dehydration in Nursing Home Residents: Evaluation of the Dehydration Risk Appraisal Checklist (2010) Research in Gerontological Nursing, 4 (2), pp. 148-157; Moher, D., Liberati, A., Tetzlaff, J., Altman, D.G., Grp, P., Preferred Reporting Items for Systematic Reviews and Meta-Analyses: The PRISMA Statement (Reprinted from Annals of Internal Medicine) (2009) Physical Therapy, 89 (9), pp. 873-880; Moody, O., Bennett, R.,

Elderly in care homes at risk of dehydration (2015) The Times, p. 1. , https://www.thetimes.co.uk/article/elderly-in-care-homes-at-risk-of-dehydration-cnzmm253fb9, Retrieved from; Mukand, J.A., Cai, C., Zielinski, A., Danish, M., Berman, J., The effects of dehydration on rehabilitation outcomes of elderly orthopedic patients (2003) Archives of Physical Medicine and Rehabilitation, 84 (1), pp. 58-61; Global Health and Aging (2011), NIH Publication No. 11-7737; O'Neill, P.A., Faragher, E.B., Davies, I., Wears, R., Mclean, K.A., Fairweather, D.S., Reduced survival with increasing plasma osmolality in elderly continuing-care patients (1990) Age and Ageing, 19 (1), pp. 68-71; Palmisano, P., Accogli, M., Zaccaria, M., Vergari, A., De Masi, G.D.L., Negro, L., De Blasi, S., Relationship between seasonal weather changes, risk of dehydration, and incidence of severe bradyarrhythmias requiring urgent temporary transvenous cardiac pacing in an elderly population (2014) International Journal of Biometeorology, 58, pp. 1513-1520; Patterson, S.M., VanderKaay, M.M., Shanholtzer, B.A., Patterson, C.A., Influence of acute fluid loading on stress-induced hemoconcentration and cardiovascular reactivity (2008) Journal of Behavioural Medicine, 31 (4), pp. 319-330; Paul, L., Brewster, S., Wyke, S., McFadyen, A.K., Sattar, N., Gill, J.M., Gray, C.M., Increasing physical activity in older adults using STARFISH, an interactive smartphone application (app); a pilot study (2017) Journal of Rehabilitation and Assistive Technologies Engineering, 4. , 205566831769623; Picetti, D., Foster, S., Pangle, A.K., Schrader, A., George, M., Wei, J.Y., Azhar, G., Hydration health literacy in the elderly (2017) Nutrition and Healthy Aging, 4 (3), pp. 227-237; Randolph, C., Repeatable Battery for the Assessment of Neuropsychological Status (1998), Psychological Corporation San Antonio, TX; Rasche, P., Wille, M., Bröhl, C., Theis, S., Schäfer, K., Knobe, M., Mertens, A., Prevalence of health app use among older adults in germany: National survey (2018) JMIR MHealth and UHealth, 6 (1), pp. 1-11; Rey, A., L'examen clinique en psychologie (1964), Presses Universitaires de France Paris; Rochette, L.M., Patterson, S.M., Hydration status and cardiovascular function: effects of hydration enhancement on cardiovascular function at rest and during psychological stress (2005) International Journal of Psychophysiology, 56 (1), pp. 81-91; Rodriguez, G.J., Cordina, S., Vazquez, G., Suri, M., Kirmani, J., Ezzeddine, M., Qureshi, A., The Hydration Influence on the Risk of Stroke (THIRST) Study (2009) Neurocritical Care, 10 (2), pp. 187-194; Sanderson, S., Tatt, I.D., Higgins, J.P.T., Tools for assessing quality and susceptibility to bias in observational studies in epidemiology: A systematic review and annotated bibliography (2007) International Journal of Epidemiology, 36 (3), pp. 666-676; Seymour, D.G., Henschke, P.J., Cape, R.D.T., Campbell, J., Acute confusional states and dementia in the elderly: The role of dehdyration/volume depletion, physical illness and age (1980) Age, 9 (August), pp. 137-146; Sharma, V.M., Sridharan, K., Pichan, G., Panwar, M.R., Influence of heat-stress induced dehydration on mental functions (1986) Ergonomics, 29 (6), pp. 791-799; Ship, J.A., Fischer, D.J., The relationship between dehydration and parotid salivary gland function in young and older healthy adults (1997) Journals of Gerontology: Medical Sciences, 52 (5), pp. M310-M319; Silva, F.G., The aging kidney: A review - Part II (2005) International Urology and Nephrology; Stookey, J.D., Pieper, C.F., Cohen, H.J., Is the prevalence of dehydration among community-dwelling older adults really low ? Informing current debate over the fluid recommendation for adults aged 70 þ years (2005) Public Health Nutrition, 8 (8), pp. 1275-1285; Strauss, E., Sherman, E.M.S., Spreen, O., A compendium of neuropsychological tests: Administration, norms and commentary (2006), 3rd editio Oxford University Press New York; Suhr, J.A., Hall, J., Patterson, S.M., Niinisto, R.T., The relation of hydration status to cognitive

performance in healthy older adults (2004) International Journal of
Psychophysiology, 53, pp. 121-125; Suhr, J.A., Patterson, S.M., Austin, A.M.,
Heffner, K.L., The relation of hydration status to declarative memory and
working memory in older adults (2010) The Journal of Nutrition, Health & Aging,
14 (10), pp. 10-13; Wakefield, B.J., Mentes, J.C., Holman, J.E., Culp, K.R.,
Risk Factors and Outcomes Associated with Hospital Admission for Dehydration
(2008) Rehabilitation Nursing, 33 (6), pp. 233-241; Waldstein, S.R., Brown,
J.R.P., Maier, K.J., Katzel, L.I., Diagnosis of hypertension and high blood
pressure levels negatively affect cognitive function in older adults (2005)
Annals of Behavioral Medicine: A Publication of the Society of Behavioral
Medicine, 29 (3), pp. 174-180; Warren, J.L., Bacon, W.E., Harris, T., Marshall
McBean, A., Foley, D.J., Phillips, M.S., The Burden and Outcomes Associated
with Dehydration among US Elderly, 1991 (1994) American Journal of Public
Health, 84 (8), pp. 1265-1270; Watso, J., Farquhar, W., Hydration status and
cardiovascular function (2002) Society for Psychophysiological Research,
Washington D.C., p. S64; Weinberg, A.D., Pals, J.K., Levesque, P.G., Beal,
L.F., Cunningham, T.J., Minaker, K.L., Dehydration and death during febrile
episodes in the nursing home (1994) Journal of the American Dietetic
Association, 42 (9), pp. 969-971; Wildenbos, G.A., Jaspers, M.W.M., Schijven,
M.P., Dusseljee-Peute, L.W., Mobile health for older adult patients: Using an
aging barriers framework to classify usability problems (2019) International
Journal of Medical Informatics, 124 (September 2018), pp. 68-77; Wittbrodt,
M.T., Millard-Stafford, M., Dehydration Impairs Cognitive Performance: A
Meta-analysis (2018) Medicine and Science in Sports and Exercise, 50 (11), pp.
2360-2368; Xiao, H., Barber, J., Campbell, E.S., Economic burden of dehydration
among hospitalized elderly patients (2004) American Journal of Health-System
Pharmacy, 61, pp. 2534-2541},
correspondence_address1={Edmonds, C.J.; School of Psychology, Stratford Campus,
Water Lane, United Kingdom; email: c.edmonds@uel.ac.uk},
publisher={Elsevier Ireland Ltd},
issn={01674943},
coden={AGGED},
language={English},
abbrev_source_title={Arch. Gerontol. Geriatr.},
document_type={Article},
source={Scopus},
}

@ARTICLE{Carpenter202179,
author={Carpenter, J.S. and Crouse, J.J. and Scott, E.M. and Naismith, S.L. and
Wilson, C. and Scott, J. and Merikangas, K.R. and Hickie, I.B.},
title={Circadian depression: A mood disorder phenotype},
journal={Neuroscience and Biobehavioral Reviews},
year={2021},
volume={126},
pages={79-101},
doi={10.1016/j.neubiorev.2021.02.045},
note={cited By 0},
url={https://www.scopus.com/inward/record.uri?eid=2-s2.0-85103421818&doi=10.101
6%2fj.neubiorev.2021.02.045&partnerID=40&md5=696b111cd67b782798c2a02e62564c03},
affiliation={Youth Mental Health and Technology Team, Brain & Mind Centre, The
University of Sydney, Camperdown, Australia; School of Medicine, University of
Notre Dame, Sydney, Australia; Charles Perkins Centre, University of Sydney,
Sydney, New South Wales, Australia; School of Psychology, University of Sydney,

Sydney, New South Wales, Australia; Academic Psychiatry, Institute of Neuroscience, Newcastle University, United Kingdom; Diderot University, Sorbonne City, Paris, France; Genetic Epidemiology Research Branch, Intramural Research Program, National Institute of Mental Health, Bethesda, United States; Department of Mental Health, Norwegian University of Science and Technology, Trondheim, Norway},
abstract={Major mood syndromes are among the most common and disabling mental disorders. However, a lack of clear delineation of their underlying pathophysiological mechanisms is a major barrier to prevention and optimised treatments. Dysfunction of the 24-h circadian system is a candidate mechanism that has genetic, behavioural, and neurobiological links to mood syndromes. Here, we outline evidence for a new clinical phenotype, which we have called ‘circadian depression’. We propose that key clinical characteristics of circadian depression include disrupted 24-h sleep-wake cycles, reduced motor activity, low subjective energy, and weight gain. The illness course includes early age-of-onset, phenomena suggestive of bipolarity (defined by bidirectional associations between objective motor and subjective energy/mood states), poor response to conventional antidepressant medications, and concurrent cardiometabolic and inflammatory disturbances. Identifying this phenotype could be clinically valuable, as circadian-targeted strategies show promise for reducing depressive symptoms and stabilising illness course. Further investigation of underlying circadian disturbances in mood syndromes is needed to evaluate the clinical utility of this phenotype and guide the optimal use of circadian-targeted interventions. © 2021},
author_keywords={Bipolar disorder; Circadian rhythms; Depression; Mood disorders; Sleep-wake cycles},
keywords={antidepressant agent; hydrocortisone; ketamine; lithium; melatonin, behavior therapy; bipolar disorder; body weight gain; circadian rhythm; clinical feature; core temperature; depression; heredity; human; inflammation; motor activity; onset age; phenotype; priority journal; Review; sex difference; sleep waking cycle; treatment response},
references={aan het Rot, M., Hogenelst, K., Schoevers, R.A., Mood disorders in everyday life: a systematic review of experience sampling and ecological momentary assessment studies (2012) Clin. Psychol. Rev., 32 (6), pp. 510-523; Abe, M., Herzog, E.D., Block, G.D., Lithium lengthens the circadian period of individual suprachiasmatic nucleus neurons (2000) Neuroreport, 11 (14), pp. 3261-3264; Akiskal, H.S., Benazzi, F., Atypical depression: a variant of bipolar II or a bridge between unipolar and bipolar II? (2005) J. Affect. Disord., 84 (2-3), pp. 209-217; Albrecht, U., Molecular connections between circadian clocks and mood-related behaviors (2019) J. Mol. Biol.; Alloy, L.B., Boland, E.M., Ng, T.H., Whitehouse, W.G., Abramson, L.Y., Low social rhythm regularity predicts first onset of bipolar spectrum disorders among at-risk individuals with reward hypersensitivity (2015) J. Abnorm. Psychol., 124 (4), pp. 944-952; Alloy, L.B., Ng, T.H., Titone, M.K., Boland, E.M., Circadian rhythm dysregulation in bipolar Spectrum disorders (2017) Curr. Psychiatry Rep., 19 (4); Amare, A.T., Vaez, A., Hsu, Y.H., Direk, N., Kamali, Z., Howard, D.M., Hartman, C.A., Bivariate genome-wide association analyses of the broad depression phenotype combined with major depressive disorder, bipolar disorder or schizophrenia reveal eight novel genetic loci for depression (2019) Mol. Psychiatry; American Psychiatric Association, Diagnostic and Statistical Manual of Mental Disorders (2013), 5th ed; Angst, J., Gamma, A., Sellaro, R., Zhang, H., Merikangas, K., Toward validation of atypical depression in the community: results of the Zurich cohort study (2002) J. Affect. Disord., 72 (2), pp. 125-138; Angst, J., Gamma, A., Benazzi, F., Silverstein, B., Ajdacic-Gross, V.,

Eich, D., Rössler, W., Atypical depressive syndromes in varying definitions (2006) Eur. Arch. Psychiatry Clin. Neurosci., 256 (1), pp. 44-54; Angst, J., Gamma, A., Benazzi, F., Ajdacic, V., Rössler, W., Melancholia and atypical depression in the Zurich study: epidemiology, clinical characteristics, course, comorbidity and personality (2007) Acta Psychiatr. Scand. Suppl., 433, pp. 72-84; Antypa, N., Vogelzangs, N., Meesters, Y., Schoevers, R., Penninx, B.W.J.H., Chronotype associations with depression and anxiety disorders in a large cohort study (2016) Depress. Anxiety, 33 (1), pp. 75-83; Armitage, R., Hoffmann, R., Emslie, G., Rintelman, J., Moore, J., Lewis, K., Rest-activity cycles in childhood and adolescent depression (2004) J. Am. Acad. Child Adolesc. Psychiatry, 43 (6), pp. 761-769; Arnow, B.A., Blasey, C., Williams, L.M., Palmer, D.M., Rekshan, W., Schatzberg, A.F., Rush, A.J., Depression Subtypes in Predicting Antidepressant Response: A Report From the iSPOT-D Trial (2015) Am. J. Psychiatry, 172 (8), pp. 743-750; Aschoff, J., Circadian control of body temperature (1983) J. Therm. Biol., 8, pp. 143-147; Au, J., Reece, J., The relationship between chronotype and depressive symptoms: a meta-analysis (2017) J. Affect. Disord., 218, pp. 93-104; Baeken, C., De Raedt, R., Santermans, L., Zeeuws, D., Vanderhasselt, M.A., Meers, M., Vanderbruggen, N., HF-rTMS treatment decreases psychomotor retardation in medication-resistant melancholic depression (2010) Prog. Neuropsychopharmacol. Biol. Psychiatry, 34 (4), pp. 684-687; Bailine, S., Fink, M., Knapp, R., Petrides, G., Husain, M.M., Rasmussen, K., Kellner, C.H., Electroconvulsive therapy is equally effective in unipolar and bipolar depression (2010) Acta Psychiatr. Scand., 121 (6), pp. 431-436; Barandas, R., Landgraf, D., McCarthy, M.J., Welsh, D.K., Circadian clocks as modulators of metabolic comorbidity in psychiatric disorders (2015) Curr. Psychiatry Rep., 17 (12), p. 98; Barbini, B., Colombo, C., Benazzi, F., Campori, E., Bellodi, L., Smeraldi, E., The unipolar-bipolar dichotomy and the response to sleep deprivation (1998) Psychiatry Res., 79, pp. 43-50; Barclay, J.L., Tsang, A.H., Oster, H., Interaction of central and peripheral clocks in physiological regulation (2012) Prog. Brain Res., 199, pp. 163-181; Bass, J., Circadian topology of metabolism (2012) Nature, 491 (7424), pp. 348-356; Bauer, M., Grof, P., Rasgon, N., Bschore, T., Glenn, T., Whybrow, P.C., Temporal relation between sleep and mood in patients with bipolar disorder (2006) Bipolar Disord., 8, pp. 160-167; Beccuti, G., Monagheddu, C., Evangelista, A., Ciccone, G., Broglio, F., Soldati, L., Bo, S., Timing of food intake: sounding the alarm about metabolic impairments? A systematic review (2017) Pharmacol. Res., 125, pp. 132-141; Belleville, G., Cousineau, H., Levrier, K., St-Pierre-Delorme, M.-È., Meta-analytic review of the impact of cognitive-behavior therapy for insomnia on concomitant anxiety (2011) Clin. Psychol. Rev., 31 (4), pp. 638-652; Bellivier, F., Geoffroy, P.-A., Etain, B., Scott, J., Sleep- and circadian rhythm-associated pathways as therapeutic targets in bipolar disorder (2015) Expert Opin. Ther. Targets, 19 (6), pp. 747-763; Benazzi, F., Should mood reactivity be included in the DSM-IV atypical features specifier? (2002) Eur. Arch. Psychiatry Clin. Neurosci., 252 (3), pp. 135-140; Benedetti, F., Serretti, A., Colombo, C., Lorenzi, C., Tubazio, V., Smeraldi, E., A glycogen synthase kinase 3-beta promoter gene single nucleotide polymorphism is associated with age at onset and response to total sleep deprivation in bipolar depression (2004) Neurosci. Lett., 368 (2), pp. 123-126; Benedetti, F., Dallaspezia, S., Fulgosi, M.C., Barbini, B., Colombo, C., Smeraldi, E., Phase advance is an actimetric correlate of antidepressant response to sleep deprivation and light therapy in bipolar depression (2007) Chronobiol. Int., 24 (5), pp. 921-937; Birkenhäger, T.K., van den Broek, W.W., Mulder, P.G., de Lely, A., One-year outcome of psychotic depression after successful electroconvulsive therapy (2005) J. ECT, 21 (4), pp. 221-226;

Birkenhäger, T.K., van den Broek, W.W., Wijkstra, J., Bruijn, J.A., van Os, E., Boks, M., Nolen, W.A., Treatment of unipolar psychotic depression: an open study of Lithium addition in refractory psychotic depression (2009) J. Clin. Psychopharmacol., 29 (5); Blake, M.J., Sheeber, L.B., Youssef, G.J., Raniti, M.B., Allen, N.B., Systematic review and meta-analysis of adolescent cognitive–behavioral sleep interventions (2017) Clin. Child Fam. Psychol. Rev.; Blanco, C., Vesga-Lopez, O., Stewart, J.W., Liu, S.M., Grant, B.F., Hasin, D.S., Epidemiology of major depression with atypical features: results from the National Epidemiologic Survey on Alcohol and Related Conditions (NESARC) (2012) J. Clin. Psychiatry, 73 (2), pp. 224-232; Boivin, D.B., Czeisler, C.A., Dijk, D.-J., Duffy, J.F., Folkard, S., Minors, D.S., Waterhouse, J.M., Complex interaction of the sleep-wake cycle and circadian phase modulates mood in healthy subjects (1997) Arch. Gen. Psychiatry, 54, pp. 145-152; Boland, E.M., Bender, R.E., Alloy, L.B., Conner, B.T., Labelle, D.R., Abramson, L.Y., Life events and social rhythms in bipolar spectrum disorders: an examination of social rhythm sensitivity (2012) J. Affect. Disord., 139 (3), pp. 264-272; Boland, E.M., Rao, H., Dinges, D.F., Smith, R.V., Goel, N., Detre, J.A., Gehrman, P.R., Meta-analysis of the antidepressant effects of acute sleep deprivation (2017) J. Clin. Psychiatry, 78 (8). , e1020-e1034; Brown, W.A., Treatment response in melancholia (2007) Acta Psychiatr. Scand., 115 (s433), pp. 125-129; Brunelin, J., Jalenques, I., Trojak, B., Attal, J., Szekely, D., Gay, A., Poulet, E., The efficacy and safety of low frequency repetitive transcranial magnetic stimulation for treatment-resistant depression: the results from a large multicenter french RCT (2014) Brain Stimul., 7 (6), pp. 855-863; Buhr, E.D., Yoo, S.-H., Takahashi, J.S., Temperature as a universal resetting cue for mammalian circadian oscillators (2010) Science, 330 (6002), pp. 379-385; Bumb, J.M., Enning, F., Mueller, J.K., van der List, T., Rohleder, C., Findeisen, P., Leweke, F.M., Differential melatonin alterations in cerebrospinal fluid and serum of patients with major depressive disorder and bipolar disorder (2016) Compr. Psychiatry, 68, pp. 34-39; Bunney, B.G., Bunney, W.E., Mechanisms of rapid antidepressant effects of sleep deprivation therapy: clock genes and circadian rhythms (2013) Biol. Psychiatry, 73 (12), pp. 1164-1171; Burgdorf, K.S., Trabjerg, B.B., Pedersen, M.G., Nissen, J., Banasik, K., Pedersen, O.B., Ullum, H., Large-scale study of Toxoplasma and Cytomegalovirus shows an association between infection and serious psychiatric disorders (2019) Brain Behav. Immun., 79, pp. 152-158; Burgess, H.J., Park, M., Wyatt, J.K., Rizvydeen, M., Fogg, L.F., Sleep and circadian variability in people with delayed sleep-wake phase disorder versus healthy controls (2017) Sleep Med., 34, pp. 33-39; Burish, M.J., Chen, Z., Yoo, S.-H., Emerging relevance of circadian rhythms in headaches and neuropathic pain (2019) Acta Physiol., 225 (1). , e13161; Burton, C., McKinstry, B., Szentagotai Tătar, A., Serrano-Blanco, A., Pagliari, C., Wolters, M., Activity monitoring in patients with depression: a systematic review (2013) J. Affect. Disord., 145 (1), pp. 21-28; Cain, S.W., McGlashan, E.M., Vidafar, P., Mustafovska, J., Curran, S.P.N., Wang, X., Phillips, A.J.K., Evening home lighting adversely impacts the circadian system and sleep (2020) Sci. Rep., 10 (1). , 19110; Carpenter, J.S., Robillard, R., Hickie, I.B., Variations in the sleep-wake cycle from childhood to adulthood: chronobiological perspectives (2015) Chronophysiology Ther., 5, pp. 37-49; Carpenter, J.S., Robillard, R., Hermens, D.F., Naismith, S.L., Gordon, C., Scott, E.M., Hickie, I.B., Sleep-wake profiles and circadian rhythms of core temperature and melatonin in young people with affective disorders (2017) J. Psychiatr. Res., 94, pp. 131-138; Carrier, J., Semba, K., Deurveilher, S., Drogos, L., Cyr-Cronier, J., Lord, C., Sekerovick, Z., Sex differences in age-related changes in the sleep-wake cycle (2017) Front.

Neuroendocrinol., 47, pp. 66-85; Carvalho, L.A., Gorenstein, C., Moreno, R.A., Markus, R.P., Melatonin levels in drug-free patients with major depression from the southern hemisphere (2006) Psychoneuroendocrinology, 31 (6), pp. 761-768; Cassone, V.M., Melatonin's role in vertebrate circadian rhythms (1998) Chronobiol. Int., 15 (5), pp. 457-473; Cervantes, P., Gelber, S., Kin, F.N., Nair, V.N., Schwartz, G., Circadian secretion of cortisol in bipolar disorder (2001) J. Psychiatry Neurosci., 26 (5), pp. 411-416; Chan, C.H., Janicak, P.G., Davis, J.M., Altman, E., Andriukaitis, S., Hedeker, D., Response of Psychotic and Nonpsychotic Depressed Patients to Tricyclic Antidepressants [Press Release] (1987); Chan, J.W.Y., Lam, S.P., Li, S.X., Yu, M.W.M., Chan, N.Y., Zhang, J., Wing, Y.-K., Eveningness and insomnia: independent risk factors of nonremission in major depressive disorder (2014) Sleep, 37 (5), pp. 911-917; Chan, J.W.Y., Lam, S.P., Li, S.X., Chau, S.W.H., Chan, S.Y., Chan, N.Y., Wing, Y.K., Adjunctive bright light treatment with gradual advance in unipolar major depressive disorder with evening chronotype – a randomized controlled trial (2020) Psychol. Med., pp. 1-10; Chaudhari, A., Gupta, R., Makwana, K., Kondratov, R., Circadian clocks, diets and aging (2017) Nutr. Healthy Aging, 4 (2), pp. 101-112; Cheng, P., Walch, O., Huang, Y., Mayer, C., Sagong, C., Cuamatzi Castelan, A., Drake, C.L., Predicting circadian misalignment with wearable technology: validation of wrist-worn actigraphy and photometry in night shift workers (2020) Sleep; Cho, C.-H., Choi, J.-H., Woo, H.G., Lee, H.-J., A genome-wide association study of bipolar disorder using a subphenotype: sleeplessness symptom of bipolar mania (2019) Chronobiology in Medicine, 1 (1), pp. 40-48; Chu, A.L., Stochl, J., Lewis, G., Zammit, S., Jones, P.B., Khandaker, G.M., Longitudinal association between inflammatory markers and specific symptoms of depression in a prospective birth cohort (2019) Brain Behav. Immun., 76, pp. 74-81; Cipriani, A., Furukawa, T.A., Salanti, G., Chaimani, A., Atkinson, L.Z., Ogawa, Y., Geddes, J.R., Comparative efficacy and acceptability of 21 antidepressant drugs for the acute treatment of adults with major depressive disorder: a systematic review and network meta-analysis (2018) Lancet; Clayton, P.J., Grove, W.M., Coryell, W., Keller, M., Hirschfeld, R., Fawcett, J., Follow-up and family study of anxious depression (1991) Am. J. Psychiatry, 148 (11), pp. 1512-1517; Corp, S.A., Gitlin, M.J., Altshuler, L.L., A review of the use of stimulants and stimulant alternatives in treating bipolar depression and major depressive disorder (2014) J. Clin. Psychiatry, 75 (9), pp. 1010-1018; Costemale-Lacoste, J.F., Colle, R., Martin, S., Asmar, K.E., Loeb, E., Feve, B., Corruble, E., Glycogen synthase kinase-3beta genetic polymorphisms and insomnia in depressed patients: a prospective study (2018) J. Affect. Disord., 240, pp. 230-236; Crasson, M., Kjiri, S., Colin, A., Kjiri, K., L'Hermite-Baleriaux, M., Ansseau, M., Legros, J.J., Serum melatonin and urinary 6-sulfatoxymelatonin in major depression (2004) Psychoneuroendocrinology, 29, pp. 1-12; Crowe, M., Beaglehole, B., Inder, M., Social rhythm interventions for bipolar disorder: a systematic review and rationale for practice (2016) J. Psychiatr. Ment. Health Nurs., 23 (1), pp. 3-11; Crowe, M., Inder, M., Douglas, K., Carlyle, D., Wells, H., Jordan, J., Porter, R., Interpersonal and social rhythm therapy for patients with major depressive disorder (2020) Am. J. Psychother., 73 (1), pp. 29-34; Cuijpers, P., Cristea, I.A., Karyotaki, E., Reijnders, M., Huibers, M.J.H., How effective are cognitive behavior therapies for major depression and anxiety disorders? A meta-analytic update of the evidence (2016) World Psychiatry, 15 (3), pp. 245-258; Cuijpers, P., Weitz, E., Lamers, F., Penninx, B.W., Twisk, J., DeRubeis, R.J., Hollon, S.D., Melancholic and atypical depression as predictor and moderator of outcome in cognitive behavior therapy and pharmacotherapy for adult depression (2017) Depress. Anxiety, 34 (3), pp.

246-256; Cunningham, J.E.A., Shapiro, C.M., Cognitive Behavioural Therapy for Insomnia (CBT-I) to treat depression: a systematic review (2018) J. Psychosom. Res., 106, pp. 1-12; D'Mello, C., Swain, M.G., Immune-to-brain communication pathways in inflammation-associated sickness and depression (2017) Inflammation-Associated Depression: Evidence, Mechanisms and Implications. Current Topics in Behavioral Neurosciences, 31, pp. 73-94. , R. Dantzer L. Capuron Springer Cham; Daimon, K., Yamada, N., Tsujimoto, T., Takahashi, S., Circadian rhythm abnormalities of deep body temperature in depressive disorders (1992) J. Affect. Disord., 26, pp. 191-198; Dallaspezia, S., Suzuki, M., Benedetti, F., Chronobiological therapy for mood disorders (2015) Curr. Psychiatry Rep., 17 (12); Danielsson, K., Markström, A., Broman, J.E., von Knorring, L., Jansson-Fröjmark, M., Delayed sleep phase disorder in a Swedish cohort of adolescents and young adults: prevalence and associated factors (2016) Chronobiol. Int., 33 (10), pp. 1331-1339; De Berardis, D., Fornaro, M., Serroni, N., Campanella, D., Rapini, G., Olivieri, L., Di Giannantonio, M., Agomelatine beyond borders: current evidences of its efficacy in disorders other than major depression (2015) Int. J. Mol. Sci., 16 (1), pp. 1111-1130; De Crescenzo, F., Economou, A., Sharpley, A.L., Gormez, A., Quested, D.J., Actigraphic features of bipolar disorder: a systematic review and meta-analysis (2017) Sleep Med. Rev., 33, pp. 58-69; Dibner, C., Schibler, U., Albrecht, U., The mammalian circadian timing system: organization and coordination of central and peripheral clocks (2010) Annu. Rev. Physiol., 72, pp. 517-549; Diefenbach, G.J., Bragdon, L., Goethe, J.W., Treating anxious depression using repetitive transcranial magnetic stimulation (2013) J. Affect. Disord., 151 (1), pp. 365-368; Dierckx, B., Heijnen, W.T., van den Broek, W.W., Birkenhäger, T.K., Efficacy of electroconvulsive therapy in bipolar versus unipolar major depression: a meta-analysis (2012) Bipolar Disord., 14 (2), pp. 146-150; Difrancesco, S., Lamers, F., Riese, H., Merikangas, K.R., Beekman, A.T.F., van Hemert, A.M., Penninx, B., Sleep, circadian rhythm, and physical activity patterns in depressive and anxiety disorders: a 2-week ambulatory assessment study (2019) Depress. Anxiety, 36 (10), pp. 975-986; Dijk, D.J., Duffy, J.F., Novel approaches for assessing circadian rhythmicity in humans: a review (2020) J. Biol. Rhythms, 35 (5), pp. 421-438; Dmitrzak-Weglarz, M.P., Pawlak, J.M., Maciukiewicz, M., Moczko, J., Wilkosc, M., Leszczynska-Rodziewicz, A., Hauser, J., Clock gene variants differentiate mood disorders (2015) Mol. Biol. Rep., 42 (1), pp. 277-288; Dong, L., Dolsen, M.R., Martinez, A.J., Notsu, H., Harvey, A.G., A transdiagnostic sleep and circadian intervention for adolescents: six-month follow-up of a randomized controlled trial (2020) J. Child Psychol. Psychiatry, 61 (6), pp. 653-661; Dooley, L.N., Kuhlman, K.R., Robles, T.F., Eisenberger, N.I., Craske, M.G., Bower, J.E., The role of inflammation in core features of depression: insights from paradigms using exogenously-induced inflammation (2018) Neurosci. Biobehav. Rev., 94, pp. 219-237; Drago, A., Monti, B., De Ronchi, D., Serretti, A., CRY1 variations impacts on the depressive relapse rate in a sample of bipolar patients (2015) Psychiatry Investig., 12 (1), pp. 118-124; Duarte Faria, A., Cardoso, T.A., Campos Mondin, T., Souza, L.D.M., Magalhaes, P.V.S., Patrick Zeni, C., Jansen, K., Biological rhythms in bipolar and depressive disorders: a community study with drug-naïve young adults (2015) J. Affect. Disord., 186, pp. 145-148; Duffy, J.F., Cain, S.W., Chang, A.-M., Phillips, A.J.K., Münch, M.Y., Gronfier, C., Czeisler, C.A., Sex difference in the near-24-hour intrinsic period of the human circadian timing system (2011) Proc. Natl. Acad. Sci., 108, pp. 15602-15608; Duffy, A., Horrocks, J., Doucette, S., Keown-Stoneman, C., McCloskey, S., Grof, P., The developmental trajectory of bipolar disorder (2014) Br. J. Psychiatry, 204 (2), pp. 122-128; Duncan, W.C., Jr., Slonena, E., Hejazi, N.S., Brutsche,

N., Yu, K.C., Park, L., Zarate, C.A., Jr., Motor-activity markers of circadian timekeeping are related to ketamine's rapid antidepressant properties (2017) Biol. Psychiatry, 82 (5), pp. 361-369; Duncan, W.C., Jr., Slonena, E.E., Hejazi, N.S., Brutsche, N., Park, L.T., Henter, I.D., Zarate, C.A., Jr., Are 24-hour motor activity patterns associated with continued rapid response to ketamine? (2018) Neuropsychiatr. Dis. Treat., 14, pp. 2739-2748; Dunster, G.P., Swendsen, J., Merikangas, K.R., Real-time mobile monitoring of bipolar disorder: a review of evidence and future directions (2020) Neuropsychopharmacology; Eastman, C.I., Tomaka, V.A., Crowley, S.J., Sex and ancestry determine the free-running circadian period (2017) J. Sleep Res., 26 (5), pp. 547-550; Eckel, R.H., Depner, C.M., Perreault, L., Markwald, R.R., Smith, M.R., McHill, A.W., Wright, K.P., Jr., Morning circadian misalignment during Short Sleep Duration Impacts Insulin Sensitivity (2015) Curr. Biol., 25 (22), pp. 3004-3010; Emens, J., Lewy, A., Kinzie, J.M., Arntz, D., Rough, J., Circadian misalignment in major depressive disorder (2009) Psychiatry Res., 168 (3), pp. 259-261; Faurholt-Jepsen, M., Brage, S., Vinberg, M., Christensen, E.M., Knorr, U., Jensen, H.M., Kessing, L.V., Differences in psychomotor activity in patients suffering from unipolar and bipolar affective disorder in the remitted or mild/moderate depressive state (2012) J. Affect. Disord., 141 (2-3), pp. 457-463; Fava, M., Alpert, J.E., Carmin, C.N., Wisniewski, S.R., Trivedi, M.H., Biggs, M.M., Rush, A.J., Clinical correlates and symptom patterns of anxious depression among patients with major depressive disorder in STAR*D (2004) Psychol. Med., 34 (7), pp. 1299-1308; Ferguson, A., Lyall, L.M., Ward, J., Strawbridge, R.J., Cullen, B., Graham, N., Smith, D.J., Genome-wide association study of circadian rhythmicity in 71,500 UK biobank participants and polygenic association with mood instability (2018) EBioMedicine, 35, pp. 279-287; Fernandez, D.C., Fogerson, P.M., Lazzerini Ospri, L., Thomsen, M.B., Layne, R.M., Severin, D., Hattar, S., Light affects mood and learning through distinct retina-brain pathways (2018) Cell, 175 (1). , 71-84 e18; Fink, M., Rush, A.J., Knapp, R., Rasmussen, K., Mueller, M., Rummans, T.A., the Consortium for Research in, E. C. T. S. G, DSM melancholic features are unreliable predictors of ECT response: a CORE publication (2007) J. ECT, 23 (3); Fischer, D., Lombardi, D.A., Marucci-Wellman, H., Roenneberg, T., Chronotypes in the US – influence of age and sex (2017) PLoS One, 12 (6). , e0178782; Foo, J.C., Trautmann, N., Sticht, C., Treutlein, J., Frank, J., Streit, F., Rietschel, M., Longitudinal transcriptome-wide gene expression analysis of sleep deprivation treatment shows involvement of circadian genes and immune pathways (2019) BioRxiv; Forbes, E.E., Dahl, R.E., Research Review: Altered reward function in adolescent depression: What, when and how? (2012) J. Child Psychol. Psychiatry, 53 (1), pp. 3-15; Foster, R.G., Hughes, S., Peirson, S.N., Circadian photoentrainment in mice and humans (2020) Biology, 9 (7), p. 180; Fountoulakis, K.N., Karamouzis, M., lacovides, A., Nimatoudis, J., Diakogiannis, J., Kaprinis, G., Bech, P., Morning and evening plasma melatonin and dexamethasone suppression test in patients with nonseasonal major depressive disorder from northern Greece (latitude 40-41.5) (2001) Biol. Psychiatry, 44, pp. 113-117; Frank, E., Kupfer, D.J., Thase, M.E., Mallinger, A.G., Swartz, H.A., Fagiolini, A.M., Monk, T., Two-year outcomes for interpersonal and social rhythm therapy in individuals with bipolar I disorder (2005) Arch. Gen. Psychiatry, 62 (9), pp. 996-1004; Frank, E., Swartz, H.A., Boland, E., Interpersonal and social rhythm therapy: an intervention addressing rhythm dysregulation in bipolar disorder (2007) Dialogues Clin. Neurosci., 9, pp. 325-332; Frank, E., Soreca, I., Swartz, H.A., Fagiolini, A.M., Mallinger, A.G., Thase, M.E., Kupfer, D.J., The role of interpersonal and social rhythm therapy in improving occupational functioning in patients with bipolar I

disorder (2008) Am. J. Psychiatry, 165, pp. 1559-1565; Fuller, P.M., Gooley, J.J., Saper, C.B., Neurobiology of the sleep-wake cycle: sleep architecture, circadian regulation, and regulatory feedback (2006) J. Biol. Rhythms, 21; Fuller-Thomson, E., Nimigon, J., Factors associated with depression among individuals with chronic fatigue syndrome: findings from a nationally representative survey (2008) Fam. Pract., 25 (6), pp. 414-422; Fullerton, D.T., Wenzel, F.J., Lohrenz, F.N., Fahs, H., Circadian rhythm of adrenal cortical activity in depression I. A comparison of depressed patients with normal subjects (1968) Arch. Gen. Psychiatry, 19, pp. 674-681; Gale, S.D., Berrett, A.N., Erickson, L.D., Brown, B.L., Hedges, D.W., Association between virus exposure and depression in US adults (2018) Psychiatry Res., 261, pp. 73-79; Gan, Z., Wu, X., Liao, Y., Wu, Y., He, Z., Yang, Z., Zhang, Q., The association between low-grade inflammation and the clinical features of bipolar disorder in Han Chinese population (2019) Psychoneuroendocrinology, 101, pp. 286-294; Gaudiano, B.A., Young, D., Chelminski, I., Zimmerman, M., Depressive symptom profiles and severity patterns in outpatients with psychotic vs nonpsychotic major depression (2008) Compr. Psychiatry, 49 (5), pp. 421-429; Gaynes, B.N., Lloyd, S.W., Lux, L., Gartlehner, G., Hansen, R.A., Brode, S., Lohr, K.N., Repetitive transcranial magnetic stimulation for treatment-resistant depression: a systematic review and meta-analysis (2014) J. Clin. Psychiatry, 75 (5), pp. 477-489. , quiz 489; Geoffroy, P.A., Bellivier, F., Scott, J., Etain, B., Seasonality and bipolar disorder: a systematic review, from admission rates to seasonality of symptoms (2014) J. Affect. Disord., 168, pp. 210-223; Geoffroy, P.A., Lajnef, M., Bellivier, F., Jamain, S., Gard, S., Kahn, J.P., Etain, B., Genetic association study of circadian genes with seasonal pattern in bipolar disorders (2015) Sci. Rep., 5, p. 10232; Geoffroy, P.A., Godin, O., Mahee, D., Henry, C., Aubin, V., Azorin, J.M., Seasonal pattern in bipolar disorders and cardio-vascular risk factors: a study from the FACE-BD cohort (2017) Chronobiol. Int., pp. 1-10; Geoffroy, P.A., Hoertel, N., Etain, B., Bellivier, F., Delorme, R., Limosin, F., Peyre, H., Insomnia and hypersomnia in major depressive episode: prevalence, sociodemographic characteristics and psychiatric comorbidity in a population-based study (2018) J. Affect. Disord., 226, pp. 132-141; Germain, A., Kupfer, D.J., Circadian rhythm disturbances in depression (2008) Hum. Psychopharmacol., 23 (7), pp. 571-585; Gill, S., Panda, S., A smartphone app reveals erratic diurnal eating patterns in humans that can Be modulated for health benefits (2015) Cell Metab., 22 (5), pp. 789-798; Glaus, J., von Kanel, R., Lasserre, A.M., Strippoli, M.F., Vandeleur, C.L., Castelao, E., Merikangas, K.R., Mood disorders and circulating levels of inflammatory markers in a longitudinal population-based study (2017) Psychol. Med., pp. 1-13; Glaus, J., Cui, L., Hommer, R., Merikangas, K.R., Association between mood disorders and BMI/overweight using a family study approach (2019) J. Affect. Disord., 248, pp. 131-138; Glozier, N., O'Dea, B., McGorry, P.D., Pantelis, C., Amminger, G.P., Hermens, D.F., Hickie, I.B., Delayed sleep onset in depressed young people (2014) BMC Psychiatry, 14 (1), p. 33; Godin, O., Henry, C., Leboyer, M., Azorin, J.M., Aubin, V., Bellivier, F., Etain, B., Sleep quality, chronotype and metabolic syndrome components in bipolar disorders during the remission period: results from the FACE-BD cohort (2017) Chronobiol. Int., pp. 1-11; Gonzalez, R., Gonzalez, S.D., Gupta, J., Mallawaarachchi, I., Suppes, T., The relationship between circadian gene single nucleotide polymorphisms and clinical and behavioral assessments of sleep and rhythms and course of illness characteristics in subjects with bipolar type I disorder (2019) Pers. Med. Psychiatry, 13-14, pp. 11-18; Goss, A.J., Kaser, M., Costafreda, S.G., Sahakian, B.J., Fu, C.H., Modafinil augmentation therapy in unipolar and

bipolar depression: a systematic review and meta-analysis of randomized controlled trials (2013) J. Clin. Psychiatry, 74 (11), pp. 1101-1107; Grandin, L.D., The social zeitgeber theory, circadian rhythms, and mood disorders: review and evaluation (2006) Clin. Psychol. Rev., 26 (6), pp. 679-694; Greco, C.M., Sassone-Corsi, P., Circadian blueprint of metabolic pathways in the brain (2019) Nat. Rev. Neurosci., 20 (2), pp. 71-82; Gregory, A.M., Rijsdijk, F.V., Eley, T.C., Buysse, D.J., Schneider, M.N., Parsons, M., Barclay, N.L., A longitudinal twin and sibling study of associations between insomnia and depression symptoms in young adults (2016) Sleep, 39 (11), pp. 1985-1992; Grierson, A.B., Hickie, I.B., Naismith, S.L., Hermens, D.F., Scott, E.M., Scott, J., Circadian rhythmicity in emerging mood disorders: state or trait marker? (2016) Int. J. Bipolar Disord., 4 (1), p. 3. , doi:10.1186/s40345-015-0043-z; Gunaratne, P., Lloyd, A.R., Vollmer-Conna, U., Mood disturbance after infection (2013) Aust. N. Z. J. Psychiatry, 47 (12), pp. 1152-1164; Gunn, P.J., Middleton, B., Davies, S.K., Revell, V.L., Skene, D.J., Sex differences in the circadian profiles of melatonin and cortisol in plasma and urine matrices under constant routine conditions (2016) Chronobiol. Int., 33 (1), pp. 39-50; Hallam, K.T., Olver, J.S., Horgan, J.E., McGrath, C., Norman, T.R., Low doses of lithium carbonate reduce melatonin light sensitivity in healthy volunteers (2005) Int. J. Neuropsychopharmacol., 8 (2), pp. 255-259; Hallam, K.T., Olver, J.S., Norman, T.R., Effect of sodium valproate on nocturnal melatonin sensitivity to light in healthy volunteers (2005) Neuropsychopharmacology, 30 (7), pp. 1400-1404; Hallam, K.T., Olver, J.S., Chambers, V., Begg, D.P., McGrath, C., Norman, T.R., The heritability of melatonin secretion and sensitivity to bright nocturnal light in twins (2006) Psychoneuroendocrinology, 31 (7), pp. 867-875; Hallam, K.T., Begg, D.P., Olver, J.S., Norman, T.R., Abnormal dose-response melatonin suppression by light in bipolar type I patients compared with healthy adult subjects (2009) Acta Neuropsychiatr., 21 (5), pp. 246-255; Hansen, B.H., Kolle, E., Dyrstad, S.M., Holme, I., Anderssen, S.A., Accelerometer-determined physical activity in adults and older people (2012) Med. Sci. Sports Exerc., 44 (2), pp. 266-272; Haraden, D.A., Mullin, B.C., Hankin, B.L., Internalizing symptoms and chronotype in youth: a longitudinal assessment of anxiety, depression and tripartite model (2019) Psychiatry Res., 272, pp. 797-805; Haraguchi, A., Fukuzawa, M., Iwami, S., Nishimura, Y., Motohashi, H., Tahara, Y., Shibata, S., Night eating model shows time-specific depression-like behavior in the forced swimming test (2018) Sci. Rep., 8 (1). , 1081; Harvey, A.G., Talbot, L.S., Gershon, A., Sleep disturbance in bipolar disorder across the lifespan (2009) Clin. Psychol. Sci. Pract., 16 (2), pp. 256-277; Harvey, A.G., Kaplan, K.A., Soehner, A.M., Interventions for sleep disturbance in bipolar disorder (2015) Sleep Med. Clin., 10 (1), pp. 101-105; Harvey, A.G., Hein, K., Dong, L., Smith, F.L., Lisman, M., Yu, S., Buysse, D.J., A transdiagnostic sleep and circadian treatment to improve severe mental illness outcomes in a community setting: study protocol for a randomized controlled trial (2016) Trials, 17 (1), p. 606; Harvey, A.G., Hein, K., Dolsen, M.R., Dong, L., Rabe-Hesketh, S., Gumport, N.B., Blum, D.J., Modifying the impact of eveningness chronotype (“Night-Owls”) in youth: a randomized controlled trial (2018) J. Am. Acad. Child Adolesc. Psychiatry, 57 (10), pp. 742-754; Hasler, B.P., Buysse, D.J., Kupfer, D.J., Germain, A., Phase relationships between core body temperature, melatonin, and sleep are associated with depression severity: further evidence for circadian misalignment in non-seasonal depression (2010) Psychiatry Res., 178 (1), pp. 205-207; Henriksen, T.E., Skrede, S., Fasmer, O.B., Schoeyen, H., Leskauskaite, I., Bjørke-Bertheussen, J., Lund, A., Blue-blocking glasses as additive treatment for mania: a randomized placebo-controlled trial (2016) Bipolar

Disord., 18 (3), pp. 221-232; Hein, M., Prevalence and risk factors of excessive daytime sleepiness in major depression: A study with 703 individuals referred for polysomnography (2019) J. Affect. Disord., 243, pp. 23-32; Herbert, J., Cortisol and depression: three questions for psychiatry (2013) Psychol. Med., 43 (3), pp. 449-469; Hickie, I.B., Carpenter, J.S., Scott, E.M., Adolescent-onset depressive disorders and inflammation (2018) Inflammation and Immunity in Depression: Basic Science and Clinical Applications, pp. 427-443. , B.T. Baune Elsevier; Himmelhoch, J.M., Thase, M.E., Mallinger, A.G., Houck, P., Tranylcypromine versus imipramine in anergic bipolar depression (1991) Am. J. Psychiatry, 148 (7), pp. 910-916; Hoof, E.V., Cluydts, R., De Meirleir, K., Atypical depression as a secondary symptom in chronic fatigue syndrome (2003) Med. Hypotheses, 61 (1), pp. 52-55; Hori, H., Koga, N., Hidese, S., Nagashima, A., Kim, Y., Higuchi, T., Kunugi, H., 24-h activity rhythm and sleep in depressed outpatients (2016) J. Psychiatr. Res., 77, pp. 27-34; Howard, D.M., Adams, M.J., Clarke, T.K., Hafferty, J.D., Gibson, J., Shirali, M., McIntosh, A.M., Genome-wide meta-analysis of depression identifies 102 independent variants and highlights the importance of the prefrontal brain regions (2019) Nat. Neurosci., 22 (3), pp. 343-352; Huang, L., Xi, Y., Peng, Y., Yang, Y., Huang, X., Fu, Y., Ren, C., A visual circuit related to habenula underlies the antidepressive effects of light therapy (2019) Neuron, 102 (1), pp. 128-142. , e128; Hühne, A., Welsh, D.K., Landgraf, D., Prospects for circadian treatment of mood disorders (2018) Ann. Med., 50 (8), pp. 637-654; Husain, M.M., McClintock, S.M., Rush, A.J., Knapp, R.G., Fink, M., Rummans, T.A., Kellner, C.H.,). The efficacy of acute electroconvulsive therapy in atypical depression (2008) J. Clin. Psychiatry, 69 (3), pp. 406-411; Hut, R.A., Beersma, D.G., Evolution of time-keeping mechanisms: early emergence and adaptation to photoperiod (2011) Philos. Trans. R. Soc. Lond., B, Biol. Sci., 366 (1574), pp. 2141-2154; Hutchison, A.T., Heilbronn, L.K., Metabolic impacts of altering meal frequency and timing - Does when we eat matter? (2016) Biochimie, 124, pp. 187-197; Ikeda, M., Saito, T., Kondo, K., Iwata, N., Genome-wide association studies of bipolar disorder: a systematic review of recent findings and their clinical implications (2018) Psychiatry Clin. Neurosci., 72 (2), pp. 52-63; Irwin, M.R., Olmstead, R., Carroll, J.E., Sleep disturbance, sleep duration, and inflammation: a systematic review and meta-analysis of cohort studies and experimental sleep deprivation (2016) Biol. Psychiatry, 80 (1), pp. 40-52; Jansson-Frojmark, M., Norell-Clarke, A., Cognitive behavioural therapy for insomnia in psychiatric disorders (2016) Curr. Sleep Med. Rep., 2 (4), pp. 233-240; Jauhar, P., Weller, M.P.I., Psychiatric morbidity and time zone changes: a study of patients from Heathrow airport (1982) Br. J. Psychiatry, 140, pp. 231-235; Johnson, S.L., Gershon, A., Starov, V., Is energy a stronger indicator of mood for those with bipolar disorder compared to those without bipolar disorder? (2015) Psychiatry Res., 230 (1), pp. 1-4; Jones, S.H., Hare, D.J., Evershed, K., Actigraphic assessment of circadian activity and sleep patterns in bipolar disorder (2005) Bipolar Disord., 7, pp. 176-186; Jones, S.E., Lane, J.M., Wood, A.R., van Hees, V.T., Tyrrell, J., Beaumont, R.N., Weedon, M.N., Genome-wide association analyses of chronotype in 697,828 individuals provides insights into circadian rhythms (2019) Nat. Commun., 10 (1), p. 343; Kalsbeek, A., van der Spek, R., Lei, J., Endert, E., Buijs, R.M., Fliers, E., Circadian rhythms in the hypothalamo–pituitary–adrenal (HPA) axis (2012) Mol. Cell. Endocrinol., 349 (1), pp. 20-29; Kan, C., Silva, N., Golden, S.H., Rajala, U., Timonen, M., Stahl, D., Ismail, K., A systematic review and meta-analysis of the association between depression and insulin resistance (2013) Diabetes Care, 36, pp. 480-489; Kaplan, K.A., Harvey, A.G., Hypersomnia across mood disorders: a review and synthesis (2009) Sleep Med. Rev., 13 (4),

pp. 275-285; Kasper, S., Hajak, G., Wulff, K., Hoogendijk, W.J., Montejo, A.L., Smeraldi, E., Baylé, F.J., Efficacy of the novel antidepressant agomelatine on the circadian rest-activity cycle and depressive and anxiety symptoms in patients with major depressive disorder: a randomized, double-blind comparison with sertraline (2010) J. Clin. Psychiatry, 71 (2), pp. 109-120; Katz, G., Knobler, H.Y., Laibel, Z., Strauss, Z., Durst, R., Time zone change and major psychiatric morbidity: the results of a 6-year study in Jerusalem (2002) Compr. Psychiatry, 43 (1), pp. 37-40; Keller, J., Flores, B., Gomez, R.G., Solvason, H.B., Kenna, H., Williams, G.H., Schatzberg, A.F., Cortisol circadian rhythm alterations in psychotic major depression (2006) Biol. Psychiatry, 60 (3), pp. 275-281; Kennedy, S.H., Kutcher, S.P., Ralevski, E., Brown, G.M., Nocturnal melatonin and 24-hour 6-sulphatoxymelatonin levels in various phases of bipolar affective disorder (1996) Psychiatry Res., 63, pp. 219-222; Kennedy, S.H., Lam, R.W., McIntyre, R.S., Tourjman, S.V., Bhat, V., Blier, P., Uher, R., Canadian network for mood and anxiety treatments (CANMAT) 2016 clinical guidelines for the management of adults with major depressive disorder: section 3. Pharmacological treatments (2016) Can. J. Psychiatry, 61 (9), pp. 540-560; Kervezee, L., Kosmadopoulos, A., Boivin, D.B., Metabolic and cardiovascular consequences of shift work: the role of circadian disruption and sleep disturbances (2018) Eur. J. Neurosci.; Kessler, R.C., Aguilar-Gaxiola, S., Alonso, J., Chatterji, S., Lee, S., Ormel, J., Wang, P.S., The global burden of mental disorders: an update from the WHO World Mental Health (WMH) Surveys (2011) Epidemiol. Psichiatr. Soc., 18 (1), pp. 23-33; Ketchesin, K.D., Zong, W., Hildebrand, M.A., Seney, M.L., Cahill, K.M., Scott, M.R., McClung, C.A., Diurnal rhythms across the human dorsal and ventral striatum (2021) Proc. Natl. Acad. Sci., 118 (2); Kishimoto, T., Chawla, J.M., Hagi, K., Zarate, C.A., Kane, J.M., Bauer, M., Correll, C.U., Single-dose infusion ketamine and non-ketamine N-methyl-d-aspartate receptor antagonists for unipolar and bipolar depression: a meta-analysis of efficacy, safety and time trajectories (2016) Psychol. Med., 46 (7), pp. 1459-1472; Klingenberg, L., Chaput, J.P., Holmback, U., Visby, T., Jennum, P., Nikolic, M., Sjodin, A., Acute sleep restriction reduces insulin sensitivity in adolescent boys (2013) Sleep, 36 (7), pp. 1085-1090; Kohler, O., Benros, M.E., Nordentoft, M., Farkouh, M.E., Iyengar, R.L., Mors, O., Krogh, J., Effect of anti-inflammatory treatment on depression, depressive symptoms, and adverse effects: a systematic review and meta-analysis of randomized clinical trials (2014) JAMA Psychiatry, 71 (12), pp. 1381-1391; Kohler, C.A., Freitas, T.H., Stubbs, B., Maes, M., Solmi, M., Veronese, N., Carvalho, A.F., Peripheral alterations in cytokine and chemokine levels after antidepressant drug treatment for major depressive disorder: systematic review and meta-analysis (2017) Mol. Neurobiol.; Krane-Gartiser, K., Henriksen, T.E.G., Morken, G., Vaaler, A., Fasmer, O.B., Actigraphic assessment of motor activity in acutely admitted inpatients with bipolar disorder (2014) PLoS One, 9 (2). , e89574; Krane-Gartiser, K., Steinan, M.K., Langsrud, K., Vestvik, V., Sand, T., Fasmer, O.B., Morken, G., Mood and motor activity in euthymic bipolar disorder with sleep disturbance (2016) J. Affect. Disord., 202, pp. 23-31; Labrecque, N., Cermakian, N., Circadian clocks in the immune system (2015) J. Biol. Rhythms, 30 (4), pp. 277-290; Lacourt, T.E., Vichaya, E.G., Chiu, G.S., Dantzer, R., Heijnen, C.J., The high costs of low-grade inflammation: persistent fatigue as a consequence of reduced cellular-energy availability and non-adaptive energy expenditure (2018) Front. Behav. Neurosci., 12, p. 78; Lam, R.W., Teng, M.Y., Jung, Y.E., Evans, V.C., Gottlieb, J.F., Chakrabarty, T., Sit, D.K., Light therapy for patients with bipolar depression: systematic review and meta-analysis of randomized controlled trials (2020) Can. J. Psychiatry, 65 (5), pp. 290-300; Lamers, F., Rhebergen, D., Merikangas, K.R.,

de Jonge, P., Beekman, A.T., Penninx, B.W., Stability and transitions of depressive subtypes over a 2-year follow-up (2012) Psychol. Med., 42 (10), pp. 2083-2093; Lamers, F., Milaneschi, Y., de Jonge, P., Giltay, E.J., Penninx, B., Metabolic and inflammatory markers: associations with individual depressive symptoms (2017) Psychol. Med., pp. 1-11; Lamers, F., Swendsen, J., Cui, L., Husky, M., Johns, J., Zipunnikov, V., Merikangas, K.R., Mood reactivity and affective dynamics in mood and anxiety disorders (2018) J. Abnorm. Psychol., 127 (7), pp. 659-669; Lamers, F., Milaneschi, Y., Penninx, B.W.J.H., Depression Subtypes and Inflammation: Atypical Rather Than Melancholic Depression Is Linked With Immunometabolic Dysregulations (2018), 455-471; Lasserre, A.M., Glaus, J., Vandeleur, C.L., Marques-Vidal, P., Vaucher, J., Bastardot, F., Preisig, M., Depression with atypical features and increase in obesity, body mass index, waist circumference, and fat mass: a prospective, population-based study (2014) JAMA Psychiatry, 71 (8), pp. 880-888; Lasserre, A.M., Strippoli, M.F., Glaus, J., Gholam-Rezaee, M., Vandeleur, C.L., Castelao, E., Preisig, M., Prospective associations of depression subtypes with cardio-metabolic risk factors in the general population (2017) Mol. Psychiatry, 22 (7), pp. 1026-1034; Laudon, M., Frydman-Marom, A., Therapeutic effects of melatonin receptor agonists on sleep and comorbid disorders (2014) Int. J. Mol. Sci., 15 (9), pp. 15924-15950; Lee, H.J., Woo, H.G., Greenwood, T.A., Kripke, D.F., Kelsoe, J.R., A genome-wide association study of seasonal pattern mania identifies NF1A as a possible susceptibility gene for bipolar disorder (2013) J. Affect. Disord., 145 (2), pp. 200-207; LeGates, T.A., Fernandez, D.C., Hattar, S., Light as a central modulator of circadian rhythms, sleep and affect (2014) Nat. Rev. Neurosci., 15 (7), pp. 443-454; Leproult, R., Van Onderbergen, A., L'Hermite-Baleriaux, M., Van Cauter, E., Copinschi, G., Phase-shifts of 24-h rhythms of hormonal release and body temperature following early evening administration of the melatonin agonist agomelatine in healthy older men (2005) Clin. Endocrinol. (Oxf), 63 (3), pp. 298-304; Lewy, A., Circadian misalignment in mood disturbances (2009) Curr. Psychiatry Rep., 11, pp. 459-465; Lewy, A.J., Nurnberger, J.I., Jr., Wehr, T.A., Pack, D., Becker, L.E., Powell, R.-L., Newsome, D.A., Supersensitivity to light: possible trait marker for manic-depressive illness (1985) Am. J. Psychiatry, 142, pp. 725-727; Lewy, A.J., Lefler, B.J., Emens, J.S., Bauer, V.K., The circadian basis of winter depression (2006) Proc. Natl. Acad. Sci., 103 (19), pp. 7414-7419; Li, J.Z., Bunney, B.G., Meng, F., Hagenauer, M.H., Walsh, D.M., Vawter, M.P., Bunney, W.E., Circadian patterns of gene expression in the human brain and disruption in major depressive disorder (2013) Proc. Natl. Acad. Sci., 110 (24), pp. 9950-9955; Lind, M.J., Hawn, S.E., Sheerin, C.M., Aggen, S.H., Kirkpatrick, R.M., Kendler, K.S., Amstadter, A.B., An examination of the etiologic overlap between the genetic and environmental influences on insomnia and common psychopathology (2017) Depress. Anxiety; Linkowski, P., Mendlewicz, J., Leclercq, R., Brasseur, M., Hubain, P., Goldstein, J., Van Cauter, E., The 24-hour profile of adrenocorticotropin and cortisol in major depressive illness (1985) J. Clin. Endocrinol. Metab., 61 (3), pp. 429-438; Linkowski, P., Van Onderbergen, A., Kerkhofs, M., Bosson, D., Mendlewicz, J., Van Cauter, E., Twin study of the 24-h cortisol profile: evidence for genetic control of the human circadian clock (1993) American Journal of Physiology - Endocrinology and Metabolism, 27, pp. E173-E181; Linkowski, P., Kerkhofs, M., Van Onderbergen, A., Hubain, P., Copinschi, G., L'Hermite-Baleriaux, M., Van Cauter, E., The 24-hour profiles of cortisol, prolactin, and growth hormone secretion in mania (1994) Arch. Gen. Psychiatry, 51, pp. 616-624; Liu, X., Buysse, D.J., Gentzler, A.L., Kiss, E., Mayer, L., Kapornai, K., Kovacs, M., Insomnia and hypersomnia associated with depressive phenomenology and comorbidity in childhood

depression (2007) Sleep, 30 (1), pp. 83-90; Lojko, D., Rybakowski, J.K., Atypical depression: current perspectives (2017) Neuropsychiatr. Dis. Treat., 13, pp. 2447-2456; Łojko, D., Buzuk, G., Owecki, M., Ruchała, M., Rybakowski, J.K., Atypical features in depression: association with obesity and bipolar disorder (2015) J. Affect. Disord., 185, pp. 76-80; Lu, J., Zhang, Y.H., Chou, T.C., Gaus, S.E., Elmquist, J.K., Shiromani, P., Saper, C.B., Contrasting effects of ibotenate lesions of the paraventricular nucleus and subparaventricular zone on sleep-wake cycle and temperature regulation (2001) J. Neurosci., 21 (13), pp. 4864-4874; Luik, A.I., Zuurbier, L.A., Direk, N., Hofman, A., Van Someren, E.J., Tiemeier, H., 24-hour activity rhythm and sleep disturbances in depression and anxiety: a population-based study of middle-aged and older persons (2015) Depress. Anxiety, 32 (9), pp. 684-692; Luppino, F.S., Wit, L.M., Bouvy, P.F., Stijnen, T., Cuijpers, P., Penninx, B.W.J.H., Zitman, F.G., Overweight, obesity, and depression: a systematic review and meta-analysis of longitudinal studies (2010) Arch. Gen. Psychiatry, 67 (3), pp. 220-229; Lyall, L.M., Wyse, C.A., Graham, N., Ferguson, A., Lyall, D.M., Cullen, B., Smith, D.J., Association of disrupted circadian rhythmicity with mood disorders, subjective wellbeing, and cognitive function: a cross-sectional study of 91 105 participants from the UK Biobank (2018) Lancet Psychiatry, 5 (6), pp. 507-514; Maj, M., Pirozzi, R., Magliano, L., Fiorillo, A., Bartoli, L., Phenomenology and prognostic significance of delusions in major depressive disorder: a 10-year prospective follow-up study (2007) J. Clin. Psychiatry, 68 (9), pp. 1411-1417; Malhi, G.S., Bassett, D., Boyce, P., Bryant, R., Fitzgerald, P.B., Fritz, K., Singh, A.B., Royal Australian and New Zealand College of Psychiatrists clinical practice guidelines for mood disorders (2015) Aust. N. Z. J. Psychiatry, 49 (12), pp. 1087-1206; Mansur, R.B., Brietzke, E., McIntyre, R.S., Is there a “metabolic-mood syndrome”? A review of the relationship between obesity and mood disorders (2015) Neurosci. Biobehav. Rev., 52, pp. 89-104; Mathew, G.M., Hale, L., Chang, A.-M., Sex moderates relationships among school night sleep duration, social jetlag, and depressive symptoms in adolescents (2019) J. Biol. Rhythms, 34 (2), pp. 205-217; Matza, L.S., Revicki, D.A., Davidson, J.R., Stewart, J.W., Depression with atypical features in the national comorbidity survey: classification, description, and consequences (2003) Arch. Gen. Psychiatry, 60 (8), pp. 817-826; Maury, E., Hong, H.K., Bass, J., Circadian disruption in the pathogenesis of metabolic syndrome (2014) Diabetes Metab., 40 (5), pp. 338-346; McCarthy, M.J., Nievergelt, C.M., Kelsoe, J.R., Welsh, D.K., A survey of genomic studies supports association of circadian clock genes with bipolar disorder spectrum illnesses and lithium response (2012) PLoS One, 7 (2). , e32091; McClung, C.A., Circadian rhythms and mood regulation: insights from pre-clinical models (2011) Eur. Neuropsychopharmacol., 21, pp. S683-693; McClung, C.A., How might circadian rhythms control mood? Let me count the ways (2013) Biol. Psychiatry, 74 (4), pp. 242-249; McGirr, A., Berlim, M.T., Bond, D.J., Fleck, M.P., Yatham, L.N., Lam, R.W., A systematic review and meta-analysis of randomized, double-blind, placebo-controlled trials of ketamine in the rapid treatment of major depressive episodes (2015) Psychol. Med., 45, pp. 693-704; McGirr, A., Karmani, S., Arsappa, R., Berlim, M.T., Thirthalli, J., Muralidharan, K., Yatham, L.N., Clinical efficacy and safety of repetitive transcranial magnetic stimulation in acute bipolar depression (2016) World Psychiatry, 15 (1), pp. 85-86; McGlashan, E.M., Nandam, L.S., Vidafar, P., Mansfield, D.R., Rajaratnam, S.M.W., Cain, S.W., The SSRI citalopram increases the sensitivity of the human circadian system to light in an acute dose (2018) Psychopharmacology, 235 (11), pp. 3201-3209; McGlashan, E.M., Coleman, M.Y., Vidafar, P., Phillips, A.J.K., Cain, S.W., Decreased sensitivity of the circadian system to light in current,

but not remitted depression (2019) J. Affect. Disord., 256, pp. 386-392; McGrath, P.J., Stewart, J.W., Harrison, W., Wager, S., Quitkin, F.M., Phenelzine treatment of melancholia (1986) J. Clin. Psychiatry, 47 (8), pp. 420-422; McGuffin, P., Rijsdijk, F., Andrew, M., Sham, P., Katz, R., Cardno, A., The heritability of bipolar affective disorder and the genetic relationship to unipolar depression (2003) Arch. Gen. Psychiatry, 60, pp. 497-502; McIntyre, R.S., Lee, Y., Zhou, A.J., Rosenblat, J.D., Peters, E.M., Lam, R.W., Jerrell, J.M., The efficacy of psychostimulants in major depressive episodes: a systematic review and meta-analysis (2017) J. Clin. Psychopharmacol., 37 (4), pp. 412-418; Meliska, C.J., Martinez, L.F., Lopez, A.M., Sorenson, D.L., Nowakowski, S., Parry, B.L., Relationship of morningness-eveningness questionnaire score to melatonin and sleep timing, body mass index and atypical depressive symptoms in peri- and post-menopausal women (2011) Psychiatry Res., 188 (1), pp. 88-95; Melo, M.C.A., Abreu, R.L.C., Linhares Neto, V.B., de Bruin, P.F.C., de Bruin, V.M.S., Chronotype and circadian rhythm in bipolar disorder: a systematic review (2017) Sleep Med. Rev., 34, pp. 46-58; Melo, M.C., Garcia, R.F., Linhares Neto, V.B., Sa, M.B., de Mesquita, L.M., de Araujo, C.F., de Bruin, V.M., Sleep and circadian alterations in people at risk for bipolar disorder: a systematic review (2016) J. Psychiatr. Res., 83, pp. 211-219; Menculini, G., Verdolini, N., Murru, A., Pacchiarotti, I., Volpe, U., Cervino, A., Tortorella, A., Depressive mood and circadian rhythms disturbances as outcomes of seasonal affective disorder treatment: a systematic review (2018) J. Affect. Disord., 241, pp. 608-626; Merikanto, I., Lahti, T., Kronholm, E., Peltonen, M., Laatikainen, T., Vartiainen, E., Partonen, T., Evening types are prone to depression (2013) Chronobiol. Int., 30 (5), pp. 719-725; Merikanto, I., Lahti, T., Puolijoki, H., Vanhala, M., Peltonen, M., Laatikainen, T., Vartiainen, E., Partonen, T., Associations of chronotype and sleep with cardiovascular diseases and type 2 diabetes (2013) Chronobiol. Int., 30 (4), pp. 470-477; Merikangas, K.R., Swendsen, J., Hickie, I.B., Cui, L., Shou, H., Merikangas, A.K., Zipunnikov, V., Real-time mobile monitoring of the dynamic associations among motor activity, energy, mood, and sleep in adults with bipolar disorder (2018) JAMA Psychiatry; Miklowitz, D.J., Efthimiou, O., Furukawa, T.A., Scott, J., McLaren, R., Geddes, J.R., Cipriani, A., Adjunctive psychotherapy for bipolar disorder: a systematic review and component network meta-analysis (2020) JAMA Psychiatry; Milaneschi, Y., Lamers, F., Bot, M., Drent, M.L., Penninx, B.W., Leptin dysregulation is specifically associated with major depression with atypical features: evidence for a mechanism connecting obesity and depression (2017) Biol. Psychiatry, 81 (9), pp. 807-814; Milosavljevic, N., How does light regulate mood and behavioral state? (2019) Clocks Sleep, 1 (3), pp. 319-331; Mitchell, P.B., Goodwin, G.M., Johnson, G.F., Hirschfeld, R.M., Diagnostic guidelines for bipolar depression: a probabilistic approach (2008) Bipolar Disord., 10 (1), pp. 144-152; Mohawk, J.A., Green, C.B., Takahashi, J.S., Central and peripheral circadian clocks in mammals (2012) Annu. Rev. Neurosci., 35, pp. 445-462; Mondin, T.C., Cardoso, T.A., Souza, L.D.M., Jansen, K., da Silva Magalhães, P.V., Kapczinski, F., da Silva, R.A., Mood disorders and biological rhythms in young adults: a large population-based study (2017) J. Psychiatr. Res., 84, pp. 98-104; Montagner, A., Korecka, A., Polizzi, A., Lippi, Y., Blum, Y., Canlet, C., Wahli, W., Hepatic circadian clock oscillators and nuclear receptors integrate microbiome-derived signals (2016) Sci. Rep., 6, p. 20127; Monteleone, P., Martiadis, V., Maj, M., Circadian rhythms and treatment implications in depression (2011) Prog. Neuropsychopharmacol. Biol. Psychiatry, 35 (7), pp. 1569-1574; Moon, J.H., Cho, C.H., Son, G., H, Geum, D., Chung, S., Kim, H., Lee, H.J., Advanced circadian phase in mania and delayed circadian phase in

mixed mania and depression returned to normal after treatment of bipolar disorder (2016) EBioMedicine; Moriarity, D.P., Giollabhui, N.M., Ellman, L.M., Klugman, J., Coe, C.L., Abramson, L.Y., Alloy, L.B., Inflammatory proteins predict change in depressive symptoms in male and female adolescents (2019) Clin. Psychol. Sci., 7 (4), pp. 754-767; Morita, Y., Sasai-Sakuma, T., Asaoka, S., Inoue, Y., The impact of a delayed sleep-wake schedule on depression is greater in women–a web-based cross-sectional study in Japanese young adults (2015) Chronobiol. Int., 32 (7), pp. 952-958; Morris, G., Stubbs, B., Kohler, C.A., Walder, K., Slyepchenko, A., Berk, M., Carvalho, A.F., The putative role of oxidative stress and inflammation in the pathophysiology of sleep dysfunction across neuropsychiatric disorders: focus on chronic fatigue syndrome, bipolar disorder and multiple sclerosis (2018) Sleep Med. Rev., 41, pp. 255-265; Murray, J.M., Sletten, T.L., Magee, M., Gordon, C., Lovato, N., Bartlett, D.J., for the Delayed Sleep on Melatonin Study, G, Prevalence of circadian misalignment and its association with depressive symptoms in delayed sleep phase disorder (2017) Sleep, 40 (1); Murru, A., Guiso, G., Barbuti, M., Anmella, G., Verdolini, N., Samalin, L., Pacchiarotti, I., The implications of hypersomnia in the context of major depression: results from a large, international, observational study (2019) Eur. Neuropsychopharmacol., 29 (4), pp. 471-481; Nagayama, H., Sasaki, M., Ichii, S., Hanada, K., Okawa, M., Ohta, T., Atypical depressive symptoms possibly predict responsiveness to phototherapy in seasonal affective disorder (1991) J. Affect. Disord., 23 (4), pp. 185-189; Nair, N.V.P., Hariharasubraman, N., Pilapil, C., Circadian rhythm of plasma melatonin in endogenous depression (1984) Prog. Neuropsychopharmacol. Biol. Psychiatry, 8, pp. 715-718; Naismith, S.L., Hermens, D.F., Ip, T.K., Bolitho, S., Scott, E., Rogers, N.L., Hickie, I.B., Circadian profiles in young people during the early stages of affective disorder (2012) Transl. Psychiatry, 2 (5), p. e123; Naismith, S.L., Norrie, L.M., Mowszowski, L., Hickie, I.B., The neurobiology of depression in later-life: clinical, neuropsychological, neuroimaging and pathophysiological features (2012) Prog. Neurobiol., 98 (1), pp. 99-143; Nardi, B., Francesconi, G., Catena-Dell'Osso, M., Bellantuono, C., Adolescent depression: clinical features and therapeutic strategies (2013) Eur. Rev. Med. Pharmacol. Sci., 17, pp. 1546-1551; Naus, T., Burger, A., Malkoc, A., Molendijk, M., Haffmans, J., Is there a difference in clinical efficacy of bright light therapy for different types of depression? A pilot study (2013) J. Affect. Disord., 151 (3), pp. 1135-1137; Ng, T.H., Chung, K.F., Ho, F.Y., Yeung, W.F., Yung, K.P., Lam, T.H., Sleep-wake disturbance in interepisode bipolar disorder and high-risk individuals: a systematic review and meta-analysis (2015) Sleep Med. Rev., 20, pp. 46-58; Ng, T.H., Chung, K.-F., Ng, T.-K., Lee, C.-T., Chan, M.-S., Correlates and prognostic relevance of sleep irregularity in inter-episode bipolar disorder (2016) Compr. Psychiatry, 69, pp. 155-162; Norris, E.R., Karen, B., Correll, J.R., Zemanek, K.J., Lerman, J., Primelo, R.A., Kaufmann, M.W., A double-blind, randomized, placebo-controlled trial of adjunctive ramelteon for the treatment of insomnia and mood stability in patients with euthymic bipolar disorder (2013) J. Affect. Disord., 144 (1-2), pp. 141-147; Novakova, M., Prasko, J., Latalova, K., Sladek, M., Sumova, A., The circadian system of patients with bipolar disorder differs in episodes of mania and depression (2015) Bipolar Disord., 17 (3), pp. 303-314; Nunez, N.A., Singh, B., Romo-Nava, F., Joseph, B., Veldic, M., Cuellar-Barboza, A., Frye, M.A., Efficacy and tolerability of adjunctive modafinil/armodafinil in bipolar depression: a meta-analysis of randomized controlled trials (2020) Bipolar Disord., 22 (2), pp. 109-120; O'Keane, V., Frodl, T., Dinan, T.G., A review of atypical depression in relation to the course of depression and changes in HPA axis organization (2012)

Psychoneuroendocrinology, 37 (10), pp. 1589-1599; Osimo, E.F., Baxter, L.J., Lewis, G., Jones, P.B., Khandaker, G.M., Prevalence of low-grade inflammation in depression: a systematic review and metaanalysis of CRP levels (2019) Psychol. Med., 49, pp. 1958-1970; Pacchiarotti, I., Bond, D.J., Baldessarini, R.J., Nolen, W.A., Grunze, H., Licht, R.W., Vieta, E., The International Society for Bipolar Disorders (ISBD) task force report on antidepressant use in bipolar disorders (2013) Am. J. Psychiatry, 170 (11), pp. 1249-1262; Palagini, L., Bastien, C.H., Marazziti, D., Ellis, J.G., Riemann, D., The key role of insomnia and sleep loss in the dysregulation of multiple systems involved in mood disorders: a proposed model (2019) J. Sleep Res., , e12841; Park, L.T., Luckenbaugh, D.A., Pennybaker, S.J., Hopkins, M.A., Henter, I.D., Lener, M.S., Zarate, C.A., Jr., The effects of ketamine on typical and atypical depressive symptoms (2020) Acta Psychiatr. Scand., 142 (5), pp. 394-401; Parker, G., Classifying depression: should paradigms lost be regained? (2000) Am. J. Psychiatry, 157 (8), pp. 1195-1203; Parker, G., McCraw, S., Blanch, B., Hadzi-Pavlovic, D., Synnott, H., Rees, A.M., Discriminating melancholic and non-melancholic depression by prototypic clinical features (2013) J. Affect. Disord., 144 (3), pp. 199-207; Parker, G., Brotchie, H., McClure, G., Fletcher, K., Psychostimulants for managing unipolar and bipolar treatment-resistant melancholic depression: a medium-term evaluation of cost benefits (2013) J. Affect. Disord., 151 (1), pp. 360-364; Parker, G., Hadzi-Pavlovic, D., Melancholia: A Disorder of Movement and Mood (1996), Cambridge University Press New York; Parker, G., Walter, S., Seasonal variation in depressive disorders and suicidal deaths in New South Wales (1982) Br. J. Psychiatry, 140, pp. 626-632; Parker, G., Roussos, J., Mitchell, P., Wilhelm, K., Marie-Paule, A., Hadzi-Pavlovic, D., Distinguishing psychotic depression from melancholia (1997) J. Affect. Disord., 42 (2), pp. 155-167; Parker, G., Roy, K., Mitchell, P., Wilhelm, K., Malhi, G., Hadzi-Pavlovic, D., Atypical depression: a reappraisal (2002) Am. J. Psychiatry, 159, pp. 1470-1479; Parker, G., Malhi, G., Hadzi-Pavlovic, D., Parker, K., Sleeping in? The impact of age and depressive sub-type on hypersomnia (2006) J. Affect. Disord., 90 (1), pp. 73-76; Parker, G., (2019) Bipolar II Disorder: Modelling, Measuring and Managing, , 3rd ed. Cambridge University Press; Parry, B.L., Optimal management of perimenopausal depression (2010) Int. J. Womens Health, 2, pp. 143-151; Parry, B.L., Plasma melatonin circadian rhythm disturbances during pregnancy and postpartum in depressed women and women with personal or family histories of depression (2008) Am. J. Psychiatry, 165 (12), pp. 1551-1558; Pascual-Leone, A., Rubio, B., Pallardó, F., Catalá, M.D., Rapid-rate transcranial magnetic stimulation of left dorsolateral prefrontal cortex in drug-resistant depression (1996) Lancet, 348 (9022), pp. 233-237; Paykel, E.S., Rowan, P.R., Parker, R.R., Bhat, A.V., Response to phenelzine and amitriptyline in subtypes of outpatient depression (1982) Arch. Gen. Psychiatry, 39 (9), pp. 1041-1049; Penninx, B.W., Depression and cardiovascular disease: epidemiological evidence on their linking mechanisms (2017) Neurosci. Biobehav. Rev., 74, pp. 277-286; Perera, S., Eisen, R., Bhatt, M., Bhatnagar, N., de Souza, R., Thabane, L., Samaan, Z., Light therapy for non-seasonal depression: systematic review and meta-analysis (2016) British Journal of Psychiatry Open, 2 (2), pp. 116-126; Perlman, K., Benrimoh, D., Israel, S., Rollins, C., Brown, E., Tunteng, J.F., Berlim, M.T., A systematic meta-review of predictors of antidepressant treatment outcome in major depressive disorder (2019) J. Affect. Disord., 243, pp. 503-515; Perry, P.J., Pharmacotherapy for major depression with melancholic features: relative efficacy of tricyclic versus selective serotonin reuptake inhibitor antidepressants (1996) J. Affect. Disord., 39 (1), pp. 1-6; Persons, J.B., Roberts, N.A., Zalecki, C.A., Brechwald, W.A.G., Naturalistic outcome of case

formulation-driven cognitive-behavior therapy for anxious depressed outpatients (2006) Behav. Res. Ther., 44 (7), pp. 1041-1051; Perugi, G., Akiskal, H.S., Lattanzi, L., Cecconi, D., Mastrocinque, C., Patronelli, A., Bemi, E., The High Prevalence of “Soft” Bipolar (II) Features in Atypical Depression (1998) Compr. Psychiatry, 39 (2), pp. 63-71; Petrides, G., Fink, M., Husain, M.M., Knapp, R.G., Rush, A.J., Mueller, M., Kellner, C.H., ECT remission rates in psychotic versus nonpsychotic depressed patients: a report from CORE (2001) J. ECT, 17 (4), pp. 244-253; Phillips, J.L., Norris, S., Talbot, J., Birmingham, M., Hatchard, T., Ortiz, A., Blier, P., Single, repeated, and maintenance ketamine infusions for treatment-resistant depression: a randomized controlled trial (2019) Am. J. Psychiatry, 176 (5), pp. 401-409; Pinho, M., Sehmbi, M., Cudney, L.E., Kauer-Sant'anna, M., Magalhães, P.V., Reinares, M., Rosa, A.R., The association between biological rhythms, depression, and functioning in bipolar disorder: a large multi-center study (2016) Acta Psychiatr. Scand., 133 (2), pp. 102-108; Pisanu, C., Congiu, D., Melis, C., Severino, G., Angius, A., Ardau, R., Squassina, A., Involvement of core clock genes in lithium response (2017) World J. Biol. Psychiatry, pp. 1-2; Posener, J.A., DeBattista, C., Williams, G.H., Kraemer, H.C., Kalehzan, M., Schatzberg, A.F., 24-hour monitoring of cortisol and corticotropin secretion in psychotic and nonpsychotic major depression (2000) Arch. Gen. Psychiatry, 57, pp. 755-760; Posener, J.A., Charles, D., Veldhuis, J.D., Province, M.A., Williams, G.H., Schatzberg, A.F., Process irregularity of cortisol and adrenocorticotropin secretion in men with major depressive disorder (2004) Psychoneuroendocrinology, 29 (9), pp. 1129-1137; Post, R.M., Altshuler, L.L., Leverich, G.S., Frye, M.A., Nolen, W.A., Kupka, R.W., Mintz, J., Mood switch in bipolar depression: comparison of adjunctive venlafaxine, bupropion and sertraline (2006) Br. J. Psychiatry, 189, pp. 124-131; Power, R.A., Tansey, K.E., Buttenschon, H.N., Cohen-Woods, S., Bigdeli, T., Hall, L.S., Lewis, C.M., Genome-wide association for major depression through age at onset stratification: major depressive disorder working group of the psychiatric genomics consortium (2017) Biol. Psychiatry, 81 (4), pp. 325-335; Preisig, M., Strippoli, M.F., Castelao, E., Merikangas, K.R., Gholam-Rezaee, M., Marquet, P., Vandeleur, C.L., The specificity of the familial aggregation of early-onset bipolar disorder: A controlled 10-year follow-up study of offspring of parents with mood disorders (2016) J. Affect. Disord., 190, pp. 26-33; Prusoff, B.A., Weissman, M.M., Klerman, G.L., Rounsaville, B.J., Research diagnostic criteria subtypes of depression. Their role as predictors of differential response to psychotherapy and drug treatment (1980) Arch. Gen. Psychiatry, 37 (7), pp. 796-801; Radua, J., Pertusa, A., Cardoner, N., Climatic relationships with specific clinical subtypes of depression (2010) Psychiatry Res., 175 (3), pp. 217-220; Ratheesh, A., Davey, C., Hetrick, S., Alvarez-Jimenez, M., Voutier, C., Bechdolf, A., Cotton, S.M., A systematic review and meta-analysis of prospective transition from major depression to bipolar disorder (2017) Acta Psychiatr. Scand.; Reinke, H., Asher, G., Circadian clock control of liver metabolic functions (2016) Gastroenterology, 150 (3), pp. 574-580; Reiter, R.J., Calvo, J.R., Karbownik, M., Qi, W., Tan, D.X., Melatonin and its relation to the immune system and inflammation (2000) Ann. N. Y. Acad. Sci., 917, pp. 376-386; Reiter, R.J., Tan, D.X., Korkmaz, A., Ma, S., Obesity and metabolic syndrome: association with chronodisruption, sleep deprivation, and melatonin suppression (2012) Ann. Med., 44 (6), pp. 564-577; Ren, J., Li, H., Palaniyappan, L., Liu, H., Wang, J., Li, C., Rossini, P.M., Repetitive transcranial magnetic stimulation versus electroconvulsive therapy for major depression: a systematic review and meta-analysis (2014) Prog. Neuropsychopharmacol. Biol. Psychiatry, 51, pp. 181-189; Rhen, T., Cidlowski,

J.A., Antiinflammatory action of glucocorticoids- new mechanisms for old drugs (2005) N. Engl. J. Med., 353, pp. 1711-1723; Rice, F., Riglin, L., Lomax, T., Souter, E., Potter, R., Smith, D.J., Thapar, A., Adolescent and adult differences in major depression symptom profiles (2019) J. Affect. Disord., 243, pp. 175-181; Rice, F., Riglin, L., Thapar, A.K., Heron, J., Anney, R., O'Donovan, M.C., Thapar, A., Characterizing developmental trajectories and the role of neuropsychiatric genetic risk variants in early-onset depression (2019) JAMA Psychiatry, 76 (3); Ringel, B.L., Szuba, M.P., Potential mechanisms of the sleep therapies for depression (2001) Depress. Anxiety, 14, pp. 29-36; Robillard, R., Naismith, S.L., Rogers, N.L., Ip, T.K., Hermens, D.F., Scott, E.M., Hickie, I.B., Delayed sleep phase in young people with unipolar or bipolar affective disorders (2013) J. Affect. Disord., 145 (2), pp. 260-263; Robillard, R., Naismith, S.L., Rogers, N.L., Scott, E.M., Ip, T.K., Hermens, D.F., Hickie, I.B., Sleep-wake cycle and melatonin rhythms in adolescents and young adults with mood disorders: comparison of unipolar and bipolar phenotypes (2013) Eur. Psychiatry, 28 (7), pp. 412-416; Robillard, R., Naismith, S.L., Smith, K.L., Rogers, N.L., White, D., Terpening, Z., Hickie, I.B., Sleep-wake cycle in young and older persons with a lifetime history of mood disorders (2014) PLoS One, 9 (2). , e87763; Robillard, R., Hermens, D.F., Naismith, S.L., White, D., Rogers, N.L., Ip, T.K., Hickie, I.B., Ambulatory sleep-wake patterns and variability in young people with emerging mental disorders (2015) J. Psychiatry Neurosci., 40 (1), pp. 28-37; Robillard, R., Carpenter, J.S., Feilds, K.L., Hermens, D.F., White, D., Naismith, S.L., Hickie, I.B., Parallel changes in mood and melatonin rhythm following an adjunctive multimodal chronobiological intervention with agomelatine in people with depression: a proof of concept open label study (2018) Front. Psychiatry, 9, p. 624; Robillard, R., Carpenter, J.S., Rogers, N.L., Fares, S., Grierson, A.B., Hermens, D.F., Hickie, I.B., Circadian rhythms and psychiatric profiles in young adults with unipolar depressive disorders (2018) Transl. Psychiatry, 8 (1), p. 213; Rosenblat, J.D., McIntyre, R.S., Bipolar disorder and immune dysfunction: epidemiological findings, proposed pathophysiology and clinical implications (2017) Brain Sci., 7 (11); Rubin, R.T., Kevin Heist, K.E., McGeoy, S.S., Hanada, K., Lesser, I.M., Neuroendocrine aspects of primary endogenous depression XI: serum melatonin measures in patients and matched control subjects (1992) Arch. Gen. Psychiatry, 49, pp. 558-567; Rudolf, S., Greggersen, W., Kahl, K.G., Huppe, M., Schweiger, U., Elevated IL-6 levels in patients with atypical depression but not in patients with typical depression (2014) Psychiatry Res., 217 (1-2), pp. 34-38; Rupp, A.C., Ren, M., Altimus, C.M., Fernandez, D.C., Richardson, M., Turek, F., Schmidt, T.M., Distinct ipRGC subpopulations mediate light's acute and circadian effects on body temperature and sleep (2019) Elife, p. 8; Salloum, N.C., Fava, M., Freeman, M.P., Flynn, M., Hoeppner, B., Hock, R.S., Papakostas, G.I., Efficacy of intravenous ketamine treatment in anxious versus nonanxious unipolar treatment-resistant depression (2019) Depress. Anxiety, 36 (3), pp. 235-243; Salvatore, P., Indic, P., Murray, G., Baldessarini, R.J., Biological rhythms and mood disorders (2012) Dialogues Clin. Neurosci., 14, pp. 369-379; Satyanarayanan, S.K., Chien, Y.C., Chang, J.P., Huang, S.Y., Guu, T.W., Su, H., Su, K.P., Melatonergic agonist regulates circadian clock genes and peripheral inflammatory and neuroplasticity markers in patients with depression and anxiety (2019) Brain Behav. Immun.; Saxvig, I.W., Pallesen, S., Wilhelmsen-Langeland, A., Molde, H., Bjorvatn, B., Prevalence and correlates of delayed sleep phase in high school students (2012) Sleep Med., 13 (2), pp. 193-199; Scheiermann, C., Kunisaki, Y., Frenette, P.S., Circadian control of the immune system (2013) Nat. Rev. Immunol., 13 (3), pp. 190-198; Schmidt, C., Collette, F., Cajochen, C.,

Peigneux, P., A time to think: circadian rhythms in human cognition (2007) Cogn. Neuropsychol., 24 (7), pp. 755-789; Schuch, F., Vancampfort, D., Firth, J., Rosenbaum, S., Ward, P., Reichert, T., Stubbs, B., Physical activity and sedentary behavior in people with major depressive disorder: a systematic review and meta-analysis (2017) J. Affect. Disord., 210, pp. 139-150; Scott, L.J., Muglia, P., Kong, X.Q., Guan, W., Flickinger, M., Upmanyu, R., Boehnke, M., Genome-wide association and meta-analysis of bipolar disorder in individuals of European ancestry (2008) Proc. Natl. Acad. Sci., 106 (18), pp. 7501-7506. , doi: 10.173/pnas.0813386106; Scott, J., Naismith, S., Grierson, A., Carpenter, J., Hermens, D., Scott, E., Hickie, I., Sleep-wake cycle phenotypes in young people with familial and non-familial mood disorders (2016) Bipolar Disorders, 18 (8), pp. 642-649; Scott, J., Murray, G., Henry, C., Morken, G., Scott, E., Angst, J., Hickie, I.B., Activation in bipolar disorders: a systematic review (2017) JAMA Psychiatry, 74 (2), pp. 189-196; Scott, J., Etain, B., Bellivier, F., Can an integrated science approach to precision medicine research improve lithium treatment in bipolar disorders? (2018) Front. Psychiatry, 9, p. 360; Serretti, A., Benedetti, F., Mandelli, L., Lorenzi, C., Pirovano, A., Colombo, C., Smeraldi, E., Genetic dissection of psychopathological symptoms: insomnia in mood disorders and CLOCK gene polymorphism (2003) Am. J. Med. Genet. Part B Neuropsychiatr. Genet., 121B (1), pp. 35-38; Shafii, M., MacMillan, D.R., Key, M.P., Derrick, A.M., Kaufman, N., Nahinsky, I.D., Nocturnal serum melatonin profile in major depression in children and adolescents (1996) Arch. Gen. Psychiatry, 53, pp. 1009-1013; Shen, G.H.C., Alloy, L.B., Abramson, L.Y., Sylvia, L.G., Social rhythm regularity and the onset of affective episodes in bipolar spectrum individuals (2008) Bipolar Disord., 10, pp. 520-529; Shi, J., Wittke-Thompson, J.K., Badner, J.A., Hattori, E., Potash, J.B., Willour, V.L., Liu, C., Clock genes may influence bipolar disorder susceptibility and dysfunctional circadian rhythm (2008) Am. J. Med. Genet. Part B Neuropsychiatr. Genet., 147 (7), pp. 1047-1055; Shou, H., Cui, L., Hickie, I., Lameira, D., Lamers, F., Zhang, J., Merikangas, K.R., Dysregulation of objectively assessed 24-hour motor activity patterns as a potential marker for bipolar I disorder: results of a community-based family study (2017) Transl. Psychiatry, 7 (8). , e1211; Simon, S.L., Behn, C.D., Cree-Green, M., Kaar, J.L., Pyle, L., Hawkins, S.M.M., Nadeau, K.J., Too late and not enough: school year sleep duration, timing, and circadian misalignment are associated with reduced insulin sensitivity in adolescents with overweight/obesity (2019) J. Pediatr., 205, pp. 257-264. , e251; Simonneaux, V., Ribelayga, C., Generation of the melatonin endocrine message in mammals: a review of the complex regulation of melatonin synthesis by norepinephrine, peptides, and other pineal transmitters (2003) Pharmacol. Rev., 55 (2), pp. 325-395; Sivertsen, B., Harvey, A.G., Pallesen, S., Hysing, M., Mental health problems in adolescents with delayed sleep phase: results from a large population-based study in Norway (2015) J. Sleep Res., 24 (1), pp. 11-18; Sklar, P., Ripke, S., Scott, L.J., Andreassen, O.A., Cichon, S., Craddock, N., Purcell, S.M., Large-scale genome-wide association analysis of bipolar disorder identifies a new susceptibility locus near ODZ4 (2011) Nat. Genet., 43 (10), pp. 977-983; Sloan, E.P., Flint, A.J., Reinish, L., Shapiro, C.M., Circadian rhythms and psychiatric disorders in the elderly (1996) J. Geriatr. Psychiatry Neurol., 9, pp. 164-170; Slyepchenko, A., Allega, O.R., Leng, X., Minuzzi, L., Eltayebani, M.M., Skelly, M., Frey, B.N., Association of functioning and quality of life with objective and subjective measures of sleep and biological rhythms in major depressive and bipolar disorder (2019) Aust. N. Z. J. Psychiatry, , 4867419829228; Smagula, S.F., Krafty, R.T., Thayer, J.F., Buysse, D.J., Hall, M.H., Rest-activity rhythm profiles associated with manic-hypomanic

and depressive symptoms (2018) J. Psychiatr. Res., 102, pp. 238-244; Soehner, A.M., Kaplan, K.A., Harvey, A.G., Prevalence and clinical correlates of co-occurring insomnia and hypersomnia symptoms in depression (2014) J. Affect. Disord., 167, pp. 93-97; Soreca, I., Wallace, M.L., Frank, E., Hasler, B.P., Levenson, J.C., Kupfer, D.J., Sleep duration is associated with dyslipidemia in patients with bipolar disorder in clinical remission (2012) J. Affect. Disord., 141 (2-3), pp. 484-487; Soria, V., Martinez-Amoros, E., Escaramis, G., Valero, J., Perez-Egea, R., Garcia, C., Urretavizcaya, M., Differential association of circadian genes with mood disorders: CRY1 and NPAS2 are associated with unipolar major depression and CLOCK and VIP with bipolar disorder (2010) Neuropsychopharmacology, 35 (6), pp. 1279-1289; Souetre, E., Salvati, E., Wehr, T.A., Sack, D.A., Krebs, B., Darcourt, G., Twenty-four-hour profiles of body temperature and plasma TSH in bipolar patients during depression and during remission and in normal control subjects (1988) Am. J. Psychiatry, 145 (9), pp. 1133-1137; Souetre, E., Salvati, E., Belugou, J.-L., Pringuey, D., Candito, M., Krebs, B., Darcourt, G., Circadian rhythms in depression and recovery: evidence for blunted amplitude as the main chronobiological abnormality (1989) Psychiatry Res., 28, pp. 263-278; Sperry, S.H., Kwapil, T.R., What can daily life assessment tell us about the bipolar spectrum? (2017) Psychiatry Res., 252, pp. 51-56; Stein, D.J., Picarel-Blanchot, F., Kennedy, S.H., Efficacy of the novel antidepressant agomelatine for anxiety symptoms in major depression (2013) Hum. Psychopharmacol. Clin. Exp., 28 (2), pp. 151-159; Steinan, M.K., Morken, G., Lagerberg, T.V., Melle, I., Andreassen, O.A., Vaaler, A.E., Scott, J., Delayed sleep phase: an important circadian subtype of sleep disturbance in bipolar disorders (2016) J. Affect. Disord., 191, pp. 156-163; Stenvers, D.J., Scheer, F., Schrauwen, P., la Fleur, S.E., Kalsbeek, A., Circadian clocks and insulin resistance (2019) Nat. Rev. Endocrinol., 15 (2), pp. 75-89; Stewart, J.W., Treating depression with atypical features (2007) J. Clin. Psychiatry, 68, pp. 25-29; Stewart, J.W., Thase, M.E., Treating DSM-IV depression with atypical features (2007) J. Clin. Psychiatry, 68 (4). , e10; Stewart, J.W., Garfinkel, R., Nunes, E.V., Donovan, S., Klein, D.F., Atypical features and treatment response in the national institute of mental health treatment of depression collaborative research program (1998) J. Clin. Psychopharmacol., 18 (6), pp. 429-434; Stokkan, K.-A., Yamazaki, S., Tei, H., Sakaki, Y., Menaker, M., Entrainment of the circadian clock in the liver by feeding (2001) Science, 291 (5503). , 490; Strawbridge, R., Young, A.H., Cleare, A.J., Biomarkers for depression: recent insights, current challenges and future prospects (2017) Neuropsychiatr. Dis. Treat., 13, pp. 1245-1262; Sugawara, H., Sakamoto, K., Harada, T., Ishigooka, J., Predictors of efficacy in lithium augmentation for treatment-resistant depression (2010) J. Affect. Disord., 125 (1-3), pp. 165-168; Sullivan, P.F., Neale, M.C., Kendler, K.S., Genetic epidemiology of major depression: review and meta-analysis (2000) Am. J. Psychiatry, 157, pp. 1552-1562; Sundberg, I., Ramklint, M., Stridsberg, M., Papadopoulos, F.C., Ekselius, L., Cunningham, J.L., Salivary melatonin in relation to depressive symptom severity in young adults (2016) PLoS One, 11 (4). , e0152814; Swanson, L.M., Burgess, H.J., Huntley, E.D., Bertram, H., Mooney, A., Zollars, J., Todd Arnedt, J., Relationships between circadian measures, depression, and response to antidepressant treatment: A preliminary investigation (2017) Psychiatry Res., 252, pp. 262-269; Takaesu, Y., Circadian rhythm in bipolar disorder: a review of the literature (2018) Psychiatry Clin. Neurosci.; Takaesu, Y., Inoue, Y., Murakoshi, A., Komada, Y., Otsuka, A., Futenma, K., Inoue, T., Prevalence of circadian rhythm sleep-wake disorders and associated factors in euthymic patients with bipolar disorder (2016) PLoS One, 11 (7). , e0159578; Takaesu, Y., Inoue, Y., Ono, K., Murakoshi, A., Futenma, K., Komada, Y., Inoue, T.,

Circadian rhythm sleep-wake disorders as predictors for bipolar disorder in patients with remitted mood disorders (2017) J. Affect. Disord., 220, pp. 57-61; Takei, N., O'Callaghan, E., Sham, P., Glover, G., Tamura, A., Murray, R., Seasonality of admissions in the psychoses: effect of diagnosis, sex, and age at onset (1992) Br. J. Psychiatry, 161, pp. 506-511; Taylor, W.D., Aizenstein, H.J., Alexopoulos, G.S., The vascular depression hypothesis: mechanisms linking vascular disease with depression (2013) Mol. Psychiatry, 18 (9), pp. 963-974; Taylor, D., Sparshatt, A., Varma, S., Olofinjana, O., Antidepressant efficacy of agomelatine: Meta-analysis of published and unpublished studies (2014) Br. Med. J., 348, p. g1888; Teichman, E.M., O'Riordan, K.J., Gahan, C.G.M., Dinan, T.G., Cryan, J.F., When rhythms meet the blues: circadian interactions with the microbiota-gut-brain axis (2020) Cell Metab., 31 (3), pp. 448-471; Thompson, C., Franey, C., Arendt, J., Checkley, S.A., A comparison of melatonin secretion in depressed patients and normal subjects (1988) Br. J. Psychiatry, 152 (2), pp. 260-265; Thompson, R.J., Mata, J., Jaeggi, S.M., Buschkuehl, M., Jonides, J., Gotlib, I.H., The Everyday Emotional Experience of Adults With Major Depressive Disorder: Examining Emotional Instability, Inertia, and Reactivity [Press Release] (2012); Tonon, A.C., Fuchs, D.F.P., Barbosa Gomes, W., Levandovski, R., Pio de Almeida Fleck, M., Hidalgo, M.P.L., da Silva Alencastro, L., Nocturnal motor activity and light exposure: objective actigraphy-based marks of melancholic and non-melancholic depressive disorder (2017) Brief report. Psychiatry Res, 258, pp. 587-590; Toomey, R., Panizzon, M.S., Kremen, W.S., Franz, C.E., Lyons, M.J., A twin-study of genetic contributions to morningness-eveningness and depression (2015) Chronobiol. Int., 32 (3), pp. 303-309; Tseng, P.T., Chen, Y.W., Tu, K.Y., Chung, W., Wang, H.Y., Wu, C.K., Lin, P.Y., Light therapy in the treatment of patients with bipolar depression: a meta-analytic study (2016) Eur. Neuropsychopharmacol., 26 (6), pp. 1037-1047; Tsujimoto, T., Yamada, N., Shimoda, K., Hanada, K., Takahashi, S., Circadian rhythms in depression- Part II: circadian rhythms in inpatients with various mental disorders (1990) J. Affect. Disord., 18, pp. 199-210; Tuunainen, A., Kripke, D.F., Endo, T., Light therapy for non-seasonal depression (2004) Cochrane Database Syst. Rev., 2004 (2). , CD004050-CD004050; Uher, R., Dernovsek, M.Z., Mors, O., Hauser, J., Souery, D., Zobel, A., Farmer, A., Melancholic, atypical and anxious depression subtypes and outcome of treatment with escitalopram and nortriptyline (2011) J. Affect. Disord., 132 (1-2), pp. 112-120; Ulbricht, C.M., Chrysanthopoulou, S.A., Levin, L., Lapane, K.L., The use of latent class analysis for identifying subtypes of depression: a systematic review (2018) Psychiatry Res., 266, pp. 228-246; Utge, S.J., Soronen, P., Loukola, A., Kronholm, E., Ollila, H.M., Pirkola, S., Paunio, T., Systematic analysis of circadian genes in a population-based sample reveals association of TIMELESS with depression and sleep disturbance (2010) PLoS One, 5 (2). , e9259; Vadnie, C.A., McClung, C.A., Circadian rhythm disturbances in mood disorders: insights into the role of the suprachiasmatic nucleus (2017) Neural Plast., 2017; Valerio, M.P., Martino, D.J., Differential response to lithium between melancholic and non-melancholic unipolar depression (2018) Psychiatry Res., 269, pp. 183-184; van den Ameele, S., van Diermen, L., Staels, W., Coppens, V., Dumont, G., Sabbe, B., Morrens, M., The effect of mood-stabilizing drugs on cytokine levels in bipolar disorder: a systematic review (2016) J. Affect. Disord., 203, pp. 364-373; van Londen, L., Goekoop, J.G., Kerkhof, G.A., Zwinderman, K.H., Wiegant, V.M., De Wied, D., Weak 24-h periodicity of body temperature and increased plasma vasopressin in melancholic depression (2001) Eur. Neuropsychopharmacol., 11 (1), pp. 7-14; van Loo, H.M., de Jonge, P., Romeijn, J.W., Kessler, R.C., Schoevers, R.A., Data-driven subtypes of major depressive disorder: a

systematic review (2012) BMC Med., 10, p. 156; van Mill, J.G., Hoogendijk, W.J., Vogelzangs, N., van Dyck, R., Penninx, B.W., Insomnia and sleep duration in a large cohort of patients with major depressive disorder and anxiety disorders (2010) J. Clin. Psychiatry, 71 (3), pp. 239-246; Varma, V.R., Dey, D., Leroux, A., Di, J., Urbanek, J., Xiao, L., Zipunnikov, V., Re-evaluating the effect of age on physical activity over the lifespan (2017) Prev. Med., 101, pp. 102-108; Veltman, E.M., de Boer, A., Dols, A., van Exel, E., Stek, M.L., Sienaert, P., Rhebergen, D., Melancholia as predictor of electroconvulsive therapy outcome in later life (2019) J. ECT, 35 (4); Verduijn, J., Milaneschi, Y., Peyrot, W.J., Hottenga, J.J., Abdellaoui, A., de Geus, E.J.C., Penninx, B., Using clinical characteristics to identify which patients with major depressive disorder have a higher genetic load for three psychiatric disorders (2017) Biol. Psychiatry, 81 (4), pp. 316-324; von Zerssen, D., Barthelmes, H., Dirlich, G., Doerr, P., Emrich, H.M., von Lindern, L., Pirke, K.M., Circadian rhythms in endogenous depression (1985) Psychiatry Res., 16, pp. 51-63; Walker, W.H., 2nd, Walton, J.C., DeVries, A.C., Nelson, R.J., Circadian rhythm disruption and mental health (2020) Transl. Psychiatry, 10 (1), p. 28; Wang, M., Wei, J., Yang, X., Ni, P., Wang, Y., Zhao, L., Ma, X., The level of IL-6 was associated with sleep disturbances in patients with major depressive disorder (2019) Neuropsychiatr. Dis. Treat., 15, pp. 1695-1700; Wang, C., Zhou, Y., Zheng, W., Liu, W., Zhan, Y., Li, H., Ning, Y., Association between depression subtypes and response to repeated-dose intravenous ketamine (2019) Acta Psychiatr. Scand., 140 (5), pp. 446-457; Wehr, T.A., Goodwin, F.K., Wirz-Justice, A., Breitmaier, J., Craig, C., 48-hour sleep-wake cycles in manic-depressive illness (1982) Arch. Gen. Psychiatry, 39, pp. 559-565; Weiss, C., Woods, K., Filipowicz, A., Ingram, K.K., Sleep quality, sleep structure, and PER3 genotype mediate chronotype effects on depressive symptoms in young adults (2020) Front. Psychol., 11. , 2028-2028; White, K.H., Rumble, M.E., Benca, R.M., Sex differences in the relationship between depressive symptoms and actigraphic assessments of sleep and rest-activity rhythms in a population-based sample (2017) Psychosom. Med., 79 (4), pp. 479-484; Wijkstra, J., Lijmer, J., Balk, F.J., Geddes, J.R., Nolen, W.A., Pharmacological treatment for unipolar psychotic depression: systematic review and meta-analysis (2006) Br. J. Psychiatry, 188 (5), pp. 410-415; Wirz-Justice, A., Benedetti, F., Perspectives in affective disorders: clocks and sleep (2019) Eur. J. Neurosci.; Wirz-Justice, A., Ajdacic, V., Rössler, W., Steinhausen, H.C., Angst, J., Prevalence of seasonal depression in a prospective cohort study (2019) Eur. Arch. Psychiatry Clin. Neurosci., 269 (7), pp. 833-839; Wood, J., Birmaher, B., Axelson, D., Ehmann, M., Kalas, C., Monk, K., Nimgainkar, V.L., Replicable differences in preferred circadian phase between bipolar disorder patients and control individuals (2009) Psychiatry Res., 166 (2-3), pp. 201-209; Wray, N.R., Gottesman, I.I., Using summary data from the danish national registers to estimate heritabilities for schizophrenia, bipolar disorder, and major depressive disorder (2012) Front. Genet., 3, p. 118; Wray, N.R., Ripke, S., Mattheisen, M., Trzaskowski, M., Byrne, E.M., Abdellaoui, A., Major Depressive Disorder Working Group of the Psychiatric Genomics, C, Genome-wide association analyses identify 44 risk variants and refine the genetic architecture of major depression (2018) Nat. Genet., 50 (5), pp. 668-681; Wright, K.P., Jr., Drake, A.L., Frey, D.J., Fleshner, M., Desouza, C.A., Gronfier, C., Czeisler, C.A., Influence of sleep deprivation and circadian misalignment on cortisol, inflammatory markers, and cytokine balance (2015) Brain Behav. Immun., 47, pp. 24-34; Wulff, K., Sleep and circadian rhythm disruption in psychiatric and neurodegenerative disease (2010) Nat. Rev. Neurosci., 11 (8), pp. 589-599; Wyse, C.A., Celis Morales, C.A., Graham, N.,

Fan, Y., Ward, J., Curtis, A.M., Pell, J.P., Adverse metabolic and mental health outcomes associated with shiftwork in a population-based study of 277,168 workers in UK biobank (2017) Ann. Med., pp. 1-10; Yatham, L.N., Vieta, E., Goodwin, G.M., Bourin, M., de Bodinat, C., Laredo, J., Calabrese, J., Agomelatine or placebo as adjunctive therapy to a mood stabiliser in bipolar I depression: randomised double-blind placebo-controlled trial (2016) Br. J. Psychiatry, 208 (1), pp. 78-86; Yatham, L.N., Kennedy, S.H., Parikh, S.V., Schaffer, A., Bond, D.J., Frey, B.N., Berk, M., Canadian Network for Mood and Anxiety Treatments (CANMAT) and International Society for Bipolar Disorders (ISBD) 2018 guidelines for the management of patients with bipolar disorder (2018) Bipolar Disord., 20 (2), pp. 97-170; Yoshikawa, T., Honma, S., Lithium lengthens circadian period of cultured brain slices in area specific manner (2016) Behav. Brain Res., 314, pp. 30-37; Zafra-Tanaka, J.H., Goicochea-Lugo, S., Villarreal-Zegarra, D., Taype-Rondan, A., Characteristics and quality of clinical practice guidelines for depression in adults: a scoping review (2019) BMC Psychiatry, 19 (1), p. 76; Zanardi, R., Franchini, L., Gasperini, M., Perez, J., Smeraldi, E., Double-blind controlled trial of sertraline versus paroxetine in the treatment of delusional depression (1996) Am. J. Psychiatry, 153 (12), pp. 1631-1633; Zhang, J., Paksarian, D., Lamers, F., Hickie, I.B., He, J., Merikangas, K.R., Sleep patterns and mental health correlates in US adolescents (2016) J. Pediatr.; Zhou, X., Ravindran, A.V., Qin, B., Del Giovane, C., Li, Q., Bauer, M., Xie, P., Comparative efficacy, acceptability, and tolerability of augmentation agents in treatment-resistant depression: systematic review and network meta-analysis (2015) J. Clin. Psychiatry, 76 (4). , e487-498; Zhu, B., Shi, C., Park, C.G., Zhao, X., Reutrakul, S., Effects of sleep restriction on metabolism-related parameters in healthy adults: a comprehensive review and meta-analysis of randomized controlled trials (2019) Sleep Med. Rev., 45, pp. 18-30},
correspondence_address1={Hickie, I.B.; Brain and Mind Centre, 94 Mallett St., Australia; email: ian.hickie@sydney.edu.au},
publisher={Elsevier Ltd},
issn={01497634},
coden={NBRED},
pubmed_id={33689801},
language={English},
abbrev_source_title={Neurosci. Biobehav. Rev.},
document_type={Review},
source={Scopus},
}

@ARTICLE{Wang2021,
author={Wang, Y. and Collins, W.B.},
title={Systematic evaluation of mobile fitness apps: Apps as the Tutor, Recorder, Game Companion, and Cheerleader},
journal={Telematics and Informatics},
year={2021},
volume={59},
doi={10.1016/j.tele.2020.101552},
art_number={101552},
note={cited By 0},
url={https://www.scopus.com/inward/record.uri?eid=2-s2.0-85098720352&doi=10.1016%2fj.tele.2020.101552&partnerID=40&md5=609c8b751961ce724e1c9ead8053ddaf},
affiliation={University of Southern California, Annenberg School for Communication and Journalism, Los Angeles, CA, United States; Purdue

University, Brian Lamb School of Communication, West Lafayette, IN, United States},
abstract={Mobile fitness applications are innovating the ways in which smartphone users self-manage their health. Prior research found that app functions may impact app efficacy. However, research to date has not sought to systematically investigate how different combinations of app functions impact user response to apps, especially adoption intent. This article describes two studies on mobile fitness app characteristics and user attitudes. Study One used content analysis and hierarchical cluster analysis on 98 iPhone fitness apps and identified four app clusters: “Tutor”, “Recorder”, “Game Companion”, and “Cheerleader.” Tracking was the predominant function in current market, but tracking-focused Recorder apps received lowest user ratings among all app clusters. Users favored Tutor apps that combine exercise education and tracking, and Game Companion apps that combine gamification, tracking, and social functions. Function combinations, rather than standalone functions, impact app success. Following a Reasoned Action Approach, Study Two found various effects of individual differences (age, gender, BMI, eHealth literacy, smartphone experience, function preference) on user attitude toward different fitness app types. A comparison between two studies demonstrated a mismatch between market offerings and user needs regarding app functions. Implications of results for mobile fitness app design to improve consumer health and for theories are discussed. © 2020 Elsevier Ltd},
author_keywords={Gamification; mHealth; Mobile app; Tailored design; Technology adoption; User experience},
keywords={Commerce; Hierarchical clustering; Hierarchical systems; Human computer interaction; Smartphones, Consumer healths; Content analysis; Individual Differences; Social function; Systematic evaluation; User attitudes; User need; User rating, Health},
references={Ajzen, I., The theory of planned behavior (1991) Org. Behav. Hum. Decis. Process., 50 (2), pp. 179-211; Anderberg, M.R., (2014) Probability and mathematical statistics: A series of monographs and textbooks, 19. , Academic Press New York, NY; Arnhold, M., Quade, M., Kirch, W., Mobile applications for diabetics: A systematic review and expert-based usability evaluation considering the special requirements of diabetes patients age 50 years or older (2014) J. Med. Internet Res., 16 (4); Azar, K.M.J., Lesser, L.I., Laing, B.Y., Stephens, J., Aurora, M.S., Burke, L.E., Palaniappan, L.P., Mobile applications for weight management (2013) Am. J. Preventive Med., 45 (5), pp. 583-589; Baker, R., Camosso-Stefinovic, J., Gillies, C., Shaw, E.J., Cheater, F., Flottorp, S., Robertson, N., Tailored interventions to overcome identified barriers to change: effects on professional practice and health care outcomes (2010) Cochrane Database Syst. Rev., 3; Bull, F.C., Kreuter, M.W., Scharff, D.P., Effects of tailored, personalized and general health messages on physical activity (1999) Patient Educ. Counsel., 36 (2), pp. 181-192; Cameron, D., Harvey, A., eHEALS: the ehealth literacy scale (2006) J Med Internet Res, 8 (4); Cialdini, R.B., Reno, R.R., Kallgren, C.A., A focus theory of normative conduct: Recycling the concept of norms to reduce littering in public places (1990) J. Personality Soc. Psychol., 58, pp. 1015-1026; Coll, C.V.N., Domingues, M.R., Gonçalves, H., Bertoldi, A.D., Perceived barriers to leisure-time physical activity during pregnancy: a literature review of quantitative and qualitative evidence (2017) J. Sci. Med. Sport, 20 (1), pp. 17-25; Deng, Z., Mo, X., Liu, S., Comparison of the middle-aged and older users’ adoption of mobile health services in China (2014) Int. J. Med. Inf., 83 (3), pp. 210-224; DiClemente, R.J., Salazar, L.F., Crosby, R.A., Health Behavior Theory for Public Health (2013), Jones & Bartlett Publishers; Donker,

T., Petrie, K., Proudfoot, J., Clarke, J., Birch, M.R., Christensen, H., Smartphones for smarter delivery of mental health programs: a systematic review (2013) J. Med. Internet Res., 15 (11); Duran, B.S., Odell, P.L., (2013) Cluster analysis: A survey, 100. , Springer Science & Business Media; Eagly, A.H., Chaiken, S., The Psychology of Attitudes (1993), Harcourt brace Jovanovich college publishers; Ernsting, C., Dombrowski, S.U., Oedekoven, M., Kanzler, M., Kuhlmey, A., Gellert, P., Using smartphones and health apps to change and manage health behaviors: a population-based survey (2017) J. Med. Internet Res., 19 (4); Fanning, J., Mullen, S.P., McAuley, E., Increasing physical activity with mobile devices: a meta-analysis (2012) J. Med. Internet Res., 14 (6); Farrow, M., Lutteroth, C., Rouse, P.C., Bilzon, J.L.J., Virtual-reality exergaming improves performance during high-intensity interval training (2019) Eur. J. Sport Sci., 19 (6), pp. 719-727; Fishbein, M., The role of theory in HIV prevention (2000) AIDS Care, 12 (3), pp. 273-278; Fishbein, M., Ajzen, I., (2010), Predicting and changing behavior: The reasoned action approach; Gibbons, F.X., Behavioral intentions, expectations, and willingness (2006) Health Behavior Constructs: Theory, Measurement, and Research, , National Cancer Institute Bethesda, MD; Goad, T., Towner, B., Jones, E., Bulger, S., Instructional tools for online physical education: using mobile technologies to enhance learning (2019) J. Phys. Educ., Recreation Dance, 90 (6), pp. 40-47; Gourlan, M., Bernard, P., Bortolon, C., Romain, A.J., Lareyre, O., Carayol, M., Ninot, G., Boiché, J., Efficacy of theory-based interventions to promote physical activity. A meta-analysis of randomised controlled trials (2016) Health Psychol. Rev., 10 (1), pp. 50-66; Goyal, S., Cafazzo, J.A., Mobile phone health apps for diabetes management: current evidence and future developments (2013) QJM: Int. J. Med., 106 (12), pp. 1067-1069; Hamari, J., Koivisto, J., Pakkanen, T., Do persuasive technologies persuade?-a review of empirical studies (2014) International Conference on Persuasive Technology, pp. 118-136. , Springer Cham; https://www.healthypeople.gov/2020/topics-objectives/topic/health-communication-and-health-information-technology, Healthy People 2020, U.S. Department of Health and Human Services, Office of Disease Prevention and Health Promotion, n.d.-a. Health communication and health information technology. Retrieved from <>; https://www.healthypeople.gov/2020/topics-objectives/topic/physical-activity, Healthy People 2020, U.S. Department of Health and Human Services, Office of Disease Prevention and Health Promotion, n.d.-b. Physical activities. Retrieved from <>; Holland, R.W., Verplanken, B., Van Knippenberg, A.D., On the nature of attitude-behavior relations: the strong guide, the weak follow (2002) Eur. J. Soc. Psychol., 32 (6), pp. 869-876; Hung, H.-C., Shwu-Ching Young, S., Lin, K.-C., Exploring the effects of integrating the iPad to improve students’ motivation and badminton skills: a WISER model for physical education (2018) Technol., Pedagogy Educ., 27 (3), pp. 265-278; Huotari, K., Hamari, J., A definition for gamification: anchoring gamification in the service marketing literature (2017) Electr. Markets, 27 (1), pp. 21-31; Hsiao, C.-H., Chang, J.-J., Tang, K.-Y., Exploring the influential factors in continuance usage of mobile social Apps: satisfaction, habit, and customer value perspectives (2016) Telematics Inf., 33 (2), pp. 342-355; Kim, H.-W., Chan, H.C., Gupta, S., Value-based Adoption of mobile internet: an empirical investigation (2007) Decis. Support Syst., 43 (1), pp. 111-126; Koivisto, J., Hamari, J., (2019), http://ceur-ws.org/Vol-2359/paper10.pdf, Gamification of physical activity: A systematic literature review of comparison studies. In: 3rd International GamiFIN Conference, GamiFIN 2019. CEUR-WS. <>; Kranz, M., MöLler, A., Hammerla, N., Diewald, S., PlöTz, T., Olivier, P., Roalter, L., The mobile fitness coach:

Toward individualized skill assessment using personalized mobile devices (2013) Pervasive Mobile Comput., 9 (2), pp. 203-215; Kreuter, M.W., Oswald, D.L., Bull, F.C., Clark, E.M., Are tailored health education materials always more effective than non-tailored materials? (2000) Health Educ. Res., 15 (3), pp. 305-315; Lee, H.E., Cho, J., What motivates users to continue using diet and fitness apps? Application of the uses and gratifications approach (2016) Health Commun., 32 (12), pp. 1445-1453; Leonard, T., Shuval, K., de Oliveira, A., Skinner, C.S., Eckel, C., Murdoch, J.C., Health behavior and behavioral economics: economic preferences and physical activity stages of change in a low-income African-American community (2013) Am J Health Promot, 27 (4), pp. 211-221; Leung, L., Wei, R., More than just talk on the move: uses and gratifications of the cellular phone (2000) Journalism Mass Commun. Q., 77 (2), pp. 308-320; Liao, G.Y., Chien, Y.T., Chen, Y.J., Hsiung, H.F., Chen, H.J., Hsieh, M.H., Wu, W.J., What to build for middle-agers to come? Attractive and necessary functions of exercise-promotion mobile phone apps: a cross-sectional study (2017) JMIR mHealth uHealth, 5 (5); Lim, S.L., Bentley, P.J., (2013), pp. 2672-2679. , Investigating app store ranking algorithms using a simulation of mobile app ecosystems. In: Evolutionary Computation (CEC), 2013 IEEE Congress on Evolutionary Computation; Lim, S.L., Bentley, P.J., Kanakam, N., Ishikawa, F., Honiden, S., Investigating country differences in mobile app user behavior and challenges for software engineering (2015) IIEEE Trans. Software Eng., 41 (1), pp. 40-64; Lustria, M.L.A., Cortese, J., Noar, S.M., Glueckauf, R.L., Computer-tailored health interventions delivered over the web: review and analysis of key components (2009) Patient Educ. Counsel., 74 (2), pp. 156-173; Mendiola, M.F., Kalnicki, M., Lindenauer, S., Valuable features in mobile health apps for patients and consumers: Content analysis of apps and user ratings (2015) JMIR mHealth uHealth, 3 (2); Miller, A.S., Cafazzo, J.A., Seto, E., A game plan: Gamification design principles in mHealth applications for chronic disease management (2016) Health Inf. J., 22 (2), pp. 184-193; Mohr, D.C., Schueller, S.M., Montague, E., Burns, M.N., Rashidi, P., The behavioral intervention technology model: an integrated conceptual and technological framework for eHealth and mHealth interventions (2014) J. Med. Internet Res., 16 (6); Mooi, E., Sarstedt, M., (2011) A Concise Guide to Market Research, pp. 237-284. , Springer Berlin Heidelberg Berlin, Heidelberg; Muessig, K.E., Pike, E.C., LeGrand, S., Hightow-Weidman, L.B., Mobile phone applications for the care and prevention of HIV and other sexually transmitted diseases: a review (2013) J. Med. Internet Res., 15 (1); Murtagh, F., Legendre, P., Ward's hierarchical agglomerative clustering method: which algorithms implement ward's criterion? (2014) J Classif, 31 (3), pp. 274-295; Norusis, M.J., (2010) Chapter 16: Cluster analysis. PASW Statistics 18 Statistical Procedures Companion, pp. 361-391. , Prentice Hall Upper Saddle River, NJ; Oinas-Kukkonen, H., Harjumaa, M., Persuasive systems design: key issues, process model, and system features (2009) Commun. Assoc. Inf. Syst., 24 (1), p. 28; Oppenheimer, D.M., Meyvis, T., Davidenko, N., Instructional manipulation checks: detecting satisficing to increase statistical power (2009) J. Exp. Soc. Psychol., 45 (4), pp. 867-872; Papastergiou, M., Natsis, P., Vernadakis, N., Antoniou, P., Introducing tablets and a mobile fitness application into primary school physical education (2020) Educ. Inf. Technol., pp. 1-18; Peng, W., Kanthawala, S., Yuan, S., Hussain, S.A., A qualitative study of user perceptions of mobile health apps (2016) BMC Publ. Health, 16 (1), pp. 1-11; (2019), https://www.pewresearch.org/internet/fact-sheet/mobile/#:~:text=The%20vast%20majority%20of%20Americans,smartphone%20ownership%20conducted%20in%202011, Pew Research Center Mobile Fact Sheet. Retrieved from <>; Reiner, M., Niermann, C., Jekauc, D., Woll, A., Long-term health benefits of physical activity–a

systematic review of longitudinal studies (2013) BMC Publ. Health, 13 (1), p. 813; Richtering, S.S., Hyun, K., Neubeck, L., Coorey, G., Chalmers, J., Usherwood, T., eHealth literacy: predictors in a population with moderate-to-high cardiovascular risk (2017) JMIR Hum. Factors, 4 (1); Rimal, R.N., Real, K., How behaviors are influenced by perceived norms: a test of the theory of normative social behavior (2005) Commun. Res., 32 (3), pp. 389-414; Sama, P.R., Eapen, Z.J., Weinfurt, K.P., Shah, B.R., Schulman, K.A., An evaluation of mobile health application tools (2014) JMIR mHealth uHealth, 2 (2); Sharifi, N., Mahdavi, R., Ebrahimi-Mameghani, M., Perceived barriers to weight loss programs for overweight or obese women (2013) Health Promotion Perspect., 3 (1), p. 11; Shin, Y., Jang, H., Pender, N.J., Psychometric evaluation of the exercise self-efficacy scale among Korean adults with chronic diseases (2001) Res. Nurs. Health, 24 (1), pp. 68-76; https://www.statista.com/statistics/779919/health-apps-available-google-play-worldwide/, Statista, 2020a. Number of mHealth apps available at Google Play from 1st quarter 2015 to 1st quarter 2020. Retrieved <>; https://www.statista.com/statistics/779910/health-apps-available-ios-worldwide/, Statista, 2020b. Number of mHealth apps available in the Apple App Store from 1st quarter 2015 to 1st quarter 2020. Retrieved from <>; Teo, A.C., Tan, G.W.H., Cheah, C.M., Ooi, K.B., Yew, K.T., Can the demographic and subjective norms influence the adoption of mobile banking? (2012) Int. J. Mobile Commun., 10 (6), pp. 578-597; Trost, S.G., Owen, N., Bauman, A.E., Sallis, J.F., Brown, W., Correlates of adults’ participation in physical activity: review and update (2002) Med. Sci. Sports Exercise, 34 (12), pp. 1996-2001; CDC, U.S., https://www.cdc.gov/nchs/data/nhanes/databriefs/adultweight.pdf, Centers for Disease Control and Prevention, n.d., Healthy weight, overweight, and obesity among U.S. adults (CDC Publication No. 03-0260 (7/03)). Retrieved from <>; CDC, U.S., (1996), https://www.cdc.gov/nccdphp/sgr/pdf/sgraag.pdf, Centers for Disease Control and Prevention Physical activity and health: A report of the Surgeon General. Retrieved from <>; Vickey, T., Breslin, J., Williams, A., Fitness - there's an app for that: Review of mobile fitness apps (2012) Int. J. Sport Soc., 3 (4); Wang, Y., Humor and camera view on mobile short-form video apps influence user experience and technology-adoption intent, an example of TikTok (DouYin) (2020) Comput. Hum. Behav., 110, p. 106373; Wang, J., Wang, Y., Wei, C., Yao, N., Yuan, A., Shan, Y., Yuan, C., Smartphone interventions for long-term health management of chronic diseases: an integrative review (2014) Telemedicine e-Health, 20 (6), pp. 570-583; Xi, N., Hamari, J., Does gamification satisfy needs? A study on the relationship between gamification features and intrinsic need satisfaction (2019) Int. J. Inf. Manage., 46, pp. 210-221; Yang, H., Bon appétit for apps: young american consumers' acceptance of mobile applications (2013) J. Comput. Inf. Syst., 53 (3), pp. 85-96; Yim, O., Ramdeen, K.T., Hierarchical cluster analysis: comparison of three linkage measures and application to psychological data (2015) Tutorials Quant. Methods Psychol., 11, pp. 8-21},
correspondence_address1={Wang, Y.; University of Southern California, United States; email: yunwenwa@usc.edu},
publisher={Elsevier Ltd},
issn={07365853},
coden={TEINE},
language={English},
abbrev_source_title={Telematics Inf},
document_type={Article},
source={Scopus},
}

```
@ARTICLE{Quintiliani2021,
author={Quintiliani, L.M. and Whiteley, J.A. and Murillo, J. and Lara, R. and Jean, C. and Quinn, E.K. and Kane, J. and Crouter, S.E. and Heeren, T.C. and Bowen, D.J.},
title={Community health worker-delivered weight management intervention among public housing residents: A feasibility study},
journal={Preventive Medicine Reports},
year={2021},
volume={22},
doi={10.1016/j.pmedr.2021.101360},
art_number={101360},
note={cited By 0},
url={https://www.scopus.com/inward/record.uri?eid=2-s2.0-85102971592&doi=10.1016%2fj.pmedr.2021.101360&partnerID=40&md5=71bd11aab3edaf530dd927eb91046cac},
affiliation={Boston University, Department of Medicine, Section of General Internal Medicine, Boston Medical Center, United States; University of Massachusetts Boston, College of Nursing and Health Sciences, Department of Exercise and Health Sciences, United States; Section of General Internal Medicine, Boston Medical Center, United States; Boston University, School of Public Health, Biostatics and Epidemiology Data Analytics Center, United States; Boston Housing Authority, United States; Department of Kinesiology, Recreation, and Sport Studies, The University of Tennessee, Knoxville, United States; Department of Biostatistics, Boston University, School of Public Health, United States; Department of Bioethics and Humanities, University of Washington, United States},
abstract={Community health worker-led interventions may be an optimal approach to promote behavior change among populations with low incomes due to the community health workers’ unique insights into participants’ social and environmental contexts and potential ability to deliver interventions widely. The objective was to determine the feasibility (implementation, acceptability, preliminary efficacy) of a weight management intervention for adults living in public housing developments. In 2016–2018, in Boston Massachusetts, we conducted a 3-month, two-group randomized trial comparing participants who received a tailored feedback report (control group) to participants who received the same report plus behavioral counseling. Community health workers provided up to 12 motivational interviewing-based counseling sessions in English or Spanish for diet and physical activity behaviors using a website designed to guide standardized content delivery. 102 participants enrolled; 8 (7.8%) were lost at 3-month follow up. Mean age was 46.5 (SD = 11.9) years; the majority were women (88%), Hispanic (67%), with ≤ high school degree (62%). For implementation, among intervention group participants (n = 50), 5 completed 0 sessions and 45 completed a mean of 4.6 (SD = 3.1) sessions. For acceptability, most indicated they would be very likely (79%) to participate again. For preliminary efficacy, adjusted linear regression models showed mean changes in weight (-0.94 kg, p = 0.31), moderate-to-vigorous physical activity (+11.7 min/day, p = 0.14), and fruit/vegetable intake (+2.30 servings/day, p < 0.0001) in the intervention vs. control group. Findings indicate a low-income public housing population was reached through a community health worker-led intervention with sufficient implementation and acceptability and promising beneficial changes in weight, nutrition, and physical activity outcomes. © 2021 The Authors},
author_keywords={Community health workers;  Diet;  Physical activity;  Public housing;  Weight management},
```

keywords={adult; article; controlled study; feasibility study; female; follow up; fruit vegetable; health auxiliary; high school; Hispanic; housing; human; human experiment; linear regression analysis; lowest income group; major clinical study; Massachusetts; middle aged; motivational interviewing; nutrition; physical activity; randomized controlled trial; resident},
references={Benjamin, E.J., Muntner, P., Alonso, A., Bittencourt, M.S., Callaway, C.W., Carson, A.P., Chamberlain, A.M., Virani, S.S., Heart disease and stroke statistics—2019 Update: a report from the american heart association (2019) Circulation, 139 (10); Hales, C.M., Fryar, C.D., Carroll, M.D., Freedman, D.S., Aoki, Y., Ogden, C.L., Differences in obesity prevalence by demographic characteristics and urbanization level among adults in the United States, 2013–2016 (2018) JAMA, 319 (23), pp. 2419-2429; West, D.S., Elaine Prewitt, T., Bursac, Z., Felix, H.C., Weight loss of black, white, and hispanic men and women in the diabetes prevention program (2008) Obesity, 16 (6), pp. 1413-1420; Digenis-Bury, E.C., Brooks, D.R., Chen, L., Ostrem, M., Horsburgh, C.R., Use of a population-based survey to describe the health of Boston public housing residents (2008) Am. J. Public Health, 98 (1), pp. 85-91; Chambers, E.C., Rosenbaum, E., Cardiovascular health outcomes of latinos in the affordable housing as an obesity mediating environment (AHOME) study: a study of rental assistance use (2014) J. Urban Health, 91 (3), pp. 489-498; (2002), https://www.ncbi.nlm.nih.gov/books/NBK221225/, Institute of Medicine Committee on Assuring the Health of the Public in the 21st Century. Understanding Population Health and Its Determinants. The Future of the Public's Health in the 21st Century. National Academies Press (US). Retrieved from; Naja-Riese, A., Keller, K.J.M., Bruno, P., Foerster, S.B., Puma, J., Whetstone, L., (2019), https://doi.org/10.1093/tbm/ibz115, … Sugerman, S. The SNAP-Ed Evaluation Framework: demonstrating the impact of a national framework for obesity prevention in low-income populations. Translational Behavioral Medicine, 9(5), 970–979; LeBlanc, E.S., Patnode, C.D., Webber, E.M., Redmond, N., Rushkin, M., O'Connor, E.A., Behavioral and pharmacotherapy weight loss interventions to prevent obesity-related morbidity and mortality in adults: Updated evidence report and systematic review for the US Preventive Services Task Force (2018) JAMA, 320 (11), pp. 1172-1191; Venditti, E.M., Behavioral lifestyle interventions for the primary prevention of type 2 diabetes and translation to Hispanic/Latino communities in the United States and Mexico (2017) Nutr. Rev., 75, pp. 85-93; (2020), https://www.apha.org/apha-communities/member-sections/community-health-workers, Community Health Workers; Rorie, J.-A., Smith, A., Evans, T., Horsburgh, C.R., Jr, Brooks, D.R., Goodman, R., Using resident health advocates to improve public health screening and follow-up among public housing residents, Boston, 2007–2008 (2011) Prevent. Chronic Dis., 8 (1), p. A15; Quintiliani, L.M., DeBiasse, M.A., Branco, J.M., Bhosrekar, S.G., Rorie, J.-A.-L., Bowen, D.J., Enhancing physical and social environments to reduce obesity among public housing residents: rationale, trial design, and baseline data for the Healthy Families study (2014) Contem. Clin. Trials, 39 (2), pp. 201-210; Islam, N., Shapiro, E., Wyatt, L., Riley, L., Zanowiak, J., Ursua, R., Trinh-Shevrin, C., Evaluating community health workers’ attributes, roles, and pathways of action in immigrant communities (2017) Prev. Med., 103, pp. 1-7; Olaniran, A., Smith, H., Unkels, R., Bar-Zeev, S., van den Broek, N., Who is a community health worker? – A systematic review of definitions (2017) Global Health Action, 10 (1), p. 1272223; Kim, K., Choi, J.S., Choi, E., Nieman, C.L., Joo, J.H., Lin, F.R., Gitlin, L.N., Han, H.-R., Effects of community-based health worker interventions to improve chronic disease management and care among vulnerable

populations: A systematic review (2016) Am. J. Public Health, 106 (4), pp. e3-e28; Yeary, K.H.K., Cornell, C.E., Moore, P.C., Gauss, C.H., Prewitt, T.E., Turner, J., The WORD: outcomes of a behavioral weight loss maintenance effectiveness trial in rural black adults of faith (2020) Obesity, 28 (3), pp. 510-520; Early, J., Gonzalez, C., Gordon-Dseagu, V., Robles-Calderon, L., Use of mobile health (mHealth) technologies and interventions among community health workers globally: a scoping review (2019) Health Promot. Pract., 20 (6), pp. 805-817; Brooks, D.R., Burtner, J.L., Borrelli, B., Heeren, T.C., Evans, T., Davine, J.A., (2017), https://doi.org/10.1093/ntr/ntx193, Twelve-month outcomes of a group-randomized community health advocate-led smoking cessation intervention in public housing. Nicotine & Tobacco Research; Jemmott, L.S., Jemmott, J.B., Chittamuru, D., Icard, L.D., Effects of a sexual HIV risk reduction intervention for african american mothers and their adolescent sons: a randomized controlled trial (2019) J. Adolesc. Health, 65 (5), pp. 643-650; Stone, R., Stone, J.D., Collins, T., Barletta-Sherwin, E., Martin, O., Crosby, R., Colorectal cancer screening in African American HOPE VI public housing residents (2019) Fam. Commun. Health, 42 (3), pp. 227-234; Henshaw, M.M., Borrelli, B., Gregorich, S.E., Heaton, B., Tooley, E.M., Santo, W., Cheng, N.F., Garcia, R.I., Randomized trial of motivational interviewing to prevent early childhood caries in public housing (2018) JDR Clinical and Translational Research, 3 (4), pp. 353-365; Bowen, D.J., Quintiliani, L.M., Bhosrekar, S.G., Goodman, R., Smith, E., Changing the housing environment to reduce obesity in public housing residents: a cluster randomized trial (2018) BMC Public Health, 18 (1), p. 883; Bowen, D.J., Kreuter, M., Spring, B., Cofta-Woerpel, L., Linnan, L., Weiner, D., Bakken, S., Fernandez, M., How we design feasibility studies (2009) Am. J. Prev. Med., 36 (5), pp. 452-457; Orsmond, G.I., Cohn, E.S., The distinctive features of a feasibility study: Objectives and guiding questions (2015) OTJR Occupat. Participat. Health, 35 (3), pp. 169-177; Harris, P.A., Taylor, R., Thielke, R., Payne, J., Gonzalez, N., Conde, J.G., Research electronic data capture (REDCap)-A metadata-driven methodology and workflow process for providing translational research informatics support (2009) J. Biomed. Inform., 42 (2), pp. 377-381; Quintiliani, L.M., Whiteley, J.A., Results of a nutrition and physical activity peer counseling intervention among nontraditional college students (2016) J. Cancer Educ., 31 (2), pp. 366-374; Quintiliani, L.M., Russinova, Z.L., Bloch, P.P., Truong, V., Xuan, Z., Pbert, L., Lasser, K.E., Patient navigation and financial incentives to promote smoking cessation in an underserved primary care population: a randomized controlled trial protocol (2015) Contem. Clin. Trials, 45, pp. 449-457; Sorensen, G., Emmons, K., Hunt, M.K., Barbeau, E., Goldman, R., Peterson, K., Kuntz, K., Berkman, L., Model for incorporating social context in health behavior interventions: applications for cancer prevention for working-class, multiethnic populations (2003) Prev. Med., 37 (3), pp. 188-197; Emmons, K., Stoddard, A., Gutheil, C., Suarez, E., Lobb, R., Fletcher, R., Cancer prevention for working class, multi-ethnic populations through health centers: the healthy directions study (2003) Cancer Causes Control, 14 (8), pp. 727-737; Sorensen, G., Barbeau, E., Stoddard, A.M., Hunt, M.K., Kaphingst, K., Wallace, L., Promoting behavior change among working-class, multiethnic workers: results of the healthy directions-small business study (2005) Am. J. Public Health, 95 (8), pp. 1389-1395; Wells, E.E., Feng, Y.I., Carrera, M., Smith, E., Goodman, R., Whiteley, J.A., Quintiliani, L.M., Identifying barriers and facilitators to nutrition and physical activity among public housing residents using photovoice (2019) Progr. Commun. Health Partnerships Res. Educ. Action, 13 (1), pp. 59-71; Bowen, D., Quintiliani, L., Socioecoconomic influences on affordable housing residents: Problem definition and possible solutions (2019) Different

Strategies of Housing Design, , V. Svalova IntechOpen; Rifas-Shiman, S.L., Willett, W.C., Lobb, R., Kotch, J., Dart, C., Gillman, M.W., PrimeScreen, a brief dietary screening tool: reproducibility and comparability with both a longer food frequency questionnaire and biomarkers (2001) Public Health Nutr., 4 (2), pp. 249-254; Hedrick, V.E., Savla, J., Comber, D.L., Flack, K.D., Estabrooks, P.A., Nsiah-Kumi, P.A., Ortmeier, S., Davy, B.M., Development of a brief questionnaire to assess habitual beverage intake (BEVQ-15): sugar-sweetened beverages and total beverage energy intake (2012) J. Acad. Nutr. Dietet., 112 (6), pp. 840-849; Crouter, S.E., Kuffel, E., Haas, J.D., Frongillo, E.A., Bassett, D.R., Refined two-regression model for the ActiGraph accelerometer (2010) Med. Sci. Sports Exerc., 42 (5), pp. 1029-1037; Hibbing, P.R., (2018), https://CRAN.R-project.org/package=AGread, van Hees, V. T. Two regression: Process data from wearable research devices using two regression algorithms. Retrieved from https://CRAN.R-project.org/package=TwoRegression. Retrieved from; Hibbing, P.R., (2019), https://CRAN.R-project.org/package=AGread, van Hees, V. T., LaMunion, S. R., Judge, D., Maygarden, J., & LLC, A. AGread: Read data files from ActiGraph monitors: Retrieved from Retrieved from https://CRAN.R-project.org/package=AGread; Sallis, J.F., Pinski, R.B., Grossman, R.M., Patterson, T.L., Nader, P.R., The development of self-efficacy scales for health related diet and exercise behaviors (1988) Health Educ. Res., 3 (3), pp. 283-292; Moser, A., Stuck, A.E., Silliman, R.A., Ganz, P.A., Clough-Gorr, K.M., The eight-item modified medical outcomes study social support survey: psychometric evaluation showed excellent performance (2012) J. Clin. Epidemiol., 65 (10), pp. 1107-1116; Levesque, C.S., Williams, G.C., Elliot, D., Pickering, M.A., Bodenhamer, B., Finley, P.J., Validating the theoretical structure of the treatment self-regulation questionnaire (TSRQ) across three different health behaviors (2007) Health Educ. Res., 22 (5), pp. 691-702; Gans, K.M., Risica, P.M., Keita, A.D., Dionne, L., Mello, J., Stowers, K.C., Papandonatos, G., Gorham, G., Multilevel approaches to increase fruit and vegetable intake in low-income housing communities: final results of the ‘Live Well, Viva Bien’ cluster-randomized trial (2018) Int. J. Behav. Nutr. Phys. Activity, 15 (1); Ahluwalia, J.S., Nollen, N., Kaur, H., James, A.S., Mayo, M.S., Resnicow, K., Pathway to health: cluster-randomized trial to increase fruit and vegetable consumption among smokers in public housing (2007) Health Psychol., 26 (2), pp. 214-221; Gudzune, K.A., Opara, O., Martinez, J.C., Doshi, R.S., Levine, D.M., Latkin, C.A., Clark, J.M., (2020), https://doi.org/10.1177/1178638820909329, Social network intervention reduces added sugar intake among Baltimore public housing Residents: A feasibility study. Nutrition and Metabolic Insights, 13; Hall, K.L., Oh, A., Perez, L.G., Rice, E.L., Patel, M., Czajkowski, S., Klesges, L., The ecology of multilevel intervention research (2018) Transl. Behav. Med., 8 (6), pp. 968-978; Bennett, G.G., Foley, P., Levine, E., Whiteley, J., Askew, S., Steinberg, D.M., Batch, B., Puleo, E., Behavioral treatment for weight gain prevention among black women in primary care practice: a randomized controlled trial (2013) JAMA Int. Med., 173 (19), p. 1770; Azar, K.M.J., Bennett, G.G., Nolting, L.A., Rosas, L.G., Burke, L.E., Ma, J., A framework for examining the function of digital health technologies for weight management (2018) Transl. Behav. Med., 8 (2), pp. 280-294; Delichatsios, H.K., Friedman, R.H., Glanz, K., Tennstedt, S., Smigelski, C., Pinto, B.M., Kelley, H., Gillman, M.W., Randomized trial of a “talking computer” to improve adults’ eating habits (2001) Am. J. Health Promot., 15 (4), pp. 215-224},
correspondence_address1={Quintiliani, L.M.801 Massachusetts Ave. Crosstown 2, United States; email: lisa.quintiliani@bmc.org},

publisher={Elsevier Inc.},
issn={22113355},
language={English},
abbrev_source_title={Preventive Med. Reports},
document_type={Article},
source={Scopus},
}

@ARTICLE{Meltzer2021,
author={Meltzer, L.J. and Williamson, A.A. and Mindell, J.A.},
title={Pediatric sleep health: It matters, and so does how we define it},
journal={Sleep Medicine Reviews},
year={2021},
volume={57},
doi={10.1016/j.smrv.2021.101425},
art_number={101425},
note={cited By 0},
url={https://www.scopus.com/inward/record.uri?eid=2-s2.0-85101019037&doi=10.1016%2fj.smrv.2021.101425&partnerID=40&md5=c9c368d4e89e2e933a81ca860c86992d},
affiliation={National Jewish Health, Denver, CO 80206, United States; Sleep Center, Children's Hospital of Philadelphia, Philadelphia, PA, United States; Center for Sleep and Circadian Neurobiology, Perelman School of Medicine, University of Pennsylvania, Philadelphia, PA, United States; Department of Psychology, Saint Joseph's University, Philadelphia, PA, United States},
abstract={In 2014, Buysse published a novel definition of sleep health, raising awareness for the importance of this construct for individuals, populations, clinical care, and research. However, the original definition focused on adults, with the recommendation that it should be adapted for children and adolescents. As children live within a complex and dynamic system, and may not always have control over their own sleep, this theoretical review will examine and apply Buysse's five dimensions of sleep health within the context of pediatrics. In addition, using examples from the pediatric sleep literature we introduce a modified definition that takes into consideration the influence of the socio-ecological system within which children live, and the sleep-related behaviors that are critical in supporting or hindering sleep health. Finally, we discuss how the proposed theoretical framework, Peds B-SATED, can be applied to clinical practice, research, and training in the field of pediatric sleep. © 2021 Elsevier Ltd},
author_keywords={Adolescents; Duration; Infant; School-age children; Sleep-efficiency; Sleep-related behaviors; Sleepiness; Timing; Toddler},
references={Buysse, D.J., Sleep health: can we define it? Does it matter? (2014) Sleep, 37 (1), pp. 9-17; Sadeh, A., Anders, T.F., Infant sleep problems: origins, assessment, interventions (1993) Infant Ment Health J, 14 (1), pp. 17-34; Sadeh, A., Tikotzky, L., Scher, A., Parenting and infant sleep (2010) Sleep Med Rev, 14 (2), pp. 89-96; Newton, A.T., Honaker, S.M., Reid, G.J., Risk and protective factors and processes for behavioral sleep problems among preschool and early school-aged children: a systematic review (2020) Sleep Med Rev, 52, p. 101303; Bronfenbrenner, U., The ecology of human development: experiments by nature and design (1979), p. 1979. , Harvard University Press Cambridge, MA; Williamson, A.A., Mindell, J.A., Hiscock, H., Quach, J., Sleep problem trajectories and cumulative socio-ecological risks: birth to school-age (2019) J Pediatr, 215, pp. 229-237; Tietze, A.L., Blankenberg, M., Hechler, T., Michel, E., Koh, M., Schluter, B., Sleep disturbances in children with multiple disabilities (2012) Sleep Med Rev, 16, pp. 117-127; Quach, J.L., Nguyen, C.D.,

Williams, K.E., Sciberras, E., Bidirectional associations between child sleep problems and internalizing and externalizing difficulties from preschool to early adolescence (2018) JAMA Pediatr, 172 (2); Spilsbury, J.C., Patel, S.R., Morris, N., Ehayaei, A., Intille, S.S., Household chaos and sleep-disturbing behavior of family members: results of a pilot study of African American early adolescents (2017) Sleep Health, 3 (2), pp. 84-89; Singh, G.K., Kenney, M.K., Rising prevalence and neighborhood, social, and behavioral determinants of sleep problems in US children and adolescents, 2003-2012 (2013) Sleep Disord, 2013, p. 394320; Bottino, C.J., Rifas-Shiman, S.L., Kleinman, K.P., Oken, E., Redline, S., Gold, D., The association of urbanicity with infant sleep duration (2012) Health Place, 18 (5), pp. 1000-1005; Jarrin, D.C., McGrath, J.J., Quon, E.C., Objective and subjective socioeconomic gradients exist for sleep in children and adolescents (2014) Health Psychol, 33 (3), pp. 301-305; Sheehan, C., Powers, D., Margerison-Zilko, C., McDevitt, T., Cubbin, C., Historical neighborhood poverty trajectories and child sleep (2018) Sleep Health, 4 (2), pp. 127-134; El-Sheikh, M., Bagley, E.J., Keiley, M., Elmore-Staton, L., Chen, E., Buckhalt, J.A., Economic adversity and children's sleep problems: multiple indicators and moderation of effects (2013) Health Psychol, 32 (8), pp. 849-859; School start times for adolescents (2014) Pediatrics, 134 (3), pp. 642-649; Short, M.A., Gradisar, M., Wright, H., Lack, L.C., Dohnt, H., Carskadon, M.A., Time for bed: parent-set bedtimes associated with improved sleep and daytime functioning in adolescents (2011) Sleep, 34 (6), pp. 797-800; Gangwisch, J.E., Babiss, L.A., Malaspina, D., Turner, J.B., Zammit, G.K., Posner, K., Earlier parental set bedtimes as a protective factor against depression and suicidal ideation (2010) Sleep, 33 (1), pp. 97-106; Peltz, J.S., Rogge, R.D., Connolly, H., Parents still matter: the influence of parental enforcement of bedtime on adolescents' depressive symptoms (2020) Sleep, 43 (5); Owens, J., Insufficient sleep in adolescents and young adults: an update on causes and consequences (2014) Pediatrics, 134 (3), pp. e921-e932; Wahlstrom, K.L., Owens, J.A., School start time effects on adolescent learning and academic performance, emotional health and behaviour (2017) Curr Opin Psychiatr, 30 (6), pp. 485-490; Williams, K.E., Berthelsen, D., Walker, S., Nicholson, J.M., A developmental cascade model of behavioral sleep problems and emotional and attentional self-regulation across early childhood (2017) Behav Sleep Med, 15 (1), pp. 1-21; Williams, K.E., Nicholson, J.M., Walker, S., Berthelsen, D., Early childhood profiles of sleep problems and self-regulation predict later school adjustment (2016) Br J Educ Psychol, 86 (2), pp. 331-350; Berger, R.H., Miller, A.L., Seifer, R., Cares, S.R., LeBourgeois, M.K., Acute sleep restriction effects on emotion responses in 30- to 36-month-old children (2012) J Sleep Res, 21 (3), pp. 235-246; Baum, K.T., Desai, A., Field, J., Miller, L.E., Rausch, J., Beebe, D.W., Sleep restriction worsens mood and emotion regulation in adolescents (2014) JCPP (J Child Psychol Psychiatry), 55 (2), pp. 180-190; Benitez, I., Roure, N., Pinilla, L., Sapina-Beltran, E., Buysse, D.J., Barbe, F., Validation of the satisfaction, alertness, timing, efficiency and duration (SATED) questionnaire for sleep health measurement (2020) Ann Am Thorac Soc, 17 (3), pp. 338-343; Ravyts, S.G., Dzierzewski, J.M., Perez, E., Donovan, E.K., Dautovich, N.D., Sleep health as measured by RU SATED: a psychometric evaluation (2019) Behav Sleep Med, pp. 1-9; Becker, N.B., Martins, R.I.S., Jesus, S.N., Chiodelli, R., Stephen, R.M., Sleep health assessment: a scale validation (2018) Psychiatr Res, 259, pp. 51-55; Dong, L., Martinez, A.J., Buysse, D.J., Harvey, A.G., A composite measure of sleep health predicts concurrent mental and physical health outcomes in adolescents prone to eveningness (2019) Sleep Health, 5 (2), pp. 166-174; Knutson, K.L., Phelan, J., Paskow, M.J., Roach, A., Whiton, K., Langer, G., The national sleep

foundation's sleep health index (2017) Sleep Health, 3 (4), pp. 234-240; Ohayon, M.M., Paskow, M., Roach, A., Filer, C., Hillygus, D.S., Chen, M.C., The national sleep foundation's sleep satisfaction tool (2019) Sleep Health, 5 (1), pp. 5-11; Healthy people 2020 2010 https://www.healthypeople.gov/2020/topics-objectives/topic/sleep-health/objectives, [updated 2010]. Available from:; Healthy people 2030 2020 https://www.healthypeople.gov/sites/default/files/ObjectivesPublicComment508.pdf, [updated 2020]. Available from:; Sleep and Health (2019), https://www.cdc.gov/healthyschools/sleep.htm, [updated 2019]. Available from:; Paruthi, S., Brooks, L.J., D'Ambrosio, C., Hall, W.A., Kotagal, S., Lloyd, R.M., Recommended amount of sleep for pediatric populations: a consensus statement of the american academy of sleep medicine (2016) J Clin Sleep Med, 12 (6), pp. 785-786; Carskadon, M.A., Orav, E.J., Dement, W.C., Evolution of sleep and daytime sleepiness in adolescents (1983) Sleep/wake disorders: natural history, epidemiology, and long-term evolution, pp. 201-216. , C. Guilleminault E. Lugaresi Raven Press New York; Bei, B., Manber, R., Allen, N.B., Trinder, J., Wiley, J.F., Too long, too short, or too variable? Sleep intraindividual variability and its associations with perceived sleep quality and mood in adolescents during naturalistically unconstrained sleep (2017) Sleep, 40 (2); El-Sheikh, M., Philbrook, L.E., Kelly, R.J., Hinnant, J.B., Buckhalt, J.A., What does a good night's sleep mean? Nonlinear relations between sleep and children's cognitive functioning and mental health (2019) Sleep, 42 (6); James, S., Hale, L., Sleep duration and child well-being: a nonlinear association (2017) J Clin Child Adolesc Psychol, 46 (2), pp. 258-268; Richards, A., Inslicht, S.S., Metzler, T.J., Mohlenhoff, B.S., Rao, M.N., O'Donovan, A., Sleep and cognitive performance from teens to old age: more is not better (2017) Sleep, 40 (1); Guglielmo, D., Gazmararian, J.A., Chung, J., Rogers, A.E., Hale, L., Racial/ethnic sleep disparities in US school-aged children and adolescents: a review of the literature (2018) Sleep Health, 4 (1), pp. 68-80; Smith, J.P., Hardy, S.T., Hale, L.E., Gazmararian, J.A., Racial disparities and sleep among preschool aged children: a systematic review (2019) Sleep Health, 5 (1), pp. 49-57; El-Sheikh, M., Tu, K.M., Saini, E.K., Fuller-Rowell, T.E., Buckhalt, J.A., Perceived discrimination and youths' adjustment: sleep as a moderator (2016) J Sleep Res, 25 (1), pp. 70-77; Bagley, E.J., Tu, K.M., Buckhalt, J.A., El-Sheikh, M., Community violence concerns and adolescent sleep (2016) Sleep Health, 2 (1), pp. 57-62; Heissel, J.A., Sharkey, P.T., Torrats-Espinosa, G., Grant, K., Adam, E.K., Violence and vigilance: the acute effects of community violent crime on sleep and cortisol (2018) Child Dev, 89 (4), pp. e323-e331; Crosby, B., LeBourgeois, M.K., Harsh, J., Racial differences in reported napping and nocturnal sleep in 2- to 8-year-old children (2005) Pediatrics, 115 (1), pp. 225-232; Lavigne, J.V., Arend, R., Rosenbaum, D., Smith, A., Weissbluth, M., Binns, H.J., Sleep and behavior problems among preschoolers (1999) J Dev Behav Pediatr, 20 (3), pp. 164-169; Mindell, J.A., Sadeh, A., Wiegand, B., How, T.H., Goh, D.Y., Cross-cultural differences in infant and toddler sleep (2010) Sleep Med, 11 (3), pp. 274-280; Buckley, A.W., Rodriguez, A.J., Jennison, K., Buckley, J., Thurm, A., Sato, S., Rapid eye movement sleep percentage in children with autism compared with children with developmental delay and typical development (2010) Arch Pediatr Adolesc Med, 164 (11), pp. 1032-1037; Reynaud, E., Vecchierini, M.F., Heude, B., Charles, M.A., Plancoulaine, S., Sleep and its relation to cognition and behaviour in preschool-aged children of the general population: a systematic review (2018) J Sleep Res, 27 (3); Astill, R.G., Van der Heijden, K.B., van Ijzendoorn, M.H., Van Someren, E.J., Sleep, cognition, and behavioral problems in school-age children: a century of research meta-analyzed (2012) Psychol

Bull, 138 (6), pp. 1109-1138; Dewald, J.F., Meijer, A.M., Oort, F.J., Kerkhof, G.A., Bogels, S.M., The influence of sleep quality, sleep duration and sleepiness on school performance in children and adolescents: a meta-analytic review (2010) Sleep Med Rev, 14 (3), pp. 179-189; Matthews, K.A., Pantesco, E.J., Sleep characteristics and cardiovascular risk in children and adolescents: an enumerative review (2016) Sleep Med, 18, pp. 36-49; Lauderdale, D.S., Knutson, K.L., Yan, L.L., Liu, K., Rathouz, P.J., Self-reported and measured sleep duration: how similar are they? (2008) Epidemiology, 19 (6), pp. 838-845; Jackson, C.L., Patel, S.R., Jackson, W.B., Lutsey, P.L., Redline, S., Agreement between self-reported and objectively measured sleep duration among white, black, Hispanic, and Chinese adults in the United States: multi-Ethnic Study of Atherosclerosis (2018) Sleep, 41 (6); Meltzer, L.J., Forrest, C.B., de la Motte, A., Mindell, J.A., Bevans, K.B., Development and validation of the Pediatric Sleep Practices Questionnaire: a self-report measure for youth ages 8-17 years (2021) Behav Sleep Med, 19 (1), pp. 126-143; Mindell, J.A., Meltzer, L.J., Carskadon, M.A., Chervin, R.D., Developmental aspects of sleep hygiene: findings from the 2004 national sleep foundation sleep in America poll (2009) Sleep Med, 10 (7), pp. 771-779; Waters, E., Stewart-Brown, S., Fitzpatrick, R., Agreement between adolescent self-report and parent reports of health and well-being: results of an epidemiological study (2003) Child Care Health Dev, 29 (6), pp. 501-509; Wolfson, A.R., Carskadon, M.A., Sleep schedules and daytime functioning in adolescents (1998) Child Dev, 69 (4), pp. 875-887; Poulain, T., Vogel, M., Meigen, C., Spielau, U., Hiemisch, A., Kiess, W., Parent-child agreement in different domains of child behavior and health (2020) PloS One, 15 (4); Anders, T.F., Halpern, L.F., Hua, J., Sleeping through the night: a developmental perspective (1992) Pediatrics, 90 (4), pp. 554-560; Burnham, M.M., Goodlin-Jones, B.L., Gaylor, E.E., Anders, T.F., Nighttime sleep-wake patterns and self-soothing from birth to one year of age: a longitudinal intervention study (2002) J Child Psychol Psychiatry Allied Discip, 43 (6), pp. 713-725; Sadeh, A., Lavie, P., Scher, A., Tirosh, E., Epstein, R., Actigraphic home-monitoring of sleep-disturbed and control infants and young children: a new method for pediatric assessment of sleep-wake patterns (1991) Pediatrics, 87, pp. 494-499; Winser, M.A., McBean, A.L., Montgomery-Downs, H.E., Minimum duration of actigraphy-defined nocturnal awakenings necessary for morning recall (2013) Sleep Med, 14 (7), pp. 688-691; Sadeh, A., Mindell, J., Rivera, L., My child has a sleep problem": a cross-cultural comparison of parental definitions (2011) Sleep Med, 12 (5), pp. 478-482; Henderson, J.M., Motoi, G., Blampied, N.M., Sleeping through the night: a community survey of parents' opinions about and expectations of infant sleep consolidation (2013) J Paediatr Child Health, 49 (7), pp. 535-540; Henderson, J.M.T., France, K.G., Owens, J.L., Blampied, N.M., Sleeping through the night: the consolidation of self-regulated sleep across the first year of life (2010) Pediatrics, 126 (5), pp. e1081-e1087; Thomas, K.A., Burr, R.L., Spieker, S., Light and maternal influence in the entrainment of activity circadian rhythm in infants 4-12 weeks of age (2016) Sleep Biol Rhythm, 14 (3), pp. 249-255; Tsai, S.Y., Thomas, K.A., Lentz, M.J., Barnard, K.E., Light is beneficial for infant circadian entrainment: an actigraphic study (2012) J Adv Nurs, 68 (8), pp. 1738-1747; Golem, D., Eck, K.M., Delaney, C.L., Clark, R.L., Shelnutt, K.P., Olfert, M.D., "My stuffed animals help me": the importance, barriers, and strategies for adequate sleep behaviors of school-age children and parents (2019) Sleep Health, 5 (2), pp. 152-160; Martinez, S.M., Thompson-Lastad, A., Latino parents' insight on optimal sleep for their preschool-age child: does context matter? (2015) Acad Pediatr, 15 (6), pp. 636-643; Kalil, A., Dunifon, R., Crosby, D., Su, J.H., Work hours, schedules,

and insufficient sleep among mothers and their young children (2014) J Marriage Fam, 76, pp. 891-904; Lee, S., Hale, L., Berger, L.M., Buxton, O.M., Maternal perceived work schedule flexibility predicts child sleep mediated by bedtime routines (2019) J Child Fam Stud, 28, pp. 245-259; Boles, R.E., Halbower, A.C., Daniels, S., Gunnarsdottir, T., Whitesell, N., Johnson, S.L., Family chaos and child functioning in relation to sleep problems among children at risk for obesity (2017) Behav Sleep Med, 15 (2), pp. 114-128; Hagenauer, M.H., Perryman, J.I., Lee, T.M., Carskadon, M.A., Adolescent changes in the homeostatic and circadian regulation of sleep (2009) Develomental Neuroscience, 31 (4), pp. 276-284; Wittmann, M., Dinich, J., Merrow, M., Roenneberg, T., Social jetlag: misalignment of biological and social time (2006) Chronobiol Int, 23 (1-2), pp. 497-509; Wheaton, A.G., Ferro, G.A., Croft, J.B., School start times for middle school and high school students - United States, 2011-12 School Year (2015) MMWR Morb Mortal Wkly Rep, 64 (30), pp. 809-813; Crowley, S.J., Wolfson, A.R., Tarokh, L., Carskadon, M.A., An update on adolescent sleep: new evidence informing the perfect storm model (2018) J Adolesc, 67, pp. 55-65; Jenni, O.G., Carskadon, M.A., Normal human sleep at different ages: infants to adolescents (2005) SRS basics of sleep guide, pp. 11-19. , Sleep Researcher Society Westchester, IL; Jenni, O.G., LeBourgeois, M.K., Understanding sleep-wake behavior and sleep disorders in children: the value of a model (2006) Curr Opin Psychiatr, 19 (3), pp. 282-287; Jenni, O.G., Achermann, P., Carskadon, M.A., Homeostatic sleep regulation in adolescents (2005) Sleep, 28 (11), pp. 1446-1454; Mindell, J.A., Leichman, E.S., Composto, J., Lee, C., Bhullar, B., Walters, R.M., Development of infant and toddler sleep patterns: real-world data from a mobile application (2016) J Sleep Res, 25 (5), pp. 508-516; Staton, S., Rankin, P.S., Harding, M., Smith, S.S., Westwood, E., LeBourgeois, M.K., Many naps, one nap, none: a systematic review and meta-analysis of napping patterns in children 0-12 years (2019) Sleep Med Rev, 50, p. 101247; Akacem, L.D., Simpkin, C.T., Carskadon, M.A., Wright, K.P., Jr., Jenni, O.G., Achermann, P., The timing of the circadian clock and sleep differ between napping and non-napping toddlers (2015) PloS One, 10 (4); Mindell, J.A., Sadeh, A., Kwon, R., Goh, D.Y., Cross-cultural differences in the sleep of preschool children (2013) Sleep Med, 14 (12), pp. 1283-1289; Liu, J., Feng, R., Ji, X., Cui, N., Raine, A., Mednick, S.C., Midday napping in children: associations between nap frequency and duration across cognitive, positive psychological well-being, behavioral, and metabolic health outcomes (2019) Sleep, 42 (9); Dahl, R.E., The impact of inadequate sleep on children's daytime cognitive function (1996) Semin Pediatr Neurol, 3 (1), pp. 44-50; Beebe, D.W., Cognitive, behavioral, and functional consequences of inadequate sleep in children and adolescents (2011) Pediatr Clin, 58 (3), pp. 649-665; Ji, X., Li, J., Liu, J., The relationship between midday napping and neurocognitive function in early adolescents (2019) Behav Sleep Med, 17 (5), pp. 537-551; Becker, S.P., Sidol, C.A., Van Dyk, T.R., Epstein, J.N., Beebe, D.W., Intraindividual variability of sleep/wake patterns in relation to child and adolescent functioning: a systematic review (2017) Sleep Med Rev, 34, pp. 94-121; Tkachenko, O., Dinges, D.F., Interindividual variability in neurobehavioral response to sleep loss: a comprehensive review (2018) Neurosci Biobehav Rev, 89, pp. 29-48; Ronnlund, H., Elovainio, M., Virtanen, I., Matomaki, J., Lapinleimu, H., Poor parental sleep and the reported sleep quality of their children (2016) Pediatrics, 137 (4); Mindell, J.A., Williamson, A.A., Benefits of a bedtime routine in young children: sleep, development, and beyond (2018) Sleep Med Rev, 40, pp. 93-108; Mindell, J.A., Li, A.M., Sadeh, A., Kwon, R., Goh, D.Y., Bedtime routines for young children: a dose-dependent association with sleep outcomes (2015) Sleep, 38 (5), pp. 717-722; Mindell, J.A., Sadeh, A., Kohyama, J., How, T.H., Parental

behaviors and sleep outcomes in infants and toddlers: a cross-cultural comparison (2010) Sleep Med, 11 (4), pp. 393-399; Palmer, C.A., Clementi, M.A., Meers, J.M., Alfano, C.A., Co-sleeping among school-aged anxious and non-anxious children: associations with sleep variability and timing (2018) J Abnorm Child Psychol, 46 (6), pp. 1321-1332; Yau, S., Pickering, R.M., Gringras, P., Elphick, H., Evans, H.J., Farquhar, M., Sleep in infants and toddlers with Down syndrome compared to typically developing peers: looking beyond snoring (2019) Sleep Med, 63, pp. 88-97; Sadeh, A., Hen-Gal, S., Tikotzky, L., Young children's reactions to war-related stress: a survey and assessment of an innovative intervention (2008) Pediatrics, 121 (1), pp. 46-53; Kushnir, J., Sadeh, A., Assessment of brief interventions for nighttime fears in preschool children (2012) Eur J Pediatr, 171 (1), pp. 67-75; Fitness CoNatCoSMa, Sports drinks and energy drinks for children and adolescents: are they appropriate? (2011) Pediatrics, 127 (6), pp. 1182-1189; Ahluwalia, N., Herrick, K., Caffeine intake from food and beverage sources and trends among children and adolescents in the United States: review of national quantitative studies from 1999 to 2011 (2015) Adv Nutr, 6 (1), pp. 102-111; Ahluwalia, N., Herrick, K., Moshfegh, A., Rybak, M., Caffeine intake in children in the United States and 10-y trends: 2001-2010 (2014) Am J Clin Nutr, 100 (4), pp. 1124-1132; Temple, J.L., Review: trends, safety, and recommendations for caffeine use in children and adolescents (2019) J Am Acad Child Adolesc Psychiatry, 58 (1), pp. 36-45; Buxton, O.M., Chang, A.M., Spilsbury, J.C., Bos, T., Emsellem, H., Knutson, K.L., Sleep in the modern family: protective family routines for child and adolescent sleep (2015) Sleep Health, 1 (1), pp. 15-27; Brambilla, P., Giussani, M., Pasinato, A., Venturelli, L., Privitera, F., Miraglia Del, G.E., Sleep habits and pattern in 1-14 years old children and relationship with video devices use and evening and night child activities (2017) Ital J Pediatr, 43 (1), p. 7; Gamble, A.L., D'Rozario, A.L., Bartlett, D.J., Williams, S., Bin, Y.S., Grunstein, R.R., Adolescent sleep patterns and night-time technology use: results of the Australian Broadcasting Corporation's Big Sleep Survey (2014) PloS One, 9 (11); Dube, N., Khan, K., Loehr, S., Chu, Y., Veugelers, P., The use of entertainment and communication technologies before sleep could affect sleep and weight status: a population-based study among children (2017) Int J Behav Nutr Phys Activ, 14 (1), p. 97; Falbe, J., Davison, K.K., Franckle, R.L., Ganter, C., Gortmaker, S.L., Smith, L., Sleep duration, restfulness, and screens in the sleep environment (2015) Pediatrics, 135 (2), pp. e367-e375; Power, S., Taylor, C., Horton, K., Sleepless in school? The social dimensions of young people's bedtime rest and routines (2017) J Youth Stud, 20 (8), pp. 945-958; Cespedes, E.M., Gillman, M.W., Kleinman, K., Rifas-Shiman, S.L., Redline, S., Taveras, E.M., Television viewing, bedroom television, and sleep duration from infancy to mid-childhood (2014) Pediatrics, 133 (5), pp. e1163-e1171; Browne, D., Thompson, D.A., Madigan, S., Digital media use in children: clinical vs scientific responsibilities (2020) JAMA Pediatr, 174 (2), pp. 111-112; Williamson, A.A., Meltzer, L.J., Fiks, A.G., A stimulus package to address the pediatric sleep debt crisis in the united states (2020) JAMA Pediatr, 174 (2), pp. 115-116; Marcus, C.L., Brooks, L.J., Draper, K.A., Gozal, D., Halbower, A.C., Jones, J., Diagnosis and management of childhood obstructive sleep apnea syndrome (2012) Pediatrics, 130 (3), pp. 576-584; Owens, J.A., Dalzell, V., Use of the 'BEARS' sleep screening tool in a pediatric residents' continuity clinic: a pilot study (2005) Sleep Med, 6, pp. 63-69; Bruni, O., Ottaviano, S., Guidetti, V., Romoli, M., Innocenzi, M., Cortesi, F., The Sleep Disturbance Scale for Children (SDSC) Construction and validation of an instrument to evaluate sleep disturbances in childhood and adolescence (1996) J Sleep Res, 5 (4), pp. 251-261; Lewandowski, A.S.,

Toliver-Sokol, M., Palermo, T.M., Evidence-based review of subjective pediatric sleep measures (2011) J Pediatr Psychol, 36 (7), pp. 780-793; Spruyt, K., Gozal, D., Pediatric sleep questionnaires as diagnostic or epidemiological tools: a review of currently available instruments (2011) Sleep Med Rev, 15 (1), pp. 19-32; Meltzer, L.J., Assessment of sleep problems (2020) Assessment of childhood disorders, , E.A. Youngstrom M.J. Prinstein E.J. Mash R.A. Barkley 5th ed. Guilford Press New York; Owens, J.A., Spirito, A., McGuinn, M., The Children's Sleep Habits Questionnaire (CSHQ): psychometric properties of a survey instrument for school-aged children (2000) Sleep, 23 (8), pp. 1043-1051; Meltzer, L.J., Avis, K.T., Biggs, S., Reynolds, A.C., Crabtree, V.M., Bevans, K.B., The Children's Report of Sleep Patterns (CRSP): a self-report measure of sleep for school-aged children (2013) J Clin Sleep Med, 9 (3), pp. 235-245; Meltzer, L.J., Biggs, S., Reynolds, A., Avis, K.T., Crabtree, V.M., Bevans, K.B., The children's report of sleep patterns - sleepiness scale: a self-report measure for school-aged children (2012) Sleep Med, 13 (4), pp. 385-389; Meltzer, L.J., Brimeyer, C., Russell, K., Avis, K.T., Biggs, S., Reynolds, A.C., The children's report of sleep patterns: validity and reliability of the sleep hygiene index and sleep disturbance scale in adolescents (2014) Sleep Med, 15 (12), pp. 1500-1507; Wolfson, A.R., Carskadon, M.A., Acebo, C., Seifer, R., Fallone, G., Labyak, S.E., Evidence for the validity of a sleep habits survey for adolescents (2003) Sleep, 26 (2), pp. 213-216; Sadeh, A., A brief screening questionnaire for infant sleep problems: validation and findings for an Internet sample (2004) Pediatrics, 113 (6), pp. e570-e577; Mindell, J.A., Gould, R.A., Tikotzy, L., Leichman, E.S., Walters, R.M., Norm-referenced scoring system for the Brief Infant Sleep Questionnaire - Revised (BISQ-R) (2019) Sleep Med, 63, pp. 106-114; Meltzer, L.J., Moore, M., Mindell, J.A., The need for interdisciplinary pediatric sleep clinics (2008) Behav Sleep Med, 6 (4), pp. 268-282; Moore, K.A., Mbwana, K., Theokas, C., Lippman, L., Bloch, M., Vandivere, S., Child well being: an index based on data of individual children. Washington, DC (2011), Report No.: 2011-10; Matricciani, L., Paquet, C., Galland, B., Short, M., Olds, T., Children's sleep and health: a meta-review (2019) Sleep Med Rev, 46, pp. 136-150; Uong, E.C., Jeffe, D.B., Gozal, D., Arens, R., Holbrook, C.R., Palmer, J., Development of a measure of knowledge and attitudes about obstructive sleep apnea in children (OSAKA-KIDS) (2005) Arch Pediatr Adolesc Med, 159 (2), pp. 181-186; Papp, K.K., Penrod, C.E., Strohl, K.P., Knowledge and attitudes of primary care physicians toward sleep and sleep disorders (2002) Sleep Breath, 6 (3), pp. 103-109; Mindell, J.A., Bartle, A., Wahab, N.A., Ahn, Y., Ramamurthy, M.B., Huong, H.T., Sleep education in medical school curriculum: a glimpse across countries (2011) Sleep Med, 12 (9), pp. 928-931; Gamaldo, C.E., Salas, R.E., Sleep medicine education: are medical schools and residency programs napping on the job? (2008) Nat Clin Pract Neurol, 4 (6), pp. 344-345; Mindell, J.A., Bartle, A., Ahn, Y., Ramamurthy, M.B., Huong, H.T., Kohyama, J., Sleep education in pediatric residency programs: a cross-cultural look (2013) BMC Res Notes, 6, p. 130; Faruqui, F., Khubchandani, J., Price, J.H., Bolyard, D., Reddy, R., Sleep disorders in children: a national assessment of primary care pediatrician practices and perceptions (2011) Pediatrics, 128 (3), pp. 539-546; Meltzer, L.J., Phillips, C., Mindell, J.A., Clinical psychology training in sleep and sleep disorders (2009) J Clin Psychol, 65 (3), pp. 305-318; Mindell, J.A., Owens, J.A., Sleep problems in pediatric practice: clinical issues for the pediatric nurse practitioner (2003) J Pediatr Health Care, 17 (6), pp. 324-331; Bonuck, K.A., Schwartz, B., Schechter, C., Sleep health literacy in head start families and staff: exploratory study of knowledge, motivation, and competencies to promote healthy sleep (2016) Sleep Health, 2 (1), pp. 19-24;

Buckhalt, J.A., Wolfson, A.R., El-Sheik, M., Children's sleep and school psychology practice (2009) Sch Psychol Q, 24 (1), pp. 60-69; Chung, K.F., Chan, M.S., Lam, Y.Y., Lai, C.S., Yeung, W.F., School-based sleep education programs for short sleep duration in adolescents: a systematic review and meta-analysis (2017) J Sch Health, 87 (6), pp. 401-408; Blunden, S.L., Chapman, J., Rigney, G.A., Are sleep education programs successful? The case for improved and consistent research efforts (2012) Sleep Med Rev, 16, pp. 355-370; Meltzer, L.J., Wainer, A., Engstrom, E., Pepa, L., Mindell, J.A., Seeing the whole elephant: a scoping review of behavioral treatments for pediatric insomnia (2021) Sleep Med Rev., 56, p. 101410; Malow, B.A., MacDonald, L.L., Fawkes, D.B., Alder, M.L., Katz, T., Teaching children with autism spectrum disorder how to sleep better: a pilot educational program for parents (2016) Clinical Practice in Pediatric Psychology, 4 (2), pp. 125-136; Malow, B.A., Adkins, K.W., Reynolds, A., Weiss, S.K., Loh, A., Fawkes, D., Parent-based sleep education for children with autism spectrum disorders (2014) J Autism Dev Disord, 44 (1), pp. 216-228; Bonuck, K.A., Blank, A., True-Felt, B., Chervin, R., Promoting sleep health among families of young children in Head Start: protocol for a social-ecological approach (2016) Prev Chronic Dis, 13, p. E121; Wilson, K.E., Miller, A.L., Bonuck, K., Lumeng, J.C., Chervin, R.D., Evaluation of a sleep education program for low-income preschool children and their families (2014) Sleep, 37 (6), pp. 1117-1125; Mindell, J.A., Leichman, E.S., Walters, R., Bhullar, B., Development and dissemination of a consumer health information website on infant and toddler sleep. under review; Meltzer, L.J., Wainer, A., Engstrom, E., Pepa, L., Mindell, J.A., Seeing the whole elephant: a scoping review of behavioral treatments for pediatric insomnia (2020) Sleep Med Rev, 56, p. 101410; Jackson, C.L., Walker, J.R., Brown, M.K., Das, R., Jones, N.L., A workshop report on the causes and consequences of sleep health disparities (2020) Sleep, 43 (8); Kelly, R.J., El-Sheik, M., Reciprocal relations between children's sleep and their adjustment over time (2014) Dev Psychol, 50 (4), pp. 1137-1147; Lovato, N., Gradisar, M., A meta-analysis and model of the relationship between sleep and depression in adolescents: recommendations for future research and clinical practice (2014) Sleep Med Rev, 18 (6), pp. 521-529; Bub, K.L., Buckhalt, J.A., El-Sheikh, M., Children's sleep and cognitive performance: a cross-domain analysis of change over time (2011) Dev Psychol, 47 (6), pp. 1504-1514; Friedman, N.P., Corley, R.P., Hewitt, J.K., Wright, K.P., Jr., Individual differences in childhood sleep problems predict later cognitive executive control (2009) Sleep, 32 (3), pp. 323-333; Dutil, C., Chaput, J.P., Inadequate sleep as a contributor to type 2 diabetes in children and adolescents (2017) Nutr Diabetes, 7 (5), p. e266; El-Sheikh, M., Bub, K.L., Kelly, R.J., Buckhalt, J.A., Children's sleep and adjustment: a residualized change analysis (2013) Dev Psychol, 49 (8), pp. 1591-1601; Hash, J.B., Oxford, M.L., Fleming, C.B., Ward, T.M., Spieker, S.J., Sleep problems, daily napping behavior, and social-emotional functioning among young children from families referred to child protective services (2019) Behav Sleep Med, pp. 1-13; Cremone, A., McDermott, J.M., Spencer, R.M.C., Naps enhance executive attention in preschool-aged children (2017) J Pediatr Psychol, 42 (8), pp. 837-845; Miller, A.L., Seifer, R., Crossin, R., LeBourgeois, M.K., Toddler's self-regulation strategies in a challenge context are nap-dependent (2015) J Sleep Res, 24 (3), pp. 279-287; Hoyniak, C.P., Bates, J.E., Staples, A.D., Rudasill, K.M., Molfese, D.L., Molfese, V.J., Child sleep and socioeconomic context in the development of cognitive abilities in early childhood (2019) Child Dev, 90 (5), pp. 1718-1737; Hart, C.N., Cairns, A., Jelalian, E., Sleep and obesity in children and adolescents (2011) Pediatr Clin, 58 (3), pp. 715-733; Chaput, J.P., Gray, C.E., Poitras, V.J., Carson, V., Gruber, R., Olds,

T., Systematic review of the relationships between sleep duration and health
indicators in school-aged children and youth (2016) Appl Physiol Nutr Metabol,
41 (6), pp. S266-S282; Chaput, J.P., Gray, C.E., Poitras, V.J., Carson, V.,
Gruber, R., Birken, C.S., Systematic review of the relationships between sleep
duration and health indicators in the early years (0-4 years) (2017) BMC Publ
Health, 17, p. 855; Short, M.A., Blunden, S., Rigney, G., Matricciani, L.,
Coussens, S., Reynolds, M., Cognition and objectively measured sleep duration
in children: a systematic review and meta-analysis (2018) Sleep Health, 4 (3),
pp. 292-300; Magee, L., Hale, L., Longitudinal associations between sleep
duration and subsequent weight gain: a systematic review (2012) Sleep Med Rev,
16 (3), pp. 231-241; Quist, J.S., Sjodin, A., Chaput, J.P., Hjorth, M.F., Sleep
and cardiometabolic risk in children and adolescents (2016) Sleep Med Rev, 29,
pp. 76-100; Lemola, S., Perkinson-Gloor, N., Brand, S., Dewald-Kaufmann, J.F.,
Grob, A., Adolescents' electronic media use at night, sleep disturbance, and
depressive symptoms in the smartphone age (2015) J Youth Adolesc, 44 (2), pp.
405-418; Mindell, J.A., Lee, C., Sadeh, A., Young child and maternal sleep in
the Middle East (2017) Sleep Med, 32, pp. 75-82; Lee, S., Hale, L., Chang,
A.M., Nahmod, N.G., Master, L., Berger, L.M., Longitudinal associations of
childhood bedtime and sleep routines with adolescent body mass index (2019)
Sleep, 42 (1); Seifert, S.M., Schaechter, J.L., Hershorin, E.R., Lipshultz,
S.E., Health effects of energy drinks on children, adolescents, and young
adults (2011) Pediatrics, 127 (3), pp. 511-528; Forrest, C.B., Meltzer, L.J.,
Marcus, C.L., de la Motte, A., Kratchman, A., Buysse, D.J., Development and
validation of the PROMIS Pediatric Sleep Disturbance and Sleep-Related
Impairment item banks (2018) Sleep, 41 (6); Melendres, M.C.S., Lutz, J.M.,
Rubin, E.D., Marcus, C.L., Daytime sleepiness and hyperactivity in children
with suspected sleep-disordered breathing (2004) Pediatrics, 114 (3), pp.
768-775; Janssen, K.C., Phillipson, S., O'Connor, J., Johns, M.W., Validation
of the epworth sleepiness scale for children and adolescents using rasch
analysis (2017) Sleep Med, 33, pp. 30-35; Chervin, R.D., Hedger, K., Dillon,
J.E., Pituch, K.J., Pediatric sleep questionnaire (PSQ): validity and
reliability of scales for sleep-disordered breathing, snoring, sleepiness, and
behavioral problems (2000) Sleep Med, 1 (1), pp. 21-32; Drake, C., Nickel, C.,
Burduvali, E., Roth, T., Jefferson, C., Pietro, B., The pediatric daytime
sleepiness scale (PDSS): sleep habits and school outcomes in middle-school
children (2003) Sleep, 26 (4), pp. 455-458; Carskadon, M.A., Vieira, C., Acebo,
C., Association between puberty and delayed phase preference (1993) Sleep, 16
(3), pp. 258-262},
correspondence_address1={Meltzer, L.J.; National Jewish Health, 1400 Jackson
Street, G311, United States; email: meltzerL@njhealth.org},
publisher={W.B. Saunders Ltd},
issn={10870792},
coden={SMREF},
language={English},
abbrev_source_title={Sleep Med. Rev.},
document_type={Review},
source={Scopus},
}

@ARTICLE{Kariuki2021,
author={Kariuki, J.K. and Gibbs, B.B. and Erickson, K.I. and Kriska, A. and
Sereika, S. and Ogutu, D. and Milton, H. and Wagner, L.V. and Rao, N. and
Peralta, R. and Bobb, J. and Bermudez, A. and Hirshfield, S. and Goetze, T. and
Burke, L.E.},

title={The feasibility and acceptability of a web-based physical activity for the heart (PATH) intervention designed to reduce the risk of heart disease among inactive African Americans: Protocol for a pilot randomized controlled trial},
journal={Contemporary Clinical Trials},
year={2021},
volume={104},
doi={10.1016/j.cct.2021.106380},
art_number={106380},
note={cited By 0},
url={https://www.scopus.com/inward/record.uri?eid=2-s2.0-85103734768&doi=10.1016%2fj.cct.2021.106380&partnerID=40&md5=4966db9fdf3d0213e5d9dcf3e999651b},
affiliation={University of PittsburghPA, United States; Ritiko Cloud-based Home-care SoftwareMA, United States; New York UniversityNY, United States; Medtronic Medical Device CompanyMN, United States; SUNY Downstate Medical CenterNY, United States; Primary Health NetworkPA, United States},
abstract={Background: Many studies have used the internet to promote physical activity (PA) in several settings, including the home environment, but few have been tailored for African Americans (AAs). To address this research gap, we conducted focus groups with AAs to inform the development of a web-based intervention, Physical Activity for The Heart (PATH), that leverages openly accessible platforms, such as YouTube, to promote PA in any setting. Purpose: To describe the rationale and design of a pilot randomized clinical trial (RCT), that examines the feasibility and acceptability of the PATH intervention among 30 AA adults aged 40--70 years without history of cardiovascular disease. Methods: A 12-week, single-site, wait-listed RCT with subjects randomized 1:1 to either: 1) treatment group - participants receive the PATH intervention, including the online portal and twice a month phone calls from a PA coach, or 2) attention control group - participants receive a self-help PA handout and twice a month general health newsletter. All participants self-monitor step count using actigraphy. The primary outcomes of this 12-week, pilot RCT are recruitment, retention, and adherence to self-monitoring (Actigraph wear time) and the intervention protocol (PATH utilization). The secondary outcomes include changes in PA (step count, moderate-to-vigorous PA, exercise self-efficacy), and cardiometabolic risk (HbA1C, HDL, LDL, total cholesterol, type 2 diabetes risk score, percent body fat, weight, and waist circumference) from baseline to 12 weeks. Conclusions: This study will provide PATH intervention feasibility and acceptability data among inactive AA adults and will inform a future, full-scale RCT testing efficacy. © 2021 Elsevier Inc.},
author_keywords={Cardiometabolic risk; Physical activity; Physical activity tracker; Randomized controlled trial; Self-monitoring; Technology; Web-based},
references={Tipton, C.M., The history of “exercise is medicine” in ancient civilizations (2014) Adv. Physiol. Educ., 38 (2), pp. 109-117; CDC, Facts about Physical Activity | Physical Activity | CDC. Centers for Disease Control and Prevention (2014), https://www.cdc.gov/physicalactivity/data/facts.htm, Published 2014. Accessed February 7, 2017; U.S. Department of Health and Human Services, Physical Activity Guidelines for Americans (2018), https://health.gov/paguidelines/second-edition/pdf/Physical_Activity_Guidelines_2nd_edition.pdf, Accessed November 13, 2018; National Center for Health Statistics, Leisure-Time Physical Activity (1997); Siddiqi, Z., Tiro, J.A., Shuval, K., Understanding impediments and enablers to physical activity among African American adults: a systematic review of qualitative studies (2011) Health Educ. Res., 26 (6), pp. 1010-1024; Durant, N.H., Joseph, R.P.,

Cherrington, A., Recommendations for a culturally relevant internet-based tool to promote physical activity among overweight young African American women, Alabama, 2010–2011 (2014) Prev. Chronic Dis., 11, pp. 130-169; Mendoza-Vasconez, A.S., Linke, S., Munoz, M., Promoting physical activity among underserved populations (2016) Curr. Sports Med. Rep., 15 (4), pp. 290-297; Pekmezi, D., Ainsworth, C., Joseph, R., Rationale, design, and baseline findings from HIPP: a randomized controlled trial testing a home-based, individually-tailored physical activity print intervention for African American women in the deep south (2016) Contemp. Clin. Trials, 47, pp. 340-348; King, A.C., Haskell, W.L., Taylor, C.B., Kraemer, H.C., DeBusk, R.F., Group- vs home-based exercise training in healthy older men and women (1991) JAMA, 266 (11), pp. 1535-1542. , https://www.ncbi.nlm.nih.gov/pubmed/1880885; Jahangiry, L., Farhangi, M.A., Shab-Bidar, S., Rezaei, F., Pashaei, T., Web-based physical activity interventions: a systematic review and meta-analysis of randomized controlled trials (2017) Public Health, 152, pp. 36-46; Joseph, R.P., Durant, N.H., Benitez, T.J., Pekmezi, D.W., Internet-based physical activity interventions (2014) Am. J. Lifestyle Med., 8 (1), pp. 42-67; Newton, R.L., Perri, M.G., A randomized pilot trial of exercise promotion in sedentary African-American adults (2004) Ethn. Dis., 14 (4), pp. 548-557. , http://www.ncbi.nlm.nih.gov/pubmed/15724775, (Accessed 4 December 2018); Pekmezi, D., Ainsworth, C., Joseph, R.P., Pilot trial of a home-based physical activity program for African American women HHS public access (2017) Med. Sci. Sports Exerc., 49 (12), pp. 2528-2536; Pekmezi, D., Ainsworth, C., Desmond, R., Physical activity maintenance following home-based, individually tailored print interventions for African American women (2018) Heal. Promot. Pract. Mon. XXXX, 20 (2), pp. 1-9; Whitt-Glover, M.C., Kumanyika, S.K., Systematic review of interventions to increase physical activity and physical fitness in African-Americans (2009) Am. J. Health Promot., 23 (6_suppl), pp. S33-S56; Kariuki, J.K., Gibbs, B.B., Davis, K.K., Mecca, L.P., Hayman, L.L., Burke, L.E., Recommendations for a culturally salient web-based physical activity program for African Americans (2019) Transl J. Am. Coll. Sport Med., 4 (2). , https://journals.lww.com/acsm-tj/Fulltext/2019/01150/Recommendations_for_a_Culturally_Salient_Web_Based.1.aspx; Conn, V.S., Coon Sells, T.G., Effectiveness of interventions to increase physical activity among minority populations: an umbrella review (2016) J. Natl. Med. Assoc., 108 (1), pp. 54-68; Pekmezi, D.W., Barbera, B.L., Bodenlos, J.S., WN, S.C.G.J., Brantley, P.J., Promoting physical activity in low income African Americans: project LAPS (2009) J. Health Dispar. Res. Pract., 3 (2). , https://digitalscholarship.unlv.edu/cgi/viewcontent.cgi?referer=https://www.google.com/&httpsredir=1&article=1061&context=jhdrp, Accessed May 30, 2018; Hills, A.P., Byrne, N.M., State of the Science: A Focus on Physical Activity (2006), 15. , http://apjcn.nhri.org.tw/server/APJCN/15Suppl//40.pdf, Accessed June 7, 2019; Mcintosh, T., Hunter, D.J., Royce, S., Barriers to physical activity in obese adults: a rapid evidence assessment (2016) UK J. Res. Nurs., 21 (4), pp. 271-287; Bandura, A., Social Foundations of Thought and Action: A Social Cognitive Theory (1986), Prentice-Hall; Pescatello, L.S., American College of Sports Medicine. ACSM's Guidelines for Exercise Testing and Prescription (2014), https://www.worldcat.org/title/acsms-guidelines-for-exercise-testing-and-prescription/oclc/857906042, 9th ed. Wolters Kluwer/Lippincott Williams & Wilkins Health Accessed October 24, 2018; Wilson, P., Overview of established risk factors for cardiovascular disease (2019) UptoDate, , https://www.uptodate.com/contents/overview-of-established-risk-factors-for-cardiovascular-disease, Accessed March 24, 2021; Be, S.A., Active Your Way a Guide

for Adults Based on the 2008 Physical Activity Guidelines for Americans https://health.gov/paguidelines/pdf/adultguide.pdf, Accessed January 10, 2018; Kriska, A.M., Knowler, W.C., LaPorte, R.E., Development of questionnaire to examine relationship of physical activity and diabetes in Pima Indians (1990) Diabetes Care, 13 (4), pp. 401-411; National Cancer Institute, Automated Self-Administered 24-Hour (ASA24®) Dietary Assessment Tool (2016), https://epi.grants.cancer.gov/asa24/, Published 2016. Accessed July 12, 2017; Canadian Society for Exercise Physiology, Physical Activity Readiness Questionnaire (PAR-Q) (2002), https://www2.fgcu.edu/mariebcollege/RS/files/EIM_PAR_Q1.pdf, Published 2002. Accessed January 21, 2018; Bang, H., Edwards, A.M., Bomback, A.S., Development and validation of a patient self-assessment score for diabetes risk (2009) Ann. Intern. Med., 151 (11), p. 775; Mcauley, E., The role of efficacy cognitions in the prediction of exercise behavior in middle-aged adults (1992) J. Behav. Med., 15 (1); Paffenbarger, R.S., Wing, A.L., Hyde, R.T., Physical activity as an index of heart attack risk in college alumni (1978) Am. J. Epidemiol., 108 (3), pp. 161-175. , http://www.ncbi.nlm.nih.gov/pubmed/707484, Accessed January 31, 2018; Yu, L., Buysse, D.J., Germain, A., Development of short forms from the PROMISTM sleep disturbance and sleep-related impairment item banks (2011) Behav. Sleep Med., 10 (1), pp. 6-24; Van Dam, N.T., Earleywine, M., Validation of the Center for Epidemiologic Studies Depression Scale-Revised (CESD-R): pragmatic depression assessment in the general population (2011) Psychiatry Res., 186, pp. 128-132; Salsman, J.M., Butt, Z., Pilkonis, P.A., Emotion assessment using the NIH toolbox (2013) Neurology, 80 (11 Suppl. 3), p. S76; Balestroni, G., Bertolotti, G., EuroQol-5D (EQ-5D): an instrument for measuring quality of life (2012) Monaldi Arch Chest Dis. Card. Ser., 78 (3), pp. 155-159; Nes, B.M., Janszky, I., Vatten, L.J., Nilsen, T.I.L., Aspenes, S.T., WislØff, U., Estimating V̇O2peak from a nonexercise prediction model: the HUNT study, Norway (2011) Med. Sci. Sports Exerc., 43 (11), pp. 2024-2030; Hall, J.M., Fowler, C.F., Barrett, F., Humphry, R.W., Van Drimmelen, M., MacRury, S.M., HbA1C determination from HemaSpotTM blood collection devices: comparison of home prepared dried blood spots with standard venous blood analysis (2019) Diabet. Med., , Published online October 17, 2019:dme.14110; (2011), myhealthywaist.org, International Chair on Cardiometabolic Risk. Waist Circumference Measurement Guidelines. Published 2011. Accessed November 10, 2020 http://www.myhealthywaist.org/index.php?id=90; Omron Healthcare, 10 Series Wireless Upper Arm Blood Pressure Monitor (2020), https://omronhealthcare.com/products/10-series-wireless-upper-arm-blood-pressure-monitor-bp7450/?bvstate=pg:2/ct:r, OMRON Healthcare Published 2020. Accessed July 31, 2020; CoreMedicaLabs, HemaSpot-SE™ Collection Instruction (2020), https://www.coremedicalabs.com/blood-ds/, Accessed November 10, 2020; Lim, C.Y., In, J., Randomization in clinical studies (2019) Korean J. Anesthesiol., 72 (3), pp. 221-232; ActiGraph, L.L.C., ActiGraph Link | ActiGraph (2018), http://actigraphcorp.com/products-showcase/activity-monitors/actigraph-link/, Published 2018. Accessed January 25, 2018; YouTube LLC, YouTube (2017), https://www.youtube.com/, Published 2017. Accessed November 9, 2017; Bandura, A., Self-efficacy: toward a unifying theory of behavioral change (1977) Psychol. Rev., 84 (2), pp. 191-215; Guo, Y., Bian, J., Leavitt, T., Assessing the quality of mobile exercise apps based on the American college of sports medicine guidelines: a reliable and valid scoring instrument (2017) J. Med. Internet Res., 19 (3); Borg, G., An Introduction to Borg's RPE-Scale (1985), http://www.worldcat.org/title/introduction-to-borgs-rpe-scale/oclc/13790520?referer=di&ht=edition, Mouvement Publications Accessed September 13, 2017; Covington, P., Adams, J., Sargin, E., Deep neural networks for YouTube

recommendations (2016) Proceedings of the 10th ACM Conference on Recommender Systems - RecSys ‘16, pp. 191-198. , ACM Press; ActiGraph, Centrepoint Data Hub Real-World Insights in near Real Time (2017), https://s3.amazonaws.com/actigraphcorp.com/wp-content/uploads/2018/02/22120735/ActiGraph_CentrePointDataHub_Tri-FoldBrochure2017_FINAL_Pages_WEB.pdf, Accessed January 24, 2019; Pescatello, L.S., Arena, R., Riebe, D., Thompson, P.D., ACSM'S Guidelines for Exercise Testing and Prescription www.acsm.org, Accessed July 28, 2020; (2018), https://health.gov/paguidelines/guidelines/chapter6.aspx, Office of Disease Prevention and Health Promotion. Physical Activity Guidelines: Safe and Active. Published 2018. Accessed January 28, 2018 Health.gov; Bandura, A., (2019), http://pitt.idm.oclc.org/login?url=https://search.proquest.com/docview/619147930?accountid=14709, Self-Efficacy: The Exercise of Control. W H Freeman/Times Books/ Henry Holt & Co Accessed January 16; Sasaki, J.E., John, D., Freedson, P.S., Validation and comparison of ActiGraph activity monitors (2011) J. Sci. Med. Sport, 14 (5), pp. 411-416; Harris, P.A., Taylor, R., Thielke, R., Payne, J., Gonzalez, N., Conde, J.G., Research electronic data capture (REDCap)-A metadata-driven methodology and workflow process for providing translational research informatics support (2009) J. Biomed. Inform., 42 (2), pp. 377-381; Booth, F.W., Lees, S.J., Fundamental questions about genes, inactivity, and chronic diseases (2007) Physiol. Genomics, 28 (2), pp. 146-157. , (doi:00174.2006 [pii); Kraus, W.E., Bittner, V., Appel, L., The National Physical Activity Plan: a call to action from the American Heart Association: a science advisory from the American Heart Association (2015) Circulation, 131 (21), pp. 1932-1940; Pekmezi, D.W., Williams, D.M., Dunsiger, S., Feasibility of using computer-tailored and internet-based interventions to promote physical activity in underserved populations (2010) Telemed. J. E Health, 16 (4), pp. 498-503; Unwin, T., de Bastion, G., Digital divide (2009) International Encyclopedia of Human Geography, pp. 191-197; Anderson, M., Perrin, A., 10% of Americans Don't Use the Internet | Pew Research Center (2019), https://www.pewresearch.org/fact-tank/2019/04/22/some-americans-dont-use-the-internet-who-are-they/, Pew Research Center Published 2019. Accessed June 9, 2020; Larsen, B., Marcus, B., Pekmezi, D., Hartman, S., Gilmer, T., A web-based physical activity intervention for spanish-speaking latinas: a costs and cost-effectiveness analysis (2017) J. Med. Internet Res., 19 (2); Marcus, B.H., Hartman, S.J., Pekmezi, D., Using interactive Internet technology to promote physical activity in Latinas: rationale, design, and baseline findings of Pasos Hacia La Salud (2015) Contemp. Clin. Trials, 44, pp. 149-158; Pekmezi, D., Marcus, B., Meneses, K., Developing an intervention to address physical activity barriers for African-American women in the deep south (USA) (2013) Women's Health (Lond. Engl.), 9 (3), pp. 301-312; Schunk, D.H., Self-efficacy, motivation, and performance (1995) J. Appl. Sport Psychol., 7 (2). , https://pdfs.semanticscholar.org/5b0f/dd0ea7a7a80d06dd1631c90634d23bf34e5e.pdf, Accessed January 31, 2018},
correspondence_address1={Kariuki, J.K.; University of Pittsburgh, 3500 Victoria Street, United States; email: kigok@pitt.edu},
publisher={Elsevier Inc.},
issn={15517144},
pubmed_id={33798731},
language={English},
abbrev_source_title={Contemp. Clin. Trials},
document_type={Article},
source={Scopus},
}

@ARTICLE{Epstein2021,
author={Epstein, E. and Patel, N. and Maysent, K. and Taub, P.R.},
title={Cardiac Rehab in the COVID Era and Beyond: mHealth and Other Novel Opportunities},
journal={Current Cardiology Reports},
year={2021},
volume={23},
number={5},
doi={10.1007/s11886-021-01482-7},
art_number={42},
note={cited By 0},
url={https://www.scopus.com/inward/record.uri?eid=2-s2.0-85102377238&doi=10.1007%2fs11886-021-01482-7&partnerID=40&md5=ceb69e7c3be0e8c6e3dfb2748747c4f4},
affiliation={University of California, 9300 Campus Point Drive, La Jolla, San Diego, CA 92037, United States},
abstract={Purpose of Review: The COVID-19 pandemic has forced many center-based cardiac rehabilitation (CBCR) programs to close or limit their usual offerings. In order for patients to continue to benefit from CR, programs need to rapidly adapt to the current environment. This review highlights ways CR has evolved, and reviews the history of CR and recent advancements in telemedicine including remote patient monitoring, and mobile health that can be applied to CR. Recent Findings: Despite that initial studies indicate that home-based CR (HBCR) is safe and effective, HBCR has faced several challenges that have prevented it from becoming more widely implemented. Many previous concerns can now be addressed through the use of new innovations in home-based healthcare delivery. Summary: Since its inception, CR has become increasingly recognized as an important tool to improve patient mortality and quality of life in a broad range of cardiac diseases. While there has been little need to modify the delivery of CR since the 1950s, COVID-19 now serves as the necessary impetus to make HBCR an equal alternative to CBCR. © 2021, The Author(s), under exclusive licence to Springer Science+Business Media, LLC part of Springer Nature.},
author_keywords={Cardiac rehabilitation; COVID-19; Mobile health; Prevention; Secondary prevention; Telemedicine},
keywords={human; pandemic; quality of life; telemedicine, COVID-19; Humans; Pandemics; Quality of Life; SARS-CoV-2; Telemedicine},
references={Pashkow, F.J., Issues in contemporary cardiac rehabilitation: a historical perspective (1993) J Am Coll Cardiol, 21, pp. 822-834. , COI: 1:STN:280:DyaK3s7ntFCrug%3D%3D, PID: 8436764; Hellerstein, H.K., Ford, A.B., Rehabilitation of the cardiac patient (1957) J Am Med Assoc, 164, pp. 225-231. , COI: 1:STN:280:DyaG2s%2Fmt1ehug%3D%3D, PID: 13415965; (2020), https://www.cdc.gov/nchs/fastats/leading-causes-of-death.htm; The Top 10 Causes of Death, , https://www.who.int/en/news-room/fact-sheets/detail/the-top-10-causes-of-death; Balady, G.J., Cardiac rehabilitation programs (1994) Circulation, 90, pp. 1602-1607; Horgan, J., Bethell, H., Working party report on cardiac rehabilitation (1992) Br Heart J, 67, pp. 412-418. , COI: 1:STN:280:DyaK3s%2FgtFyhug%3D%3D, PID: 1389724; Anderson, L., Oldridge, N., Exercise-based cardiac rehabilitation for coronary heart disease: Cochrane systematic review and meta-analysis (2016) J Am Coll Cardiol, 67, pp. 1-12. , PID: 26764059; Rauch, B., Davos, C.H., The prognostic effect of cardiac rehabilitation in the era of acute revascularisation and statin therapy: a systematic review and meta-analysis of randomized and non-randomized studies--The Cardiac Rehabilitation Outcome Study (CROS) (2016) Eur J Prev

Cardiol, 23, pp. 1914-1939. , PID: 27777324; de Vries, H., Kemps, H.M.C., van Engen-Verheul, M.M., Kraaijenhagen, R.A., Peek, N., Cardiac rehabilitation and survival in a large representative community cohort of Dutch patients (2015) Eur Heart J, 36, pp. 1519-1528. , PID: 25888007; Freeman, A.M., Taub, P.R., Lo, H.C., Ornish, D., Intensive cardiac rehabilitation: an underutilized resource (2019) Curr Cardiol Rep, 21, p. 19. , PID: 30828747; Edelmann, F., Exercise training improves exercise capacity and diastolic function in patients with heart failure with preserved ejection fraction: results of the Ex-DHF (Exercise training in Diastolic Heart Failure) pilot study (2011) J Am Coll Cardiol, 58, pp. 1780-1791. , PID: 21996391; Klempfner, R., Participation in an exercise-based cardiac rehabilitation program and functional improvement of heart failure patients with preserved versus reduced left ventricular systolic function (2018) Isr Med Assoc J, 20, pp. 358-362. , PID: 29911756; Carlson, J.J., Johnson, J.A., Franklin, B.A., VanderLaan, R.L., Program participation, exercise adherence, cardiovascular outcomes, and program cost of traditional versus modified cardiac rehabilitation (2000) Am J Cardiol, 86, pp. 17-23. , COI: 1:STN:280:DC%2BD3czislSqtQ%3D%3D, PID: 10867086; Smith Sidney, C., AHA/ACCF secondary prevention and risk reduction therapy for patients with coronary and other atherosclerotic vascular disease: 2011 update (2011) Circulation, 124, pp. 2458-2473. , PID: 22052934; Businessweek, B., Pritikin Health Resort | Voted Best Weight Loss Resort, , https://www.pritikin.com/; Smart, N., Marwick, T.H., Exercise training for patients with heart failure: a systematic review of factors that improve mortality and morbidity (2004) Am J Med, 116, pp. 693-706. , PID: 15121496; Ornish, D., Intensive lifestyle changes for reversal of coronary heart disease (1998) JAMA, 280, pp. 2001-2007. , COI: 1:STN:280:DyaK1M%2FnvFGmtw%3D%3D, PID: 9863851; Dominique, W., Abstract P325: effectiveness of the first outpatient Pritikin Intensive Cardiac Rehabilitation (ICR) program (2019) Circulation, 139, p. AP325; Khera, A., Continuity of care and outpatient management for patients with and at high risk for cardiovascular disease during the COVID-19 pandemic: a scientific statement from the American Society for Preventive Cardiology (2020) Am J Prev Cardiol, 1, p. 100009. , PID: 32835347; Beatty, A.L., Geographic variation in cardiac rehabilitation participation in Medicare and veterans affairs populations: opportunity for improvement (2018) Circulation, 137, pp. 1899-1908. , PID: 29305529; Schultz, W.M., Socioeconomic status and cardiovascular outcomes: challenges and interventions (2018) Circulation, 137, pp. 2166-2178. , PID: 29760227; Shanshan, L., Sex and racial disparities in cardiac rehabilitation referral at hospital discharge and gaps in long-term mortality (2018) J am Heart Assoc, 7 (8). , https://doi.org/10.1161/JAHA.117.008088; Woolhandler, S., Himmelstein, D.U., Intersecting U.S. epidemics: COVID-19 and lack of health insurance (2020) Ann Intern Med, 173, pp. 63-64. , PID: 32259195; (2020), https://covid.cdc.gov/covid-data-tracker/, COVID-19 cases, deaths, and trends in the US; Aragam, K.G., Gaps in referral to cardiac rehabilitation of patients undergoing percutaneous coronary intervention in the United States (2015) J Am Coll Cardiol, 65, pp. 2079-2088. , PID: 25975470; Franklin, B.A., Reed, P.S., Gordon, S., Timmis, G.C., Instantaneous electrocardiography. A simple screening technique for cardiac exercise programs (1989) Chest, 96, pp. 174-177. , COI: 1:STN:280:DyaL1M3ovFWltQ%3D%3D, PID: 2736972; Haskell, W.L., The efficacy and safety of exercise programs in cardiac rehabilitation (1994) Med Sci Sports Exerc, 26, pp. 815-823. , COI: 1:STN:280:DyaK2M%2FitVCmtA%3D%3D, PID: 7934753; Pavy, B., Safety of exercise training for cardiac patients: results of the French registry of complications during cardiac rehabilitation (2006) Arch Intern Med, 166, pp. 2329-2334. , PID: 17130385; Thomas, R.J., Home-based cardiac rehabilitation: a scientific statement from the American Association of

Cardiovascular and Pulmonary Rehabilitation, the American Heart Association, and the American College of Cardiology (2019) J Am Coll Cardiol, 74, pp. 133-153. , PID: 31097258; Miller, N.H., Haskell, W.L., Berra, K., DeBusk, R.F., Home versus group exercise training for increasing functional capacity after myocardial infarction (1984) Circulation, 70, pp. 645-649. , COI: 1:STN:280:DyaL2M%2Fgt1ahtw%3D%3D, PID: 6478567; Bell, J.M., (1998) A Comparison of a Multi-Disciplinary Home Based Cardiac Rehabilitation Programme with Comprehensive Conventional Rehabilitation in Post-Myocardial Infarction Patients, , https://spiral.imperial.ac.uk/bitstream/10044/1/8585/1/Jennifer_M_Bell-1999-PhD-Thesis.pdf; Moholdt, T., Bekken Vold, M., Grimsmo, J., Slørdahl, S.A., Wisløff, U., Home-based aerobic interval training improves peak oxygen uptake equal to residential cardiac rehabilitation: a randomized, controlled trial (2012) PLoS One, 7. , COI: 1:CAS:528:DC%2BC38XhtV2msLjO, PID: 22815970; Daskapan, A., Arikan, H., Caglar, N., Tunali, N., Ataman, S., Comparison of supervised exercise training and home-based exercise training in chronic heart failure (2005) Saudi Med J, 26, pp. 842-847. , PID: 15951880; Oerkild, B., Home-based cardiac rehabilitation is as effective as centre-based cardiac rehabilitation among elderly with coronary heart disease: results from a randomised clinical trial (2011) Age Ageing, 40, pp. 78-85. , PID: 20846961; Piotrowicz, E., A new model of home-based telemonitored cardiac rehabilitation in patients with heart failure: effectiveness, quality of life, and adherence (2010) Eur J Heart Fail, 12, pp. 164-171. , PID: 20042423; Dalal, H.M., Home-based versus hospital-based rehabilitation after myocardial infarction: a randomized trial with preference arms--Cornwall Heart Attack Rehabilitation Management Study (CHARMS) (2007) Int J Cardiol, 119, pp. 202-211. , COI: 1:STN:280:DC%2BD2szivFSgsg%3D%3D, PID: 17196274; Jolly, K., The Birmingham Rehabilitation Uptake Maximisation study (BRUM): a randomised controlled trial comparing home-based with centre-based cardiac rehabilitation (2009) Heart, 95, pp. 36-42. , COI: 1:STN:280:DC%2BD1cjnsFSrsQ%3D%3D, PID: 18332063; O’Connor, C.M., Efficacy and safety of exercise training in patients with chronic heart failure: HF-ACTION randomized controlled trial (2009) JAMA, 301, pp. 1439-1450. , PID: 19351941; Snoek, J.A., Effectiveness of home-based mobile guided cardiac rehabilitation as alternative strategy for nonparticipation in clinic-based cardiac rehabilitation among elderly patients in Europe: A randomized clinical trial (2020) JAMA Cardiol, , https://doi.org/10.1001/jamacardio.2020.5218, This randomized controlled trial in 179 patients demonstrates that home-based cardiac rehabilitation can be safely and effectively implemented in a group of patients who had previously refused referral to center-based cardiac rehabilitation; https://www.cms.gov/newsroom/press-releases/trump-administration-drives-telehealth-services-medicaid-and-medicare, Trump Administration Drives Telehealth Services in Medicaid and Medicare; Koonin, L.M., Trends in the use of Telehealth during the emergence of the COVID-19 pandemic-United States, January-March 2020 (2020) MMWR Morb Mortal Wkly Rep, 69, pp. 1595-1599. , COI: 1:CAS:528:DC%2BB3cXit1Kms7nL, PID: 33119561; Hollander Judd, E., Sites Frank D. The transition from reimagining to recreating health care is now Catalyst Non-Issue Content, 1 (2). , https://doi.org/10.1056/CAT.20.0093; Behere, S., Baffa, J.M., Penfil, S., Slamon, N., Real-world evaluation of the Eko electronic teleauscultation system (2019) Pediatr Cardiol, 40, pp. 154-160. , PID: 30171267; Attia Zachi, I., Abstract 13447: prospective analysis of utility of signals from an Ecg-enabled stethoscope to automatically detect a low ejection fraction using neural network techniques trained from the standard 12-lead Ecg (2019) Circulation, 140, p. A13447; White Brent, E., Abstract

13831: handheld wireless digital phonocardiography for machine learning-based detection of mitral regurgitation (2019) Circulation, 140, p. A13831; Croteau, J., (2019) Can at-home lab testing answer your health needs? Forbes Magazine, , https://www.forbes.com/sites/jeannecroteau/2019/09/30/can-at-homelab-testing-answer-your-health-needs/, Accessed Sept 30 2019; Yilmaz, G., A wearable stethoscope for long-term ambulatory respiratory health monitoring (2020) Sensors, 20 (18). , (,)., https://doi.org/10.3390/s20185124; Cao, M., Ambulatory monitoring of heart sounds via an implanted device is superior to auscultation for prediction of heart failure events (2020) J Card Fail, 26, pp. 151-159. , PID: 31634574; Abraham, W.T., Wireless pulmonary artery haemodynamic monitoring in chronic heart failure: a randomised controlled trial (2011) Lancet, 377, pp. 658-666. , PID: 21315441; Tison, G.H., Passive detection of atrial fibrillation using a commercially available smartwatch (2018) JAMA Cardiol, 3, pp. 409-416. , PID: 29562087; Strik, M., Validating QT-interval measurement using the Apple watch ECG to enable remote monitoring during the COVID-19 pandemic (2020) Circulation, 142, pp. 416-418. , COI: 1:CAS:528:DC%2BB3cXhsVygs77I, PID: 32478565; Spaccarotella, C.A.M., Multichannel electrocardiograms obtained by a smartwatch for the diagnosis of ST-segment changes (2020) JAMA Cardiol, , https://doi.org/10.1001/jamacardio.2020.3994; Effectiveness of continuous glucose monitoring in a clinical care environment: evidence from the Juvenile Diabetes Research Foundation continuous glucose monitoring (JDRF-CGM) trial (2010) Diabetes Care, 33, pp. 17-22. , COI: 1:CAS:528:DC%2BC3cXht1KitL4%3D; Beck, R.W., Effect of continuous glucose monitoring on glycemic control in adults with type 1 diabetes using insulin injections: the DIAMOND randomized clinical trial (2017) JAMA, 317, pp. 371-378. , COI: 1:CAS:528:DC%2BC2sXhtlSnsL7N, PID: 28118453; Battelino, T., The use and efficacy of continuous glucose monitoring in type 1 diabetes treated with insulin pump therapy: a randomised controlled trial (2012) Diabetologia, 55, pp. 3155-3162. , COI: 1:CAS:528:DC%2BC38XhsF2qt7zN, PID: 22965294; Pratley, R.E., Effect of continuous glucose monitoring on hypoglycemia in older adults with type 1 diabetes: a randomized clinical trial (2020) JAMA, 323, pp. 2397-2406. , COI: 1:CAS:528:DC%2BB3cXht1GgsLvM, PID: 32543682; Ettefagh, L., The prevalence of impaired glucose metabolism in patients referred to cardiac rehabilitation (2013) J Cardiopulm Rehabil Prev, 33, pp. 42-46. , PID: 23221808; Mourot, L., Cardiovascular rehabilitation in patients with diabetes (2010) J Cardiopulm Rehabil Prev, 30, pp. 157-164. , PID: 20040882; https://www.who.int/goe/publications/goe_mhealth_web.pdf, mHealth: new horizons for health through mobile technologies. World Health Organization; Conceptualizing a Data Infrastructure for the Capture, Use, and Sharing of Patient-Generated Health Data in Care Delivery and Research through 2024, , https://www.healthit.gov/sites/default/files/onc_pghd_final_white_paper.pdf, Accessed Feb 25 2021; Treskes, R.W., Effect of smartphone-enabled health monitoring devices vs regular follow-up on blood pressure control among patients after myocardial infarction: a randomized clinical trial (2020) JAMA Netw Open, 3. , PID: 32297946, This randomized controlled trial demonstrates that patients can successfully utilize mobile health (mHealth) to generate data; Gill, S., Panda, S., A smartphone app reveals erratic diurnal eating patterns in humans that can be modulated for health benefits (2015) Cell Metab, 22, pp. 789-798. , COI: 1:CAS:528:DC%2BC2MXhsFKqsbnF, PID: 26411343; Lu, X., Yang, H., Xia, X., Lu, X., Lin, J., Liu, F., Gu, D., Interactive mobile health intervention and blood pressure management in adults (2019) Hypertension, 74, pp. 697-704. , COI: 1:CAS:528:DC%2BC1MXhsFCqur%2FO, PID: 31327259; Rehman, H., Kamal, A.K., Sayani, S., Morris, P.B., Merchant, A.T., Virani, S.S., Using

mobile health (mHealth) technology in the management of diabetes mellitus, physical inactivity, and smoking (2017) Curr Atheroscler Rep, 19, p. 16. , PID: 28243807;
https://www.acc.org/latest-in-cardiology/articles/2017/08/03/08/27/managing-hyperlipidemia-on-the-go, Managing hyperlipidemia on the go: using mobile technology to lower cholesterol levels-American College of Cardiology. n.d., Accessed Nov 15 2020; Maddison, R., Pfaeffli, L., Whittaker, R., Stewart, R., Kerr, A., Jiang, Y., Kira, G., Rawstorn, J., A mobile phone intervention increases physical activity in people with cardiovascular disease: results from the HEART randomized controlled trial (2015) Eur J Prev Cardiol, 22, pp. 701-709. , PID: 24817694; Nes, B.M., Gutvik, C.R., Lavie, C.J., Nauman, J., Wisløff, U., Personalized activity intelligence (PAI) for prevention of cardiovascular disease and promotion of physical activity (2017) Am J Med, 130, pp. 328-336. , PID: 27984009; Zisko, N., Skjerve, K.N., Tari, A.R., Sandbakk, S.B., Wisløff, U., Nes, B.M., Nauman, J., Personal activity intelligence (PAI), sedentary behavior and cardiovascular risk factor clustering-the HUNT Study (2017) Prog Cardiovasc Dis, 60, pp. 89-95. , PID: 28274818; Nauman, J., Nes, B.M., Zisko, N., Revdal, A., Myers, J., Kaminsky, L.A., Wisløff, U., Personal activity intelligence (PAI): a new standard in activity tracking for obtaining a healthy cardiorespiratory fitness level and low cardiovascular risk (2019) Prog Cardiovasc Dis, 62, pp. 179-185. , PID: 30797801; Widmer, R.J., Digital health intervention during cardiac rehabilitation: a randomized controlled trial (2017) Am Heart J, 188, pp. 65-72. , PID: 28577682, This randomized controlled trial demonstrates that mobile health can improve outcomes cardiac rehabilitation; Wu, C., Li, Y., Chen, J., Hybrid versus traditional cardiac rehabilitation models: a systematic review and meta-analysis (2018) Kardiol Pol, 76, pp. 1717-1724. , PID: 30251248; Imran Hafiz, M., Home-based cardiac rehabilitation alone and hybrid with center-based cardiac rehabilitation in heart failure: a systematic review and meta-analysis (2019) J Am Heart Assoc, 8. , COI: 1:STN:280:DC%2BB3MvptFSjtQ%3D%3D, PID: 31423874, This systematic review and meta-analysis demonstrates that the hybrid model of cardiac rehabilitation may result a greater improvement cardiorespiratory fitness compared to center-based cardiac rehabilitation (usual care},
correspondence_address1={Taub, P.R.; University of California, 9300 Campus Point Drive, La Jolla, United States; email: ptaub@health.ucsd.edu},
publisher={Springer},
issn={15233782},
coden={CCRUA},
pubmed_id={33704611},
language={English},
abbrev_source_title={Curr. Cardiol. Rep.},
document_type={Review},
source={Scopus},
}

@ARTICLE{Aragaki2021263,
author={Aragaki, D. and Luo, J. and Weiner, E. and Zhang, G. and Darvish, B.},
title={Cardiopulmonary Telerehabilitation},
journal={Physical Medicine and Rehabilitation Clinics of North America},
year={2021},
volume={32},
number={2},
pages={263-276},
doi={10.1016/j.pmr.2021.01.004},

note={cited By 1},
url={https://www.scopus.com/inward/record.uri?eid=2-s2.0-85100617624&doi=10.1016%2fj.pmr.2021.01.004&partnerID=40&md5=1ef906f9668b83df41a23779a020243b},
affiliation={Department of Medicine, Division of Physical Medicine and Rehabilitation, David Geffen School of Medicine at UCLA, Los Angeles, CA, United States; Physical Medicine and Rehabilitation Service, VA Greater Los Angeles Healthcare System, 11301 Wilshire Boulevard (117), Los Angeles, CA 90073, United States; Physical Medicine and Rehabilitation Residency (PGY-2), Greater Los Angeles VA Healthcare System, West Los Angeles VA Medical Center, 11301 Wilshire Boulevard (117), Los Angeles, CA 90073, United States; Cardiopulmonary Telehealth Service, Physical Medicine and Rehabilitation Service, Greater Los Angeles Healthcare System, West Los Angeles VA Medical Center, 11301 Wilshire Boulevard (117), Los Angeles, CA 90073, United States},
author_keywords={Cardiac rehabilitation; Home-based rehabilitation; Pulmonary rehabilitation; Telehealth; Telerehabilitation},
keywords={evidence based medicine; health program; heart rehabilitation; high risk population; home rehabilitation; human; patient care; priority journal; process optimization; pulmonary rehabilitation; remote sensing; Review; social network; telehealth; telerehabilitation; virtual reality},
references={Bernocchi, P., Vitacca, M., La Rovere, M.T., Home-based telerehabilitation in older patients with chronic obstructive pulmonary disease and heart failure: a randomised controlled trial (2018) Age Ageing, 47 (1), pp. 82-88; Hawkins, N.M., Virani, S., Ceconi, C., Heart failure and chronic obstructive pulmonary disease: the challenges facing physicians and health services (2013) Eur J Heart Fail, 34, pp. 2795-2803; Piepoli, M.F., Corrà, U., Adamopoulos, S., Secondary prevention in the clinical management of patients with cardiovascular diseases. Core components, standards and outcome measures for referral and delivery: a policy statement from the cardiac rehabilitation section of the European Association for Cardiovascular Prevention & Rehabilitation. Endorsed by the Committee for Practice Guidelines of the European Society of Cardiology (2014) Eur J Prev Cardiol, 21 (6), pp. 664-681; Thomas, R.J., Beatty, A.L., Beckie, T.M., Home-based cardiac rehabilitation: a scientific statement from the American Association of Cardiovascular and Pulmonary Rehabilitation, the American Heart Association, and the American College of Cardiology (2019) J Am Coll Cardiol, 74, pp. 133-153; Moulson, N., Bewick, D., Selway, T., Cardiac rehabilitation during the COVID-19 era: guidance on implementing virtual care (2020) Can J Cardiol, 36 (8), pp. 1317-1321; Scherrenberg, M., Wilhelm, M., Hansen, D., The future is now: a call for action for cardiac telerehabilitation in the COVID-19 pandemic from the secondary prevention and rehabilitation section of the European Association of Preventive Cardiology (2020) Eur J Prev Cardiol, , 2047487320939671; Kumar, K.R., Pina, I.L., Cardiac rehabilitation in older adults: new options (2020) Clin Cardiol, 43 (2), pp. 163-170; Drwal, K.R., Forman, D.E., Wakefield, B.J., Cardiac rehabilitation during COVID-19 pandemic: highlighting the value of home-based programs (2020) Telemed J E Health, 26 (11), pp. 1322-1324; Jacome, C., Marques, A., Oliveira, A., Letter to the editor pulmonary telerehabilitation: an international call for action (2020) Pulmonol, 26 (6), pp. 335-337; Spruit, M.A., Singh, S.J., Garvey, C., An official American Thoracic Society/European Respiratory Society statement: key concepts and advances in pulmonary rehabilitation (2013) Am J Respir Crit Care Med, 188, pp. e13-e64; Rochester, C.I., Vogiatzis, I., Holland, A.E., An official American Thoracic Society/European Respiratory Society policy statement: enhancing implementation, use, and delivery of pulmonary rehabilitation (2015) Am J Respir Crit Care Med, 192, pp. 1373-1386; Nishi, S.P., Zhang, W., Kuo, Y.F.,

Pulmonary rehabilitation utilization in older adults with chronic obstructive pulmonary disease, 2003 to 2012 (2016) J Cardiopulm Rehabil Prev, 36, pp. 375-382; Garvey, C., Novitch, R.S., Porte, P., Editorial healing pulmonary rehabilitation in the United States: a call to action for ATS members (2019) Am J Respir Crit Care Med, 8, pp. 944-946; Vasilopoulou, M., Pappaioannou, A.I., Kaltsakas, G., Home-based maintenance telerehabilitation reduces the risk for acute exacerbations of COPD, hospitalisations, and emergency department visits (2017) Eur Respir J, 49, p. 1602129; Beckie, T.M., Utility of home-based cardiac rehabilitation for older adults (2019) Clin Geriatr Med, 35 (4), pp. 499-516; Anderson, M., Perrin, A., Tech adoption climbs among older adults. In: Pew Research Center Internet & Technology (2017), https://www.pewresearch.org/internet/2017/05/17/technology-use-among-seniors/, Available at: Accessed December 13, 2020; Mureddu, G.F., Ambrosetti, M., Venturini, E., Cardiac rehabilitation activities during the COVID-19 pandemic in Italy. Position Paper of the AICPR (Italian Association of Clinical Cardiology, Prevention and Rehabilitation) (2020) Monaldi Arch Chest Dis, 90 (2); Huang, C., Wang, Y., Li, X., Clinical features of patients infected with 2019 novel coronavirus in Wuhan, China (2020) Lancet, 395, pp. 497-506; Zhai, Z., Li, C., Chen, Y., Prevention and treatment of venous thromboembolism associated with coronavirus disease 2019 infection: a consensus statement before guidelines (2020) Thromb Haemost, 120 (6), pp. 937-948; Johnston, K., Grimmer-Somers, K., Pulmonary rehabilitation: overwhelming evidence but lost in translation? (2010) Physiother Can, 62, pp. 368-373; Thorpe, O., Johnston, K., Kumar, S., Barriers and enablers to physical activity participation in patients with COPD: a systematic review (2012) J Cardiopulm Rehabil Prev, 32, pp. 359-369; Walters, J.A., Cameron-Tucker, H., Courtney-Pratt, H., Supporting health behavior change in chronic obstructive pulmonary disease with telephone health-monitoring: insights from a qualitative study (2012) BMC Fam Pract, 13, p. 55; McLean, S., Nurmatov, U., Liu, J.L., Telehealthcare for chronic obstructive pulmonary disease. Cochrane Review and meta-analysis (2012) Br J Gen Pract, 62 (604), pp. e739-e749; Zanaboni, P., Hoaas, H., Lien, L.A., Long-term exercise maintenance in COPD via telerehabilitation: a two-year pilot study (2016) J Telemed Telecare, 23 (1), pp. 74-82; Lundell, S., Holmner, Å., Rehn, B., Telehealthcare in COPD: a systematic review and meta-analysis on physical outcomes and dyspnea (2015) Respir Med, 109 (1), pp. 11-26; Waschki, B., Kirsten, A., Holz, O., Physical activity is the strongest predictor of all-cause mortality in patients with COPD: a prospective cohort study (2011) Chest, 140, pp. 331-342; Chan, C., Yamabayashi, C., Syed, N., Exercise telemonitoring and telerehabilitation compared with traditional cardiac and pulmonary rehabilitation: a systematic review and meta-analysis (2016) Physiother Can, 68 (3), pp. 242-251; Huang, K., Liu, W., He, D., Telehealth interventions versus center-based cardiac rehabilitation of coronary artery disease: a systematic review and meta-analysis (2015) Eur J Prev Cardiol, 22 (8), pp. 959-971; Anderson, L., Sharp, G.A., Norton, R.J., Home-based versus centre-based cardiac rehabilitation (2017) Cochrane Database Syst Rev, 6 (6), p. CD007130; Batalik, L., Filakova, K., Batalikova, K., Remotely monitored telerehabilitation for cardiac patients: a review of the current situation (2020) World J Clin Cases, 8 (10), pp. 1818-1831; Avila, A., Claes, J., Goetschalckx, K., Home-based rehabilitation with telemonitoring guidance for patients with coronary artery disease (short-term results of the TRiCH Study): randomized controlled trial (2018) J Med Internet Res, 20 (6), p. e225; Brouwers, R.W.M., van Exel, H.J., van Hal, J.M.C., Cardiac telerehabilitation as an alternative to centre-based cardiac rehabilitation (2020) Neth Heart J, 28 (9), pp. 443-451; Maddison, R., Rawstorn, J.C., Stewart, R.A.H., Effects and

costs of real-time cardiac telerehabilitation: randomised controlled non-inferiority trial (2019) Heart, 105 (2), pp. 122-129; Hwang, R., Bruning, J., Morris, N.R., Home-based telerehabilitation is not inferior to a centre-based program in patients with chronic heart failure: a randomised trial (2017) J Physiother, 63 (2), pp. 101-107; Buckingham, S.A., Taylor, R.S., Jolly, K., Home-based versus centre-based cardiac rehabilitation: abridged Cochrane systematic review and meta-analysis (2016) Open Heart, 3 (2), p. e000463; Arthur, H.M., Smith, K.M., Kodis, J., A controlled trial of hospital versus home-based exercise in cardiac patients (2002) Med Sci Sports Exerc, 34, pp. 1544-1550; Frederix, I., Hansen, D., Coninx, K., Effect of comprehensive cardiac telerehabilitation on one-year cardiovascular rehospitalization rate, medical costs and quality of life: a cost-effectiveness analysis (2016) Eur J Prev Cardiol, 23 (7), pp. 674-682; Frederix, I., Solmi, F., Piepoli, M.F., Cardiac telerehabilitation: a novel cost-efficient care delivery strategy that can induce long-term health benefits (2017) Eur J Prev Cardiol, 24 (16), pp. 1708-1717; Kraal, J.J., Van den Akker-Van Marle, M.E., Abu-Hanna, A., Clinical and cost-effectiveness of home-based cardiac rehabilitation compared to conventional, centre-based cardiac rehabilitation: Results of the FIT@Home study (2017) Eur J Prev Cardiol, 24 (12), pp. 1260-1273; Mytinger, M., Nelson, R.K., Zuhl, M., Exercise prescription guidelines for cardiovascular disease patients in the absence of a baseline stress test (2020) J Cardiovasc Dev Dis, 7 (2), p. 15; Suaya, J.A., Stason, W.B., Ades, P.A., Cardiac rehabilitation and survival in older coronary patients (2009) J Am Coll Cardiol, 54, pp. 25-33; Hammill, B.G., Curtis, L.H., Schulman, K.A., Relationship between cardiac rehabilitation and long-term risks of death and myocardial infarction among elderly Medicare beneficiaries (2010) Circulation, 121, pp. 63-70; Gilchrist, S.C., Barac, A., Ades, P.A., Cardio-oncology rehabilitation to manage cardiovascular outcomes in cancer patients and survivors. a scientific statement from the American Heart Association (2019) Circulation, 138, pp. e997-e1012; Nicholls, S.J., Nelson, M., Astley, C., Optimising secondary prevention and cardiac rehabilitation for atherosclerotic cardiovascular disease during the COVID-19 pandemic: a position statement from the Cardiac Society of Australia and New Zealand (CSANZ) (2020) Heart Lung Circ, 29 (7), pp. e99-e104; Vigorito, C., Faggiano, P., Mureddu, G.F., COVID-19 pandemic: what consequences for cardiac rehabilitation? (2020) Monaldi Arch Chest Dis, 90 (1); Sheehy, L.M., Considerations for postacute rehabilitation for survivors of COVID-19 (2020) JMIR Public Health Surveill, 6 (2), p. e19462; CDC Coronavirus Disease 2019 (COVID-19) overview and infection prevention and control priorities in non-US healthcare settings https://www.cdc.gov/coronavirus/2019-ncov/hcp/non-us-settings/overview/index.html#transmission, Available at: Accessed November 29, 2020; O'Neil, S., Thomas, A., Pettit-Mee, R., Exercise prescription techniques in cardiac rehabilitation centers in midwest states (2018) J Clin Physiol, 7, pp. 8-14; Vromen, T., Spee, R., Kraal, J., Exercise training programs in Dutch cardiac rehabilitation centres (2013) Neth Heart J, 21, pp. 138-143; Ambrosetti, M., Abreu, A., Corrà, U., Secondary prevention through comprehensive cardiovascular rehabilitation: from knowledge to implementation. 2020 update. A position paper from the Secondary Prevention and Rehabilitation Section of the European Association of Preventive Cardiology (2020) Eur J Prev Cardiol, , 2047487320913379; Scherr, J., Wolfarth, B., Christle, J.W., Associations between Borg's rating of perceived exertion and physiological measures of exercise intensity (2013) Eur J Appl Physiol, 113, pp. 147-155; Myers, J., Principles of exercise prescription for patients with chronic heart failure (2008) Heart Fail Rev, 13, pp. 61-68; Gibbons, R.J., Balady, G.J., Bricker, J.T., ACC/AHA 2002 guideline

update for exercise testing: summary article. A report of the American College of Cardiology/American Heart Association Task Force on Practice Guidelines (Committee to Update the 1997 Exercise Testing Guidelines) [published correction appears in J Am Coll Cardiol. 2006 Oct 17;48(8):1731] (2002) J Am Coll Cardiol, 40 (8), pp. 1531-1540; Snoek, J.A., Prescott, E.I., van der Velde, A.E., Effectiveness of home-based mobile guided cardiac rehabilitation as alternative strategy for nonparticipation in clinic-based cardiac rehabilitation among elderly patients in Europe: a randomized clinical trial (2020) JAMA Cardiol, p. e205218; Frederix, I., Vanhees, L., Dendale, P., A review of telerehabilitation for cardiac patients (2015) J Telemed Telecare, 21 (1), pp. 45-53; Hwang, R., Bruning, J., Morris, N., A systematic review of the effects of telerehabilitation in patients with cardiopulmonary diseases (2015) J Cardiopulm Rehabil Prev, 35 (6), pp. 380-389; Hermann, M., Pekacka-Egli, A.-M., Witassek, F., Feasibility and efficacy of cardiopulmonary rehabilitation after COVID-19 (2020) Am J Phys Med Rehabil, 99 (10), pp. 865-869},
correspondence_address1={Aragaki, D.; Physical Medicine and Rehabilitation Service, 11301 Wilshire Boulevard (117), United States; email: Dixie.aragaki@va.gov},
publisher={W.B. Saunders},
issn={10479651},
coden={PMRAF},
pubmed_id={33814057},
language={English},
abbrev_source_title={Phys. Med. Rehabil. Clin. North Am.},
document_type={Review},
source={Scopus},
}

@ARTICLE{Zahid2021,
author={Zahid, A. and Poulsen, J.K. and Sharma, R. and Wingreen, S.C.},
title={A systematic review of emerging information technologies for sustainable data-centric health-care},
journal={International Journal of Medical Informatics},
year={2021},
volume={149},
doi={10.1016/j.ijmedinf.2021.104420},
art_number={104420},
note={cited By 0},
url={https://www.scopus.com/inward/record.uri?eid=2-s2.0-85102066719&doi=10.1016%2fj.ijmedinf.2021.104420&partnerID=40&md5=c3a9012ffda190ac9aed2d85b367b740},
affiliation={Department of Accounting and Information Systems, College of Business and Law, University of Canterbury, Christchurch, New Zealand; Help 4 U Limited, Christchurch, New Zealand; College of Technological Innovation, Zayed University, Abu Dhabi, United Arab Emirates},
abstract={Background: Of the Sustainable Development Goals (SDGs), the third presents the opportunity for a predictive universal digital healthcare ecosystem, capable of informing early warning, assisting in risk reduction and guiding management of national and global health risks. However, in reality, the existing technology infrastructure of digital healthcare systems is insufficient, failing to satisfy current and future data needs. Objective: This paper systematically reviews emerging information technologies for data modelling and analytics that have potential to achieve Data-Centric Health-Care (DCHC) for the envisioned objective of sustainable healthcare. The goal of this review is to: 1) identify emerging information technologies with potential for

data modelling and analytics, and 2) explore recent research of these technologies in DCHC. Findings: A total of 1619 relevant papers have been identified and analysed in this review. Of these, 69 were probed deeply. Our analysis found that the extant research focused on elder care, rehabilitation, chronic diseases, and healthcare service delivery. Use-cases of the emerging information technologies included providing assistance, monitoring, self-care and self-management, diagnosis, risk prediction, well-being awareness, personalized healthcare, and qualitative and/or quantitative service enhancement. Limitations identified in the studies included vendor hardware specificity, issues with user interface and usability, inadequate features, interoperability, scalability, and compatibility, unjustifiable costs and insufficient evaluation in terms of validation. Conclusion: Achievement of a predictive universal digital healthcare ecosystem in the current context is a challenge. State-of-the-art technologies demand user centric design, data privacy and protection measures, transparency, interoperability, scalability, and compatibility to achieve the SDG objective of sustainable healthcare by 2030. © 2021 Elsevier B.V.},
author_keywords={Data analytics; Data modelling; Data-centric health-care; Emerging technologies},
keywords={Diagnosis; Ecosystems; Health risks; Information analysis; Interoperability; Privacy by design; Scalability; User interfaces, Emerging information technologies; Healthcare services; Personalized healthcare; Protection measures; Service enhancement; State-of-the-art technology; Technology infrastructure; User-centric designs, Health care},
references={WHO Guideline: Recommendations on Digital Interventions for Health System Strengthening (2019), https://www.who.int/publications-detail/who-guideline-recommendations-on-digital-interventions-for-health-system-strengthening, Retrieved from; Svensson, T., Digital Health: Answer to Challenges of UN SDG 3 | CFI.Co (2019), https://cfi.co/sustainability/2019/11/digital-health-answer-to-challenges-of-un-sdg-3/, November 11 Retrieved February 28, 2020, from; Mavrogiorgou, A., Kiourtis, A., Perakis, K., Pitsios, S., Kyriazis, D., IoT in healthcare: achieving interoperability of High-quality data acquired by IoT medical devices (2019) Sensors, 19 (9), p. 1978; Munir, M., Baumbach, S., Gu, Y., Dengel, A., Ahmed, S., data analytics: industrial perspective & solutions for streaming data (2018) Series in Machine Perception and Artificial Intelligence, 83, pp. 144-168. , M. Last H. Bunke A. Kandel WORLD SCIENTIFIC; Wang, C., Li, F., Wang, L., Zhou, W., Zhu, B., Zhang, X., The impact of population aging on medical expenses: a big data study based on the life table (2018) Biosci. Trends, 11 (6), pp. 619-631; Windmann, S., Maier, A., Niggemann, O., Frey, C., Bernardi, A., Gu, C., Big Data Analysis of Manufacturing Processes (2015), November 19 659, 012055; Khan, K.S., Kunz, R., Kleijnen, J., Antes, G., Five steps to conducting a systematic review (2003) J. R. Soc. Med., 96 (3), pp. 118-121; Okoli, C., A Guide to Conducting a Standalone Systematic Literature Review (2015), p. 37. , Communications of the Association for Information Systems; Okoli, C., The View from Giants’ Shoulders: Developing Theory With Theory-Mining Systematic Literature Reviews (SSRN Scholarly Paper No. ID 2699362) (2015), Social Science Research Network Rochester, NY; Siddaway, A.P., Wood, A.M., Hedges, L.V., How to Do a systematic review: a Best practice Guide for conducting and reporting narrative reviews, meta-analyses, and meta-syntheses (2019) Annu. Rev. Psychol., 70 (1), pp. 747-770; Xiao, Y., Watson, M., Guidance on conducting a systematic literature review (2019) J. Plann. Educat. Res., 39 (1), pp. 93-112; Mehta, N., Pandit, A., Concurrence of big data analytics and healthcare: a systematic review (2018) Int. J. Med.

Inf., 114, pp. 57-65; Webster, J., Watson, R.T., Analyzing the past to prepare for the future: writing a literature review (2002) Analyzing the Past to Prepare for the Future: Writing a Literature Review, (2), pp. xiii-xxiii. , Scopus. Retrieved from Scopus; Wang, J., Zhang, W., Shi, Y., Duan, S., Liu, J., Industrial Big Data Analytics: Challenges, Methodologies, and Applications (2018); Weiss, P.L., Kizony, R., Feintuch, U., Katz, N., Virtual Reality in Neurorehabilitation (2006), February; Choi, S., Jung, K., Noh, S.D., Virtual reality applications in manufacturing industries: past research, present findings, and future directions (2015) Conc. Eng., 23 (1), pp. 40-63; Lee, Y., Lee, C.H., Augmented reality for personalized nanomedicines (2018) Biotechnol. Adv., 36 (1), pp. 335-343; Adenuga, K.I., Adenuga, R.O., Ziraba, A., Mbuh, P.E., Healthcare augmentation: social adoption of augmented reality glasses in medicine (2019) Proceedings of the 2019 8th International Conference on Software and Information Engineering - ICSIE’ 19, Cairo, Egypt: ACM Press, pp. 71-74; Wu, H.-K., Lee, S.W.-Y., Chang, H.-Y., Liang, J.-C., Current status, opportunities and challenges of augmented reality in education (2013) Comput. Educ., 62, pp. 41-49; Rosenberg, L.B., The Use of Virtual Fixtures as Perceptual Overlays to Enhance Operator Performance in Remote Environments (1992), https://apps.dtic.mil/docs/citations/ADA292450, Retrieved from; Steuer, J., Defining virtual reality: dimensions determining telepresence (1992) Communication, 42 (4), pp. 73-93; Codreanu, I.A., Florea, A.M., A Proposed Serious Game Architecture to Self-Management HealthCare for Older Adults (2016), pp. 437-440. , Scopus; Lv, Z., Esteve, C., Chirivella, J., Gagliardo, P., Serious game based personalized healthcare system for dysphonia rehabilitation (2017) Pervasive Mob. Comput., 41, pp. 504-519. , Scopus; Debarba, H.G., De Oliveira, M.E., Ladermann, A., Chague, S., Charbonnier, C., Augmented reality visualization of joint movements for physical examination and rehabilitation (2018) Virt. Real. 3D User Interfaces, pp. 537-538. , Scopus; Debarba, H.G., De Oliveira, M.E., Ladermann, A., Chague, S., Charbonnier, C., Augmented reality visualization of joint movements for rehabilitation and sports medicine (2018) 20th Symposium on Virtual and Augmented Reality, pp. 114-121. , Scopus; Palanica, A., Docktor, M.J., Lee, A., Fossat, Y., Using mobile virtual reality to enhance medical comprehension and satisfaction in patients and their families (2019) Perspect. Med. Ed., 8 (2), pp. 123-127. , Scopus; Fonseca, M., Cardoso, H., Ferreira, N., Loureiro, T., Gomes, I., Quaresma, C., VR4Neuropain: interactive rehabilitation system (2019) 12th International Joint Conference on Biomedical Engineering Systems and Technologies (BIOSTEC), Scopus, pp. 285-290. , Retrieved from Scopus; Bohlen, C.F., Brinkmann, A., Mavers, S., Hellmers, S., Hein, A., Virtual Reality Integrated Multi-Depth-Camera-System for Real-Time Telepresence and Telemanipulation in Caregiving (2020), pp. 294-297. , December 1 IEEE Computer Society; Boyd, N., Hawkins, J., Yang, T., Valcourt, S.A., Augmented reality: telehealth demonstration application (2020) Practice and Experience in Advanced Research Computing, pp. 452-455. , Association for Computing Machinery New York, NY, USA; Burle, A.Q., Lafayette, T.B.G., Fonseca, J.R., Teichrieb, V., Gama, A.E.F.D., Real-time approach for gait analysis using the kinect v2 sensor for clinical assessment purpose (2020) 2020 22nd Symposium on Virtual and Augmented Reality (SVR), pp. 144-153; Kim, J.H., Oh, S.H., Serious virtual reality game for isometric eye training of strabismic patients (2020) 2020 International Conference on Information and Communication Technology Convergence (ICTC), pp. 468-473; Lin, P.-J., Huang, Y.-H., Hung, S., Virtual reality training system for rehabilitation of patients with stroke based on compensatory movement detection (2020) 2020 IEEE 2nd Eurasia Conference on Biomedical Engineering, Healthcare and Sustainability (ECBIOS), pp. 68-72;

Niederriter, B., Rong, A., Aqlan, F., Yang, H., Sensor-based virtual reality for clinical decision support in the assessment of mental disorders (2020) 2020 IEEE Conference on Games (CoG), pp. 666-669; Orciuoli, F., Orciuoli, F.J., Peduto, A., A Mobile clinical DSS based on augmented reality and deep learning for the home cares of patients afflicted by bedsores (2020) Proced. Comput. Sci., 175, pp. 181-188; Popa, D.-L., Mocanu, M.-L., Popa, R.-T., Bărbulescu, L.-F., Bărbulescu, L.N., Ciobîrcă, C., Augmented and mixed reality for collaborative cardiac planning using 3D and 4D medical imaging data (2020) 2020 24th International Conference on System Theory, Control and Computing (ICSTCC), pp. 384-389; Thomos, M., Charisis, V., Hadjidimitriou, S., Iakovakis, D., Bostantzopoulou, S., Hadjileontiadis, L., HoloPrognosis—An AR-based serious exercise game for early stage Parkinson's Disease patients (2020) Universal Access in Human-Computer Interaction. Design Approaches and Supporting Technologies, pp. 417-430. , M. Antona C. Stephanidis Springer International Publishing Cham; Wang, Y.-W., Chen, C.-H., Lin, Y.-C., Balance rehabilitation system for Parkinson's Disease patients based on augmented reality (2020) 2020 IEEE Eurasia Conference on IOT, Communication and Engineering (ECICE), pp. 191-194; Turbyne, C., de Koning, P., Zantvoord, J., Denys, D., Body integrity identity disorder using augmented reality: a symptom reduction study (2021) BMJ Case Reports, 14 (1); Nilsson, N.J., Artificial Intelligence: A New Synthesis (1998), Morgan Kaufmann Publishers Inc San Francisco, CA, USA; Lawrence, D.R., Palacios-González, C., Harris, J., Artificial intelligence: the shylock syndrome (2016) Camb. Q. Healthc. Ethics, 25 (2), pp. 250-261; Liu, Y., Tang, P., The Prospect for the application of the surgical navigation system based on artificial intelligence and augmented reality (2018) International Conference on Artificial Intelligence and Virtual Reality (AIVR), Taichung, Taiwan: IEEE, pp. 244-246; Awad, M., Khanna, R., Machine learning (2015) Efficient Learning Machines: Theories, Concepts, and Applications for Engineers and System Designers, pp. 1-18. , M. Awad R. Khanna Apress Berkeley, CA; Nilsson, N.J., Principles of Artificial Intelligence (1980), Morgan Kaufmann Publishers Inc San Francisco, CA, USA; Russell, S., Norvig, P., Artificial Intelligence: A Modern Approach (2009), 3rd ed. Pearson; Amodei, D., Anubhai, R., Battenberg, E., Case, C., Casper, J., Catanzaro, B., Deep Speech 2: End-to-End Speech Recognition in English and Mandarin (2015), ArXiv:1512.02595 [Cs]. Retrieved from; LeCun, Y., Bengio, Y., Hinton, G., Deep learning (2015) Nature, 521 (7553), pp. 436-444; Sharma, R., Kshetri, N., Digital healthcare: historical development, applications, and future research directions (2020) Int. J. Inf. Manage., 53; De Grey, A.D.N.J., Artificial intelligence and medical research: time to aim Higher? (2016) Rejuvenation Res., 19 (2), pp. 105-106; Handelman, G.S., Kok, H.K., Chandra, R.V., Razavi, A.H., Lee, M.J., Asadi, H., eDoctor: machine learning and the future of medicine (2018) Intern. Med., 284 (6), pp. 603-619; Kantarjian, H., Yu, P.P., Artificial intelligence, Big data, and cancer (2015) Oncology, 1 (5), pp. 573-574; Krittanawong, C., Bomback, A.S., Baber, U., Bangalore, S., Messerli, F.H., Wilson Tang, W.H., Future direction for using artificial intelligence to predict and manage hypertension (2018) Curr. Hypertens. Rep., 20 (9), p. 75; Tseng, J.C.C., Lin, B.-H., Lin, Y.-F., Tseng, V.S., Day, M.-L., Wang, S.-C., An Interactive Healthcare System With Personalized Diet and Exercise Guideline Recommendation (2016), pp. 525-532. , Scopus; Syeda-Mahmood, T.F., Walach, E., Beymer, D., Gilboa-Solomon, F., Moradi, M., Kisilev, P., Medical Sieve: A Cognitive Assistant for Radiologists and Cardiologists (2016), Medical Imaging: Computer-Aided Diagnosis; Alaa, A.M., Moon, K.H., Hsu, W., Van Der Schaar, M., ConfidentCare: a clinical decision support system for personalized breast cancer screening (2016) IEEE Trans. Multimedia, 18 (10), pp. 1942-1955. , Scopus; Daowd, A., Abidi, S.R.,

Abusharekh, A., Raza ABIDI, S.S., A personalized risk stratification platform for population lifetime healthcare (2018) Studies Health Technol. Inform., 247, pp. 920-924. , Scopus; De Silva, D., Ranasinghe, W., Bandaragoda, T., Adikari, A., Mills, N., Iddamalgoda, L., Machine learning to support social media empowered patients in cancer care and cancer treatment decisions (2018) PLoS ONE, 13 (10). , Scopus; Baek, J.-W., Jung, H., Chung, K., Context mining based mental health model for lifecare platform (2019) Medico-Legal Update, 19 (1), pp. 674-679. , Scopus; Chen, J., Zheng, X., A system architecture for smart health services and applications (2015) Lecture Notes Comput. Sci., 9426, pp. 449-456. , Scopus; Hijazi, S., Page, A., Kantarci, B., Soyata, T., Machine learning in cardiac health monitoring and decision support (2016) Computer, 49 (11), pp. 38-48. , Scopus; Castro, D., Coral, W., Rodriguez, C., Cabra, J., Colorado, J., Wearable-based human activity recognition using an IoT approach (2017) J. Sens. Actuator Networks, 6 (4). , Scopus; Alfian, G., Syafrudin, M., Ijaz, M.F., Syaekhoni, M.A., Fitriyani, N.L., Rhee, J., A personalized healthcare monitoring system for diabetic patients by utilizing BLE-based sensors and real-time data processing (2018) Sensors (Switzerland), 18 (7). , Scopus; Öksüz, N., Shcherbatyi, I., Kowatsch, T., Maass, W., A data-analytical system to predict therapy success for obese children (2018) 39th International Conference on Information Systems (ICIS), San Francisco, pp. 1-17. , https://cocoa.ethz.ch/downloads/2018/10/None_Oksuz%20et%20al%202018%20Data-analytical%20System%20Obese%20Children.pdf, Scopus. Retrieved from; Magyar, G., Balsa, J., Cláudio, A.P., Carmo, M.B., Neves, P., Alves, P., Anthropomorphic virtual assistant to support self-care of type 2 diabetes in older people: a perspective on the role of artificial intelligence (2019) 14th International Joint Conference on Computer Vision, Imaging and Computer Graphics Theory and Applications (VISIGRAPP), 1, pp. 323-331. , Scopus. Retrieved from Scopus; Amin, P., Anikireddypally, N.R., Khurana, S., Vadakkemadathil, S., Wu, W., Personalized Health Monitoring Using Predictive Analytics (2019), pp. 271-278. , Scopus; Jayawant, S., Medic: an artificially intelligent system to provide healthcare services to society and medical assistance to doctors (2016) International Conference on Communication and Electronics Systems (ICCES), Presented at the International Conference on Communication and Electronics Systems (ICCES). Scopus; Xue, Q., Chuah, M.C., Deep Learning Based Medical Diagnosis System Using Multiple Data Sources (2018), pp. 699-706. , Scopus; Sahoo, A.K., Pradhan, C., Barik, R.K., Dubey, H., deepReco: deep learning based health recommender system using collaborative filtering (2019) Computation, 7 (2). , Scopus; Lawanot, W., Inoue, M., Yokemura, T., Mongkolnam, P., Nukoolkit, C., Daily stress and mood recognition system using deep learning and fuzzy clustering for promoting better Well-being (2019) Presented at the IEEE International Conference on Consumer Electronics (ICCE), Scopus; Rączkowski, Ł., Możejko, M., Zambonelli, J., Szczurek, E., ARA: accurate, reliable and active histopathological image classification framework with bayesian deep learning (2019) Sci. Rep., 9 (1). , Scopus; Uddin, M.Z., Hassan, M.M., Activity recognition for cognitive assistance using body sensors data and deep convolutional neural network (2019) IEEE Sensors, 19 (19), pp. 8413-8419. , Scopus; Ali, F., El-Sappagh, S., Islam, S.M.R., Kwak, D., Ali, A., Imran, M., Kwak, K.-S., A smart healthcare monitoring system for heart disease prediction based on ensemble deep learning and feature fusion (2020) Inform. Fusion, 63, pp. 208-222. , Scopus; Bargshady, G., Zhou, X., Deo, R.C., Soar, J., Whittaker, F., Wang, H., Enhanced deep learning algorithm development to detect pain intensity from facial expression images (2020) Expert Syst. Appl., 149; Luo, J., Ye, M., Xiao, C., Ma, F., HiTANet: Hierarchical Time-Aware Attention Networks for Risk Prediction on Electronic Health Records (2020), pp. 647-656.

, Scopus; Vidhya, K., Shanmugalakshmi, R., Deep learning based big medical data analytic model for diabetes complication prediction (2020) J. Amb. Intell. Human. Comput., 11 (11), pp. 5691-5702; Yu, K., Xie, X., Predicting Hospital readmission: a joint ensemble-learning model (2020) IEEE J. Biomed. Health. Inf., 24 (2), pp. 447-456. , Scopus; Hossain, M.E., Uddin, S., Khan, A., Network analytics and machine learning for predictive risk modelling of cardiovascular disease in patients with type 2 diabetes (2021) Expert Syst. Appl., p. 164. , Scopus; Motwani, A., Shukla, P.K., Pawar, M., Smart predictive healthcare framework for remote patient monitoring and recommendation using deep learning with novel cost optimization (2021) Information and Communication Technology for Intelligent Systems, pp. 671-682. , T. Senjyu P.N. Mahalle T. Perumal A. Joshi Springer Singapore; Liu, Y., Zhang, L., Yang, Y., Zhou, L., Ren, L., Wang, F., A novel Cloud-based framework for the elderly healthcare services using digital twin (2019) IEEE Access, 7, pp. 49088-49101; Glaessgen, E., Stargel, D., The digital twin paradigm for future NASA and U.S. Air force vehicles (2012) 53rd AIAA/ASME/ASCE/AHS/ASC Structures, Structural Dynamics and Materials Conference, Presented at the 53rd AIAA/ASME/ASCE/AHS/ASC Structures, Structural Dynamics and Materials Conference, Honolulu, Hawaii; Grieves, M., Vickers, J., Digital twin: mitigating unpredictable, undesirable emergent behavior in complex systems (2017) Transdisciplinary Perspectives on Complex Systems: New Findings and Approaches, pp. 85-113. , F.-J. Kahlen S. Flumerfelt A. Alves Springer International Publishing Cham; Martinez-Velazquez, R., Gamez, R., Saddik, A.E., Cardio twin: a digital twin of the human heart running on the edge (2019) IEEE International Symposium on Medical Measurements and Applications (MeMeA), Istanbul. Scopus, pp. 1-6; Rivera, L.F., Jiménez, M., Angara, P., Villegas, N.M., Tamura, G., Müller, H.A., Towards continuous monitoring in personalized healthcare through digital twins (2019) Proceedings of the 29th Annual International Conference on Computer Science and Software Engineering, USA: IBM Corp, pp. 329-335; Ahmadi-Assalemi, G., Al-Khateeb, H., Maple, C., Epiphaniou, G., Alhaboby, Z.A., Alkaabi, S., Alhaboby, D., Digital twins for precision healthcare (2020) Cyber Defence in the Age of AI, Smart Societies and Augmented Humanity, pp. 133-158. , H. Jahankhani S. Kendzierskyj N. Chelvachandran J. Ibarra Springer International Publishing Cham; Angulo, C., Gonzalez-Abril, L., Raya, C., Ortega, J.A., A proposal to evolving towards digital twins in healthcare (2020) Bioinformatics and Biomedical Engineering, pp. 418-426. , I. Rojas O. Valenzuela F. Rojas L.J. Herrera F. Ortuño Springer International Publishing Cham; Gochhait, D.S., Bende, A., Leveraging digital twin technology in the healthcare industry – a machine learning based approach (2020) Europ. J. Mol. Clin. Med., 7 (6), pp. 2547-2557; Elayan, H., Aloqaily, M., Guizani, M., Digital twin for intelligent context-aware IoT healthcare systems (2021) IEEE Internet Things J., , 1–1; He, K., Ge, D., He, M., Big data analytics for genomic medicine (2017) Mol. Sci., 18 (2), p. 412; Alonso, S.G., de la, T., Díez, I., Rodrigues, J.J.P.C., Hamrioui, S., López-Coronado, M., A systematic review of techniques and sources of Big data in the healthcare Sector (2017) Med. Syst., 41 (11), p. 183; Ketchersid, T., Big data in nephrology: friend or foe? (2013) Blood Purif., 36 (3-4), pp. 160-164; Olivera, P., Danese, S., Jay, N., Natoli, G., Peyrin-Biroulet, L., Big data in IBD: a look into the future (2019) Nat. Rev. Gastroenterol. Hepatol., 16 (5), pp. 312-321; Baro, E., Degoul, S., Beuscart, R., Chazard, E., Toward a Literature-Driven Definition of Big Data in Healthcare [Research Article] (2015); Huang, Q., Jing, S., Yi, J., Zhen, W., Innovative Testing and Measurement Solutions for Smart Grid (2016), 1st ed. Wiley-IEEE Press Singapore; Laney, D., 3D Data Management: Controlling Data Volume, Velocity and Variety (2001),

https://blogs.gartner.com/doug-laney/files/2012/01/ad949-3D-Data-Management-Controlling-Data-Volume-Velocity-and-Variety.pdf, June 2 Retrieved August 22, 2019, from; Koutkias, V., Thiessard, F., Section Editors for the IMIA Yearbook Special Section, Big data - smart health strategies: findings from the yearbook 2014 Special theme (2014) Yearbook Med. Inform., 23 (1), pp. 48-51; Wang, L., Alexander, C.A., Big data analytics in healthcare systems (2019) Math. Eng. Manage. Sci., 4 (1), pp. 17-26; Khan, N., Yaqoob, I., Hashem, I.A.T., Inayat, Z., Ali, W.K.M., Alam, M., Big data: survey, technologies, opportunities, and challenges (2014) Sci. World, 2014; Klonoff, D.C., Twelve modern digital technologies that are transforming decision making for diabetes and all areas of health care (2013) Diabetes Sci. Technol., 7 (2), pp. 291-295; Fang, R., Pouyanfar, S., Yang, Y., Chen, S.-C., Iyengar, S.S., Computational health informatics in the Big data age: a survey (2016) ACM Comput. Surv. (CSUR), 49 (1), pp. 1-36; Sakr, S., Elgammal, A., Towards a comprehensive data analytics framework for smart healthcare services (2016) Big Data Res., 4, pp. 44-58; Mahmud, S., Iqbal, R., Doctor, F., Cloud enabled data analytics and visualization framework for health-shocks prediction (2016) Future Generat. Comp. Syst., 65, pp. 169-181; Hossain, M.S., Muhammad, G., Healthcare Big data voice pathology assessment framework (2016) IEEE Access, 4, pp. 7806-7815; Zhang, Y., Qiu, M., Tsai, C.-W., Hassan, M.M., Alamri, A., Health-CPS: healthcare cyber-physical system assisted by Cloud and Big data (2017) IEEE Systems, 11 (1), pp. 88-95; Hossain, M.S., Rahman, M.A., Muhammad, G., Towards energy-aware cloud-oriented cyber-physical therapy system (2017) Future Generat. Comp. Syst.; Pramanik, M.I., Lau, R.Y.K., Demirkan, H., Azad, M.A.K., Smart health: Big data enabled health paradigm within smart cities (2017) Expert Syst. Appl., 87, pp. 370-383; Shaban-Nejad, A., Lavigne, M., Okhmatovskaia, A., Buckeridge, D.L., PopHR: a knowledge-based platform to support integration, analysis, and visualization of population health data (2017) New York Acad. Sci., 1387 (1), pp. 44-53; Sahoo, P.K., Mohapatra, S.K., Wu, S.-L., SLA based healthcare big data analysis and computing in cloud network (2018) J. Parallel Distrib. Comput., 119, pp. 121-135; Jain, P., Agarwal, A., Behara, R., Baechle, C., HPCC based framework for COPD readmission risk analysis (2019) Big Data, 6 (1). , Scopus; Lopez-Perez, L., Hernandez, L., Ottaviano, M., Martinelli, E., Poli, T., Licitra, L., BD2Decide: Big data and models for personalized head and neck cancer decision support (2019) IEEE Symposium on Computer-Based Medical Systems, 2019-June, pp. 67-68. , Scopus; Akbar, W., Wu, W.-P., Faheem, M., Saleem, S., Javed, A., Saleem, M.A., Predictive analytics model based on multiclass classification for asthma severity by using random Forest algorithm (2020) 2020 International Conference on Electrical, Communication, and Computer Engineering (ICECCE), pp. 1-4; Jing, S., Hou, K., Yan, J., Ho, Z.-P., An application of big data mining and fuzzy prediction of total knee replacement surgery patients attributes-fuzzy TKR patients attributes prediction (2020) J. Nonlin. Convex Analysis, 21 (8), pp. 1689-1702. , Scopus. Retrieved from Scopus; Satti, F.A., Ali, T., Hussain, J., Khan, W.A., Khattak, A.M., Lee, S., Ubiquitous health profile (UHPr): a big data curation platform for supporting health data interoperability (2020) Computing, 102 (11), pp. 2409-2444; Sivaparthipan, C.B., Muthu, B.A., Manogaran, G., Maram, B., Sundarasekar, R., Krishnamoorthy, S., Innovative and efficient method of robotics for helping the parkinson's disease patient using IoT in big data analytics (2020) Transact. Emerg. Telecommun. Technol., 31 (12); Ali, F., El-Sappagh, S., Islam, S.M.R., Ali, A., Attique, M., Imran, M., Kwak, K.-S., An intelligent healthcare monitoring framework using wearable sensors and social networking data (2021) Future Generat. Comp. Syst., 114, pp. 23-43; Sun, K., Yang, D., Human health big data evaluation based on FPGA

processor and big data decision algorithm (2021) Microprocess. Microsyst., 81; Blas, H.S.S., Mendes, A.S., Encinas, F.G., Silva, L.A., González, G.V., A multi-agent system for data fusion techniques applied to the internet of things enabling physical rehabilitation monitoring (2021) Appl. Sci., 11 (1), p. 331; Alaraj, A., Luciano, C.J., Bailey, D.P., Elsenousi, A., Roitberg, B.Z., Bernardo, A., Virtual reality cerebral aneurysm clipping simulation with real-time haptic feedback (2015) Neurosurgery, 11, pp. 52-58; Moisseiev, E., Mannis, M.J., Evaluation of a portable artificial vision device among patients with Low vision (2016) JAMA Ophthalmol., 134 (7), pp. 748-752; Moshtael, H., Aslam, T., Underwood, I., Dhillon, B., High tech aids Low vision: a review of image processing for the visually impaired (2015) Translat. Vision Sci. Technol., 4 (4). , 6–6; Loria, K., Therapists Have Created a Virtual “heroin Cave” in an Attempt to Help Addicts (2016), https://www.businessinsider.com/university-of-houston-heroin-cave-virtual-reality-2016-3, March 4 Retrieved October 29, 2019, from Business Insider website; Golab, M.R., Breedon, P.J., Vloeberghs, M., A wearable headset for monitoring electromyography responses within spinal surgery (2016) Eur. Spine J., 25 (10), pp. 3214-3219; Kim, A.H., Kendrick, D.E., Moorehead, P.A., Nagavalli, A., Miller, C.P., Liu, N.T., Endovascular aneurysm repair simulation can lead to decreased fluoroscopy time and accurately delineate the proximal seal zone (2016) Vasc. Surgery, 64 (1), pp. 251-258; Saratzis, A., Calderbank, T., Sidloff, D., Bown, M.J., Davies, R.S., Role of simulation in endovascular aneurysm repair (EVAR) training: a preliminary study (2017) Vasc. Endovasc. Surgery, 53 (2), pp. 193-198; Velu, J.F., Groot Jebbink, E., de Vries, J.-P.P.M., Slump, C.H., Geelkerken, R.H., Validation of the simbionix PROcedure rehearsal studio sizing module: a comparison of software for endovascular aneurysm repair sizing and planning (2017) Vascular, 25 (1), pp. 80-85; Lee, C.K., Kim, Y., Lee, N., Kim, B., Kim, D., Yi, S., Feasibility study of utilization of action camera, GoPro hero 4, google glass, and panasonic HX-A100 in spine surgery (2017) Spine, 42 (4), pp. 275-280; Yoon, J.W., Chen, R.E., Han, P.K., Si, P., Freeman, W.D., Pirris, S.M., Technical feasibility and safety of an intraoperative head-up display device during spine instrumentation (2017) Med. Robotics Comput. Ass. Surg. (MRCAS), 13 (3); Sheth, A., Jaimini, U., Yip, H.Y., How will the internet of things enable augmented personalized health? (2018) IEEE Intell. Syst., 33 (1), pp. 89-97; Felipe Heckler, W., Varella de Carvalho, J., Cinara da Costa, C., Luis Victória Barbosa, J., A model to patient abandonment prediction in the pulmonary rehabilitation (2020) XVI Brazilian Symposium on Information Systems, pp. 1-8. , Association for Computing Machinery New York, NY, USA; Mesko, B., The role of artificial intelligence in precision medicine (2017) Expert Rev. Prec. Med. Drug Develop., 2 (5), pp. 239-241; Qu, Z., Lau, C.W., Nguyen, Q.V., Zhou, Y., Catchpoole, D.R., Visual analytics of genomic and cancer data: a systematic review (2019) Cancer Inf., 18; Sharma, R., Zhang, C., Wingreen, S.C., Kshetri, N., Zahid, A., Design of blockchain-based precision health-care using soft systems methodology (2019) Indus. Manage. Data Syst., 120 (3), pp. 608-632; Big Data and Analytics in the Automotive Industry | Automotive Analytics Thought Piece (2015), https://www2.deloitte.com/content/dam/Deloitte/uk/Documents/manufacturing/deloitte-uk-automotive-analytics.pdf, Deloitte LLP Retrieved from; Goal 3: Ensure Healthy Lives and Promote Well-Being for All at All Ages (2015), https://www.un.org/sustainabledevelopment/health/, Retrieved February 28, 2020, from United Nations Sustainable Development website; THE AGE OF ANALYTICS, COMPETING IN A DATA-DRIVEN WORLD (2016), https://www.mckinsey.com/~/media/McKinsey/Business%20Functions/McKinsey%20Analytics/Our%20Insights/The%20age%20of%20analytics%20Competing%20in%20a%20data%20dr

iven%20world/MGI-The-Age-of-Analytics-Full-report.ashx, Retrieved from; The Sustainable Development Goal Report (2019), https://unstats.un.org/sdgs/report/2019/, Retrieved from},
correspondence_address1={Zahid, A.; Department of Accounting and Information Systems, New Zealand; email: arnob07@yahoo.com},
publisher={Elsevier Ireland Ltd},
issn={13865056},
coden={IJMIF},
language={English},
abbrev_source_title={Int. J. Med. Informatics},
document_type={Article},
source={Scopus},
}

@ARTICLE{Aggarwal20211,
author={Aggarwal, M. and Ornish, D. and Josephson, R. and Brown, T.M. and Ostfeld, R.J. and Gordon, N. and Madan, S. and Allen, K. and Khetan, A. and Mahmoud, A. and Freeman, A.M. and Aspry, K.},
title={Closing Gaps in Lifestyle Adherence for Secondary Prevention of Coronary Heart Disease},
journal={American Journal of Cardiology},
year={2021},
volume={145},
pages={1-11},
doi={10.1016/j.amjcard.2021.01.005},
note={cited By 0},
url={https://www.scopus.com/inward/record.uri?eid=2-s2.0-85101160913&doi=10.1016%2fj.amjcard.2021.01.005&partnerID=40&md5=11a8cfea101a95597f1c0aa84bf86ff4},
affiliation={Division of Cardiology, University of Florida, Gainesville, FL, United States; Preventive Medicine Research Institute, Sausalito, California, Department of Medicine, University of California, San Francisco, San Francisco, CA, United States; Division of Cardiology, Cleveland Medical Center and Case Western Reserve University, Cleveland, OH, United States; Division of Cardiovascular Disease, University of Alabama at Birmingham, Birmingham, AL, United States; Division of Cardiology, Montefiore Health System, Bronx, NY, United States; Medical and Science Office, INTERVENT International, Savannah, GA and Centre for Exercise Science and Sports Medicine, School of Therapeutic Sciences, Faculty of Health Sciences, University of the Witwatersrand, Johannesburg, South Africa; Division of Cardiology, Montefiore Health System, Bronx, NY, United States; Geisel School of Medicine at Dartmouth, Hanover, NH, United States; Division of Cardiology, Cleveland Medical Center and Case Western Reserve University, Cleveland, OH, United States; Division of Cardiology, University of Florida, Gainesville, FL, United States; Division of Cardiology, Department of Medicine, National Jewish Health, Denver, CO, United States; Lifespan Cardiovascular Institute, and Department of Medicine, Division of Cardiology, Brown University, Alpert Medical School, Providence, RI, United States},
abstract={The secondary prevention (SP) of coronary heart disease (CHD) has become a major public health and economic burden worldwide. In the United States, the prevalence of CHD has risen to 18 million, the incidence of recurrent myocardial infarctions (MI) remains high, and related healthcare costs are projected to double by 2035. In the last decade, practice guidelines and performance measures for the SP of CHD have increasingly emphasized evidence-based lifestyle (LS) interventions, including healthy dietary

patterns, regular exercise, smoking cessation, weight management, depression screening, and enrollment in cardiac rehabilitation. However, data show large gaps in adherence to healthy LS behaviors and low rates of enrollment in cardiac rehabilitation in patients with established CHD. These gaps may be related, since behavior change interventions have not been well integrated into traditional ambulatory care models in the United States. The chronic care model, an evidence-based practice framework that incorporates clinical decision support, self-management support, team-care delivery and other strategies for delivering chronic care is well suited for both chronic CHD management and prevention interventions, including those related to behavior change. This article reviews the evidence base for LS interventions for the SP of CHD, discusses current gaps in adherence, and presents strategies for closing these gaps via evidence-based and emerging interventions that are conceptually aligned with the elements of the chronic care model. © 2021},
keywords={Article; clinical decision support system; diet therapy; dietary compliance; evidence based practice; health care system; heart rehabilitation; human; intensive care; ischemic heart disease; lifestyle modification; long term care; medical information system; patient compliance; physical activity; priority journal; secondary prevention; self care; smoking cessation; stress management},
references={Delling, F.N., Djousse, L., Elkind, M.S.V., Ferguson, J.F., Fornage, M., Khan, S.S., Kissela, B.M., Heard, D.G., Heart disease and stroke statistics. A report from the American Heart Association. Circulation 2020;141(9):e139–598; Sandesara, P.B., Lambert, C.T., Gordon, N.F., Fletcher, G.F., Franklin, B.A., Wenger, N.K., Sperling, L., Cardiac rehabilitation and risk reduction. Time to rebrand and reinvigorate (2015) J Am Coll Cardiol, 65, pp. 389-395; Anderson, L., Thompson, D.R., Oldridge, N., Zwisler, A.D., Rees, K., Martin, N., Taylor, R.S., Exercise-based cardiac rehabilitation for coronary heart disease (2016) Cochrane Database Syst Rev, , 001810.001002/14651858.CD14001800.pub 14651853; Critchley, J., Capewell, S., Smoking cessation for the secondary prevention of coronary heart disease (2004) Cochrane Database Syst Rev, , 003010.001002/14651858.CD14003041.pub14651852; de Lorgeril, M., Salen, P., Martin, J.L., Monjaud, I., Delaye, J., Mamelle, N., Mediterranean diet, traditional risk factors, and the rate of cardiovascular complications after myocardial infarction: final report of the Lyon Diet Heart Study (1999) Circulation, 99, pp. 779-785; Li, S., Chiuve, S.E., Flint, A., Pai, J.K., Forman, J.P., Hu, F.B., Willett, W.C., Rimm, E.B., Better diet quality and decreased mortality among myocardial infarction survivors (2013) JAMA Intern Med, 173, pp. 1808-1818; Baigent, C., Blackwell, L., Collins, R., Emberson, J., Godwin, J., Peto, R., Buring, J., Zanchetti, A., Aspirin in the primary and secondary prevention of vascular disease: collaborative meta-analysis of individual participant data from randomised trials (2009) Lancet, 373, pp. 1849-1860; Fulcher, J., O'Connell, R., Voysey, M., Emberson, J., Blackwell, L., Mihaylova, B., Simes, J., Keech., A., Efficacy and safety of LDL-lowering therapy among men and women: meta-analysis of individual data from 174,000 participants in 27 randomised trials (2015) Lancet, 385, pp. 1397-1405; Freemantle, N., Cleland, J., Young, P., Mason, J., Harrison, J., Beta Blockade after myocardial infarction: systematic review and meta regression analysis (1999) BMJ, 318, pp. 1730-1737; Stergiopaulos, K., Boden, W.E., Hartigan, P., Percutaneous coronary intervention outcomes in patients with stable obstructive coronary disease and myocardial ischemia (2014) JAMA Intern Med, 174, pp. 232-240; Ockene Judith, K., Schneider Kristin, L., Lemon Stephenie, C., Ockene Ira, S., Can we improve adherence to preventive therapies for cardiovascular health? (2011) Circulation, 124, pp. 1276-1282; Glasgow, R.E., Orleans, C.T.,

Wagner, E.H., Does the chronic care model serve also as a template for improving prevention? (2001) Milbank Q, 79, pp. 579-612. , iv-v; Lionetti, V., Tuana, B.S., Casieri, V., Parikh, M., Pierce, G.N., Importance of functional food compounds on cardioprotection through action on the epigenome (2019) Eur Heart J, 40, pp. 575-582; Sacks, F.M., Lichtenstein, A.H., Wu, J.H.Y., Appel, L.J., Creager, M.A., Kris-Etherton, P.M., Miller, M., Van Horn, L.V., Dietary fats and cardiovascular disease: A presidential advisory from the American Heart Association (2017) Circulation, 136, pp. e1-e23; Van Horn, L., Carson, J.A., Appel, L.J., Burke, L.E., Economos, C., Karmally, W., Lancaster, K., Kris-Etherton, P., Recommended dietary pattern to achieve adherence to the American Heart Association/American College of Cardiology (AHA/ACC) guidelines:A scientific statement from the American Heart Association (2016) Circulation, 134, pp. e505-e529; Ornish, D., Scherwitz, L.W., Billings, J.H., Brown, S.E., Gould, L., Merritt, T.A., Sparler, S., Brand, R.J., Intensive lifestyle changes for reversal of coronary heart disease (1998) JAMA, 280, pp. 2001-2007; Li, S., Flint, A., Pai, J.K., Forman, J.P., Hu, F.B., Willett, W.C., Rexrode, K.M., Rimm, E.B., Low carbohydrate diet from plant or animal sources and mortality among myocardial infarction survivors (2014) J Am Heart Assoc, 3; Ma, Y., Li, W., Olendzki, B.C., Pagoto, S.L., Merriam, P.A., Chiriboga, D.E., Griffith, J.A., Ockene, I.S., Dietary quality 1 year after a diagnosis of coronary heart disease (2008) J Am Diet Assoc, 108, pp. 240-246; Vadiveloo, M., Lichtenstein, A.H., Anderson, C., Aspry, K.E., Foraker, R., Griggs, S., Hayman, L.L., Thorndike, A.N., Rapid diet assessment screening tools for cardiovascular disease risk reduction across healthcare settings: A scientific statement from the American Heart Association (2020) Circ Cardiovasc Quality Outcomes, 13 (9); Beydoun, M.A., Wang, Y., Do nutrition knowledge and beliefs modify the association of socio-economic factors and diet quality among US adults? (2008) Prev Med, 46, pp. 145-153; Noar, S.M., Benac, C.N., Harris, M.S., Does tailoring matter? Meta-analytic review of tailored print health behavior change interventions (2007) Psychol Bull, 133, pp. 673-693; McCarroll, R., Effectiveness of mobile health interventions for promoting healthy eating in adults (2017) Prev Med, 105, pp. 156-168. , 110.1016/j.ypmed.2017.1008.1022; Sikand, G., Cole, R.E., Handu, D., deWaal, D., Christaldi, J., Johnson, E., Arpino, L.M., Ekvall, S.M., Clinical and cost benefits of medical nutrition therapy by registered dietitian nutritionists for management of dyslipidemia: a systematic review and meta-analysis (2018) J Clin Lipidol, 12, pp. 1113-1122; Aggarwal, M., Grady, A., Desai, D., Hartog, K., Correa, L., Ostfeld, R.J., Freeman, A.M., Barnard, N.D., Successful implementation of healthful nutrition initiatives into hospitals (2020) Am J Med, 133, pp. 19-25; Martin, S.L., Connelly, N., Parsons, C., Blackstone, K., Simply delivered meals: a tale of collaboration (2018) Am J Manag Care, 24, pp. 301-304; Ross, R., Blair, S.N., Arena, R., Church, T.S., Despres, J.P., Franklink, A.B., Haskell, W.L., Wisløff, U., Importance of assessing cardiorespiratory fitness in clinical practice: A case for fitness as a clinical vital sign: a scientific statement from the American Heart Association (2016) Circulation, 134, pp. e653-e699; Stewart, R.A.H., Held, C., Hadziosmanovic, N., Armstrong, P.W., Cannon, C.P., Granger, C.B., Hagstrom, E., White, H.D., for the STABILITY Investigators. Physical activity and mortality in patients with stable coronary heart disease (2017) J Am Coll Cardiol, 70, pp. 1689-1700; Arnson, Y., Rozanski, A., Gransar, H., Hayes, S.W., Friedman, J.D., Thomson, L.E.J., Berman, D.S., Impact of exercise on the relationship between CAC scores and all-cause mortality (2017) JACC Cardiovasc Imaging, 10, pp. 1461-1468; Janssen, V., De Gucht, V., Dusseldorp, E., Maes, S., Lifestyle modification programmes for patients with coronary heart disease: a systematic review and meta-analysis of randomized

controlled trials (2013) Eur J Prev Cardiol, 20, pp. 620-640; Bock, B.C., Carmona-Barros, R.E., Esler, J.L., Tilkemeier, P.L., Program participation and physical activity maintenance after cardiac rehabilitation (2003) Behav Modif, 27, pp. 37-53; Golightly, Y.M., Allen, K.D., Ambrose, K.R., Stiller, J.L., Evenson, K.R., Voisin, C., Hootman, J.M., Callahan, L.F., Physical activity as a vital sign: a systematic review (2017) Prev Chronic Dis, 14, p. E123. , 110.5888/pcd5814.170030; Effects of physical activity counseling in primary care: the Activity Counseling Trial: a randomized controlled trial (2001) JAMA, 286, pp. 677-687; Chow, C.K., Redfern, J., Hillis, G.S., Thakkar, J., Santo, K., Hackett, M.L., Jan, S., Thiagalingam, A., Effect of lifestyle-focused text messaging on risk factor modification in patients with coronary heart disease: a randomized clinical trial (2015) JAMA, 314, pp. 1255-1263; Sullivan, A.N., Lachman, M.E., Behavior change with fitness technology in sedentary adults: a review of the evidence for increasing physical activity (2016) Front Public Health, 4, p. 289. , 210.3389/fpubh.2016.00289; Moore, S.M., Charvat, J.M., Gordon, N.H., Roberts, B.L., Pashkow, F., Ribisl, P., Rocco, M., Effects of a CHANGE intervention to increase exercise maintenance following cardiac events (2006) Ann Behav Med, 31, p. 53; An EPIC Based BPA to Enhance Quit Line Referral and Use (2017), ClinicalTrials.gov: U.S. National Library of Medicine; Stead, L.F., Buitrago, D., Preciado, N., Sanchez, G., Hartmann-Boyce, J., Lancaster, T., doi.org/000110.001002/14651858.CD14000165.pub14651853, Physician advice for smoking cessation. Cochrane Database Syst Rev 2013:Cd000165; Matkin, W., Ordóñez-Mena, J.M., Hartmann-Boyce, J., doi.org/10.1002/14651858.CD14002850.pub14651854, Telephone counselling for smoking cessation. Cochrane Database Syst Rev 2019; Whittaker, R., McRobbie, H., Bullen, C., Rodgers, A., Gu, Y., Dobson, R., Mobile phone text messaging and app-based interventions for smoking cessation (2019) Cochrane Database Syst Rev, 10. , 006610.001002/14651858.CD14006611.pub14651855; Levine, G.N., Lange, R.A., Noel, C., Bairey-Merz, C.N., Davidson, R.J., Jamerson, K., Mehta, P.K., Smith, S.C., Meditation and cardiovascular risk reduction: a scientific statement from the AHA (2017) J Am Heart Assoc, 6; Scott-Sheldon, L., Gathright, E.C., Donahue, M.L., Balletto, B., Feulner, M.M., DeCosta, J., Cruess, D.G., Salmoirago-Blotcher, E., Mindfulness-based Interventions for adults with cardiovascular disease: a systematic review and meta-analysis (2020) Ann Behav Med, 54, pp. 67-73; Kerst, A., Zielasek, J., Gaebel, W., Smartphone applications for depression: A systematic literature review and a survey of health care professionals' attitudes towards their use in clinical practice (2019) Eur Arch Psychiatry Clin Neurosci, 270, p. 139; Gould, L.K., Ornish, D.O., Scherwitz, L., Brown, S., Patterson, E., Hess, M.J., Mullani, N., Billings, J., Changes in myocardial perfusion abnormalities by positron emission tomography after long term intense risk factor modification (1995) JAMA, 274, pp. 894-901; Ades, P.A., Keteyian, S.J., Wright, J.S., Hamm, L.F., Lui, K., Newlin, K., Shepard, D.S., Thomas, R.J., Increasing cardiac rehabilitation participation from 20% to 70%: A road map from the Million Hearts Cardiac Rehabilitation collaborative (2017) Mayo Clin Proc, 92, pp. 234-242; Anderson, L., Sharp, G.A., Norton, R.J., Dalal, H., Dean, S.G., Jolly, K., Cowie, A., Taylor, R.S., Home-based versus centre-based cardiac rehabilitation Cochrane (2010) Database Syst Rev, , 007110.001002/14651858.CD14007130.pub14651852; Forman, D.E., LaFond, K., Panch, T., Allsup, K., Manning, K., Sattelmair, J., Utility and efficacy of a smartphone application to enhance the learning and behavior goals of traditional cardiac rehabilitation: a feasibility study (2014) J Cardiopulm Rehabil Prev, 34, pp. 327-334; Maddison, R., Rawstorn, J.C., Stewart, R.A.H., Benatar, J., Whittaker, R., Rolleston, A., Jiang, Y., Gant, N., Effects and

costs of real-time cardiac telerehabilitation: randomised controlled
non-inferiority trial (2019) Heart, 105, pp. 122-129; Maddison, R., Rawstorn,
J.C., Shariful Islam, S.M., Ball, K., Tighe, S., Gant, N., Whittaker, R.M.,
Chow, C.K., mHealth interventions for exercise and risk factor modification in
cardiovascular disease (2019) Exerc Sport Sci Rev, 47, pp. 86-90; Afshin, A.,
Babalola, D., McLean, M., Yu, Z., Ma, W., Chen, C.Y., Arabi, M., Mozaffarian,
D., Information technology and lifestyle: a systematic evaluation of internet
and mobile interventions for improving diet, physical activity, obesity,
tobacco, and alcohol use (2016) J Am Heart Assoc, 5; Gordon, N.F., Salmon,
R.D., Sperling, L.S., Wright, B.S., Faircloth, G.C., Gordon, T.L., Berk, M.R.,
Franklin, B.A., Multicenter study of temporal trends in the achievement of
atherosclerotic cardiovascular disease risk factor goals during cardiac
rehabilitation (2017) J Cardiopulm Rehabil Prev, 37, pp. 11-21},
correspondence_address1={Aspry, K.; Lifespan Cardiovascular Institute, United
States; email: kaspry@lifespan.org},
publisher={Elsevier Inc.},
issn={00029149},
coden={AJCDA},
pubmed_id={33454343},
language={English},
abbrev_source_title={Am. J. Cardiol.},
document_type={Article},
source={Scopus},
}

@ARTICLE{Blair2021,
author={Blair, C.K. and Harding, E. and Wiggins, C. and Kang, H. and Schwartz,
M. and Tarnower, A. and Du, R. and Kinney, A.Y.},
title={A home-based mobile health intervention to replace sedentary time with
light physical activity in older cancer survivors: Randomized controlled pilot
trial},
journal={JMIR Cancer},
year={2021},
volume={7},
number={2},
doi={10.2196/18819},
art_number={e18819},
note={cited By 0},
url={https://www.scopus.com/inward/record.uri?eid=2-s2.0-85104106311&doi=10.219
6%2f18819&partnerID=40&md5=24ff17b5c8af6b5915f02bd111e7a031},
affiliation={Department of Internal Medicine, University of New Mexico,
Albuquerque, NM, United States; University of New Mexico Comprehensive Cancer
Center, Albuquerque, NM, United States; School of Public Health, Rutgers
University, Piscataway, NJ, United States; Rutgers Cancer Institute of New
Jersey, Rutgers University, New Brunswick, NJ, United States},
abstract={Background: Older cancer survivors are at risk of the development or
worsening of both age- and treatment-related morbidity. Sedentary behavior
increases the risk of or exacerbates these chronic conditions. Light-intensity
physical activity (LPA) is more common in older adults and is associated with
better health and well-being. Thus, replacing sedentary time with LPA may
provide a more successful strategy to reduce sedentary time and increase
physical activity. Objective: This study primarily aims to evaluate the
feasibility, acceptability, and preliminary efficacy of a home-based mobile
health (mHealth) intervention to interrupt and replace sedentary time with LPA

(standing and stepping). The secondary objective of this study is to examine changes in objective measures of physical activity, physical performance, and self-reported quality of life. Methods: Overall, 54 cancer survivors (aged 60-84 years) were randomized in a 1:1:1 allocation to the tech support intervention group, tech support plus health coaching intervention group, or waitlist control group. Intervention participants received a Jawbone UP2 activity monitor for use with their smartphone app for 13 weeks. Tech support and health coaching were provided via 5 telephone calls during the 13-week intervention. Sedentary behavior and physical activity were objectively measured using an activPAL monitor for 7 days before and after the intervention. Results: Participants included survivors of breast cancer (21/54, 39%), prostate cancer (16/54, 30%), and a variety of other cancer types; a mean of 4.4 years (SD 1.6) had passed since their cancer diagnosis. Participants, on average, were 70 years old (SD 4.8), 55% (30/54) female, 24% (13/54) Hispanic, and 81% (44/54) overweight or obese. Malfunction of the Jawbone trackers occurred in one-third of the intervention group, resulting in enrollment stopping at 54 rather than the initial goal of 60 participants. Despite these technical issues, the retention in the intervention was high (47/54, 87%). Adherence was high for wearing the tracker (29/29, 100%) and checking the app daily (28/29, 96%) but low for specific aspects related to the sedentary features of the tracker and app (21%-25%). The acceptability of the intervention was moderately high (81%). There were no significant between-group differences in total sedentary time, number of breaks, or number of prolonged sedentary bouts. There were no significant between-group differences in physical activity. The only significant within-group change occurred within the health coaching group, which increased by 1675 daily steps (95% CI 444-2906; P=.009). This increase was caused by moderate-intensity stepping rather than light-intensity stepping (+15.2 minutes per day; 95% CI 4.1-26.2; P=.008). Conclusions: A home-based mHealth program to disrupt and replace sedentary time with stepping was feasible among and acceptable to older cancer survivors. Future studies are needed to evaluate the optimal approach for replacing sedentary behavior with standing and/or physical activity in this population. © 2021 Cindy K Blair, Elizabeth Harding, Charles Wiggins, Huining Kang, Matthew Schwartz, Amy Tarnower, Ruofei Du, Anita Y Kinney.},
author_keywords={Activity monitor; Cancer survivors; Consumer wearable; Light-intensity physical activity; Mobile health; Mobile phone; Physical activity; Sedentary behavior},
references={(2019), https://www.cancer.org/content/dam/cancer-org/research/cancer-facts-and-statistics/cancer-treatment-and-survivorship-facts-and-figures/cancer-treatment-and-survivorship-facts-and-figures-2019-2021.pdf, Cancer Treatment & Survivorship: Facts & Figures 2019-2021. American Cancer Society. [accessed 2021-03-22]; Avis, NE, Deimling, GT., Cancer survivorship and aging (2008) Cancer, 113 (12), pp. 3519-3529. , Dec 15;(Suppl): [FREE Full text] [doi] [Medline: 19058151]; Carver, JR, Shapiro, CL, Ng, A, Jacobs, L, Schwartz, C, Virgo, KS, American Society of Clinical Oncology clinical evidence review on the ongoing care of adult cancer survivors: cardiac and pulmonary late effects (2007) J Clin Oncol, 25 (25), pp. 3991-4008. , ASCO Cancer Survivorship Expert Panel. Sep 01; [doi] [Medline: 17577017]; Deimling, GT, Arendt, JA, Kypriotakis, G, Bowman, KF., Functioning of older, long-term cancer survivors: the role of cancer and comorbidities (2009) J Am Geriatr Soc, 57, pp. 289-292. , Suppl 2: [doi] [Medline: 20122020]; Hewitt, M, Rowland, JH, Yancik, R., Cancer survivors in the United States: age, health, and disability (2003) J Gerontol A Biol Sci Med Sci, 58 (1), pp. 82-91. , Jan; [doi] [Medline: 12560417]; Guralnik, JM,

Ferrucci, L, Simonsick, EM, Salive, ME, Wallace, RB., Lower-extremity function in persons over the age of 70 years as a predictor of subsequent disability (1995) N Engl J Med, 332 (9), pp. 556-561. , Mar 02; [doi] [Medline: 7838189]; Yabroff, KR, Kim, Y., Time costs associated with informal caregiving for cancer survivors (2009) Cancer, 115 (18), pp. 4362-4373. , Sep 15;(Suppl): [FREE Full text] [doi] [Medline: 19731345]; Guy, GPJ, Ekwueme, DU, Yabroff, KR, Dowling, EC, Li, C, Rodriguez, JL, Economic burden of cancer survivorship among adults in the United States (2013) J Clin Oncol, 31 (30), pp. 3749-3757. , Oct 20; [FREE Full text] [doi] [Medline: 24043731]; Vissers, PAJ, Thong, MSY, Pouwer, F, Zanders, MMJ, Coebergh, JWW, van de Poll-Franse, LV., The impact of comorbidity on Health-Related Quality of Life among cancer survivors: analyses of data from the PROFILES registry (2013) J Cancer Surviv, 7 (4), pp. 602-613. , Dec; [doi] [Medline: 23918453]; Smith, AW, Reeve, BB, Bellizzi, KM, Harlan, LC, Klabunde, CN, Amsellem, M, Cancer, comorbidities, and health-related quality of life of older adults (2008) Health Care Financ Rev, 29 (4), pp. 41-56. , [FREE Full text] [Medline: 18773613]; George, SM, Alfano, CM, Groves, J, Karabulut, Z, Haman, KL, Murphy, BA, Objectively measured sedentary time is related to quality of life among cancer survivors (2014) PLoS One, 9 (2). , [FREE Full text] [doi] [Medline: 24505335]; Owen, N, Healy, GN, Matthews, CE, Dunstan, DW., Too much sitting: the population health science of sedentary behavior (2010) Exerc Sport Sci Rev, 38 (3), pp. 105-113. , Jul; [FREE Full text] [doi] [Medline: 20577058]; Dunlop, DD, Song, J, Arnston, EK, Semanik, PA, Lee, J, Chang, RW, Sedentary time in US older adults associated with disability in activities of daily living independent of physical activity (2015) J Phys Act Health, 12 (1), pp. 93-101. , Jan; [FREE Full text] [doi] [Medline: 24510000]; Healy, GN, Matthews, CE, Dunstan, DW, Winkler, EAH, Owen, N., Sedentary time and cardio-metabolic biomarkers in US adults: NHANES 2003-06 (2011) Eur Heart J, 32 (5), pp. 590-597. , Mar; [FREE Full text] [doi] [Medline: 21224291]; Lyden, K, Keadle, SK, Staudenmayer, J, Braun, B, Freedson, PS., Discrete features of sedentary behavior impact cardiometabolic risk factors (2015) Med Sci Sports Exerc, 47 (5), pp. 1079-1086. , May; [FREE Full text] [doi] [Medline: 25202848]; Healy, GN, Wijndaele, K, Dunstan, DW, Shaw, JE, Salmon, J, Zimmet, PZ, Objectively measured sedentary time, physical activity, and metabolic risk: the Australian Diabetes, Obesity and Lifestyle Study (AusDiab) (2008) Diabetes Care, 31 (2), pp. 369-371. , Feb; [doi] [Medline: 18000181]; (2008) U.S. Department of Health and Human Services, , https://www.europarc.org/wp-content/uploads/2018/03/Physical-Activity-Guidelines-Advisory-Committee-Report-2008.pdf, Physical Activity Guidelines Advisory Committee Report 2008. [accessed 2021-03-22]; Lee, IM, Shiroma, EJ, Lobelo, F, Puska, P, Blair, SN, Katzmarzyk, PT, Lancet Physical Activity Series Working Group. Effect of physical inactivity on major non-communicable diseases worldwide: an analysis of burden of disease and life expectancy (2012) Lancet, 380 (9838), pp. 219-229. , Jul 21; [FREE Full text] [doi] [Medline: 22818936]; Blair, CK, Robien, K, Inoue-Choi, M, Rahn, W, Lazovich, D., Physical inactivity and risk of poor quality of life among elderly cancer survivors compared to women without cancer: the Iowa Women's Health Study (2016) J Cancer Surviv, 10 (1), pp. 103-112. , Feb; [FREE Full text] [doi] [Medline: 26008207]; Hamilton, MT, Hamilton, DG, Zderic, TW., Role of low energy expenditure and sitting in obesity, metabolic syndrome, type 2 diabetes, and cardiovascular disease (2007) Diabetes, 56 (11), pp. 2655-2667. , Nov; [FREE Full text] [doi] [Medline: 17827399]; Hamilton, MT, Healy, GN, Dunstan, DW, Zderic, TW, Owen, N., Too little exercise and too much sitting: inactivity physiology and the need for new recommendations on sedentary behavior (2008) Curr Cardiovasc Risk Rep, 2 (4), pp. 292-298. , Jul; [FREE Full text] [doi] [Medline: 22905272]; Tremblay,

MS, Aubert, S, Barnes, JD, Saunders, TJ, Carson, V, Latimer-Cheung, AE, Sedentary Behavior Research Network (SBRN) - Terminology Consensus Project process and outcome (2017) Int J Behav Nutr Phys Act, 14 (1), p. 75. , SBRN Terminology Consensus Project Participants. Jun 10; [FREE Full text] [doi] [Medline: 28599680]; Stamatakis, E, Hamer, M, Dunstan, DW., Screen-based entertainment time, all-cause mortality, and cardiovascular events: population-based study with ongoing mortality and hospital events follow-up (2011) J Am Coll Cardiol, 57 (3), pp. 292-299. , Jan 18; [FREE Full text] [doi] [Medline: 21232666]; Hawkes, AL, Lynch, BM, Owen, N, Aitken, JF., Lifestyle factors associated concurrently and prospectively with co-morbid cardiovascular disease in a population-based cohort of colorectal cancer survivors (2011) Eur J Cancer, 47 (2), pp. 267-276. , Jan; [doi] [Medline: 21074408]; Arem, H, Pfeiffer, RM, Moore, SC, Brinton, LA, Matthews, CE., Body mass index, physical activity, and television time in relation to mortality risk among endometrial cancer survivors in the NIH-AARP Diet and Health Study cohort (2016) Cancer Causes Control, 27 (11), pp. 1403-1409. , Nov; [FREE Full text] [doi] [Medline: 27730319]; Campbell, MK, Demark-Wahnefried, W, Symons, M, Kalsbeek, WD, Dodds, J, Cowan, A, Fruit and vegetable consumption and prevention of cancer: the Black Churches United for Better Health project (1999) Am J Public Health, 89 (9), pp. 1390-1396. , Sep; [doi] [Medline: 10474558]; Ratjen, I, Schafmayer, C, di Giuseppe, R, Waniek, S, Plachta-Danielzik, S, Koch, M, Postdiagnostic physical activity, sleep duration, and TV watching and all-cause mortality among long-term colorectal cancer survivors: a prospective cohort study (2017) BMC Cancer, 17 (1), p. 701. , Oct 25; [FREE Full text] [doi] [Medline: 29070017]; Phillips, SM, Awick, EA, Conroy, DE, Pellegrini, CA, Mailey, EL, McAuley, E., Objectively measured physical activity and sedentary behavior and quality of life indicators in survivors of breast cancer (2015) Cancer, 121 (22), pp. 4044-4052. , Nov 15; [FREE Full text] [doi] [Medline: 26308157]; van Roekel, EH, Winkler, EA, Bours, MJ, Lynch, BM, Willems, PJ, Meijer, K, Associations of sedentary time and patterns of sedentary time accumulation with health-related quality of life in colorectal cancer survivors (2016) Prev Med Rep, 4, pp. 262-269. , Dec;: [FREE Full text] [doi] [Medline: 27419042]; Lynch, BM, Cerin, E, Owen, N, Hawkes, AL, Aitken, JF., Television viewing time of colorectal cancer survivors is associated prospectively with quality of life (2011) Cancer Causes Control, 22 (8), pp. 1111-1120. , Aug; [doi] [Medline: 21656163]; Dunstan, DW, Kingwell, BA, Larsen, R, Healy, GN, Cerin, E, Hamilton, MT, Breaking up prolonged sitting reduces postprandial glucose and insulin responses (2012) Diabetes Care, 35 (5), pp. 976-983. , May; [FREE Full text] [doi] [Medline: 22374636]; Howard, BJ, Fraser, SF, Sethi, P, Cerin, E, Hamilton, MT, Owen, N, Impact on hemostatic parameters of interrupting sitting with intermittent activity (2013) Med Sci Sports Exerc, 45 (7), pp. 1285-1291. , Jul; [doi] [Medline: 23439415]; Sardinha, LB, Santos, DA, Silva, AM, Baptista, F, Owen, N., Breaking-up sedentary time is associated with physical function in older adults (2015) J Gerontol A Biol Sci Med Sci, 70 (1), pp. 119-124. , Jan; [doi] [Medline: 25324221]; Larsen, RN, Kingwell, BA, Sethi, P, Cerin, E, Owen, N, Dunstan, DW., Breaking up prolonged sitting reduces resting blood pressure in overweight/obese adults (2014) Nutr Metab Cardiovasc Dis, 24 (9), pp. 976-982. , Sep; [doi] [Medline: 24875670]; Lynch, BM, Dunstan, DW, Vallance, JK, Owen, N., Don't take cancer sitting down: a new survivorship research agenda (2013) Cancer, 119 (11), pp. 1928-1935. , Jun 01; [FREE Full text] [doi] [Medline: 23504979]; Buman, MP, Hekler, EB, Haskell, WL, Pruitt, L, Conway, TL, Cain, KL, Objective light-intensity physical activity associations with rated health in older adults (2010) Am J Epidemiol, 172 (10), pp. 1155-1165. , Nov 15; [FREE Full text] [doi] [Medline: 20843864]; Blair, CK,

Morey, MC, Desmond, RA, Cohen, HJ, Sloane, R, Snyder, DC, Light-intensity activity attenuates functional decline in older cancer survivors (2014) Med Sci Sports Exerc, 46 (7), pp. 1375-1383. , Jul; [FREE Full text] [doi] [Medline: 24389524]; van Waart, H, Stuiver, MM, van Harten, WH, Geleijn, E, Kieffer, JM, Buffart, LM, Effect of low-intensity physical activity and moderate- to high-intensity physical exercise during adjuvant chemotherapy on physical fitness, fatigue, and chemotherapy completion rates: results of the PACES randomized clinical trial (2015) J Clin Oncol, 33 (17), pp. 1918-1927. , Jun 10; [doi] [Medline: 25918291]; Van Roekel, EH, Bours, MJL, Breedveld-Peters, JJL, Meijer, K, Kant, I, Van Den Brandt, PA, Light physical activity is associated with quality of life after colorectal cancer (2015) Med Sci Sports Exerc, 47 (12), pp. 2493-2503. , Dec; [doi] [Medline: 25970666]; van Roekel, EH, Duchâteau, J, Bours, MJL, van Delden, L, Breedveld-Peters, JJL, Koole, JL, Longitudinal associations of light-intensity physical activity with quality of life, functioning and fatigue after colorectal cancer (2020) Qual Life Res, 29 (11), pp. 2987-2998. , Nov 2; [FREE Full text] [doi] [Medline: 32617891]; Dunlop, DD, Song, J, Semanik, PA, Sharma, L, Bathon, JM, Eaton, CB, Relation of physical activity time to incident disability in community dwelling adults with or at risk of knee arthritis: prospective cohort study (2014) Br Med J, 348. , Apr 29;: [FREE Full text] [doi] [Medline: 24782514]; Thraen-Borowski, KM, Trentham-Dietz, A, Edwards, DF, Koltyn, KF, Colbert, LH., Dose-response relationships between physical activity, social participation, and health-related quality of life in colorectal cancer survivors (2013) J Cancer Surviv, 7 (3), pp. 369-378. , Sep; [FREE Full text] [doi] [Medline: 23546822]; Conroy, DE, Wolin, KY, Blair, CK, Demark-Wahnefried, W., Gender-varying associations between physical activity intensity and mental quality of life in older cancer survivors (2017) Support Care Cancer, 25 (11), pp. 3465-3473. , Nov; [FREE Full text] [doi] [Medline: 28620700]; Matthews, CE, Keadle, SK, Troiano, RP, Kahle, L, Koster, A, Brychta, R, Accelerometer-measured dose-response for physical activity, sedentary time, and mortality in US adults (2016) Am J Clin Nutr, 104 (5), pp. 1424-1432. , Nov; [FREE Full text] [doi] [Medline: 27707702]; Matthews, CE, Moore, SC, Sampson, J, Blair, A, Xiao, Q, Keadle, SK, Mortality benefits for replacing sitting time with different physical activities (2015) Med Sci Sports Exerc, 47 (9), pp. 1833-1840. , Sep; [FREE Full text] [doi] [Medline: 25628179]; Reid, N, Daly, RM, Winkler, EAH, Gardiner, PA, Eakin, EG, Owen, N, Associations of monitor-assessed activity with performance-based physical function (2016) PLoS One, 11 (4). , [FREE Full text] [doi] [Medline: 27073888]; Elosua, R, Redondo, A, Segura, A, Fiol, M, Aldasoro, E, Vega, G, Dose-response association of physical activity with acute myocardial infarction: do amount and intensity matter? (2013) Prev Med, 57 (5), pp. 567-572. , Nov; [doi] [Medline: 23954185]; Gourlan, M, Bernard, P, Bortolon, C, Romain, AJ, Lareyre, O, Carayol, M, Efficacy of theory-based interventions to promote physical activity. A meta-analysis of randomised controlled trials (2016) Health Psychol Rev, 10 (1), pp. 50-66. , Apr 10; [doi] [Medline: 25402606]; Glanz, K, Bishop, DB., The role of behavioral science theory in development and implementation of public health interventions (2010) Annu Rev Public Health, 31, pp. 399-418. , [doi] [Medline: 20070207]; McGarrigle, L, Todd, C., Promotion of physical activity in older people using mhealth and ehealth technologies: rapid review of reviews (2020) J Med Internet Res, 22 (12). , Dec 29; [FREE Full text] [doi] [Medline: 33372894]; Compernolle, S, DeSmet, A, Poppe, L, Crombez, G, De Bourdeaudhuij, I, Cardon, G, Effectiveness of interventions using self-monitoring to reduce sedentary behavior in adults: a systematic review and meta-analysis (2019) Int J Behav Nutr Phys Act, 16 (1), p. 63. , Aug 13; [FREE Full text] [doi] [Medline:

31409357]; Stockwell, S, Schofield, P, Fisher, A, Firth, J, Jackson, SE, Stubbs, B, Digital behavior change interventions to promote physical activity and/or reduce sedentary behavior in older adults: A systematic review and meta-analysis (2019) Exp Gerontol, 120, pp. 68-87. , Jun;: [doi] [Medline: 30836130]; Gardner, B, Smith, L, Lorencatto, F, Hamer, M, Biddle, SJ., How to reduce sitting time? A review of behaviour change strategies used in sedentary behaviour reduction interventions among adults (2016) Health Psychol Rev, 10 (1), pp. 89-112. , [FREE Full text] [doi] [Medline: 26315814]; Lyons, EJ, Lewis, ZH, Mayrsohn, BG, Rowland, JL., Behavior change techniques implemented in electronic lifestyle activity monitors: a systematic content analysis (2014) J Med Internet Res, 16 (8), p. e192. , Aug 15; [FREE Full text] [doi] [Medline: 25131661]; Duncan, M, Murawski, B, Short, CE, Rebar, AL, Schoeppe, S, Alley, S, Activity trackers implement different behavior change techniques for activity, sleep, and sedentary behaviors (2017) Interact J Med Res, 6 (2), p. e13. , Aug 14; [FREE Full text] [doi] [Medline: 28807889]; Demark-Wahnefried, W, Pinto, BM, Gritz, ER., Promoting health and physical function among cancer survivors: potential for prevention and questions that remain (2006) J Clin Oncol, 24 (32), pp. 5125-5131. , Nov 10; [doi] [Medline: 17093274]; Courneya, KS, Rogers, LQ, Campbell, KL, Vallance, JK, Friedenreich, CM., Top 10 research questions related to physical activity and cancer survivorship (2015) Res Q Exerc Sport, 86 (2), pp. 107-116. , Jun; [doi] [Medline: 25629322]; Lynch, BM, Nguyen, NH, Moore, MM, Reeves, MM, Rosenberg, DE, Boyle, T, A randomized controlled trial of a wearable technology-based intervention for increasing moderate to vigorous physical activity and reducing sedentary behavior in breast cancer survivors: The ACTIVATE Trial (2019) Cancer, 125 (16), pp. 2846-2855. , Aug 15; [FREE Full text] [doi] [Medline: 31012970]; Gomersall, SR, Skinner, TL, Winkler, E, Healy, GN, Eakin, E, Fjeldsoe, B., Feasibility, acceptability and efficacy of a text message-enhanced clinical exercise rehabilitation intervention for increasing 'whole-of-day' activity in people living with and beyond cancer (2019) BMC Public Health, 19, p. 542. , Jun 03;(Suppl 2): [FREE Full text] [doi] [Medline: 31159752]; Trinh, L, Arbour-Nicitopoulos, KP, Sabiston, CM, Berry, SR, Loblaw, A, Alibhai, SMH, RiseTx: testing the feasibility of a web application for reducing sedentary behavior among prostate cancer survivors receiving androgen deprivation therapy (2018) Int J Behav Nutr Phys Act, 15 (1), p. 49. , Jun 07; [FREE Full text] [doi] [Medline: 29880049]; Visser, M, Koster, A., Development of a questionnaire to assess sedentary time in older persons - a comparative study using accelerometry (2013) BMC Geriatr, 13, p. 80. , Jul 30;: [FREE Full text] [doi] [Medline: 23899190]; Schmitz, KH, Courneya, KS, Matthews, C, Demark-Wahnefried, W, Galvão, DA, Pinto, BM, American College of Sports Medicine. American College of Sports Medicine roundtable on exercise guidelines for cancer survivors (2010) Med Sci Sports Exerc, 42 (7), pp. 1409-1426. , Jul; [doi] [Medline: 20559064]; Surveillance, Epidemiology, and End Results (SEER) Program, , https://seer.cancer.gov/, National Cancer Institute. [accessed 2020-02-23]; Bandura, A., (1977) Social learning theory, , Prentice Hall, N. J: Englewood Cliffs; Bandura, A., (1986) Social foundations of thought and action : a social cognitive theory, , Prentice-Hall, N.J: Englewood Cliffs; A; Gardiner, PA, Eakin, EG, Healy, GN, Owen, N., Feasibility of reducing older adults' sedentary time (2011) Am J Prev Med, 41 (2), pp. 174-177. , Aug; [doi] [Medline: 21767725]; Tudor-Locke, C, Craig, CL, Aoyagi, Y, Bell, RC, Croteau, KA, De Bourdeaudhuij, I, How many steps/day are enough? For older adults and special populations (2011) Int J Behav Nutr Phys Act, 8, p. 80. , Jul 28;: [FREE Full text] [doi] [Medline: 21798044]; Buman, MP, Winkler, EAH, Kurka, JM, Hekler, EB, Baldwin, CM, Owen, N, Reallocating time to sleep, sedentary behaviors, or active behaviors: associations with cardiovascular disease risk

biomarkers, NHANES 2005-2006 (2014) Am J Epidemiol, 179 (3), pp. 323-334. , Feb 01; [doi] [Medline: 24318278]; Chastin, SFM, Granat, MH., Methods for objective measure, quantification and analysis of sedentary behaviour and inactivity (2010) Gait Posture, 31 (1), pp. 82-86. , Jan; [doi] [Medline: 19854651]; Kozey-Keadle, S, Libertine, A, Lyden, K, Staudenmayer, J, Freedson, PS., Validation of wearable monitors for assessing sedentary behavior (2011) Med Sci Sports Exerc, 43 (8), pp. 1561-1567. , Aug; [doi] [Medline: 21233777]; Grant, PM, Ryan, CG, Tigbe, WW, Granat, MH., The validation of a novel activity monitor in the measurement of posture and motion during everyday activities (2006) Br J Sports Med, 40 (12), pp. 992-997. , Dec; [FREE Full text] [doi] [Medline: 16980531]; Sellers, C, Dall, P, Grant, M, Stansfield, B., Validity and reliability of the activPAL3 for measuring posture and stepping in adults and young people (2016) Gait Posture, 43, pp. 42-47. , Jan;: [doi] [Medline: 26669950]; Lyden, K, Keadle, SK, Staudenmayer, J, Freedson, PS., The activPALTM accurately classifies activity intensity categories in healthy adults (2017) Med Sci Sports Exerc, 49 (5), pp. 1022-1028. , May; [FREE Full text] [doi] [Medline: 28410327]; Guralnik, JM, Simonsick, EM, Ferrucci, L, Glynn, RJ, Berkman, LF, Blazer, DG, A short physical performance battery assessing lower extremity function: association with self-reported disability and prediction of mortality and nursing home admission (1994) J Gerontol, 49 (2), pp. 85-94. , Mar; [doi] [Medline: 8126356]; George, SM, Alfano, CM, Wilder Smith, A, Irwin, ML, McTiernan, A, Bernstein, L, Sedentary behavior, health-related quality of life, and fatigue among breast cancer survivors (2013) J Phys Act Health, 10 (3), pp. 350-358. , Mar; [FREE Full text] [doi] [Medline: 22820125]; Balboa-Castillo, T, León-Muñoz, LM, Graciani, A, Rodríguez-Artalejo, F, Guallar-Castillón, P., Longitudinal association of physical activity and sedentary behavior during leisure time with health-related quality of life in community-dwelling older adults (2011) Health Qual Life Outcomes, 9, p. 47. , Jun 27;: [FREE Full text] [doi] [Medline: 21708011]; Baker, F, Haffer, SC, Denniston, M., Health-related quality of life of cancer and noncancer patients in Medicare managed care (2003) Cancer, 97 (3), pp. 674-681. , Feb 01; [FREE Full text] [doi] [Medline: 12548610]; Walters, SJ, Munro, JF, Brazier, JE., Using the SF-36 with older adults: a cross-sectional community-based survey (2001) Age Ageing, 30 (4), pp. 337-343. , Jul; [doi] [Medline: 11509313]; Johnston, RI., Quality Metric, , https://www.qualitymetric.com/, [accessed 2021-04-07]; Patient-Reported Outcomes Measurement Information System, , http://www.healthmeasures.net/explore-measurement-systems/promis/intro-to-promis, PROMIS: [accessed 2021-03-22]; https://www.facit.org/, FACIT Measurement System. [accessed 2021-03-22]; Sangha, O, Stucki, G, Liang, MH, Fossel, AH, Katz, JN., The Self-Administered Comorbidity Questionnaire: a new method to assess comorbidity for clinical and health services research (2003) Arthritis Rheum, 49 (2), pp. 156-163. , Apr 15; [FREE Full text] [doi] [Medline: 12687505]; Lyden, K., Package 'activpalProcessing', , https://cran.r-project.org/web/packages/activpalProcessing/index.html, [accessed 2021-03-22]; Edwardson, CL, Yates, T, Biddle, SJH, Davies, MJ, Dunstan, DW, Esliger, DW, Effectiveness of the Stand More AT (SMArT) Work intervention: cluster randomised controlled trial (2018) Br Med J, p. 363. , Oct 10;: [FREE Full text] [doi] [Medline: 30305278]; Edwardson, CL, Winkler, EA, Bodicoat, DH, Yates, T, Davies, MJ, Dunstan, DW, Considerations when using the activPAL monitor in field-based research with adult populations (2017) J Sport Health Sci, 6 (2), pp. 162-178. , Jun; [FREE Full text] [doi] [Medline: 30356601]; Fitzsimons, CF, Kirk, A, Baker, G, Michie, F, Kane, C, Mutrie, N., Using an individualised consultation and activPAL" feedback to reduce sedentary time in older Scottish adults: results of a feasibility and pilot study (2013)

Prev Med, 57 (5), pp. 718-720. , Nov; [doi] [Medline: 23891853]; Rosenberg, DE, Gell, NM, Jones, SMW, Renz, A, Kerr, J, Gardiner, PA, The feasibility of reducing sitting time in overweight and obese older adults (2015) Health Educ Behav, 42 (5), pp. 669-676. , Oct; [FREE Full text] [doi] [Medline: 25794518]; (2018) Physical Activity Guidelines Advisory Committee Scientific Report, , http://fitnessmedicoitaliano.it/wp-content/uploads/2016/07/ATTIVIT%C3%A0-FISICA-2018-LINEE-GUIDA.pdf, 2018 U.S. Department of Health and Human Services. [accessed 2021-03-22]; Bull, FC, Al-Ansari, SS, Biddle, S, Borodulin, K, Buman, MP, Cardon, G, World Health Organization 2020 guidelines on physical activity and sedentary behaviour Br J Sports Med, 54 (24), pp. 1451-1462. , 2020 Dec 25; [FREE Full text] [doi] [Medline: 33239350]; Hays, RD, Morales, LS., The RAND-36 measure of health-related quality of life (2001) Ann Med, 33 (5), pp. 350-357. , Jul; [doi] [Medline: 11491194]; Kwon, S, Perera, S, Pahor, M, Katula, JA, King, AC, Groessl, EJ, What is a meaningful change in physical performance? Findings from a clinical trial in older adults (the LIFE-P study) (2009) J Nutr Health Aging, 13 (6), pp. 538-544. , Jun; [FREE Full text] [doi] [Medline: 19536422]; Lyons, EJ, Swartz, MC, Lewis, ZH, Martinez, E, Jennings, K., Feasibility and acceptability of a wearable technology physical activity intervention with telephone counseling for mid-aged and older adults: a randomized controlled pilot trial (2017) JMIR Mhealth Uhealth, 5 (3), p. e28. , Mar 06; [FREE Full text] [doi] [Medline: 28264796]; Wu, HS, Gal, R, van Sleeuwen, NC, Brombacher, AC, IJsselsteijn, WA, May, AM, Breast cancer survivors' experiences with an activity tracker integrated into a supervised exercise program: qualitative study (2019) JMIR Mhealth Uhealth, 7 (2). , Feb 21; [FREE Full text] [doi] [Medline: 30789349]; Matson, TE, Renz, AD, Takemoto, ML, McClure, JB, Rosenberg, DE., Acceptability of a sitting reduction intervention for older adults with obesity (2018) BMC Public Health, 18 (1), p. 706. , Jun 07; [FREE Full text] [doi] [Medline: 29879948]; Gardner, B, Lally, P, Wardle, J., Making health habitual: the psychology of 'habit-formation' and general practice (2012) Br J Gen Pract, 62 (605), pp. 664-666. , Dec; [FREE Full text] [doi] [Medline: 23211256]; Lally, P, Wardle, J, Gardner, B., Experiences of habit formation: a qualitative study (2011) Psychol Health Med, 16 (4), pp. 484-489. , Aug; [doi] [Medline: 21749245]; Matson, TE, Anderson, ML, Renz, AD, Greenwood-Hickman, MA, McClure, JB, Rosenberg, DE., Changes in self-reported health and psychosocial outcomes in older adults enrolled in sedentary behavior intervention study (2019) Am J Health Promot, 33 (7), pp. 1053-1057. , Sep; [doi] [Medline: 30957508]; Lynch, BM, Dunstan, DW, Healy, GN, Winkler, E, Eakin, E, Owen, N., Objectively measured physical activity and sedentary time of breast cancer survivors, and associations with adiposity: findings from NHANES (2003-2006) (2010) Cancer Causes Control, 21 (2), pp. 283-288. , Feb; [doi] [Medline: 19882359]; Lynch, BM, Dunstan, DW, Winkler, E, Healy, GN, Eakin, E, Owen, N., Objectively assessed physical activity, sedentary time and waist circumference among prostate cancer survivors: findings from the National Health and Nutrition Examination Survey (2003-2006) (2011) Eur J Cancer Care (Engl), 20 (4), pp. 514-519. , Jul; [doi] [Medline: 20597954]; Dall, PM, McCrorie, PRW, Granat, MH, Stansfield, BW., Step accumulation per minute epoch is not the same as cadence for free-living adults (2013) Med Sci Sports Exerc, 45 (10), pp. 1995-2001. , Oct; [doi] [Medline: 23568091]; Stansfield, B, Hajarnis, M, Sudarshan, R., Characteristics of very slow stepping in healthy adults and validity of the activPAL3" activity monitor in detecting these steps (2015) Med Eng Phys, 37 (1), pp. 42-47. , Jan; [doi] [Medline: 25455167]; Brickwood, KJ, Watson, G, O'Brien, J, Williams, AD., Consumer-based wearable activity trackers increase physical activity participation: systematic review and meta-analysis (2019) JMIR Mhealth Uhealth,

7 (4). , Apr 12; [FREE Full text] [doi] [Medline: 30977740]; Martin, A, Fitzsimons, C, Jepson, R, Saunders, DH, van der Ploeg, HP, Teixeira, PJ, Interventions with potential to reduce sedentary time in adults: systematic review and meta-analysis (2015) Br J Sports Med, 49 (16), pp. 1056-1063. , EuroFIT consortium. Aug; [doi] [Medline: 25907181]; Prince, SA, Saunders, TJ, Gresty, K, Reid, RD., A comparison of the effectiveness of physical activity and sedentary behaviour interventions in reducing sedentary time in adults: a systematic review and meta-analysis of controlled trials (2014) Obes Rev, 15 (11), pp. 905-919. , Nov; [FREE Full text] [doi] [Medline: 25112481]; Edwardson, CL, Henson, J, Biddle, SJH, Davies, MJ, Khunti, K, Maylor, B, activPAL and Actigraph assessed sedentary behavior and cardiometabolic health markers (2020) Med Sci Sports Exerc, 52 (2), pp. 391-397. , Feb; [doi] [Medline: 31479008]; Swain, CTV, Nguyen, NH, Eagles, T, Vallance, JK, Boyle, T, Lahart, IM, Postdiagnosis sedentary behavior and health outcomes in cancer survivors: a systematic review and meta-analysis (2020) Cancer, 126 (4), pp. 861-869. , Feb 15; [FREE Full text] [doi] [Medline: 31714596]; Chia, GLC, Anderson, A, McLean, LA., Behavior change techniques incorporated in fitness trackers: content analysis (2019) JMIR Mhealth Uhealth, 7 (7). , Jul 23; [FREE Full text] [doi] [Medline: 31339101]},
correspondence_address1={Blair, C.K.; Department of Internal Medicine, 1 University of New Mexico, MSC07-4025, United States; email: CiBlair@salud.unm.edu},
publisher={JMIR Publications Inc.},
issn={23691999},
language={English},
abbrev_source_title={JMIR Cancer},
document_type={Article},
source={Scopus},
}

@ARTICLE{McMahon2021,
author={McMahon, J. and Thompson, D.R. and Pascoe, M.C. and Brazil, K. and Ski, C.F.},
title={eHealth interventions for reducing cardiovascular disease risk in men: A systematic review and meta-analysis},
journal={Preventive Medicine},
year={2021},
volume={145},
doi={10.1016/j.ypmed.2020.106402},
art_number={106402},
note={cited By 0},
url={https://www.scopus.com/inward/record.uri?eid=2-s2.0-85099628290&doi=10.1016%2fj.ypmed.2020.106402&partnerID=40&md5=984989ea0b3e0ee6d1b7268854903f35},
affiliation={School of Nursing and Midwifery, Queen's University Belfast, Belfast, United Kingdom; Institute for Health & Sport, Victoria University, Melbourne, Australia; Integrated Care Academy, University of Suffolk, Ipswich, United Kingdom},
abstract={Men remain at a higher risk of developing cardiovascular disease (CVD) than women and behavioral risk factor modification is an important preventive measure. However, engaging men in behavior change interventions is challenging. Although men often indicate a preference for gender-specific information and support, this rarely occurs. eHealth interventions have the potential to address this gap, though their effectiveness for reducing CVD risk in men is unclear. Therefore, the aim of this systematic review and

meta-analysis was to evaluate the effectiveness of eHealth interventions for reducing CVD risk in men. A search of published randomised controlled trials with no date restrictions up to July 2020 was conducted to identify those targeting at least two major CVD risk factors. Nine trials were identified and reviewed. Study quality ranged from low to unclear, with one trial at a high risk of bias. Compared to those in a control group or receiving printed materials, participants randomised to an eHealth intervention had statistically significant improvements in BMI (Z=-2.75, p=0.01), body weight (Z=-3.25, p=0.01), waist circumference (Z=-2.30, p=0.02) and systolic (Z=-3.57, p=0.01) and diastolic (Z=-3.56, p=0.01) blood pressure. Though less evident, there were also improvements in physical activity and diet in favour of the intervention group. This review suggests that eHealth interventions can reduce CVD risk in adult men through behavior change. However, we were unable to determine the association between intervention characteristics and outcomes. Also, overall, participant adherence to the intervention was poor. Both of these issues should be considered in future studies. © 2021 Elsevier Inc.},
author_keywords={Cardiovascular risk; eHealth interventions; Men; Meta-analysis; Systematic review},
keywords={glucose; hemoglobin A1c; high density lipoprotein cholesterol; low density lipoprotein cholesterol; triacylglycerol, behavior change; body mass; body weight; caloric intake; cardiorespiratory fitness; cardiovascular disease; cardiovascular risk; cholesterol blood level; clinical effectiveness; diastolic blood pressure; disease association; glucose blood level; health promotion; hemoglobin blood level; human; physical activity; primary prevention; priority journal; processed food; resting heart rate; Review; risk reduction; sedentary lifestyle; systematic review; systolic blood pressure; telehealth; triacylglycerol blood level; waist circumference},
references={Alageel, S., Gulliford, M.C., McDermott, L., Wright, A.J., Multiple health behaviour change interventions for primary prevention of cardiovasculasr disease in primary care: systematic review and meta-analysis (2017) BMJ Open, 7; Borenstein, M., Hedges, L.V., Higgins, J.P.T., Rothstein, H.R., Introduction to Meta-Analysis (2009), John Wiley & Sons Chichester; Bottorff, J.L., Seaton, C.L., Johnson, S.T., Caperchione, C.M., Oliffe, J.L., More, K., Jaffer-Hirji, H., Tillotson, S.M., An updated review of interventions that include promotion of physical activity for adult men (2015) Sports Med., 45, pp. 775-800; Craig, P., Dieppe, P., Macintyre, S., Michie, S., Nazareth, I., Petticrew, M., Developing and evaluating complex interventions: the new Medical Research Council guidance (2008) BMJ, 337, p. a1655; Crane, M.M., Lutes, L.D., Ward, D.S., Bowling, J.M., Tate, D.F., A randomized trial testing the efficacy of a novel approach to weight loss among men with overweight and obesity (2015) Obesity, 23, pp. 2398-2405; Darker, C.D., French, D., Eves, F.F., Sniehotta, F.F., An intervention to promote walking amongst the general population based on an 'extended' Theory of Planned Behavior: a waiting list randomized controlled trial (2009) Psychol. Health, 25, pp. 71-88; Davies, C.A., Spence, J.C., Vandelanotte, C., Caperchione, C.M., Mummery, W., Meta-analysis of internet-delivered interventions to increase physical activity levels (2012) Int. J. Behav. Nutr. Phys. Act., 9, p. 52; De Vries, H., Brug, J., Computer-tailored interventions motivating people to adopt health promoting behaviours: introduction to a new apparoach (1999) Patient Educ. Couns., 36, pp. 99-105; Drew, R.J., Morgan, P.J., Pollock, E.R., Young, M.D., Impact of male-only lifestyle interventions on men's mental health: a systematic review and meta-analysis (2020) Obes. Rev., 21, p. e13014; Duncan, M.J., Vandelanotte, C., Rosenkranz, R.R., Caperchione, C.M., Ding, H., Ellison, M., George, E.S.,

Karunanithi, M., Effectiveness of a website and mobile phone based physical activity and nutrition intervention for middle-aged males: trial protocol and baseline findings of the ManUp study (2012) BMC Public Health, 12, p. 656; Duncan, M., Vandelanotte, C., Kolt, G.S., Rosenkranz, R.R., Caperchione, C.M., George, E.S., Ding, H., Karunanithi, M., Effectiveness of a web- and mobile phone-based intervention to promote physical activity and healthy eating in middle-aged males: randomized controlled trial of the ManUp study (2014) J. Med. Internet Res., 16, p. e136; Ebrahim, S., Taylor, F., Ward, K., Beswick, A., Burke, M., Davey Smith, G., Multiple risk factor interventions for primary prevention of coronary heart disease (Review) (2011) Cochrane Database Syst. Rev., 1; Gavarkovs, A.G., Burke, S.M., Petrella, R.J., Engaging men in chronic disease prevention and management programs: a scoping review (2016) Am. J. Mens Health, 10, pp. N145-N154; George, E.S., Increasing physical activity and reducing sedentary time in middle-aged males (2013), University of Western Sydney PhD dissertation; Higgins, J.P.T., Altman, D.G., Gotzsche, P.C., Juni, P., Moher, D., Oxman, A.D., Savovic, J., Weeks, L., The Cochrane Collaboration's tool for assessing risk of bias in randomised trials (2011) BMJ, 343, p. d5928; Higgins, J.P.T., Thomas, J., Chandler, J., Cumpston, M., Li, T., Page, M.J., Welch, V.A., Cochrane Handbook for Systematic Reviews of Interventions (2019), 2nd ed John Wiley & Sons Chichester; Hutchesson, M.J., Rollo, M.E., Krukowski, R., Ells, L., Harvey, J., Morgan, P.J., Callister, R., Collins, C.E., eHealth interventions for the prevention and treatment of overweight and obesity in adults: a systematic review with meta-analysis (2015) Obes. Rev., 16, pp. 376-392; Jahangiry, L., Farhangi, M.A., Shab-Bidar, S., Rezaei, F., Pashaei, T., Web-based physical activity interventions: a systematic review and meta-analysis of randomized controlled trials (2017) Public Health, 152, pp. 36-46; Kelders, S.M., Kok, R.N., Ossebaard, H.C., Van Gemert-Pijnen, J.E., Persuasive system design does matter: a systematic review of adherence to web-based interventions (2012) J. Med. Internet Res., 14, p. e152; Kroeze, W., Werkman, A., Brug, J., A systematic review of randomized trials on the effectiveness of computer-tailored education on physical activity and dietary behaviors (2006) Ann. Behav. Med., 31, pp. 205-223; Lau, Y., Chee, D.G.H., Chow, X.P., Cheng, L.J., Wong, S.N., Personalised eHealth interventions in adults with overweight and obesity: a systematic review and meta-analysis of randomised controlled trials (2020) Prev. Med., 132; Malik, S.H., Blake, H., Suggs, L.S., A systematic review of workplace health promotion interventions for increasing physical activity (2014) Br. J. Health Psychol., 19, pp. 149-180; Martin, A., Fitzsimons, C., Jepson, R., Saunders, D.H., Van Der Ploeg, H.P., Teixeira, P.J., Gray, C.M., Mutrie, N., Interventions with potential to reduce sedentary time in adults: systematic review and meta-analysis (2015) Br. J. Sports Med., 49, pp. 1056-1063; McGuinness, L.A., robvis: An R Package and Web Application for Visualising Risk-of-Bias Assessments (2019), https://github.com/mcguinlu/robvis; Moher, D., Liberati, A., Tetzlaff, J., Altman, D.G., PRISMA Group, Preferred reporting items for systematic reviews and meta-analyses: the PRISMA statement (2009) Ann. Intern. Med., 151, pp. 264-269; Moinuddin, A., Goel, A., Alcohol consumption and gender: a critical review (2016) J. Psychol. Psychotherapy, 6, pp. 1-4; Möller, H.-J., Effectiveness studies: advantages and disadvantages (2011) Dialogues Clin. Neurosci., 13, pp. 199-207; Morgan, P.J., Lubans, D.R., Collins, C.E., Warren, J.M., Callister, R., The SHED-IT randomized controlled trial: evaluation of an Internet-based weight-loss program for men (2009) Obesity, 17, pp. 2025-2032; Morgan, P.J., Collins, C.E., Plotnikoff, R.C., Cook, A.T., Berthon, B., Mitchell, S., Callister, R., Efficacy of a workplace-based weight loss program for overweight male shift workers: the Workplace POWER (Preventing Obesity

Without Eating like a Rabbit) randomized controlled trial (2011) Prev. Med., 52, pp. 317-325; Morgan, P.J., Callister, R., Collins, C.E., Plotnikoff, R.C., Young, M.D., Berry, N., McElduff, P., Aguiar, E., The SHED-IT community trial: a randomized controlled trial of internet- and paper-based weight loss programs tailored for overweight and obese men (2013) Ann. Behav. Med., 45, pp. 139-152; Muellmann, S., Forberger, S., Möllers, T., Zeeb, H., Pischke, C.R., Effectiveness of eHealth interventions for the promotion of physical activity in older adults: a systematic review protocol (2016) Syst. Rev., 5, p. 47; Norman, G.J., Zabinski, M.F., Adams, M.A., Rosenberg, D.E., Yaroch, A.L., Atienza, A.A., A review of eHealth interventions for physical activity and dietary behavior change (2007) Am. J. Prev. Med., 33, pp. 336-345; Pagoto, S.L., Schneider, K.L., Oleski, J.L., Luciani, J.M., Bodenlos, J.S., Whited, M.C., Male inclusion in randomized controlled trials of lifestyle weight loss interventions (2012) Obesity, 20, pp. 1234-1239; Patrick, K., Calfas, K.J., Norman, G.J., Rosenberg, D., Zabinski, M.F., Sallis, J.F., Rock, C.L., Dillon, L.W., Outcomes of a 12-month web-based intervention for overweight and obese men (2011) Ann. Behav. Med., 42, pp. 391-401; Tanaka, M., Adachi, Y., Adachi, K., Sato, C., Effects of a non-face-to-face behavioral weight-control program among Japanese overweight males: a randomized controlled trial (2010) Int. J. Behav. Med., 17, pp. 17-24; Turco, J.V., Inal-Veith, A., Fuster, V., Cardiovascular health promotion: an issue that can no longer wait (2018) J. Am. Coll. Cardiol., 72, pp. 908-913; van Gemert-Pijnen, L., Kelders, S.M., Kip, H., Sanderman, R., eHealth Research, Theory and Development: A Multidisciplinary Approach (2018), Routledge London; Vari, R., Scazzocchio, B., D'Amore, A., Giovannini, C., Gessani, S., Masella, R., Gender-related differences in lifestyle may affect health status (2016) Ann. Ist. Super. Sanita, 52, pp. 158-166; Veverka, D.V., Anderson, J., Auld, G.W., Coulter, G.R., Kennedy, C., Chapman, P.L., Use of the Stages of Change Model in improving nutrition and exercise habits in enlisted Air Force men (2003) Mil. Med., 168, pp. 373-379; Virani, S.S., Alonso, A., Benjamin, E.J., Bittencourt, M.S., Callaway, C.W., Carson, A.P., Chamberlain, A.M., Cheng, S., Heart disease and stroke statistics - 2020 update: a report from the American Heart Association (2020) Circulation, 141, pp. e139-e596; Webb, T.L., Joseph, J., Yardley, L., Michie, S., Using the Internet to promote health behavior change: a systematic review and meta-analysis of the impact of theoretical basis, use of behavior change techniques, and mode of delivery on efficacy (2010) J. Med. Internet Res., 12, p. e4; White, A., McKee, M., Richardson, N., De Visser, R., Madsen, S.A., De Sousa, B.C., Hogston, R., Makara, P., Europe's men need their own health strategy (2011) BMJ, 343, p. d7397; Widmer, R.J., Collins, N.M., Collins, C.S., West, C.P., Lerman, L.O., Lerman, A., Digital health interventions for the prevention of cardiovascular disease: a systematic review and meta-analysis (2015) Mayo Clin. Proc., 90, pp. 469-480; World Health Organization, Cardiovascular Diseases (CVDs) (2017), https://www.who.int/en/news-room/fact-sheets/detail/cardiovascular-diseases-(cvds); Young, M.D., Morgan, P.J., Plotnikoff, R.C., Callister, R., Collins, C.E., Effectiveness of male-only weight loss and weight loss maintenance interventions: a systematic review with meta-analysis (2012) Obes. Rev., 13, pp. 393-408; Yousaf, O., Grunfeld, E.A., Hunter, M.S., A systematic review of the factors associated with delays in medical and psychological help-seeking among men (2015) Health Psychol. Rev., 9, pp. 264-276},
correspondence_address1={Ski, C.F.; School of Nursing and Midwifery, United Kingdom; email: C.Ski@qub.ac.uk},
publisher={Academic Press Inc.},
issn={00917435},

```
coden={PVTMA},
pubmed_id={33388336},
language={English},
abbrev_source_title={Prev. Med.},
document_type={Review},
source={Scopus},
}

@ARTICLE{Thornton2021,
author={Thornton, L. and Gardner, L.A. and Osman, B. and Green, O. and
Champion, K.E. and Bryant, Z. and Teesson, M. and Kay-Lambkin, F. and Chapman,
C. and The Health4Life Team},
title={A multiple health behavior change, self-monitoring mobile app for
adolescents: Development and usability study of the Health4Life app},
journal={JMIR Formative Research},
year={2021},
volume={5},
number={4},
doi={10.2196/25513},
art_number={e25513},
note={cited By 0},
url={https://www.scopus.com/inward/record.uri?eid=2-s2.0-85104112552&doi=10.219
6%2f25513&partnerID=40&md5=c9b26f749227b401596b3c10b5019889},
affiliation={The Matilda Centre for Research in Mental Health and Substance
Use, The University of Sydney, Sydney, NSW, Australia; Priority Research Centre
for Brain and Mental Health, The University of Newcastle, Newcastle, NSW,
Australia; School of Public Health and Community Medicine, University of New
South Wales, Sydney, NSW, Australia; Department of Exercise Physiology, School
of Medical Sciences, Faculty of Medicine, University of New South Wales,
Sydney, Sydney, NSW, Australia; Department of Preventive Medicine, Northwestern
University Feinberg School of Medicine, Chicago, IL, United States; National
Drug Research Institute, Curtin University, Perth, WA, Australia; School of
Psychology, Centre for Youth Substance Abuse Research, The University of
Queensland, Brisbane, QLD, Australia},
abstract={Background: The link between chronic diseases and the Big 6 lifestyle
risk behaviors (ie, poor diet, physical inactivity, smoking, alcohol use,
sedentary recreational screen time, and poor sleep) is well established. It is
critical to target these lifestyle risk behaviors, as they often co-occur and
emerge in adolescence. Smartphones have become an integral part of everyday
life, and many adolescents already use mobile apps to monitor their lifestyle
behaviors and improve their health. Smartphones may be a valuable platform for
engaging adolescents with interventions to prevent key chronic disease risk
behaviors. Objective: The aim of this paper is to describe the development,
usability, and acceptability of the Health4Life app, a self-monitoring
smartphone app for adolescents that concurrently targets the Big 6 lifestyle
behaviors. Methods: The development of the Health4Life app was an iterative
process conducted in collaboration with adolescents and experts. The
development process consisted of three stages: scoping the literature; end user
consultations, which included a web-based survey (N=815; mean age 13.89, SD
0.89 years) and a focus group (N=12) among adolescents; and app development and
beta testing. Following this development work, 232 adolescents were asked to
rate the usability and acceptability of the app. Results: The process resulted
in a self-monitoring smartphone app that allows adolescent users to track and
set goals for the Big 6 health behaviors, using in-app rewards and
```

notifications to enhance engagement. The overall adolescent feedback was positive in terms of user-friendly design, content, relevance, and helpfulness. Commonly identified areas for improvement were to increase interactive features and display recorded health behaviors differently to improve interpretability. Conclusions: The Health4Life app is a co-designed, self-monitoring smartphone app for adolescents that concurrently targets the Big 6 lifestyle behaviors. Adolescents rated the app as highly acceptable and usable. The app has the potential to efficiently and effectively modify important risk factors for chronic disease among young people and is currently being evaluated in a world-first trial of 6640 secondary school students in 71 schools across Australia. © 2021 Louise Thornton, Lauren Anne Gardner, Bridie Osman, Olivia Green, Katrina Elizabeth Champion, Zachary Bryant, Maree Teesson, Frances Kay-Lambkin, Cath Chapman, The Health4Life Team.},
author_keywords={Adolescents; Chronic disease; Health promotion; Mhealth; Mobile phone},
references={(2014) Australia's health 2014. The 14th biennial health report of the Australian Institute of Health and Welfare, , https://www.aihw.gov.au/getmedia/d2946c3e-9b94-413c-898c-aa5219903b8c/16507.pdf.aspx?inline=true, Australian Institute of Health and Welfare (AIHW). [accessed 2021-03-18]; Wilcox, S., (2015) Chronic diseases in Australia: blueprint for preventive action, , https://www.vu.edu.au/sites/default/files/AHPC/pdfs/Chronic-Diseases-in-Australia-Blueprint-for-preventive-action.pdf, Mitchell Institute and Australian Health Policy Collaboration. [accessed 2021-03-18]; de la Haye, K, D'Amico, EJ, Miles, JNV, Ewing, B, Tucker, JS., Covariance among multiple health risk behaviors in adolescents (2014) PLoS One, 9 (5). , [FREE Full text] [doi] [Medline: 24858838]; Ezzati, M, Riboli, E., Behavioral and dietary risk factors for noncommunicable diseases (2013) N Engl J Med, 369 (10), pp. 954-964. , Sep 05; [doi] [Medline: 24004122]; (2013) Global action plan for the prevention and control of noncommunicable diseases 2013-2020, pp. 1-103. , World Health Organization. Geneva: World Health Organization; Ding, D, Rogers, K, van der Ploeg, H, Stamatakis, E, Bauman, AE., Traditional and emerging lifestyle risk behaviors and all-cause mortality in middle-aged and older adults: evidence from a large population-based Australian cohort (2015) PLoS Med, 12 (12). , Dec 8; [FREE Full text] [doi] [Medline: 26645683]; Lynch, BM, Owen, N., Too much sitting and chronic disease risk: steps to move the science forward (2015) Ann Intern Med, 162 (2), pp. 146-147. , Jan 20; [doi] [Medline: 25599352]; Cappuccio, FP, Cooper, D, D'Elia, L, Strazzullo, P, Miller, MA., Sleep duration predicts cardiovascular outcomes: a systematic review and meta-analysis of prospective studies (2011) Eur Heart J, 32 (12), pp. 1484-1492. , Jun; [doi] [Medline: 21300732]; Prochaska, JJ, Spring, B, Nigg, CR., Multiple health behavior change research: an introduction and overview (2008) Prev Med, 46 (3), pp. 181-188. , Mar; [FREE Full text] [doi] [Medline: 18319098]; Fleig, L, Lippke, S, Pomp, S, Schwarzer, R., Intervention effects of exercise self-regulation on physical exercise and eating fruits and vegetables: a longitudinal study in orthopedic and cardiac rehabilitation (2011) Prev Med, 53 (3), pp. 182-187. , Sep; [FREE Full text] [doi] [Medline: 21784096]; Johnson, SS, Paiva, AL, Mauriello, L, Prochaska, JO, Redding, C, Velicer, WF., Coaction in multiple behavior change interventions: consistency across multiple studies on weight management and obesity prevention (2014) Health Psychol, 33 (5), pp. 475-480. , May; [FREE Full text] [doi] [Medline: 24274806]; Spring, B, Moller, AC, Coons, MJ., Multiple health behaviours: overview and implications (2012) J Public Health (Oxf), 34, pp. 3-10. , Mar; Suppl 1: [FREE Full text] [doi] [Medline: 22363028]; Barratt, MJ, Lenton, S., Beyond recruitment? Participatory

online research with people who use drugs (2010) Int J Internet Res Ethics, 3, pp. 69-86. , [FREE Full text]; (2016) Aussie teens and kids online, , https://apo.org.au/node/61324, Australian Communications and Media Authority. Office of the Children's eSafety Commissioner. [accessed 2021-03-18]; (2016) Australian mobile phone owners aged 14-17, , http://www.roymorgan.com/findings/6929-australian-teenagers-and-their-mobile-phones-june-2016-201608220922, Roy Morgan Research. [accessed 2021-03-18]; Bort-Roig, J, Gilson, ND, Puig-Ribera, A, Contreras, RS, Trost, SG., Measuring and influencing physical activity with smartphone technology: a systematic review (2014) Sports Med, 44 (5), pp. 671-686. , May; [doi] [Medline: 24497157]; Mateo, GF, Granado-Font, E, Ferré-Grau, C, Montaña-Carreras, X., Mobile phone apps to promote weight loss and increase physical activity: a systematic review and meta-analysis (2015) J Med Internet Res, 17 (11), p. e253. , Nov 10; [FREE Full text] [doi] [Medline: 26554314]; Rathbone, AL, Clarry, L, Prescott, J., Assessing the efficacy of mobile health apps using the basic principles of cognitive behavioral therapy: systematic review (2017) J Med Internet Res, 19 (11), p. e399. , Nov 28; [FREE Full text] [doi] [Medline: 29187342]; Schoeppe, S, Alley, S, Van Lippevelde, W, Bray, NA, Williams, SL, Duncan, MJ, Efficacy of interventions that use apps to improve diet, physical activity and sedentary behaviour: a systematic review (2016) Int J Behav Nutr Phys Act, 13 (1), p. 127. , Dec 07; [FREE Full text] [doi] [Medline: 27927218]; Shin, JC, Kim, J, Grigsby-Toussaint, D., Mobile phone interventions for sleep disorders and sleep quality: systematic review (2017) JMIR Mhealth Uhealth, 5 (9), p. e131. , Sep 07; [FREE Full text] [doi] [Medline: 28882808]; Schoeppe, S, Alley, S, Rebar, AL, Hayman, M, Bray, NA, Van Lippevelde, W, Apps to improve diet, physical activity and sedentary behaviour in children and adolescents: a review of quality, features and behaviour change techniques (2017) Int J Behav Nutr Phys Act, 14 (1), p. 83. , Jun 24; [FREE Full text] [doi] [Medline: 28646889]; Quante, M, Khandpur, N, Kontos, EZ, Bakker, JP, Owens, JA, Redline, S., A qualitative assessment of the acceptability of smartphone applications for improving sleep behaviors in low-income and minority adolescents (2019) Behav Sleep Med, 17 (5), pp. 573-585. , Feb 05; [FREE Full text] [doi] [Medline: 29400557]; Wang, K, Varma, DS, Prosperi, M., A systematic review of the effectiveness of mobile apps for monitoring and management of mental health symptoms or disorders (2018) J Psychiatr Res, 107, pp. 73-78. , Dec;: [doi] [Medline: 30347316]; Smith, JJ, Morgan, PJ, Plotnikoff, RC, Dally, KA, Salmon, J, Okely, AD, Smart-phone obesity prevention trial for adolescent boys in low-income communities: the ATLAS RCT (2014) Pediatrics, 134 (3), pp. 723-731. , Sep; [doi] [Medline: 25157000]; Quelly, SB, Norris, AE, DiPietro, JL., Impact of mobile apps to combat obesity in children and adolescents: a systematic literature review (2016) J Spec Pediatr Nurs, 21 (1), pp. 5-17. , Jan; [FREE Full text] [doi] [Medline: 26494019]; Dute, DJ, Bemelmans, WJE, Breda, J., Using mobile apps to promote a healthy lifestyle among adolescents and students: a review of the theoretical basis and lessons learned (2016) JMIR Mhealth Uhealth, 4 (2), p. e39. , May 05; [FREE Full text] [doi] [Medline: 27150850]; Brouwer, W, Kroeze, W, Crutzen, R, de Nooijer, J, de Vries, NK, Brug, J, Which intervention characteristics are related to more exposure to internet-delivered healthy lifestyle promotion interventions? A systematic review (2011) J Med Internet Res, 13 (1), p. e2. , Jan 06; [FREE Full text] [doi] [Medline: 21212045]; Champion, KE, Newton, NC, Spring, B, Wafford, QE, Parmenter, BJ, Teesson, M., A systematic review of school-based eHealth interventions targeting alcohol use, smoking, physical inactivity, diet, sedentary behaviour and sleep among adolescents: a review protocol (2017) Syst Rev, 6 (1), p. 246. , Dec 06; [FREE Full text] [doi] [Medline: 29208040];

Newton, NC, Teesson, M, Vogl, LE, Andrews, G., Internet-based prevention for alcohol and cannabis use: final results of the Climate Schools course (2010) Addiction, 105 (4), pp. 749-759. , Apr; [doi] [Medline: 20148791]; Newton, NC, Vogl, LE, Teesson, M, Andrews, G., CLIMATE Schools: alcohol module: cross-validation of a school-based prevention programme for alcohol misuse (2009) Aust N Z J Psychiatry, 43 (3), pp. 201-207. , Mar; [doi] [Medline: 19221908]; Teesson, M, Newton, NC, Slade, T, Carragher, N, Barrett, EL, Champion, KE, Combined universal and selective prevention for adolescent alcohol use: a cluster randomized controlled trial (2017) Psychol Med, 47 (10), pp. 1761-1770. , Jul; [doi] [Medline: 28222825]; Kelman, HC., Compliance, identification, and internalization three processes of attitude change (2016) J Confl Resolut, 2 (1), pp. 51-60. , Jul; [doi]; Champion, KE, Gardner, LA, McGowan, C, Chapman, C, Thornton, L, Parmenter, B, A web-based intervention to prevent multiple chronic disease risk factors among adolescents: co-design and user testing of the Health4Life school-based program (2020) JMIR Form Res, 4 (7), p. e19485. , Health4Life Team Jul 28; [FREE Full text] [doi] [Medline: 32720898]; Brannon, EE, Cushing, CC., A systematic review: is there an app for that? Translational science of pediatric behavior change for physical activity and dietary interventions (2015) J Pediatr Psychol, 40 (4), pp. 373-384. , May; [doi] [Medline: 25502745]; Ward, MC, White, DT, Druss, BG., A meta-review of lifestyle interventions for cardiovascular risk factors in the general medical population (2015) J Clin Psychiatry, 76, pp. 477-486. , Apr 22;(04): [doi]; Norris, SL, Engelgau, MM, Narayan, KM., Effectiveness of self-management training in type 2 diabetes: a systematic review of randomized controlled trials (2001) Diabetes Care, 24 (3), pp. 561-587. , Mar 01; [doi] [Medline: 11289485]; Burke, LE, Wang, J, Sevick, MA., Self-monitoring in weight loss: a systematic review of the literature (2011) J Am Diet Assoc, 111 (1), pp. 92-102. , Jan; [FREE Full text] [doi] [Medline: 21185970]; Jenkins, RJ, McAlaney, J, McCambridge, J., Corrigendum to "Change over time in alcohol consumption in control groups in brief intervention studies: Systematic review and meta-regression study" [Drug Alcohol Depend. 100 (2009) 107-114] Drug Alcohol Depend, 108 (1-2), p. 151. , 2010 Apr; [doi]; Swendeman, D, Ramanathan, N, Baetscher, L, Medich, M, Scheffler, A, Comulada, W., Smartphone self-monitoring to support self-management among people living with HIV: perceived benefits and theory of change from a mixed-methods randomized pilot study (2015) J Acquir Immune Defic Syndr, 69, pp. 80-91. , (Suppl 1): [doi]; Thornton, L, Kay-Lambkin, F, Tebbutt, B, Hanstock, TL, Baker, AL., A mobile phone-based healthy lifestyle monitoring tool for people with mental health problems (myhealthpa): development and pilot testing (2018) JMIR Cardio, 2 (2), p. e10228. , Oct 01; [FREE Full text] [doi] [Medline: 31758772]; Rotondi, AJ, Eack, SM, Hanusa, BH, Spring, MB, Haas, GL., Critical design elements of e-health applications for users with severe mental illness: singular focus, simple architecture, prominent contents, explicit navigation, and inclusive hyperlinks (2015) Schizophr Bull, 41 (2), pp. 440-448. , Mar; [FREE Full text] [doi] [Medline: 24375458]; Teesson, M, Newton, NC, Slade, T, Chapman, C, Birrell, L, Mewton, L, Combined prevention for substance use, depression, and anxiety in adolescence: a cluster-randomised controlled trial of a digital online intervention (2020) Lancet Digit Health, 2 (2), pp. 74-84. , Feb; [doi]; Teesson, M, Champion, KE, Newton, NC, Kay-Lambkin, F, Chapman, C, Thornton, L, Study protocol of the Health4Life initiative: a cluster randomised controlled trial of an eHealth school-based program targeting multiple lifestyle risk behaviours among young Australians (2020) BMJ Open, 10 (7), p. e035662. , Health4Life Team. Jul 13; [FREE Full text] [doi] [Medline: 32665344]; Rose, T, Barker, M, Jacob, CM, Morrison, L, Lawrence, W, Strömmer, S, A systematic

review of digital interventions for improving the diet and physical activity behaviors of adolescents (2017) J Adolesc Health, 61 (6), pp. 669-677. , Dec; [FREE Full text] [doi] [Medline: 28822682]; Willmott, TJ, Pang, B, Rundle-Thiele, S, Badejo, A., Weight management in young adults: systematic review of electronic health intervention components and outcomes (2019) J Med Internet Res, 21 (2), p. e10265. , Feb 06; [FREE Full text] [doi] [Medline: 30724736]; Shilts, MK, Horowitz, M, Townsend, MS., An innovative approach to goal setting for adolescents: guided goal setting (2004) J Nutr Educ Behav, 36 (3), pp. 155-156. , May; [doi]; McEwan, D, Harden, SM, Zumbo, BD, Sylvester, BD, Kaulius, M, Ruissen, GR, The effectiveness of multi-component goal setting interventions for changing physical activity behaviour: a systematic review and meta-analysis (2016) Health Psychol Rev, 10 (1), pp. 67-88. , Nov 13; [doi] [Medline: 26445201]; Shilts, MK, Horowitz, M, Townsend, MS., Guided goal setting: effectiveness in a dietary and physical activity intervention with low-income adolescents (2009) Int J Adolesc Med Health, 21 (1), pp. 111-122. , [doi] [Medline: 19526701]; Hsu, MSH, Rouf, A, Allman-Farinelli, M., Effectiveness and behavioral mechanisms of social media interventions for positive nutrition behaviors in adolescents: a systematic review (2018) J Adolesc Health, 63 (5), pp. 531-545. , Nov; [doi] [Medline: 30197198]; Sawyer, A, Lewthwaite, H, Gucciardi, DF, Hill, K, Jenkins, S, Cavalheri, V., Behaviour change techniques to optimise participation in physical activity or exercise in adolescents and young adults with chronic cardiorespiratory conditions: a systematic review (2019) Intern Med J, 49 (10), pp. 1209-1220. , Oct; [doi] [Medline: 30324769]; Ashton, LM, Sharkey, T, Whatnall, MC, Williams, RL, Bezzina, A, Aguiar, EJ, Effectiveness of interventions and behaviour change techniques for improving dietary intake in young adults: a systematic review and meta-analysis of RCTs (2019) Nutrients, 11 (4), p. 825. , Apr 11; [FREE Full text] [doi] [Medline: 30979065]; Morrison, LG, Hargood, C, Pejovic, V, Geraghty, AWA, Lloyd, S, Goodman, N, Correction: the effect of timing and frequency of push notifications on usage of a smartphone-based stress management intervention: an exploratory trial (2018) PLoS One, 13 (5), p. e0169162. , May 22; [FREE Full text] [doi] [Medline: 29787600]; Freyne, J, Yin, J, Brindal, E, Hendrie, GA, Berkovsky, S, Noakes, M., Push notifications in diet apps: influencing engagement times and tasks (2017) Int J Hum-comput Int, , Jan 11. [doi]; Alkhaldi, G, Hamilton, FL, Lau, R, Webster, R, Michie, S, Murray, E., The effectiveness of prompts to promote engagement with digital interventions: a systematic review (2016) J Med Internet Res, 18 (1), p. e6. , Jan 08; [FREE Full text] [doi] [Medline: 26747176]; Bidargaddi, N, Pituch, T, Maaieh, H, Short, C, Strecher, V., Predicting which type of push notification content motivates users to engage in a self-monitoring app (2018) Prev Med Rep, 11, pp. 267-273. , Sep;: [FREE Full text] [doi] [Medline: 30109172]; Partridge, SR, Redfern, J., Strategies to engage adolescents in digital health interventions for obesity prevention and management (2018) Healthcare (Basel), 6 (3), p. 70. , Jun 21; [FREE Full text] [doi] [Medline: 29933550]; Torous, J, Nicholas, J, Larsen, ME, Firth, J, Christensen, H., Clinical review of user engagement with mental health smartphone apps: evidence, theory and improvements (2018) Evid Based Ment Health, 21 (3), pp. 116-119. , Aug 05; [doi] [Medline: 29871870]; Zichermann, G, Cunningham, C., (2011) Gamification by design: implementing game mechanics in web and mobile apps, , Sebastopol, California: O'Reilly; Krikelas, J., Information-seeking behavior: patterns and concepts (1983) Drexel Library Q, 19 (2), pp. 5-20. , [FREE Full text]; Werbach, K, Hunter, D., (2012) For the win: how game thinking can revolutionize your business, , Pennsylvania, United States: Wharton Digital Press; Rabbi, M, Philyaw-Kotov, M, Lee, J, Mansour, A, Dent, L, Wang, X, SARA: a mobile app to

engage users in health data collection (2017) Proc ACM Int Conf Ubiquitous Comput, 2017, pp. 781-789. , Sep;: [FREE Full text] [doi] [Medline: 29503985]; Vogl, LE, Teesson, M, Newton, NC, Andrews, G., Developing a school-based drug prevention program to overcome barriers to effective program implementation: The CLIMATE schools: alcohol module (2012) Open J Prev Med, pp. 410-422. , 02(03): [doi]; Smith, A., Americans and their cell phones (2011) Pew Research Center, ,
https://www.pewresearch.org/internet/wp-content/uploads/sites/9/media/Files/Reports/2011/Cell-Phones-2011.pdf, [accessed 2021-03-18]; Thomas, DR., A general inductive approach for analyzing qualitative evaluation data (2016) Am J Eval, 27 (2), pp. 237-246. , Jun 30; [doi]; (2013) Guide to understanding 2013 Index of Community Socio-educational Advantage (ICSEA) values, ,
https://docs.acara.edu.au/resources/Guide-to-understanding-2013-ICSEA-values.pdf, ACARA. Australian Curriculum Assessment and Reporting Authority. [accessed 2021-03-18]; Brooke, J., (1996) SUS-A quick and dirty usability scale, , London: Taylor and Francis; Peres, SC, Pham, T, Phillips, R., Validation of the System Usability Scale (SUS): SUS in the wild (2013) Proceedings of the Human Factors and Ergonomics Society Annual Meeting, ,
https://journals.sagepub.com/doi/10.1177/1541931213571043, Sep 30. [accessed 2021-03-18]; Sauro, J., A practical guide to the systems usibility scale, ,
https://measuringu.com/product/susedu/, MeasuringU. [accessed 2021-03-18]; Martin, A, Caon, M, Adorni, F, Andreoni, G, Ascolese, A, Atkinson, S, A mobile phone intervention to improve obesity-related health behaviors of adolescents across Europe: iterative co-design and feasibility study (2020) JMIR Mhealth Uhealth, 8 (3). , Mar 02; [FREE Full text] [doi] [Medline: 32130179]; Kulas, JT, Stachowski, AA., Middle category endorsement in odd-numbered Likert response scales: associated item characteristics, cognitive demands, and preferred meanings (2009) J Res Pers, 43 (3), pp. 489-493. , Jun; [doi]; Alwin, DF., (2007) Margins of error: a study of reliability in survey measurement, , Hoboken, New Jersey: John Wiley & Sons, Inc; Borgers, N, de Leeuw, E, Hox, J., Children as respondents in survey research: cognitive development and response quality 1 (2016) Bull Methodol Sociol, 66 (1), pp. 60-75. , Jul 22; [doi]; Borgers, N, Hox, J, Sikkel, D., Response effects in surveys on children and adolescents: the effect of number of response options, negative wording, and neutral mid-point (2004) Quality & Quantity, 38 (1), pp. 17-33. , Feb; [doi]; Krosnick, JA, Fabrigar, LR., (1997) Designing rating scales for effective measurement in surveys, ,
https://pprg.stanford.edu/wp-content/uploads/1997-Designing-rating-scales-for-effective-measurement-in-surveys.pdf, Hoboken, New Jersey, United States: John Wiley & Sons, Inc; [accessed 2021-03-18]; Vaillancourt, PM., Stability of children's survey responses (1973) Publ Opin Q, pp. 373-387. , [doi]},
correspondence_address1={Gardner, L.A.; The Matilda Centre for Research in Mental Health and Substance Use, 160 City Road, Australia; email: lauren.gardner@sydney.edu.au},
publisher={JMIR Publications Inc.},
issn={2561326X},
language={English},
abbrev_source_title={JMIR Form. Res.},
document_type={Article},
source={Scopus},
}

@ARTICLE{Laranjo2021422,
author={Laranjo, L. and DIng, D. and Heleno, B. and Kocaballi, B. and Quiroz,

J.C. and Tong, H.L. and Chahwan, B. and Neves, A.L. and Gabarron, E. and Dao, K.P. and Rodrigues, D. and Neves, G.C. and Antunes, M.L. and Coiera, E. and Bates, D.W.},
title={Do smartphone applications and activity trackers increase physical activity in adults? Systematic review, meta-analysis and metaregression},
journal={British Journal of Sports Medicine},
year={2021},
volume={55},
number={8},
pages={422-432},
doi={10.1136/bjsports-2020-102892},
note={cited By 2},
url={https://www.scopus.com/inward/record.uri?eid=2-s2.0-85098495097&doi=10.1136%2fbjsports-2020-102892&partnerID=40&md5=dc42313da23b8a118cbcc72523120cfb},
affiliation={Faculty of Medicine and Health, Westmead Applied Research Centre, The University of Sydney, Sydney, NSW, Australia; Centre for Health Informatics - Australian Institute of Health Innovation, Macquarie University, Sydney, NSW, Australia; Faculty of Medicine and Health, The University of Sydney, Sydney, NSW, Australia; CEDOC, Chronic Diseases Research Centre, NOVA Medical School, Faculdade de Ciências Médicas, Lisbon, Portugal; Faculty of Engineering and IT, University of Technology Sydney, Sydney, NSW, Australia; Centre for Big Data Research in Health, University of New South Wales, Sydney, NSW, Australia; Institute of Global Health Innovation, Imperial College London, London, United Kingdom; Norwegian Centre for Integrated Care and Telemedicine, University Hospital of North Norway, Tromso, Norway; Nova Medical School, Universidade Nova de Lisboa, Lisboa, Portugal; Escola Superior Tecnologias da Saude, Instituto Politécnico de Lisboa, Lisboa, Portugal; Division of General Internal Medicine and Primary Care, Brigham and Women's Hospital, Boston, MA, United States},
abstract={Objective To determine the effectiveness of physical activity interventions involving mobile applications (apps) or trackers with automated and continuous self-monitoring and feedback. Design Systematic review and meta-analysis. Data sources PubMed and seven additional databases, from 2007 to 2020. Study selection Randomised controlled trials in adults (18-65 years old) without chronic illness, testing a mobile app or an activity tracker, with any comparison, where the main outcome was a physical activity measure. Independent screening was conducted. Data extraction and synthesis We conducted random effects meta-analysis and all effect sizes were transformed into standardised difference in means (SDM). We conducted exploratory metaregression with continuous and discrete moderators identified as statistically significant in subgroup analyses. Main outcome measures Physical activity: daily step counts, min/week of moderate-to-vigorous physical activity, weekly days exercised, min/week of total physical activity, metabolic equivalents. Results Thirty-five studies met inclusion criteria and 28 were included in the meta-analysis (n=7454 participants, 28% women). The meta-analysis showed a small-to-moderate positive effect on physical activity measures (SDM 0.350, 95% CI 0.236 to 0.465, I 2 =69%, T 2 =0.051) corresponding to 1850 steps per day (95% CI 1247 to 2457). Interventions including text-messaging and personalisation features were significantly more effective in subgroup analyses and metaregression. Conclusion Interventions using apps or trackers seem to be effective in promoting physical activity. Longer studies are needed to assess the impact of different intervention components on long-term engagement and effectiveness. ©},
author_keywords={accelerometer; app; behaviour; effectiveness; physical

activity},
references={Kyu, H.H., Bachman, V.F., Alexander, L.T., Physical activity and risk of breast cancer, colon cancer, diabetes, ischemic heart disease, and ischemic stroke events: Systematic review and dose-response meta-analysis for the global burden of disease study 2013 (2016) BMJ, 354, p. i3857; Arem, H., Moore, S.C., Patel, A., Leisure time physical activity and mortality: A detailed pooled analysis of the dose-response relationship (2015) JAMA Intern Med, 175, pp. 959-967; Lear, S.A., Hu, W., Rangarajan, S., The effect of physical activity on mortality and cardiovascular disease in 130 000 people from 17 high-income, middle-income, and low-income countries: The PURE study (2017) Lancet, 390, pp. 2643-2654; Ekelund, U., Tarp, J., Steene-Johannessen, J., Dose-Response associations between accelerometry measured physical activity and sedentary time and all cause mortality: Systematic review and harmonised meta-analysis (2019) BMJ, 366, p. l4570; Guthold, R., Stevens, G.A., Riley, L.M., Worldwide trends in insufficient physical activity from 2001 to 2016: A pooled analysis of 358 population-based surveys with 1-9 million participants (2018) Lancet Glob Health, 6, pp. 1077-1086; Kohl, H.W., Craig, C.L., Lambert, E.V., The pandemic of physical inactivity: Global action for public health (2012) Lancet, 380, pp. 294-305; Ding, D., Lawson, K.D., Kolbe-Alexander, T.L., The economic burden of physical inactivity: A global analysis of major non-communicable diseases (2016) Lancet, 388, pp. 1311-1324; Michie, S., Richardson, M., Johnston, M., The behavior change technique taxonomy (V1) of 93 hierarchically clustered techniques: Building an international consensus for the reporting of behavior change interventions (2013) Ann Behav Med, 46, pp. 81-95; Michie, S., Abraham, C., Whittington, C., Effective techniques in healthy eating and physical activity interventions: A meta-regression (2009) Health Psychol, 28, pp. 690-701; Kang, M., Marshall, S.J., Barreira, T.V., Effect of pedometer-based physical activity interventions: A meta-analysis (2009) Res Q Exerc Sport, 80, pp. 648-655; Bravata, D.M., Smith-Spangler, C., Sundaram, V., Using pedometers to increase physical activity and improve health: A systematic review (2007) JAMA, 298, pp. 2296-2304; Sim, I., Mobile devices and health (2019) N Engl J Med, 381, pp. 956-968; Simblett, S., Greer, B., Matcham, F., Barriers to and facilitators of engagement with remote measurement technology for managing health: Systematic review and content analysis of findings (2018) J Med Internet Res, 20, p. 10480; Ledger, D., McCaffrey, D., Inside Wearables: How the science of human behavior change offers the secret to long-term engagement (2014) Endeavour Partners LLC; Tudor-Locke, C., Lutes, L., Why do pedometers work?: A reflection upon the factors related to successfully increasing physical activity (2009) Sports Med, 39, pp. 981-993; Higgins, J.P., Smartphone applications for patients' health and fitness (2016) Am J Med, 129, pp. 11-19; Cotton, V., Patel, M.S., Gamification use and design in popular health and fitness mobile applications (2019) Am J Health Promot, 33, pp. 448-451; Davergne, T., Pallot, A., Dechartres, A., Use of wearable activity Trackers to improve physical activity behavior in patients with rheumatic and musculoskeletal diseases: A systematic review and meta-analysis (2019) Arthritis Care Res, 71, pp. 758-767; Braakhuis, H.E.M., Berger, M.A.M., Bussmann, J.B.J., Effectiveness of healthcare interventions using objective feedback on physical activity: A systematic review and meta-analysis (2019) J Rehabil Med, 51, pp. 151-159; Hodkinson, A., Kontopantelis, E., Adeniji, C., Accelerometer-and Pedometer-Based physical activity interventions among adults with cardiometabolic conditions: A systematic review and meta-analysis (2019) JAMA Netw Open, 2, p. 1912895; Kirk, M.A., Amiri, M., Pirbaglou, M., Wearable technology and physical activity behavior change in adults with chronic cardiometabolic disease: A systematic

review and meta-analysis (2019) Am J Health Promot, 33, pp. 778-791; Yerrakalva, D., Yerrakalva, D., Hajna, S., Effects of mobile health APP interventions on sedentary time, physical activity, and fitness in older adults: Systematic review and meta-analysis (2019) J Med Internet Res, 21, p. 14343; Oliveira, S., Sherrington, C., Ryz, E., Effect of interventions using physical activity trackers on physical activity in people aged 60 years and over: A systematic review and meta-analysis (2019) Br J Sports Med; Romeo, A., Edney, S., Plotnikoff, R., Can smartphone Apps increase physical activity? (2019) Systematic Review and Meta-analysis. J Med Internet Res, 21, p. 12053; Feter, N., Dos Santos, T.S., Caputo, E.L., What is the role of smartphones on physical activity promotion? (2019) A Systematic Review and Meta-analysis. Int J Public Health, 64, pp. 679-690; Flores Mateo, G., Granado-Font, E., Ferré-Grau, C., Mobile phone Apps to promote weight loss and increase physical activity: A systematic review and meta-analysis (2015) J Med Internet Res, 17, p. 253; Zh, L., Lyons, E.J., Jarvis, J.M., Using an electronic activity monitor system as an intervention modality: A systematic review (2015) BMC Public Health, 15, p. 585; Gal, R., May, A.M., Van Overmeeren, E.J., The effect of physical activity interventions comprising Wearables and smartphone applications on physical activity: A systematic review and meta-analysis (2018) Sports Med Open, 4, p. 42; Lynch, C., Bird, S., Lythgo, N., Changing the physical activity behavior of adults with fitness Trackers: A systematic review and meta-analysis (2020) Am J Health Promot, 34, pp. 418-430; Brickwood, K.-J., Watson, G., O'Brien, J., Consumer-Based wearable activity Trackers increase physical activity participation: Systematic review and meta-analysis (2019) JMIR Mhealth Uhealth, 7, p. 11819; Liberati, A., Altman, D.G., Tetzlaff, J., The PRISMA statement for reporting systematic reviews and meta-analyses of studies that evaluate healthcare interventions: Explanation and elaboration (2009) BMJ, 339, p. b2700; Higgins, J., Green, S., (2011) Cochrane Handbook for Systematic Reviews of Interventions, , Chichester: John Wiley & Sons; Higgins, J.P.T., Altman, D.G., Gøtzsche, P.C., The Cochrane collaboration's tool for assessing risk of bias in randomised trials (2011) BMJ, 343, p. d5928; Cohen, J., Statistical power analysis for the behavioral sciences (2013) Routledge, , ISBN: 1134742703; Borenstein, M., Hedges, L.V., Higgins, J.P., Introduction to meta-analysis (2011) John Wiley & Sons, , ISBN: 1119964377; Guyatt, G.H., Oxman, A.D., Vist, G.E., Grade: An emerging consensus on rating quality of evidence and strength of recommendations (2008) BMJ, 336, pp. 924-926; Tong, H.L., Coiera, E., Laranjo, L., Using a mobile social networking APP to promote physical activity: A qualitative study of users' perspectives (2018) J Med Internet Res, 20, p. 11439; Wyke, S., Bunn, C., Andersen, E., The effect of a programme to improve men's sedentary time and physical activity: The European fans in training (EuroFIT) randomised controlled trial (2019) PLoS Med, 16, p. 1002736; Difrancisco-Donoghue, J., Jung, M.-K., Stangle, A., Utilizing wearable technology to increase physical activity in future physicians: A randomized trial (2018) Prev Med Rep, 12, pp. 122-127; Pope, Z., (2018) Use of Wearable Technology and Social Media to Improve Physical Activity and Dietary Behaviors among College Students: A 12-week Randomized Pilot Study, , University of Minnesota; Vandelanotte, C., Duncan, M.J., Maher, C.A., The effectiveness of a web-based Computer-Tailored physical activity intervention using Fitbit activity Trackers: Randomized trial (2018) J Med Internet Res, 20, p. 11321; Ashton, L.M., Morgan, P.J., Hutchesson, M.J., Feasibility and preliminary efficacy of the 'HEYMAN' healthy lifestyle program for young men: A pilot randomised controlled trial (2017) Nutr J, 16, p. 2; Brakenridge, C.L., Fjeldsoe, B.S., Young, D.C., Evaluating the effectiveness of organisational-level strategies with or without an activity tracker to reduce

office workers' sitting time: A cluster-randomised trial (2016) Int J Behav Nutr Phys Act, 13, p. 115; Finkelstein, E.A., Haaland, B.A., Bilger, M., Effectiveness of activity trackers with and without incentives to increase physical activity (TRIPPA): A randomised controlled trial (2016) Lancet Diabetes Endocrinol, 4, pp. 983-995; Poirier, J., Bennett, W.L., Jerome, G.J., Effectiveness of an activity Tracker-and Internet-based adaptive walking program for adults: A randomized controlled trial (2016) J Med Internet Res, 18, p. 34; Ashe, M.C., Winters, M., Hoppmann, C.A., "not just another walking program": Everyday Activity Supports You (EASY) model-A randomized pilot study for a parallel randomized controlled trial (2015) Pilot Feasibility Stud, 1, p. 4; Cadmus-Bertram, L.A., Marcus, B.H., Patterson, R.E., Randomized trial of a Fitbit-Based physical activity intervention for women (2015) Am J Prev Med, 49, pp. 414-418; Martin, S.S., Feldman, D.I., Blumenthal, R.S., MActive: A randomized clinical trial of an automated mHealth intervention for physical activity promotion (2015) J Am Heart Assoc, 4; Thorndike, A.N., Mills, S., Sonnenberg, L., Activity monitor intervention to promote physical activity of physicians-in-training: Randomized controlled trial (2014) PLoS One, 9, p. 100251; Patel, M.S., Small, D.S., Harrison, J.D., Effectiveness of behaviorally designed Gamification interventions with social incentives for increasing physical activity among overweight and obese adults across the United States: The step up randomized clinical trial (2019) JAMA Intern Med, 9, pp. 1624-1629; Ellingson, L.D., Lansing, J.E., Deshaw, K.J., Evaluating motivational interviewing and habit formation to enhance the effect of activity Trackers on healthy adults' activity levels: Randomized intervention (2019) JMIR Mhealth Uhealth, 7, p. 10988; Zhang, J., Jemmott Iii, J.B., Mobile app-based small-group physical activity intervention for young African American women: A pilot randomized controlled trial (2019) Prev Sci, 20, pp. 863-872; Patel, M.S., Volpp, K.G., Rosin, R., A randomized, controlled trial of Lottery-Based financial incentives to increase physical activity among overweight and obese adults (2018) Am J Health Promot, 32, pp. 1568-1575; Robinson, S.A., Bisson, A.N., Hughes, M.L., Time for change: Using implementation intentions to promote physical activity in a randomised pilot trial (2019) Psychol Health, 34, pp. 232-254; Fanning, J., Roberts, S., Hillman, C.H., A smartphone "app"-delivered randomized factorial trial targeting physical activity in adults (2017) J Behav Med, 40, pp. 712-729; Patel, M.S., Benjamin, E.J., Volpp, K.G., Effect of a Game-Based intervention designed to enhance social incentives to increase physical activity among families: The be fit randomized clinical trial (2017) JAMA Intern Med, 177, pp. 1586-1593; John, L.K., Milkman, K.L., Gino, F., The role of incentive salience in habit formation (2016) Harvard Business School, pp. 16-90; King, A.C., Hekler, E.B., Grieco, L.A., Effects of three Motivationally targeted mobile device applications on initial physical activity and sedentary behavior change in midlife and older adults: A randomized trial (2016) PLoS One, 11, p. 0156370; Melton, B.F., Buman, M.P., Vogel, R.L., Wearable devices to improve physical activity and sleep: A randomized controlled trial of College-Aged African American women (2016) Journal of Black Studies, 47, pp. 610-625; Patel, M.S., Asch, D.A., Rosin, R., Individual versus team-based financial incentives to increase physical activity: A randomized, controlled trial (2016) J Gen Intern Med, 31, pp. 746-754; Patel, M.S., Volpp, K.G., Rosin, R., A randomized trial of social comparison feedback and financial incentives to increase physical activity (2016) Am J Health Promot, 30, pp. 416-424; Walsh, J.C., Corbett, T., Hogan, M., An mHealth intervention using a smartphone APP to increase walking behavior in young adults: A pilot study (2016) JMIR Mhealth Uhealth, 4, p. 109; Cowdery, J., Majeske, P., Frank, R., Exergame Apps and physical activity: The results of the ZOMBIE trial (2015) Am

J Health Educ, 46, pp. 216-222; Wang, J.B., Cadmus-Bertram, L.A., Natarajan, L., Wearable Sensor/Device (Fitbit one) and SMS Text-Messaging prompts to increase physical activity in overweight and obese adults: A randomized controlled trial (2015) Telemed J e Health, 21, pp. 782-792; Glynn, L.G., Hayes, P.S., Casey, M., Effectiveness of a smartphone application to promote physical activity in primary care: The smart move randomised controlled trial (2014) Br J Gen Pract, 64, pp. 384-391; Guyatt, G.H., Oxman, A.D., Vist, G.E., Grade: An emerging consensus on rating quality of evidence and strength of recommendations (2008) BMJ, 336, pp. 924-926; Goode, A.P., Hall, K.S., Batch, B.C., The impact of interventions that integrate Accelerometers on physical activity and weight loss: A systematic review (2017) Ann Behav Med, 51, pp. 79-93; Fanning, J., Mullen, S.P., McAuley, E., Increasing physical activity with mobile devices: A meta-analysis (2012) J Med Internet Res, 14, p. 161; Foster, C., Richards, J., Thorogood, M., Remote and web 2 (2013) 0 Interventions for Promoting Physical Activity. Cochrane Database Syst Rev, 9, p. Cd010395; Palmer, M., Sutherland, J., Barnard, S., The effectiveness of smoking cessation, physical activity/diet and alcohol reduction interventions delivered by mobile phones for the prevention of non-communicable diseases: A systematic review of randomised controlled trials (2018) PLoS One, 13, p. 0189801; Direito, A., Carraça, E., Rawstorn, J., MHealth technologies to influence physical activity and sedentary behaviors: Behavior change techniques, systematic review and meta-analysis of randomized controlled trials (2017) Ann Behav Med, 51, pp. 226-239; Williams, S.L., French, D.P., What are the most effective intervention techniques for changing physical activity self-efficacy and physical activity behaviour-and are they the same? (2011) Health Educ Res, 26, pp. 308-322; Yardley, L., Spring, B.J., Riper, H., Understanding and promoting effective engagement with digital behavior change interventions (2016) Am J Prev Med, 51, pp. 833-842; McEwan, D., Beauchamp, M.R., Kouvousis, C., Examining the active ingredients of physical activity interventions underpinned by theory versus no stated theory: A meta-analysis (2019) Health Psychol Rev, 13, pp. 1-17; Head, K.J., Noar, S.M., Iannarino, N.T., Efficacy of text messaging-based interventions for health promotion: A meta-analysis (2013) Soc Sci Med, 97, pp. 41-48; Fjeldsoe, B., Neuhaus, M., Winkler, E., Systematic review of maintenance of behavior change following physical activity and dietary interventions (2011) Health Psychol, 30, pp. 99-109; Short, C., Rebar, A., Plotnikoff, R., (2015) Designing Engaging Online Behaviour Change Interventions: A Proposed Model of User Engagement; Eysenbach, G., The law of attrition (2005) J Med Internet Res, 7, p. 11; Tang, M.Y., Smith, D.M., Mc Sharry, J., Behavior change techniques associated with changes in Postintervention and maintained changes in self-efficacy for physical activity: A systematic review with meta-analysis (2019) Ann Behav Med, 53, pp. 801-815; Ogilvie, D., Foster, C.E., Rothnie, H., Interventions to promote walking: Systematic review (2007) BMJ, 334, p. 1204; Krebs, P., Prochaska, J.O., Rossi, J.S., A meta-analysis of computer-tailored interventions for health behavior change (2010) Prev Med, 51, pp. 214-221; Chen, J.H., Asch, S.M., Machine Learning and Prediction in Medicine-Beyond the Peak of Inflated Expectations (2017) N Engl J Med, 376, pp. 2507-2509; Topol, E.J., High-Performance medicine: The convergence of human and artificial intelligence (2019) Nat Med, 25, pp. 44-56; Insel, T.R., Digital phenotyping: Technology for a new science of behavior (2017) JAMA, 318, pp. 1215-1216; Ekelund, U., Tarp, J., Steene-Johannessen, J., Dose-Response associations between accelerometry measured physical activity and sedentary time and all cause mortality: Systematic review and harmonised meta-analysis (2019) BMJ, 366, p. l4570; Lee, I.-M., Shiroma, E.J., Kamada, M., Association of step volume and intensity with

all-cause mortality in older women (2019) JAMA Intern Med, , [Epub ahead of print: 29 May 2019]; (2019) The State of the Smartphone. Deloitte, , Global mobile consumer survey: UK cut | plateauing at the peak; (2018) Deloitte, , Global mobile consumer survey: US edition | a new era in mobile continues; Rodriguez, J.A., Clark, C.R., Bates, D.W., Digital health equity as a necessity in the 21st century cures act era (2020) JAMA, 323, p. 2381; Bates, D.W., Landman, A., Levine, D.M., Health Apps and health policy: What is needed? (2018) JAMA, 320, pp. 1975-1976; Gordon, W.J., Landman, A., Zhang, H., Beyond validation: Getting health apps into clinical practice (2020) NPJ Digit Med, 3, p. 14; Patnode, C.D., Evans, C.V., Senger, C.A., Behavioral counseling to promote a healthful diet and physical activity for cardiovascular disease prevention in adults without known cardiovascular disease risk factors: Updated evidence report and systematic review for the US preventive services Task force (2017) JAMA, 318, pp. 175-193},
correspondence_address1={Laranjo, L.; Faculty of Medicine and Health, Australia; email: liliana.Laranjo@mq.edu.au},
publisher={BMJ Publishing Group},
issn={03063674},
coden={BJSMD},
pubmed_id={33355160},
language={English},
abbrev_source_title={Br. J. Sports Med.},
document_type={Review},
source={Scopus},
}

@ARTICLE{Valle2021,
author={Valle, C.G. and Pinto, B.M. and LaRose, J.G. and Diamond, M. and Horrell, L.N. and Nezami, B.T. and Hatley, K.E. and Coffman, E.M. and Polzien, K. and Hales, D.P. and Deal, A.M. and Rini, C.M. and Rosenstein, D.L. and Tate, D.F.},
title={Promoting physical activity in young adult cancer survivors using mHealth and adaptive tailored feedback strategies: Design of the Improving Physical Activity after Cancer Treatment (IMPACT) randomized controlled trial},
journal={Contemporary Clinical Trials},
year={2021},
volume={103},
doi={10.1016/j.cct.2021.106293},
art_number={106293},
note={cited By 0},
url={https://www.scopus.com/inward/record.uri?eid=2-s2.0-85100489099&doi=10.1016%2fj.cct.2021.106293&partnerID=40&md5=7e4ec60d28113842433d40c2b217d3e4},
affiliation={Department of Nutrition, Gillings School of Global Public Health and School of Medicine, University of North Carolina at Chapel Hill, Chapel Hill, NC, United States; Lineberger Comprehensive Cancer Center, University of North Carolina at Chapel Hill, Chapel Hill, NC, United States; School of Nursing, University of South Carolina, Columbia, SC, United States; Department of Health Behavior and Policy, School of Medicine, Virginia Commonwealth University, Richmond, VA, United States; Department of Health Behavior, Gillings School of Global Public Health, University of North Carolina at Chapel Hill, Chapel Hill, NC, United States; Department of Medical Social Sciences, Feinberg School of Medicine, Robert H. Lurie Comprehensive Cancer Center, Northwestern University, Chicago, IL, United States; Departments of Psychiatry and Medicine, School of Medicine, University of North Carolina at Chapel Hill,

Chapel Hill, NC, United States},
abstract={Introduction: Despite the health benefits of physical activity for cancer survivors, nearly 60% of young adult cancer survivors (YACS) are physically inactive. Few physical activity interventions have been designed specifically for YACS. Purpose: To describe the rationale and design of the IMPACT (IMproving Physical Activity after Cancer Treatment) trial, which tests the efficacy of a theory-based, mobile physical activity intervention for YACS. Methods: A total of 280 physically inactive YACS (diagnosed at ages 18–39) will be randomized to a self-help control or intervention condition. All participants will receive an activity tracker and companion mobile app, cellular-enabled scale, individual videochat session, and access to a Facebook group. Intervention participants will also receive a 6-month mobile intervention based on social cognitive theory, which targets improvements in behavioral capability, self-regulation, self-efficacy, and social support, and incorporates self-regulation strategies and behavior change techniques. The program includes: behavioral lessons; adaptive goal-setting in response to individuals' changing activity patterns; tailored feedback based on objective data and self-report measures; tailored text messages; and Facebook prompts encouraging peer support. Assessments occur at baseline, 3, 6, and 12 months. The primary outcome is total physical activity min/week at 6 months (assessed via accelerometry); secondary outcomes include total physical activity at 12 months, sedentary behavior, weight, and psychosocial measures. Conclusions: IMPACT uniquely focuses on physical activity in YACS using an automated tailored mHealth program. Study findings could result in a high-reach, physical activity intervention for YACS that has potential to be adopted on a larger scale and reduce cancer-related morbidity. ClinicalTrials.gov Identifier: NCT03569605. © 2021 Elsevier Inc.},
author_keywords={Adaptive interventions; Physical activity; Social media; Tailored feedback; Technology; Young adult cancer survivors},
keywords={accelerometry; adult; anthropometry; Article; autoregulation; behavior modification; cancer survivor; cancer therapy; conceptual framework; controlled study; evidence based practice; feedback system; female; human; major clinical study; male; mobile application; peer group; physical activity; questionnaire; randomized controlled trial; sedentary lifestyle; Social Cognitive Theory; social media; social support},
references={Miller, K.D., Siegel, R.L., Lin, C.C., Mariotto, A.B., Kramer, J.L., Rowland, J.H., Cancer treatment and survivorship statistics, 2016 (2016) CA Cancer J. Clin., 66, pp. 271-289; Cancer Facts & Figures, American Cancer Society (2019), https://www.cancer.org/research/cancer-facts-statistics/all-cancer-facts-figures/cancer-facts-figures-2019.html; Tai, E., Buchanan, N., Townsend, J., Fairley, T., Moore, A., Richardson, L.C., Health status of adolescent and young adult cancer survivors (2012) Cancer., 118, pp. 4884-4891; Nass, S.J., Beaupin, L.K., Demark-Wahnefried, W., Fasciano, K., Ganz, P.A., Hayes-Lattin, B., Identifying and addressing the needs of adolescents and young adults with cancer: summary of an Institute of Medicine workshop (2015) Oncologist., 20, pp. 186-195; Department of Health US, Human Services (US HHS) NI of H National Cancer Institute &LIVESTRONGTM Young Adult Alliance. Closing the gap: research and care imperatives for adolescents and young adults with cancer. Report of the Adolescent and Young Adult Oncology Progress Review Group (2006), NIH Publication Bethesda, MD 06–6067; Speck, R.M., Courneya, K.S., Mâsse, L.C., Duval, S., Schmitz, K.H., An update of controlled physical activity trials in cancer survivors: a systematic review and meta-analysis (2010) J. Cancer Surviv., 4, pp. 87-100; Fong, D.Y.T., Ho, J.W.C., Hui, B.P.H., Lee, A.M.,

Macfarlane, D.J., Leung, S.S.K., Physical activity for cancer survivors: meta-analysis of randomised controlled trials (2012) BMJ., 344; Jones, L.W., Liang, Y., Pituskin, E.N., Battaglini, C.L., Scott, J.M., Hornsby, W.E., Effect of exercise training on peak oxygen consumption in patients with cancer: a meta-analysis (2011) Oncologist., 16, pp. 112-120; Schmitz, K.H., Holtzman, J., Courneya, K.S., Mâsse, L.C., Duval, S., Kane, R., Controlled physical activity trials in cancer survivors: a systematic review and meta-analysis (2005) Cancer Epidemiol. Biomark. Prev., 14, pp. 1588-1595; Campbell, K.L., Winters-Stone, K.M., Wiskemann, J., May, A.M., Schwartz, A.L., Courneya, K.S., Exercise guidelines for cancer survivors: consensus statement from international multidisciplinary roundtable (2019) Med. Sci. Sports Exerc., 51, pp. 2375-2390; Craft, L.L., Vaniterson, E.H., Helenowski, I.B., Rademaker, A.W., Courneya, K.S., Exercise effects on depressive symptoms in cancer survivors: a systematic review and meta-analysis (2012) Cancer Epidemiol. Biomark. Prev., 21, pp. 3-19; Demark-Wahnefried, W., Rogers, L.Q., Alfano, C.M., Thomson, C.A., Courneya, K.S., Meyerhardt, J.A., Practical clinical interventions for diet, physical activity, and weight control in cancer survivors (2015) CA Cancer J. Clin., 65, pp. 167-189; Ballard-Barbash, R., Hunsberger, S., Alciati, M.H., Blair, S.N., Goodwin, P.J., McTiernan, A., Physical activity, weight control, and breast cancer risk and survival: clinical trial rationale and design considerations (2009) J. Natl. Cancer Inst., 101, pp. 630-643; Rabin, C., Politi, M., Need for health behavior interventions for young adult cancer survivors (2010) Am. J. Health Behav., 34, pp. 70-76; Coups, E.J., Ostroff, J.S., A population-based estimate of the prevalence of behavioral risk factors among adult cancer survivors and noncancer controls (2005) Prev. Med., 40, pp. 702-711; Phillips-Salimi, C.R., Andrykowski, M.A., Physical and mental health status of female adolescent/young adult survivors of breast and gynecological cancer: a national, population-based, case-control study (2013) Support Care Cancer, 21, pp. 1597-1604; Zebrack, B., Information and service needs for young adult cancer survivors (2009) Support Care Cancer, 17, pp. 349-357; Zebrack, B., Hamilton, R., Wilder Smith, A., Psychosocial outcomes and service use among young adults with cancer. Seminars in oncology (2009) Elsevier, pp. 468-477; Zebrack, B.J., Block, R., Hayes-Lattin, B., Embry, L., Aguilar, C., Meeske, K.A., Psychosocial service use and unmet need among recently diagnosed adolescent and young adult cancer patients (2013) Cancer., 119, pp. 201-214; Keegan, T.H.M., Lichtensztajn, D.Y., Kato, I., Kent, E.E., Wu, X.-C., West, M.M., Unmet adolescent and young adult cancer survivors information and service needs: a population-based cancer registry study (2012) J. Cancer Surviv., 6, pp. 239-250; Murnane, A., Gough, K., Thompson, K., Holland, L., Conyers, R., Adolescents and young adult cancer survivors: exercise habits, quality of life and physical activity preferences (2015) Support Care Cancer, 23, pp. 501-510; Rabin, C., Simpson, N., Morrow, K., Pinto, B., Behavioral and psychosocial program needs of young adult Cancer survivors (2011) Qual. Health Res., 21, p. 796; Bélanger, L.J., Plotnikoff, R.C., Clark, A., Courneya, K.S., A survey of physical activity programming and counseling preferences in young-adult Cancer survivors (2012) Cancer Nurs., 35, p. 48; Valle, C.G., Tate, D.F., Mayer, D.K., Allicock, M., Cai, J., A randomized trial of a Facebook-based physical activity intervention for young adult cancer survivors (2013) J. Cancer Surviv., 7, pp. 355-368; Rabin, C., Dunsiger, S., Ness, K.K., Marcus, B.H., Internet-based physical activity intervention targeting young adult Cancer survivors (2011) J Adolesc Young Adult Oncol., 1, pp. 188-194; Rabin, C., Pinto, B., Fava, J., Randomized trial of a physical activity and meditation intervention for young adult cancer survivors (2016) J Adolesc Young Adult Oncol., 5, pp. 41-47; Bélanger, L.J., Kerry Mummery, W., Clark, A.M., Courneya, K.S., Effects of

targeted print materials on physical activity and quality of life in young adult cancer survivors during and after treatment: an exploratory randomized controlled trial (2014) J. Adolesc. Young Adult Oncol., 3, pp. 83-91; Pugh, G., Hough, R., Gravestock, H., Davies, C., Horder, R., Fisher, A., The development and user evaluation of health behaviour change resources for teenage and young adult Cancer survivors (2019) Res Involv Engagem., 5, p. 9; Devine, K.A., Viola, A.S., Coups, E.J., Wu, Y.P., Digital health interventions for adolescent and young adult cancer survivors (2018) JCO Clin Cancer Inform., 2, pp. 1-15; Harris, P.A., Taylor, R., Thielke, R., Payne, J., Gonzalez, N., Conde, J.G., Research electronic data capture (REDCap)–a metadata-driven methodology and workflow process for providing translational research informatics support (2009) J. Biomed. Inform., 42, pp. 377-381; Harris, P.A., Taylor, R., Minor, B.L., Elliott, V., Fernandez, M., O'Neal, L., The REDCap consortium: Building an international community of software platform partners (2019) J Biomed Inform., 95, p. 103208; Thomas, S., Reading, J., Shephard, R.J., Revision of the physical activity readiness questionnaire (PAR-Q) (1992) Can. J. Sport Sci., 17, p. 338; Bandura, A., Social Foundations of Thought and Action: A Social Cognitive Theory (1986), Prentice Hall; Bandura, A., Social cognitive theory: an agentic perspective (2001) Annu. Rev. Psychol., 52, pp. 1-26; Pinto, B.M., Ciccolo, J.T., Physical activity motivation and cancer survivorship (2011) Physical Activity and Cancer., pp. 367-387; Pinto, B.M., Floyd, A., Methodologic issues in exercise intervention research in oncology (2007) Semin. Oncol. Nurs., 23, pp. 297-304; Stacey, F.G., James, E.L., Chapman, K., Courneya, K.S., Lubans, D.R., A systematic review and meta-analysis of social cognitive theory-based physical activity and/or nutrition behavior change interventions for cancer survivors (2015) J. Cancer Surviv., 9, pp. 305-338; Pinto, B.M., Frierson, G.M., Rabin, C., Trunzo, J.J., Marcus, B.H., Home-based physical activity intervention for breast cancer patients (2005) J. Clin. Oncol., 23, pp. 3577-3587; Rogers, L.Q., Hopkins-Price, P., Vicari, S., Pamenter, R., Courneya, K.S., Markwell, S., A randomized trial to increase physical activity in breast cancer survivors (2009) Med. Sci. Sports Exerc., 41, pp. 935-946; Demark-Wahnefried, W., Clipp, E.C., Lipkus, I.M., Lobach, D., Snyder, D.C., Sloane, R., Main outcomes of the FRESH START trial: a sequentially tailored, diet and exercise mailed print intervention among breast and prostate cancer survivors (2007) J. Clin. Oncol., 25, pp. 2709-2718; Mosher, C.E., Fuemmeler, B.F., Sloane, R., Kraus, W.E., Lobach, D.F., Snyder, D.C., Change in self-efficacy partially mediates the effects of the FRESH START intervention on cancer survivors’ dietary outcomes (2008) Psycho-Oncology., 17, pp. 1014-1023; Mosher, C.E., Lipkus, I., Sloane, R., Snyder, D.C., Lobach, D.F., Demark-Wahnefried, W., Long-term outcomes of the FRESH START trial: exploring the role of self-efficacy in cancer survivors’ maintenance of dietary practices and physical activity (2013) Psycho-Oncology., 22 (4), pp. 876-885; Rabin, C., Pinto, B.M., Frierson, G.M., Mediators of a randomized controlled physical activity intervention for breast cancer survivors (2006) Journal of Sport and Exercise Psychology., 28, p. 269; Valle, C.G., Tate, D.F., Mayer, D.K., Allicock, M., Cai, J., Exploring mediators of physical activity in young adult Cancer survivors: evidence from a randomized trial of a Facebook-based physical activity intervention (2015) J Adolesc Young Adult Oncol., 4, pp. 26-33; Barber, F.D., Social support and physical activity engagement by Cancer survivors (2012) Clin. J. Oncol. Nurs., 16, pp. 84-98; McDonough, M.H., Beselt, L.J., Daun, J.T., Shank, J., Culos-Reed, S.N., Kronlund, L.J., The role of social support in physical activity for cancer survivors: a systematic review (2019) Psychooncology; Valle, C.G., Tate, D.F., Engagement of young adult cancer survivors within a Facebook-based physical activity intervention (2017)

Transl. Behav. Med., 7, pp. 667-679; Wing, R.R., Tate, D.F., Espeland, M.A., Lewis, C.E., LaRose, J.G., Gorin, A.A., Innovative self-regulation strategies to reduce weight gain in young adults: the study of novel approaches to weight gain prevention (SNAP) randomized clinical trial (2016) JAMA Intern. Med., 176, pp. 755-762; Wing, R.R., Tate, D., Espeland, M., Gorin, A., LaRose, J.G., Robichaud, E.F., Weight gain prevention in young adults: design of the study of novel approaches to weight gain prevention (SNAP) randomized controlled trial (2013) BMC Public Health, 13, p. 300; Gokee LaRose, J., Tate, D.F., Gorin, A.A., Wing, R.R., Preventing weight gain in young adults: a randomized controlled pilot study (2010) Am. J. Prev. Med., 39, pp. 63-68; McAlister, A.L., Perry, C.L., Parcel, G.S., How individuals, environments, and health behavior interact (2008) Health Behavior and Health Education: Theory, Research, and Practice, pp. 167-188. , K. Glanz B.K. Rimer K. Viswanath Jossey-Bass San Francisco, CA; Rabin, C., Simpson, N., Morrow, K., Pinto, B., Intervention format and delivery preferences among young adult cancer survivors (2013) Int J Behav Med., 20, pp. 304-310; Michie, S., Richardson, M., Johnston, M., Abraham, C., Francis, J., Hardeman, W., The behavior change technique taxonomy (v1) of 93 hierarchically clustered techniques: building an international consensus for the reporting of behavior change interventions (2013) Ann. Behav. Med., 46, pp. 81-95; Michie, S., Abraham, C., Whittington, C., McAteer, J., Gupta, S., Effective techniques in healthy eating and physical activity interventions: a meta-regression (2009) Health Psychol., 28, pp. 690-701; Finne, E., Glausch, M., Exner, A.-K., Sauzet, O., Stölzel, F., Seidel, N., Behavior change techniques for increasing physical activity in cancer survivors: a systematic review and meta-analysis of randomized controlled trials (2018) Cancer Manag. Res., 10, pp. 5125-5143; Michie, S., Ashford, S., Sniehotta, F.F., Dombrowski, S.U., Bishop, A., French, D.P., A refined taxonomy of behaviour change techniques to help people change their physical activity and healthy eating behaviours: the CALO-RE taxonomy (2011) Psychol. Health, 26, pp. 1479-1498; Olander, E.K., Fletcher, H., Williams, S., Atkinson, L., Turner, A., French, D.P., What are the most effective techniques in changing obese individuals’ physical activity self-efficacy and behaviour: a systematic review and meta-analysis (2013) Int. J. Behav. Nutr. Phys. Act., 10, p. 29; Williams, S.L., French, D.P., What are the most effective intervention techniques for changing physical activity self-efficacy and physical activity behaviour–and are they the same? (2011) Health Educ. Res., 26, pp. 308-322; Thompson, W.G., Kuhle, C.L., Koepp, G.A., McCrady-Spitzer, S.K., Levine, J.A., “Go4Life” exercise counseling, accelerometer feedback, and activity levels in older people (2014) Arch. Gerontol. Geriatr., 58, pp. 314-319; Cadmus-Bertram, L.A., Marcus, B.H., Patterson, R.E., Parker, B.A., Morey, B.L., Randomized trial of a Fitbit-based physical activity intervention for women (2015) Am. J. Prev. Med., 49, pp. 414-418; Danaher, B.G., Seeley, J.R., Methodological issues in research on web-based behavioral interventions (2009) Ann. Behav. Med., 38, pp. 28-39; Glasgow, R.E., eHealth evaluation and dissemination research (2007) Am. J. Prev. Med., 32, pp. S119-S126; Sung, J., Christensen, H.I., Grinter, R.E., Robots in the wild: understanding long-term use (2009) Proceedings of the 4th ACM/IEEE international conference on Human robot interaction - HRI ‘09. New York, p. 45. , ACM Press New York, USA; Shin, G., Feng, Y., Jarrahi, M.H., Gafinowitz, N., Beyond novelty effect: a mixed-methods exploration into the motivation for long-term activity tracker use (2019) JAMIA Open, 2, pp. 62-72; Ferguson, T., Rowlands, A.V., Olds, T., Maher, C., The validity of consumer-level, activity monitors in healthy adults worn in free-living conditions: a cross-sectional study (2015) Int. J. Behav. Nutr. Phys. Act., 12, p. 42; Lewis, C.E., Smith, D.E., Wallace, D.D., Williams, O.D., Bild, D.E.,

Jacobs, D.R., Seven-year trends in body weight and associations with lifestyle and behavioral characteristics in black and white young adults: the CARDIA study (1997) Am. J. Public Health, 87, pp. 635-642; Truesdale, K.P., Stevens, J., Lewis, C.E., Schreiner, P.J., Loria, C.M., Cai, J., Changes in risk factors for cardiovascular disease by baseline weight status in young adults who maintain or gain weight over 15 years: the CARDIA study (2006) Int. J. Obes., 30, pp. 1397-1407; Loprinzi, P.D., Light-intensity physical activity and all-cause mortality (2017) Am. J. Health Promot., 31, pp. 340-342; Wu, Y.P., Yi, J., McClellan, J., Kim, J., Tian, T., Grahmann, B., Barriers and facilitators of healthy diet and exercise among adolescent and young adult cancer survivors: implications for behavioral interventions (2015) J Adolesc Young Adult Oncol., 4, pp. 184-191; Joseph, R.P., Keller, C., Adams, M.A., Ainsworth, B.E., Print versus a culturally-relevant Facebook and text message delivered intervention to promote physical activity in African American women: a randomized pilot trial (2015) BMC Womens Health, 15, p. 30; Ferrer, D.A., Ellis, R., A review of physical activity interventions delivered via facebook (2017) J. Phys. Act. Health, 14, pp. 823-833; Evenson, K.R., Wen, F., Performance of the ActiGraph accelerometer using a national population-based sample of youth and adults (2015) BMC Res Notes., 8, p. 7; Thraen-Borowski, K.M., Trentham-Dietz, A., Edwards, D.F., Koltyn, K.F., Colbert, L.H., Dose-response relationships between physical activity, social participation, and health-related quality of life in colorectal cancer survivors (2013) J. Cancer Surviv., 7, pp. 369-378; Van Roekel, E.H., Bours, M.J.L., Breedveld-Peters, J.J.L., Meijer, K., Kant, I., Van Den Brandt, P.A., Light physical activity is associated with quality of life after colorectal cancer (2015) Med. Sci. Sports Exerc., 47, pp. 2493-2503; Blair, C.K., Morey, M.C., Desmond, R.A., Cohen, H.J., Sloane, R., Snyder, D.C., Light-intensity activity attenuates functional decline in older cancer survivors (2014) Med. Sci. Sports Exerc., 46, pp. 1375-1383; Evenson, K.R., Terry, J.W., Assessment of differing definitions of accelerometer nonwear time (2009) Res. Q. Exerc. Sport, 80, pp. 355-362; Choi, L., Ward, S.C., Schnelle, J.F., Buchowski, M.S., Assessment of wear/nonwear time classification algorithms for triaxial accelerometer (2012) Med. Sci. Sports Exerc., 44, pp. 2009-2016; Choi, L., Liu, Z., Matthews, C.E., Buchowski, M.S., Validation of accelerometer wear and nonwear time classification algorithm (2011) Med. Sci. Sports Exerc., 43, pp. 357-364; Loprinzi, P.D., Smith, B., Comparison between wrist-worn and waist-worn Accelerometry (2017) J. Phys. Act. Health, 14, pp. 539-545; Koster, A., Shiroma, E.J., Caserotti, P., Matthews, C.E., Chen, K.Y., Glynn, N.W., Comparison of sedentary estimates between activPAL and hip- and wrist-worn ActiGraph (2016) Med. Sci. Sports Exerc., 48, pp. 1514-1522; Kamada, M., Shiroma, E.J., Harris, T.B., Lee, I.-M., Comparison of physical activity assessed using hip- and wrist-worn accelerometers (2016) Gait Posture., 44, pp. 23-28; Troiano, R.P., Berrigan, D., Dodd, K.W., Mâsse, L.C., Tilert, T., McDowell, M., Physical activity in the United States measured by accelerometer (2008) Med. Sci. Sports Exerc., 40, pp. 181-188; Godin, G., Shephard, R.J., A simple method to assess exercise behavior in the community (1985) Can J Appl Sport Sci., 10, pp. 141-146; Jacobs, D.R., Ainsworth, B.E., Hartman, T.J., Leon, A.S., A simultaneous evaluation of 10 commonly used physical activity questionnaires (1993) Med. Sci. Sports Exerc., 25, pp. 81-91; Miller, D.J., Freedson, P.S., Kline, G.M., Comparison of activity levels using the Caltrac (R) accelerometer and five questionnaires (1994) Med. Sci. Sports Exerc., 26, p. 376; Godin, G., Jobin, J., Bouillon, J., Assessment of leisure time exercise behavior by self-report: a concurrent validity study (1986) Can J Public Health., 77, pp. 359-362; Swain, C.T.V., Nguyen, N.H., Eagles, T., Vallance,

J.K., Boyle, T., Lahart, I.M., Postdiagnosis sedentary behavior and health outcomes in cancer survivors: a systematic review and meta-analysis (2020) Cancer, 126, pp. 861-869; Rosenberg, D.E., Norman, G.J., Wagner, N., Patrick, K., Calfas, K.J., Sallis, J.F., Reliability and validity of the Sedentary Behavior Questionnaire (SBQ) for adults (2010) J. Phys. Act. Health, 7, pp. 697-705; Centers for Disease Control and Prevention, 2001 Behavioral Risk Factor Surveillance System Questionnaire, Section 13: Demographics, Question 10. (2001), Centers for Disease Control and Prevention; Health Information National Trends Survey, (2007), http://hints.cancer.gov, [cited 1 Jul 2019]. Available:; Fanelli Kuczmarski, M., Kuczmarski, R.J., Najjar, M., Effects of age on validity of self-reported height, weight, and body mass index: findings from the third National Health and nutrition examination survey, 1988–1994 (2001) J. Am. Diet. Assoc., 101, pp. 28-34; Short, C.E., James, E.L., Girgis, A., McElduff, P., Plotnikoff, R.C., Move more for life: the protocol for a randomised efficacy trial of a tailored-print physical activity intervention for post-treatment breast cancer survivors (2012) BMC Cancer, 12, p. 172; Sallis, J.F., Pinski, R.B., Grossman, R.M., Patterson, T.L., Nader, P.R., The development of self-efficacy scales for healthrelated diet and exercise behaviors (1988) Health Educ. Res., 3, pp. 283-292; Rovniak, L.S., Anderson, E.S., Winett, R.A., Stephens, R.S., Social cognitive determinants of physical activity in young adults: a prospective structural equation analysis (2002) Ann. Behav. Med., 24, pp. 149-156; Sallis, J.F., Grossman, R.M., Pinski, R.B., Patterson, T.L., Nader, P.R., The development of scales to measure social support for diet and exercise behaviors (1987) Prev. Med., 16, pp. 825-836; Ware, J.E., Kosinski, M., Turner-Bowker, D.M., Gandek, B., User's Manual for the SF-12v2 Health Survey (2007), Lincoln, RI QualityMetric Incorporated; Cella, D.F., Tulsky, D.S., Gray, G., Sarafian, B., Linn, E., Bonomi, A., The Functional Assessment of Cancer Therapy scale: development and validation of the general measure (1993) J. Clin. Oncol., 11, pp. 570-579; Overcash, J., Extermann, M., Parr, J., Perry, J., Balducci, L., Validity and reliability of the FACT-G scale for use in the older person with cancer (2001) Am. J. Clin. Oncol., 24, p. 591; Winstead-Fry, P., Schultz, A., Psychometric analysis of the Functional Assessment of Cancer Therapy-General (FACT-G) scale in a rural sample (1997) Cancer J. Clin., 79, pp. 2446-2452; McNair, D.M., Heuchert, J.W.P., POMS: Profile of Mood States Technical Update (2006), Multi-Health Systems North Tonawanda, NY; Radloff, L.S., The CES-D scale: a self-report depression scale for research in the general population (1977) Appl. Psychol. Meas., 1, pp. 385-401; Wagner, L.I., Butt, Z., Lai, J., Cella, D., Measuring patient self-reported cognitive function: development of the functional assessment of cancer therapy–cognitive function instrument (2009) J Support Oncol., 7, pp. W32-W39; Grieve, R., Indian, M., Witteveen, K., Anne Tolan, G., Marrington, J., Face-to-face or Facebook: can social connectedness be derived online? (2013) Comput Human Behav., 29, pp. 604-609; Hamilton, J.G., Wu, L.M., Austin, J.E., Valdimarsdottir, H., Basmajian, K., Vu, A., Economic survivorship stress is associated with poor health-related quality of life among distressed survivors of hematopoietic stem cell transplantation (2013) Psychooncology., 22, pp. 911-921; Scherwitz, L., Perkins, L., Chesney, M., Hughes, G., Cook-medley hostility scale and subsets: relationship to demographic and psychosocial characteristics in young adults in the CARDIA study (1991) Psychosom. Med., 53, pp. 36-49; Schmitz, K.H., Courneya, K.S., Matthews, C., American college of sports medicine roundtable on exercise guidelines for Cancer survivors: corrigendum (2011) Med. Sci. Sports Exerc., 43; Martin, C.L., Tate, D.F., Valle, C.G., Nonadherence to daily self-weighing and activity tracking is associated with weight fluctuations among African American breast

cancer survivors (2018) PLoS One, 13; Kramer, J.-N., Künzler, F., Mishra, V., Presset, B., Kotz, D., Smith, S., Investigating intervention components and exploring states of receptivity for a smartphone app to promote physical activity: protocol of a microrandomized trial (2019) JMIR Res Protoc., 8; Ellison, N.B., Steinfield, C., Lampe, C., The benefits of Facebook "friends:" social capital and college students' use of online social network sites (2007) J Comput-Mediat Comm., 12, pp. 1143-1168; Valle, C.G., Deal, A.M., Tate, D.F., Preventing weight gain in African American breast cancer survivors using smart scales and activity trackers: a randomized controlled pilot study (2017) J. Cancer Surviv., 11, pp. 133-148; Facebook, Statement of Rights and Responsibilities. (2018), https://www.facebook.com/terms.php, Facebook; Shrout, P.E., Bolger, N., Mediation in experimental and nonexperimental studies: new procedures and recommendations (2002) Psychol. Methods, 7, pp. 422-445; MacKinnon, D.P., Lockwood, C.M., Hoffman, J.M., West, S.G., Sheets, V., A comparison of methods to test mediation and other intervening variable effects (2002) Psychol. Methods, 7, pp. 83-104; Preacher, K.J., Hayes, A.F., SPSS and SAS procedures for estimating indirect effects in simple mediation models (2004) Behav. Res. Methods Instrum. Comput., 36, pp. 717-731; Maher, C.A., Lewis, L.K., Ferrar, K., Marshall, S., De Bourdeaudhuij, I., Vandelanotte, C., Are health behavior change interventions that use online social networks effective? A systematic review (2014) J Med Internet Res., 16; Laranjo, L., Arguel, A., Neves, A.L., Gallagher, A.M., Kaplan, R., Mortimer, N., The influence of social networking sites on health behavior change: a systematic review and meta-analysis (2015) J. Am. Med. Inform. Assoc., 22, pp. 243-256; Yang, Q., Are social networking sites making health behavior change interventions more effective? A meta-analytic review (2017) J Health Commun., 22, pp. 223-233; Finkelstein, E.A., Haaland, B.A., Bilger, M., Sahasranaman, A., Sloan, R.A., Nang, E.E.K., Effectiveness of activity trackers with and without incentives to increase physical activity (TRIPPA): a randomised controlled trial (2016) Lancet Diabetes Endocrinol., 4, pp. 983-995; Brickwood, K.-J., Watson, G., O'Brien, J., Williams, A.D., Consumer-based wearable activity trackers increase physical activity participation: systematic review and meta-analysis (2019) JMIR Mhealth Uhealth., 7; Lynch, C., Bird, S., Lythgo, N., Selva-Raj, I., Changing the physical activity behavior of adults with fitness trackers: a systematic review and meta-analysis (2019) Am. J. Health Promot., , 890117119895204; Patel, M.S., Asch, D.A., Volpp, K.G., Wearable devices as facilitators, not drivers, of health behavior change (2015) JAMA., 313, pp. 459-460; Chiauzzi, E., Rodarte, C., DasMahapatra, P., Patient-centered activity monitoring in the self-management of chronic health conditions (2015) BMC Med., 13, p. 77; Lyons, E.J., Lewis, Z.H., Mayrsohn, B.G., Rowland, J.L., Behavior change techniques implemented in electronic lifestyle activity monitors: a systematic content analysis (2014) J. Med. Internet Res., 16; Adams, M.A., Hurley, J.C., Todd, M., Bhuiyan, N., Jarrett, C.L., Tucker, W.J., Adaptive goal setting and financial incentives: a 2 × 2 factorial randomized controlled trial to increase adults' physical activity (2017) BMC Public Health, 17, p. 286; Adams, M.A., Sallis, J.F., Norman, G.J., Hovell, M.F., Hekler, E.B., Perata, E., An adaptive physical activity intervention for overweight adults: a randomized controlled trial (2013) PLoS One, 8; https://www.pewinternet.org/fact-sheet/mobile/, Demographics of Mobile Device Ownership and Adoption in the United States | Pew Research Center [Internet]. 12 Jun 2019 [cited 2 Oct 2019]. Available:; Kerr, J., Marinac, C.R., Ellis, K., Godbole, S., Hipp, A., Glanz, K., Comparison of accelerometry methods for estimating physical activity (2017) Med. Sci. Sports Exerc., 49, pp. 617-624},
correspondence_address1={Valle, C.G.; Department of Nutrition, CB #7461, United

```
States; email: carmina.valle@unc.edu},
publisher={Elsevier Inc.},
issn={15517144},
pubmed_id={33515784},
language={English},
abbrev_source_title={Contemp. Clin. Trials},
document_type={Article},
source={Scopus},
}

@ARTICLE{Green2021,
author={Green, J. and Huberty, J. and Puzia, M. and Stecher, C.},
title={The effect of meditation and physical activity on the mental health
impact of covid-19-related stress and attention to news among mobile app users
in the united states: Cross-sectional survey},
journal={JMIR Mental Health},
year={2021},
volume={8},
number={4},
doi={10.2196/28479},
art_number={e28479},
note={cited By 0},
url={https://www.scopus.com/inward/record.uri?eid=2-s2.0-85104133152&doi=10.219
6%2f28479&partnerID=40&md5=00c854ee8cef402702f5fdb1d9debcdd},
affiliation={College of Health Solutions, Arizona State University, Phoenix,
AZ, United States; Behavioral Research and Analytics, LLC, Salt Lake City, UT,
United States},
abstract={Background: The COVID-19 pandemic has been declared an international
public health emergency, and it may have long-lasting effects on people's
mental health. There is a need to identify effective health behaviors to
mitigate the negative mental health impact of COVID-19. Objective: The
objectives of this study were to (1) examine the regional differences in mental
health and COVID-19-related worry, attention to news, and stress, in light of
the state-level prevalence of COVID-19 cases; (2) estimate the associations
between mental health and COVID-19-related worry, attention to news, and stress
and health behavior engagement (ie, physical activity, mindfulness meditation);
and (3) explore the mediating effect of health behavior engagement on the
associations between mental health and COVID-19-related worry, attention to
news, and stress. Methods: A cross-sectional survey was distributed to a sample
of US adult paying subscribers to the Calm app (data were collected from April
22 to June 3, 2020). The survey assessed COVID-19-related worry, attention to
news, and stress; health behavior engagement; and mental health (ie, perceived
stress, posttraumatic stress disorder, and anxiety and depression). Statistical
analyses were performed using R software. Differences in COVID-19-related
worry, attention to news, and stress and mental health by location were
assessed using t tests and chi-square tests. Logistic and ordinary least
squares models were used to regress mental health and health behavior on
COVID-19-related worry, attention to news, and stress; moreover, causal
mediation analysis was used to estimate the significance of the mediation
effects. Results: The median age of the respondents (N=8392) was 47 years (SD
13.8). Participants in the Mid-Atlantic region (New Jersey, New York, and
Pennsylvania) reported higher levels of stress, more severe depression
symptoms, greater worry about COVID-19, paying more attention to
COVID-19-related news, and more stress related to social distancing
```

recommendations than participants living in other regions. The association between worry about COVID-19 and perceived stress was significantly mediated by changes in physical activity (P<.001), strength of meditation habit (P<.001), and stopping meditation (P=.046). The association between worry about COVID-19 and posttraumatic stress disorder symptoms was significantly mediated by changes in physical activity (P<.001) and strength of meditation habit (P<.001). Conclusions: Our findings describe the mental health impact of COVID-19 and outline how continued participation in health behaviors such as physical activity and mindfulness meditation reduce worsening of mental health due to the COVID-19 pandemic. These data have important implications for public health agencies and health organizations to promote the maintenance of health habits to reduce the residual mental health burden of the COVID-19 pandemic. © 2021 Jennifer Green, Jennifer Huberty, Megan Puzia, Chad Stecher.},
author_keywords={Coronavirus; COVID-19; Health behavior; Mental health; MHealth; Mindfulness meditation},
references={Mental health and psychosocial considerations during the COVID-19 outbreak, , https://www.who.int/docs/default-source/coronaviruse/mental-health-considerations.pdf, World Health Organization. 2020 Mar 18. [accessed 2021-04-05]; Fiorillo, A, Gorwood, P., The consequences of the COVID-19 pandemic on mental health and implications for clinical practice (2020) Eur Psychiatry, 63 (1), p. e32. , Apr 01; [FREE Full text] [doi] [Medline: 32234102]; Bao, Y, Sun, Y, Meng, S, Shi, J, Lu, L., nCoV epidemic: address mental health care to empower society (2019) Lancet, 395 (10224), pp. e37-e38. , 2020 Feb 22; [FREE Full text] [doi] [Medline: 32043982]; Rajkumar, RP., COVID-19 and mental health: A review of the existing literature (2020) Asian J Psychiatr, 52, p. 102066. , Aug;: [FREE Full text] [doi] [Medline: 32302935]; Fitzpatrick, KM, Harris, C, Drawve, G., Fear of COVID-19 and the mental health consequences in America (2020) Psychol Trauma, 12 (S1), pp. S17-S21. , Aug; [doi] [Medline: 32496100]; Wang, C, Pan, R, Wan, X, Tan, Y, Xu, L, Ho, CS, Immediate psychological responses and associated factors during the initial stage of the 2019 coronavirus disease (COVID-19) epidemic among the general population in China Int J Environ Res Public Health, 17 (5), p. 1729. , 2020 Mar 06; [FREE Full text] [doi] [Medline: 32155789]; Brooks, SK, Webster, RK, Smith, LE, Woodland, L, Wessely, S, Greenberg, N, The psychological impact of quarantine and how to reduce it: rapid review of the evidence (2020) Lancet, 395 (10227), pp. 912-920. , Mar 14; [FREE Full text] [doi] [Medline: 32112714]; Salisbury, H., Helen Salisbury: Fear in the time of covid (2020) BMJ, 368, p. m1286. , Mar 30;:. [doi] [Medline: 32229528]; Thombs, B, Tao, L, Wu, Y, Levis, B, Sun, Y, Bourgeault, A, Preliminary COVID-19 Fears Questionnaire: systemic sclerosis and chronic medical conditions versions (2020) OSF Preprints, , Preprint posted on April 6, [doi]; Thombs, BD, Bonardi, O, Rice, DB, Boruff, JT, Azar, M, He, C, Curating evidence on mental health during COVID-19: a living systematic review (2020) J Psychosom Res, 133, p. 110113. , Jun;: [FREE Full text] [doi] [Medline: 32354463]; Holmes, EA, O'Connor, RC, Perry, VH, Tracey, I, Wessely, S, Arseneault, L, Multidisciplinary research priorities for the COVID-19 pandemic: a call for action for mental health science (2020) Lancet Psychiatry, 7 (6), pp. 547-560. , Jun; [FREE Full text] [doi] [Medline: 32304649]; Inkster, B, O'Brien, R, Selby, E, Joshi, S, Subramanian, V, Kadaba, M, Digital health management during and beyond the COVID-19 pandemic: opportunities, barriers, and recommendations (2020) JMIR Ment Health, 7 (7), p. e19246. , Jul 06; [FREE Full text] [doi] [Medline: 32484783]; Lean, M, Fornells-Ambrojo, M, Milton, A, Lloyd-Evans, B, Harrison-Stewart, B, Yesufu-Udechuku, A, Self-management interventions for people with severe mental illness: systematic review and

meta-analysis (2019) Br J Psychiatry, 214 (5), pp. 260-268. , May 22; [FREE Full text] [doi] [Medline: 30898177]; Edenfield, TM, Saeed, SA., An update on mindfulness meditation as a self-help treatment for anxiety and depression (2012) PRBM, p. 131. , Oct:. [doi]; Keng, S, Smoski, MJ, Robins, CJ., Effects of mindfulness on psychological health: a review of empirical studies (2011) Clin Psychol Rev, 31 (6), pp. 1041-1056. , Aug; [FREE Full text] [doi] [Medline: 21802619]; Paluska, SA, Schwenk, TL., Physical activity and mental health: current concepts (2000) Sports Med, 29 (3), pp. 167-180. , Mar; [doi] [Medline: 10739267]; Beaulac, J, Carlson, A, Boyd, RJ., Counseling on physical activity to promote mental health: practical guidelines for family physicians (2011) Can Fam Physician, 57 (4), pp. 399-401. , Apr; [FREE Full text] [Medline: 21490348]; Saxena, S, Van Ommeren, M, Tang, KC, Armstrong, TP., Mental health benefits of physical activity (2009) J Ment Health, 14 (5), pp. 445-451. , Jul 06; [doi]; (1999), https://www.cdc.gov/nccdphp/sgr/index.htm, Physical activity and health: a report of the Surgeon General. US Centers for Disease Control and Prevention. [accessed 2021-04-06]; Hall, G, Laddu, DR, Phillips, SA, Lavie, CJ, Arena, R., A tale of two pandemics: how will COVID-19 and global trends in physical inactivity and sedentary behavior affect one another? (2021) Prog Cardiovasc Dis, 64, pp. 108-110. , [FREE Full text] [doi] [Medline: 32277997]; Burtscher, J, Burtscher, M, Millet, GP., (Indoor) isolation, stress, and physical inactivity: vicious circles accelerated by COVID-19? (2020) Scand J Med Sci Sports, 30 (8), pp. 1544-1545. , Aug 06; [FREE Full text] [doi] [Medline: 32374894]; Carmody, J, Baer, RA., Relationships between mindfulness practice and levels of mindfulness, medical and psychological symptoms and well-being in a mindfulness-based stress reduction program (2008) J Behav Med, 31 (1), pp. 23-33. , Feb; [doi] [Medline: 17899351]; Lacaille, J, Sadikaj, G, Nishioka, M, Carrière, K, Flanders, J, Knäuper, B., Daily Mindful Responding Mediates the Effect of Meditation Practice on Stress and Mood: The Role of Practice Duration and Adherence (2018) J Clin Psychol, 74 (1), pp. 109-122. , Jan 31; [doi] [Medline: 28759111]; Wood, W., Habit in Personality and Social Psychology (2017) Pers Soc Psychol Rev, 21 (4), pp. 389-403. , Nov 24; [doi] [Medline: 28737111]; Wood, W, Rünger, D., Psychology of habit (2016) Annu Rev Psychol, 67, pp. 289-314. , [doi] [Medline: 26361052]; Verplanken, B, Orbell, S., Reflections on past behavior: a self-report index of habit strength (2003) J Appl Soc Psychol, 33 (6), p. 1330. , Jun; [doi]; Behan, C., The benefits of meditation and mindfulness practices during times of crisis such as COVID-19 (2020) Ir J Psychol Med, 37 (4), pp. 256-258. , Dec 14; [FREE Full text] [doi] [Medline: 32406348]; Torous, J, Jän Myrick, K, Rauseo-Ricupero, N, Firth, J., Digital mental health and COVID-19: using technology today to accelerate the curve on access and quality tomorrow (2020) JMIR Ment Health, 7 (3), p. e18848. , Mar 26; [FREE Full text] [doi] [Medline: 32213476]; Zhou, X, Snoswell, CL, Harding, LE, Bambling, M, Edirippulige, S, Bai, X, The role of telehealth in reducing the mental health burden from COVID-19 (2020) Telemed J E Health, 26 (4), pp. 377-379. , Apr 23; [FREE Full text] [doi] [Medline: 32202977]; Huberty, J, Green, J, Glissmann, C, Larkey, L, Puzia, M, Lee, C., Efficacy of the mindfulness meditation mobile app "Calm" to reduce stress among college students: randomized controlled trial (2019) JMIR mHealth uHealth, 7 (6), p. e14273. , Jun 25; [FREE Full text] [doi] [Medline: 31237569]; Yang, E, Schamber, E, Meyer, RML, Gold, JI., Happier healers: randomized controlled trial of mobile mindfulness for stress management (2018) J Altern Complement Med, 24 (5), pp. 505-513. , May; [doi] [Medline: 29420050]; Economides, M, Martman, J, Bell, MJ, Sanderson, B., Improvements in stress, affect, and irritability following brief use of a mindfulness-based smartphone app: a randomized controlled trial (2018)

Mindfulness (N Y), 9 (5), pp. 1584-1593. , Mar 1; [FREE Full text] [doi] [Medline: 30294390]; Puzia, ME, Huberty, J, Eckert, R, Larkey, L, Mesa, R., Associations between global mental health and response to an app-based meditation intervention in myeloproliferative neoplasm patients (2020) Integr Cancer Ther, 19, p. 1534735420927780. , Jun 20;: [FREE Full text] [doi] [Medline: 32564631]; Huberty, J, Eckert, R, Larkey, L, Kurka, J, Rodríguez De Jesús, SA, Yoo, W, Smartphone-based meditation for myeloproliferative neoplasm patients: feasibility study to inform future trials (2019) JMIR Form Res, 3 (2), p. e12662. , Apr 29; [FREE Full text] [doi] [Medline: 31033443]; OSF, , https://osf.io/dsgrv/, Calm COVID-19 survey. [accessed 2021-04-06]; Form: CDC COVID-19 community survey question bank (DRAFT), , https://cde.nlm.nih.gov/formView?tinyId=Kcceysolt, US National Library of Medicine. [accessed 2021-04-06]; Vartti, A, Oenema, A, Schreck, M, Uutela, A, de Zwart, O, Brug, J, SARS knowledge, perceptions, and behaviors: a comparison between Finns and the Dutch during the SARS outbreak in 2003 (2009) Int J Behav Med, 16 (1), pp. 41-48. , Jan 31; [FREE Full text] [doi] [Medline: 19184625]; (2020) US General Services Administration, , https://catalog.data.gov/dataset/zip-code-data, ZIP code data. [accessed 2020-01-06]; US Census Bureau, , https://www2.census.gov/geo/pdfs/maps-data/maps/reference/us-regdiv.pdf, Census Regions and Divisions of the United States. [accessed 2021-04-06]; GitHub, , https://github.com/CSSEGISandData/COVID-19/tree/master/csse-covid-19-data/csse-covid-19-daily-reports-us, csse-covid-19-daily-reports-us. [accessed 2021-04-06]; Gardner, B, Abraham, C, Lally, P, de Bruijn, G., Towards parsimony in habit measurement: testing the convergent and predictive validity of an automaticity subscale of the Self-Report Habit Index (2012) Int J Behav Nutr Phys Act, 9, p. 102. , Aug 30;: [FREE Full text] [doi] [Medline: 22935297]; Cohen, S, Kamarck, T, Mermelstein, R., A global measure of perceived stress (1983) J Health Soc Behav, 24 (4), p. 385. , Dec; [doi]; Cohen, S., Perceived stress in a probability sample of the United States (1988) The Claremont Symposium on Applied Social Psychology. The Social Psychology of Health, pp. 31-67. , Spacapan S, Oskamp S, editors. Thousand Oaks, CA: Sage Publications; Lee, E., Review of the psychometric evidence of the perceived stress scale (2012) Asian Nurs Res (Korean Soc Nurs Sci), 6 (4), pp. 121-127. , Dec; [FREE Full text] [doi] [Medline: 25031113]; Hosey, MM, Leoutsakos, JS, Li, X, Dinglas, VD, Bienvenu, OJ, Parker, AM, Screening for posttraumatic stress disorder in ARDS survivors: validation of the Impact of Event Scale-6 (IES-6) (2019) Crit Care, 23 (1), p. 276. , Aug 07; [FREE Full text] [doi] [Medline: 31391069]; Zigmond, AS, Snaith, RP., The hospital anxiety and depression scale (1983) Acta Psychiatr Scand, 67 (6), pp. 361-370. , Jun; [doi] [Medline: 6880820]; The R Project for statistical computing, , https://www.r-project.org/, [accessed 2021-04-06]; Banerjee, M, Cavanagh, K, Strauss, C., Barriers to mindfulness: a path analytic model exploring the role of rumination and worry in predicting psychological and physical engagement in an online mindfulness-based intervention (2018) Mindfulness (N Y), 9 (3), pp. 980-992. , [FREE Full text] [doi] [Medline: 29875884]; Stanton, R, To, QG, Khalesi, S, Williams, SL, Alley, SJ, Thwaite, TL, Depression, anxiety and stress during COVID-19: associations with changes in physical activity, sleep, tobacco and alcohol use in Australian adults (2020) Int J Environ Res Public Health, 17 (11), p. 4065. , Jun 07; [FREE Full text] [doi] [Medline: 32517294]; Schuch, FB, Bulzing, RA, Meyer, J, Vancampfort, D, Firth, J, Stubbs, B, Associations of moderate to vigorous physical activity and sedentary behavior with depressive and anxiety symptoms in self-isolating people during the COVID-19 pandemic: a cross-sectional survey in Brazil (2020) Psychiatry Res,

292, p. 113339. , Oct;: [FREE Full text] [doi] [Medline: 32745795]; Constandt, B, Thibaut, E, De Bosscher, V, Scheerder, J, Ricour, M, Willem, A., Exercising in times of lockdown: an analysis of the impact of COVID-19 on levels and patterns of exercise among adults in Belgium (2020) Int J Environ Res Public Health, 17 (11), p. 4144. , Jun 10; [FREE Full text] [doi] [Medline: 32532013]; Ammar, A, Brach, M, Trabelsi, K, Chtourou, H, Boukhris, O, Masmoudi, L, Effects of COVID-19 home confinement on eating behaviour and physical activity: results of the ECLB-COVID19 international online survey (2020) Nutrients, 12 (6), p. 1583. , May 28; [FREE Full text] [doi] [Medline: 32481594]; Almandoz, JP, Xie, L, Schellinger, JN, Mathew, MS, Gazda, C, Ofori, A, Impact of COVID-19 stay-at-home orders on weight-related behaviours among patients with obesity (2020) Clin Obes, 10 (5), p. e12386. , Oct; [FREE Full text] [doi] [Medline: 32515555]; Shechter, A, Diaz, F, Moise, N, Anstey, DE, Ye, S, Agarwal, S, Psychological distress, coping behaviors, and preferences for support among New York healthcare workers during the COVID-19 pandemic (2020) Gen Hosp Psychiatry, 66, pp. 1-8. , Sep;: [FREE Full text] [doi] [Medline: 32590254]; Nelson, BW, Pettitt, A, Flannery, JE, Allen, NB., Rapid assessment of psychological and epidemiological correlates of COVID-19 concern, financial strain, and health-related behavior change in a large online sample (2020) PLoS One, 15 (11), p. e0241990. , [FREE Full text] [doi] [Medline: 33175882]; Cheval, B, Sivaramakrishnan, H, Maltagliati, S, Fessler, L, Forestier, C, Sarrazin, P, Relationships between changes in self-reported physical activity, sedentary behaviour and health during the coronavirus (COVID-19) pandemic in France and Switzerland (2021) J Sports Sci, 39 (6), pp. 699-704. , Mar; [doi] [Medline: 33118469]; https://www.cdc.gov/coronavirus/2019-ncov/daily-life-coping/managing-stress-anxiety.html, COVID-19: coping with stress. US Centers for Disease Control and Prevention. [accessed 2021-03-23]; Boyd, JE, Lanius, RA, McKinnon, MC., Mindfulness-based treatments for posttraumatic stress disorder: a review of the treatment literature and neurobiological evidence (2018) J Psychiatry Neurosci, 43 (1), pp. 7-25. , Jan 01; [doi]; Hegberg, NJ, Hayes, JP, Hayes, SM., Exercise intervention in PTSD: a narrative review and rationale for implementation (2019) Front Psychiatry, 10, p. 133. , Mar 21;: [FREE Full text] [doi] [Medline: 30949075]; Penedo, FJ, Dahn, JR., Exercise and well-being: a review of mental and physical health benefits associated with physical activity (2005) Curr Opin Psychiatry, 18 (2), pp. 189-193. , Mar; [doi] [Medline: 16639173]; Rebar, AL, Stanton, R, Geard, D, Short, C, Duncan, MJ, Vandelanotte, C., A meta-meta-analysis of the effect of physical activity on depression and anxiety in non-clinical adult populations (2015) Health Psychol Rev, 9 (3), pp. 366-378. , Jul 03; [doi] [Medline: 25739893]; Warburton, DER, Nicol, CW, Bredin, SSD., Health benefits of physical activity: the evidence (2006) CMAJ, 174 (6), pp. 801-809. , Mar 14; [FREE Full text] [doi] [Medline: 16534088]; Nieman, DC, Wentz, LM., The compelling link between physical activity and the body's defense system (2019) J Sport Health Sci, 8 (3), pp. 201-217. , May; [FREE Full text] [doi] [Medline: 31193280]; Morey, JN, Boggero, IA, Scott, AB, Segerstrom, SC., Current directions in stress and human immune function (2015) Curr Opin Psychol, 5, pp. 13-17. , Oct 01;: [FREE Full text] [doi] [Medline: 26086030]; Mani, M, Kavanagh, DJ, Hides, L, Stoyanov, SR., Review and evaluation of mindfulness-based iPhone apps (2015) JMIR mHealth uHealth, 3 (3), p. e82. , Aug 19; [FREE Full text] [doi] [Medline: 26290327]; Plaza, I, Demarzo, MMP, Herrera-Mercadal, P, García-Campayo, J., Mindfulness-based mobile applications: literature review and analysis of current features (2013) JMIR mHealth uHealth, 1 (2), p. e24. , Nov 01; [FREE Full text] [doi] [Medline: 25099314]; Fanning, J, Mullen, SP, McAuley, E., Increasing physical activity

with mobile devices: a meta-analysis (2012) J Med Internet Res, 14 (6), p. e161. , Nov 21; [FREE Full text] [doi] [Medline: 23171838]},
correspondence_address1={Huberty, J.; College of Health Solutions, 500 N. 3rd St., United States; email: jeni.green@asu.edu},
publisher={JMIR Publications Inc.},
issn={23687959},
language={English},
abbrev_source_title={JMIR Ment. Heal.},
document_type={Review},
source={Scopus},
}

@ARTICLE{NúñezdeArenas-Arroyo2021776,
author={Núñez de Arenas-Arroyo, S. and Cavero-Redondo, I. and Alvarez-Bueno, C. and Sequí-Domínguez, I. and Reina-Gutiérrez, S. and Martínez-Vizcaíno, V.},
title={Effect of eHealth to increase physical activity in healthy adults over 55 years: A systematic review and meta-analysis},
journal={Scandinavian Journal of Medicine and Science in Sports},
year={2021},
volume={31},
number={4},
pages={776-789},
doi={10.1111/sms.13903},
note={cited By 1},
url={https://www.scopus.com/inward/record.uri?eid=2-s2.0-85097601058&doi=10.1111%2fsms.13903&partnerID=40&md5=d3829bb88926d037ddb66c012be60b52},
affiliation={Health and Social Research Center, Universidad de Castilla- La Mancha, Cuenca, Spain; Universidad Politécnica y artística del Paraguay, Asunción, Paraguay; Facultad de Ciencias de la Salud, Universidad Autónoma de Chile, Talca, Chile},
abstract={To estimate the effect of eHealth interventions on increasing physical activity (PA) in healthy adults over 55 years, a systematic review and meta-analysis was conducted following the Preferred Reporting Items for Systematic Reviews and Meta-Analysis (PRISMA) guidelines. MEDLINE, Cochrane, Web of Science, SPORTDiscus, and Scopus databases were searched, from inception to February 2020, for experimental studies reporting the effect of eHealth interventions on steps/day, daily moderate-to-vigorous physical activity (MVPA min/day), PA min/week, and MVPA min/week among adults over 55 years. The DerSimonian and Laird method was used to compute a pooled effect size (ES) estimate and the respective 95% confidence interval (95% CI). Eighteen studies were included in this meta-analysis with adults whose age ranged from 58 to 74.2 years. The interventions lasted between four and 52 weeks. The ES estimates of eHealth interventions on increasing PA were 0.59 (95% CI: 0.15-1.02) for steps/day, 0.49 (95% CI: 0.17-0.80) for daily MVPA, 0.13 (95% CI: 0.01-0.24) for total weekly PA and 0.31 (95% CI: 0.13-0.48) for weekly MVPA. Considering clinical improvements, the mean change difference estimates were an increase of 1616.28 steps/day (95% CI: 386.25-2846.31), 7.41 minutes of daily MVPA (95% CI: 3.24-11.57), 40.54 minutes of total weekly PA (95% CI: −8.71 to 89.79) and 56.35 minutes of weekly MVPA (95% CI: 17.43-95.27). In conclusion, eHealth interventions are effective in increasing PA levels among adults over 55 years, resulting in increased steps/day, MVPA min/day, PA min/week and MVPA min/week. © 2020 John Wiley & Sons A/S. Published by John Wiley & Sons Ltd},
author_keywords={eHealth; healthy aging; older adults; physical activity;

telemedicine},
keywords={adult; aged; effect size; experimental study; female; healthy aging; human; male; Medline; meta analysis; physical activity; practice guideline; review; Scopus; systematic review; telemedicine; Web of Science},
references={(2015) Informe Mundial Sobre el Envejecimiento y la Salud, , Geneva, Switzerland, World Health Organization; (2015) World population ageing, 1950-2050, 40, pp. 40-1307. , Choice Rev Online, –40-1307; Ekelund, U., Tarp, J., Steene-Johannessen, J., Dose-response associations between accelerometry measured physical activity and sedentary time and all cause mortality: systematic review and harmonised meta-analysis (2019) BMJ, 366, p. l4570; (2010) Global Recommendations on Physical Activity for Health, , Geneva, World Health Organization, 60; Dipietro, L., Campbell, W.W., Buchner, D.M., Physical activity, injurious falls, and physical function in aging: an umbrella review (2019) Med Sci Sports Exerc, 51, pp. 1303-1313; Chase, J.A.D., Interventions to increase physical activity among older adults: a meta-analysis (2015) Gerontologist, 55, pp. 706-718; Im, J.Y., Bang, H.S., Seo, D.Y., The effects of 12 weeks of a combined exercise program on physical function and hormonal status in elderly Korean women (2019) Int J Environ Res Public Health, 16, p. 4196; Gianfredi, V., Blandi, L., Cacitti, S., Depression and objectively measured physical activity: a systematic review and meta-analysis (2020) Int J Environ Res Public Health, 17, p. 3738; McDowell, C.P., Dishman, R.K., Gordon, B.R., Herring, M.P., Physical activity and anxiety: a systematic review and meta-analysis of prospective cohort studies (2019) Am J Prev Med, 57, pp. 545-556; (2016) Global Diffusion of eHealth: Making Universal Health Coverage Achievable, , Geneva, Switzerland, World Health Organization; (2019) WHO Guideline: Recommendations on Digital Interventions for Health System Strengthening, 2, pp. 1-3. , Geneva, Switzerland, World Health Organization; Muellmann, S., Forberger, S., Möllers, T., Bröring, E., Zeeb, H., Pischke, C.R., Effectiveness of eHealth interventions for the promotion of physical activity in older adults: a systematic review (2018) Prev Med, 108, pp. 93-110; Jonkman, N.H., van Schooten, K.S., Maier, A.B., Pijnappels, M., eHealth interventions to promote objectively measured physical activity in community-dwelling older people (2018) Maturitas, 113, pp. 32-39; Elavsky, S., Knapova, L., Klocek, A., Smahel, D., Mobile health interventions for physical activity, sedentary behavior, and sleep in adults aged 50 years and older: a systematic literature review (2019) J Aging Phys Act, 27, pp. 565-593; Buyl, R., Beogo, I., Fobelets, M., e-Health interventions for healthy aging: a systematic review (2020) Syst Rev, 9, p. 128; Norman, G.J., Zabinski, M.F., Adams, M.A., Rosenberg, D.E., Yaroch, A.L., Atienza, A.A., A review of ehealth interventions for physical activity and dietary behavior change (2007) Am J Prev Med, 33, pp. 336-345; (2018) Archive:Internet access and use statistics—households and individuals—statistics explained; Poushter, J., (2016) Smartphone ownership and internet usage continues to climb in emerging economies | Pew Research Center, pp. 1-9. , Pewglobal; (2017) Demographics of mobile device ownership and adoption in the United States | Pew Research Center, , Pew Res Cent., 1; Kwan, R.Y.C., Salihu, D., Lee, P.H., The effect of e-health interventions promoting physical activity in older people: a systematic review and meta-analysis (2020) Eur Rev Aging Phys Act, 17, p. 7; Yerrakalva, D., Yerrakalva, D., Hajna, S., Griffin, S., Effects of mobile health app interventions on sedentary time, physical activity, and fitness in older adults: systematic review and meta-analysis (2019) J Med Internet Res, 21, pp. 1-13; Moher, D., Liberati, A., Tetzlaff, J., Altman, D.G., Preferred reporting items for systematic reviews and meta-analyses: the PRISMA statement

(2009) Ann Intern Med, 151, p. 264; Higgins, J.P.T., Thomas, J., Chandler, J., (2019) Cochrane Handbook for Systematic Reviews of Interventions Version 6.0, p. 649. , Cochrane, p; Sterne, J.A.C., Savović, J., Page, M.J., RoB 2: a revised tool for assessing risk of bias in randomised trials (2019) BMJ, 366, p. 14898; Lachenbruch, P.A., Cohen, J., Statistical power analysis for the behavioral sciences (2nd ed.) (1989) J Am Stat Assoc, 84, p. 1096; Sterne, J.A.C., Egger, M., Smith, G.D., Systematic reviews in health care: investigating and dealing with publication and other biases in meta-analysis (2001) BMJ, 323, pp. 101-105; Ashe, M.C., Winters, M., Hoppmann, C.A., 'Not just another walking program': Everyday Activity Supports You (EASY) model-a randomized pilot study for a parallel randomized controlled trial (2015) Pilot Feasibility Stud, 1, p. 4; Bickmore, T.W., Silliman, R.A., Nelson, K., A randomized controlled trial of an automated exercise coach for older adults (2013) J Am Geriatr Soc, 61, pp. 1676-1683; Irvine, A.B., Gelatt, V.A., Seeley, J.R., Macfarlane, P., Gau, J.M., Web-based intervention to promote physical activity by sedentary older adults: randomized controlled trial (2013) J Med Internet Res, 15; Cadmus-Bertram, L.A., Marcus, B.H., Patterson, R.E., Parker, B.A., Morey, B.L., Randomized trial of a fitbit-based physical activity intervention for women (2015) Am J Prev Med, 49, pp. 414-418; Kim, B.H., Glanz, K., Text messaging to motivate walking in older African Americans: a randomized controlled trial (2013) Am J Prev Med, 44, pp. 71-75; King, A.C., Friedman, R., Marcus, B., Ongoing physical activity advice by humans versus computers: the Community Health Advice by Telephone (CHAT) trial (2007) Health Psychol, 26, pp. 718-727; King, A.C., Ahn, D.K., Oliveira, B.M., Atienza, A.A., Castro, C.M., Gardner, C.D., Promoting physical activity through hand-held computer technology (2008) Am J Prev Med, 34, pp. 138-142; Kolt, G.S., Schofield, G.M., Kerse, N., Garrett, N., Oliver, M., Effect of telephone counseling on physical activity for low-active older people in primary care: a randomized, controlled trial (2007) J Am Geriatr Soc, 55, pp. 986-992; Martin, S.S., Feldman, D.I., Blumenthal, R.S., mActive: a randomized clinical trial of an automated mHealth intervention for physical activity promotion (2015) J Am Heart Assoc, 4. , https://doi.org/10.1161/JAHA.115.002239; Peels, D.A., Bolman, C., Golsteijn, R.H.J., Long-term efficacy of a printed or a Web-based tailored physical activity intervention among older adults (2013) Int J Behav Nutr Phys Act, 10, p. 104; Peels, D.A., van Stralen, M.M., Bolman, C., The differentiated effectiveness of a printed versus a Web-based tailored physical activity intervention among adults aged over 50 (2014) Health Educ Res, 29, pp. 870-882; Pinto, B.M., Goldstein, M.G., Ashba, J., Sciamanna, C.N., Jette, A., Randomized controlled trial of physical activity counseling for older primary care patients (2005) Am J Prev Med, 29, pp. 247-255; Roberts, L.M., Jaeger, B.C., Baptista, L.C., Wearable technology to reduce sedentary behavior and CVD risk in older adults: a pilot randomized clinical trial (2019) Clin Interv Aging, 14, pp. 1817-1828; Rowley, T.W., Lenz, E.K., Swartz, A.M., Miller, N.E., Maeda, H., Strath, S.J., Efficacy of an individually tailored, internet-mediated physical activity intervention in older adults: a randomized controlled trial (2019) J Appl Gerontol, 38, pp. 1011-1022; Suboc, T.B., Strath, S.J., Dharmashankar, K., Relative importance of step count, intensity, and duration on physical activity's impact on vascular structure and function in previously sedentary older adults (2014) J Am Heart Assoc, 3. , https://doi.org/10.1161/JAHA.113.000702; Van Dyck, D., Plaete, J., Cardon, G., Crombez, G., De Bourdeaudhuij, I., Effectiveness of the self-regulation eHealth intervention 'MyPlan1.0'. on physical activity levels of recently retired Belgian adults: a randomized controlled trial (2016) Health Educ Res, 31, pp. 653-664; van Dyck, D., Herman, K., Poppe, L., Crombez, G., de Bourdeaudhuij,

I., Gheysen, F., Results of MYPLAN 2.0 on physical activity in older Belgian adults: randomized controlled trial (2019) J Med Internet Res, 21; van Stralen, M.M., de Vries, H., Bolman, C., Mudde, A.N., Lechner, L., Exploring the efficacy and moderators of two computer-tailored physical activity interventions for older adults: a randomized controlled trial (2010) Ann Behav Med, 39, pp. 139-150; Wijsman, C.A., Westendorp, R.G., Verhagen, E.A., Effects of a web-based intervention on physical activity and metabolism in older adults: randomized controlled trial (2013) J Med Internet Res, 15; (2010) Quality assessment tool for quantitative studies, pp. 1-4. , Effective Public Health Practice Project McMaster University Toronto; Fiuza-Luces, C., Garatachea, N., Berger, N.A., Lucia, A., Exercise is the real polypill (2013) Physiology, 28, pp. 330-358; Conn, V.S., Hafdahl, A.R., Mehr, D.R., Interventions to increase physical activity among healthy adults: meta-analysis of outcomes (2011) Am J Public Health, 101, pp. 751-758; Simoni, J.M., Ronen, K., Aunon, F.M., Health behavior theory to enhance eHealth intervention research in HIV: rationale and review (2018) Curr HIV/AIDS Rep, 15, pp. 423-430; Morrison, L.G., Theory-based strategies for enhancing the impact and usage of digital health behaviour change interventions: a review (2015) Digit Health, 1. , 205520761559533; Amagasa, S., Fukushima, N., Kikuchi, H., Older adults’ daily step counts and time in sedentary behavior and different intensities of physical activity (2020) J Epidemiol; Lee, I.M., Shiroma, E.J., Kamada, M., Bassett, D.R., Matthews, C.E., Buring, J.E., Association of step volume and intensity with all-cause mortality in older women (2019) JAMA Intern Med, 179, pp. 1105-1112; Saint-Maurice, P.F., Troiano, R.P., Bassett, D.R., Association of daily step count and step intensity with mortality among US adults (2020) JAMA, 323, pp. 1151-1160; Cavero-Redondo, I., Tudor-Locke, C., Álvarez-Bueno, C., Cunha, P.G., Aguiar, E.J., Martínez-Vizcaíno, V., Steps per day and arterial stiffness: systematic review and meta-analysis (2019) Hypertension, 73, pp. 350-363; Araiza, P., Hewes, H., Gashetewa, C., Vella, C.A., Burge, M.R., Efficacy of a pedometer-based physical activity program on parameters of diabetes control in type 2 diabetes mellitus (2006) Metabolism, 55, pp. 1382-1387; Glynn, N.W., Gmelin, T., Santanasto, A.J., Impact of baseline fatigue on a physical activity intervention to prevent mobility disability (2020) J Am Geriatr Soc, 68, pp. 619-624; Hupin, D., Roche, F., Gremeaux, V., Even a low-dose of moderate-to-vigorous physical activity reduces mortality by 22% in adults aged ≥60 years: a systematic review and meta-analysis (2015) Br J Sports Med, 49, pp. 1262-1267; Le Masurier, G.C., Tudor-locke, C., Comparison of pedometer and accelerometer accuracy under controlled conditions (2003) Med Sci Sports Exerc, 35, pp. 867-871},
correspondence_address1={Cavero-Redondo, I.; Health and Social Research Center, Spain; email: ivan.cavero@uclm.es; Cavero-Redondo, I.; Universidad Politécnica y artística del ParaguayParaguay; email: ivan.cavero@uclm.es},
publisher={Blackwell Munksgaard},
issn={09057188},
coden={SMSSE},
pubmed_id={33280182},
language={English},
abbrev_source_title={Scand. J. Med. Sci. Sports},
document_type={Review},
source={Scopus},
}

@ARTICLE{Liu2021,
author={Liu, S.},

title={Mobile sensor network and wearable devices application in athlete muscle and physical fitness image monitoring},
journal={Microprocessors and Microsystems},
year={2021},
volume={82},
doi={10.1016/j.micpro.2021.103856},
art_number={103856},
note={cited By 0},
url={https://www.scopus.com/inward/record.uri?eid=2-s2.0-85099164728&doi=10.1016%2fj.micpro.2021.103856&partnerID=40&md5=624e22a91acd613cb69496cbc91bc528},
affiliation={Changsha Medical University, Changsha, Hunan 410219, China},
abstract={Mobile sensors have become a connection with more of the new devices and the fast pace. A new group of these devices is a wearable device, wireless sensor networks, and integrating them into sensors and wearable devices is the concept of bringing a new experience to daily living activities. Potential physiological characteristics and training that may affect the strength will contain considerations for improving the expression of maximal expression of Power and limited time. Various factors and strengths related to profit to achieve exercise capacity and great strength. Strong muscle strength contributes to the overall performance improvement of the relevant players in the time characteristics. In the proposed method using K-Nearest Neighbor (KNN)algorithm are using Sports training is physical strength, skill, tactics, psychology, includes the development of intelligence and ability. Physical training is an important part of the training of competitive sports. Physical conditions may also perform directly affects the players. Depletion might not be necessary for the maximum strength benefit of it is not necessary to increase the maximum muscle strength. In practice, programming can combine heavy chains and light loads with improving board strength and power consumption characteristics. Compared to multiple sets, a single set produces excellent training allowances, but the athlete's training status and response relationships must be considered. © 2021},
author_keywords={Athlete's training; Mobile sensors; Physical conditions; Physiological characteristics},
keywords={Muscle; Nearest neighbor search; Sports; Wireless sensor networks, Competitive sports; Daily living activities; K nearest neighbor algorithm; Mobile sensor networks; Physical conditions; Physical strength; Physiological characteristics; Time characteristics, Wearable sensors},
references={Leilei, W., Huina, C., Physical education image analysis based on virtual crowd simulation and FPGA (2020) Microprocess Microsyst, 79. , ISSN 0141-9331; Zhou, Z., Yu, H., Shi, H., Human activity recognition based on improved bayesian convolution network to analyze health care data using wearable IoT device (2020) IEEE Access, 2020 (99). , 1–1; Nweke, H.F., Teh, Y.W., Mujtaba, G., Al-garadi, M.A., Data fusion and multiple classifier systems for human activity detection and health monitoring: review and open research directions (2019) Inf. Fusion, 46, pp. 147-170; Janidarmian, M., Fekr, A.R., Radecki, K., Zilic, Z., A comprehensive analysis on wearable acceleration sensors in human activity recognition (2017) Sensors, 17 (3), p. 529; Gravina, R., Alinia, P., Ghasemzadeh, H., Fortino, G., 'Multi-sensor fusion in body sensor networks: state-of-the-art and research challenges (2017) Inf. Fusion, 35, pp. 68-80; Mahmud, T., Akash, S.S., Fattah, S.A., Zhu, W.-P., Ahmad, M.O., Human activity recognition from multi-modal wearable sensor data using deep multi-stage LSTM architecture based on temporal feature aggregation (2020) 2020 IEEE 63rd International Midwest Symposium on Circuits and Systems (MWSCAS); Takei, K., Honda, W., Harada, S., Arie, T., Akita, S., Toward flexible and

wearable human-interactive health-monitoring devices (2015) Adv. Healthc.
Mater., 4 (4), pp. 487-500; Xu, S., Assembly of micro/nanomaterials into
complex, three-dimensional architectures by compressive buckling (2015) Science
(80-.), 347 (6218), pp. 154-159; Zhong, Y., Hu, W., The research on
WebGIS-based information integration and data analysis platform for China's
physical fitness and the national fitness program (2016) 2016 2nd IEEE
International Conference on Computer and Communications (ICCC), Chengdu, pp.
135-139; Wang, D.-H., Yuan, H., Xiao-dong, W., Software development of swimming
athletes' physical fitness monitor system (2009) 2009 ISECS International
Colloquium on Computing, Communication, Control, and Management; Mou, Y., Zhou,
L., Chen, W., Zhao, X., Liu, Y., Yang, C., The analysis on college students'
physical fitness testing data — Two cases study (2017) 2017 International
Conference on Security, Pattern Analysis, and Cybernetics (SPAC), Shenzhen, pp.
252-255; Ma, L., Multi-level estimation and fuzzy evaluation of physical
fitness and health effect of college students in regular institutions of higher
learning based on classification and regression tree algorithm (2018) 2018
International Conference on Virtual Reality and Intelligent Systems (ICVRIS),
Changsha, pp. 201-205; Gao, S., Lu, M., Miao, N., Physical fitness clustering
analysis based on self-organizing feature maps network (2018) 2018 4th Annual
International Conference on Network and Information Systems for Computers
(ICNISC), Wuhan, China, pp. 261-264; Kwak, H.-M., Park, S.-H., Yoon, Y.-R., An
integrated system for body shape analysis and physical fitness test -HIMS
(2005) 2005 IEEE Engineering in Medicine and Biology 27th Annual Conference,
Shanghai, pp. 3742-3745; Hsu, C.-T., Chang, Y.-H., Chen, J.-S., Lin, H.-H.,
Chou, J.-Y., Implementation of IoT device on public fitness equipment for
health physical fitness improvement (2020) 2020 International Conference on
Mathematics and Computers in Science and Engineering (MACE), Madrid, Spain, pp.
236-239; Wang, G., Mining analysis for college students' physical performance
and fitness tests (2018) 2018 International Conference on Virtual Reality and
Intelligent Systems (ICVRIS), Changsha, pp. 191-195; Sahoo, S., Tiwari, V.N.,
Narayanan, R., Endurance based personalized fitness planner (2016) 2016 38th
Annual International Conference of the IEEE Engineering in Medicine and Biology
Society (EMBC), Orlando, FL, pp. 5270-5274; Hao, H., Web-based design and
development of undergraduate physical fitness detection system (2010) 2010
International Conference on Artificial Intelligence and Education (ICAIE),
Hangzhou, pp. 765-767; Alqudah, H., Cardiovascular fitness strengthening using
a portable device (2016) 2016 38th Annual International Conference of the IEEE
Engineering in Medicine and Biology Society (EMBC), Orlando, FL, pp. 2692-2695;
Steinmetzer, T., Wilberg, S., Bönninger, I., Travieso, C.M., Analyzing gait
symmetry with automatically synchronized wearable sensors in daily life (2020)
Microprocess Microsyst, 77. , ISSN 0141-9331},
publisher={Elsevier B.V.},
issn={01419331},
coden={MIMID},
language={English},
abbrev_source_title={Microprocessors Microsyst},
document_type={Article},
source={Scopus},
}

@ARTICLE{Jones2021,
author={Jones, R.A. and Lawlor, E.R. and Birch, J.M. and Patel, M.I. and
Werneck, A.O. and Hoare, E. and Griffin, S.J. and van Sluijs, E.M.F. and Sharp,
S.J. and Ahern, A.L.},

title={The impact of adult behavioural weight management interventions on mental health: A systematic review and meta-analysis},
journal={Obesity Reviews},
year={2021},
volume={22},
number={4},
doi={10.1111/obr.13150},
art_number={e13150},
note={cited By 1},
url={https://www.scopus.com/inward/record.uri?eid=2-s2.0-85093980680&doi=10.1111%2fobr.13150&partnerID=40&md5=88fad9fadd04432f9795491372a88d84},
affiliation={MRC Epidemiology Unit, University of Cambridge, Cambridge, United Kingdom; UKCRC Centre for Diet and Activity Research (CEDAR), University of Cambridge, Cambridge, United Kingdom; School of Clinical Medicine, University of Cambridge, Cambridge, United Kingdom; Department of Nutrition, School of Public Health, University de São Paulo (USP), São Paulo, Brazil; Food and Mood Centre, Centre for Innovation in Mental and Physical Health and Clinical Treatment, School of Medicine, Faculty of Health, Deakin University, Melbourne, VIC, Australia; Primary Care Unit, Department of Public Health and Primary Care, University of Cambridge, Cambridge, United Kingdom},
abstract={There is good evidence that behavioural weight management interventions improve physical health; however, the impact on mental health remains unclear. We evaluated the impact of behavioural weight management interventions on mental health-related outcomes in adults with overweight or obesity at intervention-end and 12 months from baseline. Eligible studies were randomized controlled trials (RCTs) or cluster RCTs of adult behavioural weight loss interventions reporting affect, anxiety, binge eating, body image, depression, emotional eating, quality of life, self-esteem and stress. We searched seven databases from inception to 7 May 2019 and included 43 articles reporting 42 RCTs. Eighteen studies were deemed to be at high risk of bias. We conducted random-effects meta-analyses, stratified analyses and meta-regression using Stata. Interventions generated greater improvements than comparators for depression, mental health-related quality of life and self-efficacy at intervention-end and 12 months from baseline. There was no difference between groups for anxiety, overall quality of life, self-esteem or stress at intervention-end. There was insufficient evidence to assess the impact on anxiety, binge eating, body image, emotional eating, affect, life satisfaction, self-esteem or stress at intervention-end and/or 12 months from baseline. Although evidence suggests that interventions benefit some aspects of mental health, high-quality, transparently reported RCTs measuring a range of mental health outcomes over longer durations are required to strengthen the evidence base. © 2020 The Authors. Obesity Reviews published by John Wiley & Sons Ltd on behalf of World Obesity Federation},
author_keywords={interventions; mental health; obesity; weight management},
keywords={adult; adult disease; aged; anxiety disorder; behavior therapy; behavioral weight management; binge eating disorder; body image; body weight management; clinical outcome; depression; distress syndrome; emotional eating; female; human; life satisfaction; male; mental disease; meta analysis; obesity; outcome assessment; quality of life; randomized controlled trial (topic); Review; self concept; self esteem; systematic review},
references={Fang, X., Wei, J., He, X., Quantitative association between body mass index and the risk of cancer: a global meta-analysis of prospective cohort studies (2018) Int J Cancer, 143 (7), pp. 1595-1603. ,

https://doi.org/10.1002/ijc.31553; Obesity and overweight; (2014) Obesity: identification, assessment and management, , https://www.nice.org.uk/guidance/cg189/, Clinical Guideline [CG189]; Flegal, K., Kit, B., Orpana, H., Graubard, B., Association of all-cause mortality with overweight and obesity using standard body mass index categories: a systematic review and meta-analysis (2013) JAMA, 309 (1), pp. 71-82. , https://doi.org/10.1001/jama.2012.113905; Laxy, M., Holle, R., Döring, A., Peters, A., Hunger, M., The longitudinal association between weight change and health-related quality of life: The KORA S4/F4 cohort study (2014) Int J Public Health, 59 (2), pp. 279-288. , https://doi.org/10.1007/s00038-013-0506-x; Williams, E.P., Mesidor, M., Winters, K., Dubbert, P.M., Wyatt, S.B., Overweight and obesity: prevalence, consequences, and causes of a growing public health problem (2015) Curr Obes Rep, 4 (3), pp. 363-370. , https://doi.org/10.1007/s13679-015-0169-4; Rajan, T.M., Menon, V., Psychiatric disorders and obesity: a review of association studies (2017) J Postgrad Med, 63 (3), pp. 182-190. , https://doi.org/10.4103/jpgm.JPGM_712_16; Avila, C., Holloway, A.C., Hahn, M.K., An overview of links between obesity and mental health (2015) Curr Obes Rep, 4 (3), pp. 303-310. , https://doi.org/10.1007/s13679-015-0164-9; Ma, C., Avenell, A., Bolland, M., Effects of weight loss interventions for adults who are obese on mortality, cardiovascular disease, and cancer: systematic review and meta-analysis (2017) BMJ, 359, p. j4849. , https://doi.org/10.1136/bmj.j4849; LeBlanc, E.S., Patnode, C.D., Webber, E.M., Redmond, N., Rushkin, M., O'Connor, E.A., Behavioral and pharmacotherapy weight loss interventions to prevent obesity-related morbidity and mortality in adults updated evidence report and systematic review for the US preventive services task force (2018) JAMA - J Am Med Assoc, 320 (11), pp. 1172-1191. , https://doi.org/10.1001/jama.2018.7777; Alhalel, N., Schueller, S.M., O'Brien, M.J., Association of changes in mental health with weight loss during intensive lifestyle intervention: does the timing matter? (2018) Obes Sci Pract, 4 (2), pp. 153-158. , https://doi.org/10.1002/osp4.157; Webber, K.H., Casey, E.M., Mayes, L., Katsumata, Y., Mellin, L., A comparison of a behavioral weight loss program to a stress management program: a pilot randomized controlled trial (2016) Nutrition, 32 (7-8), pp. 904-909. , https://doi.org/10.1016/j.nut.2016.01.008; Fuller, N.R., Burns, J., Sainsbury, A., Examining the association between depression and obesity during a weight management programme (2017) Clin Obes, 7 (6), pp. 354-359. , https://doi.org/10.1111/cob.12208; Janney, C.A., Greenberg, J.M., Moin, T., Does mental health influence weight loss in adults with prediabetes? Findings from the VA Diabetes Prevention Program (2018) Gen Hosp Psychiatry, 53 (July–August 2018), pp. 32-37. , https://doi.org/10.1016/j.genhosppsych.2018.04.001; Young, M.D., Morgan, P.J., Effect of a gender-tailored ehealth weight loss program on the depressive symptoms of overweight and obese men: pre-post study (2018) J Med Internet Res, 20 (1), pp. 1-7. , https://doi.org/10.2196/mental.8920; Rand, K., Vallis, M., Aston, M., “It is not the diet; it is the mental part we need help with.” A multilevel analysis of psychological, emotional, and social well-being in obesity (2017) Int J Qual Stud Health Well-being, 12 (1), pp. 1-14. , https://doi.org/10.1080/17482631.2017.1306421; Peckmezian, T., Hay, P., A systematic review and narrative synthesis of interventions for uncomplicated obesity: weight loss, well-being and impact on eating disorder (2017) J Eat Disord, 5 (15), pp. 212-213. , https://doi.org/10.1016/j.enfcli.2017.08.004; Walls, H.L., Peeters, A., Proietto, J., McNeil, J.J., Public health campaigns and obesity—a critique (2011) BMC Public Health, 11, pp. 1-7. , https://doi.org/10.1186/1471-2458-11-136; Pearl, R.L., Weight bias and stigma:

public health implications and structural solutions (2018) Soc Issues Policy Rev, 12 (1), pp. 146-182. , https://doi.org/10.1111/sipr.12043; Lazzeretti, L., Assessment of psychological predictors of weight loss: how and what for? (2015) World J Psychiatry, 5 (1), pp. 56-67. , https://doi.org/10.5498/wjp.v5.i1.56; Booth, A.O., Wang, X., Turner, A.I., Nowson, C.A., Torres, S.J., Diet-induced weight loss has no effect on psychological stress in overweight and obese adults: a meta-analysis of randomized controlled trials (2018) Nutrients, 10 (5), p. 613. , https://doi.org/10.3390/nu10050613; Baillot, A., Saunders, S., Brunet, J., Romain, A.J., Trottier, A., Bernard, P., A systematic review and meta-analysis of the effect of exercise on psychosocial outcomes in adults with obesity: a call for more research (2018) Ment Health Phys Act, 14, pp. 1-10. , https://doi.org/10.1016/j.mhpa.2017.12.004; Warkentin, L.M., Das, D., Majumdar, S.R., Johnson, J.A., Padwal, R.S., The effect of weight loss on health-related quality of life: systematic review and meta-analysis of randomized trials (2014) Obes Rev, 15 (3), pp. 169-182. , https://doi.org/10.1111/obr.12113; Lasikiewicz, N., Myrissa, K., Hoyland, A., Lawton, C.L., Psychological benefits of weight loss following behavioural and/or dietary weight loss interventions. A systematic research review (2014) Appetite, 72, pp. 123-137. , https://doi.org/10.1016/j.appet.2013.09.017; Fabricatore, A., Wadden, T., Higginbotham, A., Intentional weight loss and changes in symptoms of depression: a systematic review and meta-analysis (2011) Int J Obes (Lond), 35 (11), pp. 1363-1376. , https://doi.org/10.1038/ijo.2011.2; Blaine, B.E., Rodman, J., Newman, J.M., Weight loss treatment and psychological well-being: a review and meta-analysis (2007) J Health Psychol, 12 (1), pp. 66-82. , https://doi.org/10.1177/1359105307071741; Jacob, A., Moullec, G., Lavoie, K.L., Impact of cognitive-behavioral interventions on weight loss and psychological outcomes: a meta-analysis (2018) Health Psychol, 37 (5), pp. 417-432. , https://doi.org/10.1037/hea0000576; Fruh, S.M., Obesity: risk factors, complications, and strategies for sustainable long-term weight management (2017) J Am Assoc Nurse Pract, 29 (S1), pp. S3-S14. , https://doi.org/10.1002/2327-6924.12510; Moher, D., Liberati, A., Tetzlaff, J., Altman, D.G., Preferred Reporting Items for Systematic Reviews and Meta-Analyses: the PRISMA statement (2009) BMJ, 339 (jul21 1), p. b2535. , https://doi.org/10.1136/bmj.b2535; Jones, R.A., Lawlor, E.R., Griffin, S.J., Van Sluijs, E.M.F., Ahern, A.L., Impact of adult weight management interventions on mental health: a systematic review and meta-analysis protocol (2020) BMJ Open, 10 (1), pp. 1-6. , https://doi.org/10.1136/bmjopen-2019-031857; Hartmann-Boyce, J., Johns, D.J., Jebb, S.A., Summerbell, C., Aveyard, P., Behavioural weight management programmes for adults assessed by trials conducted in everyday contexts: systematic review and meta-analysis (2014) Obes Rev, 15 (11), pp. 920-932. , https://doi.org/10.1111/obr.12220; Tronieri, J.S., Wadden, T.A., Chao, A.M., Tsai, A.G., Primary care interventions for obesity: review of the evidence (2019) Curr Obes Rep, 8 (2), pp. 128-136. , https://doi.org/10.1007/s13679-019-00341-5; Drew, R.J., Morgan, P.J., Pollock, E.R., Young, M.D., Impact of male-only lifestyle interventions on men's mental health: a systematic review and meta-analysis (2020) Obes Rev, 21 (November 2019), pp. 1-16. , https://doi.org/10.1111/obr.13014; Hartmann-Boyce, J., Johns, D.J., Jebb, S.A., Aveyard, P., Effect of behavioural techniques and delivery mode on effectiveness of weight management: systematic review, meta-analysis and meta-regression (2014) Obes Rev, 15 (7), pp. 598-609. , https://doi.org/10.1111/obr.12165; Higgins, J., Thomas, J., Chandler, J., (2019) Cochrane Handbook for Systematic Reviews of Interventions Version 6.0 [Updated July 2019], , www.training.cochrane.org/handbook, eds., The Cochrane

Collaboration; (2019) Stata Statistical Software: Release 16, , College Station, TX, StataCorp LLC; Ahern, A.L., Wheeler, G.M., Aveyard, P., Extended and standard duration weight-loss programme referrals for adults in primary care (WRAP): a randomised controlled trial (2017) Lancet, 389 (10085), pp. 2214-2225. , https://doi.org/10.1016/s0140-6736(17)30647-5; Alfaris, N., Wadden, T.A., Sarwer, D.B., Effects of a 2-year behavioral weight loss intervention on sleep and mood in obese individuals treated in primary care practice (2015) Obesity, 23 (3), pp. 558-564. , https://doi.org/10.1002/oby.20996; Damschroder, L.J., Lutes, L.D., Kirsh, S., Small-changes obesity treatment among veterans: 12-month outcomes (2014) Am J Prev Med, 47 (5), pp. 541-553. , https://doi.org/10.1016/j.amepre.2014.06.016; Domene, P.A., Moir, H.J., Pummell, E., Knox, A., Easton, C., The health-enhancing efficacy of Zumba® fitness: an 8-week randomised controlled study (2016) J Sports Sci, 34 (15), pp. 1396-1404. , https://doi.org/10.1080/02640414.2015.1112022; Godino, J.G., Merchant, G., Norman, G.J., Using social and mobile tools for weight loss in overweight and obese young adults (Project SMART): a 2-year parallel group randomized controlled trial (2016) Lancet Diabetes Endocrinol, 4 (9), pp. 747-755. , https://doi.org/10.1016/S2213-8587(16)30105-X; Gray, C.M., Hunt, K., Mutrie, N., Anderson, A.S., Treweek, S., Wyke, S., Weight management for overweight and obese men delivered through professional football clubs: a pilot randomized trial (2013) Int J Behav Nutr Phys Act, 10 (121), pp. 1-17; Haapala, I., Barengo, N.C., Biggs, S., Surakka, L., Manninen, P., Weight loss by mobile phone: a 1-year effectiveness study (2009) Public Health Nutr, 12 (12), pp. 2382-2391. , https://doi.org/10.1017/S1368980009005230; Herring, L.Y., Wagstaff, C., Scott, A., The efficacy of 12 weeks supervised exercise in obesity management (2014) Clin Obes, 4 (4), pp. 220-227. , https://doi.org/10.1111/cob.12063; Huber, J.M., Shapiro, J.S., Wieland, M.L., Telecoaching plus a portion control plate for weight care management: a randomized trial (2015) Trials, 16 (323), pp. 1-9. , https://doi.org/10.1186/s13063-015-0880-1; Imayama, I., Alfano, C.M., Kong, A., Dietary weight loss and exercise interventions effects on quality of life in overweight/obese postmenopausal women: a randomized controlled trial (2011) Int J Behav Nutr Phys Act, 8 (1), p. 118. , https://doi.org/10.1186/1479-5868-8-118; Jamal, S.N., Moy, F.M., Mohamed, M.N.A., Mukhtar, F., Effectiveness of a group support lifestyle modification (GSLiM) programme among obese adults in workplace: a randomised controlled trial (2016) PLoS ONE, 11 (8), pp. 1-22. , https://doi.org/10.1371/journal.pone.0160343; Jane, M., Foster, J., Hagger, M., Ho, S., Kane, R., Pal, S., Psychological effects of belonging to a Facebook weight management group in overweight and obese adults: results of a randomised controlled trial (2018) Health Soc Care Community, 26, pp. 714-724. , https://doi.org/10.1111/hsc.12584; Ash, S., Reeves, M., Bauer, J., A randomised control trial comparing lifestyle groups, individual counselling and written information in the management of weight and health outcomes over 12 months (2006) Int J Obes (Lond), 30 (10), pp. 1557-1564. , https://doi.org/10.1038/sj.ijo.0803263; Kalarchian, M.A., Marcus, M.D., Courcoulas, A.P., Cheng, Y., Levine, M.D., Preoperative lifestyle intervention in bariatric surgery: Initial results from a randomized, controlled trial (2013) Obesity, 21 (2), pp. 254-260. , https://doi.org/10.1002/oby.20069; Kraschnewski, J.L., Stuckey, H.L., Rovniak, L.S., Efficacy of a weight-loss website based on positive deviance: a randomized trial (2011) Am J Prev Med, 41 (6), pp. 610-614. , https://doi.org/10.1016/j.amepre.2011.08.012; Laing, B.Y., Mangione, C.M., Tseng, C.H., Effectiveness of a smartphone application for

weight loss compared with usual care in overweight primary care patients (2014) Ann Intern Med, 161 (10), pp. S5-S12. , https://doi.org/10.7326/M13-3005; McRobbie, H., Hajek, P., Peerbux, S., Tackling obesity in areas of high social deprivation: clinical effectiveness and cost-effectiveness of a task-based weight management group programme—a randomised controlled trial and economic evaluation (2016) Health Technol Assess (Rockv), 20 (79), pp. 1-150. , https://doi.org/10.3310/hta20790; Morgan, P.J., Collins, C.E., Plotnikoff, R.C., Efficacy of a workplace-based weight loss program for overweight male shift workers: the Workplace POWER (Preventing Obesity Without Eating like a Rabbit) randomized controlled trial (2011) Prev Med (Baltim), 52 (5), pp. 317-325. , https://doi.org/10.1016/j.ypmed.2011.01.031; Morgan, P.J., Collins, C.E., Plotnikoff, R.C., The impact of a workplace-based weight loss program on work-related outcomes in overweight male shift workers (2012) J Occup Environ Med, 54 (2), pp. 122-127. , https://doi.org/10.1097/JOM.0b013e31824329ab; Morgan, P.J., Callister, R., Collins, C.E., The SHED-IT community trial: a randomized controlled trial of internet- and paper-based weight loss programs tailored for overweight and obese men (2013) Ann Behav Med, 45 (2), pp. 139-152. , https://doi.org/10.1007/s12160-012-9424-z; Nanchahal, K., Townsend, J., Letley, L., Haslam, D., Wellings, K., Haines, A., Weight-management interventions in primary care: a pilot randomised controlled trial (2009) Br J Gen Pract, 59 (562), pp. e157-e166. , https://doi.org/10.3399/bjgp09X420617; Nurkkala, M., Kaikkonen, K., Vanhala, M.L., Karhunen, L., Keränen, A.M., Korpelainen, R., Lifestyle intervention has a beneficial effect on eating behavior and long-term weight loss in obese adults (2015) Eat Behav, 18, pp. 179-185. , https://doi.org/10.1016/j.eatbeh.2015.05.009; O'Brien, T., Jenkins, C., Amella, E., Mueller, M., Moore, M., Hathaway, D., An internet-assisted weight loss intervention for older overweight and obese rural women: a feasibility study (2016) CIN - Comput Informatics Nurs, 34 (11), pp. 513-519. , https://doi.org/10.1097/CIN.0000000000000275; Astbury, N.M., Aveyard, P., Nickless, A., Doctor referral of overweight people to low energy total diet replacement treatment (DROPLET): pragmatic randomised controlled trial (2018) BMJ, 362, pp. 1-10. , https://doi.org/10.1136/bmj.k3760; Petrella, R.J., Gill, D.P., Zou, G., Hockey fans in training: a pilot pragmatic randomized controlled trial (2017) Med Sci Sports Exerc, 49 (12), pp. 2506-2516. , https://doi.org/10.1249/MSS.0000000000001380; Rshikesan, P.B., Subramanya, P., Nidhi, R., Yoga practice for reducing the male obesity and weight related psychological difficulties—a randomized controlled trial (2016) J Clin Diagn Res, 10 (11), pp. 22-28. , https://doi.org/10.7860/JCDR/2016/22720.8940; Rubin, R.R., Peyrot, M., Wang, N.Y., Patient-reported outcomes in the practice-based opportunities for weight reduction (POWER) trial (2013) Qual Life Res, 22 (9), pp. 2389-2398. , https://doi.org/10.1007/s11136-013-0363-3; Samuel-Hodge, C.D., Johnston, L.F., Gizlice, Z., Randomized trial of a behavioral weight loss intervention for low-income women: the weight wise program (2009) Obesity, 17 (10), pp. 1891-1899. , https://doi.org/10.1038/oby.2009.128; Scholz, U., Ochsner, S., Luszczynska, A., Comparing different boosters of planning interventions on changes in fat consumption in overweight and obese individuals: a randomised controlled trial (2013) Int J Psychol, 48 (4), pp. 604-615; Steinberg, D.M., Tate, D.F., Bennett, G.G., Ennett, S., Samuel-Hodge, C., Ward, D.S., Daily self-weighing and adverse psychological outcomes: a randomized controlled trial (2014) Am J Prev Med, 46 (1), pp. 24-29. , https://doi.org/10.1016/j.amepre.2013.08.006; Tapsell, L.C., Lonergan, M., Batterham, M.J., Effect of interdisciplinary care on weight loss: a randomised controlled trial (2017) BMJ Open, 7 (e014533), pp. 1-12. , https://doi.org/10.1136/bmjopen-2016-014533; Teixeira, P.J., Silva, M.N.,

Coutinho, S.R., Mediators of weight loss and weight loss maintenance in middle-aged women (2010) Obesity, 18 (4), pp. 725-735. , https://doi.org/10.1038/oby.2009.281; White, C., Drummond, S., De Looy, A., Comparing advice to decrease both dietary fat and sucrose, or dietary fat only, on weight loss, weight maintenance and perceived quality of life (2010) Int J Food Sci Nutr, 61 (3), pp. 282-294. , https://doi.org/10.3109/09637480903397355; Xenaki, N., Bacopoulou, F., Kokkinos, A., Nicolaides, N.C., Chrousos, G.P., Darviri, C., Impact of a stress management program on weight loss, mental health and lifestyle in adults with obesity: a randomized controlled trial (2018) J Mol Biochem, 7 (2), pp. 78-84; Barnes, R.D., White, M.A., Martino, S., Grilo, C.M., A randomized controlled trial comparing scalable weight loss treatments in primary care (2014) Obesity, 22 (12), pp. 2508-2516. , https://doi.org/10.1002/oby.20889; Jebb, S.A., Ahern, A.L., Olson, A.D., Primary care referral to a commercial provider for weight loss treatment versus standard care: a randomised controlled trial (2011) Lancet, 378 (9801), pp. 1485-1492. , https://doi.org/10.1016/S0140-6736(11)61344-5; Hunt, K., Wyke, S., Gray, C.M., A gender-sensitised weight loss and healthy living programme for overweight and obese men delivered by Scottish Premier League football clubs (FFIT): a pragmatic randomised controlled trial (2014) Lancet, 383 (9924), pp. 1211-1221. , https://doi.org/10.1016/S0140-6736(13)62420-4; Sellman, D., Schroder, R., Deering, D., Elmslie, J., Foulds, J., Frampton, C., Psychosocial enhancement of the Green Prescription for obesity recovery: a randomised controlled trial (2017) N Z Med J, 130 (1450), pp. 44-54; Berry, D., Colindres, M., Sanchez-Lugo, L., Sanchez, M., Neal, M., Smith-Miller, C., Adapting, feasibility testing, and pilot testing a weight management intervention for recently immigrated Spanish-speaking women and their 2- to 4-year-old children (2011) Hisp Heal Care Int, 9 (4), pp. 186-193. , https://doi.org/10.1891/1540-4153.9.4.186; Camolas, J., Santos, O., Moreira, P., do Carmo, I., INDIVIDUO: results from a patient-centered lifestyle intervention for obesity surgery candidates (2017) Obes Res Clin Pract, 11 (4), pp. 475-488. , https://doi.org/10.1016/j.orcp.2016.08.003; Cleo, G., Glasziou, P., Beller, E., Isenring, E., Thomas, R., Habit-based interventions for weight loss maintenance in adults with overweight and obesity: a randomized controlled trial (2019) Int J Obes (Lond), 43 (2), pp. 374-383. , https://doi.org/10.1038/s41366-018-0067-4; Cramer, H., Thoms, M.S., Anheyer, D., Lauche, R., Dobos, G., Yoga in women with abdominal obesity - a randomized controlled trial (2016) Dtsch Arztebl Int, 113 (39), pp. 645-652. , https://doi.org/10.3238/arztebl.2016.0645; Crane, M.M., Ward, D.S., Lutes, L.D., Bowling, J.M., Tate, D.F., Theoretical and behavioral mediators of a weight loss intervention for men (2016) Ann Behav Med, 50 (3), pp. 460-470. , https://doi.org/10.1007/s12160-016-9774-z; Schulz, K.F., Altman, D.G., Moher, D., CONSORT 2010 Statement: updated guidelines for reporting parallel group randomised trials (2010) BMJ, 8 (18), pp. 1-9; Kane, R.L., Wang, J., Garrard, J., Reporting in randomized clinical trials improved after adoption of the CONSORT statement (2007) J Clin Epidemiol, 60 (3), pp. 241-249. , https://doi.org/10.1016/j.jclinepi.2006.06.016; Williams, R.L., Wood, L.G., Collins, C.E., Callister, R., Effectiveness of weight loss interventions—is there a difference between men and women: a systematic review (2015) Obes Rev, 16 (2), pp. 171-186. , https://doi.org/10.1111/obr.12241; Lakens, D., Hilgard, J., Staaks, J., On the reproducibility of meta-analyses: six practical recommendations (2016) BMC Psychol, 4 (1), pp. 1-10. , https://doi.org/10.1186/s40359-016-0126-3; Pagoto, S.L., Schneider, K.L., Oleski, J.L., Luciani, J.M., Bodenlos, J.S., Whited, M.C., Male inclusion in

randomized controlled trials of lifestyle weight loss interventions (2012) Obesity, 20 (6), pp. 1234-1239. , https://doi.org/10.1038/oby.2011.140; Ford, N.D., Patel, S.A., Narayan, K.M.V., Obesity in low- and middle-income countries: burden, drivers, and emerging challenges (2017) Annu Rev Public Health, 38 (1), pp. 145-164. , https://doi.org/10.1146/annurev-publhealth-031816-044604},
correspondence_address1={Jones, R.A.; MRC Epidemiology Unit, United Kingdom; email: rj397@cam.ac.uk},
publisher={Blackwell Publishing Ltd},
issn={14677881},
coden={ORBEB},
pubmed_id={33103340},
language={English},
abbrev_source_title={Obes. Rev.},
document_type={Review},
source={Scopus},
}

@ARTICLE{Dunn2021279,
author={Dunn, S.L. and Robbins, L.B. and Tintle, N.L. and Collins, E.G. and Bronas, U.G. and Goodyke, M.P. and Luong, A. and Gutierrez-Kapheim, M. and DeVon, H.A.},
title={Heart up! RCT protocol to increase physical activity in cardiac patients who report hopelessness: Amended for the COVID-19 pandemic},
journal={Research in Nursing and Health},
year={2021},
volume={44},
number={2},
pages={279-294},
doi={10.1002/nur.22106},
note={cited By 0},
url={https://www.scopus.com/inward/record.uri?eid=2-s2.0-85099110047&doi=10.1002%2fnur.22106&partnerID=40&md5=fd1f025ee86fd261dcbd21c9a70acc63},
affiliation={Department of Biobehavioral Nursing Science, College of Nursing, University of Illinois at Chicago, Chicago, IL, United States; College of Nursing, Michigan State University, East Lansing, MI, United States; Department of Statistics, Dordt University, Sioux Center, IA, United States; School of Nursing, University of California Los Angeles, Los Angeles, CA, United States},
abstract={Hopelessness is associated with decreased physical activity (PA) and increased adverse events and death in patients with ischemic heart disease (IHD). Rates of PA in patients with IHD continue to be low in both hospital-based cardiac rehabilitation and home settings. While researchers have investigated strategies to increase PA among patients with IHD, interventions to promote PA specifically in IHD patients who report hopelessness are lacking. We describe the protocol for a NIH-funded randomized controlled trial designed to establish the effectiveness of a 6-week intervention (Heart Up!) to promote increased PA in IHD patients who report hopelessness. Participants (n = 225) are randomized to one of three groups: (1) motivational social support (MSS) from a nurse, (2) MSS from a nurse plus significant other support (SOS), or (3) attention control. Aims are to: (1) test the effectiveness of 6 weeks of MSS and MSS with SOS on increasing mean minutes per day of moderate to vigorous PA; (2) determine the effects of change in moderate to vigorous PA on hopelessness; and (3) determine if perceived social support and motivation (exercise self-regulation) mediate the effects of the intervention on PA. A total of 69

participants have been enrolled to date. The protocol has been consistently and accurately used by research personnel. We address the protocol challenges presented by the COVID-19 pandemic and steps taken to maintain fidelity to the intervention. Findings from this study could transform care for IHD patients who report hopelessness by promoting self-management of important PA goals that can contribute to better health outcomes. © 2021 Wiley Periodicals LLC},
author_keywords={cardiac; COVID-19; hopelessness; physical activity; research protocol},
keywords={adult; attitude; exercise; heart muscle ischemia; human; motivation; motivational interviewing; psychology; randomized controlled trial (topic); social support; text messaging, Adult; Attitude; COVID-19; Exercise; Humans; Motivation; Motivational Interviewing; Myocardial Ischemia; Randomized Controlled Trials as Topic; Social Support; Text Messaging},
references={Abramson, L., Metalsky, G., Alloy, L., Hopelessness depression: A theory-based subtype of depression (1989) Psychological Review, 96 (2), pp. 358-372. , https://doi.org/10.1037/0033-295x.96.2.358; (2020), https://www.actigraphcorp.com/research-database/, Actigraph research database; Aliabad, H.O., Vafaeinasab, M., Morowatisharifabad, M.A., Afshani, S.A., Firoozabadi, M.G., Forouzannia, S.K., Maintenance of physical activity and exercise capacity after rehabilitation in coronary heart disease: A randomized controlled trial (2014) Global Journal of Health Science, 6 (6), p. 198. , https://doi.org/10.5539/gjhs.v6n6p198; Anderson, L., Oldridge, N., Thompson, D.R., Zwisler, A.D., Rees, K., Martin, N., Taylor, R.S., Exercise-based cardiac rehabilitation for coronary heart disease: Cochrane systematic review and meta-analysis (2016) Journal of the American College of Cardiology, 67 (1), pp. 1-12. , https://doi.org/10.1016/j.jacc.2015.10.044; Beckie, T.M., Beckstead, J.W., Predicting cardiac rehabilitation attendance in a gender-tailored randomized clinical trial (2010) Journal of Cardiopulmonary Rehabilitation, 30 (3), pp. 147-156. , https://doi.org/10.1097/HCR.0b013e3181d0c2ce; Bellg, A.J., Borrelli, B., Resnick, B., Hecht, J., Minicucci, D.S., Ory, M., Ogedegbe, G., Czajkowski, S., Enhancing treatment fidelity in health behavior change studies: Best practices and recommendations from the NIH Behavior Change Consortium (2004) Health Psychology, 23 (5), pp. 443-451. , https://doi.org/10.1037/0278-6133.23.5.443; Burke, L.E., Ma, J., Azar, K.M.J., Bennett, G.G., Peterson, E.D., Zheng, Y., Riley, W., Quinn, C.C., Current science on consumer use of mobile health for cardiovascular disease prevention: A scientific statement from the American Heart Association (2015) Circulation, 132 (12), pp. 1157-1213. , https://doi.org/10.1161/CIR.0000000000000232; Cella, D., Riley, W., Stone, A., Rothrock, N., Reeve, B., Yount, S., Amtmann, D., Hays, R., The Patient-Reported Outcomes Measurement Information System (PROMIS) developed and tested its first wave of adult self-reported health outcome item banks: 2005–2008 (2010) Journal of Clinical Epidemiology, 63 (11), pp. 1179-1194. , https://doi.org/10.1016/j.jclinepi.2010.04.011; (2020), https://www.cdc.gov/coronavirus/2019-ncov/need-extra-precautions/groups-at-higher-risk.html, Coronavirus disease 2019 (COVID-19); Charlson, M.E., Pompei, P., Ales, K.L., MacKenzie, C.R., A new method of classifying prognostic comorbidity in longitudinal studies: Development and validation (1987) Journal of Chronic Diseases, 40 (5), pp. 373-383. , https://doi.org/10.1016/0021-9681(87)90171-8; Chow, N., Fleming-Dutra, K., Gierke, R., Hall, A., Hughes, M., Pilishvili, T., Ritchey, M., Ussery, E., Preliminary estimates of the prevalence of selected underlying health conditions among patients with coronavirus disease 2019—United States, February 12–March 28, 2020 (2020) Morbidity and Mortality Weekly Report, 69 (13), pp. 382-386. , https://doi.org/10.15585/mmwr.mm6913e2; Chow,

C.K., Redfern, J., Hillis, G.S., Thakkar, J., Santo, K., Hackett, M.L., Jan, S., Thiagalingam, A., Effect of lifestyle-focused text messaging on risk factor modifications in patients with coronary heart disease: A randomized clinical trial (2015) Journal of the American Medical Association, 314 (12), pp. 1255-1263. , https://doi.org/10.1001/jama.2015.10945; Clark, A.M., King-Shier, K.M., Spaling, M.A., Duncan, A.S., Stone, J.A., Jaglal, S.B., Thompson, D.R., Angus, J.E., Factors influencing participation in cardiac rehabilitation programmes after referral and initial attendance: Qualitative systematic review and meta-synthesis (2013) Clinical Rehabilitation, 27 (10), pp. 948-959. , https://doi.org/10.1177/0269215513481046; Cohen, S., Gottlieb, B., Underwood, L., Social relationships and health: Challenges for measurement and intervention (2001) Advances in Mind-Body Medicine, 17 (2), pp. 129-141. , https://doi.org/10.1093/med:psych/9780195126709.001.0001; Dalal, H.M., Zawada, A., Jolly, K., Moxham, T., Taylor, R.S., Home based versus centre based cardiac rehabilitation: Cochrane systematic review and meta-analysis (2010) BMJ, 340. , https://doi.org/10.1136/bmj.b5631; Davis, M., Brennan, J.M., Vish, N., Adams, J., Muldoon, M., Renbarger, T., Garner, J., Lessons learned from study of depression in cardiovascular patients in an acute-care heart and vascular hospital (2013) Proceedings (Baylor University. Medical Center), 26 (1), pp. 6-9. , https://doi.org/10.1080/08998280.2013.11928898; Deci, E.L., Ryan, R.M., Self-Determination Theory: A macrotheory of human motivation, development, and health (2008) Canadian Psychology, 49 (3), pp. 182-185. , https://doi.org/10.1037/a0012801; De Smedt, D., Clays, E., Annemans, L., Doyle, F., Kotseva, K., Pająk, A., Prugger, C., De Bacquer, D., Health related quality of life in coronary patients and its association with their cardiovascular risk profile: Results from the EUROASPIRE III survey (2013) International Journal of Cardiology, 168 (2), pp. 898-903. , https://doi.org/10.1016/j.ijcard.2012.10.053; Dunn, S.L., Corser, W., Stommel, M., Holmes-Rovner, M., Hopelessness and depression in the early recovery period after hospitalization for acute coronary syndrome (2006) Journal of cardiopulmonary rehabilitation and prevention, 26 (3), pp. 153-159. , https://doi.org/10.1097/00008483-200605000-00007; Dunn, S.L., Dunn, L.M., Rieth, N.P., Olamijulo, G.B., Swieringa, L.L., Holden, T.P., Clark, J.A., Tintle, N.L., Impact of home- and hospital-based exercise in cardiac rehabilitation on hopelessness in patients with coronary heart disease (2017) Journal of Cardiopulmonary Rehabilitation and Prevention, 37 (1), pp. 39-48; Dunn, S.L., DeVon, H.A., Collins, E.G., Luong, A.T., Buursma, M., Gutierrez-Kapheim, M., Bronas, U.B., Suicide risk management protocol for a randomized controlled trial of cardiac patients reporting hopelessness (2020) Nursing Research, 70 (1), pp. 72-79. , https://doi.org/10.1097/NNR.0000000000000474; Dunn, S.L., Olamijulo, G.B., Fuglseth, H.L., Holden, T.P., Swieringa, L.L., Sit, M.J., Rieth, N.P., Tintle, N.L., The State-Trait Hopelessness Scale: Development and testing (2014) Western Journal of Nursing Research, 36 (4), pp. 552-570. , https://doi.org/10.1177/0193945913507634; Dunn, S.L., Robbins, L.B., Smith, S.W., Ranganathan, R., DeVon, H.A., Collins, E.G., Hong, H.G., Tintle, N.L., Enhancing physical activity in cardiac patients who report hopelessness: Feasibility testing of an intervention (2019) Health Education Journal, 78 (2), pp. 226-237. , https://doi.org/10.1177/0017896918813610; Dunn, S.L., Stommel, M., Corser, W., Holmes-Rovner, M., Hopelessness and its effect on cardiac rehabilitation exercise participation following hospitalization for acute coronary syndrome (2009) Journal of cardiopulmonary rehabilitation and prevention, 29 (1), pp. 32-39. , https://doi.org/10.1097/HCR.0b013e31819276ba; (2019), https://euroqol.org/publications/user-guides, EQ-5D-5L User Guide,

2019, Retrieved from; Freedson, P.S., Melanson, E., Sirard, J., Calibration of the Computer Science and Applications, Inc. accelerometer (1998) Medicine and science in sports and exercise, 30 (5), pp. 777-781; Gaalema, D.E., Higgins, S.T., Shepard, D.S., Suaya, J.A., Savage, M.P.D., Ades, P.A., State-by-state variations in cardiac rehabilitation participation are associated with educational attainment, income, and program availability (2014) Journal of cardiopulmonary rehabilitation and prevention, 34 (4), pp. 248-254. , https://doi.org/10.1097/HCR.0000000000000059; Gebbia, V., Piazza, D., Valerio, M.R., Borsellino, N., Firenze, A., Patients with cancer and COVID-19: A WhatsApp Messenger-based survey of patients' queries, needs, fears, and actions taken (2020) JCO Global Oncology, 6, pp. 722-729. , https://doi.org/10.1200/GO.20.00118; Gillham, S., Endacott, R., Impact of enhanced secondary prevention on health behaviour in patients following minor stroke and transient ischaemic attack: A randomized controlled trial (2010) Clinical Rehabilitation, 24 (9), pp. 822-830. , https://doi.org/10.1177/0269215510367970; Goel, K., Lennon, R.J., Tilbury, R.T., Squires, R.W., Thomas, R.J., Impact of cardiac rehabilitation on mortality and cardiovascular events after percutaneous coronary intervention in the community (2011) Circulation, 123 (21), pp. 2344-2352. , https://doi.org/10.1161/CIRCULATIONAHA.110.983536; Gottlieb, B.H., Bergen, A.E., Social support concepts and measures (2010) Journal of Psychosomatic Research, 69 (5), pp. 511-520. , https://doi.org/10.1016/j.jpsychores.2009.10.001; Herdman, M., Gudex, C., Lloyd, A., Janssen, M.F., Kind, P., Parkin, D., Bonsel, G., Badia, X., Development and preliminary testing of the new five-level version of EQ-5D (EQ-5D-5L) (2011) Quality of Life Research, 20 (10), pp. 1727-1736. , https://doi.org/10.1007/s11136-011-9903-x; Holm, S., A simple sequential rejective multiple test procedure (1979) Scandinavian Journal of Statistics, 6, pp. 65-70. , http://www.jstor.org/stable/4615733; Hughes, J.W., Casey, E., Doe, V.H., Glickman, E.L., Stein, P.K., Josephson, R., Waechter, D., Rosneck, J., Depression and heart rate variability in cardiac rehabilitation patients: Exploring the roles of physical activity and fitness (2010) Perceptual and Motor Skills, 111 (2), pp. 608-624. , https://doi.org/10.2466/13.15.PMS.111.5.608-624; Janssen, V., De Gucht, V., van Exel, H., Maes, S., Beyond resolutions? A randomized controlled trial of a self-regulation lifestyle programme for post-cardiac rehabilitation patients (2013) European Journal of Prevention Cardiology, 20 (3), pp. 431-441. , https://doi.org/10.1177/2047487312441728; Jones, N.L., Schneider, P.L., Kaminsky, L.A., Riggin, K., Taylor, A.M., An assessment of the total amount of physical activity of patients participating in a phase III cardiac rehabilitation program (2007) Journal of Cardiopulmonary Rehabilitation and Prevention, 27 (2), pp. 81-85; De Jonge, P., Mangano, D., Whooley, M.A., Differential association of cognitive and somatic depressive symptoms with heart rate variability in patients with stable coronary heart disease: findings from the Heart and Soul Study (2007) Psychosomatic Medicine, 69 (8), pp. 735-739. , https://doi.org/10.1097/PSY.0b013e31815743ca; Kangelaris, K.N., Vittinghoff, E., Otte, C., Na, B., Auerbach, A.D., Whooley, M.A., Association between a serotonin transporter gene variant and hopelessness among men in the Heart and Soul Study (2010) Journal of General Internal Medicine, 25 (10), pp. 1030-1037. , https://doi.org/10.1007/s11606-010-1403-0; Karmali, K.N., Davies, P., Taylor, F., Beswick, A., Martin, N., Ebrahim, S., Promoting patient uptake and adherence in cardiac rehabilitation (2014) Cochrane Database of Systematic Reviews, (6). , https://doi.org/10.1002/14651858.CD007131.pub3; Kroenke, K., Spitzer, R.L., The PHQ-9: A new depression diagnostic and severity measure

(2002) Psychiatric Annals, 32 (9), pp. 509-515. , https://doi.org/10.3928/0048-5713-20020901-06; Kroenke, K., Spitzer, R.L., Williams, J.B., The PHQ-9: Validity of a brief depression severity measure (2001) Journal of General Internal Medicine, 16 (9), pp. 606-613. , https://doi.org/10.1046/j.1525-1497.2001.016009606.x; Marchionni, N., Fattirolli, F., Fumagalli, S., Oldridge, N., Del Lungo, F., Morosi, L., Burgisser, C., Masotti, G., Improved exercise tolerance and quality of life with cardiac rehabilitation of older patients after myocardial infarction: Results of a randomized, controlled trial (2003) Circulation, 107 (17), pp. 2201-2206. , https://doi.org/10.1161/01.CIR.0000066322.21016.4A; Marklund, D., Ryan, R.M., Tobin, V.J., Rollnick, S., Motivational interviewing and Self–Determination Theory (2005) Journal of Social and Clinical Psychology, 24 (6), pp. 811-831. , https://doi.org/10.1521/jscp.2005.24.6.811; Mattioli, A.V., Puviani, B.M., Nasi, M., Farinetti, A., COVID-19 pandemic: The effects of quarantine on cardiovascular risk (2020) European Journal of Clinical Nutrition, 74, pp. 852-855. , https://doi.org/10.1038/s41430-020-0646-z; McGrady, A., McGinnis, R., Badenhop, D., Bentle, M., Rajput, M., Effects of depression and anxiety on adherence to cardiac rehabilitation (2009) Journal of cardiopulmonary rehabilitation and prevention, 29 (6), pp. 358-364. , https://doi.org/10.1097/HCR.0b013e3181be7a8f; Meijer, A., Conradi, H.J., Bos, E.H., Thombs, B.D., van Melle, J.P., de Jonge, P., Prognostic association of depression following myocardial infarction with mortality and cardiovascular events: A meta-analysis of 25 years of research (2011) General Hospital Psychiatry, 33 (3), pp. 203-216. , https://doi.org/10.1016/j.genhosppsych.2011.02.007; Midence, L., Arthur, H.M., Oh, P., Stewart, D.E., Grace, S.L., Women's health behaviours and psychosocial well-being by cardiac rehabilitation program model: A randomized controlled trial (2016) Canadian Journal of Cardiology, 32 (8), pp. 956-962. , https://doi.org/10.1016/j.cjca.2015.10.007; Miller, W.R., Rollnick, S., (2013) Motivational interviewing: Helping people change, , 3rd ed., Guilford Press; Moore, S.M., Dolansky, M.A., Ruland, C.M., Pashkow, F.J., Blackburn, G.G., Predictors of women's exercise maintenance after cardiac rehabilitation (2003) Journal of Cardiopulmonary Rehabilitation and Prevention, 23 (1), pp. 40-49. , https://doi.org/10.1097/00008483-200301000-00008; Murphy, B.M., Worcester, M.U.C., Higgins, R.O., Elliott, P.C., Le Grande, M.R., Mitchell, F., Navaratnam, H., Goble, A.J., Reduction in 2-year recurrent risk score and improved behavioral outcomes after participation in the "Beating Heart Problems" self-management program (2013) Journal of Cardiopulmonary Rehabilitation, 33 (4), pp. 220-228. , https://doi.org/10.1097/HCR.0b013e31828c7812; (2014), https://www.ninr.nih.gov/sites/files/docs/NINR%20DSM%20Policy%202014%20FINAL.pdf, Policy of the National Institute of Nursing Research for data and safety monitoring of extramural clinical trials, Retrieved from; Núñez, J.E., Núñez, E., Fácila, L., Bertomeu, V., Llàcer, À., Bodí, V., Sanchis, J., Chorro, F.J., Prognostic value of Charlson comorbidity index at 30 days and 1 year after acute myocardial infarction (2004) Revista Española de Cardiología (English Edition), 57 (9), pp. 842-849. , https://doi.org/10.1016/S1885-5857(06)60649; Oliveira, J., Ribeiro, F., Gomes, H., Effects of a home-based cardiac rehabilitation program on the physical activity levels of patients with coronary artery disease (2008) Journal of Cardiopulmonary Rehabilitation and Prevention, 28 (6), pp. 392-396. , https://doi.org/10.1097/HCR.0b013e31818c3b83; Pedersen, S.S., Denollet, J., Daemen, J., van de Sande, M., de Jaegere, P.T., Serruys, P.W., Erdman, R.A.M., van Domburg, R.T., Fatigue, depressive symptoms, and hopelessness as predictors

of adverse clinical events following percutaneous coronary intervention with paclitaxel-eluting stents (2007) Journal of Psychosomatic Research, 62 (4), pp. 455-461. , https://doi.org/10.1016/j.jpsychores.2006.12.018; Pinto, B.M., Dunsiger, S.I., Farrell, N., Marcus, B.H., Todaro, J.F., Psychosocial outcomes of an exercise maintenance intervention after phase II cardiac rehabilitation (2013) Journal of Cardiopulmonary Rehabilitation and Prevention, 33 (2), pp. 91-98. , https://doi.org/10.1097/HCR.0b013e3182825531; Posner, K., Brown, G.K., Stanley, B., Brent, D.A., Yershova, K.V., Oquendo, M.A., Currier, G.W., Mann, J.J., The Columbia–Suicide Severity Rating Scale: Initial validity and internal consistency findings from three multisite studies with adolescents and adults (2011) American Journal of Psychiatry, 168 (12), pp. 1266-1277. , https://doi.org/10.1176/appi.ajp.2011.10111704; Radovanovic, D., Seifert, B., Urban, P., Eberli, F.R., Rickli, H., Bertel, O., Puhan, M.A., Erne, P., Validity of Charlson Comorbidity Index in patients hospitalised with acute coronary syndrome. Insights from the nationwide AMIS Plus registry 2002-2012 (2014) Heart (British Cardiac Society), 100 (4), pp. 288-294. , https://doi.org/10.1136/heartjnl-2013-304588; Russell, K.L., Bray, S.R., Promoting self-determined motivation for exercise in cardiac rehabilitation: The role of autonomy support (2010) Rehabilitation Psychology, 55 (1), pp. 74-80. , https://doi.org/10.1037/a0018416; Ryan, R.M., Deci, E.L., Self-Determination Theory and the facilitation of intrinsic motivation, social development, and well-being (2000) American Psychologist, 55 (1), pp. 68-78. , https://doi.org/10.1037/0003-066x.55.1.68; Ryan, R.M., Edward, L., An overview of Self-Determination Theory: An organismic-dialectical perspective (2002) Handbook of self-determination research, pp. 3-33. , University of Rochester Press; Shapiro, J.R., Koro, T., Doran, N., Thompson, S., Sallis, J.F., Calfas, K., Patrick, K., Text4Diet: A randomized controlled study using text messaging for weight loss behaviors (2012) Prevention Medicine, 55 (5), pp. 412-417. , https://doi.org/10.1016/j.ypmed.2012.08.011; Smith, S.C., Jr., Benjamin, E.J., Bonow, R.O., Braun, L.T., Creager, M.A., Franklin, B.A., Gibbons, R.J., Taubert, K.A., AHA/ACCF secondary prevention and risk reduction therapy for patients with coronary and other atherosclerotic vascular disease: 2011 update: A guideline from the American Heart Association and American College of Cardiology Foundation (2011) Circulation, 124 (22), pp. 2458-2473. , https://doi.org/10.1161/CIR.0b013e318235eb4d; Smolderen, K.G., Spertus, J.A., Reid, K.J., Buchanan, D.M., Krumholz, H.M., Denollet, J., Vaccarino, V., Chan, P.S., The association of cognitive and somatic depressive symptoms with depression recognition and outcomes after myocardial infarction (2009) Circulation: Cardiovascular Quality and Outcomes, 2 (4), pp. 328-337. , https://doi.org/10.1161/CIRCOUTCOMES.109.868588; Surtees, P.G., Wainwright, N.W., Luben, R.N., Wareham, N.J., Bingham, S.A., Khaw, K.T., Depression and ischemic heart disease mortality: Evidence from the EPIC-Norfolk United Kingdom prospective cohort study (2008) American Journal of Psychiatry, 165 (4), pp. 515-523. , https://doi.org/10.1176/appi.ajp.2007.07061018; (2016), https://cssrs.columbia.edu/documents/clinical-triage-guidelines-using-c-ssrs/, Guidelines For Triage Using the C-SSRS, Retrieved from; (2018), https://health.gov/sites/default/files/2019-09/Physical_Activity_Guidelines_2nd_edition.pdf, . Physical activity guidelines for Americans (2nd ed.). Washington, DC U.S. Department of Health and Human Services. Retrieved from; Vaglio, J., Conard, M., Poston, W.S., O'Keefe, J., Haddock, C.K., House, J., Spertus, J.A., Testing the performance of the ENRICHD Social Support Instrument in cardiac patients (2004) Health and Quality of Life Outcomes, 2 (1), pp. 1-5. , https://doi.org/10.1186/1477-7525-2-24; Whooley, M.A., Depressive symptoms, health behaviors, and risk of cardiovascular events in patients with coronary

heart disease (2008) Journal of the American Medical Association, 300 (20), pp. 2379-2388. , https://doi.org/10.1001/jama.2008.711; Win, S., Parakh, K., Eze-Nliam, C.M., Gottdiener, J.S., Kop, W.J., Ziegelstein, R.C., Depressive symptoms, physical inactivity and risk of cardiovascular mortality in older adults: The Cardiovascular Health Study (2011) Heart, 97 (6), pp. 500-505. , https://doi.org/10.1136/hrt.2010.209767; Ye, S., Muntner, P., Shimbo, D., Judd, S.E., Richman, J., Davidson, K.W., Safford, M.M., Behavioral mechanisms, elevated depressive symptoms, and the risk for myocardial infarction or death in individuals with coronary heart disease: The REGARDS (Reason for Geographic and Racial Differences in Stroke) study (2013) Journal of the American College of Cardiology, 61 (6), pp. 622-630. , https://doi.org/10.1016/j.jacc.2012.09.058},
correspondence_address1={Dunn, S.L.; Department of Biobehavioral Nursing Science, United States; email: sdunn01@uic.edu},
publisher={John Wiley and Sons Inc},
issn={01606891},
pubmed_id={33428224},
language={English},
abbrev_source_title={Res. Nurs. Health},
document_type={Article},
source={Scopus},
}

@ARTICLE{Cota2021870,
author={Cota, B.C. and Suhett, L.G. and Leite, N.N. and Pereira, P.F. and Ribeiro, S.A.V. and Franceschini, S.D.C.C.},
title={Cardiometabolic risk and health behaviours in adolescents with normal-weight obesity: A systematic review},
journal={Public Health Nutrition},
year={2021},
volume={24},
number={5},
pages={870-881},
doi={10.1017/S1368980020004863},
note={cited By 0},
url={https://www.scopus.com/inward/record.uri?eid=2-s2.0-85099497131&doi=10.1017%2fS1368980020004863&partnerID=40&md5=03ea0a3147f50e7c08d497846917990f},
affiliation={Departamento de Nutrição e Saúde, Universidade Federal de Viçosa, Ed. Centro de Ciências Biológicas II, Campus Universitário, s/n°, Av. Peter Henry Rolfs, Viçosa, Minas Gerais, 36570-900, Brazil},
abstract={Objective: To analyse the presence of cardiometabolic risk factors in adolescents with normal-weight obesity (NWO), as well as to investigate health behaviours related to the phenotype. Design: The study was conducted according to the Preferred Reporting Items for Systematic reviews and Meta-Analyses guidelines and the bibliographic search was carried out in the PubMed, Scielo and ScienceDirect databases. Setting: School, university and population. Participants: Adolescents between 10 and 19 years old. Results: A total of eight papers were included. Most studies have found a relationship between NWO and the presence of cardiometabolic risk factors, such as high waist circumference, unfavourable lipid and glycid profile. As for health behaviours, three of the eight studies included evaluated eating habits; however, the results were not conclusive. In addition, four studies analysed the practice of physical activity or physical fitness, which was lower in NWO. Conclusions: The available evidence indicates that NWO is related to the early development of

cardiometabolic changes, physical inactivity and less physical fitness in adolescents. The results also reveal the importance of early detection of the phenotype, as well as the need for further research on the associated factors to prevent future diseases. Registration (PROSPERO: CRD42020161204). © Authors 2020.},
author_keywords={Adiposity; Adolescent; Normal-weight obese; Normal-weight obesity; Obesity; Teenager},
references={(2020) Health Topics: Adolescent Health, , https://www.who.int/westernpacific/health-topics/adolescent-health, (accessed July 2020), WHO, Geneva: World Health Organization; (2007) Growth Reference Data for 5-19 Years, , https://www.who.int/growthref/en/, (accessed July 2020), WHO, Geneva: World Health Organization; Krebs, N.F., Himes, J.H., Jacobson, D., Assessment of child and adolescent overweight and obesity (2007) Pediatrics, 120, pp. S193-S228; Kelishadi, R., Gouya, M.M., Ardalan, G., First reference curves of waist and hip circumferences in an Asian population of youths: CASPIAN study (2007) J Trop Pediatr, 53, pp. 158-164; Okorodudu, D.O., Jumean, M.F., Montori, V.M., Diagnostic performance of body mass index to identify obesity as defined by body adiposity: A systematic review and meta-analysis (2010) Int J Obes, 34, pp. 791-799; Javed, A., Jumean, M., Murad, M.H., Diagnostic performance of body mass index to identify obesity as defined by body adiposity in children and adolescents: A systematic review and meta-analysis (2015) Pediatr Obes, 10, pp. 234-244; De Lorenzo, A., Martinoli, R., Vaia, F., Normal weight obese (NWO) women: An evaluation of a candidate new syndrome (2006) Nutr Metab Cardiovasc Dis, 16, pp. 513-523; Kapoor, N., Furler, J., Paul, T.V., Normal weight obesity: An underrecognized problem in individuals of South Asian Descent (2019) Clin Ther, 41, pp. 1638-1642; Correa-Rodríguez, M., González-Ruíz, K., Rincón-Pabón, D., Normal-weight obesity is associated with increased cardiometabolic risk in young adults (2020) Nutrients, 12, p. 1106; Lee, H.S., Park, Y.M., Han, K., Obesity-related hypertension: Findings from the Korea National Health and Nutrition Examination Survey 2008-2010 (2020) PLoS One, 15, p. 0230616; De Lorenzo, A., Del Gobbo, V., Premrov, M.G., Normal-weight obese syndrome: Early inflammation? (2007) Am J Clin Nutr, 85, pp. 40-45; Di Renzo, L., Bigioni, M., Del Gobbo, V., Interleukin-1 (IL-1) receptor antagonist gene polymorphism in normal weight obese syndrome: Relationship to body composition and IL-1 alpha and beta plasma levels (2007) Pharmacol Res, 55, pp. 131-138; Romero-Corral, A., Somers, V.K., Sierra-Johnson, J., Normal weight obesity: A risk factor for cardiometabolic dysregulation and cardiovascular mortality (2010) Eur Heart J, 31, pp. 737-746; Shea, J.L., King, M.T.C., Yi, Y., Body fat percentage is associated with cardiometabolic dysregulation in BMI-defined normal weight subjects (2012) Nutr Metab Cardiovasc Dis, 22, pp. 741-747; Madeira, F.B., Silva, A.A., Veloso, H.F., Normal weight obesity is associated with metabolic syndrome and insulin resistance in young adults from a middle-income country (2013) PLoS One, 8, p. 60673; Marques-Vidal, P., Pécoud, A., Hayoz, D., Normal weight obesity: Relationship with lipids, glycaemic status, liver enzymes and inflammation (2010) Nutr Metab Cardiovasc Dis, 20, pp. 669-675; Kang, S., Kyung, C., Park, J.S., Subclinical vascular inflammation in subjects with normal weight obesity and its association with body fat: An 18 F-FDG-PET/CT study (2014) Cardiovasc Diabetol, 13, p. 70; Oliveros, E., Somers, V.K., Sochor, O., The concept of normal weight obesity (2014) Prog Cardiovasc Dis, 56, pp. 426-433; Männistö, S., Harald, K., Kontto, J., Dietary and lifestyle characteristics associated with normal-weight obesity: The National FINRISK 2007 Study (2014) Br J Nutr, 111, pp. 887-894; Olafsdottir, A.S., Torfadottir, J.E., Arngrimsson, S.A., Health behavior and metabolic risk factors associated with normal weight

obesity in adolescents (2016) PLoS One, 11, p. 0161451; Karelis, A.D., St-Pierre, D.H., Conus, F., Metabolic and body composition factors in subgroups of obesity: What do we know? (2004) J Clin Endocrinol Metab, 89, pp. 2569-2575; Moher, D., Liberati, A., Tetzlaff, J., Preferred reporting items for systematic reviews and meta-analyses: The PRISMA statement (2009) PLoS Med, 6, p. 1000097; Ouzzani, M., Hammady, H., Fedorowicz, Z., Rayyan-a web and mobile app for systematic reviews (2016) Syst Rev, 5, p. 210; (2005) Nutrition in Adolescence: Issues and Challenges for the Health Sector: Issues in Adolescent Health and Development, , https://apps.who.int/iris/handle/10665/43342, (accessed July 2020), WHO, Geneva: World Health Organization; (2020), https://www.nhlbi.nih.gov/health-topics/study-quality-assessment-tools, (accessed September 2020), Study Quality Assessment Tools, NHLBI, NIH; Wiklund, P., Törmäkangas, T., Shi, Y., Normal-weight obesity and cardiometabolic risk: A 7-year longitudinal study in girls from prepuberty to early adulthood (2017) Obesity, 25, pp. 1077-1082; Cheng, S., Wiklund, P., The effects of muscle mass and muscle quality on cardio-metabolic risk in peripubertal girls: A longitudinal study from childhood to early adulthood (2018) Int J Obes, 42, pp. 648-654; Musálek, M., Pařízková, J., Godina, E., Poor skeletal robustness on lower extremities and weak lean mass development on upper arm and calf: Normal weight obesity in middle-school-aged children (9 to 12) (2018) Front Pediatr, 6, p. 371; Mlbm, B., Oliveira, B.R., Fonseca, J.M., (2020) Assessment of Blood Biomarkers in Adolescents Classified by Body Mass Index and Body Fat Percentage, p. 00084719. , Cadernos de Saúde Pública 36; García-Hermoso, A., Agostinis-Sobrinho, C., Camargo-Villalba, G.E., Normal-weight obesity is associated with poorer cardiometabolic profile and lower physical fitness levels in children and adolescents (2020) Nutrients, 12, p. 1171; Serrano, H.M.S., Carvalho, G.Q., Pereira, P.F., Body composition, biochemical and clinical changes of adolescents with excessive adiposity (2010) Arquivos Brasileiros Cardiologia, 95, pp. 464-472; Yaguchi-Tanaka, Y., Kawagoshi, Y., Sasaki, S., Cross-sectional study of possible association between rapid eating and high body fat rates among female Japanese college students (2013) J Nutr Sci Vitaminol, 59, pp. 243-249; Graça, P., Mateus, M.P., Lima, R.M., The Mediterranean diet concept and the promotion of healthy eating in Portuguese schools (2013) Revista Nutrícias, 13, pp. 6-9; Barnett, A., Chan, L.Y.S., Bruce, L.C., A preliminary study of the 20-m multistage shuttle run as a predictor of peak VO2 in Hong Kong Chinese Students (1993) Pediatr Exerc Sci, 5, pp. 42-50; Sallis, J.F., Strikmiller, P.K., Harsha, D.W., Validation of interviewer-and self-administered physical activity checklists for fifth grade students (1996) Med Sci Sports Exerc, 28, pp. 840-851; Ainsworth, B.E., Haskell, W.L., Leon, A.S., Compendium of physical activities: Classification of energy costs of human physical activities (1993) Med Sci Sports Exerc, 25, pp. 71-80; Benedetti, T.R.B., Antunes, P.D., Rodriguez-Añez, C.R., Reproducibility and validity of the International Physical Activity Questionnaire (IPAQ) in elderly men (2007) Rev Brasileira Med Do Esporte, 13, pp. 11-16; Frisancho, A.R., (1990) Anthropometric Standards for the Assessment of Growth and Nutritional Status, , Michigan: University of Michigan Press; Jean, N., Somers, V.K., Sochor, O., Normal-weight obesity: Implications for cardiovascular health (2014) Curr Atheroscler Rep, 16, p. 464; Conus, F., Rabasa-Lhoret, R., Péronnet, F., Characteristics of metabolically obese normal-weight (MONW) subjects (2007) Appl Physiol Nutr Metab, 32, pp. 4-12; Marques-Vidal, P., Chiolero, A., Paccaud, F., Large differences in the prevalence of normal weight obesity using various cut-offs for excess body fat (2008) E Spen Eur e J Clin Nutr Metab, 3, pp. 159-162; Katzmarzyk, P.T., Srinivasan, S.R., Chen, W., Body mass index, waist circumference, and clustering of cardiovascular disease risk

factors in a biracial sample of children and adolescents (2004) Pediatrics, 114, pp. 198-205; Nechuta, S.J., Shu, X.-O., Li, H.-L., Combined impact of lifestyle-related factors on total and cause-specific mortality among Chinese women: Prospective cohort study (2010) PLoS Med, 7, p. 1000339; Balagopal, P.B., De Ferranti, S.D., Cook, S., Nontraditional risk factors and biomarkers for cardiovascular disease: Mechanistic, research, and clinical considerations for youth: A scientific statement from the American Heart Association (2011) Circulation, 123, pp. 2749-2769; Schulte, H., Cullen, P., Assmann, G., Obesity, mortality and cardiovascular disease in the Münster Heart Study (PROCAM) (1999) Atherosclerosis, 144, pp. 199-209; Kannel, W.B., The Framingham study: ITS 50-year legacy and future promise (2000) J Atheroscler Thromb, 6, pp. 60-66; Lauer, R.M., Clarke, W.R., Mahoney, L.T., Childhood predictors for high adult blood pressure (1993) Muscatine Study Pediatr Clin North Am, 40, pp. 23-40; Berenson, G.S., Wattigney, W.A., Bao, W., Epidemiology of early primary hypertension and implications for prevention: The Bogalusa Heart Study (1994) J Hum Hypertens, 8, pp. 303-311; Cook, N.R., Gillman, M.W., Rosner, B.A., Prediction of young adult blood pressure from childhood blood pressure, height, and weight (1997) J Clin Epidemiol, 50, pp. 571-579; Raitakari, O.T., Juonala, M., Kähönen, M., Cardiovascular risk factors in childhood and carotid artery intima-media thickness in adulthood: The Cardiovascular Risk in Young Finns Study (2003) JAMA, 290, pp. 2277-2283; Hartiala, O., Magnussen, C.G., Kajander, S., Adolescence risk factors are predictive of coronary artery calcification at middle age: The cardiovascular risk in young Finns study (2012) J Am Coll Cardiol, 60, pp. 1364-1370; Jakubowski, K.P., Cundiff, J.M., Matthews, K.A., Cumulative childhood adversity and adult cardiometabolic disease: A meta-analysis (2018) Health Psychol, 37, pp. 701-715; Weihrauch-Blüher, S., Schwarz, P., Klusmann, J.-H., Childhood obesity: Increased risk for cardiometabolic disease and cancer in adulthood (2019) Metab Clin Exp, 92, pp. 147-152; (2014) Strategies for the Care of People with Chronic Disease, , Brazil, Departamento de Atenção Básica. Brasília: Ministério da Saúde; (2011) Strategic Action Plan for Tackling Chronic Non-Communicable Diseases (NCDs) in Brazil: 2011-2022, , Brazil, 1a edição. Brasília, DF: Ministério da Saúde; Barnes, A.S., Obesity and sedentary lifestyles (2012) Tex Heart Inst J, 39, pp. 224-227; Power, C., Pinto Pereira, S.M., Law, C., Obesity and risk factors for cardiovascular disease and type 2 diabetes: Investigating the role of physical activity and sedentary behaviour in mid-life in the 1958 British cohort (2014) Atherosclerosis, 233, pp. 363-369; Barroso, T.A., Marins, L.B., Alves, R., Association of Central Obesity with the Incidence of Cardiovascular Diseases and Risk Factors (2017) Int J Cardiovasc Sci, 30, pp. 416-424; Mac Ananey, O., McLoughlin, B., Leonard, A., Inverse relationship between physical activity, adiposity, and arterial stiffness in healthy middle-aged subjects (2015) J Phys Act Health, 12, pp. 1576-1581; Garcia-Pastor, T., Salinero, J.J., Sanz-Frias, D., Body fat percentage is more associated with low physical fitness than with sedentarism and diet in male and female adolescents (2016) Physiol Behav, 165, pp. 166-172; Edwardson, C.L., Gorely, T., Davies, M.J., Association of sedentary behaviour with metabolic syndrome: A meta-analysis (2012) PLoS One, 7, p. 34916; Hu, F.B., Li, T.Y., Colditz, G.A., Television watching and other sedentary behaviors in relation to risk of obesity and type 2 diabetes mellitus in women (2003) JAMA, 289, pp. 1785-1791; Mahan, L.K., Raymond, J.L., Israel weight management nutrition (2012) Krause's Food & Nutrition Therapy, pp. 477-478. , Rio de Janeiro: Elsevier Editora Ltda; Lubans, D., Richards, J., Hillman, C., Physical activity for cognitive and mental health in youth: A systematic review of mechanisms (2016) Pediatrics, 138, p. 20161642; De Souza, T.P., Ferreira, F.C., Barbosa, M.R., Ingestão alimentar e excesso de

adiposidade de mulheres sedentárias [Food intake and excess of adiposity in healthy women] (2016) Braspen J, 31, pp. 316-321; Gallagher, M.L., Ingestion: Nutrients and their metabolism (2012) Krause's Food & Nutrition Therapy, pp. 36-38. , Rio de Janeiro: Elsevier Editora Ltda; Bernaud, F.S.R., Rodrigues, T.C., Fibra alimentar: Ingestão adequada e efeitos sobre a saúde do metabolismo [Dietary fiber-adequate intake and effects on metabolism health] (2013) Arq Bras Endocrinol Metab, 57, pp. 397-405},
correspondence_address1={Cota, B.C.; Departamento de Nutrição e Saúde, s/n°, Av. Peter Henry Rolfs, Brazil; email: brunaclementec@gmail.com},
publisher={Cambridge University Press},
issn={13689800},
coden={PHNUF},
pubmed_id={33256881},
language={English},
abbrev_source_title={Public Health Nutr.},
document_type={Review},
source={Scopus},
}

@ARTICLE{Xu2021,
author={Xu, R. and Cavallo, D.},
title={Social network analysis of the effects of a social media-based weight loss intervention targeting adults of low socioeconomic status: Single-arm intervention trial},
journal={Journal of Medical Internet Research},
year={2021},
volume={23},
number={4},
doi={10.2196/24690},
art_number={e24690},
note={cited By 0},
url={https://www.scopus.com/inward/record.uri?eid=2-s2.0-85104084216&doi=10.2196%2f24690&partnerID=40&md5=8b608952d5b1b4e9d53ad8846eab7b45},
affiliation={Department of Allied Health Sciences, College of Agriculture, Health and Natural Resources, University of Connecticut, Storrs, CT, United States; Department of Nutrition, School of Medicine, Case Western Reserve University, Cleveland, OH, United States},
abstract={Background: Obesity is a known risk factor for cardiovascular disease risk factors, including hypertension and type II diabetes. Although numerous weight loss interventions have demonstrated efficacy, there is considerably less evidence about the theoretical mechanisms through which they work. Delivering lifestyle behavior change interventions via social media provides unique opportunities for understanding mechanisms of intervention effects. Server data collected directly from web-based platforms can provide detailed, real-time behavioral information over the course of intervention programs that can be used to understand how interventions work. Objective: The objective of this study was to demonstrate how social network analysis can facilitate our understanding of the mechanisms underlying a social media-based weight loss intervention. Methods: We performed secondary analysis by using data from a pilot study that delivered a dietary and physical activity intervention to a group of participants via Facebook. We mapped out participants' interaction networks over the 12-week intervention period and linked participants'network characteristics (eg, in-degree, out-degree, network constraint) to participants' changes in theoretical mediators (ie, dietary knowledge,

perceived social support, self-efficacy) and weight loss by using regression analysis. We also performed mediation analyses to explore how the effects of social network measures on weight loss could be mediated by the aforementioned theoretical mediators. Results: In this analysis, 47 participants from 2 waves completed the study and were included. We found that increases in the number of posts, comments, and reactions significantly predicted weight loss ($\beta=-.94$, $P=.04$); receiving comments positively predicted changes in self-efficacy ($\beta=7.81$, $P=.009$), and the degree to which one's network neighbors are tightly connected with each other weakly predicted changes in perceived social support ($\beta=7.70$, $P=.08$). In addition, change in self-efficacy mediated the relationship between receiving comments and weight loss ($\beta=-.89$, $P=.02$). Conclusions: Our analyses using data from this pilot study linked participants' network characteristics with changes in several important study outcomes of interest such as self-efficacy, social support, and weight. Our results point to the potential of using social network analysis to understand the social processes and mechanisms through which web-based behavioral interventions affect participants' psychological and behavioral outcomes. Future studies are warranted to validate our results and to further explore the relationship between network dynamics and study outcomes in similar and larger trials. © 2021 Journal of Medical Internet Research. All rights reserved.},
author_keywords={Electronic health; Social media intervention; Social network analysis; Weight loss intervention},
references={(2013) Managing overweight and obesity in adults: Systematic evidence review from the obesity expert panel, , https://www.nhlbi.nih.gov/health-topics/managing-overweight-obesity-in-adults, National Heart, Lung, and Blood Institute. Nov. [accessed 2020-11-03]; McLaren, L., Socioeconomic status and obesity (2007) Epidemiol Rev, 29, pp. 29-48. , [doi] [Medline: 17478442]; Sommer, I, Griebler, U, Mahlknecht, P, Thaler, K, Bouskill, K, Gartlehner, G, Socioeconomic inequalities in non-communicable diseases and their risk factors: An overview of systematic reviews (2015) BMC Public Health, 15, p. 914. , Sep 18; [FREE Full text] [doi] [Medline: 26385563]; Stephens, SK, Cobiac, LJ, Veerman, JL., Improving diet and physical activity to reduce population prevalence of overweight and obesity: An overview of current evidence (2014) Prev Med, 62, pp. 167-178. , May; [doi] [Medline: 24534460]; Beauchamp, A, Backholer, K, Magliano, D, Peeters, A., The effect of obesity prevention interventions according to socioeconomic position: A systematic review (2014) Obes Rev, 15 (7), pp. 541-554. , Jul; [doi] [Medline: 24629126]; Hillier-Brown, FC, Bambra, CL, Cairns, J, Kasim, A, Moore, HJ, Summerbell, CD., A systematic review of the effectiveness of individual, community and societal-level interventions at reducing socio-economic inequalities in obesity among adults (2014) Int J Obes (Lond), 38 (12), pp. 1483-1490. , Dec; [FREE Full text] [doi] [Medline: 24813369]; McGill, R, Anwar, E, Orton, L, Bromley, H, Lloyd-Williams, F, O'Flaherty, M, Erratum to: Are interventions to promote healthy eating equally effective for all? Systematic review of socioeconomic inequalities in impact (2015) BMC Public Health, 15, p. 894. , Sep 15; [FREE Full text] [doi] [Medline: 26373840]; (2019) Pew Research Center Internet and Technology, , http://www.pewinternet.org/fact-sheet/socialmedia/, Social media fact sheet. Jun 12. [accessed 2020-11-04]; Baruth, M, Sharpe, PA, Parra-Medina, D, Wilcox, S., Perceived barriers to exercise and healthy eating among women from disadvantaged neighborhoods: Results from a focus groups assessment (2014) Women Health, 54 (4), pp. 336-353. , [FREE Full text] [doi] [Medline: 24617795]; Griffiths, F, Lindenmeyer, A, Powell, J, Lowe, P, Thorogood, M., Why are health care interventions delivered over the internet? A systematic review

of the published literature (2006) J Med Internet Res, 8 (2), p. e10. , Jun 23; [FREE Full text] [doi] [Medline: 16867965]; Fox, S., (2014) Pew Research Center, , https://www.pewresearch.org/fact-tank/2014/01/15/the-social-life-of-health-information/, The social life of health information, Jan 15. [accessed 2020-11-15]; Heo, Y, Park, J, Kim, J, Park, H., The emerging viewertariat in South Korea: The Seoul mayoral TV debate on Twitter, Facebook, and blogs (2016) Telematics and Informatics, 33 (2), pp. 570-583. , May; [doi]; Rus, HM, Cameron, LD., Health Communication in Social Media: Message Features Predicting User Engagement on Diabetes-Related Facebook Pages (2016) Ann Behav Med, 50 (5), pp. 678-689. , Oct; [doi] [Medline: 27059761]; Nam, Y, Lee, Y, Park, HW., Measuring web ecology by Facebook, Twitter, blogs and online news: 2012 general election in South Korea (2014) Qual Quant, 49 (2), pp. 675-689. , Mar 16; [doi]; Waring, ME, Jake-Schoffman, DE, Holovatska, MM, Mejia, C, Williams, JC, Pagoto, SL., Social Media and Obesity in Adults: A Review of Recent Research and Future Directions (2018) Curr Diab Rep, 18 (6), p. 34. , Apr 18; [doi] [Medline: 29671135]; Herring, SJ, Cruice, JF, Bennett, GG, Davey, A, Foster, GD., Using technology to promote postpartum weight loss in urban, low-income mothers: A pilot randomized controlled trial (2014) J Nutr Educ Behav, 46 (6), pp. 610-615. , [FREE Full text] [doi] [Medline: 25069621]; Herring, SJ, Cruice, JF, Bennett, GG, Rose, MZ, Davey, A, Foster, GD., Preventing excessive gestational weight gain among African American women: A randomized clinical trial (2016) Obesity (Silver Spring), 24 (1), pp. 30-36. , Jan; [FREE Full text] [doi] [Medline: 26592857]; Turner-McGrievy, GM, Tate, DF., Weight loss social support in 140 characters or less: Use of an online social network in a remotely delivered weight loss intervention (2013) Transl Behav Med, 3 (3), pp. 287-294. , Sep; [FREE Full text] [doi] [Medline: 24073180]; Cavallo, DN, Tate, DF, Ward, DS, DeVellis, RF, Thayer, LM, Ammerman, AS., Social support for physical activity-role of Facebook with and without structured intervention (2014) Transl Behav Med, 4 (4), pp. 346-354. , Dec; [FREE Full text] [doi] [Medline: 25584083]; Leahey, T, Kumar, R, Weinberg, B, Wing, R., Teammates and social influence affect weight loss outcomes in a team-based weight loss competition (2012) Obesity (Silver Spring), 20 (7), pp. 1413-1418. , Jul; [FREE Full text] [doi] [Medline: 22310234]; Hansen, MT., The Search-Transfer Problem: The Role of Weak Ties in Sharing Knowledge across Organization Subunits (1999) Administrative Science Quarterly, 44 (1), p. 82. , Mar; [doi]; Alexiev, AS, Jansen, JJ, Van den Bosch, FAJ, Volberda, HW., Top management team advice seeking and exploratory innovation: The moderating role of TMT heterogeneity (2010) Journal of Management Studies, 47 (7), pp. 1343-1364. , Jan 29; [doi]; Stewart, SA, Abidi, SSR., Applying social network analysis to understand the knowledge sharing behaviour of practitioners in a clinical online discussion forum (2012) J Med Internet Res, 14 (6), p. e170. , Dec 04; [FREE Full text] [doi] [Medline: 23211783]; Burt, RS., (1995) Structural Holes: The Social Structure of Competition, pp. 1-324. , Cambridge, MA: Harvard University Press; Aug 11; Lin, N., Building a theory of social capital (1999) Connections, 22 (1), pp. 28-51. , [doi]; Hall, A, Wellman, B., (1985) Social networks and social support. Social support and health, , https://psycnet.apa.org/record/1985-97489-002, Cambridge, MA: Academic Press; [accessed 2020-11-03]; Wellman, B, Gulia, M., The network basis of social support: A network is more than the sum of its ties (1999) Networks in the Global Village, pp. 83-118. , Philadelphia, PA: Routledge; Kelman, HC., Compliance, identification, and internalization three processes of attitude change (2016) Journal of Conflict Resolution, 2 (1), pp. 51-60. , Jul; [doi]; Friedkin, NE., (2006) A Structural Theory of Social Influence, pp. 1-254. ,

Cambridge, United Kingdom: Cambridge University Press; Cavallo, D, Martinez, R, Hooper, M, Flocke, S., Feasibility of a social media-based weight loss intervention designed for low-SES adults Transl Behav Med, 2020, p. ibaa070. , [doi]; McKee, HA, Porter, JE., (2009) The Ethics of Internet Research: A Rhetorical, Case-Based Process, , New York, USA: Peter Lang Inc., International Academic Publishers; New edition; Sams, S, Lim, YS, Park, HW., e-Research applications for tracking online socio-political capital in the Asia-Pacific region (2011) Asian Journal of Communication, 21 (5), pp. 450-466. , Oct; [doi]; Pagoto, S, Nebeker, C., How scientists can take the lead in establishing ethical practices for social media research (2019) J Am Med Inform Assoc, 26 (4), pp. 311-313. , Apr 01; [FREE Full text] [doi] [Medline: 30698793]; Pagoto, S, Waring, ME, Xu, R., A Call for a Public Health Agenda for Social Media Research (2019) J Med Internet Res, 21 (12), p. e16661. , Dec 19; [FREE Full text] [doi] [Medline: 31855185]; Thomas, S, Reading, J, Shephard, R., Revision of the Physical Activity Readiness Questionnaire (PAR-Q) (1992) Can J Sport Sci, 17 (4), pp. 338-345. , Dec; [Medline: 1330274]; Grytics analytical platform, , https://grytics.com/, [accessed 2021-03-29]; Hu, Y., Efficient, high-quality force-directed graph drawing (2005) Mathematica Journal, 10 (1), pp. 37-71. , [doi]; Batagelj, V, Zaversnik, M., An O(m) algorithm for cores decomposition of networks (2011) Advances in Data Analysis and Classification, , https://arxiv.org/abs/cs/0310049, [accessed 2021-03-29]; Chambers, D, Wilson, P, Thompson, C, Harden, M., Social network analysis in healthcare settings: A systematic scoping review (2012) PLoS One, 7 (8), p. e41911. , [FREE Full text] [doi] [Medline: 22870261]; Valente, TW., Network interventions (2012) Science, 337 (6090), pp. 49-53. , Jul 06; [doi] [Medline: 22767921]; Kaonga, NN, Labrique, A, Mechael, P, Akosah, E, Ohemeng-Dapaah, S, Sakyi Baah, J, Using social networking to understand social networks: Analysis of a mobile phone closed user group used by a Ghanaian health team (2013) J Med Internet Res, 15 (4), p. e74. , Apr 03; [FREE Full text] [doi] [Medline: 23552721]; Takahashi, Y, Uchida, C, Miyaki, K, Sakai, M, Shimbo, T, Nakayama, T., Potential benefits and harms of a peer support social network service on the internet for people with depressive tendencies: Qualitative content analysis and social network analysis (2009) J Med Internet Res, 11 (3), p. e29. , Jul 23; [FREE Full text] [doi] [Medline: 19632979]; Cavallo, DN, Sisneros, JA, Ronay, AA, Robbins, CL, Jilcott Pitts, SB, Keyserling, TC, Assessing the Feasibility of a Web-Based Weight Loss Intervention for Low-Income Women of Reproductive Age: A Pilot Study (2016) JMIR Res Protoc, 5 (1), p. e30. , Feb 26; [FREE Full text] [doi] [Medline: 26920252]; Pagoto, SL, Waring, ME, Schneider, KL, Oleski, JL, Olendzki, E, Hayes, RB, Twitter-Delivered Behavioral Weight-Loss Interventions: A Pilot Series (2015) JMIR Res Protoc, 4 (4), p. e123. , Oct 23; [FREE Full text] [doi] [Medline: 26500186]; Valle, CG, Tate, DF., Engagement of young adult cancer survivors within a Facebook-based physical activity intervention (2017) Transl Behav Med, 7 (4), pp. 667-679. , Dec; [FREE Full text] [doi] [Medline: 28374211]; Merchant, G, Weibel, N, Patrick, K, Fowler, JH, Norman, GJ, Gupta, A, Click "like" to change your behavior: A mixed methods study of college students' exposure to and engagement with Facebook content designed for weight loss (2014) J Med Internet Res, 16 (6), p. e158. , Jun 24; [FREE Full text] [doi] [Medline: 24964294]; Napolitano, MA, Whiteley, JA, Mavredes, MN, Faro, J, DiPietro, L, Hayman, LL, Using social media to deliver weight loss programming to young adults: Design and rationale for the Healthy Body Healthy U (HBHU) trial (2017) Contemp Clin Trials, 60, pp. 1-13. , Sep; [FREE Full text] [doi] [Medline: 28611007]; Barnett, GA, Xu, WW, Chu, J, Jiang, K, Huh, C, Park, JY, Measuring international relations in social media conversations (2017) Government Information Quarterly, 34 (1), pp. 37-44. , Jan; [doi];

Turner-McGrievy, GM, Tate, DF., Weight loss social support in 140 characters or less: Use of an online social network in a remotely delivered weight loss intervention (2013) Transl Behav Med, 3 (3), pp. 287-294. , Sep; [FREE Full text] [doi] [Medline: 24073180]; Hales, SB, Davidson, C, Turner-McGrievy, GM., Varying social media post types differentially impacts engagement in a behavioral weight loss intervention (2014) Transl Behav Med, 4 (4), pp. 355-362. , Dec; [FREE Full text] [doi] [Medline: 25584084]; Vardaman, JM, Amis, JM, Dyson, BP, Wright, PM, Van de Graaff Randolph, R., Interpreting change as controllable: The role of network centrality and self-efficacy (2012) Human Relations, 65 (7), pp. 835-859. , Jun 28; [doi]; Burt, RS, Lin, N, Cook, K., Closure as social capital (2001) Social capital: Theory and research, pp. 31-55. , Philadelphia, PA: Routledge; Linde, JA, Rothman, AJ, Baldwin, AS, Jeffery, RW., The impact of self-efficacy on behavior change and weight change among overweight participants in a weight loss trial (2006) Health Psychol, 25 (3), pp. 282-291. , May; [doi] [Medline: 16719599]; Roach, JB, Yadrick, M, Johnson, JT, Boudreaux, L, Forsythe, WA, Billon, W., Using self-efficacy to predict weight loss among young adults (2003) Journal of the American Dietetic Association, 103 (10), pp. 1357-1359. , Oct; [doi]; Franz, MJ, VanWormer, JJ, Crain, AL, Boucher, JL, Histon, T, Caplan, W, Weight-loss outcomes: A systematic review and meta-analysis of weight-loss clinical trials with a minimum 1-year follow-up (2007) J Am Diet Assoc, 107 (10), pp. 1755-1767. , Oct; [doi] [Medline: 17904936]; Edelmann, N., What is lurking? A literature review of research on lurking (2015) The Psychology of Social Networking, pp. 159-174. , Berlin, Boston: Sciendo Migration},
correspondence_address1={Xu, R.; Department of Allied Health Sciences, Koons Hall 326, United States; email: Ran.2.xu@uconn.edu},
publisher={JMIR Publications Inc.},
issn={14388871},
pubmed_id={33835033},
language={English},
abbrev_source_title={J. Med. Internet Res.},
document_type={Article},
source={Scopus},
}

@ARTICLE{Cava20211637,
author={Cava, E. and Neri, B. and Carbonelli, M.G. and Riso, S. and Carbone, S.},
title={Obesity pandemic during COVID-19 outbreak: Narrative review and future considerations},
journal={Clinical Nutrition},
year={2021},
volume={40},
number={4},
pages={1637-1643},
doi={10.1016/j.clnu.2021.02.038},
note={cited By 0},
url={https://www.scopus.com/inward/record.uri?eid=2-s2.0-85102815042&doi=10.1016%2fj.clnu.2021.02.038&partnerID=40&md5=8e8583835baf52f06ab4ae68024399a2},
affiliation={Unit of Dietetic and Clinical Nutrition, University Hospital “Maggiore della Carità”, Novara, Italy; S. Camillo – Forlanini Hospital, Rome, Italy; Department of Kinesiology & Health Sciences, College of Humanities & Sciences, Virginia Commonwealth University, Richmond, VA, United States; VCU Pauley Heart Center, Division of Cardiology, Department of Internal Medicine,

Virginia Commonwealth University, Richmond, VA, United States},
abstract={The high prevalence of obesity and obesity-related comorbidities has reached pandemic proportions, particularly in Western countries. Obesity increases the risk to develop several chronic noncommunicable disease, ultimately contributing to reduced survival. Recently, obesity has been recognized as major risk factor for coronavirus disease-19 (COVID-19)-related prognosis, contributing to worse outcomes in those with established COVID-19. Particularly, obesity has been associated with higher hospitalization rates in acute or intensive care and greater risk for invasive mechanical ventilation than lean people. Obesity is characterized by metabolic impairments and chronic low-grade systemic inflammation that causes a pro-inflammatory microenvironment, further aggravating the cytokine production and risk of cytokine storm response during Sars-Cov2 sepsis or other secondary infections. Moreover, the metabolic dysregulations are closely related to an impaired immune system and altered response to viral infection that can ultimately lead to a greater susceptibility to infections, longer viral shedding and greater duration of illness and severity of the disease. In individuals with obesity, maintaining a healthy diet, remaining physically active and reducing sedentary behaviors are particularly important during COVID-19-related quarantine to reduce metabolic and immune impairments. Moreover, such stategies are of utmost importance to reduce the risk for sarcopenia and sarcopenic obesity, and to prevent a reduction and potentially even increase cardiorespiratory fitness, a well-known independent risk factor for cardiovascular and metabolic diseases and recently found to be a risk factor also for hospitalizations secondary to COVID-19. Such lifestyle strategies may ultimately reduce morbility and mortality in patients with infectious disease, especially in those with concomitant obesity. The aim of this review is to discuss how obesity might increase the risk of COVID-19 and potentially affect its prognosis once COVID-19 is diagnosed. We therefore advocate for implementation of strategies aimed at preventing obesity in the first place, but also to minimize the metabolic anomalies that may lead to a compromized immune response and chronic low-grade systemic inflammation, especially in patients with COVID-19. © 2021 Elsevier Ltd and European Society for Clinical Nutrition and Metabolism},
author_keywords={COVID-19; Immune system; Low-grade inflammation; Obesity; Pandemic; Sars-CoV-2},
keywords={age; cardiorespiratory fitness; cardiovascular disease; China; comorbidity; coronavirus disease 2019; diabetes mellitus; diet; disease duration; disease severity; epidemic; food quality; France; home quarantine; hospitalization; human; hypertension; immune response; immune system; infection risk; infection sensitivity; inflammation; Italy; lung disease; metabolic disorder; obesity; pandemic; physical activity; prevalence; prognosis; psychoneuroimmunology; Review; risk factor; sedentary lifestyle; Severe acute respiratory syndrome coronavirus 2; United States; vitamin supplementation},
references={Hales, C.M., Carroll, M.D., Fryar, C.D., Ogden, C.L., Prevalence of obesity and severe obesity among adults: United States, 2017–2018. NCHS Data Brief, no 360 (2020), National Center for Health Statistics Hyattsville, MD; Preliminary estimates of the prevalence of selected underlying health conditions among patients with coronavirus disease 2019 — United States, February 12–march 28, 2020 (2020) MMWR Morb Mortal Wkly Rep, 69, pp. 382-386; Cummings, M.J., Baldwin, M.R., Abrams, D., Jacobson, S.D., Meyer, B.J., Balough, E.M., Epidemiology, clinical course, and outcomes of critically ill adults with COVID-19 in New York City: a prospective cohort study (2020) Lancet, 395 (10239), pp. 1763-1770; Ebinger, J.E., Achamallah, N., Ji, H., Sun,

N., Botting, P., Nguyen, T.T., Pre-existing traits associated with Covid-19 illness severity (2020) PloS One, 15 (7). , Published 2020 Jul 23; Steinberg, E., Wright, E., Kushner, B., Young adults with COVID-19, obesity is associated with adverse outcomes (2020) West J Emerg Med, 21 (4), pp. 752-755. , Published 2020 Jun 15; Palaiodimos, L., Kokkinidis, D.G., Li, W., Karamanis, D., Ognibene, J., Arora, S., Severe obesity, increasing age and male sex are independently associated with worse in-hospital outcomes, and higher in-hospital mortality, in a cohort of patients with COVID-19 in the Bronx, New York (2020) Metabolism, 108, p. 154262; Moriconi, D., Masi, S., Rebelos, E., Virdis, A., Manca, M.L., De Marco, S., Obesity prolongs the hospital stay in patients affected by COVID-19, and may impact on SARS-COV-2 shedding (2020) Obes Res Clin Pract, 14 (3), pp. 205-209; Suleyman, G., Fadel, R.A., Malette, K.M., Hammond, C., Abdulla, H., Entz, A., Clinical characteristics and morbidity associated with coronavirus disease 2019 in a series of patients in metropolitan detroit (2020) JAMA Netw Open, 3 (6). , Published 2020 Jun 1; Kalligeros, M., Shehadeh, F., Mylona, E.K., Benitez, G., Beckwith, C.G., Chan, P.A., Association of obesity with disease severity among patients with coronavirus disease 2019 (2020) Obesity, 28 (7), pp. 1200-1204; Cai, Q., Chen, F., Wang, T., Luo, F., Liu, X., Wu, Q., Obesity and COVID-19 severity in a designated hospital in shenzhen, China (2020) Diabetes Care, 43 (7), pp. 1392-1398; Ortiz-Brizuela, E., Villanueva-Reza, M., González-Lara, M.F., Tamez-Torres, K.M., Román-Montes, C.M., Díaz-Mejía, B.A., Clinical and epidemiological characteristics of patients diagnosed with covid-19 in A tertiary care center in Mexico city: a prospective cohort study (2020) Rev Invest Clin, 72 (3), pp. 165-177; Guan, W.J., Ni, Z.Y., Hu, Y., Liang, W.H., Ou, C.Q., He, J.X., Clinical characteristics of coronavirus disease 2019 in China (2020) N Engl J Med, 382 (18), pp. 1708-1720; Zhou, F., Yu, T., Du, R., Fan, G., Liu, Y., Liu, Z., Clinical course and risk factors for mortality of adult inpatients with COVID-19 in Wuhan, China: a retrospective cohort study (2020) Lancet, 395 (10229), pp. 1054-1062. , [published correction appears in Lancet. 2020 Mar 28;395(10229):1038]; Onder, G., Rezza, G., Brusaferro, S., Case-fatality rate and characteristics of patients dying in relation to COVID-19 in Italy (2020) J Am Med Assoc, , [published online ahead of print, 2020 Mar 23]; Garg, S., Kim, L., Whitaker, M., O'Halloran, A., Cummings, C., Holstein, R., Hospitalization rates and characteristics of patients hospitalized with laboratory-confirmed coronavirus disease 2019 - COVID-NET, 14 states, March 1-30, 2020 (2020) MMWR Morb Mortal Wkly Rep, 69 (15), pp. 458-464. , Published 2020 Apr 17; “Covid-Net, COVID-19-Associated hospitalization surveillance Network, centers for disease control and prevention. WEBSITE https://gis.cdc.gov/grasp/COVIDNet/COVID19_5.html, (Accessed 31 December 2020); Richardson, S., Hirsch, J.S., Narasimhan, M., Crawford, J.M., McGinn, T., Davidson, K.W., Presenting characteristics, comorbidities, and outcomes among 5700 patients hospitalized with COVID-19 in the New York city area (2020) J Am Med Assoc, , Erratum in: JAMA. 2020 May 26;323(20):2098. PMID: 32320003; PMCID: PMC7177629; Petrilli, C.M., Jones, S.A., Yang, J., Rajagopalan, H., O'Donnell, L., Chernyak, Y., Factors associated with hospital admission and critical illness among 5279 people with coronavirus disease 2019 in New York City: prospective cohort study (2020) BMJ, 369, p. m1966; Lighter, J., Phillips, M., Hochman, S., Sterling, S., Johnson, D., Francois, F., Obesity in patients younger than 60 years is a risk factor for Covid-19 hospital admission (2020) Clin Infect Dis; Simonnet, A., Chetboun, M., Poissy, J., Raverdy, V., Noulette, J., Duhamel, A., High prevalence of obesity in severe acute respiratory syndrome coronavirus-2 (SARS-CoV-2) requiring invasive mechanical ventilation (2020) Obesity, , Erratum in: Obesity

(Silver Spring). 2020 Oct;28(10):1994; Caussy, C., Wallet, F., Laville, M., Disse, E., Obesity is associated with severe forms of COVID-19 (2020) Obesity, , 10.1002/oby.22842; Zheng, K.I., Gao, F., Wang, X.B., Sun, Q.F., Pan, K.H., Wang, T.Y., Obesity as a risk factor for greater severity of COVID-19 in patients with metabolic associated fatty liver disease (2020) Metabolism, p. 154244; Peng, Y.D., Meng, K., Guan, H.Q., Leng, L., Zhu, R.R., Wang, B.Y., Clinical characteristics and outcomes of 112 cardiovascular disease patients infected by 2019-nCoV (2020) Zhonghua Xinxueguanbing Zazhi, 48, p. E004; https://www.epicentro.iss.it/en/coronavirus/bollettino/Report-COVID-2019_7_September_2020.pdf; Heialy, S.A., Hachim, M., Senok, A., Gaudet, M., Abou Tayoun, A., Hamoudi, R., Regulation of angiotensin converting enzyme 2 (ACE2) in obesity: implications for COVID-19 (2020) Front Physiol, 11 555039; Patel, A.B., Verma, A., COVID-19 and angiotensin-converting enzyme inhibitors and angiotensin receptor blockers: what is the evidence? (2020) J Am Med Assoc, , [published online ahead of print, 2020 Mar 24] 10.1001/jama.2020.4812; Honce, R., Schultz-Cherry, S., Impact of obesity on influenza A virus pathogenesis, immune response, and evolution (2019) Front Immunol, 10, p. 1071. , Published 2019 May 10; Maier, H.E., Lopez, R., Sanchez, N., Ng, S., Gresh, L., Ojeda, S., Obesity increases the duration of influenza A virus shedding in adults (2018) J Infect Dis, 218 (9), pp. 1378-1382; Andersen, C.J., Murphy, K.E., Fernandez, M.L., Impact of obesity and metabolic syndrome on immunity (2016) Adv Nutr, 7 (1), pp. 66-75. , Published 2016 Jan 15; Luzi, L., Radaelli, M.G., Influenza and obesity: its odd relationship and the lessons for COVID-19 pandemic (2020) Acta Diabetol, pp. 1-6; Ryan, P.M., Caplice, N.M., Is adipose tissue a reservoir for viral spread, immune activation and cytokine amplification in COVID-19 ? (2020) Obesity, , 10.1002/oby.22843; Movahed, M.R., Khoubyari, R., Hashemzadeh, M., Hashemzadeh, M., Obesity is strongly and independently associated with a higher prevalence of pulmonary embolism (2019) Respir Investig, 57 (4), pp. 376-379; Lavie, C.J., Ozemek, C., Carbone, S., Katzmarzyk, P.T., Blair, S.N., Sedentary behavior, exercise, and cardiovascular health (2019) Circ Res, 124 (5), pp. 799-815; Rundle, A.G., Park, Y., Herbstman, J.B., Kinsey, E.W., Wang, Y.C., COVID-19-Related school closings and risk of weight gain among children (2020) Obesity, , 10.1002/oby.22813; Qiu, J., Shen, B., Zhao, M., Wang, Z., Xie, B., Xu, Y., A nationwide survey of psychological distress among Chinese people in the COVID-19 epidemic: implications and policy recommendations (2020) Gen Psychiatr, 33 (2). , Published 2020 Mar 6; Huang, Y., Zhao, N., Generalized anxiety disorder, depressive symptoms and sleep quality during COVID-19 outbreak in China: a web-based cross-sectional survey (2020) Psychiatr Res, 288, p. 112954; Nieman, D.C., Wentz, L.M., The compelling link between physical activity and the body's defense system (2019) J Sport Health Sci, 8 (3), pp. 201-217; Zheng, Q., Cui, G., Chen, J., Gao, H., Wei, Y., Uede, T., Regular exercise enhances the immune response against microbial antigens through up-regulation of toll-like receptor signaling pathways (2015) Cell Physiol Biochem, 37 (2), pp. 735-746; Reidy, P.T., Yonemura, N.M., Madsen, J.H., McKenzie, A.L., Mahmassani, Z.S., Rondina, M.T., An accumulation of muscle macrophages is accompanied by altered insulin sensitivity after reduced activity and recovery (2019) Acta Physiol, 226 (2); Wong, G.C.L., Narang, V., Lu, Y., Camous, X., Nyunt, M.S.Z., Carre, C., Hallmarks of improved immunological responses in the vaccination of more physically active elderly females (2019) Exerc Immunol Rev, 25, pp. 20-33; Nieman, D.C., Wentz, L.M., The compelling link between physical activity and the body's defense system (2019) J Sport Health Sci, 8 (3), pp. 201-217; Beaudart, C., Dawson, A., Shaw, S.C., Harvey, N.C., Kanis, J.A., Binkley, N., Nutrition and physical activity in the prevention and treatment of sarcopenia:

systematic review (2017) Osteoporos Int, 28 (6), pp. 1817-1833; Landi, F., Calvani, R., Picca, A., Tosato, M., Martone, A.M., D'Angelo, E., Impact of habitual physical activity and type of exercise on physical performance across ages in community-living people (2018) PloS One, 13 (1). , Published 2018 Jan 25; Tate, D.F., Lyons, E.J., Valle, C.G., High-tech tools for exercise motivation: use and role of technologies such as the internet, mobile applications, social media, and video games (2015) Diabetes Spectr, 28 (1), pp. 45-54; Chen, P., Mao, L., Nassis, G.P., Harmer, P., Ainsworth, B.E., Li, F., Coronavirus disease (COVID-19): the need to maintain regular physical activity while taking precautions (2020) J Sport Health Sci, 9 (2), pp. 103-104; Codella, R., Luzi, L., Inverardi, L., Ricordi, C., The anti-inflammatory effects of exercise in the syndromic thread of diabetes and autoimmunity (2015) Eur Rev Med Pharmacol Sci, 19 (19), pp. 3709-3722; Barazzoni, R., Bischoff, S.C., Breda, J., Wickramasinghe, K., Krznaric, Z., Nitzan, D., ESPEN expert statements and practical guidance for nutritional management of individuals with SARS-CoV-2 infection (2020) Clin Nutr, , S0261-5614(20)30140-0; Frühbeck, G., Baker, J.L., Busetto, L., Dicker, D., Goossens, G.H., Halford, J.C.G., European association for the study of obesity position statement on the global COVID-19 pandemic (2020) Obes Facts, pp. 1-5; Dantzer, R., Neuroimmune interactions: from the brain to the immune system and vice versa (2018) Physiol Rev, 98 (1), pp. 477-504; Kim, S.W., Su, K.P., Using psychoneuroimmunity against COVID-19 (2020) Brain Behav Immun, , S0889-1591(20)30391-30393; Ryan, D.H., Ravussin, E., Heymsfield, S., COVID 19 and the patient with obesity - the editors speak out (2020) Obesity, 28 (5), p. 847; Brooks, S.K., Webster, R.K., Smith, L.E., Woodland, L., Wessely, S., Greenberg, N., The psychological impact of quarantine and how to reduce it: rapid review of the evidence (2020) Lancet, 395 (10227), pp. 912-920; Martineau, A.R., Jolliffe, D.A., Hooper, R.L., Greenberg, L., Aloia, J.F., Bergman, P., Vitamin D supplementation to prevent acute respiratory tract infections: systematic review and meta-analysis of individual participant data (2017) BMJ, 356, p. i6583. , Published 2017 Feb 15; Billingsley, H.E., Carbone, S., Lavie, C.J., Dietary fats and chronic noncommunicable diseases (2018) Nutrients, 10 (10), p. 1385. , Published 2018 Sep 30; Cava, E., Carbone, S., Coronavirus disease 2019 pandemic and alterations of body composition (2021) Curr Opin Clin Nutr Metab Care},
correspondence_address1={Cava, E.; AOU Maggiore della Carità di Novara, Corso Mazzini 18, Italy; email: edda.cava@maggioreosp.novara.it},
publisher={Churchill Livingstone},
issn={02615614},
coden={CLNUD},
language={English},
abbrev_source_title={Clin. Nutr.},
document_type={Review},
source={Scopus},
}

@ARTICLE{Julien2021,
author={Julien, C.A. and Lavoie, K.L. and Ribeiro, P.A.B. and Dragomir, A.I. and Mercier, L.A. and Garneau, P.Y. and Pescarus, R. and Bacon, S.L.},
title={Behavioral weight management interventions in metabolic and bariatric surgery: A systematic review and meta-analysis investigating optimal delivery timing},
journal={Obesity Reviews},
year={2021},
volume={22},

number={4},
doi={10.1111/obr.13168},
art_number={e13168},
note={cited By 0},
url={https://www.scopus.com/inward/record.uri?eid=2-s2.0-85099051089&doi=10.1111%2fobr.13168&partnerID=40&md5=e92949b0c54ab98297326f5b7b6fa72c},
affiliation={Department of Psychology, University of Quebec at Montreal, Montréal, Canada; Montreal Behavioural Medicine Centre, Montreal North Island Integrated Health and Social Services University Centre (CIUSSS-NIM), Montréal, Canada; Department of Surgery, University of Montreal, Montréal, Canada; General and Bariatric Surgery Division, Montreal North Island Integrated Health and Social Services University Centre (CIUSSS-NIM), Montréal, Canada; Department of Health, Kinesiology & Applied Physiology, Concordia University, Montréal, Canada},
abstract={Metabolic and bariatric surgery (MBS) yields unprecedented clinical outcomes, though variability is high in weight change and health benefits. Behavioral weight management (BWM) interventions may optimize MBS outcomes. However, there is a lack of an evidence base to inform their use in practice, particularly regarding optimal delivery timing. This paper evaluated the efficacy of BWM conducted pre- versus post- versus pre- and post-MBS. The review followed the Preferred Reporting Items for Systematic Reviews and Meta-Analyses statement and included pre- and/or post-operative BWM interventions in adults reporting anthropometric and/or body composition data. Thirty-six studies (2,919 participants) were included. Post-operative BWM yielded greater decreases in weight (standardized mean difference [SMD] = −0.41; 95% confidence interval [CI]: −0.766 to −0.049, p < 0.05; I2 = 93.5%) and body mass index (SMD = −0.60; 95% CI: −0.913 to −0.289, p < 0.001; I2 = 87.8%) relative to comparators. There was no effect of BWM delivered pre- or joint pre- and post-operatively. The risk of selection and performance bias was generally high. Delivering BWM after MBS appears to confer the most benefits on weight, though there was high variability in study characteristics and risk of bias across trials. This provides insight into the type of support that should be considered post-operatively. © 2021 World Obesity Federation},
author_keywords={behavioral weight management; BMI; metabolic and bariatric surgery; weight},
keywords={adult; bariatric surgery; body composition; body mass; body weight loss; comparative effectiveness; female; human; male; meta analysis; Preferred Reporting Items for Systematic Reviews and Meta-Analyses; review; systematic review},
references={Sogg, S., Atwood, M.E., Cassin, S.E., The role of psychosocial interventions in supporting medical and surgical treatments for severe obesity (2018) Psychological Care in Severe Obesity: A Practical and Integrated Approach, pp. 18-41. , Cassin SE, Hawa R, Sockalingam S, eds., Cambridge, United Kingdom, Cambridge University Press; Wharton, S., Lau, D.C.W., Vallis, M., Obesity in adults: a clinical practice guideline (2020) CMAJ, 192 (31), pp. E875-e891; Report card on access to obesity treatment for adults in Canada 2019, , https://obesitycanada.ca/wp-content/uploads/2019/04/OC-Report-Card-2019-Eng-F-web.pdf, Published April, 2019. Accessed July 29, 2020; Booth, H.P., Prevost, A.T., Gulliford, M.C., Access to weight reduction interventions for overweight and obese patients in UK primary care: population-based cohort study (2015) BMJ Open, 5 (1). , https://doi.org/10.1136/bmjopen-2014-006642; Block, J.P., DeSalvo, K.B., Fisher, W.P., Are physicians equipped to address the obesity epidemic? Knowledge and attitudes of internal medicine residents (2003) Prev

Med, 36 (6), pp. 669-675; O'Brien, P.E., Hindle, A., Brennan, L., Long-term outcomes after bariatric surgery: a systematic review and meta-analysis of weight loss at 10 or more years for all bariatric procedures and a single-centre review of 20-year outcomes after adjustable gastric banding (2019) Obes Surg, 29 (1), pp. 3-14; Mechanick, J.I., Apovian, C., Brethauer, S., Clinical practice guidelines for the perioperative nutrition, metabolic, and nonsurgical support of patients undergoing bariatric procedures—2019 update: cosponsored by American Association of Clinical Endocrinologists/American College of Endocrinology, The Obesity Society, American Society for Metabolic & Bariatric Surgery, Obesity Medicine Association, and American Society of Anesthesiologists—executive summary (2019) Endocr Pract, 25 (12), pp. 1346-1359; Kubik, J.F., Gill, R.S., Laffin, M., Karmali, S., The Impact of Bariatric Surgery on Psychological Health (2013) Journal Obes, 2013, p. 837989. , https://doi.org/10.1155/2013/837989; Sharples, A.J., Mahawar, K., Systematic review and meta-analysis of randomised controlled trials comparing long-term outcomes of Roux-en-Y gastric bypass and sleeve gastrectomy (2020) Obes Surg, 30 (2), pp. 664-672; Doumouras, A.G., Hong, D., Lee, Y., Tarride, J.E., Paterson, J.M., Anvari, M., Association between bariatric surgery and all-cause mortality: a population-based matched cohort study in a universal health care system (2020) Ann Intern Med, 173 (9), pp. 694-703. , https://doi.org/10.7326/M19-3925; Chang, S.H., Stoll, C.R., Song, J., Varela, J.E., Eagon, C.J., Colditz, G.A., The effectiveness and risks of bariatric surgery: an updated systematic review and meta-analysis, 2003–2012 (2014) JAMA Surg, 149 (3), pp. 275-287; Kang, J.H., Le, Q.A., Effectiveness of bariatric surgical procedures: A systematic review and network meta-analysis of randomized controlled trials (2017) Medicine, 96 (46). , https://doi.org/10.1097/md.0000000000008632; Wolfe, B.M., Kvach, E., Eckel, R.H., Treatment of obesity: weight loss and bariatric surgery (2016) Circ Res, 118 (11), pp. 1844-1855; Courcoulas, A.P., Christian, N.J., Belle, S.H., Weight change and health outcomes at 3 years after bariatric surgery among individuals with severe obesity (2013) JAMA, 310 (22), pp. 2416-2425; Sjostrom, L., Review of the key results from the Swedish Obese Subjects (SOS) trial—a prospective controlled intervention study of bariatric surgery (2013) J Intern Med, 273 (3), pp. 219-234; King, W.C., Hinerman, A.S., Belle, S.H., Wahed, A.S., Courcoulas, A.P., Comparison of the performance of common measures of weight regain after bariatric surgery for association with clinical outcomes (2018) JAMA, 320 (15), pp. 1560-1569; Suter, M., Calmes, J.M., Paroz, A., Giusti, V., A 10-year experience with laparoscopic gastric banding for morbid obesity: high long-term complication and failure rates (2006) Obes Surg, 16 (7), pp. 829-835; Cadena-Obando, D., Ramírez-Rentería, C., Ferreira-Hermosillo, A., Are there really any predictive factors for a successful weight loss after bariatric surgery? (2020) BMC Endocrine Disorders, 20 (1). , https://doi.org/10.1186/s12902-020-0499-4; Voorwinde, V., Steenhuis, I.H.M., Janssen, I.M.C., Monpellier, V.M., van Stralen, M.M., Definitions of long-term weight regain and their associations with clinical outcomes (2020) Obes Surg, 30 (2), pp. 527-536; Christou, N.V., Look, D., Maclean, L.D., Weight gain after short- and long-limb gastric bypass in patients followed for longer than 10 years (2006) Ann Surg, 244 (5), pp. 734-740; Goodpaster, B.H., Delany, J.P., Otto, A.D., Effects of diet and physical activity interventions on weight loss and cardiometabolic risk factors in severely obese adults: a randomized trial (2010) JAMA, 304 (16), pp. 1795-1802; Jacob, A., Moullec, G., Lavoie, K.L., Impact of cognitive-behavioral interventions on weight loss and psychological outcomes: a meta-analysis (2018) Health Psychol, 37 (5), pp. 417-432; Stewart, F., Avenell, A., Behavioural interventions for severe obesity before and/or

after bariatric surgery: a systematic review and meta-analysis (2016) Obes Surg, 26 (6), pp. 1203-1214; David, L.A., Sijercic, I., Cassin, S.E., Preoperative and post-operative psychosocial interventions for bariatric surgery patients: A systematic review (2020) Obesity Reviews, 21 (4). , https://doi.org/10.1111/obr.12926; Liu, R.H., Do behavioral interventions delivered before bariatric surgery impact weight loss in adults? A systematic scoping review (2016) Bariatr Surg Pract P, 11 (2), pp. 39-48; Egberts, K., Brown, W.A., Brennan, L., O'Brien, P.E., Does exercise improve weight loss after bariatric surgery? A systematic review (2012) Obes Surg, 22 (2), pp. 335-341; Livhits, M., Mercado, C., Yermilov, I., Exercise following bariatric surgery: systematic review (2010) Obes Surg, 20 (5), pp. 657-665; Groller, K.D., Systematic review of patient education practices in weight loss surgery (2017) Surg Obes Relat Dis, 13 (6), pp. 1072-1085; Beck, N.N., Johannsen, M., Stoving, R.K., Mehlsen, M., Zachariae, R., Do postoperative psychotherapeutic interventions and support groups influence weight loss following bariatric surgery? A systematic review and meta-analysis of randomized and nonrandomized trials (2012) Obes Surg, 22 (11), pp. 1790-1797; Livhits, M., Mercado, C., Yermilov, I., Is social support associated with greater weight loss after bariatric surgery?: a systematic review (2011) Obes Rev, 12 (2), pp. 142-148; Gerber, P., Anderin, C., Thorell, A., Weight loss prior to bariatric surgery: an updated review of the literature (2015) Scand J Surg, 104 (1), pp. 33-39; Rudolph, A., Hilbert, A., Post-operative behavioural management in bariatric surgery: a systematic review and meta-analysis of randomized controlled trials (2013) Obes Rev, 14 (4), pp. 292-302; Moher, D., Liberati, A., Tetzlaff, J., Altman, D.G., Preferred Reporting Items for Systematic Reviews and Meta-Analyses: the PRISMA statement (2010) Int J Surg (London, England), 8 (5), pp. 336-341; Daniels, P., Burns, R.D., Brusseau, T.A., Effect of a randomised 12-week resistance training programme on muscular strength, cross-sectional area and muscle quality in women having undergone Roux-en-Y gastric bypass (2018) J Sports Sci, 36 (5), pp. 529-535; Baillot, A., Mampuya, W.M., Dionne, I.J., Comeau, E., Meziat-Burdin, A., Langlois, M.F., Impacts of supervised exercise training in addition to interdisciplinary lifestyle management in subjects awaiting bariatric surgery: a randomized controlled study (2016) Obes Surg, 26 (11), pp. 2602-2610; Stegen, S., Derave, W., Calders, P., Van Laethem, C., Pattyn, P., Physical fitness in morbidly obese patients: effect of gastric bypass surgery and exercise training (2011) Obes Surg, 21 (1), pp. 61-70; Higgins, J.P.T., Altman, D.G., Sterne, J.A.C., Chapter 8: Assessing the risk of bias in included studies (2017) Cochrane Handbook for Systematic Reviews of Interventions, , http://www.training.cochrane.org/handbook, (Eds)., Higgins JPT, Churchill R, Chandler J, Cumpston MS, ed. In, version 5.0.2., Cochrane, Accessed July, 2020; (2015) Comprehensive Meta-analysis (Version 3) [computer program], , Englewood, NJ Biostat; Deeks, J.J., Higgins, J.P.T., Altman, D.G., Chapter 9: Analysing data and undertaking meta-analyses (2017) Cochrane Handbook for Systematic Reviews of Interventions, , http://www.training.cochrane.org/handbook, (Eds) on behalf of the Cochrane Statistical Methods Group., Higgins JPT, Churchill R, Chandler J, Cumpston MS, ed. In, version 5.2.0 (updated June 2017)., Chichester, UK, Cochrane, Accessed July, 2020; Higgins, J.P., Thompson, S.G., Deeks, J.J., Altman, D.G., Measuring inconsistency in meta-analyses (2003) BMJ (Clinical Research Ed), 327 (7414), pp. 557-560; Kalarchian, M.A., Marcus, M.D., Courcoulas, A.P., Cheng, Y., Levine, M.D., Preoperative lifestyle intervention in bariatric surgery: initial results from a randomized, controlled trial (2013) Obesity (Silver Spring, md), 21 (2), pp. 254-260; Kalarchian, M.A., Marcus, M.D., Courcoulas, A.P., Cheng, Y., Levine, M.D., Preoperative lifestyle intervention in bariatric surgery: a

randomized clinical trial (2016) Surg Obes Relat Dis, 12 (1), pp. 180-187; Camolas, J., Santos, O., Moreira, P., do Carmo, I., INDIVIDUO: results from a patient-centered lifestyle intervention for obesity surgery candidates (2017) Obes Res Clin Pract, 11 (4), pp. 475-488. , https://doi.org/10.1016/j.orcp.2016.08.003; Bond, D.S., Vithiananthan, S., Thomas, J.G., Bari-Active: a randomized controlled trial of a preoperative intervention to increase physical activity in bariatric surgery patients (2015) Surg Obes Relat Dis, 11 (1), pp. 169-177; Bond, D., Thomas, J., Vithiananthan, S., Intervention-related increases in preoperative physical activity are maintained 6-months after bariatric surgery: results from the Bari-Active trial (2017) Int J Obes (Lond), 41 (3), pp. 467-470; Gade, H., Hjelmesæth, J., Rosenvinge, J.H., Friborg, O., Effectiveness of a Cognitive Behavioral Therapy for Dysfunctional Eating among Patients Admitted for Bariatric Surgery: A Randomized Controlled Trial (2014) Journal Obes, 2014, pp. 1-6. , https://doi.org/10.1155/2014/127936; Gade, H., Friborg, O., Rosenvinge, J.H., Småstuen, M.C., Hjelmesæth, J., The impact of a preoperative cognitive behavioural therapy (CBT) on dysfunctional eating behaviours, affective symptoms and body weight 1 year after bariatric surgery: a randomised controlled trial (2015) Obes Surg, 25 (11), pp. 2112-2119; Hjelmesæth, J., Rosenvinge, J.H., Gade, H., Friborg, O., Effects of cognitive behavioral therapy on eating behaviors, affective symptoms, and weight loss after bariatric surgery: a randomized clinical trial (2019) Obes Surg, 29 (1), pp. 61-69; Parikh, M., Dasari, M., McMacken, M., Ren, C., Fielding, G., Ogedegbe, G., Does a preoperative medically supervised weight loss program improve bariatric surgery outcomes? A pilot randomized study (2012) Surg Endosc, 26 (3), pp. 853-861; Heinberg, L.J., Schauer, P.R., Pilot testing of a portion-controlled, commercially available diet on presurgical weight loss and metabolic outcomes in patients undergoing bariatric surgery (2014) Obes Surg, 24 (10), pp. 1817-1820; Marcon, E.R., Baglioni, S., Bittencourt, L., Lopes, C.L.N., Neumann, C.R., Trindade, M.R.M., What is the best treatment before bariatric surgery? Exercise, exercise and group therapy, or conventional waiting: a randomized controlled trial (2017) Obes Surg, 27 (3), pp. 763-773; Lemanu, D.P., Singh, P.P., Shao, R.Y., Text messaging improves preoperative exercise in patients undergoing bariatric surgery (2018) ANZ J Surg, 88 (7-8), pp. 733-738. , https://doi.org/10.1111/ans.14418; Marc-Hernandez, A., Ruiz-Tovar, J., Aracil, A., Guillen, S., Moya-Ramon, M., Impact of exercise on body composition and cardiometabolic risk factors in patients awaiting bariatric surgery (2019) Obes Surg, 29 (12), pp. 3891-3900; Kalarchian, M.A., Marcus, M.D., Courcoulas, A.P., Cheng, Y., Levine, M.D., Josbeno, D., Optimizing long-term weight control after bariatric surgery: a pilot study (2012) Surg Obes Relat Dis, 8 (6), pp. 710-715; Kalarchian, M., Marcus, M., Courcoulas, A., Lutz, C., Cheng, Y., Sweeny, G., Structured dietary intervention to facilitate weight loss after bariatric surgery: a randomized, controlled pilot study (2016) Obesity (Silver Spring, MD), 24 (9), pp. 1906-1912; Chacko, S.A., Yeh, G.Y., Davis, R.B., Wee, C.C., A mindfulness-based intervention to control weight after bariatric surgery: preliminary results from a randomized controlled pilot trial (2016) Complement Ther Med, 28, pp. 13-21; Dodsworth, A., Warren-Forward, H., Baines, S., Feasibility of a protein-enriched diet after laparoscopic adjustable gastric banding: results from a pilot intervention (2012) E-SPEN, 7 (2), pp. 57-63. , https://doi.org/10.1016/j.clnme.2012.02.004; Gallé, F., Maida, P., Cirella, A., Giuliano, E., Belfiore, P., Liguori, G., Does post-operative psychotherapy contribute to improved comorbidities in bariatric patients with borderline personality disorder traits and bulimia tendencies? A prospective study (2017)

Obes Surg, pp. 1-7; Nijamkin, M.P., Campa, A., Sosa, J., Baum, M., Himburg, S., Johnson, P., Comprehensive nutrition and lifestyle education improves weight loss and physical activity in Hispanic Americans following gastric bypass surgery: a randomized controlled trial (2012) J Acad Nutr Diet, 112 (3), pp. 382-390; Marchesi, F., De Sario, G., Reggiani, V., Road running after gastric bypass for morbid obesity: rationale and results of a new protocol (2015) Obes Surg, 25 (7), pp. 1162-1170; Papalazarou, A., Yannakoulia, M., Kavouras, S.A., Lifestyle intervention favorably affects weight loss and maintenance following obesity surgery (2010) Obesity (Silver Spring, MD), 18 (7), pp. 1348-1353; Sarwer, D.B., Moore, R.H., Spitzer, J.C., Wadden, T.A., Raper, S.E., Williams, N.N., A pilot study investigating the efficacy of postoperative dietary counseling to improve outcomes after bariatric surgery (2012) Surg Obes Relat Dis, 8 (5), pp. 561-568; Coen, P.M., Tanner, C.J., Helbling, N.L., Clinical trial demonstrates exercise following bariatric surgery improves insulin sensitivity (2015) J Clin Invest, 125 (1), pp. 248-257; Shah, M., Snell, P.G., Rao, S., High-volume exercise program in obese bariatric surgery patients: a randomized, controlled trial (2011) Obesity, 19 (9), pp. 1826-1834; Wild, B., Hünnemeyer, K., Sauer, H., A 1-year videoconferencing-based psychoeducational group intervention following bariatric surgery: results of a randomized controlled study (2015) Surg Obes Relat Dis, 11 (6), pp. 1349-1360; Wild, B., Hunnemeyer, K., Sauer, H., Sustained effects of a psychoeducational group intervention following bariatric surgery: follow-up of the randomized controlled BaSE study (2017) Surg Obes Relat Dis, 13 (9), pp. 1612-1618; Hassannejad, A., Khalaj, A., Mansournia, M.A., Rajabian Tabesh, M., Alizadeh, Z., The effect of aerobic or aerobic-strength exercise on body composition and functional capacity in patients with BMI ≥35 after bariatric surgery: a randomized control trial (2017) Obes Surg, pp. 1-10; Campanha-Versiani, L., Pereira, D.A.G., Ribeiro-Samora, G.A., The effect of a muscle weight-bearing and aerobic exercise program on the body composition, muscular strength, biochemical markers, and bone mass of obese patients who have undergone gastric bypass surgery (2017) Obes Surg, pp. 1-9; Herring, L., Stevinson, C., Carter, P., The effects of supervised exercise training 12–24 months after bariatric surgery on physical function and body composition: a randomised controlled trial (2017) Int J Obes (Lond), 41 (6), pp. 909-916; Tucker, J.A., Samo, J.A., Rand, C.S.W., Woodward, E.R., Behavioral interventions to promote adaptive eating behavior and lifestyle changes following surgery for obesity: results of a two-year outcome evaluation (1991) Int J Eat Disord, 10 (6), pp. 689-698. , https://doi.org/10.1002/1098-108X(199111)10:6<689::AID-EAT2260100607>3.0.CO;2–6 ; Huck, C.J., Effects of supervised resistance training on fitness and functional strength in patients succeeding bariatric surgery (2015) J Strength Cond Res, 29 (3), pp. 589-595; Mangieri, C.W., Johnson, R.J., Sweeney, L.B., Choi, Y.U., Wood, J.C., Mobile health applications enhance weight loss efficacy following bariatric surgery (2019) Obes Res Clin Pract, 13 (2), pp. 176-179; Gallé, F., Cirella, A., Salzano, A.M., Di Onofrio, V., Belfiore, P., Liguori, G., Analyzing the effects of psychotherapy on weight loss after laparoscopic gastric bypass or laparoscopic adjustable gastric banding in patients with borderline personality disorder: a prospective study (2017) Scand J Surg, 106 (4), pp. 299-304; Mundbjerg, L.H., Stolberg, C.R., Cecere, S., Supervised physical training improves weight loss after Roux-en-Y gastric bypass surgery: a randomized controlled trial (2018) Obesity (Silver Spring, md), 26 (5), pp. 828-837; Lauti, M., Kularatna, M., Pillai, A., Hill, A.G., MacCormick, A.D., A randomised trial of text message support for reducing weight regain following sleeve gastrectomy (2018) Obes Surg, 28 (8), pp. 2178-2186; Lent, M.R., Campbell, L.K., Kelly, M.C., The feasibility of a behavioral group intervention

after weight-loss surgery: A randomized pilot trial (2019) PLoS One, 14 (10). , https://doi.org/10.1371/journal.pone.0223885; Hanvold, S.E., Vinknes, K.J., Loken, E.B., Does lifestyle intervention after gastric bypass surgery prevent weight regain? A randomized clinical trial (2019) Obes Surg, 29 (11), pp. 3419-3431; Ogden, J., Hollywood, A., Pring, C., The impact of psychological support on weight loss post weight loss surgery: a randomised control trial (2015) Obes Surg, 25 (3), pp. 500-505; Creel, D.B., Schuh, L.M., Reed, C.A., A randomized trial comparing two interventions to increase physical activity among patients undergoing bariatric surgery (2016) Obesity, 24 (8), pp. 1660-1668; Lier, H., Biringer, E., Stubhaug, B., Tangen, T., The impact of preoperative counseling on postoperative treatment adherence in bariatric surgery patients: a randomized controlled trial (2012) Patient Educ Couns, 87 (3), pp. 336-342; Swenson, B.R., Saalwachter Schulman, A., Edwards, M.J., The effect of a low-carbohydrate, high-protein diet on post laparoscopic gastric bypass weight loss: a prospective randomized trial (2007) J Surg Res, 142 (2), pp. 308-313; Kalarchian, M.A., Marcus, M.D., Psychosocial interventions pre and post bariatric surgery (2015) Eur Eat Disord Rev, 23 (6), pp. 457-462; Kalarchian, M., Turk, M., Elliott, J., Gourash, W., Lifestyle Management for Enhancing Outcomes after Bariatric Surgery (2014) Curr Diab Rep, 14 (10). , https://doi.org/10.1007/s11892-014-0540-y, 540; Leahey, T.M., Bond, D.S., Irwin, S.R., Crowther, J.H., Wing, R.R., When is the best time to deliver behavioral intervention to bariatric surgery patients: before or after surgery? (2009) Surg Obes Relat Dis, 5 (1), pp. 99-102; Groven, K.S., Glenn, N.M., The experience of regaining weight following weight loss surgery: a narrative-phenomenological exploration (2016) Health Care Women Int, 37 (11), pp. 1185-1202; Lynch, A., “When the honeymoon is over, the real work begins”: gastric bypass patients' weight loss trajectories and dietary change experiences (2016) Soc Sci Med, 151, pp. 241-249; Schulz, K.B., Bernhofer, E.I., Satava, M.E., Patient perception of surgical preparation and recovery following bariatric surgery: a phenomenological study (2019) Bariatric Times: Clin Dev Metab Insights Total Bariatric pat Care, 16 (12), pp. 16-19; Mechanick, J.I., Youdim, A., Jones, D.B., Clinical practice guidelines for the perioperative nutritional, metabolic, and nonsurgical support of the bariatric surgery patient—2013 update: Cosponsored by American Association of Clinical Endocrinologists, The Obesity Society, and American Society for Metabolic & Bariatric Surgery (2013) Obesity (Silver Spring, MD), 21, pp. S1-S27; Heber, D., Greenway, F.L., Kaplan, L.M., Livingston, E., Salvador, J., Still, C., Endocrine and nutritional management of the post-bariatric surgery patient: an Endocrine Society Clinical Practice Guideline (2010) J Clin Endocrinol Metab, 95 (11), pp. 4823-4843; Jumbe, S., Meyrick, J., Contrasting views of the post-bariatric surgery experience between patients and their practitioners: a qualitative study (2018) Obes Surg, 28 (8), pp. 2447-2456; Coulman, K.D., MacKichan, F., Blazeby, J.M., Donovan, J.L., Owen-Smith, A., Patients’ experiences of life after bariatric surgery and follow-up care: a qualitative study (2020) BMJ Open, 10 (2). , https://doi.org/10.1136/bmjopen-2019-035013; Rosa Fortin, M.M., Brown, C., Ball, G.D., Chanoine, J.P., Langlois, M.F., Weight management in Canada: an environmental scan of health services for adults with obesity (2014) BMC Health Serv Res, 14 (1), p. 69. , https://doi.org/10.1186/1472-6963-14-69; Parretti, H.M., Hughes, C.A., Jones, L.L., The rollercoaster of follow-up care’ after bariatric surgery: a rapid review and qualitative synthesis (2019) Obes Rev, 20 (1), pp. 88-107; Altman, D.G., Schulz, K.F., Moher, D., The revised CONSORT statement for reporting randomized trials: explanation and elaboration (2001) Ann Intern Med, 134 (8), pp. 663-694; Bacon, S.L., Campbell, T.S., Lavoie, K.L., Rethinking how to

expand the evidence base for health behavior change in cardiovascular disease prevention (2020) J am Coll Cardiol, 75 (20), pp. 2619-2622; Bartholomew, L.K., Mullen, P.D., Five roles for using theory and evidence in the design and testing of behavior change interventions (2011) J Public Health Dent, 71, pp. S20-S33; Bluethmann, S.M., Bartholomew, L.K., Murphy, C.C., Vernon, S.W., Use of theory in behavior change interventions (2017) Health Educ Behav, 44 (22), pp. 245-253; Davis, R., Campbell, R., Hildon, Z., Hobbs, L., Michie, S., Theories of behaviour and behaviour change across the social and behavioural sciences: a scoping review (2015) Health Psychol Rev, 9 (3), pp. 323-344; Swift, D.L., Johannsen, N.M., Lavie, C.J., Earnest, C.P., Blair, S.N., Church, T.S., Effects of clinically significant weight loss with exercise training on insulin resistance and cardiometabolic adaptations (2016) Obesity (Silver Spring, MD), 24 (4), pp. 812-819; Turk, M.W., Yang, K., Hravnak, M., Sereika, S.M., Ewing, L.J., Burke, L.E., Randomized clinical trials of weight loss maintenance: a review (2009) J Cardiovasc Nurs, 24 (1), pp. 58-80; Neter, J.E., Stam, B.E., Kok, F.J., Grobbee, D.E., Geleijnse, J.M., Influence of weight reduction on blood pressure: a meta-analysis of randomized controlled trials (2003) Hypertension, 42 (5), pp. 878-884; Williamson, D.A., Bray, G.A., Ryan, D.H., Is 5% weight loss a satisfactory criterion to define clinically significant weight loss? (2015) Obesity (Silver Spring, MD), 23 (12), pp. 2319-2320; Czajkowski, S.M., Powell, L.H., Adler, N., From ideas to efficacy: the ORBIT model for developing behavioral treatments for chronic diseases (2015) Health Psychol, 34 (10), pp. 971-982; Craig, P., Dieppe, P., Macintyre, S., Michie, S., Nazareth, I., Petticrew, M., Developing and evaluating complex interventions: the new Medical Research Council guidance (2013) Int J Nurs Stud, 50 (5), pp. 587-592; Skea, Z.C., Aceves-Martins, M., Robertson, C., De Bruin, M., Avenell, A., Acceptability and feasibility of weight management programmes for adults with severe obesity: a qualitative systematic review (2019) BMJ Open, 9 (9). , https://doi.org/10.1136/bmjopen-2019-029473; Des Jarlais, D.C., Lyles, C., Crepaz, N., Improving the reporting quality of nonrandomized evaluations of behavioral and public health interventions: the TREND statement (2004) Am J Public Health, 94 (3), pp. 361-366; Michie, S., van Stralen, M.M., West, R., The behaviour change wheel: A new method for characterising and designing behaviour change interventions (2011) Implement Sci, 6 (42), p. 42. , https://doi.org/10.1186/1748-5908-6-42; O'Kane, M., Parretti, H.M., Hughes, C.A., Guidelines for the follow-up of patients undergoing bariatric surgery (2016) Clin Obes, 6 (3), pp. 210-224; Lemstra, M., Bird, Y., Nwankwo, C., Rogers, M., Moraros, J., Weight loss intervention adherence and factors promoting adherence: a meta-analysis (2016) Patient Prefer Adherence, 10, pp. 1547-1559},
correspondence_address1={Bacon, S.L.; Montreal Behavioural Medicine Centre, Canada; email: simon.bacon@concordia.ca; Bacon, S.L.; Department of Health, Canada; email: simon.bacon@concordia.ca},
publisher={Blackwell Publishing Ltd},
issn={14677881},
coden={ORBEB},
pubmed_id={33403754},
language={English},
abbrev_source_title={Obes. Rev.},
document_type={Review},
source={Scopus},
}

@ARTICLE{Bautmans2021,

author={Bautmans, I. and Salimans, L. and Njemini, R. and Beyer, I. and Lieten, S. and Liberman, K.},
title={The effects of exercise interventions on the inflammatory profile of older adults: A systematic review of the recent literature},
journal={Experimental Gerontology},
year={2021},
volume={146},
doi={10.1016/j.exger.2021.111236},
art_number={111236},
note={cited By 0},
url={https://www.scopus.com/inward/record.uri?eid=2-s2.0-85099456015&doi=10.1016%2fj.exger.2021.111236&partnerID=40&md5=d0a1d8142b7b00cd798df67e69afaac6},
affiliation={Frailty in Ageing Research Group, Vrije Universiteit Brussel, Laarbeeklaan 103, Brussels, 1090, Belgium; Gerontology Department, Vrije Universiteit Brussel, Laarbeeklaan 103, Brussels, 1090, Belgium; Geriatrics Department, Universitair Ziekenhuis Brussel, Laarbeeklaan 10&, Brussels, 1090, Belgium},
abstract={Introduction: Inflammageing – characterized by age-related chronic low-grade inflammation is considered to be positively influenced by physical exercises. The aim of this systematic review is to provide an update of the most recent literature regarding exercise effects on the inflammatory profile in older adults. Methods: This review is an update of an earlier published literature review and was performed according to the NICE guidelines. Databases PubMed and Web-of-Science were systematically searched by two independent authors screening for papers published since 2016. Effect sizes of outcome parameters related to the inflammatory profile were calculated where possible. Results & discussion: Twenty-three articles were included. Resistance training (RT) was the most investigated type of exercise (13 articles: 8 in healthy, 1 in frail and 4 in older adults with a specific condition or disease). Aerobic training (AT) was investigated in 8 articles, including 5 studies in older adults with a specific disease or condition. Combined resistance & aerobic training (CT) was investigated in 7 articles: 3 were in healthy, 1 in frail and 3 in older adults with a specific condition or disease. 1 study investigated the effects of Tai Chi in older adults with mild cognitive impairment. In frail older subjects, IGF-1 - sole marker investigated - significantly increased after 8 weeks RT and CT, whereas AT showed no significant effects compared to control. Most consistent exercise effects consisted in lowering of circulating levels of CRP, IL-6 and TNF-α; which seemed more prominent in healthy older adults compared to those with a specific disease or condition. None of the studies reported an exacerbation of inflammation following exercise and all studied exercise protocols were feasible and safe for older adults. Conclusions: Overall, significant anti-inflammatory effects of exercise in older persons were reported. Literature remains extremely scarce regarding the exercise-induced effects in frail older persons. Therefore, there is an urgent need for more studies focusing on the frail elderly. There is growing literature data on exercise interventions in older adults with a specific condition or disease; however, it appears more challenging to reduce inflammageing through exercise in these specific patient groups. Importantly, the exercise interventions performed in all studies appeared to be feasible and safe for older patients, thus the presence of a specific condition or disease should not be considered as a contra-indication to perform physical exercise. © 2021 Elsevier Inc.},
author_keywords={Aged; Exercise; Inflammation},
references={Arrieta, H., Hervas, G., Rezola-Pardo, C., Ruiz-Litago, F.,

Iturburu, M., Yanguas, J.J., Serum myostatin levels are higher in fitter, more active, and non-frail long-term nursing home residents and increase after a physical exercise intervention (2019) Gerontology, 65 (3), pp. 229-239; Boeselt, T., Nell, C., Lütteken, L., Kehr, K., Koepke, J., Apelt, S., Benefits of high-intensity exercise training to patients with chronic obstructive pulmonary disease: a controlled study (2017) J Respiration, 93 (5), pp. 301-310; Bruunsgaard, H., Skinhoj, P., Qvist, J., Pedersen, B.K., Elderly humans show prolonged in vivo inflammatory activity during pneumococcal infections (1999) J. Infect. Dis., 180 (2), pp. 551-554; Calvani, R., Marini, F., Cesari, M., Tosato, M., Anker, S.D., von Haehling, S., Biomarkers for physical frailty and sarcopenia: state of the science and future developments (2015) J. Cachexia. Sarcopenia Muscle, 6 (4), pp. 278-286; Camillo, C.A., Burtin, C., Hornikx, M., Demeyer, H., De Bent, K., van Remoortel, H., Physiological responses during downhill walking: a new exercise modality for subjects with chronic obstructive pulmonary disease? (2015) Chron Respir Dis, 12 (2), pp. 155-164; Campo, R.A., Light, K.C., O'Connor, K., Nakamura, Y., Lipschitz, D., LaStayo, P.C., Blood pressure, salivary cortisol, and inflammatory cytokine outcomes in senior female cancer survivors enrolled in a tai chi chih randomized controlled trial (2015) J. Cancer Surviv., 9 (1), pp. 115-125; Cao Dinh, H., Njemini, R., Onyema, O.O., Beyer, I., Liberman, K., De Dobbeleer, L., Strength endurance training but not intensive strength training reduces senescence-prone T cells in peripheral blood in community-dwelling elderly women (2019) J. Gerontol. A Biol. Sci. Med. Sci., 74 (12), pp. 1870-1878; Chen, N., Li, Q., Liu, J., Jia, S., Irisin, an exercise-induced myokine as a metabolic regulator: an updated narrative review (2016) Diabetes Metab. Res. Rev., 32 (1), pp. 51-59; Chen, H.-T., Chung, Y.-C., Chen, Y.-J., Ho, S.-Y., Wu, H.-J., Effects of different types of exercise on body composition, muscle strength, and IGF-1 in the elderly with Sarcopenic obesity (2017) J Journal of the American Geriatrics Society, 65 (4), pp. 827-832; Colaianni, G., Cinti, S., Colucci, S., Grano, M., Irisin and musculoskeletal health (2017) Ann. N. Y. Acad. Sci., 1402 (1), pp. 5-9; Desai, M., Mohamed, A., Davenport, A., Desai, M., Mohamed, A., Davenport, A., A pilot study investigating the effect of pedalling exercise during dialysis on 6-min walking test and hand grip and pinch strength (2019) J International Journal of Artificial Organs, 42 (4), pp. 161-166; Dong, Z.J., Zhang, H.L., Yin, L.X., Effects of intradialytic resistance exercise on systemic inflammation in maintenance hemodialysis patients with sarcopenia: a randomized controlled trial (2019) Int. Urol. Nephrol., 51 (8), pp. 1415-1424; Ferrucci, L., Fabbri, E., Inflammageing: chronic inflammation in ageing, cardiovascular disease, and frailty (2018) Nat. Rev. Cardiol., 15 (9), pp. 505-522; Ferrucci, L., Guralnik, J.M., Studenski, S., Fried, L.P., Cutler, G.B., Jr., Walston, J.D., Designing randomized, controlled trials aimed at preventing or delaying functional decline and disability in frail, older persons: a consensus report (2004) J. Am. Geriatr. Soc., 52 (4), pp. 625-634; Fischer, C.P., Interleukin-6 in acute exercise and training: what is the biological relevance? (2006) Exerc. Immunol. Rev., 12, pp. 6-33; Flack, K.D., Davy, B.M., DeBerardinis, M., Boutagy, N.E., McMillan, R.P., Hulver, M.W., Resistance exercise training and in vitro skeletal muscle oxidative capacity in older adults (2016) Physiological reports, 4 (13); Forti, L.N., Van Roie, E., Njemini, R., Coudyzer, W., Beyer, I., Delecluse, C., Load-specific inflammation mediating effects of resistance training in older persons (2016) J. Am. Med. Dir. Assoc., 17 (6), pp. 547-552; Forti, L.N., Van Roie, E., Njemini, R., Coudyzer, W., Beyer, I., Delecluse, C., Effects of resistance training at different loads on inflammatory markers in young adults (2017) Eur. J. Appl. Physiol., 117 (3), pp. 511-519; Fox, J.,

Rioux, B.V., Goulet, E.D.B., Johanssen, N.M., Swift, D.L., Bouchard, D.R., Effect of an acute exercise bout on immediate post-exercise irisin concentration in adults: a meta-analysis (2018) Scand. J. Med. Sci. Sports, 28 (1), pp. 16-28; Franca, E.E., Ribeiro, L.C., Lamenha, G.G., Magalhaes, I.K., Figueiredo, T.G., Costa, M.J., Oxidative stress and immune system analysis after cycle ergometer use in critical patients (2017) Clinics (Sao Paulo), 72 (3), pp. 143-149; Frank, P., Andersson, E., Ponten, M., Ekblom, B., Ekblom, M., Sahlin, K., Strength training improves muscle aerobic capacity and glucose tolerance in elderly (2016) Scand. J. Med. Sci. Sports, 26 (7), pp. 764-773; Gabay, C., Kushner, I., Acute-phase proteins and other systemic responses to inflammation (1999) N. Engl. J. Med., 340 (6), pp. 448-454; Hasegawa, N., Fujie, S., Horii, N., Uchida, M., Kurihara, T., Sanada, K., Aerobic exercise training-induced changes in serum C1q/TNF-related protein levels are associated with reduced arterial stiffness in middle-aged and older adults (2018) Am J Physiol Regul Integr Comp Physiol, 314 (1), pp. R94-R101; Herrstedt, A., Bay, M.L., Simonsen, C., Sundberg, A., Egeland, C., Thorsen-Streit, S., Exercise-mediated improvement of depression in patients with gastro-esophageal junction cancer is linked to kynurenine metabolism 2019 (2019), (-May-. 579-87 pp.); Hsieh, P.L., Tseng, C.H., Tseng, Y.J., Yang, W.S., Resistance training improves muscle function and cardiometabolic risks but not quality of life in older people with type 2 diabetes mellitus: a randomized controlled trial (2016) J Geriatr Phys Ther., 41 (2), pp. 65-76; Jamaluddin, M.S., Weakley, S.M., Yao, Q., Chen, C., Resistin: functional roles and therapeutic considerations for cardiovascular disease (2012) Br. J. Pharmacol., 165 (3), pp. 622-632; Kim, H., Suzuki, T., Kim, M., Kojima, N., Ota, N., Shimotoyodome, A., Effects of exercise and milk fat globule membrane (MFGM) supplementation on body composition, physical function, and hematological parameters in community-dwelling frail Japanese women: a randomized double blind, placebo-controlled, follow-up trial. PLoS One. 2015;10(2):e0116256; Kim, D.I., Lee, D.H., Hong, S., Jo, S.W., Won, Y.S., Jeon, J.Y., Six weeks of combined aerobic and resistance exercise using outdoor exercise machines improves fitness, insulin resistance, and chemerin in the Korean elderly: a pilot randomized controlled trial (2018) Arch. Gerontol. Geriatr., 75, pp. 59-64; Labandeira-Garcia, J.L., Costa-Besada, M.A., Labandeira, C.M., Villar-Cheda, B., Rodríguez-Perez, A.I., Insulin-like growth factor-1 and neuroinflammation (2017) Front. Aging Neurosci., 9; Lakens, D., Calculating and reporting effect sizes to facilitate cumulative science: a practical primer for t-tests and ANOVAs (2013) Front. Psychol., 4, p. 863; Lee, W.J., IGF-I exerts an anti-inflammatory effect on skeletal muscle cells through down-regulation of TLR4 signaling (2011) Immune Netw, 11 (4), p. 223; Lee, J.S., Kim, C.G., Seo, T.B., Kim, H.G., Yoon, S.J., Effects of 8-week combined training on body composition, isokinetic strength, and cardiovascular disease risk factors in older women (2015) Aging Clin. Exp. Res., 27 (2), pp. 179-186; Liao, M.T., Liu, W.C., Lin, F.H., Huang, C.F., Chen, S.Y., Liu, C.C., Intradialytic aerobic cycling exercise alleviates inflammation and improves endothelial progenitor cell count and bone density in hemodialysis patients (2016) Medicine (Baltimore), 95 (27); Liberman, K., Forti, L.N., Beyer, I., Bautmans, I., The effects of exercise on muscle strength, body composition, physical functioning and the inflammatory profile of older adults: a systematic review (2017) Curr Opin Clin Nutr Metab Care, 20 (1), pp. 30-53; Lima, L.G., Bonardi, J.M., Campos, G.O., Bertani, R.F., Scher, L.M., Louzada-Junior, P., Effect of aerobic training and aerobic and resistance training on the inflammatory status of hypertensive older adults (2015) Aging Clin. Exp. Res., 27 (4), pp. 483-489; Marzetti, E., Landi, F., Marini, F., Cesari, M., Buford, T.W., Manini, T.M.,

Patterns of circulating inflammatory biomarkers in older persons with varying levels of physical performance: a partial least squares-discriminant analysis approach. Front Med (Lausanne). 2014;1:27; Mejias-Pena, Y., Estebanez, B., Rodriguez-Miguelez, P., Fernandez-Gonzalo, R., Almar, M., de Paz, J.A., Impact of resistance training on the autophagy-inflammation-apoptosis crosstalk in elderly subjects (2017) Aging (Albany NY), 9 (2), pp. 408-418; Chupel, M.U., Minuzzi, L.G., Furtado, G., Santos, M.L., Hogervorst, E., Filaire, E., Exercise and taurine in inflammation, cognition, and peripheral markers of blood-brain barrier integrity in older women (2018) J Applied physiology, nutrition, and metabolism, 43 (7), pp. 733-741; NICE, Methodology checklist: randomised controlled trials (2012), http://publications.nice.org.uk/the-guidelines-manual-appendices-bi-pmg6b, Available from:; Nishida, Y., Tanaka, K., Hara, M., Hirao, N., Tanaka, H., Tobina, T., Effects of home-based bench step exercise on inflammatory cytokines and lipid profiles in elderly Japanese females: a randomized controlled trial (2015) Arch. Gerontol. Geriatr., 61 (3), pp. 443-451; Ouzzani, M., Hammady, H., Fedorowicz, Z., Elmagarmid, A., Rayyan—a web and mobile app for systematic reviews (2016) Systematic Reviews, 5 (1); Pawelec, G., Bronikowski, A., Cunnane, S.C., Ferrucci, L., Franceschi, C., Fülöp, T., The conundrum of human immune system “senescence” (2020) Mech. Ageing Dev, p. 111357; Pedersen, B.K., Febbraio, M.A., Muscle as an endocrine organ: focus on muscle-derived interleukin-6 (2008) Physiol. Rev., 88 (4), pp. 1379-1406; Ruiz, J.R., Gil-Bea, F., Bustamante-Ara, N., Rodriguez-Romo, G., Fiuza-Luces, C., Serra-Rexach, J.A., Resistance training does not have an effect on cognition or related serum biomarkers in nonagenarians: a randomized controlled trial (2015) Int. J. Sports Med, 36 (1), pp. 54-60; Strandberg, E., Edholm, P., Ponsot, E., Wahlin-Larsson, B., Hellmen, E., Nilsson, A., Influence of combined resistance training and healthy diet on muscle mass in healthy elderly women: a randomized controlled trial (2015) J. Appl. Physiol., 119 (8), pp. 918-925; Sungkarat, S., Boripuntakul, S., Kumfu, S., Lord, S.R., Chattipakorn, N., Tai chi improves cognition and plasma BDNF in older adults with mild cognitive impairment: a randomized controlled trial (2018) Neurorehabil. Neural Repair, 32 (2), pp. 142-149; Tannou, T., Koeberle, S., Manckoundia, P., Aubry, R., Multifactorial immunodeficiency in frail elderly patients: contributing factors and management (2019) Med. Mal. Infect., 49 (3), pp. 167-172; Theodorou, A.A., Panayiotou, G., Volaklis, K.A., Douda, H.T., Paschalis, V., Nikolaidis, M.G., Aerobic, resistance and combined training and detraining on body composition, muscle strength, lipid profile and inflammation in coronary artery disease patients (2016) Res Sports Med, 24 (3), pp. 171-184; Tomeleri, C.M., Ribeiro, A.S., Souza, M.F., Schiavoni, D., Schoenfeld, B.J., Venturini, D., Resistance training improves inflammatory level, lipid and glycemic profiles in obese older women: a randomized controlled trial (2016) Exp. Gerontol., 84, pp. 80-87; Tomeleri, C.M., Souza, M.F., Burini, R.C., Cavaglieri, C.R., Ribeiro, A.S., Antunes, M., Resistance training reduces metabolic syndrome and inflammatory markers in older women: a randomized controlled trial (2018) J Diabetes, 10 (4), pp. 328-337; Tomeleri, C.M., Ribeiro, A.S., Cavaglieri, C.R., Deminice, R., Schoenfeld, B.J., Schiavoni, D., Correlations between resistance training-induced changes on phase angle and biochemical markers in older women (2018) Scand. J. Med. Sci. Sports, 28 (10), pp. 2173-2182; Tucci, H.T., Figueiredo, D.S., de Paula Carvalho, R., Souza, A.C.F., Vassao, P.G., Renno, A.C.M., Quadriceps femoris performance after resistance training with and without photobiomodulation in elderly women: a randomized clinical trial (2019) Lasers Med. Sci., 34 (8), pp. 1583-1594; Varadhan, R., Yao, W., Matteini, A., Beamer, B.A., Xue, Q.L., Yang, H., Simple biologically informed inflammatory

index of two serum cytokines predicts 10 year all-cause mortality in older adults (2014) J. Gerontol. A Biol. Sci. Med. Sci, 69 (2), pp. 165-173; Vatic, M., Von Haehling, S., Ebner, N., Inflammatory biomarkers of frailty (2020) Exp. Gerontol., 133; Weigert, C., Lehmann, R., Hartwig, S., Lehr, S., The secretome of the working human skeletal muscle—a promising opportunity to combat the metabolic disaster? (2014) Proteomics Clin Appl, 8 (1-2), pp. 5-18; Windsor, M.T., Bailey, T.G., Perissiou, M., Greaves, K., Jha, P., Leicht, A.S., cute inflammatory responses to exercise in patients with abdominal aortic aneurysm (2017) J Medicine and science in sports and exercise, 50 (4), pp. 649-658; Yamada, M., Nishiguchi, S., Fukutani, N., Aoyama, T., Arai, H., Mail-based intervention for sarcopenia prevention increased anabolic hormone and skeletal muscle mass in community-dwelling Japanese older adults: the INE (Intervention by Nutrition and Exercise) study (2015) J. Am. Med. Dir. Assoc., 16 (8), pp. 654-660; Zaidi, H., Byrkjeland, R., Njerve, I.U., Akra, S., Solheim, S., Arnesen, H., Effects of exercise training on inflammasome-related mediators and their associations to glucometabolic variables in patients with combined coronary artery disease and type 2 diabetes mellitus: sub-study of a randomized control trial (2019) Diab. Vasc. Dis. Res., 16 (4), pp. 360-368; Zhang, J., Zhang, W., Can irisin be a linker between physical activity and brain function? (2016) Biomolecular Concepts, 7 (4), pp. 253-258},
correspondence_address1={Bautmans, I.; Frailty in Ageing Research Group, Laarbeeklaan 103, Belgium; email: ivan.bautmans@vub.be},
publisher={Elsevier Inc.},
issn={05315565},
coden={EXGEA},
pubmed_id={33453323},
language={English},
abbrev_source_title={Exp. Gerontol.},
document_type={Article},
source={Scopus},
}

@ARTICLE{Goroso2021,
author={Goroso, D.G. and Watanabe, W.T. and Napoleone, F. and da Silva, D.P. and Salinet, J.L. and da Silva, R.R. and Puglisi, J.L.},
title={Remote monitoring of heart rate variability for obese children},
journal={Biomedical Signal Processing and Control},
year={2021},
volume={66},
doi={10.1016/j.bspc.2021.102453},
art_number={102453},
note={cited By 0},
url={https://www.scopus.com/inward/record.uri?eid=2-s2.0-85100381761&doi=10.1016%2fj.bspc.2021.102453&partnerID=40&md5=ac958e02c763285e7498c68cca5f1f2b},
affiliation={Research and Technology Center, University of Mogi das Cruzes, Mogi das Cruzes, Brazil; University of Paraíba Valley, São José dos Campos, São Paulo, Brazil; Engineering, Modelling and Applied Social Sciences Centre, Federal ABC University, São Bernardo do Campo, São Paulo, Brazil; Center for Biomedical Engineering, University of Campinas, Campinas, São Paulo, Brazil; College of Medicine, California Northstate University, Elk Grove, Sacramento, CA, United States},
abstract={Background: Heart rate variability (HRV) is a measurement of time variation between heartbeats. HRV analysis in children and adolescents with overweight and obesity is used as an indicator of cardiovascular system

function. Because of that it is important to monitor HR with a non-invasive and reliable method. This paper describes a less traumatic and non-expensive method for online monitoring of HR and calculation of HRV parameters in eutrophic, overweight and obese children. Methods: Cardiac activity from eutrophic (n = 6), overweight (n = 11), and obese (n = 9) children, (10.3 ± 1.3 years old) were performed with a smartphone App connected via Bluetooth to a smartwatch sensor during night-, morning- and afternoon/evening-time for sixty days. Signals were transmitted from the smartphone to an online monitoring system. HRV analysis was performed on the web platform, eighteen parameters were calculated (time domain, frequency domain, Poincaré Plot and Sample Entropy). Normality, Two-way repeated measures Anova and Bonferroni tests were applied, p < 0.05 were considered statistically significant. Results: Each group showed statistical differences in the time domain parameters SDNN and RMSDD, p < 0.05 in all cases. The parameters SD2 and ratio SD1-SD2 were significantly different in all cases (p < 0.05). Likewise, there were differences between night-, morning- and afternoon/evening-time for all groups in the same parameters SDNN and RMSDD (p < 0.05 in all cases) and SD2 (p < 0.05), while the ratio SD1-SD2 was marginally significant (p = 0.052). Conclusions: The described methodology provides a new, inexpensive and easy to implement approach for remote monitoring and analysis of cardiovascular parameters from children. © 2021 Elsevier Ltd},
author_keywords={Children obesity; Heart rate variability; Internet-of-things; Remote monitoring},
keywords={Eutrophication; Frequency domain analysis; Heart; Partial discharges; Remote control; Smartphones, Cardiovascular parameters; Children and adolescents; Frequency domains; Heart rate variability; On-line monitoring system; Online monitoring; Statistical differences; Time-domain parameters, Time domain analysis},
references={Working group of ESC Heart rate variability. Standards of measurement, physiological interpretation, and clinical use (1996) Eur. Heart J., 17, pp. 354-381; Goroso, D.G., Silva, R.R., Battistella, L.R., Odstrcil, M., Paolini, M., Monitoring heart rate variability online using e-health oriented 3G mobile telephone services (2013) J. Phys. Conf. Ser. (Online), 477; Chana, M., Estève, D., Fourniols, J.Y., Escriba, C., Campo, E., Smart wearable systems: current status and future challenges (2012) Artif. Intell. Med., 56, pp. 137-156; Patel, S., Park, H., Bonato, P., Chan, L., Rodgers, M., A review of wearable sensors and systems with application in rehabilitation (2012) J. Neuroeng. Rehabil., 9, p. 21; Guiry, J.J., van der Ven, P., Nelson, J., Warmerdam, E.H., Riper, H., Activity recognition with smartphone support (2014) Med. Eng. Phys., 36 (6), pp. 670-675; Iosa, M., New technologies for stroke rehabilitation (2013) Stroke Res. Treat., 2013. , 2 pages; Aceto, G., Persico, V., Pescapé, A., The role of Information and Communication Technologies in healthcare: taxonomies, perspectives, and challenges (2018) J. Netw. Comput. Appl., 107, pp. 125-154; Yach, D., Hawkes, C., Gould, C.L., Hofman, K.J., The global burden of chronic diseases: overcoming impediments to prevention and control (2004) J. Am. Med. Assoc [Internet], 291 (21), pp. 2616-2622; WHO, Consideration of the Evidence on Childhood Obesity for the Commission on Ending Childhood Obesity: Report of the Ad Hoc Working Group on Science and Evidence for Ending Childhood Obesity (2016), World Health Organization Geneva; WHO, Obesity and Overweight (2018), http://www.who.int/en/news-room/fact-sheets/detail/obesity-and-overweight, WHO Geneva Available from:; Ezzati, M., Worldwide trends in body-mass index, underweight, overweight, and obesity from 1975 to 2016: a pooled analysis of 2416 population-based measurement studies in 128·9 million children,

adolescents, and adults (2017) Lancet, 390, pp. 2627-2642; Mainardi, L., On the quantification of heart rate variability spectral parameters using time–frequency and time-varying methods (2009) Philos. Trans. R. Soc. Lond. A: Math. Phys. Eng. Sci., 367 (1887), pp. 255-275; Borresen, J., Lambert, M., Autonomic control of heart rate during and after exercise: measurements and implications for monitoring training status (2008) Sports Med., 38 (8), pp. 633-646; Buchheit, M., Monitoring training status with HR measures: do all roads lead to Rome? (2014) Front. Physiol., 5, p. 73; Plews, D.J., Laursen, P.B., Kilding, A.E., Buchheit, M., Heart rate variability in elite triathletes, is variation in variability the key to effective training? A case comparison (2012) Eur. J. Appl. Physiol., 112, pp. 3729-3741; Orini, M., Bailón, R., Mainardi, L., Laguna, P., Synthesis of HRV signals characterized by predetermined time-frequency structure by means of time-varying ARMA models (2012) Biomed. Signal Process. Control, 7, pp. 141-150; Silva, D.P., Bissaco, M.A.S., Silva, R.R., Martini-Rodrigues, S.C., Goroso, D.G., Biomedical data monitoring for prevention and health care: development of a novel smartphone application for data acquisition (2018) J. Netw. Comput. Appl., , Submit: August 28; Shaffer, F., Ginsberg, J.P., An overview of heart rate variability metrics and norms (2017) Front. Public Health, 5, p. 258; Castaneda, D., Esparza, A., Ghamari, M., Soltanpur, C., Nazeran, H.I., A review on wearable photoplethysmography sensors and their potential future applications in health care (2018) Int. J. Biosens. Bioelectron., 4 (4), pp. 195-202; Bolanos, M., Nazeran, H., Haltiwanger, E., Comparison of heart rate variability signal features derived from electrocardiography and photoplethysmography in healthy individuals (2006) Article in Conference Proceedings: Annual International Conference of the IEEE Engineering in Medicine and Biology Society. IEEE Engineering in Medicine and Biology Society. Conference · February; Lopez, L.J.R., Goroso, D.G., Monitoring system of assessment of energy expenditure to humans in free-living condition by mean of information and communication technologies (2010) Glob. Telemed. Health Updates: Knowledge Resour., 3, pp. 551-555; Malik, M., (Chairman of Writing Committee of Task Force of the European Society of Cardiology and the North American Society of Pacing Electrophysiology): heart rate variability: standards of measurement, physiological interpretation, and clinical use (1996) Circulation, 93, pp. 1043-1065; Bourdillon, N., Schmitt, L., Yazdani, S., Vesin, J.M., Millet, G.P., Minimal window duration for accurate HRV recording in athletes (2017) Front. Neurosci., 11 (August), p. 456. , PubMed PMID: 28848382; PubMed Central PMCID: PMC5554345; Swainson, M.G., Batterham, A.M., Tsakirides, C., Rutherford, Z.H., Hind, K., Prediction of whole-body fat percentage and visceral adipose tissue mass from five anthropometric variables (2017) PLoS One, 12 (5), p. e0177175; Buchan, D.S., Baker, J.S., Utility of Body Mass Index, Waist-to-Height-Ratio and cardiorespiratory fitness thresholds for identifying cardiometabolic risk in 10.4-17.6-year-old children (2017) Obes. Res. Clin. Pract., 11 (5), pp. 567-575; Savva, S.C., Lamnisos, D., Kafatos, A.G., Predicting cardiometabolic risk: waist-to-height ratio or BMI. A meta-analysis (2013) Diabetes Metab. Syndr. Obes., 24 (6), pp. 403-419; Malliani, A., Montano, N., Heart rate variability as a clinical tool (2002) Ital. Heart J., 3 (8), pp. 439-445; Santos, L.H.C., Cognitive and behavioral screening of children with learning disabilities: a preliminary study (2012) Rev. Paul. Pediatr., 30 (1), pp. 93-99; Ouvrier, R.A., Goldsmith, R.F., Ouvrier, S., Williams, I.C., The value of the Mini-Mental State Examination in childhood: a preliminary study (1993) J. Child Neurol., 8, pp. 145-148; Xi, Establishing international blood pressure references among nonoverweight children and adolescents aged 6 to 17 years (2016) Circulation, 133 (4), pp. 398-408; Plews, D.J., Scott, B., Altini, M.,

Wood, M., Kilding, A.E., Laursen, P.B., Comparison of heart-rate-Variability recording with smartphone photoplethysmography, polar H7 chest strap, and electrocardiography (2017) Int. J. Sports Physiol. Perform., 12 (10), pp. 1324-1328; Etiwy, M., Akhrass, Z., Gillinov, L., Alashi, A., Wang, R.R., Blackburn, G., Gillinov, S.M., Desai, M.Y., Accuracy of wearable heart rate monitors in cardiac rehabilitation (2019) Cardiovasc. Diagn. Ther., 9 (3), pp. 262-271; Hermand, E., Cassirame, J., Ennequin, G., Hue, O., Validation of a photoplethysmographic heart rate monitor: polar OH1 (2019) Int. J. Sports Med., 40 (7), pp. 462-467; Sollers, J.J., Buchanan, T.W., Mowrer, S.M., Hill, L.K., Thayer, J.F., Comparison of the ratio of the standard deviation of the r-r interval and the root mean squared successive differences (SD/RMSSD) to the low frequency-to-high frequency (LF/HF) ratio in a patient population and normal healthy controls (2007) Biomed. Sci. Instrum., 43, pp. 158-163; Bartels, R., Neumamm, L.L., Peçanha, T., Silva-Carvalho, A.R., SinusCor: an advanced tool for heart rate variability analysis (2017) Biomed. Eng. Online, 16, p. 110; Kobayashi, H., Park, B.J., Miyazaki, Y.Y., Normative references of heart rate variability and salivary alpha-amylase in a healthy young male population (2012) J. Physiol. Anthropol., 31, p. 9; Guzik, P., Piskorski, J., Krauze, T., Schneider, R., Wesseling, K.H., Wykrętowicz, A., Wysocki, H., Correlations between the Poincaré plot and conventional heart rate variability parameters assessed during paced breathing (2007) J. Physiol. Sci., 57 (1), pp. 63-71. , Epub 2007 Feb 3; Maculan, A.M., Towards a knowledge-based economy in Brazil (2016) Trends and Challenges in Science and Higher Education. Knowledge Studies in Higher Education, p. 3. , H. Horta M. Heitor J. Salmi Springer, Cham; Puddy, R.W., Hall, D.M., Translating economic evidence for public health: knowledge brokers and the interactive systems framework (2017) Am. J. Prev. Med., 53 (5), pp. e185-e189; Lopes, W.S., Silva, D.P., Watanabe, W.T., Rodrigues, H.C., Bastos, S.H., Silva, R.R., Goroso, D.G., Economic impact of remote monitoring system of the obesity in children: costs of monitoring and conventional practices (2020) VIII Latin American Conference on Biomedical Engineering and XLII National Conference on Biomedical Engineering, 75, pp. 1417-1427. , C. González Díaz Springer Cham CLAIB 2019. IFMBE Proceedings; Nyamdorj, R., Qiao, Q., Lam, T.H., Tuomilehto, J., Ho, S.Y., Pitkäniemi, J., BMI compared with central obesity indicators in relation to diabetes and hypertension in Asians. Decoda Study Group (2008) Obesity, 16 (7), pp. 1622-1635; Nyamdorj, R., Qiao, Q., Söderberg, S., Pitkäniemi, J.M., Zimmet, P.Z., Shaw, J.E., BMI compared with central obesity indicators as a predictor of diabetes incidence in Mauritius (2008) Obesity, 17 (2), pp. 342-348; Ribeiro, J.C., Guerra, S., Oliveira, J., Andersen, L.B., Duarte, J.A., Mota, J., Body fatness and clustering of cardiovascular disease risk factors in Portuguese children and adolescents (2004) Am. J. Hum. Biol., 16 (5), pp. 556-562; Silvetti, M.S., Drago, F., Ragonese, P., Heart rate variability in healthy children and adolescents is partially related to age and gender (2001) Int. J. Cardiol., 81 (2-3), pp. 169-174; Gąsior, J.S., Sacha, J., Pawłowski, M., Zieliński, J., Jeleń, P.J., Tomik, A., Książczyk, T.M., Dąbrowski, M.J., Normative values for heart rate variability parameters in school-aged children: simple approach considering differences in average heart rate (2018) Front. Physiol., 10 (9), p. 1495},
correspondence_address1={Goroso, D.G.; Mogi das Cruzes University, Av. Cândido Xavier de Almeida Souza, 200, Centro Cívico, Brazil; email: danielg@umc.br},
publisher={Elsevier Ltd},
issn={17468094},
language={English},
abbrev_source_title={Biomed. Signal Process. Control},

document_type={Article},
source={Scopus},
}

@ARTICLE{Jones2021398,
author={Jones, L. and Hui, A. and Phan, C.-M. and Read, M.L. and Azar, D. and Buch, J. and Ciolino, J.B. and Naroo, S.A. and Pall, B. and Romond, K. and Sankaridurg, P. and Schnider, C.M. and Terry, L. and Willcox, M.},
title={CLEAR - Contact lens technologies of the future},
journal={Contact Lens and Anterior Eye},
year={2021},
volume={44},
number={2},
pages={398-430},
doi={10.1016/j.clae.2021.02.007},
note={cited By 3},
url={https://www.scopus.com/inward/record.uri?eid=2-s2.0-85103103940&doi=10.1016%2fj.clae.2021.02.007&partnerID=40&md5=fc9226bc13eb245a95c4bb1851297432},
affiliation={Centre for Ocular Research & Education (CORE), School of Optometry & Vision Science, University of Waterloo, Waterloo, Canada; Centre for Eye and Vision Research (CEVR), 17W Hong Kong Science Park, Hong Kong; School of Optometry and Vision Science, UNSW Sydney, Sydney, NSW, Australia; Eurolens Research, Division of Pharmacy and Optometry, Faculty of Biology, Medicine and Health, The University of Manchester, Manchester, United Kingdom; Department of Ophthalmology and Visual Sciences, University of Illinois College of Medicine, Chicago, IL, United States; Verily Life Sciences, San Francisco, CA, United States; Johnson & Johnson Vision Care, Jacksonville, FL, United States; Department of Ophthalmology, Massachusetts Eye and Ear Infirmary, Harvard Medical School, Boston, MA, United States; College of Health and Life Sciences, Aston University, Birmingham, B4 7ET, United Kingdom; Brien Holden Vision Institute, Sydney, Australia; C Schnider Insights & More, Brush Prairie, WA, United States; School of Optometry and Vision Sciences, Cardiff University, United Kingdom},
abstract={Contact lenses in the future will likely have functions other than correction of refractive error. Lenses designed to control the development of myopia are already commercially available. Contact lenses as drug delivery devices and powered through advancements in nanotechnology will open up further opportunities for unique uses of contact lenses. This review examines the use, or potential use, of contact lenses aside from their role to correct refractive error. Contact lenses can be used to detect systemic and ocular surface diseases, treat and manage various ocular conditions and as devices that can correct presbyopia, control the development of myopia or be used for augmented vision. There is also discussion of new developments in contact lens packaging and storage cases. The use of contact lenses as devices to detect systemic disease has mostly focussed on detecting changes to glucose levels in tears for monitoring diabetic control. Glucose can be detected using changes in colour, fluorescence or generation of electric signals by embedded sensors such as boronic acid, concanavalin A or glucose oxidase. Contact lenses that have gained regulatory approval can measure changes in intraocular pressure to monitor glaucoma by measuring small changes in corneal shape. Challenges include integrating sensors into contact lenses and detecting the signals generated. Various techniques are used to optimise uptake and release of the drugs to the ocular surface to treat diseases such as dry eye, glaucoma, infection and allergy. Contact lenses that either mechanically or

electronically change their shape are being investigated for the management of presbyopia. Contact lenses that slow the development of myopia are based upon incorporating concentric rings of plus power, peripheral optical zone(s) with add power or non-monotonic variations in power. Various forms of these lenses have shown a reduction in myopia in clinical trials and are available in various markets. © 2021},
author_keywords={Augmented vision; Biosensing; Contact lens evidence-based academic reports (CLEAR); Diagnosis; Drug delivery; Theranostic},
keywords={alpha tocopherol; boronic acid derivative; concanavalin A; cyclodextrin; glucose; interleukin 17; interleukin 6; interleukin 8; liposome; matrix metalloproteinase; metal nanoparticle; reactive oxygen metabolite; tumor necrosis factor, Article; bacterial keratitis; cancer diagnosis; color vision defect; device approval; diabetic retinopathy; drug uptake; dry eye; electrochemical analysis; eye allergy; glaucoma; glucose blood level; human; intraocular pressure; lacrimal gland; limit of detection; microemulsion; molecular imprinting; ocular blood vessel; osmolarity; presbyopia; priority journal; refraction error; screening; systemic disease},
references={Hagan, S., Martin, E., Enriquez-de-Salamanca, A., Tear fluid biomarkers in ocular and systemic disease: potential use for predictive, preventive and personalised medicine (2016) EPMA J., 7, p. 15; Kallo, G., Emri, M., Varga, Z., Ujhelyi, B., Tozser, J., Csutak, A., Changes in the chemical barrier composition of tears in Alzheimer's disease reveal potential tear diagnostic biomarkers (2016) PLoS One, 11 (6), p. e0158000; Evans, V., Vockler, C., Friedlander, M., Walsh, B., Willcox, M.D., Lacryglobin in human tears, a potential marker for cancer (2001) Graefes Arch Clin Exp Ophthalmol, 29 (3), pp. 161-163; de Freitas Campos, C., Cole, N., Van Dyk, D., Walsh, B.J., Diakos, P., Almeida, D., Proteomic analysis of dog tears for potential cancer markers (2008) Res Vet Sci, 85 (2), pp. 349-352; You, J., Willcox, M.D., Madigan, M.C., Wasinger, V., Schiller, B., Walsh, B.J., Tear fluid protein biomarkers (2013) Adv Clin Chem, 62, pp. 151-196; Mrugacz, M., Kaczmarski, M., Bakunowicz-Lazarczyk, A., Zelazowska, B., Wysocka, J., Minarowska, A., IL-8 and IFN-gamma in tear fluid of patients with cystic fibrosis (2006) J Interferon Cytokine Res, 26 (2), pp. 71-75; Mrugacz, M., Zelazowska, B., Bakunowicz-Lazarczyk, A., Kaczmarski, M., Wysocka, J., Elevated tear fluid levels of MIP-1alpha in patients with cystic fibrosis (2007) J Interferon Cytokine Res, 27 (6), pp. 491-495; Mrugacz, M., CCL4/MIP-1beta levels in tear fluid and serum of patients with cystic fibrosis (2010) J Interferon Cytokine Res, 30 (7), pp. 509-512; Aihara, M., Kubota, N., Kadowaki, T., Study of the correlation between tear glucose concentrations and blood glucose concentrations (2018) Diabetes, 67, p. 944; Zhao, Z., Liu, J., Shi, B., He, S., Yao, X., Willcox, M.D., Advanced glycation end product (age) modified proteins in tears of diabetic patients (2010) Mol Vis, 16, pp. 1576-1584; Liu, J., Shi, B., He, S., Yao, X., Willcox, M.D., Zhao, Z., Changes to tear cytokines of type 2 diabetic patients with or without retinopathy (2010) Mol Vis, 16, pp. 2931-2938; Coyle, P.K., Sibony, P., Johnson, C., Oligoclonal IgG in tears (1987) Neurology, 37 (5), pp. 853-856; Devos, D., Forzy, G., de Seze, J., Caillez, S., Louchart, P., Gallois, P., Silver stained isoelectrophoresis of tears and cerebrospinal fluid in multiple sclerosis (2001) J Neurol, 248 (8), pp. 672-675; Salvisberg, C., Tajouri, N., Hainard, A., Burkhard, P.R., Lalive, P.H., Turck, N., Exploring the human tear fluid: discovery of new biomarkers in multiple sclerosis (2014) Proteomics Clin Appl, 8 (3-4), pp. 185-194; Comoglu, S.S., Guven, H., Acar, M., Ozturk, G., Kocer, B., Tear levels of tumor necrosis factor-alpha in patients with Parkinson's disease (2013) Neurosci Lett, 553,

pp. 63-67; Hamm-Alvarez, S.F., Janga, S.R., Edman, M.C., Feigenbaum, D., Freire, D., Mack, W.J., Levels of oligomeric alpha-synuclein in reflex tears distinguish Parkinson's disease patients from healthy controls (2019) Biomark Med, 13 (17), pp. 1447-1457; Huang, D., Luo, Q., Yang, H., Mao, Y., Changes of lacrimal gland and tear inflammatory cytokines in thyroid-associated ophthalmopathy (2014) Invest Ophthalmol Vis Sci, 55 (8), pp. 4935-4943; Cai, K., Wei, R., Interleukin-7 expression in tears and orbital tissues of patients with graves' ophthalmopathy (2013) Endocrine, 44 (1), pp. 140-144; Tseng, R.C., Chen, C.C., Hsu, S.M., Chuang, H.S., Contact-lens biosensors (2018) Sensors (Basel), 18 (8), p. 2651; Kim, Y.S., Functionalized eyewear device for detecting biomarker in tears (2019) Google Patents. US10830776B2, , University of Central Florida Research Foundation Inc USA; Pugh, R.B., Toner, A., Humphreys, S.R., Otts, D.B., Neeley, W.C., Blink detection system for electronic ophthalmic lens (2014) Google Patents. US9498124B2, , Johnson and Johnson Vision Care Inc USA; Chen, R., Kalinli, O., Interface using eye tracking contact lenses (2014) Us9244285b2, , Sony Interactive Entertainment Inc USA; (2015), A. Shtukater. Smart contact lens. Google Patents. US10845620B2. USA;; (2018), RB. Pugh. Ophthalmic lens with retinal vascularization monitoring system. Google Patents. US10159461B2. USA: Johnson & Johnson Vision Inc.;; Badugu, R., Jeng, B.H., Reece, E.A., Lakowicz, J.R., Contact lens to measure individual ion concentrations in tears and applications to dry eye disease (2018) Anal Biochem, 542, pp. 84-94; Yetisen, A.K., Jiang, N., Castaneda Gonzalez, C.M., Erenoglu, Z.I., Dong, J., Dong, X., Scleral lens sensor for ocular electrolyte analysis (2020) Adv Mater, 32 (6), p. e1906762; Saeedi, P., Petersohn, I., Salpea, P., Malanda, B., Karuranga, S., Unwin, N., Global and regional diabetes prevalence estimates for 2019 and projections for 2030 and 2045: results from the international diabetes federation diabetes atlas, 9(th) edition (2019) Diabetes Res Clin Pract, 157, p. 107843; Heinemann, L., Finger pricking and pain: a never ending story (2008) J Diabetes Sci Technol, 2 (5), pp. 919-921; Liao, Y.-T., Yao, H., Lingley, A., Parviz, B., Otis, B.P., A 3-μw cmos glucose sensor for wireless contact-lens tear glucose monitoring (2012) Ieee J Solid-St Circ, 47 (1), pp. 335-344; Otis, B., Liao, Y., Amirparviz, B., Yao, H., Wireless powered contact lens with biosensor (2012) Google Patents. US8608310B2, , University of Washington Center for Commercialization USA; Elsherif, M., Hassan, M.U., Yetisen, A.K., Butt, H., Wearable contact lens biosensors for continuous glucose monitoring using smartphones (2018) ACS Nano, 12 (6), pp. 5452-5462; Cummins, B.M., Garza, J.T., Cote, G.L., Optimization of a Concanavalin A-based glucose sensor using fluorescence anisotropy (2013) Anal Chem, 85 (11), pp. 5397-5404; (2019), M. Lamrani. Glucose sensing contact lens. Google Patents. US10478104B2. USA: Menicon Co Ltd;; Senior, M., Novartis signs up for Google smart lens (2014) Nat Biotechnol, 32 (9), p. 856; Park, S., Boo, H., Chung, T.D., Electrochemical non-enzymatic glucose sensors (2006) Anal Chim Acta, 556 (1), pp. 46-57; Liu, M., Liu, R., Chen, W., Graphene wrapped Cu2O nanocubes: non-enzymatic electrochemical sensors for the detection of glucose and hydrogen peroxide with enhanced stability (2013) Biosens Bioelectron, 45, pp. 206-212; Feng, D., Wang, F., Chen, Z., Electrochemical glucose sensor based on one-step construction of gold nanoparticle–chitosan composite film (2009) Sensor Actuat B-Chem, 138 (2), pp. 539-544; Park, J., Kim, J., Kim, S.Y., Cheong, W.H., Jang, J., Park, Y.G., Soft, smart contact lenses with integrations of wireless circuits, glucose sensors, and displays (2018) Sci Adv, 4 (1), p. eaap9841; Shende, P., Sahu, P., Gaud, R., A technology roadmap of smart biosensors from conventional glucose monitoring systems (2017) Ther Deliv, 8 (6), pp. 411-423; Yang, Y., Rim, Y.S., (2019), j. Yang High performance chemical and bio sensors using metal oxide

semiconductors. US 20180059051 A1; Sei, K.H., Young, C.S., Beom, H.M., Keon, J.L., Dohee, K., Smart contact lenses and smart glasses (2016) Google Patents. US10399291B2, , Phi Biomed Co Ltd USA; Miyashita, M., Ito, N., Ikeda, S., Murayama, T., Oguma, K., Kimura, J., Development of urine glucose meter based on micro-planer amperometric biosensor and its clinical application for self-monitoring of urine glucose (2009) Biosens Bioelectron, 24 (5), pp. 1336-1340; Koudelka, M., Gernet, S., De Rooij, N.F., Planar amperometric enzyme-based glucose microelectrode (1989) Sensor Actuator, 18 (2), pp. 157-165; Clark, L.C., Jr., Lyons, C., Electrode systems for continuous monitoring in cardiovascular surgery (1962) Ann N Y Acad Sci, 102 (1), pp. 29-45; Kajisa, T., Sakata, T., Glucose-responsive hydrogel electrode for biocompatible glucose transistor (2017) Sci Technol Adv Mater, 18 (1), pp. 26-33; Yao, H., Liao, Y., Lingley, A.R., Afanasiev, A., Lähdesmäki, I., Otis, B.P., A contact lens with integrated telecommunication circuit and sensors for wireless and continuous tear glucose monitoring (2012) J Micromechanics Microengineering, 22 (7), p. 075007; Alexeev, V.L., Das, S., Finegold, D.N., Asher, S.A., Photonic crystal glucose-sensing material for noninvasive monitoring of glucose in tear fluid (2004) Clin Chem, 50 (12), pp. 2353-2360; Sun, X., Zhai, W., Fossey, J.S., James, T.D., Boronic acids for fluorescence imaging of carbohydrates (2016) Chem Commun, 52 (17), pp. 3456-3469; March, W., Lazzaro, D., Rastogi, S., Fluorescent measurement in the non-invasive contact lens glucose sensor (2006) Diabetes Technol Ther, 8 (3), pp. 312-317; Aslan, K., Lakowicz, J.R., Geddes, C.D., Tunable plasmonic glucose sensing based on the dissociation of con a-aggregated dextran-coated gold colloids (2004) Anal Chim Acta, 517 (1-2), pp. 139-144; Villena Gonzales, W., Mobashsher, A.T., Abbosh, A., The progress of glucose monitoring-a review of invasive to minimally and non-invasive techniques, devices and sensors (2019) Sensors (Basel), 19 (4), p. 800; Zhu, H., Li, L., Zhou, W., Shao, Z., Chen, X., Advances in non-enzymatic glucose sensors based on metal oxides (2016) J Mater Chem B, 4 (46), pp. 7333-7349; Guo, S., Wu, K., Li, C., Wang, H., Sun, Z., Xi, D., Integrated contact lens sensor system based on multifunctional ultrathin mos2 transistors (2020) Matter; Zhang, J., Hodge, W., Hutnick, C., Wang, X., Noninvasive diagnostic devices for diabetes through measuring tear glucose (2011) J Diabetes Sci Technol, 5 (1), pp. 166-172; Badugu, R., Lakowicz, J.R., Geddes, C.D., A glucose-sensing contact lens: from bench top to patient (2005) Curr Opin Biotechnol, 16 (1), pp. 100-107; Vaddiraju, S., Burgess, D.J., Tomazos, I., Jain, F.C., Papadimitrakopoulos, F., Technologies for continuous glucose monitoring: current problems and future promises (2010) J Diabetes Sci Technol, 4 (6), pp. 1540-1562; McQueen, R.B., Ellis, S.L., Campbell, J.D., Nair, K.V., Sullivan, P.W., Cost-effectiveness of continuous glucose monitoring and intensive insulin therapy for type 1 diabetes (2011) Cost Eff Resour Alloc, 9, p. 13; Diabetes, C., Complications Trial Research, G., Nathan, D.M., Genuth, S., Lachin, J., Cleary, P., The effect of intensive treatment of diabetes on the development and progression of long-term complications in insulin-dependent diabetes mellitus (1993) N Engl J Med, 329 (14), pp. 977-986; Comstock, J., Alphabet's verily shelves glucose-sensing contact lens project with novartis (2018); March, W.F., (2002), M.F. Mowery-McKee. Ocular analyte sensor. Google Patents. US6980842B2. USA: Eyesense AG;; Geddes, C.D., Badugu, R., Lakowiccz, J.R., Quaternary nitrogen heterocyclic compounds for detecting aqueous monosaccharides in physiological fluids (2012) Google Patents. US8569502B2, , Geddes Chris Dr USA; Morris, C.A., Carney, F.P., Lane, J.D., Kits for assays of rapid screening of diabetes (2007) Google Patents. US7429462B2, , Novartis AG USA; Zhang, J., (2009), W.G. Hodge. Contact lens integrated with a biosensor for the detection of glucose and other components in tears. Google Patents.

US8385998B2. USA;; Ibey, B.L., Yadavalli, V.K., Rounds, R.M., Beier, H.T., Cote, G.L., Pishko, M.V., Method for glucose monitoring using fluorescence quenching (2010) Google Patents. US8088595B2, , Texas A&M University System Penn State Research Foundation USA; (2012), W.F.A. Besling. Flexible eye insert and glucose measuring system. Google Patents. US9737245B2. USA: NXP BV;; (2017), C.A. Yildirim. A contact lens design sensing tear glucose level. Google Patents. WO2017116350A1; (2014), Z. Liu Contact lenses having two-electrode electrochemical sensors. Google Patents. US20140194713A1. USA: Verily Life Sciences LLC;; Gandhi, H., Gao, H., Baig, M., Chen, R., Functional contact lens and related systems and methods (2015) Google Patents. US20170042480A1, , Medella Health Inc USA; Zhou, L., Beuerman, R.W., The power of tears: how tear proteomics research could revolutionize the clinic (2017) Expert Rev Proteomics, 14 (3), pp. 189-191; Zhou, L., Zhao, S.Z., Koh, S.K., Chen, L., Vaz, C., Tanavde, V., In-depth analysis of the human tear proteome (2012) J Proteomics, 75 (13), pp. 3877-3885; Molloy, M.P., Bolis, S., Herbert, B.R., Ou, K., Tyler, M.I., van Dyk, D.D., Establishment of the human reflex tear two-dimensional polyacrylamide gel electrophoresis reference map: new proteins of potential diagnostic value (1997) Electrophoresis, 18 (15), pp. 2811-2815; Goedegebuure, P.S., Watson, M.A., Viehl, C.T., Fleming, T.P., Mammaglobin-based strategies for treatment of breast cancer (2004) Curr Cancer Drug Targets, 4 (6), pp. 531-542; Bohm, D., Keller, K., Pieter, J., Boehm, N., Wolters, D., Siggelkow, W., Comparison of tear protein levels in breast cancer patients and healthy controls using a de novo proteomic approach (2012) Oncol Rep, 28 (2), pp. 429-438; Lebrecht, A., Boehm, D., Schmidt, M., Koelbl, H., Grus, F.H., Surface-enhanced laser desorption/ionisation time-of-flight mass spectrometry to detect breast cancer markers in tears and serum (2009) Cancer Genomics Proteomics, 6 (2), pp. 75-83; Lebrecht, A., Boehm, D., Schmidt, M., Koelbl, H., Schwirz, R.L., Grus, F.H., Diagnosis of breast cancer by tear proteomic pattern (2009) Cancer Genomics Proteomics, 6 (3), pp. 177-182; Herman, G., Glucose-monitoring contact lens would feature transparent sensor (2016), Oregon State University; Sit, A.J., Continuous monitoring of intraocular pressure: rationale and progress toward a clinical device (2009) J Glaucoma, 18 (4), pp. 272-279; Matlach, J., Bender, S., Konig, J., Binder, H., Pfeiffer, N., Hoffmann, E.M., Investigation of intraocular pressure fluctuation as a risk factor of glaucoma progression (2019) Clin Ophthalmol, 13, pp. 9-16; Asrani, S., Zeimer, R., Wilensky, J., Gieser, D., Vitale, S., Lindenmuth, K., Large diurnal fluctuations in intraocular pressure are an independent risk factor in patients with glaucoma (2000) J Glaucoma, 9 (2), pp. 134-142; Gillmann, K., Bravetti, G.E., Niegowski, L.J., Mansouri, K., Using sensors to estimate intraocular pressure: a review of intraocular pressure telemetry in clinical practice (2019) Expert Rev Ophthalmol, 14 (6), pp. 263-276; Leonardi, M., Leuenberger, P., Bertrand, D., Bertsch, A., Renaud, P., First steps toward noninvasive intraocular pressure monitoring with a sensing contact lens (2004) Invest Ophthalmol Vis Sci, 45 (9), pp. 3113-3117; Mansouri, K., Medeiros, F.A., Tafreshi, A., Weinreb, R.N., Continuous 24-hour monitoring of intraocular pressure patterns with a contact lens sensor: safety, tolerability, and reproducibility in patients with glaucoma (2012) Arch Ophthalmol, 130 (12), pp. 1534-1539; Leonardi, M., Pitchon, E.M., Bertsch, A., Renaud, P., Mermoud, A., Wireless contact lens sensor for intraocular pressure monitoring: assessment on enucleated pig eyes (2009) Acta Ophthalmol (Copenh), 87 (4), pp. 433-437; De Smedt, S., Mermoud, A., Schnyder, C., 24-hour intraocular pressure fluctuation monitoring using an ocular telemetry sensor: tolerability and functionality in healthy subjects (2012) J Glaucoma, 21 (8), pp. 539-544; Lorenz, K., Korb, C., Herzog, N., Vetter, J.M., Elflein, H., Keilani, M.M., Tolerability of 24-hour

intraocular pressure monitoring of a pressure-sensitive contact lens (2013) J Glaucoma, 22 (4), pp. 311-316; Hollo, G., Kothy, P., Vargha, P., Evaluation of continuous 24-hour intraocular pressure monitoring for assessment of prostaglandin-induced pressure reduction in glaucoma (2014) J Glaucoma, 23 (1), pp. e6-e12; Marando, C.M., Mansouri, K., Kahook, M.Y., Seibold, L.K., Tolerability and functionality of a wireless 24-hour ocular telemetry sensor in african american glaucoma patients (2019) J Glaucoma, 28 (2), pp. 119-124; Morales-Fernandez, L., Garcia-Bella, J., Martinez-de-la-Casa, J.M., Sanchez-Jean, R., Saenz-Frances, F., Arriola-Villalobos, P., Changes in corneal biomechanical properties after 24 hours of continuous intraocular pressure monitoring using a contact lens sensor (2018) Can J Ophthalmol, 53 (3), pp. 236-241; Tojo, N., Hayashi, A., Influence of ocular dimensional change on 24-hour intraocular pressure measurement with contact lens sensor (2019) J Glaucoma, 28 (9), pp. 808-810; Mottet, B., Aptel, F., Romanet, J.P., Hubanova, R., Pepin, J.L., Chiquet, C., 24-hour intraocular pressure rhythm in young healthy subjects evaluated with continuous monitoring using a contact lens sensor (2013) JAMA Ophthalmol, 131 (12), pp. 1507-1516; Agnifili, L., Mastropasqua, R., Frezzotti, P., Fasanella, V., Motolese, I., Pedrotti, E., Circadian intraocular pressure patterns in healthy subjects, primary open angle and normal tension glaucoma patients with a contact lens sensor (2015) Acta Ophthalmol (Copenh), 93 (1), pp. e14-e21; Cutolo, C.A., De Moraes, C.G., Liebmann, J.M., Mansouri, K., Traverso, C.E., Ritch, R., The effect of therapeutic IOP-lowering interventions on the 24-hour ocular dimensional profile recorded with a sensing contact lens (2019) J Glaucoma, 28 (3), pp. 252-257; Dunbar, G., Shen, B., Aref, A., The Sensimed Triggerfish contact lens sensor: efficacy, safety, and patient perspectives (2017) Clin Ophthalmol, 11, pp. 875-882; Chen, G.Z., Chan, I.S., Leung, L.K., Lam, D.C., Soft wearable contact lens sensor for continuous intraocular pressure monitoring (2014) Med Eng Phys, 36 (9), pp. 1134-1139; Pang, Y., Li, Y., Wang, X., Qi, C., Yang, Y., Ren, T.-L., A contact lens promising for non-invasive continuous intraocular pressure monitoring (2019) RSC Adv, 9 (9), pp. 5076-5082; Sanchez, I., Laukhin, V., Moya, A., Martin, R., Ussa, F., Laukhina, E., Prototype of a nanostructured sensing contact lens for noninvasive intraocular pressure monitoring (2011) Invest Ophthalmol Vis Sci, 52 (11), pp. 8310-8315; Zhang, Y., Chen, Y., Man, T., Huang, D., Li, X., Zhu, H., High resolution non-invasive intraocular pressure monitoring by use of graphene woven fabrics on contact lens (2019) Microsyst Nanoeng, 5, p. 39; Puers, R., Capacitive sensors: when and how to use them (1993) Sens Actuators A Phys, 37-38, pp. 93-105; Kim, J., Kim, M., Lee, M.S., Kim, K., Ji, S., Kim, Y.T., Wearable smart sensor systems integrated on soft contact lenses for wireless ocular diagnostics (2017) Nat Commun, 8, p. 14997; Huang, Y.-C., Yeh, G.-T., Yang, T.-S., Chiou, J.-C., A contact lens sensor system with a micro-capacitor for wireless intraocular pressure monitoring (2013) IEEE SENSORS, 2013, pp. 1-4; Kouhani, M., Wu, J., Tavakoli, A., Weber, A.J., Li, W., Wireless, passive strain sensor in a doughnut-shaped contact lens for continuous non-invasive self-monitoring of intraocular pressure (2020) Lab Chip, 20 (2), pp. 332-342; Elsheikh, A., Clamp, J., Device for monitoring intraocular pressure (2015) Google Patents. US9247877B2, , Contact Lens Precision Laboratories Ltd University of Dundee USA; Steer, R., Feeling the pressure (2017) The ophthalmologist, , Texere Publishing Limited; Elsheikh, A., Clinical study success for novel contact lens device to improve glaucoma treatment (2017), University Press Release; Campigotto, A., Leahy, S., Zhao, G., Campbell, R.J., Lai, Y., Non-invasive intraocular pressure monitoring with contact lens (2020) Br J Ophthalmol, 104 (9), pp. 1324-1328; An, H., Chen, L., Liu, X., Zhao, B., Zhang, H., Wu, Z., Microfluidic contact lenses for

unpowered, continuous and non-invasive intraocular pressure monitoring (2019) Sens Actuators A Phys, 295, pp. 177-187; AlQattan, B., Yetisen, A.K., Butt, H., Direct laser writing of nanophotonic structures on contact lenses (2018) ACS Nano, 12 (6), pp. 5130-5140; Wasilewicz, R., Varidel, T., Simon-Zoula, S., Schlund, M., Cerboni, S., Mansouri, K., First-in-human continuous 24-hour measurement of intraocular pressure and ocular pulsation using a novel contact lens sensor (2020) Br J Ophthalmol, 104 (11), pp. 1519-1523; Wolffsohn, J.S., Arita, R., Chalmers, R., Djalilian, A., Dogru, M., Dumbleton, K., TFOS DEWS II diagnostic methodology report (2017) Ocul Surf, 15 (3), pp. 539-574; Downie, L.E., Bandlitz, S., Bergmanson, J.P.G., Craig, J.P., Dutta, D., Maldonado-Codina, C., CLEAR - anatomy and physiology of the anterior eye (2021) Contact Lens Anterior Eye, 44 (2). , In press; Potvin, R., Makari, S., Rapuano, C.J., Tear film osmolarity and dry eye disease: a review of the literature (2015) Clin Ophthalmol, 9, pp. 2039-2047; Lemp, M.A., Bron, A.J., Baudouin, C., Benitez Del Castillo, J.M., Geffen, D., Tauber, J., Tear osmolarity in the diagnosis and management of dry eye disease (2011) Am J Ophthalmol, 151 (5), pp. 792-798. , e1; Chiou, J.C., The development of smart contact lens system: taking dry eye syndrome diagnosis as an example (2019) Future Tech Expo 2019; Tsangarides, C.P., Yetisen, A.K., da Cruz Vasconcellos, F., Montelongo, Y., Qasim, M.M., Wilkinson, T.D., Computational modelling and characterisation of nanoparticle-based tuneable photonic crystal sensors (2014) RSC Adv, 4 (21), pp. 10454-10461; Yetisen, A.K., Montelongo, Y., da Cruz Vasconcellos, F., Martinez-Hurtado, J.L., Neupane, S., Butt, H., Reusable, robust, and accurate laser-generated photonic nanosensor (2014) Nano Lett, 14 (6), pp. 3587-3593; Yetisen, A.K., Butt, H., Volpatti, L.R., Pavlichenko, I., Humar, M., Kwok, S.J., Photonic hydrogel sensors (2016) Biotechnol Adv, 34 (3), pp. 250-271; Hu, Y., Jiang, X., Zhang, L., Fan, J., Wu, W., Construction of near-infrared photonic crystal glucose-sensing materials for ratiometric sensing of glucose in tears (2013) Biosens Bioelectron, 48, pp. 94-99; Roy, N.S., Wei, Y., Kuklinski, E., Asbell, P.A., The growing need for validated biomarkers and endpoints for dry eye clinical research (2017) Invest Ophthalmol Vis Sci, 58 (6), pp. BIO1-BIO19; Munje, R.D., Muthukumar, S., Jagannath, B., Prasad, S., A new paradigm in sweat based wearable diagnostics biosensors using room temperature ionic liquids (RTILs) (2017) Sci Rep, 7 (1), p. 1950; Borderie, V.M., Gineys, R., Goldschmidt, P., Batellier, L., Laroche, L., Chaumeil, C., Association of anti-herpes simplex virus IgG in tears and serum with clinical presentation in patients with presumed herpetic simplex keratitis (2012) Cornea, 31 (11), pp. 1251-1256; Grus, F.H., Dick, B., Augustin, A.J., Pfeiffer, N., Analysis of the antibody repertoire in tears of dry-eye patients (2001) Ophthalmologica, 215 (6), pp. 430-434; Kwon, J., Surenkhuu, B., Raju, I., Atassi, N., Mun, J., Chen, Y.F., Pathological consequences of anti-citrullinated protein antibodies in tear fluid and therapeutic potential of pooled human immune globulin-eye drops in dry eye disease (2020) Ocul Surf, 18 (1), pp. 80-97; Zandbelt, M., te Boome, L., Klasen, I., van de Putte, L., van den Hoogen, F., Tear fluid measurement of anti-SS-a and anti-SS-b antibody in anti-SS-a and anti-SS-b seronegative Sjogren's syndrome patients (2009) Clin Exp Rheumatol, 27 (3), p. 536; Choi, E., Choi, Y., Nejad, Y.H.P., Shin, K., Park, J., Label-free specific detection of immunoglobulin g antibody using nanoporous hydrogel photonic crystals (2013) Sensor Actuat B-Chem, 180, pp. 107-113; Craig, J.P., Willcox, M.D., Argueso, P., Maissa, C., Stahl, U., Tomlinson, A., The TFOS international workshop on contact lens discomfort: report of the contact lens interactions with the tear film subcommittee (2013) Invest Ophthalmol Vis Sci, 54 (11). , TFOS123-156; Ballard, Z., Bazargan, S., Jung, D., Sathianathan, S., Clemens, A., Shir, D., Contact lens-based lysozyme

detection in tear using a mobile sensor (2020) Lab Chip, 20 (8), pp. 1493-1502; von Thun Und Hohenstein-Blaul, N., Funke, S., Grus, F.H., Tears as a source of biomarkers for ocular and systemic diseases (2013) Exp Eye Res, 117, pp. 126-137; Morgan, P., Murphy, P.J., Gifford, K., Gifford, P., Golebiowski, B., Johnson, L., CLEAR - effect of contact lens materials and designs on the anatomy and physiology of the eye (2021) Contact Lens Anterior Eye, 44 (2). , In press; Tsubota, K., Tear dynamics and dry eye (1998) Prog Retin Eye Res, 17 (4), pp. 565-596; Su, Y., Liang, Q., Su, G., Wang, N., Baudouin, C., Labbe, A., Spontaneous eye blink patterns in dry eye: clinical correlations (2018) Invest Ophthalmol Vis Sci, 59 (12), pp. 5149-5156; Jansen, M.E., Begley, C.G., Himebaugh, N.H., Port, N.L., Effect of contact lens wear and a near task on tear film break-up (2010) Optom Vis Sci, 87 (5), pp. 350-357; Gisler, C., Ridi, A., Hennebert, J., Weinreb, R.N., Mansouri, K., Automated detection and quantification of circadian eye blinks using a contact lens sensor (2015) Transl Vis Sci Technol, 4 (1), p. 4; Wang, Y., Zhao, Q., Du, X., Structurally coloured contact lens sensor for point-of-care ophthalmic health monitoring (2020) J Mater Chem B, 8 (16), pp. 3519-3526; Braun, R.J., King-Smith, P.E., Begley, C.G., Li, L., Gewecke, N.R., Dynamics and function of the tear film in relation to the blink cycle (2015) Prog Retin Eye Res, 45, pp. 132-164; Li, W., Graham, A.D., Selvin, S., Lin, M.C., Ocular surface cooling corresponds to tear film thinning and breakup (2015) Optom Vis Sci, 92 (9), pp. e248-e256; Su, T.Y., Chang, S.W., Yang, C.J., Chiang, H.K., Direct observation and validation of fluorescein tear film break-up patterns by using a dual thermal-fluorescent imaging system (2014) Biomed Opt Express, 5 (8), pp. 2614-2619; Tan, L.L., Sanjay, S., Morgan, P.B., Static and dynamic measurement of ocular surface temperature in dry eyes (2016) J Ophthalmol, 2016; Moreddu, R., Elsherif, M., Butt, H., Vigolo, D., Yetisen, A.K., Contact lenses for continuous corneal temperature monitoring (2019) RSC Adv, 9 (20), pp. 11433-11442; (2017), H.J. Lai. System for measuring and analyzing ocular temperature, receiving analyzer and methods for using the same. Google Patents. US9642533B2. USA: Ubiquity Biomedical Corp;; Pugh, R.B., Toner, A., Humphreys, S.R., Otts, D.B., Neeley, W.C., Ophthalmic lens with retinal vascularization monitoring system (2018) Google Patents. US10159461B2, , Johnson & Johnson Vision Inc. USA; Murphy, P.J., Lau, J.S., Sim, M.M., Woods, R.L., How red is a white eye? Clinical grading of normal conjunctival hyperaemia (2007) Eye (Lond), 21 (5), pp. 633-638; Ho, H., (2012), B. Amirparviz. Contact lens with integrated pulse oximeter. In: Office UP, ed. Google Patents. US8971978B2. USA: Verily Life Sciences LLC25; Jacobs, D.S., Carrasquillo, K.G., Cottrell, P.D., Fernández-Velázquez, F.J., Gil-Cazorla, R., Jalbert, I., CLEAR - medical use of contact lenses (2021) Contact Lens Anterior Eye, 44 (2). , In press; Kusama, S., Sato, K., Yoshida, S., Nishizawa, M., Self-moisturizing smart contact lens employing electroosmosis (2019) Adv Mater Technol, 5 (1), p. 1900889; Lee, S., Jo, I., Kang, S., Jang, B., Moon, J., Park, J.B., Smart contact lenses with graphene coating for electromagnetic interference shielding and dehydration protection (2017) ACS Nano, 11 (6), pp. 5318-5324; Kaur, S., Kim, Y.J., Milton, H., Mistry, D., Syed, I.M., Bailey, J., Graphene electrodes for adaptive liquid crystal contact lenses (2016) Opt Express, 24 (8), pp. 8782-8787; Moser, T., Celma, C., Lebert, A., Charrault, E., Brooke, R., Murphy, P.J., Hydrophilic organic electrodes on flexible hydrogels (2016) ACS Appl Mater Interfaces, 8 (1), pp. 974-982; Liu, C.H., Chang, Y.C., Norris, T.B., Zhong, Z., Graphene photodetectors with ultra-broadband and high responsivity at room temperature (2014) Nat Nanotechnol, 9 (4), pp. 273-278; Huang, J.F., Zhong, J., Chen, G.P., Lin, Z.T., Deng, Y., Liu, Y.L., A hydrogel-based hybrid theranostic contact lens for fungal keratitis (2016) ACS Nano, 10 (7), pp. 6464-6473; Alex, A.,

Edwards, A., Hays, J.D., Kerkstra, M., Shih, A., de Paiva, C.S., Factors predicting the ocular surface response to desiccating environmental stress (2013) Invest Ophthalmol Vis Sci, 54 (5), pp. 3325-3332; Friedman, N.J., Butron, K., Robledo, N., Loudin, J., Baba, S.N., Chayet, A., A nonrandomized, open-label study to evaluate the effect of nasal stimulation on tear production in subjects with dry eye disease (2016) Clin Ophthalmol, 10, pp. 795-804; Gumus, K., Schuetzle, K.L., Pflugfelder, S.C., Randomized controlled crossover trial comparing the impact of sham or intranasal tear neurostimulation on conjunctival goblet cell degranulation (2017) Am J Ophthalmol, 177, pp. 159-168; Loudin, J.D., Franke, M., HAMILTON, D.N., Doraiswamy, A., Ackermann, D.M., Contact lens for increasing tear production (2017) Google Patents. AU2020250288A1, , Oculeve Inc Australia; Dogru, M., Kojima, T., Simsek, C., Tsubota, K., Potential role of oxidative stress in ocular surface inflammation and dry eye disease (2018) Invest Ophthalmol Vis Sci, 59 (14). , DES163-DES8; Seen, S., Tong, L., Dry eye disease and oxidative stress (2018) Acta Ophthalmol (Copenh), 96 (4), pp. e412-e420; Stoddard, A.R., Koetje, L.R., Mitchell, A.K., Schotanus, M.P., Ubels, J.L., Bioavailability of antioxidants applied to stratified human corneal epithelial cells (2013) J Ocul Pharmacol Ther, 29 (7), pp. 681-687; Brzheskiy, V.V., Efimova, E.L., Vorontsova, T.N., Alekseev, V.N., Gusarevich, O.G., Shaidurova, K.N., Results of a multicenter, randomized, double-masked, placebo-controlled clinical study of the efficacy and safety of visomitin eye drops in patients with dry eye syndrome (2015) Adv Ther, 32 (12), pp. 1263-1279; Janagam, D.R., Wu, L., Lowe, T.L., Nanoparticles for drug delivery to the anterior segment of the eye (2017) Adv Drug Deliv Rev, 122, pp. 31-64; Choi, S.W., Cha, B.G., Kim, J., Therapeutic contact lens for scavenging excessive reactive oxygen species on the ocular surface (2020) ACS Nano, 14 (2), pp. 2483-2496; Walkey, C., Das, S., Seal, S., Erlichman, J., Heckman, K., Ghibelli, L., Catalytic properties and biomedical applications of cerium oxide nanoparticles (2015) Environ Sci Nano, 2 (1), pp. 33-53; Bian, F., Pelegrino, F.S., Henriksson, J.T., Pflugfelder, S.C., Volpe, E.A., Li, D.Q., Differential effects of dexamethasone and doxycycline on inflammation and MMP production in murine alkali-burned corneas associated with dry eye (2016) Ocul Surf, 14 (2), pp. 242-254; Lopez, C., Park, S., Edwards, S., Vong, S., Hou, S., Lee, M., Matrix metalloproteinase-deactivating contact lens for corneal melting (2019) ACS Biomater Sci Eng, 5 (3), pp. 1195-1199; Jamerson, E.C., Elhusseiny, A.M., ElSheikh, R.H., Eleiwa, T.K., El Sayed, Y.M., Role of matrix metalloproteinase 9 in ocular surface disorders (2020) Eye Contact Lens, 46, pp. S57-S63; Lanza, N.L., Valenzuela, F., Perez, V.L., Galor, A., The matrix metalloproteinase 9 point-of-care test in dry eye (2016) Ocul Surf, 14 (2), pp. 189-195; Dohlman, T.H., Ciralsky, J.B., Lai, E.C., Tear film assessments for the diagnosis of dry eye (2016) Curr Opin Allergy Clin Immunol, 16 (5), pp. 487-491; Barnett, M., Courey, C., Fadel, D., Lee, K., Michaud, L., Montani, G., CLEAR - scleral lenses (2021) Contact Lens Anterior Eye, 44 (2). , In press; Bains, K.K., Fukuoka, H., Hammond, G.M., Sotozono, C., Quantock, A.J., Recovering vision in corneal epithelial stem cell deficient eyes (2019) Cont Lens Anterior Eye, 42 (4), pp. 350-358; Bobba, S., Contact lens delivery of stem cells for restoring the ocular surface (2016) Biomaterials and regenerative medicine in ophthalmology, , Woodhead Publishing; Levis, H.J., Kureshi, A.K., Massie, I., Morgan, L., Vernon, A.J., Daniels, J.T., Tissue engineering the cornea: the evolution of RAFT (2015) J Funct Biomater, 6 (1), pp. 50-65; Pellegrini, G., Traverso, C.E., Franzi, A.T., Zingirian, M., Cancedda, R., De Luca, M., Long-term restoration of damaged corneal surfaces with autologous cultivated corneal epithelium (1997) Lancet, 349 (9057), pp. 990-993; Deshpande, P., Notara, M., Bullett, N., Daniels, J.T., Haddow, D.B., MacNeil, S., Development

of a surface-modified contact lens for the transfer of cultured limbal epithelial cells to the cornea for ocular surface diseases (2009) Tissue Eng Part A, 15 (10), pp. 2889-2902; Brown, K.D., Low, S., Mariappan, I., Abberton, K.M., Short, R., Zhang, H., Plasma polymer-coated contact lenses for the culture and transfer of corneal epithelial cells in the treatment of limbal stem cell deficiency (2014) Tissue Eng Part A, 20 (3-4), pp. 646-655; Di Girolamo, N., Bosch, M., Zamora, K., Coroneo, M.T., Wakefield, D., Watson, S.L., A contact lens-based technique for expansion and transplantation of autologous epithelial progenitors for ocular surface reconstruction (2009) Transplantation, 87 (10), pp. 1571-1578; Vasquez Quintero, A., Perez-Merino, P., De Smet, H., Artificial iris performance for smart contact lens vision correction applications (2020) Sci Rep, 10 (1), p. 14641; Raducanu, B.C., Zaliasl, S., Stanzione, S., van Liempd, C., Quintero, A.V., De Smet, H., An artificial iris ASIC with high voltage liquid crystal driver, 10-nA light range detector and 40-nA blink detector for LCD flicker removal (2020) Ieee Solid-state Circuits Lett, 3, pp. 506-509; Vásquez Quintero, A., Verplancke, R., De Smet, H., Vanfleteren, J., Stretchable electronic platform for soft and smart contact lens applications (2017) Adv Mater Technol, 2 (8), p. 1700073; Vásquez Quintero, A., Arai, R., Yamazaki, Y., Sato, T., De Smet, H., Near-field communication powered hydrogel-based smart contact lens (2020) Adv Mater Technol, 5 (12); Birol, G., Wang, S., Budzynski, E., Wangsa-Wirawan, N.D., Linsenmeier, R.A., Oxygen distribution and consumption in the macaque retina (2007) Am J Physiol Heart Circ Physiol, 293 (3), pp. H1696-H1704; Arden, G.B., Jyothi, S., Hogg, C.H., Lee, Y.F., Sivaprasad, S., Regression of early diabetic macular oedema is associated with prevention of dark adaptation (2011) Eye (Lond), 25 (12), pp. 1546-1554; Sahni, J.N., Czanner, G., Gutu, T., Taylor, S.A., Bennett, K.M., Wuerger, S.M., Safety and acceptability of an organic light-emitting diode sleep mask as a potential therapy for retinal disease (2017) Eye (Lond), 31 (1), pp. 97-106; Cook, C.A., Martinez-Camarillo, J.C., Yang, Q., Scianmarello, N.E., Humayun, M.S., Tai, Y., Phototherapeutic contact lens for diabetic retinopathy (2018) IEEE Micro Electro Mechanical Systems Belfast, pp. 62-65. , CHAUTEST; Sivaprasad, S., Vasconcelos, J.C., Prevost, A.T., Holmes, H., Hykin, P., George, S., Clinical efficacy and safety of a light mask for prevention of dark adaptation in treating and preventing progression of early diabetic macular oedema at 24 months (CLEOPATRA): a multicentre, phase 3, randomised controlled trial (2018) Lancet Diabetes Endocrinol, 6 (5), pp. 382-391; (1970), H.I. Zeltzer. Method of improving color discrimination. Google Patents. US3586423A. USA;; Hovis, J.K., Sirkka, D., Color discrimination of deutan and protan observers through tinted soft contact lenses (1990) Int Contact Lens Clin, 17 (11-12), pp. 287-295; Harris, D.A., Lane, G., Green, E., Color discrimination (2000) Office USP, ed. Google patents. US6089712A, , ChromaGen Vision LLC USA 6; Swarbrick, H.A., Nguyen, P., Nguyen, T., Pham, P., The ChromaGen contact lens system: colour vision test results and subjective responses (2001) Ophthalmic Physiol Opt, 21 (3), pp. 182-196; Karepov, S., Ellenbogen, T., Metasurface-based contact lenses for color vision deficiency (2020) Opt Lett, 45 (6), pp. 1379-1382; Urtti, A., Challenges and obstacles of ocular pharmacokinetics and drug delivery (2006) Adv Drug Deliv Rev, 58 (11), pp. 1131-1135; Zhang, X., Cao, X., Qi, P., Therapeutic contact lenses for ophthalmic drug delivery: major challenges (2020) J Biomater Sci Polym Ed, 31 (4), pp. 549-560; Alvarez-Lorenzo, C., Anguiano-Igea, S., Varela-Garcia, A., Vivero-Lopez, M., Concheiro, A., Bioinspired hydrogels for drug-eluting contact lenses (2019) Acta Biomater, 84, pp. 49-62; Muntz, A., Subbaraman, L.N., Sorbara, L., Jones, L., Tear exchange and contact lenses: a review (2015) J Optom, 8 (1), pp. 2-11; Li, C.-C.,

Chauhan, A., Modeling ophthalmic drug delivery by soaked contact lenses (2006) Ind Eng Chem Res, 45 (10), pp. 3718-3734; Davis, S.A., Sleath, B., Carpenter, D.M., Blalock, S.J., Muir, K.W., Budenz, D.L., Drop instillation and glaucoma (2018) Curr Opin Ophthalmol, 29 (2), pp. 171-177; Sklubalova, Z., Zatloukal, Z., Study of eye drops dispensing and dose variability by using plastic dropper tips (2006) Drug Dev Ind Pharm, 32 (2), pp. 197-205; Winfield, A.J., Jessiman, D., Williams, A., Esakowitz, L., A study of the causes of non-compliance by patients prescribed eyedrops (1990) Br J Ophthalmol, 74 (8), pp. 477-480; Walsh, K., Jones, L., The use of preservatives in dry eye drops (2019) Clin Ophthalmol, 13, pp. 1409-1425; Epstein, S.P., Ahdoot, M., Marcus, E., Asbell, P.A., Comparative toxicity of preservatives on immortalized corneal and conjunctival epithelial cells (2009) J Ocul Pharmacol Ther, 25 (2), pp. 113-119; Maulvi, F.A., Soni, T.G., Shah, D.O., A review on therapeutic contact lenses for ocular drug delivery (2016) Drug Deliv, 23 (8), pp. 3017-3026; Minami, T., Ishida, W., Kishimoto, T., Nakajima, I., Hino, S., Arai, R., In vitro and in vivo performance of epinastine hydrochloride-releasing contact lenses (2019) PLoS One, 14 (1), p. e0210362; Zaki, M., Pardo, J., Carracedo, G., A review of international medical device regulations: contact lenses and lens care solutions (2019) Cont Lens Anterior Eye, 42 (2), pp. 136-146; Maulvi, F.A., Choksi, H.H., Desai, A.R., Patel, A.S., Ranch, K.M., Vyas, B.A., pH triggered controlled drug delivery from contact lenses: addressing the challenges of drug leaching during sterilization and storage (2017) Colloids Surf B Biointerfaces, 157, pp. 72-82; Baenninger, P.B., Survey on bandage contact lens practice in the united kingdom (2014) J Clin Exp Ophthalmol, 5 (1), p. 2; Miller, D.D., Hasan, S.A., Simmons, N.L., Stewart, M.W., Recurrent corneal erosion: a comprehensive review (2019) Clin Ophthalmol, 13, pp. 325-335; Karlgard, C.C.S., Jones, L.W., Moresoli, C., Survey of bandage lens use in North America, october-december 2002 (2004) Eye Contact Lens, 30 (1), p. 25; Soluri, A., Hui, A., Jones, L., Delivery of ketotifen fumarate by commercial contact lens materials (2012) Optom Vis Sci, 89 (8), pp. 1140-1149; Karlgard, C.C., Wong, N.S., Jones, L.W., Moresoli, C., In vitro uptake and release studies of ocular pharmaceutical agents by silicon-containing and p-HEMA hydrogel contact lens materials (2003) Int J Pharm, 257 (1-2), pp. 141-151; Hui, A., Boone, A., Jones, L., Uptake and release of ciprofloxacin-HCl from conventional and silicone hydrogel contact lens materials (2008) Eye Contact Lens, 34 (5), pp. 266-271; Karlgard, C.C., Jones, L.W., Moresoli, C., Ciprofloxacin interaction with silicon-based and conventional hydrogel contact lenses (2003) Eye Contact Lens, 29 (2), pp. 83-89; Karlgard, C., Jones, L., Moresoli, C., Uptake and release of ciloxan from silicone-hydrogel and conventional hydrogel contact lens materials (2001) Invest Ophthalmol Vis Sci, 42 (4), p. s592; Bajgrowicz, M., Phan, C.M., Subbaraman, L.N., Jones, L., Release of ciprofloxacin and moxifloxacin from daily disposable contact lenses from an in vitro eye model (2015) Invest Ophthalmol Vis Sci, 56 (4), pp. 2234-2242; Dixon, P., Chauhan, A., Effect of the surface layer on drug release from delefilcon-A (Dailies Total1((r))) contact lenses (2017) Int J Pharm, 529 (1-2), pp. 89-101; Tian, X., Iwatsu, M., Sado, K., Kanai, A., Studies on the uptake and release of fluoroquinolones by disposable contact lenses (2001) CLAO J, 27 (4), pp. 216-220; Tian, X., Iwatsu, M., Kanai, A., Disposable 1-day Acuvue® contact lenses for the delivery of lomefloxacin to rabbits’ eyes (2001) CLAO J, 27 (4), pp. 212-215; Phan, C.M., Subbaraman, L.N., Jones, L., In vitro uptake and release of natamycin from conventional and silicone hydrogel contact lens materials (2013) Eye Contact Lens, 39 (2), pp. 162-168; Boone, A., Hui, A., Jones, L., Uptake and release of dexamethasone phosphate from silicone hydrogel and group I, II, and IV hydrogel contact lenses (2009) Eye Contact

Lens, 35 (5), pp. 260-267; Hui, A., Bajgrowicz-Cieslak, M., Phan, C.M., Jones, L., In vitro release of two anti-muscarinic drugs from soft contact lenses (2017) Clin Ophthalmol, 11, pp. 1657-1665; Jaishankar, D., Buhrman, J.S., Valyi-Nagy, T., Gemeinhart, R.A., Shukla, D., Extended release of an anti-heparan sulfate peptide from a contact lens suppresses corneal Herpes simplex virus-1 infection (2016) Invest Ophthalmol Vis Sci, 57 (1), pp. 169-180; Sharma, M., Bhowmick, R., Gappa-Fahlenkamp, H., Drug-loaded nanoparticles embedded in a biomembrane provide a dual-release mechanism for drug delivery to the eye (2016) J Ocul Pharmacol Ther, 32 (9), pp. 565-573; Peng, C.C., Burke, M.T., Chauhan, A., Transport of topical anesthetics in vitamin E loaded silicone hydrogel contact lenses (2012) Langmuir, 28 (2), pp. 1478-1487; Gulsen, D., Chauhan, A., Ophthalmic drug delivery through contact lenses (2004) Invest Ophthalmol Vis Sci, 45 (7), pp. 2342-2347; Gulsen, D., Li, C.C., Chauhan, A., Dispersion of DMPC liposomes in contact lenses for ophthalmic drug delivery (2005) Curr Eye Res, 30 (12), pp. 1071-1080; Dominguez-Godinez, C.O., Martin-Gil, A., Carracedo, G., Guzman-Aranguez, A., González-Méijome, J.M., Pintor, J., In vitro and in vivo delivery of the secretagogue diadenosine tetraphosphate from conventional and silicone hydrogel soft contact lenses (2013) J Optom, 6 (4), pp. 205-211; Dominguez-Godinez, C., Carracedo, G., Pintor, J., Diquafosol delivery from silicone hydrogel contact lenses: improved effect on tear secretion (2018) J Ocul Pharmacol Ther, 34 (1-2), pp. 170-176; Dixon, P., Fentzke, R.C., Bhattacharya, A., Konar, A., Hazra, S., Chauhan, A., In vitro drug release and in vivo safety of vitamin E and cysteamine loaded contact lenses (2018) Int J Pharm, 544 (2), pp. 380-391; Horne, R.R., Rich, J.T., Bradley, M.W., Pitt, W.G., Latanoprost uptake and release from commercial contact lenses (2020) J Biomater Sci Polym Ed, 31 (1), pp. 1-19; Horne, R.R., Judd, K.E., Pitt, W.G., Rapid loading and prolonged release of latanoprost from a silicone hydrogel contact lens (2017) J Drug Deliv Sci Technol, 41, pp. 410-418; Mohammadi, S., Jones, L., Gorbet, M., Extended latanoprost release from commercial contact lenses: in vitro studies using corneal models (2014) PLoS One, 9 (9), p. e106653; Hui, A., Contact lenses for ophthalmic drug delivery (2017) Clin Exp Optom, 100 (5), pp. 494-512; Phan, C.M., Bajgrowicz-Cieslak, M., Subbaraman, L.N., Jones, L., Release of moxifloxacin from contact lenses using an in vitro eye model: impact of artificial tear fluid composition and mechanical rubbing (2016) Transl Vis Sci Technol, 5 (6), p. 3; Zarbin, M.A., Montemagno, C., Leary, J.F., Ritch, R., Nanotechnology in ophthalmology (2010) Can J Ophthalmol, 45 (5), pp. 457-476; Kang-Mieler, J.J., Rudeen, K.M., Liu, W., Mieler, W.F., Advances in ocular drug delivery systems (2020) Eye (Lond), 34 (8), pp. 1371-1379; Khan, I., Saeed, K., Khan, I., Nanoparticles: properties, applications and toxicities (2019) Arab J Chem, 12 (7), pp. 908-931; Husseini, G.A., Pitt, W.G., Micelles and nanoparticles for ultrasonic drug and gene delivery (2008) Adv Drug Deliv Rev, 60 (10), pp. 1137-1152; Mc Carthy, D.J., Malhotra, M., O'Mahony, A.M., Cryan, J.F., O'Driscoll, C.M., Nanoparticles and the blood-brain barrier: advancing from in-vitro models towards therapeutic significance (2015) Pharm Res, 32 (4), pp. 1161-1185; Honda, M., Asai, T., Oku, N., Araki, Y., Tanaka, M., Ebihara, N., Liposomes and nanotechnology in drug development: focus on ocular targets (2013) Int J Nanomedicine, 8, pp. 495-503; Panzarini, E., Inguscio, V., Tenuzzo, B.A., Carata, E., Dini, L., Nanomaterials and autophagy: New insights in cancer treatment (2013) Cancers, 5 (1), pp. 296-319; Balamurugan, K., Chintamani, P., Lipid nano particulate drug delivery: an overview of the emerging trend (2018) The Pharma Innovation Journal, 7 (7), pp. 779-789; Jung, H.J., Abou-Jaoude, M., Carbia, B.E., Plummer, C., Chauhan, A., Glaucoma therapy by extended release of timolol from nanoparticle loaded silicone-hydrogel

contact lenses (2013) J Control Release, 165 (1), pp. 82-89; Kharaghani, D., Dutta, D., Gitigard, P., Tamada, Y., Katagiri, A., Phan, D.-N., Development of antibacterial contact lenses containing metallic nanoparticles (2019) Polym Test, 79; Mourad, R., Helaly, F., Darwesh, O., El-Sawy, S., Antimicrobial and physicomechanical natures of silver nanoparticles incorporated into silicone-hydrogel films (2019) Cont Lens Anterior Eye, 42 (3), pp. 325-333; Maulvi, F.A., Lakdawala, D.H., Shaikh, A.A., Desai, A.R., Choksi, H.H., Vaidya, R.J., In vitro and in vivo evaluation of novel implantation technology in hydrogel contact lenses for controlled drug delivery (2016) J Control Release, 226, pp. 47-56; Maulvi, F.A., Mangukiya, M.A., Patel, P.A., Vaidya, R.J., Koli, A.R., Ranch, K.M., Extended release of ketotifen from silica shell nanoparticle-laden hydrogel contact lenses: in vitro and in vivo evaluation (2016) J Mater Sci Mater Med, 27 (6), p. 113; Mun, J., Jw, M., Jeong, S., Cho, S., Joo, C.-K., Hahn, S.K., Drug-eluting contact lens containing cyclosporine-loaded cholesterol-hyaluronate micelles for dry eye syndrome (2019) RSC Adv, 9 (29), pp. 16578-16585; Ealias, A.M., Saravanakumar, M., A review on the classification, characterisation, synthesis of nanoparticles and their application (2017) IOP Conf Ser Mater Sci Eng., 263. , 032019; Carvalho, I.M., Marques, C.S., Oliveira, R.S., Coelho, P.B., Costa, P.C., Ferreira, D.C., Sustained drug release by contact lenses for glaucoma treatment-a review (2015) J Control Release, 202, pp. 76-82; Choi, S.W., Kim, J., Therapeutic contact lenses with polymeric vehicles for ocular drug delivery: a review (2018) Materials (Basel), 11 (7), p. 1125; Singh, R.B., Ichhpujani, P., Thakur, S., Jindal, S., Promising therapeutic drug delivery systems for glaucoma: a comprehensive review (2020) Ther Adv Ophthalmol, 12; Maulvi, F.A., Soni, T.G., Shah, D.O., Extended release of timolol from ethyl cellulose microparticles laden hydrogel contact lenses (2015) Open Pharm Sci J, 2 (1); Garhwal, R., Shady, S.F., Ellis, E.J., Ellis, E.J., Leahy, C.D., McCarthy, S.P., Sustained ocular delivery of ciprofloxacin using nanospheres and conventional contact lens materials (2012) Invest Ophthalmol Vis Sci, 53 (3), pp. 1341-1352; Kapoor, Y., Thomas, J.C., Tan, G., John, V.T., Chauhan, A., Surfactant-laden soft contact lenses for extended delivery of ophthalmic drugs (2009) Biomaterials, 30 (5), pp. 867-878; Nasr, F.H., Khoee, S., Dehghan, M.M., Chaleshtori, S.S., Shafiee, A., Preparation and evaluation of contact lenses embedded with polycaprolactone-based nanoparticles for ocular drug delivery (2016) Biomacromolecules, 17 (2), pp. 485-495; Phan, C.M., Subbaraman, L., Liu, S., Gu, F., Jones, L., In vitro uptake and release of natamycin Dex-b-PLA nanoparticles from model contact lens materials (2014) J Biomater Sci Polym Ed, 25 (1), pp. 18-31; ElShaer, A., Mustafa, S., Kasar, M., Thapa, S., Ghatora, B., Alany, R.G., Nanoparticle-laden contact lens for controlled ocular delivery of Prednisolone: formulation optimization using statistical experimental design (2016) Pharmaceutics, 8 (2), p. 14; Mehta, P., Al-Kinani, A.A., Haj-Ahmad, R., Arshad, M.S., Chang, M.W., Alany, R.G., Electrically atomised formulations of timolol maleate for direct and on-demand ocular lens coatings (2017) Eur J Pharm Biopharm, 119, pp. 170-184; Bin Sahadan, M.Y., Tong, W.Y., Tan, W.N., Leong, C.R., Bin Misri, M.N., Chan, M., Phomopsidione nanoparticles coated contact lenses reduce microbial keratitis causing pathogens (2019) Exp Eye Res, 178, pp. 10-14; Jung, H.J., Chauhan, A., Temperature sensitive contact lenses for triggered ophthalmic drug delivery (2012) Biomaterials, 33 (7), pp. 2289-2300; Hu, X., Tan, H., Chen, P., Wang, X., Pang, J., Polymer micelles laden hydrogel contact lenses for ophthalmic drug delivery (2016) J Nanosci Nanotechnol, 16 (6), pp. 5480-5488; Paradiso, P., Colaco, R., Mata, J.L.G., Krastev, R., Saramago, B., Serro, A.P., Drug release from liposome coated hydrogels for soft contact lenses: the blinking and temperature effect (2017) J

Biomed Mater Res B Appl Biomater, 105 (7), pp. 1799-1807; Danion, A., Brochu, H., Martin, Y., Vermette, P., Fabrication and characterization of contact lenses bearing surface-immobilized layers of intact liposomes (2007) J Biomed Mater Res A, 82 (1), pp. 41-51; Venkatesh, N., Bhowmik, H., Kuila, A., Metallic nanoparticle: a review (2018) Biomed J Sci Tech Res, 4 (2), pp. 3765-3775; Huang, Y.W., Cambre, M., Lee, H.J., The toxicity of nanoparticles depends on multiple molecular and physicochemical mechanisms (2017) Int J Mol Sci, 18 (12), p. 2702; Malik, M.A., Wani, M.Y., Hashim, M.A., Microemulsion method: a novel route to synthesize organic and inorganic nanomaterials: 1st nano update (2012) Arab J Chem, 5 (4), pp. 397-417; Ganguli, A.K., Ganguly, A., Vaidya, S., Microemulsion-based synthesis of nanocrystalline materials (2010) Chem Soc Rev, 39 (2), pp. 474-485; McClements, D.J., Nanoemulsions versus microemulsions: terminology, differences, and similarities (2012) Soft Matter, 8 (6), pp. 1719-1729; Agrawal, O.P., Agrawal, S., An overview of new drug delivery system: microemulsion (2012) Asian J Pharm Sci Tech, 2, pp. 5-12; Rane, A.V., Kanny, K., Abitha, V., Thomas, S., Methods for synthesis of nanoparticles and fabrication of nanocomposites. Synthesis of inorganic nanomaterials (2018), pp. 121-139. , Woodhead Publishing; Maulvi, F.A., Desai, A.R., Choksi, H.H., Patil, R.J., Ranch, K.M., Vyas, B.A., Effect of surfactant chain length on drug release kinetics from microemulsion-laden contact lenses (2017) Int J Pharm, 524 (1-2), pp. 193-204; Lee, S.H., Kim, H.J., Kim, D.H., Chang, W.S., Vales, T.P., Kim, H.J., Thermo-sensitive nanogel-laden bicontinuous microemulsion drug-eluting contact lenses (2019) J Biomed Mater Res B Appl Biomater, 107 (4), pp. 1159-1169; Li, C.C., Abrahamson, M., Kapoor, Y., Chauhan, A., Timolol transport from microemulsions trapped in HEMA gels (2007) J Colloid Interface Sci, 315 (1), pp. 297-306; Xu, W., Jiao, W., Li, S., Tao, X., Mu, G., Bimatoprost loaded microemulsion laden contact lens to treat glaucoma (2019) J Drug Deliv Sci Technol, 54; Torres-Luna, C., Hu, N., Koolivand, A., Fan, X., Zhu, Y., Domszy, R., Effect of a cationic surfactant on microemulsion globules and drug release from hydrogel contact lenses (2019) Pharmaceutics, 11 (6), p. 262; Li, Y., Huang, C., Yang, X., Zhang, X., Ofloxacin laden microemulsion contact lens to treat conjunctivitis (2020) J Biomater Sci Polym Ed, 31 (12), pp. 1566-1579; Wei, N., Dang, H., Huang, C., Sheng, Y., Timolol loaded microemulsion laden silicone contact lens to manage glaucoma: in vitro and in vivo studies (2020) J Dispers Sci Technol, pp. 1-9; Kapoor, Y., Chauhan, A., Ophthalmic delivery of cyclosporine a from Brij-97 microemulsion and surfactant-laden p-HEMA hydrogels (2008) Int J Pharm, 361 (1-2), pp. 222-229; Sekar, P., Chauhan, A., Effect of vitamin-e integration on delivery of prostaglandin analogs from therapeutic lenses (2019) J Colloid Interface Sci, 539, pp. 457-467; Ubani-Ukoma, U.G., Schultz, G., Silva, B.O., Chauhan, A., Evaluating the potential of drug eluting contact lenses for treatment of bacterial keratitis using an ex vivo corneal model (2019) Int J Pharm, 565, pp. 499-508; Kim, J., Peng, C.C., Chauhan, A., Extended release of dexamethasone from silicone-hydrogel contact lenses containing vitamin E (2010) J Control Release, 148 (1), pp. 110-116; Peng, C.C., Burke, M.T., Carbia, B.E., Plummer, C., Chauhan, A., Extended drug delivery by contact lenses for glaucoma therapy (2012) J Control Release, 162 (1), pp. 152-158; Paradiso, P., Serro, A.P., Saramago, B., Colaco, R., Chauhan, A., Controlled release of antibiotics from vitamin e-loaded silicone-hydrogel contact lenses (2016) J Pharm Sci, 105 (3), pp. 1164-1172; Rad, M.S., Mohajeri, S.A., Simultaneously load and extended release of betamethasone and ciprofloxacin from vitamin e-loaded silicone-based soft contact lenses (2016) Curr Eye Res, 41 (9), pp. 1185-1191; Lee, D., Cho, S., Park, H.S., Kwon, I., Ocular drug delivery through pHEMA-hydrogel contact lenses co-loaded with lipophilic vitamins (2016) Sci Rep, 6, p. 34194; Dixon,

P.G., Mondal, K., Konar, A., Chauhan, A., Hazra, S., Controlled delivery of pirfenidone through vitamin E-loaded contact lens ameliorates corneal inflammation (2018) Drug Deliv Transl Res, 8 (5), pp. 1114-1126; Alvarez-Lorenzo, C., Concheiro, A., Molecularly imprinted polymers for drug delivery (2004) J Chromatogr B Analyt Technol Biomed Life Sci, 804 (1), pp. 231-245; Hiratani, H., Alvarez-Lorenzo, C., The nature of backbone monomers determines the performance of imprinted soft contact lenses as timolol drug delivery systems (2004) Biomaterials, 25 (6), pp. 1105-1113; Alvarez-Lorenzo, C., Hiratani, H., Gomez-Amoza, J.L., Martinez-Pacheco, R., Souto, C., Concheiro, A., Soft contact lenses capable of sustained delivery of timolol (2002) J Pharm Sci, 91 (10), pp. 2182-2192; Hiratani, H., Fujiwara, A., Tamiya, Y., Mizutani, Y., Alvarez-Lorenzo, C., Ocular release of timolol from molecularly imprinted soft contact lenses (2005) Biomaterials, 26 (11), pp. 1293-1298; Ali, M., Horikawa, S., Venkatesh, S., Saha, J., Hong, J.W., Byrne, M.E., Zero-order therapeutic release from imprinted hydrogel contact lenses within in vitro physiological ocular tear flow (2007) J Control Release, 124 (3), pp. 154-162; Gonzalez-Chomon, C., Silva, M., Concheiro, A., Alvarez-Lorenzo, C., Biomimetic contact lenses eluting olopatadine for allergic conjunctivitis (2016) Acta Biomater, 41, pp. 302-311; Alvarez-Lorenzo, C., Yañez, F., Barreiro-Iglesias, R., Concheiro, A., Imprinted soft contact lenses as norfloxacin delivery systems (2006) J Control Release, 113 (3), pp. 236-244; Hui, A., Sheardown, H., Jones, L., Acetic and acrylic acid molecular imprinted model silicone hydrogel materials for ciprofloxacin-HCl delivery (2012) Materials (Basel), 5 (1), pp. 85-107; Hui, A., Willcox, M., Jones, L., In vitro and in vivo evaluation of novel ciprofloxacin-releasing silicone hydrogel contact lenses (2014) Invest Ophthalmol Vis Sci, 55 (8), pp. 4896-4904; Malakooti, N., Alexander, C., Alvarez-Lorenzo, C., Imprinted contact lenses for sustained release of polymyxin b and related antimicrobial peptides (2015) J Pharm Sci, 104 (10), pp. 3386-3394; Andrade-Vivero, P., Fernandez-Gabriel, E., Alvarez-Lorenzo, C., Concheiro, A., Improving the loading and release of nsaids from pHEMA hydrogels by copolymerization with functionalized monomers (2007) J Pharm Sci, 96 (4), pp. 802-813; Malaekeh-Nikouei, B., Ghaeni, F.A., Motamedshariaty, V.S., Mohajeri, S.A., Controlled release of prednisolone acetate from molecularly imprinted hydrogel contact lenses (2012) J Appl Polym Sci Symp, 126 (1), pp. 387-394; Tieppo, A., Pate, K.M., Byrne, M.E., In vitro controlled release of an anti-inflammatory from daily disposable therapeutic contact lenses under physiological ocular tear flow (2012) Eur J Pharm Biopharm, 81 (1), pp. 170-177; Malaekeh-Nikouei, B., Vahabzadeh, S.A., Mohajeri, S.A., Preparation of a molecularly imprinted soft contact lens as a new ocular drug delivery system for dorzolamide (2013) Curr Drug Deliv, 10 (3), pp. 279-285; Yan, F., Liu, Y., Han, S., Zhao, Q., Liu, N., Bimatoprost imprinted silicone contact lens to treat glaucoma (2020) AAPS PharmSciTech, 21 (2), p. 63; Ali, M., Byrne, M.E., Controlled release of high molecular weight hyaluronic acid from molecularly imprinted hydrogel contact lenses (2009) Pharm Res, 26 (3), pp. 714-726; White, C.J., McBride, M.K., Pate, K.M., Tieppo, A., Byrne, M.E., Extended release of high molecular weight hydroxypropyl methylcellulose from molecularly imprinted, extended wear silicone hydrogel contact lenses (2011) Biomaterials, 32 (24), pp. 5698-5705; Hiratani, H., Mizutani, Y., Alvarez-Lorenzo, C., Controlling drug release from imprinted hydrogels by modifying the characteristics of the imprinted cavities (2005) Macromol Biosci, 5 (8), pp. 728-733; Tieppo, A., White, C.J., Paine, A.C., Voyles, M.L., McBride, M.K., Byrne, M.E., Sustained in vivo release from imprinted therapeutic contact lenses (2012) J Control Release, 157 (3), pp. 391-397; Behl, G., Iqbal, J., O'Reilly, N.J., McLoughlin, P., Fitzhenry, L.,

Synthesis and characterization of poly(2-hydroxyethylmethacrylate) contact lenses containing chitosan nanoparticles as an ocular delivery system for dexamethasone sodium phosphate (2016) Pharm Res, 33 (7), pp. 1638-1648; Lee, D., Lee, N., Kwon, I., Efficient loading of ophthalmic drugs with poor loadability into contact lenses using functional comonomers (2018) Biomater Sci, 6 (10), pp. 2639-2646; Kakisu, K., Matsunaga, T., Kobayakawa, S., Sato, T., Tochikubo, T., Development and efficacy of a drug-releasing soft contact lens (2013) Invest Ophthalmol Vis Sci, 54 (4), pp. 2551-2561; Kim, G., Kim, H.J., Noh, H., Influence of solution pH on drug release from ionic hydrogel lens (2019) Macromol Res, 27 (2), pp. 191-197; Kanemoto, M., Sato, T., Aoyama, A., Matsunaga, T., Uno, K., Toshida, H., The interaction and compatibility between a soft contact lens and an ophthalmic drug (2006) Eye Contact Lens, 32 (4), pp. 192-196; Kamberi, M., Tsutsumi, K., Kotegawa, T., Kawano, K., Nakamura, K., Niki, Y., Influences of urinary pH on ciprofloxacin pharmacokinetics in humans and antimicrobial activity in vitro versus those of sparfloxacin (1999) Antimicrob Agents Chemother, 43 (3), pp. 525-529; Tranoudis, I., Efron, N., Water properties of soft contact lens materials (2004) Cont Lens Anterior Eye, 27 (4), pp. 193-208; Luensmann, D., Jones, L., Protein deposition on contact lenses: the past, the present, and the future (2012) Cont Lens Anterior Eye, 35 (2), pp. 53-64; Xu, J.L., Sun, F., Cyclodextrin-containing hydrogels for contact lenses as a platform for drug incorporation and release (2010) Acta Biomater, 6 (2), pp. 486-493; Phan, C.M., Subbaraman, L.N., Jones, L., In vitro drug release of natamycin from beta-cyclodextrin and 2-hydroxypropyl-beta-cyclodextrin-functionalized contact lens materials (2014) J Biomater Sci Polym Ed, 25 (17), pp. 1907-1919; Ciolino, J.B., Hoare, T.R., Iwata, N.G., Behlau, I., Dohlman, C.H., Langer, R., A drug-eluting contact lens (2009) Invest Ophthalmol Vis Sci, 50 (7), pp. 3346-3352; Ciolino, J.B., Stefanescu, C.F., Ross, A.E., Salvador-Culla, B., Cortez, P., Ford, E.M., In vivo performance of a drug-eluting contact lens to treat glaucoma for a month (2014) Biomaterials, 35 (1), pp. 432-439; Ciolino, J.B., Ross, A.E., Tulsan, R., Watts, A.C., Wang, R.F., Zurakowski, D., Latanoprost-eluting contact lenses in glaucomatous monkeys (2016) Ophthalmology, 123 (10), pp. 2085-2092; Ross, A.E., Bengani, L.C., Tulsan, R., Maidana, D.E., Salvador-Culla, B., Kobashi, H., Topical sustained drug delivery to the retina with a drug-eluting contact lens (2019) Biomaterials, 217; Maulvi, F.A., Singhania, S.S., Desai, A.R., Shukla, M.R., Tannk, A.S., Ranch, K.M., Contact lenses with dual drug delivery for the treatment of bacterial conjunctivitis (2018) Int J Pharm, 548 (1), pp. 139-150; Maulvi, F.A., Shaikh, A.A., Lakdawala, D.H., Desai, A.R., Pandya, M.M., Singhania, S.S., Design and optimization of a novel implantation technology in contact lenses for the treatment of dry eye syndrome: in vitro and in vivo evaluation (2017) Acta Biomater, 53, pp. 211-221; Koffler, B., McDonald, M., Nelinson, D., Group, L., Improved signs, symptoms, and quality of life associated with dry eye syndrome: hydroxypropyl cellulose ophthalmic insert patient registry (2010) Eye Contact Lens, 36 (3), pp. 170-176; McDonald, M., D'Aversa, G., Perry, H.D., Wittpenn, J.R., Donnenfeld, E.D., Nelinson, D.S., Hydroxypropyl cellulose ophthalmic inserts (lacrisert) reduce the signs and symptoms of dry eye syndrome and improve patient quality of life (2009) Trans Am Ophthalmol Soc, 107, pp. 214-221; Luchs, J., Nelinson, D., Macy, J., Group, L., Efficacy of hydroxypropyl cellulose ophthalmic inserts (LACRISERT) in subsets of patients with dry eye syndrome: findings from a patient registry (2010) Cornea, 29 (12), pp. 1417-1427; McDonald, M., D'Aversa, G., Perry, H.D., Wittpenn, J.R., Nelinson, D.S., Correlating patient-reported response to hydroxypropyl cellulose ophthalmic insert (LACRISERT(r)) therapy with clinical outcomes:

tools for predicting response (2010) Curr Eye Res, 35 (10), pp. 880-887; Pitt, W.G., Jack, D.R., Zhao, Y., Nelson, J.L., Pruitt, J.D., Loading and release of a phospholipid from contact lenses (2011) Optom Vis Sci, 88 (4), pp. 502-506; de Paiva, C.S., Pflugfelder, S.C., Ng, S.M., Akpek, E.K., Topical cyclosporine A therapy for dry eye syndrome (2019) Cochrane Database Syst Rev, 9, p. CD010051; Peng, C.C., Chauhan, A., Extended cyclosporine delivery by silicone-hydrogel contact lenses (2011) J Control Release, 154 (3), pp. 267-274; Choi, J.H., Li, Y., Jin, R., Shrestha, T., Choi, J.H.S., Lee, W.J., The efficiency of cyclosporine a-eluting contact lenses for the treatment of dry eye (2019) Curr Eye Res, 44 (5), pp. 486-496; Jones, L., Downie, L.E., Korb, D., Benitez-Del-Castillo, J.M., Dana, R., Deng, S.X., TFOS DEWS II management and therapy report (2017) Ocul Surf, 15 (3), pp. 575-628; Peng, C.C., Kim, J., Chauhan, A., Extended delivery of hydrophilic drugs from silicone-hydrogel contact lenses containing vitamin E diffusion barriers (2010) Biomaterials, 31 (14), pp. 4032-4047; Macoul, K.L., Pavan-Langston, D., Pilocarpine ocusert system for sustained control of ocular hypertension (1975) Arch Ophthalmol, 93 (8), pp. 587-590; Brandt, J.D., Sall, K., DuBiner, H., Benza, R., Alster, Y., Walker, G., Six-month intraocular pressure reduction with a topical bimatoprost ocular insert: results of a phase ii randomized controlled study (2016) Ophthalmology, 123 (8), pp. 1685-1694; Peng, C.C., Ben-Shlomo, A., Mackay, E.O., Plummer, C.E., Chauhan, A., Drug delivery by contact lens in spontaneously glaucomatous dogs (2012) Curr Eye Res, 37 (3), pp. 204-211; Taniguchi, E.V., Kalout, P., Pasquale, L.R., Kohane, D.S., Ciolino, J.B., Clinicians’ perspectives on the use of drug-eluting contact lenses for the treatment of glaucoma (2014) Ther Deliv, 5 (10), pp. 1077-1083; Xu, J., Xue, Y., Hu, G., Lin, T., Gou, J., Yin, T., A comprehensive review on contact lens for ophthalmic drug delivery (2018) J Control Release, 281, pp. 97-118; Busin, M., Spitznas, M., Sustained gentamicin release by presoaked medicated bandage contact lenses (1988) Ophthalmology, 95 (6), pp. 796-798; Kalayci, D., Basci, N., Kortunay, S., Hasiripi, H., Bozkurt, A., Penetration of topical ciprofloxacin by presoaked medicated soft contact lenses (1999) CLAO J, 25 (3), pp. 182-184; Kennedy, S.M., Deshpande, P., Gallagher, A.G., Horsburgh, M.J., Allison, H.E., Kaye, S.B., Antimicrobial activity of poly-epsilon-lysine peptide hydrogels against Pseudomonas aeruginosa (2020) Invest Ophthalmol Vis Sci, 61 (10), p. 18; Gallagher, A.G., McLean, K., Stewart, R.M.K., Wellings, D.A., Allison, H.E., Williams, R.L., Development of a poly-epsilon-lysine contact lens as a drug delivery device for the treatment of fungal keratitis (2017) Invest Ophthalmol Vis Sci, 58 (11), pp. 4499-4505; Gallagher, A.G., Alorabi, J.A., Wellings, D.A., Lace, R., Horsburgh, M.J., Williams, R.L., A novel peptide hydrogel for an antimicrobial bandage contact lens (2016) Adv Healthc Mater, 5 (16), pp. 2013-2018; Lace, R., Doherty, K.G., Dutta, D., Willcox, M.D.P., Williams, R.L., Poly-ε-lysine or Mel4 antimicrobial surface modification on a novel peptide hydrogel bandage contact lens (2020) Adv Mater Interfaces, 7; Bielory, L., Friedlaender, M.H., Allergic conjunctivitis (2008) Immunol Allergy Clin North Am, 28 (1), pp. 43-58. , vi; Abelson, M.B., McLaughlin, J.T., Gomes, P.J., Antihistamines in ocular allergy: Are they all created equal? (2011) Curr Allergy Asthma Rep, 11 (3), pp. 205-211; Gomes, P.J., Trends in prevalence and treatment of ocular allergy (2014) Curr Opin Allergy Clin Immunol, 14 (5), pp. 451-456; Pitt, A.D., Smith, A.F., Lindsell, L., Voon, L.W., Rose, P.W., Bron, A.J., Economic and quality-of-life impact of seasonal allergic conjunctivitis in oxfordshire (2004) Ophthalmic Epidemiol, 11 (1), pp. 17-33; Lemp, M.A., Contact lenses and allergy (2008) Curr Opin Allergy Clin Immunol, 8 (5), pp. 457-460; Najmi, H., Mobarki, Y., Mania, K., Altowairqi, B., Basehi, M., Mahfouz, M.S., The correlation between keratoconus

and eye rubbing: a review (2019) Int J Ophthalmol, 12 (11), pp. 1775-1781; Ackerman, S., Smith, L.M., Gomes, P.J., Ocular itch associated with allergic conjunctivitis: latest evidence and clinical management (2016) Ther Adv Chronic Dis, 7 (1), pp. 52-67; Wolffsohn, J.S., Emberlin, J.C., Role of contact lenses in relieving ocular allergy (2011) Cont Lens Anterior Eye, 34 (4), pp. 169-172; Xue, Y., Zhang, W., Lei, Y., Dang, M., Novel polyvinyl pyrrolidone-loaded olopatadine HCl-laden doughnut contact lens to treat allergic conjunctivitis (2020) J Pharm Sci, 109 (5), pp. 1714-1724; Vistakon, Safety study of a contact lens with ketotifen in healthy, normal volunteers (a). ClinicalTrials.gOv (2007), NIH-US National Library of Medicine; Vistakon, Safety study of a contact lens with ketotifen in healthy, normal volunteers (b). ClinicalTrials.gOv (2009), NIH- US National Library of Medicine; Vistakon, Evaluation of efficacy and safety of an anti-allergy drug with a contact lens in allergic conjunctivitis (a). ClinicalTrials.gOv (2007), NIH - US National Library of Medicine; Vistakon, Evaluation of efficacy and safety of an anti-allergy drug with a contact lens in the treatment of allergic conjunctivitis (b). ClinicalTrials.gOv (2007), NIH-US National Library of Medicine; Raja, R.R., Mahadevan, S., Alli, A., Molock, F., Pall, B., Methods and ophthalmic devices used in the treatment of ocular allergies (2017) Google Patents. US10045975B2, , Johnson and Johnson Vision Care Inc USA; Pall, B., Gomes, P., Yi, F., Torkildsen, G., Management of ocular allergy itch with an antihistamine-releasing contact lens (2019) Cornea, 38 (6), pp. 713-717; Linsley, C.S., Wu, B.M., Recent advances in light-responsive on-demand drug-delivery systems (2017) Ther Deliv, 8 (2), pp. 89-107; Li, C., Wang, J., Wang, Y., Gao, H., Wei, G., Huang, Y., Recent progress in drug delivery (2019) Acta Pharm Sin B, 9 (6), pp. 1145-1162; Li, L., Scheiger, J.M., Levkin, P.A., Design and applications of photoresponsive hydrogels (2019) Adv Mater, 31 (26), p. e18073333; Geng, S., Wang, Y., Wang, L., Kouyama, T., Gotoh, T., Wada, S., A light-responsive self-assembly formed by a cationic azobenzene derivative and SDS as a drug delivery system (2017) Sci Rep, 7 (1), p. 39202; Behar-Cohen, F., Baillet, G., de Ayguavives, T., Garcia, P.O., Krutmann, J., Pena-Garcia, P.O., Ultraviolet damage to the eye revisited: eye-sun protection factor (e-spf(r)), a new ultraviolet protection label for eyewear (2014) Clin Ophthalmol, 8, pp. 87-104; Söderberg, P., Talebizadeh, N., Yu, Z., Galichanin, K., Does infrared or ultraviolet light damage the lens? (2016) Eye, 30 (2), pp. 241-246; Kim, J., Him, H., Lee, S., (2017), D. Kim. Thermo-responsive hydrogel contact lens for controlled drug delivery and manufacturing method thereof. Google Patents. KR102009571B1. Korea;; Kim, Y.-J., Matsunaga, Y.T., Thermo-responsive polymers and their application as smart biomaterials (2017) J Mater Chem B, 5 (23), pp. 4307-4321; Kopecek, J., Hydrogels from soft contact lenses and implants to self-assembled nanomaterials (2009) J Polym Sci Part A: Polym Chem, 47 (22), pp. 5929-5946; Matsumoto, K., Sakikawa, N., Miyata, T., Thermo-responsive gels that absorb moisture and ooze water (2018) Nat Commun, 9 (1), pp. 1-7; Villa, C., Martello, F., Erratico, S., Tocchio, A., Belicchi, M., Lenardi, C., P(NIPAAM-co-HEMA) thermoresponsive hydrogels: an alternative approach for muscle cell sheet engineering (2017) J Tissue Eng Regen Med, 11 (1), pp. 187-196; Liu, Y.Y., Lu, J., Shao, Y.H., Preparation and characterization of poly(n-isopropylacrylamide)-modified poly(2-hydroxyethyl acrylate) hydrogels by interpenetrating polymer networks for sustained drug release (2006) Macromol Biosci, 6 (6), pp. 452-458; Ashraf, S., Park, H.-K., Park, H.-K., Lee, S.-H., Snapshot of phase transition in thermoresponsive hydrogel pnipam: role in drug delivery and tissue engineering (2016) Macromol Res, 24 (4), pp. 297-304; Fu, R., Klinngam, W., Heur, M., Edman, M.C., Hamm-Alvarez, S.F., Tear proteases and protease inhibitors: potential biomarkers and disease drivers in ocular surface

disease (2020) Eye Contact Lens, 46, pp. S70-S83; Ahlen, M., Tummala, G.K., Mihranyan, A., Nanoparticle-loaded hydrogels as a pathway for enzyme-triggered drug release in ophthalmic applications (2018) Int J Pharm, 536 (1), pp. 73-81; Kim, H.J., Zhang, K., Moore, L., Ho, D., Diamond nanogel-embedded contact lenses mediate lysozyme-dependent therapeutic release (2014) ACS Nano, 8 (3), pp. 2998-3005; Willcox, M.D., Microbial adhesion to silicone hydrogel lenses: a review (2013) Eye Contact Lens, 39 (1), pp. 61-66; Stapleton, F., Keay, L., Jalbert, I., Cole, N., The epidemiology of contact lens related infiltrates (2007) Optom Vis Sci, 84 (4), pp. 257-272; Stapleton, F., Keay, L., Edwards, K., Naduvilath, T., Dart, J.K., Brian, G., The incidence of contact lens-related microbial keratitis in australia (2008) Ophthalmology, 115 (10), pp. 1655-1662; Schein, O.D., McNally, J.J., Katz, J., Chalmers, R.L., Tielsch, J.M., Alfonso, E., The incidence of microbial keratitis among wearers of a 30-day silicone hydrogel extended-wear contact lens (2005) Ophthalmology, 112 (12), pp. 2172-2179; Willcox, M., Keir, N., Maseedupally, V., Masoudi, S., McDermott, A., Mobeen, R., CLEAR - contact lens wettability, cleaning, disinfection and interaction with tears (2021) Contact Lens Anterior Eye, 44 (2). , In press; Willcox, M.D.P., Hume, E.B.H., Vijay, A.K., Petcavich, R., Ability of silver-impregnated contact lenses to control microbial growth and colonisation (2010) J Optom, 3 (3), pp. 143-148; Kharaghani, D., Dutta, D., Gitigard, P., Tamada, Y., Katagiri, A., Phan, D.-N., Development of antibacterial contact lenses containing metallic nanoparticles (2019) Polym Test, 79, p. 106034; Nissen, S., Furkert, F.H., antimicrobial efficacy of a silver layer on hydrogel lenses citation] (2000) Ophthalmologe, 97 (9), pp. 640-643; Mourad, R., Helaly, F., Darwesh, O., Sawy, S.E., Antimicrobial and physicomechanical natures of silver nanoparticles incorporated into silicone-hydrogel films (2019) Cont Lens Anterior Eye, 42 (3), pp. 325-333; Willcox, M.D., Hume, E.B., Aliwarga, Y., Kumar, N., Cole, N., A novel cationic-peptide coating for the prevention of microbial colonization on contact lenses (2008) J Appl Microbiol, 105 (6), pp. 1817-1825; Dutta, D., Vijay, A.K., Kumar, N., Willcox, M.D., Melimine-coated antimicrobial contact lenses reduce microbial keratitis in an animal model (2016) Invest Ophthalmol Vis Sci, 57 (13), pp. 5616-5624; Dutta, D., Ozkan, J., Willcox, M.D., Biocompatibility of antimicrobial melimine lenses: rabbit and human studies (2014) Optom Vis Sci, 91 (5), pp. 570-581; Dutta, D., Kamphuis, B., Ozcelik, B., Thissen, H., Pinarbasi, R., Kumar, N., Development of silicone hydrogel antimicrobial contact lenses with Mel4 peptide coating (2018) Optom Vis Sci, 95 (10), pp. 937-946; Dutta, D., Cole, N., Kumar, N., Willcox, M.D., Broad spectrum antimicrobial activity of melimine covalently bound to contact lenses (2013) Invest Ophthalmol Vis Sci, 54 (1), pp. 175-182; Dutta, D., Zhao, T., Cheah, K.B., Holmlund, L., Willcox, M.D.P., Activity of a melimine derived peptide Mel4 against Stenotrophomonas, Delftia, Elizabethkingia, Burkholderia and biocompatibility as a contact lens coating (2017) Cont Lens Anterior Eye, 40 (3), pp. 175-183; Ren, D., Sims, J.J., Wood, T.K., Inhibition of biofilm formation and swarming of Bacillus subtilis by (5Z)-4-bromo-5-(bromomethylene)-3-butyl-2(5H)-furanone (2002) Lett Appl Microbiol, 34 (4), pp. 293-299; Jones, M.B., Jani, R., Ren, D., Wood, T.K., Blaser, M.J., Inhibition of Bacillus anthracis growth and virulence-gene expression by inhibitors of quorum-sensing (2005) J Infect Dis, 191 (11), pp. 1881-1888; George, M., Pierce, G., Gabriel, M., Morris, C., Ahearn, D., Effects of quorum sensing molecules of Pseudomonas aeruginosa on organism growth, elastase b production, and primary adhesion to hydrogel contact lenses (2005) Eye Contact Lens, 31 (2), pp. 54-61; Hentzer, M., Wu, H., Andersen, J.B., Riedel, K., Rasmussen, T.B., Bagge, N., Attenuation of Pseudomonas aeruginosa

virulence by quorum sensing inhibitors (2003) EMBO J, 22 (15), pp. 3803-3815; Read, R., Kumar, N., Willcox, M., Griesser, H., Muir, B., Thissen, H., Antimicrobial coatings (2003); Zhu, H., Kumar, A., Ozkan, J., Bandara, R., Ding, A., Perera, I., Fimbrolide-coated antimicrobial lenses: their in vitro and in vivo effects (2008) Optom Vis Sci, 85 (5), pp. 292-300; Taylor, R.L., Willcox, M.D., Williams, T.J., Verran, J., Modulation of bacterial adhesion to hydrogel contact lenses by albumin (1998) Optom Vis Sci, 75 (1), pp. 23-29; Thissen, H., Gengenbach, T., du Toit, R., Sweeney, D.F., Kingshott, P., Griesser, H.J., Clinical observations of biofouling on PEO coated silicone hydrogel contact lenses (2010) Biomaterials, 31 (21), pp. 5510-5519; Svenson, S., Theranostics: are we there yet? (2013) Mol Pharm, 10 (3), pp. 848-856; Jeelani, S., Reddy, R.C., Maheswaran, T., Asokan, G.S., Dany, A., Anand, B., Theranostics: A treasured tailor for tomorrow (2014) J Pharm Bioallied Sci, 6, pp. S6-S8; Mansouri, K., Weinreb, R., Continuous 24-hour intraocular pressure monitoring for glaucoma–time for a paradigm change (2012) Swiss Med Wkly, 142 (1314), p. w13545; Ascaso, F.J., Huerva, V., Noninvasive continuous monitoring of tear glucose using glucose-sensing contact lenses (2016) Optom Vis Sci, 93 (4), pp. 426-434; Badugu, R., Reece, E.A., Lakowicz, J.R., Glucose-sensitive silicone hydrogel contact lens toward tear glucose monitoring (2018) J Biomed Opt, 23 (5), pp. 1-9; Farandos, N.M., Yetisen, A.K., Monteiro, M.J., Lowe, C.R., Yun, S.H., Contact lens sensors in ocular diagnostics (2015) Adv Healthc Mater, 4 (6), pp. 792-810; Keum, D.H., Kim, S.K., Koo, J., Lee, G.H., Jeon, C., Mok, J.W., Wireless smart contact lens for diabetic diagnosis and therapy (2020) Sci Adv, 6 (17), p. eaba3252; Prescott, J.H., Lipka, S., Baldwin, S., Sheppard, N.F., Jr., Maloney, J.M., Coppeta, J., Chronic, programmed polypeptide delivery from an implanted, multireservoir microchip device (2006) Nat Biotechnol, 24 (4), pp. 437-438; Jinabhai, A., O'Donnell, C., Tromans, C., Radhakrishnan, H., Optical quality and visual performance with customised soft contact lenses for keratoconus (2014) Ophthalmic Physiol Opt, 34 (5), pp. 528-539; Jinabhai, A.N., Customised aberration-controlling corrections for keratoconic patients using contact lenses (2020) Clin Exp Optom, 103 (1), pp. 31-43; Shokrollahzadeh, F., Hashemi, H., Jafarzadehpur, E., Mirzajani, A., Khabazkhoob, M., Yekta, A., Corneal aberration changes after rigid gas permeable contact lens wear in keratokonic patients (2016) J Curr Ophthalmol, 28 (4), pp. 194-198; Yang, B., Liang, B., Liu, L., Liao, M., Li, Q., Dai, Y., Contrast sensitivity function after correcting residual wavefront aberrations during RGP lens wear (2014) Optom Vis Sci, 91 (10), pp. 1271-1277; Charman, W.N., Wavefront technology: past, present and future (2005) Cont Lens Anterior Eye, 28 (2), pp. 75-92; Carones, F., Vigo, L., Scandola, E., Wavefront-guided treatment of abnormal eyes using the LADARVision platform (2003) J Refract Surg, 19 (6), pp. S703-S708; Myrowitz, E.H., Chuck, R.S., A comparison of wavefront-optimized and wavefront-guided ablations (2009) Curr Opin Ophthalmol, 20 (4), pp. 247-250; Bakaraju, R.C., Ehrmann, K., Ho, A., Papas, E., Inherent ocular spherical aberration and multifocal contact lens optical performance (2010) Optom Vis Sci, 87 (12), pp. 1009-1022; Lopes-Ferreira, D., Fernandes, P., Queiros, A., Gonzalez-Meijome, J.M., Combined effect of ocular and multifocal contact lens induced aberrations on visual performance: center-distance versus center-near design (2018) Eye Contact Lens, 44, pp. S131-S137; Dietze, H.H., Cox, M.J., On- and off-eye spherical aberration of soft contact lenses and consequent changes of effective lens power (2003) Optom Vis Sci, 80 (2), pp. 126-134; Dietze, H.H., Cox, M.J., Correcting ocular spherical aberration with soft contact lenses (2004) J Opt Soc Am A Opt Image Sci Vis, 21 (4), pp. 473-485; Efron, S., Efron, N., Morgan, P.B., Optical and visual performance of aspheric soft contact lenses (2008) Optom Vis Sci, 85

(3), pp. 201-210; Lindskoog Pettersson, A., Martensson, L., Salkic, J., Unsbo, P., Brautaset, R., Spherical aberration in relation to visual performance in contact lens wear (2011) Cont Lens Anterior Eye, 34 (1), pp. 12-16. , quiz 50-51; Legras, R., Chateau, N., Charman, W.N., Assessment of just-noticeable differences for refractive errors and spherical aberration using visual simulation (2004) Optom Vis Sci, 81 (9), pp. 718-728; Lu, F., Mao, X., Qu, J., Xu, D., He, J.C., Monochromatic wavefront aberrations in the human eye with contact lenses (2003) Optom Vis Sci, 80 (2), pp. 135-141; Rae, S.M., Price, H.C., The effect of soft contact lens wear and time from blink on wavefront aberration measurement variation (2009) Clin Exp Optom, 92 (3), pp. 274-282; Marsack, J.D., Ravikumar, A., Nguyen, C., Ticak, A., Koenig, D.E., Elswick, J.D., Wavefront-guided scleral lens correction in keratoconus (2014) Optom Vis Sci, 91 (10), pp. 1221-1230; Sabesan, R., Johns, L., Tomashevskaya, O., Jacobs, D.S., Rosenthal, P., Yoon, G., Wavefront-guided scleral lens prosthetic device for keratoconus (2013) Optom Vis Sci, 90 (4), pp. 314-323; Thibos, L.N., Cheng, X., Bradley, A., Design principles and limitations of wave-front guided contact lenses (2003) Eye Contact Lens, 29 (1), pp. S167-S170. , discussion S90-1, S92-94; Hastings, G.D., Applegate, R.A., Nguyen, L.C., Kauffman, M.J., Hemmati, R.T., Marsack, J.D., Comparison of wavefront-guided and best conventional scleral lenses after habituation in eyes with corneal ectasia (2019) Optom Vis Sci, 96 (4), pp. 238-247; Kumar, M., Shetty, R., Dutta, D., Rao, H.L., Jayadev, C., Atchison, D.A., Effects of a semi-scleral contact lens on refraction and higher order aberrations (2019) Cont Lens Anterior Eye, 42 (6), pp. 670-674; Vincent, S.J., Fadel, D., Optical considerations for scleral contact lenses: a review (2019) Cont Lens Anterior Eye, 42 (6), pp. 598-613; Fricke, T.R., Tahhan, N., Resnikoff, S., Papas, E., Burnett, A., Ho, S.M., Global prevalence of presbyopia and vision impairment from uncorrected presbyopia: systematic review, meta-analysis, and modelling (2018) Ophthalmology, 125 (10), pp. 1492-1499; Papas, E., Contact lens technology to 2020 and beyond: a review of recent patent literature (2017) Clin Exp Optom, 100 (5), pp. 529-536; Bailey, J., Morgan, P.B., Gleeson, H.F., Jones, J.C., Switchable liquid crystal contact lenses for the correction of presbyopia (2018) Crystals, 8 (1), p. 29; (2009), D.M. Win-Hall. Accommodating or focusable contact lens. Google Patents. US7866815B2. USA;; Waite, S.B., Gupta, A., Schnell, U., Roulet, J.-C., Saint-Ghislain, M., Troller, S., Fluidic module for accommodating soft contact lens (2017) Google Patents. US20170131571A1, , Onefocus Vision Inc USA; Egan, W., Nibauer, L., Fluid filled adjustable contact lenses (2011) Google Patents. US9500884B2, , Adiens Beacon Inc. USA; Biederman, W.J., Yeager, D.J., Otis, B., Pletcher, N., Capacitive gaze tracking for auto-accommodation in a contact lens (2016) Google Patents. US9442310B2, , Google LLC Verily Life Sciences LLC USA; Sako, Y., Hayashi, K., Nakamura, T., Onuma, T., Kon, T., Tange, A., Pair of contact lenses, contact lens and storage medium (2018) Google Patents. US10495904B2, , Sony Corp USA; Flitsch, F.A., Otts, D.B., Pugh, R.B., Riall, J.D., Toner, A., Biocompatibility of biomedical energization elements (2018) Google Patents. US20180159091A1, , Johnson and Johnson Vision Care Inc. USA; Markus, D.T., (2018), M.C. Hayes. Piezoelectric energy harvesting contact lens. Google Patents. US10025118B1. USA;; Pugh, R.B., (2009), D.B. Otts. Ophthalmic lens media insert. Google Patents. US7931832B2. USA: Johnson and Johnson Vision Care Inc;; Honoré, F., Otis, B., Nelson, A., Reader communication with contact lens sensors and display device (2013) Google Patents. US9128305B2, , Google LLC Verily Life Sciences LLC USA; Guillon, M., Maissa, C.A., Multifocal contact lenses manufactured from a responsive polymer gel (2012) Google Patents. US8167427B2, , Optometric Technology Group Ltd USA; Etzkorn, J., Linhardt, J.G., Otis, B., Eye-mountable device to provide automatic accommodation and

method of making same (2015) Google Patents. US9690118B2, , Google LLC Verily Life Sciences LLC USA; Blum, R., Kokonaski, W., Electro-active contact lens system (2004) Google Patents. EP1947501B1, , E Vision LLC Europe; Lindhardt, J.G., Liu, Z., Kennedy, S.B., Flexible conductor for use within a contact lens (2017) Google Patents. US10670887B2, , Verily Life Sciences LLC USA; Burkert, T., Nolan, J.C., Lawrenson, M.J., Anderson, L., Controlling a lens for adjustable vision correction (2015) Google Patents. US10416478B2, , Telefonaktiebolaget LM Ericsson AB USA; Jones, J.C., Gleeson, H.F., Morgan, P.B., A control system for a switchable lens (2017) Google Patents. US20200026099A1, , Dynamic Vision Systems Ltd USA; Lin, Y.R., Hung, C.C., Chiu, H.Y., Chang, B.H., Li, B.R., Cheng, S.J., Noninvasive glucose monitoring with a contact lens and smartphone (2018) Sensors (Basel), 18 (10); Ye, M., Sato, S., Optical properties of liquid crystal lens of any size (2002) Jpn J Appl Phys, 41 (Part 2, No. 5B), pp. L571-L573; De Smet, J., Avci, A., Beernaert, R., Cuypers, D., De Smet, H., Design and wrinkling behavior of a contact lens with an integrated liquid crystal light modulator (2012) J Display Tech, 8 (5), pp. 299-305; Milton, H.E., Gleeson, H.F., Morgan, P.B., Goodby, J.W., Cowling, S., Clamp, J.H., Switchable liquid crystal contact lenses: dynamic vision for the ageing eye (2014) Emerging Liquid Crystal Technologies IX. 9004, International Society for Optics and Photonics, , 90040H; Chamberlain, P., Peixoto-de-Matos, S.C., Logan, N.S., Ngo, C., Jones, D., Young, G., A 3-year randomized clinical trial of misight lenses for myopia control (2019) Optom Vis Sci, 96 (8), pp. 556-567; Sankaridurg, P., Bakaraju, R.C., Naduvilath, T., Chen, X., Weng, R., Tilia, D., Myopia control with novel central and peripheral plus contact lenses and extended depth of focus contact lenses: 2 year results from a randomised clinical trial (2019) Ophthalmic Physiol Opt, 39 (4), pp. 294-307; Walline, J.J., Walker, M.K., Mutti, D.O., Jones-Jordan, L.A., Sinnott, L.T., Giannoni, A.G., Effect of high add power, medium add power, or single-vision contact lenses on myopia progression in children: the blink randomized clinical trial (2020) JAMA, 324 (6), pp. 571-580; Vincent, S.J., Cho, P., Chan, K.Y., Fadel, D., Ghorbani-Mojarrad, N., González-Meijome, J.M., CLEAR - orthokeratology (2021) Contact Lens Anterior Eye, 44 (2). , In press; Richdale, K., Cox, I., Kollbaum, P., Bullimore, M.A., Bakaraju, R.C., Gifford, P., CLEAR - contact lens optics (2021) Contact Lens Anterior Eye, 44 (2). , In press; Wildsoet, C.F., Chia, A., Cho, P., Guggenheim, J.A., Polling, J.R., Read, S., IMI - interventions myopia institute: interventions for controlling myopia onset and progression report (2019) Invest Ophthalmol Vis Sci, 60 (3), pp. M106-M131; Wolffsohn, J.S., Calossi, A., Cho, P., Gifford, K., Jones, L., Jones, D., Global trends in myopia management attitudes and strategies in clinical practice - 2019 update (2020) Cont Lens Anterior Eye, 43 (1), pp. 9-17; Douglass, A., Keller, P.R., He, M., Downie, L.E., Knowledge, perspectives and clinical practices of Australian optometrists in relation to childhood myopia (2020) Clin Exp Optom, 103 (2), pp. 155-166; Wei, X., Brennan, N.A., Chehab, K.A., Roffman, J.H., Wooley, B., Asymmetric lens design and method for preventing and/or slowing myopia progression (2017) Google Patents. AU2017202382B2, , Johnson and Johnson Vision Care Inc Australia; Lau, M.C., Brennan, N., Ophthalmic lens comprising lenslets for preventing and/or slowing myopia progression (2018) Google Patents. US20180275427A1, , Johnson and Johnson Vision Care Inc USA; Brennan, N.A., Chehab, K.A., Cheng, X., Moody, K., Roffman, J.H., Wei, X., Mask lens design and method for preventing and/or slowing myopia progression (2017) Google Patents. US10012848B2, , Johnson and Johnson Vision Care Inc USA; Wu, I.T., Lens with star shaped optical zone to increase defocus image area (2018) Google Patents. US10558061B2, , Brighten Optix Corp USA; Read, S.A., Collins, M.J., Carney, L.G., A review of

astigmatism and its possible genesis (2007) Clin Exp Optom, 90 (1), pp. 5-19; Chen, C., Cheung, S.W., Cho, P., Myopia control using toric orthokeratology (TO-SEE study) (2013) Invest Ophthalmol Vis Sci, 54 (10), pp. 6510-6517; University of Alabama at Birmingham, Customizing myopia control with multifocal toric contact lens. ClinicalTrials.gOv (2020), NIH-US National Library of Medicine; Tan, Q., Ng, A.L., Cheng, G.P., Woo, V.C., Cho, P., Combined atropine with orthokeratology for myopia control: study design and preliminary results (2019) Curr Eye Res, 44 (6), pp. 671-678; Kinoshita, N., Konno, Y., Hamada, N., Kanda, Y., Shimmura-Tomita, M., Kakehashi, A., Additive effects of orthokeratology and atropine 0.01% ophthalmic solution in slowing axial elongation in children with myopia: first year results (2018) Jpn J Ophthalmol, 62 (5), pp. 544-553; Vincent, S.J., Tan, Q., Ng, A.L.K., Cheng, G.P.M., Woo, V.C.P., Cho, P., Higher order aberrations and axial elongation in combined 0.01% atropine with orthokeratology for myopia control (2020) Ophthalmic Physiol Opt, 40 (6), pp. 728-737; Tan, Q., Ng, A.L., Choy, B.N., Cheng, G.P., Woo, V.C., Cho, P., One-year results of 0.01% atropine with orthokeratology (AOK) study: a randomised clinical trial (2020) Ophthalmic Physiol Opt, 40 (5), pp. 557-566; Huang, J., Mutti, D.O., Jones-Jordan, L.A., Walline, J.J., Bifocal & atropine in myopia study: baseline data and methods (2019) Optom Vis Sci, 96 (5), pp. 335-344; Saw, S.M., Matsumura, S., Hoang, Q.V., Prevention and management of myopia and myopic pathology (2019) Invest Ophthalmol Vis Sci, 60 (2), pp. 488-499; Kubota, R.W., Fehr, J.-N., Noronha, G., Haroud, K., Sauvet, J., Bernhard, H., Optical designs of electronic contact lens to decrease myopia progression (2018) Google Patents. WO2020028177A1; Zimmerman, A.B., Lust, K.L., Bullimore, M.A., Visual acuity and contrast sensitivity testing for sports vision (2011) Eye Contact Lens, 37 (3), pp. 153-159; Cervino, A., Gonzalez-Meijome, J.M., Linhares, J.M., Hosking, S.L., Montes-Mico, R., Effect of sport-tinted contact lenses for contrast enhancement on retinal straylight measurements (2008) Ophthalmic Physiol Opt, 28 (2), pp. 151-156; Erickson, G.B., Horn, F.C., Barney, T., Pexton, B., Baird, R.Y., Visual performance with sport-tinted contact lenses in natural sunlight (2009) Optom Vis Sci, 86 (5), pp. 509-516; Horn, F.C., Erickson, G.B., Karben, B., Moore, B., Comparison of low-contrast visual acuity between eye black and maxsight tinted contact lenses (2011) Eye Contact Lens, 37 (3), pp. 147-152; Buch, J.R., Toubouti, Y., Cannon, J., Randomized crossover trial evaluating the impact of senofilcon a photochromic lens on driving performance (2020) Optom Vis Sci, 97 (1), pp. 15-23; Hammond, B.R., Jr., Buch, J., Hacker, L., Cannon, J., Toubouti, Y., Renzi-Hammond, L.M., The effects of light scatter when using a photochromic vs. Non-photochromic contact lens (2020) J Optom, 13 (4), pp. 227-234; Renzi-Hammond, L., Buch, J.R., Cannon, J., Hacker, L., Toubouti, Y., Hammond, B.R., A contra-lateral comparison of the visual effects of a photochromic vs. Non-photochromic contact lens (2020) Cont Lens Anterior Eye, 43 (3), pp. 250-255; Vincent, S.J., The use of contact lens telescopic systems in low vision rehabilitation (2017) Cont Lens Anterior Eye, 40 (3), pp. 131-142; Arianpour, A., Schuster, G.M., Tremblay, E.J., Stamenov, I., Groisman, A., Legerton, J., Wearable telescopic contact lens (2015) Appl Opt, 54 (24), pp. 7195-7204; Byer, A., Magnification limitations of a contact lens telescope (1986) Am J Optom Physiol Opt, 63 (1), pp. 71-75; Tremblay, E.J., Stamenov, I., Beer, R.D., Arianpour, A., Ford, J.E., Switchable telescopic contact lens (2013) Opt Express, 21 (13), pp. 15980-15986; Moshtael, H., Aslam, T., Underwood, I., Dhillon, B., High tech aids low vision: a review of image processing for the visually impaired (2015) Transl Vis Sci Technol, 4 (4), p. 6; Culham, L.E., Chabra, A., Rubin, G.S., Clinical performance of electronic, head-mounted, low-vision devices (2004) Ophthalmic Physiol Opt, 24 (4), pp.

281-290; Hicks, S.L., Wilson, I., Muhammed, L., Worsfold, J., Downes, S.M., Kennard, C., A depth-based head-mounted visual display to aid navigation in partially sighted individuals (2013) PLoS One, 8 (7), p. e67695; Everingham, M.R., Thomas, B.T., Troscianko, T., Head-mounted mobility aid for low vision using scene classification techniques (1998) Int J Virtual Real, 3 (4), pp. 1-10; Zhao, Y., Hu, M., Hashash, S., Azenkot, S., Understanding low vision people's visual perception on commercial augmented reality glasses (2017) Proceedings of the 2017 CHI Conference on Human Factors in Computing Systems, Denver, Colorado, USA: Association for Computing Machinery, pp. 4170-4181; Kinateder, M., Gualtieri, J., Dunn, M.J., Jarosz, W., Yang, X.D., Cooper, E.A., Using an augmented reality device as a distance-based vision aid-promise and limitations (2018) Optom Vis Sci, 95 (9), pp. 727-737; Hwang, A.D., Peli, E., An augmented-reality edge enhancement application for Google glass (2014) Optom Vis Sci, 91 (8), pp. 1021-1030; Sullivan, M., The making of mojo, ar contact lenses that give your eyes superpowers (2020), Fast Company; Dorrier, J., Mojo vision's augmented reality contact lenses kick off a race to ar on your eye (2020), Singularity Hub; Perry, T.S., Augmented reality in a contact lens: it's the real deal (2020), IEEE Spectrum; Martin, P., Mojo vision nanoleds for invisible computing (2020) Spie opto. 11302, , SPIE; Poggio, E.C., Glynn, R.J., Schein, O.D., Seddon, J.M., Shannon, M.J., Scardino, V.A., The incidence of ulcerative keratitis among users of daily-wear and extended-wear soft contact lenses (1989) N Engl J Med, 321 (12), pp. 779-783; Stapleton, F., Keay, L., Edwards, K., Holden, B., The epidemiology of microbial keratitis with silicone hydrogel contact lenses (2013) Eye Contact Lens, 39 (1), pp. 79-85; Lim, C.H., Carnt, N.A., Farook, M., Lam, J., Tan, D.T., Mehta, J.S., Risk factors for contact lens-related microbial keratitis in Singapore (2016) Eye (Lond), 30 (3), pp. 447-455; Stapleton, F., Naduvilath, T., Keay, L., Radford, C., Dart, J., Edwards, K., Risk factors and causative organisms in microbial keratitis in daily disposable contact lens wear (2017) PLoS One, 12 (8), p. e0181343; Lam, D.S., Houang, E., Fan, D.S., Lyon, D., Seal, D., Wong, E., Incidence and risk factors for microbial keratitis in Hong Kong: comparison with Europe and North America (2002) Eye (Lond), 16 (5), pp. 608-618; Stapleton, F., Edwards, K., Keay, L., Naduvilath, T., Dart, J.K., Brian, G., Risk factors for moderate and severe microbial keratitis in daily wear contact lens users (2012) Ophthalmology, 119 (8), pp. 1516-1521; Wu, Y.T., Willcox, M., Zhu, H., Stapleton, F., Contact lens hygiene compliance and lens case contamination: a review (2015) Cont Lens Anterior Eye, 38 (5), pp. 307-316; Szczotka-Flynn, L.B., Pearlman, E., Ghannoum, M., Microbial contamination of contact lenses, lens care solutions, and their accessories: a literature review (2010) Eye Contact Lens, 36 (2), pp. 116-129; (2002), D. Py. Contact lens applicator and cartridge used in connection therewith. In: States U, ed. Google Patents. US6739636B2. USA: Medinstill Development LLC;; (2002), T. Faxe. Package with an applicator for a contact lens. In: States U, ed. Google Patents. US6401915B1. USA;; Greenwood, A.J., (2019), G.S.J.T. Smith. Contact lens packaging. In: USA, ed. Google Patents. US20190046353A1. USA: Johnson and Johnson Vision Care Inc.25; (2006), S.D. Newman. Packaging for disposable soft contact lenses. Google Patents. US7086526B2. USA: Clearlab International Pte Ltd;; Nomachi, M., Sakanishi, K., Ichijima, H., Cavanagh, H.D., Evaluation of diminished microbial contamination in handling of a novel daily disposable flat pack contact lens (2013) Eye Contact Lens, 39 (3), pp. 234-238; Chang, D.C., Grant, G.B., O'Donnell, K., Wannemuehler, K.A., Noble-Wang, J., Rao, C.Y., Multistate outbreak of fusarium keratitis associated with use of a contact lens solution (2006) JAMA, 296 (8), pp. 953-963; Radford, C.F., Lehmann, O.J., Dart, J.K., Acanthamoeba keratitis: multicentre survey in england 1992-6. National

acanthamoeba keratitis study group (1998) Br J Ophthalmol, 82 (12), pp. 1387-1392; Schein, O.D., Glynn, R.J., Poggio, E.C., Seddon, J.M., Kenyon, K.R., The relative risk of ulcerative keratitis among users of daily-wear and extended-wear soft contact lenses. A case-control study. Microbial keratitis study group (1989) N Engl J Med, 321 (12), pp. 773-778; Stapleton, F., Dart, J.K., Minassian, D., Risk factors with contact lens related suppurative keratitis (1993) CLAO J, 19 (4), pp. 204-210; Lakkis, C., Anastasopoulos, F., Terry, C., Borazjani, R., Time course of the development of contact lens case and contact lens contamination (2009) InvestOphthalmol Vis Sci, 50 (13), p. 6352. , (abstract); Dantam, J., McCanna, D.J., Subbaraman, L.N., Papinski, D., Lakkis, C., Mirza, A., Microbial contamination of contact lens storage cases during daily wear use (2016) Optom Vis Sci, 93 (8), pp. 925-932; Newman, S.D., Coon, J.C., McCallum, K.K., Christy, B.V., Cheong, D.C., Oliver, J.M., Contact lens package and storage case, holder, and system and method of making and using (2010) States U, ed. Google patents. US8069979B2, , 1-800 Contacts Inc USA; O'Hara, J., Peterson, C.L., Maraia, M.L., Fehr, R.G., Stone, M.W., (2007), T.H. VanHoozer Jr. Countdown timer contact lens case Google Patents US7628269B2. USA; (2007), B. Song. Contact lnes case with data storing feature. In: States U, ed. Google Patents. US7661383B2. USA;; Josephson, J.E., Myers, R., Sheardown, H., Limited use, self-destructive contact lens case (2011) States U, ed. Google patents. US20110180429A1, , Compliance Case Corp USA; Wiley, L., Bridge, D.R., Wiley, L.A., Odom, J.V., Elliott, T., Olson, J.C., Bacterial biofilm diversity in contact lens-related disease: emerging role of Achromobacter, Stenotrophomonas, and Delftia (2012) Invest Ophthalmol Vis Sci, 53 (7), pp. 3896-3905; Amos, C.F., George, M.D., Clinical and laboratory testing of a silver-impregnated lens case (2006) Cont Lens Anterior Eye, 29 (5), pp. 247-255; Dantam, J., Zhu, H., Stapleton, F., Biocidal efficacy of silver-impregnated contact lens storage cases in vitro (2011) Invest Ophthalmol Vis Sci, 52 (1), pp. 51-57; Datta, A., Willcox, M., Stapleton, F., In vitro antimicrobial efficacy of silver lens cases used with a multipurpose disinfecting solution (2019) Transl Vis Sci Technol, 8 (3), p. 52; Vijay, A.K., Zhu, H., Willcox, M., Ketelson, H., Stapleton, F., Bacterial biofilm in silver-impregnated contact lens cases (2020) Cont Lens Anterior Eye, 43 (4), pp. 408-412; Wu, Y.T., Zhu, H., Willcox, M., Stapleton, F., Impact of cleaning regimens in silver-impregnated and hydrogen peroxide lens cases (2011) Eye Contact Lens, 37 (6), pp. 365-369; Dantam, J., Zhu, H., Willcox, M., Ozkan, J., Naduvilath, T., Thomas, V., In vivo assessment of antimicrobial efficacy of silver-impregnated contact lens storage cases (2012) Invest Ophthalmol Vis Sci, 53 (3), pp. 1641-1648; Datta, A., Willcox, M.D.P., Stapleton, F., In vivo efficacy of silver-impregnated barrel contact lens storage cases (2020) Cont Lens Anterior Eye; Tran, P.L., Huynh, E., Pham, P., Lacky, B., Jarvis, C., Mosley, T., Organoselenium polymer inhibits biofilm formation in polypropylene contact lens case material (2017) Eye Contact Lens, 43 (2), pp. 110-115; Qu, W., Hooymans, J.M., Qiu, J., de-Bont, N., Gelling, O.J., van der Mei, H.C., Nonadhesive, silica nanoparticles-based brush-coated contact lens cases–compromising between ease of cleaning and microbial transmission to contact lenses (2013) J Biomed Mater Res B Appl Biomater, 101 (4), pp. 640-647; Xiao, A., Dhand, C., Leung, C.M., Beuerman, R.W., Ramakrishna, S., Lakshminarayanan, R., Strategies to design antimicrobial contact lenses and contact lens cases (2018) J Mater Chem B, 6 (15), pp. 2171-2186; Mannoor, M.S., Tao, H., Clayton, J.D., Sengupta, A., Kaplan, D.L., Naik, R.R., Graphene-based wireless bacteria detection on tooth enamel (2012) Nat Commun, 3, p. 763; Willcox, M.D., Carnt, N., Diec, J., Naduvilath, T., Evans, V., Stapleton, F., Contact lens case contamination during daily wear of silicone hydrogels (2010)

Optom Vis Sci, 87 (7), pp. 456-464; Govindji-Bhatt, N., Dobson, C., Contact lens case changes color to signal bacterial contamination (2015) Interscience Conference on Antimicrobial Agents and Chemotherapy, San Diego; Verma, M.S., Chen, P.Z., Jones, L., Gu, F.X., “Chemical nose” for the visual identification of emerging ocular pathogens using gold nanostars (2014) Biosens Bioelectron, 61, pp. 386-390; LeBlanc, S.A., Verma, M.S., Jones, L.W., Gu, F., Detecting bacteria colorimetrically on contact lens cases using immobilized gold nanoparticles (2015) Invest Ophth Vis Sci, 56 (7). , e-Abstract 2268, <Go to ISI>://WOS:000362882205269},
correspondence_address1={Jones, L.; Centre for Ocular Research & Education (CORE), Canada; email: lwjones@uwaterloo.ca},
publisher={Elsevier B.V.},
issn={13670484},
coden={CLAEA},
pubmed_id={33775384},
language={English},
abbrev_source_title={Contact Lens Anterior Eye},
document_type={Article},
source={Scopus},
}

@ARTICLE{Jones2021,
author={Jones, S.L. and Hue, W. and Kelly, R.M. and Barnett, R. and Henderson, V. and Sengupta, R.},
title={Determinants of Longitudinal Adherence in Smartphone-Based Self-Tracking for Chronic Health Conditions: Evidence from Axial Spondyloarthritis},
journal={Proceedings of the ACM on Interactive, Mobile, Wearable and Ubiquitous Technologies},
year={2021},
volume={5},
number={1},
doi={10.1145/3448093},
art_number={3448093},
note={cited By 0},
url={https://www.scopus.com/inward/record.uri?eid=2-s2.0-85103675763&doi=10.1145%2f3448093&partnerID=40&md5=846b45138025dafd812bf624f48d4ee1},
affiliation={University of Bath, Bath, United Kingdom; University of Melbourne, Parkville, Melbourne, Australia; Royal National Hospital for Rheumatic Diseases, Bath, United Kingdom},
abstract={The use of interactive mobile and wearable technologies for understanding and managing health conditions is a growing area of interest for patients, health professionals and researchers. Self-tracking technologies such as smartphone apps and wearable devices for measuring symptoms and behaviours generate a wealth of patient-centric data with the potential to support clinical decision making. However, the utility of self-tracking technologies for providing insight into patients' conditions is impacted by poor adherence with data logging. This paper explores factors associated with adherence in smartphone-based tracking, drawing on two studies of patients living with axial spondyloarthritis (axSpA), a chronic rheumatological condition. In Study 1, 184 axSpA patients used the uMotif health tracking smartphone app for a period of up to 593 days. In Study 2, 108 axSpA patients completed a survey about their experience of using self-tracking technologies. We identify six significant correlates of self-tracking adherence, providing insight into the determinants of tracking behaviour. Specifically, our data provides evidence that adherence

correlates with the age of the user, the types of tracking devices that are being used (smartphone OS and physical activity tracker), preferences for types of data to record, the timing of interactions with a self-tracking app, and the reported symptom severity of the user. We discuss how these factors may have implications for those designing, deploying or using mobile and wearable tracking technologies to support monitoring and management of chronic diseases. © 2021 ACM.},
author_keywords={adherence; chronic conditions; engagement; healthcare; personal informatics; rheumatic disease; self-report; self-tracking; smartphones; wearable devices},
keywords={Decision making; Health; Smartphones; User experience, Clinical decision making; Health professionals; Monitoring and management; Patients' conditions; Physical activity; Tracking devices; Tracking technology; Wearable devices, Wearable technology},
references={Defning spinal mobility in ankylosing spondylitis (as) The Bath As Metrology Index, , [n.d.], ([n. d.]); Ancker, J.S., Witteman, H.O., Hafeez, B., Provencher, T., Graaf De, M.Van, Wei, E., You get reminded you're a sick person": Personal data tracking and patients with multiple chronic conditions (2015) Journal of Medical Internet Research, 17 (8), p. e202. , 2015; Ayobi, A., Marshall, P., Cox, A.L., Chen, Y., Quantifying the body and caring for the mind: Self-tracking in multiple sclerosis (2017) Proceedings of the 2017 Chi Conference on Human Factors in Computing Systems, pp. 6889-6901. , https://doi.org/10.1145/3025453.3025869, Association for Computing Machinery, New York, NY, USA; Bardram, J.E., Frost, M., Szántó, K., Faurholt-Jepsen, M., Vinberg, M., Vedel Kessing, L., Designing mobile health technology for bipolar disorder: A field trial of the monarca system (2013) Proceedings of the Sigchi Conference on Human Factors in Computing Systems, pp. 2627-2636. , https://doi.org/10.1145/2470654.2481364, Association for Computing Machinery, New York, NY, USA; Barnett, R., Jones, S., Young, M., Sengupta, R., Association of self-reported pain, fatigue, sleep, exercise, mood and stress in spondyloarthritis: Initial analyses from the project nightingale study (2019) BRITSpA 5th Annual Scientifc Meeting; Bentley, F., Tollmar, K., Stephenson, P., Levy, L., Jones, B., Robertson, S., Price, E., Wilson, J., Health mashups: Presenting statistical patterns between wellbeing data and context in natural language to promote behavior change (2013) Acm Trans. Comput.-Hum. Interact., 20 (5), 27p. , https://doi.org/10.1145/2503823, Nov. 2013); Beukenhorst, A.L., Howells, K., Cook, L., McBeth, J., O'Neill, T.W., Parkes, M.J., Sanders, C., Dixon, W.G., Engagement and participant experiences with consumer smartwatches for health research: Longitudinal observational feasibility study (2020) Jmir Mhealth Uhealth, 8 (1), p. e14368. , https://doi.org/10.2196/14368, 29 Jan 2020; Bielik, P., Tomlein, M., Krátky, P., Mitrík, Š., Barla, M., Bieliková, M., Move2play: An innovative approach to encouraging people to be more physically active (2012) Proceedings of the 2nd Acm Sighit International Health Informatics Symposium (IHI '12), pp. 61-70. , https://doi.org/10.1145/2110363.2110374, Association for Computing Machinery, New York, NY, USA; Boll, S., Heuten, W., Meyer, J., From tracking to personal health (2016) Interactions, 23 (1), pp. 72-75. , https://doi.org/10.1145/2843586, Dec. 2016); Boukhechba, M., Cai, L., Chow, P.I., Fua, K., Gerber, M.S., Teachman, B.A., Barnes, L.E., Contextual analysis to understand compliance with smartphone-based ecological momentary assessment (2018) Proceedings of the 12th Eai International Conference on Pervasive Computing Technologies for Healthcare (PervasiveHealth '18), pp. 232-238. , https://doi.org/10.1145/3240925.3240967, Association for Computing Machinery, New York, NY, USA; Braun, V., Clarke, V., Refecting on refexive thematic

analysis (2019) Qualitative Research in Sport, Exercise and Health, 11 (4), pp. 589-597. , 2019; Brooke, J., Sus: A retrospective (2013) Journal of Usability Studies, 8 (2), pp. 29-40. , 2013; Caldeira, C., Chen, Y., Seniors and self-tracking technology (2019) Perspectives on Human-Computer Interaction Research with Older People, Sergio Sayago (Ed.), pp. 67-79. , https://doi.org/10.1007/978-3-030-06076-3-5, Springer International Publishing, Cham; Chipidza, F.E., Wallwork, R.S., Stern, T.A., Impact of the doctor-patient relationship (2015) The Primary Care Companion for Cns Disorders, 17, p. 5. , 2015; Choe, E.K., Lee, B., Zhu, H., Riche, N.H., Baur, D., Understanding self-refection: How people refect on personal data through visual data exploration (2017) Proceedings of the 11th Eai International Conference on Pervasive Computing Technologies for Healthcare (PervasiveHealth '17), pp. 173-182. , https://doi.org/10.1145/3154862.3154881, Association for Computing Machinery, New York, NY, USA; Choe, E.K., Lee, N.B., Lee, B., Pratt, W., Kientz, J.A., Understanding quantifed-selfers' practices in collecting and exploring personal data (2014) Proceedings of the Sigchi Conference on Human Factors in Computing Systems (CHI '14), pp. 1143-1152. , https://doi.org/10.1145/2556288.2557372, Association for Computing Machinery, New York, NY, USA; Chung, C.-F., Cook, J., Bales, E., Zia, J., Munson, S.A., More than telemonitoring: Health provider use and nonuse of life-log data in irritable bowel syndrome and weight management (2015) Journal of Medical Internet Research, 17 (8), p. e203. , https://doi.org/10.2196/jmir.4364, 21 Aug 2015); Clawson, J., Pater, J.A., Miller, A.D., Mynatt, E.D., Mamykina, L., No longer wearing: Investigating the abandonment of personal health-tracking technologies on craigslist (2015) Proceedings of the 2015 International Conference on Ubiquitous Computing, pp. 647-658. , http://dx.doi.org/10.1145/2750858.2807554, 2015; Consolvo, S., McDonald, D.W., Toscos, T., Chen, M.Y., Froehlich, J., Harrison, B., Klasnja, P., Landay, J.A., Activity sensing in the wild: A field trial of ubift garden (2008) Proceedings of the Sigchi Conference on Human Factors in Computing Systems (CHI '08), pp. 1797-1806. , https://doi.org/10.1145/1357054.1357335, Association for Computing Machinery, New York, NY, USA; Cordeiro, F., Epstein, D.A., Thomaz, E., Bales, E., Jagannathan, A.K., Abowd, G.D., Fogarty, J., Barriers and negative nudges: Exploring challenges in food journaling (2015) Proceedings of the 33rd Annual Acm Conference on Human Factors in Computing Systems (CHI '15), pp. 1159-1162. , https://doi.org/10.1145/2702123.2702155, Association for Computing Machinery, New York, NY, USA; Cuttone, A., Petersen, M.K., Larsen, J.E., Four data visualization heuristics to facilitate refection in personal informatics (2014) Universal Access in Human-Computer Interaction. Design for All and Accessibility Practice, Constantine Stephanidis and Margherita Antona (Eds.), pp. 541-552. , Springer International Publishing, Cham; Davies, T., Jones, S.L., Kelly, R.M., Patient perspectives on self-management technologies for chronic fatigue syndrome (2019) Proceedings of the 2019 Chi Conference on Human Factors in Computing Systems (CHI '19), pp. 1-13. , https://doi.org/10.1145/3290605.3300452, Association for Computing Machinery, New York, NY, USA; Doherty, A., Jackson, D., Hammerla, N., Plötz, T., Olivier, P., Granat, M.H., White, T., Owen, C.G., Large scale population assessment of physical activity using wrist worn accelerometers: The uk biobank study (2017) PloS One, 12 (2), p. 0169649. , 2017; Dohr, A., Engler, J., Bentley, F., Whalley, R., Gluballoon: An unobtrusive and educational way to better understand one's diabetes (2012) Proceedings of the 2012 Acm Conference on Ubiquitous Computing (UbiComp '12), pp. 665-666. , https://doi.org/10.1145/2370216.2370357, Association for Computing Machinery, New York, NY, USA; Drewnowski, A., Monsen, E., Birkett, D., Gunther, S.,

Vendeland, S., Su, J., Marshall, G., Health screening and health promotion programs for the elderly (2003) Disease Management & Health Outcomes, 11 (5), pp. 299-309. , 2003; Druce, K.L., Gibson, D.S., McEleney, K., Meleck, S., James, B., Hellman, B., Dixon, W.G., McBeth, J., P60daily fuctuations in fatigue severity and c-reactive protein in people with rheumatic diseases (2020) Rheumatology, 59 (2). , https://doi.org/10.1093/rheumatology/keaa111.059, 04 2020); Druce, K.L., McBeth, J., Veer Der, S.Van, Selby, D.A., Vidgen, B., Georgatzis, K., Hellman, B., Dixon, W.G., Recruitment and ongoing engagement in a uk smartphone study examining the association between weather and pain: Cohort study (2017) Jmir Mhealth Uhealth, 5 (11), p. e168. , https://doi.org/10.2196/mhealth.8162, 01 Nov 2017); Epstein, D.A., Ping, A., Fogarty, J., Munson, S.A., A lived informatics model of personal informatics (2015) Proceedings of the 2015 Acm International Joint Conference on Pervasive and Ubiquitous Computing (UbiComp '15), pp. 731-742. , https://doi.org/10.1145/2750858.2804250, Association for Computing Machinery, New York, NY, USA; Fadhil, A., (2019) Beyond Technical Motives: Perceived User Behavior in Abandoning Wearable Health & Wellness Trackers, , arXiv preprint, (2019); Frost, M., Doryab, A., Faurholt-Jepsen, M., Vedel Kessing, L., Bardram, J.E., Supporting disease insight through data analysis: Refnements of the monarca self-Assessment system (2013) Proceedings of the 2013 Acm International Joint Conference on Pervasive and Ubiquitous Computing (UbiComp '13), pp. 133-142. , https://doi.org/10.1145/2493432.2493507, Association for Computing Machinery, New York, NY, USA; Gershon, A., Kaufmann, C.N., Torous, J., Depp, C., Ketter, T.A., Electronic ecological momentary assessment (ema) in youth with bipolar disorder: Demographic and clinical predictors of electronic ema adherence (2019) Journal of Psychiatric Research, 116, pp. 14-18. , 2019; Götz, F.M., Stieger, S., Reips, U.-D., Users of the main smartphone operating systems (ios, android) difer only little in personality (2017) PloS One, 12 (5), p. 0176921. , 2017; Hanson, V.L., Infuencing technology adoption by older adults (2010) Interacting with Computers, 22 (6), pp. 502-509. , 2010; Harari, G.M., Wang, W., Müller, S.R., Wang, R., Campbell, A.T., Participants' compliance and experiences with self-tracking using a smartphone sensing app (2017) Proceedings of the 2017 Acm International Joint Conference on Pervasive and Ubiquitous Computing and Proceedings of the 2017 Acm International Symposium on Wearable Computers (UbiComp '17), pp. 57-60. , https://doi.org/10.1145/3123024.3123164, Association for Computing Machinery, New York, NY, USA; Hue, W., Jones, S., Sengupta, R., Exploring the future role of self-tracking data in the rheumatology clinic (2019) Studies in Health Technology and Informatics, 259, pp. 33-38. , 2019; Inder, M.L., Crowe, M.T., Joyce, P.R., Moor, S., Carter, J.D., Luty, S.E., I really don't know whether it is still there": Ambivalent acceptance of a diagnosis of bipolar disorder (2010) Psychiatric Quarterly, 81 (2), pp. 157-165. , 2010; Jaana, M., Pare, G., Use of mobile health technologies for self-tracking purposes among seniors: A comparison to the general adult population in canada (2020) Proceedings of the 53rd Hawaii International Conference on System Sciences; Karkar, R., Schroeder, J., Epstein, D.A., Pina, L.R., Scofeld, J., Fogarty, J., Kientz, J.A., Zia, J., Tummytrials: A feasibility study of using self-experimentation to detect individualized food triggers (2017) Proceedings of the 2017 Chi Conference on Human Factors in Computing Systems, pp. 6850-6863. , https://doi.org/10.1145/3025453.3025480, Association for Computing Machinery, New York, NY, USA; Kelley, C., Lee, B., Wilcox, L., Self-tracking for mental wellness: Understanding expert perspectives and student experiences (2017) Proceedings of the 2017 Chi Conference on Human Factors in Computing Systems

(CHI '17), pp. 629-641. , https://doi.org/10.1145/3025453.3025750, Association for Computing Machinery, New York, NY, USA; Lakshminarayana, R., Wang, D., Burn, D., Ray Chaudhuri, K., Galtrey, C., Valle Guzman, N., Hellman, B., Stamford, J., Using a smartphone-based self-management platform to support medication adherence and clinical consultation in parkinson's disease (2017) Npj Parkinson's Disease, 3 (1), pp. 1-10. , 2017; Richard Landis, J., Koch, G.G., The measurement of observer agreement for categorical data (1977) Biometrics, 33 (1), pp. 159-174. , http://www.jstor.org/stable/2529310, 1977; Lazar, A., Koehler, C., Tanenbaum, T.J., Nguyen, D.H., Why we use and abandon smart devices (2015) Proceedings of the 2015 Acm International Joint Conference on Pervasive and Ubiquitous Computing (UbiComp '15), pp. 635-646. , https://doi.org/10.1145/2750858.2804288, Association for Computing Machinery, New York, NY, USA; Ledger, D., McCafrey, D., Inside wearables: How the science of human behavior change ofers the secret to long-term engagement (2014) Endeavour Partners, 200 (93), p. 1. , 2014; Leventhal, E.A., Leventhal, H., Schaefer, P., Easterling, D., Conservation of energy, uncertainty reduction, and swift utilization of medical care among the elderly (1993) Journal of Gerontology, 48 (2), pp. P78-P86. , 1993; Li, I., Dey, A.K., Forlizzi, J., Understanding my data, myself: Supporting self-refection with ubicomp technologies (2011) Proceedings of the 13th International Conference on Ubiquitous Computing (UbiComp '11), pp. 405-414. , https://doi.org/10.1145/2030112.2030166, Association for Computing Machinery, New York, NY, USA; Marcum, C.S., Age diferences in daily social activities (2013) Research on Aging, 35 (5), pp. 612-640. , 2013; Marin, T.S., Kourbelis, C., Foote, J., Newman, P., Brown, A., Daniel, M., Cofee, N.T., Versace, V.L., Examining adherence to activity monitoring devices to improve physical activity in adults with cardiovascular disease: A systematic review (2019) European Journal of Preventive Cardiology, 26 (4), pp. 382-397. , 2019; Matthews, M., Murnane, E., Snyder, J., Quantifying the changeable self: The role of self-tracking in coming to terms with and managing bipolar disorder (2017) Human-Computer Interaction, 32 (5-6), pp. 413-446. , 2017; McKillop, M., Mamykina, L., Elhadad, N., Designing in the dark: Eliciting self-tracking dimensions for understanding enigmatic disease (2018) Proceedings of the 2018 Chi Conference on Human Factors in Computing Systems (CHI '18), pp. 1-15. , https://doi.org/10.1145/3173574.3174139, Association for Computing Machinery, New York, NY, USA; McMahon, S.K., Lewis, B., Oakes, M., Guan, W., Wyman, J.F., Rothman, A.J., Older adults' experiences using a commercially available monitor to self-track their physical activity (2016) Jmir MHealth and UHealth, 4 (2), p. e35. , 2016; Mentis, H.M., Komlodi, A., Schrader, K., Phipps, M., Gruber-Baldini, A., Yarbrough, K., Shulman, L., Crafting a view of self-tracking data in the clinical visit (2017) Proceedings of the 2017 Chi Conference on Human Factors in Computing Systems (CHI '17), pp. 5800-5812. , https://doi.org/10.1145/3025453.3025589, Association for Computing Machinery, New York, NY, USA; Meyer, J., Wasmann, M., Heuten, W., Ali, A.E., Boll, S.C.J., Identifcation and classifcation of usage patterns in long-term activity tracking (2017) Proceedings of the 2017 Chi Conference on Human Factors in Computing Systems, pp. 667-678. , https://doi.org/10.1145/3025453.3025690, Association for Computing Machinery, New York, NY, USA; Min, J.-K., Doryab, A., Wiese, J., Amini, S., Zimmerman, J., Hong, J.I., Toss 'n' turn: Smartphone as sleep and sleep quality detector (2014) Proceedings of the Sigchi Conference on Human Factors in Computing Systems (CHI '14), pp. 477-486. , https://doi.org/10.1145/2556288.2557220, Association for Computing Machinery, New York, NY, USA; Mishra, S.R., Klasnja, P., MacDufe Woodburn, J., Hekler, E.B., Omberg, L., Kellen, M., Mangravite, L., Supporting coping with

parkinson's disease through self tracking (2019) Proceedings of the 2019 Chi Conference on Human Factors in Computing Systems (CHI '19), pp. 1-16. , https://doi.org/10.1145/3290605.3300337, Association for Computing Machinery, New York, NY, USA; Murnane, E.L., Jiang, X., Kong, A., Park, M., Shi, W., Soohoo, C., Vink, L., Landay, J.A., Designing ambient narrative-based interfaces to refect and motivate physical activity (2020) Proceedings of the 2020 Chi Conference on Human Factors in Computing Systems (CHI '20), pp. 1-14. , https://doi.org/10.1145/3313831.3376478, Association for Computing Machinery, New York, NY, USA; Peck, B.M., Age-related diferences in doctor-patient interaction and patient satisfaction (2011) Current Gerontology and Geriatrics Research, 2011. , 2011; Platt, F.W., Gaspar, D.L., Coulehan, J.L., Fox, L., Adler, A.J., Wayne Weston, W., Smith, R.C., Stewart, M., Tell me about yourself": The patient-centered interview (2001) Annals of Internal Medicine, 134 (11), pp. 1079-1085. , 2001; Pryss, R., Reichert, M., Schlee, W., Spiliopoulou, M., Langguth, B., Probst, T., Diferences between android and ios users of the trackyourtinnitus mobile crowdsensing mhealth platform (2018) 2018 Ieee 31st International Symposium on Computer-based Medical Systems (CBMS), pp. 411-416. , https://doi.org/10.1109/CBMS.2018.00078; Quisel, T., Foschini, L., Zbikowski, S.M., Juusola, J.L., The association between medication adherence for chronic conditions and digital health activity tracking: Retrospective analysis (2019) Journal of Medical Internet Research, 21 (3), p. e11486. , 2019; Rahman, M.M, Nathan, V., Nemati, E., Vatanparvar, K., Ahmed, M., Kuang, J., Towards reliable data collection and annotation to extract pulmonary digital biomarkers using mobile sensors (2019) Proceedings of the 13th Eai International Conference on Pervasive Computing Technologies for Healthcare (PervasiveHealth'19), pp. 179-188. , https://doi.org/10.1145/3329189.3329204, Association for Computing Machinery, New York, NY, USA; Ramsey, A.T., Loebach Wetherell, J., Depp, C., Dixon, D., Lenze, E., Feasibility and acceptability of smartphone assessment in older adults with cognitive and emotional difculties (2016) Journal of Technology in Human Services, 34 (2), pp. 209-223. , 2016; Reade, S., Spencer, K., Sergeant, J.C., Sperrin, M., Schultz, D.M, Ainsworth, J., Lakshminarayana, R., McBeth, J., Cloudy with a chance of pain: Engagement and subsequent attrition of daily data entry in a smartphone pilot study tracking weather, disease severity, and physical activity in patients with rheumatoid arthritis (2017) Jmir MHealth and UHealth, 5 (3), p. e37. , 2017; Rintala, A., Wampers, M., Myin-Germeys, I., Viechtbauer, W., Response compliance and predictors thereof in studies using the experience sampling method (2019) Psychological Assessment, 31 (2), p. 226. , 2019; Rintala, A., Wampers, M., Myin-Germeys, I., Viechtbauer, W., Momentary predictors of compliance in studies using the experience sampling method (2020) Psychiatry Research, p. 112896. , 2020); Sanders, J.P., Loveday, A., Pearson, N., Edwardson, C., Yates, T., Biddle, S.J.H., Esliger, D.W., Devices for self-monitoring sedentary time or physical activity: A scoping review (2016) Journal of Medical Internet Research, 18 (5), p. e90. , 2016; Schroeder, J., Chung, C.-F., Epstein, D.A., Karkar, R., Parsons, A., Murinova, N., Fogarty, J., Munson, S.A., Examining self-tracking by people with migraine: Goals, needs, and opportunities in a chronic health condition (2018) Proceedings of the 2018 Designing Interactive Systems Conference (DIS '18), pp. 135-148. , https://doi.org/10.1145/3196709.3196738, Association for Computing Machinery, New York, NY, USA; Seppen, B.F., Wiegel, J., L'Ami, M.J., Dos Santos Rico, S.D., Catarinella, F.S., Turkstra, F., Boers, M., Bos, W.H., Feasibility of self-monitoring rheumatoid arthritis with a smartphone app: Results of two mixed-methods pilot studies (2020) Jmir Form Res, 4 (9), p. e20165. , https://doi.org/10.2196/20165, 21 Sep 2020; Shaw, H., Ellis, D.A., Kendrick,

L.-R., Ziegler, F., Wiseman, R., Predicting smartphone operating system from personality and individual diferences (2016) Cyberpsychology, Behavior, and Social Networking, 19 (12), pp. 727-732. , 2016; Shih, P.C., Han, K., Poole, E.S., Rosson, M.B., Carroll, J.M., Use and adoption challenges of wearable activity trackers (2015) IConference 2015 Proceedings, , 2015; Siebert, S., Sengupta, R., Tsoukas, A., (2016) Axial Spondyloarthritis, , Oxford University Press; Sieper, J., Poddubnyy, D., Axial spondyloarthritis (2017) The Lancet, 390, pp. 73-84. , 10089 (2017); Sokolovsky, A.W., Mermelstein, R.J., Hedeker, D., Factors predicting compliance to ecological momentary assessment among adolescent smokers (2014) Nicotine & Tobacco Research, 16 (3), pp. 351-358. , 2014; Stawarz, K., Cox, A.L., Blandford, A., Beyond self-tracking and reminders: Designing smartphone apps that support habit formation (2015) Proceedings of the 33rd Annual Acm Conference on Human Factors in Computing Systems, pp. 2653-2662. , https://doi.org/10.1145/2702123.2702230, Association for Computing Machinery, New York, NY, USA; Stawarz, K., Rodríguez, M.D., Cox, A.L., Blandford, A., Understanding the use of contextual cues: Design implications for medication adherence technologies that support remembering (2016) Digital Health, 2, pp. 2055207616678707. , 2016; Strand, V., Singh, J.A., Patient burden of axial spondyloarthritis (2017) Journal of Clinical Rheumatology, 23 (7), p. 383. , 2017; Swendeman, D., Ramanathan, N., Baetscher, L., Medich, M., Schefer, A., Comulada, W.S., Estrin, D., Smartphone self-monitoring to support self-management among people living with hiv: Perceived benefts and theory of change from a mixed-methods, randomized pilot study (2015) Journal of Acquired Immune Defciency Syndromes (1999), 69 (1), p. S80. , 2015; Tang, L.M., Day, M., Engelen, L., Poronnik, P., Bauman, A., Kay, J., Daily & hourly adherence: Towards understanding activity tracker accuracy (2016) Proceedings of the 2016 Chi Conference Extended Abstracts on Human Factors in Computing Systems (CHI Ea '16), pp. 3211-3218. , https://doi.org/10.1145/2851581.2892438, Association for Computing Machinery, New York, NY, USA; Tang, L.M., Meyer, J., Epstein, D.A., Bragg, K., Engelen, L., Bauman, A., Kay, J., Defning adherence: Making sense of physical activity tracker data (2018) Proc. Acm Interact. Mob. Wearable Ubiquitous Technol., 2 (1), 22p. , https://doi.org/10.1145/3191769, March 2018); Thom, D.H., Ribisl, K.M., Stewart, A.L., Luke, D.A., Further validation and reliability testing of the trust in physician scale (1999) Medical Care, pp. 510-517. , and The Stanford Trust Study Physicians, (1999); Torous, J., Friedman, R., Keshavan, M., Smartphone ownership and interest in mobile applications to monitor symptoms of mental health conditions (2014) Jmir MHealth and UHealth, 2 (1), p. e2. , 2014; Tran, T., Nathan-Roberts, D., Design considerations in wearable technology for patients with bipolar disorder (2018) Proceedings of the Human Factors and Ergonomics Society Annual Meeting, 62 (1), pp. 1187-1191. , https://doi.org/10.1177/1541931218621273arXiv:https://doi.org/10.1177/1541931218621273; Trost, S.G., McIver, K.L., Pate, R.R., Conducting accelerometer-based activity assessments in feld-based research (2005) Medicine and Science in Sports and Exercise, 37 (11), pp. S531-S543. , 2005; Turner-McGrievy, G.M., Dunn, C.G., Wilcox, S., Boutté, A.K., Hutto, B., Hoover, A., Muth, E., Defning adherence to mobile dietary self-monitoring and assessing tracking over time: Tracking at least two eating occasions per day is best marker of adherence within two diferent mobile health randomized weight loss interventions (2019) Journal of the Academy of Nutrition and Dietetics, 119 (9), pp. 1516-1524. , 2019; Van Berkel, N., Ferreira, D., Kostakos, V., The experience sampling method on mobile devices (2017) Acm Comput. Surv., 50 (6), 40p. , https://doi.org/10.1145/3123988, Dec. 2017); Wang, E.J., Zhu, J., Jain, M., Lee, T.-J., Saba, E., Nachman, L., Patel, S.N., Seismo: Blood pressure

monitoring using built-in smartphone accelerometer and camera (2018) Proceedings of the 2018 Chi Conference on Human Factors in Computing Systems (CHI '18), pp. 1-9. , https://doi.org/10.1145/3173574.3173999, Association for Computing Machinery, New York, NY, USA; Ward, D.S., Evenson, K.R., Vaughn, A., Rodgers, A.B., Troiano, R.P., Accelerometer use in physical activity: Best practices and research recommendations (2005) Medicine & Science in Sports & Exercise, 37 (11), pp. S582-S588. , 2005; West, P., Van Kleek, M., Giordano, R., Weal, M., Shadbolt, N., Information quality challenges of patient-generated data in clinical practice (2017) Frontiers in Public Health, 5, p. 284. , 2017; Xu, X., Tupy, S., Robertson, S., Miller, A.L., Correll, D., Tivis, R., Nigg, C.R., Successful adherence and retention to daily monitoring of physical activity: Lessons learned (2018) PloS One, 13 (9), p. 0199838. , 2018; Yingling, L.R., Mitchell, V., Ayers, C.R., Peters-Lawrence, M., Wallen, G.R., Brooks, A.T., Troendle, J.F., Henry, J., Adherence with physical activity monitoring wearable devices in a community-based population: Observations from the washington, dc, cardiovascular health and needs assessment Translational Behavioral Medicine, 7, p. 4. , [n.d.], ([n. d.])},
publisher={Association for Computing Machinery},
issn={24749567},
language={English},
abbrev_source_title={Proc. ACM Interact. Mob. Wearable Ubiquitous Technol.},
document_type={Article},
source={Scopus},
}

@ARTICLE{Meegahapola2021,
author={Meegahapola, L. and Ruiz-Correa, S. and Robledo-Valero, V.D.C. and Hernandez-Huerfano, E.E. and Alvarez-Rivera, L. and Chenu-Abente, R. and Gatica-Perez, D.},
title={One More Bite? Inferring Food Consumption Level of College Students Using Smartphone Sensing and Self-Reports},
journal={Proceedings of the ACM on Interactive, Mobile, Wearable and Ubiquitous Technologies},
year={2021},
volume={5},
number={1},
doi={10.1145/3448120},
art_number={3448120},
note={cited By 0},
url={https://www.scopus.com/inward/record.uri?eid=2-s2.0-85103680840&doi=10.1145%2f3448120&partnerID=40&md5=3e40f7d94aacb4a3382d622420c40d40},
affiliation={Idiap Research Institute, Epfl, Switzerland; Ipicyt, Mexico; University of Trento, Italy},
abstract={While the characterization of food consumption level has been extensively studied in nutrition and psychology research, advancements in passive smartphone sensing have not been fully utilized to complement mobile food diaries in characterizing food consumption levels. In this study, a new dataset regarding the holistic food consumption behavior of 84 college students in Mexico was collected using a mobile application combining passive smartphone sensing and self-reports. We show that factors such as sociability and activity types and levels have an association to food consumption levels. Finally, we define and assess a novel ubicomp task, by using machine learning techniques to infer self-perceived food consumption level (eating as usual, overeating, undereating) with an accuracy of 87.81% in a 3-class classification task by

using passive smartphone sensing and self-report data. Furthermore, we show that an accuracy of 83.49% can be achieved for the same classification task by using only smartphone sensing data and time of eating, which is an encouraging step towards building context-aware mobile food diaries and making food diary based apps less tedious for users. © 2021 ACM.},
author_keywords={eating behavior; food consumption; health, well-being; overeating; passive mobile sensing},
keywords={Learning systems; Smartphones; Students, Classification tasks; College students; Context-Aware; Food consumption; Machine learning techniques; Me-xico; Mobile applications; Smartphone sensing, Food supply},
references={(2018) Why Stress Causes People to Overeat, , https://www.health.harvard.edu/staying-healthy/why-stress-causes-people-to-overeat, Retrieved April 29, 2020 from; (2019) Lose It!, , https://www.loseit.com, Retrieved April 28, 2020 from; (2019) MyFitnessPal, , https://www.myftnesspal.com, Retrieved April 28, 2020 from; (2019) Put down That Mobile Phone-It's Making You Fat! Scientists Warn Using A Smartphone at Mealtimes Can Lead to An Expanding Waistline, , https://www.dailymail.co.uk/news/article-6738357/Scientists-warn-using-smartphone-mealtimes-lead-expanding-waistline.html, Retrieved October 27, 2020 from; (2020) Commuter Health, , https://www.apple.com/ios/health, Retrieved May 1, 2020 from; (2020) Google Fit-Coaching You to a Healthier and More Active Life, , https://www.google.com/ft, Retrieved February 12, 2020 from; (2020) A More Personal Health App. For A More Informed You, , https://www.apple.com/ios/health, Retrieved February 12, 2020 from; (2020) Samsung Health App, , https://www.samsung.com/us/support/owners/app/samsung-health, Retrieved February 12, 2020 from; Abdullah, S., Murnane, E.L., Matthews, M., Kay, M., Kientz, J.A., Gay, G., Choudhury, T., Cognitive rhythms: Unobtrusive and continuous sensing of alertness using a mobile phone (2016) Proceedings of the 2016 Acm International Joint Conference on Pervasive and Ubiquitous Computing (UbiComp '16), pp. 178-189. , ACM, New York, NY, USA; Afague, T., Boushey, C., Guerrero, R., Ahmad, Z., Kerr, D., Delp, E., Feasibility and use of the mobile food record for capturing eating occasions among children ages 3-10 years in guam (2015) Nutrients, 7 (6), pp. 4403-4415. , Jun 2015); Alshurafa, N., Jain, J., Alharbi, R., Iakovlev, G., Spring, B., Pfammatter, A., Is more always better? Discovering incentivized mhealth intervention engagement related to health behavior trends (2018) Proc. Acm Interact. Mob. Wearable Ubiquitous Technol., 2 (4), 26p. , Dec. 2018); Andrew, A.H., Borriello, G., Fogarty, J., Simplifying mobile phone food diaries (2013) 2013 7th International Conference on Pervasive Computing Technologies for Healthcare and Workshops., pp. 260-263; Atzmueller, M., Hilgenberg, K., Towards capturing social interactions with sdcf: An extensible framework for mobile sensing and ubiquitous data collection (2013) Proceedings of the 4th International Workshop on Modeling Social Media (MSM '13), 4p. , Association for Computing Machinery New York, NY, USA; Azevedo, M., AraÃºjo, C., Reichert, F., Siqueira, F., Silva, M., Hallal, P., Gender diferences in leisure-time physical activity (2007) International Journal of Public Health, 52 (2), pp. 8-15. , 2007); Azrin, N.H., Kellen, M.J., Brooks, J., Ehle, C., Vinas, V., Relationship between rate of eating and degree of satiation (2008) Child & Family Behavior Therapy, 30 (4), pp. 355-364. , 2008; Bae, S., Ferreira, D., Sufoletto, B., Puyana, J.C., Kurtz, R., Chung, T., Dey, A.K., Detecting drinking episodes in young adults using smartphone-based sensors (2017) Proc. Acm Interact. Mob. Wearable Ubiquitous Technol., 1 (2), 36p. , June 2017); Barlacchi, G., Perentis, C., Mehrotra, A., Musolesi, M., Lepri, B., Are you getting sick? Predicting infuenza-like symptoms using human

mobility behaviors (2017) Epj Data Science, 6 (12), p. 27. , 2017); Bauman, A., Ma, G., Cuevas, F., Omar, Z., Waqanivalu, T., Phongsavan, P., Keke, K., Bhushan, A., Cross-national comparisons of socioeconomic diferences in the prevalence of leisure-time and occupational physical activity, and active commuting in six asia-pacifc countries (2011) Journal of Epidemiology & Community Health, 65 (1), pp. 35-43. , 2011; Bayat, A., Pomplun, M., Tran, D.A., A study on human activity recognition using accelerometer data from smart-phones (2014) Procedia Computer Science, 34, pp. 450-457. , (2014), The 9th International Conference on Future Networks and Communications (FNC'14)/The 11th International Conference on Mobile Systems and Pervasive Computing (MobiSPC'14)/Afliated Workshops; Bedri, A., Li, R., Haynes, M., Kosaraju, R.P., Grover, I., Prioleau, T., Beh, M.Y., Abowd, G., Earbit: Using wearable sensors to detect eating episodes in unconstrained environments (2017) Proc. Acm Interact. Mob. Wearable Ubiquitous Technol., 1 (3), 20p. , Sept. 2017); Bedrick, E.J., (2005) Biserial Correlation, , American Cancer Society; Benesty, J., Chen, J., Huang, Y., Cohen, I., (2009) Pearson Correlation Coefcient, pp. 1-4. , Springer Berlin Heidelberg, Berlin, Heidelberg; Berke, E.M., Choudhury, T., Ali, S., Rabbi, M., Objective measurement of sociability and activity: Mobile sensing in the community (2011) The Annals of Family Medicine, 9 (4), pp. 344-350. , 2011; Biel, J.-I., Martin, N., Labbe, D., Gatica-Perez, D., Bites'n'bits: Inferring eating behavior from contextual mobile data (2018) Proc. Acm Interact. Mob. Wearable Ubiquitous Technol., 1 (4), 33p. , Jan. 2018); Bisogni, C.A., Falk, L.W., Madore, E., Blake, C.E., Jastran, M., Sobal, J., Devine, C.M., Dimensions of everyday eating and drinking episodes (2007) Appetite, 48 (2), pp. 218-231. , 2007; Bogomolov, A., Lepri, B., Ferron, M., Pianesi, F., Pentland, A.S., Daily stress recognition from mobile phone data, weather conditions and individual traits (2014) Proceedings of the 22Nd Acm International Conference on Multimedia (MM '14), pp. 477-486. , ACM, New York, NY, USA; Bongers, P., De Graaf, A., Jansen, A., Emotional' does not even start to cover it: Generalization of overeating in emotional eaters (2016) Appetite, 96, pp. 611-616. , 2016; Bongers, P., Jansen, A., Havermans, R., Roefs, A., Nederkoorn, C., Happy eating. The underestimated role of overeating in a positive mood (2013) Appetite, 67, pp. 74-80. , 2013; Bossard, L., Guillaumin, M., Van Gool, L., Food-101-mining discriminative components with random forests (2014) Computer Vision-ECCV 2014, pp. 446-461. , David Fleet, Tomas Pajdla, Bernt Schiele, and Tinne Tuytelaars (Eds.). Springer International Publishing, Cham; Boukhechba, M., Daros, A.R., Fua, K., Chow, P.I., Teachman, B.A., Barnes, L.E., Demonicsalmon: Monitoring mental health and social interactions of college students using smartphones (2018) Smart Health, 9-10, pp. 192-203. , (2018), CHASE 2018 Special Issue; Bruch, H., Psychological aspects of overeating and obesity (1964) Psychosomatics, 5 (5), pp. 269-274. , 1964; Carroll, E.A., Czerwinski, M., Roseway, A., Kapoor, A., Johns, P., Rowan, K., Schraefel, M.C., Food and mood: Just-in-time support for emotional eating (2013) 2013 Humaine Association Conference on Afective Computing and Intelligent Interaction., pp. 252-257; Chua, J., Touyz, S., Hill, A.J., Negative mood-induced overeating in obese binge eaters: An experimental study (2004) International Journal of Obesity and Related Metabolic Disorders: Journal of the International Association for the Study of Obesity 28, 5, pp. 606-610. , 2004); Cruwys, T., Bevelander, K.E., Hermans, R.C.J., Social modeling of eating: A review of when and why social infuence afects food intake and choice (2015) Appetite, 86, pp. 3-18. , (2015),. Social Infuences on Eating; Dasarathy, B.V., Sensor fusion potential exploitation-innovative architectures and illustrative applications (1997) Proc. Ieee, 85 (1), pp. 24-38. , 1997; De Wit, J.B.F., Marijn Stok, F., Smolenski, D.J., De Ridder,

D.D.T., De Vet, E., Gaspar, T., Johnson, F., Luszczynska, A., Food culture in the home environment: Family meal practices and values can support healthy eating and self-regulation in young people in four european countries (2015) Applied Psychology: Health and Well-Being, 7 (1), pp. 22-40. , 2015; Denning, T., Andrew, A., Chaudhri, R., Hartung, C., Lester, J., Borriello, G., Duncan, G., Balance: Towards a usable pervasive wellness application with accurate activity inference (2009) Proceedings of the 10th Workshop on Mobile Computing Systems and Applications (HotMobile '09), 6p. , Association for Computing Machinery, New York, NY, USA; Epling, W.F., David Pierce, W., Activity-based anorexia: A biobehavioral perspective (1988) International Journal of Eating Disorders, 7 (4), pp. 475-485. , 1988; Ferdous, R., Osmani, V., Mayora, O., Smartphone app usage as a predictor of perceived stress levels at workplace (2015) 2015 9th International Conference on Pervasive Computing Technologies for Healthcare (PervasiveHealth)., pp. 225-228; Field, A.E., Barr Taylor, C., Celio, A., Colditz, G.A., Comparison of self-report to interview assessment of bulimic behaviors among preadolescent and adolescent girls and boys (2004) International Journal of Eating Disorders, 35 (1), pp. 86-92. , 2004; Garcia-Segovia, P., Harrington, R.J., Seo, H.-S., Infuences of table setting and eating location on food acceptance and intake (2015) Food Quality and Preference, 39, pp. 1-7. , 2015; Gong, J., Huang, Y., Chow, P.I., Fua, K., Gerber, M.S., Teachman, B.A., Barnes, L.E., Understanding behavioral dynamics of social anxiety among college students through smartphone sensors (2019) Information Fusion, 49, pp. 57-68. , 2019; Gooding, H.C., Milliren, C., Shay, C.M., Richmond, T.K., Field, A.E., Gillman, M.W., Achieving cardiovascular health in young adulthoodâ€ "which adolescent factors matter? (2016) Journal of Adolescent Health, 58 (1), pp. 119-121. , 2016; Sander, G., Senn, S.J., Rothman, K.J., Carlin, J.B., Charles, P., Goodman, S.N., Altman, D.G., Statistical tests, p values, confdence intervals, and power: A guide to misinterpretations (2016) European Journal of Epidemiology, 31 (4), pp. 337-350. , 2016; Guthold, R., Cowan, M.J., Autenrieth, C.S., Kann, L., Riley, L.M., Physical activity and sedentary behavior among schoolchildren: Aâ 34-country comparison (2010) The Journal of Pediatrics, 157 (1), pp. 43-49. , (2010),.e1; Harari, G., Mueller, S., Stachl, C., Wang, R., Wang, W., Buehner, M., Rentfrow, P., Gosling, S., Sensing sociability: Individual diferences in young adults' conversation, calling, texting, and app use behaviors in daily life (2019) Journal of Personality and Social Psychology, 119 (5). , 2019); Harari, G.M., Gosling, S.D., Wang, R., Chen, F., Chen, Z., Campbell, A.T., Patterns of behavior change in students over an academic term: A preliminary study of activity and sociability behaviors using smartphone sensing methods (2017) Computers in Human Behavior, 67, pp. 129-138. , 2017; Hemminki, S., Nurmi, P., Tarkoma, S., Gravity and linear acceleration estimation on mobile devices (2014) Proceedings of the 11th International Conference on Mobile and Ubiquitous Systems: Computing, Networking and Services (MOBIQUITOUS '14), pp. 50-59. , ICST (Institute for Computer Sciences, Social-Informatics and Telecommunications Engineering), Brussels, BEL; Herman, C.P., Polivy, J., Leone, T., 6-The psychology of overeating (2005) Food, Diet and Obesity, pp. 115-136. , David J. Mela (Ed.). Woodhead Publishing; Herman, C.P., Polivy, J., Dieting as an exercise in behavioral economics (2003) Time and Decision: Economic and Psychological Perspectives on Intertemporal Choice, , 2003; Hetherington, M.M., Cues to overeat: Psychological factors infuencing overconsumption (2007) Proceedings of the Nutrition Society, 66 (1), pp. 113-123. , 2007; Higgs, S., Thomas, J., Social infuences on eating (2016) Current Opinion in Behavioral Sciences, 9, pp. 1-6. , (2016),. Diet, behavior and brain function; Huang, Y.-L., Song, W.O., Schemmel, R.A., Hoerr, S.M., What

do college students eat? Food selection and meal pattern (1994) Nutrition Research, 14 (8), pp. 1143-1153. , 1994; Lee, H.M., Hwang, J.H., A study on lifestyles, dietary habits, nutrition knowledge and dietary behaviors of male university students according to residence type (2007) Korean Journal of Community Nutrition, pp. 381-395. , 2007; Ifand, J.R., Preuss, H.G., Marcus, M.T., Rourke, K.M., Taylor, W.C., Burau, K., Jacobs, W.S., Manso, G., Refned food addiction: A classic substance use disorder (2009) Medical Hypotheses, 72 (5), pp. 518-526. , 2009; Jastran, M.M., Bisogni, C.A., Sobal, J., Blake, C., Devine, C.M., Eating routines. Embedded, value based, modifable, and refective (2009) Appetite, 52 (1), pp. 127-136. , 2009; Jiang, S., Min, W., Liu, L., Luo, Z., Multi-scale multi-view deep feature aggregation for food recognition (2020) Ieee Transactions on Image Processing, 29, pp. 265-276. , 2020); Jung, J., Wellard-Cole, L., Cai, C., Koprinska, I., Yacef, K., Allman-Farinelli, M., Kay, J., Foundations for systematic evaluation and benchmarking of a mobile food logger in a large-scale nutrition study (2020) Proc. Acm Interact. Mob. Wearable Ubiquitous Technol., 4 (2), 25p. , June 2020; Keenan, G.S., Childs, L., Rogers, P.J., Hetherington, M.M., Brunstrom, J.M., The portion size efect: Women demonstrate an awareness of eating more than intended when served larger than normal portions (2018) Appetite, 126, pp. 54-60. , 2018; Kim, T., T test as a parametric statistic (2015) Korean Journal of Anesthesiology, 68 (11), p. 540. , 2015); Klenk, S., Reifegerste, D., Renatus, R., Gender diferences in gratifcations from ftness app use and implications for health interventions (2017) Mobile Media & Communication, 5 (2), pp. 178-193. , 2017; Kromhout, D., Keys, A., Aravanis, C., Buzina, R., Fidanza, F., Giampaoli, S., Jansen, A., Pekkarinen, M., Food consumption patterns in the 1960s in seven countries (1989) The American Journal of Clinical Nutrition, 49 (5), pp. 889-894. , 05 1989); Lakens, D., Lakens d. Calculating and reporting efect sizes to facilitate cumulative science: A practical primer for t-tests and anovas. Front psychol 4: 863 (2013) Frontiers in Psychology, 4 (11), p. 863. , 2013); Lee, D.K., Alternatives to p value: Confdence interval and efect size (2016) Korean Journal of Anesthesiology; Lee, I.-M., Shiroma, E.J., Using accelerometers to measure physical activity in large-scale epidemiological studies: Issues and challenges (2014) British Journal of Sports Medicine, 48 (3), pp. 197-201. , 2014; Liang, Y., Li, J., (2017) Computer Vision-Based Food Calorie Estimation: Dataset, Method, and Experiment, , ArXiv abs/1705.07632 (2017); LiKamWa, R., Liu, Y., Lane, N.D., Zhong, L., Moodscope: Building a mood sensor from smartphone usage patterns (2013) Proceeding of the 11th Annual International Conference on Mobile Systems, Applications, and Services (MobiSys '13), pp. 389-402. , ACM, New York, NY, USA; Lim, S.L., Bentley, P., Kanakam, N., Ishikawa, F., Honiden, S., Investigating country diferences in mobile app user behavior and challenges for software engineering (2014) Ieee Transactions on Software Engineering, 41 (9). , 2014); Long, E.V., Vartanian, L.R., Herman, C.P., Polivy, J., What does it mean to overeat? (2020) Eating Behaviors, 37, p. 101390. , 2020); Lu, H., Frauendorfer, D., Rabbi, M., Mast, M.S., Chittaranjan, G.T., Campbell, A.T., Gatica-Perez, D., Choudhury, T., Stresssense: Detecting stress in unconstrained acoustic environments using smartphones (2012) Proceedings of the 2012 Acm Conference on Ubiquitous Computing (UbiComp '12), pp. 351-360. , ACM, New York, NY, USA; Madan, A., Moturu, S.T., Lazer, D., Pentland, A.S., Social sensing: Obesity, unhealthy eating and exercise in face-to-face networks (2010) Wireless Health 2010 (WH '10), pp. 104-110. , Association for Computing Machinery, New York, NY, USA; McCrory, M.A., Fuss, P.J., Hays, N.P., Vinken, A.G., Greenberg, A.S., Roberts, S.B., Overeating in america: Association between restaurant food consumption and body fatness in healthy adult men and women ages 19 to 80 (1999) Obesity Research, 7 (6), pp.

564-571. , 1999; Meegahapola, L., Gatica-Perez, D., Smartphone sensing for the well-being of young adults: A review (2021) Ieee Access, 9, pp. 3374-3399. , 2021); Mela, D., (2005) Food, Diet, and Obesity, , https://www.elsevier.com/books/food-diet-And-obesity/mela/978-1-85573-958-1, Elsevier; Min, W., Jiang, S., Liu, L., Rui, Y., Jain, R., A survey on food computing (2019) Acm Comput. Surv., 52 (5), 36p. , Sept. 2019); Min, W., Liu, L., Luo, Z., Jiang, S., Ingredient-guided cascaded multi-Attention network for food recognition (2019) Proceedings of the 27th Acm International Conference on Multimedia (MM '19), pp. 1331-1339. , Association for Computing Machinery, New York, NY, USA; Mirtchouk, M., McGuire, D., Deierlein, A., Kleinberg, S., Automated estimation of food type from body-worn audio and motion sensors in free-living environments (2019) Proceedings of Machine Learning Research, 106 (8), pp. 641-662. , 2019); Mobius, (2019) 11 Surprising Mobile Health Statistics, , https://www.mobius.md/blog/2019/03/11-mobile-health-statistics, Retrieved April 28, 2020 from; Morshed, M.B., Kulkarni, S.S., Li, R., Saha, K., Roper, L.G., Nachman, L., Lu, H., Abowd, G.D., A real-time eating detection system for capturing eating moments and triggering ecological momentary assessments to obtain further context: System development and validation study (2020) Jmir Mhealth Uhealth, 4 (3), p. e20625. , 18 dec 2020; Moturu, S.T., Khayal, I., Aharony, N., Pan, W., Pentland, A.S., Using social sensing to understand the links between sleep, mood, and sociability (2011) Proceedings of Ieee International Conference on Social Computing; Pan, S.J., Yang, Q., A survey on transfer learning (2010) Ieee Transactions on Knowledge and Data Engineering, 22 (10), pp. 1345-1359. , 2010; Parcel, G.S., Muraskin, L.D., Endert, C.M., Community education: Study group report (1988) Journal of Adolescent Health Care, 9 (6), pp. S41-S45. , 1988; Patel, K.A., Schlundt, D.G., Impact of moods and social context on eating behavior (2001) Appetite, 36 (2), pp. 111-118. , 2001; Pekka, Kouvo, A., Linked or divided by the web?: Internet use and sociability in four european countries (2007) Information Communication & Society, 10 (2), pp. 219-241. , 2007; Pentina, I., Zhang, L., Bata, H., Chen, Y., Exploring privacy paradox in information-sensitive mobile app adoption: A cross-cultural comparison (2016) Computers in Human Behavior, 65, pp. 409-419. , 2016; Phan, T.-T., Gatica-Perez, D., Healthy fondue dinner: Analysis and inference of food and drink consumption patterns on instagram (2017) Proceedings of the 16th International Conference on Mobile and Ubiquitous Multimedia (MUM '17), pp. 327-338. , Association for Computing Machinery, New York, NY, USA; Polivy, J., Herman, C.P., Overeating in restrained and unrestrained eaters (2020) Frontiers in Nutrition, 7, p. 30. , 2020); Polivy, J., Herman, C.P., Deo, R., Getting a bigger slice of the pie. Efects on eating and emotion in restrained and unrestrained eaters (2010) Appetite, 55 (3), pp. 426-430. , 2010; Quick, V.M., Byrd-Bredbenner, C., Disturbed eating behaviours and associated psychographic characteristics of college students (2013) Journal of Human Nutrition and Dietetics, 26 (S1), pp. 53-63. , 2013; Rachuri, K.K., Musolesi, M., Mascolo, C., Rentfrow, P.J., Longworth, C., Aucinas, A., Emotionsense: A mobile phones based adaptive platform for experimental social psychology research (2010) Proceedings of the 12th Acm International Conference on Ubiquitous Computing (UbiComp '10), pp. 281-290. , ACM, New York, NY, USA; Rahman, T., Czerwinski, M., Gilad-Bachrach, R., Johns, P., Predicting about-to-eat" moments for just-in-time eating intervention (2016) Proceedings of the 6th International Conference on Digital Health Conference (DH '16), pp. 141-150. , Association for Computing Machinery, New York, NY, USA; Ramirez-Esparza, N., Mehl, M.R., Alvarez-Bermudez, J., Pennebaker, J.W., Are mexicans more or less sociable than americans? Insights from a naturalistic observation study (2009) Journal of Research in

Personality, 43 (1), pp. 1-7. , 2009; Rice, M.E., Harris, G.T., Comparing efect sizes in follow-up studies: Roc area cohen's d and r (2005) Law and Human Behavior, 29 (5), pp. 615-620. , 01 Oct 2005); Richards, A., Kattelmann, K.K., Ren, C., Motivating 18-to 24-year-olds to increase their fruit and vegetable consumption (2006) Journal of the American Dietetic Association, 106 (9), pp. 1405-1411. , 2006; Robinson, E., Aveyard, P., Daley, A., Jolly, K., Lewis, A., Lycett, D., Higgs, S., Eating attentively: A systematic review and meta-Analysis of the efect of food intake memory and awareness on eating (2013) The American Journal of Clinical Nutrition, 97 (4), pp. 728-742. , 02 2013); Robinson, E., Harris, E., Thomas, J., Aveyard, P., Higgs, S., Reducing high calorie snack food in young adults: A role for social norms and health based messages (2013) The International Journal of Behavioral Nutrition and Physical Activity, 10 (6), p. 73. , 2013); Ronel, N., Libman, G., Eating disorders and recovery: Lessons from overeaters anonymous (2003) Clinical Social Work Journal, 31 (6), pp. 155-171. , 2003); Ruddock, H.K., Hardman, C.A., Guilty pleasures: The efect of perceived overeating on food addiction attributions and snack choice (2018) Appetite, 121, pp. 9-17. , 2018; Russell, A., Hart, C., Robinson, C., Olsen, S., Children's sociable and aggressive behaviour with peers: A comparison of the us and australia, and contributions of temperament and parenting styles (2003) International Journal of Behavioral Development, 27 (1), pp. 74-86. , 2003; Sahoo, D., Hao, W., Ke, S., Xiongwei, W., Le, H., Achananuparp, P., Lim, E.-P., Hoi, S.C.H., Foodai: Food image recognition via deep learning for smart food logging (2019) Proceedings of the 25th Acm Sigkdd International Conference on Knowledge Discovery & Data Mining (KDD '19), pp. 2260-2268. , ACM, New York, NY, USA; Salazar Vazquez, B.Y., Salazar Vazquez, M.A., Lopez Gutierrez, G., Acosta Rosales, K., Cabrales, P., Vadillo-Ortega, F., Intaglietta, M., Schmid-Schoenbein, G.W., Control of overweight and obesity in childhood through education in meal time habits. The 'good manners for a healthy future' programme (2016) Pediatric Obesity, 11 (6), pp. 484-490. , 2016; Salvy, S.-J., De La Haye, K., Bowker, J.C., Hermans, R.C.J., Infuence of peers and friends on children's and adolescents' eating and activity behaviors (2012) Physiology & Behavior, 106 (3), pp. 369-378. , (2012),. Proceedings from the 2011 meeting of the Society for the Study of Ingestive Behavior (SSIB); Santani, D., Do, T., Labhart, F., Landolt, S., Kuntsche, E., Gatica-Perez, D., Drinksense: Characterizing youth drinking behavior using smartphones (2018) Ieee Transactions on Mobile Computing, 17 (10), pp. 2279-2292. , Oct 2018); Schwarz, N., (2012) Retrospective and Concurrent Self-Reports: The Rationale for Real-Time Data Capture; Selye, H., (1976) Stress without Distress, pp. 137-146. , Springer US, Boston, MA; Servia-Rodríguez, S., Rachuri, K.K., Mascolo, C., Rentfrow, P.J., Lathia, N., Sandstrom, G.M., Mobile sensing at the service of mental well-being: A large-scale longitudinal study (2017) Proceedings of the 26th International Conference on World Wide Web (WWW '17). International World Wide Web Conferences Steering Committee, Republic and Canton of Geneva, Che, pp. 103-112; Seto, E., Hua, J., Wu, L., Shia, V., Eom, S., Wang, M., Li, Y., Models of individual dietary behavior based on smartphone data: The infuence of routine, physical activity, emotion, and food environment (2016) Plos One, 11 (4), pp. 1-16. , 04 2016); Sharma, R., Pavlovic, V.I., Huang, T.S., Toward multimodal human-computer interface (1998) Proc. Ieee, 86 (5), pp. 853-869. , 1998; Shema, A., Acuna, D.E., Show me your app usage and i will tell who your close friends are: Predicting user's context from simple cellphone activity (2017) Proceedings of the 2017 Chi Conference Extended Abstracts on Human Factors in Computing Systems (CHI Ea '17), pp. 2929-2935. , Association for Computing Machinery, New York, NY, USA; Sheppard-Sawyer, C., McNally, R., Fischer, J., Film-induced sadness as a trigger for disinhibited

eating (2000) The International Journal of Eating Disorders, 28 (10), pp. 215-220. , 2000); Singh, M., Mood, food, and obesity (2014) Frontiers in Psychology, 5 (9), p. 925. , 2014); Stiglic, N., Viner, R.M., Efects of screentime on the health and well-being of children and adolescents: A systematic review of reviews (2019) Bmj Open, 9, p. 1. , 2019; Stinson, E.J., Votruba, S.B., Venti, C.A., Perez, M., Krakof, J., Gluck, M.E., Food insecurity is associated with maladaptive eating behaviors and objectively measured overeating (2018) Obesity (Silver Spring, Md.), 26, pp. 1841-1848. , 2018; Taylor, R., Interpretation of the correlation coefcient: A basic review (1990) Journal of Diagnostic Medical Sonography, 6 (1), pp. 35-39. , 1990; Teo, E., Goh, D., Vijayakumar, K.M., Liu, J.C.J., To message or browse? Exploring the impact of phone use patterns on male adolescents' consumption of palatable snacks (2018) Frontiers in Psychology, 8, p. 2298. , 2018; Heatherton, T.F., Baumeister, R.F., Binge eating as escape from self-Awareness (1991) Psychol Bull., 9, pp. 86-108. , 1991); Thomas, J.G., Doshi, S., Crosby, R.D., Lowe, M.R., Ecological momentary assessment of obesogenic eating behavior: Combining person-specifc and environmental predictors (2011) Obesity, 19 (8), pp. 1574-1579. , 2011; Thomaz, E., Essa, I., Abowd, G.D., A practical approach for recognizing eating moments with wrist-mounted inertial sensing (2015) Proceedings of the 2015 Acm International Joint Conference on Pervasive and Ubiquitous Computing (UbiComp '15), pp. 1029-1040. , Association for Computing Machinery, New York, NY, USA; Trost, S.G., State of the art reviews: Measurement of physical activity in children and adolescents (2007) American Journal of Lifestyle Medicine, 1 (4), pp. 299-314. , 2007; Tulu, B., Ruiz, C., Allard, J., Acheson, J., Busch, A., Roskusku, A., Heeringa, G., Pagoto, S., (2017) SlipBuddy: A Mobile Health Intervention to Prevent Overeating., 1. , 2017); Turner, P., Lefevre, C., Instagram use is linked to increased symptoms of orthorexia nervosa (2017) Eating and Weight Disorders-Studies on Anorexia, Bulimia and Obesity, 22 (3). , 2017); Van Strien, T., Peter Herman, C., Verheijden, M.W., Eating style, overeating, and overweight in a representative dutch sample. Does external eating play a role? (2009) Appetite, 52 (2), pp. 380-387. , 2009; Van Strien, T., Peter Herman, C., Verheijden, M.W., Eating style, overeating and weight gain. A prospective 2-year follow-up study in a representative dutch sample (2012) Appetite, 59 (3), pp. 782-789. , 2012; Vandewater, E.A., Shim, M., Caplovitz, A.G., Linking obesity and activity level with children's television and video game use (2004) Journal of Adolescence, 27 (1), pp. 71-85. , (2004),. Video Games and Public Health; Vartanian, L., Reily, N., Spanos, S., Herman, C., Polivy, J., Self-reported overeating and attributions for food intake (2017) Psychology & Health, 32 (1), pp. 1-10. , 2017); Vartanian, L.R., Reily, N.M., Spanos, S., McGuirk, L.C., Herman, C.P., Polivy, J., Hunger, taste, and normative cues in predictions about food intake (2017) Appetite, 116, pp. 511-517. , 2017; Vereecken, C.A., Todd, J., Roberts, C., Mulvihill, C., Maes, L., Television viewing behaviour and associations with food habits in diferent countries (2006) Public Health Nutrition, 9 (2), pp. 244-250. , 2006; Vickerstaf, V., Omar, R., Ambler, G., Methods to adjust for multiple comparisons in the analysis and sample size calculation of randomised controlled trials with multiple primary outcomes (2019) Bmc Medical Research Methodology, 19 (6). , 2019); Vu, T., Lin, F., Alshurafa, N., Xu, W., Wearable food intake monitoring technologies: A comprehensive review (2017) Computers, 6 (1), p. 4. , Jan 2017); Walker, M., Thornton, L., De Choudhury, M., Teevan, J., Bulik, C.M., Levinson, C.A., Zerwas, S., Facebook use and disordered eating in college-Aged women (2015) Journal of Adolescent Health, 57 (2), pp. 157-163. , 2015; Wammes, B., Breedveld, B., Kremers, S., Brug, J., The 'balance intervention' for promoting caloric compensatory behaviours in response to

overeating: A formative evaluation (2006) Health Education Research, 21 (4), pp. 527-537. , 04 2006); Wammes, B., French, S., Brug, J., What young dutch adults say they do to keep from gaining weight: Self-reported prevalence of overeating, compensatory behaviours and specifc weight control behaviours (2007) Public Health Nutrition, 10 (8), pp. 790-798. , 2007; Wang, R., Wang, W., DaSilva, A., Huckins, J.F., Kelley, W.M., Heatherton, T.F., Campbell, A.T., Tracking depression dynamics in college students using mobile phone and wearable sensing (2018) Proc. Acm Interact. Mob. Wearable Ubiquitous Technol., 2 (1), 26p. , March 2018); Wardle, J., Haase, A., Steptoe, A., Nillapun, M., Jonwutiwes, K., Bellisle, F., Gender diferences in food choice: The contribution of health beliefs and dieting (2004) Annals of Behavioral Medicine: a Publication of the Society of Behavioral Medicine, 27 (5), pp. 107-116. , 2004); Westerterp, K.R., 4-Physical activity and obesity (2005) Food, Diet and Obesity, pp. 76-85. , David J. Mela (Ed.). Woodhead Publishing; (2020) Obesity and Overweight, , https://www.who.int/news-room/fact-sheets/detail/obesity-And-overweight, World Health Organization (WHO), Retrieved April 28, 2020 from; Williamson, D.A., Gleaves, D.H., Lawson, O.J., Biased perception of overeating in bulimia nervosa and compulsive binge eaters (1991) Journal of Psychopathology and Behavioral Assessment, 13, pp. 257-268. , 1991; Yan, T., Chu, D., Ganesan, D., Kansal, A., Liu, J., Fast app launching for mobile devices using predictive user context (2012) Proceedings of the 10th International Conference on Mobile Systems, Applications, and Services (MobiSys '12), pp. 113-126. , Association for Computing Machinery, New York, NY, USA; Yatani, K., (2016) Efect Sizes and Power Analysis in Hci, pp. 87-110. , Springer International Publishing, Cham; Yue, Y., Lan, T., Yeh, A.G.O., Li, Q.-Q., Zooming into individuals to understand the collective: A review of trajectory-based travel behaviour studies (2014) Travel Behaviour and Society, 1 (2), pp. 69-78. , 2014; Yun, T.C., Ahmad, S.R., Quee, D.K.S., Dietary habits and lifestyle practices among university students in universiti brunei darussalam (2018) The Malaysian Journal of Medical Sciences: Mjms, 25, pp. 56-66. , 2018; Zeni, M., Zaihrayeu, I., Giunchiglia, F., Multi-device activity logging (2014) Proceedings of the 2014 Acm International Joint Conference on Pervasive and Ubiquitous Computing: Adjunct Publication (UbiComp '14 Adjunct), pp. 299-302. , Association for Computing Machinery, New York, NY, USA; Zhang, S., Stogin, W., Alshurafa, N., I sense overeating: Motif-based machine learning framework to detect overeating using wrist-worn sensing (2018) Information Fusion, 41, pp. 37-47. , 2018; Zhu, K., Zhang, X., Xiang, B., Zhang, L., Exploiting user context and network information for mobile application usage prediction (2015) Proceedings of the 7th International Workshop on Hot Topics in Planet-Scale MObile Computing and Online Social NeTworking (HOTPOST '15), pp. 25-30. , Association for Computing Machinery, New York, NY, USA},
publisher={Association for Computing Machinery},
issn={24749567},
language={English},
abbrev_source_title={Proc. ACM Interact. Mob. Wearable Ubiquitous Technol.},
document_type={Article},
source={Scopus},
}

@ARTICLE{Gan2021,
author={Gan, Y. and Wang, T. and Javaheri, A. and Momeni-Ortner, E. and Dehghani, M. and Hosseinzadeh, M. and Rawassizadeh, R.},
title={11 Years with Wearables: Qantitative Analysis of Social Media, Academia,

News Agencies, and Lead User Community from 2009-2020 on Wearable Technologies},
journal={Proceedings of the ACM on Interactive, Mobile, Wearable and Ubiquitous Technologies},
year={2021},
volume={5},
number={1},
doi={10.1145/3448096},
art_number={3448096},
note={cited By 0},
url={https://www.scopus.com/inward/record.uri?eid=2-s2.0-85103671883&doi=10.1145%2f3448096&partnerID=40&md5=3ac0fe2c7e76c360740d0c6f8e632621},
affiliation={Boston University, United States; Independent Scientist, France; University of Vienna, Austria; EMentalist, EMentalist Labs, Austria; University College Cork, Ireland; Iran University of Medical Sciences, Iran; Duy Tan University, Viet Nam},
abstract={The role of wearable technology in our daily lives is rapidly growing and many users are cumulatively becoming dependent on it. To provide insight into the future of wearable technologies and various community attitudes towards them, we implemented an in-depth quantitative investigation of opinions from academic texts (DBLP and PubMed), social media (Twitter), news media (Google News and Bing News), and entrepreneurship communities (Kickstarter and Indiegogo) over a 10-year period. Our results indicate that unlike academia, the news media, entrepreneurship communities, and social media all hold overall positive attitudes towards wearable technologies. Secondly, there are diverse perspectives towards various wearable products across different platforms. Specifically, "XR"technologies received the most attention, while "Exoskeleton"ignited the most heated debates. Thirdly, we discovered that the lifetime of a hyped wearable technology lasts approximately three years. Furthermore, the news media and entrepreneurship community's attitudes towards wearable technologies did not have a strong impact on public opinion. Finally, among all types of wearable technologies, "fashion design"and "healthcare"products were the most enlightening for the market. © 2021 ACM.},
author_keywords={market analysis; sentiment analysis; text mining; wearable technology},
keywords={Exoskeleton (Robotics); Product design; Social aspects; Social networking (online), Daily lives; Fashion design; Lead users; News agencies; Positive attitude; Public opinions; Quantitative investigation; Social media, Wearable technology},
references={Alfna, I., Sigmawaty, D., Nurhidayati, F., Nizar Hidayanto, A., Utilizing hashtags for sentiment analysis of tweets in the political domain (2017) Proceedings of the 9th International Conference on Machine Learning and Computing., pp. 43-47; Amft, O., Lukowicz, P., From backpacks to smartphones: Past, present, and future of wearable computers (2009) Ieee Pervasive Computing, 8 (3), pp. 8-13. , 2009; Anderson, E.F., McLoughlin, L., Liarokapis, F., Peters, C., Petridis, P., De Freitas, S., Developing serious games for cultural heritage: A state-of-the-Art review (2010) Virtual Reality, 14 (4), pp. 255-275. , 2010; Arvanitis, T.N., Williams, D.D., Knight, J.F., Baber, C., Gargalakos, M., Sotiriou, S., Bogner, F.X., A human factors study of technology acceptance of a prototype mobile augmented reality system for science education (2011) Advanced Science Letters, 4 (11-12), pp. 3342-3352. , 2011; Balazs, J.A., Velásquez, J.D., Opinion mining and information fusion: A survey (2016) Information Fusion, 27, pp. 95-110. , 2016; Bartlett-Bragg, A., Wearable technologies: Shaping the future of learning (2014) Training & Development, 41

(3), p. 13. , 2014; Baziotis, C., Pelekis, N., Doulkeridis, C., Datastories at semeval-2017 task 4: Deep lstm with attention for message-level and topic-based sentiment analysis (2017) Proceedings of the 11th International Workshop on Semantic Evaluation (SemEval-2017)., pp. 747-754; Kumar Bhagat, K., Liou, W.-K., Chang, C.-Y., A cost-efective interactive 3d virtual reality system applied to military live fring training (2016) Virtual Reality, 20 (2), pp. 127-140. , 2016; Nick, B., Why google glass broke (2015) The New York Times, , https://www.nytimes.com/2015/02/05/style/why-google-glass-broke.html; Blain-Moraes, S., Schaf, R., Gruis, K.L., Huggins, J.E., Wren, P.A., Barriers to and mediators of brain-computer interface user acceptance: Focus group fndings (2012) Ergonomics, 55 (5), pp. 516-525. , 2012; Blei, D.M., Ng, A.Y., Jordan, M.I., Latent dirichlet allocation (2003) Journal of Machine Learning Research, 3, pp. 993-1022. , Jan (2003); Bovitz, G.L., Druckman, J.N., Lupia, A., When can a news organization lead public opinion?-ideology versus market forces in decisions to make news (2002) Public Choice, 113 (1-2), pp. 127-155. , 2002; Bryson, D., Smart clothes and wearable technology for the health and well-being market (2009) Smart Clothes and Wearable Technology, pp. 335-345. , Elsevier; Cain, P., Unlock the full potential of wearables with organic tfts (2015) Information Display, 31 (1), pp. 22-26. , 2015; Canhoto, A.I., Arp, S., Exploring the factors that support adoption and sustained use of health and ftness wearables (2017) Journal of Marketing Management, 33 (1-2), pp. 32-60. , 2017; Casselman, J., Onopa, N., Khansa, L., Wearable healthcare: Lessons from the past and a peek into the future (2017) Telematics and Informatics, 34 (7), pp. 1011-1023. , 2017; Castillo, A.G., Telan, S.M., Palaoag, T., Cloud-based data mining framework: A model to improve maternal healthcare (2018) Proceedings of the 2nd International Conference on Cryptography, Security and Privacy., pp. 21-28; Cawley, J.C., Homce, G.T., Protecting miners from electrical arcing injury (2007) 2007 Ieee Industry Applications Annual Meeting, pp. 1373-1380; Chang, J., Gerrish, S., Wang, C., Boyd-Graber, J.L., Blei, D.M., Reading tea leaves: How humans interpret topic models (2009) Advances in Neural Information Processing Systems., pp. 288-296; Cheung, M.L., Chau, K.Y., Huen, M., Lam, S., Tse, G., Yan Ho, K., Flint, S.W., Lee, K.Y., Examining consumers' adoption of wearable healthcare technology: The role of health attributes (2019) International Journal of Environmental Research and Public Health, 16 (13), p. 2257. , 2019; Choi, J., Kim, S., Is the smartwatch an it product or a fashion product? A study on factors afecting the intention to use smartwatches (2016) Computers in Human Behavior, 63, pp. 777-786. , 2016; Cohen, J., A coefcient of agreement for nominal scales (1960) Educational and Psychological Measurement, 20 (1), pp. 37-46. , 1960; Coorevits, L., Coenen, T., The rise and fall of wearable ftness trackers (2016) Academy of Management; Cox, D.F., Rich, S.U., Perceived risk and consumer decision-making-the case of telephone shopping (1964) Journal of Marketing Research, 1 (4), pp. 32-39. , 1964; Dehghani, M., Dangelico, R.M., Smart wearable technologies: State of the art and evolution over time through patent analysis and clustering (2018) International Journal of Product Development, 22 (4), pp. 293-313. , 2018; Dehghani, M., Kim, K.J., Past and present research on wearable technologies: Bibliometric and cluster analyses of published research from 2000 to 2016 (2019) International Journal of Innovation and Technology Management, 16 (1), p. 1950007. , 2019; Milad, D., Joon, K.K., Maria, D.R., Will smartwatches last? Factors contributing to intention to keep using smart wearable technology (2018) Telematics and Informatics, 35 (2), pp. 480-490. , 2018; Milad, D., Leung, T.K., Maria, Z.I., Reza, R., What are the perceived experiences of health ftness trackers for the elderly? A qualitative post-Adoption study (2020) International Journal of Technology Marketing, 14 (2), pp. 181-198. ,

2020); Deng, W., Hu, D., Xu, S., Liu, X., Zhao, J., Chen, Q., Liu, J., Ma, L., The efcacy of virtual reality exposure therapy for ptsd symptoms: A systematic review and meta-Analysis (2019) Journal of Afective Disorders, , 2019; Devlin, J., Chang, M.-W., Lee, K., Toutanova, K., (2018) Bert: Pre-Training of Deep Bidirectional Transformers for Language Understanding, , arXiv preprint, (2018); Duarte, D., Nuno, F., Silva, C.J.P., Vitallogger: An adaptable wearable physiology and body-Area ambiance data logger for mobile applications (2017) 2017 Ieee 14th International Conference on Wearable and Implantable Body Sensor Networks (BSN)., pp. 71-74; Duarte, D., João, P.S.C., Wearable health devices-vital sign monitoring, systems and technologies (2018) Sensors, 18 (8), p. 2414. , 2018; Mark, D., Michael, N., Katie, R., Lai Samuel, K., Jo, S., Helen, B., Harriet, K., Ridgers Nicola, D., Using the technology acceptance model to explore adolescents' perspectives on combining technologies for physical activity promotion within an intervention: Usability study (2020) Journal of Medical Internet Research, 22 (3), p. e15552. , 2020); Edelson, D.P., Sasson, C., Chan, P.S., Atkins, D.L., Aziz, K., Becker, L.B., Berg, R.A., Cheng, A., Interim guidance for basic and advanced life support in adults, children, and neonates with suspected or confrmed covid-19: From the emergency cardiovascular care committee and get with the guidelines®-resuscitation adult and pediatric task forces of the american heart association in collaboration with the american academy of pediatrics, american association for respiratory care, american college of emergency physicians, the society of critical care anesthesiologists, and american society of anesthesiologists: Supporting organizations: American association of critical care nurses and national ems physicians (2020) Circulation, , 2020); Fisch, M.J., Chung, A.E., Accordino, M.K., Using technology to improve cancer care: Social media, wearables, and electronic health records (2016) American Society of Clinical Oncology Educational Book, 36, pp. 200-208. , 2016; Fleiss, J.L., Measuring nominal scale agreement among many raters (1971) Psychological Bulletin, 76 (5), p. 378. , 1971; Folgieri, R., Zampolini, R., Bci promises in emotional involvement in music and games (2015) Computers in Entertainment (CIE), 12 (1), pp. 1-10. , 2015; Frangoul, A., Spending on wearables predicted to hit $52 billion next year as prices fall (2020) Cnbc, , https://www.cnbc.com/2019/10/30/spending-on-wearables-predicted-to-hit-52-billion-next-year.html; Gafni, R., Framework for quality metrics in mobile-wireless information systems (2008) Interdisciplinary Journal of Information, Knowledge & Management, 3. , 2008; Ghose, A., Sokol, D.D., Unlocking platform technology to combat health pandemics (2020) Yale Journal on Regulation, , https://www.whitecase.com/sites/default/fles/2020-03/unlocking-platform-technology-to-combat-health-pandemics.pdf, 2020); Gimhae, G.-N., Six human factors to acceptability of wearable computers (2013) International Journal of Multimedia and Ubiquitous Engineering, 8, p. 3. , 2013; Grifths, T.L., Steyvers, M., Finding scientifc topics (2004) Proceedings of the National Academy of Sciences, 101, pp. 5228-5235. , 2004; Hadaya, J., Schumm, M., Livingston, E.H., Testing individuals for coronavirus disease 2019 (covid-19) (2020) Jama (Published Online), , 2020); Hermsen, S., Moons, J., Kerkhof, P., Wiekens, C., De Groot, M., Determinants for sustained use of an activity tracker: Observational study (2017) Jmir MHealth and UHealth, 5 (10), p. e164. , 2017; Homayounfar, M., Malekijoo, A., Visuri, A., Dobbins, C., Peltonen, E., Pinsky, E., Teymourian, K., Rawassizadeh, R., Understanding smartwatch battery utilization in the wild (2020) Sensors, 20 (13), p. 3784. , 2020); Horton, R.L., The structure of perceived risk: Some further progress (1976) Journal of the Academy of Marketing Science, 4 (4), pp. 694-706. , 1976; Hwang, C., Chung, T.-L., Sanders, E.A., Attitudes and purchase intentions for smart clothing:

Examining us consumers' functional, expressive, and aesthetic needs for solar-powered clothing (2016) Clothing and Textiles Research Journal, 34 (3), pp. 207-222. , 2016; Iskender, E., Bati, G.B., Comparing turkish universities entrepreneurship and innovativeness index's rankings with sentiment analysis results on social media (2015) Procedia-Social and Behavioral Sciences, 195, pp. 1543-1552. , 2015; Javaheri, A., Moghadamnejad, N., Keshavarz, H., Javaheri, E., Dobbins, C., Momeni-Ortner, E., Rawassizadeh, R., Public vs media opinion on robots and their evolution over recent years (2020) Ccf Transactions on Pervasive Computing and Interaction, pp. 1-17. , 2020); Jiang, H., Chen, X., Zhang, S., Zhang, X., Kong, W., Zhang, T., Software for wearable devices: Challenges and opportunities (2015) 2015 Ieee 39th Annual Computer Software and Applications Conference, 3, pp. 592-597; Kalantari, M., Rauschnabel, P., Exploring the early adopters of augmented reality smart glasses: The case of microsoft hololens (2018) Augmented Reality and Virtual Reality, pp. 229-245. , Springer; Kenwright, B., Virtual reality: Ethical challenges and dangers [opinion] (2018) Ieee Technology and Society Magazine, 37 (4), pp. 20-25. , 2018; Keshavarz, H., Abadeh, M.S., Rawassizadeh, R., (2020) SEFR: A Fast Linear-Time Classifer for Ultra-Low Power Devices, , arXiv preprint, (2020); Khakurel, J., Melkas, H., Porras, J., Tapping into the wearable device revolution in the work environment: A systematic review (2018) Information Technology & People, , 2018; Khakurel, J., Porras, J., Melkas, H., Fu, B., A comprehensive framework of usability issues related to the wearable devices (2020) Convergence of Ict and Smart Devices for Emerging Applications, pp. 21-66. , Springer; Kim, E.H.-J., Jeong, Y.K., Kim, Y., Kang, K.Y., Song, M., Topic-based content and sentiment analysis of ebola virus on twitter and in the news (2016) Journal of Information Science, 42 (6), pp. 763-781. , 2016; Kim, K.J., Shin, D.-H., An acceptance model for smart watches (2015) Internet Research, 25 (4), pp. 527-541. , 2015; Ko, E., Sung, H., Yun, H., Comparative analysis of purchase intentions toward smart clothing between korean and us consumers (2009) Clothing and Textiles Research Journal, 27 (4), pp. 259-273. , 2009; Landis, J.R., Koch, G.G., The measurement of observer agreement for categorical data (1977) Biometrics, pp. 159-174. , 1977; Lee, J.-Y., Bakri, I., Kim, J.-H., Son, S.-Y., Tochihara, Y., The impact of frefghter personal protective equipment and treadmill protocol on maximal oxygen uptake (2013) Journal of Occupational and Environmental Hygiene, 10 (7), pp. 397-407. , 2013; Lennox, R.J., Veríssimo, D., Twardek, W.M., Davis, C.R., Jaric, I., Sentiment analysis as a measure of conservation culture in scientifc literature (2020) Conservation Biology, 34 (2), pp. 462-471. , 2020); Lunney, A., Cunningham, N.R., Eastin, M.S., Wearable ftness technology: A structural investigation into acceptance and perceived ftness outcomes (2016) Computers in Human Behavior, 65, pp. 114-120. , 2016; Mak, J.N., Wolpaw, J.R., Clinical applications of brain-computer interfaces: Current state and future prospects (2009) Ieee Reviews in Biomedical Engineering, 2, pp. 187-199. , 2009; Malu, M., Findlater, L., Toward accessible health and ftness tracking for people with mobility impairments (2016) Proceedings of the 10th Eai International Conference on Pervasive Computing Technologies for Healthcare., pp. 170-177; Mercer, K., Giangregorio, L., Schneider, E., Chilana, P., Li, M., Grindrod, K., Acceptance of commercially available wearable activity trackers among adults aged over 50 and with chronic illness: A mixed-methods evaluation (2016) Jmir MHealth and UHealth, 4 (1), p. e7. , 2016; Mercier, D., Bhardwaj, A., Dengel, A., Ahmed, S., (2019) Senticite: An Approach for Publication Sentiment Analysis, , arXiv preprint, (2019); Millard, I., Glaser, H., Salvadores, M., Shadbolt, N., Consuming multiple linked data sources: Challenges and experiences (2010) First International Workshop on Consuming Linked Data (COLD2010), , 2010; Mimno, D.,

Wallach, H.M., Talley, E., Leenders, M., McCallum, A., Optimizing semantic coherence in topic models (2011) Proceedings of the Conference on Empirical Methods in Natural Language Processing, pp. 262-272. , Association for Computational Linguistics; Moore, M.M., Real-world applications for brain-computer interface technology (2003) Ieee Transactions on Neural Systems and Rehabilitation Engineering, 11 (2), pp. 162-165. , 2003; Nasir, S., Yurder, Y., Consumers' and physicians' perceptions about high tech wearable health products (2015) Procedia-Social and Behavioral Sciences, 195, pp. 1261-1267. , 2015; Nigel, N., Connie, S., Jen, K.H., Michael, L., The acceptance, challenges, and future applications of wearable technology and virtual reality to support people with autism spectrum disorders (2017) Recent Advances in Technologies for Inclusive Well-Being, pp. 221-241. , Springer; Newman, D., Bonilla, E.V., Buntine, W., Improving topic coherence with regularized topic models (2011) Advances in Neural Information Processing Systems., pp. 496-504; Oliver, N., Letouzé, E., Sterly, H., Delataille, S., De Nadai, M., Lepri, B., Lambiotte, R., Colizza, V., (2020) Mobile Phone Data and COVID-19: Missing An Opportunity?, , arXiv preprint, 2020); Olsson, T., Kärkkäinen, T., Lagerstam, E., Ventä-Olkkonen, L., User evaluation of mobile augmented reality scenarios (2012) Journal of Ambient Intelligence and Smart Environments, 4 (1), pp. 29-47. , 2012; Page, T., A forecast of the adoption of wearable technology (2015) International Journal of Technology Difusion (IJTD), 6 (2), pp. 12-29. , 2015; Paradiso, J.A., Landay, J.A., Guest editors' introduction: Cross-reality environments (2009) Ieee Pervasive Computing, 8 (3), pp. 14-15. , 2009; Pevnick, J.M., Birkeland, K., Zimmer, R., Elad, Y., Kedan, I., Wearable technology for cardiology: An update and framework for the future (2018) Trends in Cardiovascular Medicine, 28 (2), pp. 144-150. , 2018; Pinto, A., Oliveira, H.G., Alves, A.O., Comparing the performance of diferent nlp toolkits in formal and social media text (2016) 5th Symposium on Languages, Applications and Technologies (SLATE'16); Podgorski, D., Majchrzycka, K., Dabrowska, A., Gralewicz, G., Okrasa, M., Towards a conceptual framework of osh risk management in smart working environments based on smart ppe, ambient intelligence and the internet of things technologies (2017) International Journal of Occupational Safety and Ergonomics, 23 (1), pp. 1-20. , 2017; Price Solla De, D.J, Networks of scientifc papers (1965) Science, pp. 510-515. , 1965; Rauschnabel, P.A., Hein, D.W.E., He, J., Ro, Y.K., Rawashdeh, S., Krulikowski, B., Fashion or technology? A fashnology perspective on the perception and adoption of augmented reality smart glasses (2016) I-Com, 15 (2), pp. 179-194. , 2016; Rawassizadeh, R., Keshavarz, H., Pazzani, M., Ghost imputation: Accurately reconstructing missing data of the of period (2020) Ieee Transactions on Knowledge and Data Engineering (Online Frst), , 2020); Rawassizadeh, R., Momeni, E., Dobbins, C., Gharibshah, J., Pazzani, M., Scalable daily human behavioral pattern mining from multivariate temporal data (2016) Ieee Transactions on Knowledge and Data Engineering, 28 (11), pp. 3098-3112. , 2016; Rawassizadeh, R., Momeni, E., Dobbins, C., Mirza-Babaei, P., Rahnamoun, R., Lesson learned from collecting quantifed self information via mobile and wearable devices (2015) Journal of Sensor and Actuator Networks, 4 (4), pp. 315-335. , 2015; Rawassizadeh, R., Pierson, T.J., Peterson, R., Kotz, D., Nocloud: Exploring network disconnection through on-device data analysis (2018) Ieee Pervasive Computing, 17 (1), pp. 64-74. , 2018; Rawassizadeh, R., Price, B.A., Petre, M., Wearables: Has the age of smartwatches fnally arrived? (2014) Commun. Acm, 58 (1), pp. 45-47. , 2014; Rawassizadeh, R., Tomitsch, M., Nourizadeh, M., Momeni, E., Peery, A., Ulanova, L., Pazzani, M., Energy-efcient integration of continuous context sensing and prediction into smartwatches (2015) Sensors, 15 (9), pp. 22616-22645. , 2015; Reza, R., Chelsea, D.,

Mohammad, A., Michael, P., Indexing multivariate mobile data through spatio-temporal event detection and clustering (2019) Sensors, 19 (3), p. 448. , 2019; Siimon, R., Why google glass failed: A marketing lesson (2015) Forbes Magazine, , https://www.forbes.com/sites/siimonreynolds/2015/02/05/why-google-glass-failed; Röder, M., Both, A., Hinneburg, A., Exploring the space of topic coherence measures (2015) Proceedings of the Eighth Acm International Conference on Web Search and Data Mining., pp. 399-408; Rogers, E.M., (2010) Difusion of Innovations, , Simon and Schuster; Vlad, S., Google glass gets a second chance in factories, where its likely to remain (2017) The Verge, , https://www.theverge.com/2017/7/18/15988258/google-glass-2-enterprise-edition-factories; Scott, D.M., (2009) The New Rules of Marketing and PR: How to Use Social Media, Blogs, News Releases, Online Video, and Viral Marketing to Reach Buyers Directly, , John Wiley & Sons; Simeone, A.L., Khamis, M., Esteves, A., Daiber, F., Kljun, M., Copic Pucihar, K., Isokoski, P., Gugenheimer, J., International workshop on cross-reality (xr) interaction (2020) Companion Proceedings of the 2020 Conference on Interactive Surfaces and Spaces., pp. 111-114; Soosaraei, M., Khasseh, A.A., Fakhar, M., Hezarjaribi, H.Z., A decade bibliometric analysis of global research on leishmaniasis in web of science database (2018) Annals of Medicine and Surgery, 26, pp. 30-37. , 2018; Sorbello, R., Tramonte, S., Giardina, M.E., La Bella, V., Spataro, R., Allison, B., Guger, C., Chella, A., A human-humanoid interaction through the use of bci for locked-in als patients using neuro-biological feedback fusion (2017) Ieee Transactions on Neural Systems and Rehabilitation Engineering, 26 (2), pp. 487-497. , 2017; Stock, B., Dos Santos Ferreira, T.P., Ernst, C.-P.H., Does perceived health risk infuence smartglasses usage? (2016) The Drivers of Wearable Device Usage, pp. 13-23. , Springer; Thorp, E.O., The invention of the frst wearable computer (1998) Digest of Papers. Second International Symposium on Wearable Computers (Cat. No. 98EX215)., pp. 4-8; Tom Dieck, M.C., Jung, T., Han, D.-I., Mapping requirements for the wearable smart glasses augmented reality museum application (2016) Journal of Hospitality and Tourism Technology, , 2016; Vladimir, T., Trenton, S., Sebastian, K., Applying universal design principles to themes for wearables (2015) International Conference on Universal Access in Human-Computer Interaction., pp. 550-560; Turney, P.D., Thumbs up or thumbs down?: Semantic orientation applied to unsupervised classifcation of reviews (2002) Proceedings of the 40th Annual Meeting on Association for Computational Linguistics., pp. 417-424; Van Heek, J., Schaar, A.K., Trevisan, B., Bosowski, P., Ziefe, M., User requirements for wearable smart textiles: Does the usage context matter (medical vs. Sports)? (2014) Proceedings of the 8th International Conference on Pervasive Computing Technologies for Healthcare., pp. 205-209; Vanderveld, A., Pandey, A., Han, A., Parekh, R., An engagement-based customer lifetime value system for e-commerce (2016) Proceedings of the 22nd Acm Sigkdd International Conference on Knowledge Discovery and Data Mining., pp. 293-302; Vaswani, A., Shazeer, N., Parmar, N., Uszkoreit, J., Jones, L., Gomez, A.N., Kaiser, L., Polosukhin, I., Attention is all you need (2017) Advances in Neural Information Processing Systems., pp. 5998-6008; Vincent, A., Armstrong, M., (2010) Predicting Break-Points in Trading Strategies with Twitter, , Available at SSRN 1685150 (2010); Von Bloh, J., Broekel, T., Özgun, B., Sternberg, R., New (s) data for entrepreneurship research? An innovative approach to use big data on media coverage (2019) Small Business Economics, pp. 1-22. , 2019; Wallach, H.M., Mimno, D.M., McCallum, A., Rethinking lda: Why priors matter (2009) Advances in Neural Information Processing Systems., pp. 1973-1981; Wang, X., White, L., Chen, X., Gao, Y., Li, H., Luo, Y., An empirical study of wearable technology acceptance in healthcare

(2015) Industrial Management & Data Systems, 115 (9), pp. 1704-1723. , 2015; Wolf, T., Debut, L., Sanh, V., Chaumond, J., Delangue, C., Moi, A., Cistac, P., Funtowicz, M., (2019) Transformers: State-of-the-Art Natural Language Processing, , arXiv preprint, (2019); Wu, L.-H., Wu, L.-C., Chang, S.-C., Exploring consumers' intention to accept smartwatch (2016) Computers in Human Behavior, 64, pp. 383-392. , 2016; Xi, Y., Chen, G., Li, B., Tang, Y., Topic evolution analysis based on cluster topic model (2016) Journal of Advanced Computational Intelligence and Intelligent Informatics, 20 (1), pp. 66-75. , 2016; Kumar Yadav, S., Bharadwaj, B., Pal, S., (2012) Mining Education Data to Predict Student's Retention: A Comparative Study, , arXiv preprint, (2012); Yang, H., Yu, J., Zo, H., Choi, M., User acceptance of wearable devices: An extended perspective of perceived value (2016) Telematics and Informatics, 33 (2), pp. 256-269. , 2016; Yang, R., Shin, E., Newman, M.W., Ackerman, M.S., When ftness trackers don't'ft' end-user difculties in the assessment of personal tracking device accuracy (2015) Proceedings of the 2015 Acm International Joint Conference on Pervasive and Ubiquitous Computing., pp. 623-634; Yun, J., Patel, S.N., Reynolds, M.S., Abowd, G.D., Design and performance of an optimal inertial power harvester for human-powered devices (2010) Ieee Transactions on Mobile Computing, 10 (5), pp. 669-683. , 2010; Zhang, M., Luo, M., Nie, R., Zhang, Y., Technical attributes, health attribute, consumer attributes and their roles in adoption intention of healthcare wearable technology (2017) International Journal of Medical Informatics, 108, pp. 97-109. , 2017; Zhao, D., Lucas, J., Virtual reality simulation for construction safety promotion (2015) International Journal of Injury Control and Safety Promotion, 22 (1), pp. 57-67. , 2015},
publisher={Association for Computing Machinery},
issn={24749567},
language={English},
abbrev_source_title={Proc. ACM Interact. Mob. Wearable Ubiquitous Technol.},
document_type={Article},
source={Scopus},
}

@ARTICLE{Pogosova2021,
author={Pogosova, N. and Yufereva, Y. and Sokolova, O. and Yusubova, A. and Suvorov, A. and Saner, H.},
title={Telemedicine intervention to improve long-term risk factor control and body composition in persons with high cardiovascular risk: Results from a randomized trial},
journal={Global Heart},
year={2021},
volume={16},
number={1},
doi={10.5334/GH.825},
art_number={825},
note={cited By 0},
url={https://www.scopus.com/inward/record.uri?eid=2-s2.0-85104164919&doi=10.5334%2fGH.825&partnerID=40&md5=c7d612be1373c8c56e78cb564d0f5885},
affiliation={Federal State Institution, Natl. Medical Research Center of Cardiology of the Ministry of Healthcare of the Russian Federation, Moscow, Russian Federation; Children's Diagnostics and Treatment Center after N.A. Semashko, Moscow, Russian Federation; City Hospital #4, Moscow, Russian Federation; Institute for Social and Preventive Medicine, University of Bern, Bern, Switzerland},

abstract={Background: Telehealth strategies are increasingly used to support people at high cardiovascular risk long-term, but is it unclear if these interventions are effective at improving cardiovascular risk. Objective: To evaluate the effects of a telemedicine technology-based program on risk factor control and body composition in patients at high cardiovascular risk. Methods: This is a population based randomized controlled trial. 100 patients at high and very high cardiovascular risk were randomly assigned to a telemedicine technology-based program consisting of: Comprehensive counseling on risk factors delivered by a physician; biweekly remote support via phone delivered by a trained nurse during the first three months after enrollment; and a control group receiving routine care with individual single-session counseling on patients' current risk factors without further support. The follow-up period was 1 year. Results: Mean age of participants was 59.9 ± 4.5 years, 80% were women. Weight (-0.582; p < 0.001), waist circumference (-0.429; p = 0.01), body mass index (-0.216; p < 0.001) diastolic blood pressure (-0.881; p = 0.04), total cholesterol (-0.149; p = 0.01) and LDL cholesterol (-0.123; p = 0.003) were lower in the intervention group compared to the control group after 12-month. Body fat mass was also lower (-0.352; p = 0.01) and lean mass was higher (0.92; p = 0.03) in the intervention group. Anxiety scores (-2.5; p < 0.002) and depression scores (-2.6; p < 0.001) were also lower in the intervention group. Conclusions: Among older people at high cardiovascular risk, the addition of telehealth strate-gies using remote support by phone calls over a period of 3 month resulted in small but significant improvements of cardiovascular risk factors, body composition, anxiety, and depression which are maintained long-term. Such telehealth strategies may offer an advantage over standard institution-based interventions. © 2021 The Author(s).},
author_keywords={Anxiety; Depression; High cardiovascular risk; Obesity; Physical inactivity; Preventive counseling; Risk factors; Smoking; Telemedicine technology; Unhealthy nutrition},
references={Roth, GA, Johnson, C, Abajobir, A, Global, regional and national burden of cardiovascular disease for 10 causes, 1990 to 2015 (2017) J Am Coll Cardiol, 70 (1), pp. 1-25. , https://doi.org/10.1016/j.jacc.2017.04.052; Maruthur, NM, Wang, N-Y, Appel, LJ., Lifestyle interventions reduce coronary heart disease risk: Results from the PREMIER Trial (2009) Circulation, 119 (15), pp. 2026-2031. , https://doi.org/10.1161/CIRCULATIONAHA.108.809491; Estruch, R, Ros, E, Salas-Salvado, J, Primary prevention of cardiovascular disease with aMediterranean diet supplemented with extra-virgin olive oil or nuts (2018) The New England Journal of Medicine, 378 (25), p. e34. , https://doi.org/10.1056/nejmoa18003894; Cardiovascular prevention. National guidelines (2011) Cardiovascular Therapy and Prevention, 10 (6), pp. 3-64. , Russian Scientific Society of Cardiology; Piepoli, MF, Hoes, AW, Agewall, S, 2016 European Guidelines on cardiovascular disease prevention in clinical practice: The Sixth Joint Task Force of the European Society of Cardiology and Other Societies on Cardiovascular Disease Prevention in Clinical Practice (constituted by representatives of 10 societies and by invited experts) developed with the special contribution of the European Association for Cardiovascular Prevention & Rehabilitation (EACPR) (2016) Eur Heart J, 37 (29), pp. 2315-2381. , https://doi.org/10.1093/eurheartj/ehw106; Vijver, S, Oti, S, Addo, J, de Graft-Alkins, A, Agyemang, Ch, Review of community-based interventions for prevention of cardiovascular diseases in low- and middle-income countries (2012) Ethnicity & Health, 17 (6). , https://doi.org/10.1080/13557858.2012.754409; Beishuizen, CR, Stephan, BC, van Gool, WA, Web-Based interventions targeting cardiovascular risk factors in middle-aged and older people: A systematic review and meta-analysis (2016) J

Med Internet Res, 18 (3), p. e55. , https://doi.org/10.2196/jmir.5218; Piette, JD, List, J, Rana, GK, Townsend, W, Striplin, D, Heisler, M., Mobile health devices as tools for worldwide cardiovascular risk reduction and disease management (2015) Circulation, 132 (21), pp. 2012-2027. , https://doi.org/10.1161/CIRCULATIONAHA.114.008723; Patel, A, Praveen, D, Maharani, A, Association of multifaceted mobile technology-enabled primary care intervention with cardiovascular disease risk management in rural Indonesia (2019) JAMA Cardiol, 4 (10), pp. 987-986. , https://doi.org/10.1001/jamacardio.2019.2974, [Epub ahead of print]; Krishnamurthi, R, Hale, L, Barker-Collo, S, Mobile technology for primary stroke prevention (2018) Stroke, , https://doi.org/10.1161/STROKEAHA.118.023058, [Epub ahead of print]; Hallberg, I, Ranerup, A, Kjellgren, K., Supporting the self-management of hypertension: Patients' experiences of using a mobile phone-based system (2016) J Hum Hypertens, 30 (2), pp. 141-146. , https://doi.org/10.1038/jhh.2015.37; Lu, X, Yang, H, Xia, X, Interactive mobile health intervention and blood pressure management in adults (2019) Hypertension, 74 (3), pp. 697-704. , https://doi.org/10.1161/HYPERTENSIONAHA.119.13273, Epub 2019 Jul 22; Lin, JS, O'Connor, EA, Evans, CV, Behavioral counseling to promote a healthy lifestyle for cardiovascular disease prevention in persons with cardiovascular risk factors: Updated systematic review for the U.S (2014), https://doi.org/10.1001/jama.2018.7777, Preventive Services Task Force 5. Aug. Report No.: 13-05179-EF-1; LeBlanc, ES, Patnode, CD, Webber, EM, Behavioral and pharmacotherapy weight loss interventions to prevent obesity-related morbidity and mortality in adults: An updated systematic review for the U.S (2018), Preventive Services Task Force. Sep. Report No.: 18-05239-EF-1; Conroy, RM, Pyörälä, K, Fitzgerald, AP, Estimation of ten-year risk of fatal cardiovascular disease in Europe: The SCORE project (2003) Eur Heart J, 24 (11), pp. 987-1003. , https://doi.org/10.1016/S0195-668X(03)00114-3; Perk, J, De Backer, G, Gohlke, H, European Guidelines on cardiovascular disease prevention in clinical practice (version 2012). The Fifth Joint Task Force of the European Society of Cardiology and other societies on cardiovascular disease prevention in clinical practice (constituted by representatives of nine societies and by invited experts) (2012) Eur Heart J, 33 (13), pp. 1635-1701. , https://doi.org/10.1093/eurheartj/ehs092; Craig, CL, Marshall, AL, Sjöström, M, International physical activity questionnaire: 12-country reliability and validity (2003) Med Sci Sports Exerc, 35 (8), pp. 1381-1395. , https://doi.org/10.1249/01.MSS.0000078924.61453.FB; Zigmond, AS, Snaith, RP., Hospital anxiety and depression scale (1983) Acta Psychiat Scand, 67, pp. 361-370. , https://doi.org/10.1111/j.1600-0447.1983.tb09716.x; Cowie, MR, Bax, J, Bruining, N, e-Health: A position statement of the European Society of Cardiology (2016) Eur Heart J, 37 (1), pp. 63-66. , https://doi.org/10.1093/eurheartj/ehv416; Muromtseva, GA, Kontsevaya, AV, Konstantinov, VV, The prevalence of non-infectious diseases risk factors in Russian population in 2012-2013 years. The results of ECVD-RF (2014) Cardiovascular Therapy and Prevention, 13 (6), pp. 4-11. , https://doi.org/10.15829/1728-8800-2014-4-21-28, 21; Hutchesson, MJ, Rollo, ME, Krukowski, R, Health interventions for the prevention and treatment of overweight and obesity in adults: A systematic review with meta-analysis (2015) Obes Rev, 16 (5), pp. 376-392. , https://doi.org/10.1111/obr.12268, Epub 2015 Mar 5; Akbari, M, Lankarani, KB, Naghibzadeh-Tahami, A, The effects of mobile health interventions on lipid profiles among patients with metabolic syndrome and related disorders: A systematic review and meta-analysis of randomized controlled trials (2019) Diabetes Metab Syndr, 13 (3), pp. 1949-1955. ,

https://doi.org/10.1016/j.dsx.2019.04.011, Epub 2019 Apr 12; Ma, Y, Cheng, HY, Cheng, L, The effectiveness of electronic health interventions on blood pressure control, self-care behavioural outcomes and psychosocial well-being in patients with hypertension: A systematic review and meta-analysis (2019) Int J Nurs Stud, 92, pp. 27-46. , https://doi.org/10.1016/j.ijnurstu.2018.11.007, Epub 2018 Dec 31; Pogosova, NV, Yufereva Yu, M, Yusubova, AI, Preventive counselling with the use of remote technologies provides effective control of metabolic risk factors in patients with high and very high cardiovascular risk (2018) Kardiologiia, 58 (10), pp. 34-44. , https://doi.org/10.18087/cardio.2018.10.10183; Pogosova, NV, Yufereva Yu, M, Yusubova, AI, The effectiveness of preventive counseling with the use of remote technologies on medical awareness of cardiovascular ris factors in paients with high and very high cardiovascular risk (2019) Kardiologiia, 59 (10S), pp. 31-38. , https://doi.org/10.18087/cardio.n472; Byambasukh, O, Eisenga, MF, Gansevoort, RT, Body fat estimates from bioelectrical impedance equations in cardiovascular risk assessment: The PREVEND cohort study (2019) Eur J Prev Cardiol, 26 (9), pp. 905-916. , https://doi.org/10.1177/2047487319833283, Epub 2019 Feb 21; Feldman, DI, Theodore Robison, W, Pacor, JM, Harnessing mHealth technologies to increase physical activity and prevent cardiovascular disease (2018) Clin Cardiol, 41, pp. 985-991. , https://doi.org/10.1002/clc.22968; Andre, N, Wibawanti, R, Siswanto, BB., Mobile phone-based intervention in hypertension management (2019) Int J Hypertens, 2019. , https://doi.org/10.1155/2019/9021017},
correspondence_address1={Saner, H.; Institute for Social and Preventive Medicine, Switzerland; email: hugo.saner@med.unibe.ch},
publisher={Ubiquity Press},
issn={22118160},
pubmed_id={33833945},
language={English},
abbrev_source_title={Glo. Heart},
document_type={Article},
source={Scopus},
}

@ARTICLE{Céspedes2021,
author={Céspedes, N. and Irfan, B. and Senft, E. and Cifuentes, C.A. and Gutierrez, L.F. and Rincon-Roncancio, M. and Belpaeme, T. and Múnera, M.},
title={A Socially Assistive Robot for Long-Term Cardiac Rehabilitation in the Real World},
journal={Frontiers in Neurorobotics},
year={2021},
volume={15},
doi={10.3389/fnbot.2021.633248},
art_number={633248},
note={cited By 0},
url={https://www.scopus.com/inward/record.uri?eid=2-s2.0-85103616004&doi=10.3389%2ffnbot.2021.633248&partnerID=40&md5=024eb7908aa3905e197ce9d357cb4b8c},
affiliation={Biomedical Engineering Department, Colombian School of Engineering Julio Garavito, Bogotá, Colombia; Centre for Robotics and Neural Systems, University of Plymouth, Plymouth, United Kingdom; Department of Computer Sciences, University of Wisconsin-Madison, Madison, WI, United States; Fundación Cardioinfantil-Instituto de Cardiología, Bogotá, Colombia; IDLab-imec, Ghent University, Ghent, Belgium},
abstract={What are the benefits of using a socially assistive robot for

long-term cardiac rehabilitation? To answer this question we designed and conducted a real-world long-term study, in collaboration with medical specialists, at the Fundación Cardioinfantil-Instituto de Cardiología clinic (Bogotá, Colombia) lasting 2.5 years. The study took place within the context of the outpatient phase of patients' cardiac rehabilitation programme and aimed to compare the patients' progress and adherence in the conventional cardiac rehabilitation programme (control condition) against rehabilitation supported by a fully autonomous socially assistive robot which continuously monitored the patients during exercise to provide immediate feedback and motivation based on sensory measures (robot condition). The explicit aim of the social robot is to improve patient motivation and increase adherence to the programme to ensure a complete recovery. We recruited 15 patients per condition. The cardiac rehabilitation programme was designed to last 36 sessions (18 weeks) per patient. The findings suggest that robot increases adherence (by 13.3%) and leads to faster completion of the programme. In addition, the patients assisted by the robot had more rapid improvement in their recovery heart rate, better physical activity performance and a higher improvement in cardiovascular functioning, which indicate a successful cardiac rehabilitation programme performance. Moreover, the medical staff and the patients acknowledged that the robot improved the patient motivation and adherence to the programme, supporting its potential in addressing the major challenges in rehabilitation programmes. © Copyright © 2021 Céspedes, Irfan, Senft, Cifuentes, Gutierrez, Rincon-Roncancio, Belpaeme and Múnera.},
author_keywords={cardiac rehabilitation; human-robot interaction; human-robot interface; long-term interaction; social assistive robotics; social robot},
keywords={Heart; Medical robotics; Motivation; Social robots, Cardiac rehabilitations; Colombia; Immediate feedbacks; Long term study; Physical activity; Real-world; Recovery heart rates; Socially assistive robots, Patient rehabilitation},
references={Aamot, I.L., Forbord, S.H., Karlsen, T., Støylen, A., Does rating of perceived exertion result in target exercise intensity during interval training in cardiac rehabilitation? A study of the Borg scale versus a heart rate monitor (2014) J. Sci. Med. Sport, 17, pp. 541-545. , 23988787; Aguirre, A., Sierra, S.D., Múnera, M., Cifuentes, C.A., Online system for gait parameters estimation using a lrf sensor for assistive devices (2020) IEEE Sens. J; Altenhoener, T., Leppin, A., Grande, G., Romppel, M., Social inequality in patients? physical and psychological state and participation in rehabilitation after myocardial infarction in Germany (2005) Int. J. Rehabil. Res, 28, pp. 251-257. , 16046919; Anderson, L., Thompson, D.R., Oldridge, N., Zwisler, A., Rees, K., Martin, N., Exercise-based cardiac rehabilitation for coronary heart disease (2016) Cochrane Syst. Rev, 67, pp. 1-12. , 26764059; (2021) Spatio-Temporal Parameters - What Do They Mean, , https://www.aposhealth.co.uk/blog/apostherapy/spatio-temporal-parameters-what-do-they-mean/, AposHealth® UK. Available online at:, (accessed February 26, 2021; Beswick, A.D., Rees, K., West, R.R., Taylor, F.C., Burke, M., Griebsch, I., Improving uptake and adherence in cardiac rehabilitation: literature review (2005) J. Adv. Nurs, 49, pp. 538-555. , 15713186; Bethell, H., Lewin, R., Dalal, H., Cardiac rehabilitation in the United Kingdom (2009) Heart, 95, pp. 271-275; Bickmore, T.W., Picard, R.W., Establishing and maintaining long-term human-computer relationships (2005) ACM Trans. Comput. Hum. Interact, 12, pp. 293-327; Borg, G., (1998) Borg's Perceived Exertion and Pain Scales, , Champaign, IL, Human Kinetics; Carlson, J., Johnson, J., Franklin, B., VanderLaan, R., Program participation, exercise adherence, cardiovascular outcomes, and program cost of traditional versus modified cardiac

rehabilitation (2000) Am. J. Cardiol, 86, pp. 17-23. , 10867086; Carnethon, M., Sternfeld, B., Liu, K., Jcob, D., Schreiner, P., Williams, D., Correlates of heart rate recovery over 20 years in a population sample (2015) J. Investig. Dermatol, 135, pp. 612-615. , 21796053; Carrillo, F.M., Butchart, J., Kruse, N., Scheinberg, A., Wise, L., McCarthy, C., Physiotherapists' acceptance of a socially assistive robot in ongoing clinical deployment (2018) 2018 27th IEEE International Symposium on Robot and Human Interactive Communication (RO-MAN), pp. 850-855. , Nanjing; Casas, J., Céspedes, N., Cifuentes, C., Gutierrez, L.F., Rincón-Roncancio, M., Múnera, M., Expectation vs. reality: Attitudes towards a socially assistive robot in cardiac rehabilitation (2019) Appl. Sci, 9, p. 4651; Casas, J., Gomez, N.C., Senft, E., Irfan, B., Gutiérrez, L.F., Rincón, M., Architecture for a social assistive robot in cardiac rehabilitation (2018) 2018 IEEE 2nd Colombian Conference on Robotics and Automation (CCRA), pp. 1-6. , c, Barranquilla; Casas, J., Irfan, B., Senft, E., Gutiérrez, L., Rincon-Roncancio, M., Munera, M., Social assistive robot for cardiac rehabilitation: a pilot study with patients with angioplasty (2018) Companion of the 2018 ACM/IEEE International Conference on Human-Robot Interaction, pp. 79-80. , a, Chicago, IL, ACM; Casas, J., Irfan, B., Senft, E., Gutierrez, L., Rincon-Roncancio, M., Munera, M., Towards a sar system for personalized cardiac rehabilitation: a patient with PCI (2018) 2018 ACM/IEEE International Conference on Human-Robot Interaction Personal Robots for Exercising and Coaching Workshop, , b, Chicago, IL, ACM; Casas, J., Senft, E., Gutierrez, L.F., Rincon-Rocancio, M., Munera, M., Belpaeme, T., Social assistive robots: assessing the impact of a training assistant robot in cardiac rehabilitation (2020) Int. J. Soc. Robot, 13, pp. 1-15; Clark, A.M., Hartling, L., Vandermeer, B., McAlister, F.A., Meta-analysis: secondary prevention programs for patients with coronary artery disease (2005) Ann. Intern. Med, 143, pp. 659-672. , 23426558; Cohen, J., (1988) Statistical Power Analysis for the Behavioral Sciences, , New York, NY, Routledge Academic, 17695343; Cooper, A.F., Weinman, J., Hankins, M., Jackson, G., Horne, R., Assessing patients' beliefs about cardiac rehabilitation as a basis for predicting attendance after acute myocardial infarction (2007) Heart, 93, pp. 53-58. , 16905630; Eriksson, J., Matarić, M.J., Winstein, C.J., Hands-off assistive robotics for post-stroke arm rehabilitation (2005) 9th International Conference on Rehabilitation Robotics, 2005, ICORR 2005, pp. 21-24. , Chicago, IL; Fasola, J., Matarić, M.J., Robot exercise instructor: a socially assistive robot system to monitor and encourage physical exercise for the elderly (2010) Proceedings - IEEE International Workshop on Robot and Human Interactive Communication, pp. 416-421. , Viareggio; Fasola, J., Mataric, M.J., Using socially assistive human-robot interaction to motivate physical exercise for older adults (2012) Proc. IEEE, 100, pp. 2512-2526; Fasola, J., Matarić, M.J., A socially assistive robot exercise coach for the elderly (2013) J. Hum. Robot Interact, 2, pp. 3-32; Feil-Seifer, D., Matarić, M.J., Defining socially assistive robotics (2005) 9th International Conference on Rehabilitation Robotics (ICORR), pp. 465-468. , Chicago, IL; Gadde, P., Kharrazi, H., Patel, H., MacDorman, K.F., Toward monitoring and increasing exercise adherence in older adults by robotic intervention: a proof of concept study (2011) J. Robot, 2011, pp. 1-11; Galve, E., Alegria, E., Cordero, A., Facila, L., de Bobadilla, J.F., Lluis-Ganella, C., Temas de actualidad en cardiologia: riesgo vascular y rehabilitacion cardiaca (2014) Revista Espanola de Cardiologia, 67, pp. 203-210; Giuliano, C., Parmenter, B.J., Baker, M., Mitchell, B.L., Williams, A.D., Lyndon, K., Cardiac rehabilitation for patients with coronary artery disease: a practical guide to enhance patient outcomes through continuity of care (2017) Clin. Med. Insights Cardiol, 11, pp. 1-7. , 28638244; Glass, G.V., McGaw, B., Smith, M.L., (1981)

Meta-Analysis Insocial Research, , Newbury Park, CA, Sage; Gockley, R., Bruce, A., Forlizzi, J., Michalowski, M., Mundell, A., Rosenthal, S., Designing robots for long-term social interaction (2005) 2005 IEEE/RSJ International Conference on Intelligent Robots and Systems, pp. 1338-1343. , Edmonton, AB; Gockley, R., Mataric, M.J., Encouraging physical therapy compliance with a hands-off mobile robot (2006) Proceedings of the 1st ACM SIGCHI/SIGART Conference on Human-Robot Interaction, HRI?06, pp. 150-155. , New York, NY, Association for Computing Machinery; Hammill, B.G., Curtis, L.H., Schulman, K.A., Whellan, D.J., Relationship between cardiac rehabilitation and long-term risks of death and myocardial infarction among elderly medicare beneficiaries (2010) Circulation, 121, pp. 63-70. , 20026778; Irfan, B., Céspedes Gomez, N., Casas, J., Senft, E., Gutiérrez, L.F., Rincon-Roncancio, M., Using a personalised socially assistive robot for cardiac rehabilitation: a long-term case study (2020) 29th IEEE International Symposium on Robot and Human Interactive Communication (RO-MAN), pp. 124-130. , IEEE; James, G.S., The comparison of several groups of observations when the ratios of the population variances are unknown (1951) Biometrika, 38, pp. 324-329; Johansen, S., The welch-james approximation to the distribution of the residual sum of squares in a weighted linear regression (1980) Biometrika, 67, pp. 85-92; Jolly, K., Taylor, R., Lip, G., Greenfield, S., Raftery, J., Mant, J., The Birmingham rehabilitation uptake maximisation study (brum). Home-based compared with hospital-based cardiac rehabilitation in a multi-ethnic population: cost-effectiveness and patient adherence (2007) Health Technol. Assess, 11, pp. 1-118. , 17767899; Kang, K.I., Freedman, S., Mataric, M.J., Cunningham, M.J., Lopez, B., A hands-off physical therapy assistance robot for cardiac patients (2005) 9th International Conference on Rehabilitation Robotics, 2005 (ICORR 2005), pp. 337-340. , Chicago, IL; Keselman, H.J., Wilcox, R.R., Lix, L.M., A generally robust approach to hypothesis testing in independent and correlated groups designs (2003) Psychophysiology, 40, pp. 586-596; Kidd, C.D., Breazeal, C., A robotic weight loss coach (2007) Proceedings of the 22nd National Conference on Artificial Intelligence, pp. 1985-1986. , Vancouver, BC, AAAI Press; Kraus, W., Keteyian, S., (2007) Cardiac Rehabilitation, , Totowa, NJ, Humana Press; Lane, G.W., Noronha, D., Rivera, A., Craig, K., Yee, C., Mills, B., Effectiveness of a social robot, “Paro,” in a VA long-term care setting (2016) Psychol. Serv, 13, pp. 292-299. , 27195530; Langer, A., Feingold-Polak, R., Mueller, O., Kellmeyer, P., Levy-Tzedek, S., Trust in socially assistive robots: considerations for use in rehabilitation (2019) Neurosci. Biobehav. Rev, 104, pp. 231-239. , 31348963; Lara, J.S., Casas, J., Aguirre, A., Munera, M., Rincon-Roncancio, M., Irfan, B., Human-robot sensor interface for cardiac rehabilitation (2017) 2017 International Conference on Rehabilitation Robotics (ICORR), pp. 1013-1018. , London, 28813954; Lawler, P.R., Filion, K.B., Eisenberg, M.J., Efficacy of exercise-based cardiac rehabilitation post-myocardial infarction: a systematic review and meta-analysis of randomized controlled trials (2011) Am. Heart J, 162, pp. 571.e2-584.e2. , 21982647; Leite, I., Martinho, C., Paiva, A., Social robots for long-term interaction: a survey (2013) Int. J. Soc. Robot, 5, pp. 291-308; Lemaignan, S., Garcia, F., Jacq, A., Dillenbourg, P., From real-time attention assessment to “with-me-ness” in human-robot interaction (2016) 2016 11th ACM/IEEE International Conference on Human-Robot Interaction (HRI), pp. 157-164. , Christchurch, IEEE; Li, J., The benefit of being physically present (2015) Int. J. Hum. Comput. Stud, 77, pp. 23-37; Maclean, N., Pound, P., A critical review of the concept of patient motivation in the literature on physical rehabilitation (2000) Soc. Sci. Med, 50, pp. 495-506. , 10641802; Martin, C.M., McConahay, D.R., Maximal treadmill exercise electrocardiography. Correlations with coronary

arteriography and cardiac hemodynamics (1972) Circulation, 46, pp. 956-962. , 5081147; Matarić, M., Eriksson, J., Feil-Seifer, D., Winstein, C.J., Socially assistive robotics for post-stroke rehabilitation (2007) J. Neuroeng. Rehabil, 4, pp. 1-9. , a, 17309795; Matarić, M., Scassellati, B., Socially assistive robotics (2016) Springer Handbook of Robotics, pp. 1973-1993. , Siciliano B., Khatib O., (eds), Cham, Springer, eds; Matarić, M., Tapus, A., Feil-Seifer, D., Personalized socially assistive robotics (2007) Workshop on Intelligent Systems for Assisted Cognition, , b, Rochester, NY, in; McKee, G., Biddle, M., Donnell, S.O., Mooney, M., Brien, F.O., Moser, D.K., Cardiac rehabilitation after myocardial infarction: What influences patients? intentions to attend? (2014) Eur. J. Cardiovasc. Nurs, 13, pp. 329-337. , 23818214; Oldridge, N.B., Guyatt, G.H., Fischer, M.E., Rimm, A.A., Cardiac rehabilitation after myocardial infarction: combined experience of randomized clinical trials (1988) JAMA, 260, pp. 945-950. , 3398199; Piepoli, M.F., Corre, U., Benzer, W., Bjarnason-Wehrens, B., Dendale, P., Gaita, D., Secondary prevention through cardiac rehabilitation: from knowledge to implementation. A position paper from the cardiac rehabilitation section of the European Association of Cardiovascular Prevention and Rehabilitation (2010) Eur. J. Cardiovasc. Prev. Rehabil, 17, pp. 1-17. , 19952757; Pinto, M.J., Aguirre, A., Cifuentes, C.A., Munera, M., Wearable sensors for monitoring exercise and fatigue estimation in rehabilitation (2020) Internet of Medical Things: Paradigm of Wearable Devices, , Cardona M., Solanki V.K., García Cena C.E., (eds), Boca Raton, FL, Taylor & Francis Group, eds; Riek, L.D., Healthcare robotics (2017) Commun. ACM, 60, pp. 68-78; Ruano-Ravina, A., Pena-Gil, C., Abu-Assi, E., Raposeiras, S., van 't Hof, A., Meindersma, E., Participation and adherence to cardiac rehabilitation programs. A systematic review (2016) Int. J. Cardiol, 223, pp. 436-443. , 27557484; Šabanović, S., Bennett, C.C., Chang, W., Huber, L., Paro robot affects diverse interaction modalities in group sensory therapy for older adults with dementia (2013) 2013 IEEE 13th International Conference on Rehabilitation Robotics (ICORR), pp. 1-6. , Seattle, WA, IEEE, 24187245; Scane, K., Alter, D., Oh, P., Brooks, D., Adherence to a cardiac rehabilitation home program model of care: a comparison to a well-established traditional on-site supervised program (2012) Appl. Physiol. Nutr. Metab, 37, pp. 206-213. , 22360343; Schafer, R., Anderson, J., (1987) Clinical Biomechanics, , Baltimore, MD, Williams & Wilkins; Shahsavari, H., Shahriari, M., Alimohammadi, N., Motivational factors of adherence to cardiac rehabilitation (2012) Iran. J. Nurs. Midwif. Res, 17, pp. 318-324. , 23833634; She, J., Nakamura, H., Makino, K., Ohyama, Y., Hashimoto, H., Selection of suitable maximum-heart-rate formulas for use with Karvonen formula to calculate exercise intensity (2014) Int. J. Automat. Comput, 12, pp. 62-69; Siegert, R.J., Taylor, W.J., Theoretical aspects of goal-setting and motivation in rehabilitation (2004) Disabil. Rehabil, 26, pp. 1-8. , 14660192; Suaya, J., Stason, W., Ades, P., Normand, S., Shepard, D., Cardiac rehabilitation and survival in older coronary patients (2009) J. Am. Coll. Cardiol, 54, pp. 25-33. , 19555836; Süssenbach, L., Riether, N., Schneider, S., Berger, I., Kummert, F., Lutkebohle, I., A robot as fitness companion: towards an interactive action-based motivation model (2014) The 23rd IEEE International Symposium on Robot and Human Interactive Communication, pp. 286-293. , Edinburgh; Swift-Spong, K., Short, E., Wade, E., Mataric, M.J., Effects of comparative feedback from a socially assistive robot on self-efficacy in post-stroke rehabilitation (2015) 2015 IEEE International Conference on Rehabilitation Robotics (ICORR), pp. 764-769. , Nanyang; Taylor, R., Brown, A., Ebrahim, S.A., Exercise-based cardiac rehabilitation in patients with coronary heart disease: meta-analysis outcomes revisited (2012) Future Cardiol, 8, pp. 729-751. , 23013125; Taylor, R.S.,

Brown, A., Ebrahim, S., Jolliffe, J., Noorani, H., Rees, K., Exercise-based rehabilitation for patients with coronary heart disease: systematic review and meta-analysis of randomized controlled trials (2004) Am. J. Med, 116, pp. 682-692. , 15121495; Thomas, R.J., King, M., Lui, K., Oldridge, N., Pina, I.L., Spertus, J., AACVPR/ACC/AHA 2007 performance measures on cardiac rehabilitation for referral to and delivery of cardiac rehabilitation/secondary prevention services (2007) J. Cardiopulm. Rehabil. Prev, 27, pp. 260-290. , 17903645; Thompson, D., (2002) Stride Analysis, , https://ouhsc.edu/bserdac/dthompso/web/gait/knmatics/stride.htm, Available online at:, (accessed June 16, 2020; Turk-Adawi, K., Supervia, M., Lopez-Jimenez, F., Pesah, E., Ding, R., Britto, R.R., Cardiac rehabilitation availability and density around the globe (2019) eClinicalmedicine, 13, pp. 31-45. , 31517261; Turk-Adawi, K.I., Oldridge, N.B., Tarima, S.S., Stason, W.B., Shepard, D.S., Cardiac rehabilitation patient and organizational factors: what keeps patients in programs? (2013) J. Am. Heart Assoc, 2, p. e000418. , 24145743; Vasco, V., Willems, C., Chevalier, P., De Tommaso, D., Gower, V., Gramatica, F., Train with me: a study comparing a socially assistive robot and a virtual agent for a rehabilitation task (2019) International Conference on Social Robotics (ICSR 2019), , Madrid, Springer; Venkatesh, V., Morris, M.G., Davis, G.B., Davis, F.D., User acceptance of information technology: toward a unified view (2003) MIS Q, 27, pp. 425-478; Villacorta, P.J., The welchADF package for robust hypothesis testing in unbalanced multivariate mixed models with heteroscedastic and non-normal data (2017) R J, 9, pp. 309-328; Welch, B.L., The significance of the difference between two means when the population variances are unequal (1938) Biometrika, 29, pp. 350-362. , 7568575; Welch, B.L., On the comparison of several mean values: an alternative approach (1951) Biometrika, 38, pp. 330-336; Winkle, K., Caleb-Solly, P., Turton, A., Bremner, P., Social robots for engagement in rehabilitative therapies: design implications from a study with therapists (2018) Proceedings of the 2018 ACM/IEEE International Conference on Human-Robot Interaction, HRI?18, pp. 289-297. , New York, NY, Association for Computing Machinery; (2011) World Report on Disability, 91. , Geneva, The World Bank},
correspondence_address1={Múnera, M.; Biomedical Engineering Department, Colombia; email: marcela.munera@escuelaing.edu.co},
publisher={Frontiers Media S.A.},
issn={16625218},
language={English},
abbrev_source_title={Front. Neurorobotics},
document_type={Article},
source={Scopus},
}

@ARTICLE{Jörres2021,
author={Jörres, M. and Gunga, H.-C. and Steinach, M.},
title={Physiological Changes, Activity, and Stress During a 100-km–24-h Walking-March},
journal={Frontiers in Physiology},
year={2021},
volume={12},
doi={10.3389/fphys.2021.640710},
art_number={640710},
note={cited By 0},
url={https://www.scopus.com/inward/record.uri?eid=2-s2.0-85103085713&doi=10.3389%2ffphys.2021.640710&partnerID=40&md5=c05110ed668b3f9542bcc046394769fc},

affiliation={Charité – Universitätsmedizin Berlin, Freie Universität Berlin, Humboldt-Universität zu Berlin, Berlin Institute of Health, Institute of Physiology, Center for Space Medicine and Extreme Environments, Germany},
abstract={Background: Long-endurance exercises like ultramarathons are known to elicit various metabolic and physiological changes in the human body. However, little is known about very long-duration exercise at low intensities regarding healthy human subjects. Aim: The purpose of this study was to evaluate changes in body composition and metabolism in long-endurance but low-intensity events. Methods: Twenty-five male and 18 female healthy recreational athletes (age 34.6 ± 8.8 years; BMI: 22.4 ± 2.0 kg/m2) of the “100 km Mammutmarsch” were recruited for participation during the events in 2014–2016. Other than classical ultramarathons, the “Mammutmarsch” is a hiking event, in which participants were required to walk but not run or jog. It was expected to complete the 100-km distance within 24 h, resulting in a calculated mean speed of 4.17 km/h, which fits to the mean speed observed (4.12 ± 0.76 km/h). As not all participants reached the finish line, comparison of finishers (FIN, n = 11) and non-finishers (NON, n = 21) allowed differential assessment of performance. Body composition measured through bioelectrical impedance analysis (BIA) was determined pre- and post-event, and serum samples were taken pre-event, at 30, 70, and 100 km to determine NT-pro-BNP, troponin T, C-reactive protein (CRP), cortisol, low-density lipoprotein (LDL), high-density lipoprotein (HDL), triglycerides, total cholesterol, total creatine kinase (CK), CK-MB, aminotransferase (AST), ALT, and sodium levels. Nineteen participants wore actimeter armbands (SenseWear®) to gain information about body activity and exercise intensity [metabolic equivalent of task (MET)]. Sixteen participants wore mobile heart rate monitors to assess mean heart rate during the race. Serum parameter alterations over the course of the race were analyzed with mixed-effects ANOVA and additional t-tests. All serum parameters were analyzed for correlation concerning different MET levels, speed, age, BMI, baseline NT-pro-BNP, mean heart rate during the race, and sex with linear regression analysis. Results: We found significant elevations for muscle and cardiac stress markers (CRP, CK, CK-MB, AST, ALT, cortisol, and NT-pro-BNP) as well as decreasing markers of lipid metabolism (cholesterol, triglycerides, LDL). Although the intensity level demanded from our participants was low compared with other studies on (ultra-) marathons, the alteration of tested parameters was similar to those of high-intensity exercise, e.g., NT-pro-BNP showed a fourfold increase (p < 0.01) and LDL decreased by 20% (p = 0.05). Besides the duration of exercise, age, BMI, training status, and sex are relevant parameters that influence the elevation of stress factors. Notably, our data indicate that NT-pro-BNP might be a marker for cardiovascular fitness also in healthy adults. Conclusion: This low-intensity long-endurance walk evoked a strong systemic reaction and large cell stress and shifted to a favorable lipid profile, comparable to higher intensity events. Despite increasing cardiac stress parameters, there were no indications of cardiac cell damage. Remarkably, the duration seems to have a greater influence on stress markers and metabolism than intensity. © Copyright © 2021 Jörres, Gunga and Steinach.},
author_keywords={body composition; cardiac stress; endurance; exercise performance; low intensity; physiological changes; ultra-endurance; ultramarathon},
references={Aadahl, M., Kjaer, M., Jørgensen, T., Associations between overall physical activity level and cardiovascular risk factors in an adult population (2007) Eur. J. Epidemiol, 22, pp. 369-378; Achten, J., Gleeson, M., Jeukendrup, A.E., Determination of the exercise intensity that elicits maximal fat oxidation (2002) Med. Sci. Sports Exerc, 34, pp. 92-97; Ainsworth, B.E.,

Haskell, W.L., Leon, A.S., Jacobs, D.R., Jr., Montoye, H.J., Sallis, J.F., Compendium of physical activities: classification of energy costs of human physical activities (1993) Med. Sci. Sports Exerc, 25, pp. 71-80; Allen, J., Sun, Y., Woods, J.A., Exercise and the regulation of inflammatory responses (2015) Prog. Mol. Biol. Transl. Sci, 135, pp. 337-354; Alpert, S.S., A limit on the energy transfer rate from the human fat store in hypophagia (2005) J. Theor. Biol, 233, pp. 1-13; Anton, S.D., Duncan, G.E., Limacher, M.C., Martin, A.D., Perri, M.G., How much walking is needed to improve cardiorespiratory fitness? An examination of the 2008 Physical Activity Guidelines for Americans (2011) Res. Q. Exerc. Sport, 82, pp. 365-370; Arai, S.R., Butzlaff, A., Stotts, N.A., Puntillo, K.A., Quench the thirst: lessons from clinical thirst trials (2014) Biol. Res. Nurs, 16, pp. 456-466; Arakawa, K., Hosono, A., Shibata, K., Ghadimi, R., Fuku, M., Goto, C., Changes in blood biochemical markers before, during, and after a 2-day ultramarathon (2016) Open Access J. Sports Med, 7, pp. 43-50; Arocha Rodulfo, J.I., Sedentary lifestyle a disease from xxi century (2019) Clin. Investig. Arterioscler, 31, pp. 233-240; Baird, M.F., Graham, S.M., Baker, J.S., Bickerstaff, G.F., Creatine-kinase- and exercise-related muscle damage implications for muscle performance and recovery (2012) J. Nutr. Metab, 2012; Banfi, G., Colombini, A., Lombardi, G., Lubkowska, A., Metabolic markers in sports medicine (2012) Adv. Clin. Chem, 56, pp. 1-54; Bassett, D.R., Jr., Howley, E.T., Limiting factors for maximum oxygen uptake and determinants of endurance performance (2000) Med. Sci. Sports Exerc, 32, pp. 70-84; Baur, D.A., Bach, C.W., Hyder, W.J., Ormsbee, M.J., Fluid retention, muscle damage, and altered body composition at the Ultraman triathlon (2016) Eur. J. Appl. Physiol, 116, pp. 447-458; Benda, N.M., Hopman, M.T., van Dijk, A.P., Oxborough, D., George, K.P., Thijssen, D.H., Impact of prolonged walking exercise on cardiac structure and function in cardiac patients versus healthy controls (2016) Eur. J. Prev. Cardiol, 23, pp. 1252-1260; Beyer, S., Gender differences in the accuracy of self-evaluations of performance (1990) J. Pers. Soc. Psychol, 59, pp. 960-970; Cerqueira, É., Marinho, D.A., Neiva, H.P., Lourenço, O., Inflammatory effects of high and moderate intensity exercise-a systematic review (2020) Front. Physiol, 10 (1550); Coker, R.H., Weaver, A.N., Coker, M.S., Murphy, C.J., Gunga, H.C., Steinach, M., Metabolic responses to the yukon arctic ultra: longest and coldest in the world (2017) Med. Sci. Sports Exerc, 49, pp. 357-362; Cowie, M.R., Jourdain, P., Maisel, A., Dahlstrom, U., Follath, F., Isnard, R., Clinical applications of B-type natriuretic peptide (BNP) testing (2003) Eur. Heart J, 24, pp. 1710-1718; Coyle, E.F., Coggan, A.R., Hemmert, M.K., Ivy, J.L., Muscle glycogen utilization during prolonged strenuous exercise when fed carbohydrate (1986) J. Appl. Physiol, 61, pp. 165-172; Davis, C.E., Williams, D.H., Oganov, R.G., Tao, S.C., Rywik, S.L., Stein, Y., Sex difference in high density lipoprotein cholesterol in six countries (1996) Am. J. Epidemiol, 143, pp. 1100-1106; (2020), http://www.dakks.de/as/ast/d/D-ML-17085-01-00.pdf, (accessed January 06, 2020), Available online at; Doyen, B., Matelot, D., Carré, F., Asymptomatic bradycardia amongst endurance athletes (2019) Phys. Sportsmed, 47, pp. 249-252; Edwards, S., Clow, A., Evans, P., Hucklebridge, F., Exploration of the awakenin/g cortisol response in relation to diurnal cortisol secretory activity (2001) Life Sci, 68, pp. 2093-2103; Eijsvogels, T., George, K., Shave, R., Gaze, D., Levine, B.D., Hopman, M.T., Effect of prolonged walking on cardiac troponin levels (2010) Am. J. Cardiol, 105, pp. 267-272; Eijsvogels, T.M.H., Maessen, M.F.H., Exercise for coronary heart disease patients: little is good, more is better, vigorous is best (2017) J. Am. Coll. Cardiol, 70, pp. 1701-1703; Emed, L.G., Passaglia, D.G., Guerios, S.T., João, P.G., Moser, A.I., Abdalla, D.S., Acute modification in plasma lipid levels in ultramarathon

runners (2016) J. Sports Sci, 34, pp. 1657-1661; Fallon, K.E., The acute phase response and exercise: the ultramarathon as prototype exercise (2001) Clin. J. Sport Med, 11, pp. 38-43; Fikenzer, K., Fikenzer, S., Laufs, U., Werner, C., Effects of endurance training on serum lipids (2018) Vascul. Pharmacol, 101, pp. 9-20; Ginsburg, G.S., Agil, A., O'Toole, M., Rimm, E., Douglas, P.S., Rifai, N., Effects of a single bout of ultraendurance exercise on lipid levels and susceptibility of lipids to peroxidation in triathletes (1996) JAMA, 276, pp. 221-225; Di Angelantonio, E., Bhupathiraju, S.H.N., Wormser, D., Gao, P., Kaptoge, S., Body-mass index and all-cause mortality: individual-participant-data meta-analysis of 239 prospective studies in four continents (2016) Lancet, 388, pp. 776-786; (2020), http://iau-ultramarathon.org, (accessed July 13, 2019), Available online at; Jaffrin, M.Y., Morel, H., Body fluid volumes measurements by impedance: a review of bioimpedance spectroscopy (BIS) and bioimpedance analysis (BIA) methods (2008) Med. Eng. Phys, 30, pp. 1257-1269; Jensen-Urstad, K., Saltin, B., Ericson, M., Storck, N., Jensen-Urstad, M., Pronounced resting bradycardia in male elite runners is associated with high heart rate variability (1997) Scand. J. Med. Sci. Sports, 7, pp. 274-278; Kang, S.J., Ha, G.C., Ko, K.J., Association between resting heart rate, metabolic syndrome and cardiorespiratory fitness in Korean male adults (2017) J. Exerc. Sci. Fit, 15, pp. 27-31; Kannel, W.B., Kannel, C., Paffenbarger, R.S., Jr., Cupples, L.A., Heart rate and cardiovascular mortality: the Framingham Study (1987) Am. Heart J, 113, pp. 1489-1494; Kasprowicz, K., Ziemann, E., Ratkowski, W., Laskowski, R., Kaczor, J.J., Dadci, R., Running a 100-km ultra-marathon induces an inflammatory response but does not raise the level of the plasma iron-regulatory protein hepcidin (2013) J. Sports Med. Phys. Fitness, 53, pp. 533-537; Khan, F.Y., Rhabdomyolysis: a review of the literature (2009) Neth. J. Med, 67, pp. 272-283; Kim, J., So, W.Y., High body mass index is associated with the extent of muscle damage after eccentric exercise (2018) Int. J. Environ. Res. Public Health, 15 (1378); Kim, Y.J., Shin, Y.O., Lee, J.B., Lee, Y.H., Shin, K.A., Kim, A.C., The effects of running a 308 km ultra-marathon on cardiac markers (2014) Eur. J. Sport Sci, 14, pp. S92-S97; Kłapcińska, B., Waśkiewicz, Z., Chrapusta, S.J., Sadowska-Krêpa, E., Czuba, M., Langfort, J., Metabolic responses to a 48-h ultra-marathon run in middle-aged male amateur runners (2013) Eur. J. Appl. Physiol, 113, pp. 2781-2793; Knechtle, B., Nikolaidis, P.T., Physiology and pathophysiology in ultra-marathon running (2018) Front. Physiol, 9 (634); Knechtle, B., Salas Fraire, O., Andonie, J.L., Kohler, G., Effect of a multistage ultra-endurance triathlon on body composition: World Challenge Deca Iron Triathlon 2006 (2008) Br. J. Sports Med, 42, pp. 121-125. , a discussion 125; Knechtle, B., Scheer, V., Nikolaidis, P.T., Sousa, C.V., Participation and performance trends in the oldest 100-km Ultramarathon in the World (2020) Int. J. Environ. Res. Public Health, 17 (1719); Knechtle, B., Schwanke, M., Knechtle, P., Kohler, G., Decrease in body fat during an ultra-endurance triathlon is associated with race intensity (2008) Br. J. Sports Med, 42, pp. 609-613. , b; Knechtle, B., Valeri, F., Zingg, M.A., Rosemann, T., Rüst, C.A., What is the age for the fastest ultra-marathon performance in time-limited races from 6 h to 10 days? (2014) Age, 36 (9715); Koller, A., Summer, P., Moser, H., Regular exercise and subclinical myocardial injury during prolonged aerobic exercise (1999) JAMA, 282 (1816); Koohsari, M.J., Shibata, A., Ishii, K., Kurosawa, S., Yasunaga, A., Hanibuchi, T., Built environment correlates of objectively-measured sedentary behaviours in densely-populated areas (2020) Health Place, 66; La Gerche, A., Connelly, K.A., Mooney, D.J., MacIsaac, A.I., Prior, D.L., Biochemical and functional abnormalities of left and right ventricular function after

ultra-endurance exercise (2008) Heart, 94, pp. 860-866; Lee, J.B., Kim, J.H., Murota, H., Perspiration functions in different ethnic, age, and sex populations: modification of Sudomotor function (2016) Curr. Probl. Dermatol, 51, pp. 109-119; Legaz-Arrese, A., George, K., Carranza-García, L.E., Munguía-Izquierdo, D., Moros-García, T., Serrano-Ostáriz, E., The impact of exercise intensity on the release of cardiac biomarkers in marathon runners (2011) Eur. J. Appl. Physiol, 111, pp. 2961-2967; Lin, C.Y., Koohsari, M.J., Liao, Y., Ishii, K., Shibata, A., Nakaya, T., Workplace neighbourhood built environment and workers' physically-active and sedentary behaviour: a systematic review of observational studies (2020) Int. J. Behav. Nutr. Phys. Act, 17 (148); Lukaski, H.C., Bolonchuk, W.W., Estimation of body fluid volumes using tetrapolar bioelectrical impedance measurements (1988) Aviat. Space Environ. Med, 59, pp. 1163-1169; Maisel, A.S., Krishnaswamy, P., Nowak, R.M., McCord, J., Hollander, J.E., Duc, P., Rapid measurement of B-type natriuretic peptide in the emergency diagnosis of heart failure (2002) N. Engl. J. Med, 347, pp. 161-167; (2018), https://mammutmarsch.de/, (accessed July 8, 2017), Available online at; Mann, S., Beedie, C., Jimenez, A., Differential effects of aerobic exercise, resistance training and combined exercise modalities on cholesterol and the lipid profile: review, synthesis and recommendations (2014) Sports Med, 44, pp. 211-221; Marklund, P., Mattsson, C.M., Wåhlin-Larsson, B., Ponsot, E., Lindvall, B., Lindvall, L., Extensive inflammatory cell infiltration in human skeletal muscle in response to an ultraendurance exercise bout in experienced athletes (2013) J. Appl. Physiol, 114, pp. 66-72; Maggioni, M.A., Rundfeldt, L.C., Gunga, H.-C., Joerres, M., Merati, G., Steinach, M., The advantage of supine and standing heart rate variability analysis to assess training status and performance in a walking ultramarathon (2020) Front. Physiol, 11 (731); Mayer-Davis, E.J., Costacou, T., Obesity and sedentary lifestyle: modifiable risk factors for prevention of type 2 diabetes (2001) Curr. Diab. Rep, 1, pp. 170-176; Mehta, R., Gaze, D., Mohan, S., Williams, K.L., Sprung, V., George, K., Post-exercise cardiac troponin release is related to exercise training history (2012) Int. J. Sports Med, 33, pp. 333-337; Mendes, M.A., da Silva, I., Ramires, V., Reichert, F., Martins, R., Ferreira, R., Metabolic equivalent of task (METs) thresholds as an indicator of physical activity intensity (2018) PLoS One, 13 (e0200701); Nakagawa, O., Ogawa, Y., Itoh, H., Suga, S., Komatsu, Y., Kishimoto, I., Rapid transcriptional activation and early mRNA turnover of brain natriuretic peptide in cardiocyte hypertrophy. Evidence for brain natriuretic peptide as an “emergency” cardiac hormone against ventricular overload (1995) J. Clin. Invest, 96, pp. 1280-1287; Neal, R.C., Ferdinand, K.C., Ycas, J., Miller, E., Relationship of ethnic origin, gender, and age to blood creatine kinase levels (2009) Am. J. Med, 122, pp. 73-78; Nichols, S., Taylor, C., Page, R., Kallvikbacka-Bennett, A., Nation, F., Goodman, T., Is cardiorespiratory fitness related to cardiometabolic health and all-cause mortality risk in patients with coronary heart disease? A care CR Study (2018) Sports Med. Open, 4 (22); Noakes, T.D., Sharwood, K., Speedy, D., Hew, T., Reid, S., Dugas, J., Three independent biological mechanisms cause exercise-associated hyponatremia: evidence from 2,135 weighed competitive athletic performances (2005) Proc. Natl. Acad. Sci. U.S.A, 102, pp. 18550-18555; Obling, K.H., Hansen, A.L., Overgaard, K., Normann, K., Sandbaek, A., Maindal, H.T., Association between self-reported and objectively measured physical fitness level in a middle-aged population in primary care (2015) Prev. Med. Rep, 2, pp. 462-466; Ohba, H., Takada, H., Musha, H., Nagashima, J., Mori, N., Awaya, T., Effects of prolonged strenuous exercise on plasma levels of atrial natriuretic peptide and brain natriuretic peptide in healthy men (2001) Am. Heart J, 141, pp. 751-758; Popovic, B., Popovic, D., Macut, D., Antic,

I.B., Isailovic, T., Ognjanovic, S., Acute response to endurance exercise stress: focus on catabolic/anabolic interplay between cortisol, testosterone, and sex hormone binding globulin in professional athletes (2019) J. Med. Biochem, 38, pp. 6-12; Saklayen, M.G., The global epidemic of the metabolic syndrome (2018) Curr. Hypertens Rep, 20 (12); Salvagno, G.L., Schena, F., Gelati, M., Danese, E., Cervellin, G., Guidi, G.C., The concentration of high-sensitivity troponin I, galectin-3 and NT-proBNP substantially increase after a 60-km ultramarathon (2014) Clin. Chem. Lab. Med, 52, pp. 267-272; Santos-Lozano, A., Hernández-Vicente, A., Pérez-Isaac, R., Santín-Medeiros, F., Cristi-Montero, C., Casajús, J.A., Is the SenseWear Armband accurate enough to quantify and estimate energy expenditure in healthy adults? (2017) Ann. Transl. Med, 5 (97); Sarko, J., Pollack, C.V., Jr., Cardiac troponins (2002) J. Emerg. Med, 23, pp. 57-65; Saugy, J., Place, N., Millet, G.Y., Degache, F., Schena, F., Millet, G.P., Alterations of Neuromuscular Function after the World's Most Challenging Mountain Ultra-Marathon (2013) PLoS One, 8 (e65596); Scharhag, J., George, K., Shave, R., Urhausen, A., Kindermann, W., Exercise-associated increases in cardiac biomarkers (2008) Med. Sci. Sports Exerc, 40, pp. 1408-1415; Scharhag, J., Herrmann, M., Urhausen, A., Haschke, M., Herrmann, W., Kindermann, W., Independent elevations of N-terminal pro-brain natriuretic peptide and cardiac troponins in endurance athletes after prolonged strenuous exercise (2005) Am. Heart J, 150, pp. 1128-1134; Scharhag, J., Herrmann, M., Weissinger, M., Herrmann, W., Kindermann, W., N-terminal B-type natriuretic peptide concentrations are similarly increased by 30 minutes of moderate and brisk walking in patients with coronary artery disease (2007) Clin. Res. Cardiol, 96, pp. 218-226; Scharhag, J., Urhausen, A., Schneider, G., Herrmann, M., Schumacher, K., Haschke, M., Reproducibility and clinical significance of exercise-induced increases in cardiac troponins and N-terminal pro brain natriuretic peptide in endurance athletes (2006) Eur J. Cardiovasc. Prev. Rehabil, 13, pp. 388-397; Scott, J.M., Esch, B.T., Shave, R., Warburton, D.E., Gaze, D., George, K., Cardiovascular consequences of completing a 160-km ultramarathon (2009) Med. Sci. Sports Exerc, 41, pp. 26-34; Sedaghat-Hamedani, F., Kayvanpour, E., Frankenstein, L., Mereles, D., Amr, A., Buss, S., Biomarker changes after strenuous exercise can mimic pulmonary embolism and cardiac injury—a metaanalysis of 45 studies (2015) Clin. Chem, 61, pp. 1246-1255; Segal, K.R., Van Loan, M., Fitzgerald, P.I., Hodgdon, J.A., Van Itallie, T.B., Lean body mass estimation by bioelectrical impedance analysis: a four-site cross-validation study (1988) Am. J. Clin. Nutr, 47, pp. 7-14; Serrano-Ostáriz, E., Terreros-Blanco, J.L., Legaz-Arrese, A., George, K., Shave, R., Bocos-Terraz, P., The impact of exercise duration and intensity on the release of cardiac biomarkers (2011) Scand. J. Med. Sci. Sports, 21, pp. 244-249; Shave, R., George, K.P., Atkinson, G., Hart, E., Middleton, N., Whyte, G., Exercise-induced cardiac troponin T release: a meta-analysis (2007) Med. Sci. Sports Exerc, 39, pp. 2099-2106; Sun, S.S., Chumlea, W.C., Heymsfield, S.B., Lukaski, H.C., Schoeller, D., Friedl, K., Development of bioelectrical impedance analysis prediction equations for body composition with the use of a multicomponent model for use in epidemiologic surveys (2003) Am. J. Clin. Nutr, 77, pp. 331-340; Van Hoye, K., Boen, F., Lefevre, J., Validation of the SenseWear Armband in different ambient temperatures (2014) J. Sports Sci, 33, pp. 1007-1018; Vilela, E.M., Bettencourt-Silva, R., Nunes, J.P., Ribeiro, V.G., BNP and NT-proBNP elevation after running—a systematic review (2015) Acta Cardiol, 70, pp. 501-509; Wachira, L.M., Muthuri, S.K., Ochola, S.A., Onywera, V.O., Tremblay, M.S., Screen-based sedentary behaviour and adiposity among school children: results from International Study of Childhood Obesity, Lifestyle and the Environment (ISCOLE) - Kenya (2018) PLoS One, 13 (e0199790);

Waśkiewicz, Z., Kłapcińska, B., Sadowska-Krêpa, E., Czuba, M., Kempa, K., Kimsa, E., Acute metabolic responses to a 24-h ultra-marathon race in male amateur runners (2012) Eur. J. Appl. Physiol, 112, pp. 1679-1688; Whyte, G., Age, sex and (the) race: gender and geriatrics in the ultra-endurance age (2014) Extrem. Physiol. Med, 3 (1); Williams, D.P., Jarczok, M.N., Ellis, R.J., Hillecke, T.K., Thayer, J.F., Koenig, J., Two-week test-retest reliability of the Polar§RS800CXTM to record heart rate variability (2017) Clin. Physiol. Funct. Imaging, 37, pp. 776-781; Wishnofsky, M., Caloric equivalents of gained or lost weight (1958) Am. J. Clin. Nutr, 6, pp. 542-546; Wu, H.J., Chen, K.T., Shee, B.W., Chang, H.C., Huang, Y.J., Yang, R.S., Effects of 24 h ultra-marathon on biochemical and hematological parameters (2004) World J. Gastroenterol, 10, pp. 2711-2714; Yoon, J.H., Park, Y., Ahn, J., Shin, K.A., Kim, Y.J., Changes in the markers of cardiac damage in men following long-distance and ultra-long-distance running races (2016) J. Sports Med. Phys. Fitness, 56, pp. 295-301; Zingg, M.A., Knechtle, B., Rosemann, T., Rüst, C.A., Performance differences between sexes in 50-mile to 3,100-mile ultramarathons (2015) Open Access J. Sports Med, 6, pp. 7-21},
correspondence_address1={Jörres, M.; Charité – Universitätsmedizin Berlin, Germany; email: marc.joerres@charite.de},
publisher={Frontiers Media S.A.},
issn={1664042X},
language={English},
abbrev_source_title={Front. Physiol.},
document_type={Article},
source={Scopus},
}

@ARTICLE{Valli2021705,
author={Valli, G. and Minnock, D. and Tarantino, G. and Neville, R.D.},
title={Delayed effect of different exercise modalities on glycaemic control in type 1 diabetes mellitus: A systematic review and meta-analysis},
journal={Nutrition, Metabolism and Cardiovascular Diseases},
year={2021},
volume={31},
number={3},
pages={705-716},
doi={10.1016/j.numecd.2020.12.006},
note={cited By 0},
url={https://www.scopus.com/inward/record.uri?eid=2-s2.0-85100397111&doi=10.1016%2fj.numecd.2020.12.006&partnerID=40&md5=f62054ffbc4a1a1c9964eabc0712f710},
affiliation={Neuromuscular Physiology Laboratory, Department of Biomedical Sciences, University of Padova, Italy; School of Public Health, Physiotherapy and Sports Science, University College Dublin, Ireland},
abstract={Background and aims: Despite the crucial role of exercise in the prevention of comorbidities and complications in type 1 diabetes mellitus (T1DM), people living with the disease are often insufficiently physically active, mainly due to the fear of hypoglycaemia. Research using continuous glucose monitoring (CGM) devices has shown that exercise affects glycaemic control in T1DM for over 24 h. The aim of this systematic review and meta-analysis is, therefore, to investigate the delayed effects of different exercise modalities on glycaemic control in adults with T1DM. Methods and results: The literature search of experimental studies was conducted on PubMed, SPORTDiscus and EMBASE from January 2000 to September 2019. Twelve studies using CGM devices were included. Compared to endurance, intermittent exercise

increased the time spent in hypoglycaemia (0.62, 0.07 to 1.18; standardised effect size, 95% CI) and reduced the mean interstitial glucose concentration (−0.88, −1.45 to −0.33). No differences emerged in the time spent in hyperglycaemia (−0.07, −0.58 to 0.45) or in the proportion of exercisers experiencing hypoglycaemic events (0.82, 0.45 to 1.49; proportion ratio, 95% CI) between conditions. The systematic review also found a reduced risk of hypoglycaemia if exercise is performed in the morning rather than in the afternoon, and with a 50% rapid-acting insulin reduction. It was not possible to determine the benefits of resistance exercise. Conclusions: For the first time, we systematically investigated the delayed effect of exercise in adults with T1DM, highlighted undetected effects, shortcomings in the existing literature, and provided suggestions to design future comparable studies. © 2020 The Italian Diabetes Society, the Italian Society for the Study of Atherosclerosis, the Italian Society of Human Nutrition and the Department of Clinical Medicine and Surgery, Federico II University},
author_keywords={Continuous glucose monitoring; Exercise; Glycaemic control; Hypoglycaemia; Type 1 diabetes},
keywords={hemoglobin A1c; short acting insulin; antidiabetic agent; biological marker; insulin, aerobic exercise; Article; blood glucose monitoring; brain perfusion; chronic fatigue syndrome; exercise; glucose blood level; glycemic control; high intensity interval training; human; hyperglycemia; hypoglycemia; insulin dependent diabetes mellitus; maximal oxygen uptake; medical record review; meta analysis; motor activity; peak oxygen uptake; physical education; Preferred Reporting Items for Systematic Reviews and Meta-Analyses; randomized controlled trial (topic); resistance training; running; sprinting exercise; systematic review; walking; adolescent; adult; blood; blood glucose monitoring; drug effect; female; healthy lifestyle; insulin dependent diabetes mellitus; male; metabolism; middle aged; time factor; young adult, Adolescent; Adult; Biomarkers; Blood Glucose; Blood Glucose Self-Monitoring; Diabetes Mellitus, Type 1; Exercise; Female; Glycemic Control; Healthy Lifestyle; Humans; Hypoglycemic Agents; Insulin; Male; Middle Aged; Time Factors; Young Adult},
references={McCrimmon, R.J., Sherwin, R.S., Hypoglycemia in type 1 diabetes (2010) Diabetes, 59 (10), pp. 2333-2339; Palladino, D.K., Helgeson, V.S., Adolescents, parents and physicians: a comparison of perspectives on type 1 diabetes self-care (2013) Can J Diabetes, 37 (3), pp. 175-181; Riddell, M.C., Gallen, I.W., Smart, C.E., Taplin, C.E., Adolfsson, P., Lumb, A.N., Exercise management in type 1 diabetes: a consensus statement (2017) Lancet Diabetes Endocrinol, 5 (5), pp. 377-390; Campbell, M.D., Walker, M., Bracken, R.M., Turner, D., Stevenson, E.J., Gonzalez, J.T., Insulin therapy and dietary adjustments to normalize glycemia and prevent nocturnal hypoglycemia after evening exercise in type 1 diabetes: a randomized controlled trial (2015) BMJ Open Diabetes Res Care, 3 (1); Donnelly, R., Emslie-Smith, A.M., Gardner, I.D., Morris, A.D., Vascular complications of diabetes (2000) Br Med J, 320 (7241), p. 1062; Alvarado-Martel, D., Velasco, R., Sánchez-Hernández, R.M., Carrillo, A., Nóvoa, F.J., Wägner, A.M., Quality of life and type 1 diabetes: a study assessing patients' perceptions and self-management needs (2015) Patient Prefer Adherence, 9, pp. 1315-1323; Costacou, T., The epidemiology of cardiovascular disease in adults with type 1 diabetes (2017) Curr Diabetes Rev, 13 (6), pp. 520-527; Booth, F.W., Gordon, S.E., Carlson, C.J., Hamilton, M.T., Waging war on modern chronic diseases: primary prevention through exercise biology (2000) J Appl Physiol, 88 (2), p. 774; Kruk, J., Physical activity in the prevention of the most frequent chronic diseases: an analysis of the recent evidence

(2007) Asian Pac J Cancer Prev APJCP, 8 (3), pp. 325-338; Bohn, B., Herbst, A., Pfeifer, M., Krakow, D., Zimny, S., Kopp, F., Impact of physical activity on glycemic control and prevalence of cardiovascular risk factors in adults with type 1 diabetes: a cross-sectional multicenter study of 18,028 patients (2015) Diabetes Care, 38 (8), pp. 1536-1543; Riddell, M.C., Burr, J., Evidence-based risk assessment and recommendations for physical activity clearance: diabetes mellitus and related comorbidities. Applied Physiology (2011) Nutr Metab, 36 (1), pp. 154-189; Tikkanen-Dolenc, H., Wadén, J., Forsblom, C., Harjutsalo, V., Thorn, L.M., Saraheimo, M., Physical activity reduces risk of premature mortality in patients with type 1 diabetes with and without kidney disease (2017) Diabetes Care, 40 (12), pp. 1727-1732; Yardley, J.E., Hay, J., Abou-Setta, A.M., Marks, S.D., McGavock, J., A systematic review and meta-analysis of exercise interventions in adults with type 1 diabetes (2014) Diabetes Res Clin Pract, 106 (3), pp. 393-400; Kennedy, A., Nirantharakumar, K., Chimen, M., Pang, T.T., Hemming, K., Andrews, R.C., Does exercise improve glycaemic control in type 1 diabetes? A systematic review and meta-analysis (2013) PloS One, 8 (3), p. e58861; Driscoll, K.A., Corbin, K.D., Maahs, D.M., Pratley, R., Bishop, F.K., Kahkoska, A., Biopsychosocial aspects of weight management in type 1 diabetes: a review and next steps (2017) Curr Diabetes Rep, 17 (8), pp. 1-9; Katzmarzyk, P.T., Janssen, I., Ardern, C.I., Physical inactivity, excess adiposity and premature mortality (2003) Obes Rev, 4 (4), pp. 257-290; Brazeau, A.-S., Rabasa-Lhoret, R., Strychar, I., Mircescu, H., Barriers to physical activity among patients with type 1 diabetes (2008) Diabetes Care, 31 (11), pp. 2108-2109; Martyn-Nemeth, P., Schwarz Farabi, S., Mihailescu, D., Nemeth, J., Quinn, L., Fear of hypoglycemia in adults with type 1 diabetes: impact of therapeutic advances and strategies for prevention - a review (2016) J Diabetes Complicat, 30 (1), pp. 167-177; Hansen, D., Niebauer, J., Cornelissen, V., Barna, O., Neunhäuserer, D., Stettler, C., Exercise prescription in patients with different combinations of cardiovascular disease risk factors: a consensus statement from the EXPERT working group (2018) Sports Med, 48 (8), pp. 1781-1797; Kalra, S., Mukherjee, J.J., Venkataraman, S., Bantwal, G., Shaikh, S., Saboo, B., Hypoglycemia: the neglected complication (2013) Indian J Endocrinol Metabol, 17 (5), pp. 819-834; Moher, D., Liberati, A., Tetzlaff, J., Altman, D.G., Grp, P., Preferred reporting items for systematic reviews and meta-analyses: the PRISMA statement (2009) J Clin Epidemiol, 62 (10), pp. 1006-1012; Higgins, J.P.T., Thomas, J., Chandler, J., Cumpston, M., Li, T., Page, M.J., Cochrane Handbook for systematic reviews of interventions version 6.0 (updated July 2019) (2019), http://www.training.cochrane.org/handbook, Cochrane (Accessed 10 January 2020); Shamseer, L., Moher, D., Clarke, M., Ghersi, D., Liberati, A., Petticrew, M., Preferred reporting items for systematic review and meta-analysis protocols (PRISMA-P) 2015: elaboration and explanation (2015) BMJ – Br Med J, 349 (1), p. g7647. , g; Houlder, S.K., Yardley, J.E., Continuous glucose monitoring and exercise in type 1 diabetes: past, present and future (2018) Biosensors, 8 (3), p. 73; Ouzzani, M., Hammady, H., Fedorowicz, Z., Elmagarmid, A., Rayyan-a web and mobile app for systematic reviews (2016) Syst Rev, 5 (1), p. 210; Ryan, R., Hill, S., How to GRADE the quality of the evidence. Cochrane consumers and communication group version 3.0 (Updated December 2016) (2016), http://cccrg.cochrane.org/author-resources, Cochrane (Accessed 10 January 2020); Hopkins, W.G., Marshall, S.W., Batterham, A.M., Hanin, J., Progressive statistics for studies in sports medicine and exercise science (2009) Med Sci Sports Exerc, 41 (1), pp. 3-12; Wasserman, S., Book reviews: statistical methods for meta-analysis Larry V. Hedges and Ingram Olkin New York (1985), Academic Press xxii + 369 pp. Journal of Educational Statistics; 1988. pp. 75–

78; Higgins, J.P.T., Commentary: heterogeneity in meta-analysis should be expected and appropriately quantified (2008) Int J Epidemiol, 37 (5), pp. 1158-1160; Higgins, J.P.T., Thompson, S.G., Quantifying heterogeneity in a meta-analysis (2002) Stat Med, 21 (11), pp. 1539-1558; Higgins, J.P.T., Thompson, S.G., Deeks, J.J., Altman, D.G., Measuring inconsistency in meta-analyses (2003) BMJ, 327 (7414), pp. 557-560; Schwarzer, G., Rücker, G., Carpenter, J.R., Meta-analysis with R. Report. Cham (2015), Springer; Iscoe, K.E., Riddell, M.C., Continuous moderate-intensity exercise with or without intermittent high-intensity work: effects on acute and late glycaemia in athletes with Type 1 diabetes mellitus (2011) Diabet Med, 28 (7), pp. 824-832; Moser, O., Tschakert, G., Mueller, A., Groeschl, W., Pieber, T.R., Obermayer-Pietsch, B., Effects of high-intensity interval exercise versus moderate continuous exercise on glucose homeostasis and hormone response in patients with type 1 diabetes mellitus using novel ultra-long-acting insulin (2015) PloS One, 10 (8); Campbell, M.D., West, D.J., Bain, S.C., Kingsley, M.I.C., Foley, P., Kilduff, L., Simulated games activity vs continuous running exercise: a novel comparison of the glycemic and metabolic responses in T1DM patients (2015) Scand J Med Sci Sports, 25 (2), pp. 216-222; Bally, L., Zueger, T., Buehler, T., Dokumaci, A.S., Speck, C., Pasi, N., Metabolic and hormonal response to intermittent high-intensity and continuous moderate intensity exercise in individuals with type 1 diabetes: a randomised crossover study (2016) Diabetologia, 59 (4), pp. 776-784; Zaharieva, D., Yavelberg, L., Jamnik, V., Cinar, A., Turksoy, K., Riddell, M.C., The effects of basal insulin suspension at the start of exercise on blood glucose levels during continuous versus circuit-based exercise in individuals with type 1 diabetes on continuous subcutaneous insulin infusion (2017) Diabetes Technol Therapeut, 19 (6), pp. 370-378; Maran, A., Pavan, P., Bonsembiante, B., Brugin, E., Ermolao, A., Avogaro, A., Continuous glucose monitoring reveals delayed nocturnal hypoglycemia after intermittent high-intensity exercise in nontrained patients with type 1 diabetes (2010) Diabetes Technol Therapeut, 12 (10), pp. 763-768; Yardley, J.E., Kenny, G.P., Perkins, B.A., Riddell, M.C., Balaa, N., Malcolm, J., Resistance versus aerobic exercise (2013) Diabetes Care, 36 (3), pp. 537-542; Reddy, R., El Youssef, J., Winters-Stone, K., Branigan, D., Leitschuh, J., Castle, J., The impact of exercise on sleep in adults with type 1 diabetes (2018) Diabetes Obes Metabol, 20 (2), pp. 443-447; Campbell, M.D., Walker, M., Trenell, M.I., Jakovljevic, D.G., Stevenson, E.J., Bracken, R.M., Large pre-and postexercise rapid-acting insulin reductions preserve glycemia and prevent early- but not late-onset hypoglycemia in patients with type 1 diabetes (2013) Diabetes Care, 36 (8), pp. 2217-2224; Gomez, A.M., Gomez, C., Aschner, P., Veloza, A., Muñoz, O., Rubio, C., Effects of performing morning versus afternoon exercise on glycemic control and hypoglycemia frequency in type 1 diabetes patients on sensor-augmented insulin pump therapy (2015) J Diabetes Sci Technol, 9 (3), pp. 619-624; Yardley, J.E., Kenny, G.P., Perkins, B.A., Riddell, M.C., Malcolm, J., Boulay, P., Effects of performing resistance exercise before versus after aerobic exercise on glycemia in type 1 diabetes (2012) Diabetes Care, 35 (4), pp. 669-675; Campbell, M.D., Walker, M., Trenell, M.I., Stevenson, E.J., Turner, D., Bracken, R.M., A low-glycemic index meal and bedtime snack prevents postprandial hyperglycemia and associated rises in inflammatory markers, providing protection from early but not late nocturnal hypoglycemia following evening exercise in type 1 diabetes (2014) Diabetes Care, 37 (7), pp. 1845-1853; Kemmer, F.W., Prevention of hypoglycemia during exercise in type I diabetes (1992) Diabetes Care, 15 (11), pp. 1732-1735; Tuominen, J.A., Karonen, S.L., Melamies, L., Bolli, G., Koivisto, V.A., Exercise-induced hypoglycaemia in IDDM patients treated with a short-acting

insulin analogue (1995) Diabetologia, 38 (1), pp. 106-111; Purdon, C., Brousson, M., Nyveen, S.L., Miles, P.D., Halter, J.B., Vranic, M., The roles of insulin and catecholamines in the glucoregulatory response during intense exercise and early recovery in insulin-dependent diabetic and control subjects (1993) J Clin Endocrinol Metab, 76 (3), pp. 566-573; Sigal, R.J., Purdon, C., Fisher, S.J., Halter, J.B., Vranic, M., Marliss, E.B., Hyperinsulinemia prevents prolonged hyperglycemia after intense exercise in insulin-dependent diabetic subjects (1994) J Clin Endocrinol Metab, 79 (4), pp. 1049-1057; Guelfi, K.J., Jones, T.W., Fournier, P.A., The decline in blood glucose levels is less with intermittent high-intensity compared with moderate exercise in individuals with type 1 diabetes (2005) Diabetes Care, 28 (6), pp. 1289-1294; Moussa, E., Zouhal, H., Vincent, S., Proiux, J., Delamarche, P., Gratas-Delamarche, A., Effect of sprint duration (6 s or 30 s) on plasma glucose regulation in untrained male subjects (2003) J Sports Med Phys Fit, 43 (4), p. 546; Heinemann, L., Freckmann, G., CGM versus FGM; or, continuous glucose monitoring is not flash glucose monitoring (2015) Journal of Diabetes Science and Technology, 9 (5), pp. 947-950; Bussau, V.A., Ferreira, L.D., Jones, T.W., Fournier, P.A., The 10-s maximal sprint (2006) Diabetes Care, 29 (3), p. 601; Riebe, D., Ehrman, J.K., Liguori, G., Magal, M., American College of Sports M. ACSM's guidelines for exercise testing and prescription (2017), Wolters Kluwer Philadelphia, [Pennsylvania]; Battelino, T., Danne, T., Bergenstal, R.M., Amiel, S.A., Beck, R., Biester, T., Clinical targets for continuous glucose monitoring data interpretation: recommendations from the international consensus on time in range (2019) Diabetes Care, 42 (8), pp. 1593-1603; White, I.R., Network meta-analysis (2015) STATA J, 15 (4), pp. 951-985; McGauran, N., Wieseler, B., Kreis, J., Schüler, Y.B., Kölsch, H., Kaiser, T., Reporting bias in medical research - a narrative review (2010) Trials, 11, p. 37},
correspondence_address1={Valli, G.; Neuromuscular Physiology laboratory, via F. Marzolo, 3, Italy; email: giacomo.valli@phd.unipd.it},
publisher={Elsevier B.V.},
issn={09394753},
pubmed_id={33549457},
language={English},
abbrev_source_title={Nutr. Metab. Cardiovasc. Dis.},
document_type={Article},
source={Scopus},
}

@ARTICLE{Gillies2021,
author={Gillies, K. and Kearney, A. and Keenan, C. and Treweek, S. and Hudson, J. and Brueton, V.C. and Conway, T. and Hunter, A. and Murphy, L. and Carr, P.J. and Rait, G. and Manson, P. and Aceves-Martins, M.},
title={Strategies to improve retention in randomised trials},
journal={Cochrane Database of Systematic Reviews},
year={2021},
volume={2021},
number={3},
doi={10.1002/14651858.MR000032.pub3},
art_number={MR000032},
note={cited By 6},
url={https://www.scopus.com/inward/record.uri?eid=2-s2.0-85102607252&doi=10.1002%2f14651858.MR000032.pub3&partnerID=40&md5=356ed79ab863b6a908db2a14e744c2ae},
affiliation={Health Services Research Unit, University of Aberdeen, Aberdeen,

United Kingdom; Dept. of Health Data Science, University of Liverpool, Liverpool, United Kingdom; Campbell UK & Ireland, Centre for Evidence and Social Innovation, Queen's University, Belfast, United Kingdom; Department of Adult Nursing, Florence Nightingale Faculty of Nursing Midwifery and Palliative Care, King's College, London, United Kingdom; Clinical Research Facility Galway, National University of Ireland Galway, Galway, Ireland; School of Nursing and Midwifery, National University of Ireland Galway, Galway, Ireland; Research Department of Primary Care and Population Health, University College London, London, United Kingdom; Health Services Research Unit (HSRU), University of Aberdeen, Aberdeen, United Kingdom},
abstract={Background: Poor retention of participants in randomised trials can lead to missing outcome data which can introduce bias and reduce study power, affecting the generalisability, validity and reliability of results. Many strategies are used to improve retention but few have been formally evaluated. Objectives: To quantify the effect of strategies to improve retention of participants in randomised trials and to investigate if the effect varied by trial setting. Search methods: We searched the Cochrane Central Register of Controlled Trials (CENTRAL), MEDLINE, Scopus, PsycINFO, CINAHL, Web of Science Core Collection (SCI-expanded, SSCI, CPSI-S, CPCI-SSH and ESCI) either directly with a specified search strategy or indirectly through the ORRCA database. We also searched the SWAT repository to identify ongoing or recently completed retention trials. We did our most recent searches in January 2020. Selection criteria: We included eligible randomised or quasi-randomised trials of evaluations of strategies to increase retention that were embedded in 'host' randomised trials from all disease areas and healthcare settings. We excluded studies aiming to increase treatment compliance. Data collection and analysis: We extracted data on: the retention strategy being evaluated; location of study; host trial setting; method of randomisation; numbers and proportions in each intervention and comparator group. We used a risk difference (RD) and 95% confidence interval (CI) to estimate the effectiveness of the strategies to improve retention. We assessed heterogeneity between trials. We applied GRADE to determine the certainty of the evidence within each comparison. Main results: We identified 70 eligible papers that reported data from 81 retention trials. We included 69 studies with more than 100,000 participants in the final meta-analyses, of which 67 studies evaluated interventions aimed at trial participants and two evaluated interventions aimed at trial staff involved in retention. All studies were in health care and most aimed to improve postal questionnaire response. Interventions were categorised into broad comparison groups: Data collection; Participants; Sites and site staff; Central study management; and Study design. These intervention groups consisted of 52 comparisons, none of which were supported by high-certainty evidence as determined by GRADE assessment. There were four comparisons presenting moderate-certainty evidence, three supporting retention (self-sampling kits, monetary reward together with reminder or prenotification and giving a pen at recruitment) and one reducing retention (inclusion of a diary with usual follow-up compared to usual follow-up alone). Of the remaining studies, 20 presented GRADE low-certainty evidence and 28 presented very low-certainty evidence. Our findings do provide a priority list for future replication studies, especially with regard to comparisons that currently rely on a single study. Authors' conclusions: Most of the interventions we identified aimed to improve retention in the form of postal questionnaire response. There were few evaluations of ways to improve participants returning to trial sites for trial follow-up. None of the comparisons are supported by high-certainty evidence. Comparisons in the review where the evidence certainty could be improved with

the addition of well-done studies should be the focus for future evaluations.
Copyright © 2021 The Cochrane Collaboration. Published by John Wiley & Sons, Ltd.},
keywords={Article; behavior; certification; data collection method; human; incentive; money; motivational interviewing; patient information; priority journal; publication; questionnaire; randomized controlled trial (topic); reward; study design; theory},
references={Evaluation of theory informed cover letetr comapred to standard cover letter Personal Communication, , Hagen S., x-wiley/crsRef/16631898; Arundel, C., Coleman, E., Fairhurst, C., Peckham, E., Bailey, D., Gilbody, S., The effectiveness of a contingent financial incentive to improve trial follow up; a randomised study within a trial (SWAT) (2019) F1000Research, 8, p. 1937. , x-wiley/crsRef/16406398; Ashby, R., Turner, G., Cross, B., Mitchell, N., Torgerson, D., A randomized trial of electronic reminders showed a reduction in the time to respond to postal questionnaires (2011) Journal of Clinical Epidemiology, 64, pp. 208-212. , x-wiley/crsRef/16406400; Avenell, A., Grant, A.M., McGee, M., McPherson, G., Campbell, M.K., McGee, M.A., The effects of an open design on trial participant recruitment, compliance and retention - a randomized controlled trial comparison with a blinded, placebo-controlled design (2004) Clinical Trials, 1 (6), pp. 490-498. , x-wiley/crsRef/16406402; Bailey, J., Evaluating the effectiveness of incentives in the "Sexunzipped" trial Personal communication - email, , x-wiley/crsRef/16406404; Bauer, J.E., Rezaishiraz, H., Head, K., Cowell, J., Bepler, G., Aiken, M., Obtaining DNA from a geographically dispersed cohort of current and former smokers: use of mail-based mouthwash collection and monetary incentives (2004) Nicotine and Tobacco Research, 6 (3), pp. 439-446. , x-wiley/crsRef/16406406; Bean, M.K., Thornton, L.M., Jeffers, A.J., Gow, R.W., Mazzeo, S.E., Impact of motivational interviewing on engagement in a parent-exclusive paediatric obesity intervention: randomized controlled trial of NOURISH+MI (2019) Pediatric Obesity, 14 (4). , x-wiley/crsRef/16406408; Bell, K., Clark, L., Fairhurst, C., Mitchell, N., Lenaghan, E., Blacklock, J., Enclosing a pen reduced time to response to questionnaire mailings (2016) Journal of Clinical Epidemiology, 74, pp. 144-150. , x-wiley/crsRef/16406410; Bradshaw, L.E., Montgomery, A.A., Williams, H.C., Chalmers, J.R., Haines, R.H., Two-by-two factorial randomised study within a trial (SWAT) to evaluate strategies for follow-up in a randomised prevention trial (2020) Trials., 21 (1), p. 529. , x-wiley/crsRef/16406412; Brubaker, L., Jelovsek, J.E., Lukacz, E.S., Balgobin, S., Ballard, A., Weidner, A.C., Recruitment and retention: a randomized controlled trial of video-enhanced versus standard consent processes within the E-OPTIMAL study (2019) Clinical Trials, 16 (5), pp. 481-489. , x-wiley/crsRef/16406414; Clark, L., Ronaldson, S., Dyson, L., Hewitt, C., Torgerson, D., Adamson, J., Electronic prompts significantly increase response rates to postal questionnaires: a randomized trial within a randomized trial and meta-analysis (2015) Journal of Clinical Epidemiology, 68 (12), pp. 1446-1450. , x-wiley/crsRef/16406416; Cochrane, A., Welch, C., Fairhurst, C., Cockayne, S., Torgerson, D.J., An evaluation of a personalised text message reminder compared to a standard text message on postal questionnaire response rates: an embedded randomised controlled trial (2020) F1000Research, 9, p. 154. , x-wiley/crsRef/16406418; Cockayne, S., Torgerson, D., A randomised controlled trial to assess the effectiveness of offering study results as an incentive to increase response rates to postal questionnaires (2005) BMC Medical Research Methodology, 5 (1), p. 34. , x-wiley/crsRef/3188687; Cockayne, S., Fairhurst, C., Adamson, J., Hewitt, C., Hull, R., Hicks, K., An optimised patient information sheet did not significantly increase recruitment or retention in a

falls prevention study: an embedded randomised recruitment trial (2017) Trials, 18 (1), p. 144. , x-wiley/crsRef/16406420; Cook, J., Cook, J.A., Bongard, E., Heneghan, C., SWAT 90 evaluation: Does the time at which a participant incentive is given affect the retention rate? https://www.qub.ac.uk/sites/TheNorthernIrelandNetworkforTrialsMethodologyResearch/FileStore/Filetoupload,902359,en.pdf, , Butler CC., [16406421], x-wiley/crsRef/16631899; Couper, P.M., Peytchev, A., Strecher, J.V., Rothert, K., Anderson, J., Following up non respondents to an online weight management intervention: randomized trial comparing mail versus telephone (2007) Journal of Medical Internet Research, 9 (2). , x-wiley/crsRef/16406423; Cunningham, J.A., Wild, T.C., Cordingley, J., Using collaterals to validate self-reports of problem drinkers: any impact on client attrition and quantity of drinking reported? (2004) Addictive Behaviors, 29 (3), pp. 615-621. , x-wiley/crsRef/16406425; Cunningham-Burley, R., Roche, J., Fairhurst, C., Cockayne, S., Hewitt, C., Iles-Smith, H., Enclosing a pen to improve response rate to postal questionnaire: an embedded randomised controlled trial (2020) F1000Research, 9, p. 577. , x-wiley/crsRef/16406427; Dinglas, V.D., Huang, M., Sepulveda, K.A., Pinedo, M., Hopkins, R.O., Colantuoni, E., Personalized contact strategies and predictors of time to survey completion: analysis of two sequential randomized trials (2015) BMC Medical Research Methodology, 15, p. 5. , x-wiley/crsRef/16406429; Dorling, J., Hewer, O., Hurd, M., Bari, V., Bosiak, B., Bowler, U., Two speeds of increasing milk feeds for very preterm or very low-birthweight infants: the SIFT RCT (2020) Health Technology Assessment, 24 (81), pp. 1-94. , x-wiley/crsRef/16406431; Dorman, P., Slattery, J., Farrell, B., Dennis, M.S., Sandercock, P.A., A randomised comparison of the EuroQol and Short Form-36 after stroke. United Kingdom collaborators in the International Stroke Trial (1997) BMJ, 315 (7106), p. 461. , x-wiley/crsRef/3188694; Edwards, P., Roberts, I., Sandercock, P., Frost, C., Follow-up by mail in clinical trials: does questionnaire length matter? (2004) Controlled Clinical Trials, 25 (1), pp. 31-52. , x-wiley/crsRef/16406433; Edwards, L., Salisbury, C., Horspool, K., Foster, A., Garner, K., Montgomery, A.A., Increasing follow-up questionnaire response rates in a randomized controlled trial of telehealth for depression: three embedded controlled studies (2016) Trials, 17 (1), p. 107. , x-wiley/crsRef/16406435; Fouad, M.N., Johnson, R.E., Nagy, M.C., Person, S.D., Partridge, E.E., Adherence and retention in clinical trials: a community-based approach (2014) Cancer, 120 Suppl 7, pp. 1106-1112. , x-wiley/crsRef/16406437; Gates, S., Williams, M., Withers, E., Williamson, E., Mt-Isa, S., Lamb, S., Does a monetary incentive improve the response to a postal questionnaire in a randomised controlled trial? The MINT incentive study (2009) Trials, 10 (1), p. 44. , x-wiley/crsRef/16406439; Gattellari, M., Ward, J.E., Does a deadline improve men's participation in self-administered health surveys? A randomized controlled trial in general practice (2004) Journal of Public Health, 26 (4), pp. 384-387. , x-wiley/crsRef/16406441; Glassman, A.R., Beaulieu, W.T., Stockdale, C.R., Beck, R.W., Bressler, N.M., Labriola, L.T., Effect of telephone calls from a centralized coordinating center on participant retention in a randomized clinical trial (2020) Clinical Trials, 17 (2), pp. 195-201. , x-wiley/crsRef/16406443; Goulao, B., Duncan, A., Floate, R., Clarkson, J., Ramsay, C., Three behavior change theory-informed randomized studies within a trial to improve response rates to trial postal questionnaires (2020) Journal of Clinical Epidemiology, 122, pp. 35-41. , x-wiley/crsRef/16406445; Goulao, B., Duncan, A., Floate, R., Clarkson, J., Ramsay, C., Three behavior change theory-informed randomized studies within a trial to improve response rates to trial postal questionnaires (2020) Journal of Clinical Epidemiology, 122, pp. 35-41. , x-wiley/crsRef/16406447; Greig, A., Gardiner, M.D., Sierakowski, A.,

Zweifel, C.J., Pinder, R.M., Furniss, D., Randomized feasibility trial of replacing or discarding the nail plate after nail-bed repair in children (2017) British Journal of Surgery, 104 (12), pp. 1634-1639. , x-wiley/crsRef/16406449; Griffin, J., Lall, R., Bruce, J., Withers, E., Finnegan, S., Lamb, S.E., Comparison of alternative falls data collection methods in the Prevention of Falls Injury Trial (PreFIT) (2019) Journal of Clinical Epidemiology, 106, pp. 32-40. , x-wiley/crsRef/16406451; Guarino, P., Elbourne, D., Carpenter, J., Peduzzi, P., Consumer involvement in consent document development: a multicenter cluster randomized trial to assess study participants' understanding (2006) Clinical Trials, 3 (1), pp. 19-30. , x-wiley/crsRef/16406453; Hardy, P., Bell, J.L., Brocklehurst, P., Evaluation of the effects of an offer of a monetary incentive on the rate of questionnaire return during follow-up of a clinical trial: a randomised study within a trial (2016) BMC Medical Research Methodology, 16, p. 82. , x-wiley/crsRef/16406455; Henderson, M., Wight, D., Nixon, C., Hart, G., Retaining young people in a longitudinal sexual health survey: a trial of strategies to maintain participation (2010) BMC Medical Research Methodology, 10, p. 9. , x-wiley/crsRef/16406457; James, S., Parker, A., Cockayne, S., Rodgers, S., Fairhurst, C., Torgerson, D., Including a pen and/or cover letter, containing social incentive text, had no effect on questionnaire response rate: a factorial randomised controlled Study within a Trial (2020) F1000Research, 9, p. 623. , x-wiley/crsRef/16406459; Keding, A., Brabyn, S., MacPherson, H., Richmond, S.J., Torgerson, D.J., Text message reminders to improve questionnaire response rates (2016) Journal of Clinical Epidemiology, 79, pp. 90-95. , x-wiley/crsRef/16406461; Kenton, L., Dennis, C.L., Weston, J., Kiss, A., The effect of incentives and high priority mailing on postal questionnaire response rates: a mini-RCT (2007) Abstracts from the 28th Meeting of the Society of Clinical Trials; 2007 May 20-23; Montreal, 4, pp. 371-455. , x-wiley/crsRef/16406463; Kenyon, S., Pike, K., Jones, D., Taylor, D., Salt, A., Marlow, N., The effect of a monetary incentive on return of a postal health and development questionnaire: a randomised trial (2005) BMC Health Services Research, 5 (1), p. 55. , x-wiley/crsRef/16406465; Khadjesari, Z., Murray, E., Kalaitzaki, E., White, I., Mc Cambridge, J., Thompson, S., Impact and costs of incentives to reduce attrition in online trials: two randomised controlled trials (2011) Journal of Medical Internet Research, 13 (1). , x-wiley/crsRef/16406467; Land, S.R., Ritter, M.W., Costantino, J.P., Julian, T.B., Cronin, W.M., Haile, S.R., Compliance with patient-reported outcomes in multicenter clinical trials: methodologic and practical approaches (2007) Journal of Clinical Oncology, 25 (32), pp. 5113-5120. , x-wiley/crsRef/16406469; Lewis, H., Keding, A., Bosanquet, K., Gilbody, S., Torgerson, D., An randomized controlled trial of Post-it® notes did not increase postal response rates in older depressed participants (2017) Journal of Evaluation in Clinical Practice, 23 (1), pp. 102-107. , x-wiley/crsRef/16406471; Liénard, J.L., Quinaux, E., Fabre-Guillevin, E., Piedbois, P., Jouhaud, A., Decoster, G., Impact of on-site initiation visits on patient recruitment and data quality in a randomized trial of adjuvant chemotherapy for breast cancer (2006) Clinical Trials, 3 (5), pp. 486-492. , x-wiley/crsRef/16406473; MacLennan, G., McDonald, A., McPherson, G., Treweek, S., Avenell, A., Advance telephone calls ahead of reminder questionnaires increase response rate in non-responders compared to questionnaire reminders only: The RECORD phone trial (2014) Trials, 15, p. 13. , x-wiley/crsRef/16406475; Beasley, M., MamMOTH trial Personal communication, , x-wiley/crsRef/16406477; Man, M.S., Tilbrook, H.E., Jayakody, S., Hewitt, C.E., Cox, H., Cross, B., Electronic reminders did not improve postal questionnaire

response rates or response times: a randomized controlled trial (2011) Journal of Clinical Epidemiology, 64 (1001). , x-wiley/crsRef/16406479; Marques, E., Johnson, E.C., Gooberman-Hill, R., Blom, A.W., Noble, S., Using resource use logs to reduce the amount of missing data in economic evaluations alongside trials (2013) Value Health, 16 (1), pp. 195-201. , x-wiley/crsRef/16406481; Marsh, P., Kendrick, D., Using a diary to record near misses and minor injuries?which method of administration is best? (1999) Injury Prevention, 5, pp. 305-309. , x-wiley/crsRef/16406483; Marson, A.G., A randomised controlled trial examining the longer-term outcomes of standard versus new antiepileptic drugs: the SANAD trial (2007) NIHR HTA Report, 11 (37), pp. 111-113. , x-wiley/crsRef/16406485; McCambridge, J., Kalaitzaki, E., White, R.I., Khadjesari, Z., Murray, E., Linke, S., Impact of length or relevance of questionnaires on attrition in online trials: randomized controlled trial (2011) Journal of Medical Internet Research, 13 (4). , x-wiley/crsRef/16406487; McColl, E.M., Eccles, M.P., Rousseau, N.S., Steen, I.N., Parkin, D.W., Grimshaw, J.M., From the generic to the condition-specific? Instrument order effects in quality of life assessment (2003) Medical Care, 41 (7), pp. 777-790. , x-wiley/crsRef/16406489; McColl, E.M., Eccles, M.P., Rousseau, N.S., Steen, I.N., Parkin, D.W.D., Grimshaw, J.M., From the generic to the condition-specific? Instrument order effects in quality of life assessment (2003) Medical Care, 41 (7), pp. 777-790. , x-wiley/crsRef/16406491; Mitchell, N., Hewitt, C.E., Torgerson, D.J., A controlled trial of envelope colour for increasing response rates in older women (2011) Aging Clinical and Experimental Research, 23 (3), pp. 236-240. , x-wiley/crsRef/16406493; Mitchell, N., Hewitt, C.E., Lenaghan, E., Platt, E., Shepstone, L., Torgerson, D.J., Prior notification of trial participants by newsletter increased response rates: a randomized controlled trial (2012) Journal of Clinical Epidemiology, 65 (12), pp. 1348-1352. , x-wiley/crsRef/16406495; Mitchell, A.S., Cook, L., Dean, A., Fairhurst, C., Northgraves, M., Torgerson, D., Using pens as an incentive for questionnaire return in an orthopaedic trial: an embedded randomised controlled retention trial (2020) F1000Research, 9, p. 321. , x-wiley/crsRef/16406497; Mitchell, A.S., Cook, L., Dean, A., Fairhurst, C., Northgraves, M., Togerson, D., An embedded randomised controlled retention trial of personalised text messages compared to non-personalised text messages in an orthopaedic setting (2020) F1000Research, 9, p. 591. , x-wiley/crsRef/16406499; Nakash, R.A., (2007) A Study of Response and Non-Response to Postal Questionnaire Follow-up in Clinical Trials: Chapter 6: A Randomised Controlled Trial of a Method of Improving Response to Postal Questionnaire Follow-up in a Clinical Trial [PhD thesis], , Warwick, UK, University of Warwick, Warwick Medical School, x-wiley/crsRef/3188763; Evaluation of theory informed cover letter compared to standard cover letter Personal communication, , Goodman K., x-wiley/crsRef/16631900; Renfroe, E.G., Heywood, G., Foreman, L., Schron, E., Powell, J., Baessler, C., The end-of-study patient survey: methods influencing response rate in the AVID trial (2002) Controlled Clinical Trials, 23 (5), pp. 521-533. , x-wiley/crsRef/16406502; Renfroe, E.G., Powell, J., Olarte, A., Morris, M., Hallstrom, A., Method of administering patient surveys affects response rate (1999) Controlled Clinical Trials, 20, p. s62. , x-wiley/crsRef/16406503; Rodgers, S., Sbizzera, I., Cockayne, S., Fairhurst, C., Lamb, S.E., Vernon, W., A study update newsletter or Post-it® note did not increase postal questionnaire response rates in a falls prevention trial: an embedded randomised factorial trial (2019) F1000Research, 7, p. 1083. , x-wiley/crsRef/16406505; Salvesen KA adn Vatten, L.J., Effect of a newspaper article on the response to a postal questionnaire (1992) Journal of Epidemiology and Community Health, 46 (1), p. 86. , x-wiley/crsRef/16406507;

Sarathy, P.P., Kottam, L., Parker, A., Brealey, S., Coleman, E., Keding, A., Timing of electronic reminders did not improve trial participant questionnaire response: a randomized trial and meta-analyses (2020) Journal of Clinical Epidemiology, 6. , eprint., x-wiley/crsRef/16406509; Severi, E., Free, C., Knight, R., Robertson, S., Edwards, P., Hoile, E., Two controlled trials to increase participant retention in a randomized controlled trial of mobile phone-based smoking cessation support in the United Kingdom (2011) Clinical Trials, 8 (5), pp. 654-660. , x-wiley/crsRef/16406511; Sharp, L., Cochran, C., Cotton, S.C., Gray, N.M., Gallagher, M.E., Enclosing a pen with a postal questionnaire can significantly increase the response rate (2006) Journal of Clinical Epidemiology, 59 (7), pp. 747-754. , x-wiley/crsRef/16406513; Starr, K., McPherson, G., Forrest, M., Cotton, S.C., SMS text pre-notification and delivery of reminder e-mails to increase response rates to postal questionnaires in the SUSPEND trial: a factorial design, randomised controlled trial (2015) Trials, 16, p. 295. , x-wiley/crsRef/16406515; Subar, A.F., Ziegler, R.G., Thompson, F.E., Johnson, C.C., Weissfeld, J.L., Reding, D., Is shorter always better? Relative importance of questionnaire length and cognitive ease on response rates and data quality for two dietary questionnaires (2001) American Journal of Epidemiology, 153 (4), pp. 404-409. , x-wiley/crsRef/16406517; Tai, S.S., Nazareth, I., Haines, A., Jowett, C., A randomized trial of the impact of telephone and recorded delivery reminders on the response rate to research questionnaires (1997) Journal of Public Health Medicine, 19 (2), pp. 219-221. , x-wiley/crsRef/16406519; Tilbrook, H.E., Becque, T., Buckley, H., MacPherson, H., Bailey, M., Torgerson, D.J., Randomized trial within a trial of yellow 'post-it notes' did not improve questionnaire response rates among participants in a trial of treatments for neck pain (2015) Journal of Evaluation in Clinical Practice, 21 (2), pp. 202-204. , x-wiley/crsRef/16406521; Tranberg, M., Bech, B.H., Blaakær, J., Jensen, J.S., Svanholm, H., Andersen, B., Preventing cervical cancer using HPV self-sampling: direct mailing of test-kits increases screening participation more than timely opt-in procedures - a randomized controlled trial (2018) BMC Cancer, 18 (1), p. 273. , x-wiley/crsRef/16406523; Treweek, S., Gallant, S., Anderson, A.S., SWAT 76 evaluation: randomised evaluation of sending pre-notification cards to trial participants before a face-to-face primary outcome measurement https://www.qub.ac.uk/sites/TheNorthernIrelandNetworkforTrialsMethodologyResearch/FileStore/Filetoupload,864300,en.pdf, , x-wiley/crsRef/16406525; Watson, A.J., Cook, J., Hudson, J., Kilonzo, M., Wood, J., Bruhn, H., A pragmatic multicentre randomised controlled trial comparing stapled haemorrhoidopexy with traditional excisional surgery for haemorrhoidal disease: the eTHoS study (2017) Health Technology Assessment, 21 (70), pp. 1-224. , x-wiley/crsRef/16406527; Whiteside, K., Flett, L., Mitchell, A., Fairhurst, C., Cockayne, S., Rodgers, S., Using pens as an incentive for trial recruitment of older adults: An embedded randomised controlled trial (2019) F1000Research, 8, p. 315. , x-wiley/crsRef/16406529; Young, B., Bedford, L., das Nair, R., Gallant, S., Littleford, R., Robertson, J.F., Unconditional and conditional monetary incentives to increase response to mailed questionnaires: A randomized controlled study within a trial (SWAT) (2020) Journal of Evaluation in Clinical Practice, 26 (3), pp. 893-902. , x-wiley/crsRef/16406531; Abboah-Offei, M., Bristowe, K., Vanderpuye-Donton, N.A., Ansa, G., Oppong-Agyei, Y.D., Abas, M., Phase II mixed methods' feasibility cluster randomised controlled trial of a novel community-based enhanced care intervention to improve person-centred outcomes for people living with HIV in Ghana (2020) AIDS Care, 32, pp. 107-118. , x-wiley/crsRef/16406533; Alexander, G.L., Divine, G.W., Couper, M.P.,

McClure, J.B., Stopponi, M.A., Fortman, K.K., Effect of incentives and mailing features on online health program enrollment (2008) American Journal of Preventive Medicine, 34 (5), pp. 382-388. , x-wiley/crsRef/16406535; Arnevik, E., Wilberg, T., Johansen, M., Monsen, J., Karterud, S., Psychotherapy for personality disorders: short-term day hospital psychotherapy versus outpatient individual therapy - a randomized controlled study (2009) European Psychiatry, 24, pp. 71-78. , Urnes Ø,, x-wiley/crsRef/3188809; Arundel, C., Jefferson, L., Bailey, M., Cockayne, S., Hicks, K., Loughrey, L., A randomized, embedded trial of pre?notification of trial participation did not increase recruitment rates to a falls prevention trial (2017) Journal of Evaluation in Clinical Practice, 23 (1), pp. 73-78. , x-wiley/crsRef/16406537; Atherton, H., Oakeshott, P., Aghaizu, A., Hay, P., Kerry, S., Use of an online questionnaire for follow-up of young female students recruited to a randomised controlled trial of chlamydia screening (2010) Journal of Epidemiology and Community Health, 64 (580). , x-wiley/crsRef/3188811; Aysola, J., Tahirovic, E., Troxel, A.B., Asch, D.A., Gangemi, K., Hodlofski, A.T., A randomized controlled trial of opt-In versus opt-out enrollment into a diabetes behavioral intervention (2018) American Journal of Health Promotion, 32 (3), pp. 745-752. , x-wiley/crsRef/16406539; Barry, M.J., Walker-Corkery, E., Chang, Y., Tyll, L.T., Cherkin, D.C., Fowler, F.J., Measurement of overall and disease-specific health status: does the order of questionnaires make a difference (1996) Journal of Health Services Research and Policy, 1 (1), pp. 20-27. , x-wiley/crsRef/3188813; Bednarek, P., Nichols, M., Carlson, N., Edelman, A., Creinin, M., Truitt, S., Effect of "observed start" vs. traditional "Sunday start" on hormonal contraceptive continuation rates after medical abortion (2008) Contraception, 78, pp. 26-30. , x-wiley/crsRef/3188815; Bisla, J., Ambler, G., Frank, B., Gulati, S., Hocken, P., James, M., Successful and unsuccessful recruitment and retainment strategies in a UK multicentre drug trial for a rare chronic pain condition which performed above target (2020) British Journal of Pain, 14 (3), pp. 171-179. , x-wiley/crsRef/16406541; Bowen, D., Thornquist, M., Anderson, K., Barnett, M., Valanis, B., Goodman, G., The use of incentives to increase participation in a chemoprevention trial (1995) Controlled Clinical Trials, 16, p. 41s. , x-wiley/crsRef/16406543; Bowen, D., Thornquist, M., Goodman, G., Omenn, G.S., Anderson, K., Barnett, M., Effects of incentive items on participation in a randomized chemoprevention trial (2000) Journal of Health and Psychology, 5 (1), pp. 109-115. , x-wiley/crsRef/16406544; Bromley, K.J., Ogollah, R., Konstantinou, K., Foster, N., Lewis, M., The use of regular text messaging over one year to collect primary outcome data in a randomised controlled trial (2019) Trials, 20, p. 12. , x-wiley/crsRef/16406546; Chaffin, M., Valle, L.A., Funderburk, B., Gurwitch, R., Silovsky, J., Bard, D., A motivational intervention can improve retention in PCIT for low-motivation child welfare clients (2009) Child Maltreatment, 14 (4), pp. 356-368. , x-wiley/crsRef/3188685; Chee, W., Ji, X., Kim, S., Park, S., Zhang, J., Chee, E., Recruitment and retention of Asian Americans in web-based physical activity promotion programs: a discussion paper (2019) Computers, Informatics, Nursing, 37 (9), pp. 455-462. , x-wiley/crsRef/16406548; Cheung, Y.T., Weng, X., Wang, M.P., Ho, S.Y., Kwong, A.C., Lai, V.M., Effect of prepaid and promised financial incentive on follow-up survey response in cigarette smokers: a randomized controlled trial (2019) BMC Medical Research Methodology, 19, p. 138. , x-wiley/crsRef/16406550; Cox, K.L., Burke, V., Gorely, T.J., Beilin, L.J., Puddey, I.B., Controlled comparison of retention and adherence in home- vs center-initiated exercise interventions in women ages 40?65 years: the S.W.E.A.T. study (Sedentary Women Exercise Adherence Trial) (2003) Preventive Medicine, 36, pp. 17-29. ,

x-wiley/crsRef/3188817; Cox, K.L., Burke, V., Beilin, L.J., Grove, J.R., Blanksby, B.A., Puddey, I.B., Blood pressure rise with swimming versus walking in older women: the Sedentary Women Exercise Adherence Trial 2 (SWEAT 2) (2006) Journal of Hypertension, 24 (2), pp. 307-314. , x-wiley/crsRef/16406552; Cox, K.L., Burke, V., Beilin, L.J., Derbyshire, A.J., Grove, J.R., Blanksby, B.A., Short and long-term adherence to swimming and walking programs in older women - the Sedentary Women Exercise Adherence Trial (SWEAT 2) (2008) Preventive Medicine, 46 (6), pp. 511-517. , x-wiley/crsRef/3188691; Cox, K.L., Burke, V., Beilin, L.J., Grove, J.R., Blanksby, B.A., Puddey, I.B., Blood pressure rise with swimming versus walking in older women: the Sedentary Women Exercise AdherenceTrial 2 (SWEAT 2) (2006) Journal of Hypertension, 24 (2), pp. 307-314. , x-wiley/crsRef/3188692; Day, R., Cella, D., Ganz, P., Constantino, J., Electronic monitoring of participant adherence in the NSABP breast cancer prevention trial (BCPT) (1998) Controlled Clinical Trials, 19, p. 69s. , x-wiley/crsRef/3188819; Diaz, E., Levine, H.B., Sullivan, M.C., Sernyak, M.J., Hawkins, K.A., Cramer, J.A., Use of the Medication Event Monitoring System to estimate medication compliance in patients with schizophrenia (2001) Journal of Psychiatry & Neuroscience, 26 (4), pp. 325-329. , [PMID, x-wiley/crsRef/16406554 11590972; Eaker, S., Adami, H., Granath, F., Wilander, E., Sparen, P., A large population-based randomized controlled trial to increase attendance at screening for cervical cancer (2004) Cancer Epidemiology, Biomarkers & Prevention, 13 (3), pp. 346-354. , x-wiley/crsRef/3188821; Edelstein, S., Minutillo, A., Pyle, L., Anderson, B., Wilfley, D., Van Buren, D., Rewarding youth behaviour: implementation of incentives in the TODAY trial (2005) Clinical Trials, 2, p. s23. , x-wiley/crsRef/3188823; Edwards, K.E., Hagen, S.M., Hannam, J., Kruger, C., Yu, R., Merry, A.F., A randomized comparison between records made with an anesthesia information management system and by hand, and evaluation of the Hawthorne effect (2013) Canadian Journal of Anaesthesia, 60 (10), pp. 990-997. , x-wiley/crsRef/16406556; Farabee, D., Hawken, A., Calhoun, S., Veliz, R., Grossman, J., Zhang, Y., Tracking and locating itinerant subjects with a rechargeable incentive card: results of a randomized trial (2016) Substance Use & Misuse, 51 (5), pp. 658-663. , x-wiley/crsRef/16406558; Ford, M.E., Havstad, S., Vernon, S.W., Davis, S.D., Kroll, D., Lamerato, L., Enhancing adherence among older African American men enrolled in a longitudinal cancer screening trial (2006) Gerontologist, 46 (4), pp. 545-550. , x-wiley/crsRef/3188698; Ford, M.E., Randolph, V., Hopkins-Johnson, L., Eason, S.L., Havstad, S., Jankowski, M., Design of a case management approach to enhance cancer screening trial retention among older African American men (2004) Journal of Aging and Health, 16 (5), pp. 39s-57s. , x-wiley/crsRef/3188699; Galárraga, O., Sosa-Rubí, S.G., Kuo, C., Gozalo, P., González, A., Saavedra, B., Punto Seguro: a randomized controlled pilot using conditional economic incentives to reduce sexually transmitted infection risks in Mexico (2017) AIDS and Behavior, 21 (12), pp. 3440-3456. , x-wiley/crsRef/16406560; Guarino, P., Elbourne, D., Carpenter, J., Peduzzi, P., Consumer involvement in consent document development: a multicenter cluster randomized trial to assess study participants' understanding (2006) Clinical Trials, 3 (1), pp. 19-30. , x-wiley/crsRef/16406562; Grabowski, J.P., Rhoades, H.P., Elk, R.P., Schmitz, J.P., Davis, C.P., Creson, D.M., Fluoxetine is ineffective for treatment of cocaine dependence or concurrent opiate and cocaine dependence: two placebo-controlled, double-blind trials (1995) Journal of Clinical Psychopharmacology, 15 (3), pp. 163-174. , x-wiley/crsRef/3188825; Haines, R.H., Chalmers, J.R., Swinden, R., Bradshaw, L.E., Montgomery, A.M., Williams, H.C., Maximising participant retention in a randomised prevention trial (2019)

Trials, 20 (suppl.1), pp. P-189. , x-wiley/crsRef/16406564; Hall, S., Hall, R., Borden, B., Hanson, R., Follow-up strategies in the behavioural treatment of overweight (1975) Behaviour Research and Therapy, 13, pp. 167-172. , x-wiley/crsRef/3188827; Hall, S., Bass, A., Monroe, J., Continued contact and monitoring as follow-up strategies: a long-term study of obesity treatment (1978) Addictive Behaviours, 3, pp. 139-147. , x-wiley/crsRef/3188829; Henderson, M., Waldram, M., Thomas, A., AlAlmmary, F., Di Brito, S., Ottman, S., Can financial incentives improve living donor follow-up?: a pilot randomized controlled trial (2019) Transplant International, 32 (suppl.2), p. 199. , x-wiley/crsRef/16406566; Hoang, U., Sheldenkar, A., Leon, J., Emmett, E., Mckevitt, C., Wolfe, C., The effect of personalized care on long term loss to follow-up in a stroke register: WSC-0469 (2014) International Journal of Stroke, 9, p. 262. , x-wiley/crsRef/16406568; Hoffman, S., Burke, A., Helzlsouer, K., Comstock, G., Controlled trial of the effect of length, incentives, and follow-up techniques on response to a mailed questionnaire (1998) American Journal of Epidemiology, 148 (10), pp. 1007-1011. , x-wiley/crsRef/3188831; Hopkins, K., Podolak, J., Class-of-mail and the effects of monetary gratuity on the response rates of mailed questionnaires (1983) Journal of Experimental Education, 51 (4), pp. 169-170. , x-wiley/crsRef/3188833; Hughes, J.F.R., Free reprints to increase the return of follow-up questionnaires (1989) Abstracts from the Tenth Meeting of the Society of Clinical Trials, 1989 May 15-18; Minneapolis, 10, pp. 316-354. , x-wiley/crsRef/3188703; Hunter, R.F., Murray, J.M., Gough, A., Tang, J., Patterson, C., French, D.P., Effectiveness and cost-effectiveness of a loyalty scheme for physical activity behaviour change maintenance: results from a cluster randomised controlled trial (2018) International Journal of Behavioral Nutrition and Physical Activity, 15 (1), p. 127. , x-wiley/crsRef/16406570; Iglesias, C., Torgerson, D., Does length of questionnaire matter? A randomised trial of response rates to a mailed questionnaire (2000) Journal of Health Services Research & Policy, 5 (4), pp. 219-221. , x-wiley/crsRef/3188835; Iglesias, C.P., Birks, Y.F., Torgerson, D.J., Improving the measurement of quality of life in older people: the York SF-12 (2001) QJM: an International Journal of Medicine, 94, pp. 695-698. , x-wiley/crsRef/3188837; Johnson, G., Oxman, M., Schmader, K., Levin, M., Concato, J., Williams, H., Use of an automated telephone response system for follow-up in a long-term multi-centre clinical trial (2004) Clinical Trials, 2, p. 211. , x-wiley/crsRef/3188839; Juraskova, I., Butow, P., Bonner, C., Bell, M.L., Smith, A.N., Seccombe, M., Improving decision making about clinical trial participation - a randomised controlled trial of a decision aid for women considering participation in the IBIS-II breast cancer prevention trial (2014) British Journal of Cancer, 111 (1), pp. 1-7. , x-wiley/crsRef/16406572; Karras-Jean Gilles, J., Astuto, J., Gjicali, K., Allen, L.R., Sample retention in an urban context: exploring influential factors within a longitudinal randomized evaluation (2019) American Journal of Evaluation, 40 (2), pp. 268-290. , x-wiley/crsRef/16406574; Katz, K., El-Mohandes, A., McNeely Johnson, D., Jarrett, M., Rose, A., Retention of low income mothers in a parenting intervention study (2001) Journal of Community Health, 26 (3), pp. 203-218. , x-wiley/crsRef/3188841; Kim, M., Lee, H., Kiang, P., Aronowitz, T., Sheldon, L.K., Shi, L., A Storytelling intervention in a mobile, web-based platform: a pilot randomized controlled trial to evaluate the preliminary effectiveness to promote human papillomavirus vaccination in Korean American college women (2020) Health Education & Behavior, 47 (2), pp. 258-263. , x-wiley/crsRef/16406576; Kiwanuka, N., Mpendo, J., Asiimwe, S., Ssempiira, J., Nalutaaya, A., Nambuusi, B., A randomized trial to assess retention rates using mobile phone reminders versus physical contact

tracing in a potential HIV vaccine efficacy population of fishing communities around Lake Victoria, Uganda (2018) BMC Infectious Diseases, 18, p. 591. , x-wiley/crsRef/16406578; Kramer, F.M., Jeffery, R.W., Snell, M.K., Monetary incentives to improve follow-up data collection (1986) Psychological Reports, 58 (3), pp. 739-742. , x-wiley/crsRef/16406580; Kuhlmann, T., Reips, U.D., Wienert, J., Lippke, S., Using visual analogue scales in ehealth: non-response effects in a lifestyle intervention (2016) Journal of Medical Internet Research, 18 (6). , x-wiley/crsRef/16406582; Lannin, N.A., Anderson, C., Lim, J., Paice, K., Price, C., Faux, S., Telephone follow-up was more expensive but more efficient than postal in a national stroke registry (2013) Journal of Clinical Epidemiology, 66 (8), pp. 896-902. , x-wiley/crsRef/16406584; Leidy, N.K., Legro, M., Schmier, J., Zyczynski, T., McHorney, C., Coyne, K., Does order of administration affect subject response on generic and condition-specific measures of health related quality of life? (2000) Quality of Life Research, 9, p. 337. , x-wiley/crsRef/3188843; Leigh Brown, A.P., Lawrie, H.E., Kennedy, A.D., Webb, J.A., Torgerson, D.J., Grant, A.M., Cost effectiveness of a prize draw on response to a postal questionnaire: results of a randomised trial among orthopaedic outpatients in Edinburgh (1997) Journal of Epidemiology and Community Health, 51 (4), pp. 463-464. , x-wiley/crsRef/3188733; Leighton, P.A., Brealey, S.D., Dias, J.J., Interventions to improve retention in a surgical, clinical trial: a pragmatic, stakeholder?driven approach (2018) Journal of Evidence-based Medicine, 11 (1), pp. 12-19. , x-wiley/crsRef/16406586; Litchfield, J., Freeman, J., Schou, H., Elsley, M., Fuller, R., Chubb, B., Is the future for clinical trials internet-based? A cluster randomized clinical trial (2005) Clinical Trials., 1, pp. 72-79. , x-wiley/crsRef/16406588; Malden, S., Reilly, J.J., Gibson, A.M., Bardid, F., Summerbell, C., De Craemer, M., A feasibility cluster randomised controlled trial of a preschool obesity prevention intervention: ToyBox-Scotland Pilot and Feasibility Studies, 5 (1), p. 128. , 2019 Dec 1;, x-wiley/crsRef/16406590; McAuley, E., Courneya, K., Rudolph, D., Cox, C.L., Enhancing exercise in middle aged males and females (1994) Preventive Medicine, 23, pp. 498-506. , x-wiley/crsRef/3188847; McBee, W., Clemons, T., Chew, E., SanGiovanni, J.P., A multi-level approach for retention of participants in a long-term clinical trial (2009) Clinical Trials, 6, pp. 493-524. , x-wiley/crsRef/3188849; Muñoz, R.F., Leykin, Y., Barrera, A.Z., Brown, C.H., Bunge, E.L., The impact of phone calls on follow-up rates in an online depression prevention study Internet interventions., 8, pp. 10-14. , 2017 Jun 1;, x-wiley/crsRef/16406592; Murray, J.M., French, D.P., Patterson, C.C., Kee, F., Gough, A., Tang, J., Predicting outcomes from engagement with specific components of an internet-based physical activity intervention with financial incentives: process analysis of a cluster randomized controlled trial (2019) Journal of Medical Internet Research, 21 (4). , x-wiley/crsRef/16406594; Murray, J.M., French, D.P., Kee, F., Gough, A., Tang, J., Hunter, R.F., Mechanisms of physical activity behavior change in an incentive-based intervention: mediation analysis (2020) Health Psychology, 39 (4), pp. 281-297. , x-wiley/crsRef/16406596; Nicholas, A., Bailey, J.V., Stevenson, F., Murray, E., The Sexunzipped Trial: young people?s views of participating in an online randomized controlled trial (2013) Journal of Medical Internet Research, 15 (12). , x-wiley/crsRef/16406598; Nielson, C.M., Rivelli, J.S., Fuoco, M.J., Gawlik, V.R., Jimenez, R., Petrik, A.F., Effectiveness of automated and live phone reminders after mailed-FIT outreach in a pilot randomized trial (2018) Preventive Medicine Reports, 12, pp. 210-213. , x-wiley/crsRef/16406600; Novak, L.A., Belsher, B.E., Freed, M.C., McCutchan, P.K., Liu, X., Evatt, D.P., Impact of financial reimbursement on retention rates in military clinical trial

research: a natural experiment within a multi-site randomized effectiveness trial with active duty service members (2019) Contemporary Clinical Trials Communications, 15, p. 100353. , x-wiley/crsRef/16406602; Nuzzolese, I., Montemurro, F., Attrition in metastatic breast cancer: a metric to be reported in randomised clinical trials? (2020) Lancet Oncology, 21 (1), pp. 21-24. , x-wiley/crsRef/16406604; Parker, A., Arundel, C., Beard, D., Bower, P., Brocklehurst, P., Coleman, E., PROMoting THE USE OF SWATs (PROMETHEUS): routinely embedding recruitment and retention interventions within randomised trials (2019) Trials, 20, pp. 46-47. , x-wiley/crsRef/16406606; Paul, J., Iveson, T., Midgley, R., Harkin, A., Masterton, M., Alexander, L., Choice of randomisation time-point in non-inferiority studies of reduced treatment duration: experience from the SCOT study (2011) Trials, 12, pp. 1-2. , x-wiley/crsRef/16406608; Phiri, S.C., Mudhune, S., Prust, M.L., Haimbe, P., Shakwelele, H., Chisenga, T., Impact of the Umoyo mother-infant pair model on HIV-positive mothers? social support, perceived stigma and 12-month retention of their HIV-exposed infants in PMTCT care: evidence from a cluster randomized controlled trial in Zambia (2019) Trials, 20 (1), p. 505. , x-wiley/crsRef/16406610; Pieper, D., Kotte, N., Ober, P., The effect of a voucher incentive on a survey response rate in the clinical setting: a quasi-randomized controlled trial (2018) BMC Medical Research Methodology, 18 (1), pp. 1-4. , x-wiley/crsRef/16406612; Poling, J., Oliveto, A., Petry, N., Sofuoglu, M., Gonsai, K., Gonzalez, G., Six-month trial of bupropion with contingency management for cocaine dependence in a methadone-maintained population (2006) Archives of General Psychiatry, 63, pp. 219-228. , x-wiley/crsRef/3188851; Price, A., Bryson, H., Smith, A., Mensah, F., Goldfeld, S., Processes for engaging and retaining women who are experiencing adversity in longitudinal health services research (2019) BMC health services research, 19 (1), p. 833. , x-wiley/crsRef/16406614; Puffer, S., Porthouse, J., Birks, Y., Morton, V., Torgerson, D., Increasing response rates to postal questionnaires: a randomised trial of variations in design (2004) Journal of Health Services Research and Policy, 9 (4), p. 213?7. , x-wiley/crsRef/3188853; Rhoades, H., Creson, D., Elk, R., Schmitz, J., Grabowski, J., Retention, HIV risk, and illicit drug use during treatment: methadone dose and visit frequency (1998) American Journal of Public Health, 88 (1), pp. 34-39. , x-wiley/crsRef/3188855; Roberts, P., Roberts, C., Sibbald, B., Torgerson, D., The effect of a direct payment or a lottery on questionnaire response rates: a randomised controlled trial (2000) Journal of Epidemiology and Community Health, 54, pp. 71-72. , x-wiley/crsRef/3188857; Rodgers, S., Sbizzera, I., Cockayne, S., Fairhurst, C., Lamb, S.E., Vernon, W., A study update newsletter or Post-it® note did not increase postal questionnaire response rates in a falls prevention trial: an embedded randomised factorial trial (2019) F1000Research, 7, p. 1083. , x-wiley/crsRef/16406616; Rodgers, M., Meisel, Z., Wiebe, D., Crits-Christoph, P., Rhodes, K.V., Wireless participant incentives using reloadable bank cards to increase clinical trial retention with abused women drinkers: a natural experiment (2019) Journal of Interpersonal Violence, 34 (13), pp. 2774-2796. , x-wiley/crsRef/16406618; Rolfson, O., Salomonsson, R., Dahlberg, L.E., Garellick, G., Internet-based follow-up questionnaire for measuring patient-reported outcome after total hip replacement surgery?reliability and response rate (2011) Value in Health, 14 (2), pp. 316-321. , x-wiley/crsRef/16406620; Sano, M., Egelko, S., Donohue, M., Ferris, S., Kaye, J., Hayes, T.L., Developing dementia prevention trials: baseline report of the Home-Based Assessment study (2013) Alzheimer Disease and Associated Disorders, 27 (4), pp. 356-362. , x-wiley/crsRef/16406622; Schmitz, J., Sayre, S., Stotts, A., Rothfleisch, J., Mooney, M., Medication compliance

during a smoking cessation clinical trial: a brief intervention using MEMS feedback (2005) Journal of Behavioral Medicine, 28 (2), pp. 139-147. , x-wiley/crsRef/3188859; Shulman, G.P., Buck, B.E., Gahm, G.A., Reger, G.M., Norr, A.M., Effectiveness of the intent to complete and intent to attend intervention to predict and prevent posttraumatic stress disorder treatment drop out among soldiers (2019) Journal of Traumatic Stress, 32 (5), pp. 784-790. , x-wiley/crsRef/16406624; Smeeth, L., Fletcher, A.E., Stirling, S., Nunes, M., Breeze, E., Ng, E., Randomised comparison of three methods of administering a screening questionnaire to elderly people: findings from the MRC trial of the assessment and management of older people in the community (2001) BMJ, 323 (7326), p. 1403. , x-wiley/crsRef/3188861; Smith, V., Clarke, M., Begley, C., Devane, D., SWAT-1: The effectiveness of a?site visit? intervention on recruitment rates in a multi-centre randomised trial (2015) Trials, 16 (1), p. 211. , x-wiley/crsRef/16406626; Stoner, T., Dowd, B., Carr, W., Maldonado, G., Church, T., Mandel, J., Do vouchers improve breast cancer screening rates? Results from a randomized trial (1998) Health Services Research, 33 (1), pp. 11-28. , x-wiley/crsRef/3188863; Svoboda, P., A comparison of two questionnaires for assessing outcome after head injury Personal email communication from P Edwards in April 2010, , x-wiley/crsRef/3188805; Tariq, M.B., Jones, M.H., Strnad, G., Sosic, E., Spindler, K.P., A last-ditch effort and personalized surgeon letter improves PROMs follow-up rate in sports medicine patients: a crossover randomized controlled trial (2021) Journal of Knee Surgery, 34 (2), pp. 130-136. , x-wiley/crsRef/16406628; Tassopoulos, C., The challenges of follow-up in a multi-centre randomised controlled trial (2007) Clinical Trials, 4, pp. 371-455. , x-wiley/crsRef/3188865; Trevena, L., Irwig, L., Barratt, A., Impact of privacy legislation on the number and characteristics of people who are recruited for research: a randomised controlled trial (2006) Journal of Medical Ethics, 32 (8), pp. 473-477. , x-wiley/crsRef/16406630; von Allmen, R.S., Tinner, C., Schmidli, J., Tevaearai, H.T., Dick, F., Randomized controlled comparison of cross-sectional survey approaches to optimize follow-up completeness in clinical studies (2019) PLOS One, 14 (3). , x-wiley/crsRef/16406632; Wagstaff, A., van Doorslaer, E., Burger, R., SMS nudges as a tool to reduce tuberculosis treatment delay and pretreatment loss to follow-up. A randomized controlled trial PLOS One, 146. , 2019 Jun 20;, x-wiley/crsRef/16406634; Wensing, M., Schattenberg, G., Initial nonresponders had an increased response rate after repeated questionnaire mailings (2005) Journal of Clinical Epidemiology, 58 (9), pp. 959-961. , x-wiley/crsRef/16406636; Weston, D., Parsons, V., Ntani, G., Rushton, L., Madan, I., Mixed contact methods to improve response to a postal questionnaire (2017) Occupational Medicine, 67 (4), pp. 305-307. , x-wiley/crsRef/16406638; Wood, J., Bruhn, H., Cook, J.A., McDonald, A., Norrie, J., Watson, A.J., PTU-225 Strategies to improve response rates to patient reported outcome measures in a surgical rct (2015) Gut, 64, pp. A161-A162. , x-wiley/crsRef/16406640; Wood, J., Cook, J.A., Hudson, J., McDonald, A., Bruhn, H., Watson, A.J., Do higher monetary incentives improve response rates part-way through a randomised control trial? (2017) Trials, 18, p. P65. , x-wiley/crsRef/16406642; Wu, A.W., Jacobson, D.L., Berzon, R.A., Revicki, D.A., van der Horst, C., Fichtenbaum, C.J., The effect of mode of administration on medical outcomes study health ratings and EuroQol scores in AIDS (1997) Quality of Life Research, 6 (1), pp. 3-10. , x-wiley/crsRef/3188867; Letley, L., Garratt, A., Questionnaire length and internal reliability: a randomised study (2000) Society for Social Medicine 43rd Annual Scientific Meeting; 2000 6-8 Sept; Norwich, , x-wiley/crsRef/3188735; Sutherland, H.J., Beaton, M., Mazer,

R., Kriukov, V., Boyd, N.F., A randomized trial of the total design method for the postal follow-up of women in a cancer prevention trial (1996) European Journal of Cancer Prevention, 5 (3), pp. 165-168. , [PMID, x-wiley/crsRef/16406644 8818605; O'Neill, L., Knapp, P., Doyle, S., Guinan, E., Hussey, J., SWAT 100: Patient and family co-developed participant information to improve recruitment rates, retention, and patient understanding of a randomised trial https://www.qub.ac.uk/sites/TheNorthernIrelandNetworkforTrialsMethodologyResearch/FileStore/Filetoupload,914713,en.pdf, , x-wiley/crsRef/16631901; Hynes, S., Dwyer, C., Joyce, R., SWAT 105: Effects of a patient-designed-and-informed participant information sheet versus a standard, researcher-designed information sheet on recruitment to a randomised trial https://www.qub.ac.uk/sites/TheNorthernIrelandNetworkforTrialsMethodologyResearch/FileStore/Filetoupload,926069,en.pdf, , x-wiley/crsRef/16631902; Shiely, F., SWAT 107: Effects of a multi-trial programmable animation platform on the efficiency and success of pre-screening and subsequent recruitment to a randomised trial https://www.qub.ac.uk/sites/TheNorthernIrelandNetworkforTrialsMethodologyResearch/FileStore/Filetoupload,949779,en.pdf, , x-wiley/crsRef/16631903; Parker, A., Brealey, S., Sharma, H.K., Welch, C., Keding, A., Flett, L., Hunkins, D., Witts, J., SWAT 109: The effectiveness of a text message reminder which participants can respond to, compared with a?no reply? text message on questionnaire response rates https://www.qub.ac.uk/sites/TheNorthernIrelandNetworkforTrialsMethodologyResearch/FileStore/Filetoupload,943284,en.pdf, , McDaid C., x-wiley/crsRef/16631904; Ooms, A., SWAT 110: Printing the primary outcomE on Pink PapER versus standard paper to increase participant engagement to postal questionnaires (PEPPER) https://www.qub.ac.uk/sites/TheNorthernIrelandNetworkforTrialsMethodologyResearch/FileStore/Filetoupload,949651,en.pdf, , x-wiley/crsRef/16631905; Woodford J & von Essen, L., SWAT 112: Effects on recruitment of a personalised compared with a standard study invitation letter https://www.qub.ac.uk/sites/TheNorthernIrelandNetworkforTrialsMethodologyResearch/FileStore/Filetoupload,939618,en.pdf, , x-wiley/crsRef/16631906; Arundel, C., Chetter, I., Fairhurst, C., Joshi, K., McCaffery, J., Mott, A., Wilkinson, J., SWAT 119: Effects on retention of giving trial participants a thank you card following each study visit https://www.qub.ac.uk/sites/TheNorthernIrelandNetworkforTrialsMethodologyResearch/FileStore/Filetoupload,959362,en.pdf, , x-wiley/crsRef/16631907; Dhanjal, G., SWAT 121: What are the effects on retention and follow-up of courtesy telephone calls versus postcards to trial participants following enrolment? https://www.qub.ac.uk/sites/TheNorthernIrelandNetworkforTrialsMethodologyResearch/FileStore/Filetoupload,957826,en.pdf, , x-wiley/crsRef/16631908; Agus, A., SWAT 51: Promoting group identity to improve questionnaire return rate https://www.qub.ac.uk/sites/TheNorthernIrelandNetworkforTrialsMethodologyResearch/FileStore/Filetoupload,758574,en.pdf, , x-wiley/crsRef/16631909; Anand, R., SWAT 54: Giving trial participants a thank you note or card after each study visit https://www.qub.ac.uk/sites/TheNorthernIrelandNetworkforTrialsMethodologyResearch/FileStore/Filetoupload,758633,en.pdf, , x-wiley/crsRef/16631910; Azuara-Blanco, M., SWAT 63: Does local radio and social media advertisement increase recruitment? https://www.qub.ac.uk/sites/TheNorthernIrelandNetworkforTrialsMethodologyResearch/FileStore/Filetoupload,906043,en.pdf, , x-wiley/crsRef/16631911; Backhouse, M., Torgerson, D., Parker, A., SWAT 79: Effect of birthday cards with or

without nudge on retention and data completion rates in trials involving children https://www.qub.ac.uk/sites/TheNorthernIrelandNetworkforTrialsMethodologyResearch/FileStore/Filetoupload,864304,en.pdf, , Cockayne S., x-wiley/crsRef/16631912; BenSaaud, A., Tawfick, W., Jordan, F., SWAT 81: A Telephone Reminder to Enhance Adherence to Interventions in Randomised Trials https://www.qub.ac.uk/sites/TheNorthernIrelandNetworkforTrialsMethodologyResearch/FileStore/Filetoupload,959437,en.pdf, , x-wiley/crsRef/16631913; Treweek, S., Gillies, K., Innes, K., MacLennan, G., SWAT 82: Sending Christmas cards to trial participants to improve retention https://www.qub.ac.uk/sites/TheNorthernIrelandNetworkforTrialsMethodologyResearch/FileStore/Filetoupload,846275,en.pdf, , x-wiley/crsRef/16631914; Sutton, C., Cotterill, S., D Rhodes, S., Hammond, A., SWAT 86: Advance notification of trial participants before outcome data collection to improve retention https://www.qub.ac.uk/sites/TheNorthernIrelandNetworkforTrialsMethodologyResearch/FileStore/Filetoupload,902355,en.pdf, , Forshaw,, x-wiley/crsRef/16631915; Hopewell, S., Cureton, L., Greenall, G., SWAT 87: Do participants complete the original or the reminder postal follow up questionnaire? https://www.qub.ac.uk/sites/TheNorthernIrelandNetworkforTrialsMethodologyResearch/FileStore/Filetoupload,902356,en.pdf, , x-wiley/crsRef/16631916; Starr, K., McRae, D., Gillies, K., Cooper, C., Wolfe, A., SWAT 89: Including a theoretically informed leaflet in a participant takehome pack of questionnaires to increase response rate https://www.qub.ac.uk/sites/TheNorthernIrelandNetworkforTrialsMethodologyResearch/FileStore/Filetoupload,911406,en.pdf, , x-wiley/crsRef/16631917; Tew, G., Tilbrook, H., Paul, S., Howe, L., Parker, A., SWAT 92: Pen incentive to enhance retention in a randomised trial https://www.qub.ac.uk/sites/TheNorthernIrelandNetworkforTrialsMethodologyResearch/FileStore/Filetoupload,903297,en.pdf, , Bell K., x-wiley/crsRef/16631918; kKnapp, P., SWAT 97: TRECA (TRials Engagement in Children and Adolescents) https://www.qub.ac.uk/sites/TheNorthernIrelandNetworkforTrialsMethodologyResearch/FileStore/Filetoupload,903301,en.pdf, , x-wiley/crsRef/16631919; Bensaaud, A., Gibson, I., Jones, J., Flaherty, G., Sultan, S., Tawfick, W., Jordan, F., A telephone reminder to enhance adherence to interventions in cardiovascular randomized trials: a protocol for a study within a trial (SWAT) (2020) Journal of Evidence-based Medicine, 13 (1), pp. 81-84; Booker, C., Harding, S., Benzeval, M., A systematic review of the effect of retention methods on response rates in population-based cohort studies (2011) BMC Public Health., 11, p. 249; Brunsdon, D., Biesty, L., Brocklehurst, P., Brueton, V., Devane, D., Elliott, J., What are the most important unanswered research questions in trial retention? A James Lind Alliance Priority Setting Partnership: the PRioRiTy II (Prioritising Retention in Randomised Trials) study (2019) Trials, 20 (1), p. 593; Tetzlaff, J.M., Altman, D.G., Dickersin, K., Moher, D., SPIRIT 2013: new guidance for content of clinical trial protocols (2013) Lancet, 381 (9861), pp. 91-92. , Chan A-W; Edwards, P.J., Roberts, I., Clarke, M.J., DiGuiseppi, C., Wentz, R., Kwan, I., Methods to increase response to postal and electronic questionnaires (2009) Cochrane Database of Systematic Reviews, (3). , Issue, Art. MR000008; Egger, M., Smith, G.D., Schneider, M., Minder, C., Bias in meta-analysis detected by a simple, graphical test (1997) BMJ, 315 (7109), pp. 629-634; Fewtrell, M.S., Kennedy, K., Singhal, A., Martin, R.M., Ness, A., Hadders-Algra, M., How much loss to follow-up is acceptable in long-term randomised trials and prospective studies? (2008) Archives of Disease in Childhood, 93 (6), pp. 458-461; Guyatt, G.H., Oxman, A.D., Vist, G.E., Kunz, R., Falck?Ytter, Y., Alonso?Coello, P., GRADE: an emerging consensus on rating

quality of evidence and strength of recommendations (2008) BMJ, 336, pp. 924-926; Higgins, J.P., Thompson, S.G., Deeks, J.J., Altman, D.G., Measuring inconsistency in meta-analyses (2003) BMJ, 327 (7414), pp. 557-560; Higgins, J.P., Altman, D., Chapter 8: Assessing risk of bias in included studies (2008) Cochrane Handbook of Systematic Reviews of Interventions, pp. 188-241. , Higgins JP, Green S, editors(s)., Chichester, John Wiley & Sons; Higgins, J.P.T., Deeks, J.J., Chapter 16: Special topics in statistics (2008) Cochrane Handbook for Systematic Reviews of Interventions, pp. 482-529. , Altman DG (editors)., Higgins JPT, Green S, editors(s)., Chichester, John Wiley & Sons; Hoffmann, T.C., Glasziou, P.P., Boutron, I., Milne, R., Perera, R., Moher, D., Better reporting of interventions: template for intervention description and replication (TIDieR) checklist and guide (2014) BMJ, 348, p. g1687. , doi.org/10.1136/bmj.g1687; Hollis, S., Campbell, F., What is meant by intention to treat analysis? Survey of published randomised controlled trials (1999) BMJ, 319 (7211), pp. 670-674; Madurasinghe, V.W., Eldridge, S., Guidelines for reporting embedded recruitment trials (2016) Trials, 17, p. 27. , doi.org/10.1186/s13063-015-1126-y; Moher, D., Hopewell, S., Schulz, K.F., Montori, V., Gøtzsche, P.C., Devereaux, P.J., CONSORT 2010 Explanation and Elaboration: updated guidelines for reporting parallel group randomised trials (2010) BMJ, 340, p. c869. , [PMID, 20332511; Murray, E., Khadjesari, Z., White, I.R., Kalaitzaki, E., Godfrey, C., McCambridge, J., Methodological challenges in online trials (2009) Journal of Medical Internet Research, 11 (2); PROMETHEUS https://www.york.ac.uk/healthsciences/research/trials/research/swats/prometheus/; (2012) Review Manager (RevMan), , Version 5.2. Copenhagen The Nordic Cochrane Centre, The Cochrane Collaboration; Robotham, D., Satkunanathan, S., Reynolds, J., Stahl, D., Wykes, T., Using digital notifications to improve attendance in clinic: systematic review and meta-analysis (2016) BMJ Open, 6 (10); Santesso, N., Glenton, C., Dahm, P., Garner, P., Akl, E.A., Alper, B., GRADE guidelines 26: informative statements to communicate the findings of systematic reviews of interventions (2020) Journal of Clinical Epidemiology, 119, pp. 126-135; Schulz, K.F., Grimes, D.A., Sample size slippages in randomised trials: exclusions and the lost and wayward (2002) Lancet, 359 (9308), pp. 781-785; Skea, Z.C., Newlands, R., Gillies, K., Exploring non-retention in clinical trials: a meta-ethnographic synthesis of studies reporting participant reasons for drop out (2019) BMJ Open, 9 (6); Sterne, J., Egger, M., Moher, D., Chapter 10: Addressing reporting biases (2008) Cochrane Handbook for Systematic Reviews of Interventions, pp. 297-333. , Higgins JP, Green S, editors(s)., Chichester, John Wiley & Sons; SWAT repository www.qub.ac.uk/sites/TheNorthernIrelandNetworkforTrialsMethodologyResearch/SWATSWARInformation/Repositories/SWATStore/, , Accessed on 1 December 2020; Treweek, S., Pitkethly, M., Cook, J., Fraser, C., Mitchell, E., Sullivan, F., Strategies to improve recruitment to randomised trials (2018) Cochrane Database of Systematic Reviews, (2). , Issue, Art. MR000013; Treweek, S., Bevan, S., Bower, P., Briel, M., Campbell, M., Christie, J., Trial Forge Guidance 2: How to decide if a further Study Within A Trial (SWAT) is needed (2020) Trials, 21, p. 33; Trial Forge - A systematic way to improve trial efficiency wwww.trialforge.org; Walsh, M., Srinathan, S.K., McAuley, D.F., Mrkobrada, M., Levine, O., Ribic, C., The statistical significance of randomized controlled trial results is frequently fragile: a case for a Fragility Index (2014) Journal of Clinical Epidemiology, 67 (6), pp. 622-628; Walsh, M., Sackett, D., Deveraux, P.J., When RCT participants are lost to follow up. Why even a few can matter (2015) Clinical Trials, 12, p. 537?9; Walters, S.J., Bonacho Dos Anjos Henriques-Cadby, I., Bortolami, O., Flight, L., Hind, D., Jacques, R.M.,

Recruitment and retention of participants in randomised controlled trials: a review of trials funded and published by the United Kingdom Health Technology Assessment Programme (2017) BMJ Open, 7, p. 3; Brueton, V.C., Rait, G., Tierney, J., Meredith, S., Darbyshire, J., Harding, S., Strategies to reduce attrition in randomised trials (2011) Cochrane Database of Systematic Reviews, (2). , Issue, Art. MR000032; Brueton, V.C., Tierney, J., Stenning, S., Harding, S., Meredith, S., Nazareth, I., Strategies to improve retention in randomised trials (2013) Cochrane Database of Systematic Reviews, (12). , Issue, Art. MR000032},
correspondence_address1={Gillies, K.; Health Services Research Unit, United Kingdom; email: k.gillies@abdn.ac.uk; Gillies, K.email: k.gillies@abdn.ac.uk},
publisher={John Wiley and Sons Ltd},
issn={14651858},
pubmed_id={33675536},
language={English},
abbrev_source_title={Cochrane Database Syst. Rev.},
document_type={Article},
source={Scopus},
}

@ARTICLE{Taher2021,
author={Taher, M. and Yule, C. and Bonaparte, H. and Kwiecien, S. and Collins, C. and Naylor, A. and Juraschek, S.P. and Bailey-Davis, L. and Chang, A.R.},
title={Telehealth versus self-directed lifestyle intervention to promote healthy blood pressure: A protocol for a randomised controlled trial},
journal={BMJ Open},
year={2021},
volume={11},
number={3},
doi={10.1136/bmjopen-2020-044292},
art_number={e044292},
note={cited By 0},
url={https://www.scopus.com/inward/record.uri?eid=2-s2.0-85102122247&doi=10.1136%2fbmjopen-2020-044292&partnerID=40&md5=f22fafc11a28ac9164325c5437f1aa8d},
affiliation={Kidney Health Research Institute, Geisinger, Danville, PA, United States; Center for Professionalism, Geisinger, Danville, PA, United States; Obesity Institute, Geisinger, Danville, PA, United States; Medicine, Beth Israel Deaconess Medical Center, Boston, MA, United States; Department of Population Health Sciences, Geisinger, Danville, PA, United States},
abstract={Introduction Weight loss, consumption of a Dietary Approaches to Stop Hypertension dietary pattern, reduced sodium intake and increased physical activity have been shown to lower blood pressure (BP). Use of web-based tools and telehealth to deliver lifestyle counselling could be potentially scalable solutions to improve BP through behavioural modification though limited data exists to support these approaches in clinical practice. Methods and analysis This randomised controlled trial will compare the efficacy of a telehealth versus self-directed lifestyle intervention in lowering 24-hour SBP in patients with overweight/obesity (body mass index ≥25 kg/m 2) and 24-hour SBP 120-160 mm Hg. All participants receive personalised recommendations to improve dietary quality based on a web-based Food Frequency Questionnaire, access to an online comprehensive weight management programme and a smartphone dietary app. The telehealth arm additionally includes weekly calls with registered dietitian nutritionists who use motivational interviewing. The primary outcome is change from baseline to 12 weeks in 24-hour SBP. Secondary outcomes include changes

from baseline in 24-hour diastolic BP, daytime SBP, nighttime SP, daytime diastolic BP, nighttime diastolic BP, total Healthy Eating Index-2015 score, weight, waist circumference and physical activity. Other prespecified outcomes will include change in individual components of the Healthy Eating Index-2015 score, and satisfaction with the Healthy BP research study measured on a 5-point Likert scale. Ethics and dissemination The study has been approved by the Geisinger Institutional Review Board. Results will be disseminated through peer-reviewed publications and conference presentations. Trial registration number NCT03700710. © 2021 BMJ Publishing Group. All rights reserved.},
author_keywords={cardiology; clinical trials; hypertension; nephrology; nutrition & dietetics},
references={Whelton, P.K., Carey, R.M., Aronow, W.S., 2017 ACC/AHA/AAPA/ABC/ACPM/AGS/APhA/ASH/ASPC/NMA/PCNA guideline for the prevention, detection, evaluation, and management of high blood pressure in adults: A report of the American College of Cardiology/ American Heart Association Task force on clinical practice guidelines (2017) Hypertension; Virani, S.S., Alonso, A., Benjamin, E.J., Heart disease and stroke Statistics-2020 update: A report from the American Heart Association (2020) Circulation, 141, pp. e139-e596; Sacks, F.M., Svetkey, L.P., Vollmer, W.M., Effects on blood pressure of reduced dietary sodium and the dietary approaches to stop hypertension (DASH) diet DASH-Sodium Collaborative Research Group (2001) N Engl J Med, 344, pp. 3-10; Appel, L.J., Champagne, C.M., Harsha, D.W., Effects of comprehensive lifestyle modification on blood pressure control: Main results of the premier clinical trial (2003) JAMA, 289, pp. 2083-2093; Tuomilehto, J., Jousilahti, P., Rastenyte, D., Urinary sodium excretion and cardiovascular mortality in Finland: A prospective study (2001) Lancet, 357, pp. 848-851; Muntner, P., Carey, R.M., Gidding, S., Potential U.S population impact of the 2017 ACC/AHA high blood pressure guideline (2018) J Am Coll Cardiol, 71, pp. 109-118; Berman, A.H., Kolaas, K., Petersén, E., Clinician experiences of healthy lifestyle promotion and perceptions of digital interventions as complementary tools for lifestyle behavior change in primary care (2018) BMC Fam Pract, 19, p. 139; Rubio-Valera, M., Pons-Vigués, M., Martínez-Andrés, M., Barriers and facilitators for the implementation of primary prevention and health promotion activities in primary care: A synthesis through meta-ethnography (2014) PLoS One, 9; Villinger, K., Wahl, D.R., Boeing, H., The effectiveness of app-based mobile interventions on nutrition behaviours and nutrition-related health outcomes: A systematic review and meta-analysis (2019) Obes Rev, 20, pp. 1465-1484; Appel, L.J., Clark, J.M., Yeh, H.-C., Comparative effectiveness of weight-loss interventions in clinical practice (2011) N Engl J Med, 365, pp. 1959-1968; Thomas, J.G., Bond, D.S., Raynor, H.A., Comparison of smartphone-based behavioral obesity treatment with gold standard group treatment and control: A randomized trial (2019) Obesity, 27, pp. 572-580; Tucker, K.L., Sheppard, J.P., Stevens, R., Self-monitoring of blood pressure in hypertension: A systematic review and individual patient data meta-analysis (2017) PLoS Med, 14, p. e1002389; Omboni, S., McManus, R.J., Bosworth, H.B., Evidence and recommendations on the use of telemedicine for the management of arterial hypertension: An international expert position paper (2020) Hypertension, 76, pp. 1368-1383; Miller, E.R., Erlinger, T.P., Young, D.R., Results of the diet, exercise, and weight loss intervention trial (DEW-IT) (2002) Hypertension, 40, pp. 612-618; Chang, A.R., Bailey-Davis, L., Hetherington, V., Remote dietary counseling using smartphone applications in patients with stages 1-3a chronic kidney disease: A mixed methods feasibility study (2020) J Ren Nutr, 30, pp. 53-60; Barenbaum, S.R., Mathews, S.E., Saunders, K.H., Utility of BMIQ, a novel web-based weight management programme,

at an academic weight management centre (2020) Obes Sci Pract, 6, pp. 134-138; Kristal, A.R., Kolar, A.S., Fisher, J.L., Evaluation of web-based, self-administered, graphical food frequency questionnaire (2014) J Acad Nutr Diet, 114, pp. 613-621; Carver, C., Scheier, M., (1998) On the Self Regulation of Behavior, , New York: Cambridge University Press; Lave, J., Wenger, E., (1991) Situated Learning: Legitimate Peripheral Participation, , New York: Cambridge University Press; Baena Diéz, J.M., Carrera Morodo, M., Corral Roca, M., Impact of the new criteria of the ACC/AHA on the diagnostic prevalence of hypertension (2020) Med Clin, 154, pp. 254-256; De Greeff, A., Shennan, A.H., Validation of the Spacelabs 90227 OnTrak device according to the European and British hypertension societies as well as the American protocols (2020) Blood Press Monit, 25, pp. 110-114; Craig, C.L., Marshall, A.L., Sjöström, M., International physical activity questionnaire: 12-country reliability and validity (2003) Med Sci Sports Exerc, 35, pp. 1381-1395; Reedy, J., Lerman, J.L., Krebs-Smith, S.M., Evaluation of the healthy eating Index-2015 (2018) J Acad Nutr Diet, 118, pp. 1622-1633; Griebeler, M.L., Levis, S., Beringer, L.M., Self-reported versus measured height and weight in Hispanic and non-Hispanic menopausal women (2011) J Womens Health, 20, pp. 599-604; Carranza Leon, B.G., Jensen, M.D., Hartman, J.J., Self-Measured vs Professionally measured waist circumference (2016) Ann Fam Med, 14, pp. 262-266; Sikand, G., Cole, R.E., Handu, D., Clinical and cost benefits of medical nutrition therapy by registered dietitian nutritionists for management of dyslipidemia: A systematic review and meta-analysis (2018) J Clin Lipidol, 12, pp. 1113-1122; Hardy, S.T., Loehr, L.R., Butler, K.R., Reducing the blood pressure-related burden of cardiovascular disease: Impact of achievable improvements in blood pressure prevention and control (2015) J Am Heart Assoc, 4, p. e002276; Juraschek, S.P., Miller, E.R., Weaver, C.M., Effects of sodium reduction and the DASH diet in relation to baseline blood pressure (2017) J Am Coll Cardiol, 70, pp. 2841-2848},
correspondence_address1={Chang, A.R.; Kidney Health Research Institute, United States; email: achang@geisinger.edu},
publisher={BMJ Publishing Group},
issn={20446055},
pubmed_id={33658261},
language={English},
abbrev_source_title={BMJ Open},
document_type={Article},
source={Scopus},
}

@ARTICLE{Breeman2021,
author={Breeman, L.D. and Keesman, M. and Atsma, D.E. and Chavannes, N.H. and Janssen, V. and van Gemert-Pijnen, L. and Kemps, H. and Kraaij, W. and Rauwers, F. and Reijnders, T. and Scholte op Reimer, W. and Wentzel, J. and Kraaijenhagen, R.A. and Evers, A.W.M.},
title={A multi-stakeholder approach to eHealth development: Promoting sustained healthy living among cardiovascular patients},
journal={International Journal of Medical Informatics},
year={2021},
volume={147},
doi={10.1016/j.ijmedinf.2020.104364},
art_number={104364},
note={cited By 0},
url={https://www.scopus.com/inward/record.uri?eid=2-s2.0-85098103156&doi=10.101

6%2fj.ijmedinf.2020.104364&partnerID=40&md5=915269fc4b75be510316a5632a3833c9},
affiliation={Health, Medical, and Neuropsychology Unit, Leiden University, Netherlands; Department of Cardiology, Leiden University Medical Center, Netherlands; Department of Public Health and Primary Care, Leiden University Medical Center, Netherlands; Centre for eHealth and Wellbeing Research, Department of Psychology, Health and Technology, University of Twente, Netherlands; Department of Cardiology, Máxima Medical Center Veldhoven, Netherlands; Department of Industrial Design, Eindhoven University of Technology, Netherlands; Leiden Institute for Advanced Computer Science, Leiden University, Netherlands; Department of Cardiology, Academic Medical Center, Netherlands; Faculty of Health, Amsterdam University of Applied Sciences, Netherlands; Vital10, Amsterdam, Netherlands; NDDO Institute for Prevention and Early Diagnostics (NIPED), Amsterdam, Netherlands; Healthy Society, Medial Delta, Leiden University, TU Delft, Erasmus University, Netherlands},
abstract={Background: Healthy living is key in the prevention and rehabilitation of cardiovascular disease (CVD). Yet, supporting and maintaining a healthy lifestyle is exceptionally difficult and people differ in their needs regarding optimal support for healthy lifestyle interventions. Objective: The goals of this study were threefold: to uncover stakeholders’ needs and preferences, to translate these to core values, and develop eHealth technology based on these core values. Our primary research question is: What type of eHealth application to support healthy living among people with (a high risk of) CVD would provide the greatest benefit for all stakeholders? Methods: User-centered design principles from the CeHRes roadmap for eHealth development were followed to guide the uncovering of important stakeholder values. Data were synthesized from various qualitative studies (i.e., literature studies, interviews, think-aloud sessions, focus groups) and usability tests (i.e., heuristic evaluation, cognitive walkthrough, think aloud study). We also developed an innovative application evaluation tool to perform a competitor analysis on 33 eHealth applications. Finally, to make sure to take into account all end-users needs and preferences in eHealth technology development, we created personas and a customer journey. Results: We uncovered 10 universal values to which eHealth-based initiatives to support healthy living in the context of CVD prevention and rehabilitation should adhere to (e.g., providing social support, stimulating intrinsic motivation, offering continuity of care). These values were translated to 14 desired core attributes and then prototype designs. Interestingly, we found that the primary attribute of good eHealth technology was not a single intervention principle, but rather that the technology should be in the form of a digital platform disseminating various interventions, i.e., a ‘one-stop-shop’. Conclusion: Various stakeholders in the field of cardiovascular prevention and rehabilitation may benefit most from utilizing one personalized eHealth platform that integrates a variety of evidence-based interventions, rather than a new tool. Instead of a one-size-fits-all approach, this digital platform should aid the matchmaking between patients and specific interventions based on personal characteristics and preferences. © 2020 The Author(s)},
author_keywords={Cardiovascular disease; eHealth; Health behavior; Lifestyle; Mobile application; User-centered design},
keywords={Patient treatment; User centered design, Cardiovascular disease; Cardiovascular prevention; Cognitive walkthrough; e-Health applications; Heuristic evaluation; Intrinsic motivation; Multi-stakeholder approach; Personal characteristics, eHealth, adult; Article; behavior change; cardiovascular disease; cardiovascular risk; counseling; health behavior; health care personnel; health promotion; healthy lifestyle; human; mobile

application; motivation; patient care; priority journal; prophylaxis; rehabilitation care; social support; stakeholder engagement; telehealth},
references={Wilkins, E., Wilson, L., Wickramasinghe, K., Bhatnagar, P., Leal, J., Luengo-Fernandez, R., Burns, R., Townsend, N., European Cardiovascular Disease Statistics (2017), 2017 edition ed. European Heart Network Brussels, Belgium; Piepoli, M.F., Hoes, A.W., Agewall, S., Albus, C., Brotons, C., Catapano, A.L., Cooney, M.T., Binno, S., 2016 European Guidelines on cardiovascular disease prevention in clinical practice: The Sixth Joint Task Force of the European Society of Cardiology and Other Societies on Cardiovascular Disease Prevention in Clinical Practice (constituted by representatives of 10 societies and by invited experts). Developed with the special contribution of the European Association for Cardiovascular Prevention & Rehabilitation (EACPR) (2016) Eur. Heart J., 37, pp. 2315-2381; Janssen, V., De Gucht, V., Dusseldorp, E., Maes, S., Lifestyle modification programmes for patients with coronary heart disease: a systematic review and meta-analysis of randomized controlled trials (2013) Eur. J. Prev. Cardiol., 20, pp. 620-640; Granja, C., Janssen, W., Johansen, M.A., Factors determining the success and failure of eHealth interventions: systematic review of the literature (2018) J. Med. Internet Res., 20; Thomas, J.G., Bond, D.S., Review of innovations in digital health technology to promote weight control (2014) Curr. Diab. Rep., 14; Janssen, V., De Gucht, V., van Exel, H., Maes, S., A self-regulation lifestyle program for post-cardiac rehabilitation patients has long-term effects on exercise adherence (2014) J. Behav. Med., 37, pp. 308-321; Ahern, A.L., Olson, A.D., Aston, L.M., Jebb, S.A., Weight Watchers on prescription: an observational study of weight change among adults referred to Weight Watchers by the NHS (2011) BMC Public Health, 11; Velsen, L., van, J.W., van, J.E.G.-P., Designing eHealth that matters via a multidisciplinary requirements development approach (2013) JMIR Res. Prot., 2, p. e21; Velsen, L., van, M.E., Bara, C.D., op den Akker, H., Boerema, S., Hermens, H., Understanding the acceptance of an eHealth technology in the early stages of development: an end-user walkthrough approach and two case studies (2018) JMIR Form. Res., 2; Gemert-Pijnen, J.E., van, N.N., van Limburg, M., Ossebaard, H.C., Kelders, S.M., Eysenbach, G., Seydel, E.R., A holistic framework to improve the uptake and impact of eHealth technologies (2011) J. Med. Internet Res., 13, p. e111; Kelders, S.M., van Zyl, L.E., Ludden, G.D.S., The concept and components of engagement in different domains applied to eHealth: a systematic scoping review (2020) Front. Psychol., 11, p. 926; Hennemann, S., Beutel, M.E., Zwerenz, R., Ready for eHealth? Health professionals' acceptance and adoption of eHealth interventions in inpatient routine care (2017) J. Health Commun., 22, pp. 274-284; Porter, M.E., What is value in health care? (2010) N. Engl. J. Med., 363, pp. 2477-2481; Keesman, M., Janssen, V., Kemps, H., Hollander, M., Reimer, W.S.O., Gemert-Pijnen, L.V., Hoes, A., Evers, A., BENEFIT consortium, BENEFIT for all: an ecosystem to facilitate sustained healthy living and reduce the burden of cardiovascular disease (2019) Eur. J. Prev. Cardiol., 26, pp. 606-608; Hagger, M.S., Chatzisarantis, N.L., An integrated behavior change model for physical activity (2014) Exerc. Sport Sci. Rev., 42, pp. 62-69; Marteau, T.M., Hollands, G.J., Fletcher, P.C., Changing human behavior to prevent disease: the importance of targeting automatic processes (2012) Science, 337, pp. 1492-1495; Kelders, S.M., Kok, R.N., Ossebaard, H.C., Van Gemert-Pijnen, J.E., Persuasive system design does matter: a systematic review of adherence to web-based interventions (2012) J. Med. Internet Res., 14, p. e152; Oinas-Kukkonen, H., Harjumaa, M., Persuasive Systems Design: key issues, process model, and system features (2009) Commun. Assoc. Inf. Syst., 24, pp. 485-500; Deci, E.L., Ryan, R.M., Self-Determination Theory: A macrotheory of human motivation, development, and

health (2008) Can. Psychol., 49, pp. 182-185; Cerasoli, C.P., Nicklin, J.M., Ford, M.T., Intrinsic motivation and extrinsic incentives jointly predict performance: a 40-year meta-analysis (2014) Psychol. Bull., 140, pp. 980-1008; Vliet, M.H.M., Keesman, M., Kraaijenhagen, R.A., Janssen, V.R., Evers, A.W.M., Material Incentives and Active Health Behavior Change Interventions to Promote Short-Term and Long-Term Weight Loss: A Meta-Analysis (2020), manuscript in preparation; Hsieh, H.F., Shannon, S.E., Three approaches to qualitative content analysis (2005) Qual. Health Res., 15, pp. 1277-1288; Groeneveld, R.G.A., Master Thesis: How Technology Can Be Supportive and Motivating for Patients With Chronic Heart Failure (2018), https://essay.utwente.nl/77885/, Science and Technology, University of Twente; Municipal Medical and Health Care Service, GGD AppStore (2019), https://www.ggdappstore.nl/Appstore/OverGGDappstore, Available at:; Stoyanov, S.R., Hides, L., Kavanagh, D.J., Zelenko, O., Tjondronegoro, D., Mani, M., Mobile app rating scale: a new tool for assessing the quality of health mobile apps (2015) JMIR Mhealth Uhealth, 3, p. e27; Artsenfederatie, K., Medical App Checker: A Guide to Assessing Mobile Medical Apps (2019), https://www.knmg.nl/actualiteit-opinie/nieuws/nieuwsbericht/medical-app-checker-a-guide-to-assessing-mobile-medical-apps.htm, Available at:; de Korte, E., Wiezer, N., Roozeboom, M.B., Vink, P., Kraaij, W., Behavior change techniques in mHealth apps for the mental and physical health of employees: systematic assessment (2018) J. Med. Internet Res., 20, p. 98; Achterkamp, R., Dekker-Van Weering, M.G., Evering, R.M., Tabak, M., Timmerman, J.G., Hermens, H.J., Vollenbroek-Hutten, M.M., Strategies to improve effectiveness of physical activity coaching systems: development of personas for providing tailored feedback (2018) Health Informatics J., 24, pp. 92-102; van, L.V., van, L.G.-P., Nijland, N., Beaujean, D., van, J.S., Personas: the linking pin in holistic design for eHealth (2012) The Fourth International Conference on eHealth, Telemedicine, and Social Medicine (eTELEMED) Valencia, Spain; LeRouge, C., Ma, J., Sneha, S., Tolle, K., User profiles and personas in the design and development of consumer health technologies (2013) Int. J. Med. Inform., 82, pp. e251-e268; Holden, R.J., Kulanthaivel, A., Purkayastha, S., Goggins, K.M., Kripalani, S., Know thy eHealth user: development of biopsychosocial personas from a study of older adults with heart failure (2017) Int. J. Med. Inform., 108, pp. 158-167; Wentzel, J., Sieverink, F., Groeneveld, R., Gemert-Pijnen, L., Internal Document: Results of a Cognitive Walkthrough of the Personal Health Environment (2018), University of Twente, Centre for eHealth and Wellbeing Research, Department of Psychology, Health and Technology Netherlands; Aveyard, P., Lewis, A., Tearne, S., Hood, K., Christian-Brown, A., Adab, P., Begh, R., Jebb, S.A., Screening and brief intervention for obesity in primary care: a parallel, two-arm, randomised trial (2016) Lancet, 388, pp. 2492-2500; Jaspers, M.W.M., Steen, T., van den Bos, C., Geenen, M., The think aloud method: a guide to user interface design (2004) Int. J. Med. Inform., 73, pp. 781-795; Michie, S., Ashford, S., Sniehotta, F.F., Dombrowski, S.U., Bishop, A., French, D.P., A refined taxonomy of behaviour change techniques to help people change their physical activity and healthy eating behaviours: the CALO-RE taxonomy (2011) Psychol. Health, 26, pp. 1479-1498; Hagger, M.S., Chatzisarantis, N.L., An integrated behavior change model for physical activity (2014) Exerc. Sport Sci. Rev., 42, pp. 62-69; French, D.P., Olander, E.K., Chisholm, A., Mc Sharry, J., Which behaviour change techniques are most effective at increasing older adults' self-efficacy and physical activity behaviour? A systematic review (2014) Ann. Behav. Med., 48, pp. 225-234; Shilts, M.K., Horowitz, M., Townsend, M.S., Goal setting as a strategy for dietary and physical activity behavior change: a review of the literature

(2004) Am. J. Health Promot., 19, pp. 81-93; Webb, T.L., Joseph, J., Yardley, L., Michie, S., Using the internet to promote health behavior change: a systematic review and meta-analysis of the impact of theoretical basis, use of behavior change techniques, and mode of delivery on efficacy (2010) J. Med. Internet Res., 12, p. e4; Mitchell, M.S., Orstad, S.L., Biswas, A., Oh, P.I., Jay, M., Pakosh, M.T., Faulkner, G., Financial incentives for physical activity in adults: systematic review and meta-analysis (2020) Br. J. Sports Med., 54, p. 1259; Giles, E.L., Robalino, S., McColl, E., Sniehotta, F.F., Adams, J., The effectiveness of financial incentives for health behaviour change: systematic review and meta-analysis (2014) PLoS One, 9; van Woezik, A.F.G., Braakman-Jansen, L.M.A., Kulyk, O., Siemons, L., van Gemert-Pijnen, J.E.W.C., Tackling wicked problems in infection prevention and control: a guideline for co-creation with stakeholders (2016) Antimicrob. Resist. Infect. Control, 5, p. 20; Cruz-Martinez, R.R., Wentzel, J., Asbjornsen, R.A., Noort, P.D., Niekerk, J.M., Sanderman, R., van Gemert-Pijnen, J.E.W.C., Supporting self-management of cardiovascular diseases through remote monitoring technologies: metaethnography review of frameworks, models, and theories used in research and development (2020) J. Med. Internet Res., 22; Asbjørnsen, R.A., Wentzel, J., Smedsrød, M.L., Hjelmesæth, J., Clark, M.M., van Gemert-Pijnen, J.E., Solberg-Nes, L., Design for long-term weight loss maintenance: identifying persuasive design principles and behavior change techniques supporting end-user values and needs in eHealth Interventions (2020) J. Med. Internet Res., , preprint; Beerlage-de Jong, N., van Gemert-Pijnen, L., Wentzel, J., Hendrix, R., Siemons, L., Technology to support integrated antimicrobial stewardship programs: a user centered and stakeholder driven development approach (2017) Infect. Dis. Rep., 9, p. 6829; Flores, M., Glusman, G., Brogaard, K., Price, N.D., Hood, L., P4 medicine: how systems medicine will transform the healthcare sector and society (2013) Per. Med., 10, pp. 565-576; Botti, A., Monda, A., Sustainable value co-creation and digital health: the case of trentino eHealth ecosystem (2020) Sustainability, 12, p. 5263; Harris, K.K., Zopey, M., Friedman, T.C., Metabolic effects of smoking cessation (2016) Nat. Rev. Endocrinol., 12, pp. 299-308; Barnum, C., The ‘magic number 5’: is it enough for web-testing? (2002) Inf. Des. J., 11; Holzinger, A., Errath, M., Searle, G., Thurnher, B., Slany, W., From extreme programming and usability engineering to extreme usability in software engineering education (XP+UE /spl rarr/ XU) (2005) 29th Annual International Computer Software and Applications Conference (COMPSAC'05) Edinburgh (UK), pp. 169-172},
correspondence_address1={Evers, A.W.M.; Leiden University, Pieter de la Court Building, P.O. box 9555, Netherlands; email: A.Evers@fsw.leidenuniv.nl},
publisher={Elsevier Ireland Ltd},
issn={13865056},
coden={IJMIF},
pubmed_id={33373949},
language={English},
abbrev_source_title={Int. J. Med. Informatics},
document_type={Article},
source={Scopus},
}

@ARTICLE{Balbim2021,
author={Balbim, G.M. and Marques, I.G. and Marquez, D.X. and Patel, D. and Sharp, L.K. and Kitsiou, S. and Nyenhuis, S.M.},
title={Using fitbit as an mHealth intervention tool to promote physical activity: Potential challenges and solutions},

journal={JMIR mHealth and uHealth},
year={2021},
volume={9},
number={3},
doi={10.2196/25289},
art_number={e25289},
note={cited By 0},
url={https://www.scopus.com/inward/record.uri?eid=2-s2.0-85101884227&doi=10.2196%2f25289&partnerID=40&md5=fb538240e36917623279b5cde697ea2d},
affiliation={Department of Kinesiology and Nutrition, College of Applied Health Sciences, University of Illinois at Chicago, Chicago, IL, United States; Rheumatology Division, School of Medicine, University of Sao Paulo, Sao Paulo, Brazil; Department of Medicine, College of Medicine, University of Illinois at Chicago, Chicago, IL, United States; College of Pharmacy, University of Illinois at Chicago, Chicago, IL, United States; Department of Biomedical and Health Information Sciences, College of Applied Health Sciences, University of Illinois at Chicago, Chicago, IL, United States; Division of Pulmonary, Critical Care, Sleep and Allergy, Department of Medicine, University of Illinois at Chicago, Chicago, IL, United States},
abstract={Consumer-based physical activity (PA) trackers, also known as wearables, are increasingly being used in research studies as intervention or measurement tools. One of the most popular and widely used brands of PA trackers is Fitbit. Since the release of the first Fitbit in 2009, hundreds of experimental studies have used Fitbit devices to facilitate PA self-monitoring and behavior change via goal setting and feedback tools. Fitbit’s ability to capture large volumes of PA and physiological data in real time creates enormous opportunities for researchers. At the same time, however, it introduces a number of challenges (eg, technological, operational, logistical), most of which are not sufficiently described in study publications. Currently, there are no technical reports, guidelines, nor other types of publications discussing some of these challenges and offering guidance to researchers on how to best incorporate Fitbit devices in their study design and intervention to achieve their research goals. As a result, researchers are often left alone to discover and address some of these issues during the study through “trial and error.” This paper aims to address this gap. Drawing on our cumulative experience of conducting multiple studies with various Fitbit PA trackers over the years, we present and discuss various key challenges associated with the use of Fitbit PA trackers in research studies. Difficulties with the use of Fitbit PA trackers are encountered throughout the entire research process. Challenges and solutions are categorized in 4 main categories: study preparation, intervention delivery, data collection and analysis, and study closeout. Subsequently, we describe a number of empirically tested strategies used in 4 of our interventional studies involving participants from a broad range of demographic characteristics, racial/ethnic backgrounds, and literacy levels. Researchers should be prepared to address challenges and issues in a timely fashion to ensure that the Fitbit effectively assists participants and researchers in achieving research and outcome goals. © Guilherme M Balbim, Isabela G Marques, David X Marquez, Darshilmukesh Patel, Lisa K Sharp, Spyros Kitsiou, Sharmilee M Nyenhuis.},
author_keywords={Adults; Fitbit; Fitness trackers; Intervention; Interventional studies; Older adults; Physical activity; Smartphones; Wearable},
references={Piercy, KL, Troiano, RP, Ballard, RM, Carlson, SA, Fulton, JE, Galuska, DA, The Physical Activity Guidelines for Americans (2018) JAMA, 320

(19), pp. 2020-2028. , Nov 20; [doi] [Medline: 30418471]; Clarke, TC, Schiller, JS, Norris, T., (2017) Early release of selected estimates based on data from January-June 2017 National Health Interview Survey, , https://www.cdc.gov/nchs/data/nhis/earlyrelease/earlyrelease201712.pdf, U.S. Department of Health and Human Services. Dec. [accessed 2021-02-03]; Guthold, R, Stevens, G, Riley, L, Bull, FC., Worldwide trends in insufficient physical activity from 2001 to 2016: a pooled analysis of 358 population-based surveys with 1·9 million participants (2018) The Lancet Global Health, 6 (10), pp. e1077-e1086. , Oct; [doi]; https://www.statista.com/statistics/543070/number-of-wearable-users-in-the-us/, of wearable device users in the United States from 2014 to 2022. Statista. 2020. [accessed 2021-02-03]; Emery, E, Woodhead, E, Molinari, V, Hunt, MG., Treatment adherence in late-life (2010) Handbook of Assessment in Clinical Gerontology, pp. 427-457. , Lichtenberg PA, editor. 2nd ed. Cambridge, MA: Academic Press; Arvidsson, D, Fridolfsson, J, Börjesson, M., Measurement of physical activity in clinical practice using accelerometers (2019) J Intern Med, 286 (2), pp. 137-153. , Aug 16; [doi] [Medline: 30993807]; Hickey, AM, Freedson, PS., Utility of Consumer Physical Activity Trackers as an Intervention Tool in Cardiovascular Disease Prevention and Treatment (2016) Prog Cardiovasc Dis, 58 (6), pp. 613-619. , May; [doi] [Medline: 26943981]; Gupta, L., Fitbit’s Unit Sales Shipments (2016-2021) (2020) Business Quant, , https://businessquant.com/fitbit-unit-sales-shipments, [accessed 2021-02-03]; Earwear and Wristbands Drive First Quarter Growth in the Worldwide Wearables Market, , https://www.idc.com/getdoc.jsp?containerId=prUS46432620, Says IDC. IDC. 2020 May 28. [accessed 2021-02-03]; Ringeval, M, Wagner, G, Denford, J, Paré, G, Kitsiou, S., Fitbit-Based Interventions for Healthy Lifestyle Outcomes: Systematic Review and Meta-Analysis (2020) J Med Internet Res, 22 (10), p. e23954. , Oct 12; [FREE Full _text] [doi] [Medline: 33044175]; (2021) Fitabase, , https://www.fitabase.com/research-library/, Fitbit Research Library. [accessed 2021-02-03]; Marques, IG, Kitsiou, S, Gerber, BS, Buchholz, SW, Bustamante, EE, Marquez, DX., Feasibility of a Latin Dance Program with mHealth for Middle-Aged and Older Latinxs (BAILA TECH) (2021) Translational Journal of the ACSM, 6 (1), p. e000143. , [doi]; Marques, IG, Marquez, DX., Feasibility of a mobile health-infused dance program for middle-aged and older Latinos (2018) Annals of Behavioral Medicine, 52, p. S216; Nyenhuis, SM, Balbim, GM, Dixit, D, Kitsiou, S, Sharp, LK., Physical activity monitor wear-time among low-income minority women in a randomized clinical trial Annals of Behavioral Medicine, 2020, p. S216; Nabulsi, N, Smart, M, Kuo, T, Gupta, I, Ziebart, B, Di Eugenio, B, PAM7 Developing an Automated Virtual Walking Coach for Underserved, Sedentary Patients in Primary Care: Analysis of Pilot Data (2020) Value in Health, 23, p. S11. , May;:. [doi]; Kitsiou, S., (2018) Patient-centered Mobile Health Intervention to Improve Self-care in Patients With Chronic Heart Failure (iCardia4HF), , https://clinicaltrials.gov/ct2/show/NCT03642275?term=NCT03642275&draw=2&rank=1, U.S. National Library of Medicine: ClinicalTrials.gov. [accessed 2021-02-03]; Marquez, DX, Wilbur, J, Hughes, SL, Berbaum, ML, Wilson, RS, Buchner, DM, B.A.I.L.A. - a Latin dance randomized controlled trial for older Spanish-speaking Latinos: rationale, design, and methods (2014) Contemp Clin Trials, 38 (2), pp. 397-408. , Jul; [FREE Full _text] [doi] [Medline: 24969395]; Wilbur, J, Miller, AM, Fogg, L, McDevitt, J, Castro, CM, Schoeny, ME, Randomized Clinical Trial of the Women's Lifestyle Physical Activity Program for African-American Women: 24- and 48-Week Outcomes (2016) Am J Health Promot, 30 (5), pp. 335-345. , May 13; [doi] [Medline: 27404642]; Juniper, E, Guyatt, G, Cox, F, Ferrie, P, King, D., Development and validation of the Mini

Asthma Quality of Life Questionnaire (1999) Eur Respir J, 14 (1), pp. 32-38. , Jul; [FREE Full _text] [doi] [Medline: 10489826]; Riegel, B, Dickson, V, Faulkner, KM., The Situation-Specific Theory of Heart Failure Self-Care: Revised and Updated (2016) J Cardiovasc Nurs, 31 (3), pp. 226-235. , [doi] [Medline: 25774844]; Nyenhuis, SM, Shah, N, Ma, J, Marquez, DX, Wilbur, J, Cattamanchi, A, Identifying barriers to physical activity among African American women with asthma (2019) Cogent Medicine, 6 (1), p. 1582399. , Feb 27; [doi]; Nyenhuis, S, Balbim, G, Ma, J, Marquez, D, Wilbur, J, Sharp, L, A Walking Intervention Supplemented With Mobile Health Technology in Low-Active Urban African American Women With Asthma: Proof-of-Concept Study (2020) JMIR Form Res, 4 (3), p. e13900. , Mar 11; [FREE Full _text] [doi] [Medline: 32159520]; Kitsiou, S, Nieto, A, Mishra, A, Gerber, BS, Stamos, T., Smartphone Ownership and Use of Mobile Health Technologies for Self-Management of Heart Failure (2018) 2018 Presented at: Annual Symposium of the American Medical Informatics Association, , November 3-7, San Francisco, CA; Kitsiou, S, Thomas, M, Marai, G, Maglaveras, N, Kondos, G, Arena, R, Development of an innovative mHealth platform for remote physical activity monitoring and health coaching of cardiac rehabilitation patients (2017) 2017 Presented at: 2017 IEEE EMBS International Conference on Biomedical & Health Informatics (BHI), , February 16-19, Orlando, FL. [doi]; Buchholz, SW, Wilbur, J, Halloway, S, Schoeny, M, Johnson, T, Vispute, S, Study protocol for a sequential multiple assignment randomized trial (SMART) to improve physical activity in employed women (2020) Contemp Clin Trials, 89, p. 105921. , Feb;:. [doi] [Medline: 31899371]; Prendergast, H, Lara, B, Khosla, S, Kitsiou, S, Del Rios, M, Durazo-Arvizu, R, Preliminary Data from a Randomized Controlled Trial for a Hypertension Education and Empowerment Intervention (TOUCHED) in an Urban, Academic Emergency Department: Opportunities in the era of COVID-19 (2020) Journal of the National Medical Association, 112 (5), pp. S16-S17. , Oct; [doi]; Kitsiou, S, Gerber, B, Buchholz, S, Kansal, M, Chen, J, Nawrocki, L, Development and Pilot Testing of a Tailored Text Messaging Intervention to Improve Self-efficacy and Health Beliefs About Self-care in People with Heart Failure (2020) Journal of Cardiac Failure, 26 (10), p. S89. , Oct; [doi]; (2021), https://www.fitabase.com/, Fitabase. [accessed 2021-02-03]; Arora, S, Yttri, J, Nilse, W., Privacy and Security in Mobile Health (mHealth) Research (2014) Alcohol Res, 36 (1), pp. 143-151. , [FREE Full _text] [Medline: 26259009]; (2021) Fitbit, , https://www.fitbit.com/global/us/products/find-your-fit, Find the product that's right for you. [accessed 2021-02-03]; Marques, IG, Cantoral, J, Guzman, J, Balbim, GM, Marquez, DX., Using a physical activity wearable tracker: Older Latinos' perceptions (2018) Innov Aging, 2, p. 681. , (Suppl 1); (2021) Fitbit, , https://www.fitbit.com/us/home, [accessed 2021-02-03]; Eysenbach, G., The law of attrition (2005) J Med Internet Res, 7 (1), p. e11. , Mar 31; [FREE Full _text] [doi] [Medline: 15829473]; Harrison, D, Marshall, P, Berthouze, N, Bird, J., Problems related to running longitudinal studies with commercial devices (2014), Presented at: UBICOMP '14 ADJUNCT; September 13-17, 2014; Seattle, WA. [doi]; Gupta, I, Di, EB, Ziebart, B, Liu, B, Gerber, B, Sharp, L., Modeling health coaching dialogues for behavioral goal extraction (2019) 2020 Presented at: 2019 IEEE International Conference on Bioinformatics and Biomedicine (BIBM), , November 18-21, San Diego, CA. [doi]; Troiano, RP, Berrigan, D, Dodd, KW, Mâsse, LC, Tilert, T, McDowell, M., Physical activity in the United States measured by accelerometer (2008) Med Sci Sports Exerc, 40 (1), pp. 181-188. , Jan; [doi] [Medline: 18091006]},
correspondence_address1={Kitsiou, S.; Department of Biomedical and Health Information Sciences, 1919 W Taylor St (MC 530), United States; email: skitsiou@uic.edu},

publisher={JMIR Publications Inc.},
issn={22915222},
pubmed_id={33646135},
language={English},
abbrev_source_title={JMIR mHealth uHealth},
document_type={Review},
source={Scopus},
}

@ARTICLE{Chatterjee2021,
author={Chatterjee, A. and Gerdes, M. and Prinz, A. and Martinez, S.},
title={Human coaching methodologies for automatic electronic coaching (eCoaching) as behavioral interventions with information and communication technology: Systematic review},
journal={Journal of Medical Internet Research},
year={2021},
volume={23},
number={3},
doi={10.2196/23533},
art_number={e23533},
note={cited By 0},
url={https://www.scopus.com/inward/record.uri?eid=2-s2.0-85103593582&doi=10.2196%2f23533&partnerID=40&md5=6ac6df2c746f2095bf3199e3fb7b65b6},
affiliation={Department for Information and Communication Technologies, Centre for E-Health, University of Agder, Grimstad, Norway; Department of Health and Nursing Science, Centre for E-Health, University of Agder, Grimstad, Norway},
abstract={Background: We systematically reviewed the literature on human coaching to identify different coaching processes as behavioral interventions and methods within those processes. We then reviewed how those identified coaching processes and the used methods can be utilized to improve an electronic coaching (eCoaching) process for the promotion of a healthy lifestyle with the support of information and communication technology (ICT). Objective: This study aimed to identify coaching and eCoaching processes as behavioral interventions and the methods behind these processes. Here, we mainly looked at processes (and corresponding models that describe coaching as certain processes) and the methods that were used within the different processes. Several methods will be part of multiple processes. Certain processes (or the corresponding models) will be applicable for both human coaching and eCoaching. Methods: We performed a systematic literature review to search the scientific databases EBSCOhost, Scopus, ACM, Nature, SpringerLink, IEEE Xplore, MDPI, Google Scholar, and PubMed for publications that included personal coaching (from 2000 to 2019) and persuasive eCoaching as behavioral interventions for a healthy lifestyle (from 2014 to 2019). The PRISMA (Preferred Reporting Items for Systematic Reviews and Meta-Analyses) framework was used for the evidence-based systematic review and meta-analysis. Results: The systematic search resulted in 79 publications, including 72 papers and seven books. Of these, 53 were related to behavioral interventions by eCoaching and the remaining 26 were related to human coaching. The most utilized persuasive eCoaching methods were personalization (n=19), interaction and cocreation (n=17), technology adoption for behavior change (n= 17), goal setting and evaluation (n=16), persuasion (n=15), automation (n=14), and lifestyle change (n=14). The most relevant methods for human coaching were behavior (n=23), methodology (n=10), psychology (n=9), and mentoring (n=6). Here, "n" signifies the total number of articles where the respective method

was identified. In this study, we focused on different coaching methods to understand the psychology, behavioral science, coaching philosophy, and essential coaching processes for effective coaching. We have discussed how we can integrate the obtained knowledge into the eCoaching process for healthy lifestyle management using ICT. We identified that knowledge, coaching skills, observation, interaction, ethics, trust, efficacy study, coaching experience, pragmatism, intervention, goal setting, and evaluation of coaching processes are relevant for eCoaching. Conclusions: This systematic literature review selected processes, associated methods, strengths, and limitations for behavioral interventions from established coaching models. The identified methods of coaching point toward integrating human psychology in eCoaching to develop effective intervention plans for healthy lifestyle management and overcome the existing limitations of human coaching. © 2021 Journal of Medical Internet Research. All rights reserved.},
author_keywords={Coaching; Electronic coaching; Healthy lifestyle; Human behavior; Persuasive technology},
keywords={adoption; automation; behavior change; behavioral science; ethics; healthy lifestyle; human; lifestyle modification; Medline; mentoring; persuasive communication; pragmatism; Preferred Reporting Items for Systematic Reviews and Meta-Analyses; review; Scopus; skill; SpringerLink; systematic review; trust},
references={Bartlett, JE., Advances in coaching practices: A humanistic approach to coach and client roles (2007) Journal of Business Research, 60 (1), pp. 91-93. , Jan; [doi]; Flaherty, J., Coaching: Evoking Excellence in Others (2nd edition) (2006) Dev and Learning in Org, 20 (6). , Nov; [doi]; Cox, E., (2013) Coaching Understood: A Pragmatic Inquiry into the Coaching Process, , Thousand Oaks, CA, USA: SAGE Publications Ltd; Stober, D, Grant, A., (2010) Evidence Based Coaching Handbook: Putting Best Practices to Work for Your Clients, , Hoboken, NJ, USA: John Wiley & Sons; Côté, J., The Development of Coaching Knowledge (2016) International Journal of Sports Science & Coaching, 1 (3), pp. 217-222. , Nov 07; [doi]; Murphy, SA., Recourse to Executive Coaching: The Mediating Role of Human Resources (2016) International Journal of Police Science & Management, 7 (3), pp. 175-186. , Jul 31; [doi]; Green, LS, Oades, LG, Grant, AM., Cognitive-behavioral, solution-focused life coaching: Enhancing goal striving, well-being, and hope (2006) The Journal of Positive Psychology, 1 (3), pp. 142-149. , Jul; [doi]; Whitten, H., (2011) Cognitive Behavioural Coaching Techniques For Dummies, , Hoboken, NJ, USA: John Wiley & Sons; Cunningham, GB, Sagas, M., People make the difference: The influence of the coaching staff's human capital and diversity on team performance (2004) European Sport Management Quarterly, 4 (1), pp. 3-21. , Mar; [doi]; Grant, A, Palmer, S., Integrating positive psychology and coaching psychology into counselling psychology (2015) Counselling Psychology Review, 30 (3), pp. 22-25. , Sep; [FREE Full text]; Rutten, GM, Meis, JJM, Hendriks, MRC, Hamers, FJM, Veenhof, C, Kremers, SPJ., The contribution of lifestyle coaching of overweight patients in primary care to more autonomous motivation for physical activity and healthy dietary behaviour: results of a longitudinal study (2014) Int J Behav Nutr Phys Act, 11, p. 86. , Jul 16;: [FREE Full text] [doi] [Medline: 25027848]; Brötz, D., Exercise, Physical Activity, and Self-Determination Theory: A Systematic Review (2013) physioscience, 9, pp. 37-37. , Feb 26;(01): [FREE Full text] [doi]; Venditti, EM, Wylie-Rosett, J, Delahanty, LM, Mele, L, Hoskin, MA, Edelstein, SL, Diabetes Prevention Program Research Group. Short and long-term lifestyle coaching approaches used to address diverse participant barriers to weight loss and physical activity adherence (2014) Int J Behav Nutr Phys Act, 11, p. 16. , Feb 12;: [FREE Full text] [doi] [Medline: 24521153];

Gillison, F, Stathi, A, Reddy, P, Perry, R, Taylor, G, Bennett, P, Processes of behavior change and weight loss in a theory-based weight loss intervention program: a test of the process model for lifestyle behavior change (2015) Int J Behav Nutr Phys Act, 12, p. 2. , Jan 16;: [FREE Full text] [doi] [Medline: 25592314]; Lakerveld, J, Bot, SD, Chinapaw, MJ, van Tulder, MW, Kostense, PJ, Dekker, JM, Motivational interviewing and problem solving treatment to reduce type 2 diabetes and cardiovascular disease risk in real life: a randomized controlled trial (2013) Int J Behav Nutr Phys Act, 10, p. 47. , Apr 19;: [FREE Full text] [doi] [Medline: 23597082]; Standing, R, Maulder, P., The Effectiveness of Progressive and Traditional Coaching Strategies to Improve Sprint and Jump Performance Across Varying Levels of Maturation within a General Youth Population (2019) Sports (Basel), 7 (8), p. 186. , Jul 30; [FREE Full text] [doi] [Medline: 31366104]; Castro Sweet, CM, Chiguluri, V, Gumpina, R, Abbott, P, Madero, EN, Payne, M, Outcomes of a Digital Health Program With Human Coaching for Diabetes Risk Reduction in a Medicare Population (2018) J Aging Health, 30 (5), pp. 692-710. , Jun 01; [FREE Full text] [doi] [Medline: 28553807]; den Braber, N, Vollenbroek-Hutten, MMR, Oosterwijk, MM, Gant, CM, Hagedoorn, IJM, van Beijnum, BF, Requirements of an Application to Monitor Diet, Physical Activity and Glucose Values in Patients with Type 2 Diabetes: The Diameter (2019) Nutrients, 11 (2), p. 409. , Feb 15; [FREE Full text] [doi] [Medline: 30781348]; https://www.who.int/ehealth/en/, Digital health. World Health Organization. [accessed 2020-07-17]; Chatterjee, A, Gerdes, MW, Martinez, SG., Identification of Risk Factors Associated with Obesity and Overweight-A Machine Learning Overview (2020) Sensors (Basel), 20 (9), p. 2734. , May 11; [FREE Full text] [doi] [Medline: 32403349]; Aranki, D, Peh, GX, Kurillo, G, Bajcsy, R., The Feasibility and Usability of RunningCoach: A Remote Coaching System for Long-Distance Runners (2018) Sensors (Basel), 18 (1), p. 175. , Jan 10; [FREE Full text] [doi] [Medline: 29320436]; Chatterjee, A, Gerdes, M, Martinez, S., eHealth Initiatives for The Promotion of Healthy Lifestyle and Allied Implementation Difficulties 2019 Oct Presented at: 2019 International Conference on Wireless and Mobile Computing, Networking and Communications (WiMob), , October 21-23, 2019; Barcelona, Spain. [doi]; Petsani, D, Kostantinidis, E, Bamidis, P., Designing an E-coaching System for Older People to Increase Adherence to Exergame-based Physical Activity (2018) Proceedings of the 4th International Conference on Information and Communication Technologies for Ageing Well and e-Health. 2018 Presented at: 4th International Conference on Information and Communication Technologies for Ageing Well and e-Health, pp. 258-263. , Portugal [doi]; Benítez-Guijarro, A, Ruiz-Zafra, Á, Callejas, Z, Medina-Medina, N, Benghazi, K, Noguera, M., General Architecture for Development of Virtual Coaches for Healthy Habits Monitoring and Encouragement (2018) Sensors (Basel), 19 (1), p. 108. , Dec 30; [FREE Full text] [doi] [Medline: 30598011]; Montenegro, C, López Zorrilla, A, Mikel Olaso, J, Santana, R, Justo, R, Lozano, JA, A Dialogue-Act Taxonomy for a Virtual Coach Designed to Improve the Life of Elderly (2019) MTI, 3 (3), p. 52. , Jul 11; [doi]; Hansel, B, Giral, P, Gambotti, L, Lafourcade, A, Peres, G, Filipecki, C, A Fully Automated Web-Based Program Improves Lifestyle Habits and HbA1c in Patients With Type 2 Diabetes and Abdominal Obesity: Randomized Trial of Patient E-Coaching Nutritional Support (The ANODE Study) (2017) J Med Internet Res, 19 (11), p. e360. , Nov 08; [FREE Full text] [doi] [Medline: 29117929]; Beun, RJ, Fitrianie, S, Griffioen-Both, F, Spruit, S, Horsch, C, Lancee, J, Talk and Tools: the best of both worlds in mobile user interfaces for E-coaching (2017) Pers Ubiquit Comput, 21 (4), pp. 661-674. , May 19; [doi]; Boratto, L, Carta, S, Mulas, F, Pilloni, P., An e-coaching ecosystem: design and effectiveness analysis of the engagement of remote coaching on

athletes (2017) Pers Ubiquit Comput, 21 (4), pp. 689-704. , May 23; [doi]; Kamphorst, B, Kalis, A., Why option generation matters for the design of autonomous e-coaching systems (2014) AI & Soc, 30 (1), pp. 77-88. , Jan 17; [doi]; Anaby, D, Mercerat, C, Tremblay, S., Enhancing Youth Participation Using the PREP Intervention: Parents' Perspectives (2017) Int J Environ Res Public Health, 14 (9), p. 1005. , Sep 02; [FREE Full text] [doi] [Medline: 28869491]; Lim, S, Oh, H, Kim, J, Lee, J, Park, J., LSTM-Guided Coaching Assistant for Table Tennis Practice (2018) Sensors (Basel), 18 (12), p. 4112. , Nov 23; [FREE Full text] [doi] [Medline: 30477175]; Klaassen, R, Bul, KCM, Op den Akker, R, van der Burg, GJ, Kato, PM, Di Bitonto, P., Design and Evaluation of a Pervasive Coaching and Gamification Platform for Young Diabetes Patients (2018) Sensors (Basel), 18 (2), p. 402. , Jan 30; [FREE Full text] [doi] [Medline: 29385750]; Nooitgedagt, A, Beun, R, Dignum, F., e-Coaching for Intensive Cardiac Rehabilitation (2017) Persuasive Technology: Development and Implementation of Personalized Technologies to Change Attitudes and Behaviors. PERSUASIVE 2017. Lecture Notes in Computer Science, 10171, pp. 31-42. , de Vries P, Oinas-Kukkonen H, Siemons L, Beerlage-de Jong N, van Gemert-Pijnen L, editors. Cham: Springer; Beun, R, Brinkman, W, Fitrianie, S., Improving Adherence in Automated e-Coaching (2016) Persuasive Technology. PERSUASIVE 2016. Lecture Notes in Computer Science, 9638, pp. 276-287. , Meschtscherjakov A, De Ruyter B, Fuchsberger Murer M, Tscheligi M, editors. Cham: Springer; Pratiwi, P, Tjondronegoro, D., Towards personalisation of physical activity e-coach using stage-matched behaviour change and motivational interviewing strategies (2017) 2017 Presented at: 2017 IEEE Life Sciences Conference (LSC), pp. 5-8. , December 13-15, Sydney, Australia [doi]; Nápoles, AM, Santoyo-Olsson, J, Chacón, L, Stewart, AL, Dixit, N, Ortiz, C., Feasibility of a Mobile Phone App and Telephone Coaching Survivorship Care Planning Program Among Spanish-Speaking Breast Cancer Survivors (2019) JMIR Cancer, 5 (2), p. e13543. , Jul 09; [FREE Full text] [doi] [Medline: 31290395]; Partridge, SR, Allman-Farinelli, M, McGeechan, K, Balestracci, K, Wong, ATY, Hebden, L, Process evaluation of TXT2BFiT: a multi-component mHealth randomised controlled trial to prevent weight gain in young adults (2016) Int J Behav Nutr Phys Act, 13 (1), p. 7. , Jan 19; [FREE Full text] [doi] [Medline: 26785637]; Middelweerd, A, van der Laan, DM, van Stralen, MM, Mollee, JS, Stuij, M, te Velde, SJ, What features do Dutch university students prefer in a smartphone application for promotion of physical activity? A qualitative approach (2015) Int J Behav Nutr Phys Act, 12, p. 31. , Mar 01;: [FREE Full text] [doi] [Medline: 25889577]; Partridge, SR, McGeechan, K, Bauman, A, Phongsavan, P, Allman-Farinelli, M., Improved eating behaviours mediate weight gain prevention of young adults: moderation and mediation results of a randomised controlled trial of TXT2BFiT, mHealth program (2016) Int J Behav Nutr Phys Act, 13, p. 44. , Apr 02;: [FREE Full text] [doi] [Medline: 27039178]; Müller, AM, Khoo, S., Non-face-to-face physical activity interventions in older adults: a systematic review (2014) Int J Behav Nutr Phys Act, 11 (1), p. 35. , Mar 10; [FREE Full text] [doi] [Medline: 24612748]; Schoeppe, S, Alley, S, Van Lippevelde, W, Bray, NA, Williams, SL, Duncan, MJ, Efficacy of interventions that use apps to improve diet, physical activity and sedentary behaviour: a systematic review (2016) Int J Behav Nutr Phys Act, 13 (1), p. 127. , Dec 07; [FREE Full text] [doi] [Medline: 27927218]; Greaves, C, Gillison, F, Stathi, A, Bennett, P, Reddy, P, Dunbar, J, Waste the waist: a pilot randomised controlled trial of a primary care based intervention to support lifestyle change in people with high cardiovascular risk (2015) Int J Behav Nutr Phys Act, 12, p. 1. , Jan 16;: [FREE Full text] [doi] [Medline: 25592201]; Stephenson, A, McDonough, SM, Murphy, MH, Nugent, CD, Mair, JL., Using computer, mobile and wearable

technology enhanced interventions to reduce sedentary behaviour: a systematic review and meta-analysis (2017) Int J Behav Nutr Phys Act, 14 (1), p. 105. , Aug 11; [FREE Full text] [doi] [Medline: 28800736]; Michaelides, A, Major, J, Pienkosz, E, Wood, M, Kim, Y, Toro-Ramos, T., Usefulness of a Novel Mobile Diabetes Prevention Program Delivery Platform With Human Coaching: 65-Week Observational Follow-Up (2018) JMIR Mhealth Uhealth, 6 (5), p. e93. , May 03; [FREE Full text] [doi] [Medline: 29724709]; Brandt, CJ, Søgaard, GI, Clemensen, J, Søndergaard, J, Nielsen, JB., Determinants of Successful eHealth Coaching for Consumer Lifestyle Changes: Qualitative Interview Study Among Health Care Professionals (2018) J Med Internet Res, 20 (7), p. e237. , Jul 05; [FREE Full text] [doi] [Medline: 29980496]; Mao, AY, Chen, C, Magana, C, Caballero Barajas, K, Olayiwola, JN., A Mobile Phone-Based Health Coaching Intervention for Weight Loss and Blood Pressure Reduction in a National Payer Population: A Retrospective Study (2017) JMIR Mhealth Uhealth, 5 (6), p. e80. , Jun 08; [FREE Full text] [doi] [Medline: 28596147]; Karhula, T, Vuorinen, A, Rääpysjärvi, K, Pakanen, M, Itkonen, P, Tepponen, M, Telemonitoring and Mobile Phone-Based Health Coaching Among Finnish Diabetic and Heart Disease Patients: Randomized Controlled Trial (2015) J Med Internet Res, 17 (6), p. e153. , Jun 17; [FREE Full text] [doi] [Medline: 26084979]; Wayne, N, Perez, DF, Kaplan, DM, Ritvo, P., Health Coaching Reduces HbA1c in Type 2 Diabetic Patients From a Lower-Socioeconomic Status Community: A Randomized Controlled Trial (2015) J Med Internet Res, 17 (10), p. e224. , Oct 05; [FREE Full text] [doi] [Medline: 26441467]; Modave, F, Bian, J, Leavitt, T, Bromwell, J, Harris Iii, C, Vincent, H., Low Quality of Free Coaching Apps With Respect to the American College of Sports Medicine Guidelines: A Review of Current Mobile Apps (2015) JMIR Mhealth Uhealth, 3 (3), p. e77. , Jul 24; [FREE Full text] [doi] [Medline: 26209109]; van der Weegen, S, Verwey, R, Spreeuwenberg, M, Tange, H, van der Weijden, T, de Witte, L., It's LiFe! Mobile and Web-Based Monitoring and Feedback Tool Embedded in Primary Care Increases Physical Activity: A Cluster Randomized Controlled Trial (2015) J Med Internet Res, 17 (7), p. e184. , Jul 24; [FREE Full text] [doi] [Medline: 26209025]; Verwey, R, van der Weegen, S, Spreeuwenberg, M, Tange, H, van der Weijden, T, de Witte, L., A monitoring and feedback tool embedded in a counselling protocol to increase physical activity of patients with COPD or type 2 diabetes in primary care: study protocol of a three-arm cluster randomised controlled trial (2014) BMC Fam Pract, 15, p. 93. , May 12;: [FREE Full text] [doi] [Medline: 24885096]; Karppinen, P, Oinas-Kukkonen, H, Alahäivälä, T, Jokelainen, T, Keränen, AM, Salonurmi, T, Persuasive user experiences of a health Behavior Change Support System: A 12-month study for prevention of metabolic syndrome (2016) Int J Med Inform, 96, pp. 51-61. , Dec;: [doi] [Medline: 26992482]; Karppinen, P, Oinas-Kukkonen, H, Alahäivälä, T, Jokelainen, T, Teeriniemi, A, Salonurmi, T, Opportunities and challenges of behavior change support systems for enhancing habit formation: A qualitative study (2018) J Biomed Inform, 84, pp. 82-92. , Aug;: [FREE Full text] [doi] [Medline: 29936091]; Teeriniemi, A, Salonurmi, T, Jokelainen, T, Vähänikkilä, H, Alahäivälä, T, Karppinen, P, A randomized clinical trial of the effectiveness of a Web-based health behaviour change support system and group lifestyle counselling on body weight loss in overweight and obese subjects: 2-year outcomes (2018) J Intern Med, 284 (5), pp. 534-545. , Nov 04; [FREE Full text] [doi] [Medline: 29974563]; Kamphorst, BA., E-coaching systems (2017) Pers Ubiquit Comput, 21 (4), pp. 625-632. , May 23; [doi]; Lentferink, AJ, Oldenhuis, HK, de Groot, M, Polstra, L, Velthuijsen, H, van Gemert-Pijnen, JE., Key Components in eHealth Interventions Combining Self-Tracking and Persuasive eCoaching to Promote a Healthier Lifestyle: A Scoping Review (2017) J Med Internet Res, 19 (8), p. e277. , Aug 01; [FREE Full text] [doi] [Medline:

28765103]; Ochoa, SF, Gutierrez, FJ., Architecting E-Coaching Systems: A First Step for Dealing with Their Intrinsic Design Complexity (2018) Computer, 51 (3), pp. 16-23. , Mar; [doi]; Fischer, X, Donath, L, Zwygart, K, Gerber, M, Faude, O, Zahner, L., Coaching and Prompting for Remote Physical Activity Promotion: Study Protocol of a Three-Arm Randomized Controlled Trial (Movingcall) (2019) Int J Environ Res Public Health, 16 (3), p. 331. , Jan 25; [FREE Full text] [doi] [Medline: 30691013]; Pascal, A, Sass, M, Gregory, JB., I'm only human: The role of technology in coaching (2015) Consulting Psychology Journal: Practice and Research, 67 (2), pp. 100-109. , [doi]; van Berkel, J, Boot, CRL, Proper, KI, Bongers, PM, van der Beek, AJ., Effectiveness of a worksite mindfulness-related multi-component health promotion intervention on work engagement and mental health: results of a randomized controlled trial (2014) PLoS One, 9 (1), p. e84118. , [FREE Full text] [doi] [Medline: 24489648]; Gerdes, M, Martinez, S, Tjondronegoro, D., Conceptualization of a personalized ecoach for wellness promotion Proceedings of the 11th EAI International Conference on Pervasive Computing Technologies for Healthcare - PervasiveHealth '17. 2017 Presented at: 11th EAI International Conference on Pervasive Computing Technologies for Healthcare, pp. 365-374. , May 2017; Barcelona, Spain [doi]; Rutjes, H, Willemsen, MC, Kersten - van Dijk, ET, de Ruyter, B, IJsselsteijn, WA., Better together: opportunities for technology in health coaching from the coach's perspective (2017) 2017 Presented at: 2017 ECHW Conference, , January 26-27, Amsterdam, Netherlands; Kamphorst, BA, Klein, MCA, Van Wissen, A., Autonomous e-coaching in the wild: empirical validation of a model-based reasoning system AAMAS '14: Proceedings of the 2014 international conference on Autonomous agents and multi-agent systems. 2014 Presented at: 2014 international conference on Autonomous agents and multi-agent systems, pp. 725-732. , May 2014; Paris, France; Moher, D, Liberati, A, Tetzlaff, J, Altman, DG, Preferred reporting items for systematic reviews and meta-analyses: the PRISMA statement (2009) PLoS Med, 6 (7), p. e1000097. , PRISMA Group. Jul 21; [FREE Full text] [doi] [Medline: 19621072]; Wendler, R., The maturity of maturity model research: A systematic mapping study (2012) Information and Software Technology, 54 (12), pp. 1317-1339. , Dec; [doi]; Potrac, P, Brewer, C, Jones, R, Armour, K, Hoff, J., Toward an Holistic Understanding of the Coaching Process (2000) Quest, 52 (2), pp. 186-199. , May; [doi]; Richards, B., Intelligent coaching: Unleashing human potential (2003) The Journal for Quality and Participation, 26 (1), pp. 13-16. , [FREE Full text]; Knight, J., Instructional Coaching for Implementing Visible Learning: A Model for Translating Research into Practice (2019) Education Sciences, 9 (2), p. 101. , May 10; [doi]; Szymanska, K., Anxiety and the coaching relationship: How to recognise the signs and what to do next (2007) The Coaching Psychologist, 3 (2), pp. 85-89. , [FREE Full text]; Beun, R, Griffioen-both, F, Ahn, R, Fitrianie, S, Lancee, J., Modeling interaction in automated e-coaching a case from insomnia therapy (2014) COGNITIVE 2014: The Sixth International Conference on Advanced Cognitive Technologies and Applications, , Wilmington, DE: IARIA; Cavanagh, M, Spence, G., Mindfulness in Coaching (2012) The Wiley-Blackwell Handbook of the Psychology of Coaching and Mentoring, pp. 112-134. , Hoboken, NJ, USA: Wiley; Roša, Lace, N., The Open Innovation Model of Coaching Interaction in Organisations for Sustainable Performance within the Life Cycle (2018) Sustainability, 10 (10), p. 3516. , (Rosha) A Sep 30; [doi]; Guyenet, SJ., (2017) The Hungry Brain: Outsmarting the Instincts That Make Us Overeat, , New York, NY, USA: Macmillan; Kovalchuck, V, Vorotnykova, I., E-Coaching, E-Mentoring for Lifelong Professional Development of Teachers within the System of Post-Graduate Pedagogical Education (2017) Turkish Online Journal of Distance Education, p. 214. , [doi]; Shneiderman, B, Plaisant, C, Cohen, M,

Jacobs, S, Elmqvist, N, Diakopoulos, N., (2016) Designing the User Interface: Strategies for Effective Human-Computer Interaction, , London, United Kingdom: Pearson; Baber, C, Khattab, A, Russell, M, Hermsdörfer, J, Wing, A., Creating Affording Situations: Coaching through Animate Objects (2017) Sensors (Basel), 17 (10), p. 2308. , Oct 11; [FREE Full text] [doi] [Medline: 29019939]; Jablonski, RA, Winstead, V, Geldmacher, DS., Description of Process and Content of Online Dementia Coaching for Family Caregivers of Persons with Dementia (2019) Healthcare (Basel), 7 (1), p. 13. , Jan 19; [FREE Full text] [doi] [Medline: 30669444]},
correspondence_address1={Chatterjee, A.; Department for Information and Communication Technologies, Norway; email: ayan.chatterjee@uia.no},
publisher={JMIR Publications Inc.},
issn={14388871},
pubmed_id={33759793},
language={English},
abbrev_source_title={J. Med. Internet Res.},
document_type={Review},
source={Scopus},
}

@ARTICLE{Yen2021791,
author={Yen, H.-Y.},
title={Smart wearable devices as a psychological intervention for healthy lifestyle and quality of life: a randomized controlled trial},
journal={Quality of Life Research},
year={2021},
volume={30},
number={3},
pages={791-802},
doi={10.1007/s11136-020-02680-6},
note={cited By 0},
url={https://www.scopus.com/inward/record.uri?eid=2-s2.0-85093937356&doi=10.1007%2fs11136-020-02680-6&partnerID=40&md5=850edcbd676bd622b602bd2fabb772aa},
affiliation={School of Gerontology Health Management, College of Nursing, Taipei Medical University, 250 Wuxing St., Taipei, 11031, Taiwan},
abstract={Purpose: Creating a healthy lifestyle is important across different life stages. Commercial smart wearable devices are an innovative and interesting approach as an early psychological intervention for modifying health-related behaviors. Therefore, the purpose of this study was to explore the effects of smart wearable devices on health-promoting lifestyles and quality of life. Methods: The study design was a three-parallel randomized controlled trial with a 3-month intervention. Two commercial smart wearable devices (smartwatches and smart bracelets) with different levels of complicated functions were applied as a psychological intervention in comparison with a smartphone app as the control group. Participants were healthy young adults with a median age of 26 years. Outcome measurements were conducted by self-administered questionnaires. Chi-square tests and ANOVA were performed for testing the difference of participants at baseline, and generalized estimating equations were performed for testing the effect of the intervention. Results: At the beginning, 81 participants were recruited and 73 participants completed the study. Results of a healthy lifestyle demonstrated significant group effects of exercise and a significant effect of the interaction for self-actualization and stress management in the experimental group with a smartwatch (Self-actualization: MD = 0.35[- 0.10,0.80]; Exercise: MD = 0.21[-

0.33 0.75]; Stress management: MD = 0.36[- 0.04,0.76]) by comparing with only using mobile app (Self-actualization: MD = - 0.03[- 0.25,0.18]; Exercise: MD = - 0.12[- 0.38,0.14]; Stress management, MD = - 0.28[- 0.55,0.00]). The significant effect of group-by-time interaction for self-actualization was found in the experimental group with a smart bracelet (MD = 0.05[- 0.30,0.20]) by comparing with the control group. The GEE-adjusted model indicated significant effects of the interaction on the comprehensive, physical, and mental quality of life in the experimental group with the smartwatch (Comprehensive: MD = 0.24[- 0.04,0.52]; Physical: MD = 0.67[0.26,1.09]; Mental: MD = 0.72[0.29,1.16]) by comparing with the control group (Comprehensive: MD = - 1.57[- 2.55, - 0.59]; Physical: MD = 0.25[0.00,0.50]; Mental: MD = 0.08[- 0.11,0.27]). Conclusion: From a psychological perspective, smart wearable devices have potential benefits of shaping a healthy lifestyle and improving the quality of life. Enhancing the utility of commercial well-designed smart wearable devices is an innovative and effective strategy for promoting public health. © 2020, Springer Nature Switzerland AG.},
author_keywords={Health behavior; Mobile health; Physical activity; Sedentary behavior; Stress management},
references={(2018) Noncommunicable Diseases, , https://www.who.int/news-room/fact-sheets/detail/noncommunicable-diseases, Retrieved from; Hunter, D.J., Reddy, K.S., Noncommunicable Diseases (2013) The New England Journal of Medicine, 369 (14), pp. 1336-1343; Maher, C., Ryan, J., Ambrosi, C., Edney, S., Users’ experiences of wearable activity trackers: A cross-sectional study (2017) BMC Public Health, 17 (1), pp. 1-9; Naslund, J.A., Aschbrenner, K.A., Bartels, S.J., Wearable devices and smartphones for activity tracking among people with serious mental illness (2016) Mental Health and Physical Activity, 10, pp. 10-17; Wadden, T.A., Tronieri, J.S., Butryn, M.L., Lifestyle modification approaches for the treatment of obesity in adults (2020) The American psychologist, 75 (2), pp. 235-251; Jakicic, J.M., Davis, K.K., Rogers, R.J., King, W.C., Marcus, M.D., Helsel, D., Belle, S.H., Effect of wearable technology combined with a lifestyle intervention on long-term weight loss: The IDEA randomized clinical trial (2016) JAMA - Journal of the American Medical Association, 316 (11), pp. 1161-1171; Arigo, D., Promoting physical activity among women using wearable technology and online social connectivity: a feasibility study (2015) Health Psychology and Behavioral Medicine, 3 (1), pp. 391-409; Lin, F., Continued use of wearables for wellbeing with a cultural probe* (2019) Service Industries Journal, 39 (15-16), pp. 1140-1166; Gualtieri, L., Rosenbluth, S., Phillips, J., Can a Free Wearable Activity Tracker Change Behavior? The Impact of Trackers on Adults in a Physician-Led Wellness Group (2016) JMIR Research Protocols, 5 (4); Wu, Q., Sum, K., Nathan-Roberts, D., How Fitness trackers facilitate health behavior change (2016) Proceedings of the Human Factors and Ergonomics Society, 2014, pp. 1067-1071; Wen, D., Zhang, X., Liu, X., Lei, J., Evaluating the consistency of current mainstream wearable devices in health monitoring: A comparison under free-living conditions (2017) Journal of Medical Internet Research, 19 (3), pp. 1-20; Mohr, D.C., Burns, M.N., Schueller, S.M., Clarke, G., Klinkman, M., Behavioral Intervention Technologies: Evidence review and recommendations for future research in mental health (2013) General Hospital Psychiatry, 35 (4), pp. 332-338; Cadmus-Bertram, L.A., Marcus, B.H., Patterson, R.E., Parker, B.A., Morey, B.L., Randomized Trial of a Fitbit-Based Physical Activity Intervention for Women (2015) American Journal of Preventive Medicine, 49 (3), pp. 414-418; Neațu, A.M., Public health and behavioral economics nudging behaviors through wearables (2015) International Journal of Economic Practices and Theories, 5 (5), pp. 518-526; Mohr, D.C., Schueller, S.M., Riley, W.T., Brown, C.H., Cuijpers, P.,

Duan, N., Cheung, K., Trials of intervention principles: Evaluation methods for evolving behavioral intervention technologies (2015) Journal of Medical Internet Research, 17 (7); Mohr, D.C., Schueller, S.M., Montague, E., Burns, M.N., Rashidi, P., The behavioral intervention technology model: An integrated conceptual and technological framework for ehealth and mhealth interventions (2014) Journal of Medical Internet Research, 16 (6); Walker, S.N., Sechrist, K.R., Pender, N.J., The Health-Promoting Lifestyle Profile (1987) Nursing Research; Sullivan, A.N., Lachman, M.E., Behavior change with fitness technology in sedentary adults: a review of the evidence for increasing physical activity (2017) Frontiers in Public Health, 4 (289), pp. 1-16; Chaddha, A., Jackson, E.A., Richardson, C.R., Franklin, B.A., Technology to help promote physical activity (2017) American Journal of Cardiology, 119 (1), pp. 149-152; Lyons, E.J., Swartz, M.C., Lewis, Z.H., Martinez, E., Jennings, K., Feasibility and acceptability of a wearable technology physical activity intervention with telephone counseling for mid-aged and older adults: a randomized controlled pilot trial (2017) JMIR mHealth and uHealth, 5 (3); Smith, E.N., Santoro, E., Moraveji, N., Susi, M., Crum, A.J., Integrating wearables in stress management interventions: promising evidence from a randomized trial (2019) International Journal of Stress Management; Handel, B., Kolstad, J., Wearable Technologies and Health Behaviors: New Data and New Methods to Understand Population Health (2017) The American economic review, 107 (5), pp. 481-485; Yurkiewicz, I.R., Simon, P., Liedtke, M., Dahl, G., Dunn, T., Effect of fitbit and ipad wearable technology in health-related quality of life in adolescent and young adult cancer patients (2018) Journal of Adolescent and Young Adult Oncology, 7 (5), pp. 579-583; Karimi, M., Brazier, J., Health, Health-Related Quality of Life, and Quality of Life: What is the Difference? (2016) PharmacoEconomics, 34 (7), pp. 645-649; McColl-Kennedy, J.R., Hogan, S.J., Witell, L., Snyder, H., Cocreative customer practices: Effects of health care customer value cocreation practices on well-being (2017) Journal of Business Research; Knight, A., Bidargaddi, N., Commonly available activity tracker apps and wearables as a mental health outcome indicator: A prospective observational cohort study among young adults with psychological distress (2018) Journal of Affective Disorders, 236 (April), pp. 31-36; de Arriba-Pérez, F., Santos-Gago, J.M., Caeiro-Rodríguez, M., Ramos-Merino, M., Study of stress detection and proposal of stress-related features using commercial-off-the-shelf wrist wearables (2019) Journal of Ambient Intelligence and Humanized Computing, 10 (12), pp. 4925-4945; Rosenberger, M.E., Buman, M.P., Haskell, W.L., McConnell, M.V., Carstensen, L.L., Twenty-four Hours of Sleep, Sedentary Behavior, and Physical Activity with Nine Wearable Devices (2016) Medicine and Science in Sports and Exercise, 48 (3), pp. 457-465; Kang, S.G., Kang, J.M., Ko, K.P., Park, S.C., Mariani, S., Weng, J., Validity of a commercial wearable sleep tracker in adult insomnia disorder patients and good sleepers (2017) Journal of Psychosomatic Research, 97 (2016), pp. 38-44; Kortlik, J.W., Williams, H.A., The incorporation of effect size in information technology, learning, and performance research (2003) Journal Spring; Walker, S., Sechrist, K., Pender, N., (1995) Health Promotion Model - Instruments to Measure Health Promoting Lifestyle : Health-Promoting Lifestyle Profile [HPLP II] (Adult Version), , Journal of Nursing Research; Teng, H.L., Yen, M., Fetzer, S., Health promotion lifestyle profile-II: Chinese version short form (2010) Journal of Advanced Nursing; Harper, A., Power, M., Orley, J., Herrman, H., Schofield, H., Murphy, B., Sartorius, N., Development of the world health organization WHOQOL-BREF quality of life assessment (1998) Psychological Medicine; Yao, G., Chung, C.W., Yu, C.F., Wang, J.D., Development and verification of validity and reliability of the WHOQOL-BREF Taiwan version

(2002) Journal of the Formosan Medical Association; Espinoza, J., Chen, A., Orozco, J., Deavenport-Saman, A., Yin, L., Effect of personal activity trackers on weight loss in families enrolled in a comprehensive behavioral family-lifestyle intervention program in the federally qualified health center setting: A randomized controlled trial (2017) Contemporary Clinical Trials Communications, 7 (June), pp. 86-94; Cowdery, J., Majeske, P., Frank, R., Brown, D., Exergame Apps and Physical Activity: The Results of the ZOMBIE Trial (2015) American Journal of Health Education, 46 (4), pp. 216-222; Chen, J.-L., Guedes, C.M., Lung, A.E., Smartphone-based healthy weight management intervention for Chinese American adolescents short-term efficacy and factors associated with decreased weight (2018) Journal of Adolescent Health, 64 (4), pp. 443-449; Lewis, Z.H., Pritting, L., Picazo, A.L., JeanMarie-Tucker, M., The utility of wearable fitness trackers and implications for increased engagement: An exploratory, mixed methods observational study (2020) Digital Health; Millings, A., Morris, J., Rowe, A., Easton, S., Martin, J.K., Majoe, D., Mohr, C., Can the effectiveness of an online stress management program be augmented by wearable sensor technology? (2015) Internet Interventions, 2 (3), pp. 330-339; Amarantos, E., Martinez, A., Dwyer, J., Nutrition and quality of life in older adults (2001) Journals of Gerontology - Series A Biological Sciences and Medical Sciences; Lennefer, T., Lopper, E., Wiedemann, A.U., Hess, U., Hoppe, A., Improving Employees’ Work-Related Well-Being and Physical Health Through a Technology-Based Physical Activity Intervention: A Randomized Intervention- Control Group Study (2019) Journal of Occupational Health Psychology; Coughlin, S.S., Stewart, J., Use of Consumer Wearable Devices to Promote Physical Activity A Review of Health Intervention Studies (2016) Journal of environment and health sciences; Baron, K.G., Duffecy, J., Berendsen, M.A., Cheung Mason, I., Lattie, E.G., Manalo, N.C., Feeling validated yet? A scoping review of the use of consumer-targeted wearable and mobile technology to measure and improve sleep (2018) Sleep Medicine Reviews, 40, pp. 151-159; Randriambelonoro, M., Chen, Y., Pu, P., Can Fitness Trackers Help Diabetic and Obese Users Make and Sustain Lifestyle Changes? (2017) Computer, 50 (3), pp. 20-29; Jiang, X., Ming, W.K., You, J.H.S., The cost-effectiveness of digital health interventions on the management of cardiovascular diseases: Systematic review (2019) Journal of Medical Internet Research, 21 (6), pp. 1-11; Stephenson, A., McDonough, S.M., Murphy, M.H., Nugent, C.D., Mair, J.L., Using computer, mobile and wearable technology enhanced interventions to reduce sedentary behaviour: a systematic review and meta-analysis (2017) The international journal of behavioral nutrition and physical activity, 14 (1), p. 105},
correspondence_address1={Yen, H.-Y.; School of Gerontology Health Management, 250 Wuxing St., Taiwan; email: yenken520@gmail.com},
publisher={Springer Science and Business Media Deutschland GmbH},
issn={09629343},
coden={QLREE},
pubmed_id={33104939},
language={English},
abbrev_source_title={Qual. Life Res.},
document_type={Article},
source={Scopus},
}

@ARTICLE{Cho2021,
author={Cho, S. and Ensari, I. and Weng, C. and Kahn, M.G. and Natarajan, K.},
title={Factors affecting the quality of person-generated wearable device data

and associated challenges: Rapid systematic review},
journal={JMIR mHealth and uHealth},
year={2021},
volume={9},
number={3},
doi={10.2196/20738},
art_number={e20738},
note={cited By 0},
url={https://www.scopus.com/inward/record.uri?eid=2-s2.0-85103230396&doi=10.2196%2f20738&partnerID=40&md5=9559982c65cdffc60d253c7f11aa1724},
affiliation={Department of Biomedical Informatics, Columbia University, New York, NY, United States; Data Science Institute, Columbia University, New York, NY, United States; Department of Pediatrics, University of Colorado Anschutz Medical Campus, Denver, CO, United States},
abstract={Background: There is increasing interest in reusing person-generated wearable device data for research purposes, which raises concerns about data quality. However, the amount of literature on data quality challenges, specifically those for person-generated wearable device data, is sparse. Objective: This study aims to systematically review the literature on factors affecting the quality of person-generated wearable device data and their associated intrinsic data quality challenges for research. Methods: The literature was searched in the PubMed, Association for Computing Machinery, Institute of Electrical and Electronics Engineers, and Google Scholar databases by using search terms related to wearable devices and data quality. By using PRISMA (Preferred Reporting Items for Systematic Reviews and Meta-Analyses) guidelines, studies were reviewed to identify factors affecting the quality of wearable device data. Studies were eligible if they included content on the data quality of wearable devices, such as fitness trackers and sleep monitors. Both research-grade and consumer-grade wearable devices were included in the review. Relevant content was annotated and iteratively categorized into semantically similar factors until a consensus was reached. If any data quality challenges were mentioned in the study, those contents were extracted and categorized as well. Results: A total of 19 papers were included in this review. We identified three high-level factors that affect data quality-deviceand technical-related factors, user-related factors, and data governance-related factors. Device- and technical-related factors include problems with hardware, software, and the connectivity of the device; user-related factors include device nonwear and user error; and data governance-related factors include a lack of standardization. The identified factors can potentially lead to intrinsic data quality challenges, such as incomplete, incorrect, and heterogeneous data. Although missing and incorrect data are widely known data quality challenges for wearable devices, the heterogeneity of data is another aspect of data quality that should be considered for wearable devices. Heterogeneity in wearable device data exists at three levels: heterogeneity in data generated by a single person using a single device (within-person heterogeneity); heterogeneity in data generated by multiple people who use the same brand, model, and version of a device (between-person heterogeneity); and heterogeneity in data generated from multiple people using different devices (between-person heterogeneity), which would apply especially to data collected under a bring-your-own-device policy. Conclusions: Our study identifies potential intrinsic data quality challenges that could occur when analyzing wearable device data for research and three major contributing factors for these challenges. As poor data quality can compromise the reliability and accuracy of research results, further

investigation is needed on how to address the data quality challenges of wearable devices. © 2021 JMIR Publications. All rights reserved.},
author_keywords={Data accuracy; Data quality; Fitness trackers; Mobile phone; Patient generated health data; Wearable device},
references={Nittas, V, Mütsch, M, Ehrler, F, Puhan, MA., Electronic patient-generated health data to facilitate prevention and health promotion: a scoping review protocol (2018) BMJ Open, 8 (8), p. e021245. , Aug 10; [FREE Full text] [doi] [Medline: 30099392]; McCarthy, J., (2019) One in Five U.S. Adults Use Health Apps, Wearable Trackers, , https://news.gallup.com/poll/269096/one-five-adults-health-apps-wearable-trackers.aspx, Gallup. Dec 11. [accessed 2020-09-20]; Wood, WA, Bennett, AV, Basch, E., Emerging uses of patient generated health data in clinical research (2015) Mol Oncol, 9 (5), pp. 1018-1024. , May; [FREE Full text] [doi] [Medline: 25248998]; Hickey, AM, Freedson, PS., Utility of Consumer Physical Activity Trackers as an Intervention Tool in Cardiovascular Disease Prevention and Treatment (2016) Prog Cardiovasc Dis, 58 (6), pp. 613-619. , [doi] [Medline: 26943981]; Haghi, M, Thurow, K, Stoll, R., Wearable Devices in Medical Internet of Things: Scientific Research and Commercially Available Devices (2017) Healthc Inform Res, 23 (1), pp. 4-15. , Jan; [FREE Full text] [doi] [Medline: 28261526]; Izmailova, ES, Wagner, JA, Perakslis, ED., Wearable Devices in Clinical Trials: Hype and Hypothesis (2018) Clin Pharmacol Ther, 104 (1), pp. 42-52. , Jul; [FREE Full text] [doi] [Medline: 29205294]; Codella, J, Partovian, C, Chang, HY, Chen, CH., Data quality challenges for person-generated health and wellness data (2018) IBM J. Res. & Dev, 62 (1). , Jan 1; 3:1-3:8. [doi]; Lim, WK, Davila, S, Teo, JX, Yang, C, Pua, CJ, Blöcker, C, Beyond fitness tracking: The use of consumer-grade wearable data from normal volunteers in cardiovascular and lipidomics research (2018) PLoS Biol, 16 (2), p. e2004285. , Dec; [FREE Full text] [doi] [Medline: 29485983]; McDonald, L, Mehmud, F, Ramagopalan, SV., Sleep and BMI: Do (Fitbit) bands aid? (2018) F1000Res, 7, p. 511. , [FREE Full text] [doi] [Medline: 30271578]; Cheung, YK, Hsueh, PS, Ensari, I, Willey, JZ, Diaz, KM., Quantile Coarsening Analysis of High-Volume Wearable Activity Data in a Longitudinal Observational Study (2018) Sensors (Basel), 18 (9). , Sep 12; [FREE Full text] [doi] [Medline: 30213093]; Burg, MM, Schwartz, JE, Kronish, IM, Diaz, KM, Alcantara, C, Duer-Hefele, J, Does Stress Result in You Exercising Less? Or Does Exercising Result in You Being Less Stressed? Or Is It Both? Testing the Bi-directional Stress-Exercise Association at the Group and Person (N of 1) Level (2017) Ann Behav Med, 51 (6), pp. 799-809. , Dec; [FREE Full text] [doi] [Medline: 28290065]; Henriksen, A, Haugen, MM, Woldaregay, AZ, Muzny, M, Hartvigsen, G, Hopstock, LA, Using Fitness Trackers and Smartwatches to Measure Physical Activity in Research: Analysis of Consumer Wrist-Worn Wearables (2018) J Med Internet Res, 20 (3), p. e110. , Mar 22; [FREE Full text] [doi] [Medline: 29567635]; Safran, C., Update on Data Reuse in Health Care (2017) Yearb Med Inform, 26 (1), pp. 24-27. , Aug; [FREE Full text] [doi] [Medline: 29063535]; Data Sources. All of Us, , https://www.researchallofus.org/data/data-sources/#mobile-health-data, National Institutes of Health (NIH). [accessed 2021-02-27]; Menai, M, Brouard, B, Vegreville, M, Chieh, A, Schmidt, N, Oppert, J, Cross-Sectional and longitudinal associations of objectively-measured physical activity on blood pressure: evaluation in 37 countries (2017) Health Promot Perspect, 7 (4), pp. 190-196. , [FREE Full text] [doi] [Medline: 29085795]; Kim, K, Nikzad, N, Quer, G, Wineinger, NE, Vegreville, M, Normand, A, Real World Home Blood Pressure Variability in Over 56, 000 Individuals With Nearly 17 Million Measurements (2018) Am J Hypertens, 31 (5), pp. 566-573. , Apr 13; [FREE Full text] [doi] [Medline: 29365036]; Weiner, MG, Embi, PJ., Toward reuse of clinical data for

research and quality improvement: the end of the beginning? (2009) Ann Intern Med, 151 (5), pp. 359-360. , Sep 01; [doi] [Medline: 19638404]; Zozus, M, Kahn, M, Weiskopf, N., Data Quality in Clinical Research (2019) Clinical Research Informatics, pp. 213-248. , New York, NY: Springer International Publishing; Hicks, JL, Althoff, T, Sosic, R, Kuhar, P, Bostjancic, B, King, AC, Best practices for analyzing large-scale health data from wearables and smartphone apps (2019) NPJ Digit Med, 2, p. 45. , [FREE Full text] [doi] [Medline: 31304391]; Abdolkhani, R, Borda, A, Gray, K., Quality Management of Patient Generated Health Data in Remote Patient Monitoring Using Medical Wearables - A Systematic Review (2018) Stud Health Technol Inform, 252, pp. 1-7. , [Medline: 30040674]; Kahn, MG, Callahan, TJ, Barnard, J, Bauck, AE, Brown, J, Davidson, BN, A Harmonized Data Quality Assessment Terminology and Framework for the Secondary Use of Electronic Health Record Data (2016) EGEMS (Wash DC), 4 (1), p. 1244. , [FREE Full text] [doi] [Medline: 27713905]; Fiorini, N, Canese, K, Starchenko, G, Kireev, E, Kim, W, Miller, V, Best Match: New relevance search for PubMed (2018) PLoS Biol, 16 (8), p. e2005343. , Aug; [FREE Full text] [doi] [Medline: 30153250]; Piasecki, J, Waligora, M, Dranseika, V., Google Search as an Additional Source in Systematic Reviews (2018) Sci Eng Ethics, 24 (2), pp. 809-810. , Apr; [FREE Full text] [doi] [Medline: 29249022]; Wright, SP, Hall, BTS, Collier, SR, Sandberg, K., How consumer physical activity monitors could transform human physiology research (2017) Am J Physiol Regul Integr Comp Physiol, 312 (3), pp. R358-R367. , Mar 01; [doi] [Medline: 28052867]; Bietz, MJ, Bloss, CS, Calvert, S, Godino, JG, Gregory, J, Claffey, MP, Opportunities and challenges in the use of personal health data for health research (2016) J Am Med Inform Assoc, 23 (e1), pp. e42-e48. , Apr; [doi] [Medline: 26335984]; Fawcett, T., Mining the Quantified Self: Personal Knowledge Discovery as a Challenge for Data Science (2015) Big Data, 3 (4), pp. 249-266. , Dec; [doi] [Medline: 27441406]; Düking, P, Fuss, FK, Holmberg, H, Sperlich, B., Recommendations for Assessment of the Reliability, Sensitivity, and Validity of Data Provided by Wearable Sensors Designed for Monitoring Physical Activity (2018) JMIR Mhealth Uhealth, 6 (4), p. e102. , Apr 30; [FREE Full text] [doi] [Medline: 29712629]; Guo, A, Ma, J., Context-Aware Scheduling in Personal Data Collection From Multiple Wearable Devices (2017) IEEE Access, 5, pp. 2602-2614. , [doi]; Constantinou, V, Felber, AE, Chan, JL., Applicability of consumer activity monitor data in marathon events: an exploratory study (2017) J Med Eng Technol, 41 (7), pp. 534-540. , Oct; [doi] [Medline: 28954563]; Hardy, J, Veinot, TC, Yan, X, Berrocal, VJ, Clarke, P, Goodspeed, R, User acceptance of location-tracking technologies in health research: Implications for study design and data quality (2018) J Biomed Inform, 79, pp. 7-19. , Mar;: [FREE Full text] [doi] [Medline: 29355784]; Banerjee, T, Sheth, A., IoT Quality Control for Data and Application Needs (2017) IEEE Intell. Syst, 32 (2), pp. 68-73. , Mar; [doi]; Karkouch, A, Mousannif, H, Al Moatassime, H, Noel, T., Data quality in internet of things: A state-of-the-art survey (2016) Journal of Network and Computer Applications, 73, pp. 57-81. , Sep;: [doi]; Liang, Z, Ploderer, B, Chapa-Martell, M., Is Fitbit Fit for Sleep-tracking? Sources of Measurement Errors and Proposed Countermeasures (2017) Proc 11th EAI Int Conf Pervasive Comput Technol Healthc, pp. 476-479. , USA: ACM; 2017 Presented at: May 23-26, New York, NY [doi]; Reinerman-Jones, L, Harris, J, Watson, A., Considerations for using fitness trackers in psychophysiology research (2017) Human Interface and the Management of Information: Information, Knowledge and Interaction Design. HIMI 2017, pp. 598-606. , New York, NY: Springer; Jülicher, T, Delisle, M., Step into "The Circle" - A Close Look at Wearables and Quantified Self (2018) Big Data Context, pp. 81-91. , New York, NY: Springer; Beukenhorst, AL, Sergeant, JC, Little, MA, McBeth, J, Dixon, WG., Consumer

Smartwatches for Collecting Self-Report and Sensor Data: App Design and Engagement (2018) Stud Health Technol Inform, 247, pp. 291-295. , [Medline: 29677969]; Cleland, I, Donnelly, MP, Nugent, CD, Hallberg, J, Espinilla, M, Garcia-Constantino, M., Collection of a Diverse, Realistic and Annotated Dataset for Wearable Activity Recognition (2018) 2018 Presented at: 2018 IEEE International Conference on Pervasive Computing and Communications Workshops, pp. 555-560. , March 19-23, Athens, Greece; Mezghani, E, Exposito, E, Drira, K, Da Silveira, M, Pruski, C., A Semantic Big Data Platform for Integrating Heterogeneous Wearable Data in Healthcare (2015) J Med Syst, 39 (12), p. 185. , Dec; [doi] [Medline: 26490143]; Lai, AM, Hsueh, PS, Choi, YK, Austin, RR., Present and Future Trends in Consumer Health Informatics and Patient-Generated Health Data (2017) Yearb Med Inform, 26 (1), pp. 152-159. , Aug; [FREE Full text] [doi] [Medline: 29063559]; Oh, J, Lee, U., Exploring UX issues in Quantified Self technologies (2015) 2015 Presented at: 2015 Eighth International Conference on Mobile Computing and Ubiquitous Networking (ICMU), pp. 53-59. , January 20-22, Hakodate, Japan [doi]; Wickel, EE., Reporting the reliability of accelerometer data with and without missing values (2014) PLoS One, 9 (12), p. e114402. , [FREE Full text] [doi] [Medline: 25478692]; Stephens, S, Beyene, J, Tremblay, MS, Faulkner, G, Pullnayegum, E, Feldman, BM., Strategies for Dealing with Missing Accelerometer Data (2018) Rheum Dis Clin North Am, 44 (2), pp. 317-326. , May; [doi] [Medline: 29622298]; Sun, H., (2011) Enterprise Information Management: Best Practices in Data Governance, , https://www.oracle.com/technetwork/articles/entarch/oea-best-practices-data-gov-400760.pdf, Oracle Corporation; May; West, P, Van, KM, Giordano, R, Weal, M, Shadbolt, N., Information Quality Challenges of Patient-Generated Data in Clinical Practice (2017) Front Public Health, 5, p. 284. , [FREE Full text] [doi] [Medline: 29209601]; Khare, R, Ruth, B, Miller, M, Tucker, J, Utidjian, L, Razzaghi, H., Predicting Causes of Data Quality Issues in a Clinical Data Research Network (2018) AMIA Joint Summits Translational Science, pp. 113-121. , May 18 Presented at: March 12-15, 2018; San Francisco; Ong, T, Pradhananga, R, Holve, E, Kahn, MG., A Framework for Classification of Electronic Health Data Extraction-Transformation-Loading Challenges in Data Network Participation (2017) EGEMS (Wash DC), 5 (1), p. 10. , Jun 13; [FREE Full text] [doi] [Medline: 29930958]; Mailey, EL, Gothe, NP, Wójcicki, TR, Szabo, AN, Olson, EA, Mullen, SP, Influence of allowable interruption period on estimates of accelerometer wear time and sedentary time in older adults (2014) J Aging Phys Act, 22 (2), pp. 255-260. , Apr; [FREE Full text] [doi] [Medline: 23752299]; Evenson, KR, Terry, JW., Assessment of differing definitions of accelerometer nonwear time (2009) Res Q Exerc Sport, 80 (2), pp. 355-362. , Jun; [FREE Full text] [doi] [Medline: 19650401]; Tang, LM, Meyer, J, Epstein, DA, Bragg, K, Engelen, L, Bauman, A, Defining Adherence: Making Sense of Physical Activity Tracker Data (2018) ACM on Interactive, Mobile, Wearable and Ubiquitous Technologies, 2 (1), pp. 1-22. , Mar; [doi]; Low, CA, Dey, AK, Ferreira, D, Kamarck, T, Sun, W, Bae, S, Estimation of Symptom Severity During Chemotherapy From Passively Sensed Data: Exploratory Study (2017) J Med Internet Res, 19 (12), p. e420. , [FREE Full text] [doi] [Medline: 29258977]; Collins, T, Woolley, SI, Oniani, S, Pires, IM, Garcia, NM, Ledger, SJ, Version Reporting and Assessment Approaches for New and Updated Activity and Heart Rate Monitors (2019) Sensors (Basel), 19 (7). , Apr 10; [FREE Full text] [doi] [Medline: 30974755]; Evenson, KR, Goto, MM, Furberg, RD., Systematic review of the validity and reliability of consumer-wearable activity trackers (2015) Int J Behav Nutr Phys Act, 12 (1), p. 159. , [FREE Full text] [doi] [Medline: 26684758]; Liberati, A, Altman, DG, Tetzlaff, J, Mulrow, C, Gøtzsche, PC, Ioannidis, JPA, The PRISMA statement for reporting systematic reviews and

meta-analyses of studies that evaluate healthcare interventions: explanation and elaboration (2009) BMJ, 339, p. b2700. , [FREE Full text] [Medline: 19622552]; Ancker, JS, Shih, S, Singh, MP, Snyder, A, Edwards, A, Kaushal, R, Root causes underlying challenges to secondary use of data (2011) AMIA Annu Symp Proc, 2011, pp. 57-62. , HITEC investigators. ;: [FREE Full text] [Medline: 22195055]},
correspondence_address1={Cho, S.; Department of Biomedical Informatics, 622 West 168th Street PH20, United States; email: sc3901@cumc.columbia.edu},
publisher={JMIR Publications Inc.},
issn={22915222},
pubmed_id={33739294},
language={English},
abbrev_source_title={JMIR mHealth uHealth},
document_type={Review},
source={Scopus},
}

@ARTICLE{Buoitestella2021452,
author={Buoitestella, A. and Ajčevic, M. and Furlanis, G. and Cillotto, T. and Menichelli, A. and Accardo, A. and Manganotti, P.},
title={Smart technology for physical activity and health assessment during COVID-19 lockdown},
journal={Journal of Sports Medicine and Physical Fitness},
year={2021},
volume={61},
number={3},
pages={452-460},
doi={10.23736/S0022-4707.20.11373-2},
note={cited By 1},
url={https://www.scopus.com/inward/record.uri?eid=2-s2.0-85100496844&doi=10.23736%2fS0022-4707.20.11373-2&partnerID=40&md5=5888047c3e805f27ed51a05d96fcddff},
affiliation={Department of Medicine, Surgery and Health Sciences, Clinical Unit of Neurology, Cattinara University Hospital (ASUGI), Trieste, Italy; Department of Engineering and Architecture, University of Trieste, Trieste, Italy; Department of Medicine, Surgery and Health Sciences, Rehabilitation Unit, Maggiore City Hospital, Trieste, Italy},
abstract={BACKGROUND: The worldwide lockdown in response to COVID-19 pandemics has often led to physical inactivity and social distancing. When combined, these factors may affect quality and life and increase the risk of different diseases. Therefore, assessment of physical activity levels should be encouraged to monitor and identify those at a higher risk of inactivity. Smart technology is a promising tool to assess physical activity and health and may be particularly useful during a period of general lockdown. METHODS: An online survey was developed and shared among the Italian general population to collect data about physical activity and daily routine changes between January 2020 and the lockdown period from March 23 to March 29. Participants were asked to provide data such as daily step count (Steps) or heart rate (HR) measured and collected by their smart technology devices. RESULTS: Four hundred participants were included in the final analysis. During the lockdown, the number of steps dropped from 8284±4390 to 3294±3994 steps (P<0.001), while mean peak HR decreased from 61.3±18.2% to 55.9±17.3% (P<0.001). CONCLUSIONS: These findings provide objective data about the effects of the COVID-19 lockdown on physical activity, thus encouraging the use of smart devices to monitor and promote healthy lifestyles while faced with a confinement condition. © 2020 EDIZIONI

MINERVA MEDICA.},
author_keywords={Exercise; Sars-cov-2; Wearable electronic devices},
keywords={activity tracker; adult; epidemiology; exercise; female; health survey; heart rate; human; Italy; male; middle aged; mobile application; pandemic; physiologic monitoring; prevention and control; quarantine; smartphone; young adult, Adult; COVID-19; Exercise; Female; Fitness Trackers; Health Surveys; Heart Rate; Humans; Italy; Male; Middle Aged; Mobile Applications; Monitoring, Physiologic; Pandemics; Physical Distancing; Quarantine; SARS-CoV-2; Sedentary Behavior; Smartphone; Young Adult},
references={Caso, V, Federico, A., No lockdown for neurological diseases during COVID19 pandemic infection (2020) Neurol Sci, 41, pp. 999-1001; Grasselli, G, Pesenti, A, Cecconi, M., Critical Care Utilization for the COVID-19 Outbreak in Lombardy, Italy: Early Experience and Forecast During an Emergency Response (2020) JAMA, 323, pp. 1545-1546; Booth, FW, Roberts, CK, Laye, MJ., Lack of exercise is a major cause of chronic diseases (2012) Compr Physiol, 2, pp. 1143-1211; Ding, D, Kolbe-Alexander, T, Nguyen, B, Katzmarzyk, PT, Pratt, M, Lawson, KD., The economic burden of physical inactivity: A systematic review and critical appraisal (2017) Br J Sports Med, 51, pp. 1392-1409; Lavie, CJ, Ozemek, C, Carbone, S, Katzmarzyk, PT, Blair, SN., Sedentary Behavior, Exercise, and Cardiovascular Health (2019) Circ Res, 124, pp. 799-815; López-Blanco, R, Velasco, MA, Méndez-Guerrero, A, Romero, JP, Del Castillo, MD, Serrano, JI, Smartwatch for the analysis of rest tremor in patients with Parkinson's disease (2019) J Neurol Sci, 401, pp. 37-42; Lawrie, S, Dong, Y, Steins, D, Xia, Z, Esser, P, Sun, S, Evaluation of a smartwatch-based intervention providing feedback of daily activity within a research-naive stroke ward: A pilot randomised controlled trial (2018) Pilot Feasibility Stud, 4, p. 157. , Smart Watch Activity Feedback Trial Committee (SWAFT); Midaglia, L, Mulero, P, Montalban, X, Adherence and Satisfaction of Smartphone- A nd Smartwatch-Based Remote Active Testing and Passive Monitoring in People With Multiple Sclerosis: Nonrandomized Interventional Feasibility Study (2019) J Med Internet Res, 21, p. 14863; Menta, AK, Subbiah, IM, Subbiah, V., Bringing wearable devices into oncology practice: Fitting smart technology in the clinic (2018) Discov Med, 26, pp. 261-270; Degroote, L, De Bourdeaudhuij, I, Verloigne, M, Poppe, L, Crombez, G., The Accuracy of Smart Devices for Measuring Physical Activity in Daily Life: Validation Study (2018) JMIR Mhealth Uhealth, 6, p. e10972; Henriksen, A, Haugen Mikalsen, M, Woldaregay, AZ, Muzny, M, Hartvigsen, G, Hopstock, LA, Using Fitness Trackers and Smartwatches to Measure Physical Activity in Research: Analysis of Consumer Wrist-Worn Wearables (2018) J Med Internet Res, 20, p. e110; Strain, T, Wijndaele, K, Brage, S., Physical Activity Surveillance Through Smartphone Apps and Wearable Trackers: Examining the UK Potential for Nationally Representative Sampling (2019) JMIRMhealth Uhealth, 7, p. e11898; Lasorsa, I, D Antrassi, P, Ajčević, M, Stellato, K, Di Lenarda, A, Marceglia, S, Personalized support for chronic conditions. Anovel approach for enhancing self-management and improving lifestyle (2016) Appl Clin Inform, 7, pp. 633-645; Ho, A, Quick, O., Leaving patients to their own devices? Smart technology, safety and therapeutic relationships (2018) BMC Med Ethics, 19, p. 18; Narici, M, De Vito, G, Franchi, M, Paoli, A, Moro, T, Marcolin, G, Impact of sedentarism due to the COVID-19 home confinement on neuromuscular, cardiovascular and metabolic health: Physiological and pathophysiological implications and recommendations for physical and nutritional countermeasures (2020) Eur J Sport Sci, pp. 1-22. , [Epub ahead of print]; Jurak, G, Morrison, SA, Leskošek, B, Kovač, M, Hadžić, V, Vodičar, J, Physical activity recommendations during the coronavirus

disease-2019 virus outbreak (2020) J Sport Health Sci, 9, pp. 325-327; Buoite Stella, A, Yardley, J, Francescato, MP, Morrison, SA., Fluid Intake Habits in Type 1 Diabetes Individuals during Typical Training Bouts (2018) Ann Nutr Metab, 73, pp. 10-18; Tanaka, H, Monahan, KD, Seals, DR., Age-predicted maximal heart rate revisited (2001) J Am Coll Cardiol, 37, pp. 153-156; Minetto, MA, Motta, G, Gorji, NE, Lucini, D, Biolo, G, Pigozzi, F, Reproducibility and validity of the Italian version of the International Physical Activity Questionnaire in obese and diabetic patients (2018) J Endocri-nol Invest, 41, pp. 343-349; Tudor-Locke, C, Craig, CL, Brown, WJ, Clemes, SA, De Cocker, K, Giles-Corti, B, How many steps/day are enough? For adults (2011) Int J Behav Nutr Phys Act, 8, p. 79; Apolone, G, Mosconi, P., The Italian SF-36 Health Survey: Translation, validation and norming (1998) J Clin Epidemiol, 51, pp. 1025-1036; Ammar, A, Brach, M, Trabelsi, K, Effects of COVID-19 home confinement on physical activity and eating behaviour Preliminary results of the ECLB-COVID19 international online-survey (2020) medRxiv, , [Epub ahead of print]; Lee, PH, Macfarlane, DJ, Lam, TH, Stewart, SM., Validity of the International Physical Activity Questionnaire Short Form (IPAQ-SF): A systematic review (2011) Int J Behav Nutr Phys Act, 8, p. 115; Ahmad, MH, Salleh, R, Mohamad Nor, NS, Baharuddin, A, Rodzlan Hasani, WS, Omar, A, Comparison between self-reported physical activity (IPAQ-SF) and pedometer among overweight and obese women in the MyBFF@home study (2018) BMC Womens Health, 18, p. 100; Huang, Y, Zhao, N., Generalized anxiety disorder, depressive symptoms and sleep quality during COVID-19 outbreak in China: A web-based cross-sectional survey (2020) Psychiatry Res, 288, p. 112954; Altena, E, Baglioni, C, Espie, CA, Ellis, J, Gavriloff, D, Holzinger, B, Dealing with sleep problems during home confinement due to the COVID-19 outbreak: Practical recommendations from a task force of the European CBT-IAcademy (2020) J Sleep Res, 29, p. e13052; Cellini, N, Canale, N, Mioni, G, Costa, S., Changes in sleep pattern, sense of time and digital media use during COVID-19 lockdown in Italy (2020) J Sleep Res, 29, p. e13074; Buoite Stella, A, Francescato, MP, Sims, ST, Morrison, SA., Fluid intake behavior in athletes during typical training bouts (2017) J Sports Med Phys Fitness, 57, pp. 1504-1512; Jenkins, M, Hargreaves, EA, Hodge, K., The Role of Psychological Flexibility in Physical Activity Maintenance (2019) J Sport Exerc Psychol, 41, pp. 185-193; Khan, S, Siddique, R, Li, H, Ali, A, Shereen, MA, Bashir, N, Impact of coronavirus outbreak on psychological health (2020) J Glob Health, 10, p. 010331; Haxhi, J, Balducci, S, Pugliese, G., Invest in METs, Not in Meds (2019) Am J Med, 132, p. e756; Pillay, JD, Kolbe-Alexander, TL, van Mechelen, W, Lambert, EV., Steps that count: The association between the number and intensity of steps accumulated and fitness and health measures (2014) J Phys Act Health, 11, pp. 10-17; Kraus, WE, Janz, KF, Powell, KE, Campbell, WW, Jakicic, JM, Troiano, RP, Daily Step Counts for Measuring Physical Activity Exposure and Its Relation to Health (2019) Med Sci Sports Exerc, 51, pp. 1206-1212. , 2018 PHYSICAL ACTIVITY GUIDELINES ADVISORY COMMITTEE; Lee, IM, Shiroma, EJ, Kamada, M, Bassett, DR, Matthews, CE, Buring, JE., Association of Step Volume and Intensity With All-Cause Mortality in Older Women (2019) JAMA Intern Med, 179, pp. 1105-1112; Tudor-Locke, C, Craig, CL, Thyfault, JP, Spence, JC., A step-defined sedentary lifestyle index: <5000 steps/day (2013) Appl Physiol Nutr Metab, 38, pp. 100-114; Jiménez-Pavón, D, Carbonell-Baeza, A, Lavie, CJ., Physical exercise as therapy to fight against the mental and physical consequences of COVID-19 quarantine: Special focus in older people (2020) Prog Cardiovasc Dis, 63, pp. 386-388; Bassett, DRJr, Toth, LP, LaMunion, SR, Crouter, SE., Step Counting: AReview of Measurement Considerations and Health-Related Applications (2017) Sports Med, 47, pp. 1303-1315; Hargreaves, EA, Mutrie, N, Fleming, JD.,

AWeb-Based Intervention to Encourage Walking (StepWise): Pilot Randomized Controlled Trial (2016) JMIR Res Protoc, 5, p. e14; Sember, V, Jurak, G, Kovač, M, urić, S, Starc, G., Decline of physical activity in early adolescence: A3-year cohort study (2020) PLoS One, 15, p. e0229305},
correspondence_address1={Buoitestella, A.; Department of Medicine, Strada di Fiume 447, Italy; email: abuoitestella@units.it},
publisher={Edizioni Minerva Medica},
issn={00224707},
coden={JMPFA},
pubmed_id={33092330},
language={English},
abbrev_source_title={J. Sports Med. Phys. Fitness},
document_type={Article},
source={Scopus},
}

@ARTICLE{Robinson2021,
author={Robinson, A. and Slight, R.D. and Husband, A.K. and Slight, S.P.},
title={Designing the optimal digital health intervention for patients' use before and after elective orthopedic surgery: Qualitative study},
journal={Journal of Medical Internet Research},
year={2021},
volume={23},
number={3},
doi={10.2196/25885},
art_number={e25885},
note={cited By 0},
url={https://www.scopus.com/inward/record.uri?eid=2-s2.0-85102683965&doi=10.2196%2f25885&partnerID=40&md5=809483483f3be437c482637384758e2a},
affiliation={School of Pharmacy, Newcastle University, Newcastle upon Tyne, United Kingdom; Population Health Sciences Institute, Newcastle University, Newcastle upon Tyne, United Kingdom; Newcastle upon Tyne Hospitals, NHS Foundation Trust, Newcastle upon Tyne, United Kingdom},
abstract={Background: Health behavior changes made by patients during the perioperative period can impact the outcomes and success of elective surgeries. However, there remains a limited understanding of how best to support patients during this time, particularly through the use of digital health interventions. Recognizing and understanding the potential unmet needs of elective orthopedic surgery patients is central to motivating healthier behavior change, improving recovery, and optimizing overall surgical success in the short and long term. Objective: The aim of this study is to explore patient perspectives on technology features that would help support them to change their lifestyle behaviors during the pre- and postoperative periods, and that could potentially maintain long-term healthy lifestyles following recovery. Methods: Semistructured interviews with pre- and postoperative elective orthopedic patients were conducted between May and June 2020 using telephone and video call-based software. Patient perspectives on the use of digital technologies to complement current surgical care and support with lifestyle behavior changes were discussed. Interviews were audio recorded and transcribed verbatim. Reflexive thematic analysis enabled the development of themes from the data, with QSR NVivo software (version 12) facilitating data management. Ethical approval was obtained from the National Health Service Health Research Authority. Results: A total of 18 participants were interviewed. Four themes were developed from the data regarding the design and functionality of digital

technologies to best support the perioperative journey. These center around an intervention's ability to incorporate interactive, user-centered features; direct a descriptive and structured recovery; enable customizable, patient-controlled settings; and deliver both general and specific surgical advice in a timely manner. Interventions that are initiated preoperatively and continued postoperatively were perceived as beneficial. Interventions designed with personalized milestones were found to better guide patients through a structured recovery. Individualized tailoring of preparatory and recovery information was desired by patients with previously high levels of physical activity before surgery. The use of personalized progression-based exercises further encouraged physical recovery; game-like rewards and incentives were regarded as motivational for making and sustaining health behavior change. In-built video calling and messaging features offered connectivity with peers and clinicians for supported care delivery. Conclusions: Specific intervention design and functionality features can provide better, structured support for elective orthopedic patients across the entire surgical journey and beyond. This study provides much-needed evidence relating to the optimal design and timing of digital interventions for elective orthopedic surgical patients. Findings from this study suggest a desire for personalized perioperative care, in turn, supporting patients to make health behavior changes to optimize surgical success. These findings should be used to influence future co-design projects to enable the design and implementation of patient-focused, tailored, and targeted digital health technologies within modern health care settings. © 2021 Journal of Medical Internet Research. All rights reserved.},
author_keywords={Behavior change; Digital technology; EHealth; MHealth; Mobile phone; Orthopedic surgery; Perioperative care; Prehabilitation; Qualitative research},
references={Al-Obaidi, B, Babu, S, Al-Hadithy, N., Smartphones in orthopaedics: a decade of innovation (2019) Br J Hosp Med (Lond), 80 (5), pp. 274-277. , May 02; [doi] [Medline: 31059338]; (2017), https://www.iqvia.com/insights/the-iqvia-institute/reports/the-growing-value-of-digital-health-in-the-united-kingdom, The growing value of digital health in the United Kingdom. The IQVIA Institute. Nov 01. [accessed 2021-02-02]; Armstrong, KA, Coyte, PC, Brown, M, Beber, B, Semple, JL., Effect of home monitoring via mobile app on the number of in-person visits following ambulatory surgery: a randomized clinical trial (2017) JAMA Surg, 152 (7), pp. 622-627. , Mar 22; [doi] [Medline: 28329223]; Schnall, R, Mosley, JP, Iribarren, SJ, Bakken, S, Carballo-Diéguez, A, Brown, IW., Comparison of a user-centered design, self-management app to existing mhealth apps for persons living with HIV (2015) JMIR Mhealth Uhealth, 3 (3), p. e91. , [FREE Full _text] [doi] [Medline: 26385783]; Birnbaum, F, Lewis, D, Rosen, RK, Ranney, ML., Patient engagement and the design of digital health (2015) Acad Emerg Med, 22 (6), pp. 754-756. , Jun; [FREE Full _text] [doi] [Medline: 25997375]; Brennan, L, Kessie, T, Caulfield, B., Patient experiences of rehabilitation and the potential for an mHealth system with biofeedback after breast cancer surgery: qualitative study (2020) JMIR Mhealth Uhealth, 8 (7), p. e19721. , Jul 29; [FREE Full text] [doi] [Medline: 32687476]; Burns, D, Razmjou, H, Shaw, J, Richards, R, McLachlin, S, Hardisty, M, Adherence tracking with smart watches for shoulder physiotherapy in rotator cuff pathology: protocol for a longitudinal cohort study (2020) JMIR Res Protoc, 9 (7), p. e17841. , Jul 05; [FREE Full _text] [doi] [Medline: 32623366]; Apolinário-Hagen, J, Menzel, M, Hennemann, S, Salewski, C., Acceptance of mobile health apps for disease management among people with multiple sclerosis: web-based survey study (2018) JMIR Form Res, 2 (2), p. e11977. , Dec 12; [FREE Full _text] [doi] [Medline:

30684408]; Santo, K, Singleton, A, Rogers, K, Thiagalingam, A, Chalmers, J, Chow, CK, Medication reminder applications to improve adherence in coronary heart disease: a randomised clinical trial (2019) Heart, 105 (4), pp. 323-329. , Feb 27; [doi] [Medline: 30150326]; Sua, YS, Jiang, Y, Thompson, DR, Wang, W., Effectiveness of mobile phone-based self-management interventions for medication adherence and change in blood pressure in patients with coronary heart disease: a systematic review and meta-analysis (2020) Eur J Cardiovasc Nurs, 19 (3), pp. 192-200. , Mar 19; [doi] [Medline: 31856596]; McDonald, S, Yates, D, Durrand, JW, Kothmann, E, Sniehotta, FF, Habgood, A, Exploring patient attitudes to behaviour change before surgery to reduce peri-operative risk: preferences for short- vs. long-term behaviour change (2019) Anaesthesia, 74 (12), pp. 1580-1588. , Dec 21; [FREE Full _text] [doi] [Medline: 31637700]; Robinson, AH, Husband, AK, Slight, RD, Slight, SP., Effectiveness of digital technologies to support surgical patients in changing their health behaviours: a systematic review (2020) BJS Open, pp. 1-4. , Dec 28: [FREE Full _text] [doi]; Truntzer, J, Vopat, B, Feldstein, M, Matityahu, A., Smoking cessation and bone healing: optimal cessation timing (2015) Eur J Orthop Surg Traumatol, 25 (2), pp. 211-215. , Feb 31; [doi] [Medline: 24879610]; Sørensen, LT., Wound healing and infection in surgery. The clinical impact of smoking and smoking cessation: a systematic review and meta-analysis (2012) Arch Surg, 147 (4), pp. 373-383. , Apr 01; [doi] [Medline: 22508785]; Luna, IE, Kehlet, H, Wede, HR, Hoevsgaard, SJ, Aasvang, EK., Objectively measured early physical activity after total hip or knee arthroplasty (2019) J Clin Monit Comput, 33 (3), pp. 509-522. , Jun 23; [doi] [Medline: 30039461]; Jansson, MM, Rantala, A, Miettunen, J, Puhto, A, Pikkarainen, M., The effects and safety of telerehabilitation in patients with lower-limb joint replacement: a systematic review and narrative synthesis (2020) J Telemed Telecare, p. 1357633X20917868. , Apr 21:. [doi] [Medline: 32316837]; Popat, R, Mohan, AT, Branford, OA., Current uses of smartphones and apps in orthopaedic surgery (2013) Br J Hosp Med (Lond), 74 (12), pp. 672-676. , Dec; [doi] [Medline: 24326713]; Chen, EA, Ellahie, A, Barsi, J., Smartphone applications in orthopaedic surgery: a review of the literature and application analysis (2019) Current Orthopaedic Practice, 30, p. 230. , [doi]; Watson, HA, Tribe, RM, Shennan, AH., The role of medical smartphone apps in clinical decision-support: a literature review (2019) Artif Intell Med, 100, pp. 101707-101704. , Sep;: [doi] [Medline: 31607347]; Morkel, RW, Mann, TN, Du Preez, G, Du Toit, J., Orthopaedic referrals using a smartphone app: uptake, response times and outcome (2019) S Afr Med J, 109 (11), p. 859. , Oct 31; [doi]; De La Cruz Monroy, MFI, Mosahebi, A., The use of smartphone applications (Apps) for enhancing communication with surgical patients: a systematic review of the literature (2019) Surg Innov, 26 (2), pp. 244-259. , Apr; [doi] [Medline: 30602332]; Sharif, FS, Rahman, A, Tonner, E, Ahmed, H, Haq, I, Abbass, R, Can technology optimise the pre-operative pathway for elective hip and knee replacement surgery: a qualitative study (2020) Perioper Med (Lond), 9 (1), p. 33. , Nov 16; [FREE Full _text] [doi] [Medline: 33292556]; Tang, M, Li, Z, Dai, Y, Li, X., What kind of a mobile health app do patients truly want? A pilot study among ambulatory surgery patients (2019) Patient Prefer Adherence, 13, pp. 2039-2046. , [FREE Full _text] [doi] [Medline: 31824139]; Datillo, JR, Gittings, DJ, Sloan, M, Hardaker, WM, Deasey, MJ, Sheth, NP., "Is There An App For That?" Orthopaedic patient preferences for a smartphone application (2017) Appl Clin Inform, 8 (3), pp. 832-844. , Aug 16; [FREE Full _text] [doi] [Medline: 28885637]; Michard, F, Gan, T, Kehlet, H., Digital innovations and emerging technologies for enhanced recovery programmes (2017) Br J Anaesth, 119 (1), pp. 31-39. , Jul 01; [FREE Full _text] [doi] [Medline: 28605474]; Tong, A, Sainsbury, P, Craig, J., Consolidated criteria

for reporting qualitative research (COREQ): a 32-item checklist for interviews and focus groups (2007) Int J Qual Health Care, 19 (6), pp. 349-357. , Dec; [doi] [Medline: 17872937]; Robinson, A, Oksuz, U, Slight, R, Slight, S, Husband, A., Digital and Mobile Technologies to Promote Physical Health Behavior Change and Provide Psychological Support for Patients Undergoing Elective Surgery: Meta-Ethnography and Systematic Review (2020) JMIR Mhealth Uhealth, 8 (12), p. e19237. , Dec 01; [FREE Full _text] [doi] [Medline: 33258787]; Robinson, A, Husband, AK, Slight, RD, Slight, SP., Digital support for patients undergoing bariatric surgery: narrative review of the roles and challenges of online forums (2020) JMIR Perioper Med, 3 (2), p. e17230. , Jul 15; [FREE Full _text] [doi] [Medline: 33393926]; Braun, V, Clarke, V., Reflecting on reflexive thematic analysis (2019) Qualitative Research in Sport, Exercise and Health, 11 (4), pp. 589-597. , Jun 13; [doi]; Braun, V, Clarke, V., What can "thematic analysis" offer health and wellbeing researchers? (2014) Int J Qual Stud Health Well-being, 9, p. 26152. , [FREE Full _text] [doi] [Medline: 25326092]; Cook, DJ, Thompson, JE, Prinsen, SK, Dearani, JA, Deschamps, C., Functional recovery in the elderly after major surgery: assessment of mobility recovery using wireless technology (2013) Ann Thorac Surg, 96 (3), pp. 1057-1061. , Sep; [doi] [Medline: 23992697]; Appelboom, G, Taylor, BE, Bruce, E, Bassile, CC, Malakidis, C, Yang, A, Mobile phone-connected wearable motion sensors to assess postoperative mobilization (2015) JMIR Mhealth Uhealth, 3 (3), p. e78. , Jul 28; [FREE Full _text] [doi] [Medline: 26220691]; Lyman, S, Hidaka, C, Fields, K, Islam, W, Mayman, D., Monitoring patient recovery after THA or TKA using mobile technology (2020) HSS J, 16, pp. 358-365. , Dec;(Suppl 2): [doi] [Medline: 33380968]; Mehta, N, Steiner, C, Fields, KG, Nawabi, DH, Lyman, SL., Using mobile tracking technology to visualize the trajectory of recovery after hip arthroscopy: a case report (2017) HSS J, 13 (2), pp. 194-200. , Jul; [FREE Full _text] [doi] [Medline: 28690471]; Michie, S, Whittington, C, Hamoudi, Z, Zarnani, F, Tober, G, West, R., Identification of behaviour change techniques to reduce excessive alcohol consumption (2012) Addiction, 107 (8), pp. 1431-1440. , Aug; [doi] [Medline: 22340523]; Michie, S, van Stralen, MM, West, R., The behaviour change wheel: a new method for characterising and designing behaviour change interventions (2011) Implement Sci, 6, p. 42. , Apr 23;: [FREE Full _text] [doi] [Medline: 21513547]; Argent, R, Slevin, P, Bevilacqua, A, Neligan, M, Daly, A, Caulfield, B., Wearable sensor-based exercise biofeedback for orthopaedic rehabilitation: a mixed methods user evaluation of a prototype system (2019) Sensors (Basel), 19 (2), pp. 1-432. , Jan 21; [FREE Full _text] [doi] [Medline: 30669657]; Lee, M, Suh, D, Son, J, Kim, J, Eun, S, Yoon, B., Patient perspectives on virtual reality-based rehabilitation after knee surgery: importance of level of difficulty (2016) J Rehabil Res Dev, 53 (2), pp. 239-252. , [FREE Full _text] [doi] [Medline: 27149529]; Michie, S, Abraham, C, Whittington, C, McAteer, J, Gupta, S., Effective techniques in healthy eating and physical activity interventions: a meta-regression (2009) Health Psychol, 28 (6), pp. 690-701. , Nov; [doi] [Medline: 19916637]; Jessen, S, Mirkovic, J, Ruland, CM., Creating gameful design in mhealth: a participatory co-design approach (2018) JMIR Mhealth Uhealth, 6 (12), p. e11579. , Dec 14; [FREE Full _text] [doi] [Medline: 30552080]; Amann, J, Fiordelli, M, Scheel-Sailer, A, Brach, M, Rubinelli, S., Opportunities and challenges of a self-management app to support people with spinal cord injury in the prevention of pressure injuries: qualitative study (2020) JMIR Mhealth Uhealth, 8 (12), p. e22452. , Dec 09; [FREE Full _text] [doi] [Medline: 33295876]; Barsom, EZ, Jansen, M, Tanis, PJ, van de Ven, AWH, Blussé van Oud-Alblas, M, Buskens, CJ, Video consultation during follow up care: effect on quality of care and

patient- and provider attitude in patients with colorectal cancer (2021) Surg Endosc, 35 (3), pp. 1278-1287. , Mar; [doi] [Medline: 32198552]; Li, HL, Chan, YC, Huang, JX, Cheng, SW., Pilot study using telemedicine video consultation for vascular patients' care during the Covid-19 period (2020) Ann Vasc Surg, 68, pp. 76-82. , Oct;: [FREE Full _text] [doi] [Medline: 32562832]; Rabinovich, L, Molton, JS, Ooi, WT, Paton, NI, Batra, S, Yoong, J., Perceptions and acceptability of digital interventions among tuberculosis patients in cambodia: qualitative study of video-based directly observed therapy (2020) J Med Internet Res, 22 (7), p. e16856. , Jul 27; [FREE Full _text] [doi] [Medline: 32716309]; Hasselberg, M, Beer, N, Blom, L, Wallis, LA, Laflamme, L., Image-based medical expert teleconsultation in acute care of injuries. A systematic review of effects on information accuracy, diagnostic validity, clinical outcome, and user satisfaction (2014) PLoS One, 9 (6), p. e98539. , [FREE Full _text] [doi] [Medline: 24887257]; Vlahu-Gjorgievska, E, Nagapuri, S, Win, KT., Tele-monitoring technology as a tool for monitoring and management of patients with congestive heart failure (2019) Acad J Interdiscip Stud, 23, p. e1. , Feb 12;:. [doi]; Almathami, HKY, Win, KT, Vlahu-Gjorgievska, E., Barriers and facilitators that influence telemedicine-based, real-time, online consultation at patients' homes: systematic literature review (2020) J Med Internet Res, 22 (2), p. e16407. , Feb 20; [FREE Full _text] [doi] [Medline: 32130131]; Sahu, D, Rathod, V., Telerehabilitation during COVID 19: Shoulder rehabilitation: letter to the editor regarding Menendez et al: "Orthopedic surgery post COVID-19: an opportunity for innovation and transformation" (2020) J Shoulder Elbow Surg, 29 (10), pp. 1957-1958. , Oct; [FREE Full _text] [doi] [Medline: 32659460]; Cugelman, B., Gamification: what it is and why it matters to digital health behavior change developers (2013) JMIR Serious Games, 1 (1), p. e3. , Dec 12; [FREE Full _text] [doi] [Medline: 25658754]; Cotton, V, Patel, MS., Gamification use and design in popular health and fitness mobile applications (2019) Am J Health Promot, 33 (3), pp. 448-451. , Mar; [FREE Full _text] [doi] [Medline: 30049225]; Kontadakis, G, Chasiouras, D, Proimaki, D, Halkiadakis, M, Fyntikaki, M, Mania, K., Gamified platform for rehabilitation after total knee replacement surgery employing low cost and portable inertial measurement sensor node (2018) Multimed Tools Appl, 79 (5-6), pp. 3161-3188. , Sep 12; [doi]; Ryu, JH, Park, JW, Nahm, FS, Jeon, YT, Oh, AY, Lee, HJ, The effect of gamification through a virtual reality on preoperative anxiety in pediatric patients undergoing general anesthesia: a prospective, randomized, and controlled trial (2018) J Clin Med, 7 (9), p. 284. , Sep 17; [FREE Full _text] [doi] [Medline: 30227602]; Jessen, S, Mirkovic, J, Nes, LS., Mystrengths, a strengths-focused mobile health tool: participatory design and development (2020) JMIR Form Res, 4 (7), p. e18049. , Jul 24; [FREE Full _text] [doi] [Medline: 32706651]; King, D, Greaves, F, Exeter, C, Darzi, A., 'Gamification': influencing health behaviours with games (2013) J R Soc Med, 106 (3), pp. 76-78. , Mar; [FREE Full _text] [doi] [Medline: 23481424]; Lister, C, West, JH, Cannon, B, Sax, T, Brodegard, D., Just a fad? Gamification in health and fitness apps (2014) JMIR Serious Games, 2 (2), p. e9. , Aug 04; [FREE Full _text] [doi] [Medline: 25654660]; Mitchell, M, White, L, Lau, E, Leahey, T, Adams, MA, Faulkner, G., Evaluating the carrot rewards app, a population-level incentive-based intervention promoting step counts across two canadian provinces: quasi-experimental study (2018) JMIR Mhealth Uhealth, 6 (9), p. e178. , Sep 20; [FREE Full _text] [doi] [Medline: 30148712]; Elliott, M, Eck, F, Khmelev, E, Derlyatka, A, Fomenko, O., Physical activity behavior change driven by engagement with an incentive-based app: evaluating the impact of sweatcoin (2019) JMIR Mhealth Uhealth, 7 (7), p. e12445. , Jul 08; [FREE Full _text] [doi] [Medline: 31287064]; Hajat, C, Hasan, A, Subel, S, Noach, A.,

The impact of short-term incentives on physical activity in a UK behavioural incentives programme (2019) NPJ Digit Med, 2 (1), p. 91. , Sep 16; [FREE Full _text] [doi] [Medline: 31531396]; Fox, LG, Barnes, D., Boosterbuddy: using gamification as a compensatory strategy for motivational deficits (2016) Psychiatr Serv, 67 (1), pp. 141-142. , Jan; [doi] [Medline: 26725495]; Roepke, AM, Jaffee, SR, Riffle, OM, McGonigal, J, Broome, R, Maxwell, B., Randomized controlled trial of superbetter, a smartphone-based/internet-based self-help tool to reduce depressive symptoms (2015) Games Health J, 4 (3), pp. 235-246. , Jun; [doi] [Medline: 26182069]; Edwards, EA, Lumsden, J, Rivas, C, Steed, L, Edwards, LA, Thiyagarajan, A, Gamification for health promotion: systematic review of behaviour change techniques in smartphone apps (2016) BMJ Open, 6 (10), p. e012447. , Oct 04; [FREE Full text] [doi] [Medline: 27707829]; Oinas-Kukkonen, H, Harjumaa, M., Persuasive systems design: key issues, process model, and system features (2009) Commun Assoc Inf Syst, 24, p. e1. , [doi]; Parada, F, Martínez, V, Espinosa, HD, Bauer, S, Moessner, M., Using persuasive systems design model to evaluate (2020) Telemed J E Health, 26 (2), pp. 251-254. , Feb; [FREE Full _text] [doi] [Medline: 30668227]; Hassan, L, Xi, N, Gurkan, B, Koivisto, J, Hamari, J., Gameful self-regulation: a study on how gamified self-tracking features evoke gameful experiences Proceedings of the 53rd Hawaii International Conference on System Sciences. 2020 Presented at: 53rd Hawaii International Conference on System Sciences, pp. 1-10. , https://tinyurl.com/3yprrpa2, Jan 7-10, 2020; Maui, Hawaii [doi]; Asbjørnsen, RA, Wentzel, J, Smedsrød, ML, Hjelmesæth, J, Clark, MM, Solberg Nes, L, Identifying persuasive design principles and behavior change techniques supporting end user values and needs in ehealth interventions for long-term weight loss maintenance: qualitative study (2020) J Med Internet Res, 22 (11), p. e22598. , Nov 30; [FREE Full _text] [doi] [Medline: 33252347]; Kelders, SM, Kok, RN, Ossebaard, HC, Van Gemert-Pijnen, JEWC., Persuasive system design does matter: a systematic review of adherence to web-based interventions (2012) J Med Internet Res, 14 (6), p. e152. , Nov 14; [FREE Full _text] [doi] [Medline: 23151820]; Mirkovic, J, Kristjansdottir, OB, Stenberg, U, Krogseth, T, Stange, KC, Ruland, CM., Patient insights into the design of technology to support a strengths-based approach to health care (2016) JMIR Res Protoc, 5 (3), p. e175. , Aug 24; [FREE Full _text] [doi] [Medline: 27558951]; Robinson, A, Slight, R, Husband, A, Slight, S., The value of teachable moments in surgical patient care and the supportive role of digital technologies (2020) Perioper Med (Lond), 9, p. 2. , [FREE Full _text] [doi] [Medline: 32042404]; Grocott, MPW, Edwards, M, Mythen, MG, Aronson, S., Peri-operative care pathways: re-engineering care to achieve the 'triple aim' (2019) Anaesthesia, 74, pp. 90-99. , Jan 02; Suppl 1: [FREE Full _text] [doi] [Medline: 30604413]; Levy, N, Grocott, MPW, Carli, F., Patient optimisation before surgery: a clear and present challenge in peri-operative care (2019) Anaesthesia, 74, pp. 3-6. , Jan; Suppl 1: [FREE Full _text] [doi] [Medline: 30604412]; Güldenpfennig, F, Mayer, P, Panek, P, Fitzpatrick, G., An autonomy-perspective on the design of assistive technology experiences of people with multiple sclerosis (2019) Proceedings of the 2019 CHI Conference on Human Factors in Computing Systems, p. e1. , Glasgow, Scotland Uk. May Presented at: CHI: Conference on Human Factors in Computing Systems; May 2019; United Kingdom [doi]; Calvo, RA, Peters, D, Johnson, D, Rogers, Y., Autonomy in technology design (2014) Proceedings of CHI'14 Extended Abstracts on Human Factors in Computing Systems, p. e1. , https://dl.acm.org/doi/proceedings/10.1145/2559206, Apr Presented at: CHI'14 Extended Abstracts on Human Factors in Computing Systems; April 2014; Toronto Ontario Canada [doi]; Burton, E, Clayville, K, Goldsmith, J, Mattei, N, The heart of the matter: patient autonomy as a model for the wellbeing of

technology users (2019) Proceedings of the 2019 AAAI/ACM Conference on AI, Ethics, and Society, , https://dl.acm.org/doi/proceedings/10.1145/3306618, editors Jan Presented at: AIES '19: Proceedings of the 2019 AAAI/ACM Conference on AI, Ethics, and Society; January 2019; Honolulu HI USA [doi]; Ruland, CM., Handheld technology to improve patient care: evaluating a support system for preference-based care planning at the bedside (2002) J Am Med Inform Assoc, 9 (2), pp. 192-201. , [FREE Full _text] [doi] [Medline: 11861634]; Solem, IKL, Varsi, C, Eide, H, Kristjansdottir, OB, Mirkovic, J, Børøsund, E, Patients' needs and requirements for ehealth pain management interventions: qualitative study (2019) J Med Internet Res, 21 (4), p. e13205. , Apr 01; [FREE Full _text] [doi] [Medline: 30877780]; Brewer, LC, Fortuna, KL, Jones, C, Walker, R, Hayes, SN, Patten, CA, Back to the future: achieving health equity through health informatics and digital health (2020) JMIR Mhealth Uhealth, 8 (1), p. e14512. , Jan 14; [FREE Full _text] [doi] [Medline: 31934874]; Menendez, ME, Jawa, A, Haas, DA, Warner, JJP, Orthopedic surgery post COVID-19: an opportunity for innovation and transformation (2020) J Shoulder Elbow Surg, 29 (6), pp. 1083-1086. , Codman Shoulder Society. Jun; [FREE Full _text] [doi] [Medline: 32312643]; Archibald, MM, Ambagtsheer, RC, Casey, MG, Lawless, M., Using zoom videoconferencing for qualitative data collection: perceptions and experiences of researchers and participants (2019) Int J Qual Methods, , Sep 11. [doi]; Lobe, B, Morgan, D, Hoffman, KA., Qualitative data collection in an era of social distancing (2020) Int J Qual Methods, , Jul 07. [doi]; Greenhalgh, T, Wherton, J, Shaw, S, Morrison, C., Video consultations for covid-19 (2020) Br Med J, 368, p. e1. , Mar 12;: [FREE Full _text] [doi] [Medline: 32165352]},
correspondence_address1={Slight, S.P.; School of Pharmacy, United Kingdom; email: Sarah.slight@newcastle.ac.uk},
publisher={JMIR Publications Inc.},
issn={14388871},
pubmed_id={33683208},
language={English},
abbrev_source_title={J. Med. Internet Res.},
document_type={Article},
source={Scopus},
}

@ARTICLE{Patel2021478,
author={Patel, M.L. and Wakayama, L.N. and Bennett, G.G.},
title={Self-Monitoring via Digital Health in Weight Loss Interventions: A Systematic Review Among Adults with Overweight or Obesity},
journal={Obesity},
year={2021},
volume={29},
number={3},
pages={478-499},
doi={10.1002/oby.23088},
note={cited By 0},
url={https://www.scopus.com/inward/record.uri?eid=2-s2.0-85101351971&doi=10.1002%2foby.23088&partnerID=40&md5=654f1d4975015ab84f6057d4296ef864},
affiliation={Stanford Prevention Research Center, Stanford University School of Medicine, Stanford, CA, United States; Integrated Care Psychology, San Francisco VA Health Care System, San Francisco, CA, United States; Department of Psychology and Neuroscience, Duke University, Durham, NC, United States; Duke Digital Health Science Center, Duke Global Health Institute, Durham, NC, United States},

abstract={Objective: Self-monitoring is a core component of behavioral obesity treatment, but it is unknown how digital health has been used for self-monitoring, what engagement rates are achieved in these interventions, and how self-monitoring and weight loss are related. Methods: This systematic review examined digital self-monitoring in behavioral weight loss interventions among adults with overweight or obesity. Six databases (PubMed, Embase, Scopus, PsycInfo, CINAHL, and ProQuest Dissertations & Theses) were searched for randomized controlled trials with interventions ≥ 12 weeks, weight outcomes ≥ 6 months, and outcomes on self-monitoring engagement and their relationship to weight loss. Results: Thirty-nine studies from 2009 to 2019 met inclusion criteria. Among the 67 interventions with digital self-monitoring, weight was tracked in 72% of them, diet in 81%, and physical activity in 82%. Websites were the most common self-monitoring modality, followed by mobile applications, wearables, electronic scales, and, finally, text messaging. Few interventions had digital self-monitoring engagement rates ≥ 75% of days. Rates were higher in digital- than in paper-based arms in 21 out of 34 comparisons and lower in just 2. Interventions with counseling had similar rates to standalone interventions. Greater digital self-monitoring was linked to weight loss in 74% of occurrences. Conclusions: Self-monitoring via digital health is consistently associated with weight loss in behavioral obesity treatment. © 2021 The Obesity Society},
references={Jensen, M.D., Ryan, D.H., Apovian, C.M., 2013 AHA/ACC/TOS guideline for the management of overweight and obesity in adults: a report of the American College of Cardiology/American Heart Association Task Force on practice guidelines and the Obesity Society (2014) Circulation, 129 (25), pp. S102-S138; Carver, C.S., Scheier, M.F., Control theory: a useful conceptual framework for personality–social, clinical, and health psychology (1982) Psychol Bull, 92, pp. 111-135; Bandura, A., Social cognitive theory of self-regulation (1991) Organ Behav Hum Decis Process, 50, pp. 248-287; Clark, M.M., Abrams, D.B., Niaura, R.S., Eaton, C.A., Rossi, J.S., Self-efficacy in weight management (1991) J Consult Clin Psychol, 59, pp. 739-744; Kitsantas, A., The role of self-regulation strategies and self-efficacy perceptions in successful weight loss maintenance (2000) Psychol Health, 15, pp. 811-820; Michie, S., Abraham, C., Whittington, C., McAteer, J., Gupta, S., Effective techniques in healthy eating and physical activity interventions: a meta-regression (2009) Health Psychol, 28, pp. 690-701; Harkin, B., Webb, T.L., Chang, B.P.I., Does monitoring goal progress promote goal attainment? A meta-analysis of the experimental evidence (2016) Psychol Bull, 142, pp. 198-229; Spring, B., Champion, K.E., Acabchuk, R., Hennessy, E.A., Self-regulatory behaviour change techniques in interventions to promote healthy eating, physical activity, or weight loss: a meta-review Health Psychol Rev, , [published online February 17, 2020]; Burke, L.E., Swigart, V., Warziski Turk, M., Derro, N., Ewing, L.J., Experiences of self-monitoring: successes and struggles during treatment for weight loss (2009) Qual Health Res, 19, pp. 815-828; Rosenbaum, D.L., Clark, M.H., Convertino, A.D., Call, C.C., Forman, E.M., Butryn, M.L., Examination of nutrition literacy and quality of self-monitoring in behavioral weight loss (2018) Ann Behav Med, 52, pp. 809-816; https://www.pewresearch.org/internet/fact-sheet/mobile/, Published June 12, 2019. Accessed October 12, 2020; One in five U.S. adults use health apps, wearable trackers, , https://news.gallup.com/poll/269096/one-five-adults-health-apps-wearable-trackers.aspx, Published December 11, 2019. Accessed October 12, 2020; Lanpher, M.G., Askew, S., Bennett, G.G., Health literacy and weight change in a digital health intervention for women: a randomized controlled trial in primary care practice

(2016) J Health Commun, 21, pp. 34-42; Pirotta, S., Joham, A., Hochberg, L., Strategies to reduce attrition in weight loss interventions: a systematic review and meta-analysis (2019) Obes Rev, 20, pp. 1400-1412; Hales, C.M., Carroll, M.D., Fryar, C.D., Ogden, C.L., Prevalence of obesity and severe obesity among adults: United States, 2017–2018, , National Center for Health Statistics; 2020; World Health Organization, , https://www.who.int/en/news-room/fact-sheets/detail/obesity-and-overweight, Obesity and overweight. Published April 1, 2020. Accessed October 12, 2020; Bradley, L.E., Smith-Mason, C.E., Corsica, J.A., Kelly, M.C., Hood, M.M., Remotely delivered interventions for obesity treatment (2019) Curr Obes Rep, 8, pp. 354-362; Podina, I.R., Fodor, L.A., Critical review and meta-analysis of multicomponent behavioral e-health interventions for weight loss (2018) Health Psychol, 37, pp. 501-515; Schippers, M., Adam, P., Smolenski, D., Wong, H., Wit, J., A meta-analysis of overall effects of weight loss interventions delivered via mobile phones and effect size differences according to delivery mode, personal contact, and intervention intensity and duration (2017) Obes Rev, 18, pp. 450-459; Skinner, R., Gonet, V., Currie, S., Hoddinott, P., Dombrowski, S.U., A systematic review with meta-analyses of text message-delivered behaviour change interventions for weight loss and weight loss maintenance (2020) Obes Rev, 21; Semper, H., Povey, R., Clark-Carter, D., A systematic review of the effectiveness of smartphone applications that encourage dietary self-regulatory strategies for weight loss in overweight and obese adults (2016) Obes Rev, 17, pp. 895-906; Dounavi, K., Tsoumani, O., Mobile health applications in weight management: a systematic literature review (2019) Am J Prev Med, 56, pp. 894-903; Fawcett, E., Van Velthoven, M.H., Meinert, E., Long-term weight management using wearable technology in overweight and obese adults: systematic review (2020) JMIR mHealth uHealth, 8; Yen, H.Y., Chiu, H.L., The effectiveness of wearable technologies as physical activity interventions in weight control: a systematic review and meta-analysis of randomized controlled trials (2019) Obes Rev, 20, pp. 1485-1493; Beleigoli, A.M., Andrade, A.Q., Cançado, A.G., Paulo, M.N., Diniz, M.D.F.H., Ribeiro, A.L., Web-based digital health interventions for weight loss and lifestyle habit changes in overweight and obese adults: systematic review and meta-analysis (2019) J Med Internet Res, 21; Burke, L.E., Wang, J., Sevick, M.A., Self-monitoring in weight loss: a systematic review of the literature (2011) J Am Diet Assoc, 111, pp. 92-102; Zheng, Y., Klem, M.L., Sereika, S.M., Danford, C.A., Ewing, L.J., Burke, L.E., Self-weighing in weight management: a systematic literature review (2015) Obesity (Silver Spring), 23, pp. 256-265; Pagoto, S., Waring, M.E., A call for a science of engagement: comment on Rus and Cameron (2016) Ann Behav Med, 50, pp. 690-691; Moher, D., Liberati, A., Tetzlaff, J., Altman, D.G., Preferred reporting items for systematic reviews and meta-analyses: the PRISMA statement (2009) Ann Intern Med, 151, pp. 264-269; Bennett, G.G., Steinberg, D.M., Stoute, C., Electronic health (eHealth) interventions for weight management among racial/ethnic minority adults: a systematic review (2014) Obes Rev, 15, pp. 146-158; Higgins, J.P.T., Thomas, J., Chandler, J., Cochrane Handbook for Systematic Reviews of Interventions, , www.training.cochrane.org/handbook, eds., version 6.0. Updated September 2020. Accessed October 12, 2020; Allen, J.K., Stephens, J., Dennison Himmelfarb, C.R., Stewart, K.J., Hauck, S., Randomized controlled pilot study testing use of smartphone technology for obesity treatment (2013) J Obes, 2013, p. 151597; Anton, S.D., LeBlanc, E., Allen, H.R., Use of a computerized tracking system to monitor and provide feedback on dietary goals for calorie-restricted diets: the POUNDS LOST study (2012) J Diabetes Sci Technol, 6, pp. 1216-1225; Bennett, G.G., Steinberg, D., Askew, S., Effectiveness of an

app and provider counseling for obesity treatment in primary care (2018) Am J Prev Med, 55, pp. 777-786; Burke, L.E., Styn, M.A., Sereika, S.M., Using mHealth technology to enhance self-monitoring for weight loss: a randomized trial (2012) Am J Prev Med, 43, pp. 20-26; Conroy, M.B., Yang, K., Elci, O.U., Physical activity self-monitoring and weight loss: 6-month results of the SMART trial (2011) Med Sci Sports Exerc, 43, pp. 1568-1574; Turk, M.W., Elci, O.U., Wang, J., Self-monitoring as a mediator of weight loss in the SMART randomized clinical trial (2013) Int J Behav Med, 20, pp. 556-561; Wang, J., (2010) Social Problem Solving and Adherence to Self-Monitoring in Association With Changes in Weight and Cardiometabolic Risk Factors in a Behavioral Weight Loss Trial, , http://d-scholarship.pitt.edu/8870/, Dissertation., University of Pittsburgh; Wang, J., Sereika, S.M., Chasens, E.R., Ewing, L.J., Matthews, J.T., Burke, L.E., Effect of adherence to self-monitoring of diet and physical activity on weight loss in a technology-supported behavioral intervention (2012) Patient Prefer Adherence, 6, pp. 221-226; Carter, M.C., Burley, V.J., Cade, J.E., Weight loss associated with different patterns of self-monitoring using the mobile phone app My Meal Mate (2017) JMIR mHealth uHealth, 5; Crane, M.M., Lutes, L.D., Ward, D.F., Bowling, J.M., Tate, D.F., A randomized trial testing the efficacy of a novel approach to weight loss among men with overweight and obesity (2015) Obesity (Silver Spring), 23, pp. 2398-2405; Dunn, C.G., Turner-McGrievy, G.M., Wilcox, S., Hutto, B., Dietary self-monitoring through calorie tracking but not through a digital photography app is associated with significant weight loss: the 2SMART pilot study—a 6-month randomized trial (2019) J Acad Nutr Diet, 119, pp. 1525-1532.; Turner-McGrievy, G.M., Dunn, C.G., Wilcox, S., Defining adherence to mobile dietary self-monitoring and assessing tracking over time: tracking at least two eating occasions per day is best marker of adherence within two different mobile health randomized weight loss interventions (2019) J Acad Nutr Diet, 119, pp. 1516-1524; Goldstein, S.P., Goldstein, C.M., Bond, D.S., Raynor, H.A., Wing, R.R., Thomas, J.G., Associations between self-monitoring and weight change in behavioral weight loss interventions (2019) Health Psychol, 38, pp. 1128-1136; Harvey, J., Krukowski, R., Priest, J., West, D., Log often, lose more: electronic dietary self-monitoring for weight loss (2019) Obesity (Silver Spring), 27, pp. 380-384; Hutchesson, M.J., Tan, C.Y., Morgan, P., Callister, R., Collins, C., Enhancement of self-monitoring in a web-based weight loss program by extra individualized feedback and reminders: randomized trial (2016) J Med Internet Res, 18; Jakicic, J.M., Davis, K.K., Rogers, R.J., Effect of wearable technology combined with a lifestyle intervention on long-term weight loss: the IDEA randomized clinical trial (2016) JAMA, 316, pp. 1161-1171; Jospe, M.R., Roy, M., Brown, R.C., The effect of different types of monitoring strategies on weight loss: a randomized controlled trial (2017) Obesity (Silver Spring), 25, pp. 1490-1498; Krukowski, R.A., Harvey-Berino, J., Bursac, Z., Ashikaga, T., West, D.S., Patterns of success: online self-monitoring in a web-based behavioral weight control program (2013) Health Psychol, 32, pp. 164-170; Leahey, T.M., Thomas, G., Fava, J.L., Adding evidence-based behavioral weight loss strategies to a statewide wellness campaign: a randomized clinical trial (2014) Am J Public Health, 104, pp. 1300-1306; Unick, J.L., Leahey, R., Kent, K., Wing, R.R., Examination of whether early weight loss predicts 1-year weight loss among those enrolled in an Internet-based weight loss program (2015) Int J Obes, 39, pp. 1558-1560. , (Lond); Leahey, T.M., Subak, L.L., Fava, J., Benefits of adding small financial incentives or optional group meetings to a web-based statewide obesity initiative (2015) Obesity (Silver Spring), 23, pp. 70-76; Lin, P.-H., Wang, Y., Levine, E., A text messaging-assisted randomized lifestyle weight loss clinical trial among overweight adults in Beijing (2014)

Obesity (Silver Spring), 22, pp. E29-E37; Lin, P.-H., Grambow, S., Intille, S., The association between engagement and weight loss through personal coaching and cell phone interventions in young adults: randomized controlled trial (2018) JMIR mHealth uHealth, 6; Ma, J., Xiao, L., Blonstein, A.C., Measurement of self-monitoring web technology acceptance and use in an e-health weight-loss trial (2013) Telemed J E Health, 19, pp. 739-745; Melchart, D., Wuhr, E., Weidenhammer, W., High and low responders in a comprehensive lifestyle program for weight loss - secondary analysis of a randomized controlled trial (2017) Compl Med Res, 24, pp. 232-239; Morgan, P.J., Lubans, D.R., Collins, C.E., Warren, J.M., Callister, R., The SHED-IT randomized controlled trial: evaluation of an internet-based weight-loss program for men (2009) Obesity (Silver Spring), 17, pp. 2025-2032; Morgan, P.J., Lubans, D.R., Collins, C.E., Warren, J.M., Callister, R., 12-month outcomes and process evaluation of the SHED-IT RCT: an internet-based weight loss program targeting men (2011) Obesity (Silver Spring), 19, pp. 142-151; Morgan, P.J., Callister, R., Collins, C.E., The SHED-IT community trial: a randomized controlled trial of internet-and paper-based weight loss programs tailored for overweight and obese men (2013) Ann Behav Med, 45, pp. 139-152; Nezami, B.T., Ward, D.S., Lytle, L.A., Ennett, S.T., Tate, D.F., A mHealth randomized controlled trial to reduce sugar-sweetened beverage intake in preschool-aged children (2018) Pediatr Obes, 13, pp. 668-676; Nezami, B.T., (2016) Healthy Weight Behaviors and Weight Change in Parents and Preschool-Aged Children, , https://cdr.lib.unc.edu/concern/dissertations/1831ck42z, Dissertation., The University of North Carolina at Chapel Hill, Accessed October 12, 2020; Patel, M.L., Hopkins, C.M., Brooks, T.L., Bennett, G.G., Comparing self-monitoring strategies for weight loss in a smartphone app: randomized controlled trial (2019) JMIR mHealth uHealth, 7; Patel, M.L., (2018) Comparing Self-Monitoring Strategies for Weight Loss: Does Developing Mastery Before Diet Tracking Enhance Engagement?, , https://dukespace.lib.duke.edu/dspace/handle/10161/17513, Dissertation., Duke University, Accessed October 12, 2020; Patel, M.L., Brooks, T.L., Bennett, G.G., Consistent self-monitoring in a commercial app-based intervention for weight loss: results from a randomized trial (2020) J Behav Med, 43, pp. 391-401; Patel, M.L., Hopkins, C.M., Bennett, G.G., Early weight loss in a standalone mHealth intervention predicting treatment success (2019) Obes Sci Pract, 5, pp. 231-237; Pellegrini, C.A., Verba, S.D., Otto, A.D., Helsel, D.L., Davis, K.K., Jakicic, J.M., The comparison of a technology-based system and an in-person behavioral weight loss intervention (2012) Obesity (Silver Spring), 20, pp. 356-363; Pellegrini, C.A., (2010) The Comparison of a Technology-Based System and an In-Person Behavioral Weight Loss Intervention in Overweight and Obese Adults, , http://d-scholarship.pitt.edu/8870/, Dissertation., University of Pittsburgh, Accessed October 12, 2020; Richardson, C.R., Goodrich, D.E., Larkin, A.R., A comparative effectiveness trial of three walking self-monitoring strategies (2016) Transl J Am Coll Sports, 1, pp. 133-142. , Med; Ross, K.M., Wing, R.R., Impact of newer self-monitoring technology and brief phone-based intervention on weight loss: a randomized pilot study (2016) Obesity (Silver Spring), 24, pp. 1653-1659; Shapiro, J.R., Koro, T., Doran, N., Text4Diet: a randomized controlled study using text messaging for weight loss behaviors (2012) Prev Med, 55, pp. 412-417; Shuger, S.L., Barry, V.W., Sui, X., Electronic feedback in a diet- and physical activity-based lifestyle intervention for weight loss: a randomized controlled trial (2011) Int J Behav Nutr Phys Act, 8, p. 41; Barry, V.W., (2011) An Assessment of Obesity Through Lifestyle Self-Monitoring and Cardiorespiratory Fitness, , https://scholarcommons.sc.edu/etd/1194/, Dissertation., University of South

Carolina, Accessed October 12, 2020; Spring, B., Pellegrini, C.A., Pfammatter, A., Effects of an abbreviated obesity intervention supported by mobile technology: the ENGAGED randomized clinical trial (2017) Obesity (Silver Spring), 25, pp. 1191-1198; Steinberg, D.M., (2012) Effect of Daily Self-Weighing on Weight Loss in Adults, , https://cdr.lib.unc.edu/concern/dissertations/z316q2047, Dissertation., The University of North Carolina at Chapel Hill; Steinberg, D.M., Bennett, G.G., Askew, S., Tate, D.F., Weighing every day matters: daily weighing improves weight loss and adoption of weight control behaviors (2015) J Acad Nutr Diet, 115, pp. 511-518; Steinberg, D.M., Levine, E.L., Askew, S., Foley, P., Bennett, G.G., Daily text messaging for weight control among racial and ethnic minority women: randomized controlled pilot study (2013) J Med Internet Res, 15; Thomas, J.G., Leahey, T.M., Wing, R.R., An automated internet behavioral weight-loss program by physician referral: a randomized controlled trial (2015) Diabetes Care, 38, pp. 9-15; Thomas, J.G., Raynor, H.A., Bond, D.S., Weight loss and frequency of body-weight self-monitoring in an online commercial weight management program with and without a cellular-connected ‘smart’ scale: a randomized pilot study (2017) Obes Sci Pract, 3, pp. 365-372; Thomas, J.G., Raynor, H.A., Bond, D.S., Weight loss in Weight Watchers Online with and without an activity tracking device compared to control: a randomized trial (2017) Obesity (Silver Spring), 25, pp. 1014-1021; Turner-McGrievy, G.M., Beets, M.W., Moore, J.B., Kaczynski, A.T., Barr-Anderson, D.J., Tate, D.F., Comparison of traditional versus mobile app self-monitoring of physical activity and dietary intake among overweight adults participating in an mHealth weight loss program (2013) J Am Med Inform Assoc, 20, pp. 513-518; Turner-McGrievy, G.M., Wilcox, S., Boutté, A., The dietary intervention to enhance tracking with mobile devices (DIET Mobile) study: a 6-month randomized weight-loss trial (2017) Obesity (Silver Spring), 25, pp. 1336-1342; Watson, S., Woodside, J.V., Ware, L.J., Effect of a web-based behavior change program on weight loss and cardiovascular risk factors in overweight and obese adults at high risk of developing cardiovascular disease: randomized controlled trial (2015) J Med Internet Res, 17; Wipfli, B., Hanson, G., Anger, K., Process evaluation of a mobile weight loss intervention for truck drivers (2019) Saf Health Work, 10, pp. 95-102; Wolin, K.Y., Steinberg, D.M., Lane, I.B., Engagement with eHealth self-monitoring in a primary care-based weight management intervention (2015) PLoS One, 10; Silberman, J.M., Kaur, M., Sletteland, J., Venkatesan, A., Outcomes in a digital weight management intervention with one-on-one health coaching (2020) PLoS One, 15; Painter, S.L., Lu, W., Schneider, J., James, R., Shah, B., Drivers of weight loss in a CDC-recognized digital diabetes prevention program (2020) BMJ Open Diabetes Res Care, 8; Neriah, D.B., Geliebter, A., Weight loss following use of a smartphone food photo feature: retrospective cohort study (2019) JMIR mHealth uHealth, 7; Gordon, M.L., Althoff, T., Leskovec, J., Goal-setting and achievement in activity tracking apps: a case study of MyFitnessPal (2019) Proc Int World Wide Web Conf, pp. 571-582; Jacobs, S., Radnitz, C., Hildebrandt, T., Adherence as a predictor of weight loss in a commonly used smartphone application (2017) Obes Res Clin Pract, 11, pp. 206-214; Tate, D., Crane, M., Espeland, M., Gorin, A., LaRose, J., Wing, R., Sustaining eHealth engagement in a multi-year weight gain prevention intervention (2019) Obes Sci Pract, 5, pp. 103-110; Anderson, M., Mobile technology and home broadband 2019. Pew Research Center: Internet, Science & Tech, , https://www.pewresearch.org/internet/2019/06/13/mobile-technology-and-home-broadband-2019/, Published June 13, 2019; Hales, C.M., Fryar, C.D., Carroll, M.D., Freedman, D.S., Aoki, Y., Ogden, C.L., Differences in obesity prevalence by

demographic characteristics and urbanization level among adults in the United States, 2013-2016 (2018) JAMA, 319, pp. 2419-2429; Rosenbaum, D., Piers, A., Schumacher, L., Kase, C., Butryn, M., Racial and ethnic minority enrollment in randomized clinical trials of behavioural weight loss utilizing technology: a systematic review (2017) Obes Rev, 18, pp. 808-817; Silver, L., Smartphone ownership is growing rapidly around the world, but not always equally, , https://www.pewresearch.org/global/2019/02/05/smartphone-ownership-is-growing-rapidly-around-the-world-but-not-always-equally/, PEW Research Center Global Attitudes & Trends Website. Published February 5, 2019. Accessed October 12, 2020; Perski, O., Blandford, A., West, R., Michie, S., Conceptualising engagement with digital behaviour change interventions: a systematic review using principles from critical interpretive synthesis (2017) Transl Behav Med, 7, pp. 254-267; Sieverink, F., Kelders, S.M., van Gemert-Pijnen, J.E., Clarifying the concept of adherence to eHealth technology: systematic review on when usage becomes adherence (2017) J Med Internet Res, 19; Butryn, M.L., Godfrey, K.M., Martinelli, M.K., Roberts, S.R., Forman, E.M., Zhang, F., Digital self-monitoring: does adherence or association with outcomes differ by self-monitoring target? (2020) Obes Sci Pract, 6, pp. 126-133; Darling, K.E., Sato, A.F., Systematic review and meta-analysis examining the effectiveness of mobile health technologies in using self-monitoring for pediatric weight management (2017) Child Obes, 13, pp. 347-355; Ho, T.J.H., Lee, C.C.S., Wong, S.N., Lau, Y., Internet-based self-monitoring interventions for overweight and obese adolescents: a systematic review and meta-analysis (2018) Int J Med Inform, 120, pp. 20-30; Collins, L.M., (2018) Optimization of Behavioral, Biobehavioral, and Biomedical Interventions: The Multiphase Optimization Strategy (MOST), , Springer},
correspondence_address1={Patel, M.L.; Stanford Prevention Research Center, United States; email: michele.patel@stanford.edu},
publisher={Blackwell Publishing Inc.},
issn={19307381},
pubmed_id={33624440},
language={English},
abbrev_source_title={Obesity},
document_type={Review},
source={Scopus},
}

@ARTICLE{Wilson-Barnes202177,
author={Wilson-Barnes, S. and Gymnopoulos, L.P. and Dimitropoulos, K. and Solachidis, V. and Rouskas, K. and Russell, D. and Oikonomidis, Y. and Hadjidimitriou, S. and María Botana, J. and Brkic, B. and Mantovani, E. and Gravina, S. and Telo, G. and Lalama, E. and Buys, R. and Hassapidou, M. and Balula Dias, S. and Batista, A. and Perone, L. and Bryant, S. and Maas, S. and Cobello, S. and Bacelar, P. and Lanham-New, S.A. and Hart, K.},
title={PeRsOnalised nutriTion for hEalthy livINg: The PROTEIN project},
journal={Nutrition Bulletin},
year={2021},
volume={46},
number={1},
pages={77-87},
doi={10.1111/nbu.12482},
note={cited By 0},
url={https://www.scopus.com/inward/record.uri?eid=2-s2.0-85101264999&doi=10.1111%2fnbu.12482&partnerID=40&md5=4b4c3d5a86609f35e94f199e02ee1e29},

affiliation={School of Biosciences and Medicine, Faculty of Health and Medical Sciences, University of Surrey, Guildford, United Kingdom; Centre for Research and Technology Hellas, Thessaloniki, Greece; OCADO Group, Hatfield, United Kingdom; Intrasoft International SA, Thessaloniki, Greece; Department of Electrical and Computer Engineering, Aristotle University of Thessaloniki, Thessaloniki, Greece; Grupo CMC, Madrid, Spain; BioSense Institute, Research and Development Institute for Information Technology, Vojvodina, Serbia; Research Group on Law, Science, Technology and Society, Vrije Universiteit Brussel, Brussels, Belgium; Datawizard, Rome, Italy; PLUX Wireless Biosignals, Lisbon, Portugal; Department of Endocrinology and Metabolic Diseases, Charité Universitätsmedizin, Berlin, Germany; Department of Rehabilitation Sciences, Katholieke Universiteit Leuven, Leuven, Belgium; Department of Nutrition and Dietetics, Alexander Technological Educational Institute of Thessaloniki, Thessaloniki, Greece; Faculdade de Motricidade Humana, Universidade de Lisboa, Lisbon, Portugal; Sport Lisboa e Benfica, Lisbon, Portugal; Fluviale srl, Rome, Italy; European Association for the Study of Obesity (EASO), Middlesex, United Kingdom; AgriFood Capital BV, Hertogenbosch, Netherlands; Polo Europeo della Conoscenza, Verona, Italy; Healthium/Nutrium Software, Porto e Região, Portugal},
abstract={Personalised nutrition is a novel public health strategy aiming to promote positive diet and lifestyle changes. Tailored dietary and physical activity advice may be more appropriate than a generalised ‘one-size-fits-all’ approach as it is more biologically relevant to the individual. Information and computing technology, smartphones and mobile applications have become an integral part of modern life and thereby present the opportunity for novel methods to encourage individuals to lead a healthier lifestyle. This article introduces the European Union-funded PROTEIN project (PeRsOnalised nutriTion for hEalthy livINg) consortium and introduces the associated work packages. The primary objective of the PROTEIN project is to produce a novel adaptable mobile application suite based on sound nutrition and physical activity advice from experts in their field, accessible to all population groups, with differing health outcomes, whose behaviour can be tracked with a variety of sensors and health hazard perception. The mobile application ‘ecosystem’ that will be developed by the consortium includes a platform, mobile suite, cloud services, artificial intelligence advisor, game suite, modelling of expert’s knowledge, users’ behaviour data collection, data analysis and a dashboard for healthcare professionals. It is proposed that users will find the provision of personalised nutrition advice and real-time data capture through a smartphone application useful, and importantly, will be encouraged by this to make positive health behaviour changes. © 2021 The Authors. Nutrition Bulletin published by John Wiley & Sons Ltd on behalf of British Nutrition Foundation},
author_keywords={healthy living; m-health; personalised nutrition; public health},
references={Ahlgren, J., Nordgren, A., Perrudin, M., Consumers on the Internet: ethical and legal aspects of commercialization of personalized nutrition (2013) Genes & Nutrition, 8, pp. 349-355; Barrea, L., Annunziata, G., Bordoni, L., Nutrigenetics – personalized nutrition in obesity and cardiovascular diseases (2020) International Journal of Obesity Supplements, 10, pp. 1-13; Celis-Morales, C., Livingstone, K.M., Marsaux, C.F.M., Effect of personalized nutrition on health-related behaviour change: evidence from the Food4Me European randomized controlled trial (2017) International Journal of Epidemiology, 46, pp. 578-588; Chiauzzi, E., Rodarte, C., DasMahapatra, P., Patient-centered activity monitoring in the self-management of chronic health conditions (2015) BMC Medicine, 13, p. 77; van Dijk, M.R., Huijgen, N.A.,

Willemsen, S.P., Impact of an mHealth platform for pregnancy on nutrition and lifestyle of the reproductive population: a survey (2016) JMIR mHealth and uHealth, 4; Dimitratos, S.M., German, J.B., Schaefer, S.E., Wearable Technology to Quantify the Nutritional Intake of Adults: Validation Study (2020) JMIR mHealth and uHealth, 8 (7). , http://dx.doi.org/10.2196/16405; (2017) Technical Report Dietary Reference Values for nutrients Summary report, , https://doi.org/10.1016/S0169-023X(97)00056-6; (2020) Smarter Pregnancy, , https://www.smarterpregnancy.co.uk/, (accessed 29 October 2020); Regulation (No 1047/2012) Nutrition and Health Claims [online], 2012 (2012) Official Journal of the European Union, , https://eur-lex.europa.eu/legal-content/EN/TXT/PDF/?uri=CELEX:32012R1047&rid=1, Available from, (accessed 21 October 2020); Follett, R., Strezov, V., An Analysis of Citizen Science Based Research: Usage and Publication Patterns (2015) PLOS ONE, 10 (11). , https://doi.org/10.1371/journal.pone.0143687; Free, C., Phillips, G., Felix, L., The effectiveness of M-health technologies for improving health and health services: a systematic review protocol (2010) BMC Research Notes, 3, pp. 1-7; Gardner, C.D., Trepanowski, J.F., Del Gobbo, L.C., Effect of low-fat VS low-carbohydrate diet on 12-month weight loss in overweight adults and the association with genotype pattern or insulin secretion the DIETFITS randomized clinical trial (2018) Journal of the American Medical Association, 319, pp. 667-679; Gibney, M.J., Walsh, M.C., The future direction of personalised nutrition: my diet, my phenotype, my genes (2013) Proceedings of the Nutrition Society, 72, pp. 219-225; Gibney, M., Walsh, M., Goosens, J., Personalized nutrition: paving the way to better population health (2016) Good nutrition: perspectives for the 21st century, pp. 235-248. , M Eggersdorfer, M Kraemer, &, JB Vordaro, eds), Basel, Karger Publishers; Graikos, A., Charisis, V., Iakovakis, D., Single image-based food volume estimation using monocular depth-prediction networks (2020) Universal Access in Human-Computer Interaction. Applications and Practice. HCII 2020. Lecture Notes in Computer Science, 12189; Guenther, P.M., Casavale, K.O., Reedy, J., Update of the Healthy Eating Index: HEI-2010 (2013) Journal of the Academy of Nutrition and Dietetics, 113, pp. 569-580; Hudson, G.M., Sprow, K., Promoting physical activity during the COVID-19 pandemic: implications for obesity and chronic disease management (2020) Journal of Physical Activity and Health, 17, pp. 685-687; Kho, A., Henderson, L.E., Dressler, D.D., Use of handheld computers in medical education: a systematic review (2006) Journal of General Internal Medicine, 21, pp. 531-537; Konstantinidis, D., Dimitropoulos, K., Ioakimidis, I., A deep network for automatic video-based food bite detection (2019) Lecture Notes in Computer Science (including subseries Lecture Notes in Artificial Intelligence and Lecture Notes in Bioinformatics), pp. 586-595. , A Hofmann, Berlin, Springer; Konstantinidis, D., Dimitropoulos, K., Langlet, B., Validation of a deep learning system for the full automation of bite and meal duration analysis of experimental meal videos (2020) Nutrients, 12, p. 209; Krishna, S., Boren, S.A., Balas, E.A., Healthcare via cell phones: a systematic review (2009) Telemedicine and e-Health, 15, pp. 231-240; Mathers, J.C., Paving the way to better population health through personalised nutrition (2019) EFSA Journal, 17; Mazahery, H., von Hurst, P.R., Factors affecting 25-hydroxyvitamin D concentration in response to vitamin D supplementation (2015) Nutrients, 7, pp. 5111-5142; Shill, J., Stephens, H., Uccellini, M., Evaluating mobile phone applications for health behaviour change: A systematic review (2018) Journal of Telemedicine and Telecare, 24 (1), pp. 22-30. , http://dx.doi.org/10.1177/1357633x16673538; Merino, J., Dashti, H.S., Li, S.X., Genome-wide meta-analysis of macronutrient intake of 91,114 European ancestry participants from the cohorts for heart and aging research in genomic

epidemiology consortium (2019) Molecular Psychiatry, 24, pp. 1920-1932. , https://doi.org/10.1038/s41380-018-0079-4; (2020) Health Survey for England: Fruit and vegetables, , http://healthsurvey.hscic.gov.uk/data-visualisation/data-visualisation/explore-the-trends/fruit-vegetables.aspx?type=adult, (accessed 20 October 2020); (2020) Around one million downloads of fitness app during lockdown as people stay fit, , https://www.england.nhs.uk/2020/07/around-one-million-downloads-of-fitness-app-during-lockdown-as-people-stay-fit/, (accessed 13 October 2020); Ordovas, J.M., Ferguson, L.R., Tai, E.S., Personalised nutrition and health (2018) British Medical Journal, 361, p. bmj.k2173; Patel, M.S., Asch, D.A., Volpp, K.G., Wearable devices as facilitators, not drivers, of health behavior change (2015) Journal of the American Medical Association, 313, p. 459; (2016) Smartphone Ownership and Internet Usage Continues to Climb in Emerging Economies, , https://www.pewresearch.org/global/2016/02/22/smartphone-ownership-and-internet-usage-continues-to-climb-in-emerging-economies/, (accessed 21 October 2020); (2019) Physical activity: applying All Our Health, , https://www.gov.uk/government/publications/physical-activity-applying-all-our-health/physical-activity-applying-all-our-health, (accessed 13 October 2020); (2020) Health Profile for England 2019, , https://publichealthengland.exposure.co/health-profile-for-england-2019, (accessed 20 October 2020); (2020) Screen time stats: How your phone impacts your workday, , https://blog.rescuetime.com/screen-time-stats-2018/, (accessed 21 October 2020); (2016) Vitamin D and Health, , https://www.gov.uk/government/groups/scientific-advisory-committee-on-nutrition, London, Scientific Advisory Committee on Nutrition; Seshadri, D.R., Li, R.T., Voos, J.E., Wearable sensors for monitoring the internal and external workload of the athlete (2019) NPJ Digital Medicine, 2, p. 71; (2020) MyFitnessPal monthly iPhone downloads 2019, , https://www.statista.com/statistics/994866/myfitnesspal-monthly-downloads-iphone-great-britain/, (accessed 13 October 2020); Studer, R., Benjamins, V.R., Fensel, D., Knowledge engineering: principles and methods (1998) Data & Knowledge Engineering, 25, pp. 161-197; COVID-19: A new lens for non-communicable diseases (2020) The Lancet, 396, p. 649. , http://dx.doi.org/10.1016/s0140-6736(20)31856-0; Theodoridis, T., Solachidis, V., Dimitropoulso, K., (2020) A Cross-Modal Variational Framework for Food Image Analysis, pp. 3244-3248. , Abu Dhabi, United Arab Emirates, Institute of Electrical and Electronics Engineers (IEEE); van Dijk, M.R., Koster, M.P.H., Willemsen, S.P., Healthy preconception nutrition and lifestyle using personalized mobile health coaching is associated with enhanced pregnancy chance (2017) Reproductive BioMedicine Online, 35, pp. 453-460; Verma, M., Hontecillas, R., Tubau-Juni, N., Challenges in personalized nutrition and health (2018) Frontiers in Nutrition, 5, p. 117; Wang, H., Wang, L., Liu, C., Employee competitive attitude and competitive behavior promote job-crafting and performance: a two-component dynamic model (2018) Frontiers in Psychology, 9; (2011) mHealth, , https://www.who.int/goe/publications/goe_mhealth_web.pdf?, (accessed 13 October 2020); (2020) Coronavirus disease (COVID-19) Pandemic, , https://www.who.int/emergencies/diseases/novel-coronavirus-2019, (accessed 13 October 2020); Zhou, K., Fukuoka, P., Mintz, E., Evaluating Machine Learning–Based Automated Personalized Daily Step Goals Delivered Through a Mobile Phone App: Randomized Controlled Trial (2018) JMIR mHealth and uHealth, 6 (1). , http://dx.doi.org/10.2196/mhealth.9117; Zhang, M., Zhao, L.J., Zhou, Y., SNP rs11185644 of RXRA gene is identified for dose-response variability to vitamin D3 supplementation: a randomized clinical trial (2017) Scientific

Reports, 7, p. 40593. , http://dx.doi.org/10.1038/srep40593},
correspondence_address1={Wilson-Barnes, S.; School of Biosciences and Medicine, United Kingdom; email: s.wilson-barnes@surrey.ac.uk},
publisher={Blackwell Publishing Ltd},
issn={14719827},
coden={NBUUA},
language={English},
abbrev_source_title={Nutr. Bull.},
document_type={Article},
source={Scopus},
}

@ARTICLE{Hajna2021e111,
author={Hajna, S. and Sharp, S.J. and Cooper, A.J.M. and Williams, K.M. and van Sluijs, E.M.F. and Brage, S. and Griffin, S.J. and Sutton, S.},
title={Effectiveness of Minimal Contact Interventions: An RCT},
journal={American Journal of Preventive Medicine},
year={2021},
volume={60},
number={3},
pages={e111-e121},
doi={10.1016/j.amepre.2020.10.010},
note={cited By 0},
url={https://www.scopus.com/inward/record.uri?eid=2-s2.0-85100543093&doi=10.1016%2fj.amepre.2020.10.010&partnerID=40&md5=55da5c6fb1ac1fd48e2f285fdb8e6d72},
affiliation={MRC Epidemiology Unit, Institute of Metabolic Science, University of Cambridge School of Clinical Medicine, Cambridge, United Kingdom; Primary Care Unit, Department of Public Health and Primary Care, University of Cambridge School of Clinical Medicine, Cambridge, United Kingdom},
abstract={Introduction: Around 23% of adults worldwide are insufficiently active. Wearable devices paired with virtual coaching software could increase physical activity. The effectiveness of 3 minimal contact interventions (paper-based physical activity diaries, activity trackers, and activity trackers coupled with virtual coaching) in increasing physical activity energy expenditure and cardiorespiratory fitness were compared over 12 weeks among inactive adults. Methods: This was an open label, parallel-group RCT. Inactive adults (aged ≥18 years, N=488) were randomized to no intervention (Control; n=121), paper-based diary (Diary; n=124), activity tracker (Activity Band; n=122), or activity tracker plus virtual coaching (Activity Band PLUS; n=121) groups. Coprimary outcomes included 12-week changes in physical activity energy expenditure and fitness (May 2012–January 2014). Analyses were conducted in 2019–2020. Results: There were no differences between groups overall (physical activity energy expenditure: p=0.114, fitness: p=0.417). However, there was a greater increase in physical activity energy expenditure (4.21 kJ/kg/day, 95% CI=0.42, 8.00) in the Activity Band PLUS group than in the Diary group. There were also greater decreases in BMI and body fat percentage in the Activity Band PLUS group than in the Control group (BMI= −0.24 kg/m2, 95% CI= −0.45, −0.03; body fat= −0.48%, 95% CI= −0.88, −0.08) and in theActivity Band PLUS group than in the Diary group (BMI= −0.30 kg/m2, 95% CI= −0.50, −0.09; body fat= −0.57%, 95% CI= −0.97, −0.17). Conclusions: Coupling activity trackers with virtual coaching may facilitate increases in physical activity energy expenditure compared with a traditional paper-based physical activity diary intervention and improve some secondary outcomes compared with a traditional paper-based physical activity diary intervention or no intervention. Trial registration:

This study is registered at www.clinicaltrials.gov ISRCTN31844443. © 2020 American Journal of Preventive Medicine},
keywords={adult; Article; body fat; body mass; cardiorespiratory fitness; clinical effectiveness; controlled study; energy expenditure; female; human; male; middle aged; online system; physical activity; psychological aspect; randomized controlled trial; self monitoring; self report; social behavior},
references={Physical activity (2018), https://www.who.int/news-room/fact-sheets/detail/physical-activity, WHO Geneva, Switzerland (Accessed 19 October 2020); Lee, I.M., Shiroma, E.J., Lobelo, F., Effect of physical inactivity on major non-communicable diseases worldwide: an analysis of burden of disease and life expectancy (2012) Lancet, 380 (9838), pp. 219-229; Global recommendations on physical activity for health (2010), https://www.who.int/dietphysicalactivity/global-PA-recs-2010.pdf, WHO Geneva, Switzerland (Accessed 19 October 2020); Guthold, R., Stevens, G.A., Riley, L.M., Bull, F.C., Worldwide trends in insufficient physical activity from 2001 to 2016: a pooled analysis of 358 population-based surveys with 1•9 million participants [published correction appears in Lancet Glob Health. 2019;7(1):e36] (2018) Lancet Glob Health, 6 (10), pp. e1077-e1086; Sallis, J.F., Bull, F., Guthold, R., Progress in physical activity over the Olympic quadrennium (2016) Lancet, 388 (10051), pp. 1325-1336; Owen, N., Leslie, E., Salmon, J., Fotheringham, M.J., Environmental determinants of physical activity and sedentary behavior (2000) Exerc Sport Sci Rev, 28 (4), pp. 153-158; Hallal, P.C., Andersen, L.B., Bull, F.C., Global physical activity levels: surveillance progress, pitfalls, and prospects (2012) Lancet, 380 (9838), pp. 247-257; Sullivan, A.N., Lachman, M.E., Behavior change with fitness technology in sedentary adults: a review of the evidence for increasing physical activity (2017) Front Public Health, 4, p. 289; Henriksen, A., Haugen Mikalsen, M., Woldaregay, A.Z., Using fitness trackers and smartwatches to measure physical activity in research: analysis of consumer wrist-worn wearables (2018) J Med Internet Res, 20 (3), p. e110; Phillips, S.M., Cadmus-Bertram, L., Rosenberg, D., Buman, M.P., Lynch, B.M., Wearable technology and physical activity in chronic disease: opportunities and challenges (2018) Am J Prev Med, 54 (1), pp. 144-150; Allison, C., Smartwatches and hearables will fuel wearable growth, forecast says: the Apple watch is expected to lead the pack over the next five years (2018) Wareable, , https://www.wareable.com/smartwatches/smartwatches-hearables-ccs-insight-5782, (Accessed 7 April 2020); Lyons, E.J., Lewis, Z.H., Mayrsohn, B.G., Rowland, J.L., Behavior change techniques implemented in electronic lifestyle activity monitors: a systematic content analysis (2014) J Med Internet Res, 16 (8), p. e192; Bravata, D.M., Smith-Spangler, C., Sundaram, V., Using pedometers to increase physical activity and improve health: a systematic review (2007) JAMA, 298 (19), pp. 2296-2304; Coughlin, S.S., Stewart, J., Use of consumer wearable devices to promote physical activity: a review of health intervention studies (2016) J Environ Health Sci, 2 (6), pp. 1-6; Lewis, Z.H., Lyons, E.J., Jarvis, J.M., Baillargeon, J., Using an electronic activity monitor system as an intervention modality: a systematic review (2015) BMC Public Health, 15, p. 585; Dasgupta, K., Rosenberg, E., Joseph, L., Physician step prescription and monitoring to improve ARTERial health (SMARTER): a randomized controlled trial in patients with type 2 diabetes and hypertension (2017) Diabetes Obes Metab, 19 (5), pp. 695-704; Pellegrini, C.A., Verba, S.D., Otto, A.D., Helsel, D.L., Davis, K.K., Jakicic, J.M., The comparison of a technology-based system and an in-person behavioral weight loss intervention (2012) Obesity (Silver Spring), 20 (2), pp. 356-363; Ross, K.M., Wing, R.R., Impact of newer self-monitoring

technology and brief phone-based intervention on weight loss: a randomized pilot study (2016) Obesity (Silver Spring), 24 (8), pp. 1653-1659; Manini, T.M., Everhart, J.E., Patel, K.V., Daily activity energy expenditure and mortality among older adults (2006) JAMA, 296 (2), pp. 171-179; Thompson, A.M., Church, T.S., Janssen, I., Katzmarzyk, P.T., Earnest, C.P., Blair, S.N., Cardiorespiratory fitness as a predictor of cancer mortality among men with pre- diabetes and diabetes (2008) Diabetes Care, 31 (4), pp. 764-769; Sui, X., LaMonte, M.J., Laditka, J.N., Cardiorespiratory fitness and adiposity as mortality predictors in older adults (2007) JAMA, 298 (21), pp. 2507-2516; Cooper, A.J., Dearnley, K., Williams, K.M., Protocol for Get Moving: a randomised controlled trial to assess the effectiveness of three minimal contact interventions to promote fitness and physical activity in working adults (2015) BMC Public Health, 15, p. 296; Godin, G., Shephard, R.J., A simple method to assess exercise behavior in the community (1985) Can J Appl Sport Sci, 10 (3), pp. 141-146. , http://www.ncbi.nlm.nih.gov/pubmed/4053261; Kinmonth, A.L., Wareham, N.J., Hardeman, W., Efficacy of a theory-based behavioural intervention to increase physical activity in an at-risk group in primary care (ProActive UK): a randomised trial (2008) Lancet, 371 (9606), pp. 41-48; Hurling, R., Catt, M., Boni, M.D., Using internet and mobile phone technology to deliver an automated physical activity program: randomized controlled trial (2007) J Med Internet Res, 9 (2), p. e7; Esliger, D.W., Rowlands, A.V., Hurst, T.L., Catt, M., Murray, P., Eston, R.G., Validation of the GENEA accelerometer (2011) Med Sci Sport Exerc, 43 (6), pp. 1085-1093; (2020), http://www.icas.co.ao/global-eap/media/index.html, ICAS International. Accessed October 19; Verplanken, B., Orbell, S., Reflections on past behavior: a self-report index of habit strength 1 (2003) J Appl Soc Psychol, 33 (6), pp. 1313-1330; Gosling, S.D., Rentfrow, P.J., Swann, W.B., Jr, A very brief measure of the Big-Five personality domains (2003) J Res Pers, 37 (6), pp. 504-528; Ajzen, I., The Theory of Planned Behavior (1991) Organ Behav Hum Decis Process, 50 (2), pp. 179-211; Brage, S., Brage, N., Franks, P.W., Ekelund, U., Wareham, N.J., Reliability and validity of the combined heart rate and movement sensor actiheart (2005) Eur J Clin Nutr, 59 (4), pp. 561-570; Stegle, O., Fallert, S.V., MacKay, D.J., Brage, S., Gaussian process robust regression for noisy heart rate data (2008) IEEE Trans Bio med Eng, 55 (9), pp. 2143-2151; Brage, S., Ekelund, U., Brage, N., Hierarchy of individual calibration levels for heart rate and accelerometry to measure physical activity [published correction appears in J Appl Physiol. 2009;106(3):1026] (2007) J Appl Physiol, 103 (2), pp. 682-692; Brage, S., Brage, N., Franks, P.W., Branched equation modeling of simultaneous accelerometry and heart rate monitoring improves estimate of directly measured physical activity energy expenditure (2004) J Appl Physiol, 96 (1), pp. 343-351; Brage, S., Westgate, K., Franks, P.W., Estimation of free-living energy expenditure by heart rate and movement sensing: a doubly-labelled water study (2015) PLoS One, 10 (9); Tanaka, H., Monahan, K.D., Seals, D.R., Age-predicted maximal heart rate revisited (2001) J Am Coll Cardiol, 37 (1), pp. 153-156; Ware, J., Kosinski, M., Dewey, J., Gandek, B., How to Score and Interpret Single-Item Health Status Measures: A Manual for Users of the SF-8 Health Survey (2001), QualyMetric Boston, MA; Yiengprugsawan, V., Kelly, M., Tawatsupa, B., SF-8TM health survey. (2014) Encyclopedia of Quality of Life and Well-Being Research, , Springer Dordrecht, The Netherlands; Cohen, S., Kamarck, T., Mermelstein, R., A global measure of perceived stress (1983) J Health Soc Behav, 24 (4), pp. 385-396; Besson, H., Brage, S., Jakes, R.W., Ekelund, U., Wareham, N.J., Estimating physical activity energy expenditure, sedentary time, and physical activity intensity by self-report in adults (2010) Am J Clin Nutr, 91 (1), pp. 106-114; Rodríguez-Mañas, L., Féart,

C., Mann, G., Searching for an operational definition of frailty: a Delphi method based consensus statement: the frailty operative definition-consensus conference project (2013) J Gerontol A Biol Sci Med Sci, 68 (1), pp. 62-67; Gourlan, M., Bernard, P., Bortolon, C., Efficacy of theory-based interventions to promote physical activity. A meta-analysis of randomised controlled trials (2016) Health Psychol Rev, 10 (1), pp. 50-66; Kosma, M., Ellis, R., Cardinal, B.J., Bauer, J.J., McCubbin, J.A., The mediating role of intention and stages of change in physical activity among adults with physical disabilities: an integrative framework (2007) J Sport Exerc Psychol, 29 (1), pp. 21-38; Rhodes, R.E., Pfaeffli, L.A., Mediators of physical activity behaviour change among adult non-clinical populations: a review update (2010) Int J Behav Nutr Phys Act, 7, p. 37; Armitage, C.J., Can the theory of planned behavior predict the maintenance of physical activity (2005) Health Psychol, 24 (3), pp. 235-245; Hardeman, W., Kinmonth, A.L., Michie, S., Sutton, S., Theory of planned behaviour cognitions do not predict self-reported or objective physical activity levels or change in the ProActive trial (2011) Br J Health Psychol, 16 (1), pp. 135-150; Mok, A., Khaw, K.T., Luben, R., Wareham, N., Brage, S., Physical activity trajectories and mortality: population based cohort study (2019) BMJ, 365, p. l2323; Ekelund, U., Tarp, J., Steene-Johannessen, J., Dose–response associations between accelerometry measured physical activity and sedentary time and all cause mortality: systematic review and harmonised meta-analysis (2019) BMJ, 366, p. l4570; Jakicic, J.M., Davis, K.K., Rogers, R.J., Effect of wearable technology combined with a lifestyle intervention on long-term weight loss: the IDEA randomized clinical trial [published correction appears in JAMA. 2016;316(14):1498] (2016) JAMA, 316 (11), pp. 1161-1171; DiFrancisco-Donoghue, J., Jung, M.K., Stangle, A., Utilizing wearable technology to increase physical activity in future physicians: a randomized trial (2018) Prev Med Rep, 12, pp. 122-127; Thomas, J.G., Raynor, H.A., Bond, D.S., Weight loss in Weight Watchers Online with and without an activity tracking device compared to control: a randomized trial (2017) Obesity (Silver Spring), 25 (6), pp. 1014-1021; Britton, K.A., Massaro, J.M., Murabito, J.M., Kreger, B.E., Hoffmann, U., Fox, C.S., Body fat distribution, incident cardiovascular disease, cancer, and all-cause mortality (2013) J Am Coll Cardiol, 62 (10), pp. 921-925; Stiggelbout, M., Hopman-Rock, M., Crone, M., Lechner, L., Van Mechelen, W., Predicting older adults’ maintenance in exercise participation using an integrated social psychological model (2006) Health Educ Res, 21 (1), pp. 1-14; Martin, S.S., Feldman, D.I., Blumenthal, R.S., mActive: a randomized clinical trial of an automated mHealth intervention for physical activity promotion (2015) J Am Heart Assoc, 4 (11); Bickmore, T.W., Silliman, R.A., Nelson, K., A randomized controlled trial of an automated exercise coach for older adults (2013) J Am Geriatr Soc, 61 (10), pp. 1676-1683; Poirier, J., Bennett, W.L., Jerome, G.J., Effectiveness of an activity tracker- and internet-based adaptive walking program for adults: a randomized controlled trial (2016) J Med Internet Res, 18 (2), p. e34; Wang, J.B., Cadmus-Bertram, L.A., Natarajan, L., Wearable sensor/device (Fitbit One) and SMS text-messaging prompts to increase physical activity in overweight and obese adults: a randomized controlled trial (2015) Telemed J E Health, 21 (10), pp. 782-792; Kim, B.H., Glanz, K., Text messaging to motivate walking in older African Americans: a randomized controlled trial (2013) Am J Prev Med, 44 (1), pp. 71-75; Harris, T., Kerry, S.M., Limb, E.S., Effect of a primary care walking intervention with and without nurse support on physical activity levels in 45- to 75-year-olds: the Pedometer and Consultation Evaluation (PACE-UP) Cluster Randomised Clinical Trial (2017) PLoS Med, 14 (1); Harris, T., Kerry, S.M., Limb, E.S., Physical activity levels in adults and older adults 3–4 years after

pedometer-based walking interventions: long-term follow-up of participants from two randomised controlled trials in UK primary care (2018) PLoS Med, 15 (3); McDermott, M.M., Spring, B., Berger, J.S., Effect of a home-based exercise intervention of wearable technology and telephone coaching on walking performance in peripheral artery disease: the HONOR randomized clinical trial [published correction appears in JAMA. 2018;320(1):96] (2018) JAMA, 319 (16), pp. 1665-1676; Lynch, B.M., Nguyen, N.H., Moore, M.M., A randomized controlled trial of a wearable technology-based intervention for increasing moderate to vigorous physical activity and reducing sedentary behavior in breast cancer survivors: the ACTIVATE Trial (2019) Cancer, 125 (16), pp. 2846-2855; Maxwell-Smith, C., Cohen, P.A., Platell, C., Wearable Activity Technology And Action-Planning (WATAAP) to promote physical activity in cancer survivors: randomised controlled trial protocol (2018) Int J Clin Health Psychol, 18 (2), pp. 124-132; Cadmus-Bertram, L.A., Marcus, B.H., Patterson, R.E., Parker, B.A., Morey, B.L., Randomized trial of a Fitbit-based physical activity intervention for women (2015) Am J Prev Med, 49 (3), pp. 414-418; Little, P., Stuart, B., Hobbs, F.R., An internet-based intervention with brief nurse support to manage obesity in primary care (POWeR+): a pragmatic, parallel-group, randomised controlled trial (2016) Lancet Diabetes Endocrinol, 4 (10), pp. 821-828; Brickwood, K.J., Watson, G., O'Brien, J., Williams, A.D., Consumer-based wearable activity trackers increase physical activity participation: systematic review and meta-analysis (2019) JMIR MHealth UHealth, 7 (4), p. e11819; Liu, S., Brooks, D., Thomas, S.G., Eysenbach, G., Nolan, R.P., Effectiveness of user- and expert-driven web-based hypertension programs: an RCT (2018) Am J Prev Med, 54 (4), pp. 576-583; Lynch, C., Bird, S., Lythgo, N., Selva-Raj, I., Changing the physical activity behavior of adults with fitness trackers: a systematic review and meta-analysis (2020) Am J Health Promot, 34 (4), pp. 418-430; Heath, G.W., Parra, D.C., Sarmiento, O.L., Evidence-based intervention in physical activity: lessons from around the world (2012) Lancet, 380 (9838), pp. 272-281; Test, I.D., (2020), https://www.mayocliniclabs.com/test-catalog/Clinical+and+Interpretive/82510, NICOU: nicotine and metabolites, random, urine. Mayo Foundation for Medical Education and Research. Accessed July 6; Whitsett, T.L., Manion, C.V., Christensen, H.D., Cardiovascular effects of coffee and caffeine (1984) Am J Cardiol, 53 (7), pp. 918-922; Pharmacology of caffeine (2001) Caffeine for the Sustainment of Mental Task Performance: Formulations for Military Operations, , The National Academies Press Washington, DC; Gajewska, M., Worth, A., Urani, C., Briesen, H., Schramm, K.W., The acute effects of daily nicotine intake on heart rate - a toxicokinetic and toxicodynamic modelling study (2014) Regul Toxicol Pharmacol, 70 (1), pp. 312-324; Lefante, J.J., Jr, Harmon, G.N., Ashby, K.M., Barnard, D., Webber, L.S., Use of the SF-8 to assess health-related quality of life for a chronically ill, low-income population participating in the Central Louisiana Medication Access Program (CMAP) (2005) Qual Life Res, 14 (3), pp. 665-673},
correspondence_address1={Griffin, S.J.; Primary Care Unit, United Kingdom; email: profgp@medschl.cam.ac.uk},
publisher={Elsevier Inc.},
issn={07493797},
coden={AJPME},
pubmed_id={33612170},
language={English},
abbrev_source_title={Am. J. Prev. Med.},
document_type={Article},
source={Scopus},

}

@ARTICLE{KaplanSerin2021273,
author={Kaplan Serin, E. and Citlik Saritas, S.},
title={The Effect of the Transtheoretical Model Based Walking Exercise Training and Follow-Up on Improving Exercise Behavior and Metabolic Control in Patients with Type 2 Diabetes},
journal={Clinical Nursing Research},
year={2021},
volume={30},
number={3},
pages={273-284},
doi={10.1177/1054773820920487},
note={cited By 0},
url={https://www.scopus.com/inward/record.uri?eid=2-s2.0-85084524476&doi=10.1177%2f1054773820920487&partnerID=40&md5=a4adc5781174229c34d96e399180ccdd},
affiliation={Department of Nursing, School of Health, Munzur University, Tunceli, Turkey; Department of Nursing, Faculty of Health Science, Inonu University, Malatya, Turkey},
abstract={This study aims to determine the effects of transtheoretical model-based walking exercise training and follow-up on improving exercise behavior and metabolic control in patients with type 2 diabetes. This randomized controlled trial was conducted as a pre-test and post-test experimental model with 76 intervention and 76 control individuals. The intervention group received the transtheoretical model-based exercise training. Data were collected using patient identification form, transtheoretical model scales, pedometer and metabolic outcomes. Data were analyzed by Chi square, two-way ANOVA, Mauchly’s, Greenhouse-Geisser, Friedman, and McNamer test. The overall score means for the exercise change processes, decision-making balance and self-efficacy scales increased compared to the pre-test (p <.001). The mean HDL increased from 41.39 ± 10.35 to 49.18 ± 11.58, and average number of steps per day increased from 3264.31 ± 1933.03 to 5639.37 ± 2317.01. Consequently, this difference between the groups was significant (p <.05). © The Author(s) 2020.},
author_keywords={metabolic control; nurse; transtheoretical model; type 2 diabetes mellitus; walking exercise},
references={Andrés, A., Gómez, J., Saldaña, C., Challenges and applications of the transtheoretical model in patients with diabetes mellitus (2008) Disease Management & Health Outcomes, 16 (1), pp. 31-46. , https://doi.org/10.2165/00115677-200816010-00004; Araiza, P., Hewes, H., Gashetewa, C., Vella, C.A., Burge, M.R., Efficacy of a pedometer-based physical activity program on parameters of diabetes control in type 2 diabetes mellitus (2006) Metabolism: Clinical and Experimental, 55 (10), pp. 1382-1387. , https://doi.org/10.1016/j.metabol.2006.06.009; Avery, L., Flynn, D., van Wersch, A., Sniehotta, F.F., Trenell, M.I., Changing physical activity behavior in type 2 diabetes (2012) Diabetes Care, 35 (12), pp. 2681-2689. , https://doi.org/10.2337/dc11-2452; Ay, S., (2008) A Guide For Exercising Regulary (2004). Pro-Change Behavior Systems. Ways to Live Well: Regular Exercise Guide, , 1st ed., Atlı Printing; Ay, S., Temel, A.B., Adaptation of the transtheoretical model based exercise behavior change model scales on Turkish adults: Validity and reliability study (2015) İzmir University Medicine Journal, 1, pp. 29-45. , https://doi.org/10.18228/iütd.42535; Balducci, S., D’Errico, V., Haxhi, J., Sacchetti, M., Orlando, G., Cardelli, P., Vitale, M., Pugliese, G., Effect of a behavioral intervention strategy for adoption and

maintenance of a physically active lifestyle: The Italian diabetes and exercise study 2 (IDES_2): A randomized controlled trial (2017) Diabetes Care, 40 (11), pp. 1444-1452. , https://doi.org/10.2337/dc17-0594; Chen, S.M., Creedy, D., Lin, H.-S., Wollin, J., Effects of motivational interviewing intervention on self-management, psychological and glycemic outcomes in type 2 diabetes: A randomized controlled trial (2012) International Journal of Nursing Studies, 49 (6), pp. 637-644. , https://doi.org/10.1016/j.ijnurstu.2011.11.011; de Moura, B.P., Natali, A.J., Marins, J.C.B., Amorim, P.R.S., Different approaches of physical training used in the management of type 2 diabetes: A brief systematic review of randomised clinical trials (2011) The British Journal of Diabetes & Vascular Disease, 11 (4), pp. 210-216. , https://doi.org/10.1177/1474651411410578; Di Loreto, C., Fanelli, C., Lucidi, P., Murdolo, G., De Cicco, A., Parlanti, N., Ranchelli, A., Feo, P.D., Make your diabetic patients walk (2005) Diabetes Care, 28 (6), pp. 1295-1302. , https://doi.org/10.2337/diacare.28.6.1295; Di Loreto, C., Fanelli, C., Lucidi, P., Murdolo, G., De Cicco, A., Parlanti, N., Santeusanio, F., De Feo, P., Validation of a counseling strategy to promote the adoption and the maintenance of physical activity by type 2 diabetic subjects (2003) Diabetes Care, 26 (2), pp. 404-408. , https://doi.org/10.2337/diacare.26.2.404; Erol, S., Erdoğan, S., Application of Transtheoretic model for Improving and changing health behaviours (2007) Journal of Anatolia Nursing and Health Sciences, 10 (2), pp. 86-94. , https://doi.org/10.17049/ahsbd.26482; Gleeson-Kreig, J.M., Self-monitoring of physical activity effects on self-efficacy and behavior in people with type 2 diabetes (2006) The Diabetes Educator, 32 (1), pp. 69-77. , https://doi.org/10.1177/0145721705284285; Greef, K.D., Deforche, B., Tudor-Locke, C., Bourdeaudhuij, I.D., A cognitive-behavioural pedometer-based group intervention on physical activity and sedentary behaviour in individuals with type 2 diabetes (2010) Health Education Research, 25 (5), pp. 724-736. , https://doi.org/10.1093/her/cyq017; Hekim, M., The importance of physical activity in the prevention of type II diabetes, hypertension and obesity (2015) The Journal of International Social Research, 8 (38), pp. 1081-1086; Holmen, H., Wahl, A., Torbjørnsen, A., Jenum, A.K., Småstuen, M.C., Ribu, L., Stages of change for physical activity and dietary habits in persons with type 2 diabetes included in a mobile health intervention: The Norwegian study in renewing health (2016) BMJ Open Diabetes Research & Care, 4 (1). , https://doi.org/10.1136/bmjdrc-2016-000193, e000193; Hordern, M.D., Cooney, L.M., Beller, E.M., Prins, J.B., Marwick, T.H., Coombes, J.S., Determinants of changes in blood glucose response to short-term exercise training in patients with type 2 diabetes (2008) Clinical Science, 115 (9), pp. 273-281. , https://doi.org/10.1042/CS20070422; (2018) IBM SPSS Statistics 21 Documentation, , https://www.ibm.com/support/pages/ibm-spss-statistics-21-documentation, Retrieved October 29, 2019, from; (2015) IDF DIABETES ATLAS, 1. , http://www.diabetesatlas.org/resources/2015-atlas.html, 7th ed., (,). Retrieved from; Kim, Y.-H., Application of the transtheoretical model to identify psychological constructs influencing exercise behavior: A questionnaire survey (2007) International Journal of Nursing Studies, 44 (6), pp. 936-944. , https://doi.org/10.1016/j.ijnurstu.2006.03.008; Kirk, A.F., Barnett, J., Mutrie, N., Physical activity consultation for people with type 2 diabetes: Evidence and guidelines: (2007) Diabetic Medicine, 24 (8), pp. 809-816. , https://doi.org/10.1111/j.1464-5491.2007.02190.x; Kirk, A.F., Leese, G., Encouraging physical activity interventions among people with type 2 diabetes (2009) Journal of Diabetes Nursing, 13 (1), pp. 26-31; Kirk, A.F., MacMillan, F., Webster, N., Application of the transtheoretical model to physical activity

in older adults with type 2 diabetes and/or cardiovascular disease (2010) Psychology of Sport and Exercise, 11 (4), pp. 320-324. , https://doi.org/10.1016/j.psychsport.2010.03.001; Kirk, A.F., Mutrie, N., MacIntyre, P., Fisher, M., Increasing physical activity in people with type 2 diabetes (2003) Diabetes Care, 26 (4), pp. 1186-1192. , https://doi.org/10.2337/diacare.26.4.1186; Kirk, A.F., Mutrie, N., MacIntyre, P., Fisher, M., Effects of a 12-month physical activity counselling intervention on glycaemic control and on the status of cardiovascular risk factors in people with type 2 diabetes (2004) Diabetologia, 47 (5), pp. 821-832. , https://doi.org/10.1007/s00125-004-1396-5; Marcus, B.H., Rakowski, W., Rossi, J.S., Assessing motivational readiness and decision making for exercise (1992) Health Psychology: Official Journal of the Division of Health Psychology, American Psychological Association, 11 (4), pp. 257-261. , https://doi.org/10.1037//0278-6133.11.4.257, (, a; Marcus, B.H., Rossi, J.S., Selby, V.C., Niaura, R.S., Abrams, D.B., The stages and processes of exercise adoption and maintenance in a worksite sample (1992) Health Psychology: Official Journal of the Division of Health Psychology, American Psychological Association, 11 (6), pp. 386-395. , https://doi.org/10.1037//0278-6133.11.6.386, (, b; Marcus, B.H., Selby, V.C., Niaura, R.S., Rossi, J.S., Self-efficacy and the stages of exercise behavior change (1992) Research Quarterly for Exercise and Sport, 63 (1), pp. 60-66. , https://doi.org/10.1080/02701367.1992.10607557, (, c; Marwick, T.H., Hordern, M.D., Miller, T., Chyun, D.A., Bertoni, A.G., Blumenthal, R.S., Philippides, G., Rocchini, A., Exercise training for type 2 diabetes mellitus: Impact on cardiovascular risk: A scientific statement from the American heart association (2009) Circulation, 119 (25), pp. 3244-3262. , https://doi.org/10.1161/CIRCULATIONAHA.109.192521; Negri, C., Bacchi, E., Morgante, S., Soave, D., Marques, A., Menghini, E., Muggeo, M., Moghetti, P., Supervised walking groups to increase physical activity in type 2 diabetic patients (2010) Diabetes Care, 33 (11), pp. 2333-2335. , https://doi.org/10.2337/dc10-0877; (2014) Physical Activity Guide. 2014, 940. , http://fizikselaktivite.gov.tr/wp-content/uploads/FizikselAktiviteRehberiTR.pdf , 2014; Prochaska, J.O., DiClemente, C.C., Norcross, J.C., In search of how people change. Applications to addictive behaviors (1992) The American Psychologist, 47 (9), pp. 1102-1114. , https://doi.org/10.1037//0003-066x.47.9.1102; Sazlina, S.-G., Browning, C., Yasin, S., Interventions to promote physical activity in older people with type 2 diabetes mellitus: A systematic review (2013) Public Health Education and Promotion, 1, p. 71. , https://doi.org/10.3389/fpubh.2013.00071; Sigal, R.J., Kenny, G.P., Boulé, N.G., Wells, G.A., Prud'homme, D., Fortier, M., Reid, R.D., Jaffey, J., Effects of aerobic training, resistance training, or both on glycemic control in type 2 diabetes: A randomized trial (2007) Annals of Internal Medicine, 147 (6), pp. 357-369. , https://doi.org/10.7326/0003-4819-147-6-200709180-00005; Tosun, A.S., Zincir, H., Motivational interview method based on transtheoretical model of health behavior change in type 2 diabetes mellitus (2016) Current Approaches to Psychiatry, 8 (1), pp. 32-41. , https://doi.org/10.18863/pgy.57110; Tudor-Locke, C.E., Bell, R.C., Myers, A.M., Harris, S.B., Lauzon, N., Rodger, N.W., Pedometer-determined ambulatory activity in individuals with type 2 diabetes (2002) Diabetes Research and Clinical Practice, 55 (3), pp. 191-199. , https://doi.org/10.1016/S0168-8227(01)00317-5; Umpierre, D., Ribeiro, P.A.B., Kramer, C.K., Leitão, C.B., Zucatti, A.T.N., Azevedo, M.J., Gross, J.L., Schaan, B.D., Physical activity advice only or structured exercise training and association with HbA1c levels in type 2 diabetes: A systematic review and

meta-analysis (2011) The Journal of the American Medical Association, 305 (17), pp. 1790-1799. , https://doi.org/10.1001/jama.2011.576; Umpierre, D., Ribeiro, P.A.B., Schaan, B.D., Ribeiro, J.P., Volume of supervised exercise training impacts glycaemic control in patients with type 2 diabetes: A systematic review with meta-regression analysis (2012) Diabetologia, 56 (2), pp. 242-251. , https://doi.org/10.1007/s00125-012-2774-z; Waryasz, G.R., McDermott, A.Y., Exercise prescription and the patient with type 2 diabetes: A clinical approach to optimizing patient outcomes (2010) Journal of the American Academy of Nurse Practitioners, 22 (4), pp. 217-227. , https://doi.org/10.1111/j.1745-7599.2010.00490.x; Yanık, Y.T., Erol, Ö., Evaluation of self-efficacy levels of individuals with type 2 diabetes (2016) Journal of Anatolia Nursing and Health Sciences, 19 (3). , http://e-dergi.atauni.edu.tr/ataunihem/article/view/5000148066},
correspondence_address1={Kaplan Serin, E.; Department of Nursing, Turkey; email: emine_3354@hotmail.com},
publisher={SAGE Publications Inc.},
issn={10547738},
pubmed_id={32363944},
language={English},
abbrev_source_title={Clin. Nurs. Res.},
document_type={Article},
source={Scopus},
}

@ARTICLE{Wong2021547,
author={Wong, M.Y.C. and Chung, P.-K. and Leung, K.-M.},
title={The Relationship Between Physical Activity and Self-Compassion: a Systematic Review and Meta-analysis},
journal={Mindfulness},
year={2021},
volume={12},
number={3},
pages={547-563},
doi={10.1007/s12671-020-01513-4},
note={cited By 1},
url={https://www.scopus.com/inward/record.uri?eid=2-s2.0-85092352551&doi=10.1007%2fs12671-020-01513-4&partnerID=40&md5=f2cbb32432df7efcc1ec5fb3c57b120a},
affiliation={Hong Kong Baptist University, Hong Kong; The Education University of Hong Kong, Hong Kong},
abstract={Objectives: According to the mental health service recommendations from WHO, self-care is the basis for tackling psychological concerns. Although self-esteem has been a symbolic self-driven psychological factor that could generate significant positive well-being and associated with physical activity (PA), self-esteem’s negative effects have been criticized by the advocation of self-compassion (SC). Therefore, with both PA and SC have been found to be effective in achieving positive psychological health, they are expected to show a relationship to cope with people’s mental health problems. Methods: To fully study the relationship, a concrete systematic review and meta-analysis were conducted to review the relationship between PA and SC. The meta-analysis outcome was obtained by the pooled correlation coefficient using the random effects model, and meta-regression with mixed-effect models were used to determine the moderate effects. Results: A total of 25 studies, with n = 5622 samples, were included. Ten studies examined the association between PA and SC; five studies examined the association between PA regulation and SC; and six RCT

studies and four single group repeated measure studies examined the effect of PA on SC. The random effects model demonstrated a significant effect size on the overall relationship between PA and SC (r = 0.26), with significant heterogeneity. Subgroup analysis showed the significant effect sizes on four types of relationship, with r = 0.19, r = 0.273 and r = 0.34, r = 0.273, respectively. All potential sources of heterogeneity, including age, gender, study design and risk of bias, showed no moderating effect on the relationship. Conclusion: The relationship between PA and SC was significant while investigating the effect of non-mind-and-body interventions and constructing a mechanism of PA and SC can be done as future implications. © 2020, Springer Science+Business Media, LLC, part of Springer Nature.},
author_keywords={Physical activity; Regulated health-related behaviour; Self-compassion},
references={Arts-De Jong, M., Vanwesterop, L.L., Hoogerbrugge, N., Massuger, L.F., Maas, A.H., Vanbeek, M.H., Dehullu, J.A., Self-compassion, physical fitness and climacteric symptoms in oophorectomized BRCA1/2 mutation carriers (2018) Maturitas, 108, pp. 13-17. , https://doi.org/10.1016/j.maturitas.2017.11.002; Babenko, O., Mosewich, A., Abraham, J., Lai, H., Contributions of psychological needs, self-compassion, leisure-time exercise, and achievement goals to academic engagement and exhaustion in Canadian medical students (2018) Journal of Educational Evaluation for Health Professions, 15, p. 2. , https://doi.org/10.3352/jeehp.2018.15.2; Bluth, K., Bluth, K., Campo, R.A., Campo, R.A., Futch, W.S., Futch, W.S., Gaylord, S.A., Age and gender differences in the associations of self-compassion and emotional well-being in a large adolescent sample (2017) Journal of Youth and Adolescence, 46 (4), pp. 840-853. , …, (,).,., (,)., https://doi.org/10.1007/s10964-016-0567-2; Borenstein, M., Hedges, L.V., Higgins, J.P.T., Rothstein, H.R., (2009) Introduction to Meta-Analysis, , Oxford, Wiley; Borenstein, M., Hedges, L.V., Higgins, J.P., Rothstein, H.R., (2011) Introduction to Meta-Analysis, , Oxford, Wiley; Bosco, F.A., Aguinis, H., Singh, K., Field, J.G., Pierce, C.A., Correlational effect size benchmarks (2015) Journal of Applied Psychology, 100 (2), pp. 431-449; Braun, T.D., Park, C.L., Conboy, L.A., Psychological well-being, health behaviors, and weight loss among participants in a residential, Kripalu yoga-based weight loss program (2012) International Journal of Yoga Therapy, 22, pp. 9-22; Brown, S.A., Martin, E.E., Garcia, T.J., Winter, M.A., García, A.A., Brown, A., Cuevas, H.E., Sumlin, L.L., Managing complex research datasets using electronic tools: Ameta-analysis exemplar (2013) Computers, Informatics, Nursing: CIN, 31 (6), pp. 257-265. , https://doi.org/10.1097/NXN.0b013e318295e69c; Chen, J., Yan, L., Zhou, L., Reliability and validity of Chinese version of Self-Compassion Scale (2011) Chinese Journal of Clinical Psychology, 19 (6), pp. 734-736; Chen, Z., Zhang, G., Li, J., Goodness-of-fit test for meta-analysis (2015) Scientific Reports, 5 (1), p. 16983; Cohen, J., A power primer (1992) Psychological Bulletin, 112 (1), pp. 155-159; Conn, V.S., Depressive symptom outcomes of physical activity interventions: Meta-analysis findings (2010) Annals of Behavioral Medicine, 39 (2), pp. 128-138; Cox, A.E., Ullrich-French, S., Cole, A.N., D'Hondt-Taylor, M., The role of state mindfulness during yoga in predicting self-objectification and reasons for exercise (2016) Psychology of Sport and Exercise, 22, pp. 321-327; Cox, A.E., Ullrich-French, S., Tylka, T.L., McMahon, A.K., The roles of self-compassion, body surveillance, and body appreciation in predicting intrinsic motivation for physical activity: Cross-sectional associations, and prospective changes within a yoga context (2019) Body Image, 29, pp. 110-117. , https://doi.org/10.1016/j.bodyim.2019.03.002; Craig, C.L.,

Marshall, A.L., Sjostrom, M., Bauman, A.E., Booth, M.L., Ainsworth, B.E., Oja, P., International physical activity questionnaire: 12-country reliability and validity (2003) Medicine & Science in Sports & Exercise, 35 (8), pp. 1381-1395. , https://doi.org/10.1249/01.MSS.0000078924.61453.FB; Cramer, H., Anheyer, D., Lauche, R., Dobos, G., A systematic review of yoga for major depressive disorder (2017) Journal of Affective Disorders, 213, pp. 70-77; de Bruin, E.I., van Der Zwan, J.E., Bogels, S.M., A RCT comparing daily mindfulness meditations, biofeedback exercises, and daily physical exercise on attention control, executive functioning, mindful awareness, self-compassion, and worrying in stressed young adults (2016) Mindfulness, 7 (5), pp. 1182-1192. , https://doi.org/10.1007/s12671-016-0561-5; Décamps, G., Boujut, E., Brisset, C., French college students' sports practice and its relations with stress, coping strategies and academic success (2012) Frontiers in Psychology, 3, p. 104. , http://doi.org/10.3389/fpsyg.2012.00104; Derose, K.P., Han, B., Williamson, S., Cohen, D.A., Gender disparities in park use and physical activity among residents of high-poverty neighborhoods in los angeles (2018) Women's Health Issues, 28 (1), pp. 6-13. , &, (,).,., (,), -,., http://doi.org/10.1016/j.whi.2017.11.003; Duval, S., Tweedie, R., A nonparametric ‘trim and fill’ method of accounting for publication bias in meta-analysis (2000) Journal of the American Statistical Association, 95, pp. 89-98; Duval, S., Tweedie, R., Trim and fill: a simple funnel-plot-based method of testing and adjusting for publication bias in meta-analysis (2000) Biometrics, 56, pp. 455-463; Evers, J.L., Do we need an RCT for everything? (2017) Human Reproduction, 32 (3), pp. 483-484; Falsafi, N., A randomized controlled trial of mindfulness versus yoga: Effects on depression and/or anxiety in college students (2016) Journal of the American Psychiatric Nurses Association, 22 (6), pp. 483-497. , https://doi.org/10.1177/1078390316663307; Falsafi, N., Leopard, L., Pilot study: Use of mindfulness, self-compassion, and yoga practices with low-income and/or uninsured patients with depression and/or anxiety (2015) Journal of Holistic Nursing, 33 (4), pp. 289-297. , https://doi.org/10.1177/0898010115569351; (2017) Mental Health Review Report, , https://www.fhb.gov.hk/download/press_and_publications/otherinfo/180500_mhr/e_mhr_full_report.pdf, Accessed Apr 2020; Gilbertson, J.T., The relationship of self-regulation, exercise self-efficacy, and self-compassion with commitment to physical activity in college students (2017) Dissertation Abstracts International Section A: Humanities and Social Sciences, 78 (5). , http://ovidsp.ovid.com/ovidweb.cgi?T=JS&PAGE=reference&D=psyc14&NEWS=N&AN=2017-10858-284; Godin, G., Shephard, R.J., A simple method to assess exercise behavior in the community. Canadian Journal of Applied Sport Sciences (1985) Journal Canadien Des Sciences Appliquées Au Sport, 10 (3), pp. 141-146. , PID: 4053261; Hallion, M., Taylor, A., Roberts, R., Ashe, M., Exploring the association between physical activity participation and self-compassion in middle-aged adults (2019) Sport, Exercise, and Performance Psychology, 8 (3), pp. 305-316. , https://doi.org/10.1037/spy0000150; Harden, S.M., Steketee, A.M., Kelliher, R., Mason, K.A., Fitzwater Boyle, N., Using a studio-academic partnership to advance public health within a pragmatic yoga setting (2019) Journal of Primary Care and Community Health, 10. , https://doi.org/10.1177/2150132719874621; Hariton, E., Locascio, J.J., Randomised controlled trials - the gold standard for effectiveness research: study design: randomised controlled trials (2018) BJOG: An International Journal of Obstetrics and Gynaecology, 125 (13), p. 1716; Hendriks, T., de Jong, J., Cramer, H., The effects of yoga on positive mental health among healthy adults: a systematic review and meta-analysis (2017) The Journal of Alternative and Complementary Medicine, 23 (7), pp. 55-517; Higgins, J.P.,

Green, S., (2011) Cochrane Handbook for Systematic Reviews of Interventions, 4. , Oxford, Cochrane Collaboration; Hlatky, M.A., Boineau, R.E., Higginbotham, M.B., Lee, K.L., Mark, D.B., Califf, R.M., Pryor, D.B., A brief self-administered questionnaire to determine functional capacity (the Duke Activity Status Index) (1989) The American Journal of Cardiology, 64 (10), pp. 651-654. , …, (,).,., (,)., https://doi.org/10.1016/0002-9149(89)90496-7; Homan, K.J., Sirois, F.M., Self-compassion and physical health: Exploring the roles of perceived stress and health-promoting behaviors (2017) Health Psychology Open, 4 (2). , https://doi.org/10.1177/2055102917729542; Horan, K.A., Taylor, M.B., Mindfulness and self-compassion as tools in health behavior change: An evaluation of a workplace intervention pilot study (2018) Journal of Contextual Behavioral Science, 8, pp. 8-16. , https://doi.org/10.1016/j.jcbs.2018.02.003; Huberty, J., Green, J., Glissmann, C., Larkey, L., Puzia, M., Lee, C., Efficacy of the mindfulness meditation mobile app “calm” to reduce stress among college students: Randomized controlled trial (2019) JMIR Mhealth and Uhealth, 7 (6). , https://doi.org/10.2196/14273; Jazaieri, H., Goldin, P.R., Werner, K., Ziv, M., Gross, J.J., A randomized trial of MBSR versus aerobic exercise for social anxiety disorder (2012) Journal of Clinical Psychology, 68 (7), pp. 715-731. , https://doi.org/10.1002/jclp.21863; Jewett, R., Sabiston, C.M., Brunet, J., O’Loughlin, E.K., Scarapicchia, T., O'Loughlin, J., School sport participation during adolescence and mental health in early adulthood (2014) Journal of Adolescent Health, 55 (5), pp. 640-644; Kiernan, M., Schoffman, D.E., Lee, K., Brown, S.D., Fair, J.M., Perri, M.G., Haskell, W.L., The Stanford Leisure-Time Activity Categorical Item (L-Cat): a single categorical item sensitive to physical activity changes in overweight/obese women (2013) International Journal of Obesity (2005), 37 (12), pp. 1597-1602; Killham, M.E., Mosewich, A.D., Mack, D.E., Gunnell, K.E., Ferguson, L.J., Women athletes’ self-compassion, self-criticism, and perceived sport performance (2018) Sport, Exercise, and Performance Psychology, 7 (3), p. 297; Kohut, H., (1977) The Restoration of the Self, , New York, International Universities Press; Kolbe, L.J., Kann, L., Collins, J.L., Overview of the youth risk behavior surveillance system (1993) Public Health Reports, 108 (1), p. 2. , PID: 8210269; Li, Y., Deng, J., Lou, X., Wang, H., Wang, Y., A daily diary study of the relationships among daily self-compassion, perceived stress and health-promoting behaviours (2019) International Journal of Psychology, 55 (3), pp. 364-372. , https://doi.org/10.1002/ijop.12610; Lopez, A., Sanderman, R., Ranchor, A.V., Schroevers, M.J., Compassion for others and self-compassion: levels, correlates, and relationship with psychological well-being (2018) Mindfulness, 9 (1), pp. 325-331; Lowry, R.G., Waller, K., (2018) Self-Compassion and Self-Determined Motivation Towards Exercise: An Exploration Or Gender and Age Differences of Regular Exercisers. European College of Sports Science, , http://eprints.chi.ac.uk/id/eprint/3892, 4–7 July 2018, Dublin, Republic of Ireland; Lu, C., Stolk, R.P., Sauer, P.J.J., Sijtsma, A., Wiersma, R., Huang, G., Corpeleijn, E., Factors of physical activity among Chinese children and adolescents: a systematic review (2017) The International Journal of Behavioral Nutrition and Physical Activity, 14 (1), p. 36; Lubans, D., Richards, J., Hillman, C., Faulkner, G., Beauchamp, M., Nilsson, M., Biddle, S., Physical activity for cognitive and mental health in youth: A systematic review of mechanisms (2016) Pediatrics, 138 (3). , https://doi.org/10.1542/peds.2016-1642; Magnus, C.M.R., Kowalski, K.C., McHugh, T.L.F., The role of self-compassion in women’s self-determined motives to exercise and exercise-related outcomes (2010) Self and Identity, 9 (4), pp. 363-382. , https://doi.org/10.1080/15298860903135073; Markland, D., Tobin, V.,

A modification to the behavioural regulation in exercise questionnaire to include an assessment of a motivation (2004) Journal of Sport and Exercise Psychology, 26 (2), pp. 191-196; Marsh, I.C., Chan, S.W., MacBeth, A., Self-compassion and psychological distress in adolescents—a meta-analysis (2018) Mindfulness, 9 (4), pp. 1011-1027; Mehling, W.E., Chesney, M.A., Metzler, T.J., Goldstein, L.A., Maguen, S., Geronimo, C., A 12-week integrative exercise program improves self-reported mindfulness and interoceptive awareness in war veterans with posttraumatic stress symptoms (2018) Journal of Clinical Psychology, 74 (4), pp. 554-565; Moher, D., Shamseer, L., Clarke, M., Ghersi, D., Liberati, A., Petticrew, M., Stewart, L.A., Preferred Reporting Items for Systematic Review and Meta-Analysis Protocols (PRISMA-P) 2015 statement (2015) Systematic Reviews, 4 (1), p. 1; Mosewich, A., Crocker, P.R., Kowalski, K., Delongis, A., Applying self-compassion in sport: an intervention with women athletes (2013) Journal of Sport & Exercise Psychology, 35 (5), pp. 514-524; Mothes, H., Klaperski, S., Seelig, H., Schmidt, S., Fuchs, R., Regular aerobic exercise increases dispositional mindfulness in men: A randomized controlled trial (2014) Mental Health and Physical Activity, 7 (2), pp. 111-119. , &, (,).,., (,), -,., http://doi.org/10.1016/j.mhpa.2014.02.003; Mullan, E., Markland, D., Ingledew, D.K., A graded conceptualisation of self-determination in the regulation of exercise behaviour: development of a measure using confirmatory factor analytic procedures (1997) Personality and Individual Differences, 23 (5), pp. 745-752; Murn, L.T., Steele, M.R., What matters most? Age and gender differences in self-compassion and body attitudes among college students (2019) Counselling Psychology Quarterly, pp. 1-20. , &, (,).,., https://doi.org/10.1080/09515070.2019.1605334; Neff, K., Self-compassion: an alternative conceptualization of a healthy attitude toward oneself (2003) Self and Identity, 2 (2), pp. 85-101; Neff, K.D., The role of self-compassion in development: a healthier way to relate to oneself (2009) Human Development, 52 (4), pp. 211-214; Neff, K.D., Germer, C.K., A pilot study and randomized controlled trial of the mindful self-compassion program (2013) Journal of Clinical Psychology, 69 (1), pp. 28-44; Neff, K.D., Rude, S.S., Kirkpatrick, K.L., An examination of self-compassion in relation to positive psychological functioning and personality traits (2007) Journal of Research in Personality, 41 (4), pp. 908-916; Nieminen, P., Lehtiniemi, H., Vähäkangas, K., Huusko, A., Rautio, A., Standardised regression coefficient as an effect size index in summarising findings in epidemiological studies (2013) Epidemiology Biostatistics and Public Health, 10 (4). , https://doi.org/10.2427/8854; Nilsen, A.K.O., Anderssen, S.A., Ylvisaaker, E., Johannessen, K., Aadland, E., Physical activity among Norwegian preschoolers varies by sex, age, and season (2019) Scandinavian Journal of Medicine & Science in Sports, 29 (6), pp. 862-873. , &; Parry, G., Power, M., To RCT or not to RCT? The ongoing saga of randomised trials in quality improvement (2016) BMJ Quality & Safety, 25, pp. 221-223; Phillips, W.J., Hine, D.W., Self-compassion, physical health, and health behaviour: a meta-analysis (2019) Health Psychology Review, pp. 1-27. , &, (,).,., https://doi.org/10.1080/17437199.2019.1705872; Raes, F., Pommier, E., Neff, K.D., Van Gucht, D., Construction and factorial validation of a short form of the Self-Compassion Scale (2011) Clinical Psychology & Psychotherapy, 18 (3), pp. 250-255; Reis, N.A., Kowalski, K.C., Ferguson, L.J., Sabiston, C.M., Sedgwick, W.A., Crocker, P.R., Self-compassion and women athletes' responses to emotionally difficult sport situations: an evaluation of a brief induction (2015) Psychology of Sport and Exercise, 16, pp. 18-25; Rice, S.M., Parker, A.G., Rosenbaum, S., Bailey, A., Mawren, D., Purcell, R., Sport-related concussion and mental health outcomes in elite athletes: A systematic review (2017) Sports Medicine, pp. 1-19. , https://doi.org/10.1007/s40279-017-0810-3;

Robins, J.L., Elswick, R.K., Sturgill, J., McCain, N.L., The effects of tai chi on cardiovascular risk in women (2016) American Journal of Health Promotion, 30 (8), pp. 613-622. , https://doi.org/10.4278/ajhp.140618-QUAN-287; Semenchuk, B.N., Strachan, S.M., Fortier, M., Self-compassion and the self-regulation of exercise: Reactions to recalled exercise setbacks (2018) Journal of Sport and Exercise Psychology, 40 (1), pp. 31-39. , https://doi.org/10.1123/jsep.2017-0242; Sharma, M., Yoga as an alternative and complementary approach for stress management: a systematic review (2014) Journal of Evidence-Based Complementary & Alternative Medicine, 19 (1), pp. 59-67; Sirois, F.M., Kitner, R., Hirsch, J.K., Self-compassion, affect, and health-promoting behaviors (2015) Health Psychology, 34 (6), pp. 661-669. , &, (,).,., (,), -,., http://doi.org/10.1037/hea0000158; Spruit, A., Assink, M., van Vugt, E., van der Put, C., Stams, G.J., The effects of physical activity interventions on psychosocial outcomes in adolescents: a meta-analytic review (2016) Clinical Psychology Review, 45, pp. 56-71; Stalsberg, R., Pedersen, A.V., Are differences in physical activity across socioeconomic groups associated with choice of physical activity variables to report? (2018) International Journal of Environmental Research and Public Health, 15 (5), p. 922; Sterne, J.A.C., Egger, M., Funnel plots for detecting bias in meta-analysis: guidelines on choice of axis (2001) Journal of Clinical Epidemiology, 54 (10), pp. 1046-1055; Sun, X., Chan, D.W., Chan, L., Self-compassion and psychological well-being among adolescents in Hong Kong: Exploring gender differences (2016) Personality and Individual Differences, 101, pp. 288-292. , &, (,).,., -,., http://doi.org/10.1016/j.paid.2016.06.011; Thakur, M.B., Joshi, N., Analysis of self compassion and self esteem between adolescents engaged in physical exercise in the form of gym with those having sedentary lifestyle (2016) Journal of Psychosocial Research, 11 (1), p. 65; Thall, M.S., (2014) Exercise & Physical Activity in Middle-Aged Women: The Role of Self-Compassion, , Doctoral dissertation, The Ohio State University); Thompson, W.R., Worldwide survey of fitness trends for 2019 (2018) ACSM’s Health & Fitness Journal, 22 (6), pp. 10-17; Toobert, D.J., Hampson, S.E., Glasgow, R.E., The summary of diabetes self-care activities measure: results from 7 studies and a revised scale (2000) Diabetes Care, 23 (7), pp. 943-950; Trent, N.L., Miraglia, M., Dusek, J.A., Pasalis, E., Khalsa, S.B.S., Improvements in psychological health following a residential yoga-based program for frontline professionals (2018) Journal of Occupational and Environmental Medicine, 60 (4), pp. 357-367. , https://doi.org/10.1097/JOM.0000000000001216; Van Cauwenberg, J., Nathan, A., Barnett, A., Barnett, D.W., Cerin, E., Relationships between neighbourhood physical environmental attributes and older adults’ leisure-time physical activity: a systematic review and meta-analysis (2018) Sports Medicine, 48 (7), pp. 1635-1660; Vella, S.A., Gardner, L.A., Swann, C., Allen, M.S., Response: sports participation and interventions to reduce risk of mental health problems during childhood: a response to Crowell (2018) (2019) Child and Adolescent Mental Health, 24 (2), pp. 152-153; Ventura, A.D., Nefs, G., Browne, J.L., Friis, A.M., Pouwer, F., Speight, J., Is self-compassion related to behavioural, clinical and emotional outcomes in adults with diabetes?: Results from the second diabetes MILES-Australia (MILES-2) study (2019) Mindfulness, 10 (7), pp. 1222-1231. , https://doi.org/10.1007/s12671-018-1067-0; Walker, S.N., Sechrist, K., Pender, N., (1995) The health-promoting lifestyle profile II, , University of Nebraska Medical Center, College of Nursing, Omaha; Warburton, D.E.R., Nicol, C.W., Bredin, S.S.D., Health benefits of physical activity: The evidence (2006) Canadian Medical Association Journal, 174 (6), pp. 801-809. , http://doi.org/10.1503/cmaj.051351; Wendel-Vos, G.C., Schuit, A.J., Saris,

W.H., Kromhout, D., Reproducibility and relative validity of the short questionnaire to assess health-enhancing physical activity (2003) Journal of Clinical Epidemiology, 56 (12), pp. 1163-1169. , https://doi.org/10.1016/s0895-4356(03)00220-8; White, R.L., Babic, M.J., Parker, P.D., Lubans, D.R., Astell-Burt, T., Lonsdale, C., Domain-specific physical activity and mental health: a meta-analysis (2017) American Journal of Preventive Medicine, 52 (5), pp. 653-666; Williams, M.J., Dalgleish, T., Karl, A., Kuyken, W., Examining the factor structures of the five facet mindfulness questionnaire and the self-compassion scale (2014) Psychological Assessment, 26 (2), p. 407; Wilson, D., Bennett, E.V., Mosewich, A.D., Faulkner, G.E., Crocker, P.R., The zipper effect": exploring the interrelationship of mental toughness and self-compassion among Canadian elite women athletes (2019) Psychology of Sport and Exercise, 40, pp. 61-70; Wrosch, C., Scheier, M.F., Miller, G.E., Schulz, R., Carver, C.S., Adaptive self-regulation of unattainable goals: goal disengagement, goal reengagement, and subjective well-being (2003) Personality and Social Psychology Bulletin, 29 (12), pp. 1494-1508; Yarnell, L.M., Neff, K.D., Davidson, O.A., Mullarkey, M., Gender differences in self-compassion: examining the role of gender role orientation (2019) Mindfulness, 10 (6), pp. 1136-1152; Hou, J.J., (2007) The relationship between self-compassion and mental health among university students], , 侯佳捷. (2007). 大学生自我同情及其与心理健康的相关研究 [,., (Master’s thesis, 西南大学); Cun, Y.L., Gao, Z.C., Zhang, X., Li, Y.P., (2019) The Influence Mechanism of Yoga on University students’ School Adaptation: The Mediating Effect of Self-Compassion, (5), p. 16; Li, L.R., (2014) The Predictive Effect of Self-Compassion and Self-Esteem on Female University students’ Self-Determination Exercise Motivation, 34 (5), pp. 50-53},
correspondence_address1={Wong, M.Y.C.; Hong Kong Baptist UniversityHong Kong; email: 18481795@life.hkbu.edu.hk},
publisher={Springer},
issn={18688527},
language={English},
abbrev_source_title={Mindfulness},
document_type={Review},
source={Scopus},
}

@ARTICLE{Gamble2021,
author={Gamble, A. and Beech, B.M. and Wade, B.C. and Sutton, V.D. and Lim, C. and Sandridge, S. and Welsch, M.A.},
title={Telehealth diabetes prevention intervention for the next generation of African American youth: Protocol for a pilot trial},
journal={JMIR Research Protocols},
year={2021},
volume={10},
number={3},
doi={10.2196/25699},
art_number={e25699},
note={cited By 0},
url={https://www.scopus.com/inward/record.uri?eid=2-s2.0-85103616252&doi=10.2196%2f25699&partnerID=40&md5=42188f7fcb6badfe312393ecbe2970c3},
affiliation={Department of Preventive Medicine, John D Bower School of Population Health, University of Mississippi Medical Center, Jackson, MS, United States; Department of Pediatrics, School of Medicine, University of Mississippi Medical Center, Jackson, MS, United States; Myrlie Evers-Williams

Institute for the Elimination of Health Disparities, John D Bower School of Population Health, University of Mississippi Medical Center, Jackson, MS, United States; Department of Health Systems and Population Health Science, College of Medicine, University of Houston, Houston, TX, United States; Office of Preventive Health and Health Equity, Mississippi State Department of Health, Ridgeland, MS, United States; Division of Psychology, Department of Psychiatry and Human Behavior, University of Mississippi Medical Center, Jackson, MS, United States; Pediatric Gastroenterology, Children's Healthcare of Mississippi, Jackson, MS, United States; Department of Population Health Science, John D Bower School of Population Health, University of Mississippi Medical Center, Jackson, MS, United States},
abstract={Background: In 1999, type 2 diabetes mellitus (T2DM) was identified as an emerging epidemic in youth, and racial and ethnic minority youth were identified with high risk. Two decades later, no gold standard T2DM prevention intervention has been established for this population. Objective: This study tests the efficacy of a telehealth diabetes prevention intervention for African American (AA) families with children with risk for T2DM. Concurrently, investigators aim to evaluate an implementation strategy for the uptake of the intervention by the University of Mississippi Medical Center’s (UMMC) pediatric weight management clinic. Methods: This single-arm trial will enroll 20 parents with overweight or obesity of children (8-11 years) with overweight or obesity, both of whom are at risk for T2DM. Parents will meet in small groups (5 parents per group) weekly for 11 weeks and then monthly for 4 monthly maintenance sessions via videoconference using Wi-Fi–enabled iPads with cellular connectivity. The intervention will be adapted from the National Diabetes Prevention Program and Power to Prevent, a diabetes prevention program tailored for AA families. The same lifestyle intervention facilitated by a racially concordant lifestyle coach trained in the Diabetes Prevention Program will be delivered to all groups (n=4). Participants will be recruited in-person during patient encounters at the UMMC’s pediatric weight management clinic. Sessions will consist of dietary and physical activity behavior change strategies facilitated using problem-solving and goal-setting skills. The implementation strategy has 2 targets: the pediatric weight management clinic site and clinical team and parents of children at risk for T2DM engaged in intensive obesity treatment to prevent T2DM. The multifaceted implementation protocol includes 4 discrete strategies: creating a new clinical team, changing the service site, intervening with families, and promoting organizational readiness for change. Results: Recruitment and enrollment began in December 2020, and the intervention is scheduled to be delivered to the first cohort of parents in March 2021. The results are expected to be submitted for publication beginning in November 2021 through 2022. The primary outcome measure for the pilot trial will include changes from baseline to 12 and 30 weeks in the child BMI z score and parent BMI. The implementation evaluation will include multiple measures of feasibility, acceptability, appropriateness, fidelity, and efficacy. This protocol was approved by the UMMC’s Institutional Review Board (#2020V0249). Conclusions: The proposed intervention approach is supported by the scientific literature and is scalable given the current and future health care subsidies for telehealth. Findings from this pilot trial will begin to address critical barriers to defining a gold standard lifestyle intervention for AA families with children at risk for T2DM. If effective, the intervention could be feasibly disseminated to treat obesity and prevent T2DM in high-risk AA pediatric populations. © Abigail Gamble, Bettina M Beech, Breanna C Wade, Victor D Sutton, Crystal Lim, Shanda Sandridge, Michael A Welsch.},
author_keywords={Behavioral science; Centers for disease control and

prevention; Child obesity; Implementation science; Jackson heart study; Mobile phone; Obesity management; Prediabetic state; Preventive medicine; Telehealth},
references={Rosenbloom, AL, Joe, JR, Young, RS, Winter, WE., Emerging epidemic of type 2 diabetes in youth (1999) Diabetes Care, 22 (2), pp. 345-354. , Feb; [FREE Full _text] [doi] [Medline: 10333956]; Vespa, J, Medina, L, Armstrong, D., Demographic Turning Points for the United States: Population Projections for 2020 to 2060, ,
https://www.census.gov/library/publications/2020/demo/p25-1144.html, Washington, DC.: United States Census Bureau; 2020 Feb. [accessed 2021-03-15]; Prasad, RB, Groop, L., Genetics of type 2 diabetes-pitfalls and possibilities (2015) Genes (Basel), 6 (1), pp. 87-123. , Mar 12; [FREE Full _text] [doi] [Medline: 25774817]; Dendup, T, Feng, X, Clingan, S, Astell-Burt, T., Environmental Risk Factors for Developing Type 2 Diabetes Mellitus: A Systematic Review (2018) Int J Environ Res Public Health, 15 (1). , Jan 05; [FREE Full _text] [doi] [Medline: 29304014]; Centers for Disease Control and Prevention, ,
https://www.cdc.gov/diabetes/pdfs/data/statistics/national-diabetes-statistics-report.pdf, National Diabetes Statistics Report, 2020. 2020. [accessed 2021-03-15]; Andes, LJ, Cheng, YJ, Rolka, DB, Gregg, EW, Imperatore, G., Prevalence of Prediabetes Among Adolescents and Young Adults in the United States, 2005-2016 (2020) JAMA Pediatr, 174 (2), p. e194498. , Feb 01; [FREE Full _text] [doi] [Medline: 31790544]; Imperatore, G, Boyle, JP, Thompson, TJ, Case, D, Dabelea, D, Hamman, RF, SEARCH for Diabetes in Youth Study Group (2012) Diabetes Care, 35 (12), pp. 2515-2520. , Dec; [FREE Full _text] [doi] [Medline: 23173134]; Haire-Joshu, D, Hill-Briggs, F., The Next Generation of Diabetes Translation: A Path to Health Equity (2019) Annu Rev Public Health, 40, pp. 391-410. , Apr 01;: [doi] [Medline: 30601723]; Arslanian, S, Kim, JY, Nasr, A, Bacha, F, Tfayli, H, Lee, S, Insulin sensitivity across the lifespan from obese adolescents to obese adults with impaired glucose tolerance: Who is worse off? (2018) Pediatr Diabetes, 19 (2), pp. 205-211. , Mar; [doi] [Medline: 28726334]; Pulgaron, ER, Delamater, AM., Obesity and type 2 diabetes in children: epidemiology and treatment (2014) Curr Diab Rep, 14 (8), p. 508. , Aug; [FREE Full _text] [doi] [Medline: 24919749]; Impact of Insulin and Metformin Versus Metformin Alone on β-Cell Function in Youth With Impaired Glucose Tolerance or Recently Diagnosed Type 2 Diabetes (2018) Diabetes Care, 41 (8), pp. 1717-1725. , RISE Consortium. Aug; [FREE Full _text] [doi] [Medline: 29941500]; Nadeau, KJ, Anderson, BJ, Berg, EG, Chiang, JL, Chou, H, Copeland, KC, Youth-Onset Type 2 Diabetes Consensus Report: Current Status, Challenges, and Priorities (2016) Diabetes Care, 39 (9), pp. 1635-1642. , Sep; [FREE Full _text] [doi] [Medline: 27486237]; Bansal, N., Prediabetes diagnosis and treatment: A review (2015) World J Diabetes, 6 (2), pp. 296-303. , Mar 15; [FREE Full _text] [doi] [Medline: 25789110]; Buysschaert, M, Bergman, M., Definition of prediabetes (2011) Med Clin North Am, 95 (2), pp. 289-297. , Mar; [doi] [Medline: 21281833]; Tabák, AG, Herder, C, Rathmann, W, Brunner, EJ, Kivimäki, M., Prediabetes: a high-risk state for diabetes development (2012) Lancet, 379 (9833), pp. 2279-2290. , Jun 16; [FREE Full _text] [doi] [Medline: 22683128]; Nathan, DM, Davidson, MB, DeFronzo, RA, Heine, RJ, Henry, RR, Pratley, R, American Diabetes Association. Impaired fasting glucose and impaired glucose tolerance: implications for care (2007) Diabetes Care, 30 (3), pp. 753-759. , Mar; [doi] [Medline: 17327355]; Echouffo-Tcheugui, JB, Niiranen, TJ, McCabe, EL, Jain, M, Vasan, RS, Larson, MG, Lifetime Prevalence and Prognosis of Prediabetes Without Progression to Diabetes (2018) Diabetes Care, 41 (7), pp. e117-e118. , Jul; [FREE Full _text] [doi] [Medline: 29724784];

Albright, AL, Gregg, EW., Preventing type 2 diabetes in communities across the U.S.: the National Diabetes Prevention Program (2013) Am J Prev Med, 44 (4), pp. S346-S351. , Apr;(Suppl 4): [doi] [Medline: 23498297]; The 10-year cost-effectiveness of lifestyle intervention or metformin for diabetes prevention: an intent-to-treat analysis of the DPP/DPPOS (2012) Diabetes Care, 35 (4), pp. 723-730. , Diabetes Prevention Program Research Group. Apr; [FREE Full text] [doi] [Medline: 22442395]; Knowler, WC, Barrett-Connor, E, Fowler, SE, Hamman, RF, Lachin, JM, Walker, EA, Reduction in the incidence of type 2 diabetes with lifestyle intervention or metformin (2002) N Engl J Med, 346 (6), pp. 393-403. , Feb 7; [FREE Full _text] [doi] [Medline: 11832527]; Diabetes, PPRG, Knowler, WC, Fowler, SE, Hamman, RF, Christophi, CA, Hoffman, HJ, 10-year follow-up of diabetes incidence and weight loss in the Diabetes Prevention Program Outcomes Study (2009) Lancet, 374 (9702), pp. 1677-1686. , Nov 14; [FREE Full _text] [doi] [Medline: 19878986]; Subramanian, K, Midha, I, Chellapilla, V., Overcoming the Challenges in Implementing Type 2 Diabetes Mellitus Prevention Programs Can Decrease the Burden on Healthcare Costs in the United States (2017) J Diabetes Res, 2017, p. 2615681. , [FREE Full _text] [doi] [Medline: 28890899]; Magge, SN, Goodman, E, Armstrong, SC, The Metabolic Syndrome in Children and Adolescents: Shifting the Focus to Cardiometabolic Risk Factor Clustering (2017) Pediatrics, 140 (2). , COMMITTEE ON NUTRITION, SECTION ON ENDOCRINOLOGY, SECTION ON OBESITY. Aug; [FREE Full _text] [doi] [Medline: 28739653]; McCurley, JL, Crawford, MA, Gallo, LC., Prevention of Type 2 Diabetes in U.S. Hispanic Youth: A Systematic Review of Lifestyle Interventions (2017) Am J Prev Med, 53 (4), pp. 519-532. , Oct; [FREE Full _text] [doi] [Medline: 28688727]; Olson, ML, Rentería-Mexía, A, Connelly, MA, Vega-López, S, Soltero, EG, Konopken, YP, Decreased GlycA after lifestyle intervention among obese, prediabetic adolescent Latinos (2019) J Clin Lipidol, 13 (1), pp. 186-193. , [FREE Full _text] [doi] [Medline: 30342918]; Burnet, DL, Plaut, AJ, Wolf, SA, Huo, D, Solomon, MC, Dekayie, G, Reach-out: a family-based diabetes prevention program for African American youth (2011) J Natl Med Assoc, 103 (3), pp. 269-277. , Mar; [doi] [Medline: 21671531]; O'Connor, EA, Evans, CV, Burda, BU, Walsh, ES, Eder, M, Lozano, P., Screening for Obesity and Intervention for Weight Management in Children and Adolescents: Evidence Report and Systematic Review for the US Preventive Services Task Force (2017) JAMA, 317 (23), pp. 2427-2444. , Jun 20; [doi] [Medline: 28632873]; (2012) Power to Prevent: A Family Lifestyle Approach to Diabetes Prevention, , US Department of Health and Human Services. Amazon: CreateSpace Independent Publishing Platform; Jul 12; Huang, J, Lin, Y, Wu, N., The effectiveness of telemedicine on body mass index: A systematic review and meta-analysis (2019) J Telemed Telecare, 25 (7), pp. 389-401. , Aug; [doi] [Medline: 29804509]; Joiner, KL, Nam, S, Whittemore, R., Lifestyle interventions based on the diabetes prevention program delivered via eHealth: A systematic review and meta-analysis (2017) Prev Med, 100, pp. 194-207. , Jul;: [FREE Full _text] [doi] [Medline: 28456513]; Vadheim, LM, Patch, K, Brokaw, SM, Carpenedo, D, Butcher, MK, Helgerson, SD, Telehealth delivery of the diabetes prevention program to rural communities (2017) Transl Behav Med, 7 (2), pp. 286-291. , Jun; [FREE Full _text] [doi] [Medline: 28417426]; Grock, S, Ku, J, Kim, J, Moin, T., A Review of Technology-Assisted Interventions for Diabetes Prevention (2017) Curr Diab Rep, 17 (11), p. 107. , Sep 23; [doi] [Medline: 28942537]; Kaufman, N, Khurana, I., Using Digital Health Technology to Prevent and Treat Diabetes (2016) Diabetes Technol Ther, 18, pp. S56-S68. , Feb; Suppl 1: [FREE Full _text] [doi] [Medline: 26836430]; Fatehi, F, Armfield, NR, Dimitrijevic, M, Gray, LC., Clinical applications of videoconferencing: a scoping review of the literature for the period 2002-2012 (2014) J Telemed Telecare, 20 (7), pp. 377-383. , Oct;

[doi] [Medline: 25399998]; Verhoeven, F, van, GL, Dijkstra, K, Nijland, N, Seydel, E, Steehouder, M., The contribution of teleconsultation and videoconferencing to diabetes care: a systematic literature review (2007) J Med Internet Res, 9 (5), p. e37. , [FREE Full _text] [doi] [Medline: 18093904]; Siriwardena, LSAN, Wickramasinghe, WAS, Perera, KLD, Marasinghe, RB, Katulanda, P, Hewapathirana, R., A review of telemedicine interventions in diabetes care (2012) J Telemed Telecare, 18 (3), pp. 164-168. , Apr; [doi] [Medline: 22362832]; Banbury, A, Nancarrow, S, Dart, J, Gray, L, Parkinson, L., Telehealth Interventions Delivering Home-based Support Group Videoconferencing: Systematic Review (2018) J Med Internet Res, 20 (2), p. e25. , Feb 02; [FREE Full _text] [doi] [Medline: 29396387]; Nelson, E, Yadrich, DM, Thompson, N, Wright, S, Stone, K, Adams, N, Telemedicine Support Groups for Home Parenteral Nutrition Users (2017) Nutr Clin Pract, 32 (6), pp. 789-798. , Dec; [doi] [Medline: 29016235]; Olthuis, JV, Wozney, L, Asmundson, GJG, Cramm, H, Lingley-Pottie, P, McGrath, PJ., Distance-delivered interventions for PTSD: A systematic review and meta-analysis (2016) J Anxiety Disord, 44, pp. 9-26. , Dec;: [doi] [Medline: 27697658]; Tuckson, RV, Edmunds, M, Hodgkins, ML., Telehealth (2017) N Engl J Med, 377 (16), pp. 1585-1592. , Oct 19; [doi] [Medline: 29045204]; Tuckson, RV, Edmunds, M, Hodgkins, ML., Telehealth (2017) N Engl J Med, 377 (16), pp. 1585-1592. , Oct 19; [doi] [Medline: 29045204]; Mayberry, LS, Harper, KJ, Osborn, CY., Family behaviors and type 2 diabetes: What to target and how to address in interventions for adults with low socioeconomic status (2016) Chronic Illn, , Apr 19. [doi] [Medline: 27099387]; Curran, GM, Bauer, M, Mittman, B, Pyne, JM, Stetler, C., Effectiveness-implementation hybrid designs: combining elements of clinical effectiveness and implementation research to enhance public health impact (2012) Med Care, 50 (3), pp. 217-226. , Mar; [FREE Full _text] [doi] [Medline: 22310560]; Barker, LE, Kirtland, KA, Gregg, EW, Geiss, LS, Thompson, TJ., Geographic distribution of diagnosed diabetes in the U.S.: a diabetes belt (2011) Am J Prev Med, 40 (4), pp. 434-439. , Apr; [doi] [Medline: 21406277]; (2019) Annual Report, 2019. America's Health Rankings, , https://assets.americashealthrankings.org/app/uploads/ahr_2019annualreport.pdf, United Health Foundation. [accessed 2021-03-15]; Warren, M, Beck, S, Delgado, D., (2020) The State of Obesity: Better Policies for a Healthier Americ, 2020, , https://www.tfah.org/wp-content/uploads/2020/09/TFAHObesityReport_20.pdf, Washington, D.C: Trust for America's Health; [accessed 2021-03-15]; (2019) Chart Book: Results of the Mississippi Child & Youth Prevalence of Obesity Study, 2005 - 2017, , https://mshealthpolicy.com/wp-content/uploads/2019/07/2017-CAYPOS-Chartbook-May-2019.pdf, Center for Mississippi Health Policy. Jackson, MS: Center for Mississippi Health Policy; May. [accessed 2021-03-15]; (2019), https://www.census.gov/quickfacts/fact/table/MS,US/PST045219, U.S. Census Bureau. QuickFacts: Mississippi; United States. [accessed 2021-03-15]; (2020) Mississippi Kids Count Factbook, Census Edition, , https://childrensfoundationms.org/project/kidscount/, Jackson, MS: Children's Foundation of Mississippi; [accessed 2021-03-15]; (2016), http://www.msdh.state.ms.us/msdhsite/index.cfm/44,7357,112,63,pdf/Primary%20Care%20Needs%20Assessment.pdf, Mississippi Primary Care Needs Assessment. Jackson, MS: Office of Rural Health and Primary Care, Mississippi State Department of Health; Mar. [accessed 2021-03-15]; (2017) Telemedicine in Mississippi: Defining Boundaries for a New Frontier, , https://mshealthpolicy.com/wp-content/uploads/2017/01/Telemedicine-Issue-Brief-Jan-2017.pdf, Center for Mississippi Health Policy. Jackson, MS: Center for Mississippi Heath Policy; Jan. [accessed 2021-03-15]; (2019),

https://www.umc.edu/Healthcare/Telehealth/Telehealth_Home.html, Telehealth Care at UMMC. Jackson, MS: University of Mississippi Medical Center; [accessed 2021-03-15]; Davis, TC, Hoover, KW, Keller, S, Replogle, WH., Mississippi Diabetes Telehealth Network: A Collaborative Approach to Chronic Care Management (2020) Telemed J E Health, 26 (2), pp. 184-189. , Feb 01; [doi] [Medline: 30822265]; Lim, C., (2016) Wellness and Weight Family Group Project, , https://clinicaltrials.gov/ct2/show/NCT02677792, Feb. [accessed 2021-03-15]; O'Connor, E, Senger, C, Henninger, M, Gaynes, B, Coppola, E, Soulsby, WM., Interventions to Prevent Perinatal Depression: A Systematic Evidence Review for the U.S. Preventive Services Task Force (2019) JAMA, 321 (6). , [doi] [Medline: 30807060]; (2019) Current State Laws and Reimbursement Policies, , https://www.cchpca.org, The Center for Connected Health Policy. Telehealth Policy. West Sacramento, CA: Center for Connected Health Policy; [accessed 2021-03-15]; Ackermann, RT, Finch, EA, Brizendine, E, Zhou, H, Marrero, DG., Translating the Diabetes Prevention Program into the Community (2008) American Journal of Preventive Medicine, 35 (4), pp. 357-363. , Oct; [doi]; Gregg, E, Jakicic, J, Blackburn, G, Bloomquist, P, Bray, G, Association of the magnitude of weight loss and changes in physical fitness with long-term cardiovascular disease outcomes in overweight or obese people with type 2 diabetes: a post-hoc analysis of the Look AHEAD randomised clinical trial (2016) Lancet Diabetes Endocrinol, 4 (11), pp. 913-921. , Look AHEAD Research Group, Nov; [FREE Full _text] [doi] [Medline: 27595918]; Kolsgaard, MLP, Joner, G, Brunborg, C, Anderssen, SA, Tonstad, S, Andersen, LF., Reduction in BMI z-score and improvement in cardiometabolic risk factors in obese children and adolescents. The Oslo Adiposity Intervention Study - a hospital/public health nurse combined treatment (2011) BMC Pediatr, 11 (1). , May 27; [doi]; Mazzeo, SE, Burnette, CB, Stern, M, Thornton, LM, Bulik, CM, Evans, RK, Recruitment and retention of families interested in a parent-based pediatric obesity intervention (2019) Contemp Clin Trials Commun, 16, p. 100467. , Dec;: [FREE Full text] [doi] [Medline: 31701041]; Tripicchio, GL, Ammerman, AS, Ward, DS, Faith, MS, Truesdale, KP, Burger, KS, Clinical-Community Collaboration: A Strategy to Improve Retention and Outcomes in Low-Income Minority Youth in Family-Based Obesity Treatment (2018) Child Obes, 14 (3), pp. 141-148. , Apr; [doi] [Medline: 29584452]; Davis, AM, Sampilo, M, Gallagher, KS, Dean, K, Saroja, MB, Yu, Q, Treating rural paediatric obesity through telemedicine vs. telephone: Outcomes from a cluster randomized controlled trial (2016) J Telemed Telecare, 22 (2), pp. 86-95. , Mar; [FREE Full text] [doi] [Medline: 26026186]; Diabetes, P., The Diabetes Prevention Program (DPP): description of lifestyle intervention (2002) Diabetes Care, 25 (12), pp. 2165-2171. , Dec; [FREE Full _text] [Medline: 12453955]; Skelton, JA, Buehler, C, Irby, MB, Grzywacz, JG., Where are family theories in family-based obesity treatment?: conceptualizing the study of families in pediatric weight management (2012) Int J Obes (Lond), 36 (7), pp. 891-900. , Jul; [FREE Full _text] [doi] [Medline: 22531090]; Davison, KK, Lawson, HA, Coatsworth, JD., The Family-centered Action Model of Intervention Layout and Implementation (FAMILI): the example of childhood obesity (2012) Health Promot Pract, 13 (4), pp. 454-461. , Jul; [doi] [Medline: 21632465]; Chen, M, Wang, R, Hung, S., Predicting health-promoting self-care behaviors in people with pre-diabetes by applying Bandura social learning theory (2015) Appl Nurs Res, 28 (4), pp. 299-304. , Nov; [doi] [Medline: 26608429]; Nugent, LE, Wallston, KA., Modified social learning theory re-examined: correlates of self-management behaviors of persons with Type 2 diabetes (2016) J Behav Med, 39 (6), pp. 947-956. , Dec; [doi] [Medline: 27263097]; Salisbury, C, Thomas, C, O'Cathain, A, Rogers, A, Pope, C, Yardley, L, TElehealth in CHronic disease: mixed-methods study to develop the TECH

conceptual model for intervention design and evaluation (2015) BMJ Open, 5 (2), p. e006448. , Feb 06; [FREE Full _text] [doi] [Medline: 25659890]; (2009) Small Steps, Big Rewards”: Preventing Type 2 Diabetes, , https://medlineplus.gov/magazine/issues/fall09/articles/fall09pg10a.html, [accessed 2021-03-15]; Grossman, DC, Bibbins-Domingo, K, Curry, SJ, Barry, MJ, Davidson, KW, Screening for Obesity in Children and Adolescents: US Preventive Services Task Force Recommendation Statement (2017) JAMA, 317 (23), pp. 2417-2426. , US Preventive Services Task Force, Jun 20; [doi] [Medline: 28632874]; Aziz, Z, Absetz, P, Oldroyd, J, Pronk, NP, Oldenburg, B., A systematic review of real-world diabetes prevention programs: learnings from the last 15 years (2015) Implement Sci, 10, p. 172. , Dec 15;: [FREE Full _text] [doi] [Medline: 26670418]; (2018) Diabetes Prevention Recognition Program: Standards and Operating Procedures. Centers for Disease Control and Prevention, , https://www.cdc.gov/diabetes/prevention/pdf/dprp-standards.pdf, Atlanta, GA: Centers for Disease Control and Prevention; Mar 01. [accessed 2021-03-15]; (2013) Managing Overweight and Obesity in Adults: Systematic Evidence Review from the Obesity Expert Panel, , https://www.nhlbi.nih.gov/sites/default/files/media/docs/obesity-evidence-review.pdf, Bethesda, MD: U.S. Department of Health and Human Services, National Institutes of Health, National Heart, Lung, and Blood Institute; [accessed 2021-03-14]; Whitlock, EP, O'Connor, EA, Williams, SB, Beil, TL, Lutz, KW., Effectiveness of weight management interventions in children: a targeted systematic review for the USPSTF (2010) Pediatrics, 125 (2), pp. e396-e418. , Feb; [doi] [Medline: 20083531]; (2018) Physical Activity Guidelines Advisory Committee Scientific Report, , https://health.gov/sites/default/files/2019-09/PAG_Advisory_Committee_Report.pdf, 2018 Washington, D.C: U.S. Department of Health and Human Services; [accessed 2021-03-15]; Donnelly, JE, Blair, SN, Jakicic, JM, Manore, MM, Rankin, JW, Smith, BK, American College of Sports Medicine Position Stand. Appropriate physical activity intervention strategies for weight loss and prevention of weight regain for adults (2009) Med Sci Sports Exerc, 41 (2), pp. 459-471. , Feb; [doi] [Medline: 19127177]; Scott, HK, Jain, A, Cogburn, M., Behavior Modification (2021) StatPearls, , [Internet]. Treasure Island, FL: StatPearls Publishing; Jan 07; Bhattacharya, A, Vilardaga, R, Kientz, J, Munson, S., Lessons from Practice: Designing Tools to Facilitate Individualized Support for Quitting Smoking (2017) ACM Trans Comput Hum Interact, pp. 3057-3070. , Nov: [doi] [Medline: 291123362]; Rollnick, S, Butler, CC, Stott, N., Helping smokers make decisions: the enhancement of brief intervention for general medical practice (1997) Patient Educ Couns, 31 (3), pp. 191-203. , [Medline: 9277242]; Lorig, K, Ritter, PL, Ory, MG, Whitelaw, N., Effectiveness of a generic chronic disease self-management program for people with type 2 diabetes: a translation study (2013) Diabetes Educ, 39 (5), pp. 655-663. , [doi] [Medline: 23782621]; Lorig, KR, Sobel, DS, Ritter, PL, Laurent, D, Hobbs, M., Effect of a self-management program on patients with chronic disease (2001) Eff Clin Pract, 4 (6), pp. 256-262. , [FREE Full _text] [Medline: 11769298]; Bodenheimer, T, Lorig, K, Holman, H, Grumbach, K., Patient self-management of chronic disease in primary care (2002) JAMA, 288 (19), pp. 2469-2475. , Nov 20; [Medline: 12435261]; Pearson, ES., Goal setting as a health behavior change strategy in overweight and obese adults: a systematic literature review examining intervention components (2012) Patient Educ Couns, 87 (1), pp. 32-42. , Apr; [doi] [Medline: 21852063]; Lorig, KR, Holman, H., Self-management education: history, definition, outcomes, and mechanisms (2003) Ann Behav Med, 26 (1), pp. 1-7. , Aug; [Medline: 12867348]; Lorig, K, Laurent, DD, Plant, K, Krishnan, E, Ritter, PL., The components of action planning and their

associations with behavior and health outcomes (2014) Chronic Illn, 10 (1), pp. 50-59. , Mar; [doi] [Medline: 23838837]; (2021) Retention for Commercial Plans and Employers, , https://coveragetoolkit.org/commercial-plans/commercial-plans-delivery/commercial-plans-retention/, Coveragetoolkit.org. [accessed 2021-03-15]; Holdsworth, E, Thorogood, N, Sorhaindo, A, Nanchahal, K., A Qualitative Study of Participant Engagement With a Weight Loss Intervention (2017) Health Promot Pract, 18 (2), pp. 245-252. , Mar; [doi] [Medline: 27466266]; Millstein, R., Measuring outcomes in adult weight loss studies that include diet and physical activity: a systematic review (2014) J Nutr Metab, 2014, p. 421423. , [FREE Full _text] [doi] [Medline: 25525513]; Harris, PA, Taylor, R, Thielke, R, Payne, J, Gonzalez, N, Conde, JG., Research electronic data capture (REDCap)-a metadata-driven methodology and workflow process for providing translational research informatics support (2009) J Biomed Inform, 42 (2), pp. 377-381. , Apr; [FREE Full _text] [doi] [Medline: 18929686]; (2019), https://www.cdc.gov/nccdphp/dnpao/growthcharts/resources/sas.htm, Centers for Disease Control and Prevention. [accessed 2021-03-15]; (2014) Centers for Disease Control and Prevention, , https://www.cdc.gov/nccdphp/dnpao/growthcharts/training/bmiage/page5_1.html, Calculating BMI Using the Metric System. [accessed 2021-03-15]; Jiang, F, Hou, X, Lu, J, Zhou, J, Lu, F, Kan, K, Assessment of the performance of A1CNow(+) and development of an error grid analysis graph for comparative hemoglobin A1c measurements (2014) Diabetes Technol Ther, 16 (6), pp. 363-369. , Jun; [FREE Full _text] [doi] [Medline: 24766632]; Bastianelli, K, Ledin, S, Chen, J., Comparing the Accuracy of 2 Point-of-Care Lipid Testing Devices (2017) J Pharm Pract, 30 (5), pp. 490-497. , Oct; [doi] [Medline: 27313238]; Muntner, P, Shimbo, D, Carey, RM, Charleston, JB, Gaillard, T, Misra, S, Measurement of Blood Pressure in Humans: A Scientific Statement From the American Heart Association (2019) Hypertension, 73 (5), pp. e35-e66. , May; [doi] [Medline: 30827125]; Norman, Bohman B, Nyberg, G, Schäfer, EL., Psychometric Properties of a Scale to Assess Parental Self-Efficacy for Influencing Children's Dietary, Physical Activity, Sedentary, and Screen Time Behaviors in Disadvantaged Areas (2018) Health Educ Behav, 45 (1), pp. 132-140. , Feb; [doi] [Medline: 28385099]; Lassetter, JH, Macintosh, CI, Williams, M, Driessnack, M, Ray, G, Wisco, JJ., Psychometric testing of the healthy eating and physical activity self-efficacy questionnaire and the healthy eating and physical activity behavior recall questionnaire for children (2018) J Spec Pediatr Nurs, 23 (2), p. e12207. , Apr; [FREE Full _text] [doi] [Medline: 29316195]; Gattshall, ML, Shoup, JA, Marshall, JA, Crane, LA, Estabrooks, PA., Validation of a survey instrument to assess home environments for physical activity and healthy eating in overweight children (2008) Int J Behav Nutr Phys Act, 5, p. 3. , Jan 11;: [FREE Full _text] [doi] [Medline: 18190709]; Rhee, KE, McEachern, R, Jelalian, E., Parent readiness to change differs for overweight child dietary and physical activity behaviors (2014) J Acad Nutr Diet, 114 (10), pp. 1601-1610. , Oct; [FREE Full _text] [doi] [Medline: 24953789]; Rhee, KE, De Lago, CW, Arscott-Mills, T, Mehta, SD, Davis, RK., Factors associated with parental readiness to make changes for overweight children (2005) Pediatrics, 116 (1), pp. e94-101. , Jul; [doi] [Medline: 15995022]; Epstein, N, Baldwin, L, Bishop, D., The McMaster Family Assessment Device (1983) J Marital Fam Ther, 9 (2), pp. 171-180. , [doi]; Halliday, JA, Palma, CL, Mellor, D, Green, J, Renzaho, AMN., The relationship between family functioning and child and adolescent overweight and obesity: a systematic review (2014) Int J Obes (Lond), 38 (4), pp. 480-493. , Apr; [doi] [Medline: 24232501]; Mujahid, MS, Diez Roux, AV, Morenoff, JD, Raghunathan, T., Assessing the measurement properties of neighborhood scales:

from psychometrics to ecometrics (2007) Am J Epidemiol, 165 (8), pp. 858-867. , Apr 15; [doi] [Medline: 17329713]; Cohen, S, Kamarck, T, Mermelstein, R., A global measure of perceived stress (1983) J Health Soc Behav, 24 (4), pp. 385-396. , Dec; [Medline: 6668417]; Bevans, KB, Gardner, W, Pajer, KA, Becker, B, Carle, A, Tucker, CA, Psychometric Evaluation of the PROMIS® Pediatric Psychological and Physical Stress Experiences Measures (2018) J Pediatr Psychol, 43 (6), pp. 678-692. , Jul 01; [FREE Full _text] [doi] [Medline: 29490050]; Carskadon, MA, Acebo, C., A self-administered rating scale for pubertal development (1993) J Adolesc Health, 14 (3), pp. 190-195. , May; [doi] [Medline: 8323929]; McCoy, CE., Understanding the Intention-to-treat Principle in Randomized Controlled Trials (2017) West J Emerg Med, 18 (6), pp. 1075-1078. , Oct; [FREE Full _text] [doi] [Medline: 29085540]; Montori, VM, Guyatt, GH., Intention-to-treat principle (2001) CMAJ, 165 (10), pp. 1339-1341. , Nov 13; [FREE Full _text] [Medline: 11760981]; Gupta, SK., Intention-to-treat concept: A review (2011) Perspect Clin Res, 2 (3), pp. 109-112. , Jul; [FREE Full _text] [doi] [Medline: 21897887]; Powell, BJ, Waltz, TJ, Chinman, MJ, Damschroder, LJ, Smith, JL, Matthieu, MM, A refined compilation of implementation strategies: results from the Expert Recommendations for Implementing Change (ERIC) project (2015) Implement Sci, 10, p. 21. , Feb 12;: [FREE Full _text] [doi] [Medline: 25889199]; Proctor, EK, Powell, BJ, McMillen, JC., Implementation strategies: recommendations for specifying and reporting (2013) Implement Sci, 8, p. 139. , Dec 01;: [FREE Full _text] [doi] [Medline: 24289295]; Doherty, RB, Crowley, RA, Principles supporting dynamic clinical care teams: an American College of Physicians position paper (2013) Ann Intern Med, 159 (9), pp. 620-626. , HealthPublic Policy Committee of the American College of Physicians. Nov 05; [FREE Full _text] [doi] [Medline: 24042251]; Fortmann, AL, Walker, C, Barger, K, Robacker, M, Morrisey, R, Ortwine, K, Care Team Integration in Primary Care Improves One-Year Clinical and Financial Outcomes in Diabetes: A Case for Value-Based Care (2020) Popul Health Manag, 23 (6), pp. 467-475. , Dec; [doi] [Medline: 31944895]; Rauschenberg, C, Schick, A, Hirjak, D, Seidler, A, Paetzold, I, Apfelbacher, C, Evidence Synthesis of Digital Interventions to Mitigate the Negative Impact of the COVID-19 Pandemic on Public Mental Health: Rapid Meta-review (2021) J Med Internet Res, 23 (3), p. e23365. , Mar 10; [FREE Full _text] [doi] [Medline: 33606657]; Damschroder, LJ, Aron, DC, Keith, RE, Kirsh, SR, Alexander, JA, Lowery, JC., Fostering implementation of health services research findings into practice: a consolidated framework for advancing implementation science (2009) Implement Sci, 4, p. 50. , [FREE Full _text] [doi] [Medline: 19664226]; Damschroder, LJ, Reardon, CM, AuYoung, M, Moin, T, Datta, SK, Sparks, JB, Implementation findings from a hybrid III implementation-effectiveness trial of the Diabetes Prevention Program (DPP) in the Veterans Health Administration (VHA) (2017) Implement Sci, 12 (1), p. 94. , Jul 26; [FREE Full _text] [doi] [Medline: 28747191]; Stetler, CB, Legro, MW, Wallace, CM, Bowman, C, Guihan, M, Hagedorn, H, The role of formative evaluation in implementation research and the QUERI experience (2006) J Gen Intern Med, 21, pp. S1-S8. , Feb; Suppl 2: [FREE Full _text] [doi] [Medline: 16637954]; Nurjono, M, Shrestha, P, Lee, A, Lim, XY, Shiraz, F, Tan, S, Realist evaluation of a complex integrated care programme: protocol for a mixed methods study (2018) BMJ Open, 8 (3), p. e017111. , Mar 01; [doi]; Vastine, A, Gittelsohn, J, Ethelbah, B, Anliker, J, Caballero, B., Formative research and stakeholder participation in intervention development (2005) Am J Health Behav, 29 (1), pp. 57-69. , [doi] [Medline: 15604050]; McKleroy, VS, Galbraith, JS, Cummings, B, Jones, P, Harshbarger, C, Collins, C, Adapting evidence-based behavioral interventions for new settings and target populations (2006) AIDS Educ Prev, 18 (4), pp. 59-73. , ADAPT Team. Aug;(Suppl A): [doi] [Medline:

16987089]; Fernandez, ME, Ten Hoor, GA, van Lieshout, S, Rodriguez, SA, Beidas, RS, Parcel, G, Implementation Mapping: Using Intervention Mapping to Develop Implementation Strategies (2019) Front Public Health, 7, p. 158. , [FREE Full _text] [doi] [Medline: 31275915]; Fernandez, ME, Ruiter, RAC, Markham, CM, Kok, G., Intervention Mapping: Theory- and Evidence-Based Health Promotion Program Planning: Perspective and Examples (2019) Front Public Health, 7, p. 209. , [FREE Full _text] [doi] [Medline: 31475126]; Dane, AV, Schneider, BH., Program integrity in primary and early secondary prevention: are implementation effects out of control? (1998) Clin Psychol Rev, 18 (1), pp. 23-45. , Jan; [doi] [Medline: 9455622]; Bright, R, Sakurada, B., A Population Health Strategy for Diabetes: New Partners, New Opportunities (2016) NAM Perspectives, , Washington, D.C: National Academy of Medicine; Feb 24; Keesara, S, Jonas, A, Schulman, K., Covid-19 and Health Care's Digital Revolution (2020) N Engl J Med, , Apr 02. [doi] [Medline: 32240581]; Calton, B, Abedini, N, Fratkin, M., Telemedicine in the Time of Coronavirus (2020) J Pain Symptom Manage, , Mar 31. [doi] [Medline: 32240756]; Portnoy, J, Waller, M, Elliott, T., Telemedicine in the Era of COVID-19 (2020) J Allergy Clin Immunol Pract, 8 (5), pp. 1489-1491. , May; [FREE Full _text] [doi] [Medline: 32220575]; Hollander, JE, Carr, BG., Virtually Perfect? Telemedicine for Covid-19 (2020) N Engl J Med, 382 (18), pp. 1679-1681. , Apr 30; [doi] [Medline: 32160451]; (2016) Charting the Future Together: The NHLBI Strategic Mission. Strategic Vision, , https://www.nhlbi.nih.gov/sites/default/files/2017-11/NHLBI-Strategic-Vision-2016_FF.pdf, Bethesda, MD: National Heart, Lung, and Blood Institute, National Institutes of Health; [accessed 2021-03-15]; Centers for Medicare and Medicaid Services, , https://www.medicare.gov/coverage/telehealth, Medicare Advantage Plan, Medicare Part B (Medical Insurance), [accessed 2021-03-15]; Elliott, VL., Congressional Research Service, , https://www.senate.gov/CRSpubs/757e3b90-ff10-497c-8e8c-ac1bdbdb3aaf.pdf, Telehealth and Telemedicine: Description and Issues, [accessed 2021-03-14]},
correspondence_address1={Gamble, A.; Department of Preventive Medicine, 2500 N State St, United States; email: agamble2@umc.edu},
publisher={JMIR Publications Inc.},
issn={19290748},
language={English},
abbrev_source_title={JMIR Res. Prot.},
document_type={Article},
source={Scopus},
}

@ARTICLE{Kracht2021100,
author={Kracht, C.L. and Redman, L.M. and Casey, P.H. and Krukowski, R.A. and Andres, A.},
title={Association between Home Environment in Infancy and Child Movement Behaviors},
journal={Childhood Obesity},
year={2021},
volume={17},
number={2},
pages={100-109},
doi={10.1089/chi.2020.0319},
note={cited By 0},
url={https://www.scopus.com/inward/record.uri?eid=2-s2.0-85102880306&doi=10.1089%2fchi.2020.0319&partnerID=40&md5=61b0e22b62bee425f01d43a532d4e612},
affiliation={Pennington Biomedical Research Center, Baton Rouge, LA, United

States; Pediatrics, University of Arkansas for Medical Sciences and Arkansas Children's Nutrition Center, Little Rock, AR, United States; Department of Preventive Medicine, University of Tennessee Health Science Center, Memphis, TN, United States},
abstract={Introduction: An adequate balance of movement behaviors, including physical activity (PA), sleep, and screen time, is important for preventing excess weight gain in children. This study examined the relationship between the infant home environment and movement behaviors later in life. Methods: Pregnant women were recruited for a cohort study related to maternal and child development. The home environment was assessed for developmental stimulation, organization, and toys by the Pediatric Review of Children's Environmental Support and Stimulation (PROCESS) questionnaire when the child was 6 months of age. At 2 years of age, mother-reported child screen time, and child PA and sleep duration were estimated by accelerometry. Child behaviors were compared with the 24-hour Movement Guidelines (≥180 minutes/day of total PA, 11-14 hours/day of sleep, and ≤1 hour/day of screen time). Logistic regression was used to assess the relationship between the home environment and movement behaviors, adjusting for maternal and child covariates. Results: Mother/child dyads (n = 141) were mainly white (84.4%), and middle (32.8%) or low income (48.9%). All children (100%) met the PA guideline, some met the sleep guideline (71.6%), fewer met the screen-time guideline (44.7%), and only one-third (34.0%) met all three guidelines. Children who met the screen-time guideline lived in homes with more developmental stimulation and toys ($p < 0.05$). Children who met all 3 guidelines lived in homes with more organization and toys ($p < 0.05$). Conclusion: The infant home environment was associated with appropriate amounts of movement behaviors at 2 years. Promoting organization (i.e., routines) and toys in infancy may help facilitate nonscreen-based habits and healthy development. The clinical trial registration number is NCT01131117. © Copyright 2021, Mary Ann Liebert, Inc., publishers 2021.},
author_keywords={exercise; family; parent; television},
references={Hjorth, MF, Chaput, JP, Damsgaard, CT, Low physical activity level and short sleep duration are associated with an increased cardio-metabolic risk profile: A longitudinal study in 8-11 year old Danish children (2014) PLoS One, 9, p. e104677; Janssen, I, Leblanc, AG., Systematic review of the health benefits of physical activity and fitness in school-aged children and youth (2010) Int J Behav Nutr Phys Act, 7, p. 40; Mendoza, JA, Zimmerman, FJ, Christakis, DA., Television viewing, computer use, obesity, and adiposity in US preschool children (2007) Int J Behav Nutr Phys Act, 4, p. 44; Kaar, JL, Schmiege, SJ, Kalkwarf, HJ, Longitudinal assessment of sleep trajectories during early childhood and their association with obesity (2020) Child Obes, 16, pp. 211-217; Hart, CN, Jelalian, E, Raynor, HA., Behavioral and social routines and biological rhythms in prevention and treatment of pediatric obesity (2020) Am Psychol, 75, pp. 152-162; Tremblay, MS, Carson, V, Chaput, JP, Canadian 24-Hour Movement Guidelines for Children and Youth: An integration of physical activity, sedentary behaviour, and sleep (2016) Appl Physiol Nutr Metab, 41 (6), pp. S311-S327. , (Suppl 3); Ansari, M., (2019) WHO Guidelines on Physical Activity, Sedentary Behaviour and Sleep for Children Under 5 Years of Age, , World Health Organization: Geneva, Licence: CC BY-NC-SA 3.0 IGO. 2019; Lee, EY, Hesketh, KD, Hunter, S, Meeting new Canadian 24-Hour Movement Guidelines for the Early Years and associations with adiposity among toddlers living in Edmonton, Canada (2017) BMC Public Health, 17, p. 840. , (Suppl 5); Cliff, DP, McNeill, J, Vella, SA, Adherence to 24-Hour Movement Guidelines for the Early Years and associations with social-cognitive development among Australian preschool children (2017) BMC Public Health, 17, p. 857. , (Suppl

5); Kracht, CL, Webster, EK, Staiano, AE., Sociodemographic differences in young children meeting 24-Hour Movement Guidelines (2019) J Phys Act Health, 16, pp. 908-915; Rhodes, RE, Guerrero, MD, Vanderloo, LM, Development of a consensus statement on the role of the family in the physical activity, sedentary, and sleep behaviours of children and youth (2020) Int J Behav Nutr Phys Act, 17, p. 74; Hnatiuk, JA, DeDecker, E, Hesketh, KD, Cardon, G., Maternal-child co-participation in physical activity-related behaviours: Prevalence and cross-sectional associations with mothers and children's objectively assessed physical activity levels (2017) BMC Public Health, 17, p. 506; Emond, JA, Tantum, LK, Gilbert-Diamond, D, Household chaos and screen media use among preschool-aged children: A cross-sectional study (2018) BMC Public Health, 18, p. 1210; Boles, RE, Halbower, AC, Daniels, S, Family chaos and child functioning in relation to sleep problems among children at risk for obesity (2017) Behav Sleep Med, 15, pp. 114-128; Zhang, M, Quick, V, Jin, Y, Martin-Biggers, J., Associations of mother's behaviors and home/neighborhood environments with preschool children's physical activity behaviors (2020) Am J Health Promot, 34, pp. 83-86; Haines, J, McDonald, J, O'Brien, A, Healthy habits, happy homes: Randomized trial to improve household routines for obesity prevention among preschool-aged children (2013) JAMA Pediatr, 167, pp. 1072-1079; Khatiwada, A, Shoaibi, A, Neelon, B, Household chaos during infancy and infant weight status at 12 months (2018) Pediatr Obes, 13, pp. 607-613; Vlasblom, E, van Grieken, A, Beltman, M, Parenting support to prevent overweight during regular well-child visits in 0-3 year old children (BBOFT+ program), a cluster randomized trial on the effectiveness on child BMI and health behaviors and parenting (2020) PLoS One, 15, p. e0237564; Messito, MJ, Katzow, MW, Mendelsohn, AL, Gross, RS., Starting early program impacts on feeding at infant 10 months age: A randomized controlled trial (2020) Child Obes, 16 (S1), pp. S4-S13; Wen, LM, Rissel, C, Xu, H, Effects of telephone and short message service support on infant feeding practices, ''Tummy Time,'' and screen time at 6 and 12 months of child age: A 3-group randomized clinical trial (2020) JAMA Pediatr, 174, pp. 657-664; Kuczmarski, RJ, Ogden, CL, Guo, SS, 2000 CDC growth charts for the United States: Methods and development Vital Health Stat, (246), pp. 1-190. , 11 2002; Casey, PH, Barrett, K, Bradley, RH, Spiker, D., Pediatric clinical assessment of mother-child interaction: Concurrent and predictive validity (1993) J Dev Behav Pediatr, 14, pp. 313-317; Clark, BK, Sugiyama, T, Healy, GN, Validity and reliability of measures of television viewing time and other nonoccupational sedentary behaviour of adults: A review (2009) Obes Rev, 10, pp. 7-16; Sadeh, A, McGuire, JP, Sachs, H, Sleep and psychological characteristics of children on a psychiatric inpatient unit (1995) J Am Acad Child Adolesc Psychiatry, 34, pp. 813-819; Armstrong, B, Covington, LB, Hager, ER, Black, MM., Objective sleep and physical activity using 24-hour ankle-worn accelerometry among toddlers from low-income families (2019) Sleep Health, 5, pp. 459-465; So, K, Adamson, TM, Horne, RS., The use of actigraphy for assessment of the development of sleep/wake patterns in infants during the first 12 months of life (2007) J Sleep Res, 16, pp. 181-187; Mindell, JA, Leichman, ES, Composto, J, Development of infant and toddler sleep patterns: Real-world data from a mobile application (2016) J Sleep Res, 25, pp. 508-516; Acebo, C, Sadeh, A, Seifer, R, Sleep/wake patterns derived from activity monitoring and maternal report for healthy 1-to 5-year-old children (2005) Sleep, 28, pp. 1568-1577; Bisson, M, Tremblay, F, Pronovost, E, Accelerometry to measure physical activity in toddlers: Determination of wear time requirements for a reliable estimate of physical activity (2019) J Sports Sci, 37, pp. 298-305; Hnatiuk, JA, Ridgers, ND, Salmon, J, Hesketh, KD., Maternal correlates of young children's physical

activity across periods of the day (2017) J Sci Med Sport, 20, pp. 178-183; Hager, ER, Gormley, CE, Latta, LW, Toddler physical activity study: Laboratory and community studies to evaluate accelerometer validity and correlates (2016) BMC Public Health, 16, p. 936; Konstabel, K, Veidebaum, T, Verbestel, V, Objectively measured physical activity in European children: The IDEFICS study (2014) Int J Obes (Lond), 38, pp. S135-S143. , Suppl 2; Zhang, Z, Sousa-Sa, E, Pereira, J, Correlates of nocturnal sleep duration, nocturnal sleep variability, and nocturnal sleep problems in toddlers: Results from the GET UP! Study (2019) Sleep Med, 53, pp. 124-132; Katzmarzyk, PT, Staiano, AE., Relationship between meeting 24-Hour Movement Guidelines and cardiometabolic risk factors in children (2017) J Phys Act Health, 14, pp. 779-784; Kracht, CL, Webster, EK, Staiano, AE., Relationship between the 24-Hour Movement Guidelines and fundamental motor skills in preschoolers (2020) J Sci Med Sport, 23, pp. 1185-1190; Garriguet, D, Carson, V, Colley, RC, Physical activity and sedentary behaviour of Canadian children aged 3 to 5 (2016) Health Rep, 27, pp. 14-23; Hager, ER, Tilton, NA, Wang, Y, The home environment and toddler physical activity: An ecological momentary assessment study (2017) Pediatr Obes, 12, pp. 1-9; Hager, ER, Calamaro, CJ, Bentley, LM, Nighttime sleep duration and sleep behaviors among toddlers from low-income families: Associations with obesogenic behaviors and obesity and the role of parenting (2016) Child Obes, 12, pp. 392-400; Kuzik, N, Carson, V., The association between physical activity, sedentary behavior, sleep, and body mass index z-scores in different settings among toddlers and preschoolers (2016) BMC Pediatr, 16, p. 100; Tremblay, MS, Chaput, JP, Adamo, KB, Canadian 24-Hour Movement Guidelines for the Early Years (0-4 years): An integration of physical activity, sedentary behaviour, and sleep (2017) BMC Public Health, 17, p. 874. , (Suppl 5); Hewitt, L, Kerr, E, Stanley, RM, Okely, AD., Tummy time and infant health outcomes: A systematic review (2020) Pediatrics, 145, p. e20192168; Mendres-Smith, AE, Borrero, JC, Castillo, MI, Tummy time without the tears: The impact of parent positioning and play (2020) J Appl Behav Anal, 53, pp. 2090-2107; Adams, EL, Marini, ME, Stokes, J, INSIGHT responsive parenting intervention reduces infant's screen time and television exposure (2018) Int J Behav Nutr Phys Act, 15, p. 24; Mindell, JA, Leichman, ES, Lee, C, Implementation of a nightly bedtime routine: How quickly do things improve? (2017) Infant Behav Dev, 49, pp. 220-227; Bruijns, BA, Truelove, S, Johnson, AM, Infants' and toddlers' physical activity and sedentary time as measured by accelerometry: A systematic review and meta-analysis (2020) Int J Behav Nutr Phys Act, 17, p. 14; Staiano, AE, Webster, EK, Allen, AT, Screen-time policies and practices in early care and education centers in relationship to child physical activity (2018) Child Obes, 14, pp. 341-348; Kracht, CL, Webster, EK, Staiano, AE., A natural experiment of state-level physical activity and screen-time policy changes early childhood education (ECE) centers and child physical activity (2020) BMC Public Health, 20, p. 387},
correspondence_address1={Andres, A.; Department of Pediatrics, 15 Children s Way, Slot 512-20B, United States; email: andresaline@uams.edu},
publisher={Mary Ann Liebert Inc.},
issn={21532168},
pubmed_id={33471594},
language={English},
abbrev_source_title={Child. Obes.},
document_type={Article},
source={Scopus},
}

@ARTICLE{Nepper2021352,
author={Nepper, M.J. and McAtee, J.R. and Chai, W.},
title={Effect of a Workplace Weight-Loss Program for Overweight and Obese Healthcare Workers},
journal={American Journal of Health Promotion},
year={2021},
volume={35},
number={3},
pages={352-361},
doi={10.1177/0890117120960393},
note={cited By 0},
url={https://www.scopus.com/inward/record.uri?eid=2-s2.0-85091405302&doi=10.1177%2f0890117120960393&partnerID=40&md5=d0a747973e488863a4a0ad65e99de3d2},
affiliation={Nebraska Methodist Health System, Omaha, NE, United States; Department of Nutrition and Health Sciences, University of Nebraska-Lincoln, Lincoln, NE, United States},
abstract={Purpose: Examining the effect of a workplace weight-loss program on weight loss, and physical, behavioral and mental well-being among overweight/obese healthcare workers. Design: Quasi experimental design Setting: Single healthcare setting Participants: Forty-one (48.0 ± 11.2 years) day-time shift healthcare employees with body mass index [BMI] >29 kg/m2. Intervention: Sixteen-week program with weekly group meetings/activities and individual appointments with nutrition/health experts. Measures: Objective (weight, BMI, blood pressure) and self-reported measures were collected at baseline, conclusion of the intervention and 3 to 6 months post-intervention. Analysis: Repeated measure analysis accounting for confounders. Results: Participants had an average of 13 pounds (5.6%) weight loss (224.2 ± 6.4 vs. 211.6 ± 6.4 lbs.; P < 0.0001) upon program completion with significant decreases in BMI (37.7 ± 1.0 vs. 35.1 ± 1.0 kg/m2; P < 0.0001). Extreme obesity (BMI≥40 kg/m2) rate was reduced from 36.6% to 17.1% (P < 0.0001). There were decreases in diastolic blood pressure (76.0 ± 1.4 vs. 68.7 ± 1.5 mmHg; P = 0.001) and self-reported blood glucose (119.9 ± 4.4 vs. 105.5 ± 4.6 mg/dL; P = 0.03). Participants had improvements in weekly physical activity (25% change; P = 0.01), nutrition behavior (33% change, P < 0.0001), sleep quality (23% change, P = 0.005), and depression (72% change, P < 0.0001). Twenty-seven participants had post-intervention follow-up data. On average participants regained 8 pounds, which was less than the initial weight loss (16 lbs., N = 27). Conclusion: The results suggest the program may benefit healthcare employees. Further emphasis should be placed on post-intervention weight management to prevent weight regain. © The Author(s) 2020.},
author_keywords={healthcare setting; healthcare worker; obesity; weight loss; workplace weight-loss program},
references={Hales, C.M., Fryar, C.D., Carroll, M.D., Freedman, D.S., Ogden, C.L., Trends in obesity and severe obesity prevalence in US Youth and adults by sex and age, 2007-2008 to 2015-2016 (2018) JAMA, 319 (16), pp. 1723-1725; Flegal, K.M., Kruszon-Moran, D., Carroll, M.D., Fryar, C.D., Ogden, C.L., Trends in obesity among adults in the United States, 2005 to 2014 (2016) JAMA, 315 (21), pp. 2284-2291; Guh, D.P., Zhang, W., Bansback, N., Amarsi, Z., Birmingham, C.L., Anis, A.H., The incidence of co-morbidities related to obesity and overweight: a systematic review and meta-analysis (2009) BMC Public Health, 9 (1), p. 88; Jensen, M.D., Ryan, D.H., Apovian, C.M., 2013 AHA/ACC/TOS guideline for the management of overweight and obesity in adults: a report of the American College of Cardiology/American Heart Association Task Force on Practice Guidelines and The Obesity Society (2014) J Am Coll Cardiol, 63, pp.

2985-3023; Wilson, M.G., Holman, P.B., Hammock, A., A comprehensive review of the effects of worksite health promotion on health-related outcomes (1996) Am Journal Health Promot, 10 (6), pp. 429-435; Michaud, T.L., Nyman, J.A., Jutkowitz, E., Su, D., Dowd, B., Abraham, J.M., Effect of workplace weight management on health care expenditures and quality of life (2016) J Occup Environ Med, 58 (11), pp. 1073-1078; Gemson, D.H., Commisso, R., Fuente, J., Newman, J., Benson, S., Promoting weight loss and blood pressure control at work: impact of an education and intervention program (2008) J Occup Environ Med, 50 (3), pp. 272-281; San Mauro Martin, I., Sanz Rojo, S., Becerra, X., Garicano Vilar, E., Successful implementation of a Mediterranean weight loss program to prevent overweight and obesity in the workplace (2019) J Occup Environ Med, 61 (8), pp. e329-e332; Schroer, S., Haupt, J., Pieper, C., Evidence-based lifestyle interventions in the workplace—an overview (2014) Occup Med (Lond), 64 (1), pp. 8-12; Anderson, L.M., Quinn, T.A., Glanz, K., The effectiveness of worksite nutrition and physical activity interventions for controlling employee overweight and obesity: a systematic review (2009) Am J Prev Med, 37 (4), pp. 340-357; Giese, K.K., Cook, P.F., Reducing obesity among employees of a manufacturing plant: translating the Diabetes Prevention Program to the workplace (2014) Workplace Health Saf, 62 (4), pp. 136-141; Grimani, A., Aboagye, E., Kwak, L., The effectiveness of workplace nutrition and physical activity interventions in improving productivity, work performance and workability: a systematic review (2019) BMC Public Health, 19 (1), p. 1676; Sharma, S.V., Upadhyaya, M., Karhade, M., Are Hospital workers healthy? A study of cardiometabolic, behavioral, and psychosocial factors associated with obesity among hospital workers (2016) J Occup Environ Med, 58 (12), pp. 1231-1238; Luckhaupt, S.E., Cohen, M.A., Li, J., Calvert, G.M., Prevalence of obesity among U.S. workers and associations with occupational factors (2014) Am J Prev Med, 46 (3), pp. 237-248; Reed, J.L., Prince, S.A., Women's heart health: a focus on nurses' physical activity and sedentary behaviour (2018) Curr Opin Cardiol, 33 (5), pp. 514-520; Shan, Z., Li, Y., Zong, G., Rotating night shift work and adherence to unhealthy lifestyle in predicting risk of type 2 diabetes: results from two large US cohorts of female nurses (2018) BMJ, 363, p. k4641; Glanz, K., Bishop, D.B., The role of behavioral science theory in development and implementation of public health interventions (2010) Annu Rev Publ Health, 31, pp. 399-418; (2020) Define adult overweight and obesity, , https://www.cdc.gov/obesity/adult/defining.html, Accessed June 8, 2020; Ogedegbe, G., Pickering, T., Principles and techniques of blood pressure measurement (2010) Cardiol Clin, 28 (4), pp. 571-586; Toobert, D.J., Hampson, S.E., Glasgow, R.E., The summary of diabetes self-care activities measure: results from 7 studies and a revised scale (2000) Diabetes Care, 23 (7), pp. 943-950; (2004) Physical activity screener, , http://www.nutritionquest.com/assessment, Accessed February 1, 2017; Ainsworth, B.E., Haskell, W.L., Herrmann, S.D., Compendium of physical activities: a second update of codes and MET values (2011) Med Sci Sport Exer, 43 (8), pp. 1575-1581; Buysse, D.J., Reynolds, I.I.I.C.F., Monk, T.H., Hoch, C.C., Yeager, A.L., Kupfer, D.J., The Pittsburgh Sleep Quality Index: a new instrument for psychiatric practice and research (1989) Psychiatry Res, 28 (2), pp. 193-213; Kroenke, K., Spitzer, R.L., Williams, J.B., Lowe, B., The patient health questionnaire somatic, anxiety, and depressive symptom scales: a systematic review (2010) Gen Hosp Psychiatry, 32 (4), pp. 345-359; Cohen, J., A power primer (1992) Psychol Bull, 112 (1), pp. 115-159; Nepper, M.J., McAtee, J.R., Wheeler, L., Chai, W., Mobile phone text message intervention on diabetes self-care activities, cardiovascular disease risk awareness, and food choices among type 2 diabetes patients (2019) Nutrients, 11 (6), p. 1314; Force,

U.S.P.S.T., Curry, S.J., Krist, A.H., Behavioral weight loss interventions to prevent obesity-related morbidity and mortality in adults: us preventive services task force recommendation statement (2018) JAMA, 320 (11), pp. 1163-1171; Fitzgibbon, M.L., Stolley, M.R., Schiffer, L., Sharp, L.K., Singh, V., Dyer, A., Obesity reduction black intervention trial (ORBIT): 18-month results (2010) Obesity, 18 (12), pp. 2317-2325; Rock, C.L., Flatt, S.W., Byers, T.E., Results of the exercise and nutrition to enhance recovery and good health for you (energy) trial: a behavioral weight loss intervention in overweight or obese breast cancer survivors (2015) J Clin Oncol, 33 (28), pp. 3169-3176; Appel, L.J., Clark, J.M., Yeh, H.C., Comparative effectiveness of weight-loss interventions in clinical practice (2011) N Eng J Med, 365 (21), pp. 1959-1968; Barte, J.C., ter Bogt, N.C., Bogers, R.P., Maintenance of weight loss after lifestyle interventions for overweight and obesity, a systematic review (2010) Obesity Rev, 11 (12), pp. 899-906; Mitchell, N.S., Catenacci, V.A., Wyatt, H.R., Hill, J.O., Obesity: overview of an epidemic (2011) Psychiatr Clin North Am, 34 (4), pp. 717-732; Ramage, S., Farmer, A., Eccles, K.A., McCargar, L., Healthy strategies for successful weight loss and weight maintenance: a systematic review (2014) Appl Physiol Nutr Metab, 39 (1), pp. 1-20; Stephens, S.K., Cobiac, L.J., Veerman, J.L., Improving diet and physical activity to reduce population prevalence of overweight and obesity: an overview of current evidence (2014) Prev Med, 62, pp. 167-178; Patel, S.R., Hu, F.B., Short sleep duration and weight gain: a systematic review (2008) Obesity, 16 (3), pp. 643-653; Coughlin, J.W., Smith, M.T., Sleep, obesity, and weight loss in adults: is there a rationale for providing sleep interventions in the treatment of obesity? (2014) Int Rev Psychiatry, 26 (2), pp. 177-188; Pereira-Miranda, E., Costa, P.R.F., Queiroz, V.A.O., Pereira-Santos, M., Santana, M.L.P., Overweight and obesity associated with higher depression prevalence in adults: a systematic review and meta-analysis (2017) J Am Coll Nutr, 36 (3), pp. 223-233; Pratt, L.A., Brody, D.J., Depression and obesity in the U.S. adult household population, 2005-2010 (2014) NCHS Data Brief, (167), pp. 1-8; Linde, J.A., Simon, G.E., Ludman, E.J., A randomized controlled trial of behavioral weight loss treatment versus combined weight loss/depression treatment among women with comorbid obesity and depression (2011) Ann Behav Med, 41 (1), pp. 119-130; Pagoto, S., Schneider, K.L., Whited, M.C., Randomized controlled trial of behavioral treatment for comorbid obesity and depression in women: the Be Active Trial (2013) Int J Obes, 37 (11), pp. 1427-1434; Ma, J., Rosas, L.G., Lv, N., Effect of integrated behavioral weight loss treatment and problem-solving therapy on body mass index and depressive symptoms among patients with obesity and depression: the rainbow randomized clinical trial (2019) JAMA, 321 (9), pp. 869-879; Saksvik, I.B., Bjorvatn, B., Hetland, H., Sandal, G.M., Pallesen, S., Individual differences in tolerance to shift work—a systematic review (2011) Sleep Med Rev, 15 (4), pp. 221-235; James, S.M., Honn, K.A., Gaddameedhi, S., Van Dongen, H.P.A., Shift work: disrupted circadian rhythms and sleep-implications for health and well-being (2017) Curr Sleep Med Rep, 3 (2), pp. 104-112},
correspondence_address1={Chai, W.; Department of Nutrition and Health Sciences, United States; email: wchai2@unl.edu},
publisher={SAGE Publications Inc.},
issn={08901171},
coden={AJHPE},
pubmed_id={32969262},
language={English},
abbrev_source_title={Am. J. Health Promot.},
document_type={Article},

source={Scopus},
}

@ARTICLE{Tréhout2021325,
author={Tréhout, M. and Leroux, E. and Bigot, L. and Jego, S. and Leconte, P. and Reboursière, E. and Morello, R. and Chapon, P.-A. and Herbinet, A. and Quarck, G. and Dollfus, S.},
title={A web-based adapted physical activity program (e-APA) versus health education program (e-HE) in patients with schizophrenia and healthy volunteers: study protocol for a randomized controlled trial (PEPSY V@Si)},
journal={European Archives of Psychiatry and Clinical Neuroscience},
year={2021},
volume={271},
number={2},
pages={325-337},
doi={10.1007/s00406-020-01140-z},
note={cited By 0},
url={https://www.scopus.com/inward/record.uri?eid=2-s2.0-85085472240&doi=10.1007%2fs00406-020-01140-z&partnerID=40&md5=f1eae90b3251ed12855759c835968fb5},
affiliation={CHU de Caen Normandie, Service de Psychiatrie, Centre Esquirol, Caen, 14000, France; Normandie Univ, UNICAEN, UFR de Médecine, Caen, 14000, France; Normandie Univ, UNICAEN, ISTS EA 7466, GIP CYCERON, Caen, 14000, France; MOOVEN, Caen, 14000, France; Normandie Univ, UNICAEN/INSERM, UMR 1075, COMETE, PFRS, Caen, 14000, France; Normandie Univ, UNICAEN, UFR STAPS, Caen, 14000, France; CHU de Caen Normandie, Service de Médecine du Sport, Caen, 14000, France; CHU de Caen Normandie, Unité de Biostatistiques et Recherche Clinique, Caen, 14000, France; BodyCap, Hérouville Saint-Clair, 14200, France; MOOVEN, Saint-Mathieu-de-Treviers, 34270, France},
abstract={Patients with schizophrenia (SZ) have a high level of cardiovascular morbidity and some clinical symptoms of illness remain resistant to pharmacological approaches. A large number of studies support the effectiveness of physical activity (PA) in SZ. The aims of this trial is to assess the effects of a remote, web-based adapted PA program (e-APA) compared to a health education program (e-HE) on brain plasticity in SZ and healthy volunteers (HV) and on psychiatric, neurocognitive, circadian and physical variables. The study is an interventional, multicenter, randomized open-label trial. Forty-two SZ will be randomized to either the active group (e-APA, N = 21) or nonactive group (e-HE, N = 21), and 21 HV will be matched to SZ according to age, gender, and level of PA in both e-APA and e-HE groups. Interventions will consist of 32 sessions (2 × 60 min/week, for 16 weeks) via supervised home-based videoconferencing. Cerebral magnetic resonance imaging, psychiatric symptoms, neurocognitive and circadian rhythms assessments as well as physical tests and biological analyses will be assessed at baseline and 16 weeks after the intervention. To our knowledge, this is the first study aiming to evaluate the efficacy of APA delivered by supervised home-based videoconferencing in SZ. Moreover, using multimodal MRI, this study could clarify the pathophysiological mechanisms underlying the efficacy of APA. Finally, this innovative approach might also increase participation in long-term PA since PA-based programs are known to have low adherence and early dropout. Trial registration: ClinicalTrials.gov identifier: NCT03261817. Registered on 16 August 2017. © 2020, Springer-Verlag GmbH Germany, part of Springer Nature.},
author_keywords={Adapted physical activity; Brain plasticity; Cognition; MRI; Schizophrenia; Web},
references={Tréhout, M., Dollfus, S., Physical activity in patients with

schizophrenia: From neurobiology to clinical benefits (2018) Encephale, 44, pp. 538-547. , 10.1016/j.encep.2018.05.005 DOI; Rosenbaum, S., Tiedemann, A., Sherrington, C., Curtis, J., Ward, P.B., Physical activity interventions for people with mental illness: A systematic review and meta-analysis (2014) J Clin Psychiatry, 75, pp. 964-974. , DOI: 10.4088/JCP.13r08765; Firth, J., Cotter, J., Elliott, R., French, P., Yung, A.R., A systematic review and meta-analysis of exercise interventions in schizophrenia patients (2015) Psychol Med, 45, pp. 1343-1361. , DOI: 10.1017/s0033291714003110; Dauwan, M., Begemann, M.J., Heringa, S.M., Sommer, I.E., (2016); Firth, J., Stubbs, B., Rosenbaum, S., Vancampfort, D., Malchow, B., Schuch, F., Elliott, R., Yung, A.R., Aerobic exercise improves cognitive functioning in people with schizophrenia: A systematic review and meta-analysis (2017) Schizophr Bull, 43, pp. 546-556; Vogel, J.S., van Der Gaag, M., Slofstra, C., Knegtering, H., Bruins, J., Castelein, S., The effect of mind-body and aerobic exercise on negative symptoms in schizophrenia: A meta-analysis (2019) Psychiatry Res, 279, pp. 295-305; Lalande, D., Theriault, L., Kalinova, E., Fortin, A., Leone, M., The effect of exercise on sleep quality and psychological, physiological, and biological correlates in patients with schizophrenia: A pilot study (2016) Schizophr Res, 171, pp. 235-236; Costa, R., Bastos, T., Probst, M., Seabra, A., Vilhena, E., Corredeira, R., Sleep quality in patients with schizophrenia: The relevance of physical activity (2018) Ment Health Phys Act, 14, pp. 140-145; Lederman, O., Ward, P.B., Firth, J., Maloney, C., Carney, R., Vancampfort, D., Stubbs, B., Rosenbaum, S., Does exercise improve sleep quality in individuals with mental illness? A systematic review and meta-analysis (2019) J Psychiatr Res, 109, pp. 96-106. , DOI; Vancampfort, D., Rosenbaum, S., Ward, P.B., Stubbs, B., Exercise improves cardiorespiratory fitness in people with schizophrenia: A systematic review and meta-analysis (2015) Schizophr Res, 169, pp. 453-457. , DOI; Armstrong, H.F., Bartels, M.N., Paslavski, O., Cain, D., Shoval, H.A., Ballon, J.S., Khan, S., Kimhy, D., The impact of aerobic exercise training on cardiopulmonary functioning in individuals with schizophrenia (2016) Schizophr Res, 173, pp. 116-117. , DOI: 10.1016/j.schres.2016.03.009; Chalfoun, C., Karelis, A.D., Stip, E., Abdel-Baki, A., Running for your life: A review of physical activity and cardiovascular disease risk reduction in individuals with schizophrenia (2016) J Sports Sci, 34, pp. 1500-1515. , DOI: 10.1080/02640414.2015.1119875; Hsu, C.C., Liang, C.S., Tai, Y.M., Cheng, S.L., Incongruent changes in heart rate variability and body weight after discontinuing aerobic exercise in patients with schizophrenia (2016) Int J Psychophysiol, 109, pp. 132-137. , DOI: 10.1016/j.ijpsycho.2016.08.011; Hjorth, P., Davidsen, A.S., Kilian, R., Skrubbeltrang, C., A systematic review of controlled interventions to reduce overweight and obesity in people with schizophrenia (2014) Acta Psychiatr Scand, 130, pp. 279-289. , DOI: 10.1111/acps.12245; Holt, R.I., The prevention of diabetes and cardiovascular disease in people with schizophrenia (2015) Acta Psychiatr Scand, 132, pp. 86-96. , DOI: 10.1111/acps.12443; Kim, D.D., Lang, D.J., Warburton, D.E., Barr, A.M., Smith, G.N., Thornton, A.E., White, R.F., Procyshyn, R.M., Effects of exercise on serum triglycerides and symptoms of schizophrenia (2017) J Clin Psychopharmacol, 37, pp. 273-274. , DOI; Schmitt, A., Maurus, I., Rossner, M.J., Röh, A., Lembeck, M., von Wilmsdorff, M., Takahashi, S., Falkai, P., Effects of aerobic exercise on metabolic syndrome, cardiorespiratory fitness, and symptoms in schizophrenia include decreased mortality (2018) Front Psychiatry, 9, p. 690. , DOI: 10.3389/fpsyt.2018.00690; Vancampfort, D., Probst, M., de Hert, M., Soundy, A., Stubbs, B., Stroobants, M., de Herdt, A., Neurobiological effects of physical exercise in schizophrenia: A systematic review (2014) Disabil Rehabil, 36, pp. 1749-1754. , DOI:

10.3109/09638288.2013.874505; Firth, J., Cotter, J., Carney, R., Yung, A.R., The pro-cognitive mechanisms of physical exercise in people with schizophrenia (2017) Br J Pharmacol, 174, pp. 3161-3172. , DOI: 10.1111/bph.13772; Maurus, I., Hasan, A., Röh, A., Takahashi, S., Rauchmann, B., Keeser, D., Malchow, B., Falkai, P., Neurobiological effects of aerobic exercise, with a focus on patients with schizophrenia (2019) Eur Arch Psychiatry Clin Neurosci, 269, pp. 499-515; Farnia, V., Farshchian, F., Farshchian, N., Alikhani, M., Sadeghi Bahmani, D., Brand, S., Comparisons of voxel-based morphometric brain volumes of individuals with methamphetamine-induced psychotic disorder and schizophrenia spectrum disorder and healthy controls (2020) Neuropsychobiology, 79, pp. 170-178; Tamminga, C.A., Stan, A.D., Wagner, A.D., The hippocampal formation in schizophrenia (2010) Am J Psychiatry, 167, pp. 1178-1193; Adriano, F., Caltagirone, C., Spalletta, G., Hippocampal volume reduction in first-episode and chronic schizophrenia: A review and meta-analysis (2012) Neuroscientist, 18, pp. 180-200. , DOI; Pajonk, F.G., Wobrock, T., Gruber, O., Hippocampal plasticity in response to exercise in schizophrenia (2010) Arch Gen Psychiatry, 67, pp. 133-143. , DOI; Malchow, B., Keeser, D., Keller, K., Effects of endurance training on brain structures in chronic schizophrenia patients and healthy controls (2016) Schizophr Res, 173, pp. 182-191. , DOI: 10.1016/j.schres.2015.01.005; Scheewe, T.W., van Haren, N.E., Sarkisyan, G., Schnack, H.G., Brouwer, R.M., de Glint, M., Hulshoff Pol, H.E., Cahn, W., Exercise therapy, cardiorespiratory fitness and their effect on brain volumes: A randomised controlled trial in patients with schizophrenia and healthy controls (2013) Eur Neuropsychopharmacol, 23, pp. 675-685. , DOI: 10.1016/j.euroneuro.2012.08.008; Firth, J., Stubbs, B., Vancampfort, D., Schuch, F., Lagopoulos, J., Rosenbaum, S., Ward, P.B., Effect of aerobic exercise on hippocampal volume in humans: A systematic review and meta-analysis (2018) Neuroimage, 166, pp. 230-238. , DOI: 10.1016/j.neuroimage.2017.11.007; Haukvik, U.K., Tamnes, C.K., Söderman, E., Agartz, I., Neuroimaging hippocampal subfields in schizophrenia and bipolar disorder: A systematic review and meta-analysis (2018) J Psychiatr Res, 104, pp. 217-226. , DOI; Baglivo, V., Cao, B., Mwangi, B., Hippocampal subfield volumes in patients with first-episode psychosis (2018) Schizophr Bull, 44, pp. 552-559. , DOI; Vargas, T., Dean, D.J., Osborne, K.J., Gupta, T., Ristanovic, I., Ozturk, S., Turner, J., Mittal, V.A., Hippocampal subregions across the psychosis spectrum (2018) Schizophr Bull, 44, pp. 1091-1099. , DOI; Papiol, S., Popovic, D., Keeser, D., Hasan, A., Schneider-Axmann, T., Degenhardt, F., Rossner, M.J., Malchow, B., Polygenic risk has an impact on the structural plasticity of hippocampal subfields during aerobic exercise combined with cognitive remediation in multi-episode schizophrenia (2017) Transl Psychiatry, 7. , DOI: 10.1038/tp.2017.131; Chung, A.K., Chua, S.E., Effects on prolongation of Bazett's corrected QT interval of seven second-generation antipsychotics in the treatment of schizophrenia: A meta-analysis (2011) J Psychopharmacol, 25, pp. 646-666. , DOI: 10.1177/0269881110376685; Howell, S., Yarovova, E., Khwanda, A., Rosen, S.D., Cardiovascular effects of psychotic illnesses and antipsychotic therapy (2019) Heart, 105, pp. 1852-1859. , DOI: 10.1136/heartjnl-2017-312107; Franklin, B.A., Thompson, P.D., Al-Zaiti, S.S., Albert, C.M., Hivert, M.F., Levine, B.D., Lobelo, F., Eijsvogels, T.M.H., Exercise-related acute cardiovascular events and potential deleterious adaptations following long-term exercise training: Placing the risks into perspective-an update: A scientific statement from the american heart association (2020) Circulation, , 10.1161/cir.0000000000000749; Herbsleb, M., Schumann, A., Malchow, B., Puta, C., Schulze, P.C., Gabriel, H.W., Bär, K.J., Chronotropic incompetence of the heart is associated with exercise intolerance

in patients with schizophrenia (2018) Schizophr Res, 197, pp. 162-169. , DOI: 10.1016/j.schres.2018.02.020; Herbsleb, M., Keller-Varady, K., Wobrock, T., Hasan, A., Schmitt, A., Falkai, P., Gabriel, H.H.W., Malchow, B., The influence of continuous exercising on chronotropic incompetence in multi-episode schizophrenia (2019) Front Psychiatry, 10, p. 90. , DOI; Warburton, D.E., Charlesworth, S.A., Foulds, H.J., McKenzie, D.C., Shephard, R.J., Bredin, S.S., Qualified exercise professionals: best practice for work with clinical populations (2013) Can Fam Physician, 59, pp. 759-761. , PID: 23851545; Gudrun, D.T., Christoph, D., Bernd, D., Harald, V.S., (1990) Adapted physical activity: an interdisciplinary approach, , Springer-Verlag, Berlin; Stubbs, B., Firth, J., Berry, A., Schuch, F.B., Rosenbaum, S., Gaughran, F., Veronesse, N., Vancampfort, D., How much physical activity do people with schizophrenia engage in? A systematic review, comparative meta-analysis and meta-regression (2016) Schizophr Res, 176, pp. 431-440. , DOI; Stubbs, B., Koyanagi, A., Schuch, F., Firth, J., Rosenbaum, S., Gaughran, F., Mugisha, J., Vancampfort, D., Physical activity levels and psychosis: A mediation analysis of factors influencing physical activity target achievement among 204 186 people across 46 low- and middle-income countries (2017) Schizophr Bull, 43, pp. 536-545. , DOI; Vancampfort, D., de Hert, M., Skjerven, L.H., Gyllensten, A.L., Parker, A., Mulders, N., Nyboe, L., Probst, M., International organization of physical therapy in mental health consensus on physical activity within multidisciplinary rehabilitation programmes for minimising cardio-metabolic risk in patients with schizophrenia (2012) Disabil Rehabil, 34, pp. 1-12. , DOI: 10.3109/09638288.2011.587090; Vancampfort, D., Rosenbaum, S., Schuch, F.B., Ward, P.B., Probst, M., Stubbs, B., Prevalence and predictors of treatment dropout from physical activity interventions in schizophrenia: A meta-analysis (2016) Gen Hosp Psychiatry, 39, pp. 15-23. , DOI: 10.1016/j.genhosppsych.2015.11.008; Firth, J., Rosenbaum, S., Stubbs, B., Gorczynski, P., Yung, A.R., Vancampfort, D., Motivating factors and barriers towards exercise in severe mental illness: A systematic review and meta-analysis (2016) Psychol Med, 46, pp. 2869-2881. , DOI; Yung, A.R., Firth, J., How should physical exercise be used in schizophrenia treatment? (2017) Expert Rev Neurother, 17, pp. 213-214. , DOI: 10.1080/14737175.2017.1275571; Kolt, G.S., Rosenkranz, R.R., Vandelanotte, C., Caperchione, C.M., Maeder, A.J., Tague, R., Savage, T.N., Duncan, M.J., Using Web 2.0 applications to promote health-related physical activity: Findings from the WALK 2.0 randomised controlled trial (2017) Br J Sports Med, 51, pp. 1433-1440. , DOI: 10.1136/bjsports-2016-096890; McIntosh, J.R.D., Jay, S., Hadden, N., Whittaker, P.J., Do E-health interventions improve physical activity in young people: A systematic review (2017) Public Health, 148, pp. 140-148. , DOI: 10.1016/j.puhe.2017.04.001; Degroote, L., Plaete, J., de Bourdeaudhuij, I., Verloigne, M., van Stappen, V., de Meester, A., Poppe, L., Crombez, G., The effect of the eHealth intervention 'MyPlan 1.0' on physical activity in adults who visit general practice: a quasi-experimental trial (2018) Int J Environ Res Public Health, , DOI: 10.3390/ijerph15020228; Alvarez-Jimenez, M., Alcazar-Corcoles, M.A., González-Blanch, C., Bendall, S., McGorry, P.D., Gleeson, J.F., Online, social media and mobile technologies for psychosis treatment: A systematic review on novel user-led interventions (2014) Schizophr Res, 156, pp. 96-106. , DOI: 10.1016/j.schres.2014.03.021; Naslund, J.A., Marsch, L.A., McHugo, G.J., Bartels, S.J., Emerging mHealth and eHealth interventions for serious mental illness: A review of the literature (2015) J Ment Health, 24, pp. 321-332. , DOI; Rotondi, A.J., Eack, S.M., Hanusa, B.H., Spring, M.B., Haas, G.L., Critical design elements of e-health applications for users with severe mental illness: Singular focus, simple architecture,

prominent contents, explicit navigation, and inclusive hyperlinks (2015) Schizophr Bull, 41, pp. 440-448. , DOI; Brunette, M.F., Rotondi, A.J., Ben-Zeev, D., Gottlieb, J.D., Mueser, K.T., Robinson, D.G., Achtyes, E.D., Kane, J.M., Coordinated technology-delivered treatment to prevent rehospitalization in schizophrenia: A novel model of care (2016) Psychiatr Serv, 67, pp. 444-447. , DOI; Gaebel, W., Großimlinghaus, I., Kerst, A., Cohen, Y., Hinsche-Böckenholt, A., Johnson, B., Mucic, D., Zielasek, J., European Psychiatric Association (EPA) guidance on the quality of eMental health interventions in the treatment of psychotic disorders (2016) Eur Arch Psychiatry Clin Neurosci, 266, pp. 125-137. , DOI: 10.1007/s00406-016-0677-6; Treisman, G.J., Jayaram, G., Margolis, R.L., Pearlson, G.D., Schmidt, C.W., Mihelish, G.L., Kennedy, A., Misiuta, I.E., Perspectives on the use of ehealth in the management of patients with schizophrenia (2016) J Nerv Ment Dis, 204, pp. 620-629. , DOI: 10.1097/nmd.0000000000000471; Rotondi, A.J., Sinkule, J., Haas, G.L., Spring, M.B., Litschge, C.M., Newhill, C.E., Ganguli, R., Anderson, C.M., Designing websites for persons with cognitive deficits: Design and usability of a psychoeducational intervention for persons with severe mental illness (2007) Psychol Serv, 4, pp. 202-224. , DOI: 10.1037/1541-1559.4.3.202; Kasckow, J., Felmet, K., Appelt, C., Thompson, R., Rotondi, A., Haas, G., Telepsychiatry in the assessment and treatment of schizophrenia (2014) Clin Schizophr Relat Psychoses, 8, pp. 21-27. , DOI: 10.3371/csrp.kafe.021513; Ricci, J., Gagnon, L., Evaluation du niveau d’activité physique et de condition physique (2011) Clinic Prosport, , https://www.ffsa.asso.fr/Pages/InCadres/GestClient/Communication/SportSante/Rassemblement/Evaluation_outils/test-ricci-gagnon_actif-inactif.pdf; Garber, C.E., Blissmer, B., Deschenes, M.R., Franklin, B.A., Lamonte, M.J., Lee, I.M., Nieman, D.C., Swain, D.P., American College of Sports Medicine position stand. Quantity and quality of exercise for developing and maintaining cardiorespiratory, musculoskeletal, and neuromotor fitness in apparently healthy adults: Guidance for prescribing exercise (2011) Med Sci Sports Exerc, 43, pp. 1334-1359. , DOI: 10.1249/MSS.0b013e318213fefb; Bull, F.C., Maslin, T.S., Armstrong, T., Global Physical Activity Questionnaire (GPAQ): Nine country reliability and validity study (2009) J Phys Act Health, 6, pp. 790-804. , DOI: 10.1123/jpah.6.6.790; Borg, G., (1998) Borg's perceived exertion and pain scales. Borg's perceived exertion and pain scales, , Human Kinetics, Champaign; Yushkevich, P.A., Pluta, J.B., Wang, H., Xie, L., Ding, S.L., Gertje, E.C., Mancuso, L., Wolk, D.A., Automated volumetry and regional thickness analysis of hippocampal subfields and medial temporal cortical structures in mild cognitive impairment (2015) Hum Brain Mapp, 36, pp. 258-287. , DOI: 10.1002/hbm.22627; Wechsler, D., (1981) WAIS-R manual: Wechsler adult intelligence scale-revised, , The Psychological Corporation, New York; Wechsler, D., (2001) MEM-III, Échelle clinique de mémoire de Wechsler: manuel, , Les Éd. du Centre de psychologie appliquée, cop., Paris; Rey, A., (1964) L’examen clinique en psychologie, , Presses Universitaires de France, Paris; Stroop, J.R., Studies of interference in serial verbal reactions (1935) J Exp Psychol, 18, pp. 643-662. , DOI: 10.1037/h0054651; Reitan, R.M., Validity of the trail making test as an indicator of organic brain damage (1958) Percept Mot Ski, 8, pp. 271-276. , DOI: 10.2466/pms.1958.8.3.271; Brickenkamp, R., Zillmer, E., (1998) D2—test of attention, , Hogrefe & Huber, Göttingen; Cardebat, D., Doyon, B., Puel, M., Goulet, P., Joanette, Y., Formal and semantic lexical evocation in normal subjects. Performance and dynamics of production as a function of sex, age and educational level (1990) Acta Neurol Belg, 90, pp. 207-217. , COI: 1:STN:280:DyaK3M%2FnsF2qug%3D%3D, PID: 2124031; Kay, S.R., Fiszbein, A., Opler, L.A., The Positive and Negative Syndrome Scale

(PANSS) for schizophrenia (1987) Schizophr Bull, 13, pp. 261-276. , DOI: 10.1093/schbul/13.2.261; Dollfus, S., Mach, C., Morello, R., Self-evaluation of negative symptoms: A novel tool to assess negative symptoms (2016) Schizophr Bull, 42, pp. 571-578. , DOI: 10.1093/schbul/sbv161; Kirkpatrick, B., Strauss, G.P., Nguyen, L., Fischer, B.A., Daniel, D.G., Cienfuegos, A., Marder, S.R., The brief negative symptom scale: Psychometric properties (2011) Schizophr Bull, 37, pp. 300-305. , DOI: 10.1093/schbul/sbq059; Zigmond, A.S., Snaith, R.P., The hospital anxiety and depression scale (1983) Acta Psychiatr Scand, 67, pp. 361-370. , DOI: 10.1111/j.1600-0447.1983.tb09716.x; Guy, W., (1976) ECDEU assessment manual for psychopharmacology, , revised. US department of health, education, and welfare publication (ADM). National Institute of Mental Health, Rockville; Birchwood, M., Smith, J., Drury, V., Healy, J., Macmillan, F., Slade, M., A self-report insight scale for psychosis: Reliability, validity and sensitivity to change (1994) Acta Psychiatr Scand, 89, pp. 62-67. , DOI: 10.1111/j.1600-0447.1994.tb01487.x; Waintraud, L., Guelfi, J.D., Lancrenon, S., Rouillon, F., Validation of M. Weissman's social adaptation questionnaire in its French version (1995) Ann Med Psychol, 153, pp. 274-277. , COI: 1:STN:280:DyaK2MzksVWhtw%3D%3D; Ryff, C.D., Keyes, C.L., The structure of psychological well-being revisited (1995) J Pers Soc Psychol, 69, pp. 719-727. , DOI; Saunders, J.B., Aasland, O.G., Babor, T.F., de La Fuente, J.R., Grant, M., Development of the Alcohol Use Disorders Identification Test (AUDIT): WHO collaborative project on early detection of persons with harmful alcohol consumption-II (1993) Addiction, 88, pp. 791-804. , DOI; Heatherton, T.F., Kozlowski, L.T., Frecker, R.C., Fagerström, K.O., The fagerström test for nicotine dependence: a revision of the fagerström tolerance questionnaire (1991) Br J Addict, 86, pp. 1119-1127. , DOI; Legleye, S., Karila, L., Beck, F., Reynaud, M., Validation of the CAST, a general population Cannabis Abuse Screening Test (2007) J Subst Use, 12, pp. 233-242. , DOI: 10.1080/14659890701476532; Denise, P., Benoît, O., (2016) Actisomm. Logiciel d’analyse Des données actimétriques; Cosgrave, J., Wulff, K., Gehrman, P., Sleep, circadian rhythms, and schizophrenia: Where we are and where we need to go (2018) Curr Opin Psychiatry, 31, pp. 176-182. , DOI: 10.1097/yco.0000000000000419; Johansson, A.S., Owe-Larsson, B., Hetta, J., Lundkvist, G.B., Altered circadian clock gene expression in patients with schizophrenia (2016) Schizophr Res, 174, pp. 17-23. , DOI: 10.1016/j.schres.2016.04.029; Youngstedt, S.D., Effects of exercise on sleep (2005) Clin Sports Med, 24, p. 355. , DOI: 10.1016/j.csm.2004.12.003; Chennaoui, M., Arnal, P.J., Sauvet, F., Léger, D., Sleep and exercise: A reciprocal issue (2015) Sleep Med Rev, 20, pp. 59-72. , DOI: 10.1016/j.smrv.2014.06.008; Kimhy, D., Lauriola, V., Bartels, M.N., Armstrong, H.F., Vakhrusheva, J., Ballon, J.S., Sloan, R.P., Aerobic exercise for cognitive deficits in schizophrenia—the impact of frequency, duration, and fidelity with target training intensity (2016) Schizophr Res, 172, pp. 213-215. , DOI: 10.1016/j.schres.2016.01.055; Faul, F., Erdfelder, E., Lang, A.G., Buchner, A., G*Power 3: A flexible statistical power analysis program for the social, behavioral, and biomedical sciences (2007) Behav Res Methods, 39, pp. 175-191. , DOI; Wasserstein, R.L., Schirm, A.L., Lazar, N.A., Moving to a World beyond “p %3c 0.05 (2019) Am Stat, 73, pp. 1-19. , DOI; Zhu, W., Sadly, the earth is still round (P %3c 0.05) (2012) J Sport Health Sci, 1, pp. 9-11; Zhu, W., (2016) J Sport Health Sci, 5, pp. 77-79. , p %3c 0.05, %3c 0.01, %3c 0.001, %3c 0.0001, %3c 0.00001, %3c 0.000001, or %3c 0.0000001; Tumiel, E., Wichniak, A., Jarema, M., Lew-Starowicz, M., Nonpharmacological interventions for the treatment of cardiometabolic risk factors in people with schizophrenia—a systematic review (2019) Front Psychiatry, 10, p. 566; Marcus, B.H., Ciccolo,

J.T., Sciamanna, C.N., Using electronic/computer interventions to promote physical activity (2009) Br J Sports Med, 43, pp. 102-105; Andersson, G., Internet-delivered psychological treatments (2016) Annu Rev Clin Psychol, 12, pp. 157-179; Sharma, A., Sharma, S.D., Sharma, M., Mental health promotion: A narrative review of emerging trends (2017) Curr Opin Psychiatry, 30, pp. 339-345; van Der Stouwe, E.C.D., van Busschbach, J.T., de Vries, B., Cahn, W., Aleman, A., Pijnenborg, G.H.M., Neural correlates of exercise training in individuals with schizophrenia and in healthy individuals: A systematic review (2018) Neuroimage Clin, 19, pp. 287-301. , DOI: 10.1016/j.nicl.2018.04.018},
correspondence_address1={Dollfus, S.; CHU de Caen Normandie, France; email: dollfus-s@chu-caen.fr},
publisher={Springer Science and Business Media Deutschland GmbH},
issn={09401334},
coden={EAPNE},
pubmed_id={32458107},
language={English},
abbrev_source_title={Eur. Arch. Psychiatry Clin. Neurosci.},
document_type={Article},
source={Scopus},
}

@ARTICLE{Strassnig2021,
author={Strassnig, M.T. and Harvey, P.D. and Miller, M.L. and Depp, C.A. and Granholm, E.},
title={Real world sedentary behavior and activity levels in patients with schizophrenia and controls: An ecological momentary assessment study},
journal={Mental Health and Physical Activity},
year={2021},
volume={20},
doi={10.1016/j.mhpa.2020.100364},
art_number={100364},
note={cited By 2},
url={https://www.scopus.com/inward/record.uri?eid=2-s2.0-85096507126&doi=10.1016%2fj.mhpa.2020.100364&partnerID=40&md5=06b10fcc26400a04b49cdde2d62e2222},
affiliation={Department of Psychiatry and Behavioral Sciences, University of Miami, Miami, FL, United States; Bruce W. Carter VA Medical Center, Miami, FL, United States; University of Miami Miller School of Medicine, Miami, FL, United States; Department of Psychiatry, University of California San Diego, La Jolla, CA, United States; Psychology Service, VA San Diego, San Diego, CA, United States},
abstract={Background: People with schizophrenia often experience poor health, leading to shortened lifespans. The health of people with schizophrenia may be further exacerbated by increased sedentary behavior, which independently predicts health risk in the general population. However, the prevalence and patterns of objectively measured sedentary behavior in schizophrenia have not been studied extensively on a momentary basis. Methods: Activity of 100 patients with schizophrenia was compared to that of healthy controls (HC; n = 71) using ecological momentary assessment (EMA). EMA provides real-time, real-world monitoring of behavior. We sampled behavior seven times per day for seven days, quantifying active versus inactive behaviors and four different movement patterns (recumbent, seated, standing, and moving). Due to different employment rates between samples, we focused on surveys completed at home. Results: Four of the five most commonly reported activities for participants with schizophrenia involved sitting or lying down. When considering activity

during the last hour, participants with schizophrenia were more likely to be sitting or pacing and less likely to be standing than HC. If participants with schizophrenia only did one thing in the last hour, it was more likely to involve sitting and less likely to involve standing compared to HC. Discussion: People with schizophrenia were significantly more likely to be seated and less likely to be standing or active during the past hour than HC, despite high frequencies of seated behaviors in the HC as well. The adverse health consequences of sitting for extended periods may be especially relevant for people with schizophrenia and likely contribute to premature mortality in this population. © 2020 Elsevier Ltd},
author_keywords={Ecological momentary assessment; Everyday activities; Experience sampling method; Physical health; Schizophrenia; Sedentary behavior},
keywords={adult; Article; controlled study; ecological momentary assessment; female; home care; human; leisure; major clinical study; male; middle aged; movement (physiology); music; negative syndrome; physical activity; physical inactivity; priority journal; radio; recumbency; schizoaffective psychosis; schizophrenia; sedentary lifestyle; self care; sitting; standing; television viewing; vocation},
references={Arango, C., Garibaldi, G., Marder, S.R., Pharmacological approaches to treating negative symptoms: A review of clinical trials (2013) Schizophrenia Research, 150 (2-3), pp. 346-352; Biddle, S.J.H., García Bengoechea, E., Pedisic, Z., Bennie, J., Vergeer, I., Wiesner, G., Screen time, other sedentary behaviours, and obesity risk in adults: A review of reviews (2017) Current Obesity Reports, 6 (2), pp. 134-147; Booth, F., Lees, S., Fundamental questions about genes, inactivity, and chronic diseases (2007) Physiological Genomics, 28, pp. 146-157; Bora, E., Akdede, B.B., Alptekin, K., The relationship between cognitive impairment in schizophrenia and metabolic syndrome: A systematic review and meta-analysis (2017) Psychological Medicine, 47 (6), pp. 1030-1040; Bort-Roig, J., Gilson, N.D., Puig-Ribera, A., Contreras, R.S., Trost, S.G., Measuring and influencing physical activity with smartphone technology: A systematic review (2014) Sports Medicine, 44 (5), p. 671-686; Brownson, R.C., Boehmer, T.K., Luke, D.A., Declining rates of physical activity in the United States: What are the contributors? (2005) Annual Review of Public Health, 26, pp. 421-443; Buckley, P.F., Stahl, S.M., Pharmacological treatment of negative symptoms of schizophrenia: Therapeutic opportunity or cul-de-sac? (2007) Acta Psychiatrica Scandinavica, 115 (2), pp. 93-100; Charlson, F.J., Ferrari, A.J., Santomauro, D.F., Diminic, S., Stockings, E., Scott, J.G., Global epidemiology and burden of schizophrenia: Findings from the global burden of disease study 2016 (2018) Schizophrenia Bulletin, 44 (6), pp. 1195-1203; Correll, C.U., Solmi, M., Veronese, N., Bortolato, B., Rosson, S., Santonastaso, P., Prevalence, incidence and mortality from cardiovascular disease in patients with pooled and specific severe mental illness: A large-scale meta-analysis of 3,211,768 patients and 113,383,368 controls (2017) World Psychiatry, 16 (2), pp. 163-180; Depp, C.A., Bashem, J., Moore, R.C., Holden, J.L., Mikhael, T., Swendsen, J., GPS mobility as a digital biomarker of negative symptoms in schizophrenia: A case control study (2019) NPJ Digital Medicine, 2, p. 108; Depp, C.A., Moore, R.C., Perivoliotis, D., Granholm, E., Technology to assess and support self-management in serious mental illness (2016) Dialogues in Clinical Neuroscience, 18 (2), pp. 171-183; Depp, C.A., Moore, R.C., Perivoliotis, D., Holden, J.L., Swedndsen, J., Granholm, E.L., Social behavior, interaction appraisals, and suicidal ideation in schizophrenia: The dangers of being alone (2016) Schizophrenia Research, 172 (1-3), pp. 195-200; Fisher, M., Loewy, R., Carter, C., Lee, A., Ragland, J.D., Niendam, T.,

Neuroplasticity-based auditory training via laptop computer improves cognition in young individuals with recent onset schizophrenia (2015) Schizophrenia Bulletin, 41 (1), p. 250-258; Gould, F., Sabbag, S., Durand, D., Patterson, T.L., Harvey, P.D., Self-assessment of functional ability in schizophrenia: Milestone achievement and its relationship to accuracy of self-evaluation (2013) Psychiatry Research, 207 (1-2), pp. 19-24; Granholm, E., Holden, J.L., Mikhael, T., Link, P.C., Swendsen, J., Depp, C., What do people with schizophrenia do all day? Ecological momentary assessment of real-world functioning in schizophrenia (2020) Schizophrenia Bulletin, 46 (2), pp. 242-251; Granholm, E., Loh, C., Swendsen, J., Feasibility and validity of computerized ecological momentary assessment in schizophrenia (2008) Schizophrenia Bulletin, 34 (3), pp. 507-514; Hamilton, M.T., Hamilton, D.G., Zderic, T.W., Role of low energy expenditure and sitting in obesity, metabolic syndrome, type 2 diabetes, and cardiovascular disease (2007) Diabetes, 56 (11), pp. 2655-2667; Hamilton, M.T., Healy, G.N., Dunstan, D.W., Zderic, T.W., Owen, N., Too little exercise and too much sitting: Inactivity physiology and the need for new recommendations on sedentary behavior (2008) Current Cardiovascular Risk Reports, 2 (4), pp. 292-298; Harvey, P.D., Strassnig, M., Predicting the severity of everyday functional disability in people with schizophrenia: Cognitive deficits, functional capacity, symptoms, and health status (2012) World Psychiatry, 11 (2), pp. 73-79; Harvey, P.D., Strassnig, M.T., Cognition and disability in schizophrenia: Cognition-related skills deficits and decision-making challenges add to morbidity (2019) World Psychiatry, 18 (2), pp. 165-167; Hennekens, C.H., Increasing global burden of cardiovascular disease in general populations and patients with schizophrenia (2007) Journal of Clinical Psychiatry, 68, pp. 4-7; Janney, C.A., Ganguli, R., Richardson, C.R., Holleman, R.G., Tang, G., Cauley, J.A., Sedentary behavior and psychiatric symptoms in overweight and obese adults with schizophrenia and schizoaffective disorders (WAIST Study) (2013) Schizophrenia Research, 145 (1-3), pp. 63-68; Jones, M.T., Deckler, E., Laurrari, C., Fredrik Jarskog, L., Penn, D.L., Pinkham, A.E., Confidence, performance, and accuracy of self-assessment of social cognition: A comparison of schizophrenia patients and healthy controls (2019) Schizophrenia Research: Cognition, 19. , 002-2; Keefe, R., Davis, V.G., Atkins, A.S., Vaughan, A., Patterson, T., Narasimhan, M., Validation of a computerized test of functional capacity (2016) Schizophrenia Research, 175 (1-3), pp. 90-96; Lee, I.M., Shiroma, E.J., Lobelo, F., Puska, P., Blair, S.N., Katzmarzyk, P.T., Effect of physical inactivity on major non-communicable diseases worldwide: An analysis of burden of disease and life expectancy (2012) The Lancet, 380, pp. 219-229; Liao, Y., Skelton, K., Dunton, G., Bruening, M., A systematic review of methods and procedures used in ecological momentary assessments of diet and physical activity research in youth: An adapted STROBE checklist for reporting EMA studies (CREMAS) (2016) Journal of Medical Internet Research, 18 (6); Loewy, R., Fisher, M., Schlosser, D.A., Biagianti, B., Stuart, B., Mathalon, D.H., Intensive auditory cognitive training improves verbal memory in adolescents and young adults at clinical high risk for psychosis (2016) Schizophrenia Bulletin, 42, pp. S118-S126; Naslund, J.A., Aschbrenner, K.A., Bartels, S.J., Wearable devices and smartphones for activity tracking among people with serious mental illness (2016) Mental Health and Physical Activity, 10, p. 10-17; Naslund, J.A., Aschbrenner, K.A., Scherer, E.A., McHugo, G.J., Marsch, L.A., Bartels, S.J., Wearable devices and mobile technologies for supporting behavioral weight loss among (2016); Owen, N., Sparling, P.B., Healy, G.N., Dunstan, D.W., Matthews, C.E., Sedentary behavior: Emerging evidence for a new health risk (2010) Mayo Clinic Proceedings, 85 (12), pp. 1138-1141; Panahi, S., Tremblay, A.,

Sedentariness and health: Is sedentary behavior more than just physical inactivity? (2018) Frontiers in Public Health, 6, p. 258; Patterson, R., McNamara, E., Tainio, M., de Sá, T.H., Smith, A.D., Sharp, S.J., Sedentary behaviour and risk of all-cause, cardiovascular and cancer mortality, and incident type 2 diabetes: A systematic review and dose response meta-analysis (2018) European Journal of Epidemiology, 33 (9), pp. 811-829; de Rezende, L.F., Rodrigues Lopes, M., Rey-López, J.P., Matsudo, V.K., Luiz Odo, C., Sedentary behavior and health outcomes: An overview of systematic reviews (2014) PloS One, 9 (8); Rosenbaum, S., Morell, R., Abdel-Baki, A., Ahmadpanah, M., Anilkumar, T.V., Baie, L., Assessing physical activity in people with mental illness: 23-country reliability and validity of the simple physical activity questionnaire (SIMPAQ) (2020) BMC Psychiatry, 20 (1), p. 108; Sabbag, S., Twamley, E.M., Vella, L., Heaton, R.K., Patterson, T.L., Harvey, P.D., Assessing everyday functioning in schizophrenia: Not all informants seem equally informative (2011) Schizophrenia Research, 131 (1-3), p. 250-255; Sabbag, S., Twamley, E.W., Vella, L., Heaton, R.K., Patterson, T.L., Harvey, P.D., Predictors of the accuracy of self assessment of everyday functioning in people with schizophrenia (2012) Schizophrenia Research, 137 (1-3), pp. 190-195; Scheewe, T.W., Jörg, F., Takken, T., Deenik, J., Vancampfort, D., Backx, F.J.G., Low physical activity and cardiorespiratory fitness in people with schizophrenia: A comparison with matched healthy controls and associations with mental and physical health (2019) Frontiers in Psychiatry, 10, p. 87; Semkovska, M., Bédard, M.A., Godbout, L., Limoge, F., Stip, E., Assessment of executive dysfunction during activities of daily living in schizophrenia (2004) Schizophrenia Research, 69 (2-3), pp. 289-300; Strassnig, M., Brar, J.S., Ganguli, R., Increased caffeine and nicotine consumption in community-dwelling patients with schizophrenia (2006) Schizophrenia Research, 86 (1-3), pp. 269-275; Strassnig, M.T., Caceda, R., Newcomer, J.W., Harvey, P.D., Cognitive deficits, obesity, and disability in Schizophrenia (2012) Translational Neuroscience, 3, pp. 345-354; Strassnig, M., Cornacchio, D., Harvey, P.D., Kotov, R., Fochtmann, L., Bromet, E.J., Health status and mobility limitations are associated with residential and employment status in schizophrenia and bipolar disorder (2017) Journal of Psychiatriac Research, 94, p. 180-185; Strassnig, M., Kotov, R., Cornaccio, D., Fochtmann, L., Harvey, P.D., Bromet, E.J., Twenty-year progression of body mass index in a county-wide cohort of people with schizophrenia and bipolar disorder identified at their first episode of psychosis (2017) Bipolar Disorders, 19 (5), pp. 336-343; Strassnig, M., Kotov, R., Fochtmann, L., Kalin, M., Bromet, E.J., Harvey, P.D., Associations of independent living and labor force participation with impairment indicators in schizophrenia and bipolar disorder at 20-year follow-up (2018) Schizophrenia Research, 197, p. 150-155; Stubbs, B., Williams, J., Gaughran, F., Craig, T., How sedentary are people with psychosis? A systematic review and meta-analysis (2016) Schizophrenia Research, 171 (1-3), pp. 103-109; Thorp, A.A., Owen, N., Neuhaus, M., Dunstan, D.W., Sedentary behaviors and subsequent health outcomes in adults a systematic review of longitudinal studies, 1996-2011 (2011) American Journal of Preventative Medicine, 41 (2), pp. 207-215; Ventura, J., Welikson, T., Ered, A., Subotnik, K.L., Keefe, R., Hellemann, G.S., Virtual reality assessment of functional capacity in the early course of schizophrenia: Associations with cognitive performance and daily functioning (2020) Early intervention in psychiatry, 14 (1), pp. 106-114},
correspondence_address1={Strassnig, M.T.; Associate Professor of Psychiatry University of Miami at PBC 16158 South Military Trail Delray BeachUnited States; email: strassnigm@schmcinc.org},

publisher={Elsevier Ltd},
issn={17552966},
language={English},
abbrev_source_title={Ment. Health Phys. Act.},
document_type={Article},
source={Scopus},
}

@ARTICLE{Varma2021,
author={Varma, N. and Cygankiewicz, I. and Turakhia, M. and Heidbuchel, H. and Hu, Y. and Chen, L.Y. and Couderc, J.-P. and Cronin, E.M. and Estep, J.D. and Grieten, L. and Lane, D.A. and Mehra, R. and Page, A. and Passman, R. and Piccini, J. and Piotrowicz, E. and Piotrowicz, R. and Platonov, P.G. and Ribeiro, A.L. and Rich, R.E. and Russo, A.M. and Slotwiner, D. and Steinberg, J.S. and Svennberg, E.},
title={2021 ISHNE/ HRS/ EHRA/ APHRS collaborative statement on mHealth in Arrhythmia Management: Digital Medical Tools for Heart Rhythm Professionals: From the International Society for Holter and Noninvasive Electrocardiology/Heart Rhythm Society/European Heart Rhythm Association/Asia Pacific Heart Rhythm Society},
journal={Annals of Noninvasive Electrocardiology},
year={2021},
volume={26},
number={2},
doi={10.1111/anec.12795},
art_number={e12795},
note={cited By 1},
url={https://www.scopus.com/inward/record.uri?eid=2-s2.0-85102311526&doi=10.1111%2fanec.12795&partnerID=40&md5=0ccc609ceadc4a711da4466841d0ac20},
affiliation={Cleveland Clinic, Cleveland, OH, United States; Medical University of Lodz, Lodz, Poland; Stanford University, Palo Alto, CA, United States; Antwerp University and University Hospital, Antwerp, Belgium; Taipei Veterans General Hospital, Taipei, Taiwan; University of Minnesota, Minneapolis, MN, United States; University of Rochester, Rochester, NY, United States; Temple University, Philadelphia, PA, United States; Hasselt University, Hasselt, Belgium; University of Liverpool, Liverpool, United Kingdom; Northwestern University Feinberg School of Medicine, Chicago, IL, United States; Duke University, Durham, NC, United States; National Institute of Cardiology, Warsaw, Poland; Lund University, Lund, Sweden; Faculdade de Medicina, Centro de Telessaúde, Hospital das Clínicas, and Departamento de Clínica Médica, Universidade Federal de Minas Gerais, Belo Horizonte, Brazil; Cooper Medical School of Rowan University, Camden, NJ, United States; Cardiology Division, NewYork-Presbyterian Queens, and School of Health Policy and Research, Weill Cornell Medicine, New York, NY, United States; Karolinska University Hospital, Stockholm, Sweden},
abstract={This collaborative statement from the International Society for Holter and Noninvasive Electrocardiology/ Heart Rhythm Society/ European Heart Rhythm Association/ Asia Pacific Heart Rhythm Society describes the current status of mobile health ("mHealth") technologies in arrhythmia management. The range of digital medical tools and heart rhythm disorders that they may be applied to and clinical decisions that may be enabled are discussed. The facilitation of comorbidity and lifestyle management (increasingly recognized to play a role in heart rhythm disorders) and patient self-management are novel aspects of mHealth. The promises of predictive analytics but also operational

challenges in embedding mHealth into routine clinical care are explored. This article has been co-published with the permission of Annals of Noninvasive Electrocardiology, Cardiovascular Digital Health Journal, Circulation: Arrhythmia and Electrophysiology, European Heart Journal - Digital Health and Journal of Arrhythmia. All rights reserved. © 2021 The Authors.},
author_keywords={arrhythmias; atrial fibrillation; comorbidities; digital medicine; heart rhythm; mHealth},
references={Abraham, W.T., Adamson, P.B., Bourge, R.C., Aaron, M.F., Costanzo, M.R., Stevenson, L.W., Yadav, J.S., Wireless pulmonary artery haemodynamic monitoring in chronic heart failure: A randomised controlled trial (2011) Lancet, 377, pp. 658-666. , https://doi.org/10.1016/S0140-6736(11)60101-3; Chung, M.K., Eckhardt, L.L., Chen, L.Y., Ahmed, H.M., Gopinathannair, R., Joglar, J.A., Trulock, K.M., American Heart Association Electrocardiography and Arrhythmias Committee and Exercise, Cardiac Rehabilitation, and Secondary Prevention Committee of the Council on Clinical Cardiology; Council on Arteriosclerosis, Thrombosis and Vascular Biology; Council on Cardiovascular and Stroke Nursing; and Council on Lifestyle and Cardiometabolic Health. Lifestyle and Risk Factor Modification for Reduction of Atrial Fibrillation: A Scientific Statement from the American Heart Association (2020) Circulation, 141. , https://doi.org/10.1161/CIR.0000000000000748; Hindricks, G., Taborsky, M., Glikson, M., Heinrich, U., Schumacher, B., Katz, A., Søgaard, P., IN-TIME study group. Implant-based multiparameter telemonitoring of patients with heart failure (IN-TIME): A randomised controlled trial (2014) Lancet, 384, pp. 583-590; Slotwiner, D.J., Abraham, R.L., Al-Khatib, J.G., Anderson, H.V., Bunch, T.J., Ferrara, M.G., Wilkoff, B.L., HRS white paper on interoperability of data from cardiac implantable electronic devices (CIEDs) (2019) Heart Rhythm, 16. , https://doi.org/10.1016/j.hrthm.2019.05.002; Turakhia, M.P., Desai, S.A., Harrington, R.A., The outlook of digital health for cardiovascular medicine: challenges but also extraordinary opportunities (2016) Journal of the American Medical Association Cardiol, 1, pp. 743-744; Varma, N., Epstein, A.E., Irimpen, A., Schweikert, R., Love, C., Efficacy and safety of automatic remote monitoring for implantable cardioverter-defibrillator follow-up (2010) Circulation, 122, pp. 325-332; Varma, N., Ricci, R.P., Telemedicine and cardiac implants: What is the benefit? (2013) European Heart Journal, 34, pp. 1885-1895. , https://doi.org/10.1093/eurheartj/ehs388; Varma, N., Marrouche, N.F., Aguinaga, L., Albert, C.M., Arbelo, E., Choi, J.I., Varosy, P.D., HRS/EHRA/APHRS/LAHRS/ACC/AHA worldwide practical guidance for telehealth and arrhythmia monitoring during and after a pandemic (2020) Journal of the American College of Cardiology, 76, pp. 1363-1374. , https://doi.org/10.1016/j.jacc.2020.06.019; https://www.fcc.govhttps://www.fcc.gov/general/five-questions-you-can-ask-your-doctor-about-digital-health#ab; (2011) mHealth New horizons for health through mobile technologies, , https://www.who.int/goe/publications/goe_mhealth_web.pdf, Switzerland; (2018) mHealth: Use of appropriate digital technologies for public health, , https://apps.who.int/gb/ebwha/pdf_files/WHA71/A71_20-en.pdf?ua=1, Switzerland; Bumgarner, J.M., Lambert, C.T., Hussein, A.A., Cantillon, D.J., Baranowski, B., Wolski, K., Tarakji, K.G., Smartwatch algorithm for automated detection of atrial fibrillation (2018) Journal of the American College of Cardiology, 71, pp. 2381-2388. , https://doi.org/10.1016/j.jacc.2018.03.003; Chan, P.H., Wong, C.K., Poh, Y.C., Pun, L., Leung, W.W., Wong, Y.F., Siu, C.W., Diagnostic performance of a smartphone-based photoplethysmographic application for atrial fibrillation screening in a primary care setting (2016) Journal of the American Heart Association, 5. , https://doi.org/10.1161/JAHA.116.003428; Chan, P.H.,

Wong, C.K., Pun, L., Wong, Y.F., Wong, M.M., Chu, D.W., Siu, C.W., Head-to-head comparison of the AliveCor heart monitor and microlife watchBP office AFIB for atrial fibrillation screening in a primary care setting (2017) Circulation, 135, pp. 110-112. , https://doi.org/10.1161/circulationaha.116.024439; Cooke, G., Doust, J., Sanders, S., Is pulse palpation helpful in detecting atrial fibrillation? A systematic review (2006) The Journal of Family Practice, 55, pp. 130-134; Doliwa, P.S., Frykman, V., Rosenqvist, M., Short-term ECG for out of hospital detection of silent atrial fibrillation episodes (2009) Scandinavian Cardiovascular Journal, 43, pp. 163-168; Kearley, K., Selwood, M., Van den Bruel, A., Thompson, M., Mant, D., Hobbs, F.R., Fitzmaurice, D., Heneghan, C., Triage tests for identifying atrial fibrillation in primary care: A diagnostic accuracy study comparing single-lead ECG and modified BP monitors (2014) BMJ Open, 4; Lau, J.K., Lowres, N., Neubeck, L., Brieger, D.B., Sy, R.W., Galloway, C.D., Albert, D.E., Freedman, S.B., iPhone ECG application for community screening to detect silent atrial fibrillation: A novel technology to prevent stroke (2013) International Journal of Cardiology, 165, pp. 193-194; McManus, D.D., Chong, J.W., Soni, A., Saczynski, J.S., Esa, N., Napolitano, C., Chon, K.H., PULSE-SMART: pulse-based arrhythmia discrimination using a novel smartphone application (2016) Journal of Cardiovascular Electrophysiology, 27, pp. 51-57. , https://doi.org/10.1111/jce.12842; Proesmans, T., Mortelmans, C., Van Haelst, R., Verbrugge, F., Vandervoort, P., Vaes, B., Mobile phone-based use of the photoplethysmography technique to detect atrial fibrillation in primary care: Diagnostic accuracy study of the fibricheck app (2019) JMIR Mhealth and Uhealth, 7; Tieleman, R.G., Plantinga, Y., Rinkes, D., Bartels, G.L., Posma, J.L., Cator, R., Houben, R.P., Validation and clinical use of a novel diagnostic device for screening of atrial fibrillation (2014) Europace, 16, pp. 1291-1295; Wiesel, J., Fitzig, L., Herschman, Y., Messineo, F.C., Detection of atrial fibrillation using a modified microlife blood pressure monitor (2009) American Journal of Hypertension, 22, pp. 848-852; Ciconte, G., Saviano, M., Giannelli, L., Calovic, Z., Baldi, M., Ciaccio, C., Pappone, C., Atrial fibrillation detection using a novel three-vector cardiac implantable monitor: The atrial fibrillation detect study (2017) Europace, 19, pp. 1101-1108. , https://doi.org/10.1093/europace/euw181; Hindricks, G., Pokushalov, E., Urban, L., Taborsky, M., Kuck, K.H., Lebedev, D., Pürerfellner, H., XPECT Trial Investigators. Performance of a new leadless implantable cardiac monitor in detecting and quantifying atrial fibrillation: Results of the XPECT trial (2010) Circulation Arrhythmia and Electrophysiology, 3, pp. 141-147. , https://doi.org/10.1161/circep.109.877852; Mittal, S., Rogers, J., Sarkar, S., Koehler, J., Warman, E.N., Tomson, T.T., Passman, R.S., Real-world performance of an enhanced atrial fibrillation detection algorithm in an insertable cardiac monitor (2016) Heart Rhythm, 13, pp. 1624-1630. , https://doi.org/10.1016/j.hrthm.2016.05.010; Musat, D.L., Milstein, N., Mittal, S., Implantable loop recorders for cryptogenic stroke (plus real-world atrial fibrillation detection rate with implantable loop recorders) (2018) Cardiac Electrophysiology Clinics, 10, pp. 111-118. , https://doi.org/10.1016/j.ccep.2017.11.011; Nölker, G., Mayer, J., Boldt, L.-H., Seidl, K., Van driel, V., Massa, T., Lewalter, T., Performance of an implantable cardiac monitor to detect atrial fibrillation: Results of the DETECT AF study (2016) Journal of Cardiovascular Electrophysiology, 27, pp. 1403-1410. , https://doi.org/10.1111/jce.13089; Sakhi, R., Theuns, D.A.M.J., Szili-Torok, T., Yap, S.C., Insertable cardiac monitors: Current indications and devices (2019) Expert Review of Medical Devices, 16, pp. 45-55. , https://doi.org/10.1080/17434440.2018.1557046; Sanders, P., Purerfellner, H., Pokushalov, E., Sarkar, S., Di Bacco, M., Maus, B., Dekker, L.R., Reveal LINQ

Usability Investigators. Performance of a new atrial fibrillation detection algorithm in a miniaturized insertable cardiac monitor: Results from the Reveal LINQ Usability Study (2016) Heart Rhythm, 13, pp. 1425-1430. , https://doi.org/10.1016/j.hrthm.2016.03.005; Steinberg, J.S., Varma, N., Cygankiewicz, I., Aziz, P., Balsam, P., Baranchuk, A., Piotrowicz, R., ISHNE-HRS expert consensus statement on ambulatory ECG and external cardiac monitoring/telemetry (2017) Annals of Noninvasive Electrocardiology, 22 (3). , https://doi.org/10.1111/anec.12447; Tomson, T.T., Passman, R., The Reveal LINQ insertable cardiac monitor (2015) Expert Review of Medical Devices, 12, pp. 7-18. , https://doi.org/10.1586/17434440.2014.953059; Varma, N., Marrouche, N.F., Aguinaga, L., Albert, C.M., Arbelo, E., Choi, J.I., Varosy, P.D., HRS/EHRA/APHRS/LAHRS/ACC/AHA worldwide practical guidance for telehealth and arrhythmia monitoring during and after a pandemic (2020) Journal of the American College of Cardiology, 76, pp. 1363-1374. , https://doi.org/10.1016/j.jacc.2020.06.019; Desteghe, L., Raymaekers, Z., Lutin, M., Vijgen, J., Dilling-Boer, D., Koopman, P., Heidbuchel, H., Performance of handheld electrocardiogram devices to detect atrial fibrillation in a cardiology and geriatric ward setting (2017) Europace, 19, pp. 29-39. , https://doi.org/10.1093/europace/euw025; Doliwa, P.S., Frykman, V., Rosenqvist, M., Short-term ECG for out of hospital detection of silent atrial fibrillation episodes (2009) Scandinavian Cardiovascular Journal, 43, pp. 163-168. , https://doi.org/10.1080/14017430802593435; Hendrikx, T., Rosenqvist, M., Wester, P., Sandstrom, H., Hornsten, R., Intermittent short ECG recording is more effective than 24-hour Holter ECG in detection of arrhythmias (2014) BMC Cardiovascular Disorders, 14, p. 41. , https://doi.org/10.1186/1471-2261-14-41; Kaasenbrood, F., Hollander, M., Rutten, F.H., Gerhards, L.J., Hoes, A.W., Tieleman, R.G., Yield of screening for atrial fibrillation in primary care with a hand-held, single-lead electrocardiogram device during influenza vaccination (2016) Europace, 18, pp. 1514-1520. , https://doi.org/10.1093/europace/euv426; Poulsen, M.B., Binici, Z., Dominguez, H., Soja, A.M., Kruuse, C., Hornnes, A.H., Overgaard, K., Performance of short ECG recordings twice daily to detect paroxysmal atrial fibrillation in stroke and transient ischemic attack patients (2017) International Journal of Stroke, 12, pp. 192-196. , https://doi.org/10.1177/1747493016669883; Svennberg, E., Stridh, M., Engdahl, J., Al-Khalili, F., Friberg, L., Frykman, V., Rosenqvist, M., Safe automatic one-lead electrocardiogram analysis in screening for atrial fibrillation (2017) Europace, 19, pp. 1449-1453. , https://doi.org/10.1093/europace/euw286; Tavernier, R., Wolf, M., Kataria, V., Phlips, T., Huys, R., Taghji, P., Duytschaever, M., Screening for atrial fibrillation in hospitalised geriatric patients (2018) Heart, 104, pp. 588-593. , https://doi.org/10.1136/heartjnl-2017-311981; Tieleman, R.G., Plantinga, Y., Rinkes, D., Bartels, G.L., Posma, J.L., Cator, R., Houben, R.P., Validation and clinical use of a novel diagnostic device for screening of atrial fibrillation (2014) Europace, 16, pp. 1291-1295. , https://doi.org/10.1093/europace/euu057; Vaes, B., Stalpaert, S., Tavernier, K., Thaels, B., Lapeire, D., Mullens, W., Degryse, J., The diagnostic accuracy of the MyDiagnostick to detect atrial fibrillation in primary care (2014) BMC Family Practice, 15, p. 113. , https://doi.org/10.1186/1471-2296-15-113; Barrett, P.M., Komatireddy, R., Haaser, S., Topol, S., Sheard, J., Encina, S.J., Topol, E.J., Comparison of 24-hour Holter monitoring with 14-day novel adhesive patch electrocardiographic monitoring (2014) The American Journal of Medicine, 127. , https://doi.org/10.1016/j.amjmed.2013.10.003; Rooney, M.R., Soliman, E.Z., Lutsey, P.L., Norby, F.L., Loehr, L.R., Mosley, T.H., Chen, L.Y., Prevalence and characteristics of subclinical atrial fibrillation in a community-dwelling

elderly population: The ARIC study (2019) Circulation: Arrhythmia and Electrophysiology, 12. , https://doi.org/10.1161/CIRCEP.119.007390; Rosenberg, M.A., Samuel, M., Thosani, A., Zimetbaum, P.J., Use of a noninvasive continuous monitoring device in the management of atrial fibrillation: A pilot study (2013) Pacing and Clinical Electrophysiology, 36, pp. 328-333. , PMC3618372.10.1111/pace.12053; Stehlik, J., Schmalfuss, C., Bozkurt, B., Nativi-Nicolau, J., Wohlfahrt, P., Wegerich, S., Pham, M., Continuous wearable monitoring analytics predict heart failure hospitalization: The LINK-HF multicenter study (2020) Circulation Heart Failure, 13. , https://doi.org/10.1161/CIRCHEARTFAILURE.119.006513; Turakhia, M.P., Hoang, D.D., Zimetbaum, P., Miller, J.D., Froelicher, V.F., Kumar, U.N., Heidenreich, P.A., Diagnostic utility of a novel leadless arrhythmia monitoring device (2013) American Journal of Cardiology, 112, pp. 520-524. , https://doi.org/10.1016/j.amjcard.2013.04.017; Turakhia, M.P., Ullal, A.J., Hoang, D.D., Than, C.T., Miller, J.D., Friday, K.J., Heidenreich, P.A., Feasibility of extended ambulatory electrocardiogram monitoring to identify silent atrial fibrillation in high-risk patients: The Screening Study for Undiagnosed Atrial Fibrillation (STUDY-AF) (2015) Clinical Cardiology, 38, pp. 285-292. , PMC4654330.10.1002/clc.22387; Elliot, C.A., Hamlin, M.J., Lizamore, C.A., Validity and reliability of the hexoskin wearable biometric vest during maximal aerobic power testing in elite cyclists (2019) The Journal of Strength & Conditioning Research, 33, pp. 1437-1444. , https://doi.org/10.1519/JSC.0000000000002005; Eysenck, W., Freemantle, N., Sulke, N., A randomized trial evaluating the accuracy of AF detection by four external ambulatory ECG monitors compared to permanent pacemaker AF detection (2019) Journal of Interventional Cardiac Electrophysiology, 57, pp. 361-369. , https://doi.org/10.1007/s10840-019-00515-0; Fabregat-Andres, O., Munoz-Macho, A., Adell-Beltran, G., Ibanez-Catala, X., Macia, A., Facila, L., Evaluation of a new shirt-based electrocardiogram device for cardiac screening in soccer players: Comparative study with treadmill ergospirometry (2014) Cardiology Research, 5, pp. 101-107. , https://doi.org/10.14740/cr333w; Feito, Y., Moriarty, T.A., Mangine, G., Monahan, J., The use of a smart-textile garment during high-intensity functional training: A pilot study (2019) The Journal of Sports Medicine and Physical Fitness, 59, pp. 947-954. , https://doi.org/10.23736/S0022-4707.18.08689-9; Pagola, J., Juega, J., Francisco-Pascual, J., Moya, A., Sanchis, M., Bustamante, A., Molina, C.A., CryptoAF investigators. Yield of atrial fibrillation detection with Textile Wearable Holter from the acute phase of stroke: Pilot study of Crypto-AF registry (2018) International Journal of Cardiology, 251, pp. 45-50. , https://doi.org/10.1016/j.ijcard.2017.10.063; Bumgarner, J.M., Lambert, C.T., Hussein, A.A., Cantillon, D.J., Baranowski, B., Wolski, K., Tarakji, K.G., Smartwatch algorithm for automated detection of atrial fibrillation (2018) Journal of the American College of Cardiology, 71, pp. 2381-2388. , https://doi.org/10.1016/j.jacc.2018.03.003; Chan, P.H., Wong, C.K., Poh, Y.C., Pun, L., Leung, W.W., Wong, Y.F., Siu, C.W., Diagnostic performance of a smartphone-based photoplethysmographic application for atrial fibrillation screening in a primary care setting (2016) Journal of the American Heart Association, 5 (7). , https://doi.org/10.1161/JAHA.116.003428; Chung, E.H., Guise, K.D., QTC intervals can be assessed with the AliveCor heart monitor in patients on dofetilide for atrial fibrillation (2015) Journal of Electrocardiology, 48, pp. 8-9. , https://doi.org/10.1016/j.jelectrocard.2014.10.005; Desteghe, L., Raymaekers, Z., Lutin, M., Vijgen, J., Dilling-Boer, D., Koopman, P., Heidbuchel, H., Performance of handheld electrocardiogram devices to detect atrial fibrillation

in a cardiology and geriatric ward setting (2017) Europace, 19, pp. 29-39. , https://doi.org/10.1093/europace/euw025; Garabelli, P., Stavrakis, S., Albert, M., Koomson, E., Parwani, P., Chohan, J., Po, S., Comparison of QT interval readings in normal sinus rhythm between a smartphone heart Monitor and a 12-Lead ECG for healthy volunteers and inpatients receiving sotalol or dofetilide (2016) Journal of Cardiovascular Electrophysiology, 27, pp. 827-832. , https://doi.org/10.1111/jce.12976; Halcox, J.P.J., Wareham, K., Cardew, A., Gilmore, M., Barry, J.P., Phillips, C., Gravenor, M.B., Assessment of remote heart rhythm sampling using the alivecor heart monitor to screen for atrial fibrillation: The REHEARSE-AF Study (2017) Circulation, 136, pp. 1784-1794. , https://doi.org/10.1161/circulationaha.117.030583; Lowres, N., Neubeck, L., Salkeld, G., Krass, I., McLachlan, A.J., Redfern, J., Freedman, S.B., Feasibility and cost-effectiveness of stroke prevention through community screening for atrial fibrillation using iPhone ECG in pharmacies. The SEARCH-AF study (2014) Thrombosis and Haemostasis, 111, pp. 1167-1176. , https://doi.org/10.1160/th14-03-0231; Koltowski, L., Balsam, P., Głłowczynska, R., Rokicki, J.K., Peller, M., Maksym, J., Grabowski, M., Kardia Mobile applicability in clinical practice: A comparison of Kardia Mobile and standard 12-lead electrocardiogram records in 100 consecutive patients of a tertiary cardiovascular care center (2019) Cardiology Journal, , https://doi.org/10.5603/CJ.a2019.0001, Jan 15., Online ahead of print; Tarakji, K.G., Wazni, O.M., Callahan, T., Kanj, M., Hakim, A.H., Wolski, K., Lindsay, B.D., Using a novel wireless system for monitoring patients after the atrial fibrillation ablation procedure: the iTransmit study (2015) Heart Rhythm, 12, pp. 554-559. , https://doi.org/10.1016/j.hrthm.2014.11.015; Varma, N., Marrouche, N.F., Aguinaga, L., Albert, C.M., Arbelo, E., Choi, J.I., Varosy, P.D., HRS/EHRA/APHRS/LAHRS/ACC/AHA worldwide practical guidance for telehealth and arrhythmia monitoring during and after a pandemic (2020) Journal of the American College of Cardiology, 76, pp. 1363-1374. , https://doi.org/10.1016/j.jacc.2020.06.019; https://supportapple/en/HT208931; https://apple.com/healthcare/doc/site/applehttps://apple.com/healthcare/doc/site/applewatcharrhythmiadetection; https://cardiacrhythmnews.com/kardiamobile-6l-can-be-used-to-measure-qt-duration-in-covid-19, https://cardiacrhythmnews.com/kardiamobile-6l-can-be-used-to-measure-qt-duration-in-covid-19; Conroy, T., Guzman, J.H., Hall, B., Tsouri, G., Couderc, J.P., Detection of atrial fibrillation using an earlobe photoplethysmographic sensor (2017) Physiological Measurement, 38, pp. 1906-1918. , https://doi.org/10.1088/1361-6579/aa8830; Choi, A., Shin, H., Photoplethysmography sampling frequency: Pilot assessment of how low can we go to analyze pulse rate variability with reliability? (2017) Physiol Measurement, 38, pp. 586-600; Dörr, M., Nohturfft, V., Brasier, N., Bosshard, E., Djurdjevic, A., Gross, S., Eckstein, J., The WATCH AF trial: SmartWATCHes for detection of atrial fibrillation (2019) Journal of the American College of Cardiology Clinical Electrophysiology, 5, pp. 199-208. , https://doi.org/10.1016/j.jacep.2018.10.006; Guo, Y., Wang, H., Zhang, N., Liu, T., Liang, Z., Xia, Y., Lip, G.P.H., MAFA II investigators. Mobile photoplethysmographic technology to detect atrial fibrillation (2019) Journal of the American College of Cardiology, 74, pp. 2365-2375. , https://doi.org/10.1016/j.jacc.2019.08.019; Guo, Y., Wang, H., Zhang, H., Liu, T., Liang, Z., Xia, Y., Lip, G.Y.H., MAFA II investigators. Mobile photoplethysmographic technology to detect atrial fibrillation (2019) Journal of the American College of Cardiology, 74, pp. 2365-2375. , https://doi.org/10.1016/j.jacc.2019.08.019; McManus, D.D., Lee, J., Maitas, O.,

Esa, N., Pidikiti, R., Carlucci, A., Chon, K.H., A novel application for the detection of an irregular pulse using an iPhone 4S in patients with atrial fibrillation (2013) Heart Rhythm, 10, pp. 315-319. , PMC3698570.10.1016/j.hrthm.2012.12.001; McManus, D.D., Chong, J.W., Soni, A., Saczynski, J.S., Esa, N., Napolitano, C., Chon, K.H., PULSE-SMART: Pulse-based arrhythmia discrimination using a novel smartphone application (2016) Journal of Cardiovascular Electrophysiology, 27, pp. 51-57. , PMC4768310.10.1111/jce.12842; Nemati, S., Ghassemi, M.M., Ambai, V., Isakadze, N., Levantsevych, O., Shah, A., Clifford, G.D., Monitoring and detecting atrial fibrillation using wearable technology (2016) Conference proceedings: Annual International Conference of the IEEE Engineering in Medicine and Biology Society IEEE Engineering in Medicine and Biology Society Annual Conference, 2016, pp. 3394-3397. , https://doi.org/10.1109/embc.2016.7591456; Proesmans, T., Mortelmans, C., Van Haelst, R., Verbrugge, F., Vandervoort, P., Vaes, B., Mobile phone-based use of the photoplethysmography technique to detect atrial fibrillation in primary care: Diagnostic accuracy study of the fibricheck app (2019) Journal of Medical Internet Research mHealth and uHealth, 7. , https://doi.org/10.2196/12284; Verbrugge, F.H., Proesmans, T., Vijgen, J., Mullens, W., Rivero-Ayerza, M., Van Herendael, H., Nuyens, D., Atrial fibrillation screening with photo-plethysmography through a smartphone camera (2019) Europace, 21, pp. 1167-1175. , https://doi.org/10.1093/europace/euz119; https://www.mobihealthnews.com/content/study-apple-watch-paired-deep-neural-network-detects-atrial-fibrillation-97-percent-accuracy, https://www.mobihealthnews.com/content/study-apple-watch-paired-deep-neural-network-detects-atrial-fibrillation-97-percent-accuracyhttps:mrhythmstudy.org; Chan, P.H., Wong, C.K., Pun, L., Wong, Y.F., Wong, M.M., Siu, C.W., Head-to-head comparison of the AliveCor heart monitor and microlife WatchBP office AFIB for atrial fibrillation screening in a primary care setting (2017) Circulation, 135, pp. 110-112. , https://doi.org/10.1161/CIRCULATIONAHA.116.024439; Chen, Y., Lei, L., Wang, J.G., Atrial fibrillation screening during automated blood pressure measurement-Comment on "Diagnostic accuracy of new algorithm to detect atrial fibrillation in a home blood pressure monitor" (2017) The Journal of Clinical Hypertension, 19, pp. 1148-1151. , https://doi.org/10.1111/jch.13081; Gandolfo, C., Balestrino, M., Bruno, C., Finocchi, C., Reale, N., Validation of a simple method for atrial fibrillation screening in patients with stroke (2015) Neurological Sciences, 36, pp. 1675-1678. , https://doi.org/10.1007/s10072-015-2231-0; Kane, S.A., Blake, J.R., McArdle, F.J., Langley, F., Sims, A.J., Opportunistic detection of atrial fibrillation using blood pressure monitors: A systematic review (2016) Open Heart, 3; Kario, K., Evidence and perspectives on the 24-hour management of hypertension: Hemodynamic biomarker-initiated 'anticipation medicine' for zero cardiovascular event (2016) Progress in Cardiovascular Diseases, 59, pp. 262-281. , https://doi.org/10.1016/j.pcad.2016.04.001; Kearley, K., Selwood, M., Van den Bruel, A., Thompson, M., Mant, D., Hobbs, F.R., Heneghan, C., Triage tests for identifying atrial fibrillation in primary care: A diagnostic accuracy study comparing single-lead ECG and modified BP monitors (2014) British Medical Journal Open, 4. , https://doi.org/10.1136/bmjopen-2013-004565; Marazzi, G., Iellamo, F., Volterrani, M., Lombardo, M., Pelliccia, F., Righi, D., Rosano, G., Comparison of Microlife BP A200 Plus and Omron M6 blood pressure monitors to detect atrial fibrillation in hypertensive patients (2012) Advances in Therapy, 29, pp. 64-70. , https://doi.org/10.1007/s12325-011-0087-0; Omboni, S., Verberk, W.J., Opportunistic screening of atrial fibrillation by automatic blood pressure measurement in the community (2016) British Medical Journal

Open, 6. , https://doi.org/10.1136/bmjopen-2015-010745; Stergiou, G.S., Karpettas, N., Protogerou, A., Nasothimiou, E.G., Kyriakidis, M., Diagnostic accuracy of a home blood pressure monitor to detect atrial fibrillation (2009) Journal of Human Hypertension, 23, pp. 654-658. , https://doi.org/10.1038/jhh.2009.5; Wiesel, J., Arbesfeld, B., Schechter, D., Comparison of the Microlife blood pressure monitor with the Omron blood pressure monitor for detecting atrial fibrillation (2014) American Journal of Cardiology, 114, pp. 1046-1048. , https://doi.org/10.1016/j.amjcard.2014.07.016; Wiesel, J., Fitzig, L., Herschman, Y., Messineo, F.C., Detection of atrial fibrillation using a modified microlife blood pressure monitor (2009) American Journal of Hypertension, 22, pp. 848-852. , https://doi.org/10.1038/ajh.2009.98; Wiesel, J., Salomone, T.J., Screening for atrial fibrillation in patients >/=65 years using an automatic blood pressure monitor in a skilled nursing facility (2017) American Journal of Cardiology, 120, pp. 1322-1324. , https://doi.org/10.1016/j.amjcard.2017.07.016; Jaakkola, J., Jaakkola, S., Lahdenoja, O., Hurnanen, T., Koivisto, T., Pankaala, M., Airaksinen, K.E.J., Mobile phone detection of atrial fibrillation with mechanocardiography: The MODE-AF Study (mobile phone detection of atrial fibrillation) (2018) Circulation, 137, pp. 1524-1527. , https://doi.org/10.1161/circulationaha.117.032804; Couderc, J.-P., Kyal, S., Mestha, L.K., Xu, B., Peterson, D.R., Xia, X., Hall, B., Detection of atrial fibrillation using contactless facial video monitoring (2015) Heart Rhythm, 12, pp. 195-201; Dautov, R., Savur, C., Tsouri, G., On the Effect of Face Detection on Heart Rate Estimation in Videoplethysmography (2018) IEEE Western New York Image and Signal Processing Workshop (WNYISPW), , https://doi.org/10.1109/wnyipw.2018.8576439; Takano, C., Ohta, Y., Heart rate measurement based on a time-lapse image (2007) Medical Engineering & Physics - Journal, 29, pp. 853-857; Tsouri, G.R., Li, Z., On the benefits of alternative color spaces for noncontact heart rate measurements using standard red-green-blue cameras (2015) Journal of Biomedical Optics, 20, p. 48002; Turakhia, M.P., Diagnosing With a Camera From a Distance—Proceed Cautiously and Responsibly (2020) JAMA Cardiology, 5 (1), p. 107. , https://doi.org/10.1001/jamacardio.2019.4572; Verkruysse, W., Svaasand, L.O., Nelson, J.S., Remote plethysmographic imaging using ambient light (2008) Optics Express, 16, pp. 21434-21445; Yan, B.P., Lai, W.H.S., Chan, C.K.Y., Au, A.C.K., Freedman, B., Poh, Y.C., Poh, M.Z., High-throughput, contact-free detection of atrial fibrillation from video with deep learning (2020) Journal of the American Medical Association Cardiology, 5, pp. 105-107. , https://doi.org/10.1001/jamacardio.2019.4004; Yan, B.P., Lai, W.H.S., Chan, C.K.Y., Chan, S.C., Chan, L.H., Lam, K.M., Poh, M.Z., Contact-free screening of atrial fibrillation by a smartphone using facial pulsatile photoplethysmographic signals (2018) Journal of the American Heart Association, 5 (7). , https://doi.org/10.1161/JAHA.118.008585; Chan, J., Rea, T., Gollakota, S., Sunshine, J.E., Contactless cardiac arrest detection using smart devices (2019) NPJ Digital Medicine, 2, p. 52. , https://doi.org/10.1038/s41746-019-0128-7; Wang, A., Sunshine, J.E., Gollakota, S., Contactless infant monitoring using white noise, , https://homes.cs.washington.edu/~gshyam/Papers/whitenoise.pdf; Berge, T., Lyngbakken, M.N., Ihle-Hansen, H., Brynildsen, J., Pervez, M.O., Aagaard, E.N., Tveit, A., Prevalence of atrial fibrillation and cardiovascular risk factors in a 63–65 years old general population cohort: The Akershus Cardiac Examination (ACE) 1950 Study (2018) BMJ Open, 8; Chan, P.H., Wong, C.K., Poh, Y.C., Pun, L., Leung, W.W., Wong, Y.F., Siu, C.W., Diagnostic performance of a

smartphone-based photoplethysmographic application for atrial fibrillation screening in a primary care setting (2016) Journal of the American Heart Association, 5; Doliwa, R., Sobocinski, P., Anggårdh, R.E., Frykman Kull, V., von Arbin, M., Wallén, H., Rosenqvist, M., Improved screening for silent atrial fibrillation after ischaemic stroke (2012) Europace, 2012 (14), pp. 1112-1116. , https://doi.org/10.1093/europace/eur431; Engdahl, J., Andersson, L., Mirskaya, M., Rosenqvist, M., Stepwise screening of atrial fibrillation in a 75-year-old population: Implications for stroke prevention (2013) Circulation, 127, pp. 930-937; Guo, Y., Wang, H., Zhang, H., Liu, T., Liang, Z., Xia, Y., Lip, G.Y.H., MAFA II Investigators. Mobile photoplethysmographic technology to detect atrial fibrillation (2019) Journal of the American College of Cardiology, 74, pp. 2365-2375. , https://doi.org/10.1016/j.jacc.2019.08.019; Halcox, J.P.J., Wareham, K., Cardew, A., Gilmore, M., Barry, J.P., Phillips, C., Gravenor, M.B., Assessment of Remote heart rhythm sampling using the alivecor heart monitor to screen for atrial fibrillation: The REHEARSE-AF Study (2017) Circulation, 136, pp. 1784-1794. , https://doi.org/10.1161/circulationaha.117.030583; Heckbert, S.R., Austin, T.R., Jensen, P.N., Floyd, J.S., Psaty, B.M., Soliman, E.Z., Kronmal, R.A., Yield and consistency of arrhythmia detection with patch electrocardiographic monitoring: The Multi-Ethnic Study of Atherosclerosis (2018) Journal of Electrocardiology, 51, pp. 997-1002. , https://doi.org/10.1016/j.jelectrocard.2018.07.027; Kaasenbrood, F., Hollander, M., de Bruijn, S.H.M., Dolmans, C.P.E., Tieleman, R.G., Hoes, A.W., Rutten, F.H., Opportunistic screening versus usual care for diagnosing atrial fibrillation in general practice: A cluster randomised controlled trial (2020) British Journal of General Practice, 70 (695). , https://doi.org/10.3399/bjgp20X708161; Kemp Gudmundsdottir, K., Fredriksson, T., Svennberg, E., Al-Khalili, F., Friberg, L., Frykman, V., Engdahl, J., Stepwise mass screening for atrial fibrillation using N-terminal B-type natriuretic peptide: The STROKESTOP II study (2020) Europace, 22, pp. 24-32. , https://doi.org/10.1093/europace/euz255; Lowres, N., Neubeck, L., Salkeld, G., Krass, I., McLachlan, A.J., Redfern, J., Freedman, S.B., Feasibility and cost-effectiveness of stroke prevention through community screening for atrial fibrillation using iPhone ECG in pharmacies. The SEARCH-AF study (2014) Thrombosis & Haemostasis, 111, pp. 1167-1176. , https://doi.org/10.1160/th14-03-0231; Perez, M.V., Mahaffey, K.W., Hedlin, H., Rumsfeld, J.S., Garcia, A., Ferris, T., Turakhia, M.P., Large-scale assessment of a smartwatch to identify atrial fibrillation New England Journal of Medicine, 381 (2019), pp. 1909-1917. , https://doi.org/10.1056/NEJMoa1901183; Rooney, M.R., Soliman, E.Z., Lutsey, P.L., Norby, F.L., Loehr, L.R., Mosley, T.H., Chen, L.Y., Prevalence and characteristics of subclinical atrial fibrillation in a community-dwelling elderly population (2019) Circulation: Arrhythmia and Electrophysiology, 12 (10). , https://doi.org/10.1161/CIRCEP.119.007390; Steinhubl, S.R., Waalen, J., Edwards, A.M., Ariniello, L.M., Mehta, R.R., Ebner, G.S., Topol, E.J., Effect of a home-based wearable continuous ECG Monitoring patch on detection of undiagnosed atrial fibrillation: The mSToPS randomized clinical trial (2018) Journal of the American Medical Association, 320, pp. 146-155; Svennberg, E., Engdahl, J., Al-Khalili, F., Friberg, L., Frykman, V., Rosenqvist, M., Mass screening for untreated atrial fibrillation: The STROKESTOP study (2015) Circulation, 131, pp. 2176-2184; Tavernier, R., Wolf, M., Kataria, V., Phlips, T., Huys, R., Taghji, P., Duytschaever, M., Screening for atrial fibrillation in hospitalised geriatric patients (2018) Heart, 104, pp. 588-593. , https://doi.org/10.1136/heartjnl-2017-311981; Tieleman, R.G., Plantinga, Y.,

Rinkes, D., Bartels, G.L., Posma, J.L., Cator, R., Hofman, C., Houben, R.P., Validation and clinical use of a novel diagnostic device for screening of atrial fibrillation (2014) Europace, 16, pp. 1291-1295; Turakhia, M.P., Ullal, A.J., Hoang, D.D., Than, C.T., Miller, J.D., Friday, K.J., Heidenreich, P.A., Feasibility of extended ambulatory electrocardiogram monitoring to identify silent atrial fibrillation in high-risk patients: The Screening Study for Undiagnosed Atrial Fibrillation (STUDY-AF) (2015) Clinical Cardiology, 2015 (38), pp. 285-292. , https://doi.org/10.1002/clc.22387; Gropler, M.R.F., Dalal, A.S., Van Hare, G.F., Silva, J.N.A., Can smartphone wireless ECGs be used to accurately assess ECG intervals in pediatrics? A comparison of mobile health monitoring to standard 12-lead ECG (2018) PLoS One, 13. , https://doi.org/10.1371/journal.pone.0204403; Steinberg, J.S., Varma, N., Cygankiewicz, I., Aziz, P., Balsam, P., Baranchuk, A., Piotrowicz, R., 2017 ISHNE-HRS expert consensus statement on ambulatory ECG and external cardiac monitoring/telemetry (2017) Annals of Noninvasive Electrocardiology, 22. , https://doi.org/10.1111/anec.12447; Boriani, G., Laroche, C., Diemberger, I., Fantecchi, E., Popescu, M.I., Rasmussen, L.H., Lip, G.Y., Asymptomatic atrial fibrillation: clinical correlates, management, and outcomes in the EORP-AF Pilot General Registry (2015) The American Journal of Medicine, 128, pp. 509-518.e2; Garimella, R.S., Chung, E.H., Mounsey, J.P., Schwartz, J.D., Pursell, I., Gehi, A.K., Accuracy of patient perception of their prevailing rhythm: A comparative analysis of monitor data and questionnaire responses in patients with atrial fibrillation (2015) Heart Rhythm, 12, pp. 658-665; McCabe, P.J., Chamberlain, A.M., Rhudy, L., DeVon, H.A., Symptom representation and treatment-seeking prior to diagnosis of atrial fibrillation (2015) Western Journal of Nursing Research, 38, pp. 200-215; Strickberger, S.A., Ip, J., Saksena, S., Curry, K., Bahnson, T.D., Ziegler, P.D., Relationship between atrial tachyarrhythmias and symptoms (2005) Heart Rhythm, 2, pp. 125-131; Verma, A., Champagne, J., Sapp, J., Essebag, V., Novak, P., Skanes, A., Birnie, D., Discerning the incidence of symptomatic and asymptomatic episodes of atrial fibrillation before and after catheter ablation (DISCERN AF): A prospective, multicenter study (2013) Journal of the American Medical Association Internal Medicine, 173, pp. 149-156. , https://doi.org/10.1001/jamainternmed.2013.1561; Wong, J.A., Conen, D., Van Gelder, I., McIntyre, W.F., Crijns, H.J., Wang, J., Healey, J.S., Progression of device-detected subclinical atrial fibrillation and the risk of heart failure (2018) Journal of the American College of Cardiology, 71, pp. 2603-2611; Charitos, E.I., Stierle, U., Ziegler, P.D., Baldewig, M., Robinson, D.R., Sievers, H.H., Hanke, T., A comprehensive evaluation of rhythm monitoring strategies for the detection of atrial fibrillation recurrence: Insights from 647 continuously monitored patients and implications for monitoring after therapeutic interventions (2012) Circulation, 126, pp. 806-814. , https://doi.org/10.1161/CIRCULATIONAHA.112.098079; Ding, M., Qiu, C., Atrial fibrillation, cognitive decline, and dementia: An epidemiologic review (2018) Current Epidemiology Reports, 5, pp. 252-261. , https://doi.org/10.1007/s40471-018-0159-7; Freedman, B., Potpara, T.S., Lip, G.Y., Stroke prevention in atrial fibrillation (2016) Lancet, 388, pp. 806-817. , https://doi.org/10.1016/S0140-6736(16)31257-0; Freedman, B., Camm, J., Calkins, H., Healey, J.S., Rosenqvist, M., Wang, J., Yan, B.P., A Report of the AF-SCREEN International Collaboration (2017) Circulation, 135, pp. 1851-1867; Friberg, L., Rosenqvist, M., Less dementia with oral anticoagulation in atrial fibrillation (2018) European Heart Journal, 39, pp. 453-460. , https://doi.org/10.1093/eurheartj/ehx579; Friberg, L., Rosenqvist, M., Lindgren, A., Terént, A., Norrving, B., Asplund, K., High prevalence of atrial fibrillation among patients with ischemic stroke (2014) Stroke, 45, pp.

2599-2605. , https://doi.org/10.1161/STROKEAHA.114.006070; Giebel, G.D., Gissel, C., Accuracy of mHealth devices for atrial fibrillation screening: Systematic review (2019) JMIR Mhealth Uhealth, 7. , https://doi.org/10.2196/13641; Gudmundsdottir, K., Fredriksson, T., Svennberg, E., Al-Khalili, F., Friberg, L., Frykman, V., Engdahl, J., Stepwise mass screening for atrial fibrillation using N-terminal B-type natriuretic peptide: the STROKESTOP II study (2020) Europace, 22, pp. 24-32. , https://doi.org/10.1093/europace/euz255; January, C.T., Wann, L.S., Calkins, H., Chen, L.Y., Cigarroa, J.E., Cleveland, J.C., Yancy, C.W., 2019 AHA/ACC/HRS Focused Update of the 2014 AHA/ACC/HRS Guideline for the Management of Patients With Atrial Fibrillation: A Report of the American College of Cardiology/American Heart Association Task Force on Clinical Practice Guidelines and the Heart Rhythm Society in Collaboration With the Society of Thoracic Surgeons (2019) Circulation, 140, pp. e125-e151. , https://doi.org/10.1161/CIR.0000000000000665; Jonas, D.E., Kahwati, L.C., Yun, J.D.Y., Middleton, J.C., Coker-Schwimmer, M., Asher, G.N., Screening for atrial fibrillation with electrocardiography: Evidence report and systematic review for the US preventive services task force (2018) Journal of the American Medical Association, 320, pp. 485-498. , https://doi.org/10.1001/jama.2018.4190; Kirchhof, P., Benussi, S., Kotecha, D., Ahlsson, A., Atar, D., Casadei, B., Vardas, P., 2016 ESC Guidelines for the management of atrial fibrillation developed in collaboration with EACTS: The Task Force for the management of atrial fibrillation of the European Society of Cardiology (ESC) Developed with the special contribution of the European Heart Rhythm Association (EHRA) of the ESC. Endorsed by the European Stroke Organisation (ESO) (2016) European Heart Journal, 37, pp. 2893-2962. , https://doi.org/10.1093/eurheartj/ehw210; Lowres, N., Olivier, J., Chao, T.F., Chen, S.A., Chen, Y., Diederichsen, A., Freedman, B., Estimated stroke risk, yield, and number needed to screen for atrial fibrillation detected through single time screening: a multicountry patient-level meta-analysis of 141,220 screened individuals (2019) PLoS Medicine, 16. , https://doi.org/10.1371/journal.pmed.1002903; Martinez, C., Katholing, A., Freedman, S.B., Adverse prognosis of incidentally detected ambulatory atrial fibrillation. A cohort study (2014) Thrombosis and Haemostasis, 112, pp. 276-286; Orchard, J.J., Neubeck, L., Freedman, B., Webster, R., Patel, A., Gallagher, R., Lowres, N., Atrial fibrillation screen, management and guideline recommended therapy (AF SMART II) in the rural primary care setting: An implementation study protocol (2018) British Medical Journal Open, 8. , https://doi.org/10.1136/bmjopen-2018-023130; O'Sullivan, J.W., Grigg, S., Crawford, W., Turakhia, M.P., Perez, M., Ingelsson, E., Ashley, E.A., Accuracy of smartphone camera applications for detecting atrial fibrillation: A systematic review and meta-analysis (2020) Journal of the American Medical Association Netw Open, 3. , https://doi.org/10.1001/jamanetworkopen.2020.2064; Ramkumar, S., Nerlekar, N., D'Souza, D., Pol, D.J., Kalman, J.M., Marwick, T.H., Atrial fibrillation detection using single lead portable electrocardiographic monitoring: A systematic review and meta-analysis (2018) British Medical Journal Open, 8. , https://doi.org/10.1136/bmjopen-2018-024178; Steinhubl, S.R., Waalen, J., Edwards, A.M., Ariniello, L.M., Mehta, R.R., Ebner, G.S., Topol, E.J., Effect of a home-based wearable continuous ecg monitoring patch on detection of undiagnosed atrial fibrillation: The mSToPS randomized clinical trial (2018) Journal of the American Medical Association, 320, pp. 146-155. , #x2026;, (,).,#x2013; Svennberg, E., Engdahl, J., Al-Khalili, F., Friberg, L., Frykman, V., Rosenqvist, M., Mass screening for untreated atrial fibrillation: The STROKESTOP study (2015) Circulation, 131,

pp. 2176-2184; Tsivgoulis, G., Katsanos, A.H., Köhrmann, M., Caso, V., Perren, F., Palaiodimou, L., Alexandrov, A.V., Duration of implantable cardiac monitoring and detection of atrial fibrillation in ischemic stroke patients: A systematic review and meta-analysis (2019) Journal of Stroke, 21, pp. 302-311. , https://doi.org/10.5853/jos.2019.01067; Wolf, P.A., Abbott, R.D., Kannel, W.B., Atrial fibrillation as an independent risk factor for stroke: The Framingham Study (1991) Stroke, 22, pp. 983-988; Xiong, Q., Proietti, M., Senoo, K., Lip, G.Y., Asymptomatic versus symptomatic atrial fibrillation: A systematic review of age/gender differences and cardiovascular outcomes (2015) International Journal of Cardiology, 191, pp. 172-177. , https://doi.org/10.1016/j.ijcard.2015.05.011; https://www.heartline.comhttps://www.heartline.com/; Chan, N.Y., Choy, C.C., Screening for atrial fibrillation in 13 122 Hong Kong citizens with smartphone electrocardiogram (2017) Heart, 103, pp. 24-31. , https://doi.org/10.1136/heartjnl-2016-309993; Chan, P.H., Wong, C.K., Pun, L., Wong, Y.F., Wong, M.M., Chu, D.W., Siu, C.W., Head-to-head comparison of the alivecor heart monitor and microlife WatchBP office AFIB for atrial fibrillation screening in a primary care setting (2017) Circulation, 135, pp. 110-112. , https://doi.org/10.1161/CIRCULATIONAHA.116.024439; Charitos, E.I., Stierle, U., Ziegler, P.D., Baldewig, M., Robinson, D.R., Sievers, H.H., Hanke, T., A comprehensive evaluation of rhythm monitoring strategies for the detection of atrial fibrillation recurrence: Insights from 647 continuously monitored patients and implications for monitoring after therapeutic interventions (2012) Circulation, 126, pp. 806-814. , https://doi.org/10.1161/CIRCULATIONAHA.112.098079; Chen, L.Y., Chung, M.K., Allen, L.A., Ezekowitz, M., Furie, K.L., McCabe, P., Turakhia, M.P., American Heart Association Council on Clinical Cardiology; Council on Cardiovascular and Stroke Nursing; Council on Quality of Care and Outcomes Research; and Stroke Council. Atrial Fibrillation Burden: Moving Beyond Atrial Fibrillation as a Binary Entity: A Scientific Statement From the American Heart Association (2018) Circulation, 137, pp. e623-e644. , https://doi.org/10.1161/CIR.0000000000000568; Diener, H.C., Sacco, R.L., Easton, J.D., Granger, C.B., Bernstein, R.A., Uchiyama, S., Toyoda, K., RE-SPECT ESUS Steering Committee and Investigators. Dabigatran for Prevention of Stroke after Embolic Stroke of Undetermined Source (2019) New England Journal of Medicine, 380, pp. 1906-1917. , https://doi.org/10.1056/NEJMoa1813959; Engdahl, J., Andersson, L., Mirskaya, M., Rosenqvist, M., Stepwise screening of atrial fibrillation in a 75-year-old population: Implications for stroke prevention (2013) Circulation, 127, pp. 930-937; Furie, K.L., Goldstein, L.B., Albers, G.W., Khatri, P., Neyens, R., Turakhia, M.P., Wood, K.A., American Heart Association Stroke Council; Council on Quality of Care and Outcomes Research; Council on Cardiovascular Nursing; Council on Clinical Cardiology; Council on Peripheral Vascular Disease. Oral antithrombotic agents for the prevention of stroke in nonvalvular atrial fibrillation: a science advisory for healthcare professionals from the American Heart Association/American Stroke Association (2012) Stroke, 43, pp. 3442-3453. , https://doi.org/10.1161/STR.0b013e318266722a; Gudmundsdottir, K., Fredriksson, T., Svennberg, E., Al-Khalili, F., Friberg, L., Frykman, V., Engdahl, J., Stepwise mass screening for atrial fibrillation using N-terminal B-type natriuretic peptide: The STROKESTOP II study (2020) Europace, 22, pp. 24-32. , https://doi.org/10.1093/europace/euz255; Hannon, N., Sheehan, O., Kelly, L., Marnane, M., Merwic, K.A., Moore, A., Kelly, P.J., Stroke associated with atrial fibrillation–incidence and early outcomes in the north Dublin population stroke study (2010) Cerebrovascular Diseases, 29, pp. 43-49. ,

https://doi.org/10.1159/000255973; Hart, R.G., Sharma, M., Mundl, H., Kasner, S.E., Bangdiwala, S.I., Berkowitz, S.D., Connolly, S.J., NAVIGATE ESUS Investigators New England Journal of Medicine, 378, pp. 2191-2201. , https://doi.org/10.1056/NEJMoa1802686; January, C.T., Wann, L.S., Calkins, H., Chen, L.Y., Cigarroa, J.E., Cleveland, J.C., Jr., Yancy, C.W., 2019 AHA/ACC/HRS Focused Update of the 2014 AHA/ACC/HRS Guideline for the Management of Patients With Atrial Fibrillation: A Report of the American College of Cardiology/American Heart Association Task Force on Clinical Practice Guidelines and the Heart Rhythm Society in Collaboration With the Society of Thoracic Surgeons (2019) Circulation, 140. , https://doi.org/10.1161/CIR.0000000000000665; Kamel, H., Longstreth, W.T., Tirschwell, D.L., Kronmal, R.A., Broderick, J.P., Palesch, Y.Y., Elkind, M.S., The AtRial cardiopathy and antithrombotic drugs in prevention after cryptogenic stroke randomized trial: Rationale and methods (2019) International Journal of Stroke, 14, pp. 207-214. , https://doi.org/10.1177/1747493018799981; Kaplan, R.M., Koehler, J., Ziegler, P.D., Sarkar, S., Zweibel, S., Passman, R.S., Stroke risk as a function of atrial fibrillation duration and CHA2DS2-VASc score (2019) Circulation, 140, pp. 1639-1646. , https://doi.org/10.1161/CIRCULATIONAHA.119.041303; Kirchhof, P., Benussi, S., Kotecha, D., Ahlsson, A., Atar, D., Casadei, B., Vardas, P., 2016 ESC Guidelines for the management of atrial fibrillation developed in collaboration with EACTS: The Task Force for the management of atrial fibrillation of the European Society of Cardiology (ESC)Developed with the special contribution of the European Heart Rhythm Association (EHRA) of the ESC Endorsed by the European Stroke Organisation (ESO) (2016) European Heart Journal, 37, pp. 2893-2962. , https://doi.org/10.1093/eurheartj/ehw210; Kishore, A., Vail, A., Majid, A., Dawson, J., Lees, K.R., Tyrrell, P.J., Smith, C.J., Detection of atrial fibrillation after ischemic stroke or transient ischemic attack: A systematic review and meta-analysis (2014) Stroke, 45, pp. 520-526. , https://doi.org/10.1161/STROKEAHA.113.003433; Kolominsky-Rabas, P.L., Weber, M., Gefeller, O., Neundoerfe, R.B., Heuschmann, P.U., Epidemiology of ischemic stroke subtypes according to TOAST criteria: Incidence, recurrence, and long-term survival in ischemic stroke subtypes: A population-based study (2001) Stroke, 32, pp. 2735-2740; Lowres, N., Olivier, J., Chao, T.F., Chen, S.A., Chen, Y., Diederichsen, A., Freedman, B., Estimated stroke risk, yield, and number needed to screen for atrial fibrillation detected through single time screening: A multicountry patient-level meta-analysis of 141,220 screened individuals (2019) PLoS Medicine, 16. , https://doi.org/10.1371/journal.pmed.1002903; Proietti, M., Mairesse, G.H., Goethals, P., Scavee, C., Vijgen, J., Blankoff, I., Lip, G.Y., Belgian Heart Rhythm Week Investigators. A population screening programme for atrial fibrillation: A report from the Belgian Heart Rhythm Week screening programme (2016) Europace, 18, pp. 1779-1786. , https://doi.org/10.1093/europace/euw069; Sanna, T., Diener, H.C., Passman, R.S., Di Lazzaro, V., Bernstein, R.A., Morillo, C.A., Brachmann, J., CRYSTAL AF Investigators. Cryptogenic stroke and underlying atrial fibrillation (2014) New England Journal of Medicine, 370, pp. 2478-2486. , https://doi.org/10.1056/NEJMoa1313600; Schnabel, R.B., Haeusler, K.G., Healey, J.S., Freedman, B., Boriani, G., Brachmann, J., Yan, B., Searching for atrial fibrillation poststroke: A white paper of the Af-SCREEN international collaboration (2019) Circulation, 140, pp. 1834-1850. , https://doi.org/10.1161/CIRCULATIONAHA.119.040267; Sposato, L.A., Cipriano, L.E., Saposnik, G., Ruíz Vargas, E., Riccio, P.M., Hachinski, V., Diagnosis of atrial fibrillation after stroke and transient ischaemic attack: A systematic review and meta-analysis (2015) Lancet Neurology, 14, pp. 377-387. ,

https://doi.org/10.1016/S1474-4422(15)70027-X; Svennberg, E., Engdahl, J., Al-Khalili, F., Friberg, L., Frykman, V., Rosenqvist, M., Mass Screening for Untreated Atrial Fibrillation: The STROKESTOP Study (2015) Circulation, 131, pp. 2176-2184. , CIRCULATIONAHA. 114.014343; Tsivgoulis, G., Katsanos, A.H., Köhrmann, M., Caso, V., Perren, F., Palaiodimou, L., Alexandrov, A.V., Duration of implantable cardiac monitoring and detection of atrial fibrillation in ischemic stroke patients: A systematic review and meta-analysis (2019) Journal of Stroke, 21, pp. 302-311. , https://doi.org/10.5853/jos.2019.01067; Varma, N., Stambler, B., Chun, S., Detection of atrial fibrillation by implanted devices with wireless data transmission capability (2005) Pacing and Clinical Electrophysiology, 28, pp. S133-S136; Yano, Y., Greenland, P., Lloyd-Jones, D.M., Daoud, E.G., Koehler, J.L., Ziegler, P.D., Simulation of daily snapshot rhythm monitoring to identify atrial fibrillation in continuously monitored patients with stroke risk factors (2016) PLoS One, 11. , https://doi.org/10.1371/journal.pone.0148914; Zungsontiporn, N., Link, M.S., Newer technologies for detection of atrial fibrillation (2018) British Medical Journal, 363, p. k3946. , https://doi.org/10.1136/bmj.k3946; https://www.heartline.comhttps://www.heartline.com/; Caceres, B.A., Hickey, K.T., Bakken, S.B., Biviano, A.B., Garan, H., Goldenthal, I.L., Jia, H., Mobile Electrocardiogram Monitoring and Health-Related Quality of Life in Patients With Atrial Fibrillation: Findings From the iPhone Helping Evaluate Atrial Fibrillation Rhythm Through Technology (iHEART) Study (2020) The Journal of Cardiovascular Nursing, 35 (4), pp. 327-336. , https://doi.org/10.1097/JCN.0000000000000646; Hickey, K.T., Hauser, N.R., Valente, L.E., Riga, T.C., Frulla, A.P., Creber, M.R., Wang, D.Y., A single-center randomized, controlled trial investigating the efficacy of a mHealth ECG technology intervention to improve the detection of atrial fibrillation: The iHEART study protocol (2016) BMC Cardiovascular Disorders, 16, p. 152. , https://doi.org/10.1186/s12872-016-0327-y; Heartline study, , https://www.heartline.com/; Al-Turki, A., Marafi, M., Russo, V., Proietti, R., Essebag, V., Subclinical atrial fibrillation and risk of stroke: Past, present and future (2019) Medicina, 55, p. 611. , https://doi.org/10.3390/medicina55100611; Chen, L.Y., Chung, M.K., Allen, L.A., Ezekowitz, M., Furie, K.L., McCabe, P., Turakhia, M.P., American Heart Association Council on Clinical Cardiology; Council on Cardiovascular and Stroke Nursing; Council on Quality of Care and Outcomes Research; and Stroke Council. Atrial fibrillation burden: Moving beyond atrial fibrillation as a binary entity: A Scientific Statement from the American Heart Association (2018) Circulation, 137. , https://doi.org/10.1161/CIR.0000000000000568, e623-; Congrete, S., Bintvihok, M., Thongprayoon, C., Bathini, T., Boonpheng, B., Sharma, K., Cheungpasitporn, W., Effect of obstructive sleep apnea and its treatment of atrial fibrillation recurrence after radiofrequency catheter ablation: A meta-analysis (2018) Journal of Evidence-Based Medicine, 11, pp. 145-151. , https://doi.org/10.1111/jebm.12313; Garabelli, P., Stavrakis, S., Albert, M., Koomson, E., Parwani, P., Chohan, J., Po, S., Comparison of QT interval readings in normal sinus rhythm between a smartphone heart Monitor and a 12-lead ECG for healthy volunteers and inpatients receiving sotalol or dofetilide (2016) Cardiovascular Electrophysiology, 27, pp. 827-832; Glotzer, T.V., Hellkamp, A.S., Zimmerman, J., Sweeney, M.O., Yee, R., Marinchak, R., Lamas, G.A., Investigators, M.O.S.T., Atrial high rate episodes detected by pacemaker diagnostics predict death and stroke: Report of the atrial diagnostics ancillary study of the MOde selection trial (MOST) (2003) Circulation, 107, pp. 1614-1619; Go, A.S., Reynolds, K., Yang, J., Gupta, N., Lenane, J., Sung, S.H., Solomon, M.D., Association of burden of atrial

fibrillation with risk of ischemic stroke in adults with paroxysmal atrial fibrillation: The KP-RHYTHM study (2018) Journal of the American Medical Association Cardiology, 3, pp. 601-608; Healey, J.S., Connolly, S.J., Gold, M.R., Israel, C.W., Van Gelder, I.C., Capucci, A., Hohnloser, S.H., Subclinical atrial fibrillation and the risk of stroke (2012) New England Journal of Medicine, 366, pp. 120-129; January, C.T., Wann, L.S., Calkins, H., Chen, L.Y., Cigarroa, J.E., Cleveland, J.C., Yancy, C.W., 2019 AHA/ACC/HRS Focused Update of the 2014 AHA/ACC/HRS Guideline for the Management of Patients With Atrial Fibrillation: A Report of the American College of Cardiology/American Heart Association Task Force on Clinical Practice Guidelines and the Heart Rhythm Society in Collaboration With the Society of Thoracic Surgeons (2019) Circulation, 140. , https://doi.org/10.1161/CIR.0000000000000665, e125–; Kanagala, R., Murali, N.S., Friedman, P.A., Ammash, N.M., Gersh, B.J., Ballman, K.V., Somers, V.K., Obstructive sleep apnea and the recurrence of atrial fibrillation (2003) Circulation, 107, pp. 2589-2594; Kaplan, R.M., Koehler, J., Ziegler, P.D., Sarkar, S., Zweibel, S., Passman, R.S., Stroke risk as a function of atrial fibrillation duration and CHA2DS2-VASc score (2019) Circulation, 140, pp. 1639-1646. , https://doi.org/10.1161/CIRCULATIONAHA.119.041303; Kirchhof, P., Benussi, S., Kotecha, D., Ahlsson, A., Atar, D., Casadei, B., Vardas, P., 2016 ESC Guidelines for the management of atrial fibrillation developed in collaboration with EACTS: The Task Force for the management of atrial fibrillation of the European Society of Cardiology (ESC)Developed with the special contribution of the European Heart Rhythm Association (EHRA) of the ESC. Endorsed by the European Stroke Organisation (ESO) (2016) European Heart Journal, 37, pp. 2893-2962. , https://doi.org/10.1093/eurheartj/ehw210; Kirchhof, P., Blank, B.F., Calvert, M., Camm, A.J., Chlouverakis, G., Diener, H.C., Vardas, P., Probing oral anticoagulation in patients with atrial high rate episodes: Rationale and design of the Non-vitamin K antagonist Oral anticoagulants in patients with Atrial High rate episodes (NOAH-AFNET 6) trial (2017) American Heart Journal, 190, pp. 12-18; Lopes, R.D., Alings, M., Connolly, S.J., Beresh, H., Granger, C.B., Mazuecos, J.B., Healey, J.S., Rationale and design of the apixaban for the reduction of thrombo-embolism in patients with device-detected sub-clinical atrial fibrillation (ARTESiA) trial (2017) American Heart Journal, 189, pp. 137-145; Martin, D.T., Bersohn, M.M., Waldo, A.L., Wathen, M.S., Choucair, W.K., Lip, G.Y., Halperin, J.L., IMPACT investigators randomized trial of atrial arrhythmia monitoring to guide anticoagulation in patients with implanted defibrillator and cardiac resynchronization devices (2015) European Heart Journal, 36, pp. 1660-1668; Pathak, R.K., Middeldorp, M.E., Meredith, M., Mehta, A.B., Mahajan, R., Wong, C.X., Sanders, P., Long-term effect of goal-directed weight management in an atrial fibrillation cohort: A long-term follow-up study (LEGACY) (2015) Journal of the American College of Cardiology, 65, pp. 2159-2169. , https://doi.org/10.1016/j.jacc.2015.03.002; Perino, A.C., Fan, J., Askari, M., Heidenreich, P.A., Keung, E., Raitt, M.H., Turakhia, M.P., Practice variation in anticoagulation prescription and outcomes after device-detected atrial fibrillation (2019) Circulation, 139, pp. 2502-2512. , https://doi.org/10.1161/CIRCULATIONAHA.118.038988; Swiryn, S., Orlov, M.V., Benditt, D.G., DiMarco, J.P., Lloyd-Jones, D., Karst, E., Waldo, A.L., Clinical implications of brief device-detected atrial tachyarrhythmias in a cardiac rhythm management device population: Results from the registry of atrial tachycardia and atrial fibrillation episodes (2016) Circulation, 134, pp. 1130-1140. , https://doi.org/10.1161/CIRCULATIONAHA.115.020252, #x2026;, (,).,#x2013; Van Gelder, I.C., Healey, J.S., Crijns, H., Wang, J., Hohnloser, S.H., Gold, M.R., Connolly, S.J., Duration of device-detected subclinical

atrial fibrillation and occurrence of stroke in ASSERT (2017) European Heart Journal, 38, pp. 1339-1344; Varma, N., Stambler, B., Chun, S., Detection of atrial fibrillation by implanted devices with wireless data transmission capability (2005) Pacing and Clinical Electrophysiology, 28, pp. S133-S136; Voskoboinik, A., Kalman, J.M., De Silva, A., Nicholls, T., Costello, B., Nanayakkara, S., Kistler, P.M., Alcohol abstinence in drinkers with atrial fibrillation (2020) New England Journal of Medicine, 382, pp. 20-28. , https://doi.org/10.1056/NEJMoa181759; Andelius, L., Hansen, M.C., Lippert, F.K., Karlsson, L., Torp-Pedersen, C., Kjær Ersbøll, A., Folke, F., Smartphone activation of citizen responders to facilitate defibrillation in out-of-hospital cardiac arrest (2020) Journal of the American College of Cardiology, 76, pp. 43-53. , https://doi.org/10.1016/j.jacc.2020.04.073; Au-Yeung, W.M., Reinhall, P.G., Bardy, G.H., Brunton, S.L., Development and validation of warning system of ventricular tachyarrhythmia in patients with heart failure with heart rate variability data (2018) PloS One, 13; Billet, S., Rollin, A., Mondoly, P., Monteil, B., Fournier, P., Cariou, E., Maury, P., Hemodynamic consequences of premature ventricular contractions: Association of mechanical bradycardia and postextrasystolic potentiation with premature ventricular contraction-induced cardiomyopathy (2019) Heart Rhythm, 16, pp. 853-860; Bolle, S.R., Scholl, J., Gilbert, B., Can video mobile phones improve CPR quality when used for dispatcher assistance during simulated cardiac arrest? (2009) Acta Anaesthesiologica Scandinavica, 53, pp. 116-120; Boutilier, J.J., Brooks, S.C., Janmohamed, A., Byers, A., Buick, J.E., Zhan, C., Chan, T.C.Y., Optimizing a drone network to deliver automated external defibrillators (2017) Circulation, 135, pp. 2454-2465; Claesson, A., Bäckman, A., Ringh, M., Svensson, L., Nordberg, P., Djärv, T., Hollenberg, J., Time to delivery of an automated external defibrillator using a drone for simulated out-of-hospital cardiac arrests vs emergency medical services (2017) Journal of the American Medical Association, 317, pp. 2332-2334; Chan, J., Rea, T., Gollakota, S., Sunshine, J.E., Contactless cardiac arrest detection using smart devices (2019) NPJ Digital Medicine, 2, p. 52. , https://doi.org/10.1038/s41746-019-0128-7; Choa, M., Park, I., Chung, H.S., Yoo, S.K., Shim, H., Kim, S., The effectiveness of cardiopulmonary resuscitation instruction: Animation versus dispatcher through a cellular phone (2008) Resuscitation, 77, pp. 87-94; Chong, J.W., Esa, N., McManus, D.D., Chon, K.H., Arrhythmia discrimination using a smart phone (2018) IEEE Journal of Biomedical and Health Informatics, 19, pp. 815-824; Davis, J.C., Robertson, M.C., Ashe, M.C., Liu-Ambrose, T., Khan, K.M., Marra, C.A., International comparison of cost of falls in older adults living in the community: A systematic review (2010) Osteoporosis International, 21, pp. 1295-1306; Hatakeyama, T., Nishiyama, C., Shimamoto, T., Kiyohara, K., Kiguchi, T., Chida, I., Iwami, T., A smartphone application to reduce the time to automated external defibrillator delivery after a witnessed out-of-hospital cardiac arrest: A randomized simulation-based study (2018) Simulation in Healthcare, 13, pp. 387-393; Heinrich, S., Rapp, K., Rissmann, U., Becker, C., König, H.H., Cost of falls in old age: A systematic review (2010) Osteoporosis International, 21, pp. 891-902; Kalz, M., Lenssen, N., Felzen, M., Rossaint, R., Tabuenca, B., Specht, M., Skorning, M., Smartphone apps for cardiopulmonary resuscitation training and real incident support: A mixed-methods evaluation study (2014) Journal of Medical Internet Research, 16; Kwon, J.M., Lee, Y., Lee, Y., Lee, S., Park, J., An algorithm based on deep learning for predicting in-hospital cardiac arrest (2018) Journal of the American Heart Association, 7. , https://doi.org/10.1161/JAHA.118.008678; Lee, H., Shin, S.Y., Seo, M., Nam, G.B., Joo, S., Prediction of ventricular tachycardia one hour before occurrence using artificial neural networks (2016) Scientific Reports, 6, p. 32390;

McManus, D.D., Chong, J.W., Soni, A., Saczynski, J.S., Esa, N., Napolitano, C., Chon, K.H., PULSESMART: Pulse-based arrhythmia discrimination using a novel smartphone application (2016) Journal of Cardiovascular Electrophysiology, 27, pp. 51-57; Merchant, R.M., Abella, B.S., Abotsi, E.J., Smith, T.M., Long, J.A., Trudeau, M.E., Asch, D.A., Cell phone cardiopulmonary resuscitation: Audio instructions when needed by lay rescuers: A randomized, controlled trial (2010) Annals of Emergency Medicine, 55, pp. 538-543; Neves Briard, J., Grou-Boileau, F., El Bashtaly, A., Spenard, C., de Champlain, F., Homier, V., Automated external defibrillator geolocalization with a mobile application, verbal assistance or no assistance: A pilot randomized simulation (AED G-MAP) (2019) Prehospital Emergency Care, 23, pp. 420-429; Paal, P., Pircher, I., Baur, T., Gruber, E., Strasak, A.M., Herff, H., Mitterlechner, T., Mobile phone-assisted basic life support augmented with a metronome (2012) Journal of Emergency Medicine, 43, pp. 472-477; Praveen Kumar, D., Amgoth, T., Annavarapu, C.S.R., Machine learning algorithms for wireless sensor networks: A survey (2019) Information Fusion, 49, pp. 1-25; Reed, M.J., Grubb, N.R., Lang, C.C., O'Brien, R., Simpson, K., Padarenga, M., Coats, T., Multi-centre randomised controlled trial of a smartphone-based event recorder alongside standard care versus standard care for patients presenting to the emergency department with palpitations and pre-syncope: The IPED (Investigation of Palpitations in the ED) study (2019) EClinicalMedicine, 8, pp. 37-46; Rickard, J., Ahmed, S., Baruch, M., Klocman, B., Martin, D.O., Menon, V., Utility of a novel watch-based pulse detection system to detect pulselessness in human subjects (2011) Heart Rhythm, 8, pp. 1895-1899; Ringh, M., Rosenqvist, M., Hollenberg, J., Jonsson, M., Fredman, D., Nordberg, P., Svensson, L., Mobile-phone dispatch of laypersons for CPR in out-of-hospital cardiac arrest (2015) New England Journal of Medicine, 372, pp. 2316-2325; Ringwald, M., Crich, A., Beysard, N., Smart watch recording of ventricular tachycardia: Case study (2019) The American Journal of Emergency Medicine, 38 (4); Sakai, T., Iwami, T., Kitamura, T., Nishiyama, C., Kawamura, T., Kajino, K., Shimazu, T., Effectiveness of the new "Mobile AED Map" to find and retrieve an AED: A andomized controlled trial (2011) Resuscitation, 82, pp. 69-73; Steinberg, J.S., Varma, N., Cygankiewicz, I., Aziz, P., Balsam, P., Baranchuk, A., Piotrowicz, R., 2017 ISHNE-HRS expert consensus statement on ambulatory ECG and external cardiac monitoring/telemetry (2017) Annals of Noninvasive Electrocardiology, 22. , https://doi.org/10.1111/anec.12447; Waks, J.W., Fein, A.S., Das, S., Wide complex tachycardia recorded with a smartphone cardiac rhythm monitor (2015) Journal of the American Medical Association Internal Medicine, 175, pp. 437-439; Weisfeldt, M.L., Sitlani, C.M., Ornato, J.P., Rea, T., Aufderheide, T.P., Davis, D., Morrison, L.J., Survival after application of automatic external defibrillators before arrival of the emergency medical system: Evaluation in the resuscitation out-comes consortium population of 21 million (2010) Journal of the American College of Cardiology, 55, pp. 1713-1720; Wu, O., Briggs, A., Kemp, T., Gray, A., MacIntyre, K., Rowley, J., Willett, K., Mobile phone use for contacting emergency services in life threatening circumstances (2012) Journal of Emergency Medicine, 42, pp. 291-298; You, J.S., Park, S., Chung, S.P., Park, J.W., Performance of cellular phones with video telephony in the use of automated external defibrillators by untrained laypersons (2008) Emergency Medicine Journal, 25, pp. 597-600; Boyne, J.J., Vrijhoef, H.J., Crijns, H.J., De Weerd, G., Kragten, J., Gorgels, A.P., Tailored telemonitoring in patients with heart failure: Results of a multicentre randomized controlled trial (2012) European Journal of Heart Failure, 14, pp. 791-801; Chaudhry, S.I., Mattera, J.A., Curtis, J.P., Spertus, J.A., Herrin, J., Lin, Z., Krumholz, H.M., Telemonitoring in patients with

heart failure (2010) New England Journal of Medicine, 363, pp. 2301-2309; Koehler, F., Winkler, S., Schieber, M., Sechtem, U., Stangl, K., Böhm, M., Anker, S.D., Telemedical Interventional Monitoring in Heart Failure Investigators. Impact of remote telemedical management on mortality and hospitalizations in ambulatory patients with chronic heart failure: The Telemedical Interventional Monitoring in Heart Failure study (2011) Circulation, 123, pp. 1873-1880; Ong, M.K., Romano, P.S., Edgington, S., Aronow, H.U., Auerbach, A.D., Black, J.T., Fonarow, G.C., Better effectiveness after transition-heart failure (BEAT-HF) research group. Effectiveness of remote patient monitoring after discharge of hospitalized patients with heart failure: the better effectiveness after transition—heart failure (BEAT-HF) randomized clinical trial (2016) Journal of the American Medical Association Internal Medicine, 176, pp. 310-318; Takahashi, P.Y., Pecina, J.L., Upatising, B., Chaudhry, R., Shah, N.D., Van Houten, H., Hanson, G.J., A randomized controlled trial of telemonitoring in older adults with multiple health issues to prevent hospitalizations and emergency department visits (2012) Archives of Internal Medicine, 172, pp. 773-779; Stehlik, J., Schmalfuss, C., Bozkurt, B., Nativi-Nicolau, J., Wohlfahrt, P., Wegerich, S., Pham, M., Continuous wearable monitoring analytics predict heart failure hospitalization the LINK-HF multicenter study (2020) Circulation Heart Failure, 13. , https://doi.org/10.1161/CIRCHEARTFAILURE.119.006513; Chung, M.K., Eckhardt, L.L., Chen, L.Y., Ahmed, H.M., Gopinathannaie, R., Joglar, J.A., Trulock, K.M., Lifestyle and risk factor modification for reduction of atrial fibrillation: A scientific statement from the American Heart Association (2020) Circulation, 141. , https://doi.org/10.1161/CIR.0000000000000748; January, C.T., Wann, L.S., Calkins, H., Chen, L.Y., Cogarroa, J.E., Cleveland, J.C., Yancy, C.W., 2019 AHA/ACC/HRS Focused Update of the 2014 AHA/ACC/HRS Guideline for the Management of Patients With Atrial Fibrillation: A Report of the American College of Cardiology/American Heart Association Task Force on Clinical Practice Guidelines and the Heart Rhythm Society in Collaboration With the Society of Thoracic Surgeons (2019) Circulation, 140. , https://doi.org/10.1161/CIR.0000000000000665; Avila, C.O., Novel use of apple watch 4 to obtain 3-lead electrocardiogram and detect cardiac ischemia (2019) The Permanente Journal, 23, pp. 19-025; Chow, C.K., Redfern, J., Hillis, G.S., Thakkar, J., Santo, K., Hackett, M.L., Thiagalingam, A., Effect of lifestyle-focused text messaging on risk factor modification in patients with coronary heart disease: A randomized clinical trial (2015) Journal of the American Medical Association, 314, pp. 1255-1263; Clemmensen, P., Loumann-Nielsen, S., Sejersten, M., Telemedicine fighting acute coronary syndromes (2010) Journal of Electrocardiology, 43, pp. 615-618; Gibson, C.M., Holmes, D., Mikdad, I.G., Presser, D., Wohns, D., Yee, M.K., Krucoff, M.W., Implantable cardiac alert system for early recognition of ST-segment elevation myocardial infarction (2019) Journal of the American College of Cardiology, 73, pp. 1919-1927; Holmes, D.R., Jr., Krucoff, M.W., Mullin, C., Mikdadi, G., Presser, D., Wohns, D., Gibson, C.M., Implanted monitor alerting to reduce treatment delay in patients with acute coronary syndrome events (2019) Journal of the American College of Cardiology, 74, pp. 2047-2055; Horwitz, L.I., Moriarty, J.P., Chen, C., Fogerty, R.L., Brewster, U.C., Kanade, S., Krumholz, H.M., Quality of discharge practices and patient understanding at an academic medical center (2013) Journal of the American Medical Association Internal Medicine, 173, pp. 1715-1722; Moser, D.K., Kimble, L.P., Alberts, M.J., Alonzo, A., Croft, J.B., Dracup, K., Zerwic, J.J., American Heart Association Council on Cardiovascular Nursing and Stroke Council. Reducing delay in seeking treatment by patients with acute coronary syndrome and stroke: a scientific

statement from the American Heart Association Council on Cardiovascular Nursing and Stroke Council (2006) Circulation, 114, pp. 168-182; Park, L.G., Beatty, A., Stafford, Z., Whooley, M.A., Mobile phone interventions for the secondary prevention of cardiovascular disease (2016) Progress in Cardiovascular Diseases, 58, pp. 639-650; Pizzetti, F., Turazza, F.M., Franzosi, M.G., Barlera, S., Ledda, A., Maggioni, A.P., Tognoni, G., Incidence and prognostic significance of atrial fibrillation in acute myocardial infarction: the GISSI-3 data (2001) Heart, 86, pp. 527-532; Ritchey, M.D., Maresh, S., McNeely, J., Shaffer, T., Jackson, S.L., Keteyian, S.J., Wright, J., Tracking cardiac rehabilitation participation and completion among medicare beneficiaries to inform the efforts of a national initiative (2020) Circulation: Cardiovascular Quality and Outcome, 13; Sanchez-Ross, M., Oghlakian, G., Maher, J., Patel, B., Mazza, V., Hom, D., Klapholz, M., The STAT-MI (ST-Segment analysis using wireless technology in acute myocardial Infarction) trial improves outcomes (2011) Journal of the American College of Cardiology, 4, pp. 222-227; Shariful Islam, S.M., Farmer, A.J., Bobrow, K., Maddison, R., Whittaker, R., Pfaeffli Dale, L.A., Chow, C.K., Mobile phone text-messaging interventions aimed to prevent cardiovascular diseases (Text2PreventCVD): Systematic review and individual patient data meta-analysis (2019) Open Heart, 6; Unal, E., Giakoumidakis, K., Khan, E., Patelarou, E., Mobile phone text messaging for improving secondary prevention in cardiovascular diseases: A systematic review (2018) Heart Lung, 47, pp. 351-359; Van Heuverswyn, F., De Buyzere, M., Coeman, M., de Pooter, J., Drieghe, B., Duyschaever, M., Gheeraert, P., Feasibility and performance of a device for automatic self-detection of symptomatic acute coronary artery occlusion in outpatients with coronary artery disease: A multicentre observational study (2019) Lancet Digital Health, 1; Varnfield, M., Karunanithi, M., Lee, C.K., Honeyman, E., Arnold, D., Ding, H., Walters, D.L., Smartphone-based home care model improved use of cardiac rehabilitation in postmyocardial infarction patients: Results from a randomised controlled trial (2014) Heart, 100, pp. 1770-1779; Vollmer, W.M., Owen-Smith, A.A., Tom, J.O., Laws, R., Ditmer, D.G., Smith, D.H., Williams, A., Improving adherence to cardiovascular disease medications with information technology (2014) The American Journal of Managed Care, 20 (SP17), pp. SP502-SP510; Ziaeian, B., Araujo, K.L., Van Ness, P.H., Horwitz, L.I., Medication reconciliation accuracy and patient understanding of intended medication changes on hospital discharge (2012) Journal of General Internal Medicine, 27, pp. 1513-1520; Zwisler, A.D., Norton, R.J., Dean, S.G., Dalal, H., Tang, L.H., Wingham, J., Taylor, R.S., Home-based cardiac rehabilitation for people with heart failure: A systematic review and meta-analysis (2016) International Journal of Cardiology, 221, pp. 963-969; Abraham, W.T., Stevenson, L.W., Bourge, R.C., Lindenfeld, J.A., Bauman, J.G., Adamson, P.B., Sustained efficacy of pulmonary artery pressure to guide adjustment of chronic heart failure therapy: complete follow-up results from the CHAMPION randomized trial (2016) Lancet, 387, pp. 453-461; Albert, C., Estep, J.D., Economic impact of chronic heart failure management in today’s cost-conscious environment (2019) Cardiac Electrophysiology Clinics, 11, pp. 1-9; Benjamin, E.J., Blaha, M.J., Chiuve, S.E., Cushman, M., Das, S.R., Deo, R., Muntner, P., Heart disease and stroke statistics-2017 update: A report from the American Heart Association (2017) Circulation, 135; Boyne, J.J., Vrijhoef, H.J., Crijns, H.J., De Weerd, G., Kragten, J., Gorgels, A.P., Tailored telemonitoring in patients with heart failure: Results of a multicentre randomized controlled trial (2012) European Journal of Heart Failure, 14, pp. 791-801; Carbo, A., Gupta, M., Tamariz, L., Palacio, A., Levis, S., Nemeth, Z., Dang, S., Mobile technologies for managing heart failure: a systematic review and meta-analysis (2018) Telemedicine and e-Health, 24 (12), pp. 958-968. ,

https://doi.org/10.1089/tmj.2017.0269, Online ahead of print; Chaudhry, S.I., Mattera, J.A., Curtis, J.P., Spertus, J.A., Herrin, J., Lin, Z., Krumholz, H.M., Telemonitoring in patients with heart failure (2010) New England Journal of Medicine, 363, pp. 2301-2309; Desai, A.S., Bhimaraj, A., Bharmi, R., Jermyn, R., Bhatt, K., Shavelle, D., Heywood, J.T., Ambulatory hemodynamic monitoring reduces heart failure hospitalizations in “real-world” clinical practice (2017) Journal of the American College of Cardiology, 69, pp. 2357-2365; Dickinson, M., Allen, L., Albert, N.A., DiSalvo, T., Ewald, G.A., Vest, A.R., Givertz, M.M., Consensus Statement. Remote monitoring of patients with heart failure: A white paper from the Heart Failure Society of America Scientific Statements Committee (2018) Journal of Cardiac Failure, 24, pp. 682-694. , https://doi.org/10.1016/j.cardfail.2018.08.011; Koehler, F., Koehler, K., Deckwart, O., Prescher, S., Wegscheider, K., Kirwan, B.A., Stangl, K., Efficacy of telemedical interventional management in patients with heart failure (TIM-HF2): A randomised, controlled, parallel-group, unmasked trial (2018) Lancet, 392, pp. 1047-1057. , https://doi.org/10.1016/S0140-6736(18)31880-4; Koehler, F., Winkler, S., Schieber, M., Sechtem, U., Stangl, K., Böhm, M., Anker, S.D., Telemedical Interventional Monitoring in Heart Failure Investigators. Impact of remote telemedical management on mortality and hospitalizations in ambulatory patients with chronic heart failure: The Telemedical Interventional Monitoring in Heart Failure study (2011) Circulation, 123, pp. 1873-1880; Ong, M.K., Romano, P.S., Edgington, S., Aronow, H.U., Auerbach, A.D., Black, J.T., Fonarow, G.C., Better effectiveness after transition-heart failure (BEAT-HF) research group. Effectiveness of remote patient monitoring after discharge of hospitalized patients with heart failure: the better effectiveness after transition–heart failure (BEAT-HF) randomized clinical trial (2016) Journal of the American Medical Association Internal Medicine, 176, pp. 310-318; Ono, M., Varma, N., Remote monitoring to Improve long-term prognosis in heart failure patients with implantable cardioverter-defibrillators (2017) Expert Review of Medical Devices, 14, pp. 335-342; Takahashi, P.Y., Pecina, J.L., Upatising, B., Chaudhry, R., Shah, N.D., Van Houten, H., Hanson, G.J., A randomized controlled trial of telemonitoring in older adults with multiple health issues to prevent hospitalizations and emergency department visits (2012) Archives of Internal Medicine, 172, pp. 773-779; Carbo, A., Gupta, M., Tamariz, L., Palacio, A., Levis, S., Nemeth, Z., Dang, S., Mobile technologies for managing heart failure: A systematic review and meta-analysis (2018) Telemedicine and e-Health, 24, pp. 958-968. , https://doi.org/10.1089/tmj.2017.0269; Chaudhry, S.I., Mattera, J.A., Curtis, J.P., Spertus, J.A., Herrin, J., Lin, Z., Krumholz, H.M., Telemonitoring in patients with heart failure (2010) New England Journal of Medicine, 363, pp. 2301-2309; Cipresso, P., Serino, S., Villan, I.D., Repetto, C., Sellitti, L., Albani, A., Rivaet, G., Is your phone so smart to affect your states? An exploratory study based on psychophysiological measures (2012) Neurocomputing, 84, pp. 23-30; Hamilton, S.J., Mills, B., Birch, E.M., Thompson, S.C., Smartphones in the secondary prevention of cardiovascular disease: A systematic review (2018) BMC Cardiovascular Disorders, 18, p. 25; Molinaro, N., Massaroni, C., Lo Presti, D., Saccomandi, P., Di Tomaso, G., Zollo, L., Schena, E., Wearable textile based on silver plated knitted sensor for respiratory rate monitoring (2018) Conference Proceedings IEEE Engineering in Medicine and Biology Society (EMBC), 2865-2868. , https://doi.org/10.1109/EMBC.2018.8512958; Stehlik, J., Schmalfuss, C., Bozkurt, B., Nativi-Nicolau, J., Wohlfahrt, P., Wegerich, S., Pham, M., Continuous wearable monitoring analytics predict heart failure hospitalization the LINK-HF multicenter study (2020) Circulation Heart Failure,

13. , https://doi.org/10.1161/CIRCHEARTFAILURE.119.006513; Ades, P.A., Pashkow, F.J., Fletcher, G., Pina, I.L., Zohman, L.R., Nestor, J.R., A controlled trial of cardiac rehabilitation in the home setting using electrocardiographic and voice transtelephonic monitoring (2000) American Heart Journal, 139, pp. 543-548; Kouidi, E., Farmakiotis, A., Kouidis, N., Deligiannis, A., Transtelephonic electrocardiographic monitoring of an outpatient cardiac rehabilitation programme (2006) Clinical Rehabilitation, 20, pp. 1100-1104; Piepoli, M.F., Conraads, V., Corrà, U., Agoston, I.P., Coats, A.J., Conraads, V., Ponikowski, P.P., Exercise training in heart failure: from theory to practice. A consensus document of the Heart Failure Association and the European Association for Cardiovascular Prevention and Rehabilitation (2011) European Journal of Heart Failure, 13, pp. 347-357; Piotrowicz, E., Buchner, T., Piotrowski, W., Piotrowicz, R., Influence of Home-based telemonitored Nordic walking training on autonomic nervous system balance in heart failure patients (2015) Archives of Medical Science, 11, pp. 1205-1212; Piotrowicz, E., Pencina, M.J., Opolski, G., Zareba, W., Banach, M., Kowalik, I., Piotrowicz, R., Effects of a 9-Week Hybrid Comprehensive Telerehabilitation Program on Long-term Outcomes in Patients With Heart Failure. The Telerehabilitation in Heart Failure Patients (TELEREH-HF) Randomized Clinical Trial (2019) Journal of the American Medical Association Cardiology, 5 (3), pp. 300-308. , https://doi.org/10.1001/jamacardio.2019.5006; Piotrowicz, E., Piepoli, M.F., Jaarsma, T., Lambrinou, E., Coats, A.J., Schmid, J.P., Ponikowski, P.P., Telerehabilitation in heart failure patients: The evidence and the pitfalls (2016) International Journal of Cardiology, 220, pp. 408-413; Piotrowicz, E., Piotrowicz, R., Opolski, G., Pencina, M., Banach, M., Zaręba, W., Hybrid comprehensive telerehabilitation in heart failure patients (TELEREH-HF): A randomized, multicenter, prospective, open-label, parallel group controlled trial-Study design and description of the intervention (2019) American Heart Journal, 217, pp. 148-158; Piotrowicz, E., Piotrowski, W., Piotrowicz, R., Positive effects of the reversion of depression on the sympathovagal balance after telerehabilitation in heart failure patients (2016) Annals of Noninvasive Electrocardiology, 21, pp. 358-368; Piotrowicz, E., Stepnowska, M., Leszczyńska-Iwanicka, K., Piotrowska, D., Kowalska, M., Tylka, J., Piotrowicz, R., Quality of life in heart failure patients undergoing home-based telerehabilitation versus outpatient rehabilitation - a randomized controlled study (2015) European Journal of Cardiovascular Nursing, 14, pp. 256-263; Piotrowicz, E., Zieliński, T., Bodalski, R., Rywik, T., Dobraszkiewicz-Wasilewska, B., Sobieszczańska-Małek, M., Piotrowicz, R., Home-based telemonitored Nordic walking training is well accepted, safe, effective and has high adherence among heart failure patients, including those with cardiovascular implantable electronic devices – a randomized controlled study (2015) European Journal of Preventive Cardiology, 22, pp. 1368-1377; Ponikowski, P., Voors, A.A., Anker, S.D., Bueno, H., Cleland, J.G.F., Coats, A.J.S., van der Meer, P., ESC Scientific Document Group. 2016 ESC Guidelines for the diagnosis and treatment of acute and chronic heart failure: The Task Force for the diagnosis and treatment of acute and chronic heart failure of the European Society of Cardiology (ESC)Developed with the special contribution of the Heart Failure Association (HFA) of the ESC (2016) European Heart Journal, 37, pp. 2129-2200; Smart, N., Haluska, B., Jeffriess, L., Marwick, T.H., Predictors of a sustained response to exercise training in patients with chronic heart failure: A telemonitoring study (2005) American Heart Journal, 150, pp. 1240-1247; Thomas, R.J., Beatty, A.L., Beckie, T.M., Brewer, L.C., Brown, T.M., Forman, D.E., Whooley, M.A., Home-based cardiac rehabilitation. A scientific statement from the American Association of Cardiovascular and

Pulmonary Rehabilitation the American Heart Association, and the American College of Cardiology (2019) Journal of the American College of Cardiology, 74, pp. 133-153; Agarwal, P., Mukerji, G., Desveaux, L., Ivers, N.M., Bhattacharyya, O., Hensel, J.M., Bhatia, R.S., Mobile app for improved self-management of type 2 diabetes: Multicenter pragmatic randomized controlled trial (2019) JMIR Mhealth and Uhealth, 7; Andrès, E., Meyer, L., Zulfiqar, A.A., Hajjam, M., Talha, S., Bahougne, T., Hajjam El Hassani, A., Telemonitoring in diabetes: Evolution of concepts and technologies, with a focus on results of the more recent studies (2019) Journal of Medicine and Life, 12, pp. 203-214; Balakumar, P., Maung-U, K., Jagadeesh, G., Prevalence and prevention of cardiovascular disease and diabetes mellitus (2016) Pharmacological Research, 113 (Pt A), pp. 600-609; Chang, S.H., Wu, L.S., Chiou, M.J., Liu, J.R., Yu, K.H., Kuo, C.F., See, L.C., Association of metformin with lower atrial fibrillation risk among patients with type 2 diabetes mellitus: A population-based dynamic cohort and in vitro studies (2014) Cardiovascular Diabetology, 13, p. 123. , https://doi.org/10.1186/s12933-014-0123-x; Chao, T.F., Leu, H.B., Huang, C.C., Chen, J.W., Chan, W.L., Lin, S.J., Chen, S.A., Thiazolidinediones can prevent new onset atrial fibrillation in patients with non-insulin dependent diabetes (2012) International Journal of Cardiology, 156, pp. 199-202. , https://doi.org/10.1016/j.ijcard.2011.08.081; Cosentino, F., Grant, P.J., Aboyans, V., Bailey, C.J., Ceriello, A., Wheeler, D.C., ESC Scientific Document Group. 2019 ESC Guidelines on diabetes, pre-diabetes, and cardiovascular diseases developed in collaboration with the EASD (2020) European Heart Journal, 41, pp. 255-323. , https://doi.org/10.1093/eurheartj/ehz486; Donnelan, E., Aagaard, P., Kanj, M., Jabe, R.W., Elshazly, M., Hoosien, M., Wazni, O., Association between pre-ablation glycemic control and outcomes among patients with diabetes undergoing atrial fibrillation ablation (2019) Journal of the American Medical Association Clinical Electrophysiology, 5, pp. 397-903; Fleming, G.A., Petrie, J.R., Bergenstal, R.M., Holl, R.W., Peters, A.L., Heinemann, L., Diabetes digital app technology: Benefits, challenges, and recommendations. A Consensus Report by the European Association for the Study of Diabetes (EASD) and the American Diabetes Association (ADA) Diabetes Technology Working Group (2020) Diabetes Care, 43, pp. 250-260. , https://doi.org/10.2337/dci19-0062; Fokkert, M.J., van Dijk, P.R., Edens, M.A., Abbes, S., de Jong, D., Slingerland, R.J., Bilo, H.J.G., Performance of the FreeStyle Libre Flash glucose monitoring system in patients with type 1 and 2 diabetes mellitus (2017) BMJ Open Diabetes Research & Care, 5 (1). , https://doi.org/10.1136/bmjdrc-2016-000320; Fokkert, M., van Dijk, P., Edens, M., Barents, E., Mollema, J., Slingerland, R., Bilo, H., Improved well-being and decreased disease burden after 1-year use of flash glucose monitoring (FLARE-NL4) (2019) BMJ Open Diabetes Research and Care, 7. , https://doi.org/10.1136/bmjdrc-2019-0008093; Garabedian, L.F., Ross-Degnan, D., Wharam, J.F., Mobile phone and smartphone technologies for diabetes care and self-management (2015) Current Diabetes Reports, 15, p. 109; Goudis, C.A., Korantzopoulos, P., Ntalas, I.V., Kallergis, E.M., Liu, T., Ketikoglou, D.G., Diabetes mellitus and atrial fibrillation: Pathophysiological mechanisms and potential upstream therapies (2015) International Journal of Cardiology, 184, pp. 617-622; Gu, J., Liu, X., Wang, X., Shi, H., Tan, H., Zhou, L., Wang, Y., Beneficial effect of pioglitazone on the outcome of catheter ablation in patients with paroxysmal atrial fibrillation and type 2 diabetes mellitus (2011) Europace, 13 (9), pp. 1256-1261. , https://doi.org/10.1093/europace/eur131; Liang, X., Wang, Q., Yang, X., Cao, J., Chen, J., Mo, X., Gu, D., Effect of mobile phone intervention for diabetes

on glycaemic control: A meta-analysis (2011) Diabetic Medicine, 28, pp. 455-463; Otake, H., Suzuki, H., Honda, T., Maruyama, Y., Influences of autonomic nervous system on atrial arrhythmogenic substrates and the incidence of atrial fibrillation in diabetic heart (2009) International Heart Journal, 50 (5), pp. 627-641. , https://doi.org/10.1536/ihj.50.627; Pal, K., Eastwood, S.V., Michie, S., Farmer, A., Barnard, M.L., Peacock, R., Murray, E., Computer-based interventions to improve self-management in adults with type 2 diabetes: A systematic review and meta-analysis (2014) Diabetes Care, 37, pp. 1759-1766; Pillay, J., Armstrong, M.J., Butalia, S., Donovan, L.E., Sigal, R.J., Chordiya, P., Dryden, D.M., Behavioral programs for type 1 diabetes mellitus: A systematic review and meta-analysis (2015) Annals of Internal Medicine, 163, pp. 836-847; Quinn, C., Shardell, M., Terrin, M., Barr, E.A., Ballew, S.H., Gruber-Baldini, A.L., Cluster-randomized trial of a mobile phone personalized behavioral intervention for blood glucose control (2011) Diabetes Care, 34, pp. 1934-1942; Saffari, M., Ghanizadeh, G., Koenig, H.G., Health education via mobile text messaging for glycemic control in adults with type 2 diabetes: A systematic review and meta-analysis (2014) Primary Care Diabetes, 8, pp. 275-285; Veazie, S., Winchell, K., Gilbert, J., Paynter, R., Ivlev, I., Eden, K.B., Helfand, M., Rapid evidence review of mobile applications for self-management of diabetes (2018) Journal of General Internal Medicine, 33, pp. 1167-1176; Wang, A., Green, J.B., Halperin, J.L., Piccini, J.P., Sr., Atrial fibrillation and diabetes mellitus (2019) Journal of the American College of Cardiology, 74, pp. 1107-1115; Whaley, C.M., Bollyky, J.B., Lu, W., Painter, S., Schneider, J., Zhao, Z., Meadows, E.S., Reduced medical spending associated with increased use of a remote diabetes management program and lower mean blood glucose values (2019) Journal of Medical Economics, 22, pp. 869-877; Wilkinson, M.J., Zadourian, A., Taub, P.R., Heart failure and diabetes mellitus: Defining the problem and exploring the interrelationship (2019) American Journal of Cardiology, 124, pp. S3-S11; Wingerter, R., Steiger, N., Burrows, A., Estes, N.A.M., Impact of lifestyle modification on atrial fibrillation (2020) American Journal of Cardiology, 125, pp. 289-297. , https://doi.org/10.1016/j.amjcard.2019.10.018; Bosworth, H.B., Powers, B.J., Olsen, M.K., McCant, F., Grubber, J., Smith, V., Oddone, E.Z., Home blood pressure management and improved blood pressure control: results from a randomized controlled trial (2011) Archives of Internal Medicine, 171, pp. 1173-1180; Duan, Y., Xie, Z., Dong, F., Wu, Z., Lin, Z., Sun, N., Xu, J., Effectiveness of home blood pressure telemonitoring: a systematic review and meta-analysis of randomised controlled studies (2017) Journal of Human Hypertension, 31, pp. 427-437; Huxley, R.R., Lopez, F.L., Folsom, A.R., Agarwal, S.K., Loehr, L.R., Soliman, E.Z., Alonso, A., Absolute and attributable risks of atrial fibrillation in relation to optimal and borderline risk factors: the Atherosclerosis Risk in Communities (ARIC) study (2011) Circulation, 123, pp. 1501-1508. , https://doi.org/10.1161/CIRCULATIONAHA.110.009035; Kim, Y.-N., Shin, D.G., Park, S., Lee, C.H., Randomized clinical trial to assess the effectiveness of remote patient monitoring and physician care in reducing office blood pressure (2015) Hypertension Research, 38, pp. 491-497; McManus, R.J., Mant, J., Bray, E.P., Holder, R., Jones, M.I., Greenfield, S., Hobbs, F.D., Telemonitoring and self-management in the control of hypertension (TASMINH2): A randomised controlled trial (2010) Lancet, 376, pp. 163-172; McManus, R.J., Mant, J., Franssen, M., Nickless, A., Schwartz, C., Hodgkinson, J., Hobbs, F.D.R., Efficacy of self-monitored blood pressure, with or without telemonitoring, for titration of antihypertensive medication (TASMINH4): An unmasked randomised controlled trial (2018) Lancet, 391, pp. 949-959; Monahan, M., Jowett, S.,

Nickless, A., Franssen, M., Grant, S., Greenfield, S., McManus, R.J., Cost-effectiveness of telemonitoring and self-monitoring of blood pressure for antihypertensive titration in primary care (TASMINH4) (2019) Hypertension, 73, pp. 1231-1239; Tucker, K.L., Sheppard, J.P., Stevens, R., Bosworth, H.B., Bove, A., Bray, E.P., McManus, R.J., Self-monitoring of blood pressure in hypertension: A systematic review and individual patient data meta-analysis (2017) PLoS Medicine, 14; Williams, B., Mancia, G., Spiering, W., Agabiti Rosei, E., Azizi, M., Burnier, M., Desormais, I., ESC Scientific Document Group. 2018 ESC/ESH Guidelines for the management of arterial hypertension (2018) European Heart Journal, 39, pp. 3021-3104; Berryhill, S., Morton, C.J., Dean, A., Berryhill, A., Provencio-Dean, N., Patel, S.I., Parthasarathy, S., Effect of wearables on sleep in healthy individuals: A randomized crossover trial and validation study (2020) Journal of Clinical Sleep Medicine, 16, pp. 775-783; Burgess, H.J., Trinder, J., Kim, Y., Luke, D., Sleep and circadian influences on cardiac autonomic nervous system activity (1997) The American Journal of Physiology, 273, pp. H1761-H1768; Daghlas, I., Dashti, H.S., Lane, L., Aragam, J.L., Rutter, M.K., Saxena, R., Vetter, C., Sleep duration and myocardial infarction (2019) Journal of the American College of Cardiology, 74, pp. 1304-1314; Hirshkowitz, M., Whiton, K., Albert, S.M., Alessi, C., Bruni, O., DonCarlos, L., Ware, J.C., National Sleep Foundation’s updated sleep duration recommendations: Final report (2015) Sleep Health, 1, pp. 233-243; Huang, T., Mariani, S., Redline, S., Sleep irregularity and risk of cardiovascular events: The multi-ethnic study of atherosclerosis (2020) Journal of the American College of Cardiology, 75, pp. 991-999. , https://doi.org/10.1016/j.jacc.2019.12.054; Khosla, S., Deak, M.C., Gault, D., Goldstein, C.A., Hwang, D., Kwon, Y., Rowley, J.A., American academy of sleep medicine board of directors. Consumer sleep technology: An American Academy of Sleep Medicine Position Statement (2018) Journal of Clinical Sleep Medicine, 14, pp. 877-880; Mantua, J., Gravel, N., Spencer, R.M., Reliability of sleep measures from four personal health monitoring devices compared to research-based actigraphy and polysomnography (2016) Sensors, 16, p. 646. , https://doi.org/10.3390/s16050646; May, A.M., Blackwell, T., Stone, P.H., Stone, K.L., Cawthon, P.M., Sauer, W.H., Mehra, R., Central sleep-disordered breathing predicts incident atrial fibrillation in older men (2016) American Journal of Respiratory Critical Care Medicine, 193, pp. 783-791; May, A.M., Van Wagoner, D.R., Mehra, R., OSA and cardiac arrhythmogenesis: Mechanistic insights (2017) Chest, 151, pp. 225-241; Mehra, R., Benjamin, E.J., Shahar, E., Gottlieb, D.J., Nawabit, R., Kirchner, H.L., Redline, S., Sleep Heart Health Study. Association of nocturnal arrhythmias with sleep-disordered breathing: The Sleep Heart Health Study (2006) American Journal of Respiratory Critical Care Medicine, 173, pp. 910-916; Qureshi, W.T., Nasir, U.B., Alqalyoobi, S., O’Neal, W.T., Mawri, S., Sabbagh, S., Al-Mallah, M.H., Meta-analysis of continuous positive airway pressure as a therapy of atrial fibrillation in obstructive sleep apnea (2015) American Journal of Cardiology, 116 (11), pp. 1767-1773. , https://doi.org/10.1016/j.amjcard.2015.08.04; Selvaraj, N., Narasimhan, R., Automated prediction of the apnea-hypopnea index using a wireless patch sensor (2014) Conference Proceedings IEEE Eng Med Biol Soc, 1897-900. , https://doi.org/10.1109/EMBC.2014.6943981; Youssef, I., Kamran, H., Yacoub, M., Patel, N., Goulbourne, C., Kumar, S., Kane, J., McFarlane, S.I., Obstructive sleep apnea as a risk factor for atrial fibrillation: A meta-analysis (2018) Journal of Sleep Disorders & Therapy, 7, p. 282. , https://doi.org/10.4172/2167-0277.1000282; (2006), http://www.ncbi.nlm.nih.gov/books/NBK19961/, . 3, Extent and Health Consequences of Chronic Sleep Loss and Sleep Disorders. Available from;

Abdulla, J., Nielsen, J.R., Is the risk of atrial fibrillation higher in athletes thanin the general population? A systematic review and meta-analysis (2009) Europace, 11, pp. 1156-1159. , https://doi.org/10.1093/europace/eup197; Andersen, K., Farahmand, B., Ahlbom, A., Held, C., Ljunghall, S., Michaelsson, K., Sundstrom, J., Risk of arrhythmias in 52 755 long-distance crosscountry skiers: A cohort study (2013) European Heart Journal, 34, pp. 3624-3631. , https://doi.org/10.1093/eurheartj/eht188; Aroganam, G., Manivannan, N., Harrison, D., Review on wearable technology sensors used in consumer sport applications (2019) Sensors, 19, p. 1983. , https://doi.org/10.3390/s19091983; Brunetti, N.D., Dellegrottaglie, G., Di Giuseppe, G., Lopriore, C., Loiacono, T., Gardini, G., Patruno, S., Di Biase, M., Young football italian amateur players remote electrocardiogram screening with telemedicine (you first) study: Preliminary results (2014) International Journal of Cardiology, 176, pp. 1257-1258; Coppetti, T., Brauchlin, A., Müggler, S., Attinger-Toller, A., Templin, C., Schönrath, F., Wyss, C.A., Accuracy of smartphone apps for heart rate measurement (2017) European Journal of Preventive Cardiology, 24, pp. 1287-1293; Dobbs, W.C., Fedewa, M.V., MacDonald, H.V., Holmes, C.J., Cicone, Z.S., Plews, D.J., Esco, M.R., The accuracy of acquiring heart rate variability from portable devices: A systematic review and meta-analysis (2019) Sports Medicine, 2019 (49), pp. 417-435. , https://doi.org/10.1007/s40279-019-01061-5; Everett, B.M., Conen, D., Buring, J.E., Moorthy, M.V., Lee, I.M., Albert, C.M., Physical activity and the risk of incident atrial fibrillation in women (2011) Circulation Cardiovascular Quality Outcomes, 4, pp. 321-327; Faselis, C., Kokkinos, P., Tsimploulis, A., Pittaras, A., Myers, J., Lavie, C.J., Moore, H., Exercise capacity and atrial fibrillation risk in veterans: A cohort study (2016) Mayo Clinical Proceedings, 91 (5), pp. 558-566. , https://doi.org/10.1016/j.mayocp.2016.03.002; Jakicic, J.M., Davis, K.K., Rogers, R.J., King, W.C., Marcus, M.D., Helsel, D., Belle, S.H., Effect of wearable technology combined with a lifestyle intervention on long-term weight loss: The IDEA randomized clinical trial (2016) Journal of the American Medical Association, 316, pp. 1161-1171; Kramer, D.B., Mitchell, S.L., Monteiro, J., Jones, P.W., Sharon-Lise Normand, S.-L., Hayes, D.L., Reynolds, M.R., Patient activity and survival following implantable cardioverter-defibrillator implantation: The ALTITUDE activity study (2015) Journal of the American Heart Association, 4. , https://doi.org/10.1161/JAHA.115.001775; Li, R.T., Kling, S.R., Salata, M.J., Cupp, S.A., Sheehan, J., Voos, J.E., Wearable performance devices in sports medicine (2016) Sports Health, 8, pp. 74-78; McConnell, M.V., Turakhia, M.P., Harrington, R.A., King, A.C., Ashley, E.A., Mobile health advances in physical activity, fitness, and atrial fibrillation: Moving hearts (2018) Journal of the American College of Cardiology, 71, pp. 2691-2701; Mozaffarian, D., Furberg, C.D., Psaty, B.M., Siscovick, D., Physical activity and incidence of atrial fibrillation in older adults - The cardiovascular health study (2008) Circulation, 118, pp. 800-807; Orchard, J.J., Neubeck, L., Orchard, J.W., Puranik, R., Raju, H., Freedman, B., Semsarian, C., ECG-based cardiac screening programs: Legal, ethical, and logistical considerations (2019) Heart Rhythm, 16, pp. 1584-1591; Pathak, R.K., Elliott, A., Middeldorp, M.E., Meredith, M., Mehta, A.B., Mahajan, R., Sanders, P., Impact of CARDIOrespiratory fitness on arrhythmia recurrence in obese individuals with atrial fibrillation: The CARDIO-FIT study (2015) Journal of the American College of Cardiology, 66, pp. 985-966. , https://doi.org/10.1016/j.jacc.2015.06.488; Peake, J.M., Kerr, G., Sullivan, J.P., A Critical review of consumer wearables, mobile applications, and equipment for providing biofeedback, monitoring stress, and sleep in physically active populations (2018) Frontiers in Physiology, 9, p. 743. ,

https://doi.org/10.3389/fphys.2018.00743; Peart, D.J., Balsalobre-Fernández, C., Shaw, M.P., Use of mobile applications to collect data in sport, health, and exercise science: A narrative review (2019) The Journal of Strength & Conditioning Research, 33, pp. 1167-1177; Piercy, K.L., Troiano, R.P., Ballard, R.M., Carlson, S.A., Fulton, J.E., Galuska, D.A., Olson, R.D., The physical activity guidelines for Americans (2018) Journal of the American Medical Association, 320, pp. 2020-2028; Rosenberger, M.E., Buman, M.P., Haskell, W.L., McConnell, M.V., Carstensen, L.L., Twenty-four hours of sleep, sedentary behavior, and physical activity with nine wearable devices (2016) Medicine and Science in Sports and Exercise, 4, pp. 457-465; Seshadri, D.R., Li, R.T., Voos, J.E., Rowbottom, J.R., Alfes, C.M., Zorman, C.A., Drummond, C.K., Wearable sensors for monitoring the internal and external workload of the athlete (2019) NPJ Digital Medicine, 2, p. 71; Singh, N., Moneghetti, K.J., Christle, J.W., Hadley, D., Froelicher, V., Plews, D., Heart rate variability: An old metric with new meaning in the era of using mhealth technologies for health and exercise training guidance. Part two: Prognosis and training (2018) Arrhythmia & Electrophysiology Review, 7, pp. 247-255; Sullivan, A.N., Lachman, M.E., Behavior change with fitness technology in sedentary adults: A review of the evidence for increasing physical activity (2017) Frontiers in Public Health, 4, p. 289. , https://doi.org/10.3389/fpubh.2016.00289; (2017) The growing value of digital health: Evidence and impact on human health and the healthcare system, , https://www.iqvia.com/insights/the-iqvia-institute/reports/the-growing-value-of-digital-health; Abed, H.S., Wittert, G.A., Leong, D.P., Shirazi, M.G., Bahrami, B., Middeldorp, M.E., Sanders, P., Effect of weight reduction and cardiometabolic risk factor management on symptom burden and severity in patients with atrial fibrillation a randomized clinical trial (2013) Journal of the American Medical Association, 310, pp. 2050-2060; Boushey, C.J., Spoden, M., Zhu, F.M., Delp, E.J., Kerr, D.A., New mobile methods for dietary assessment: Review of image-assisted and image-based dietary assessment methods (2017) The Proceedings of the Nutrition Society, 76, pp. 283-294; Donnellan, E., Wazni, O.M., Kanj, M., Baranowski, B., Cremer, P., Harb, S., Saliba, W.I., Association between pre-ablation bariatric surgery and atrial fibrillation recurrence in morbidly obese patients undergoing atrial fibrillation ablation (2019) Europace, 21, pp. 1476-1483; Dounavi, K., Tsoumani, O., Mobile health applications in weight management: A systematic literature review (2019) American Journal of Preventive Medicine, 56, pp. 894-903; El-Gayar, O., Timsina, P., Nawar, N., Eid, W., Mobile applications for diabetes self-management: Status and potential (2013) Journal of Diabetes Science and Technology, 7, pp. 247-262; Fang, S., Liu, C., Zhu, F., Delp, E.J., Boushey, C.J., Single-view food portion estimation based on geometric models (2015) ISM: IEEE International Symposium on Multimedia: Proceedings IEEE International Symposium on Multimedia, 2015, pp. 385-390; Folsom, A.R., Yatsuya, H., Nettleton, J.A., Lutsey, P.L., Cushman, M., Rosamond, W.D., Investigators, A.S., Community prevalence of ideal cardiovascular health, by the American Heart Association definition, and relationship with cardiovascular disease incidence (2011) Journal of the American College of Cardiology, 57, pp. 1690-1696; Griffiths, C., Harnack, L., Pereira, M.A., Assessment of the accuracy of nutrient calculations of five popular nutrition tracking applications (2018) Public Health Nutrition, 21, pp. 1495-1502; Pathak, R.K., Middeldorp, M.E., Meredith, M., Mehta, A.B., Mahajan, R., Sanders, P., Long-term effect of goal-directed weight management in an atrial fibrillation cohort a long-term follow-up study (LEGACY) (2015) Journal of the American College of Cardiology, 65, pp. 2159-2169; Six, B.L., Schap, T.E., Zhu, F.Q.M.,

Mariappan, A., Bosch, M., Delp, E.J., Boushey, C.J., Evidence-based development of a mobile telephone food record (2010) Journal of the American Dietetic Association, 110, pp. 74-79; Zhu, F.Q., Bosch, M., Woo, I., Kim, S., Boushey, C.J., Ebert, D.S., Delp, E.J., The use of mobile devices in aiding dietary assessment and evaluation (2010) IEEE Journal of Selected Topics in Signal Processing, 4, pp. 756-766; Angaran, P., Mariano, Z., Dragan, V., Zou, L., Atzema, C.L., Mangat, I., Dorian, P., The atrial fibrillation therapies after ER visit: Outpatient care for patients with acute AF: the AFTER3 study (2015) Journal of Atrial Fibrillation, 7, p. 1187; Lin, J.S., O'Connor, E.A., Evans, C.V., Senger, C.A., Rowland, M.G., Groom, H.C., (2014) Behavioral Counseling to Promote a Healthy Lifestyle for Cardiovascular Disease Prevention in Persons With Cardiovascular Risk Factors: An Updated Systematic Evidence Review for the U.S. Preventive Services Task Force [Internet] Rockville (MD): Agency for Healthcare Research and Quality (US); U.S. Preventive Services Task Force Evidence Syntheses, formerly Systematic Evidence Reviews; Hendriks, J.M., de Wit, R., Crijns, H.J., Vrijhoef, H.J., Prins, M.H., Pisters, R., Pison, L.A., Tieleman, R.G., Nurse-led care vs. usual care for patients with atrial fibrillation: results of a randomized trial of integrated chronic care vs. routine clinical care in ambulatory patients with atrial fibrillation (2012) European Heart Journal, 33 (21), pp. 2692-2699. , https://doi.org/10.1093/eurheartj/ehs071; Chow, C.K., Ariyarathna, N., Islam, S.M., Thiagalingam, A., Redfern, J., mHealth in cardiovascular health care (2016) Heart Lung & Circulation, 25, pp. 802-807; Coorey, G.M., Neubeck, L., Mulley, J., Redfern, J., Effectiveness, acceptability and usefulness of mobile applications for cardiovascular disease self-management: Systematic review with meta-synthesis of quantitative and qualitative data (2018) European Journal of Preventive Cardiology, 25, pp. 505-521; Gandhi, S., Chen, S., Hong, L., Sun, K., Gong, E., Li, C., Yan, L.L., Schwalm, J.D., Effect of mobile health interventions on the secondary prevention of cardiovascular disease: Systematic review and meta-analysis (2017) Canadian Journal of Cardiology, 33, pp. 219-231; Guo, Y., Chen, Y., Lane, D.A., Liu, L., Wang, Y., Lip, G.Y.H., Mobile health technology for atrial fibrillation management integrating decision support, education and patient involvement: mAF App trial (2017) The American Journal of Medicine, 130, pp. 1388-1396.e6. , https://doi.org/10.1016/j.amjmed.2017.07.003; Hagglund, E., Lynga, P., Frie, F., Ullman, B., Persson, H., Melin, M., Hagerman, I., Patient-centred home-based management of heart failure. Findings from a randomised clinical trial evaluating a tablet computer for self-care, quality of life and effects on knowledge (2015) Scandinavian Cardiovascular Journal, 49, pp. 193-199; Kotecha, D., Chua, W.W.L., Fabritz, L., Hendriks, J., Casadei, B., Schotten, U., Kirchhof, P., European Society of Cardiology (ESC) Atrial Fibrillation Guidelines Taskforce, the CATCH ME consortium and the European Heart Rhythm Association (EHRA). European Society of Cardiology smartphone and tablet applications for patients with atrial fibrillation and their health care providers (2018) Europace, 20, pp. 225-233. , https://doi.org/10.1093/europace/eux299; Park, L.G., Beatty, A., Stafford, Z., Whooley, M.A., Mobile phone interventions for the secondary prevention of cardiovascular disease (2016) Progress in Cardiovascular Diseases, 58, pp. 639-650; Pfaeffli Dale, L., Dobson, R., Whittaker, R., Maddison, R., The effectiveness of mobile-health behaviour change interventions for cardiovascular disease self-management: A systematic review (2016) European Journal of Preventive Cardiology, 23, pp. 801-817; Slotwiner, D.J., Tarakji, K.G., Al-Khatib, S.M., Passman, R.S., Saxon, L.A., Peters, N.S., Marrouche, N.F., Transparent sharing of digital health data: A call to action (2019) Heart

Rhythm, 16. , https://doi.org/10.1016/j.hrthm.2019.04.042; Walsh, J.A., 3rd, Topol, E.J., Steinhubl, S.R., Novel wireless devices for cardiac monitoring (2014) Circulation, 130, pp. 573-581; Varnfield, M., Karunanithi, M., Lee, C.K., Honeyman, E., Arnold, D., Ding, H., Smith, C., Walters, D.L., Smartphone-based home care model improved use of cardiac rehabilitation in postmyocardial infarction patients: Results from a randomised controlled trial (2014) Heart, 100, pp. 1770-1779; VA to provide capability for veterans to access their VA health data on Apple iPhones, , https://www.va.gov/opa/pressrel/pressrelease.cfm?id55199; Blondon, K., Meyer, P., Lovis, C., Ehrler, F., Gamification and mHealth: A model to bolster cardiovascular disease self-management (2017) Swiss Medical Informatics, , https://doi.org/10.4414/smi.33.00398; Chow, C.K., Redfern, J., Hillis, G.S., Thakkar, J., Santo, K., Hackett, M.L., Thiagalingam, A., Effect of lifestyle-focused text messaging on risk factor modification in patients with coronary heart disease: A randomized clinical trial (2015) Journal of the American Medical Association, 314, pp. 1255-1263; Conroy, D.E., Yang, C.H., Maher, J.P., Behavior change techniques in top-ranked mobile apps for physical activity (2014) American Journal of Preventive Medicine, 46, pp. 649-652; Coorey, G.M., Neubeck, L., Mulley, J., Redfern, J., Effectiveness, acceptability and usefulness of mobile applications for cardiovascular disease self-management: Systematic review with meta-synthesis of quantitative and qualitative data (2018) European Journal of Preventive Cardiology, 25, pp. 505-521; Cugelman, B., Gamification: What it is and why it matters to digital health behavior change developers (2013) JMIR Serious Games, 1; Direito, A., Dale, L.P., Shields, E., Dobson, R., Whittaker, R., Maddison, R., Do physical activity and dietary smartphone applications incorporate evidence-based behaviour change techniques? (2014) BMC Public Health, 14, p. 646; Edwards, E.A., Lumsden, J., Rivas, C., Steed, L., Edwards, L.A., Thiyagarajan, A., Walton, R.T., Gamification for health promotion: systematic review of behaviour change techniques in smartphone apps (2016) British Medical Journal Open, 6; Gandhi, S., Chen, S., Hong, L., Sun, K., Gong, E., Li, C., Schwalm, J.-D., Effect of mobile health interventions on the secondary prevention of cardiovascular disease: Systematic review and meta-analysis (2017) Canadian Journal of Cardiology, 33, pp. 219-231. , https://doi.org/10.1016/j.cjca.2016.08.017; Gustafson, D.H., McTavish, F.M., Chih, M.Y., Atwood, A.K., Johnson, R.A., Boyle, M.G., Shah, D., A smartphone application to support recovery from alcoholism: A randomized clinical trial (2014) Journal of the American Medical Association Psychiatry, 71, pp. 566-572; Johnson, D., Deterding, S., Kuhn, K.A., Staneva, A., Stoyanov, S., Hides, L., Gamification for health and wellbeing: A systematic review of the literature (2016) Internet Interventions, 2016 (6), pp. 89-106; Nahum-Shani, I., Smith, S.N., Spring, B.J., Collins, L.M., Witkiewitz, K., Tewari, A., Murphy, S.A., Just-in-Time adaptive interventions (JITAIs) in mobile health: Key components and design principles for ongoing health behavior support (2018) Annals of Behavioral Medicine, 52, pp. 446-462; Nahum-Shani, I., Hekler, E.B., Spruijt-Metz, D., Building health behavior models to guide the development of just-in-time adaptive interventions: A pragmatic framework (2015) Health Psychology, 34s, pp. 1209-1219; Patrick, K., Raab, F., Adams, M.A., Dillon, L., Zabinski, M., Rock, C.L., Griswold, W.G., Norman, G.J., A text message-based intervention for weight loss: Randomized controlled trial (2009) Journal of Medical Internet Research, 11; Pfaeffli Dale, L., Dobson, R., Whittaker, R., Maddison, R., The effectiveness of mobile-health behaviour change interventions for cardiovascular disease self-management: A systematic review (2016) European Journal of Preventive Cardiology, 23, pp. 801-817; Riley, W., Obermayer, J.,

Jean-Mary, J., Internet and mobile phone text messaging intervention for college smokers (2008) Journal of American College Health, 57, pp. 245-248; Rumsfeld, J.S., Alexander, K.P., Goff, D.C., Jr., Graham, M.M., Ho, P.M., Masoudi, F.A., Zerwic, J.J., American Heart Association Council on Quality of Care and Outcomes Research, Council on Cardiovascular and Stroke Nursing, Council on Epidemiology and Prevention, Council on Peripheral Vascular Disease, and Stroke Council. Cardiovascular Health: The Importance of Measuring Patient-Reported Health Status A Scientific Statement From the American Heart Association (2013) Circulation, 127, pp. 2233-2249. , https://doi.org/10.1161/CIR.0b013e3182949a2e; Sardi, L., Idri, A., Fernandez-Aleman, J.L., A systematic review of gamification in e-Health (2017) Journal of Biomedical Informatics, 71, pp. 31-48; Varma, N., Piccini, J.P., Snell, J., Fischer, A., Dalal, N., Mittal, S., The relationship between level of adherence to automatic wireless remote monitoring and survival in pacemaker and defibrillator patients (2015) Journal of the American College of Cardiology, 65, pp. 2601-2610. , https://doi.org/10.1016/j.jacc.2015.04.033; Besaleva, L.I., Weaver, A.C., (2014) CrowdHelp: m-Health Application for Emergency Response Improvement through Crowdsourced and Sensor-Detected Information, , https://ieeexplore.ieee.org/document/6693335, https://doi.org/10.1109/WTS.2014.6835005; Fox, S., The Social Life of Health Information, , https://www.pewresearch.org/fact-tank/2014/01/15/the-social-life-of-health-information/; Swan, M., Emerging patient-driven health care models: An examination of health social networks, consumer personalized medicine and quantified self-tracking (2009) International Journal of Environmental Research and Public Health, 6, pp. 492-525; Swan, M., Health 2050: The realization of personalized medicine through crowdsourcing, the quantified self, and the participatory biocitizen (2012) Journal of Personalized Medicine, 2, pp. 93-118; Burke, L.E., Ma, J., Azar, K.M., Bennett, G.G., Peterson, E.D., Zheng, Y., Quinn, C.C., Current science on consumer use of mobile health for cardiovascular disease prevention: A scientific statement from the American Heart Association Circulation, 132 (2015), pp. 1157-1213; Chaudhry, S.I., Mattera, J.A., Curtis, J.P., Spertus, J.A., Herrin, J., Lin, Z., Krumholz, H.M., Telemonitoring in patients with heart failure (2010) New England Journal of Medicine, 363, pp. 2301-2309; Chow, C.K., Ariyarathna, N., Islam, S.M., Thiagalingam, A., Redfern, J., mHealth in cardiovascular health care (2016) Heart Lung & Circulation, 25, pp. 802-807; Dunton, G.F., Sustaining health-protective behaviors such as physical activity and healthy eating (2018) Journal of the American Medical Association, 320, pp. 639-640; Flores Mateo, G., Granado-Font, E., Ferre-Grau, C., Ferré-Grau, C., Montaña-Carreras, X., Mobile phone apps to promote weight loss and increase physical activity: A systematic review and meta-analysis (2015) Journal of Medical Internet Research, 17; Fukuoka, Y., Gay, C., Haskell, W., Arai, S., Vittinghoff, E., Identifying factors associated with dropout during prerandomization run-in period from an mhealth physical activity education study: The mPED trial (2015) JMIR Mhealth Uhealth, 3; Kumar, S., Nilsen, W.J., Abernethy, A., Atienza, A., Patrick, K., Pavel, M., Swendeman, D., Mobile health technology evaluation: the mHealth evidence workshop (2013) American Journal of Preventive Medicine, 45, pp. 228-236; Morgan, J.M., Kitt, S., Gill, J., McComb, J.M., Ng, G.A., Raftery, J., Cowie, M.R., Remote management of heart failure using implantable electronic devices (2017) European Heart Journal, 38, pp. 2352-2360; (2014) My heart, my life, , https://myheartmylife.org.au/; Owen, J.E., Jaworski, B.K., Kuhn, E., Makin-Byrd, K.N., Ramsey, K.M., Hoffman, J.E., mHealth in the wild: Using novel data to examine the reach, use, and impact of PTSD coach (2015) JMIR Mental

Health, 2; Perez, M.V., Mahaffey, K.W., Hedlin, H., Rumsfeld, J.S., Garcia, A., Ferris, T., Turakhia, M.P., Large-scale assessment of a smartwatch to identify atrial fibrillation New England Journal of Medicine, 381 (2019), pp. 1909-1917. , https://doi.org/10.1056/NEJMoa1901183; Simblett, S., Greer, B., Matcham, F., Curtis, H., Polhemus, A., Ferrão, J., Gamble, P., Wykes, T., Barriers to and facilitators of engagement with remote measurement technology for managing health: Systematic review and content analysis of findings (2018) Journal of Medical Internet Research, 20; Steinhubl, S.R., Muse, E.D., Topol, E.J., The emerging field of mobile health (2015) Science Translational Medicine, 7, p. 283rv283; Whitehead, L., Seaton, P., The effectiveness of self-management mobile phone and tablet apps in long-term condition management: A systematic review (2016) Journal of Medical Internet Research, 18; Varma, N., Michalski, J., Stambler, B., Pavri, B.B., Superiority of automatic remote monitoring compared with in-person evaluation for scheduled ICD follow-up in the TRUST trial - testing execution of the recommendations (2014) European Heart Journal, 35, pp. 1345-1352. , https://doi.org/10.1093/eurheartj/ehu066; Varma, N., (2020) Remote Patent Management Of Heart Failure Patients – How Long Should It Go On? Lancet Digital health, 2. , https://doi.org/10.1016/S2589-7500(19)30221-3, ISSUE 1, E2-E3; (2019) Sustaining healthy behaviours (AHA Simple 7), , https://www.heart.org/en/healthy-living/healthy-lifestyle/my-life-check--lifes-simple-7; (2014) Our healthy recipe finder app, , http://www.bhf.org.uk/heart-health/prevention/healthy-eating/our-healthy-recipe-finder-app.aspx; (2014) Heart and stroke etools for a healthier you, , http://www.heartandstroke.com/site/c.ikIQLcMWJtE/b.8324551/k.972C/_30_days.htm?utm_campaign=offline&utm_source=30days&utm_medium=vanity; (2017) Inside wearables: how the science of human behavior change offers the secret to long-term, , https://medium.com/@endeavourprtnrs/inside-wearable-how-the-science-of-human-behavior-change-offers-the-secret-to-long-term-engagement-a15b3c7d4cf3; Bhavnani, S.P., Sola, S., Adams, D., Venkateshvaran, A., Dash, P.K., Sengupta, P.P., Kadakia, A., A randomized trial of pocket-echocardiography integrated mobile health device assessments in modern structural heart disease clinics (2018) Cardiovascular Imaging, 11, pp. 546-557. , https://doi.org/10.1016/j.jcmg.2017.06.019; Coorey, G.M., Neubeck, L., Mulley, J., Redfern, J., Effectiveness, acceptability and usefulness of mobile applications for cardiovascular disease self-manegement: Systematic review with meta-synthesis of quantitative and qualitattive data (2018) European Journal of Preventive Cardiology, 25, pp. 505-521. , https://doi.org/10.1177/2047487317750913; Coorey, G.M., Neubeck, L., Mulley, J., Glinatsis, H., Belshaw, J., Kirkness, A., Neubeck, L., Mobile technology use across age groups in patients eligible for cardiac rehabilitation: Survey study (2017) JMIR Mhealth Uhealth, 5; Gallagher, R., Roach, K., Sadler, L., Glinatsis, H., Belshaw, J., Kirkness, A., Neubeck, L., Mobile technology use across age groups in patients eligible for cardiac rehabilitation: Survey study (2017) JMIR Mhealth Uhealth, 5. , https://doi.org/10.2196/mhealth.8352; Martin, T., Assessing mHealth: Opportunities and barriers to patient engagement (2012) Journal of Health Care for the Poor and Underserved, 23, pp. 935-941; Neubeck, L., Lowres, N., Benjamin, E.J., Freedman, S.B., Coorey, G., Redfern, J., The mobile revolution–using smartphone apps to prevent cardiovascular disease (2015) Nature Reviews. Cardiology, 12, pp. 350-360; Neubeck, L., Cartledge, S., Dawkes, S., Gallagher, R., Is there an app for that? Mobile phones and secondary prevention of cardiovascular disease (2017) Current Opinion Cardiology, 32, pp. 567-571; Smith, A., (2017) Record shares of Americans now

own smartphones, have home broadband, , https://www.pewresearch.org/fact-tank/2017/01/12/evolution-of-technology/, Pew Research Center; Tarakji, K.G., Vives, C.A., Patel, A.S., Fagan, D.H., Sims, J.J., Varma, N., Success of pacemaker remote monitoring using app-based technology: Does patient age matter? (2018) Pacing and Clinical Electrophysiology, 41, pp. 1329-1335; (2018), https://www.pewresearch.org/internet/fact-sheet/mobile/; Redfern, J., Santo, K., Coorey, G., Thakkar, J., Hackett, M., Thiagalingam, A., Chow, C.K., Factors influencing engagement, perceived usefulness and behavioral mechanisms associated with a text message support program (2016) PLoS One, 11; Varma, N., Remote patent management of heart failure patients – how long should it go on? (2020) Lancet Digital Health, 2. , https://doi.org/10.1016/S2589-7500(19)30221-3; Andrade, J.G., Champagne, J., Dubuc, M., Deyell, M.W., Verma, A., Macle, L., Khairy, P., Cryoballoon or radiofrequency ablation for atrial fibrillation assessed by continuous monitoring: A randomized clinical trial (2019) Circulation, 140, pp. 1779-1788; Chen, L.Y., Chung, M.K., Allen, L.A., Ezekowitz, M., Furie, K.L., McCabe, P., Turakhia, M.P., Atrial fibrillation burden: moving beyond atrial fibrillation as a binary entity: A scientific statement from the American Heart Association (2018) Circulation, 137; Glotzer, T.V., Daoud, E.G., Wyse, D.G., Singer, D.E., Ezekowitz, M.D., Hilker, C., Ziegler, P.D., The relationship between daily atrial tachyarrhythmia burden from implantable device diagnostics and stroke risk: The TRENDS study (2009) Circulation: Arrhythmia and Electrophysiology, 2, pp. 474-480; Guo, X., Vittinghoff, E., Olgin, J.E., Marcus, G.M., Pletcher, M.J., Volunteer participation in the Health eHeart Study: A comparison with the US population (2017) Science Reports, 2017 (7), p. 1956. , https://doi.org/10.1038/s41598-017-02232-y; Guo, Y., Wang, H., Zhang, H., Liu, T., Liang, Z., Xia, Y., Lip, G.Y.H., Mobile photoplethysmographic technology to detect atrial fibrillation (2019) Journal of the American College of Cardiology, 2019 (74), pp. 2365-2375. , https://doi.org/10.1016/j.jacc.2019.08.019; Guo, Y., Lane, D.A., Wang, L., Zhang, H., Wang, H., Zhang, W., Lip, G.Y.H., Mobile health technology to improve care for patients with atrial fibrillation (2020) Journal of the American College of Cardiology, 75, pp. 1523-1534; Kaplan, R.M., Koehler, J., Ziegler, P.D., Sarkar, S., Zweibel, S., Passman, R.S., Stroke risk as a function of atrial fibrillation duration and CHA2DS2-VASc score (2019) Circulation, 140, pp. 1639-1646. , https://doi.org/10.1161/CIRCULATIONAHA.119.041303; Nguyen, K.T., Olgin, J.E., Pletcher, M.J., Ng, M., Kaye, L., Moturu, S., Marcus, G.M., Smartphone-based geofencing to ascertain hospitalizations (2017) Circulation Cardiovascular Quality & Outcomes, 10. , https://doi.org/10.1161/CIRCOUTCOMES.116.003326; Passman, R., Leong-Sit, P., Andrei, A.C., Huskin, A., Tomson, T.T., Bernstein, R., Zimetbaum, P., Targeted anticoagulation for atrial fibrillation guided by continuous rhythm assessment with an insertable cardiac monitor: The rhythm evaluation for anticoagulation with continuous monitoring (REACT.COM) pilot study (2016) Journal of Cardiovascular Electrophysiology, 27, pp. 264-270. , https://doi.org/10.1111/jce.12864; Perez, M.V., Mahaffey, K.W., Hedlin, H., Rumsfeld, J.S., Garcia, A., Ferris, T., Turakhia, M.P., Large-scale assessment of a smartwatch to identify atrial fibrillation (2019) New England Journal of Medicine, 381, pp. 1909-1917. , https://doi.org/10.1056/NEJMoa1901183; Piccini, J.P., Clark, R.L., Kowey, P.R., Mittal, S., Dunnmon, P., Stockbridge, N., Ismat, F., Long-term electrocardiographic safety monitoring in clinical drug development: A report from the Cardiac Safety Research Consortium (2017) American Heart Journal, 187, pp. 156-169; Piccini, J.P., Passman, R., Turakhia,

M., Connolly, A.T., Nabutovsky, Y., Varma, N., Atrial fibrillation burden, progression, and the risk of death: a case-crossover analysis in patients with cardiac implantable electronic devices (2019) Europace, 21, pp. 404-413; Turakhia, M.P., Desai, M., Hedlin, H., Rajmane, A., Talati, N., Ferris, T., Perez, M.V., Rationale and design of a large-scale, app-based study to identify cardiac arrhythmias using a smartwatch: The Apple Heart Study (2019) American Heart Journal, 207, pp. 66-75; Van Gelder, I.C., Groenveld, H.F., Crijns, H.J., Tuininga, Y.S., Tijssen, J.G., Alings, A.M., Van den Berg, M.P., Lenient versus strict rate control in patients with atrial fibrillation (2010) New England Journal of Medicine, 362, pp. 1363-1373. , https://doi.org/10.1056/NEJMoa1001337; Varma, N., Stambler, B., Chun, S., Detection of atrial fibrillation by implanted devices with wireless data transmission capability (2005) Pacing and Clinical Electrophysiology, 28, pp. S133-S136; Varma, N., Marrouche, N.F., Aguinaga, L., Albert, C.M., Arbelo, E., Choi, J.I., Varosy, P.D., HRS/EHRA/APHRS/LAHRS/ACC/AHA Worldwide Practical Guidance for Telehealth and Arrhythmia Monitoring During and After a Pandemic (2020) Journal of the American College of Cardiology, , https://doi.org/10.1016/j.jacc.2020.06.019; Wong, J.A., Conen, D., Van Gelder, I.C., McIntyre, W.F., Crijns, H.J., Wang, J., Healey, J.S., Progression of Device-Detected Subclinical Atrial Fibrillation and the Risk of Heart Failure (2018) Journal of the American College of Cardiology, 71, pp. 2603-2611; (2020) Fitbit launches large-scale consumer health study to detect a-fib via heart rate sensors, algorithm, , https://www.mobihealthnews.com/news/fitbit-launches-large-scale-consumer-health-study-detect-fib-heart-rate-sensors-algorithm, https://www.mobihealthnews.com/news/fitbit-launches-large-scale-consumer-health-study-detect-fib-heart-rate-sensors-algorithm; https://www.iso.org/standard/63904.htmlhttps://www.iso.org/standard/63904.html; Chow, C.K., Redfern, J., Hillis, G.S., Thakkar, J., Santo, K., Hackett, M.L., Thiagalingam, A., Effect of lifestyle-focused text messaging on risk factor modification in patients with coronary heart disease: A randomized clinical trial (2015) Journal of the American Medical Association, 314, pp. 1255-1263; Ratwani, R.M., Savage, E., Will, A., Arnold, R., Khairat, S., Miller, K., Hettinger, A.Z., A usability and safety analysis of electronic health records: A multi-center study (2018) Journal of the American Medical Information Association, 25, pp. 1197-1201; Slotwiner, D.J., Abraham, R.L., Al-Khatib, S.M., Anderson, H.V., Bunch, T.J., Ferrara, M.G., Wilkoff, B.L., HRS White Paper on interoperability of data from cardiac implantable electronic devices (CIEDs) (2019) Heart Rhythm, 16. , https://doi.org/10.1016/j.hrthm.2019.05.002; Spaulding, E.M., Marvel, F.A., Lee, M.A., Yang, W.E., Demo, R., Wang, J., Martin, S.S., Corrie health digital platform for self-management in secondary prevention after acute myocardial infarction (2019) Circulation. Cardiovascular Quality & Outcomes, 12; Gollakota, S., Hassanieh, H., Ransford, B., Katabi, D., Fu, K., They can hear your heartbeats: Non-invasive security for implantable medical devices (2011) SIGCOMM Computer Communication Review, 41. , https://doi.org/10.1145/2043164.2018438; Jalali, M.S., Russell, B., Razak, S., Gordon, W.J., EARS to cyber incidents in health care (2019) Journal of the American Medical Information Association, 26, pp. 81-90; Klonoff, D.C., Cybersecurity for connected diabetes devices (2015) Journal of Diabetes Science and Technology, 9, pp. 1143-1147; Kruse, C.S., Frederick, B., Jacobson, T., Monticone, D.K., Cybersecurity in healthcare: A systematic review of modern threats and trends (2017) Technology Health Care, 25, pp. 1-10; Mansfield-Devine, S., (2016) Ransomware: taking businesses hostage, , Network Security, 8–17; (2016) Hospitals become major target for ransomware, ,

https://doi.org/10.1016/S1353-4858(16)30031-9, Network Security, 2016, 1–2; Perakslis, E.D., Cybersecurity in health care (2014) New England Journal of Medicine, 371, pp. 395-397; Saxon, L.A., Varma, N., Epstein, L.M., Ganz, L.I., Epstein, A.E., Factors influencing the decision to proceed to firmware upgrades to implanted pacemakers for cybersecurity risk mitigation (2018) Circulation, 138, pp. 1274-1276; Shanon, C.E., Communication theory of secrecy systems' (1949) Bell System Technical Journal, 28; Shuren, J., Patel, B., Gottlieb, S., FDA regulation of mobile medical apps (2018) Journal of the American Medical Association, 320, pp. 337-338; Voelker, R., FDA joins new effort to strengthen medical device cybersecurity (2018) Journal of the American Medical Association, 320, p. 1970; Bruining, N., Caiani, E., Chronaki, C., Guzik, P., Vvan der Velde, E., Acquisition and analysis of cardiovascular signals on smartphones: potential, pitfalls and perspectives: by the Task Force of the e-Cardiology Working Group of European Society of Cardiology (2014) European Journal of Preventive Cardiology, 21 (2), pp. 4-13; Crossley, G.H., Boyle, A., Vitense, H., Chang, Y., Mead, R.H., The CONNECT (Clinical Evaluation of Remote Notification to Reduce Time to Clinical Decision) trial: the value of wireless remote monitoring with automatic clinician alerts (2011) Journal of the American College of Cardiology, 57, pp. 1181-1189; Feldman, D.I., Robison, T.W., Pacor, J.M., Caddell, L.C., Feldman, E.B., Deitz, R.L., Blaha, M.J., Harnessing mHealth technologies to increase physical activity and prevent cardiovascular disease (2018) Clinical Cardiology, 41, pp. 985-991; Guedon-Moreau, L., Lacroix, D., Sadoul, N., Clementy, J., Kouakam, C., Hermida, J.S., Aliot, E., Kacet, S., Costs of remote monitoring vs. ambulatory follow-ups of implanted cardioverter defibrillators in the randomized ECOST study (2014) Europace, 16, pp. 1181-1188. , https://doi.org/10.1093/europace/euu012; Heidbuchel, H., Hindricks, G., Broadhurst, P., Van Erven, L., Fernandez-Lozano, I., Rivero-Ayerza, M., Annemans, L., EuroEco (European Health Economic Trial on Home Monitoring in ICD Patients): a provider perspective in five European countries on costs and net financial impact of follow-up with or without remote monitoring (2015) European Heart Journal, 36, pp. 158-169. , https://doi.org/10.1093/eurheartj/ehu339; Hindricks, G., Taborsky, M., Glikson, M., Heinrich, U., Schumacher, B., Katz, A., Søgaard, P., Implant-based multiparameter telemonitoring of patients with heart failure (IN-TIME): A randomised controlled trial (2014) Lancet, 384, pp. 583-590; Jiang, X., Ming, W.K., You, J.H., The Cost-Effectiveness of Digital Health Interventions on the Management of Cardiovascular Diseases: Systematic Review (2019) Journal of Medical Internet Research, 21. , https://doi.org/10.2196/13166; Mabo, P., Victor, F., Bazin, P., Ahres, S., Babuty, D., Da Costa, A., Daubert, J.C., A randomized trial of long-term remote monitoring of pacemaker recipients (the COMPAS trial) (2012) European Heart Journal, 33, pp. 1105-1111; Orchard, J.J., Neubeck, L., Freedman, B., Webster, R., Patel, A., Gallagher, R., Lowres, N., Atrial Fibrillation Screen, Management and Guideline Recommended Therapy (AF SMART II) in the rural primary care setting: an implementation study protocol (2018) British Medical Journal Open, 8; Treskes, R.W., van der Velde, E.T., Barendse, R., Bruining, N., Mobile health in cardiology: a review of currently available medical apps and equipment for remote monitoring (2016) Expert Review of Medical Devices, 13, pp. 823-830; Varma, N., Epstein, A.E., Irimpen, A., Schweikert, R., Love, C., Efficacy and safety of automatic remote monitoring for implantable cardioverter-defibrillator follow-up: the Lumos-T Safely Reduces Routine Office Device Follow-up (TRUST) trial (2010) Circulation, 2010 (122), pp. 325-332. , https://doi.org/10.1161/CIRCULATIONAHA.110.937409; Lee, T.T., Kesselheim, A.S., U.S. Food and Drug Administration Precertification Pilot Program for Digital

Health Software: Weighing the Benefits and Risks (2018) Annals of Internal Medicine, 168, pp. 730-732; Shuren, J., Patel, B., Gottlieb, S., FDA regulation of mobile medical apps (2018) Journal of the American Medical Association, 320, pp. 337-338; (2019) Digital Health. In: U.S. Food and Drug Administration [Internet], , https://www.fda.gov/medical-devices/digital-health; https://www.fda.gov/medical-devices/digital-health-software-precertification-pre-cert-program/precertification-pre-cert-pilot-program-milestones-and-next-steps; Attia, Z.I., Kapa, S., Lopez-Jimenez, F., McKie, P.M., Ladewig, D.J., Satam, G., Friedman, P.A., Screening for cardiac contractile dysfunction using an artificial intelligence-enabled electrocardiogram (2019) Nature Medicine, 25, pp. 70-74; Attia, Z.I., Noseworthy, P.A., Lopez-Jimenez, F., Asirvatham, S.J., Deshmukh, A.J., Gersh, B.J., Friedman, P.A., An artificial intelligence-enabled ECG algorithm for the identification of patients with atrial fibrillation during sinus rhythm: a retrospective analysis of outcome prediction (2019) Lancet, 394, pp. 861-867; Awan, S.E., Bennamoun, M., Sohel, F., Sanfilippo, F.M., Dwivedi, G., Machine learning-based prediction of heart failure readmission or death: Implications of choosing the right model and the right metrics (2019) European Society of Cardiology Heart Failure Journal, 6, pp. 428-435; Bumgarner, J.M., Lambert, C.T., Hussein, A.A., Cantillon, D.J., Baranowski, B., Wolski, K., Tarakji, K.G., Smartwatch algorithm for automated detection of atrial fibrillation (2018) Journal of the American College of Cardiology, 71, pp. 2381-2388; Chamsi-Pasha, M.A., Sengupta, P.P., Zoghbi, W.A., Handheld echocardiography: Current state and future perspectives (2017) Circulation, 136, pp. 2178-2188; Clifton, D.A., Niehaus, K.E., Charlton, P., Colopy, G.W., Health informatics via machine learning for the clinical management of patients (2015) Yearbook of Medical Informatics, 10, pp. 38-43; Davenport, T., Kalakota, R., The potential for artificial intelligence in healthcare (2019) Future Healthcare Journal, pp. 94-98; Frizzell, J.D., Liang, L., Schulte, P.J., Yancy, C.W., Heidenreich, P.A., Hernandez, A.F., Laskey, W.K., Prediction of 30-day all-cause readmissions in patients hospitalized for heart failure: Comparison of machine learning and other statistical approaches (2017) Journal of the American Medical Association Cardiology, 2, pp. 204-209; Goto, S., Goto, S., Pieper, K.S., Bassand, J.P., Camm, A.J., Fitzmaurice, D.A., Kakkar, A.K., New AI Prediction Model Using Serial PT-INR Measurements in AF Patients on VKAs: GARFIELD-AF (2019) European Heart Journal Cardiovasc Pharmacother, , https://doi.org/10.1093/ehjcvp/pvz076; Halcox, J.P.J., Wareham, K., Cardew, A., Gilmore, M., Barry, J.P., Phillips, C., Gravenor, M.B., Assessment of remote heart rhythm sampling using the alivecor heart monitor to screen for atrial fibrillation: The REHEARSE-AF study (2017) Circulation, 136, pp. 1784-1794; Hannun, A.Y., Rajpurkar, P., Haghpanahi, M., Tison, G.H., Bourn, C., Turakhia, M.P., Ng, A.Y., Cardiologist-level arrhythmia detection and classification in ambulatory electrocardiograms using a deep neural network (2019) Nature Medicine, 25, pp. 65-69; Kagiyama, N., Shrestha, S., Farjo, P.D., Sengupta, P.P., Artificial intelligence: Practical primer for clinical research in cardiovascular disease (2019) Journal of the American Heart Association, 8; Marcolino, M.S., Oliveira, J.A.Q., D’Agostino, M., Ribeiro, A.L., Alkmim, M.B.M., Novillo-Ortiz, D., The impact of mhealth interventions: Systematic review of systematic reviews (2018) Journal of Medical Internet Research Mhealth Uhealth, 6; Nascimento, B.R., Beaton, A.Z., Nunes, M.C.P., Tompsett, A.R., Oliveira, K.K.B., Diamantino, A.C., Sable, C., Integration of echocardiographic screening by non-physicians with remote reading in primary care (2018) Heart, 105, pp. 283-290. , https://doi.org/10.1136/heartjnl-2018-313593; Powell, J., Trust Me, I’m a Chatbot: How Artificial Intelligence in Health Care Fails the Turing Test

(2019) Journal of Medical Internet Research, 21; Ribeiro, A.L.P., Paixão, G.M.M., Gomes, P.R., Ribeiro, M.H., Ribeiro, A.H., Canazart, J.A., Macfarlane, P.W., Tele-electrocardiography and bigdata: The CODE (Clinical Outcomes in Digital Electrocardiography) study (2019) Journal of Electrocardiology, 57S, pp. S75-S78. , https://doi.org/10.1016/j.jelectrocard.2019.09.008; Ribeiro, A.H., Ribeiro, M.H., Paixão, G.M.M., Oliveira, D.M., Gomes, P.R., Canazart, J.A., Ribeiro, A.L.P., (2019) Automatic Diagnosis of the Short-Duration 12-Lead ECG using a Deep Neural Network: the CODE Study [Internet], , http://arxiv.org/abs/1904.01949, arXiv [cs.LG]; Ribeiro, A.L., Oliveira, G.M.M., Toward a patient-centered, data-driven cardiology (2019) Arquivos Brasileiros de Cardiologia, 112, pp. 371-373; Safavi, K.C., Khaniyev, T., Copenhaver, M., Seelen, M., Zenteno Langle, A.C., Zanger, J., Dunn, P., Development and validation of a machine learning model to aid discharge processes for inpatient surgical care (2019) Journal of the American Medical Association Network Open, 2; Seetharam, K., Kagiyama, N., Sengupta, P.P., Application of mobile health, telemedicine and artificial intelligence to echocardiography (2019) Echo Research and Practice, 6, pp. R41-52; Smith, S.W., Walsh, B., Grauer, K., Wang, K., Rapin, J., Li, J., Fennell, W., Taboulet, P., A deep neural network learning algorithm outperforms a conventional algorithm for emergency department electrocardiogram interpretation (2019) Journal of Electrocardiology, 52, pp. 88-95; Steinhubl, S.R., Muse, E.D., Topol, E.J., The emerging field of mobile health (2015) Science Translational Medicine, 7, p. 283rv3; Tripoliti, E.E., Karanasiou, G.S., Kalatzis, F.G., Bechlioulis, A., Goletsis, Y., Naka, K., Fotiadis, D.I., A knowledge management system targeting the management of patients with heart failure (2019) Journal of Biomedical Informatics, 94, p. 103203; Weng, S.F., Reps, J., Kai, J., Garibaldi, J.M., Qureshi, N., Can machine-learning improve cardiovascular risk prediction using routine clinical data? (2017) PLoS One, 12; Zhang, J., Gajjala, S., Agrawal, P., Tison, G.H., Hallock, L.A., Beussink-Nelson, L., Deo, R.C., Fully automated echocardiogram interpretation in clinical practice (2018) Circulation, 138, pp. 1623-1635; Yahoo! Finance. Exclusive: What Fitbit's 6 billion nights of sleep data reveals about us, (2020). , https://finance.yahoo.com/news/exclusive-fitbits-6-billion-nights-sleep-data-reveals-us-110058417.html; Boehmer, J.P., Hariharan, R., Devecchi, F.G., Smith, A.L., Molon, G., Capucci, A., Singh, J.P., A multisensor algorithm predicts heart failure events in patients with implanted devices: Results from the MultiSENSE study (2017) JACC: Heart Failure, 5, pp. 216-225. , https://doi.org/10.1016/j.jchf.2016.12.011; Choi, P.J., Oskouian, R.J., Tubbs, R.S., Telesurgery: Past, present, and future (2018) Cureus Journal of Medical Science, 10. , https://doi.org/10.7759/cureus.2716; Curry, S.J., Krist, A.H., Owens, D.K., Barry, M.J., Caughey, A.B., Davidson, K.W., Wong, J.B., US Preventive Services Task Torce, screening for atrial fibrillation with electrocardiography: US preventive services task force recommendation statement (2018) Journal of the American Medical Association, 320, pp. 478-484; Haidegger, T., Sándor, J., Benyó, Z., Surgery in space: The future of robotic telesurgery (2011) Surgical Endoscopy, 25, pp. 681-690. , https://doi.org/10.1007/s00464-010-1243-3; Jiang, X., Ming, W.K., You, J.H., The cost-effectiveness of digital health interventions on the management of cardiovascular diseases: Systematic review (2019) Journal of Medical Internet Research, 21. , https://doi.org/10.2196/13166; Pluymaekers, N.A.H.A., Hermans, A.N.L., van der Velden, R.M., den Uijl, D.W., Vorstermans, B., Buskes, S., Linz, D., On-demand app-based rate and rhythm monitoring to manage atrial fibrillation through tele-consultations during COVID-19 (2020) International Journal of Cardiology Heart & Vascular, 28, p. 100533. ,

https://doi.org/10.1016/j.ijcha.2020.100533; Rosier, A., Mabo, P., Temal, L., Van Hille, P., Dameron, O., Deléger, L., Burgun, A., Personalized and automated remote monitoring of atrial fibrillation (2016) Europace, 18, pp. 347-352. , https://doi.org/10.1093/europace/euv234; Shinoda, Y., Sato, A., Adach, T., Nishi, I., Nogami, A., Aonuma, K., Ieda, M., Early clinical experience of radiofrequency catheter ablation using an audiovisual telesupport system (2020) Heart Rhythm, 17 (5), pp. 870-875. , https://doi.org/10.1016/j.hrthm.2020.01.018; Slotwiner, D.J., Abraham, R.L., Al-Khatib, S.M., Anderson, H.V., Bunch, T.J., Ferrara, M.G., Wilkoff, B.L., HRS White Paper on interoperability of data from cardiac implantable electronic devices (CIEDs) (2019) Heart Rhythm, 16, pp. e107-e127; Turakhia, M.P., Diagnosing with a camera from a distance—proceed cautiously and responsibly (2020) Journal of the American Medical Association Cardiol, 5, p. 107; Varma, N., Marrouche, N.F., Aguinaga, L., Albert, C.M., Arbelo, E., Choi, J.I., Varosy, P.D., HRS/EHRA/APHRS/LAHRS/ACC/AHA worldwide practical guidance for telehealth and arrhythmia monitoring during and after a pandemic (2020) Journal of the American College of Cardiology, 76, pp. 1363-1374. , https://doi.org/10.1016/j.jacc.2020.06.019; Yan, B.P., Lai, W.H.S., Chan, C.K.Y., Au, A.C.K., Freedman, B., Poh, Y.C., Poh, M.Z., High-throughput, contact-free detection of atrial fibrillation from video with deep learning (2020) Journal of the American Medical Association Cardiology, 5, pp. 105-107. , https://doi.org/10.1001/jamacardio.2019.4004; (2020) Notification of enforcement discretion for telehealth remote communications during the COVID-19 nationwide public health emergency, , https://www.hhs.gov/hipaa/for-professionals/special-topics/emergency-preparedness/notification-enforcement-discretion-telehealth/index.html; (2020) Enforcement policy for non-invasive remote monitoring devices used to support patient monitoring during the coronavirus disease-2019 (COVID-19) public health emergency, , https://www.fda.gov/regulatory-information/search-fda-guidance-documents/enforcement-policy-non-invasive-remote-monitoring-devices-used-support-patient-monitoring-during; https://bodimetrics.com/product/circul-sleep-and-fitness-ring, https://bodimetrics.com/product/circul-sleep-and-fitness-ring/; https://ghr.nlm.nih.gov/primer/precisionmedicine/initiative, https://ghr.nlm.nih.gov/primer/precisionmedicine/initiative},
correspondence_address1={Varma, N.; Cleveland ClinicUnited States; email: varman@ccf.org},
publisher={Blackwell Publishing Inc.},
issn={1082720X},
coden={ANELF},
pubmed_id={33513268},
language={English},
abbrev_source_title={Ann. Noninvasive Electrocardiol.},
document_type={Review},
source={Scopus},
}

@ARTICLE{Shah2021707,
author={Shah, S.Z.A. and Karam, J.A. and Zeb, A. and Ullah, R. and Shah, A. and Haq, I.U. and Ali, I. and Darain, H. and Chen, H.},
title={Movement is Improvement: The Therapeutic Effects of Exercise and General Physical Activity on Glycemic Control in Patients with Type 2 Diabetes Mellitus: A Systematic Review and Meta-Analysis of Randomized Controlled Trials},

journal={Diabetes Therapy},
year={2021},
volume={12},
number={3},
pages={707-732},
doi={10.1007/s13300-021-01005-1},
note={cited By 0},
url={https://www.scopus.com/inward/record.uri?eid=2-s2.0-85100555510&doi=10.1007%2fs13300-021-01005-1&partnerID=40&md5=7ca922d6d4d7a0a35de083526f154c09},
affiliation={Department of Rehabilitation Medicine, Tongji Hospital, Tongji Medical College, Huazhong University of Science and Technology, Wuhan, China; Department of applied exercise science, Concordia University Chicago, 7400 Augusta St., River Forest, IL 60305, United States; School of Health Sciences, Peshawar, Pakistan; Paraplegic Centre, Phase#4, Hayatabad, Peshawar, Pakistan; Institute of Physical Medicine and Rehabilitation Sciences, Khyber Medical University, Hayatabad, Peshawar, Pakistan; WHO Collaborating Center for Training and Research in Rehabilitation, Tongji Hospital, Tongji Medical College, Huazhong University of Science and Technology, Wuhan, China},
abstract={Introduction: Exercise is considered a cornerstone in achieving an optimized blood glucose level and reducing body weight, body mass index (BMI), and waist circumference. This study aimed to investigate and quantitatively summarize the literature regarding the therapeutic effects of exercise and general physical activity on glycemic control. Methods: A systematic review and meta-analysis of the literature on the therapeutic effects of exercise on glycemic control in patients with type 2 diabetes mellitus (type 2 DM) were conducted using electronic databases. Studies with an exercise intervention lasting more than 8 weeks were included. Both qualitative and quantitative analyses were performed. Qualitative data were presented narratively in table form. Quantitative analysis was performed using a random-effects model with a 95% confidence interval and a significance level of 0.05. The Physiotherapy Evidence Database (PEDro) scale and the Cochrane Risk of Bias 2 (RoB2) tool were used to assess the quality of evidence and the risk of bias. Results: A total of 21,559 articles were identified through different databases. Out of 21559 studies, only 32 randomized controlled trials were deemed eligible for inclusion in this study. The average exercise session was 45.15 min, while the average follow-up duration was 21.94 weeks. The mean exercise frequency according to our findings was 3.25 days/week. Almost all the studies reported decreases in glycated hemoglobin (HbA1c; P < 0.0001), fasting blood glucose (P = 0.03), BMI (P = 0.04), and waist circumference (P = 0.007) after the exercise intervention. Conclusion: Exercise plays an important role in optimizing glycemic control and improving quality of life (QoL), BMI, and waist circumference in type 2 DM patients. Exercise could be a safe adjunct therapy to medical treatments in these patients. Registration: PROSPERO: CRD42020210816 © 2021, The Author(s).},
author_keywords={Diabetes mellitus, type 2; Exercise; Fasting blood glucose; Glycated hemoglobin; Meta-analysis; Physical activity; Systematic review},
references={Ijaz, S., Ajmal, M., Experiencing type II diabetes in Pakistan (2011) Pak J Social Clin Psychol, 9 (1); Sazlina, S.-G., Browning, C.J., Yasin, S., Promoting physical activity in sedentary elderly Malays with type 2 diabetes: a protocol for randomised controlled trial (2012) BMJ Open, 2 (6); Taylor, J.D., Fletcher, J.P., Tiarks, J., Impact of physical therapist-directed exercise counseling combined with fitness center-based exercise training on muscular strength and exercise capacity in people with type 2 diabetes: a randomized clinical trial (2009) Phys Ther, 89 (9), pp. 884-892; International

Diabetes Federation. IDF Diabetes Atlas, , https://www.diabetesatlas.org, 9th edn. Brussels: IDF; 2019; Short, K.R., Vittone, J.L., Bigelow, M.L., Impact of aerobic exercise training on age-related changes in insulin sensitivity and muscle oxidative capacity (2003) Diabetes, 52 (8), pp. 1888-1896. , COI: 1:CAS:528:DC%2BD3sXlvFCgtr0%3D; Khan, I., King, H., Pakistan National Diabetes Survey prevalence of glucose intolerance and associated factors in North West Frontier Province (NVVFP) of Pakistan (1999) JPMA, 49, p. 206; Rosenbloom, A.L., Joe, J.R., Young, R.S., Winter, W.E., Emerging epidemic of type 2 diabetes in youth (1999) Diabetes Care, 22 (2), pp. 345-354. , COI: 1:STN:280:DyaK1M3ms1Cqug%3D%3D; Iqbal, T., Rashid, F., Saleem, S.A., Shah, S.A., Khalid, G.H., Ishtiaq, O., Awareness about diabetes mellitus amongst diabetics (2013) JRMC, 17 (2), pp. 294-296; Qidwai, W., Ashfaq, T., Imminent epidemic of diabetes mellitus in Pakistan: Issues and challenges for health care providers (2010) JLUMHS, 9 (3), pp. 112-113; Akhter, J., The burden of diabetes in Pakistan: The National Diabetes Survey (1999) JPMA, 49 (9), p. 205; Hakeem, R., Fawwad, A., Diabetes in Pakistan: Epidemiology, determinants and prevention (2010) J Diabetol, 1 (3), p. 3; Anderson, R.M., Funnell, M.M., Arnold, M.S., Barr, P.A., Edwards, G.J., Fitzgerald, J.T., Assessing the cultural relevance of an education program for urban African Americans with diabetes (2000) Diabetes Educ, 26 (2), pp. 280-289; Gabriel, B.M., Zierath, J.R., The limits of exercise physiology: from performance to health (2017) Cell Metab, 25 (5), pp. 1000-1011. , COI: 1:CAS:528:DC%2BC2sXntVKltrg%3D; Sigal, R.J., Kenny, G.P., Wasserman, D.H., Castaneda-Sceppa, C., White, R.D., Physical activity/exercise and type 2 diabetes a consensus statement from the American Diabetes Association (2006) Diabetes Care, 29 (6), pp. 1433-1438; Sigal, R.J., Kenny, G.P., Wasserman, D.H., Castaneda-Sceppa, C., Physical activity/exercise and type 2 diabetes (2004) Diabetes Care, 27 (10), pp. 2518-2539; Sigal, R.J., Kenny, G.P., Boulé, N.G., Effects of aerobic training, resistance training, or both on glycemic control in type 2 diabetes: a randomized trial (2007) Ann Intern Med, 147 (6), pp. 357-369; Moher, D., Liberati, A., Tetzlaffprisma Group, J.D.G., Preferred reporting items for systematic reviews and meta-analyses: The PRISMA statement (2009) Plos Med, 6 (7); Ouzzani, M., Hammady, H., Fedorowicz, Z., Elmagarmid, A., Rayyan—a web and mobile app for systematic reviews (2016) Syst Rev, 5 (1), p. 210; Eng, J., Teasell, R., Miller, W., Spinal cord injury rehabilitation evidence: method of the SCIRE systematic review (2007) Top Spinal Cord Inj Rehabil, 13, pp. 1-10; Higgins, J.P.T., Altman, D.G., Assessing risk of bias in included studies (2008) Cochrane Handbook for Systematic Reviews of Interventions, pp. 187-241. , Higgins JPT, Green S, Chichester, Wiley; (2014) Review Manager, version 5.3. Copenhagen: Cochrane Collaboration; Hozo, S.P., Djulbegovic, B., Hozo, I., Estimating the mean and variance from the median, range, and the size of a sample (2005) BMC Med Res Methodol, 5 (1), p. 13; Leppink, J., O’Sullivan, P., Winston, K., Effect size—large, medium, and small (2016) Perspectives Med Educ, 5 (6), pp. 347-349; Lipsey, M.W., Wilson, D.B., (2001) Practical Meta-Analysis. Thousand Oaks: SAGE; Andrews, R., Cooper, A., Montgomery, A., Diet or diet plus physical activity versus usual care in patients with newly diagnosed type 2 diabetes: the Early ACTID randomised controlled trial (2011) The Lancet, 378 (9786), pp. 129-139. , COI: 1:STN:280:DC%2BC3MnmvVOiuw%3D%3D; Akinci, B., Yeldan, I., Satman, I., Dirican, A., Ozdincler, A.R., The effects of Internet-based exercise compared with supervised group exercise in people with type 2 diabetes: A randomized controlled study (2018) Clin Rehabil, 32 (6), pp. 799-810. , PubMed PMID: 29417832. eng; Dixit, S., Maiya, A., Shastry, B., Effect of moderate-intensity aerobic exercise on glycosylated haemoglobin among elderly patients with type 2 diabetes & peripheral neuropathy (2017) Indian J

Med Res, 145 (1), p. 129. , COI: 1:CAS:528:DC%2BC1MXosVCktA%3D%3D; Dasgupta, K., Rosenberg, E., Joseph, L., Physician step prescription and monitoring to improve ARTERial health (SMARTER): A randomized controlled trial in patients with type 2 diabetes and hypertension (2017) Diabetes Obes Metab, 19 (5), pp. 695-704. , (,):. PubMed PMID; Abdelbasset, W.K., Tantawy, S.A., Kamel, D.M., Effects of high-intensity interval and moderate-intensity continuous aerobic exercise on diabetic obese patients with nonalcoholic fatty liver disease: a comparative randomized controlled trial (2020) Medicine, 99 (10). , (,):,. PubMed PMID:, 32150108; Halvari, H., Healey, J., Olafsen, A.H., Physical activity and motivational predictors of changes in health behavior and health among DM2 and CAD patients (2017) Scand J Med Sci Sports, 27 (11), pp. 1454-1469. , (,):. PubMed PMID:, 27688002; Heiskanen, M.A., Sjöros, T.J., Heinonen, I.H., Sprint interval training decreases left-ventricular glucose uptake compared to moderate-intensity continuous training in subjects with type 2 diabetes or prediabetes (2017) Sci Rep, 7 (1), pp. 1-11. , COI: 1:CAS:528:DC%2BC1cXhtlGkt7rL; Cox, D.J., Taylor, A.G., Singh, H., Glycemic load, exercise, and monitoring blood glucose (GEM): a paradigm shift in the treatment of type 2 diabetes mellitus (2016) Diabetes Res Clin Pract, 111, pp. 28-35. , COI: 1:CAS:528:DC%2BC2MXhslKgsr7F; Sreedevi, A., Gopalakrishnan, U.A., Ramaiyer, S.K., Kamalamma, L., A randomized controlled trial of the effect of yoga and peer support on glycaemic outcomes in women with type 2 diabetes mellitus: A feasibility study (2017) BMC Complement Altern Med, 17 (1); Mensberg, P., Nyby, S., Jørgensen, P.G., Near-normalization of glycaemic control with glucagon-like peptide-1 receptor agonist treatment combined with exercise in patients with type 2 diabetes (2016) Diabetes Obes Metab, 19 (2), pp. 172-180. , (,):. PubMed PMID:, 27717126; Winding, K.M., Munch, G.W., Iepsen, U.W., Van Hall, G., Pedersen, B.K., Mortensen, S.P., The effect on glycaemic control of low-volume high-intensity interval training versus endurance training in individuals with type 2 diabetes (2018) Diabetes Obes Metab, 20 (5), pp. 1131-1139. , (,):. PubMed PMID; Brinkmann, C., Weh-Gray, O., Brixius, K., Bloch, W., Predel, H.G., Kreutz, T., Effects of exercising before breakfast on the health of T2DM patients—a randomized controlled trial (2019) Scand J Med Sci Sports, 29 (12), pp. 1930-1936. , (,):. PubMed PMID:, 31442336; Vanroy, J., Seghers, J., Bogaerts, A., Devloo, K., De Cock, S., Boen, F., Short- and long-term effects of a need-supportive physical activity intervention among patients with type 2 diabetes mellitus: a randomized controlled pilot trial (2017) PLoS One, 12 (4). , (,):,. PubMed PMID:, 28384237; Hangping, Z., Xiaona, Q., Qi, Z., The impact on glycemic control through progressive resistance training with bioDensityTM in Chinese elderly patients with type 2 diabetes: The PReTTy2 (Progressive Resistance Training in Type 2 Diabetes) trial (2019) Diabetes Res Clin Pract, 150, pp. 64-71; Church, T.S., Blair, S.N., Cocreham, S., Effects of aerobic and resistance training on hemoglobin A1c levels in patients with type 2 diabetes: a randomized controlled trial (2010) JAMA, 304 (20), pp. 2253-2262. , COI: 1:CAS:528:DC%2BC3cXhsV2htbbO; Miyamoto, T., Fukuda, K., Oshima, Y., Moritani, T., Non-locomotive physical activity intervention using a tri-axial accelerometer reduces sedentary time in type 2 diabetes (2017) Phys Sportsmed, 45 (3), pp. 245-251. , (,):. PubMed PMID:, 28664755; Bock, B.C., Thind, H., Fava, J.L., Feasibility of yoga as a complementary therapy for patients with type 2 diabetes: the Healthy Active and in Control (HA1C) study (2019) Complement Ther Med, 42, pp. 125-131. , –,. PubMed PMID:, 30670230; Di Loreto, C., Fanelli, C., Lucidi, P., Make your diabetic patients walk: long-term impact of different amounts of physical activity on type 2 diabetes (2005) Diabetes Care, 28 (6), pp. 1295-1302; Earnest, C.P., Johannsen, N.M., Swift, D.L.,

Aerobic and strength training in concomitant metabolic syndrome and type 2 diabetes (2014) Med Sci Sports Exerc, 46 (7), p. 1293. , COI: 1:CAS:528:DC%2BC2cXpvV2ksLw%3D; Alonso-Domínguez, R., García-Ortiz, L., Patino-Alonso, M.C., Effectiveness of a multifactorial intervention in increasing adherence to the Mediterranean diet among patients with diabetes mellitus type 2: a controlled and randomized study (EMID study) (2019) Nutrients, 11 (1), p. 162; Magalhães, J.P., Júdice, P.B., Ribeiro, R., Effectiveness of high-intensity interval training combined with resistance training versus continuous moderate-intensity training combined with resistance training in patients with type 2 diabetes: a one-year randomized controlled trial (2019) Diabetes Obes Metab, 21 (3), pp. 550-559. , (,):. PubMed PMID; Hsieh, P.-L., Tseng, C.-H., Tseng, Y.J., Yang, W.-S., Resistance training improves muscle function and cardiometabolic risks but not quality of life in older people with type 2 diabetes mellitus: a randomized controlled trial (2018) J Geriatr Phys Ther, 41 (2), pp. 65-76; Fayh, A.P.T., Borges, K., Cunha, G.S., Effects of n-3 fatty acids and exercise on oxidative stress parameters in type 2 diabetic: A randomized clinical trial (2018) J Int Soc Sports Nutr, 15 (18); Shakil-Ur-rehman, S., Karimi, H., Gillani, S.A., Amjad, I., Ahmad, S., Yaseen, A., Response to a supervised structured aerobic exercise training program in patients with type 2 diabetes mellitus—does gender make a difference? A randomized controlled clinical trial (2018) J Natl Med Assoc, 110 (5), pp. 431-439; Balducci, S., Zanuso, S., Cardelli, P., Changes in physical fitness predict improvements in modifiable cardiovascular risk factors independently of body weight loss in subjects with type 2 diabetes participating in the Italian Diabetes and Exercise Study (IDES) (2012) Diabetes Care, 35 (6), pp. 1347-1354; Castaneda, C., Layne, J.E., Munoz-Orians, L., A randomized controlled trial of resistance exercise training to improve glycemic control in older adults with type 2 diabetes (2002) Diabetes Care, 25 (12), pp. 2335-2341; Wycherley, T.P., Clifton, P.M., Noakes, M., Brinkworth, G.D., Weight loss on a structured hypocaloric diet with or without exercise improves emotional distress and quality of life in overweight and obese patients with type 2 diabetes (2014) J Diabetes Investig, 5 (1), pp. 94-98. , COI: 1:CAS:528:DC%2BC2cXisleltL4%3D; Francois, M.E., Pistawka, K.J., Halperin, F.A., Little, J.P., Cardiovascular benefits of combined interval training and post-exercise nutrition in type 2 diabetes (2018) J Diabetes Complications, 32 (2), pp. 226-233. , (,):. PubMed PMID:, 29198993; Zhang, L.-Y., Liu, T., Teng, Y.-Q., Effect of a 12-week aerobic exercise training on serum fetuin-A and adipocytokine levels in type 2 diabetes (2018) Exp Clin Endocrinol Diabetes, 126 (8), pp. 487-492. , COI: 1:CAS:528:DC%2BC2sXht1CjsL3F; Cassidy, S., Vaidya, V., Houghton, D., Unsupervised high-intensity interval training improves glycaemic control but not cardiovascular autonomic function in type 2 diabetes patients: a randomised controlled trial (2019) Diab Vasc Dis Res, 16 (1), pp. 69-76. , COI: 1:CAS:528:DC%2BC1MXlvFWkug%3D%3D; Savikj, M., Gabriel, B.M., Alm, P.S., Afternoon exercise is more efficacious than morning exercise at improving blood glucose levels in individuals with type 2 diabetes: a randomised crossover trial (2019) Diabetologia, 62 (2), pp. 233-237. , (,):. PubMed PMID:, 30426166; Hangping, Z., Xiaona, Q., Qi, Z., The impact on glycemic control through progressive resistance training with bioDensity(TM) in Chinese elderly patients with type 2 diabetes: The PReTTy2 (Progressive Resistance Training in Type 2 Diabetes) trial (2019) Diabetes Res Clin Pract, 150, pp. 64-71; Hemmati Maslakpak, M., Razmara, S., Niazkhani, Z., Effects of face-to-face and telephone-based family-oriented education on self-care behavior and patient outcomes in type 2 diabetes: A randomized controlled trial (2017) J Diabetes Res},

correspondence_address1={Chen, H.; Department of Rehabilitation Medicine, China; email: chenhong1129@hotmail.com},
publisher={Adis},
issn={18696953},
language={English},
abbrev_source_title={Diabetes Ther.},
document_type={Article},
source={Scopus},
}

@ARTICLE{DeSouza2021187,
author={De Souza, R.A.S. and Da Silva, A.G. and De Souza, M.F. and Ferreira Souza, L.K. and Roschel, H. and Da Silva, S.F. and Saunders, B.},
title={A systematic review of CrossFit® workouts and dietary and supplementation interventions to guide nutritional strategies and future research in CrossFit®},
journal={International Journal of Sport Nutrition and Exercise Metabolism},
year={2021},
volume={31},
number={2},
pages={187-205},
doi={10.1123/IJSNEM.2020-0223},
note={cited By 0},
url={https://www.scopus.com/inward/record.uri?eid=2-s2.0-85103773234&doi=10.1123%2fIJSNEM.2020-0223&partnerID=40&md5=46e3ccfbb0c271c6e331cbff5d84e7d5},
affiliation={Nutrition Department, University of Lavras, Lavras, Brazil; Graduate Program in Nutrition and Health, University of Lavras, Lavras, Brazil; Applied Physiology and Nutrition Research Group, School of Physical Education and Sport, Rheumatology Division, Faculdade de Medicina FMUSP, University of São Paulo, São Paulo, Brazil; Institute of Orthopaedics and Traumatology, Faculty of Medicine FMUSP, University of São Paulo, São Paulo, Brazil},
abstract={CrossFit® is a high-intensity functional training method consisting of daily workouts called “workouts of the day.” No nutritional recommendations exist for CrossFit® that are supported by scientific evidence regarding the energetic demands of this type of activity or dietary and supplement interventions. This systematic review performed in accordance with PRISMA guidelines aimed to identify studies that determined (a) the physiological and metabolic demands of CrossFit® and (b) the effects of nutritional strategies on CrossFit® performance to guide nutritional recommendations for optimal recovery, adaptations, and performance for CrossFit® athletes and direct future research in this emerging area. Three databases were searched for studies that investigated physiological responses to CrossFit® and dietary or supplementation interventions on CrossFit® performance. Various physiological measures revealed the intense nature of all CrossFit® workouts of the day, reflected in substantial muscle fatigue and damage. Dietary and supplementation studies provided an unclear insight into effective strategies to improve performance and enhance adaptations and recovery due to methodological shortcomings across studies. This systematic review showed that CrossFit® is a high-intensity sport with fairly homogenous anaerobic and aerobic characteristics, resulting in substantial metabolic stress, leading to metabolite accumulation (e.g., lactate and hydrogen ions) and increased markers of muscle damage and muscle fatigue. Limited interventional data exist on dietary and supplementation strategies to optimize CrossFit® performance, and most are moderate to very low quality with some critical methodological

limitations, precluding solid conclusions on their efficacy. High-quality work is needed to confirm the ideal dietary and supplemental strategies for optimal performance and recovery for CrossFit® athletes and is an exciting avenue for further research. © 2021 Human Kinetics, Inc.},
author_keywords={Carbohydrate; Dietary supplements; High-intensity functional training; Nutrition; Workout of the day},
references={Adeva-Andany, M., Lopez-Ojen, M., Funcasta-Calderon, R., Ameneiros-Rodriguez, E., Donapetry-Garcia, C., Vila-Altesor, M., Rodriguez-Seijas, J., Comprehensive review on lactate metabolism in human health (2014) Mitochondrion, 17, pp. 76-100. , PubMed ID: 24929216; Ahmad, A., Jusoh, N., Tengah, R.Y., Acute physiological responses and performance following subsequent CrossFit ‘CINDY’ workout with Zea Mays juice (2019) Physical Education of Students, 23 (2), pp. 57-63; Amir-Behghadami, M., Janati, A., Population, Intervention, Comparison, Outcomes and Study (PICOS) design as a framework to formulate eligibility criteria in systematic reviews (2020) Emergency Medicine Journal, 37 (6), pp. 387-387. , PubMed ID: 32253195; Barfield, J.P., Anderson, A., Effect of CrossFit™ on health-related physical fitness: A pilot study (2014) Journal of Sport and Human Performance, 2 (1), pp. 23-28; Bergeron, M.F., Nindl, B.C., Deuster, P.A., Baumgartner, N., Kane, S.F., Kraemer, W.J., O’Connor, F.G., Consortium for Health and Military Performance and American College of Sports Medicine consensus paper on extreme conditioning programs in military personnel (2011) Current Sports Medicine Reports, 10 (6), pp. 383-389. , PubMed ID: 22071400; Brescansin, B.M., Naziazeno, R.F.T., de Miranda, T.V., Análise do perfil alimentar de praticantes de crossfit na região metropolitana de belém do pará (2019) Revista Brasileira De Nutricao Esportiva, 13 (81), pp. 830-838; Burke, L.M., Practical issues in evidence-based use of performance supplements: Supplement interactions, repeated use and individual responses (2017) Sports Medicine, 47, pp. 79-100. , (Suppl. 1), PubMed ID: 28332111; Burke, L.M., Ketogenic low-CHO, high-fat diet: The future of elite endurance sport? (2020) The Journal of Physiology, , Advance online publication; Burke, L.M., Hawley, J.A., Swifter, higher, stronger: What’s on the menu? (2018) Science, 362 (6416), pp. 781-787. , PubMed ID: 30442803; Butcher, S.J., Neyedly, T.J., Horvey, K.J., Benko, C.R., Do physiological measures predict selected CrossFit® benchmark performance? (2015) Open Access Journal of Sports Medicine, 6, pp. 241-247. , PubMed ID: 26261428; Calverley, T.A., Ogoh, S., Marley, C.J., Steggall, M., Marchi, N., Brassard, P., Bailey, D.M., HIITing the brain with exercise: Mechanisms, consequences and practical recommendations (2020) The Journal of Physiology, 598 (13), pp. 2513-2530. , PubMed ID: 32347544; Carr, A.J., Hopkins, W.G., Gore, C.J., Effects of acute alkalosis and acidosis on performance: A meta-analysis (2011) Sports Medicine, 41 (10), pp. 801-814. , PubMed ID: 21923200; Carreker, J.D., Grosicki, G.J., Physiological predictors of performance on the CrossFit “Murph” challenge (2020) Sports, 8 (7), p. 92; Coco, M., Di Corrado, D., Ramaci, T., Di Nuovo, S., Perciavalle, V., Puglisi, A., Buscemi, A., Role of lactic acid on cognitive functions (2019) The Physician and Sportsmedicine, 47 (3), pp. 329-335. , PubMed ID: 30615538; Coffey, V.G., Hawley, J.A., Concurrent exercise training: Do opposites distract? (2017) The Journal of Physiology, 595 (9), pp. 2883-2896. , PubMed ID: 27506998; Cronin, C.C., O’Neal, E.K., Simpson, J.D., Miller, B.L., Green, M., Allen, J.A.H., Boman, S., Natural training hydration status, sweat rates, and perception of sweat losses during CrossFit training (2016) International Journal of Exercise Science, 9 (5), pp. 576-586; Dantas, T.S.P., Aidar, F.J., de Souza, R.F., de Matos Gama, D., Ferreira, A.R.P., Barros, N.A., da Silva Júnior, W.M., Evaluation of a CrossFit® session on postexercise blood pressure (2018) Journal of Exercise Physiology online, 21

(1), pp. 44-51; de Salles Painelli, V., Alves, V.T., Ugrinowitsch, C., Benatti, F.B., Artioli, G.G., Lancha, A.H., Roschel, H., Creatine supplementation prevents acute strength loss induced by concurrent exercise (2014) European Journal of Applied Physiology, 114 (8), pp. 1749-1755. , PubMed ID: 24840857; Downs, S.H., Black, N., The feasibility of creating a checklist for the assessment of the methodological quality both of randomised and non-randomised studies of health care interventions (1998) Journal of Epidemiology and Community Health, 52 (6), pp. 377-384. , PubMed ID: 9764259; Drake, N., Smeed, J., Carper, M.J., Crawford, D.A., Effects of short-term CrossFitTM training: A magnitude-based approach (2017) Journal of Exercise Physiology Online, 20 (2), pp. 111-133; Drum, S.N., Bellovary, B.N., Jensen, R.L., Moore, M.T., Donath, L., Perceived demands and postexercise physical dysfunction in CrossFit(R) compared to an ACSM based training session (2017) The Journal of Sports Medicine and Physical Fitness, 57 (5), pp. 604-609. , PubMed ID: 26954573; Durkalec-Michalski, K., Nowaczyk, P.M., Siedzik, K., Effect of a four-week ketogenic diet on exercise metabolism in CrossFit-trained athletes (2019) Journal of the International Society of Sports Nutrition, 16 (1), p. 16. , PubMed ID: 30953522; Durkalec-Michalski, K., Zawieja, E.E., Podgorski, T., Loniewski, I., Zawieja, B.E., Warzybok, M., Jeszka, J., The effect of chronic progressive-dose sodium bicarbonate ingestion on CrossFit-like performance: A double-blind, randomized cross-over trial (2018) PLoS One, 13 (5), p. e0197480. , PubMed ID: 29771966; Escobar, K.A., Morales, J., Vandusseldorp, T.A., The effect of a moderately low and high carbohydrate intake on crossfit performance (2016) International Journal of Exercise Science, 9 (4), pp. 460-470; Escobar, K.A., Morales, J., Vandusseldorp, T.A., Metabolic profile of a crossfit training bout (2017) Journal of Human Sport and Exercise, 12 (4), pp. 1248-1255; Fernández-Fernández, J., Solana, R.S., Moya, D., Marin, J.M.S., Ramón, M.M., Acute physiological responses during crossfit® workouts (2015) European Journal of Human Movement, 35, pp. 114-124; Fogaça, L.J., Santos, S.L., Soares, R.C., Gentil, P., Naves, J.P., Dos Santos, W.D., Mota, J.F., Effect of caffeine supplementation on exercise performance, power, markers of muscle damage, and perceived exertion in trained CrossFit men: A randomized, double-blind, placebo-controlled crossover trial (2020) The Journal of Sports Medicine and Physical Fitness, 60 (2), pp. 181-188. , PubMed ID: 31665873; Gallagher, C.N., Carpenter, K.L., Grice, P., Howe, D.J., Mason, A., Timofeev, I., Hutchinson, P.J., The human brain utilizes lactate via the tricarboxylic acid cycle: A 13C-labelled microdialysis and high-resolution nuclear magnetic resonance study (2009) Brain, 132 (10), pp. 2839-2849; Glassman, G., Understanding Crossfit (2007) CrossFit Journal, 56, pp. 1-11; Glassman, G., (2010) The CrossFit training guide, , CrossFit, Inc; Gogojewicz, A., liwicka, E., Durkalec-Michalski, K., Assessment of dietary intake and nutritional status in CrossFit-trained individuals: A descriptive study (2020) International Journal of Environmental Research and Public Health, 17 (13), p. 4772. , S; Gomez-Pinilla, F., Nguyen, T.T., Natural mood foods: The actions of polyphenols against psychiatric and cognitive disorders (2012) Nutritional Neuroscience, 15 (3), pp. 127-133. , PubMed ID: 22334236; Goodwin, M.L., Blood glucose regulation during prolonged, submaximal, continuous exercise: A guide for clinicians (2010) Journal of Diabetes Science and Technology, 4 (3), pp. 694-705. , PubMed ID: 20513337; Gregory, R.M., Hamdan, H., Torisky, D.M., Akers, J.D., A low-carbohydrate ketogenic diet combined with 6-weeks of CrossFit training improves body composition and performance (2017) International Journal of Sports and Exercise Medicine, 3 (2), pp. 1-10; Grgic, J., Grgic, I., Pickering, C., Schoenfeld, B.J., Bishop, D.J., Pedisic, Z., Wake up and smell the coffee: Caffeine supplementation and exercise performance-an

umbrella review of 21 published metaanalyses (2019) British Journal of Sports Medicine, 54 (11), pp. 681-688. , PubMed ID: 30926628; Hak, P.T., Hodzovic, E., Hickey, B., The nature and prevalence of injury during CrossFit training (2013) Journal of Strength and Conditioning Research, , Original investigation: PDF only; Hector, A.J., Phillips, S.M., Protein recommendations for weight loss in elite athletes: A focus on body composition and performance (2018) International Journal of Sport Nutrition and Exercise Metabolism, 28 (2), pp. 170-177. , PubMed ID: 29182451; Heibel, A.B., Perim, P.H.L., Oliveira, L.F., McNaughton, L.R., Saunders, B., Time to optimize supplementation: Modifying factors influencing the individual responses to extracellular buffering agents (2018) Frontiers in Nutrition, 5, p. 35. , PubMed ID: 29868599; Hopkins, B.S., Cloney, M.B., Kesavabhotla, K., Yamaguchi, J., Smith, Z.A., Koski, T.R., Dahdaleh, N.S., Impact of CrossFit-related spinal injuries (2019) Clinical Journal of Sport Medicine, 29 (6), pp. 482-485. , PubMed ID: 31688179; Impey, S.G., Hearris, M.A., Hammond, K.M., Bartlett, J.D., Louis, J., Close, G.L., Morton, J.P., Fuel for the work required: A theoretical framework for carbohydrate periodization and the glycogen threshold hypothesis (2018) Sports Medicine, 48 (5), pp. 1031-1048. , PubMed ID: 29453741; Issurin, V.B., New horizons for the methodology and physiology of training periodization (2010) Sports Medicine, 40 (3), pp. 189-206. , PubMed ID: 20199119; Jeukendrup, A.E., A step towards personalized sports nutrition: carbohydrate intake during exercise (2014) Sports Medicine, 44, pp. 25-33. , (Suppl. 1); Jones, A.M., Dietary nitrate supplementation and exercise performance (2014) Sports Medicine, 44, pp. 35-45. , (Suppl. 1); Kephart, W.C., Pledge, C.D., Roberson, P.A., Mumford, P.W., Romero, M.A., Mobley, C.B., Roberts, M.D., The three-month effects of a ketogenic diet on body composition, blood parameters, and performance metrics in crossfit trainees: A pilot study (2018) Sports, 6 (1), p. 1; Kliszczewicz, B., Quindry, C.J., Blessing, L.D., Oliver, D.G., Esco, R.M., Taylor, J.K., Acute exercise and oxidative stress: CrossFitTM vs. treadmill bout (2015) Journal of Human Kinetics, 47 (1), pp. 81-90; Kliszczewicz, B., Snarr, R.L., Esco, M., Metabolic and cardiovascular response to the CrossFit workout ‘Cindy’: A pilot study (2014) Journal of Sport and Human Performance, 2 (2), pp. 1-9; Kramer, S.J., Baur, D.A., Spicer, M.T., Vukovich, M.D., Ormsbee, M.J., The effect of six days of dietary nitrate supplementation on performance in trained CrossFit athletes (2016) Journal of the International Society of Sports Nutrition, 13 (1), p. 39; Kreider, R.B., Kalman, D.S., Antonio, J., Ziegenfuss, T.N., Wildman, R., Collins, R., Lopez, H.L., International Society of Sports Nutrition position stand: Safety and efficacy of creatine supplementation in exercise, sport, and medicine (2017) Journal of the International Society of Sports Nutrition, 14 (1), p. 18; Macnaughton, L.S., Wardle, S.L., Witard, O.C., McGlory, C., Hamilton, D.L., Jeromson, S., Tipton, K.D., The response of muscle protein synthesis following whole-body resistance exercise is greater following 40 g than 20 g of ingested whey protein (2016) Physiological Reports, 4 (15), p. e12893. , PubMed ID: 27511985; Magistretti, P.J., Allaman, I., Lactate in the brain: From metabolic end-product to signalling molecule (2018) Nature Reviews Neuroscience, 19 (4), pp. 235-249. , PubMed ID: 29515192; Maté-Muñoz, J.L., Lougedo, J.H., Barba, M., Canuelo-Marquez, A.M., Guodemar-Perez, J., Garcia-Fernandez, P., Garnacho-Castano, M.V., Cardiometabolic and muscular fatigue responses to different CrossFit(R) workouts (2018) Journal of Sports Science and Medicine, 17 (4), pp. 668-679. , PubMed ID: 30479537; Maté-Muñoz, J.L., Lougedo, J.H., Barba, M., Garcia-Fernandez, P., Garnacho-Castano, M.V., Dominguez, R., Muscular fatigue in response to different modalities of CrossFit sessions (2017) PLoS One, 12 (7), p. e0181855. , PubMed ID: 28753624; Maughan, R.J.,

Burke, L.M., Dvorak, J., Larson-Meyer, D.E., Peeling, P., Phillips, S.M., Engebretsen, L., IOC consensus statement: Dietary supplements and the high-performance athlete (2018) British Journal of Sports Medicine, 52 (7), pp. 439-455. , PubMed ID: 29540367; Maxwell, C., Ruth, K., Friesen, C., Sports nutrition knowledge, perceptions, resources, and advice given by certified CrossFit trainers (2017) Sports, 5 (2), p. 21; Moher, D., Shamseer, L., Clarke, M., Ghersi, D., Liberati, A., Petticrew, M., Group, P.-P., Preferred reporting items for systematic review and meta-analysis protocols (PRISMA-P) 2015 statement (2015) Systematic Reviews, 4 (1), p. 1; Moran, D.S., Heled, Y., Arbel, Y., Israeli, E., Finestone, A.S., Evans, R.K., Yanovich, R., Dietary intake and stress fractures among elite male combat recruits (2012) Journal of the International Society of Sports Nutrition, 9 (1), p. 6. , PubMed ID: 22413851; Moreira, J.P.A., Mendes, T.P., Costa, A.G.V., Nível de desidratação e concentração de lactato de praticantes de atividade física de alta intensidade (2019) Revista Brasileira De Nutricao Esportiva, 13 (81), pp. 648-656; Moro, T., Badiali, F., Fabbri, I., Paoli, A., Betaine supplementation does not improve muscle hypertrophy or strength following 6 weeks of cross-fit training (2020) Nutrients, 12 (6), p. 1688; Morton, R.W., Murphy, K.T., McKellar, S.R., Schoenfeld, B.J., Henselmans, M., Helms, E., Phillips, S.M., A systematic review, meta-analysis and meta-regression of the effect of protein supplementation on resistance training-induced gains in muscle mass and strength in healthy adults (2018) British Journal of Sports Medicine, 52 (6), pp. 376-384. , PubMed ID: 28698222; Mountjoy, M., Sundgot-Borgen, J.K., Burke, L.M., Ackerman, K.E., Blauwet, C., Constantini, N., Budgett, R., IOC consensus statement on relative energy deficiency in sport (RED-S): 2018 update (2018) British Journal of Sports Medicine, 52 (11), pp. 687-697. , PubMed ID: 29773536; Murawska-Cialowicz, E., Wojna, J., Zuwala-Jagiello, J., Crossfit training changes brain-derived neurotrophic factor and irisin levels at rest, after wingate and progressive tests, and improves aerobic capacity and body composition of young physically active men and women (2015) Journal of Physiology and Pharmacology, 66 (6), pp. 811-821. , PubMed ID: 26769830; Outlaw, J.J., Wilborn, C.D., Smith-Ryan, A.E., Hayward, S.E., Urbina, S.L., Taylor, L.W., Foster, C.A., Effects of a pre-and postworkout protein-carbohydrate supplement in trained crossfit individuals (2014) Springerplus, 3 (1), p. 369; Ouzzani, M., Hammady, H., Fedorowicz, Z., Elmagarmid, A., Rayyan-a web and mobile app for systematic reviews (2016) Systemtic Reviews, 5 (1), p. 210; Pastor, R., Tur, J.A., Antioxidant supplementation and adaptive response to training: A systematic review (2019) Current Pharmaceutical Design, 25 (16), pp. 1889-1912. , PubMed ID: 31267859; Peeling, P., Binnie, M.J., Goods, P.S.R., Sim, M., Burke, L.M., Evidence-based supplements for the enhancement of athletic performance (2018) International Journal of Sport Nutrition and Exercise Metabolism, 28 (2), pp. 178-187. , PubMed ID: 29465269; Perciavalle, V., Marchetta, N.S., Giustiniani, S., Borbone, C., Perciavalle, V., Petralia, M.C., Coco, M., Attentive processes, blood lactate and CrossFit® (2016) The Physician and Sportsmedicine, 44 (4), pp. 403-406. , PubMed ID: 27556548; Rountree, J.A., Krings, B.M., Peterson, T.J., Thigpen, A.G., McAllister, M.J., Holmes, M.E., Smith, J.W., Efficacy of carbohydrate ingestion on crossfit exercise performance (2017) Sports, 5 (3), p. 61; Sadowska-Krepa, E., Domaszewski, P., Pokora, I., Zebrowska, A., Gdanska, A., Podgorski, T., Effects of medium-term green tea extract supplementation combined with CrossFit workout on blood antioxidant status and serum brain-derived neurotrophic factor in young men: A pilot study (2019) Journal of the International Society of Sports Nutrition, 16 (1), p. 13. , PubMed ID: 30898134; Saunders, B., Elliott-Sale, K., Artioli, G.G., Swinton, P.A., Dolan,

E., Roschel, H., Gualano, B., β-alanine supplementation to improve exercise capacity and performance: A systematic review and meta-analysis (2017) British Journal of Sports Medicine, 51 (8), pp. 658-669. , PubMed ID: 27797728; Schwarz, N.A., Blahnik, Z.J., Prahadeeswaran, S., McKinley-Barnard, S.K., Holden, S.L., Waldhelm, A., Epicatechin supplementation inhibits aerobic adaptations to cycling exercise in humans (2018) Frontiers in Nutrition, 5, p. 132. , (-) PubMed ID: 30622947; Schwarz, N.A., Theodore, A.P., Funderburg, B.R., Waldhelm, A., McKinley-Barnard, S.K., Hudson, G.M., Acute (-)-epicatechin consumption: Effects on local vasodilation following resistance exercise and high-intensity exercise performance (2020) Sports, 8 (2), p. 22; Senefeld, J.W., Wiggins, C.C., Regimbal, R.J., Dominelli, P.B., Baker, S.E., Joyner, M.J., Ergogenic effect of nitrate supplementation: A systematic review and meta-analysis (2020) Medicine & Science in Sports & Exercise, 52 (10), pp. 2250-2261; Shaw, S.B., Dullabh, M., Forbes, G., Brandkamp, J.-L., Shaw, I., Analysis of physiological determinants during a single bout of Crossfit (2015) International Journal of Performance Analysis in Sport, 15 (3), pp. 809-815; Sigal, R.J., Kenny, G.P., Wasserman, D.H., Castaneda-Sceppa, C., White, R.D., Physical activity/exercise and type 2 diabetes: A consensus statement from the American Diabetes Association (2006) Diabetes Care, 29 (6), pp. 1433-1438. , PubMed ID: 16732040; Slentz, C.A., Tanner, C.J., Bateman, L.A., Durheim, M.T., Huffman, K.M., Houmard, J.A., Kraus, W.E., Effects of exercise training intensity on pancreatic beta-cell function (2009) Diabetes Care, 32 (10), pp. 1807-1811. , PubMed ID: 19592624; Stein, J.A., Ramirez, M., Heinrich, K.M., Acute caffeine supplementation does not improve performance in trained CrossFit® athletes (2020) Sports, 8 (4), p. 54; Stokes, T., Hector, A.J., Morton, R.W., McGlory, C., Phillips, S.M., Recent perspectives regarding the role of dietary protein for the promotion of muscle hypertrophy with resistance exercise training (2018) Nutrients, 10 (2), p. 180; Swinton, P.A., Hemingway, B.S., Saunders, B., Gualano, B., Dolan, E., A statistical framework to interpret individual response to intervention: Paving the way for personalized nutrition and exercise prescription (2018) Frontiers in Nutrition, 5, p. 41; Thomas, D.T., Erdman, K.A., Burke, L.M., American College of Sports Medicine joint position statement. Nutrition and athletic performance (2016) Medicine & Science in Sports & Exercise, 48 (3), pp. 543-568; Thomas, D.T., Erdman, K.A., Burke, L.M., Position of the Academy of Nutrition and Dietetics, Dietitians of Canada, and the American College of Sports Medicine: Nutrition and athletic performance (2016) Journal of the Academy of Nutrition and Dietetics, 116 (3), pp. 501-528; Tibana, R.A., de Almeida, L.M., Frade de Sousa, N.M., Nascimento Dda, C., Neto, I.V., de Almeida, J.A., Prestes, J., Two consecutive days of crossfit training affects pro and anti-inflammatory cytokines and osteoprotegerin without impairments in muscle power (2016) Frontiers in Physiology, 7, p. 260. , PubMed ID: 27445850; Tibana, R.A., de Sousa, N.M.F., Cunha, G.V., Prestes, J., Fett, C., Gabbett, T.J., Voltarelli, F.A., Validity of session rating perceived exertion method for quantifying internal training load during high-intensity functional training (2018) Sports, 6 (3), p. 68; Tibana, R.A., De Sousa, N.M.F., Prestes, J., Voltarelli, F.A., Lactate, heart rate and rating of perceived exertion responses to shorter and longer duration CrossFit® training sessions (2018) Journal of Functional Morphology and Kinesiology, 3 (4), p. 60; Timón, R., Olcina, G., Camacho-Cardenosa, M., Camacho-Cardenosa, A., Martinez-Guardado, I., Marcos-Serrano, M., 48-hour recovery of biochemical parameters and physical performance after two modalities of CrossFit workouts (2019) Biology of Sport, 36 (3), pp. 283-289. , PubMed ID: 31624423; Watts, M.E., Pocock, R., Claudianos, C., Brain energy and oxygen metabolism: Emerging role in normal function and disease (2018)

Frontiers in Molecular Neuroscience, 11, p. 216. , PubMed ID: 29988368;
Weisenthal, B.M., Beck, C.A., Maloney, M.D., DeHaven, K.E., Giordano, B.D., Injury rate and patterns among CrossFit athletes (2014) Orthopaedic Journal of Sports Medicine, 2 (4), p. 232596711453117},
correspondence_address1={Saunders, B.; Applied Physiology and Nutrition Research Group, Brazil; email: drbryansaunders@outlook.com},
publisher={Human Kinetics Publishers Inc.},
issn={1526484X},
coden={IJSNA},
pubmed_id={33513565},
language={English},
abbrev_source_title={Int. J. Sport Nutr. Exer. Metabol.},
document_type={Article},
source={Scopus},
}

@ARTICLE{Tannous202189,
author={Tannous, H. and Istrate, D. and Perrochon, A. and Daviet, J.-C. and Benlarbi-Delai, A. and Sarrazin, J. and Ho Ba Tho, M.-C. and Dao, T.T.},
title={GAMEREHAB@HOME: A New Engineering System Using Serious Game and Multisensor Fusion for Functional Rehabilitation at Home},
journal={IEEE Transactions on Games},
year={2021},
volume={13},
number={1},
pages={89-98},
doi={10.1109/TG.2019.2963108},
art_number={8946731},
note={cited By 0},
url={https://www.scopus.com/inward/record.uri?eid=2-s2.0-85103192723&doi=10.1109%2fTG.2019.2963108&partnerID=40&md5=b64ae2a4300fce3374e5b82ecc5677de},
affiliation={Université de Technologie de Compiègne, Sorbonne University, Compiègne, France; HAVAE EA 6310, Université de Limoges, Limoges, France; Université Pierre et Marie Curie, L2E, Sorbonne University, Paris, France},
abstract={Biomedical-connected objects, such as kinematic sensors, have been commonly used for patient monitoring in many clinical applications. Moreover, serious games have become widely used to improve patients' motivation during functional rehabilitation. In this article, we develop and evaluate a new engineering system as a solution for functional rehabilitation at home. A multisensor fusion between Kinect camera and inertial sensors was developed to animate a 3-D avatar during rehabilitation and to estimate kinematic data of different joints for clinical monitoring. Two serious game scenarios were designed for upper and lower limb rehabilitation. The developed system was evaluated through patient kinematic data and a questionnaire-based approach with a panel of eight poststroke patients and four clinical experts. The evaluation of the system showed that multisensor fusion provides useful data for clinical follow-up. The virtual game scenarios lead to a high level of immersion for patients. Feedback from clinical experts concerning the system's graphical user interfaces and the clinical relevance of the acquired data for each rehabilitation session are positive. The developed system paves the way to deploy recent technologies, such as multisensor fusion and serious games, as a solution for home-based rehabilitation, which can optimize the benefit of the involved patients and medical experts. © 2018 IEEE.},
author_keywords={Functional rehabilitation; home-based rehabilitation;

multisensor fusion; poststroke patient; real-time biofeedback; serious games},
keywords={Graphical user interfaces; Kinematics; Patient monitoring; Serious games; Surveys; Three dimensional computer graphics, Clinical application; Clinical monitoring; Engineering systems; Inertial sensor; Kinematic sensors; Medical experts; Post stroke patients; Rehabilitation at homes, Patient rehabilitation},
references={Noites, A., Effects of a phase IV home-based cardiac rehabilitation program on cardiorespiratory fitness and physical activity (2017) Heart Lung Circ., 26 (5), pp. 455-462; Rice, D., Janzen, S., McIntyre, A., Vermeer, J., Britt, E., Teasell, R., Comprehensive outpatient rehabilitation program: Hospital-based stroke outpatient rehabilitation (2016) J. Stroke Cerebrovasc. Dis., 25 (5), pp. 1158-1164; Wijkstra, P.J., Van Altena, R., Kraan, J., Otten, V., Postma, D.S., Koeter, G.H., Quality of life in patients with chronic obstructive pulmonary disease improves after rehabilitation at home (1994) Eur. Respir. J., 7 (2), pp. 269-273; Moe, A., Brataas, H.V., Interdisciplinary collaboration experiences in creating an everyday rehabilitation model: A pilot study (2016) J. Multidiscip. Healthcare, 9, pp. 173-182; Capan, N., Effect of an early supervised rehabilitation programme compared with home-based exercise after temporomandibular joint condylar discopexy: A randomized controlled trial (2017) Int. J. Oral Maxillofaial. Surgery, 46 (3), pp. 314-321; Hwang, R., Bruning, J., Morris, N.R., Mandrusiak, A., Russell, T., Home-based telerehabilitation is not inferior to a centre-based program in patients with chronic heart failure: A randomised trial (2017) J. Physiotherapy, 63 (2), pp. 101-107; Holmqvist, L.W., A randomized controlled trial of rehabilitation at home after stroke in Southwest Stockholm (1998) Stroke, 29 (3), pp. 591-597; Thorsen, A.-M., Wides Holmqvist, L., De Pedro-Cuesta, J., Von Koch, L., A randomized controlled trial of early supported discharge and continued rehabilitation at home after stroke: Five-year follow-up of patient outcome (2005) Stroke, 36 (2), pp. 297-303; Perry, J.C., Andureu, J., Cavallaro, F.I., Veneman, J., Carmien, S., Keller, T., Effective game use in neurorehabilitation: User-centered perspectives (2009) Comput. Sci., pp. 1-44; Wattanasoontorn, V., Boada, I., García, R., Sbert, M., Serious games for health (2013) Entertain. Comput., 4 (4), pp. 231-247. , Dec; Seaborn, K., Fels, D.I., Gamification in theory and action: A survey (2015) Int. J. Hum. Comput. Stud., 74, pp. 14-31; Pasch, M., Bianchi-Berthouze, N., Vandijk, B., Nijholt, A., Movementbased sports video games: Investigating motivation and gaming experience (2009) Entertain. Comput., 1 (2), pp. 49-61; González-Ortega, D., Díaz-Pernas, F.J., Martínez-Zarzuela, M., Antón-Rodríguez, M., A Kinect-based system for cognitive rehabilitation exercises monitoring (2014) Comput. Methods Programs Biomed., 113 (2), pp. 620-631; Cherniack, E.P., Not just fun and games: Applications of virtual reality in the identification and rehabilitation of cognitive disorders of the elderly (2011) Disability Rehabil., Assistive Technol., 6 (4), pp. 283-289; Webster, D., Celik, O., Systematic review of Kinect applications in elderly care and stroke rehabilitation (2014) J. Neuroeng.Rehabil., 11; Paraskevopoulos, I.T., Tsekleves, E., Craig, C., Whyatt, C., Cosmas, J., Design guidelines for developing customised serious games for Parkinson's disease rehabilitation using bespoke game sensors (2014) Entertain. Comput., 5 (4), pp. 413-424; McCallum, S., Gamification and serious games for personalized health (2012) Stud. Health Technol. Informat., 177, pp. 85-96; Burke, J.W., McNeill, M.D.J., Charles, D.K., Morrow, P.J., Crosbie, J.H., McDonough, S.M., Optimising engagement for stroke rehabilitation using serious games (2009) Vis. Comput., 25 (12), pp. 1085-1099; Connolly, T.M., Boyle, E.A., Macarthur, E., Hainey, T., Boyle, J.M., A systematic literature review of

empirical evidence on computer games and serious games (2012) Comput. Educ., 59 (2), pp. 661-686; Charsky, D., From edutainment to serious games: A change in the use of game characteristics (2010) Games Culture, 5 (2), pp. 177-198; Idriss, M., Rehabilitation-oriented serious game development and evaluation guidelines for musculoskeletal disorders (2017) JMIR Serious Games, 5 (3), p. e14; Tannous, H., Istrate, D., Ho Ba Tho, M.C., Dao, T.T., Feasibility study of a serious game based on Kinect system for functional rehabilitation of the lower limbs (2016) Eur. Res. Telemed., 5 (2), pp. 97-104. , Jun; Tannous, H., Istrate, D., Ho Ba Tho, M.C., Dao, T.T., Serious game and functional rehabilitation for the lower limbs (2016) Eur. Res. Telemed., 5 (2), pp. 65-69; Jintronix, , jintronix.com; Medimoov, , medimoov.com; SeeMe, , virtual-reality-rehabilitation.com/products/seeme/what-is-seeme; Martins, T., Carvalho, V., Soares, F., Web platform for serious games' management (2015) Procedia Comput. Sci., 64, pp. 1115-1123; Chatzitofis, A., HeartHealth: A cardiovascular disease home-based rehabilitation system (2015) Procedia Comput. Sci., 63, pp. 340-347; Vasconcelos, A., Nunes, F., Carvalho, A., Correia, C., Mobile, exerciseagnostic, sensor-based serious games for physical rehabilitation at home (2018) Proc. 12th Int. Conf. Tangible, Embedded Embodied Interact., pp. 271-278; Jonsdottir, J., Bertoni, R., Lawo, M., Montesano, A., Bowman, T., Gabrielli, S., Serious games for arm rehabilitation of persons with multiple sclerosis.Arandomized controlled pilot study (2018) Multiple Sclerosis Related Disorders., 19, pp. 25-29; Mader, S., Natkin, S., Levieux, G., How to analyse therapeutic games: The player/game/therapy model (2012) Entertainment Computing-ICEC 2012. (Volume 7522, Lecture Notes in Computer Science). Berlin, Germany: Springer, pp. 193-206; Annema, J., Verstraete, M., Vanden Abeele, V., Desmet, S., Geerts, D., Videogames in therapy:Atherapist's perspective (2010) Proc. Int. Conf. Fun Games, pp. 94-98; Tannous, H., Anewmulti-sensor fusion scheme to improve the accuracy of knee flexion kinematics for functional rehabilitation movements (2016) Sensors, 16 (11); Bohannon, R.W., Clinical measurement of range of motion review of goniometry emphasizing reliability and validity (1987), 67 (12), pp. 1867-1872; Brosseau, L., Intratester and intertester reliability and criterion validity of the parallelogram and universal goniometers for active knee flexion in healthy subjects (1997) Physiotherapy Res. Int., 2 (3), pp. 150-166. , Aug; Jennett, C., Measuring and defining the experience of immersion in games (2008) Int. J. Hum. Comput. Stud., 66 (9), pp. 641-661; Lewis, J.R., IBM computer usability satisfaction questionnaires: Psychometric evaluation and instructions for use IBM Computer Usability (1995) Int. J. Human-Comput. Interact., 7 (1), pp. 57-78; Fierro, A., Musical rhythm: How musical rhythm in a serious game can increase the immersion and how the immersion can encourage the rehabilitation process (2012) Master's Thesis, Univ. Skövde, Skövde, Sweden, p. 36; Iacovides, I., Cox, A., Kennedy, R., Cairns, P., Jennett, C., Removing the HUD: The impact of non-diegetic game elements and expertise on player involvement (2015) Proc. Annu. Symp. Comput. Interact. Play, pp. 13-22; Ling, Y., Ter Meer, L.P., Yumak, Z., Veltkamp, R.C., Usability test of exercise games designed for rehabilitation of elderly patients after hip replacement surgery: Pilot study (2017) JMIR Serious Games, 5 (4), p. e19; Kao, H.Y., Cheng, Y.T., Chien, Y.K., Develop and evaluate the mobile-based self-management application for tele-healthcare service (2014) Modern Advances in Applied Intelligence, (Volume 8481, Lecture Notes in Computer Science). Cham, Switzerland: Springer, pp. 460-469; Hu, P.J., Chau, P.Y.K., Sheng, O.R.L., Tam, K.Y., Examining the technology acceptance model using physician acceptance of telemedicine technology (1999) J. Manag. Inf. Syst., 16 (2), pp. 91-112; Dao, T.T., Tannous, H., Pouletaut, P., Gamet, D., Istrate, D., Ho Ba Tho, M.C., Interactive and connected rehabilitation systems

for E-health (2016) IRBM, 37 (5-6), pp. 289-296},
correspondence_address1={Dao, T.T.; Université de Technologie de Compiègne, France; email: tien-tuan.dao@utc.fr},
publisher={Institute of Electrical and Electronics Engineers Inc.},
issn={24751502},
language={English},
abbrev_source_title={IEEE Trans. Games},
document_type={Article},
source={Scopus},
}

@ARTICLE{Neunhäuserer2021e171,
author={Neunhäuserer, D. and Patti, A. and Niederseer, D. and Kaiser, B. and Cadamuro, J. and Lamprecht, B. and Ermolao, A. and Studnicka, M. and Niebauer, J.},
title={Systemic Inflammation, Vascular Function, and Endothelial Progenitor Cells after an Exercise Training Intervention in COPD},
journal={American Journal of Medicine},
year={2021},
volume={134},
number={3},
pages={e171-e180},
doi={10.1016/j.amjmed.2020.07.004},
note={cited By 1},
url={https://www.scopus.com/inward/record.uri?eid=2-s2.0-85091254147&doi=10.1016%2fj.amjmed.2020.07.004&partnerID=40&md5=0ed82520deaed24fcc767a2ba83c396c},
affiliation={University Institute of Sports Medicine, Prevention and Rehabilitation, Paracelsus Medical University of Salzburg, Austria; Research Institute for Molecular Sports Medicine and Rehabilitation, Paracelsus Medical University of Salzburg, Austria; Sport and Exercise Medicine Division, Department of Medicine, University of Padova, Italy; Division of Cardiology, University Heart Centre, University Hospital Zurich, Switzerland; University Clinic of Pneumology, Paracelsus Medical University of Salzburg, Austria; Department of Laboratory Medicine, Paracelsus Medical University of Salzburg, Austria; Department of Pulmonary Medicine, Faculty of Medicine, Kepler-University-Hospital, Johannes-Kepler-University, Linz, Austria},
abstract={Background: Exercise training is a cornerstone of the treatment of chronic obstructive pulmonary disease (COPD) in all disease stages. Data about the training effects with supplemental oxygen in nonhypoxemic patients remains inconclusive. In this study we set out to investigate the training and oxygen effects on inflammatory markers, vascular function, and endothelial progenitor cells in this population of increased cardiovascular risk. Methods: In this prospective, randomized, double-blind, crossover study, 29 patients with nonhypoxemic COPD performed combined endurance and strength training 3 times a week while breathing medical air or supplemental oxygen for the first 6-week period, and were then reallocated to the opposite gas for the following 6 weeks. Exercise capacity, inflammatory biomarkers, endothelial function (peripheral arterial tone analysis), and endothelial progenitor cells were assessed. Data were also analyzed for a subgroup with endothelial dysfunction (reactive hyperemia index <1.67). Results: Following 12 weeks of exercise training, patients demonstrated a significant improvement of peak work rate and an associated decrease of blood fibrinogen and leptin. Eosinophils were found significantly reduced after exercise training in patients with endothelial dysfunction. In this subgroup, peripheral arterial tone analysis revealed a

significant improvement of reactive hyperemia index. Generally, late endothelial progenitor cells were found significantly reduced after the exercise training intervention. Supplemental oxygen during training positively influenced the effect on exercise capacity without impact on inflammation and endothelial function. Conclusions: This is the first randomized controlled trial in patients with COPD to show beneficial effects of exercise training not only on exercise capacity, but also on systemic/eosinophilic inflammation and endothelial dysfunction. © 2020 Elsevier Inc.},
author_keywords={Chronic obstructive pulmonary disease; Endothelial dysfunction; Eosinophilic inflammation; EPC; Supplemental oxygen},
keywords={aged; blood; chronic obstructive lung disease; complication; controlled study; crossover procedure; double blind procedure; endothelial progenitor cell; female; human; inflammation; kinesiotherapy; male; middle aged; pathophysiology; prospective study; randomized controlled trial; vascular endothelium, Aged; Cross-Over Studies; Double-Blind Method; Endothelial Progenitor Cells; Endothelium, Vascular; Exercise Therapy; Female; Humans; Inflammation; Male; Middle Aged; Prospective Studies; Pulmonary Disease, Chronic Obstructive},
references={Chen, W., Thomas, J., Sadatsafavi, M., FitzGerald, J.M., Risk of cardiovascular comorbidity in patients with chronic obstructive pulmonary disease: a systematic review and meta-analysis (2015) Lancet Respir Med, 3 (8), pp. 631-639; Sin, D.D., Paul Man, S.F., Why are patients with chronic obstructive pulmonary disease at increased risk of cardiovascular diseases? The potential role of systemic inflammation in chronic obstructive pulmonary disease (2003) Circulation, 107 (11), pp. 1514-1519; Wedzicha, J.A., Seemungal, T.A.R., MacCallum, P.K., Acute exacerbations of chronic obstructive pulmonary disease are accompanied by elevations of plasma fibrinogen and serum IL-6 levels (2000) Thromb Haemost, 84 (2), pp. 210-215; Eickhoff, P., Valipour, A., Kiss, D., Determinants of systemic vascular function in patients with stable chronic obstructive pulmonary disease (2008) Am J Respir Crit Care Med, 178 (12), pp. 1211-1218; McDonald, C.F., Eosinophil biology in COPD (2017) N Engl J Med, 377 (17), pp. 1680-1682; Tashkin, D.P., Wechsler, M.E., Role of eosinophils in airway inflammation of chronic obstructive pulmonary disease (2018) Int J Chron Obstruct Pulmon Dis, 13, pp. 335-349; Hastie, A.T., Martinez, F.J., Curtis, J.L., Association of sputum and blood eosinophil concentrations with clinical measures of COPD severity: an analysis of the SPIROMICS cohort (2017) Lancet Respir Med, 5 (12), pp. 956-967; Sabit, R., Bolton, C.E., Edwards, P.H., Arterial stiffness and osteoporosis in chronic obstructive pulmonary disease (2007) Am J Respir Crit Care Med, 175 (12), pp. 1259-1265; Maclay, J.D., McAllister, D.A., Mills, N.L., Vascular dysfunction in chronic obstructive pulmonary disease (2009) Am J Respir Crit Care Med, 180 (6), pp. 513-520; Hamburg, N.M., Benjamin, E.J., Assessment of endothelial function using digital pulse amplitude tonometry (2009) Trends Cardiovasc Med, 19 (1), pp. 6-11; Bernardi, E., Merlo, C., Cogo, A., Endothelial function in COPD is in an intermediate position between healthy subjects and coronary artery disease patients and is related to physical activity (2018) Lung, 196 (6), pp. 669-672; MacNee, W., Maclay, J., McAllister, D., Cardiovascular injury and repair in chronic obstructive pulmonary disease (2008) Proc Am Thorac Soc, 5 (8), pp. 824-833; Koutroumpi, M., Dimopoulos, S., Psarra, K., Kyprianou, T., Nanas, S., Circulating endothelial and progenitor cells: Evidence from acute and long-term exercise effects (2012) World J Cardiol, 4 (12), pp. 312-326; Peinado, V.I., Ramírez, J., Roca, J., Rodriguez-Roisin, R., Barberà, J.A., Identification of vascular progenitor cells in pulmonary arteries of patients with chronic obstructive pulmonary disease (2006) Am J Respir Cell Mol Biol, 34

(3), pp. 257-263; Salter, B.M., Manzoor, F., Beaudin, S., Dysregulation of vascular endothelial progenitor cells lung-homing in subjects with COPD (2016) Can Respir J, 2016; Huertas, A., Palange, P., Circulating endothelial progenitor cells and chronic pulmonary diseases (2011) Eur Respir J, 37 (2), pp. 426-431; Palange, P., Testa, U., Huertas, A., Circulating haemopoietic and endothelial progenitor cells are decreased in COPD (2006) Eur Respir J, 27 (3), pp. 529-541; Clarenbach, C.F., Senn, O., Sievi, N.A., Determinants of endothelial function in patients with COPD (2013) Eur Respir J, 42 (5), pp. 1194-1204; Sievi, N.A., Franzen, D., Kohler, M., Clarenbach, C.F., Physical inactivity and arterial stiffness in COPD (2015) Int J Chron Obstruct Pulmon Dis, 10 (1), pp. 1891-1897; Adams, V., Reich, B., Uhlemann, M., Niebauer, J., Molecular effects of exercise training in patients with cardiovascular disease: focus on skeletal muscle, endothelium, and myocardium (2017) Am J Physiol Heart Circ Physiol, 313 (1), pp. H72-H88; Pucci, G., Battista, F., Schillaci, G., Aerobic physical exercise and arterial de-stiffening: a recipe for vascular rejuvenation (2012) Hypertens Res, 35 (10), pp. 964-966; Myers, J., Prakash, M., Froelicher, V., Partington, S., Atwood, E., Exercise capacity and mortality among men referred for exercise testing (2002) N Engl J Med, 346 (11), pp. 793-801; McCarthy, B., Casey, D., Devane, D., Murphy, K., Murphy, E., Lacasse, Y., Pulmonary rehabilitation for chronic obstructive pulmonary disease (2015) Cochrane Database Syst Rev, 23 (2); Spruit, M.A., Singh, S.J., Garvey, C., An official American thoracic society/European respiratory society statement: key concepts and advances in pulmonary rehabilitation (2013) Am J Respir Crit Care Med, 188 (8), pp. e13-e64; Vestbo, J., Hurd, S.S., Agustí, A.G., Global strategy for the diagnosis, management, and prevention of chronic obstructive pulmonary disease GOLD executive summary (2013) Am J Respir Crit Care Med, 187 (4), pp. 347-365; Waschki, B., Kirsten, A., Holz, O., Physical activity is the strongest predictor of all-cause mortality in patients with COPD (2011) Chest, 140 (2), pp. 331-342; Watz, H., Pitta, F., Rochester, C.L., An official European respiratory society statement on physical activity in COPD (2014) Eur Respir J, 44 (6), pp. 1521-1537; Singh, D., Agusti, A., Anzueto, A., Global strategy for the diagnosis, management, and prevention of chronic obstructive lung disease: the GOLD science committee report 2019 (2019) Eur Respir J, 53 (5); Neunhäuserer, D., Steidle-Kloc, E., Weiss, G., Supplemental oxygen during high-intensity exercise training in nonhypoxemic chronic obstructive pulmonary disease (2016) Am J Med, 129 (11), pp. 1185-1193; Neunhäuserer, D., Steidle-kloc, E., Bergamin, M., Role of breathing conditions during exercise testing on training prescription in chronic obstructive pulmonary disease (2017) Am J Phys Med Rehabil, 96 (12), pp. 908-911; Karvonen, M., Kentala, E., Mustala, O., The effects of training heart rate: a longitudinal study (1957) Ann Med Exp Biol Fenn, 35 (3), pp. 307-315; Niederseer, D., Steidle-Kloc, E., Mayr, M., Effects of a 12-week alpine skiing intervention on endothelial progenitor cells, peripheral arterial tone and endothelial biomarkers in the elderly (2016) Int J Cardiol, 214, pp. 343-347; Del Bo, C., Deon, V., Porrini, M., Campolo, J., Parolini, M., Riso, P., Intra- and interday repeatability of peripheral arterial function: suitability and potential limitations (2016) Microcirculation, 23 (7), pp. 503-511; McCrea, C.E., Skulas-Ray, A.C., Chow, M., West, S.G., Test-retest reliability of pulse amplitude tonometry measures of vascular endothelial function: implications for clinical trial design (2012) Vasc Med, 17 (1), pp. 29-36; Bonetti, P.O., Attenuation of digital reactive hyperemia in patients with early and advanced coronary artery disease (2005) J Am Coll Cardiol, 45 (3), p. 407A. , (suppl); Hills, M., Armitage, P., The two-period cross-over clinical trial (1979) Br J Clin Pharmacol, 8 (1), pp. 7-20; Schumacher, M., Schulgen, G., Methodik Klinischer Studien: Methodische

Grundlagen Der Planung, Durchführung Und Auswertung (2008), 3rd ed Springer Berlin, Heidelberg; Somfay, A., Porszasz, J., Lee, S., Casaburi, R., Effect of hyperoxia on gas exchange and lactate kinetics following exercise onset in nonhypoxemic COPD patients (2002) Chest, 121 (2), pp. 393-400; Fujimoto, K., Matsuzawa, Y., Yamaguchi, S., Koizumi, T., Kubo, K., Benefits of oxygen on exercise performance and pulmonary hemodynamics in patients with COPD with mild hypoxemia (2002) Chest, 122 (2), pp. 457-463; Lin, X., Zhang, X., Guo, J., Effects of exercise training on cardiorespiratory fitness and biomarkers of cardiometabolic health: A systematic review and meta-analysis of randomized controlled trials (2015) J Am Heart Assoc, 4 (7), pp. 1-29; Pizarro, S., García-Lucio, J., Peinado, V.I., Circulating progenitor cells and vascular dysfunction in chronic obstructive pulmonary disease (2014) PLoS One, 9 (8); França-Pinto, A., Mendes, F.A.R., De Carvalho-Pinto, R.M., Aerobic training decreases bronchial hyperresponsiveness and systemic inflammation in patients with moderate or severe asthma: a randomised controlled trial (2015) Thorax, 70 (8), pp. 732-739; Almeida-Oliveira, A.R., Aquino-Junior, J., Abbasi, A., Effects of aerobic exercise on molecular aspects of asthma: involvement of SOCS-JAK-STAT (2019) Exerc Immunol Rev, 25 (5), pp. 50-62; do Nascimento, E.S., Sampaio, L.M., Peixoto-Souza, F.S., Home-based pulmonary rehabilitation improves clinical features and systemic inflammation in chronic obstructive pulmonary disease patients (2015) Int J Chron Obstruct Pulmon Dis, 10 (1), pp. 645-653; Abd El-Kader, S.M., Al-Jiffri, O.H., Al-Shreef, F.M., Plasma inflammatory biomarkers response to aerobic versus resisted exercise training for chronic obstructive pulmonary disease patients (2016) Afr Health Sci, 16 (2), pp. 507-515; Wang, C.H., Chou, P.C., Joa, W.C., Mobile-phone-based home exercise training program decreases systemic inflammation in COPD: a pilot study (2014) BMC Pulm Med, 14 (1), pp. 1-9; Canavan, J., Garrod, R., Marshall, J., Jackson, D., Ansley, P., Jewell, A., Measurement of the acute inflammatory response to walking exercise in COPD: effects of pulmonary rehabilitation (2007) Int J Chron Obs Pulmon Dis, 2 (3), pp. 347-353; White, J., Dawson, B., Landers, G., Croft, K., Peeling, P., Effect of supplemental oxygen on post-exercise inflammatory response and oxidative stress (2013) Eur J Appl Physiol, 113 (4), pp. 1059-1067; Vivodtzev, I., Tamisier, R., Baguet, J.P., Borel, J.C., Levy, P., Pépin, J.L., Arterial stiffness in COPD (2014) Chest, 145 (4), pp. 861-875; Luk, T.H., Dai, Y.L., Siu, C.W., Effect of exercise training on vascular endothelial function in patients with stable coronary artery disease: A randomized controlled trial (2012) Eur J Prev Cardiol, 19 (4), pp. 830-839; Swift, D.L., Earnest, C.P., Blair, S.N., Church, T.S., The effect of different doses of aerobic exercise training on endothelial function in postmenopausal women with elevated blood pressure: results from the DREW study (2012) Br J Sports Med, 46 (10), pp. 753-758; Kim, C., Choi, H.E., Jung, H., Kang, S.H., Kim, J.H., Byun, Y.S., Impact of aerobic exercise training on endothelial function in acute coronary syndrome (2014) Ann Rehabil Med, 38 (3), pp. 388-395; Cornelissen, A., Onkelinx, S., Goetschalckx, K., Exercise-based cardiac rehabilitation improves endothelial function assessed by flow-mediated dilation but not by pulse amplitude tonometry (2014) Eur J Prev Cardiol, 21 (1), pp. 39-48; Katayama, K., Fujita, O., Iemitsu, M., The effect of acute exercise in hypoxia on flow-mediated vasodilation (2013) Eur J Appl Physiol, 113 (2), pp. 349-357; Vanfleteren, L.E.G.W., Spruit, M.A., Groenen, M.T.J., Arterial stiffness in patients with COPD: the role of systemic inflammation and the effects of pulmonary rehabilitation (2014) Eur Respir J, 43 (5), pp. 1306-1315; Smith, M.M., Buffington, C.A., Hamlin, R.L., Devor, S.T., Relationship between muscle sympathetic nerve activity and aortic wave reflection characteristics in aerobic- and resistance-trained subjects (2015)

Eur J Appl Physiol, 115 (12), pp. 2609-2619; Cortez-Cooper, M.Y., DeVan, A.E., Anton, M.M., Effects of high intensity resistance training on arterial stiffness and wave reflection in women (2005) Am J Hypertens, 18 (7), pp. 930-934; Heffernan, K.S., Patvardhan, E.A., Hession, M., Ruan, J., Karas, R.H., Kuvin, J.T., Elevated augmentation index derived from peripheral arterial tonometry is associated with abnormal ventricular-vascular coupling (2010) Clin Physiol Funct Imaging, 30 (5), pp. 313-317; Rakobowchuk, M., Harris, E., Taylor, A., Heavy and moderate interval exercise training alters low-flow-mediated constriction but does not increase circulating progenitor cells in healthy humans (2012) Exp Physiol, 97 (3), pp. 375-385; Mayr, M., Niederseer, D., Niebauer, J., From bench to bedside: what physicians need to know about endothelial progenitor cells (2011) Am J Med, 124 (6), pp. 489-497; Liu, Z.-J., Velazquez, O.C., Hyperoxia, endothelial progenitor cell mobilization, and diabetic wound healing (2008) Antioxid Redox Signal, 10 (11), pp. 1869-1882},
correspondence_address1={Niebauer, J.; University Institute of Sports Medicine, Lindhofstrasse 20, Austria; email: j.niebauer@salk.at},
publisher={Elsevier Inc.},
issn={00029343},
coden={AJMEA},
pubmed_id={32781050},
language={English},
abbrev_source_title={Am. J. Med.},
document_type={Article},
source={Scopus},
}

@ARTICLE{Masarwa2021,
author={Masarwa, R. and Brunetti, V.C. and Aloe, S. and Henderson, M. and Platt, R.W. and Filion, K.B.},
title={Efficacy and safety of metformin for obesity: A systematic review},
journal={Pediatrics},
year={2021},
volume={147},
number={3},
doi={10.1542/PEDS.2020-1610},
art_number={e20201610},
note={cited By 1},
url={https://www.scopus.com/inward/record.uri?eid=2-s2.0-85102536672&doi=10.1542%2fPEDS.2020-1610&partnerID=40&md5=3e026265845f756e26e11b7f4fbda347},
affiliation={Center for Clinical Epidemiology, Lady Davis Institute, Jewish General Hospital, Montreal, QC, Canada; Department of Pediatrics, McGill University, Montreal, QC, Canada; Department of Medicine, McGill University, Montreal, QC, Canada; Department of Epidemiology, Biostatistics, and Occupational Health, McGill University, Montreal, QC, Canada; Division of Endocrinology, Jewish General Hospital, McGill University, Montreal, QC, Canada; Department of Pediatrics, Centre Hospitalier Universitaire Sainte-Justine, University of Montreal, Montreal, QC, Canada; Centre Hospitalier Universitaire Sainte-Justine Research Center, Montreal, QC, Canada},
abstract={CONTEXT: The efficacy and safety of metformin for obesity in children and adolescents remains unclear. OBJECTIVE: To assess the efficacy and safety of metformin via systematic review. DATA SOURCES: Data sources included PubMed, Embase, the Cochrane Library, Scopus, and ClincalTrials.gov (inception to

November 2019). STUDY SELECTION: We selected randomized controlled trials (RCTs) in which researchers assessed the efficacy and safety of metformin with lifestyle interventions, compared with a placebo with lifestyle interventions, in children and adolescents with obesity. DATA EXTRACTION: Two researchers independently extracted data and assessed quality. The primary outcomes were mean changes from baseline in BMI, BMI z score, homeostatic model assessment of insulin resistance, and gastrointestinal adverse effects. RESULTS: Twenty-four RCTs (1623 patients; range: 16 to 151) were included. Ages ranged from 4 to 19 years, and follow-up ranged from 2 months to 2 years. Metformin resulted in a modest decrease in BMI (range of mean values: 22.70 to 1.30 vs 21.12 to 1.90), BMI z score (range of mean values: 20.37 to 20.03 vs 20.22 to 0.15), and homeostatic model assessment of insulin resistance (range of mean values: 23.74 to 1.00 vs 21.40 to 2.66). Metformin resulted in a higher frequency of gastrointestinal adverse effects (range: 2% to 74% vs 0% to 42%). LIMITATIONS: The available evidence is of varying quality, with high heterogeneity between trials, suggesting some uncertainty in the benefits of metformin in this population. CONCLUSIONS: With this systematic review of RCTs, we suggest that metformin has modest but favorable effects on weight and insulin resistance and a tolerable safety profile among children and adolescents with obesity. © 2021 American Academy of Pediatrics. All rights reserved.},
references={de Onis, M, Blössner, M, Borghi, E., Global prevalence and trends of overweight and obesity among preschool children (2010) Am J Clin Nutr, 92 (5), pp. 1257-1264; Barlow, SE, Expert committee recommendations regarding the prevention, assessment, and treatment of Expert Committee; child and adolescent overweight and obesity: summary report (2007) Pediatrics, 120, pp. S164-S192. , (suppl 4); Ogden, CL, Carroll, MD, Lawman, HG, Trends in obesity prevalence among children and adolescents in the United States, 1988-1994 through 2013-2014 (2016) JAMA, 315 (21), pp. 2292-2299; Skinner, AC, Ravanbakht, SN, Skelton, JA, Perrin, EM, Armstrong, SC., Prevalence of obesity and severe obesity in US children, 1999-2016. [published correction appears in Pediatrics. 2018; 142(3):e20181916] (2018) Pediatrics, 141 (3), p. e20173459; Caprio, S., Insulin resistance in childhood obesity (2002) J Pediatr Endocrinol Metab, 15, pp. 487-492. , (suppl 1); Berenson, GS, Srinivasan, SR, Bao, W, Newman, WP, Tracy, RE, Wattigney, WA., Association between multiple cardiovascular risk factors and atherosclerosis in children and young adults. The Bogalusa Heart Study (1998) N Engl J Med, 338 (23), pp. 1650-1656; Must, A, Jacques, PF, Dallal, GE, Bajema, CJ, Dietz, WH., Long-term morbidity and mortality of overweight adolescents. A follow-up of the Harvard Growth Study of 1922 to 1935 (1992) N Engl J Med, 327 (19), pp. 1350-1355; Boland, CL, Harris, JB, Harris, KB., Pharmacological management of obesity in pediatric patients (2015) Ann Pharmacother, 49 (2), pp. 220-232; Steinberger, J, Daniels, SR, Obesity, insulin resistance, diabetes, and cardiovascular risk in children: an American Heart Association scientific statement from the Atherosclerosis, Hypertension, and Obesity in the Young Committee (Council on Cardiovascular Disease in the Young) and the Diabetes Committee (Council on Nutrition, Physical Activity, and Metabolism) (2003) Circulation, 107 (10), pp. 1448-1453. , American Heart Association Atherosclerosis, Hypertension, and Obesity in the Young Committee (Council on Cardiovascular Disease in the Young); American Heart Association Diabetes Committee (Council on Nutrition, Physical Activity, and Metabolism); (2017) GLUCOPHAGE (metformin hydrochloride) tablets, , Bristol-Myers Squibb Company. Available;
https://www.accessdata.fda.gov/drugsatfda_docs/label/2017/020357s037s039/021202s021s023lbl.pdf, at: Accessed April 4, 2019; Type 2 diabetes in children and adolescents (2000) Diabetes Care, 23 (3), pp. 381-389. , American Diabetes

Association; Evia-Viscarra, ML, Rodea-Montero, ER, Apolinar-Jiménez, E, The effects of metformin on inflammatory mediators in obese adolescents with insulin resistance: controlled randomized clinical trial (2012) J Pediatr Endocrinol Metab, 25 (1-2), pp. 41-49; Gómez-Díaz, RA, Talavera, JO, Pool, EC, Metformin decreases plasma resistin concentrations in pediatric patients with impaired glucose tolerance: a placebo-controlled randomized clinical trial (2012) Metabolism, 61 (9), pp. 1247-1255; Kendall, D, Vail, A, Amin, R, Metformin in obese children and adolescents: the MOCA trial (2013) J Clin Endocrinol Metab, 98 (1), pp. 322-329; Burgert, TS, Duran, EJ, Goldberg-Gell, R, Short-term metabolic and cardiovascular effects of metformin in markedly obese adolescents with normal glucose tolerance (2008) Pediatr Diabetes, 9 (6), pp. 567-576; Harden, KA, Cowan, PA, Velasquez-Mieyer, P, Patton, SB., Effects of lifestyle intervention and metformin on weight management and markers of metabolic syndrome in obese adolescents (2007) J Am Acad Nurse Pract, 19 (7), pp. 368-377; Atabek, ME, Pirgon, O., Use of metformin in obese adolescents with hyperinsulinemia: a 6-month, randomized, double-blind, placebo-controlled clinical trial (2008) J Pediatr Endocrinol Metab, 21 (4), pp. 339-348; Luong, DQ, Oster, R, Ashraf, AP., Metformin treatment improves weight and dyslipidemia in children with metabolic syndrome (2015) J Pediatr Endocrinol Metab, 28 (5-6), pp. 649-655; Rynders, C, Weltman, A, Delgiorno, C, Lifestyle intervention improves fitness independent of metformin in obese adolescents (2012) Med Sci Sports Exerc, 44 (5), pp. 786-792; Moher, D, Liberati, A, Tetzlaff, J, Altman, DG, Preferred reporting items for systematic reviews and meta-analyses: the PRISMA statement (2009) BMJ, 339, p. b2535. , PRISMA Group; Stroup, DF, Berlin, JA, Morton, SC, Meta-analysis of observational studies in epidemiology: a proposal for reporting. Meta-analysis Of Observational Studies in Epidemiology (MOOSE) group (2000) JAMA, 283 (15), pp. 2008-2012; Ouzzani, M, Hammady, H, Fedorowicz, Z, Elmagarmid, A., Rayyan-a web and mobile app for systematic reviews (2016) Syst Rev, 5 (1), p. 210; Higgins, JP, Altman, DG, Gøtzsche, PC, The Cochrane Collaboration's tool for assessing risk of bias in randomised trials (2011) BMJ, 343, p. d5928. , Cochrane Bias Methods Group; Cochrane Statistical Methods Group; Hozo, SP, Djulbegovic, B, Hozo, I., Estimating the mean and variance from the median, range, and the size of a sample (2005) BMC Med Res Methodol, 5, p. 13; Freemark, M., Liver dysfunction in paediatric obesity: a randomized, controlled trial of metformin (2007) Acta Paediatr, 96 (9), pp. 1326-1332; Freemark, M, Bursey, D., The effects of metformin on body mass index and glucose tolerance in obese adolescents with fasting hyperinsulinemia and a family history of type 2 diabetes (2001) Pediatrics, 107 (4). , www.pediatrics.org/cgi/content/full/107/4/e55; Clarson, CL, Brown, HK, De Jesus, S, Effects of a comprehensive, intensive lifestyle intervention combined with metformin extended release in obese adolescents (2014) Int Sch Res Notices, 2014, p. 659410; Clarson, CL, Mahmud, FH, Baker, JE, Metformin in combination with structured lifestyle intervention improved body mass index in obese adolescents, but did not improve insulin resistance (2009) Endocrine, 36 (1), pp. 141-146; Garibay-Nieto, N, Queipo-García, G, Alvarez, F, Effects of conjugated linoleic acid and metformin on insulin sensitivity in obese children: randomized clinical trial (2017) J Clin Endocrinol Metab, 102 (1), pp. 132-140; Mauras, N, DelGiorno, C, Hossain, J, Metformin use in children with obesity and normal glucose tolerance-effects on cardiovascular markers and intrahepatic fat (2012) J Pediatr Endocrinol Metab, 25 (1-2), pp. 33-40; Rezvanian, H, Hashemipour, M, Kelishadi, R, Tavakoli, N, Poursafa, P., A randomized, triple masked, placebo-controlled clinical trial for controlling childhood obesity (2010) World J Pediatr, 6 (4), pp. 317-322; Srinivasan, S, Ambler, GR, Baur, LA, Randomized, controlled trial of metformin

for obesity and insulin resistance in children and adolescents: improvement in body composition and fasting insulin (2006) J Clin Endocrinol Metab, 91 (6), pp. 2074-2080; van der Aa, MP, Elst, MAJ, van de Garde, EMW, van Mil, EGAH, Knibbe, CAJ, van der Vorst, MMJ., Long-term treatment with metformin in obese, insulin-resistant adolescents: results of a randomized double-blinded placebo-controlled trial (2016) Nutr Diabetes, 6 (8), p. e228; Wilson, DM, Abrams, SH, Aye, T, Metformin extended release treatment of adolescent obesity: a 48-week randomized, double-blind, placebo-controlled trial with 48-week follow-up (2010) Arch Pediatr Adolesc Med, 164 (2), pp. 116-123. , Glaser Pediatric Research Network Obesity Study Group; Kay, JP, Alemzadeh, R, Langley, G, D'Angelo, L, Smith, P, Holshouser, S., Beneficial effects of metformin in normoglycemic morbidly obese adolescents (2001) Metabolism, 50 (12), pp. 1457-1461; Love-Osborne, K, Sheeder, J, Zeitler, P., Addition of metformin to a lifestyle modification program in adolescents with insulin resistance (2008) J Pediatr, 152 (6), pp. 817-822; Nadeau, KJ, Ehlers, LB, Zeitler, PS, Love-Osborne, K., Treatment of non-alcoholic fatty liver disease with metformin versus lifestyle intervention in insulin-resistant adolescents (2009) Pediatr Diabetes, 10 (1), pp. 5-13; Wiegand, S, l'Allemand, D, Hübel, H, Metformin and placebo therapy both improve weight management and fasting insulin in obese insulin-resistant adolescents: a prospective, placebo-controlled, randomized study (2010) Eur J Endocrinol, 163 (4), pp. 585-592; Lavine, JE, Schwimmer, JB, Van Natta, ML, Effect of vitamin E or metformin for treatment of nonalcoholic fatty liver disease in children and adolescents: the TONIC randomized controlled trial (2011) JAMA, 305 (16), pp. 1659-1668. , Nonalcoholic Steatohepatitis Clinical Research Network; Pastor-Villaescusa, B, Cañete, MD, Caballero-Villarraso, J, Metformin for obesity in prepubertal and pubertal children: a randomized controlled trial. [published corrections appear in Pediatrics. 2017;140(5):e20172555 and Pediatrics. 2017;140(6):e20173232] (2017) Pediatrics, 140 (1), p. e20164285; Warnakulasuriya, LS, Fernando, MMA, Adikaram, AVN, Metformin in the management of childhood obesity: a randomized control trial (2018) Child Obes, 14 (8), pp. 553-565; Yanovski, JA, Krakoff, J, Salaita, CG, Effects of metformin on body weight and body composition in obese insulin-resistant children: a randomized clinical trial (2011) Diabetes, 60 (2), pp. 477-485; Brufani, C, Crinò, A, Fintini, D, Patera, PI, Cappa, M, Manco, M., Systematic review of metformin use in obese nondiabetic children and adolescents (2013) Horm Res Paediatr, 80 (2), pp. 78-85; McDonagh, MS, Selph, S, Ozpinar, A, Foley, C., Systematic review of the benefits and risks of metformin in treating obesity in children aged 18 years and younger (2014) JAMA Pediatr, 168 (2), pp. 178-184; Park, MH, Kinra, S, Ward, KJ, White, B, Viner, RM., Metformin for obesity in children and adolescents: a systematic review (2009) Diabetes Care, 32 (9), pp. 1743-1745; Sun, J, Wang, Y, Zhang, X, He, H., The effects of metformin on insulin resistance in overweight or obese children and adolescents: a PRISMA-compliant systematic review and meta-analysis of randomized controlled trials (2019) Medicine (Baltimore), 98 (4), p. e14249; Al-Shareef, MA, Sanneh, AFNS, Aljoudi, AS., Clinical effect of metformin in children and adolescents with type 2 diabetes mellitus: a systematic review and meta-analysis (2012) J Family Community Med, 19 (2), pp. 68-73; Mühlig, Y, Wabitsch, M, Moss, A, Hebebrand, J., Weight loss in children and adolescents (2014) Dtsch Arztebl Int, 111 (48), pp. 818-824; Must, A, Anderson, SE., Body mass index in children and adolescents: considerations for population-based applications (2006) Int J Obes (Lond), 30 (4), pp. 590-594; Levri, KM, Slaymaker, E, Last, A, Metformin as treatment for overweight and obese adults: a systematic review (2005) Ann Fam Med, 3 (5), pp. 457-461; Shalitin, S, Phillip, M., Role of obesity and leptin in the pubertal process and pubertal

growth-a review (2003) Int J Obes Relat Metab Disord, 27 (8), pp. 869-874; Pau, CT, Keefe, C, Duran, J, Welt, CK., Metformin improves glucose effectiveness, not insulin sensitivity: predicting treatment response in women with polycystic ovary syndrome in an open-label, interventional study (2014) J Clin Endocrinol Metab, 99 (5), pp. 1870-1878; Knowler, WC, Barrett-Connor, E, Fowler, SE, Reduction in the incidence of type 2 diabetes with lifestyle intervention or metformin (2002) N Engl J Med, 346 (6), pp. 393-403. , Diabetes Prevention Program Research Group; Galderisi, A, Giannini, C, Weiss, R, Trajectories of changes in glucose tolerance in a multiethnic cohort of obese youths: an observational prospective analysis (2018) Lancet Child Adolesc Health, 2 (10), pp. 726-735; Weiss, R, Taksali, SE, Tamborlane, WV, Burgert, TS, Savoye, M, Caprio, S., Predictors of changes in glucose tolerance status in obese youth (2005) Diabetes Care, 28 (4), pp. 902-909; Kleber, M, deSousa, G, Papcke, S, Wabitsch, M, Reinehr, T., Impaired glucose tolerance in obese white children and adolescents: three to five year follow-up in untreated patients (2011) Exp Clin Endocrinol Diabetes, 119 (3), pp. 172-176; Copeland, KC, Silverstein, J, Moore, KR, Management of newly diagnosed type 2 diabetes mellitus (T2DM) in children and adolescents (2013) Pediatrics, 131 (2), pp. 364-382. , American Academy of Pediatrics; Akl, EA, Briel, M, You, JJ, Potential impact on estimated treatment effects of information lost to follow-up in randomised controlled trials (LOST-IT): systematic review (2012) BMJ, 344, p. e2809},
correspondence_address1={Filion, K.B.; Departments of Medicine and of Epidemiology, 3755 Cote-Sainte-Catherine, Suite H410.1, Canada; email: kristian.filion@mcgill.ca},
publisher={American Academy of Pediatrics},
issn={00314005},
coden={PEDIA},
pubmed_id={33608415},
language={English},
abbrev_source_title={Pediatrics},
document_type={Article},
source={Scopus},
}

@ARTICLE{Leyro2021,
author={Leyro, T.M. and Versella, M.V. and Yang, M.-J. and Brinkman, H.R. and Hoyt, D.L. and Lehrer, P.},
title={Respiratory therapy for the treatment of anxiety: Meta-analytic review and regression},
journal={Clinical Psychology Review},
year={2021},
volume={84},
doi={10.1016/j.cpr.2021.101980},
art_number={101980},
note={cited By 0},
url={https://www.scopus.com/inward/record.uri?eid=2-s2.0-85100314073&doi=10.1016%2fj.cpr.2021.101980&partnerID=40&md5=5143d22c3b7eeb33a2b2a0b4fe30d551},
affiliation={Department of Psychology, Rutgers, The State University of New Jersey, United States; Department of Health Outcomes and Behavior, Moffitt Cancer Center, United States; Department of Psychiatry, Rutgers Robert Wood Johnson Medical School, United States},
abstract={Objective: Respiratory abnormalities are a hallmark of anxiety symptomatology and may serve as clinically useful modifiers for alleviating anxiety symptoms. However, gold-standard anxiety treatments (e.g.,

cognitive-behavioral interventions) often do not directly address respiratory components despite their theoretical utility and clinical accessibility. This review examined the clinical effectiveness of respiratory interventions, interventions that directly target respiration abnormalities and processes, in treating trait anxiety symptoms. Methods: The final analysis included 40 randomized controlled trials including at least one measure of trait anxiety, a respiratory-focused intervention group, and a non-respiratory control-group (active or inactive treatment). Overall effects of respiratory focused interventions were examined, as well as the effect of hypothesized moderators. Results: Respiratory component interventions yielded significantly greater improvements (moderate to large effect) in anxiety symptoms than controls, with the stronger effects observed in comparison to inactive, rather than active, control conditions. Significant heterogeneity in findings suggests that variability in intervention design, population, and control comparison may obfuscate interpretation of findings. Conclusions: Evidence supports the clinical utility of respiratory interventions as either an independent anxiety treatment, or as an adjunct to other interventions. Clinical and research implications of findings along with recommendations for ongoing investigations in this domain are discussed. © 2021},
author_keywords={Anxiety; Breathing; Intervention; Meta-analysis; Respiration; Treatment},
keywords={anxiety; artificial ventilation; controlled study; human; randomized controlled trial (topic); respiratory care; review; systematic review},
references={American Psychiatric Association, Diagnostic and statistical manual of mental disorders (1994), 4th ed. Author Washington, D. C; American Psychiatric Association, Diagnostic and statistical manual of mental disorders (2013), 5th ed. Author Washington, D. C; Asmundson, G.J., Frombach, I., McQuaid, J., Pedrelli, P., Lenox, R., Stein, M.B., Dimensionality of posttraumatic stress symptoms: A confirmatory factor analysis of DSM-IV symptom clusters and other symptom models (2000) Behaviour Research and Therapy, 38 (2), pp. 203-214; Balke, B., Lillehei, J.P., Effect of hyperventilation on performance (1956) Journal of Applied Physiology, 9 (3), pp. 371-374; Baxter, A.J., Scott, K.M., Vos, T., Whiteford, H.A., Global prevalence of anxiety disorders: A systematic review and meta-regression (2013) Psychological Medicine, 43 (5), pp. 897-910; Baxter, A.J., Vos, T., Scott, K.M., Ferrari, A.J., Whiteford, H.A., The global burden of anxiety disorders in 2010 (2014) Psychological Medicine, 44 (11), pp. 2363-2374; Beck, A.T., Epstein, N., Brown, G., Steer, R.A., An inventory for measuring clinical anxiety: Psychometric properties (1988) Journal of Consulting and Clinical Psychology, 56 (6), pp. 893-897; Becker, C.B., Zayfert, C., Anderson, E., A survey of psychologists' attitudes towards and utilization of exposure therapy for PTSD (2004) Behaviour Research and Therapy, 42 (3), pp. 277-292; Bekhuis, E., Boschloo, L., Rosmalen, J.G., Schoevers, R.A., Differential associations of specific depressive and anxiety disorders with somatic symptoms (2015) Journal of Psychosomatic Research, 78 (2), pp. 116-122; Ben-Amnon, Y., Fux, M., Maoz, B., Benjamin, J., Respiratory and other symptoms in panic disorder versus other anxiety disorders (1995) Israel Journal of Psychiatry and Related Sciences, 32 (1), pp. 38-43. , https://www.ncbi.nlm.nih.gov/pubmed/7622345; Berger, B.C., The treatment of panic disorder: A comparative study between breathing retraining and cognitive behavioral therapy (2000) Doctor of philosophy dissertation, , Alliant University, California School of Professional Psychology San Diego; Berkeljon, A., Baldwin, S.A., An introduction to meta-analysis for psychotherapy outcome research (2009) Psychotherapy Research, 19 (4-5), pp. 511-518; Bernardi, L.,

Passino, C., Spadacini, G., Bonfichi, M., Arcaini, L., Malcovati, L., Bernasconi, C., Reduced hypoxic ventilatory response with preserved blood oxygenation in yoga trainees and Himalayan Buddhist monks at altitude: Evidence of a different adaptive strategy? (2007) European Journal of Applied Physiology, 99 (5), pp. 511-518; Bernardi, L., Spadacini, G., Bellwon, J., Hajric, R., Roskamm, H., Frey, A.W., Effect of breathing rate on oxygen saturation and exercise performance in chronic heart failure (1998) The Lancet, 351 (9112), pp. 1308-1311; Bindslev, L., Carlsson, J., Santesson, J., Gottlieb, I., Hypoxic pulmonary vasoconstriction in man: Effects of hyperventilation (1986) Survey of Anesthesiology, 30 (1), p. 1; Boettcher, H., Brake, C.A., Barlow, D.H., Origins and outlook of interoceptive exposure (2016) Journal of Behavior Therapy and Experimental Psychiatry, 53, pp. 41-51; Bohr, C., Hasselbalch, K., Krogh, A., Über einen in biologischer Beziehung wichtigen Einfluss, den die Kohlensäurespannung des Blutes auf dessen Sauerstoffbindung übt (1904) Acta Physiologica, 16 (2), pp. 402-412; Bonn, J.A., Readhead, C.P.A., Timmons, B.H., Enhanced adaptive behavioural response in agoraphobic patients pretreated with breathing retraining (1984) The Lancet, 324 (8404), pp. 665-669; Borenstein, M., Higgins, J.P.T., Hedges, L.V., Rothstein, H.R., Basics of meta-analysis: I2 is not an absolute measure of heterogeneity (2017) Research Synthesis Methods, 8 (1), pp. 5-18; Boswell, J.F., Farchione, T.J., Sauer-Zavala, S., Murray, H.W., Fortune, M.R., Barlow, D.H., Anxiety sensitivity and interoceptive exposure: A transdiagnostic construct and change strategy (2013) Behavior Therapy, 44 (3), pp. 417-431; Boulding, R., Stacey, R., Niven, R., Fowler, S.J., Dysfunctional breathing: A review of the literature and proposal for classification (2016) European Respiratory Review, 25 (141), pp. 287-294; Bratton, B.L., Mindfulness in childbirth: An investigation of the effects of mindfulness training on maternal satisfaction with childbirth and obstetric outcomes (2008) Doctor of Philsophy dissertation, , Western Michigan University; Briere, J., DAPS, detailed assessment of posttraumatic stress: Professional manual: Psychological assessment resources (2001); Carpenter, J.K., Andrews, L.A., Witcraft, S.M., Powers, M.B., Smits, J.A.J., Hofmann, S., (2018), G. Cognitive behavioral therapy for anxiety and related disorders: A meta-analysis of randomized placebo-controlled trials. Depression and Anxiety, 1-13. doi:; Chandla, S.S., Sood, S., Dogra, R., Das, S., Shukla, S.K., Gupta, S., Effect of short-term practice of pranayamic breathing exercises on cognition, anxiety, general well being and heart rate variability (2013) Journal of the Indian Medical Association, 111 (10), pp. 662-665. , https://www.ima-india.org/ima/left-side-bar.php?pid=347, Retrieved from; Chang, W.L., Lee, J.T., Li, C.R., Davis, A.H.T., Yang, C.C., Chen, Y.J., Effects of heart rate variability biofeedback in patients with acute ischemic stroke: A randomized controlled trial (2020) Biological Research for Nursing, 22 (1), pp. 34-44; Chen, Y.F., Huang, X.Y., Chien, C.H., Cheng, J.F., The effectiveness of diaphragmatic breathing relaxation training for reducing anxiety (2017) Perspectives in Psychiatric Care, 53 (4), pp. 329-336; Cohen, J., Statistical power analysis for the behavioral sciences (1988), 2nd ed. Erlbaum Hillsdale, NJ; Colgan, D.D., Christopher, M., Michael, P., Wahbeh, H., The body scan and mindful breathing among veterans with PTSD: Type of intervention moderates the relationship between changes in mindfulness and post-treatment depression (2016) Mindfulness, 7 (2), pp. 372-383; Collimore, K.C., Asmundson, G.J.G., Fearful responding to interoceptive exposure in social anxiety disorder (2014) Journal of Anxiety Disorders, 28 (2), pp. 195-202; Courtney, R., Breathing training for dysfunctional breathing in asthma: Taking a multidimensional approach (2017) ERJ Open Research, 3 (4), pp. 00065-02017; Courtney, R., Greenwood, K.M., Cohen, M., Relationships between measures of

dysfunctional breathing in a population with concerns about their breathing (2011) Journal of Bodywork and Movement Therapies, 15 (1), pp. 24-34; Courtney, R., van Dixhoorn, J., Greenwood, K.M., Anthonissen, E.L.M., Medically unexplained dyspnea: Partly moderated by dysfunctional (thoracic dominant) breathing pattern (2011) Journal of Asthma, 48 (3), pp. 259-265; Craske, M.G., Rowe, M., Lewin, M., Noriega-Dimitri, R., Interoceptive exposure versus breathing retraining within cognitive-behavioural therapy for panic disorder with agoraphobia (1997) British Journal of Clinical Psychology, 36 (1), pp. 85-99; Crockett, J.E., Cashwell, C.S., Tangen, J.L., Hall, K.H., Young, J.S., Breathing characteristics and symptoms of psychological distress: An exploratory study (2016) Counseling and Values, 61 (1), pp. 10-27; Cuijpers, P., Gentili, C., Banos, R.M., Garcia-Campayo, J., Botella, C., Cristea, I.A., Relative effects of cognitive and behavioral therapies on generalized anxiety disorder, social anxiety disorder and panic disorder: A meta-analysis (2016) Journal of Anxiety Disorders, 43, pp. 79-89; Delaney, E.P., Greaney, J.L., Edwards, D.G., Rose, W.C., Fadel, P.J., Farquhar, W.B., Exaggerated sympathetic and pressor responses to handgrip exercise in older hypertensive humans: Role of the muscle metaboreflex (2010) American Journal of Physiology. Heart and Circulatory Physiology, 299 (5), pp. H1318-H1327; Dhruva, A., Miaskowski, C., Abrams, D., Acree, M., Cooper, B., Goodman, S., Hecht, F.M., Yoga breathing for cancer chemotherapy–associated symptoms and quality of life: Results of a pilot randomized controlled trial (2012) The Journal of Alternative and Complementary Medicine, 18 (5), pp. 473-479; Enright, S., Chatham, K., Ionescu, A.A., Unnithan, V.B., Shale, D.J., Inspiratory muscle training improves lung function and exercise capacity in adults with cystic fibrosis (2004) Chest Journal, 126 (2), pp. 405-411; Fasko, D., Jr., Hall, G., Osborne, M.R., Kornfeld, R.W.B.H., Comeditation: An exploratory study of pulse and respiration rates and anxiety (1992) Perceptual and Motor Skills, 74 (3), pp. 895-904; Fatouleh, R., Macefield, V.G., Cardiorespiratory coupling of sympathetic outflow in humans: A comparison of respiratory and cardiac modulation of sympathetic nerve activity to skin and muscle (2013) Experimental Physiology, 98 (9), pp. 1327-1336; Gallagher, M.W., Bentley, K.H., Barlow, D.H., Perceived control and vulnerability to anxiety disorders: A meta-analytic review (2014) Cognitive Therapy and Research, 38 (6), pp. 571-584; Gatchel, R.J., Proctor, J.D., Effectiveness of voluntary heart rate control in reducing speech anxiety (1976) Journal of Consulting and Clinical Psychology, 44 (3), pp. 381-389; Gentes, E.L., Ruscio, A.M., A meta-analysis of the relation of intolerance of uncertainty to symptoms of generalized anxiety disorder, major depressive disorder, and obsessive–compulsive disorder (2011) Clinical Psychology Review, 31 (6), pp. 923-933; Giardino, N.D., Chan, L., Borson, S., Combined heart rate variability and pulse oximetry biofeedback for chronic obstructive pulmonary disease: Preliminary findings (2004) Applied Psychophysiology and Biofeedback, 29 (2), pp. 121-133; Gibson, T., The effects of Hypocapnia on psychomotor and intellectual performance (1978) Aviation, Space, and Environmental Medicine, 49 (8), pp. 943-946. , http://www.asma.org/journal, Retrieved from; Grassi, M., Caldirola, D., Vanni, G., Guerriero, G., Piccinni, M., Valchera, A., Perna, G., Baseline respiratory parameters in panic disorder: A meta-analysis (2013) Journal of Affective Disorders, 146 (2), pp. 158-173; Gruzelier, J.H., Thompson, T., Redding, E., Brandt, R., Steffert, T., Application of alpha/theta neurofeedback and heart rate variability training to young contemporary dancers: State anxiety and creativity (2014) International Journal of Psychophysiology, 93 (1), pp. 105-111; Gunter, R.W., Whittal, M.L., Dissemination of cognitive-behavioral treatments for anxiety disorders: Overcoming barriers and improving patient access (2010) Clinical Psychology

Review, 30 (2), pp. 194-202; Hagman, C., Janson, C., Emtner, M., A comparison between patients with dysfunctional breathing and patients with asthma (2008) The Clinical Respiratory Journal, 2 (2), pp. 86-91; Hakked, C.S., Balakrishnan, R., Krishnamurthy, M.N., Yogic breathing practices improve lung functions of competitive young swimmers (2017) Journal of Ayurveda and Integrative Medicine, 8 (2), pp. 99-104; Hamilton, M., A rating scale for depression (1960) Journal of Neurology, Neurosurgery, and Psychiatry, 23 (1), pp. 56-62; Hayama, Y., Inoue, T., The effects of deep breathing on "tension–anxiety"and fatigue in cancer patients undergoing adjuvant chemotherapy (2012) Complementary Therapies in Clinical Practice, 18 (2), pp. 94-98; Hayes, S.C., Luoma, J.B., Bond, F.W., Masuda, A., Lillis, J., Acceptance and commitment therapy: Model, processes and outcomes (2006) Behaviour Research and Therapy, 44 (1), pp. 1-25; Hedges, L., Olkin, I., Statistical methods for meta-analysis (1985), Academic Press New York; Hedges, L.V., Vevea, J.L., Fixed-and random-effects models in meta-analysis (1998) Psychological Methods, 3 (4), p. 486; Hellström, F., Roatta, S., Thunberg, J., Passatore, M., Djupsjöbacka, M., Responses of muscle spindles in feline dorsal neck muscles to electrical stimulation of the cervical sympathetic nerve (2005) Experimental Brain Research, 165 (3), pp. 328-342; Henriques, G., Keffer, S., Abrahamson, C., Horst, S.J., Exploring the effectiveness of a computer-based heart rate variability biofeedback program in reducing anxiety in college students (2011) Applied Psychophysiology and Biofeedback, 36 (2), pp. 101-112; Henry, J.D., Crawford, J.R., The short-form version of the depression anxiety stress scales (DASS-21): Construct validity and normative data in a large non-clinical sample (2005) British Journal of Clinical Psychology, 44 (2), pp. 227-239; Hibbert, G.A., Chan, M., Respiratory control: Its contribution to the treatment of panic attacks. A controlled study (1989) The British Journal of Psychiatry, 154 (2), pp. 232-236; Higgins, J.P., Thomas, J., Chandler, J., Cumpston, M., Li, T., Page, M.J., Welch, V.A., Cochrane handbook for systematic reviews of interventions (2019), John Wiley & Sons; Higgins, J.P.T., Thompson, S.G., Deeks, J.J., Altman, D.G., Measuring inconsistency in meta-analyses (2003) BMJ [British Medical Journal], 327 (7414), pp. 557-560; Hipol, L.J., Deacon, B.J., Dissemination of evidence-based practices for anxiety disorders in Wyoming: A survey of practicing psychotherapists (2013) Behavior Modification, 37 (2), pp. 170-188; Hofmann, S.G., Asnaani, A., Vonk, I.J., Sawyer, A.T., Fang, A., The efficacy of cognitive behavioral therapy: A review of meta-analyses (2012) Cognitive Therapy and Research, 36 (5), pp. 427-440; Hofmann, S.G., Smits, J.A., Cognitive-behavioral therapy for adult anxiety disorders: A meta-analysis of randomized placebo-controlled trials (2008) Journal of Clinical Psychiatry, 69 (4), pp. 621-632; Holaway, R.M., Heimberg, R.G., Coles, M.E., A comparison of intolerance of uncertainty in analogue obsessive-compulsive disorder and generalized anxiety disorder (2006) Journal of Anxiety Disorders, 20 (2), pp. 158-174; Keogh, E., Reidy, J., Exploring the factor structure of the mood and anxiety symptom questionnaire (MASQ) (2000) Journal of Personality Assessessment, 74 (1), pp. 106-125; Kessler, R.C., Petukhova, M., Sampson, N.A., Zaslavsky, A.M., Wittchen, H.U., Twelve-month and lifetime prevalence and lifetime morbid risk of anxiety and mood disorders in the United States (2012) International Journal of Methods in Psychiatric Research, 21 (3), pp. 169-184; King, D.W., Leskin, G.A., King, L.A., Weathers, F.W., Confirmatory factor analysis of the clinician-administered PTSD scale: Evidence for the dimensionality of posttraumatic stress disorder (1998) Psychological Assessment, 10 (2), p. 90; Kliem, S., Kröger, C., Foran, H.M., Mößle, T., Glaesmer, H., Zenger, M., Brähler, E., Dimensional latent structure of PTSD-symptoms reporting: Is it adding by subtracting? (2016) Psychological

Assessment, 28 (12), pp. 1663-1673; Kotov, R., Krueger, R.F., Watson, D., Achenbach, T.M., Althoff, R.R., Bagby, R.M., Zimmerman, M., The hierarchical taxonomy of psychopathology (HiTOP): A dimensional alternative to traditional nosologies (2017) Journal of Abnormal Psychology, 126 (4), pp. 454-477; Kotov, R., Perlman, G., Gámez, W., Watson, D., The structure and short-term stability of the emotional disorders: A dimensional approach (2015) Psychological Medicine, 45 (8), pp. 1687-1698; Lau, E.X., Rapee, R.M., Prevention of anxiety disorders (2011) Current Psychiatry Reports, 13 (4), pp. 258-266; Leander, M., Lampa, E., Rask-Andersen, A., Franklin, K., Gislason, T., Oudin, A., Janson, C., Impact of anxiety and depression on respiratory symptoms (2014) Respiratory Medicine, 108 (11), pp. 1594-1600; Lee, J., Kim, J.K., Wachholtz, A., The benefit of heart rate variability biofeedback and relaxation training in reducing trait anxiety (2015) Han'guk Simni Hakhoe chi. Kon'gang= The Korean Journal of Health Psychology, 20 (2), pp. 391-408; Lehrer, P., Vaschillo, E., Heart rate variability biofeedback: A new tool for improving autonomic homeostasis and treating emotional and psychosomatic diseases (special address in 2002) (2004) Japanese Journal of Biofeedback Research, 30, pp. 7-16; Lehrer, P.M., Vaschillo, E., Vaschillo, B., Resonant frequency biofeedback training to increase cardiac variability: Rationale and manual for training (2000) Applied Psychophysiology and Biofeedback, 25 (3), pp. 177-191; Lehrer, P.M., Vaschillo, E.G., Vaschillo, B., Lu, S.-E., Eckberg, D.L., Edelberg, R., Hamer, R.M., Heart rate variability biofeedback increases baroreflex gain and peak expiratory flow (2003) Psychosomatic Medicine, 65 (5), pp. 796-805; Lickel, J., Nelson, E., Lickel, A.H., Deacon, B., Interoceptive exposure exercises for evoking depersonalization and derealization: A pilot study (2008) Journal of Cognitive Psychotherapy, 22 (4), pp. 321-330; Lickel, J.J., The use of breathing retraining among individuals who fear anxiety-related body sensations: Safety behavior or coping aid? [doctor of philosophy dissertation] (2010), University of Wyoming; Lickel, J.J., Carruthers, B.R., Dixon, L.J., Deacon, B.J., Breathing retraining for individuals who fear respiratory sensations: Examination of safety behavior and coping aid hypotheses (2013) Journal of Cognitive Psychotherapy, 27 (2), pp. 111-125; Lin, I.M., Effects of a cardiorespiratory synchronization training mobile application on heart rate variability and electroencephalography in healthy adults (2018) International Journal of Psychophysiology, 134, pp. 168-177; Lin, I.M., Fan, S.Y., Yen, C.F., Yeh, Y.C., Tang, T.C., Huang, M.F., Tsai, Y.C., Heart rate variability biofeedback increased autonomic activation and improved symptoms of depression and insomnia among patients with major depression disorder (2019) Clinical Psychopharmacology and Neuroscience, 17 (2), pp. 222-232; Lovibond, P.F., Lovibond, S.H., The structure of negative emotional states: Comparison of the depression anxiety stress scales (DASS) with the Beck depression and anxiety inventories (1995) Behaviour Research and Therapy, 33 (3), pp. 335-343; Malatino, L.S., Bellofiore, S., Costa, M.P., Manto, G.L., Finocchiaro, F., Di Maria, G.U., Cerebral blood flow velocity after hyperventilation-induced vasoconstriction in hypertensive patients (1992) Stroke, 23 (12), pp. 1728-1732; McKinnon, A.C., Van Oudenhove, L., Tack, J., Jones, M., The association of personality, appraisal, catastrophising and vigilance with gastrointestinal symptom-specific anxiety (2015) Journal of Health Psychology, 20 (4), pp. 456-465; McNair, D., Lorr, M., Droppleman, L., Profile of mood state manual (1971), Educational and Industrial Testing Service San Diego (CA); Meuret, A.E., Ritz, T., Hyperventilation in panic disorder and asthma: Empirical evidence and clinical strategies (2010) International Journal of Psychophysiology, 78 (1), pp. 68-79; Meuret, A.E., Rosenfield, D., Seidel, A., Bhaskara, L., Hofmann, S.G., Respiratory and cognitive mediators of treatment

change in panic disorder: Evidence for intervention specificity (2010) Journal of Consulting and Clinical Psychology, 78 (5), pp. 691-704; Meuret, A.E., Wilhelm, F.H., Ritz, T., Roth, W.T., Feedback of end-tidal pCO 2 as a therapeutic approach for panic disorder (2008) Journal of Psychiatric Research, 42 (7), pp. 560-568; Michel, C.C., Lloyd, B.B., Cunningham, D.J.C., The in vivo carbon dioxide dissociation curve of true plasma (1966) Respiration Physiology, 1 (2), pp. 121-137; van Minnen, A., Hendriks, L., Olff, M., When do trauma experts choose exposure therapy for PTSD patients? A controlled study of therapist and patient factors (2010) Behaviour Research and Therapy, 48 (4), pp. 312-320; Montero-Marin, J., Garcia-Campayo, J., Lopez-Montoyo, A., Zabaleta-Del-Olmo, E., Cuijpers, P., Is cognitive-behavioural therapy more effective than relaxation therapy in the treatment of anxiety disorders? A meta-analysis (2018) Psychological Medicine, 48 (9), pp. 1427-1436; Neuman, Å., Gunnbjörnsdottir, M., Tunsäter, A., Nyström, L., Franklin, K.A., Norrman, E., Janson, C., Dyspnea in relation to symptoms of anxiety and depression: A prospective population study (2006) Respiratory Medicine, 100 (10), pp. 1843-1849; Olatunji, B.O., Cisler, J.M., Deacon, B.J., Efficacy of cognitive behavioral therapy for anxiety disorders: A review of meta-analytic findings (2010) Psychiatric Clinics, 33 (3), pp. 557-577; Olatunji, B.O., Wolitzky-Taylor, K.B., Anxiety sensitivity and the anxiety disorders: A meta-analytic review and synthesis (2009) Psychological Bulletin, 135 (6), pp. 974-999; Pai, A., Suris, A., North, C., Posttraumatic stress disorder in the DSM-5: Controversy, change, and conceptual considerations (2017) Behavioral Science, 7 (1), p. 7; Papp, L.A., Martinez, J.M., Klein, D.F., Coplan, J.D., Norman, R.G., Cole, R., Gorman, J.M., Respiratory psychophysiology of panic disorder: Three respiratory challenges in 98 subjects (1997) American Journal of Psychiatry, 154 (11), pp. 1557-1565; Paul, M., Garg, K., The effect of heart rate variability biofeedback on performance psychology of basketball players (2012) Applied Psychophysiology and Biofeedback, 37 (2), pp. 131-144; Paulus, M.P., The breathing conundrum—Interoceptive sensitivity and anxiety (2013) Depression and Anxiety, 30 (4), pp. 315-320; Penzlin, A.I., Siepmann, T., Illigens, B.M.-W., Weidner, K., Siepmann, M., Heart rate variability biofeedback in patients with alcohol dependence: A randomized controlled study (2015) Neuropsychiatric Disease and Treatment, 11, pp. 2619-2627; Pham, Q., Khatib, Y., Stansfeld, S., Fox, S., Green, T., Feasibility and efficacy of an mHealth game for managing anxiety:“flowy” randomized controlled pilot trial and design evaluation (2016) Games for Health Journal, 5 (1), pp. 50-67; Pollard, K.L., Campbell, C., Squires, M., Palsson, O., van Tilburg, M.A.L., Seasonal association of pediatric functional abdominal pain disorders and anxiety (2018) Journal of Pediatric Gastroenterology and Nutrition, 67 (1), pp. 18-22; Pompoli, A., Furukawa, T.A., Efthimiou, O., Imai, H., Tajika, A., Salanti, G., Dismantling cognitive-behaviour therapy for panic disorder: A systematic review and component network meta-analysis (2018) Psychological Medicine, 48 (12), pp. 1945-1953; Radovanovic, D., Peikert, K., Lindström, M., Domellöf, F.P., Sympathetic innervation of human muscle spindles (2015) Journal of Anatomy, 226 (6), pp. 542-548; Reiss, S., Peterson, R.A., Gursky, D.M., McNally, R.J., Anxiety sensitivity, anxiety frequency and the prediction of fearfulness (1986) Behaviour Research and Therapy, 24 (1), pp. 1-8; Risk of Bias, Risk of bias tools (2020), https://www.riskofbias.info/welcome; Ritz, T., Meuret, A.E., Bhaskara, L., Petersen, S., Respiratory muscle tension as symptom generator in individuals with high anxiety sensitivity (2013) Psychosomatic Medicine, 75 (2), pp. 187-195; Rosenthal, R., Rubin, D.B., Meta-analytic procedures for combining studies with multiple effect sizes (1986) Psychological Bulletin, 99 (3), pp. 400-406; Saigo, T., Tayama, J., Hamaguchi, T., Nakaya, N., Tomiie, T.,

Bernick, P.J., Fukudo, S., Gastrointestinal specific anxiety in irritable bowel syndrome: Validation of the Japanese version of the visceral sensitivity index for university students (2014) BioPsychoSocial Medicine, 8 (1), p. 10; Schmidt, N.B., Woolaway-Bickel, K., Trakowski, J., Santiago, H., Storey, J., Koselka, M., Cook, J., Dismantling cognitive–behavioral treatment for panic disorder: Questioning the utility of breathing retraining (2000) Journal of Consulting and Clinical Psychology, 68 (3), pp. 417-424; Schwartz, G.E., Davidson, R.J., Goleman, D.J., Patterning of cognitive and somatic processes in the self-regulation of anxiety: Effects of meditation versus exercise (1978) Psychosomatic Medicine, 40 (4), pp. 321-328; Shear, M.K., Rucci, P., Williams, J., Frank, E., Grochocinski, V.J., Bilt, J.V., Wang, T., Reliability and validity of the panic disorder severity scale: Replication and extension (2001) Journal of Psychiatric Research, 35 (5), pp. 293-296. , doi:10.1016/s0022-3956(01)00028-0; Siev, J., Chambless, D.L., Specificity of treatment effects: Cognitive therapy and relaxation for generalized anxiety and panic disorders (2007) Journal of Consulting and Clinical Psychology, 75 (4), p. 513; Simeon, D., Guralnik, O., Knutelska, M., Yehuda, R., Schmeidler, J., Basal norepinephrine in depersonalization disorder (2003) Psychiatry Research, 121 (1), pp. 93-97; Simon, N.M., Weiss, A.M., Kradin, R., Evans, K.C., Reese, H.E., Otto, M.W., Pollack, M.H., The relationship of anxiety disorders, anxiety sensitivity and pulmonary dysfunction with dyspnea-related distress and avoidance (2006) The Journal of Nervous and Mental Disease, 194 (12), pp. 951-957; Smith, E.N., Santoro, E., Moraveji, N., Susi, M., Crum, A.J., Integrating wearables in stress management interventions: Promising evidence from a randomized trial (2019) International Journal of Stress Management, 27 (2), pp. 172-182; Smith, R.E., Smoll, F.L., Cumming, S.P., Grossbard, J.R., Measurement of multidimensional sport performance anxiety in children and adults: The sport anxiety Scale-2 (2006) Journal of Sport and Exercise Psychology, 28 (4), pp. 479-501; Spicuzza, L., Porta, C., Bramanti, A., Maffeis, M., Casucci, G., Casiraghi, N., Bernardi, L., Interaction between central-peripheral chemoreflexes and cerebro-cardiovascular control (2005) Clinical Autonomic Research, 15 (6), pp. 373-381; Spielberg, C.D., Gorsuch, R.L., Lushene, R.E., Manual for the state-trait anxiety inventory (1983), https://www.apa.org/pi/about/publications/caregivers/practice-settings/assessment/tools/trait-state, Consulting Psychologist Press Palo Alto, CA Retrieved from; Spitzer, R.L., Kroenke, K., Williams, J.B., Löwe, B., A brief measure for assessing generalized anxiety disorder: The GAD-7 (2006) Archives of Internal Medicine, 166 (10), pp. 1092-1097; Stanley, T.D., Doucouliagos, H., Meta-regression analysis in economics and business (2012), 5. , Routledge; Stanley, T.D., Jarrell, S.B., Meta-regression analysis: A quantitative method of literature surveys (1989) Journal of Economic Surveys, 3 (2), pp. 161-170; Stapinski, L.A., Abbott, M.J., Rapee, R.M., Evaluating the cognitive avoidance model of generalised anxiety disorder: Impact of worry on threat appraisal, perceived control and anxious arousal (2010) Behaviour Research and Therapy, 48 (10), pp. 1032-1040; Stefanaki, C., Bacopoulou, F., Livadas, S., Kandaraki, A., Karachalios, A., Chrousos, G.P., Diamanti-Kandarakis, E., Impact of a mindfulness stress management program on stress, anxiety, depression and quality of life in women with polycystic ovary syndrome: A randomized controlled trial (2015) Stress, 18 (1), pp. 57-66; Stein, M.B., Millar, T.W., Larsen, D.K., Kryger, M.H., Irregular breathing during sleep in patients with panic disorder (1995) The American Journal of Psychiatry, 152 (8), pp. 1168-1173; Sterne, J.A.C., Harbord, R.M., Funnel plots in meta-analysis (2004) Stata Journal, 4, pp. 127-141; Tan, G., Dao, T.K., Farmer, L., Sutherland, R.J., Gevirtz, R., Heart rate variability (HRV) and posttraumatic stress

disorder (PTSD): A pilot study (2011) Applied Psychophysiology and Biofeedback, 36 (1), pp. 27-35; Taylor, V.R., Measuring healthy days; population assessment of health-related quality of life (2000); Thomas, M., McKinley, R.K., Mellor, S., Watkin, G., Holloway, E., Scullion, J., Pavord, I., Breathing exercises for asthma: A randomised controlled trial (2009) Thorax, 64 (1), pp. 55-61; Thorp, S.R., Ayers, C.R., Nuevo, R., Stoddard, J.A., Sorrell, J.T., Wetherell, J.L., Meta-analysis comparing different behavioral treatments for late-life anxiety (2009) The American Journal of Geriatric Psychiatry, 17 (2), pp. 105-115; Thurber, M.R., Effects of heart-rate variability biofeedback training and emotional regulation on music performance anxiety in university students. [dissertation] (2007), University of North Texas; Tolin, D.F., Is cognitive–behavioral therapy more effective than other therapies?: A meta-analytic review (2010) Clinical Psychology Review, 30 (6), pp. 710-720; Uliaszek, A.A., Alden, A., Zinbarg, R.E., Dimensionality in cognitive-behavioral assessment (2015) Assessment in cognitive therapy, pp. 149-171. , http://ebookcentral.proquest.com/lib/rutgers-ebooks/detail.action?docID=1760728 , Guilford Press New York, NY Retrieved from; Valenza, M.C., Valenza-Peña, G., Torres-Sánchez, I., González-Jiménez, E., Conde-Valero, A., Valenza-Demet, G., Effectiveness of controlled breathing techniques on anxiety and depression in hospitalized patients with COPD: A randomized clinical trial (2014) Respiratory Care, 59 (2), pp. 209-215; Van den Bergh, O., Zaman, J., Bresseleers, J., Verhamme, P., Van Diest, I., Anxiety, pCO2 and cerebral blood flow (2013) International Journal of Psychophysiology, 89 (1), pp. 72-77; Van Diest, I., Stegen, K., Van de Woestijne, K.P., Schippers, N., Van den Bergh, O., Hyperventilation and attention: Effects of hypocapnia on performance in a Stroop task (2000) Biological Psychology, 53 (2-3), pp. 233-252; Van Dixhoorn, J., Duivenvoorden, H.J., Pool, J., Verhage, F., Psychic effects of physical training and relaxation therapy after myocardial infarction (1990) Journal of Psychosomatic Research, 34 (3), pp. 327-337; Wahbeh, H., Goodrich, E., Goy, E., Oken, B.S., Mechanistic pathways of mindfulness meditation in combat veterans with posttraumatic stress disorder (2016) Journal of Clinical Psychology, 72 (4), pp. 365-383; Wald, J., Taylor, S., Responses to interoceptive exposure in people with posttraumatic stress disorder (PTSD): A preliminary analysis of induced anxiety reactions and trauma memories and their relationship to anxiety sensitivity and PTSD symptom severity (2008) Cognitive Behaviour Therapy, 37 (2), pp. 90-100; Wang, K., Zeng, G.Q., Li, R., Luo, Y.W., Wang, M., Hu, Y.H., Chen, X., Cycle ergometer and inspiratory muscle training offer modest benefit compared with cycle ergometer alone: A comprehensive assessment in stable COPD patients (2017) International Journal of Chronic Obstructive Pulmonary Disease, 12, pp. 2655-2668; Wang, P.S., Lane, M., Olfson, M., Pincus, H.A., Wells, K.B., Kessler, R.C., Twelve-month use of mental health services in the United States: Results from the National Comorbidity Survey Replication (2005) Archives of General Psychiatry, 62 (6), pp. 629-640; Weathers, F., Litz, B., Psychometric properties of the clinician-administered PTSD scale, CAPS-1 (1994) PTSD Research Quarterly, 5 (2), pp. 2-6. , https://www.ptsd.va.gov/publications/ptsd_rq.asp; White, B., The effects of heart rate variability biofeedback as an adjunct to therapy on trauma symptoms: ProQuest (2008); Whiteside, S.P., Deacon, B.J., Benito, K., Stewart, E., Factors associated with practitioners’ use of exposure therapy for childhood anxiety disorders (2016) Journal of Anxiety Disorders, 40, pp. 29-36; Wolitzky-Taylor, K., Zimmermann, M., Arch, J.J., De Guzman, E., Lagomasino, I., Has evidence-based psychosocial treatment for anxiety disorders permeated usual care in community mental health settings? (2015) Behaviour Research and

Therapy, 72, pp. 9-17; Yekta, Z.P., Sadeghian, F., Larijani, T.T., Mehran, A., The comparison of two types of relaxation techniques on postoperative state anxiety in candidates for the mastectomy surgery: A randomized controlled clinical trial (2017) International Journal of Community Based Nursing and Midwifery, 5 (1), p. 61. , https://ijcbnm.sums.ac.ir/, Retrieved from; Zigmond, A.S., Snaith, R.P., The hospital anxiety and depression scale (1983) Acta Psychiatrica Scandinavica, 67 (6), pp. 361-370; Zucker, T.L., Samuelson, K.W., Muench, F., Greenberg, M.A., Gevirtz, R.N., The effects of respiratory sinus arrhythmia biofeedback on heart rate variability and posttraumatic stress disorder symptoms: A pilot study (2009) Applied Psychophysiology and Biofeedback, 34 (2), pp. 135-143; Zvolensky, M.J., Bernstein, A., Cardenas, S.J., Colotla, V.A., Marshall, E.C., Feldner, M.T., Anxiety sensitivity and early relapse to smoking: A test among Mexican daily, low-level smokers (2007) Nicotine & Tobacco Research, 9 (4), pp. 483-491; Zvolensky, M.J., Forsyth, J.P., Fuse, T., Feldner, M.T., Leen-Feldner, E.W., Panic attacks and smoking: An initial investigation of panic-relevant cognitive processes (2002) Cognitive Behaviour Therapy, 31, pp. 170-182; van der Zwan, J.E., de Vente, W., Huizink, A.C., Bögels, S.M., de Bruin, E.I., Physical activity, mindfulness meditation, or heart rate variability biofeedback for stress reduction: A randomized controlled trial (2015) Applied Psychophysiology and Biofeedback, 40 (4), pp. 257-268; van der Zwan, J.E., Huizink, A.C., Lehrer, P.M., Koot, H.M., de Vente, W., The effect of heart rate variability biofeedback training on mental health of pregnant and non-pregnant women: A randomized controlled trial (2019) International Journal of Environmental Research and Public Health, 16 (6)},
correspondence_address1={Leyro, T.M.; Department of Psychology, United States; email: tml124@psych.rutgers.edu},
publisher={Elsevier Inc.},
issn={02727358},
coden={CPSRD},
language={English},
abbrev_source_title={Clin. Psychol. Rev.},
document_type={Review},
source={Scopus},
}

@ARTICLE{Armario202185,
author={Armario, P. and Brotons, C. and Elosua, R. and Alonso de Leciñana, M. and Castro, A. and Clarà, A. and Cortés, O. and Díaz Rodriguez, Á. and Herranz, M. and Justo, S. and Lahoz, C. and Pedro-Botet, J. and Pérez Pérez, A. and Santamaria, R. and Tresserras, R. and Aznar Lain, S. and Royo-Bordonada, M.Á.},
title={Statement of the Spanish Interdisciplinary Vascular Prevention Committee on the updated European Cardiovascular Prevention Guidelines [Comentario del CEIPV a la actualización de las Guías Europeas de Prevención Vascular en la Práctica Clínica]},
journal={Clinica e Investigacion en Arteriosclerosis},
year={2021},
volume={33},
number={2},
pages={85-107},
doi={10.1016/j.arteri.2020.11.004},
note={cited By 0},
url={https://www.scopus.com/inward/record.uri?eid=2-s2.0-85099951560&doi=10.1016%2fj.arteri.2020.11.004&partnerID=40&md5=469009f7559fbf01252931a67d9351f9},
affiliation={Sociedad Española-Liga Española para la Lucha contra la

Hipertensión Arterial, Madrid, Spain; Sociedad Española de Medicina de Familia y Comunitaria, Barcelona, Spain; Sociedad Española de Epidemiología, Barcelona, Spain; Sociedad Española de Neurología, Spain; Sociedad Española de Cardiología-Coordinadora Nacional Sección de Prevención, Madrid, Spain; Sociedad Española de Angiología y Cirugía Vascular, Madrid, Spain; Asociación Española Pediatría de Atención Primaria, Madrid, Spain; Sociedad Española de Médicos de Atención Primaria-Semergen, Madrid, Spain; Federación de Asociaciones de Enfermería Comunitaria y Atención Primaria-FAECAP, Madrid, Spain; Ministerio de Sanidad, Madrid, Spain; Sociedad Española de Medicina Interna, Madrid, Spain; Sociedad Española de Arteriosclerosis, Barcelona, Spain; Sociedad Española de Diabetes, Madrid, Spain; Sociedad Española de Nefrología, Spain; Sociedad Española de Salud Pública y Administración Sanitaria-SESPAS, Barcelona, Spain; Facultad de Ciencias del Deporte, Universidad Castilla La Mancha, Toledo, Spain; Instituto de Salud Carlos III, Madrid, Spain},
abstract={We present the adaptation for Spain of the updated European Cardiovascular Prevention Guidelines. In this update, greater stress is laid on the population approach, and especially on the promotion of physical activity and healthy diet through dietary, leisure and active transport policies in Spain. To estimate vascular risk, note should be made of the importance of recalibrating the tables used, by adapting them to population shifts in the prevalence of risk factors and incidence of vascular diseases, with particular attention to the role of chronic kidney disease. At an individual level, the key element is personalised support for changes in behaviour, adherence to medication in high-risk individuals and patients with vascular disease, the fostering of physical activity, and cessation of smoking habit. Furthermore, recent clinical trials with PCSK9 inhibitors are reviewed, along with the need to simplify pharmacological treatment of arterial hypertension to improve control and adherence to treatment. In the case of patients with type 2 diabetes mellitus and vascular disease or high vascular disease risk, when lifestyle changes and metformin are inadequate, the use of drugs with proven vascular benefit should be prioritised. Lastly, guidelines on peripheral arterial disease and other specific diseases are included, as is a recommendation against prescribing antiaggregants in primary prevention. © 2020 Sociedad Española de Arteriosclerosis},
author_keywords={Arterial hypertension; Clinical Practice Guidelines; Diabetes; Healthy diet; Lipid control; Prevention and control; Smoking; Vascular diseases},
references={Piepoli, M.F., Hoes, A.W., Agewall, S., Albus, C., Brotons, C., Catapano, A.L., 2016 European Guidelines on cardiovascular disease prevention in clinical practice: The Sixth Joint Task Force of the European Society of Cardiology and Other Societies on Cardiovascular Disease Prevention in Clinical Practice (constituted by representatives of 10 societies and by invited experts) Developed with the special contribution of the European Association for Cardiovascular Prevention & Rehabilitation (EACPR) (2016) Eur Heart J., 37, pp. 2315-2381; Royo-Bordonada, M.A., Armario, P., Lobos Bejarano, J.M., Pedro-Botet, J., Villar Alvarez, F., Elosua, R., Adaptación española de las guías europeas de 2016 sobre prevención de la enfermedad cardiovascular en la práctica clínica (2016) Rev Esp Salud Publica., 90, pp. e1-e24; Piepoli, M.F., Abreu, A., Albus, C., Ambrosetti, M., Brotons, C., Catapano, A.L., Update on cardiovascular prevention in clinical practice: A position paper of the European Association of Preventive Cardiology of the European Society of Cardiology* (2020) Eur J Prev Cardiol., 27, pp. 181-205; Global, regional, and national age-sex specific mortality and life expectancy, 1950-2017: a

systematic analysis for the Global Burden of Diseases Study 2017 (2018) Lancet, 392, pp. 1684-1735; Wilkins, E., Wilson, L., Wickramasinghe, K., Bhatnagar, P., Rayner, M., Towsend, N., European Cardiovascular Disease Statistics (2017), 2017 Edition European heart network Belgium; (2020) Estadística de defunciones según la causa de muerte., , https://www.ine.es/dyngs/INEbase/es/operacion.htm?c=Estadistica_C&cid=1254736176780&menu=ultiDatos&idp=1254735573175, Disponible en:; Soriano, J.B., Rojas-Rueda, D., Alonso, J., Antó, J.M., Cardona, P.-J., Fernández, E., La carga de enfermedad en España: resultados del Estudio de la Carga Global de las Enfermedades 2016 (2018) Med Clin (Barc)., 151, pp. 171-190; Castellano Vázquez, J.M., Fernández Alvira, J.M., Fuster, V., La prevención primordial, primordial en la prevención cardiovascular (2020) Rev Esp Cardiol., 73, pp. 194-196; Lloyd-Jones, D.M., Hong, Y., Labarthe, D., Mozaffarian, D., Appel, L.J., Van Horn, L., Defining and setting national goals for cardiovascular health promotion and disease reduction: the American Heart Association's strategic Impact Goal through 2020 and beyond (2010) Circulation., 121, pp. 586-613; Díez-Espino, J., Buil-Cosales, P., Babio, N., Toledo, E., Corella, D., Ros, E., Impacto de Life's Simple 7 en la incidencia de eventos cardiovasculares mayores en adultos españoles con alto riesgo de la cohorte del estudio PREDIMED (2020) Rev Esp Cardiol., 73, pp. 205-211; Conroy, R.M., Pyörälä, K., Fitzgerald, A.P., Sans, S., Menotti, A., De Backer, G., Estimation of ten-year risk of fatal cardiovascular disease in Europe: the SCORE project (2003) Eur Heart J., 24, pp. 987-1003; Baena-Díez, J.M., Subirana, I., Ramos, R., Gómez de la Cámara, A., Elosua, R., Vila, J., Evaluación de la validez de las funciones SCORE de bajo riesgo y calibrada para población española en las cohortes FRESCO (2018) Rev Esp Cardiol., 71, pp. 274-282; Vega Alonso, A.T., Ordax Díez, A., Lozano Alonso, J.E., Álamo Sanz, R., Lleras Muñoz ̃ S., García Palomar, P., Validación del índice SCORE y el SCORE para personas mayores en la cohorte de riesgo de enfermedad cardiovascular en Castilla y León (2019) Hipertens Riesgo Vasc., 36, pp. 184-192; Brotons, C., Moral, I., Fernández, D., Puig, M., Calvo Bonacho, E., Martínez Muñoz, P., Catalina Romero, C., Quevedo Aguado, L.J., Estimation of Lifetime Risk of Cardiovascular Disease (IBERLIFERISK): A New Tool for Cardiovascular Disease Prevention in Primary Care (2019) Rev Esp Cardiol (Engl Ed)., 72, pp. 562-568; Marrugat, J., Solanas, P., D'Agostino, R., Sullivan, L., Ordovas, J., Cordón, F., Estimación del riesgo coronario en España mediante la ecuación de Framingham calibrada (2003) Rev Esp Cardiol., 56, pp. 253-261; Marrugat, J., Subirana, I., Comín, E., Cabezas, C., Vila, J., Elosua, R., Validity of an adaptation of the Framingham cardiovascular risk function: the VERIFICA study (2007) J Epidemiol Community Health., 61, pp. 40-47; Marrugat, J., Subirana, I., Ramos, R., Vila, J., Marín-Ibañez, A., Guembe, M.J., Derivation and validation of a set of 10-year cardiovascular risk predictive functions in Spain: the FRESCO Study (2014) Prev Med., 61, pp. 66-74; Brotons, C., Moral, I., Fernández, D., Puig, M., Calvo Bonacho, E., Martínez Muñoz, P., Estimation of Lifetime Risk of Cardiovascular Disease (IBERLIFERISK): A New Tool for Cardiovascular Disease Prevention in Primary Care (2019) Rev Esp Cardiol (Engl Ed)., 72, pp. 562-568; Jaspers, N.E.M., Blaha, M.J., Matsushita, K., van der Schouw, Y.T., Wareham, N.J., Khaw, K.-T., Prediction of individualized lifetime benefit from cholesterol lowering, blood pressure lowering, antithrombotic therapy, and smoking cessation in apparently healthy people (2020) Eur Heart J., 41, pp. 1190-1199; Berkelmans, G.F.N., Gudbjörnsdottir, S., Visseren, F.L.J., Wild, S.H., Franzen, S., Chalmers, J., Prediction of individual life-years gained without cardiovascular events from lipid, blood pressure, glucose, and aspirin treatment based on data of more than 500 000 patients with Type 2 diabetes mellitus (2019) Eur Heart

J., 40, pp. 2899-2906; Lin, J.S., Evans, C.S., Johnson, E., Redmon, N., Coppola, E.L., Smith, N., Nontraditional risk factors in cardiovascular risk assessment: updated evidence report and systematic review for the US Preventive Services Task Force (2018) JAMA., 320, pp. 281-297; Arnett, D.K., Blumenthal, R.S., Albert, M.A., Buroker, A.B., Goldberger, Z.D., Hahn, E.J., 2019 ACC/AHA guideline on the primary prevention of cardiovascular disease: a report of the American College of Cardiology/American Heart Association Task Force on Clinical Practice Guidelines (2019) Circulation., 140, pp. e596-e646; Tada, H., Melander, O., Louie, J.Z., Catanese, J.J., Rowland, C.M., Devlin, J.J., Risk prediction by genetic risk scores for coronary heart disease is independent of self-reported family history (2016) Eur Heart J, 37, pp. 561-567; Jarmul, J., Pletcher, M.J., Hassmiller Lich, K., Wheeler, S.B., Weinberger, M., Avery, C.L., Cardiovascular Genetic Risk Testing for Targeting Statin Therapy in the Primary Prevention of Atherosclerotic Cardiovascular Disease: A Cost-Effectiveness Analysis (2018) Circ Cardiovasc Qual Outcomes., 11, p. e004171; de Gonzalo-Calvo, D., Iglesias-Gutiérrez, E., Llorente-Cortés, V., Epigenetic Biomarkers and Cardiovascular Disease: Circulating MicroRNAs (2017) Rev Esp Cardiol (Engl Ed)., 70, pp. 763-769; Vaccarino, V., Badimon, L., Bremner, J.D., Cenko, E., Cubedo, J., Dorobantu, M., (2020) Eur Heart J., 41, pp. 1687-1696; Pogosova, N., Kotseva, K., De Bacquer, D., von Känel, R., De Smedt, D., Bruthans, J., Psychosocial risk factors in relation to other cardiovascular risk factors in coronary heart disease: Results from the EUROASPIRE IV survey. A registry from the European Society of Cardiology (2017) Eur J Prev Cardiol., 24, pp. 1371-1380; Suglia, S.F., Koenen, K.C., Boynton-Jarrett, R., Chan, P.S., Clark, C.J., Danese, A., Childhood and adolescent adversity and cardiometabolic outcomes: a scientific statement from the American Heart Association (2018) Circulation, 137, pp. e15-e28; Hippisley-Cox, J., Coupland, C., Brindle, P., Development and validation of QRISK3 risk prediction algorithms to estimate future risk of cardiovascular disease: prospective cohort study (2017) BMJ., 357, p. j2099; Coronary CT angiography and 5-year risk of myocardial infarction (2018) N Engl J Med, 379, pp. 924-933; Steinarsson, A.O., Rawshani, A., Gudbjörnsdottir, S., Franzén, S., Svensson, A.-M., Sattar, N., Short-term progression of cardiometabolic risk factors in relation to age at type 2 diabetes diagnosis: a longitudinal observational study of 100,606 individuals from the Swedish National Diabetes Register (2018) Diabetologia., 61, pp. 599-606; Huo, L., Magliano, D.J., Rancière, F., Harding, J.L., Nanayakkara, N., Shaw, J.E., Impact of age at diagnosis and duration of type 2 diabetes on mortality in Australia 1997-2011 (2018) Diabetologia., 61, pp. 1055-1063; Sattar, N., Rawshani, A., Franzén, S., Rawshani, A., Svenson, A.-M., Rosengren, A., Age at diagnosis of type 2 diabetes mellitus and associations with cardiovascular and mortality risks (2019) Circulation., 139, pp. 2228-2237; Donnelly, L.A., Zhou, K., Doney, A.S.F., Jennison, C., Franks, P.W., Pearson, E.R., Rates of glycaemic deterioration in a real-world population with type 2 diabetes (2018) Diabetologia., 61, pp. 607-615; Huxley, R.R., Peters, S.A.E., Mishra, G.D., Woodward, M., Risk of all-cause mortality and vascular events in women versus men with type 1 diabetes: a systematic review and meta-analysis (2015) Lancet Diabetes Endocrinol., 3, pp. 198-206; Bebu, I., Braffett, B.H., Pop-Busui, R., Orchard, T.J., Nathan, D.M., Lachin, J.M., The relationship of blood glucose with cardiovascular disease is mediated over time by traditional risk factors in type 1 diabetes: the DCCT/EDIC study (2017) Diabetologia., 60, pp. 2084-2091; Risk factors for cardiovascular disease in type 1 diabetes (2016) Diabetes., 65, pp. 1370-1379; Lind, M., Svensson, A.-M., Kosiborod, M., Gudbjörnsdottir, S., Pidovic, A., Wedel, H., Glycemic control and excess

mortality in type 1 diabetes (2014) N Engl J Med., 37, pp. 1972-1982; Nathan, D.M., Cleary, P.A., Backlund, J.-Y.C., Genuth, S.M., Lachin, J.M., Orchard, T.J., Intensive diabetes treatment and cardiovascular disease in patients with type 1 diabetes (2005) N Engl J Med., 353, pp. 2643-2653; Rawshani, A., Sattar, N., Franzén, S., Rawshani, A., Hattersley, A.T., Svensson, A.M., Excess mortality and cardiovascular disease in young adults with type 1 diabetes in relation to age at onset: a nationwide, register-based cohort study (2018) Lancet., 392, pp. 477-486; 10. Cardiovascular Disease and Risk Management: Standards of Medical Care in Diabetes-2019 (2019) Diabetes Care., 42, pp. S103-S123; Cosentino, F., Grant, P.J., Aboyans, V., Bailey, C.J., Ceriello, A., Delgado, V., 2019 ESC Guidelines on diabetes, pre-diabetes, and cardiovascular diseases developed in collaboration with the EASD: The Task Force for diabetes, pre-diabetes, and cardiovascular diseases of the European Society of Cardiology (ESC) and the European Association for the Study of Diabetes (EASD) (2020) Eur Heart J., 41, pp. 255-323; Mach, F., Baigent, C., Catapano, A.L., Koskinas, K.C., Casula, M., Badimon, L., 2019 ESC/EAS Guidelines for the management of dyslipidaemias: lipid modification to reduce cardiovascular risk (2020) Eur Heart J., 41, pp. 111-188; Global, regional, and national burden of chronic kidney disease, 1990-2017: a systematic analysis for the Global Burden of Disease Study 2017 (2020) Lancet, 395, pp. 709-733; Ortiz, A., Sanchez-Niño, M.D., Crespo-Barrio, M., De-Sequera-Ortiz, P., Fernández-Giráldez, E., García-Maset, R., The Spanish Society of Nephrology (SENEFRO) commentary to the Spain GBD 2016 report: Keeping chronic kidney disease out of sight of health authorities will only magnify the problem (2019) Nefrologia., 39, pp. 29-34; Virani, S.S., Alonso, A., Benjamin, E.J., Bittencourt, M.S., Callaway, C.W., Carson, A.P., Heart Disease and Stroke Statistics-2020 Update: A Report From the American Heart Association (2020) Circulation., 141, pp. e139-e596; Whelton, P.K., Carey, R.M., Aronow, W.S., Casey, D.E., Collins, K.J., Himmelfarb, C.D., 2017 ACC/AHA/AAPA/ABC/ACPM/AGS/APhA/ASH/ASPC/NMA/PCNA Guideline for the Prevention, Detection, Evaluation, and Management of High Blood Pressure in Adults: A Report of the American College of Cardiology/American Heart Association Task Force on Clinical Practice Guidelines (2018) J Am Coll Cardiol., 71, pp. e127-e248; Williams, B., Mancia, G., Spiering, W., Agabiti Rosei, E., Azizi, M., Burnier, M., 2018 ESC/ESH guidelines for the management or arterial hypertension. The Task Force for the management of arterial hypertension of the European Society of Cardiology (ESC) and the European Society of Hypertension (ESH) (2018) Eur Heart J., 29, pp. 3021-3104; Mach, F., Baigent, C., Catapano, A.L., Koskinas, K.C., Casula, M., Badimon, L., 2019 ESC/EAS Guidelines for the management of dyslipidaemias: lipid modification to reduce cardiovascular risk: The Task Force for the management of dyslipidaemias of the European Society of Cardiology (ESC) and European Atherosclerosis Society (EAS) (2020) Eur Heart J., 41, pp. 111-188; 11. Microvascular Complications and Foot Care: Standards of Medical Care in Diabetes-2020 (2020) Diabetes Care., 43, pp. S135-S151; KDIGO clinical practice guideline for the evaluation and management of chronic kidney disease (2013) Kidney., 3, pp. 1-150; Matsushita, K., van der Velde, M., Astor, B.C., Woodward, M., Levey, A.S., de Jong, P.E., Association of estimated glomerular filtration rate and albuminuria with all-cause and cardiovascular mortality in general population cohorts: a collaborative meta-analysis (2010) Lancet., 375, pp. 2073-2081; Matsushita, K., Coresh, J., Sang, Y., Chalmers, J., Fox, C., Guallar, E., Estimated glomerular filtration rate and albuminuria for prediction of cardiovascular outcomes: a collaborative meta-analysis of individual participant data (2015) Lancet Diabetes Endocrinol., 3, pp. 514-525; Lees, J.S., Welsh, C.E., Celis-Morales, C.A., Mackay, D., Lewsey, J., Gray,

S.R., Glomerular filtration rate by differing measures, albuminuria and prediction of cardiovascular disease, mortality and end-stage kidney disease (2019) Nat Med., 25, pp. 1753-1760; (2016) Cardiovascular disease: risk assessment and reduction, including lipid modification (NICE Clinical Guideline CG181). NICE, , https://www.nice.org.uk/guidance/cg181, Disponible en:; Goff, D.C.J., Lloyd-Jones, D.M., Bennett, G., Coady, S., D'Agostino, R.B., Gibbons, R., 2013 ACC/AHA guideline on the assessment of cardiovascular risk: a report of the American College of Cardiology/American Heart Association Task Force on Practice Guidelines (2014) Circulation., 129, pp. S49-S73; Xu, S., Song, M., Xiong, Y., Liu, X., He, Y., Qin, Z., The association between periodontal disease and the risk of myocardial infarction: a pooled analysis of observational studies (2017) BMC Cardiovasc Disord., 17, p. 50; Li, C., Lv, Z., Shi, Z., Zhu, Y., Wu, Y., Li, L., Periodontal therapy for the management of cardiovascular disease in patients with chronic periodontitis (2014) Cochrane Database Syst Rev., 8. , CD009197; Garshick, M.S., Vaidean, G.D., Vani, A., Underberg, J.A., Newman, J.D., Berger, J.S., Cardiovascular risk factor control and lifestyle factors in young to middle-aged adults with newly diagnosed obstructive coronary artery disease (2019) Cardiology., 142, pp. 83-90; Medrano, M.J., Pastor-Barriuso, R., Boix, R., del Barrio, J.L., Damián, J., Álvarez, R., Riesgo coronario atribuible a los factores de riesgo cardiovascular en población española (2007) Rev Esp Cardiol., 60, pp. 1250-1256; Aranceta-Bartrina, J., Gianzo-Citores, M., Pérez-Rodrigo, C., Prevalence of overweight, obesity and abdominal obesity in the Spanish population aged 3 to 24 years The ENPE study (2020) Rev Esp Cardiol (Engl Ed)., 73, pp. 290-299; Farnier, M., Civeira, F., Descamps, O., How to implement clinical guidelines to optimise familial hypercholesterolaemia diagnosis and treatment (2017) Atheroscler Suppl., 26, pp. 25-35; Vallejo-Vaz, A.J., De Marco, M., Stevens, C.A.T., Akram, A., Freiberger, T., Hovingh, G.K., Overview of the current status of familial hypercholesterolaemia care in over 60 countries - The EAS Familial Hypercholesterolaemia Studies Collaboration (FHSC) (2018) Atherosclerosis, 277, pp. 234-255; Gijón-Conde, T., Gorostidi, M., Banegas, J.R., de la Sierra, A., Segura, J., Vinyoles, E., Documento de la Sociedad Española de Hipertensión-Liga Española para la Lucha contra la Hipertensión Arterial (SEH-LELHA) sobre monitorización ambulatoria de la presión arterial (MAPA) 2019 (2019) Hipertens Riesgo Vasc., 36, pp. 199-212; (2019) World population prospects 2019, , http://esa.un.org/unpd/wpp/DataQuery, Disponible en: /; Nanna, M.G., Navar, A.M., Wojdyla, D., Peterson, E.D., The Association Between Low-Density Lipoprotein Cholesterol and Incident Atherosclerotic Cardiovascular Disease in Older Adults: Results From the National Institutes of Health Pooled Cohorts (2019) J Am Geriatr Soc., 67, pp. 2560-2567; Bejan-Angoulvant, T., Saadatian-Elahi, M., Wright, J.M., Schron, E.B., Lindholm, L.H., Fagard, R., Treatment of hypertension in patients 80 years and older: the lower the better? A meta-analysis of randomized controlled trials (2010) J Hypertens., 28, pp. 1366-1372; Vaes, B., Depoortere, D., Van Pottelbergh, G., Matheï, C., Neto, J., Degryse, J., Association between traditional cardiovascular risk factors and mortality in the oldest old: untangling the role of frailty (2017) BMC Geriatr., 17, p. 234; Anjker, D., Santos-Eggimann, B., Santschi, V., Del Giovane, C., Wolfson, C., Streit, S., Screening and treatment of hypertension in older adults: less is more? (2018) Public Health Rev., 39, p. 26; Benetos, A., Petrovic, M., Strandberg, T., Hypertension management in older and frailolder patients (2019) Circ Res, 124, pp. 1045-1060; Williamson, J.D., Supiano, M.A., Applegate, W.B., Berlowitz, D.R., Campbell, R.C., Chertow, G.M., Intensive vs Standard Blood Pressure Control and Cardiovascular Disease Outcomes in Adults Aged ≥75 Years: A

Randomized Clinical Trial (2016) JAMA., 315, pp. 2673-2682; Pallarés-Carratalá, V., Divisón Garrote, J.A., Prieto Díaz, M.A., García Matarín, L., Seoane Vicente, M.C., Molina Escribano, F., [Positioning for the management of arterial hypertension in Primary Care from the critical analysis of the American (2017) and European (2018) guidelines. Spanish Society of Primary Care Physicians (SEMERGEN)] (2019) Semergen., 45, pp. 251-272; Gómez-Huelgas, R., Gómez Peralta, F., Rodríguez Mañas, L., Formiga, F., Puig Domingo, M., Mediavilla Bravo, J.J., Tratamiento de la diabetes mellitus tipo 2 en el paciente anciano (2018) Rev Clin Esp., 218, pp. 74-88; Efficacy and safety of statin therapy in older people: a meta-analysis of individual participant data from 28 randomised controlled trials (2019) Lancet., 393, pp. 407-415; Okaby, A.R., Gaziano, J.M., Djousse, L., Driver, J.A., Statins for Primary Prevention of Cardiovascular Events and Mortality in Older Men (2017) J Am Geriatr Soc., 65, pp. 2362-2368; Ramos, R., Comas-Cufí, M., Martí-Lluch, R., Balló, E., Ponjoan, A., Alves-Cabratosa, L., Statins for primary prevention of cardiovascular events and mortality in old and very old adults with and without type 2 diabetes: retrospective cohort study (2018) BMJ., 362, p. k3359; Giral, P., Neumann, A., Weill, A., Coste, J., Cardiovascular effect of discontinuing statins for primary prevention at the age of 75 years: a nationwide population-based cohort study in France (2019) Eur Heart J., 40, pp. 3516-3525; Timmis, A., Townsend, N., Gale, C.P., Torbica, A., Lattino, M., Petersen, S.E., European Society of Cardiology: Cardiovascular Disease Statistics 2019 (2020) Eur Heart J., 41, pp. 12-85; Groenhof, T.K.J., van Rijn, B.B., Franx, A., Roeters van Lennet, J.E., Bots, M.L., Lely, A.T., Preventing cardiovascular disease after hypertensive disorders of pregnancy: Searching for the how and when (2017) Eur J Prev Cardiol., 24, pp. 1735-1745; Douglas, P., Poppas, A., (2019) Overview of cardiovascular risk factors in women. UpToDate., , https://www.uptodate.com/contents/overview-of-cardiovascular-risk-factors-in-women#H3, Disponible en:; Stephenson, J., Heslehurst, N., Hall, J., Shoenaker, D.A.J.M., Hurtchison, J., Cade, J., Before the beginning: nutrition and lifestyle in the preconception period and its importance for future health (2018) Lancet., 391, pp. 1830-1841; Peters, S.A., Woodward, M., Women's reproductive factors and incident cardiovascular disease in the UK Biobank (2018) Heart., 104, pp. 1069-1075; Hyun, K.K., Redfern, J., Patel, A., Peiris, D., Brieger, D., Sullivan, D., Gender inequalities in cardiovascular risk factor assessment and management in primary healthcare (2017) Heart., 103, pp. 492-498; Benjamin, E.J., Blaha, M.J., Chiuve, S.E., Cushman, M., Das, S.R., Deo, L., Heart Disease and stroke statistics- 2017 update: a report from the American Heart Association (2017) Circulation., 135, pp. e146-e603; Willett, W., Rockström, J., Loken, B., Springmann, M., Lang, T., Vermeulen, S., Food in the Anthropocene: the EAT-Lancet Commission on healthy diets from sustainable food systems (2019) Lancet., 393, pp. 447-492; Health effects of dietary risks in 195 countries, 1990-2017: a systematic analysis for the Global Burden of Disease Study 2017 (2019) Lancet., 393, pp. 1958-1972; (2019) Mapping the health system response to childhood obesity in the WHO European Region: an overview and country perspectives., , https://www.euro.who.int/__data/assets/pdf_file/0019/401176/Childhood-obesity-web.pdf?ua=1, Disponible en:; (2017) Nota técnica. Encuesta Nacional de Salud España 2017, , https://www.mscbs.gob.es/estadEstudios/estadisticas/encuestaNacional/encuestaNac2017/ENSE2017_notatecnica.pdf, Disponible en:; Spinelli, A., Buoncristiano, M., Kovacs, V.A., Yngve, A., Spiroski, I., Obreja, G., Prevalence of Severe Obesity among Primary School Children in 21 European Countries (2019) Obes Facts., 12, pp. 244-258; Royo-Bordonada, M.Á., La publicidad que nos engorda

(2019) Alternativas Económicas., 66, pp. 46-47; Bronzwaer, S., Kass, G., Robinson, T., Tarazona, J., Verhagen, H., Verloo, D., Food safety regulatory research needs 2030 (2019) EFSA J., 17, p. e170622; Garde, A., Byrne, S., Gokani, N., Murphy, B., (2018) A child rights-based approach to food marketing: a guide for policy makers. United Nations Children's Fund (UNICEF), , https://www.unicef.org/csr/files/A_Child_Rights-Based_Approach_to_Food_Marketing_Report.pdf, Disponible en:; (2010) Conjunto de recomendaciones sobre la promoción de alimentos y bebidas no alcohólicas dirigida a los niños., , http://apps.who.int/iris/bitstream/10665/44422/1/9789243500218_spa.pdf, Disponible en:; (2018) Diario Oficial de la Unión Europea, , https://www.boe.es/doue/2018/303/L00069-00092.pdf, Disponible en:; (2019) Health Promotion and Disease Prevention Knowledge Gateway, , https://ec.europa.eu/jrc/en/health-knowledge-gateway, Disponible en:; Egnell, M., Talati, Z., Hercberg, S., Pettigrew, S., Julia, C., Objective understanding of front-of-package nutrition labels: an international comparative experimental study across 12 countries (2018) Nutrients., 10, p. 1542; Julia, C., Etilé, F., Hercberg, S., Front-of-pack Nutri-Score labelling in France: an evidence-based policy (2018) Lancet Public Health., 3, p. 164; Royo-Bordonada MÁ, Rodríguez-Artalejo, F., Bes-Rastrollo, M., Fernández-Escobar, C., González, C.A., Rivas, F., Políticas alimentarias para prevenir la obesidad y las principales enfermedades no transmisibles en España: querer es poder (2019) Gac Sanit., 33, pp. 584-592; (2018) Campaña por la eliminación de la publicidad de alimentos malos para la salud de la población infantil, , https://defiendeme.org/campana, Disponible en: /; Katzmarzyk, P.T., Church, T.S., Craig, C.L., Bouchard, C., Sitting time and mortality from all causes, cardiovascular disease, and cancer (2009) Med Sci Sports Exerc., 41, pp. 998-1000; Chau, J.Y., Grunseit, A.C., Chey, T., Stamatakis, E., Brown, J.E., Matthews, C.E., Daily sitting time and all-cause mortality: a meta-analysis (2013) PLoS One., 8. , e80000; Sakaue, A., Adachi, H., Enomoto, M., Fukami, A., Kumagai, E., Nakamura, S., Association between physical activity, occupational sitting time and mortality in a general population: an 18-year prospective survey in Tanushimaru, Japan (2020) Eur J Prev Cardiol., 27, pp. 758-766; Ekelund, U., Steene-Johannessen, J., Brown, W.J., Fagerland, M.W., Owen, N., Powell, K.E., Does physical activity attenuate, or even eliminate, the detrimental association of sitting time with mortality? A harmonised meta-analysis of data from more than 1 million men and women (2016) Lancet., 388, pp. 1302-1310; Berry, J.D., Willis, B., Gupta, S., Barlow, C.E., Lakoski, S.G., Khera, A., Lifetime risks for cardiovascular disease mortality by cardiorespiratory fitness levels measured at ages 45, 55, and 65 years in men: the Cooper Center Longitudinal Study (2011) J Am Coll Cardiol., 57, pp. 1604-1610; Fiuza-Luces, C., Garatachea, N., Berger, N.A., Lucia, A., Exercise is the real polypill (2013) Physiology., 28, pp. 330-358; Bronfenbrenner, U., The bioecological theory of human development (2001) International enciclopedia of the social and behavioral sciences, pp. 6963-6970. , N. Smelser P. Baltes Elsevier New York; (2019) Global action plan on physical activity 2018-2030: More active people for a healthier world., , https://www.who.int/ncds/prevention/physical-activity/global-action-plan-2018-2030/en, Disponible en: /; Piercy, K.L., Troiano, R.P., Ballard, R.M., Carlson, S.A., Fulton, J.E., Galuska, D.A., U.S. Department of Health and Human Services. Physical Activity Guidelines for Americans. 2nd ed. 2018 (2018) JAMA, 320, pp. 2020-2028; (2019) Scientific Report 2018. U.S. Department of Health and Human Services, , https://health.gov/paguidelines/second-edition/report, Disponible en:; Hunter, R.F., Christian, H., Veitch, J., Astell-But, T., Hipp, J.A., Schipperjin, J., The impact of interventions to promote physical activity

in urban green space: a systematic review and recommendations for future research (2015) Soc Sci Med., 124, pp. 246-256; (2015) Estrategia de actividad física para la región europea de la OMS 2016-2025, , http://www.cibr.es/salud-actividad-fisica-organismo-oficial-salud-actividad-fisica-organismo-oficial-22dcfc, Disponible en:; Aznar Laín, S., González López, E., Ruiz Peralta, E., Claramonte, M.T., Martín-Pérez Rodríguez, A., Ravelo Mireles, R., (2019) Guía rutas saludables. Red Española de Ciudades Saludables, , http://recs.es/hacia-rutas-saludables-guia-para-el-diseno-implementacion-y-evaluacion-de-un-plan-de-rutas-saludables, Disponible en: /; (2015) WHO Report on the Global Tobacco Epidemic, 2015: Raising Taxes on Tobacco., , https://www.who.int/tobacco/global_report/2015/en, /; Critselis, E., Panagiotakos, D.B., Georgousopoulou, E.N., Katsaounou, P., Chrysohoou, C., Pitsavos, C., Exposure to second hand smoke and 10-year (2002-2012) incidence of cardiovascular disease in never smokers: the ATTICA cohort study (2019) Int J Cardiol., 295, pp. 29-35; Pérez, M., Galán, I., Evaluación de las políticas de control del tabaquismo en España (Leyes 28/2005 y 42/2010). Revisión de la evidencia. Grupo de Trabajo sobre Tabaquismo de la Sociedad Española de Epidemiología. (2017), Parsán Gráfica Madrid; Hoffman, S.J., Poirier, M.J.P., Rogers Van Katwyk, S., Baral, P., Sritharan, L., Impact of the WHO Framework Convention on Tobacco Control on global cigarette consumption: quasi-experimental evaluations using interrupted time series analysis and in sample forecast event modelling (2019) BMJ, 365, p. l2287; Carroll, A.J., Labarthe, D.R., Huffman, M.D., Hitsman, B., Global tobacco prevention and control in relation to a cardiovascular health promotion and disease prevention framework: A narrative review (2016) Prev Med., 93, pp. 189-197; Gravely, S., Giovino, G.A., Craig, L., Commar, A., D'Espaignet, E.T., Schotte, S., Implementation of key demand-reduction measures of the WHO Framework Convention on Tobacco Control and change in smoking prevalence in 126 countries an association study (2017) Lancet., 2, pp. e166-e174; (2018) Austria's Reversal of Smoking Ban in World Spotlight, , https://www.iaslc.org/iaslc-news/ilcn/austrias-reversal-smoking-ban-world-spotlight, Disponible en:; Brook, R.D., Rajagopalan, S., Pope, C.A., 3rd, Brook, J.R., Bhatnagar, A., Diez-Roux, A.V., Particulate matter air pollution and cardiovascular disease: An update to the scientific statement from the American Heart Association (2010) Circulation., 121, pp. 2331-2378; Liu, C., Chen, R., Sera, F., Vicedo-Cabrera, A.M., Guo, Y., Tong, S., Ambient Particulate Air Pollution and Daily Mortality in 652 Cities (2019) N Engl J Med., 381, pp. 705-715; Lelieveld, J., Klingmüller, K., Pozzer, A., Pöschl, U., Fnais, M., Daiber, A., Cardiovascular disease burden from ambient air pollution in Europe reassessed using novel hazard ratio functions (2019) Eur Heart J., 40, pp. 1590-1596; Bañeras, J., Ferreira-González, I., Marsal, J.R., Barrabés, J.A., Ribera, A., Lidón, R.M., Short-term exposure to air pollutants increases the risk of ST elevation myocardial infarction and of infarct-related ventricular arrhythmias and mortality (2018) Int J Cardiol., 250, pp. 35-42; Tobías, A., Recio, A., Díaz, J., Linares, C., Health impact assessment of traffic noise in Madrid (Spain) (2015) Environ Res., 137, pp. 136-140; Karanasiou, A., Moreno, N., Moreno, T., Viana, M., de Leeuw, F., Querol, X., Health effects from Sahara dust episodes in Europe: literature review and research gaps (2012) Environ Int., 47, pp. 107-114; Nieuwenhuijsen, M.J., Influence of urban and transport planning and the city environment on cardiovascular disease (2018) Nat Rev Cardiol., 15, pp. 432-438; (2017) Plan Nacional de Calidad del AIRE 2017-2019 (Plan Aire II), , https://www.miteco.gob.es/es/calidad-y-evaluacion-ambiental/temas/atmosfera-y-c

alidad-del-aire/planaire2017-2019_tcm30-436347.pdf, Disponible en:; Leemrijse, C.J., Peters, R.J.G., von Birgelen, C., van Dijk, L., van Hal, J.M.C., Kuijper, F.M., The telephone lifestyle intervention «Hartcoach» has modest impact on coronary risk factors: a randomised multicentretrial (2016) Eur J Prev Cardiol., 23, pp. 1658-1660; Palacios, J., Lee, G.A., Duaso, M., Clifton, A., Norman, I.J., Richards, D., Internet-delivered self-management support for improving coronary heart disease and self-management-related outcomes: a systematic review (2017) J Cardiovasc Nurs., 32, pp. E9-E23; Simpson, S.H., Eurich, D.T., Majumdar, S.R., Padwal, R.S., Tsuyuki, R.T., Varney, J., A meta-analysis of the association between adherence to drug therapy and mortality (2006) BMJ., 333, p. 15; Palmer, M.J., Barnard, S., Perel, P., Free, C., Mobile phonebased interventions for improving adherence to medication prescribed for the primary prevention of cardiovascular disease in adults (2018) Cochrane Database Syst Rev., 6. , CD012675; Adler, A.J., Martin, N., Mariani, J., Tajer, C.D., Owolabi, O.O., Free, C., Mobile phone text messaging to improve medication adherence in secondary prevention of cardiovascular disease (2017) Cochrane Database Syst Rev., 4, p. CD011851; Kraus, W.E., Powell, K.E., Haskell, W.L., Janz, K.F., Campbell, W.W., Jakicic, J.M., Physical activity, all-cause and cardiovascular mortality, and cardiovascular disease (2019) Med Sci Sports Exerc., 51, pp. 1270-1281; King, A.C., Whitt-Glover, M.C., Marquez, D.X., Buman, M.P., Napolitano, M.A., Jakici, J., Physical activity promotion: highlights from the 2018 Physical Activity Guidelines Advisory Committee Systematic Review (2019) Med Sci Sports Exerc., 51, pp. 1340-1353; Sánchez Ruiz-Cabello, F.J., (2019) Promoción de la actividad física, , http://previnfad.aepap.org/recomendacion/actividad-fisica-rec, Disponible en:; Campbell, W.W., Kraus, W.E., Powell, K.E., Haskell, W.L., Janz, K.F., Jakicic, J.M., Highintensity interval training for cardiometabolic disease prevention (2019) Med Sci Sports Exerc., 51, pp. 1220-1226; (2018) 2018 Physical Activity Guidelines Advisory Committee Scientific Report, , https://health.gov/paguidelines/second-edition/report, Disponible en:; Gourlan, M., Bernard, P., Bortolon, C., Romain, A.J., Lareyre, O., Carayol, M., Efficacy of theory-based interventions to promote physical activity. A meta-analysis of randomised controlled trials (2016) Health Psychol Rev., 10, pp. 50-66; Jakicic, J.M., Davis, K.K., Rogers, R.J., King, W.C., Marcus, M.D., Helsel, D., Effect of wearable technology combined with a lifestyle intervention on long-term weight loss: the IDEA randomized clinical trial (2016) JAMA., 316, pp. 1161-1171; National Center for Chronic Disease Prevention and Health Promotion (US) Office on Smoking and Health (2014) The health consequences of smoking – 50 years of progress: a report of the Surgeon General, , Centers for Disease Control and Prevention (US) Georgia; (2020) El reto de los e-cigs y otros dispositivos de liberación de nicotina (ENDS), , http://educacionpapps.blogspot.com/2020/05/el-reto-de-los-e-cigs-y-otros.html, Disponible en:; Bullen, C., Howe, C., Laugesen, M., McRobbie, H., Parag, V., Williman, J., Electronic cigarettes for smoking cessation: a randomised controlled trial (2013) Lancet., 382, pp. 1629-1637; Galaviz, K.I., Weber, M.B., Straus, A., Haw, J.S., Narayan, K.M.V., Ali, M.K., Global diabetes prevention interventions: a systematic review and network meta-analysis of the real-world impact on incidence, weight, and glucose (2018) Diabetes Care., 41, pp. 1526-1534; Mente, A., Dehghan, M., Rangarajan, S., McQueen, M., Dagenais, G., Wielgosz, A., Association of dietary nutrients with blood lipids and blood pressure in 18 countries: a cross-sectional analysis from the PURE study (2017) Lancet Diabetes Endocrinol., 5, pp. 774-787; O'Connor, L.E., Kim, J.E., Campbell, W.W., Total red meat intake of ≥0.5 servings/d does not negatively influence cardiovascular disease risk factors: a systemically searched

meta-analysis of randomized controlled trials (2017) Am J Clin Nutr., 105, pp. 57-69; Fernández-Solà, J., Cardiovascular risks and benefits of moderate and heavy alcohol consumption (2015) Nat Rev Cardiol., 12, pp. 576-587; Wood, A.M., Kaptoge, S., Butterworth, A.S., Willeit, P., Warnakula, S., Bolton, T., Risk thresholds for alcohol consumption: combined analysis of individual-participant data for 599 912 current drinkers in 83 prospective studies (2018) Lancet., 391, pp. 1513-1523; Sabatine, M.S., Giugliano, R.P., Keech, A.C., Honarpour, N., Wiviott, S.D., Murphy, S.A., Evolocumab and clinical outcomes in patients with cardiovascular disease (2017) N Engl J Med., 376, pp. 1713-1722; Schwartz, G.G., Steg, P.G., Szarek, M., Bhatt, D.L., Bittner, V.A., Diaz, R., Alirocumab and cardiovascular outcomes after acute coronary syndrome (2018) N Engl J Med., 379, pp. 2097-2107; Ridker, P.M., Revkin, J., Amarenco, P., Brunell, R., Curto, M., Civeira, F., Cardiovascular efficacy and safety of bococizumab in high-risk patients (2017) N Engl J Med., 376, pp. 1527-1539; Bonaca, M.P., Nault, P., Giugliano, R.P., Keech, A.C., Pineda, A.L., Kanevsky, E., Low-density lipoprotein cholesterol lowering with evolocumab and outcomes in patients with peripheral artery disease: insights from the FOURIER trial (Further Cardiovascular Outcomes Research With PCSK9 Inhibition in Subjects With Elevated Risk) (2018) Circulation., 137, pp. 338-350; Jukema, J.W., Szarek, M., Zijlstra, L.E., de Silva, H.A., Bhatt, D.L., Bittner, V.A., Alirocumab in patients with polyvascular disease and recent acute coronary syndrome: ODYSSEY OUTCOMES trial (2019) J Am Coll Cardiol., 74, pp. 1167-1176; O'Donoghue, M.L., Fazio, S., Giugliano, R.P., Stroes, E.S.G., Kanevsky, E., Gouni-Berthold, I., Lipoprotein(a), PCSK9 inhibition, and cardiovascular risk (2019) Circulation., 139, pp. 1483-1492; Bittner, V.A., Szarek, M., Aylward, P.E., Bhatt, D.L., Diaz, R., Edelberg, J.M., Effect of alirocumab on lipoprotein(a) and cardiovascular risk after acute coronary syndrome (2020) J Am Coll Cardiol., 75, pp. 133-144; Nicholls, S.J., Puri, R., Anderson, T., Ballantyne, C.M., Cho, L., Kastelein, J.J., Effect of evolocumab on progression of coronary disease in statin-treated patients: the GLAGOV randomized clinical trial (2016) JAMA., 316, pp. 2373-2384; Cao, Y.X., Liu, H.H., Dong, Q.T., Li, S., Li, J.J., Effect of proprotein convertase subtilisin/kexin type 9 (PCSK9) monoclonal antibodies on new-onset diabetes mellitus and glucose metabolism: a systematic review and meta-analysis (2018) Diabetes Obes Metab., 20, pp. 1391-1398; Giugliano, R.P., Mach, F., Zavitz, K., Kurtz, C., Im, K., Kanevsky, E., Cognitive function in a randomized trial of evolocumab (2017) N Engl J Med., 377, pp. 633-643; Harvey, P.D., Sabbagh, M.N., Harrison, J.E., Ginsberg, H.N., Chapman, M.J., Manvelian, G., No evidence of neurocognitive adverse events associated with alirocumab treatment in 3340 patients from 14 randomized phase 2 and 3 controlled trials: a meta-analysis of individual patient data (2018) Eur Heart J., 39, pp. 374-381; Dobrzynski, J.M., Kostis, J.B., Sargsyan, D., Zinonos, S., Kostis, W.J., Effect of cholesterol lowering with statins or proprotein convertase subtilisin/kexin type 9 antibodies on cataracts: a meta-analysis (2018) J Clin Lipidol., 12, pp. 728-733; Faselis, C., Imprialos, K., Grassos, H., Pittaras, A., Kallistratos, M., Manolis, A., Is very low LDL-C harmful? (2018) Curr Pharm Des., 24, pp. 3658-3664; Annemans, L., Packard, C.J., Briggs, A., Ray, K.K., ’Highest risk highest benefit’ strategy: a pragmatic, cost-effective approach to targeting use of PCSK9 inhibitor therapies (2018) Eur Heart J., 39, pp. 2546-2550; Kazi, D.S., Penko, J., Coxson, P.G., Moran, A.E., Ollendorf, D.A., Tice, J.A., Updated cost-effectiveness analysis of PCSK9 inhibitors based on the results of the FOURIER trial (2017) JAMA., 318, pp. 748-750; Ascaso, J.F., Civeira, F., Guijarro, C., López Miranda, J., Masana, L., Mostaza, J.M., Indicaciones de los inhibidores de PCSK9 en la práctica clínica. Recomendaciones de la Sociedad Española de Arteriosclerosis (SEA), 2019 (2019)

Clin Investig Arterioscler., 31, pp. 128-139; Alexander, D.D., Miller, P.E., Van Elswyk, M.E., Kuratko, C.N., Bylsma, L.C., A meta-analysis of randomized controlled trials and prospective cohort studies of eicosapentaenoic and docosahexaenoic long-chain omega-3 fatty acids and coronary heart disease risk (2017) Mayo Clin Proc., 92, pp. 15-29; Abdelhamid, A.S., Brown, T.J., Brainard, J.S., Biswas, P., Thorpe, G.C., Moore, H.J., Omega-3 fatty acids for the primary and secondary prevention of cardiovascular disease (2018) Cochrane Database Syst Rev., 11, p. CD003177; Wen, Y.T., Dai, J.H., Gao, Q., Effects of Omega-3 fatty acid on major cardiovascular events and mortality in patients with coronary heart disease: a meta-analysis of randomized controlled trials (2014) Nutr Metab Cardiovasc Dis., 24, pp. 470-475; Mozaffarian, D., Rimm, E.B., Fish intake, contaminants, and human health: evaluating the risks and the benefits (2006) JAMA., 296, pp. 1885-1899; Bhatt, D.L., Steg, P.G., Miller, M., Brinton, E.A., Jacobson, T.A., Ketchum, S.B., Cardiovascular risk reduction with icosapent ethyl for hypertriglyceridemia (2019) N Engl J Med., 380, pp. 11-22; Bhatt, D.L., Steg, P.G., Miller, M., Brinton, E.A., Jacobson, T.A., Jiao, L., Reduction in first and total ischemic events with icosapent ethyl across baseline triglyceride tertiles (2019) J Am Coll Cardiol., 74, pp. 1159-1161; Perez-Calahorra, S., Laclaustra, M., Marco-Benedí, V., Lamiquiz-Moneo, I., Pedro-Botet, J., Plana, N., Effect of lipid-lowering treatment in cardiovascular disease prevalence in familial hypercholesterolemia (2019) Atherosclerosis., 284, pp. 245-252; Tuomilehto, J., Lindström, J., Eriksson, J.G., Valle, T.T., Hämäläinen, H., Ilanne-Parikka, P., Prevention of type 2 diabetes mellitus by changes in lifestyle among subjects with impaired glucose tolerance (2001) N Engl J Med., 344, pp. 1343-1350; Wing, R.R., Long term effects of a lifestyle intervention on weight and cardiovascular risk factors in individuals with type 2 diabetes mellitus: four-year results of the Look AHEAD trial (2010) Arch Intern Med., 170, pp. 1566-1575; Franz, M.J., Boucher, J.L., Rutten-Ramos, S., VanWormer, J.J., Lifestyle weight-loss intervention outcomes in overweight and obese adults with type 2 diabetes: a systematic review and meta-analysis of randomized clinical trials (2015) J Acad Nutr Diet., 115, pp. 1447-1463; Lean, M.E., Leslie, W.S., Barnes, A.C., Brosnahan, N., Thom, G., McCombie, L., Primary care-led weight management for remission of type 2 diabetes (DiRECT): an open-label, cluster-randomised trial (2018) Lancet., 391, pp. 541-551; Zelniker, T.A., Wiviott, S.D., Raz, I., Im, K., Goodrich, E.L., Furtado, R.H.M., Comparison of the effects of glucagon-like peptide receptor agonists and sodium-glucose cotransporter 2 inhibitors for prevention of major adverse cardiovascular and renal outcomes in type 2 diabetes mellitus (2019) Circulation., 139, pp. 2022-2031; Davies, M.J., D'Alessio, D.A., Fradkin, J., Kernan, W.N., Mathieu, C., Mingrone, G., Management of hyperglycemia in type 2 diabetes, 2018. A consensus report by the American Diabetes Association (ADA) and the European Association for the Study of Diabetes (EASD) (2018) Diabetes Care., 41, pp. 2669-2701; Mach, F., Baigent, C., Catapano, A.L., Koskinas, K.C., Casula, M., Badimon, L., 2019 ESC/EAS Guidelines for the management of dyslipidaemias: lipid modification to reduce cardiovascular risk (2020) Eur Heart J., 41, pp. 111-188; Gijón-Conde, T., Sánchez-Martínez, M., Graciani, A., Cruz, J.J., López-García, E., Ortolá, R., Impact of the European and American guideliness on hypertension prevalence, treatment, and cardiometabolic goals (2019) J Hypertens., 37, pp. 1393-13921400; Banegas, J.R., Ruilope, L.M., Estudio de mortalidad del Registro español de monitorización ambulatoria de la presión arterial. Una llamada a la traslación de la monitorización ambulatoria de la presión arterial a la práctica clínica (2018) Hipertens Riesgo Vasc.; Martín-Fernández, M., Vinyoles, E., Real, J., Soldevila, N., Muñoz, M.Á., Del-Val, J.L., The prognostic value

of blood pressure control delay in newly diagnosed hypertensive patients (2019) J Hypertens., 37, pp. 426-431; Vinoyles, E., El control de la hipertensión arterial: precoz y diligente (2020) Hipertens Riesgo Vasc., 37, pp. 53-55; Wright, J.T., Jr., Williamson, J.D., Whelton, P.K., Snyder, J.K., Sink, K.M., Rocco, M.V., A randomized trial of intensive versus standard blood-pressure control (2015) N Engl J Med., 373, pp. 2103-2116; Bangalore, S., Toklu, B., Gianos, E., Schwartzbard, A., Weintraub, H., Ogedegbe, G., Optimal systolic blood pressure target after SPRINT: insights from a network meta-analysis of randomized trials (2017) Am J Med., 130, pp. 707-719.e8; Salam, A., Atkins, E., Sundström, J., Hirakawa, Y., Ettehad, D., Emdin, C., Effects of blood pressure lowering on cardiovascular events, in the context of regression to the mean: a systematic review of randomized trials (2019) J Hypertens., 37, pp. 16-23; Schwalm, J.D., McCready, T., Lopez-Jaramillo, P., Yusoff, K., Attaran, A., Lamelas, P., A community-based comprehensive intervention to reduce cardiovascular risk in hypertension (HOPE 4): a cluster-randomised controlled trial (2019) Lancet., 394, pp. 1231-1242; Baigent, C., Blackwell, L., Collins, R., Emberson, J., Godwin, J., Peto, R., Aspirin in the primary and secondary prevention of vascular disease: collaborative meta-analysis of individual participant data from randomised trials (2009) Lancet., 373, pp. 1849-1860; Berger, J.S., Brown, D.L., Becker, R.C., Low-dose aspirin in patients with stable cardiovascular disease: a meta-analysis (2008) Am J Med., 121, pp. 43-49; O'Brien, C.W., Juraschek, S.P., Wee, C.C., Prevalence of aspirin use for primary prevention of cardiovascular disease in the United States: results from the 2017 National Health Interview Survey (2019) Ann Intern Med., 171, pp. 596-598; Gaziano, J.M., Brotons, C., Coppolecchia, R., Cricelli, C., Darius, H., Gorelick, P.B., Use of aspirin to reduce risk of initial vascular events in patients at moderate risk of cardiovascular disease (ARRIVE): a randomised, double-blind, placebo-controlled trial (2018) Lancet., 392, pp. 1036-1046; Bowman, L., Mafham, M., Wallendszus, K., Stevens, W., Buck, G., Barton, J., Effects of aspirin for primary prevention in persons with diabetes mellitus (2018) N Engl J Med., 379, pp. 1529-1539; McNeil, J.J., Wolfe, R., Woods, R.L., Tonkin, A.M., Donnan, G.A., Nelson, M.R., Effect of aspirin on cardiovascular events and bleeding in the healthy elderly (2018) N Engl J Med., 379, pp. 1509-1518; Selak, V., Jackson, R., Poppe, K., Wu, B., Harwood, M., Grey, C., Personalized prediction of cardiovascular benefits and bleeding harms from aspirin for primary prevention: a benefit-harm analysis (2019) Ann Intern Med., 171, pp. 529-539; Albus, C., Herrmann-Lingen, C., Jensen, K., Hackbusch, M., Münch, N., Kuncewicz, C., Additional effects of psychological interventions on subjective and objective outcomes compared with exercise-based cardiac rehabilitation alone in patients with cardiovascular disease: a systematic review and meta-analysis (2019) Eur J Prev Cardiol., 26, pp. 1035-1049; Reavell, J., Hopkinson, M., Clarkesmith, D., Lane, D.A., Effectiveness of cognitive behavioral therapy for depression and anxiety in patients with cardiovascular disease: a systematic review and meta-analysis (2018) Psychosom Med., 80, pp. 742-753; Connolly, S.J., Ezekowitz, M.D., Yusuf, S., Eikelboom, J., Oldgren, J., Parekh, A., Dabigatran versus warfarin in patients with atrial fibrillation (2009) N Engl J Med., 361, pp. 1139-1151; Patel, M.R., Mahaffey, K.W., Garg, J., Pan, G., Singer, D.E., Hacke, W., Rivaroxaban versus warfarin in nonvalvular atrial fibrillation (2011) N Engl J Med., 365, pp. 883-891; Granger, C.B., Alexander, J.H., McMurray, J.J., Lopes, R.D., Hylek, E.M., Hanna, M., Apixaban versus warfarin in patients with atrial fibrillation (2011) N Engl J Med., 365, pp. 981-992; Giugliano, R.P., Ruff, C.T., Braunwald, E., Murphy, S.A., Wiviott, S.D., Halperin, J.L., Edoxaban versus warfarin in patients with atrial fibrillation (2013) N Engl J Med., 369, pp. 2093-2104;

Gibson, C.M., Mehran, R., Bode, C., Halperin, J., Verheugt, F.W., Wildgoose, P., Prevention of bleeding in patients with atrial fibrillation undergoing PCI (2016) N Engl J Med., 375, pp. 2423-2434; Cannon, C.P., Bhatt, D.L., Oldgren, J., Lip, G.Y.H., Ellis, S.G., Kimura, T., Dual antithrombotic therapy with dabigatran after PCI in atrial fibrillation (2017) N Engl J Med., 377, pp. 1513-1524; Vranckx, P., Valgimigli, M., Eckardt, L., Tijssen, J., Lewalter, T., Gargiulo, G., Edoxaban-based versus vitamin K antagonist-based antithrombotic regimen after successful coronary stenting in patients with atrial fibrillation (ENTRUST-AF PCI): a randomised, open-label, phase 3 b trial (2019) Lancet., 394, pp. 1335-1343; Valgimigli, M., Bueno, H., Byrne, R.A., Collet, J.P., Costa, F., Jeppsson, A., 2017 ESC focused update on dual antiplatelet therapy in coronary artery disease developed in collaboration with EACTS: the Task Force for dual antiplatelet therapy in coronary artery disease of the European Society of Cardiology (ESC) and of the European Association for Cardio-Thoracic Surgery (EACTS) (2018) Eur Heart J., 39, pp. 213-260; Reavell, J., Hopkinson, M., Clarkesmith, D., Deirdre, A., Effectiveness of cognitive behavioral therapy for depression and anxiety in patients with cardiovascular disease: a systematic review and meta-analysis (2018) Psychosom Med., 80, pp. 742-753; Piepoli, M.F., Corrà, U., Adamopoulos, S., Benzer, W., Bjarnason-Wehrens, B., Cupples, M., Secondary prevention in the clinical management of patients with cardiovascular diseases. Core components, standards and outcome measures for referral and delivery: a policy statement from the cardiac rehabilitation section of the European Association for Cardiovascular Prevention & Rehabilitation. Endorsed by the Committee for Practice Guidelines of the European Society of Cardiology (2014) Eur J Prev Cardiol., 21, pp. 664-681; Kotseva, K., De Backer, G., De Bacquer, D., Rydén, L., Hoes, A., Grobbee, D., Lifestyle and impact on cardiovascular risk factor control in coronary patients across 27 countries: results from the European Society of Cardiology ESC-EORP EUROASPIRE V registry (2019) Eur J Prev Cardiol., 26, pp. 824-835; Kotseva, K., Wood, D., De Bacquer, D., Determinants of participation and risk factor control according to attendance in cardiac rehabilitation programmes in coronary patients in Europe: EUROASPIRE IV survey (2018) Eur J Prev Cardiol., 25, pp. 1242-1251; Székely, O., Lane, D.A., Lip, G.Y.H., Guideline-adherent secondary prevention post-acute coronary syndromes: the importance of patient uptake and persistence (2018) Eur Heart J., 39, pp. 2365-2367; Piek, A., Du, W., de Boer, R.A., Silljé, H.H.W., Novel heart failure biomarkers: why do we fail to exploit their potential? (2018) Crit Rev Clin Lab Sci., 55, pp. 246-263; Hao, Q., Tampi, M., O'Donnell, M., Foroutan, F., Siemieniuk, R.A., Guyatt, G., Clopidogrel plus aspirin versus aspirin alone for acute minor ischaemic stroke or high risk transient ischaemic attack: systematic review and meta-analysis (2018) BMJ., 363, p. k5108; Miller, W.L., Jaffe, A.S., Biomarkers in heart failure: the importance of inconvenient details (2016) ESC Heart Fail., 3, pp. 3-10; Wang, Y., Wang, Y., Zhao, X., Liu, L., Wang, D., Wang, C., Clopidogrel with aspirin in acute minor stroke or transient ischemic attack (2013) N Engl J Med., 369, pp. 11-19; Johnston, S.C., Easton, J.D., Farrant, M., Barsan, W., Conwit, R.A., Elm, J.J., Clopidogrel and aspirin in acute ischemic stroke and high-risk TIA (2018) N Engl J Med., 379, pp. 215-225; Thomas, R.J., Balady, G., Banka, G., Beckie, T.M., Chiu, J., Gokak, S., 2018 ACC/AHA clinical performance and quality measures for cardiac rehabilitation: a report of the American College of Cardiology/American Heart Association Task Force on Performance Measures (2018) J Am Coll Cardiol., 71, pp. 1814-1837; (2017) The BACPR Standards and Core Components for Cardiovascular Disease Prevention and Rehabilitation 2017 (3 rd edn), , https://www.bacpr.com/pages/page_box_contents.asp?pageid=791, Disponible en:;

Zaidat, O.O., Fitzsimmons, B.F., Woodward, B.K., Wang, Z., Killer-Oberpfalzer, M., Wakhloo, A., Effect of a balloon-expandable intracranial stent vs medical therapy on risk of stroke in patients with symptomatic intracranial stenosis: the VISSIT randomized clinical trial (2015) JAMA., 313, pp. 1240-1248; Derdeyn, C.P., Chimowitz, M.I., Lynn, M.J., Fiorella, D., Turan, T.N., Janis, L.S., Aggressive medical treatment with or without stenting in high-risk patients with intracranial artery stenosis (SAMMPRIS): the final results of a randomised trial (2014) Lancet., 383, pp. 333-341; Diener, H.C., Bogousslavsky, J., Brass, L.M., Cimminiello, C., Csiba, L., Kaste, M., Aspirin and clopidogrel compared with clopidogrel alone after recent ischaemic stroke or transient ischaemic attack in high-risk patients (MATCH): randomised, double-blind, placebo-controlled trial (2004) Lancet., 364, pp. 331-337; Hankey, G.J., Johnston, S.C., Easton, J.D., Hacke, W., Mas, J.L., Brennan, D., Effect of clopidogrel plus ASA vs. ASA early after TIA and ischaemic stroke: a substudy of the CHARISMA trial (2011) Int J Stroke., 6, pp. 3-9; Patti, G., Sticchi, A., Bisignani, A., Pelliccia, F., Pasceri, V., Speciale, G., Meta-regression to identify patients deriving the greatest benefit from dual antiplatelet therapy after stroke or transient ischemic attack without thrombolytic or thrombectomy treatment (2019) Am J Cardiol., 124, pp. 627-635; Armario, P., Jericó, J., Beneficios de la reducción del colesterol LDL en la prevención secundaria del ictus isquémico. Nuevas evidencias (2020) Clin Investig Arterioscler.; Palacio-Portilla, E.J., Roquer, J., Amaro, S., Arenillas, J.F., Ayo-Martín, O., Castellanos, M., Dislipidemias y prevención del ictus: Recomendaciones del Grupo de Estudio de Enfermedades Cerebrovasculares de la Sociedad Española de Neurología (2020) Neurologia., , En prensa; Amarenco, P., Kim, J.S., Labreuche, J., Charles, H., Giroud, M., Lee, B.C., Benefit of targeting a LDL (Low-Density Lipoprotein) cholesterol <70mg/dL during 5 years after ischemic stroke (2020) Stroke., 51, pp. 1231-1239; Amarenco, P., Kim, J.S., Labreuche, J., Charles, H., Abtan, J., Béjot, Y., A comparison of two LDL cholesterol targets after ischemic stroke (2020) N Engl J Med., 382, p. 9; Cannon, C.P., Blazing, M.A., Giugliano, R.P., McCagg, A., White, J.A., Theroux, P., Ezetimibe added to statin therapy after acute coronary syndromes (2015) N Engl J Med., 372, pp. 2387-2397; Sabatine, M.S., Giugliano, R.P., Keech, A.C., Honarpour, N., Wiviott, S.D., Murphy, S.A., Evolocumab and clinical outcomes in patients with cardiovascular disease (2017) N Engl J Med., 376, pp. 1713-1722; Schwartz, G.G., Steg, P.G., Szarek, M., Bhatt, D.L., Bittner, V.A., Diaz, R., Alirocumab and cardiovascular outcomes after acute coronary syndrome (2018) N Engl J Med., 379, pp. 2097-2107; Bohula, E.A., Wiviott, S.D., Giugliano, R.P., Blazing, M.A., Park, J.G., Murphy, S.A., Prevention of stroke with the addition of ezetimibe to statin therapy in patients with acute coronary syndrome in IMPROVE-IT (Improved Reduction of Outcomes: Vytorin Efficacy International Trial) (2017) Circulation., 136, pp. 2440-2450; Giugliano, R.P., Pedersen, T.R., Saver, J.L., Sever, P.S., Keech, A.C., Bohula, E.A., Stroke prevention with the PCSK9 (proprotein convertase subtilisin-kexin type 9) inhibitor evolocumab added to statin in high-risk patients with stable atherosclerosis (2020) Stroke., 51, pp. 1546-1554; Wang, N., Fulcher, J., Abeysuriya, N., Park, L., Kumar, S., Di Tanna, G.L., Intensive LDL cholesterol-lowering treatment beyond current recommendations for the prevention of major vascular events: a systematic review and meta-analysis of randomised trials including 327 037 participants (2020) Lancet Diabetes Endocrinol., 8, pp. 36-49; Conte, M.S., Bradbury, A.W., Kolh, P., White, J.V., Dick, F., Fitridge, R., Global vascular guidelines on the management of chronic limb-threatening ischemia (2019) J Vasc Surg., 69, pp. 3S-125S; Lin, J.S., Evans, C.V., Johnson, E., Redmond, N., Coppola, E.L., Smith, N., Nontraditional risk factors in cardiovascular disease

risk assessment: updated evidence report and systematic review for the US Preventive Services Task Force (2018) JAMA., 320, pp. 281-297; Anand, S.S., Bosch, J., Eikelboom, J.W., Connolly, S.J., Diaz, R., Widimsky, P., Rivaroxaban with or without aspirin in patients with stable peripheral or carotid artery disease: an international, randomised, double-blind, placebo-controlled trial (2018) Lancet., 391, pp. 219-229; Bonaca, M.P., Bhatt, D.L., Storey, R.F., Steg, P.G., Cohen, M., Kuder, J., Ticagrelor for prevention of ischemic events after myocardial infarction in patients with peripheral artery disease (2016) J Am Coll Cardiol., 67, pp. 2719-2728; Bonaca, M.P., Nault, P., Giugliano, R.P., Keech, A.C., Pineda, A.L., Kanevsky, E., Low-density lipoprotein cholesterol lowering with evolocumab and outcomes in patients with peripheral artery disease: insights from the FOURIER trial (Further Cardiovascular Outcomes Research With PCSK9 Inhibition in Subjects With Elevated Risk) (2018) Circulation., 137, pp. 338-350350; Frederix, I., Vandijck, D., Hens, N., De Sutter, J., Dendale, P., Economic and social impact of increased cardiac rehabilitation uptake and cardiac telerehabilitation in Belgium - a cost-benefit analysis (2018) Acta Cardiol., 73, pp. 222-229; Jin, K., Khonsari, S., Gallagher, R., Gallagher, P., Clark, A.M., Freedman, B., Telehealth interventions for the secondary prevention of coronary heart disease: a systematic review and meta-analysis (2019) Eur J Cardiovasc Nurs., 18, pp. 260-271; Coorey, G.M., Neubeck, L., Mulley, J., Redfern, J., Effectiveness, acceptability and usefulness of mobile applications for cardiovascular disease self-management: systematic review with meta-synthesis of quantitative and qualitative data (2018) Eur J Prev Cardiol., 25, pp. 505-521; Janssen, A., Wagenaar, K.P., Dendale, P., Grobbee, D.E., Accreditation of clinical centres providing primary prevention, secondary prevention and rehabilitation, and sports cardiology: a step towards optimizing quality (2019) Eur J Prev Cardiol., 26, pp. 1775-1777},
correspondence_address1={Armario, P.; Sociedad Española-Liga Española para la Lucha contra la Hipertensión ArterialSpain; email: parmariog@gmail.com},
publisher={Elsevier Doyma},
issn={02149168},
pubmed_id={33069629},
language={English; Spanish},
abbrev_source_title={Clin. Invest. Arterioscler.},
document_type={Article},
source={Scopus},
}

@ARTICLE{Herring2021,
author={Herring, L.Y. and Dallosso, H. and Schreder, S. and Smith, E.J. and Waheed, G. and Gray, L.J. and Khunti, K. and Yates, T. and Highton, P.J. and Rowlands, A.V. and Hudson, I. and Seidu, S. and Davies, M.J.},
title={Physical activity after cardiac EventS (PACES): A group education programme with subsequent text message support designed to increase physical activity in individuals with diagnosed coronary heart disease: A randomised controlled trial},
journal={Open Heart},
year={2021},
volume={8},
number={1},
doi={10.1136/openhrt-2020-001351},
art_number={e001351},
note={cited By 0},

url={https://www.scopus.com/inward/record.uri?eid=2-s2.0-85101805697&doi=10.1136%2fopenhrt-2020-001351&partnerID=40&md5=bfac1f52c84087bf774bc4184ccf1d9e},
affiliation={Leicester Diabetes Centre, University Hospitals of Leicester NHS Trust, Leicester, United Kingdom; Institute of Psychological Research, De Montfort University, Leicester, United Kingdom; Diabetes Research Centre, College of Life Sciences, University of Leicester, Leicester, United Kingdom; Department of Heath Sciences, College of Life Sciences, University of Leicester, Leicester, United Kingdom; Glenfield Hospital, University Hospitals of Leicester NHS Trust, Leicester, United Kingdom},
abstract={Aim To assess the effectiveness of a low-cost pragmatic intervention (structured education and ongoing text message support) to increase daily physical activity in participants 12-48 months after a coronary heart disease cardiac event (myocardial infarction, angina or acute coronary syndrome) diagnosis. Methods A single-centre randomised controlled trial of 291 adults randomised to a structured education programme (n=145) or usual care (n=146). The programme consisted of two 2.5 hour sessions delivered 2 weeks apart, followed by supplementary text message support. The GENEActiv accelerometer assessed the primary outcome at 12 months (change in overall physical activity (expressed in milli gravitational (mg) units) from baseline). Secondary outcomes included anthropometric, physical function, cardiovascular, biochemical and patient-reported outcome measures. Linear regression was used to compare outcome measures between groups on a modified intention-to-treat basis. Results Participants' mean age was 66.5±9.7 years, 84.5% males, 82.5% white British and 15.5% south Asian. At 12 months, there was no difference between the groups in terms of change in overall physical activity (-0.23 mg (95% CI -1.22 to 0.75), p=0.64) and the programme was well accepted (88% attendance). Exploratory analyses showed that average moderate to vigorous physical activity (MVPA) levels increased in individuals not meeting physical activity guidelines (≥150 min per week) on enrolment compared with those who did, by 8 minutes per day (8.04 (95% CI 0.99 to 15.10), p=0.03). Conclusion The programme was well attended but showed no change in physical activity levels. Results show high baseline MVPA levels and suggest that Physical Activity after Cardiac EventS education may benefit cardiac patients not currently meeting activity guidelines. Trial registration number ISRCTN91163727. © 2021 BMJ Publishing Group. All rights reserved.},
author_keywords={cardiac rehabilitation; coronary artery disease; education; public health; risk factors},
references={Darden, D., Richardson, C., Jackson, E.A., Physical activity and exercise for secondary prevention among patients with cardiovascular disease (2013) Curr Cardiovasc Risk Rep, 7, pp. 411-416; Varghese, T., Schultz, W.M., McCue, A.A., Physical activity in the prevention of coronary heart disease: Implications for the clinician (2016) Heart, 102, pp. 904-909; Anderson, L., Thompson, D.R., Oldridge, N., Exercise-based cardiac rehabilitation for coronary heart disease (2016) Cochrane Database Syst Rev, p. CD001800; (2018), British Heart Foundation. The national audit of cardiac rehabilitation (NACR) Quality and outcomes Report 2018; Fell, J., Dale, V., Doherty, P., Does the timing of cardiac rehabilitation impact fitness outcomes? (2016) An Observational Analysis. Open Heart, 3, p. e000369; Ter Hoeve, N., Huisstede, B.M.A., Stam, H.J., Does cardiac rehabilitation after an acute cardiac syndrome lead to changes in physical activity habits?Systematic review (2015) Phys Ther, 95, pp. 167-179; Herring, L.Y., Dallosso, H., Chatterjee, S., Physical activity after cardiac events (paces)-A group education programme with subsequent text-message support designed to increase physical activity in individuals with diagnosed coronary heart disease: Study protocol for a randomised controlled

trial (2018) Trials, 19, p. 537; Davies, M.J., Gray, L.J., Troughton, J., A community based primary prevention programme for type 2 diabetes integrating identification and lifestyle intervention for prevention: The Let's Prevent Diabetes cluster randomised controlled trial (2016) Prev Med, 84, pp. 48-56; Chow, C.K., Redfern, J., Hillis, G.S., Effect of Lifestyle-Focused text messaging on risk factor modification in patients with coronary heart disease: A randomized clinical trial (2015) Jama, 314, pp. 1255-1263; Park, L.G., Howie-Esquivel, J., Chung, M.L., A text messaging intervention to promote medication adherence for patients with coronary heart disease: A randomized controlled trial (2014) Patient Educ Couns, 94, pp. 261-268; Kim, B.H., Glanz, K., Text messaging to motivate walking in older African Americans: A randomized controlled trial (2013) Am J Prev Med, 44, pp. 71-75; Singh, S.J., Jones, P.W., Evans, R., Minimum clinically important improvement for the incremental shuttle walking test (2008) Thorax, 63, pp. 775-777; (2014), British Heart Foundation. Put your heart into walking. London: British heart Foundation (BHF); Redfern, J., Thiagalingam, A., Jan, S., Development of a set of mobile phone text messages designed for prevention of recurrent cardiovascular events (2014) Eur J Prev Cardiol, 21, pp. 492-499; Golubic, R., May, A.M., Benjaminsen Borch, K., Validity of electronically administered recent physical activity questionnaire (RPAQ) in ten European countries (2014) PLoS. One, 9, p. e92829; Herrmann, C., International experiences with the Hospital Anxiety and Depression Scale-A review of validation data and clinical results (1997) J Psychosom Res, 42, pp. 17-41; (2016), http://www.euroqol.org/about-eq-5d/how-to-use-eq-5d.html, EuroQol Research Foundation. How to use EQ-5D; Resnick, B., Jenkins, L.S., Testing the reliability and validity of the self-efficacy for exercise scale (2000) Nurs Res, 49, pp. 154-159; Valenti, L., Lim, L., Heller, R.F., An improved questionnaire for assessing quality of life after acute myocardial infarction (1996) Qual Life Res, 5, pp. 151-161; Bell, J.A., Hamer, M., Van Hees, V.T., Healthy obesity and objective physical activity (2015) Am J Clin Nutr, 102, pp. 268-275; Migueles, J.H., Rowlands, A.V., Huber, F., Ggir: A research community-driven open source r package for generating physical activity and sleep outcomes from multi-day raw accelerometer data (2019) J Meas Phys Behav, 2, pp. 188-196; Hildebrand, M., Van Hees, V.T., Hansen, B.H., Age group comparability of raw accelerometer output from wrist-and hip-worn monitors (2014) Med Sci Sports Exerc, 46, pp. 1816-1824; Rubin, D.B., Inference and missing data (1976) Biometrika, 63, pp. 581-592; Dibben, G.O., Dalal, H.M., Taylor, R.S., Cardiac rehabilitation and physical activity: Systematic review and meta-analysis (2018) Heart, 104, pp. 1394-1402; De Souto Barreto, P., Ferrandez, A.-M., Saliba-Serre, B., Are older adults who volunteer to participate in an exercise study fitter and healthier than nonvolunteers? The participation bias of the study population (2013) J Phys Act Health, 10, pp. 359-367; Hur, S.A., Guler, S.A., Khalil, N., Minimal important difference for physical activity and validity of the International physical activity questionnaire in interstitial lung disease (2019) Ann Am Thorac Soc, 16, pp. 107-115; Campo, G., Tonet, E., Chiaranda, G., Exercise intervention improves quality of life in older adults after myocardial infarction: Randomised clinical trial (2020) Heart, 106, pp. 1658-1664; Shepherd, C.W., While, A.E., Cardiac rehabilitation and quality of life: A systematic review (2012) J Nurs Stud, 49, pp. 755-771; De Silva, D., Helping measure person-centred care: A review of evidence about commonly used approaches and tools used to help measure person-centred care (2014) The health Foundation Inspiring improvement, , https://www.health.org.uk/publications/helping-measure-person-centred-care; Winkley, K., Stahl, D., Amiel, S.A., A multi-level model to explain attendance at structured diabetes education for people with newly diagnosed type 2

diabetes (2014) Eur J Public Health, 24; NHS Digital. National diabetes audit report 1 Care processes and treatment targets 2017-18. UK, 2018; Yates, T., Haffner, S.M., Schulte, P.J., Association between change in daily ambulatory activity and cardiovascular events in people with impaired glucose tolerance (navigator trial): A cohort analysis (2014) Lancet, 383, pp. 1059-1066},
correspondence_address1={Herring, L.Y.; Leicester Diabetes Centre, United Kingdom; email: louisa.herring@uhl-tr.nhs.uk},
publisher={BMJ Publishing Group},
issn={2398595X},
language={English},
abbrev_source_title={Open Heart},
document_type={Article},
source={Scopus},
}

@ARTICLE{Silva2021,
author={Silva, T.D.D. and Oliveira, P.M.D. and Dionizio, J.B. and Santana, A.P.D. and Bahadori, S. and Dias, E.D. and Ribeiro, C.M. and Gomes, R.D.A. and Ferreira, M. and Ferreira, C. and Moraes, Í.A.P.D. and Silva, D.M.M. and Barnabé, V. and Araújo, L.V.D. and Santana, H.B.R. and Monteiro, C.B.D.M.},
title={Comparison Between Conventional Intervention and Non-immersive Virtual Reality in the Rehabilitation of Individuals in an Inpatient Unit for the Treatment of COVID-19: A Study Protocol for a Randomized Controlled Crossover Trial},
journal={Frontiers in Psychology},
year={2021},
volume={12},
doi={10.3389/fpsyg.2021.622618},
art_number={622618},
note={cited By 0},
url={https://www.scopus.com/inward/record.uri?eid=2-s2.0-85102370921&doi=10.3389%2ffpsyg.2021.622618&partnerID=40&md5=787f5f6d5fd3a85582b21e7b281b5be0},
affiliation={Departamento de Medicina (Cardiologia), Escola Paulista de Medicina da Universidade Federal de São Paulo (UNIFESP), São Paulo, Brazil; Grupo de Pesquisa e Aplicações Tecnológicas em Reabilitação da Escola de Artes, Ciências e Humanidades da Universidade de São Paulo (PATER EACH USP), São Paulo, Brazil; Faculdade de Medicina da, Universidade Cidade de São Paulo (UNICID), São Paulo, Brazil; Serviço de Fisioterapia do Hospital São Paulo, Universidade Federal de São Paulo (HSP UNIFESP), São Paulo, Brazil; Executive Business Centre, Orthopaedic Research Institute, Bournemouth University, Bournemouth, United Kingdom},
abstract={Background: The new human coronavirus that leads to COVID-19 (coronavirus disease 2019) has spread rapidly around the world and has a high degree of lethality. In more severe cases, patients remain hospitalized for several days under treatment of the health team. Thus, it is important to develop and use technologies with the aim to strengthen conventional therapy by encouraging movement, physical activity, and improving cardiorespiratory fitness for patients. In this sense, therapies for exposure to virtual reality (VR) are promising and have been shown to be an adequate and equivalent alternative to conventional exercise programs. Aim: This is a study protocol with the aim of comparing the conventional physical therapy intervention with the use of a non-immersive VR software during COVID-19 hospitalization. Methods: Fifty patients hospitalized with confirmed diagnosis of COVID-19 will be divided in two groups under physiotherapy treatment using conventional or VR

intervention: Group A: participants with COVID-19 will start the first day of the protocol with VR tasks in the morning and then in the second period, in the afternoon, will perform the conventional exercises (n = 25) and Group B: participants with COVID-19 will start the first day with conventional exercises in the morning and in the second period, in the afternoon, will perform activity with VR (n = 25). All participants will be evaluated with different motor and physiologic scales before and after the treatment to measure improvements. Conclusion: Considering the importance of benefits from physical activity during hospitalization, VR software shows promise as a potential mechanism for improving physical activity. The results of this study may provide new insights into hospital rehabilitation. Trial Registration: ClinicalTrials.gov, identifier: NCT04537858. Registered on 01 September 2020. © Copyright © 2021 Silva, Oliveira, Dionizio, Santana, Bahadori, Dias, Ribeiro, Gomes, Ferreira, Ferreira, Moraes, Silva, Barnabé, Araújo, Santana and Monteiro.},
author_keywords={autonomic nervous system; coronavirus; hospitals; physical functional performance; rehabilitation; telerehabilitation; virtual reality exposure therapy},
references={Heart rate variability: standards of measurement, physiological interpretation and clinical use (1996) Circulation, 93, pp. 1043-1065. , Task force of the European Society of Cardiology and the North American Society of pacing and electrophysiology; Abushakra, A., Faezipour, M., Augmenting breath regulation using a mobile driven virtual reality therapy framework (2013) IEEE J. Biomed. Health Inform, 18, pp. 746-752. , 24043409; Alankus, G., Lazar, A., May, M., Kelleher, C., “Towards customizable games for stroke rehabilitation,” (2010) Proceedings of the SIGCHI Conference on Human Factors in Computing Systems, pp. 2113-2122. , Atlanta, GA; Alvarez, M.P.B., Silva, T.D.D., Favero, F.M., Valenti, V.E., Raimundo, R.D., Vanderlei, L.C.M., Autonomic modulation in duchenne muscular dystrophy during a computer task: a prospective control trial (2017) PLoS ONE, 12, p. e0169633. , 28118369; Baig, A.M., Khaleeq, A., Ali, U., Syeda, H., Evidence of the COVID-19 virus targeting the CNS: tissue distribution, host–virus interaction, and proposed neurotropic mechanisms (2020) ACS Chem. Neurosci, 11, pp. 995-998. , 32167747; Bohannon, R.W., Reference values for the timed up and go test: a descriptive meta-analysis (2006) J. Geriatr. Phys. Ther, 29, pp. 64-68. , 16914068; Boldrini, P., Bernetti, A., Fiore, P., Impact of COVID-19 outbreak on rehabilitation services and physical and rehabilitation medicine physicians' activities in Italy (2020) Eur. J. Phys. Rehabil. Med, 56, pp. 316-318. , 32175719; Bond, S., Laddu, D.R., Ozemek, C., Lavie, C.J., Arena, R., Exergaming and virtual reality for health: implications for cardiac rehabilitation (2019) Curr. Prob. Cardiol, 46, p. 100472. , 31606141; Bonnechère, B., Jansen, B., Omelina, L., Van Sint Jan, S., The use of commercial video games in rehabilitation: a systematic review (2016) Int. J. Rehabil. Res, 39, pp. 277-290. , 27508968; Brokelman, R.B., Haverkamp, D., van Loon, C., Hol, A., van Kampen, A., Veth, R., The validation of the visual analogue scale for patient satisfaction after total hip arthroplasty (2012) Eur. Orthopaed. Traumatol, 3, pp. 101-105. , 22798966; Chan, A.W., Tetzlaff, J.M., GØtzsche, P.C., Altman, D.G., Mann, H., Berlin, J.A., SPIRIT 2013 explanation and elaboration: guidance for protocols of clinical trials (2013) BMJ, 346, p. e7586. , b, 23303884; Chan, A.W., Tetzlaff, J.M., Altman, D.G., Dickersin, K., Moher, D., SPIRIT 2013: new guidance for content of clinical trial protocols (2013) Lancet, 381, pp. 91-92. , a, 23305999; Chen, N., Zhou, M., Dong, X., Qu, J., Gong, F., Han, Y., Epidemiological and clinical characteristics of 99 cases of 2019 novel coronavirus pneumonia in Wuhan, China: a descriptive study (2020) Lancet, 395, pp. 507-513. , 32007143; Coraci,

D., Fusco, A., Frizziero, A., Giovannini, S., Biscotti, L., Padua, L., Global approaches for global challenges: the possible support of rehabilitation in the management of COVID-19 (2020) J. Med. Virol, 92, pp. 1739-1740. , 32243596; Corman, V.M., Landt, O., Kaiser, M., Molenkamp, R., Meijer, A., Chu, D.K., Detection of 2019 novel coronavirus (2019-nCoV) by real-time RT-PCR (2020) Eurosurveillance, 25, p. 2000045. , 31992387; Craig, P., Dieppe, P., Macintyre, S., Michie, S., Nazareth, I., Petticrew, M., Developing and evaluating complex interventions: the new Medical Research Council guidance (2008) Int. J. Nurs. Stud, 50, pp. 587-592. , 23159157; Crocetta, T.B., de Araújo, L.V., Guarnieri, R., Massetti, T., Ferreira, F.H.I.B., De Abreu, L.C., Virtual reality software package for implementing motor learning and rehabilitation experiments (2018) Virtual Reality, 22, pp. 199-209; da Silva, T.D., Fontes, A.M.G.G., de Oliveira-Furlan, B.S., Roque, T.T., Lima, A.I.I., de Souza, B.M.M., Effect of combined therapy of virtual reality and transcranial direct current stimulation in children and adolescents with cerebral palsy: a study protocol for a triple-blinded randomized controlled crossover trial (2020) Front. Neurol, 11, p. 953. , a, 32982950; da Silva, T.D., Massetti, T., Crocetta, T.B., Monteiro, C.B.M., Carll, A., Vanderlei, L.C.M., Heart rate variability and cardiopulmonary dysfunction in patients with duchenne muscular dystrophy: a systematic review (2018) Pediatr. Cardiol, 39, pp. 869-883. , 29696428; da Silva, T.D., Ribeiro-Papa, D.C., Coe, S., Malheiros, S.R.P., Massetti, T., Meira Junior, C.M., Evaluation of speed-accuracy trade-off in a computer task to identify motor difficulties in individuals with Duchenne Muscular Dystrophy-a cross-sectional study (2020) Res. Dev. Disabil, 96, p. 103541. , b, 31830680; de Freitas, B.L., da Silva, T.D., Crocetta, T.B., Massetti, T., de Araújo, L.V., Coe, S., Analysis of different device interactions in a virtual reality task in individuals with Duchenne muscular dystrophy—a randomized controlled trial (2019) Front. Neurol, 10, p. 24. , 30761066; de Moraes, Í.A.P., Monteiro, C.B.D.M., Silva, T.D.D., Massetti, T., Crocetta, T.B., de Menezes, L.D.C., Motor learning and transfer between real and virtual environments in young people with autism spectrum disorder: a prospective randomized cross over controlled trial (2020) Autism Res, 13, pp. 307-319. , 31566888; De Wit, E., Van Doremalen, N., Falzarano, D., Munster, V.J., SARS and MERS: recent insights into emerging coronaviruses (2016) Nat. Rev. Microbiol, 14, p. 523. , 27344959; Dias, P., Silva, R., Amorim, P., Lains, J., Roque, E., Serôdio, I., Using virtual reality to increase motivation in poststroke rehabilitation (2019) IEEE Comput. Graphi. Appl, 39, pp. 64-70. , 30869599; Diccini, S., Pereira, E.M., Im, S.Y., Shida, L.Y., Bettencourt, A.R., Evaluation of pulse oximetry measurements in healthy subjects with nail polish (2011) Acta Paul. Enferm, 24, pp. 784-788; Eichhorn-Kissel, J., Dassen, T., Lohrmann, C., Comparison of the responsiveness of the care dependency scale for rehabilitation and the Barthel Index (2011) Clin. Rehabil, 25, pp. 760-767. , 21402648; Farhat, F., Hsairi, I., Baati, H., Smits-Engelsman, B.C.M., Masmoudi, K., Mchirgui, R., The effect of a motor skills training program in the improvement of practiced and non-practiced tasks performance in children with developmental coordination disorder (DCD) (2016) Hum. Mov. Sci, 46, pp. 10-22. , 26703915; Fernani, D.C.G.L., Prado, M.T.A., da Silva, T.D., Massetti, T., de Abreu, L.C., Magalhães, F.H., Evaluation of speed-accuracy trade-off in a computer task in individuals with cerebral palsy: a cross-sectional study (2017) BMC Neurol, 17, pp. 1-9. , 28750603; Ford, C.G., Manegold, E.M., Randall, C.L., Aballay, A.M., Duncan, C.L., Assessing the feasibility of implementing low-cost virtual reality therapy during routine burn care (2018) Burns, 44, pp. 886-895. , 29305105; García-Bravo, S., Cuesta-Gómez, A., Campuzano-Ruiz, R., López-Navas, M.J., Domínguez-Paniagua, J., Araújo-Narváez,

A., Virtual reality and video games in cardiac rehabilitation programs. A systematic review (2019) Disabil. Rehabil, pp. 1-10. , 31258015; Glen, K., Eston, R., Loetscher, T., Parfitt, G., Exergaming: feels good despite working harder (2017) PLoS ONE, 12, p. e0186526. , 29059227; Gomez, D.H., Bagley, J.R., Bolter, N., Kern, M., Lee, C.M., Metabolic cost and exercise intensity during active virtual reality gaming (2018) Games Health J, 7, pp. 310-316. , 30325233; Gosselink, R., Bott, J., Johnson, M., Dean, E., Nava, S., Norrenberg, M., Physiotherapy for adult patients with critical illness: recommendations of the European Respiratory Society and European Society of Intensive Care Medicine Task Force on physiotherapy for critically ill patients (2008) Intensive Care Med, 34, pp. 1188-1199. , 18283429; Guan, W.J., Ni, Z.Y., Hu, Y., Liang, W.H., Ou, C.Q., He, J.X., Clinical characteristics of coronavirus disease 2019 in China (2020) New Engl. J. Med, 382, pp. 1708-1720; Hagerty, S.L., Williams, L.M., The impact of COVID-19 on mental health: the interactive roles of brain biotypes and human connection (2020) Brain Behav. Immun. Health, 5, p. 100078; Herman, T., Giladi, N., Hausdorff, J.M., Properties of the ‘timed up and go'test: more than meets the eye (2011) Gerontology, 57, pp. 203-210. , 20484884; Hermann, M., Pekacka-Egli, A.M., Witassek, F., Baumgaertner, R., Schoendorf, S., Spielmanns, M., Feasibility and efficacy of cardiopulmonary rehabilitation following COVID-19 (2020) Am. J. Phys. Med. Rehabil, 99, pp. 865-869; Hoffman, A.J., Brintnall, R.A., Brown, J.K., Von Eye, A., Jones, L.W., Alderink, G., Virtual reality bringing a new reality to postthoracotomy lung cancer patients via a home-based exercise intervention targeting fatigue while undergoing adjuvant treatment (2014) Cancer Nurs, 37, pp. 23-33. , 23348662; Houlden, H., Edwards, M., McNeil, J., Greenwood, R., Use of the Barthel Index and the Functional Independence Measure during early inpatient rehabilitation after single incident brain injury (2006) Clin. Rehabil, 20, pp. 153-159. , 16541936; Huang, C., Wang, Y., Li, X., Ren, L., Zhao, J., Hu, Y., Clinical features of patients infected with 2019 novel coronavirus in Wuhan, China (2020) Lancet, 395, pp. 497-506. , 32502551; Jelsma, D., Geuze, R.H., Mombarg, R., Smits-Engelsman, B.C., The impact of Wii Fit intervention on dynamic balance control in children with probable Developmental Coordination Disorder and balance problems (2014) Hum. Mov. Sci, 33, pp. 404-418. , 24444657; Jung, T., Moorhouse, N., Shi, X., Amin, M.F., A virtual reality–supported intervention for pulmonary rehabilitation of patients with chronic obstructive pulmonary disease: mixed methods study (2020) J. Med. Int. Res, 22, p. e14178. , 32673224; Karloh, M., Sousa Matias, T., Fleig Mayer, A., The COVID-19 pandemic confronts the motivation fallacy within pulmonary rehabilitation programs (2020) COPD, 17, pp. 343-345. , 32657171; Kear, B.M., Guck, T.P., McGaha, A.L., Timed Up and Go (TUG) test: normative reference values for ages 20 to 59 years and relationships with physical and mental health risk factors (2017) J. Primary Care Commun. Health, 8, pp. 9-13. , 27450179; Leal, A.F., da Silva, T.D., Lopes, P.B., Bahadori, S., de Araújo, L.V., da Costa, M.V.B., The use of a task through virtual reality in cerebral palsy using two different interaction devices (concrete and abstract)–a cross-sectional randomized study (2020) J. Neuro Eng. Rehabil, 17, pp. 1-10. , 32349752; Lew, H.L., Oh-Park, M., Cifu, D.X., The war on COVID-19 pandemic: role of rehabilitation professionals and hospitals (2020) Am. J. Phys. Med. Rehabil, 99, pp. 571-572. , 32371624; Madabhavi, I., Sarkar, M., Kadakol, N., COVID-19: a review (2020) Monaldi Arch. Chest Dis, 90, pp. 248-258; Madjid, M., Safavi-Naeini, P., Solomon, S.D., Vardeny, O., Potential effects of coronaviruses on the cardiovascular system: a review (2020) JAMA Cardiol, 5, pp. 831-840. , 32219363; Martins, F.P.A., Massetti, T., Crocetta, T.B., Lopes, P.B., da Silva, A.A., Figueiredo, E.F., Analysis of motor performance in individuals with cerebral palsy using a

non-immersive virtual reality task–a pilot study (2019) Neuropsychiatric Dis. Treat, 15, pp. 417-428. , 30787616; Mazzoleni, S., Montagnani, G., Vagheggini, G., Buono, L., Moretti, F., Dario, P., Ambrosino, N., Interactive videogame as rehabilitation tool of patients with chronic respiratory diseases: preliminary results of a feasibility study (2014) Respir. Med, 108, pp. 1516-1524. , 25087837; Moraes, I.A., Silva, T.D., Massetti, T., Menezes, L.D., Ribeiro, V.F., Tropiano, L.M., Fractal correlations and linear analyses of heart rate variability in healthy young people with different levels of physical activity (2019) Cardiol. Young, 29, pp. 1236-1242. , 31475643; Novak, S., Johnson, J., Greenwood, R., Barthel revisited: making guidelines work (1996) Clin. Rehabil, 10, pp. 128-134; Paules, C.I., Marston, H.D., Fauci, A.S., Coronavirus infections—more than just the common cold (2020) JAMA, 323, pp. 707-708. , 31971553; Polechoński, J., Debska, M., Debski, P.G., Exergaming can be a health-related aerobic physical activity (2019) BioMed Res. Int, 2019, p. 1890527. , 31275964; Prado, M.T.A., Fernani, D.C.G.L., da Silva, T.D., Smorenburg, A.R., de Abreu, L.C., Monteiro, C.B.M., Motor learning paradigm and contextual interference in manual computer tasks in individuals with cerebral palsy (2017) Res. Dev. Disabil, 64, pp. 56-63. , 28351764; Rohlfs, I.C.P.D.M., Rotta, T.M., Luft, C.D.B., Andrade, A., Krebs, R.J., Carvalho, T.D., Brunel Mood Scale (BRUMS): an instrument for early detection of overtraining syndrome (2008) Revista Brasileira de Medicina do Esporte, 14, pp. 176-181; Rutkowski, S., Rutkowska, A., Kiper, P., Jastrzebski, D., Racheniuk, H., Turolla, A., Virtual reality rehabilitation in patients with chronic obstructive pulmonary disease: a randomized controlled trial (2020) Int. J. Chron. Obstruct. Pulmon. Dis, 15, pp. 117-124. , 32021150; Salonini, E., Gambazza, S., Meneghelli, I., Tridello, G., Sanguanini, M., Cazzarolli, C., Active video game playing in children and adolescents with cystic fibrosis: exercise or just fun? (2015) Respir. Care, 60, pp. 1172-1179. , 25899477; Schefold, J.C., Wollersheim, T., Grunow, J.J., Luedi, M.M., Z'Graggen, W.J., Weber-Carstens, S., Muscular weakness and muscle wasting in the critically ill (2020) J. Cachex. Sarcopenia Muscle, 11, pp. 1399-1412. , 32893974; Sheehy, L.M., Considerations for postacute rehabilitation for survivors of COVID-19 (2020) JMIR Public Health Surveill, 6, p. e19462. , 32369030; Shields, G.E., Wells, A., Doherty, P., Heagerty, A., Buck, D., Davies, L.M., Cost-effectiveness of cardiac rehabilitation: a systematic review (2018) Heart, 104, pp. 1403-1410. , 29654096; Singhal, T., A review of coronavirus disease-2019 (COVID-19) (2020) Indian J. Pediatr, 87, pp. 281-286; Smith, S.R., Jenq, G., Claflin, T., Magnant, C., Haig, A.J., Hurvitz, E., Proposed workflow for rehabilitation in a field hospital setting during the COVID-19 pandemic (2020) PM&R, 12, pp. 823-828. , 32412176; Smits-Engelsman, B.C., Jelsma, L.D., Ferguson, G.D., The effect of exergames on functional strength, anaerobic fitness, balance and agility in children with and without motor coordination difficulties living in low-income communities (2017) Hum. Mov. Sci, 55, pp. 327-337. , 27423302; Stam, H., Stucki, G., Bickenbach, J., Covid-19 and post intensive care syndrome: a call for action (2020) J. Rehabil. Med, 52, p. jrm00044. , 32286675; Stein, J., Visco, C.J., Barbuto, S., Rehabilitation medicine response to the COVID-19 pandemic (2020) Am. J. Phys. Med. Rehabil, 99, pp. 573-557. , 32433243; Sties, S.W., Gonzáles, A.I., Netto, A.S., Wittkopf, P.G., Lima, D.P., Carvalho, T.D., Validation of the Brunel Mood Scale for cardiac rehabilitation program (2014) Revista Brasileira de Medicina do Esporte, 20, pp. 281-284; Taslimipour, S., Rojhani-Shirazi, Z., Hemmati, L., Rezaei, I., Effects of a virtual reality dance training program on kyphosis angle and respiratory parameters in young women with postural hyperkyphosis: a randomized controlled clinical trial (2020) J. Sport Rehabil, 1, pp. 1-7. , 32404535; Vanderlei, L., Pastre, C.,

Hoshi, R., Carvalho, T., Godoy, M., Basic notions of heart rate variability and its clinical applicability (2009) Brazil. J. Cardiovasc. Surg, 24, pp. 205-217. , 19768301; Voutilainen, A., Pitkäaho, T., Kvist, T., Vehviläinen-Julkunen, K., How to ask about patient satisfaction? The visual analogue scale is less vulnerable to confounding factors and ceiling effect than a symmetric Likert scale (2016) J. Adv. Nurs, 72, pp. 946-957. , 26689434; Weston, S., Frieman, M.B., COVID-19: knowns, unknowns, and questions (2020) mSphere, 5, p. e00203. , 20; Williams, N., The Borg rating of perceived exertion (RPE) scale (2017) Occupat. Med, 67, pp. 404-405; Wu, Z., McGoogan, J.M., Characteristics of and important lessons from the coronavirus disease 2019 (COVID-19) outbreak in China: summary of a report of 72 314 cases from the Chinese Center for Disease Control and Prevention (2020) JAMA, 323, pp. 1239-1242; Yang, X., Yu, Y., Xu, J., Shu, H., Liu, H., Wu, Y., Clinical course and outcomes of critically ill patients with SARS-CoV-2 pneumonia in Wuhan, China: a single-centered, retrospective, observational study (2020) Lancet Respir. Med, 8, pp. 475-481. , 32105632; Zamunér, A.R., Moreno, M.A., Camargo, T.M., Graetz, J.P., Rebelo, A.C., Tamburús, N.Y., da Silva, E., Assessment of subjective perceived exertion at the anaerobic threshold with the Borg CR-10 scale (2011) J. Sports Sci. Med, 10, pp. 130-136. , 24149305; Zanaboni, P., Dinesen, B., Hjalmarsen, A., Hoaas, H., Holland, A.E., Oliveira, C.C., Long-term integrated telerehabilitation of COPD patients: a multicentre randomised controlled trial (iTrain) (2016) BMC Pulm. Med, 16, p. 126. , 27549782; Zhao, H.M., Xie, Y.X., Wang, C., Recommendations for respiratory rehabilitation in adults with coronavirus disease 2019 (2020) Chin. Med. J, 133, pp. 1595-1602. , 32251002; Zhong, L., Chuan, J., Gong, B.O., Shuai, P., Zhou, Y., Zhang, Y., Detection of serum IgM and IgG for COVID-19 diagnosis (2020) Sci. China Life Sci, 63, pp. 777-780. , 32270436; Zhou, M., Zhang, X., Qu, J., Coronavirus disease 2019 (COVID-19): a clinical update (2020) Front. Med, 14, pp. 126-135},
correspondence_address1={Silva, T.D.D.; Departamento de Medicina (Cardiologia), Brazil; email: talita.dias@unifesp.br; Silva, T.D.D.; Grupo de Pesquisa e Aplicações Tecnológicas em Reabilitação da Escola de Artes, Brazil; email: talita.dias@unifesp.br; Silva, T.D.D.; Faculdade de Medicina da, Brazil; email: talita.dias@unifesp.br},
publisher={Frontiers Media S.A.},
issn={16641078},
language={English},
abbrev_source_title={Front. Psychol.},
document_type={Article},
source={Scopus},
}

@ARTICLE{Shannon2021,
author={Shannon, O.M. and Lee, V. and Bundy, R. and Gillings, R. and Jennings, A. and Stephan, B. and Hornberger, M. and Balanos, G. and Paddick, S.M. and Hanson, S. and Hardeman, W. and Holmes, R. and Garner, N. and Aldred, S. and Siervo, M. and Mathers, J.C. and Minihane, A.M.},
title={Feasibility and acceptability of a multi-domain intervention to increase Mediterranean diet adherence and physical activity in older UK adults at risk of dementia: Protocol for the MedEx-UK randomised controlled trial},
journal={BMJ Open},
year={2021},
volume={11},
number={2},
doi={10.1136/bmjopen-2020-042823},

art_number={042823},
note={cited By 0},
url={https://www.scopus.com/inward/record.uri?eid=2-s2.0-85100686406&doi=10.1136%2fbmjopen-2020-042823&partnerID=40&md5=0ffac436227cd19aeb1402da5177ae89},
affiliation={Human Nutrition Research Centre, Population Health Sciences Institute, Newcastle University, Newcastle upon Tyne, United Kingdom; School of Sport, Exercise and Rehabilitation Sciences, University of Birmingham, Birmingham, United Kingdom; Department of Nutrition and Preventive Medicine, Norwich Medical School, University of East Anglia, Norwich, United Kingdom; Institute of Mental Health, Division of Psychiatry and Applied Psychology, School of Medicine, University of Nottingham, Nottingham, Nottinghamshire, United Kingdom; Norwich Medical School, University of East Anglia, Norwich, United Kingdom; Institute of Neuroscience, Newcastle University, Newcastle upon Tyne, Tyne and Wear, United Kingdom; Northumbria Healthcare NHS Foundation Trust, North Tyneside General Hospital, Newcastle, United Kingdom; School of Health Sciences, Norwich Medical School, University of East Anglia, Norwich, United Kingdom; Behavioural and Implementation Science Group, School of Health Sciences, University of East Anglia, Norwich, United Kingdom; Norfolk and Norwich University Hospitals NHS Foundation Trust, Norwich, United Kingdom; Centre for Human Brain Health, College of Life and Environmental Sciences, University of Birmingham, Birmingham, United Kingdom; School of Life Sciences, University of Nottingham, Nottingham, Nottinghamshire, United Kingdom},
abstract={Introduction Dementia prevalence continues to increase, and effective interventions are needed to prevent, delay or slow its progression. Higher adherence to the Mediterranean diet (MedDiet) and increased physical activity (PA) have been proposed as strategies to facilitate healthy brain ageing and reduce dementia risk. However, to date, there have been no dementia prevention trials in the UK focussed on combined dietary and PA interventions. This study aims to: (1) assess feasibility and acceptability of a theory-underpinned digital and group-based intervention for dementia risk reduction in an 'at risk' UK cohort; (2) evaluate behaviour change responses to the intervention; and, (3) provide information on cognitive, neurological, vascular and physiological outcomes to inform the design of a follow-on, full-scale efficacy trial. Methods One hundred and eight participants aged 55 to 74 years with a QRISK2 score of ≥10% will be recruited to take part in this 24-week multi-site study. Participants will be randomised into three parallel arms: (1) Control; (2) MedDiet; and, (3) MedDiet+PA. The study will evaluate a personalised website, group session and food delivery intervention to increase MedDiet adherence and PA in older adults at risk of dementia. Diet and PA will be monitored prior to, during and following the intervention. Feasibility, acceptability and hypothesised mediators will be assessed in addition to measures of cognitive function, brain structure/perfusion (MRI), vascular function and metabolic markers (blood, urine and faecal) prior to, and following, the intervention. Discussion This trial will provide insights into the feasibility, acceptability and mechanism of effect of a multi-domain intervention focussed on the MedDiet alone and PA for dementia risk reduction in an 'at risk' UK cohort. Ethics and dissemination The study has received NHS REC and HRA approval (18/NI/0191). Findings will be disseminated via conference presentations, public lectures, and peer-reviewed publications. Trial registration details ClinicalTrials.gov NCT03673722. ©},
author_keywords={dementia; nutrition & dietetics; public health},
keywords={brain derived neurotrophic factor, adult; aged; Article; behavior change; blood pressure monitoring; body mass; brain perfusion; cardiovascular risk; cognition; cohort analysis; controlled study; daily

life activity; dementia; female; human; international physical activity questionnaire; major clinical study; male; Mediterranean diet; mortality; multicenter study; nuclear magnetic resonance imaging; nutritional assessment; physical activity; practice guideline; primary medical care; program acceptability; randomized controlled trial; risk reduction; social media; spatial orientation; United Kingdom; web-based intervention},
references={(2018) Dementia: Key Facts, , https://www.who.int/en/news-room/fact-sheets/detail/dementia, World Health Organisation; (2019) Deaths Registered in England and Wales, , https://www.ons.gov.uk/peoplepopulationandcommunity/birthsdeathsandmarriages/deaths/datasets/deathsregisteredinenglandandwalesseriesdrreferencetables, ONS; Prince, M., Knapp, M., Guerchet, M., (2014) Dementia UK: Update, , UK: Alzheimer's Society; Livingston, G., Sommerlad, A., Orgeta, V., Dementia prevention, intervention, and care (2017) The Lancet, 390, pp. 2673-2734; Stevenson, E.J., Shannon, O.M., Minihane, A.M., NuBrain: UK Consortium for optimal nutrition for healthy brain ageing (2020) Nutr Bull, 45, pp. 223-229; Cummings, J.L., Morstorf, T., Zhong, K., Alzheimer's disease drug-development pipeline: Few candidates, frequent failures (2014) Alzheimers Res Ther, 6, p. 37; Deckers, K., Van Boxtel, M.P.J., Schiepers, O.J.G., Target risk factors for dementia prevention: A systematic review and Delphi consensus study on the evidence from observational studies (2015) Int J Geriatr Psychiatry, 30, pp. 234-246; Lewis, F., Karlsberg Schaffer, S., Sussex, J., The trajectory of dementia in the UK-making a difference (2014) Office of Health Economics; Bach-Faig, A., Berry, E.M., Lairon, D., Mediterranean diet pyramid today (2011) Science and Cultural Updates. Public Health Nutr, 14, pp. 2274-2284; Trichopoulou, A., Martínez-González, M.A., Tong, T.Y., Definitions and potential health benefits of the Mediterranean diet: Views from experts around the world (2014) BMC Med, 12, p. 112; Petersson, S.D., Philippou, E., Mediterranean diet, cognitive function, and dementia: A systematic review of the evidence (2016) Adv Nutr, 7, pp. 889-904; Radd-Vagenas, S., Duffy, S.L., Naismith, S.L., Effect of the Mediterranean diet on cognition and brain morphology and function: A systematic review of randomized controlled trials (2018) Am J Clin Nutr, 107, pp. 389-404; Martínez-Lapiscina, E.H., Clavero, P., Toledo, E., Mediterranean diet improves cognition: The PREDIMED-NAVARRA randomised trial (2013) J Neurol Neurosurg Psychiatry, 84, pp. 1318-1325; Martínez-Lapiscina, E.H., Galbete, C., Corella, D., Genotype patterns at CLU, CR1, PICALM and APOE, cognition and Mediterranean diet: The PREDIMED-NAVARRA trial (2014) Genes Nutr, 9, p. 393; Valls-Pedret, C., Sala-Vila, A., Serra-Mir, M., Mediterranean diet and age-related cognitive decline: A randomized clinical trial (2015) JAMA Intern Med, 175, pp. 1094-1103; Shannon, O.M., Stephan, B.C.M., Granic, A., Mediterranean diet adherence and cognitive function in older UK adults: The European prospective investigation into cancer and Nutrition-Norfolk (EPIC-Norfolk) study (2019) Am J Clin Nutr, 110, pp. 938-948; Shannon, O.M., Stephan, B.C.M., Minihane, A.-M., Nitric oxide boosting effects of the Mediterranean diet: A potential mechanism of action (2018) J Gerontol A Biol Sci Med Sci, 73, pp. 902-904; Shannon, O.M., Mendes, I., Köchl, C., Mediterranean diet increases endothelial function in adults: A systematic review and meta-analysis of randomized controlled trials (2020) J Nutr, 150, pp. 1151-1159; Cowell, O.R., Mistry, N., Deighton, K., Effects of a Mediterranean diet on blood pressure: A systematic review and meta-analysis of randomized controlled trials and observational studies (2020) J Hypertens; De Filippis, F., Pellegrini, N., Vannini, L., High-Level adherence to a Mediterranean diet beneficially impacts the gut microbiota and associated metabolome (2016) Gut, 65, pp. 1812-1821; Berti, V., Walters, M., Sterling, J.,

Mediterranean diet and 3-year Alzheimer brain biomarker changes in middle-aged adults (2018) Neurology, 90, pp. e1789-e1798; Vassilaki, M., Aakre, J.A., Syrjanen, J.A., Mediterranean diet, its components, and amyloid imaging biomarkers (2018) J Alzheimers Dis, 64, pp. 281-290; Xu, W., Wang, H.F., Wan, Y., Leisure time physical activity and dementia risk: A dose-response meta-analysis of prospective studies (2017) BMJ Open, 7, p. 014706; Sanders, L.M.J., Hortobágyi, T., La Bastide-Van Gemert, S., Dose-Response relationship between exercise and cognitive function in older adults with and without cognitive impairment: A systematic review and meta-analysis (2019) PLoS One, 14, p. 0210036; Sabia, S., Dugravot, A., Dartigues, J.-F., Physical activity, cognitive decline, and risk of dementia: 28 year follow-up of Whitehall II cohort study (2017) BMJ, 357, p. j2709; Lamb, S.E., Sheehan, B., Atherton, N., Dementia and physical activity (DAPA) trial of moderate to high intensity exercise training for people with dementia: Randomised controlled trial (2018) BMJ, 361, p. k1675; Angevaren, M., Aufdemkampe, G., Verhaar, H.J.J., Physical activity and enhanced fitness to improve cognitive function in older people without known cognitive impairment (2008) Cochrane Database Syst Rev, p. CD005381; Adlard, P.A., Perreau, V.M., Pop, V., Voluntary exercise decreases amyloid load in a transgenic model of Alzheimer's disease (2005) J Neurosci, 25, pp. 4217-4221; Cotman, C.W., Berchtold, N.C., Christie, L.-A., Exercise builds brain health: Key roles of growth factor cascades and inflammation (2007) Trends Neurosci, 30, pp. 464-472; Van Praag, H., Shubert, T., Zhao, C., Exercise enhances learning and hippocampal neurogenesis in aged mice (2005) J Neurosci, 25, pp. 8680-8685; Isaacs, K.R., Anderson, B.J., Alcantara, A.A., Exercise and the brain: Angiogenesis in the adult rat cerebellum after vigorous physical activity and motor skill learning (1992) J Cereb Blood Flow Metab, 12, pp. 110-119; Radak, Z., Hart, N., Sarga, L., Exercise plays a preventive role against Alzheimer's disease (2010) J Alzheimers Dis, 20, pp. 777-783; Ngandu, T., Lehtisalo, J., Solomon, A., A 2 year multidomain intervention of diet, exercise, cognitive training, and vascular risk monitoring versus control to prevent cognitive decline in at-risk elderly people (finger): A randomised controlled trial (2015) Lancet, 385, pp. 2255-2263; Moll Van Charante, E.P., Richard, E., Eurelings, L.S., Effectiveness of a 6-year multidomain vascular care intervention to prevent dementia (preDIVA): A cluster-randomised controlled trial (2016) Lancet, 388, pp. 797-805; Andrieu, S., Guyonnet, S., Coley, N., Effect of long-term omega 3 polyunsaturated fatty acid supplementation with or without multidomain intervention on cognitive function in elderly adults with memory complaints (MAPT): A randomised, placebo-controlled trial (2017) Lancet Neurol, 16, pp. 377-389; Hardman, R.J., Kennedy, G., Macpherson, H., A randomised controlled trial investigating the effects of Mediterranean diet and aerobic exercise on cognition in cognitively healthy older people living independently within aged care facilities: The Lifestyle Intervention in Independent Living Aged Care (LIILAC) study protocol [ACTRN12614001133628] (2015) Nutr J, 14, p. 53; Heffernan, M., Andrews, G., Fiatarone Singh, M.A., Maintain your brain: Protocol of a 3-year randomized controlled trial of a personalized multi-modal digital health intervention to prevent cognitive decline among community dwelling 55 to 77 year olds (2019) J Alzheimers Dis, 70, pp. S221-S237; Ordovas, J.M., Ferguson, L.R., Tai, E.S., Personalised nutrition and health (2018) BMJ, 361, p. 2173; Celis-Morales, C., Lara, J., Mathers, J.C., Personalising nutritional guidance for more effective behaviour change (2015) Proc Nutr Soc, 74, pp. 130-138; Celis-Morales, C., Livingstone, K.M., Marsaux, C.F., Effect of personalized nutrition on health-related behaviour change: Evidence from the Food4Me European randomized controlled trial (2017) Int J Epidemiol, 46, pp. 578-588; Jennings, A.,

Berendsen, A.M., De Groot, L.C.P.G.M., Mediterranean-Style diet improves systolic blood pressure and arterial stiffness in older adults (2019) Hypertension, 73, pp. 578-586; Justin, B.N., Turek, M., Hakim, A.M., Heart disease as a risk factor for dementia (2013) Clin Epidemiol, 5, pp. 135-145; De Bruijn, R.F.A.G., Ikram, M.A., Cardiovascular risk factors and future risk of Alzheimer's disease (2014) BMC Med, 12, p. 130; Song, R., Xu, H., Dintica, C.S., Associations between cardiovascular risk, structural brain changes, and cognitive decline (2020) J Am Coll Cardiol, 75, pp. 2525-2534; Chan, A.-W., Tetzlaff, J.M., Gøtzsche, P.C., Spirit 2013 explanation and elaboration: Guidance for protocols of clinical trials (2013) BMJ, 346, p. e7586; Knopman, D., Boland, L.L., Mosley, T., Cardiovascular risk factors and cognitive decline in middle-aged adults (2001) Neurology, 56, pp. 42-48; Raz, N., Rodrigue, K.M., Acker, J.D., Hypertension and the brain: Vulnerability of the prefrontal regions and executive functions (2003) Behav Neurosci, 117, pp. 1169-1180; Debette, S., Seshadri, S., Beiser, A., Midlife vascular risk factor exposure accelerates structural brain aging and cognitive decline (2011) Neurology, 77, pp. 461-468; Sabia, S., Fayosse, A., Dumurgier, J., Association of ideal cardiovascular health at age 50 with incidence of dementia: 25 year follow-up of Whitehall II cohort study (2019) BMJ, 366, p. l4414; Carson, N., Leach, L., Murphy, K.J., A re-examination of Montreal cognitive assessment (MoCA) cutoff scores (2018) Int J Geriatr Psychiatry, 33, pp. 379-388; Martínez-González, M.A., Corella, D., Salas-Salvadó, J., Cohort profile: Design and methods of the PREDIMED study (2012) Int J Epidemiol, 41, pp. 377-385; Rzewnicki, R., Vanden Auweele, Y., De Bourdeaudhuij, I., Addressing overreporting on the International physical activity questionnaire (IPAQ) telephone survey with a population sample (2003) Public Health Nutr, 6, pp. 299-305; Nasreddine, Z.S., Phillips, N.A., Bédirian, V., Valarie, B., The Montreal cognitive assessment, MoCA: A brief screening tool for mild cognitive impairment (2005) J Am Geriatr Soc, 53, pp. 695-699; Rattanabannakit, C., Risacher, S.L., Gao, S., The cognitive change index as a measure of self and informant perception of cognitive decline: Relation to neuropsychological tests (2016) J Alzheimers Dis, 51, pp. 1145-1155; Kroenke, K., Spitzer, R.L., Williams, J.B., The PHQ-9: Validity of a brief depression severity measure (2001) J Gen Intern Med, 16, pp. 606-613; Spitzer, R.L., Kroenke, K., Williams, J.B.W., A brief measure for assessing generalized anxiety disorder: The GAD-7 (2006) Arch Intern Med, 166, pp. 1092-1097; Galvin, J.E., Roe, C.M., Powlishta, K.K., The AD8: A brief informant interview to detect dementia (2005) Neurology, 65, pp. 559-564; Lawton, M.P., Brody, E.M., Assessment of older people: Self-maintaining and instrumental activities of daily living (1969) Gerontologist, 9, pp. 179-186; Trichopoulou, A., Costacou, T., Bamia, C., Adherence to a Mediterranean diet and survival in a Greek population (2003) N Engl J Med, 348, pp. 2599-2608; Sofi, F., Macchi, C., Abbate, R., Mediterranean diet and health status: An updated meta-analysis and a proposal for a literature-based adherence score (2014) Public Health Nutr, 17, pp. 2769-2782; (2011) Global Recommendations on Physical Activity for Health, , https://www.who.int/dietphysicalactivity/pa/en/, WHO; Lara, J., O'Brien, N., Godfrey, A., Pilot randomised controlled trial of a web-based intervention to promote healthy eating, physical activity and meaningful social connections compared with usual care control in people of retirement age recruited from workplaces (2016) PLoS One, 11, p. 0159703; Moore, S.E., McEvoy, C.T., Prior, L., Barriers to adopting a Mediterranean diet in northern European adults at high risk of developing cardiovascular disease (2018) J Hum Nutr Diet, 31, pp. 451-462; Tuomilehto, J., Lindström, J., Eriksson, J.G., Prevention of type 2 diabetes mellitus by changes in lifestyle among subjects with impaired glucose

tolerance (2001) N Engl J Med, 344, pp. 1343-1350; Knowler, W.C., Barrett-Connor, E., Fowler, S.E., Reduction in the incidence of type 2 diabetes with lifestyle intervention or metformin (2002) N Engl J Med, 346, pp. 393-403; Borek, A.J., Abraham, C., How do small groups promote behaviour change? an integrative conceptual review of explanatory mechanisms (2018) Appl Psychol Health Well Being, 10, pp. 30-61; Michie, S., Van Stralen, M.M., West, R., The behaviour change wheel: A new method for characterising and designing behaviour change interventions (2011) Implement Sci, 6, p. 42; Michie, S., Wood, C.E., Johnston, M., Behaviour change techniques: The development and evaluation of a taxonomic method for reporting and describing behaviour change interventions (a suite of five studies involving consensus methods, randomised controlled trials and analysis of qualitative data) (2015) Health Technol Assess, 19, pp. 1-188; Bellg, A.J., Borrelli, B., Resnick, B., Enhancing treatment fidelity in health behavior change studies: Best practices and recommendations from the NIH behavior change Consortium (2004) Health Psychology, 23, pp. 443-451; Tong, T.Y.N., Imamura, F., Monsivais, P., Dietary cost associated with adherence to the Mediterranean diet, and its variation by socio-economic factors in the UK Fenland study (2018) Br J Nutr, 119, pp. 685-694; Management of people with an estimated risk of 10% or more (2019) UK: National Institute for Health and Care Excellence, , https://cks.nice.org.uk/cvd-riskassessment-and-management, CVD risk assessment and management; Simpson, E., Bradley, J., Poliakov, I., Iterative development of an online dietary recall tool: INTAKE24 (2017) Nutrients, 9, p. 188; Foster, E., Lee, C., Imamura, F., Validity and reliability of an online self-report 24-h dietary recall method (Intake24): A doubly labelled water study and repeated-measures analysis (2019) J Nutr Sci, 8, p. e29; Bradley, J., Simpson, E., Poliakov, I., Comparison of INTAKE24 (an online 24-h dietary recall tool) with Interviewer-Led 24-h recall in 11-24 year-old (2016) Nutrients, 8, p. 358; Nelson, E.C., Verhagen, T., Noordzij, M.L., Health empowerment through activity trackers: An empirical smart wristband study (2016) Comput Human Behav, 62, pp. 364-374; Harrison, J., Minassian, S.L., Jenkins, L., A neuropsychological test battery for use in Alzheimer disease clinical trials (2007) Arch Neurol, 64, pp. 1323-1329; Tu, S., Wong, S., Hodges, J.R., Lost in spatial translation-A novel tool to objectively assess spatial disorientation in Alzheimer's disease and frontotemporal dementia (2015) Cortex, 67, pp. 83-94; Tu, S., Spiers, H.J., Hodges, J.R., Egocentric versus allocentric spatial memory in behavioral variant frontotemporal dementia and Alzheimer's disease (2017) J Alzheimers Dis, 59, pp. 883-892; Coughlan, G., Flanagan, E., Jeffs, S., Diagnostic relevance of spatial orientation for vascular dementia: A case study (2018) Dement Neuropsychol, 12, pp. 85-91; Ritchie, K., Carrière, I., Howett, D., Allocentric and egocentric spatial processing in middle-aged adults at high risk of late-onset Alzheimer's disease: The prevent dementia study (2018) J Alzheimers Dis, 65, pp. 885-896; Coutrot, A., Schmidt, S., Coutrot, L., Virtual navigation tested on a mobile app is predictive of real-world wayfinding navigation performance (2019) PLoS One, 14, p. 0213272; Coughlan, G., Laczó, J., Hort, J., Spatial navigation deficits-overlooked cognitive marker for preclinical Alzheimer disease? (2018) Nat Rev Neurol, 14, pp. 496-506; Coughlan, G., Coutrot, A., Khondoker, M., Toward personalized cognitive diagnostics of at-genetic-risk Alzheimer's disease (2019) Proc Natl Acad Sci U S A, 116, pp. 9285-9292; Stergiou, G., Palatini, P., Asmar, R., Blood pressure measurement and hypertension diagnosis in the 2017 us guidelines: First things first (2018) Hypertension, 71, pp. 963-965; Raitakari, O.T., Celermajer, D.S., Flow-Mediated dilatation (2000) Br J Clin Pharmacol, 50, pp. 397-404; Moore, G.F., Audrey, S., Barker, M., Process evaluation of complex interventions:

Medical Research Council guidance (2015) BMJ, 350, p. h1258; Creswell, J.W., (2014) A Concise Introduction to Mixed Methods Research, , London: SAGE Publications; Sekhon, M., Cartwright, M., Francis, J.J., Acceptability of healthcare interventions: An overview of reviews and development of a theoretical framework (2017) BMC Health Serv Res, 17, p. 88; Ajzen, I., The theory of planned behavior (1991) Organ Behav Hum Decis Process, 50, pp. 179-211; Cress, M.E., Buchner, D.M., Prohaska, T., Physical activity programs and behavior counseling in older adult populations (2004) Medicine & Science in Sports & Exercise, 36, pp. 1997-2003; Rivera-Torres, S., Fahey, T.D., Rivera, M.A., Adherence to exercise programs in older adults: Informative report (2019) Gerontol Geriatr Med, 5. , 233372141882360; Brunt, A., Albines, D., Hopkins-Rosseel, D., The effectiveness of exercise on cognitive performance in individuals with known vascular disease: A systematic review (2019) J Clin Med, 8, p. 294; Schulze, M.B., Martínez-González, M.A., Fung, T.T., Food based dietary patterns and chronic disease prevention (2018) BMJ, 361, p. k2396; Staudacher, H.M., Irving, P.M., Lomer, M.C.E., The challenges of control groups, placebos and blinding in clinical trials of dietary interventions (2017) Proc Nutr Soc, 76, pp. 203-212; Irimata, K.E., Dugger, B.N., Wilson, J.R., Impact of the presence of select cardiovascular risk factors on cognitive changes among dementia subtypes (2018) Curr Alzheimer Res, 15, pp. 1032-1044; Lara, J., McCrum, L.-A., Mathers, J.C., Association of Mediterranean diet and other health behaviours with barriers to healthy eating and perceived health among British adults of retirement age (2014) Maturitas, 79, pp. 292-298; Robinson, E., Boyland, E., Chisholm, A., Obesity, eating behavior and physical activity during COVID-19 lockdown: A study of UK adults (2021) Appetite, 156, p. 104853},
correspondence_address1={Minihane, A.M.; Department of Nutrition and Preventive Medicine, United Kingdom; email: a.minihane@uea.ac.uk},
publisher={BMJ Publishing Group},
issn={20446055},
pubmed_id={33550254},
language={English},
abbrev_source_title={BMJ Open},
document_type={Article},
source={Scopus},
}

@ARTICLE{Duan2021,
author={Duan, Y. and Shang, B. and Liang, W. and Du, G. and Yang, M. and Rhodes, R.E.},
title={Effects of eHealth-based multiple health behavior change interventions on physical activity, healthy diet, and weight in people with noncommunicable diseases: Systematic review and meta-analysis},
journal={Journal of Medical Internet Research},
year={2021},
volume={23},
number={2},
doi={10.2196/23786},
art_number={e23786},
note={cited By 0},
url={https://www.scopus.com/inward/record.uri?eid=2-s2.0-85101883086&doi=10.2196%2f23786&partnerID=40&md5=6ad3580b79adf2eda2b7b5248c59dfb6},
affiliation={Department of Sport, Physical Education and Health, Hong Kong Baptist University, Hong Kong, Hong Kong; Department of Social Sciences, Hebei

Sport University, Shijiazhuang, China; Department of Health Science, Wuhan Sports University, Wuhan, China; School of Exercise Science, Physical and Health Education, University of Victoria, Victoria, BC, Canada},
abstract={Background: Noncommunicable diseases (NCDs) are associated with the burden of premature deaths and huge medical costs globally. There is an increasing number of studies combining a multiple health behavior change (MHBC) intervention paradigm with eHealth approaches to jointly promote weight-related health behaviors among people with NCD; yet, a comprehensive summary of these studies is lacking. Objective: This review aims to meta-analyze the effectiveness and systematically summarize the characteristics of the relevant intervention studies for improving the outcomes of physical activity, healthy diet, and weight among people with NCD. Methods: Following PRISMA guidelines, 4 electronic databases (PsycINFO, PubMed, Scopus, SPORTDiscus) were systematically searched to identify eligible articles based on a series of inclusion and exclusion criteria. Article selection, quality assessment, and data extraction were independently performed by 2 authors. The standardized mean difference (SMD) was calculated to evaluate the effectiveness of interventions for 3 intervention outcomes (physical activity, healthy diet, and weight), and subsequent subgroup analyses were performed for gender, age, intervention duration, channel, and theory. Calculations were conducted, and figures were produced in SPSS 22 and Review Manager 5.3. Results: Of the 664 original hits generated by the systematic searches, 15 eligible studies with moderate to high quality were included. No potential publication bias was detected using statistical analyses. Studies varied in intervention channel, intensity, and content. The meta-analysis revealed that the eHealth MHBC interventions significantly promoted physical activity (SMD 0.85, 95% CI 0.23 to 1.47, P=.008) and healthy diet (SMD 0.78, 95% CI 0.13 to 1.43, P=.02), but did not contribute to a healthy weight status (SMD –0.13, 95% CI= –0.47 to 0.20, P=.43) among people with NCDs, compared to the control conditions. Results from subgroup analysis indicated that theory-based interventions achieved greater effect than nontheory-based interventions in promoting physical activity, and interventions with traditional approaches (SMS, telephone) were more effective than those with modern internet-based approaches in promoting healthy diet. Conclusions: The results of this review indicates that eHealth MHBC interventions achieve preliminary success in promoting physical activity and healthy diet behaviors among people with NCD. Future studies could improve the intervention design to achieve better intervention effectiveness. © Yanping Duan, Borui Shang, Wei Liang, Gaohui Du, Min Yang, Ryan E Rhodes.},
author_keywords={EHealth; Healthy diet; Meta-analysis; Multiple health behavior change; Noncommunicable disease; Physical activity; Systematic review; Weight-related},
references={(2018), https://www.who.int/news-room/fact-sheets/detail/noncommunicable-diseases, Noncommunicable diseases. World Health Organization. Jun. [accessed 2021-02-06]; Bloom, D, Chen, S, Kuhn, M, McGovern, M, Oxley, L, Prettner, K., The economic burden of chronic diseases: Estimates and projections for China, Japan, and South Korea (2020) The Journal of the Economics of Ageing, 17, p. 100163. , Oct;: [FREE Full text] [doi]; Jakovljevic, MB, Milovanovic, O., Growing Burden of Non-Communicable Diseases in the Emerging Health Markets: The Case of BRICS (2015) Front Public Health, 3, p. 65. , [FREE Full _text] [doi] [Medline: 25954740]; Broers, ER, Kop, WJ, Denollet, J, Widdershoven, J, Wetzels, M, Ayoola, I, A Personalized eHealth Intervention for Lifestyle Changes in Patients With Cardiovascular Disease: Randomized Controlled Trial

(2020) J Med Internet Res, 22 (5), p. e14570. , May 22; [FREE Full _text] [doi] [Medline: 32441658]; Prochaska, JO., Multiple Health Behavior Research represents the future of preventive medicine (2008) Prev Med, 46 (3), pp. 281-285. , Mar; [doi] [Medline: 18319100]; Geller, K, Lippke, S, Nigg, CR., Future directions of multiple behavior change research (2017) J Behav Med, 40 (1), pp. 194-202. , Feb; [doi] [Medline: 27785652]; Nigg, CR, Long, CR., A systematic review of single health behavior change interventions vs. multiple health behavior change interventions among older adults (2012) Transl Behav Med, 2 (2), pp. 163-179. , Jun; [FREE Full _text] [doi] [Medline: 24073109]; Beaglehole, R, Bonita, R, Horton, R, Adams, C, Alleyne, G, Asaria, P, Priority actions for the non-communicable disease crisis (2011) The Lancet, 377 (9775), pp. 1438-1447. , Apr 23; [doi] [Medline: 21474174]; Prochaska, JJ, Prochaska, JO., A Review of Multiple Health Behavior Change Interventions for Primary Prevention (2011) Am J Lifestyle Med, 5 (3), pp. 208-221. , May; [FREE Full _text] [doi] [Medline: 24358034]; Rhodes, RE, Quinlan, A, Mistry, CD., Do other goals influence physical activity? A systematic review examining the relationship between other goals and physical activity behavior (2016) Prev Med, 91, pp. 306-317. , Oct;: [doi] [Medline: 27568235]; van Dam, RM, Li, T, Spiegelman, D, Franco, OH, Hu, FB., Combined impact of lifestyle factors on mortality: prospective cohort study in US women (2008) BMJ, 337, p. a1440. , Sep 16;: [FREE Full _text] [doi] [Medline: 18796495]; Buis, LR, Dawood, K, Kadri, R, Dawood, R, Richardson, CR, Djuric, Z, Improving Blood Pressure Among African Americans With Hypertension Using a Mobile Health Approach (the MI-BP App): Protocol for a Randomized Controlled Trial (2019) JMIR Res Protoc, 8 (1), p. e12601. , Jan 25; [FREE Full _text] [doi] [Medline: 30681965]; Vandelanotte, C, Müller, AM, Short, CE, Hingle, M, Nathan, N, Williams, SL, Past, Present, and Future of eHealth and mHealth Research to Improve Physical Activity and Dietary Behaviors (2016) J Nutr Educ Behav, 48 (3), pp. 219-228. , Mar; e1. [doi] [Medline: 26965100]; Boogerd, EA, Arts, T, Engelen, LJ, van de Belt, TH., "What Is eHealth": Time for An Update? (2015) JMIR Res Protoc, 4 (1), p. e29. , Mar 12; [FREE Full _text] [doi] [Medline: 25768939]; Murray, E, Khadjesari, Z, White, IR, Kalaitzaki, E, Godfrey, C, McCambridge, J, Methodological challenges in online trials (2009) J Med Internet Res, 11 (2), p. e9. , Apr 03; [FREE Full _text] [doi] [Medline: 19403465]; Haberlin, C, O'Dwyer, T, Mockler, D, Moran, J, O'Donnell, DM, Broderick, J., The use of eHealth to promote physical activity in cancer survivors: a systematic review (2018) Support Care Cancer, 26 (10), pp. 3323-3336. , Oct 16; [doi] [Medline: 29909476]; Rollo, ME, Aguiar, EJ, Williams, RL, Wynne, K, Kriss, M, Callister, R, eHealth technologies to support nutrition and physical activity behaviors in diabetes self-management (2016) Diabetes Metab Syndr Obes, 9, pp. 381-390. , [FREE Full _text] [doi] [Medline: 27853384]; Duan, YP, Liang, W, Guo, L, Wienert, J, Si, GY, Lippke, S., Evaluation of a Web-Based Intervention for Multiple Health Behavior Changes in Patients With Coronary Heart Disease in Home-Based Rehabilitation: Pilot Randomized Controlled Trial (2018) J Med Internet Res, 20 (11), p. e12052. , Nov 19; [FREE Full _text] [doi] [Medline: 30455167]; Kanera, IM, Bolman, CAW, Willems, RA, Mesters, I, Lechner, L., Lifestyle-related effects of the web-based Kanker Nazorg Wijzer (Cancer Aftercare Guide) intervention for cancer survivors: a randomized controlled trial (2016) J Cancer Surviv, 10 (5), pp. 883-897. , Oct 17; [FREE Full _text] [doi] [Medline: 26984534]; Moher, D, Liberati, A, Tetzlaff, J, Altman, DG, Preferred reporting items for systematic reviews and meta-analyses: the PRISMA statement (2009) PLoS Med, 6 (7), p. e1000097. , PRISMA Group. Jul 21; [FREE Full _text] [doi] [Medline: 19621072]; Su, X, Dai, H, Zhang, F, Chen, K, Li, H, Wu, X, Research on the Application of "Internet + medical treatment" in Diabetes Hospital - community - family

Management Model (2018) Hospital Management Forum, 35 (4), pp. 74-77; Müller, AM, Alley, S, Schoeppe, S, Vandelanotte, C., (2016) Int J Behav Nutr Phys Act, 13 (1), p. 109. , Oct 10; [FREE Full _text] [doi] [Medline: 27724911]; Higgins, JPT, Altman, DG, Gøtzsche, PC, Jüni, P, Moher, D, Oxman, AD, The Cochrane Collaboration's tool for assessing risk of bias in randomised trials (2011) BMJ, 343, p. d5928. , Cochrane Bias Methods Group, Cochrane Statistical Methods Group. Oct 18;: [FREE Full _text] [doi] [Medline: 22008217]; Begg, CB, Mazumdar, M., Operating characteristics of a rank correlation test for publication bias (1994) Biometrics, 50 (4), pp. 1088-1101. , Dec; [Medline: 7786990]; DeCoster, J., Converting effect sizes Microsoft Excel Spreadsheets (2012) Stat-Help, , http://www.stat-help.com/spreadsheets.html, [accessed 2021-02-06]; Borenstein, M, Hedges, LV, Higgins, JPT, Rothstein, HR., (2009) Introduction to meta-analysis, , Hoboken, NJ: John Wiley & Sons, Ltd; Mar 11; Bantum, EO, Albright, CL, White, KK, Berenberg, JL, Layi, G, Ritter, PL, Surviving and thriving with cancer using a Web-based health behavior change intervention: randomized controlled trial (2014) J Med Internet Res, 16 (2), p. e54. , Feb 24; [FREE Full _text] [doi] [Medline: 24566820]; Field, AP, Gillett, R., How to do a meta-analysis (2010) Br J Math Stat Psychol, 63, pp. 665-694. , Nov;(Pt 3): [doi] [Medline: 20497626]; Lewis, S, Clarke, M., Forest plots: trying to see the wood and the trees (2001) BMJ, 322 (7300), pp. 1479-1480. , Jun 16; [FREE Full text] [doi] [Medline: 11408310]; Higgins, JPT, Green, S., (2008) Cochrane handbook for systematic reviews of interventions, , https://training.cochrane.org/handbook/archive/v5.0.0/, Version 5.0.0. [accessed 2021-02-06]; (2013) SPSS Statistics for Windows, , https://www.ibm.com/support/pages/spss-statistics-220-available-download, Version 22. IBM. [accessed 2021-02-06]; (2014), revman.cochrane.org, Cochrane Review Manager (RevMan). [accessed 2021-02-06]; Glasgow, RE, Kurz, D, King, D, Dickman, JM, Faber, AJ, Halterman, E, Outcomes of minimal and moderate support versions of an internet-based diabetes self-management support program (2010) J Gen Intern Med, 25 (12), pp. 1315-1322. , Dec; [FREE Full _text] [doi] [Medline: 20714820]; Swoboda, CM, Miller, CK, Wills, CE., Setting Single or Multiple Goals for Diet and Physical Activity Behaviors Improves Cardiovascular Disease Risk Factors in Adults With Type 2 Diabetes: A Pragmatic Pilot Randomized Trial (2016) Diabetes Educ, 42 (4), pp. 429-443. , Aug; [doi] [Medline: 27194048]; Migneault, JP, Dedier, JJ, Wright, JA, Heeren, T, Campbell, MK, Morisky, DE, A culturally adapted telecommunication system to improve physical activity, diet quality, and medication adherence among hypertensive African-Americans: a randomized controlled trial (2012) Ann Behav Med, 43 (1), pp. 62-73. , Feb; [doi] [Medline: 22246660]; Liu, S, Brooks, D, Thomas, SG, Eysenbach, G, Nolan, RP., Effectiveness of User- and Expert-Driven Web-based Hypertension Programs: an RCT (2018) Am J Prev Med, 54 (4), pp. 576-583. , Apr; [doi] [Medline: 29456025]; Nolan, RP, Upshur, REG, Lynn, H, Crichton, T, Rukholm, E, Stewart, DE, Therapeutic benefit of preventive telehealth counseling in the Community Outreach Heart Health and Risk Reduction Trial (2011) Am J Cardiol, 107 (5), pp. 690-696. , Mar 1; [doi] [Medline: 21215382]; Ramachandran, A, Snehalatha, C, Ram, J, Selvam, S, Simon, M, Nanditha, A, Effectiveness of mobile phone messaging in prevention of type 2 diabetes by lifestyle modification in men in India: a prospective, parallel-group, randomised controlled trial (2013) The Lancet Diabetes & Endocrinology, 1 (3), pp. 191-198. , Nov; [doi] [Medline: 24622367]; Shetty, AS, Chamukuttan, S, Nanditha, A, Raj, RKC, Ramachandran, A., Reinforcement of adherence to prescription recommendations in Asian Indian diabetes patients using short message service (SMS)-a pilot study (2011) J Assoc Physicians India, 59, pp. 711-714. , Nov;: [Medline: 22616337]; Shahid, M, Mahar, SA,

Shaikh, S, Shaikh, Z., Mobile phone intervention to improve diabetes care in rural areas of Pakistan: a randomized controlled trial (2015) J Coll Physicians Surg Pak, 25 (3), pp. 166-171. , Mar; [doi: 03.2015/JCPSP.166171] [Medline: 25772954]; Lee, MK, Yun, YH, Park, H, Lee, ES, Jung, KH, Noh, D., A Web-based self-management exercise and diet intervention for breast cancer survivors: pilot randomized controlled trial (2014) Int J Nurs Stud, 51 (12), pp. 1557-1567. , Dec; [doi] [Medline: 24856854]; Pfaeffli Dale, L, Whittaker, R, Jiang, Y, Stewart, R, Rolleston, A, Maddison, R., Text Message and Internet Support for Coronary Heart Disease Self-Management: Results From the Text4Heart Randomized Controlled Trial (2015) J Med Internet Res, 17 (10), p. e237. , Oct 21; [FREE Full _text] [doi] [Medline: 26490012]; Lindsay, S, Bellaby, P, Smith, S, Baker, R., Enabling healthy choices: is ICT the highway to health improvement? (2008) Health (London), 12 (3), pp. 313-331. , Jul; [doi] [Medline: 18579630]; Morey, MC, Snyder, DC, Sloane, R, Cohen, HJ, Peterson, B, Hartman, TJ, Effects of home-based diet and exercise on functional outcomes among older, overweight long-term cancer survivors: RENEW: a randomized controlled trial (2009) JAMA, 301 (18), pp. 1883-1891. , May 13; [FREE Full _text] [doi] [Medline: 19436015]; Sawilowsky, S., New Effect Size Rules of Thumb (2009) J. Mod. App. Stat. Meth, 8 (2), pp. 597-599. , Nov 01; [FREE Full _text] [doi]; Fu, R, Gartlehner, G, Grant, M, Shamliyan, T, Sedrakyan, A, Wilt, TJ, Conducting quantitative synthesis when comparing medical interventions: AHRQ and the Effective Health Care Program (2011) J Clin Epidemiol, 64 (11), pp. 1187-1197. , Nov; [doi] [Medline: 21477993]; Amireault, S, Fong, AJ, Sabiston, CM., Promoting Healthy Eating and Physical Activity Behaviors: A Systematic Review of Multiple Health Behavior Change Interventions Among Cancer Survivors (2018) Am J Lifestyle Med, 12 (3), pp. 184-199. , [FREE Full _text] [doi] [Medline: 30202391]; Alageel, S, Gulliford, MC, McDermott, L, Wright, AJ., Multiple health behaviour change interventions for primary prevention of cardiovascular disease in primary care: systematic review and meta-analysis (2017) BMJ Open, 7 (6), p. e015375. , Jun 15; [FREE Full _text] [doi] [Medline: 28619779]; Kwan, RYC, Salihu, D, Lee, PH, Tse, M, Cheung, DSK, Roopsawang, I, The effect of e-health interventions promoting physical activity in older people: a systematic review and meta-analysis (2020) Eur Rev Aging Phys Act, 17, p. 7. , [FREE Full text] [doi] [Medline: 32336996]; Rodriguez Rocha, NP, Kim, H., eHealth Interventions for Fruit and Vegetable Intake: A Meta-Analysis of Effectiveness (2019) Health Educ Behav, 46 (6), pp. 947-959. , Dec; [doi] [Medline: 31347403]; Sin, DYE, Guo, X, Yong, DWW, Qiu, TY, Moey, PKS, Falk, M, Assessment of willingness to Tele-monitoring interventions in patients with type 2 diabetes and/or hypertension in the public primary healthcare setting (2020) BMC Med Inform Decis Mak, 20 (1), p. 11. , Jan 28; [FREE Full _text] [doi] [Medline: 31992288]; Schulz, KF, Altman, DG, Moher, D, CONSORT 2010 statement: updated guidelines for reporting parallel group randomised trials (2010) BMJ, 340, p. c332. , CONSORT Group. Mar 23;: [FREE Full _text] [doi] [Medline: 20332509]; Sheeran, P, Gollwitzer, PM, Bargh, JA., Nonconscious processes and health (2013) Health Psychol, 32 (5), pp. 460-473. , May; [doi] [Medline: 22888816]; Hagger, MS., Non-conscious processes and dual-process theories in health psychology (2016) Health Psychol Rev, 10 (4), pp. 375-380. , Dec; [doi] [Medline: 27718880]; Rhodes, RE, McEwan, D, Rebar, AL., Theories of physical activity behaviour change: A history and synthesis of approaches (2019) Psychology of Sport and Exercise, 42, pp. 100-109. , May;: [doi]; Kang, H, Han, J, Kwon, GH., An Ecological Approach to Smart Homes for Health Care Services: Conceptual Framework of a Smart Servicescape Wheel (2019) JMIR Mhealth Uhealth, 7 (2), p. e12425. , Feb 08; [FREE Full _text] [doi] [Medline: 30735145]; Annett, J., Subjective rating scales: science or art? (2002)

Ergonomics, 45 (14), pp. 966-987. , Nov 15; [doi] [Medline: 12569049]; Rosenberger, ME, Buman, MP, Haskell, WL, McConnell, MV, Carstensen, LL., Twenty-four Hours of Sleep, Sedentary Behavior, and Physical Activity with Nine Wearable Devices (2016) Med Sci Sports Exerc, 48 (3), pp. 457-465. , Mar 17; [FREE Full _text] [doi] [Medline: 26484953]; Champion, KE, Newton, NC, Spring, B, Wafford, QE, Parmenter, BJ, Teesson, M., A systematic review of school-based eHealth interventions targeting alcohol use, smoking, physical inactivity, diet, sedentary behaviour and sleep among adolescents: a review protocol (2017) Syst Rev, 6 (1), p. 246. , Dec 06; [FREE Full _text] [doi] [Medline: 29208040]; Prestwich, A, Sniehotta, FF, Whittington, C, Dombrowski, SU, Rogers, L, Michie, S., Does theory influence the effectiveness of health behavior interventions? Meta-analysis (2014) Health Psychol, 33 (5), pp. 465-474. , May; [doi] [Medline: 23730717]; Webb, TL, Joseph, J, Yardley, L, Michie, S., Using the internet to promote health behavior change: a systematic review and meta-analysis of the impact of theoretical basis, use of behavior change techniques, and mode of delivery on efficacy (2010) J Med Internet Res, 12 (1), p. e4. , Feb 17; [FREE Full _text] [doi] [Medline: 20164043]; McEwan, D, Beauchamp, MR, Kouvousis, C, Ray, CM, Wyrough, A, Rhodes, RE., Examining the active ingredients of physical activity interventions underpinned by theory versus no stated theory: a meta-analysis (2019) Health Psychol Rev, 13 (1), pp. 1-17. , Mar; [doi] [Medline: 30412685]; Hagger, MS, Weed, M., DEBATE: Do interventions based on behavioral theory work in the real world? (2019) Int J Behav Nutr Phys Act, 16 (1), p. 36. , Apr 25; [FREE Full _text] [doi] [Medline: 31023328]; James, E, Freund, M, Booth, A, Duncan, MJ, Johnson, N, Short, CE, Comparative efficacy of simultaneous versus sequential multiple health behavior change interventions among adults: A systematic review of randomised trials (2016) Prev Med, 89, pp. 211-223. , Aug;: [doi] [Medline: 27311332]},
correspondence_address1={Shang, B.; Department of Social Sciences, 82 Xuefu Road, China; email: borui_shang_pe@qq.com},
publisher={JMIR Publications Inc.},
issn={14388871},
pubmed_id={33616534},
language={English},
abbrev_source_title={J. Med. Internet Res.},
document_type={Review},
source={Scopus},
}

@ARTICLE{McCarthy2021,
author={McCarthy, H. and Potts, H.W.W. and Fisher, A.},
title={Physical activity behavior before, during, and after COVID-19 restrictions: Longitudinal smartphone-tracking study of adults in the United Kingdom},
journal={Journal of Medical Internet Research},
year={2021},
volume={23},
number={2},
doi={10.2196/23701},
art_number={e23701},
note={cited By 1},
url={https://www.scopus.com/inward/record.uri?eid=2-s2.0-85100482624&doi=10.2196%2f23701&partnerID=40&md5=60ee08c6448007625c1ae2f6f9bb7ac6},
affiliation={University College London, London, United Kingdom; Institute of Health Informatics, University College London, London, United Kingdom;

Department of Behavioural Science and Health, University College London, London, United Kingdom},
abstract={Background: The COVID-19 pandemic led to the implementation of worldwide restrictive measures to reduce social contact and viral spread. These measures have been reported to have a negative effect on physical activity (PA). Studies of PA during the pandemic have primarily used self-reported data. The single academic study that used tracked data did not report on demographics. Objective: This study aimed to explore patterns of smartphone-tracked activity before, during, and immediately after lockdown in the United Kingdom, and examine differences by sociodemographic characteristics and prior levels of PA. Methods: Tracked longitudinal weekly minutes of PA were captured using the BetterPoints smartphone app between January and June 2020. Data were plotted by week, demographics, and activity levels at baseline. Nonparametric tests of difference were used to assess mean and median weekly minutes of activity at significant points before and during the lockdown, and as the lockdown was eased. Changes over time by demographics (age, gender, Index of Multiple Deprivation, baseline activity levels) were examined using generalized estimating equations (GEEs). Results: There were 5395 users with a mean age of 41 years (SD 12) and 61% (n=3274) were female. At baseline, 26% (n=1422) of users were inactive, 23% (n=1240) were fairly active, and 51% (n=2733) were active. There was a relatively even spread across deprivation deciles (31% [n=1693] in the least deprived deciles and 23% in the most [n=1261]). We found significant changes in PA from the week before the first case of COVID-19 was announced (baseline) to the week that social distancing restrictions were relaxed (Friedman test: χ22=2331, P<.001). By the first full week of lockdown, the median change in PA was 57 minutes less than baseline. This represents a 37% reduction in weekly minutes of PA. Overall, 63% of people decreased their level of activity between baseline and the first week of COVID-19 restrictions. Younger people showed more PA before lockdown but the least PA after lockdown. In contrast, those aged >65 years appeared to remain more active throughout and increased their activity levels as soon as lockdown was eased. Levels of PA among those classed as active at baseline showed a larger drop compared with those considered to be fairly active or inactive. Socioeconomic group and gender did not appear to be associated with changes in PA. Conclusions: Our tracked PA data suggests a significant drop in PA during the United Kingdom’s COVID-19 lockdown. Significant differences by age group and prior PA levels suggests that the government’s response to COVID-19 needs to be sensitive to these individual differences and the government should react accordingly. Specifically, it should consider the impact on younger age groups, encourage everyone to increase their PA, and not assume that people will recover prior levels of PA on their own. © Hannah McCarthy, Henry W W Potts, Abigail Fisher.},
author_keywords={Apps; Behavior; COVID-19; Fitness trackers; MHealth; Mobile apps; Pattern; Physical activity; Smartphone; Tracking},
keywords={adolescent; adult; aged; communicable disease control; exercise; female; human; longitudinal study; male; middle aged; mobile application; pandemic; public policy; smartphone; United Kingdom; very elderly; young adult, Adolescent; Adult; Aged; Aged, 80 and over; Communicable Disease Control; COVID-19; Exercise; Female; Humans; Longitudinal Studies; Male; Middle Aged; Mobile Applications; Pandemics; Public Policy; SARS-CoV-2; Smartphone; United Kingdom; Young Adult},
references={Warburton, D, Bredin, SSD., Health benefits of physical activity: a systematic review of current systematic reviews (2017) Curr Opin Cardiol, 32 (5), pp. 541-556. , Sep; [doi] [Medline: 28708630]; Ekelund, U, Tarp, J,

Steene-Johannessen, J, Hansen, BH, Jefferis, B, Fagerland, MW, Dose-response associations between accelerometry measured physical activity and sedentary time and all cause mortality: systematic review and harmonised meta-analysis (2019) BMJ, 366, p. 4570. , Aug 21;: [FREE Full _text] [doi] [Medline: 31434697]; Hammami, A, Harrabi, B, Mohr, M, Krustrup, P., Physical activity and coronavirus disease 2019 (COVID-19): specific recommendations for home-based physical training (2020) Managing Sport and Leisure, 19, pp. 1-6. , Apr 20;: [doi]; Jiménez-Pavón, D, Carbonell-Baeza, A, Lavie, CJ., Physical exercise as therapy to fight against the mental and physical consequences of COVID-19 quarantine: Special focus in older people (2020) Prog Cardiovasc Dis, 63 (3), pp. 386-388. , [FREE Full _text] [doi] [Medline: 32220590]; Lippi, G, Henry, BM, Sanchis-Gomar, F., Physical inactivity and cardiovascular disease at the time of coronavirus disease 2019 (COVID-19) (2020) Eur J Prev Cardiol, 27 (9), pp. 906-908. , Jun; [FREE Full _text] [doi] [Medline: 32270698]; Rundle, AG, Park, Y, Herbstman, JB, Kinsey, EW, Wang, YC., COVID-19-Related School Closings and Risk of Weight Gain Among Children (2020) Obesity (Silver Spring), 28 (6), pp. 1008-1009. , Jun 18; [FREE Full _text] [doi] [Medline: 32227671]; (2020) New exercise habits forming during coronavirus crisis, , https://www.sportengland.org/news/new-exercise-habits-forming-during-coronavirus-crisis, Sport England. [accessed 2020-12-23]; Ding, D, Del Pozo Cruz, B, Green, MA, Bauman, AE., Is the COVID-19 lockdown nudging people to be more active: a big data analysis (2020) Br J Sports Med, 54 (20), pp. 1183-1184. , Oct 30; [doi] [Medline: 32605932]; Ammar, A, Brach, M, Trabelsi, K, Chtourou, H, Boukhris, O, Masmoudi, L, Effects of COVID-19 Home Confinement on Eating Behaviour and Physical Activity: Results of the ECLB-COVID19 International Online Survey (2020) Nutrients, 12 (6), p. 1. , May 28; [FREE Full _text] [doi] [Medline: 32481594]; Rogers, NT, Waterlow, NR, Brindle, H, Enria, L, Eggo, RM, Lees, S, Behavioral Change Towards Reduced Intensity Physical Activity Is Disproportionately Prevalent Among Adults With Serious Health Issues or Self-Perception of High Risk During the UK COVID-19 Lockdown (2020) Front Public Health, 8, p. 575091. , [FREE Full _text] [doi] [Medline: 33102424]; Smith, L, Jacob, L, Butler, L, Schuch, F, Barnett, Y, Grabovac, I, Prevalence and correlates of physical activity in a sample of UK adults observing social distancing during the COVID-19 pandemic (2020) BMJ Open Sport Exerc Med, 6 (1), p. e000850. , Jul 01; [doi]; (2020) The Impact Of Coronavirus On Global Activity, , https://blog.fitbit.com/covid-19-global-activity/, FitBit Staff. Mar 23. [accessed 2020-12-23]; (2020) Activity bounces back as lockdowns lift, but some healthy gains are slipping, , https://blog.fitbit.com/lockdowns-lift-mobility-changes/, FitBit Staff. [accessed 2020-12-23]; (2020) Are we still moving? The effect of the global pandemic on active lifestyles, , https://www.garmin.com/en-US/blog/general/the-effect-of-the-global-pandemic-on-active-lifestyles/, Garmin. [accessed 2020-12-23]; Tison, GH, Avram, R, Kuhar, P, Abreau, S, Marcus, GM, Pletcher, MJ, Worldwide Effect of COVID-19 on Physical Activity: A Descriptive Study (2020) Ann Intern Med, 173 (9), pp. 767-770. , Nov 03; [doi]; www.BetterPoints.ltd, BetterPoints. [accessed 2020-12-23]; English indices of deprivation 2019, , imd-by-postcode.opendatacommunities.org/imd/2019, [accessed 2020-12-23]; Hekler, EB, Buman, MP, Grieco, L, Rosenberger, M, Winter, SJ, Haskell, W, Validation of Physical Activity Tracking via Android Smartphones Compared to ActiGraph Accelerometer: Laboratory-Based and Free-Living Validation Studies (2015) JMIR mHealth uHealth, 3 (2), p. e36. , Apr 15; [FREE Full _text] [doi] [Medline: 25881662]; Duncan, MJ, Wunderlich, K, Zhao, Y, Faulkner, G., Walk this way: validity evidence of iphone health application step count in

laboratory and free-living conditions (2018) J Sports Sci, 36 (15), pp. 1695-1704. , Aug; [doi] [Medline: 29179653]; (2018) Active lives adult survey, , https://sportengland-production-files.s3.eu-west-2.amazonaws.com/s3fs-public/active-lives-adult-may-17-18-report.pdf, Sport England. May 17/18 report. [accessed 2020-12-23]; Carter, EM, Potts, HWW., Predicting length of stay from an electronic patient record system: a primary total knee replacement example (2014) BMC Med Inform Decis Mak, 14, p. 26. , Apr 04;: [FREE Full _text] [doi] [Medline: 24708853]; Westgate, PM, Burchett, WW., A Comparison of Correlation Structure Selection Penalties for Generalized Estimating Equations (2017) Am Stat, 71 (4), pp. 344-353. , [FREE Full _text] [doi] [Medline: 30918414]; Jia, R, Ayling, K, Chalder, T, Massey, A, Broadbent, E, Coupland, C, Mental health in the UK during the COVID-19 pandemic: cross-sectional analyses from a community cohort study (2020) BMJ Open, 10 (9), p. e040620. , Sep 15; [FREE Full text] [doi] [Medline: 32933965]; Smith, L, Jacob, L, Butler, L, Schuch, F, Barnett, Y, Grabovac, I, Prevalence and correlates of physical activity in a sample of UK adults observing social distancing during the COVID-19 pandemic (2020) BMJ Open Sport Exerc Med, 6 (1), p. e000850. , Jul 01; [doi]; Smith, LEAR, Potts, HWW, Fear, NT, Michie, S, Rubin, GJ., Worry, behaviour and stigma following UK Government communications during the COVID-19 outbreak: results from three UK surveys (2020) Report to the Department of Health and Social Care, , https://www.gov.uk/government/organisations/department-of-health-and-social-care, [accessed 2020-12-23]; (2019) Communications Market Report 2019, , https://www.ofcom.org.uk/__data/assets/pdf_file/0028/155278/communications-market-report-2019.pdf, Ofcom. Jul 04. [accessed 2020-10-08]},
correspondence_address1={Potts, H.W.W.; Institute of Health Informatics, 222 Euston Road, United Kingdom; email: h.potts@ucl.ac.uk},
publisher={JMIR Publications Inc.},
issn={14388871},
pubmed_id={33347421},
language={English},
abbrev_source_title={J. Med. Internet Res.},
document_type={Article},
source={Scopus},
}

@ARTICLE{McLaughlin2021,
author={McLaughlin, M. and Delaney, T. and Hall, A. and Byaruhanga, J. and Mackie, P. and Grady, A. and Reilly, K. and Campbell, E. and Sutherland, R. and Wiggers, J. and Wolfenden, L.},
title={Associations between digital health intervention engagement, physical activity, and sedentary behavior: Systematic review and meta-analysis},
journal={Journal of Medical Internet Research},
year={2021},
volume={23},
number={2},
doi={10.2196/23180},
art_number={e23180},
note={cited By 0},
url={https://www.scopus.com/inward/record.uri?eid=2-s2.0-85101069694&doi=10.2196%2f23180&partnerID=40&md5=8b5bf5fb89d0de8b75ae08edbc55a02a},
affiliation={School of Medicine and Public Health, University of Newcastle, Callaghan, Australia; Hunter New England Population Health, Wallsend,

Australia; Hunter Medical Research Institute, New Lambton Heights, Australia; Priority Research Centre for Heath Behaviour, University of Newcastle, Callaghan, Australia; School of Health Sciences and Priority, Research Centre for Stroke and Brain Injury, University of Newcastle, Callaghan, Australia; Centre for Research Excellence in Stroke Recovery and Rehabilitation, Florey Institute of Neuroscience, Melbourne, Australia},
abstract={Background: The effectiveness of digital health interventions is commonly assumed to be related to the level of user engagement with the digital health intervention, including measures of both digital health intervention use and users' subjective experience. However, little is known about the relationships between the measures of digital health intervention engagement and physical activity or sedentary behavior. Objective: This study aims to describe the direction and strength of the association between engagement with digital health interventions and physical activity or sedentary behavior in adults and explore whether the direction of association of digital health intervention engagement with physical activity or sedentary behavior varies with the type of engagement with the digital health intervention (ie, subjective experience, activities completed, time, and logins). Methods: Four databases were searched from inception to December 2019. Grey literature and reference lists of key systematic reviews and journals were also searched. Studies were eligible for inclusion if they examined a quantitative association between a measure of engagement with a digital health intervention targeting physical activity and a measure of physical activity or sedentary behavior in adults (aged ≥18 years). Studies that purposely sampled or recruited individuals on the basis of pre-existing health-related conditions were excluded. In addition, studies were excluded if the individual engaging with the digital health intervention was not the target of the physical activity intervention, the study had a non-digital health intervention component, or the digital health interventions targeted multiple health behaviors. A random effects meta-analysis and direction of association vote counting (for studies not included in meta-analysis) were used to address objective 1. Objective 2 used vote counting on the direction of the association. Results: Overall, 10,653 unique citations were identified and 375 full texts were reviewed. Of these, 19 studies (26 associations) were included in the review, with no studies reporting a measure of sedentary behavior. A meta-analysis of 11 studies indicated a small statistically significant positive association between digital health engagement (based on all usage measures) and physical activity (0.08, 95% CI 0.01-0.14, SD 0.11). Heterogeneity was high, with 77% of the variation in the point estimates explained by the between-study heterogeneity. Vote counting indicated that the relationship between physical activity and digital health intervention engagement was consistently positive for three measures: subjective experience measures (2 of 3 associations), activities completed (5 of 8 associations), and logins (6 of 10 associations). However, the direction of associations between physical activity and time-based measures of usage (time spent using the intervention) were mixed (2 of 5 associations supported the hypothesis, 2 were inconclusive, and 1 rejected the hypothesis). Conclusions: The findings indicate a weak but consistent positive association between engagement with a physical activity digital health intervention and physical activity outcomes. No studies have targeted sedentary behavior outcomes. The findings were consistent across most constructs of engagement; however, the associations were weak. © Matthew Mclaughlin, Tessa Delaney, Alix Hall, Judith Byaruhanga, Paul Mackie, Alice Grady, Kathryn Reilly, Elizabeth Campbell, Rachel Sutherland, John Wiggers, Luke Wolfenden. Originally published in the Journal of Medical Internet Research

(http://www.jmir.org), 19.02.2021. This is an open-access article distributed under the terms of the Creative Commons Attribution License (https://creativecommons.org/licenses/by/4.0/)},
author_keywords={Adherence; Digital behavior change intervention; Digital health intervention; Engagement; Mobile phone; Physical activity; Sedentary behavior},
references={Ekelund, U, Tarp, J, Steene-Johannessen, J, Hansen, BH, Jefferis, B, Fagerland, MW, Dose-response associations between accelerometry measured physical activity and sedentary time and all cause mortality: systematic review and harmonised meta-analysis (2019) BMJ, p. l4570. , Aug 21: [FREE Full _text] [doi]; Biswas, A, Oh, PI, Faulkner, GE, Bajaj, RR, Silver, MA, Mitchell, MS, Sedentary Time and Its Association With Risk for Disease Incidence, Mortality, and Hospitalization in Adults (2015) Ann Intern Med, 162 (2), p. 123. , Jan 20; [FREE Full _text] [doi]; Pandey, A, Salahuddin, U, Garg, S, Ayers, C, Kulinski, J, Anand, V, Continuous Dose-Response Association Between Sedentary Time and Risk for Cardiovascular Disease (2016) JAMA Cardiol, 1 (5), p. 575. , Aug 01; [FREE Full _text] [doi]; Global action plan on physical activity 2018-2030: more active people for a healthier world (2018) Noncommunicable diseases and their risk factors, , World Health Organization Geneva: World Health Organization; Iribarren, SJ, Cato, K, Falzon, L, Stone, PW., What is the economic evidence for mHealth? A systematic review of economic evaluations of mHealth solutions (2017) PLoS One, 12 (2), p. e0170581. , Feb 2; [FREE Full _text] [doi] [Medline: 28152012]; ICT Facts and Figures 2016 Mobile network coverage and evolving technologies 2016:2016, , International Telecommunication Union. [FREE Full _text]; (2016) Monitoring and evaluating digital health interventions: a practical guide to conducting research and assessment, pp. 1-144. , WHO. [FREE Full _text] [doi]; Donkin, L, Christensen, H, Naismith, SL, Neal, B, Hickie, IB, Glozier, N., A systematic review of the impact of adherence on the effectiveness of e-therapies (2011) J Med Internet Res, 13 (3), p. e52. , Aug 05; [FREE Full _text] [doi] [Medline: 21821503]; World, HO., WHO guideline: recommendations on digital interventions for health system strengthening (2019) Sexual and reproductive health, , Geneva: World Health Organization; Müller, AM, Maher, CA, Vandelanotte, C, Hingle, M, Middelweerd, A, Lopez, ML, Physical Activity, Sedentary Behavior, and Diet-Related eHealth and mHealth Research: Bibliometric Analysis (2018) J Med Internet Res, 20 (4), p. e122. , Apr 18; [FREE Full _text] [doi] [Medline: 29669703]; mHealth: new horizons for health through mobile technologies (2011) Global Observatory for eHealth series, 3. , WHO Geneva: World Health Organanization; Müller, AM, Alley, S, Schoeppe, S, Vandelanotte, C., (2016) Int J Behav Nutr Phys Act, 13 (1), p. 109. , Oct 10; [FREE Full _text] [doi] [Medline: 27724911]; Maher, C, Lewis, L, Ferrar, K, Marshall, S, De Bourdeaudhuij, I, Vandelanotte, C., Are health behavior change interventions that use online social networks effective? A systematic review (2014) J Med Internet Res, 16 (2), p. e40. , Feb 14; [FREE Full _text] [doi] [Medline: 24550083]; Davies, C, Spence, J, Vandelanotte, C, Caperchione, C, Mummery, W., Meta-analysis of internet-delivered interventions to increase physical activity levels (2012) Int J Behav Nutr Phys Act, 9, p. 52. , Apr 30;: [FREE Full _text] [doi] [Medline: 22546283]; Jahangiry, L, Farhangi, M, Shab-Bidar, S, Rezaei, F, Pashaei, T., Web-based physical activity interventions: a systematic review and meta-analysis of randomized controlled trials (2017) Public Health, 152; Hou, S, Charlery, SR, Roberson, K., Systematic literature review of Internet interventions across health behaviors (2014) Health Psychol Behav Med, 2 (1), pp. 455-481. , Jan 01; [FREE Full _text] [doi] [Medline: 25750795]; Romeo, A, Edney, S, Plotnikoff, R, Curtis, R, Ryan, J, Sanders, I, Can Smartphone Apps Increase Physical Activity? Systematic
[truncated: 642,785 more chars]
